# The underlying process of early ecological and genetic differentiation in a facultative mutualistic *Sinorhizobium meliloti* population

**DOI:** 10.1038/s41598-017-00730-7

**Published:** 2017-04-06

**Authors:** Nicolás Toro, Pablo J. Villadas, María Dolores Molina-Sánchez, Pilar Navarro-Gómez, José M. Vinardell, Lidia Cuesta-Berrio, Miguel A. Rodríguez-Carvajal

**Affiliations:** 1 Structure, Dynamics and Function of Rhizobacterial Genomes, Grupo de Ecología Genética de la Rizosfera, Department of Soil Microbiology and Symbiotic Systems, Estación Experimental del Zaidín, Consejo Superior de Investigaciones Científicas, C/Profesor Albareda 1, 18008 Granada, Spain; 2 Departamento de Microbiología, Facultad de Biología, Universidad de Sevilla, Avenida de Reina Mercedes 6, 41012 Sevilla, Spain; 3 Departamento de Química Orgánica, Universidad de Sevilla, C/Profesor García González, 1, 41012 Sevilla, Spain

## Abstract

The question of how genotypic and ecological units arise and spread in natural microbial populations remains controversial in the field of evolutionary biology. Here, we investigated the early stages of ecological and genetic differentiation in a highly clonal sympatric *Sinorhizobium meliloti* population. Whole-genome sequencing revealed that a large DNA region of the symbiotic plasmid pSymB was replaced in some isolates with a similar synteny block carrying densely clustered SNPs and displaying gene acquisition and loss. Two different versions of this genomic island of differentiation (GID) generated by multiple genetic exchanges over time appear to have arisen recently, through recombination in a particular clade within this population. In addition, these isolates display resistance to phages from the same geographic region, probably due to the modification of surface components by the acquired genes. Our results suggest that an underlying process of early ecological and genetic differentiation in *S. meliloti* is primarily triggered by acquisition of genes that confer resistance to soil phages within particular large genomic DNA regions prone to recombination.

## Introduction

Selection, genetic drift and gene flow are evolutionary forces that model microbial genomes by driving adaptation and speciation in bacteria. According to the ecotype model^1–3^, genotypic clusters in bacterial populations evolve through the spread of acquired adaptive genes in genome-wide selective sweeps. The whole genome of the variant hitchhikes with the adaptive loci, and, after rounds of periodic selection events purging diversity genome-wide, this variant spreads, replacing the parental organism and its descendants. Likewise, the acquisition of niche-specifying genes may lead to the coexistence of distinct clusters. However, recent studies in marine bacteria and thermophilic archaea populations have suggested that adaptive loci may sweep independently of the rest of the genome, with niche-specifying variants spreading in a gene-specific sweep^4–7^. These studies identify regions of the genome containing densely clustered divergent SNPs denoted as “islands and continents of speciation”.

Rhizobia are soil bacteria; most are able to fix nitrogen and elicit the formation of root nodules on legume plants^8, 9^, within which they convert atmospheric nitrogen (N_2_) into ammonia. They mostly belong to the *Rhizobium*, *Mesorhizobium*, *Sinorhizobium* (syn. *Ensifer*) or *Bradyrhizobium* genera. *Sinorhizobium meliloti* can be found as a free-living soil organism or in symbiosis with plants of the genera *Medicago, Melilotus* and *Trigonella* ^10–13^. *S. meliloti* harbors a multireplicon genome consisting of a single circular chromosome (~3.65 Mb) and two large symbiotic (Sym) plasmids of ~1.3 (pSymA megaplasmid) and ~1.6 Mb (pSymB chromid) in size, together with smaller accessory plasmids^14^.

Like other rhizobia, *S. meliloti* has a composite lifestyle. In the soil and rhizosphere environments, its resources are generally distributed in patches, forming microgeographically overlapping niches with distinct barriers to gene flow. Moreover, the root nodules may be considered to be a microhabitat separating the variants, thereby purging diversity genome-wide in a periodic selection event. After nodule senescence, many of the undifferentiated nodule bacteria are released into the soil, increasing the prevalence of host plant-adapted genotypes^13^. The interplay between separation into different microhabitats, plant selection, and gene flow boundaries underlies the evolution of these facultative mutualistic soil bacteria. The complex populations formed by these bacteria thus constitute an excellent model for studies of the emergence, maintenance and spread of nascent clusters under natural selection.

Genomic analyses based on genome-wide sequence data have been carried out for *S. meliloti* and the closely related *S. medicae* species^15–20^. However, genome coverage was low or the isolates were obtained from different geographic sites, making it difficult to differentiate between the effects of environmental selection from those of genetic divergence due to geographic separation. *S. meliloti* strain GR4 is a nitrogen-fixing highly competitive bacterium for nodulation on alfalfa that was isolated over 35 years ago at the Estación Experimental del Zaidín (Granada, Spain) field site^21^. We recently^22^ analyzed genomic variation within closely related and sympatric S*. meliloti* isolates (the so-called GR4-type population) obtained from *M. sativa* (alfalfa) root nodules at the same field site^23^. These isolates formed a highly clonal population that had recently undergone demographic expansion. This bacterial population displayed very low levels of nucleotide sequence diversity for the chromosome, pSymB and pSymA replicons, but displayed other types of variation, such as indels, genomic island excision, the transposition of mobile elements and some recombination events.

Here, we analyzed 21 isolates representative of the genomic variation within the GR4-type population^22, 23^ and used the genomic information obtained to make inferences about how environmental selection promotes the early emergence, maintenance and spread of variation within a facultative mutualistic soil bacterial population, and the genetic basis underlying early ecological and genetic differentiation.

## Results

### Genome-wide sequence analysis

GR4-type isolates were obtained from alfalfa root nodules in the fall of 1996 and the summer of 1997^23^, at the same field site from which the reference *S. meliloti* GR4 strain was isolated^21^. The overall genomic structure and variation within these isolates were initially determined by fingerprint analyses with IS*Rm2011-2* and the group II intron RmInt1 as DNA probes, which showed that 22% of the GR4-type isolates displayed variations^22, 23^. We recently sequenced 13 isolates representative of this genomic variation and reported that one of these isolates (G6) harbored a pSymB replicon carrying two long regions with features of genetic exchange and replacement^22^. One of these regions encompassed almost 200 kb (reference strain GR4 coordinate positions 1,098,191 to 1,296,315) of sequence containing a large number of SNPs in the conserved syntenic genes, with gene acquisition and loss^22^. Here, we sequenced eight additional isolates within this population. Genome-wide sequence analysis with Illumina technology and the mapping of data reads onto the GR4 reference strain genome^22, 24^ identified another isolate (7G9) with a large stretch of DNA containing SNPs within the pSymB replicon (coordinate positions 1,091,946 to 1,262,153) and resembling that found in isolate G6. Like many other isolates from the same population^22^, 7G9 lacks the accessory plasmid pRmeGR4a, and displays low levels of polymorphism (SNPs and indels, Supplementary Table S1) in all replicons (chromosome, pSymA, pSymB and the smaller plasmid pRmeGR4b). Additional polymorphisms detected in both 7G9 and G6 out of the island of dense polymorphism in pSymB (hereafter referred to as GID, for “genomic island for differentiation”), were found to be common to the other isolates sequenced, therefore, unlikely to be responsible for a phenotype specific to these two isolates.

### Genealogy of the isolates carrying the GID

**Figure 1:**
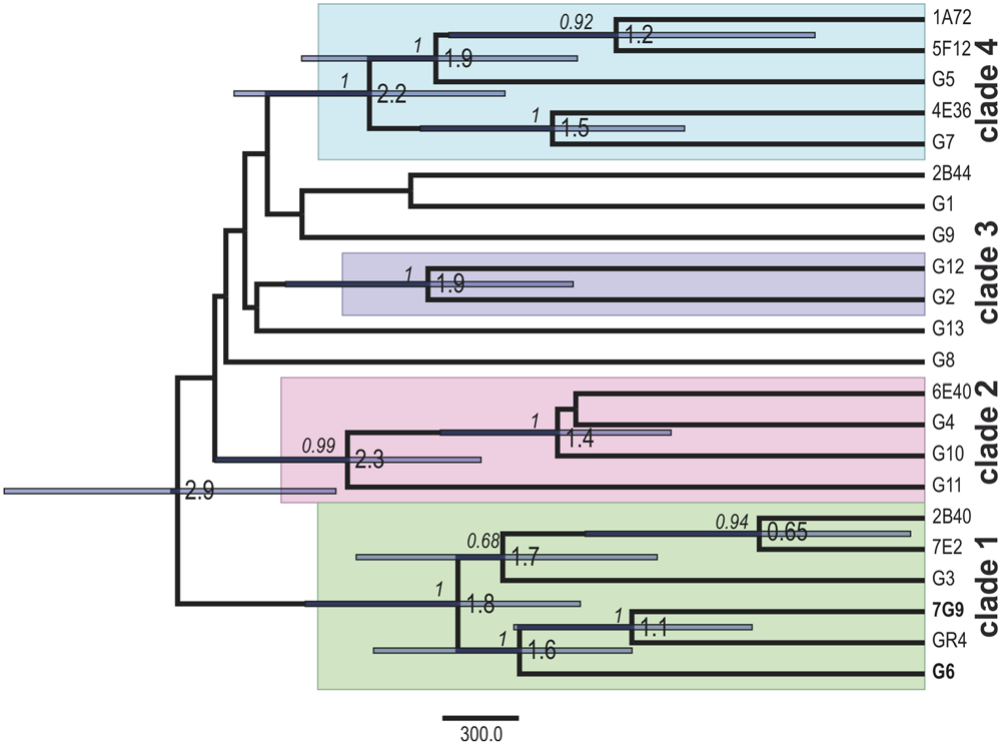
Genealogy of the 21 isolates representative of genomic variation within the *S. meliloti* GR4-type population. The maximum clade credibility tree was estimated with BEAST 2^25^, from the alignment (Supplementary Data 1) of concatenated genes carrying SNPs from the chromosome, pSymB and pSymA replicons (280,068 nts) of all isolates, as previously determined within the GR4-type population^22^. Node bars indicate the 95% credibility intervals (95% HPD) for node age, and posterior probabilities (>0.5) are indicated (in italics) on the branches. The estimated node ages (indicating the branching time in thousands of years ago) and the phylogenetically supported clades are indicated. Isolates carrying the GID are indicated in bold typeface.

We inferred the genealogy of the newly sequenced isolates and those carrying the GID, by performing Bayesian analyses^25^ on the previously determined set of data for concatenated genes carrying SNPs within this population^22^. Three of the eight isolates sequenced (7G9, 2B40 and 7E2) clustered within clade 1, which diversified recently (~1.8 thousand years ago), and includes the previously characterized isolates G6, G3, and the reference strain GR4 (Fig. 1). The GID therefore appears to present at low frequency in this population (two of 21 different isolates sequenced), but it occurs exclusively in a particular subgroup within a single clade. The low frequency of the GID and the low level of diversity throughout the genome in this population suggest that the GID was acquired recently, by recombination, rather than being a remnant from ancient clonal frame divergence.

### Characterization of the GID

**Figure 2:**
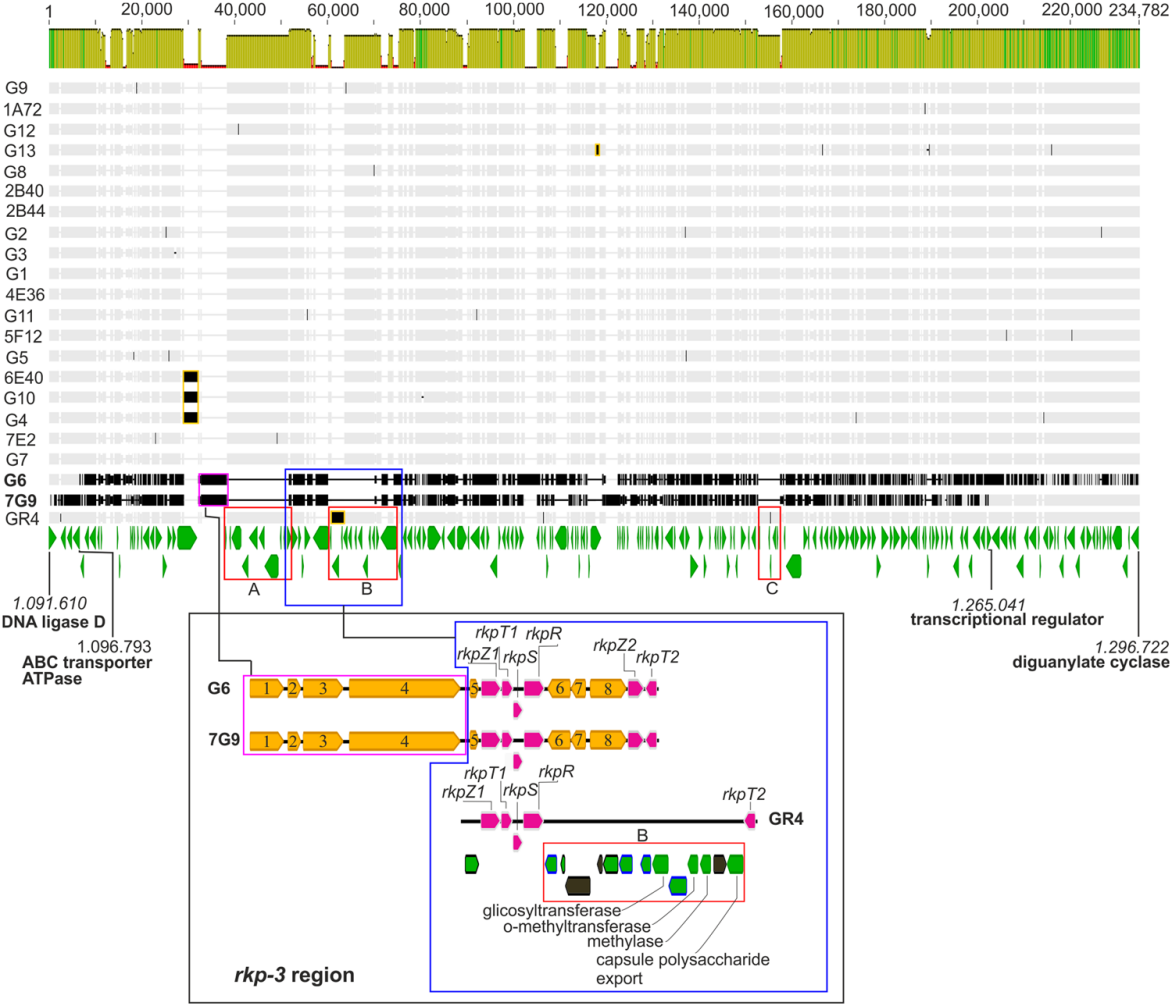
Comparison of the genome region encompassing the GID of pSymB in the GR4-type population. Alignment (Supplementary Data 2) of GR4-type isolates for the region of the genome encompassing the GID on pSymB. Mean pairwise identity is shown above the alignment (bright green: 100% identity), khaki green (between 30% and 100% identity). Transposase sequences in the alignment are framed in yellow and discordances with respect to the consensus are shown in black. The genes at the left and right boundaries are indicated (DNA ligase D, WP_015243213.1; ABC transporter ATPase, WP_015243216.1; transcriptional regulator, WP_015243340.1; and diguanylate cyclase, WP_015243358.1), together with their coordinate positions in the reference strain GR4 pSymB replicon. The genes identified in the GR4 population are shown below the alignment in green. The three major regions (A, B and C) containing genes mostly encoding hypothetical proteins absent from the isolates are indicated. An enlargement of the *rkp-3* region and its genetic organization is also shown. Genes with only distant homologs in databases are shown in orange and numbered: methyltransferases (1 and 3); a hypothetical protein (2); glycosyltransferases (4, 7 and 8); a nucleotidyl transferase (5) and a sugar phosphotransferase (6). The conserved *rkp* genes are shown in pink. In enlarged region B, the transposases are shown in black, the annotated truncated genes are framed in black, and the genes encoding hypothetical proteins are framed in blue.

*De novo* assembly of the reads for the 21 sequenced isolates and alignment with the reference GR4 strain made it possible to define the region of the genome encompassing the GID (Fig. 2). Detailed observation of the GID in the two isolates (G6 and 7G9) revealed boundary differences. The GID was larger in G6, in which it appeared to begin within the 5′end of an ABC transporter ATPase gene and to end at a diguanylate cyclase gene. By contrast, in 7G9, the left boundary of the GID seemed to extend into the 3′end of a DNA ligase D-encoding ORF, and right boundary ended in the 5′end of a transcriptional regulator. The GID of 7G9 was about 32 kb shorter than that of G6.

This region of the pSymB replicon is characterized by the presence of the *rkpZ1*, *rkpT1*, *rkpS*, *rkpR* and *rkpT2* genes within the *rkp*-*3* gene region^26–28^, with these genes arranged in a manner similar to that in *S. meliloti* strain1021, which lacks the *rkpLMNOPQ* gene cluster. These genes are potentially involved in capsular polysaccharide (KPS or K-antigen) biosynthesis and export through the cell membrane. In isolates G6 and 7G9, these genes are conserved, but highly polymorphic. Upstream from these loci, several genes encoding hypothetical proteins (region A) are absent, and have been replaced with genes encoding potential methyl- glycosyl- and nucleotidyl transferases in the isolates. These enzymes have distant homologs in databases. Similarly, between *rkpR* and *rkpT2* (region B), the isolates have a different, shorter series of genes potentially encoding a sugar phosphotransferase and several glycosyltransferases. Again, these enzymes have distant homologs in databases. The isolates also carry an *rkpZ2* gene potentially encoding a capsule polysaccharide export protein upstream from *rkpT2* and transcribed in the opposite orientation. Homologs of this gene are present in other *S. meliloti* strains.

**Figure 3:**
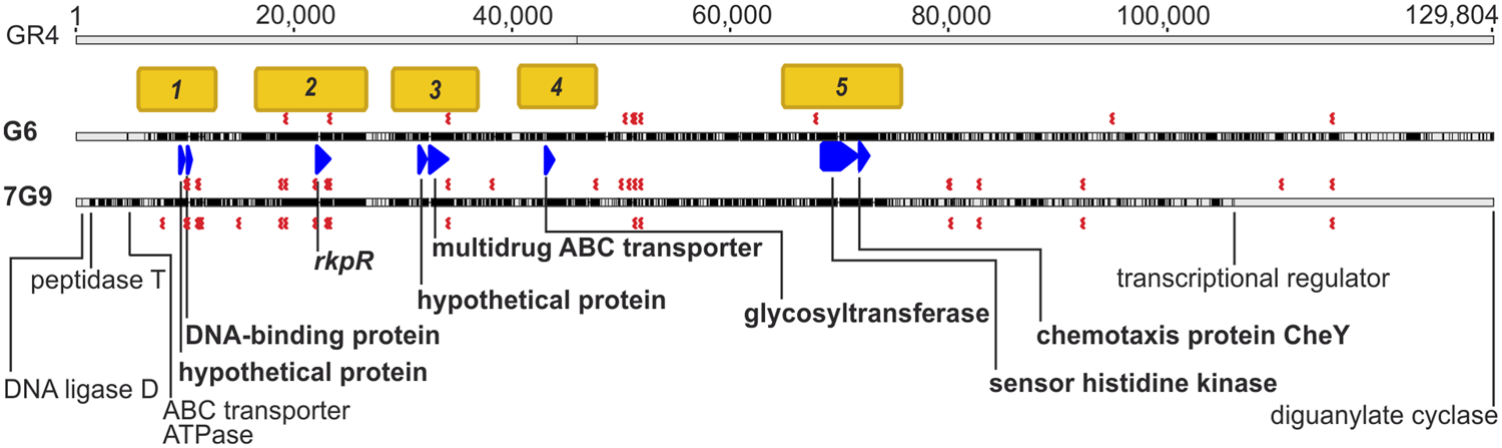
The pSymB core genes of the GID. Alignment of concatenated genes encompassing the GID conserved in all GR4-type isolates (Supplementary Data 3). Only the 7G9, G6 and reference GR4 strain sequences are shown, for the sake of clarity. The identified regions (1 to 5) displaying higher levels of nucleotide sequence diversity (orange) within the GID are shown, together with the genes displaying the highest levels of diversity within each region (blue): in Region 1 (5,750–13,028), hypothetical protein (WP_015243219.1) and DNA-binding protein (WP_003533107.1); in Region 2 (16,528–26,785), capsule polysaccharide export protein R kpR (WP_015243241.1); in Region 3 (29,035–37,036), hypothetical protein (WP_010975434.1) and multidrug ABC transporter ATPase/permease (WP_015243259.1); in Region 4 (40,537–47,788), a glycosyltransferase involved in cell wall biogenesis (WP_015243266.1); and in Region 5 (64,849–75,854), sensor histidine kinase (WP_015243309.1) and chemotaxis protein CheY (WP_015243310.1). The red symbol below each sequence indicates the position of the flexible genome removed for alignment purposes. Discordances with respect to the consensus sequence are shown in black.

By applying a sliding-window approach to the set of genes encompassing the GID present in all isolates (core genes) (Fig. 3), we identified five stretches of DNA (Regions 1 to 5) displaying high levels of nucleotide diversity (π), separated by regions of lower divergence (Supplementary Fig. S1), potentially corresponding to a signature of adaptive genes. The conserved *rkp*-*3* gene cluster (*rkpZ1*, *rkpT1*, *rkpS*, *rkpR* and *rkpT2*) was found in Region 2. DNA polymorphism analyses within these regions of high diversity identified the genes with the highest levels of divergence (Fig. 3; Supplementary Fig. S1).

**Figure 4:**
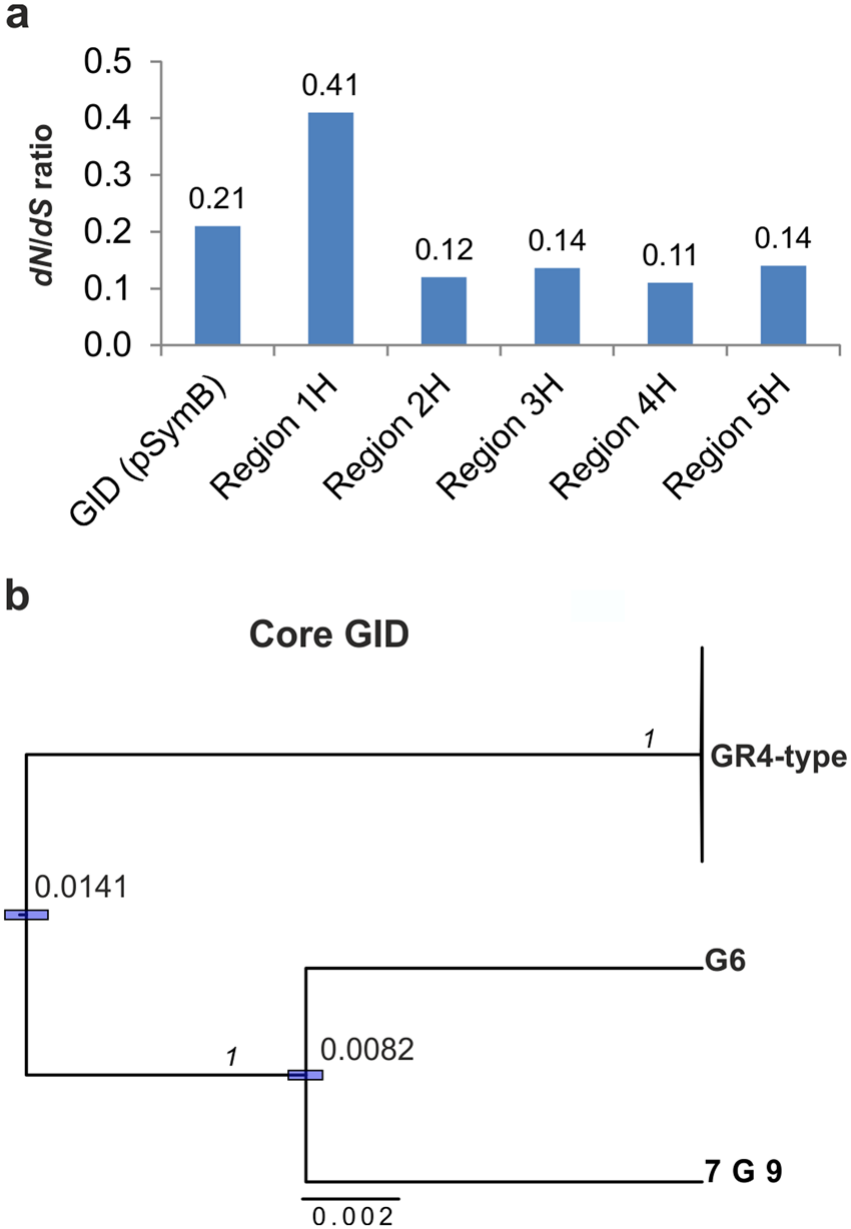
Ratio of synonymous to nonsynonymous mutations and divergence of the GID. (**a**) The calculated *dN*/*dS* ratio for the core concatenated genes carrying SNPs within the GID, and for the genes displaying higher (H) levels of nucleotide sequence diversity within each of the five regions of greater diversity (Fig. 3). (**b**) The maximum clade credibility tree estimated with BEAST 2, based on the alignment of the core GID. Node bars indicate the 95% credibility intervals (95% HPD) for node ages, which are also shown as nucleotide substitution per site; the posterior probability is shown above the branches (in italics). The cluster containing the other 19 sequenced GR4-type isolates and the reference GR4 strain was collapsed for simplicity.

The core genes of the GID carry 4,615 SNPs (synonymous/nonsynonymous), 1,638 (~35%) of which are nonsynonymous. Interestingly, all the *dN*/*dS* values (Fig. 4a) for the genes showing higher levels of nucleotide sequence diversity within the GID were well below one (0.11 to 0.41), but similar to that of the whole GID (0.21), consistent with selective constraints. Finally, the phylogenetic tree (Fig. 4b) inferred from the core genes suggests that the G6 and 7G9 GIDs have a longer divergence time than the equivalent region in the other GR4-type isolates, and that they have evolved independently for some time, possibly before their acquisition.

### The origin of the GID

**Figure 5:**
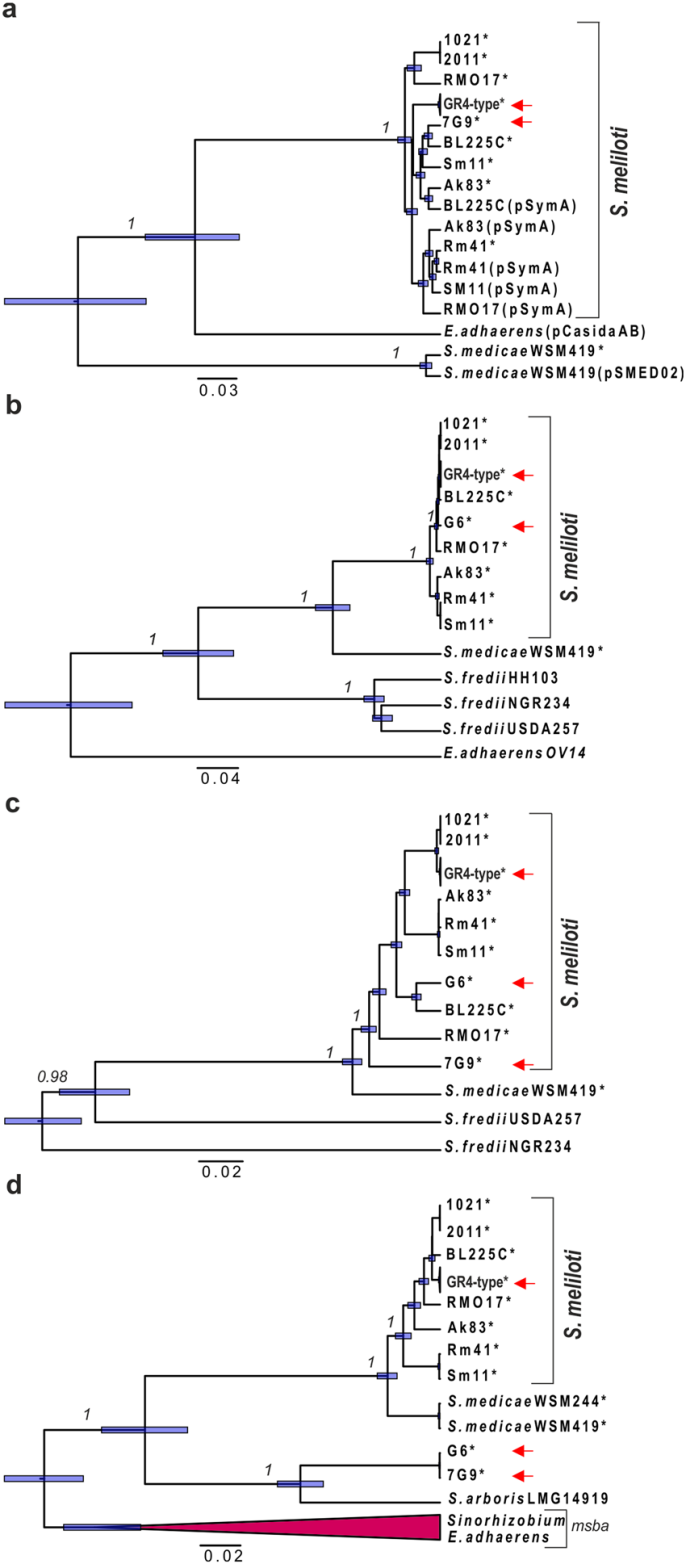
Species trees for GID core genes. The maximum clade credibility tree estimated with BEAST 2, based on the alignment of core loci (Supplementary Data 4–7) located at the left and right boundaries of the GID, and internal genes with higher levels of nucleotide diversity conserved in *Sinorhizobium* species. (**a**) Peptidase T (left boundary, WP_015243214.1). (**b**) Diguanylate cyclase (right boundary, WP_015243358.1). (**c**) Sensor histidine kinase (WP_015243309.1) within Region 5. (**d**) multidrug ABC transporter ATPase/permease (WP_015243259.1) within Region 3. We included paralogous *msba* (collapsed branches) gene sequences (homologous to *E. coli msba* gene encoding Lipid A export ATP-binding/permease protein) in the alignment, to improve the definition of the phylogenetic position of the locus harbored by the isolates. Node bars indicate the 95% credibility intervals (95% HPD) for node ages. The scale bar represents the number of nucleotide substitutions per site. The asterisks (*) indicate location in pSymB and orthologous plasmids, and the red arrows identify GR4-type isolates and the isolates G6 and 7G9. The names of the other plasmids present are given in brackets.

We investigate the origin of the GID further, by inferring species trees from the genes at its boundaries and those within the island displaying the highest levels of nucleotide sequence diversity but conserved in the *S. meliloti* strains sequenced, with orthologous genes in other *Sinorhizobium* species. We first inferred trees from loci at the left and right borders of the GID: the peptidase T gene located downstream from the DNA-ligase D gene; and the diguanylate cyclase gene, respectively (Fig. 3). As shown in Fig. 5a,b; these loci from G6 and 7G9 cluster with the *S. meliloti* species, suggesting recent acquisition of the GID by these two isolates, probably from coexisting S*. meliloti* populations. Similarly, the species tree obtained from the alignment of sensor histidine kinase sequences (Region 5) originated within the species *S. meliloti*, but those for G6 and 7G9 appeared to originate from different populations (Fig. 5c). Interestingly, the species tree inferred from the multidrug ABC transporter ATPase/permease (Region 3) indicated that the variant forms of this gene had an external common origin, with possible acquisition from the closely related *S. arboris* species (Fig. 5d). Given that there is no evidence for such rampant recombination events in the full genome sequences of the 21 isolates, we conclude that the two extant versions of the GID probably arose through multiple recombination events with sequences acquired from other sympatric populations. These events may have also occurred after the divergence of these sequences, but likely before their acquisition by the 7G9 and G6 isolates.

### The isolates carrying the GID have altered cell surface components and are resistant to phages

**Figure 6:**
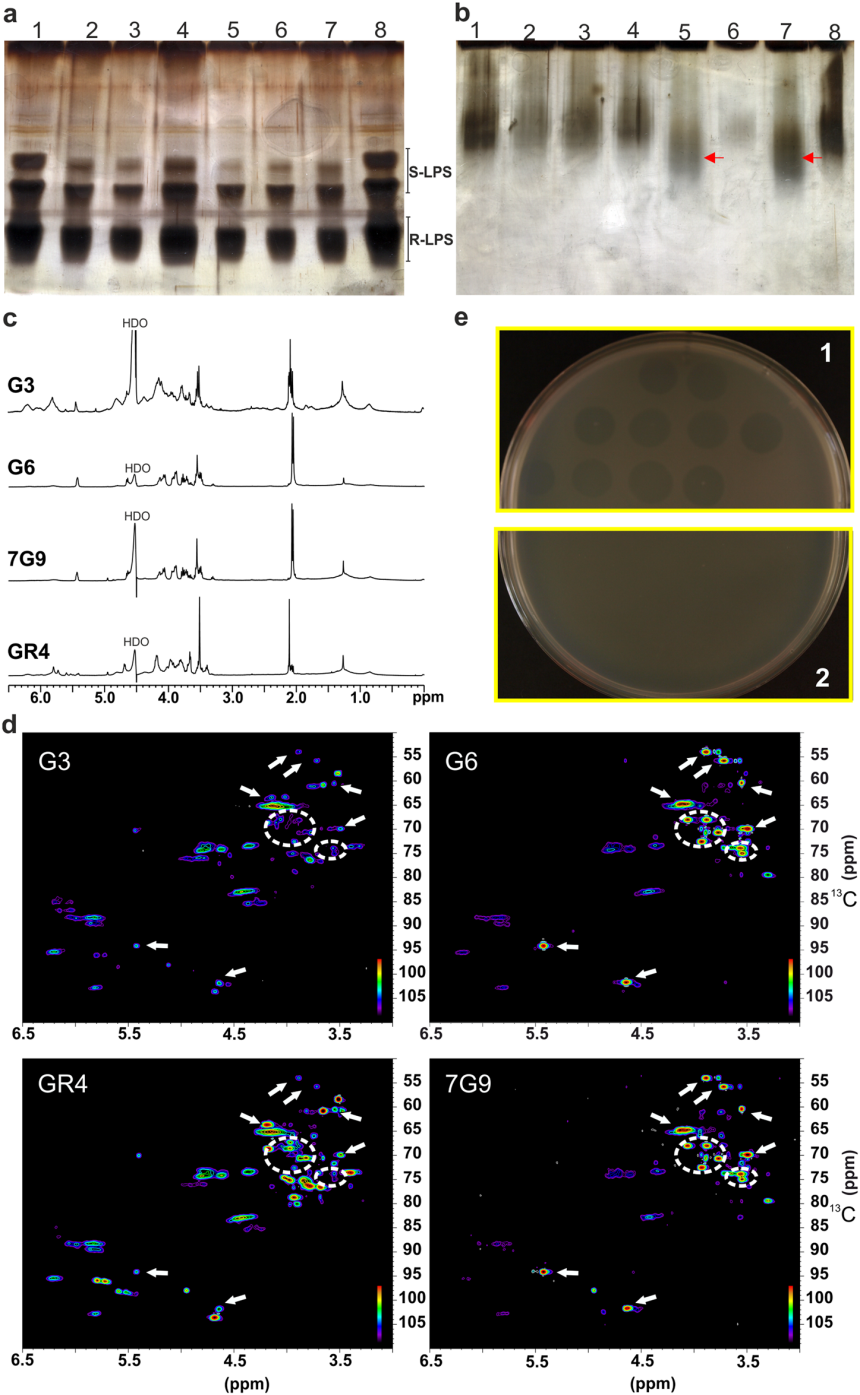
Analyses of the cell surface components of the isolates and resistance to phages. (**a**) SDS-PAGE of crude LPS extracts of various *S. meliloti* strains. The rough and smooth forms of LPS are indicated as R-LPS and S-LPS, respectively. (**b**) PAGE analysis of crude KPS extracts. For crude KPS extracts, samples were run in the absence of detergent (SDS), treated with Alcian Blue and silver-stained. Lane 1, GR4; lane 2, 2B40; lane 3, 2C67 (a field isolate with identical fingerprint to GR4 strain)^23^; lane 4, G3; lane 5, G6; lane 6, 7E2; lane 7, 7G9; lane 8, GR4. The red arrows highlight the low-molecular mass capsular polysaccharide in the G6 and 7G9 isolates. (**c**) 1H-NMR spectra (500 MHz, 323 K), and (**d**) 1H-13C HSQC spectra of the high-molecular weight fraction isolated from G3, G6, 7G9, and GR4 cells. Signals from main component present in extracts from G6 and 7G9 are marked; these signals, at lower intensities, can also be found in G3 and GR4 extracts. (**e**) Phage infection test on GR4-type isolates. An example of phage plaques on GR4-type isolates from clade 1 (plate 1) and on resistant G6 or 7G9 cells (plate 2).

Based on the above results, we hypothesized that the acquisition of the GID by the isolates might have altered the cell surface components of the isolates. We tested this hypothesis, by performing PAGE analyses on the LPS and K-antigens (KPS) of the isolates within clade 1. The isolates had essentially unchanged LPS profiles (Fig. 6a). However, the KPS profile of the 7G9 and G6 isolates differed from those of the other isolates and that of the reference GR4 strain, in having a faster migration pattern (Fig. 6b). Surface polysaccharides were extracted and the resulting high-molecular weight fractions (above 6 kDa), containing mixtures of LPS and KPS, were studied by NMR. ^1^H-NMR spectra (Fig. 6c) of these isolates show characteristic signals from polysaccharides, with signals from anomeric hydrogens located between 4.5 and 6 ppm, and signals from non-anomeric hydrogens located between 4.5 and 3 ppm. It can be seen that ^1^H-NMR spectra from G6 and 7G9 are almost identical (except for the signal from residual water, marked as HDO), and different from those from G3 and GR4. Moreover, given that spectra from G6 and 7G9 show mainly two signals from anomeric hydrogens (at 5.43 and and 4.64 ppm) and the small number of signals in the region of 4.5–3.0 ppm, the structure of this polysaccharide must be relatively simple. On the contrary, the anomeric region of G3 and GR4 present multiple signals, some of them broad. NMR analysis included the acquisition of ^1^H-^13^C HSQC (Heteronuclear Single Quantum Coherence spectroscopy) spectra (Fig. 6d). This kind of NMR experiment is more suitable for studying crowded spectra, as it adds a second dimension (^13^C chemical shifts, in this case) that allows spreading out the peaks over a much larger area. The signals in ^1^H-^13^C, which appear as spots and their intensities are indicated by a color code in Fig. 6d, correlate carbon signals with directly bound protons. ^1^H-^13^C HSQC spectra of all of the isolates show very similar signal distributions, that is, they show approximately the same signals at the same combination of ^1^H/^13^C chemical shifts, but their intensities are different. This result indicates that all of the isolates contain the same mixture of polysaccharides but in different ratio. In particular, the main component of G6 and 7G9 is a relatively simple polysaccharide (signals marked in Fig. 6d) that is present as a minor component in G3 and GR4, whereas these last isolates present mainly a mixture of more complex structures.

The observed changes in cell surface expression in the isolates may lead to resistance to predators and pathogens (e.g., bacteriophages), as previously reported^27–29^. We tested this hypothesis, by isolating phages from soil from the same geographic region on which alfalfa was grown to obtain the isolates, and screening the clade 1 isolates for phage resistance. Only 7G9 and G6 were resistant to the phages isolated (Fig. 6e), confirming our hypothesis and demonstrating the importance of bacteriophages as a major selective agent driving the acquisition of different KPS genes within the GR4-type population.

### Symbiosis is not defective in the isolates carrying the GID

**Figure 7:**
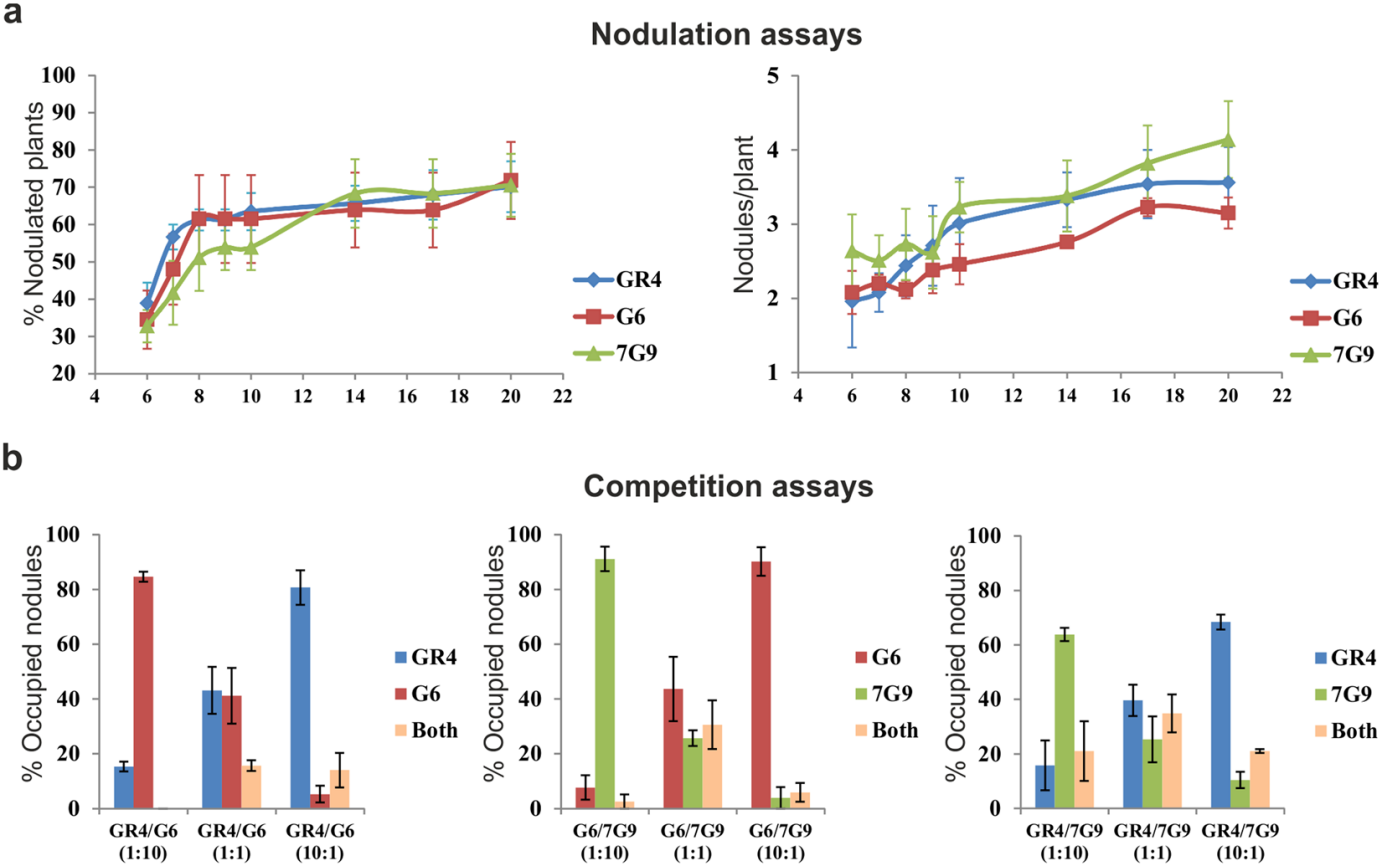
Nodulation and competition assays on *M. sativa*. (**a**) Nodulation assays. Quantification of the number of nodules per plant and the percentage of nodulated plants obtained with each of the isolates indicated and the GR4 reference strain. The x-axis indicates the number of days post-inoculation. (**b**) Competitive nodule occupancy assays. Bacterial cells were co-inoculated at ratios of 1:1, 1:10 and 10:1 and their presence within the nodules was determined by PCR, with the use of specific primers to distinguish them (Supplementary Table S2).

At the field site sampled, the nodules occupied by GR4-type isolates with an IS/group II intron (IS*Rm2011-2*/RmInt1) fingerprint identical to that of strain GR4 displayed the highest level of nodule occupancy (78%). Nodule occupancy rates were much lower (22%) for the isolates showing variations in the fingerprint^23^. We investigated whether the apparently lower nodule occupancy for the 7G9 and G6 isolates was due to an altered symbiotic phenotype caused by the change in cell surface components, by performing nodulation and competition assays on alfalfa plants. The ability of these isolates to elicit nodules on alfalfa roots was not very different from that of the GR4 reference strain (Fig. 7a). Furthermore, ability to compete for nodule formation was influenced principally by population size (Fig. 7b). Thus, the low frequency of these isolates within the root nodules may be better explained by the presence of a smaller population in the soil rather than a symbiotic defect.

## Discussion

Our study illustrates how environmental selection leads to the emergence, maintenance and spread of variation within a population of facultative mutualistic soil bacteria. Our work suggests that related versions of a genomic island carrying genes involved in capsular polysaccharide biosynthesis and transport (KPS genes) have recently been replaced with an equivalent region, through horizontal gene transfer and homologous recombination, in some isolates from the *S. meliloti* GR4-type population. The acquisition of the GID modified the cell surface of the isolates, potentially accounting for their resistance to bacteriophages isolated from the same geographic region. The symbiotic phenotype of these isolates was unaltered, so the low frequency of nodule occupancy by these isolates in the field^24^ at the time of isolation (1996–1997) probably reflects the small size of this subpopulation in the soil. Thus, the isolates carrying the GID are probably maintained within the population by negative frequency-dependent selection^30^.

The GIDs harbored by the 7G9 and G6 isolates are clearly separated phylogenetically from the equivalent region in the GR4-type genome, and they appear to be under selective constraints. The GIDs of the G6 and 7G9 isolates appear to have a common origin, but they also evolved independently for some time before their acquisition by these two isolates, as shown by their overall divergence times and different phylogenies for some of the conserved genes with higher levels of nucleotide sequence diversity. The species trees based on genes flanking the GID and conserved in *S. meliloti* and closely related species suggest that these islands were recently acquired, possibly from other sympatric *S. meliloti* populations. Nevertheless, the presence of outlier genes and other genes more closely related to those of other *Sinorhizobium* species suggest that different versions of the GID available from the sympatric gene pool were generated by multiple genetic exchanges over time. These events occurred in a particular genomic region located in the symbiotic pSymB replicon that is probably prone to recombination in the GR4-type genome. Our findings are in agreement with previous observations that suggested that the pSymB chromid has a distinctive role in intraspecies differentiation^18^.

According to a recently proposed model^31^ of microbial speciation in nature, after the arrival of adaptive genes or alleles in an ancestral population, through mutation or horizontal gene transfer, the spread of these variations depends on the balance between recombination and selection and occurs either through a gene-specific selective sweep or by clonal expansion. In the 7G9 and G6 isolates, the genes that confer local phages resistance arrived with a long stretch of divergent DNA (≈170 and 200 kb). The clustering of the 7G9 and G6 isolates harboring the GID within clade 1 and the genome-wide low diversity shown by all the sequenced isolates within the GR4-type population suggest that, after acquisition, the different versions of the GID spread by clonal expansion, at least during the early stages of ecological and genetic differentiation.

The distribution of bacteriophages in the soil, plant rhizosphere or root nodules is unknown. However, we can hypothesize that, by avoiding the pressure of local soil bacteriophages, these isolates carrying the GID may be able to spread to other sympatric niches, allowing them to search for new resources and increase their population. Moreover, as plant root nodules have a selective effect, increasing the proportion of host plant-adapted genotypes in the soil, and the isolates carrying the GID have no symbiotic defect, rounds of periodic selection by the plant should result in the gradual replacement of the current major GR4-type genotype lacking the GID with former isolates in plant nodules. The particular mosaic structure of the GID suggests that, at later stages of ecological differentiation, new recombination events within these islands and other parts of the genome will generate further diversity. Nevertheless, in each round of selection, the plant will also purge genetic diversity within these isolates, as suggested by the stable ecotype model^1–3^. Understanding the evolution of phage specificity and bacterial sensitivity into the local environment is essential to predicting the impact of phages in shaping microbial communities^32^. Thus, further studies on the coevolutionary dynamics between the GR4-type population and bacteriophages that infect distinct isolates within this population may contribute to understanding microbial diversity.

In conclusion, we provide insights into the underlying the process of early ecological and genetic differentiation in a population of facultative plant mutualistic soil bacteria. In nature, bacteriophages exert strong selection, driving the emergence, maintenance and spread of isolates that have acquired genes that confer local phages resistance within particular divergent large genomic DNA regions (GID) generated by multiples genetic exchanges over the time. The present of distinct versions of these GID caused by random mutation and further recombination events in the sympatric gene pool increase the genetic diversity of these variations, and presumably their successful spread within the population by genome-wide selective sweeps. We hypothesize that the multireplicon genome of *S. meliloti* may evolve through acquisition of these GID and further genome scans of closely related isolates may identify other regions that are good candidates for driving population differentiation.

## Methods

### Bacterial genome sequencing data used in this study

The complete annotated genome sequence of *S. meliloti* strain GR4^22, 24^, and whole-genome sequence data from 21 GR4-type isolates representing distinct variations of the GR4 strain fingerprint obtained with IS*Rm2011-2* and the group II intron RmInt1 as DNA probes^22^ were used in this study. Genome sequencing data of 13 of these GR4-type isolates (3G48, 3D13, 3F11, 7D33, 5G35, 5F20, 7G54, 2B2, 1A66, 7A75, 5D25, 1B5 and 2A8, referred to as G1 to G13, respectively) were reported previously^22^.

### Genome sequencing and assembly of the reads

In this study, eight additional GR4-type isolates (1A72, 5F12, 4E36, 2B44, 6E40, 2B40, 7E2 and 7G9) previously characterized by DNA fingerprinting^22^ were sequenced by Macrogen Inc. (South Korea), with Illumina paired-end technology, using multiplex MiSeq runs (2 × 300 bp) and a with a mean coverage of more than 80x, as previously described^22^. Illumina reads were mapped separately to each replicon with Geneious Pro Software v 8.0^33^ (Biomatters Ltd; http://www.geneious.com): the chromosome (NC_019845), pSymB (NC_019849), pSymA (NC_019848), and accessory replicons pRmeGR4b (NC_019847) and pRmeGR4a (NC_019846). Mapping was carried out with minimum identity overlaps of 100, 99, 95 and 90%, over up to five iterations. *De novo* assembly of the reads for the above isolates and the 13 previously sequenced isolates (G1 to G13) was performed with the Geneious and MIRA assembler implemented in Geneious Software. The contigs carrying the GID were identified by mapping conserved DNA sequences within the previously identified island in the G6 isolate. The potential genes within the GID were identified and annotated with Glimmer 3^34^.

### BLAST searching and phylogenetic analyses

The nr (nucleotide and protein) database maintained by NCBI was used as a target for searches with *e*-value threshold of <10^−6^ for homology. Distant homologs are referred here to sequences showing in the corresponding alignment ≤65% pairwise identity. BEAST 2^25^ software was used for Metropolis-coupled Markov chain Monte Carlo (MCMC) analysis of multiple sequence alignments as previously described in detail^22^. We applied a HKY substitution model with gamma correction of between-site rate variation for four rate categories and a strict molecular clock rate of 1 (node ages are given as nucleotide substitutions per site) or of 2.03 × 10^−8^ per site per year^22^ (node ages given in thousands of years). For the tree prior, we used a coalescent Bayesian skyline plot. Each MCMC sample was based on a run of 10,000,000 generations, sampled every 1,000 generations, with the first 1,000,000 generations discarded as burn-in. We used Tracer v1.6 to analyze the Bayesian runs, to confirm that there was a suitable effective sample size (i.e. ESS values were greater than 200)^35^ for all parameters estimated from the posterior distribution of the trees, with confirmation of the stationary state of each chain following the removal of a suitable number of burn-in runs (10%) and convergence of the runs. TreeAnnotator, from BEAST 2 software, was used to summarize the tree output file, generating a maximum clade credibility tree with a 10% burn-in and median branch lengths with standard deviations. Trees were visualized with Figtree v1.4.2 (tree.bio.ed.ac.uk).

### DNA polymorphism analyses

DNA polymorphism analyses were performed with MEGA 6.0^36^ and DnaSP v5.10.01 software^37^. Maximum likelihood analysis of natural selection, codon-by-codon, in MEGA 6.0 was used to estimate the numbers of inferred synonymous and nonsynonymous substitutions and to calculate the number of synonymous substitutions per site (*dS*) and the number of nonsynonymous substitutions per site (*dN*).

### Nodulation test and competition assays

*Medicago sativa* L. ‘Aragón’ (alfalfa) seedlings were prepared for nodulation as previously described^38^. Briefly, the seeds were surface-sterilized with 2.5% HgCl_2_, washed with sterile water and germinated on filter paper in Petri dishes. They were then placed on filter paper strips in glass tubes (20 by 200 mm) containing 10 ml of sterile nitrogen-free mineral solution. They were incubated in these tubes, under controlled light and temperature conditions (16 h light at 24–26 °C and 8 h dark at 20–22 °C). Seven-day-old plantlets were inoculated with the rhizobial suspensions to a final density in the plant growth medium of 10^6^ cells/ml as previously described^38^.

We analysed the nodulation kinetics of the isolates in three independent sets of about 15 plants grown hydroponically in test tubes, by recording the number of plants with nodules and the number of nodules per plant various numbers of days after inoculation.

For competition assays, 15 seven-day-old alfalfa plants grown hydroponically in test tubes were inoculated with 10:1; 1:1 and 1:10 mixtures of two strains of *S. meliloti*. Around 20 mature nodules were collected 20 days after plant inoculation, surface-sterilized by incubation for 5 minutes in 0.25% HgCl_2_, crushed and simultaneously plated on TY medium. The analysis was carried out three times. DNA was obtained from bacteria grown on TY medium and tested by PCR (Supplementary Table S2) to determine which strain or strains occupied the nodules.

### PAGE analysis of lipopolysaccharide (LPS) and K-antigens (KPS)

LPS extraction from bacterial cultures grown on solid TY medium, separation on SDS-PAGE gels and silver staining were performed as previously described^39^. K-antigen capsular polysaccharides (KPS) were extracted from bacterial cultures grown on solid TY medium, as previously described^40^. Samples were analyzed by PAGE, as previously described^41^, except that absolute ethanol was added to the running buffer, and the running and stacking gels, at a final concentration of 10% (v/v) in all cases. The acrylamide concentration of the running gel was 18% (w/v), and the acrylamide/*N*,*N*′-methylene bisacrylamide ratio was 29:1. Gels were fixed with Alcian Blue (0.5% in 2% acetic acid) and silver-stained.

### Isolation of KPS and LPS

KPS and LPS were isolated with a modified version of the method proposed by Yi and Hackett^42^. About 100 mg of lyophilized bacterial cells, ground in a mortar with a pestle, was suspended in 2 ml TRI Reagent (Sigma-Aldrich). The cell suspension was then incubated at room temperature for 15 minutes, for complete homogenization. We then added 2 ml of chloroform for phase separation. The mixture was then vigorously vortexed and incubated at room temperature for an additional 10 minutes. The resulting mixture was centrifuged at 6000 × *g* for 10 minutes, to separate the aqueous and organic phases, and the aqueous phase was collected. Two additional aqueous extraction steps were carried out, in 1 ml water each. The combined aqueous phase was dried on a rotary evaporator. The extract was purified by size exclusion chromatography (SEC) on a Bio-Gel P-6 (BioRad), with pyridine:acetic acid:water (10:14:986 (v/v/v)) as the eluent. Fractions containing carbohydrates were analyzed by NMR.

### NMR spectroscopy

Samples were deuterium-exchanged several times, by freeze-drying from D_2_O, and then examined in solution (1–5 mg/750 μL) in 99.98% D_2_O. Spectra were recorded at 323 K on a Bruker Avance III 500 MHz spectrometer operating at 500.40 MHz (^1^H) and 125.8 MHz (^13^C). Chemical shifts are given in ppm, using the HDO signal (4.50 ppm at 323 K) (^1^H) as a reference. The 2D heteronuclear one-bond proton-carbon correlation experiment was carried out in the ^1^H-detection mode, by single-quantum coherence (HSQC). A data matrix of 256 × 1000 points was used to digitize a spectral width of 5411 to 5681 in F2 and 20833 Hz in F1. ^13^C decoupling was achieved by the GARP scheme. Squared-sine-bell functions were applied in both dimensions, and zero-filling was used to expand the data to 1000 × 1000 points.

### Isolation of phages

The phage collection was obtained from an alfalfa (*Medicago sativa* L.) field located in the Vega area (Granada, Spain). We incubated 5 g of rhizosphere soil with 5 ml of sterilized MiliQ water for 20 hours at room temperature. Soil particles were removed by centrifugation at 18,000 × *g* for 30 minutes at 20 °C. The aqueous solution was mixed with exponential cultures of *S. meliloti* GR4, in a 1 to 5 ratio, and the mixture was incubated at 30 °C for 48 hours with shaking. Complete cell lysis was performed by adding 10% chloroform and the mixture was vigorously vortexed. Phage lysates were clarified by centrifugation at 4,000 × *g* for 30 minutes at 4 °C. Phage supernatants were added with 150 µl chloroform and stored at 4 °C for at least 8 hours.

For the isolation of individual phages, the infection was detected on solid medium plates. Cultures of *S. meliloti* GR4 in the exponential growth phase (OD_600_ 0.6) were infected with serial dilutions of the initial phage lysates and embedded into 3 ml soft agar (0.1 g/l tryptone, 0.1 g/l bacto-agar, 0.05 g/l NaCl). This mixture was spread on LB plates supplemented with 4 mM CaCl_2_, which were then incubated for 24 hours at 30 °C. Infection plaques of different sizes, appearances and morphologies were obtained. *S. meliloti* GR4 cultures grown to an OD_600_ of 0.6 were further infected with a selection of phage plaques and cultured for an additional 48 hours at 30 °C with orbital shaking. As above, we added 10% chloroform to stop phage infection and obtained phage lysates by centrifugation. This infection process was repeated until the phage preparation was homogeneous. The titer of phage preparations ranged from 2 × 10^−6^ to 1.6 × 10^−9^.

### Phage infection assay

The various isolates of *S. meliloti* were grown to exponential growth phase. We then mixed 100 µl of each culture separately with 3 ml soft agar, which was then spread on LB plates supplemented with 4 mM CaCl_2_. Once the top agar had set, we spotted 5 µl of each homogeneous phage lysate onto the plate. Plates were incubated for 24 hours at 30 °C. Phage infection was assessed by checking for an absence of bacterial growth in the area onto which the phage was spotted.

### Data Availability

Sequence reads are available from NCBI [SRA: SRP059863 and SRP091901].

## Electronic supplementary material

Supplementary Information
