# Supplementary material for: The underlying process of early ecological and genetic differentiation in a facultative mutualistic Sinorhizobium meliloti population
Source: Sci Rep. 2017 Apr 6;7:675. doi: 10.1038/s41598-017-00730-7 (PMC5429615; doi:10.1038/s41598-017-00730-7)
Supplement: Supplementary file 1 — Supplementary Information [file 41598_2017_730_MOESM1_ESM.pdf]

Supplementary information

The underlying process of early ecological and genetic differentiation in a facultative mutualistic *Sinorhizobium meliloti* population

Nicolás Toro[1,]*, Pablo J. Villadas[1], María Dolores Molina-Sánchez[1], Pilar Navarro-Gómez[2], José M. Vinardell[2], Lidia Cuesta-Berrio[3] and Miguel A. Rodríguez-Carvajal[3].

[1]Structure, Dynamics and Function of Rhizobacterial Genomes, Grupo de Ecología Genética de la Rizosfera, Department of Soil Microbiology and Symbiotic Systems, Estación Experimental del Zaidín, Consejo Superior de Investigaciones Científicas, C/ Profesor Albareda 1, 18008 Granada, Spain.

[2]Departamento de Microbiología, Facultad de Biología, Universidad de Sevilla, Avenida de Reina Mercedes 6, 41012 Sevilla, Spain.

[3]Departamento de Química Orgánica, Universidad de Sevilla. C/ Profesor García González, 1. 41012 Sevilla, Spain.

* nicolas.toro@eez.csic.es

Table S1

Figure S1

Table S2

Supplementary Data 1

Supplementary Data 2

Supplementary Data 3

Supplementary Data 4

Supplementary Data 5

Supplementary Data 6

Supplementary Data 7

Supplementary Table S1. SNPs and INDELs in the isolates G6 and 7G9

7G9 Chromosome

Minimum	Maximum	Length	Change	Coverage	Polymorphism Type	Variant Frequency	Amino Acid Change	CDS Position	Codon Change	locus_tag	product	Protein Effect	Variant P-Value (approximate)	CDS Codon Number	CDS Position Within Codon	protein_id
241.553	241.553	1	G -> A	123	SNP (transition)	96.7%							0.0			
241.555	241.555	1	C -> G	123	SNP (transversion)	98.4%							0.0			
241.557	241.557	1	A -> G	121	SNP (transition)	100.0%							0.0			
290.377	290.377	1	C -> G	86	SNP (transversion)	100.0%							6.3E-276			
558.406	558.405	0	AAC	103	Insertion	99.0% -> 100.0%	R -> RL	321	CGG -> CGG,TTG	C770_RS02575	Tellurite resistance prot	Insertion	0.0	107	3	WP_015241023.1
640.603	640.604	2	TC	131	Deletion	100.0%		1.139		C770_RS02920	Restriction endonucleas	Frame Shift	4.0E-184	380	2	WP_015241037.1
657.231	657.231	1	A -> G	93	SNP (transition)	98.9%							1.5E-311			
850.884	850.884	1	C -> T	73	SNP (transition)	95.9%		39	GGC -> GGT	C770_RS03960	patatin	None	6.2E-227	13	3	WP_013844082.1
860.531	860.531	1	T -> C	105	SNP (transition)	100.0%		999	GCT -> GCC	C770_RS04005	tetraacyldisaccharide 4'	None	0.0	333	3	WP_010968838.1
1,193,210	1,193,210	1	(C)5 -> (C)4	86	Deletion (tandem repeat)	100.0%							1.6E-112			
1,193,328	1,193,328	1	(A)4 -> (A)3	93	Deletion (tandem repeat)	100.0%							6.3E-131			
1,242,101	1,242,101	1	T -> C	100	SNP (transition)	99.0%		567	GCT -> GCC	C770_RS05815	branched-chain amino a	None	1.6E-315	189	3	WP_010969084.1
1,242,587	1,242,587	1	C -> G	106	SNP (transversion)	98.1%	G -> R	445	GGC -> CGC	C770_RS05820	molybdopterin converti	Substitution	0.0	149	1	WP_015241124.1
1,585,298	1,585,298	1	G -> T	102	SNP (transversion)	98.0%	A -> S	931	GCC -> TCC	C770_RS07535	outer membrane assem	Substitution	5.1E-317	311	1	WP_015241168.1
1,603,402	1,603,402	1	C -> G	92	SNP (transversion)	97.8%							4.2E-267			
1,696,288	1,696,288	1	A -> G	96	SNP (transition)	99.0%	F -> L	22	TTT -> CTT	C770_RS07970	hypothetical protein	Substitution	9.6E-322	8	1	WP_015241191.1
2,156,798	2,156,798	1	T -> G	99	SNP (transversion)	100.0%							0.0			
2,633,947	2,633,947	1	T -> G	92	SNP (transversion)	100.0%	L -> R	80	CTC -> CGC	C770_RS12380	transcriptional regulato	Substitution	1.6E-313	27	2	WP_015241305.1
2,657,237	2,657,237	1	G -> A	89	SNP (transition)	100.0%	V -> I	19	GTT -> ATT	C770_RS12520	hemin import ATP-bindi	Substitution	1.6E-285	7	1	WP_015241308.1
2,797,450	2,797,450	1	(C)12 -> (C)11	67	Deletion (tandem repeat)	98.5%							2.6E-91			
3,296,058	3,296,058	1	T -> G	131	SNP (transversion)	99.2%	N -> K	3.189	AAT -> AAG	C770_RS15475	hypothetical protein	Substitution	0.0	1.063	3	WP_015241352.1
3,365,771	3,365,771	1	C -> T	90	SNP (transition)	100.0%	A -> T	742	GCC -> ACC	C770_RS15790	sn-glycerol-3-phosphate	Substitution	1.0E-297	248	1	WP_013844902.1
3,530,665	3,530,664	0	CGTCCGGC	118 -> 126	Insertion	97.5% -> 97.6%				C770_RS16605	expR		0.0			
3,591,300	3,591,300	1	A -> G	116	SNP (transition)	99.1%	Y -> C	1.181	TAT -> TGT	C770_RS16805	MFS transporter	Substitution	0.0	394	2	WP_017264986.1

G6 Chromosome

Minimum	Maximum	Length	Change	Coverage	Polymorphism Type	Variant Frequency	Amino Acid Change	CDS Position	Codon Change	locus_tag	product	Protein Effect	Variant P-Value (approximate)	CDS Codon Number	CDS Position Within Codon	protein_id
558.406	558.405	0	AAC	100	Insertion	99.0%	R -> RL	321	CGG -> CGG,TTG	C770_RS02575	Tellurite resistance prot	Insertion	0.0	107	3	WP_015241023.1
758.279	758.278	0	(G)2 -> (G)3	116	Insertion (tandem repeat)	100.0%		654		C770_RS03510	acetoacetyl-CoA synthe	Frame Shift	0.0	218	3	WP_014529049.1
850.884	850.884	1	C -> T	113	SNP (transition)	99.1%		39	GGC -> GGT	C770_RS03960	patatin	None	0.0	13	3	WP_013844082.1
860.531	860.531	1	T -> C	97	SNP (transition)	100.0%		999	GCT -> GCC	C770_RS04005	tetraacyldisaccharide 4'	None	4.0E-311	333	3	WP_010968838.1
1,101,589	1,101,589	1	C -> T	103	SNP (transition)	100.0%	S -> L	1.301	TCG -> TTG	C770_RS05135	electron transfer flavop	Substitution	0.0	434	2	WP_010968999.1
1,193,210	1,193,210	1	(C)5 -> (C)4	74	Deletion (tandem repeat)	100.0%							2.5E-67			
1,193,328	1,193,328	1	(A)4 -> (A)3	77	Deletion (tandem repeat)	100.0%							4.0E-93			
1,242,101	1,242,101	1	T -> C	92	SNP (transition)	100.0%		567	GCT -> GCC	C770_RS05815	branched-chain amino a	None	2.5E-304	189	3	WP_010969084.1
1,242,587	1,242,587	1	C -> G	73	SNP (transversion)	100.0%	G -> R	445	GGC -> CGC	C770_RS05820	molybdopterin converti	Substitution	3.2E-256	149	1	WP_015241124.1
1,696,288	1,696,288	1	A -> G	77	SNP (transition)	100.0%	F -> L	22	TTT -> CTT	C770_RS07970	hypothetical protein	Substitution	1.6E-262	8	1	WP_015241191.1
2,156,798	2,156,798	1	T -> G	81	SNP (transversion)	100.0%							4.0E-276			
2,376,972	2,376,972	1	T -> C	88	SNP (transition)	100.0%		1.497	GCT -> GCC	C770_RS11280	ligand-gated channel	None	1.6E-273	499	3	WP_013844649.1
2,603,354	2,603,354	1	A -> G	89	SNP (transition)	98.9%		515	TAA -> TGA	C770_RS12250	DNA-directed RNA polyı	None	3.5E-289	172	2	WP_010969891.1
2,633,947	2,633,947	1	T -> G	81	SNP (transversion)	100.0%	L -> R	80	CTC -> CGC	C770_RS12380	transcriptional regulato	Substitution	5.0E-268	27	2	WP_015241305.1
2,657,237	2,657,237	1	G -> A	92	SNP (transition)	95.7%	V -> I	19	GTT -> ATT	C770_RS12520	hemin import ATP-bindi	Substitution	1.8E-293	7	1	WP_015241308.1
2,797,450	2,797,450	1	(C)12 -> (C)11	72	Deletion (tandem repeat)	97.2%							2.4E-95			
2,949,099	2,949,099	1	C -> T	107	SNP (transition)	100.0%		93	AAG -> AAA	C770_RS13930	hypothetical protein	None	0.0	31	3	WP_003527526.1
2,976,408	2,976,408	1	C -> A	96	SNP (transversion)	100.0%							0.0			
3,030,279	3,030,279	1	C -> T	121	SNP (transition)	99.2%		633	GGC -> GGT	C770_RS14320	MFS transporter	None	0.0	211	3	WP_003536011.1
3,296,058	3,296,058	1	T -> G	83	SNP (transversion)	98.8%	N -> K	3.189	AAT -> AAG	C770_RS15475	hypothetical protein	Substitution	2.1E-269	1.063	3	WP_015241352.1
3,299,561	3,299,561	1	G -> A	83	SNP (transition)	100.0%	V -> I	448	GTT -> ATT	C770_RS15485	aminoglycoside phosph	Substitution	1.3E-274	150	1	WP_010970345.1
3,462,427	3,462,427	1	G -> A	110	SNP (transition)	100.0%	A -> V	371	GCA -> GTA	C770_RS16280	hypothetical protein	Substitution	0.0	124	2	WP_015241359.1
3,530,665	3,530,664	0	CGTCCGGC	112 -> 114	Insertion	97.3% -> 97.4%				C770_RS16605	expR		0.0			
3,559,782	3,559,782	1	G -> C	112	SNP (transversion)	100.0%		513	GCC -> GCG	C770_RS16690	dehydrogenase	None	0.0	171	3	WP_015445683.1

7G9 pSymA

Minimum	Maximum	Length	Change	Coverage	Polymorphism Type	Variant Frequency	Amino Acid Change	CDS Position	Codon Change	locus_tag	product	Protein Effect	Variant P-Value (approximate)	CDS Codon Number	CDS Position Within Codon	protein_id
24.988	24.999	12	GACGCGGTGAG(	82	Deletion	100.0%	HLTAS -> H	447	CAC,CTC,ACC,GC	C770_RS35580	Nucleotidyltransferase/	Deletion	6.3E-132	149	3	WP_015241749.1
32.129	32.129	1	G -> A	109	SNP (transition)	96.3%		78	GGC -> GGT	C770_RS35615	Short-chain alcohol deh	None	0.0	26	3	WP_015241755.1
251.146	251.146	1	A	71	Deletion	100.0%							3.2E-107			
279.592	279.592	1	G -> A	83	SNP (transition)	100.0%	T -> M	8	ACG -> ATG	C770_RS36750	excisionase	Substitution	1.6E-299	3	2	WP_041169751.1
330.804	330.804	1	A -> T	99	SNP (transversion)	99.0%							2.5E-312			
335.704	335.704	1	T -> A	87	SNP (transversion)	100.0%							4.0E-279			
460.007	460.007	1	T -> G	74	SNP (transversion)	100.0%	E -> D	135	GAA -> GAC	C770_RS37605	transcriptional regulato	Substitution	1.0E-259	45	3	WP_015242117.1
569.644	569.644	1	G -> T	68	SNP (transversion)	100.0%							2.5E-218			
673.800	673.801	2	TC -> AT	77	Substitution	98.7%	D -> I	82	GAT -> ATT	C770_RS38590	TetR family transcriptior	Substitution	4.0E-247	28	1	WP_041169777.1
774.652	774.652	1	T -> C	88	SNP (transition)	100.0%							4.0E-291			
838.468	838.468	1	T -> A	91	SNP (transversion)	100.0%							4.0E-310			
861.442	861.442	1	A -> G	81	SNP (transition)	97.5%	I -> T	407	ATT -> ACT	C770_RS39545	cAMP-binding protein	Substitution	1.0E-273	136	2	WP_015242322.1
1,142,894	1,142,894	1	A -> G	77	SNP (transition)	97.4%		639	GCT -> GCC	C770_RS40830	alcohol dehydrogenase	None	9.2E-215	213	3	WP_014531864.1
1,227,548	1,227,548	1	A -> C	51	SNP (transversion)	100.0%	R -> S	2.199	AGA -> AGC	C770_RS41165	Serine phosphatase Rsb	Substitution	3.2E-179	733	3	WP_015242576.1
1,401,662	1,401,663	2	TT -> A	89	Deletion	97.8%		682		C770_RS42030	cysteine protease	Frame Shift	3.2E-134	228	1	WP_015242711.1

G6 pSymA

Minimum	Maximum	Length	Change	Coverage	Polymorphism Type	Variant Frequency	Amino Acid Change	CDS Position	Codon Change	locus_tag	product	Protein Effect	Variant P-Value (approximate)	CDS Codon Number	CDS Position Within Codon	protein_id
32.129	32.129	1	G -> A	79	SNP (transition)	98.7%		78	GGC -> GGT	C770_RS35615	Short-chain alcohol deh	None	7.9E-272	26	3	WP_015241755.1
279.592	279.592	1	G -> A	97	SNP (transition)	100.0%	T -> M	8	ACG -> ATG	C770_RS36750	excisionase	Substitution	0.0	3	2	WP_041169751.1
335.157	335.157	1	A -> T	91	SNP (transversion)	100.0%							4.0E-310			
335.704	335.704	1	T -> A	67	SNP (transversion)	100.0%							7.9E-222			
569.644	569.644	1	G -> T	74	SNP (transversion)	98.6%							4.7E-247			
707.254	707.254	1	G -> A	85	SNP (transition)	100.0%	C -> Y	1.067	TGT -> TAT	C770_RS38790	nitric oxide reductase	Substitution	3.2E-298	356	2	WP_015242213.1
774.652	774.652	1	T -> C	72	SNP (transition)	100.0%							1.6E-245			
861.442	861.442	1	A -> G	90	SNP (transition)	100.0%	I -> T	407	ATT -> ACT	C770_RS39545	cAMP-binding protein	Substitution	1.0E-306	136	2	WP_015242322.1
1,142,894	1,142,894	1	A -> G	82	SNP (transition)	97.6%		639	GCT -> GCC	C770_RS40830	alcohol dehydrogenase	None	3.3E-245	213	3	WP_014531864.1
1,227,548	1,227,548	1	A -> C	66	SNP (transversion)	100.0%	R -> S	2.199	AGA -> AGC	C770_RS41165	Serine phosphatase Rsb	Substitution	4.0E-225	733	3	WP_015242576.1
1,268,178	1,268,178	1	T -> C	89	SNP (transition)	98.9%	F -> L	658	TTC -> CTC	C770_RS41375	alcohol dehydrogenase	Substitution	2.2E-280	220	1	WP_015242616.1
1,374,862	1,374,862	1	T -> G	86	SNP (transversion)	98.8%		651	GCA -> GCC	C770_RS41900	amine acid ABC transpo	None	2.7E-279	217	3	WP_015242691.1

7G9 pSymB

Minimum	Maximum	Length	Change	Coverage	Polymorphism Type	Variant Frequency	Amino Acid Change	CDS Position	Codon Change	locus_tag	product	Protein Effect	Variant P-Value (approximate)	CDS Codon Number	CDS Position Within Codon	protein_id
28.796	28.795	0	ACC	93 -> 94	Insertion	97.9% -> 98.9%							1.1E-300			
317.920	317.92	1	A -> T	83	SNP (transversion)	97.6%	Q -> H	1.584	CAA -> CAT	C770_RS43515	beta-galactosidase	Substitution	1.7E-264	528	3	WP_015242835.1
497.301	497.301	1	G -> A	92	SNP (transition)	98.9%	R -> C	265	CGT -> TGT	C770_RS44370	hypothetical protein	Substitution	7.3E-281	89	1	WP_015242946.1
748.330	748.331	2	GA -> TC	78 -> 79	Substitution	98.7%	S -> E	1.885	TCG -> GAG	C770_RS45480	glycosyltransferase	Substitution	2.5E-250	629	1	WP_015243054.1
823.182	823.182	1	G -> A	78	SNP (transition)	98.7%	A -> T	151	GCC -> ACC	C770_RS45775	DNA polymerase	Substitution	9.8E-207	51	1	WP_041169873.1
878.512	878.512	1	G	87	Deletion	98.9%		788		C770_RS46000	OHCU decarboxylase	Frame Shift	2.1E-136	263	2	WP_015243103.1
913.819	913.819	1	C -> G	75	SNP (transversion)	98.7%	E -> D	552	GAG -> GAC	C770_RS46145	polysaccharide deacetyl	Substitution	1.9E-250	184	3	WP_015243112.1
959.491	959.491	1	C -> T	81	SNP (transition)	100.0%		705	GCC -> GCT	C770_RS46320	Sugar kinase ribokinase	None	6.3E-260	235	3	WP_015243143.1
1,013,670	1,013,670	1	T -> G	81	SNP (transversion)	98.8%							8.1E-271			
1,022,187	1,022,187	1	G -> A	82	SNP (transition)	100.0%							1.6E-279			
1,059,297	1,059,297	1	A -> G	89	SNP (transition)	100.0%							1.6E-285			
1,078,126	1,078,126	1	T -> C	73	SNP (transition)	97.3%	V -> A	77	GTA -> GCA	C770_RS46845	hypothetical protein	Substitution	1.0E-238	26	2	WP_015243200.1
1,091,946	1,091,946	1	G -> T	99	SNP (transversion)	99.0%	A -> S	337	GCC -> TCC	C770_RS46920	DNA ligase D	Substitution	4.0E-322	113	1	WP_015243213.1
1,091,961	1,091,961	1	T -> G	97	SNP (transversion)	97.9%	L -> V	352	TTG -> GTG	C770_RS46920	DNA ligase D	Substitution	4.7E-301	118	1	WP_015243213.1
1,091,972	1,091,972	1	A -> G	96	SNP (transition)	100.0%		363	GGA -> GGG	C770_RS46920	DNA ligase D	None	6.3E-308	121	3	WP_015243213.1
1,092,005	1,092,005	1	G -> C	88	SNP (transversion)	100.0%		396	CCG -> CCC	C770_RS46920	DNA ligase D	None	2.5E-282	132	3	WP_015243213.1
1,092,670	1,092,670	1	C -> A	71	SNP (transversion)	100.0%	T -> N	1.061	ACT -> AAT	C770_RS46920	DNA ligase D	Substitution	4.0E-242	354	2	WP_015243213.1
1,092,713	1,092,714	2	AG -> GC	69	Substitution	97.1%	LE -> LQ	1.104	TTA,GAG -> TTG,	C770_RS46920	DNA ligase D	Substitution	4.4E-230	368	3	WP_015243213.1
1,092,740	1,092,740	1	C -> T	65	SNP (transition)	100.0%		1.131	CCC -> CCT	C770_RS46920	DNA ligase D	None	3.2E-215	377	3	WP_015243213.1
1,092,743	1,092,743	1	A -> G	64	SNP (transition)	96.9%		1.134	TCA -> TCG	C770_RS46920	DNA ligase D	None	2.0E-214	378	3	WP_015243213.1
1,092,781	1,092,781	1	A -> C	68	SNP (transversion)	100.0%	D -> A	1.172	GAT -> GCT	C770_RS46920	DNA ligase D	Substitution	6.3E-232	391	2	WP_015243213.1
1,092,827	1,092,827	1	A -> G	70	SNP (transition)	100.0%		1.218	AGA -> AGG	C770_RS46920	DNA ligase D	None	1.0E-231	406	3	WP_015243213.1
1,092,836	1,092,836	1	C -> T	71	SNP (transition)	98.6%		1.227	CAC -> CAT	C770_RS46920	DNA ligase D	None	7.1E-251	409	3	WP_015243213.1
1,092,854	1,092,854	1	T -> C	74	SNP (transition)	98.6%		1.245	CAT -> CAC	C770_RS46920	DNA ligase D	None	4.7E-247	415	3	WP_015243213.1
1,092,881	1,092,881	1	C -> A	76	SNP (transversion)	96.1%		1.272	CCC -> CCA	C770_RS46920	DNA ligase D	None	4.4E-244	424	3	WP_015243213.1
1,092,885	1,092,885	1	A -> G	75	SNP (transition)	100.0%	I -> V	1.276	ATC -> GTC	C770_RS46920	DNA ligase D	Substitution	1.0E-240	426	1	WP_015243213.1
1,092,950	1,092,950	1	T -> C	72	SNP (transition)	100.0%		1.341	CTT -> CTC	C770_RS46920	DNA ligase D	None	2.5E-238	447	3	WP_015243213.1
1,092,952	1,092,952	1	C -> G	73	SNP (transversion)	100.0%	A -> G	1.343	GCC -> GGC	C770_RS46920	DNA ligase D	Substitution	2.5E-234	448	2	WP_015243213.1
1,092,971	1,092,971	1	C -> T	70	SNP (transition)	98.6%		1.362	GTC -> GTT	C770_RS46920	DNA ligase D	None	1.1E-219	454	3	WP_015243213.1
1,093,009	1,093,009	1	G -> C	73	SNP (transversion)	100.0%	S -> T	1.4	AGC -> ACC	C770_RS46920	DNA ligase D	Substitution	5.0E-227	467	2	WP_015243213.1
1,093,013	1,093,013	1	A -> C	75	SNP (transversion)	98.7%		1.404	GTA -> GTC	C770_RS46920	DNA ligase D	None	1.2E-235	468	3	WP_015243213.1
1,093,016	1,093,017	2	TG -> CA	77	Substitution	98.7%	DD -> DN	1.407	GAT,GAT -> GAC,	C770_RS46920	DNA ligase D	Substitution	2.0E-239	469	3	WP_015243213.1
1,093,095	1,093,095	1	T -> G	72	SNP (transversion)	100.0%	S -> A	1.486	TCC -> GCC	C770_RS46920	DNA ligase D	Substitution	1.6E-245	496	1	WP_015243213.1
1,093,112	1,093,112	1	A -> G	77	SNP (transition)	100.0%		1.503	GAA -> GAG	C770_RS46920	DNA ligase D	None	7.9E-255	501	3	WP_015243213.1
1,093,139	1,093,139	1	G -> A	79	SNP (transition)	100.0%		1.53	ACG -> ACA	C770_RS46920	DNA ligase D	None	2.0E-261	510	3	WP_015243213.1
1,093,475	1,093,475	1	A -> C	86	SNP (transversion)	100.0%		1.866	GCA -> GCC	C770_RS46920	DNA ligase D	None	1.6E-284	622	3	WP_015243213.1
1,093,512	1,093,512	1	A -> C	83	SNP (transversion)	100.0%							2.5E-266			
1,093,543	1,093,544	2	CC -> GG	85	Substitution	100.0%							1.0E-272			
1,093,547	1,093,547	1	G -> A	85	SNP (transition)	98.8%							1.3E-267			
1,093,549	1,093,549	1	T -> C	85	SNP (transition)	100.0%							3.2E-281			
1,093,563	1,093,565	3	ACT	83	Deletion	100.0%							1.6E-133			
1,093,582	1,093,582	1	C -> G	83	SNP (transversion)	98.8%							2.1E-269			
1,093,588	1,093,588	1	C -> G	82	SNP (transversion)	100.0%							1.6E-279			
1,093,600	1,093,600	1	C -> G	77	SNP (transversion)	100.0%							1.6E-262			
1,093,623	1,093,624	2	TT -> CA	78 -> 79	Substitution	98.7%							6.2E-253			
1,093,649	1,093,649	1	G -> A	80	SNP (transition)	100.0%							1.0E-264			
1,093,662	1,093,662	1	G -> A	80	SNP (transition)	100.0%							1.0E-240			
1,093,718	1,093,718	1	C -> G	84	SNP (transversion)	98.8%							2.1E-264			
1,093,720	1,093,720	1	G -> A	85	SNP (transition)	95.3%							2.0E-237			
1,093,730	1,093,730	1	C -> G	82	SNP (transversion)	100.0%							2.5E-271			
1,093,757	1,093,757	1	G -> A	85	SNP (transition)	98.8%							8.5E-251			
1,093,766	1,093,766	1	A -> G	87	SNP (transition)	98.9%							1.4E-282			
1,093,785	1,093,785	1	A -> C	83	SNP (transversion)	98.8%							3.3E-261			
1,093,788	1,093,788	1	A -> T	82	SNP (transversion)	98.8%							6.5E-250			
1,093,791	1,093,791	1	C -> G	82	SNP (transversion)	100.0%							4.0E-263			
1,093,823	1,093,823	1	G -> C	86	SNP (transversion)	100.0%							2.5E-267			
1,093,863	1,093,864	2	AT -> GG	87 -> 88	Substitution	95.4% -> 95.5%							3.8E-255			
1,093,868	1,093,868	1	G -> T	87	SNP (transversion)	96.6%							4.2E-256			
1,093,881	1,093,881	1	T -> C	87	SNP (transition)	100.0%							4.0E-279			
1,093,883	1,093,883	1	C -> T	88	SNP (transition)	98.9%							3.5E-277			
1,093,894	1,093,894	1	G -> T	90	SNP (transversion)	97.8%							1.6E-287			
1,093,904	1,093,904	1	G -> T	94	SNP (transversion)	98.9%							9.4E-278			
1,093,915	1,093,915	1	C -> T	93	SNP (transition)	97.8%							3.4E-279			
1,093,920	1,093,920	1	A -> T	90	SNP (transversion)	98.9%							2.3E-301			
1,093,923	1,093,923	1	A -> G	89	SNP (transition)	98.9%							3.5E-289			
1,093,964	1,093,964	1	T -> A	92	SNP (transversion)	98.9%							4.6E-299			
1,093,974	1,093,974	1	A -> T	91	SNP (transversion)	98.9%							9.1E-278			
1,093,979	1,093,979	1	T	91	Deletion	100.0%							2.5E-146			
1,093,984	1,093,983	0	AGTG	90 -> 91	Insertion	97.8% -> 98.9%							6.5E-282			
1,093,987	1,093,987	1	C -> G	90	SNP (transversion)	100.0%							1.0E-288			
1,093,994	1,093,994	1	A -> G	89	SNP (transition)	100.0%							1.3E-276			
1,093,998	1,093,998	1	G -> A	92	SNP (transition)	96.7%							2.0E-280			
1,094,060	1,094,060	1	T -> A	85	SNP (transversion)	98.8%							8.5E-251			
1,094,062	1,094,063	2	TG -> GA	84 -> 86	Substitution	98.8% -> 100.0%							2.7E-262			

1,094,084	1,094,084	1	T -> C		88	SNP (transition)	100.0%							4.0E-291			
1,094,090	1,094,089	0	TTCCT	88 -> 90		Insertion	98.9%							1.3E-276			
1,094,102	1,094,103	2	TC -> CT		88	Substitution	98.9%							7.0E-286			
1,094,109	1,094,109	1	C -> T		91	SNP (transition)	100.0%							4.0E-310			
1,094,113	1,094,113	1	T -> C		92	SNP (transition)	98.9%							4.6E-299			
1,094,157	1,094,157	1	A -> C		90	SNP (transversion)	100.0%	L -> W	1.184	TTG -> TGG	C770_RS46925	peptidase T	Substitution	1.0E-288	395	2	WP_015243214.1
1,094,180	1,094,180	1	C -> G		87	SNP (transversion)	100.0%		1.161	CCG -> CCC	C770_RS46925	peptidase T	None	2.0E-270	387	3	WP_015243214.1
1,094,240	1,094,240	1	T -> A		85	SNP (transversion)	100.0%		1.101	ATA -> ATT	C770_RS46925	peptidase T	None	3.2E-264	367	3	WP_015243214.1
1,094,247	1,094,247	1	C -> T		84	SNP (transition)	100.0%	R -> K	1.094	AGA -> AAA	C770_RS46925	peptidase T	Substitution	2.5E-286	365	2	WP_015243214.1
1,094,252	1,094,252	1	T -> G		80	SNP (transversion)	100.0%		1.089	TCA -> TCC	C770_RS46925	peptidase T	None	1.0E-256	363	3	WP_015243214.1
1,094,258	1,094,258	1	A -> G		79	SNP (transition)	100.0%		1.083	GTT -> GTC	C770_RS46925	peptidase T	None	1.6E-253	361	3	WP_015243214.1
1,094,291	1,094,291	1	C -> T		80	SNP (transition)	100.0%		1.05	GTG -> GTA	C770_RS46925	peptidase T	None	1.0E-264	350	3	WP_015243214.1
1,094,300	1,094,301	2	GC -> CT		77	Substitution	100.0%	G -> E	1.04	GGC -> GAG	C770_RS46925	peptidase T	Substitution	4.0E-247	347	2	WP_015243214.1
1,094,303	1,094,303	1	A -> G		78	SNP (transition)	100.0%		1.038	ATT -> ATC	C770_RS46925	peptidase T	None	2.5E-250	346	3	WP_015243214.1
1,094,391	1,094,391	1	G -> A		78	SNP (transition)	97.4%	T -> I	950	ACC -> ATC	C770_RS46925	peptidase T	Substitution	1.2E-255	317	2	WP_015243214.1
1,094,399	1,094,399	1	G -> A		79	SNP (transition)	98.7%		942	TAC -> TAT	C770_RS46925	peptidase T	None	5.0E-264	314	3	WP_015243214.1
1,094,564	1,094,564	1	A -> G		77	SNP (transition)	96.1%		777	CGT -> CGC	C770_RS46925	peptidase T	None	4.6E-240	259	3	WP_015243214.1
1,094,568	1,094,568	1	C -> T		79	SNP (transition)	96.2%	G -> E	773	GGA -> GAA	C770_RS46925	peptidase T	Substitution	1.3E-246	258	2	WP_015243214.1
1,094,809	1,094,810	2	TT -> GC	85 -> 86		Substitution	98.8%	GM -> GL	531	GGA,ATG -> GG	C770_RS46925	peptidase T	Substitution	8.6E-271	177	3	WP_015243214.1
1,094,839	1,094,840	2	TG -> CA		95	Substitution	100.0%	FI -> FV	501	TTC,ATC -> TTT,	C770_RS46925	peptidase T	Substitution	3.2E-295	167	3	WP_015243214.1
1,094,897	1,094,897	1	G -> C		96	SNP (transversion)	99.0%		444	GCC -> GCG	C770_RS46925	peptidase T	None	9.6E-322	148	3	WP_015243214.1
1,094,964	1,094,965	2	AA -> GT		97	Substitution	100.0%	L -> T	376	TTG -> ACG	C770_RS46925	peptidase T	Substitution	0.0	126	1	WP_015243214.1
1,094,998	1,094,999	2	GT -> TG		96	Substitution	99.0%		342	CTA,CGG -> CTC,	C770_RS46925	peptidase T	None	1.6E-317	114	3	WP_015243214.1
1,095,041	1,095,043	2	TTC -> CTT		98	Substitution	99.0%	E -> K	298	GAA -> AAG	C770_RS46925	peptidase T	Substitution	2.0E-299	100	1	WP_015243214.1
1,095,070	1,095,070	1	A -> C		106	SNP (transversion)	99.1%	F -> V	271	TTC -> GTC	C770_RS46925	peptidase T	Substitution	0.0	91	1	WP_015243214.1
1,095,133	1,095,133	1	A -> G		98	SNP (transition)	100.0%	S -> P	208	TCT -> CCT	C770_RS46925	peptidase T	Substitution	1.6E-304	70	1	WP_015243214.1
1,095,149	1,095,151	2	GAC -> TAT	89 -> 90		Substitution	97.8%	V -> I	190	GTC -> ATA	C770_RS46925	peptidase T	Substitution	1.4E-271	64	1	WP_015243214.1
1,095,218	1,095,218	1	G -> A		94	SNP (transition)	98.9%		123	CTC -> CTT	C770_RS46925	peptidase T	None	1.2E-305	41	3	WP_015243214.1
1,095,323	1,095,323	1	T -> C		88	SNP (transition)	100.0%		18	GAA -> GAG	C770_RS46925	peptidase T	None	1.0E-308	6	3	WP_015243214.1
1,095,342	1,095,342	1	C -> G		83	SNP (transversion)	100.0%							6.3E-283			
1,095,393	1,095,393	1	G -> A		81	SNP (transition)	100.0%							4.0E-276			
1,095,402	1,095,402	1	G -> C		83	SNP (transversion)	96.4%							9.2E-252			
1,095,422	1,095,423	2	CA -> TG		88	Substitution	97.7%							7.0E-286			
1,095,436	1,095,436	1	T -> C		90	SNP (transition)	98.9%							1.4E-283			
1,095,462	1,095,462	1	C -> G		93	SNP (transversion)	100.0%							2.5E-298			
1,095,474	1,095,474	1	A -> G		94	SNP (transition)	100.0%		1.233	CTT -> CTC	C770_RS46930	enzyme of poly-gamma-	None	1.6E-301	411	3	WP_015243215.1
1,095,507	1,095,507	1	G -> A		96	SNP (transition)	99.0%		1.2	ATC -> ATT	C770_RS46930	enzyme of poly-gamma-	None	3.0E-293	400	3	WP_015243215.1
1,095,527	1,095,527	1	T -> C		94	SNP (transition)	100.0%	T -> A	1.18	ACC -> GCC	C770_RS46930	enzyme of poly-gamma-	Substitution	1.6E-301	394	1	WP_015243215.1
1,095,539	1,095,539	1	A -> T		96	SNP (transversion)	100.0%	S -> T	1.168	TCA -> ACA	C770_RS46930	enzyme of poly-gamma-	Substitution	0.0	390	1	WP_015243215.1
1,095,732	1,095,732	1	G -> C		90	SNP (transversion)	100.0%		975	ACC -> ACG	C770_RS46930	enzyme of poly-gamma-	None	1.0E-315	325	3	WP_015243215.1
1,095,828	1,095,828	1	A -> C		79	SNP (transversion)	100.0%		879	GGT -> GGG	C770_RS46930	enzyme of poly-gamma-	None	1.6E-253	293	3	WP_015243215.1
1,095,963	1,095,963	1	G -> C		73	SNP (transversion)	100.0%		744	GCC -> GCG	C770_RS46930	enzyme of poly-gamma-	None	6.3E-249	248	3	WP_015243215.1
1,095,966	1,095,967	2	CT -> GG	73 -> 74		Substitution	100.0%	E -> A	740	GAG -> GCC	C770_RS46930	enzyme of poly-gamma-	Substitution	1.3E-241	247	2	WP_015243215.1
1,095,977	1,095,977	1	T -> C		75	SNP (transition)	98.7%	I -> V	730	ATC -> GTC	C770_RS46930	enzyme of poly-gamma-	Substitution	1.2E-235	244	1	WP_015243215.1
1,096,005	1,096,005	1	C -> T		74	SNP (transition)	98.6%		702	GAG -> GAA	C770_RS46930	enzyme of poly-gamma-	None	1.9E-232	234	3	WP_015243215.1
1,096,015	1,096,015	1	T -> C		75	SNP (transition)	100.0%	K -> R	692	AAG -> AGG	C770_RS46930	enzyme of poly-gamma-	Substitution	3.2E-233	231	2	WP_015243215.1
1,096,032	1,096,038	7	TTTCCCC -> CCG	75 -> 77		Substitution	100.0%	SGK -> SNR	669	TCG,GGG,AAA ->	C770_RS46930	enzyme of poly-gamma-	Substitution	3.2E-233	223	3	WP_015243215.1
1,096,050	1,096,050	1	G -> C		73	SNP (transversion)	98.6%		657	GTC -> GTG	C770_RS46930	enzyme of poly-gamma-	None	1.2E-243	219	3	WP_015243215.1
1,096,053	1,096,053	1	C -> G		71	SNP (transversion)	100.0%		654	GCG -> GCC	C770_RS46930	enzyme of poly-gamma-	None	6.3E-228	218	3	WP_015243215.1
1,096,056	1,096,056	1	A -> C		69	SNP (transversion)	100.0%		651	CGT -> CGG	C770_RS46930	enzyme of poly-gamma-	None	2.0E-228	217	3	WP_015243215.1
1,096,061	1,096,061	1	A -> C		70	SNP (transversion)	100.0%	S -> A	646	TCG -> GCG	C770_RS46930	enzyme of poly-gamma-	Substitution	1.0E-231	216	1	WP_015243215.1
1,096,070	1,096,070	1	C -> T		66	SNP (transition)	97.0%	V -> I	637	GTC -> ATC	C770_RS46930	enzyme of poly-gamma-	Substitution	1.4E-208	213	1	WP_015243215.1
1,096,075	1,096,077	3	GAT -> TGA	66 -> 67		Substitution	97.0%	ES -> DH	630	GAA,TCC -> GAT,	C770_RS46930	enzyme of poly-gamma-	Substitution	2.0E-208	210	3	WP_015243215.1
1,096,082	1,096,083	2	CG -> GC	66 -> 67		Substitution	97.0% -> 98.5%	AA -> AP	624	GCC,GCG -> GCG	C770_RS46930	enzyme of poly-gamma-	Substitution	7.0E-212	208	3	WP_015243215.1
1,096,086	1,096,087	2	GA -> AG		67	Substitution	98.5%	L -> P	620	CTC -> CCT	C770_RS46930	enzyme of poly-gamma-	Substitution	4.0E-215	207	2	WP_015243215.1
1,096,090	1,096,090	1	C -> T		68	SNP (transition)	97.1%	R -> H	617	CGC -> CAC	C770_RS46930	enzyme of poly-gamma-	Substitution	3.6E-215	206	2	WP_015243215.1
1,096,093	1,096,093	1	G -> C		67	SNP (transversion)	100.0%	P -> R	614	CCG -> CGG	C770_RS46930	enzyme of poly-gamma-	Substitution	4.0E-215	205	2	WP_015243215.1
1,096,095	1,096,095	1	C -> T		67	SNP (transition)	98.5%		612	CCG -> CCA	C770_RS46930	enzyme of poly-gamma-	None	2.7E-223	204	3	WP_015243215.1
1,096,110	1,096,110	1	A -> G		69	SNP (transition)	98.6%		597	AGT -> AGC	C770_RS46930	enzyme of poly-gamma-	None	4.4E-230	199	3	WP_015243215.1
1,096,183	1,096,183	1	C -> G		70	SNP (transversion)	100.0%	G -> A	524	GGA -> GCA	C770_RS46930	enzyme of poly-gamma-	Substitution	1.0E-224	175	2	WP_015243215.1
1,096,260	1,096,260	1	A -> G		78	SNP (transition)	98.7%		447	GCT -> GCC	C770_RS46930	enzyme of poly-gamma-	None	1.6E-237	149	3	WP_015243215.1
1,096,380	1,096,380	1	A -> C		84	SNP (transversion)	98.8%		327	CTT -> CTG	C770_RS46930	enzyme of poly-gamma-	None	5.3E-281	109	3	WP_015243215.1
1,096,593	1,096,593	1	G -> A		94	SNP (transition)	100.0%		114	GAC -> GAT	C770_RS46930	enzyme of poly-gamma-	None	6.3E-311	38	3	WP_015243215.1
1,096,596	1,096,596	1	G -> C		93	SNP (transversion)	98.9%		111	GCC -> GCG	C770_RS46930	enzyme of poly-gamma-	None	5.9E-284	37	3	WP_015243215.1
1,096,599	1,096,599	1	A -> C		95	SNP (transversion)	97.9%	H -> Q	108	CAT -> CAG	C770_RS46930	enzyme of poly-gamma-	Substitution	1.1E-294	36	3	WP_015243215.1
1,096,721	1,096,721	1	A -> G		92	SNP (transition)	100.0%							2.5E-304			
1,096,723	1,096,723	1	A -> G		93	SNP (transition)	98.9%							2.3E-302			
1,096,725	1,096,725	1	C -> T		93	SNP (transition)	100.0%							5.0E-289			
1,096,746	1,096,746	1	T -> A		94	SNP (transversion)	100.0%							4.0E-292			
1,096,767	1,096,767	1	C -> G		94	SNP (transversion)	100.0%							0.0			
1,096,792	1,096,792	1	C -> A		92	SNP (transversion)	100.0%							6.3E-286			
1,096,943	1,096,943	1	G -> C		88	SNP (transversion)	100.0%		1.524	CTC -> CTG	C770_RS46935	ABC transporter ATPase	None	6.3E-256	508	3	WP_015243216.1
1,096,949	1,096,949	1	G -> A		86	SNP (transition)	100.0%		1.518	CAC -> CAT	C770_RS46935	ABC transporter ATPase	None	1.6E-284	506	3	WP_015243216.1
1,096,952	1,096,952	1	C -> G		87	SNP (transversion)	98.9%		1.515	TCG -> TCC	C770_RS46935	ABC transporter ATPase	None	5.5E-274	505	3	WP_015243216.1
1,096,982	1,096,982	1	C -> G		89	SNP (transversion)	100.0%		1.485	GCG -> GCC	C770_RS46935	ABC transporter ATPase	None	1.6E-285	495	3	WP_015243216.1
1,097,003	1,097,005	3	TTG -> ACC	89 -> 90		Substitution	98.9%	Q -> G	1.462	CAA -> GGT	C770_RS46935	ABC transporter ATPase	Substitution	8.9E-263	488	1	WP_015243216.1
1,097,012	1,097,012	1	T -> C		85	SNP (transition)	97.6%		1.455	GAA -> GAG	C770_RS46935	ABC transporter ATPase	None	4.5E-271	485	3	WP_015243216.1
1,097,149	1,097,149	1	A -> G		81	SNP (transition)	98.8%		1.318	TTG -> CTG	C770_RS46935	ABC transporter ATPase	None	8.1E-271	440	1	WP_015243216.1
1,097,180	1,097,181	2	CA -> TG		89	Substitution	100.0%	V -> A	1.286	GTG -> GCA	C770_RS46935	ABC transporter ATPase	Substitution	2.0E-294	429	2	WP_015243216.1
1,097,183	1,097,183	1	C -> G		89	SNP (transversion)	98.9%		1.284	GCG -> GCC	C770_RS46935	ABC transporter ATPase	None	3.5E-289	428	3	WP_015243216.1
1,097,188	1,097,189	2	CT -> GG	87 -> 88		Substitution	97.7%	EV -> DL	1.278	GAA,GTG -> GAC	C770_RS46935	ABC transporter ATPase	Substitution	7.9E-288	426	3	WP_015243216.1
1,097,203	1,097,203	1	C -> T		88	SNP (transition)	100.0%	E -> K	1.264	GAA -> AAA	C770_RS46935	ABC transporter ATPase	Substitution	1.6E-273	422	1	WP_015243216.1
1,097,210	1,097,212	2	ACG -> GCC	88 -> 89		Substitution	98.9%	R -> G	1.255	CGT -> GGC	C770_RS46935	ABC transporter ATPase	Substitution	1.6E-273	419	1	WP_015243216.1
1,097,214	1,097,214	1	A -> G		89	SNP (transition)	98.9%	I -> T	1.253	ATC -> ACC	C770_RS46935	ABC transporter ATPase	Substitution	3.5E-289	418	2	WP_015243216.1
1,097,231	1,097,231	1	A -> G		93	SNP (transition)	100.0%		1.236	ATT -> ATC	C770_RS46935	ABC transporter ATPase	None	5.0E-289	412	3	WP_015243216.1
1,097,240	1,097,240	1	A -> G		90	SNP (transition)	100.0%		1.227	GAT -> GAC	C770_RS46935	ABC transporter ATPase	None	1.0E-297	409	3	WP_015243216.1
1,097,264	1,097,264	1	G -> T		87	SNP (transversion)	97.7%		1.203	CGC -> CGA	C770_RS46935	ABC transporter ATPase	None	3.7E-252	401	3	WP_015243216.1
1,097,369	1,097,369	1	T -> C		80	SNP (transition)	100.0%		1.098	ACA -> ACG	C770_RS46935	ABC transporter ATPase	None	1.0E-256	366	3	WP_015243216.1
1,097,393	1,097,393	1	G -> A		74	SNP (transition)	100.0%		1.074	GAC -> GAT	C770_RS46935	ABC transporter ATPase	None	6.3E-245	358	3	WP_015243216.1
1,097,483	1,097,483	1	G -> A		68	SNP (transition)	100.0%		984	GGC -> GGT	C770_RS46935	ABC transporter ATPase	None	6.3E-232	328	3	WP_015243216.1
1,097,504	1,097,505	2	TC -> CT		70	Substitution	100.0%	R -> K	962	AGA -> AAG	C770_RS46935	ABC transporter ATPase	Substitution	1.0E-231	321	2	WP_015243216.1
1,097,515	1,097,515	1	C -> T		67	SNP (transition)	95.5%	G -> S	952	GGC -> AGC	C770_RS46935	ABC transporter ATPase	Substitution	3.0E-207	318	1	WP_015243216.1
1,097,523	1,097,525	3	ATA -> GCC	68 -> 69		Substitution	98.5% -> 98.6%	GI -> GA	942	GGT,ATC -> GGG	C770_RS46935	ABC transporter ATPase	Substitution	1.6E-221	314	3	WP_015243216.1
1,097,563	1,097,566	3	CCCT -> GGCC	68 -> 69		Substitution	98.5% -> 98.6%	RV -> GL	901	AGG,GTT -> GGC	C770_RS46935	ABC transporter ATPase	Substitution	1.6E-228	301	1	WP_015243216.1
1,097,601	1,097,602	2	GT -> AG		70	Substitution	98.6%	T -> L	865	ACA -> CTA	C770_RS46935	ABC transporter ATPase	Substitution	1.0E-231	289	1	WP_015243216.1
1,097,605	1,097,605	1	C -> T		71	SNP (transition)	98.6%	A -> T	862	GCC -> ACC	C770_RS46935	ABC transporter ATPase	Substitution	7.1E-237	288	1	WP_015243216.1
1,097,608	1,097,608	1	G -> A		72	SNP (transition)	98.6%	P -> S	859	CCC -> TCC	C770_RS46935	ABC transporter ATPase	Substitution	3.6E-233	287	1	WP_015243216.1
1,097,613	1,097,615	3	TCC -> CAT		72	Substitution	98.6%	AD -> AC	852	GCG,GAC -> GCA	C770_RS46935	ABC transporter ATPase	Substitution	2.3E-247	284	3	WP_015243216.1
1,097,621	1,097,623	2	ACG -> GCC	74 -> 76		Substitution	97.3% -> 97.4%	R -> G	844	CGT -> GGC	C770_RS46935	ABC transporter ATPase	Substitution	1.1E-227	282	1	WP_015243216.1
1,097,660	1,097,660	1	C -> G		71	SNP (transversion)	98.6%		807	CGG -> CGC	C770_RS46935	ABC transporter ATPase	None	7.1E-237	269	3	WP_015243216.1
1,097,690	1,097,690	1	C -> T		76	SNP (transition)	98.7%		777	AAG -> AAA	C770_RS46935	ABC transporter ATPase	None	2.4E-246	259	3	WP_015243216.1
1,097,707	1,097,707	1	C -> G		76	SNP (transversion)	98.7%	G -> R	760	GGC -> CGC	C770_RS46935	ABC transporter ATPase	Substitution	7.6E-239	254	1	WP_015243216.1
1,097,735	1,097,735	1	A -> G		72	SNP (transition)	98.6%		732	GAT -> GAC	C770_RS46935	ABC transporter ATPase	None	9.1E-205	244	3	WP_015243216.1
1,097,741	1,097,741	1	C -> T		70	SNP (transition)	98.6%		726	GAG -> GAA	C770_RS46935	ABC transporter ATPase	None	7.0E-206	242	3	WP_015243216.1
1,097,900	1,097,900	1	C -> G		78	SNP (transversion)	96.2%		567	ACG -> ACC	C770_RS46935	ABC transporter ATPase	None	2.4E-243	189	3	WP_015243216.1
1,098,014	1,098,014	1	G -> C		93	SNP (transversion)	97.8%		453	CCC -> CCG	C770_RS46935	ABC transporter ATPase	None	2.1E-297	151	3	WP_015243216.1
1,098,245	1,098,245	1	C -> T		101	SNP (transition)	99.0%		222	ACG -> ACA	C770_RS46935	ABC transporter ATPase	None	1.0E-318	74	3	WP_015243216.1
1,098,254	1,098,254	1	T -> G		103	SNP (transversion)	98.1%		213	ACA -> ACC	C770_RS46935	ABC transporter ATPase	None	0.0	71	3	WP_015243216.1
1,098,257	1,098,257	1	T -> C		101	SNP (transition)	99.0%		210	CAA -> CAG	C770_RS46935	ABC transporter ATPase	None	0.0	70	3	WP_015243216.1
1,098,293	1,098,293	1	G -> A		97	SNP (transition)	99.0%		174	AAC -> AAT	C770_RS46935	ABC transporter ATPase	None	0.0	58	3	WP_015243216.1
1,098,613	1,098,613	1	C -> T		87	SNP (transition)	100.0%		726	TCG -> TCA	C770_RS46940	peptide ABC transporte	None	7.9E-288	242	3	WP_015243217.1
1,098,859	1,098,859	1	A -> C		82	SNP (transversion)	100.0%		480	GCT -> GCG	C770_RS46940	peptide ABC transporte	None	1.0E-287	160	3	WP_015243217.1
1,099,312	1,099,312	1	A -> C		74	SNP (transversion)	100.0%		27	CTT -> CTG	C770_RS46940	peptide ABC transporte	None	2.5E-252	9	3	WP_015243217.1
1,099,327	1,099,327	1	G -> T		71	SNP (transversion)	100.0%	H -> Q	12	CAC -> CAA	C770_RS46940	peptide ABC transporte	Substitution	5.0E-235	4	3	WP_015243217.1
1,099,691	1,099,691	1	C -> T		79	SNP (transition)	98.7%		639	GGG -> GGA	C770_RS46945	ABC transporter perme	None	3.1E-256	213	3	WP_010975490.1
1,099,802	1,099,802	1	C -> G		81	SNP (transversion)	100.0%		528	CTG -> CTC	C770_RS46945	ABC transporter perme	None	5.0E-268	176	3	WP_010975490.1
1,099,819	1,099,819	1	G -> A		84	SNP (transition)	98.8%		511	CTG -> TTG	C770_RS46945	ABC transporter perme	None	2.1E-264	171	1	WP_010975490.1
1,099,829	1,099,829	1	G -> A		78	SNP (transition)	97.4%		501	GGC -> GGT	C770_RS46945	ABC transporter perme	None	4.8E-248	167	3	WP_010975490.1
1,099,930	1,099,930	1	T -> G		88	SNP (transversion)	100.0%	M -> L	400	ATG -> CTG	C770_RS46945	ABC transporter perme	Substitution	1.6E-273	134	1	WP_010975490.1
1,099,982	1,099,982	1	C -> G		83	SNP (transversion)	100.0%		348	ACG -> ACC	C770_RS46945	ABC transporter perme	None	1.3E-274	116	3	WP_010975490.1
1,100,183	1,100,183	1	G -> A		82	SNP (transition)	98.8%		147	GAC -> GAT	C770_RS46945	ABC transporter perme	None	3.3E-274	49	3	WP_010975490.1
1,100,243	1,100,243	1	G -> A		77	SNP (transition)	100.0%		87	ATC -> ATT	C770_RS46945	ABC transporter perme	None	1.6E-262	29	3	WP_010975490.1
1,100,283	1,100,283	1	A -> G		81	SNP (transition)	98.8%	L -> P	47	CTG -> CCG	C770_RS46945	ABC transporter perme	Substitution	8.1E-255	16	2	WP_010975490.1
1,100,352	1,100,352	1	C -> A		87	SNP (transversion)	97.7%	R -> L	1.58	CGT -> CTT	C770_RS46950	peptide ABC transporte	Substitution	1.2E-260	527	2	WP_015243218.1
1,100,495	1,100,495	1	G -> C		96	SNP (transversion)	100.0%		1.437	GCC -> GCG	C770_RS46950	peptide ABC transporte	None	0.0	479	3	WP_015243218.1
1,100,581	1,100,581	1	A -> G		107	SNP (transition)	99.1%		1.351	TTG -> CTG	C770_RS46950	peptide ABC transporte	None	0.0	451	1	WP_015243218.1
1,100,592	1,100,592	1	T -> C		113	SNP (transition)	100.0%	E -> G	1.34	GAG -> GGG	C770_RS46950	peptide ABC transporte	Substitution	0.0	447	2	WP_015243218.1
1,100,623	1,100,623	1	T -> A		114	SNP (transversion)	95.6%	T -> S	1.309	ACT -> TCT	C770_RS46950	peptide ABC transporte	Substitution	0.0	437	1	WP_015243218.1
1,100,681	1,100,681	1	C -> G		111	SNP (transversion)	98.2%		1.251	GGG -> GGC	C770_RS46950	peptide ABC transporte	None	0.0	417	3	WP_015243218.1
1,100,684	1,100,684	1	T -> G		111	SNP (transversion)	100.0%		1.248	GCA -> GCC	C770_RS46950	peptide ABC transporte	None	0.0	416	3	WP_015243218.1
1,100,705	1,100,705	1	G -> T		104	SNP (transversion)	99.0%		1.227	GTC -> GTA	C770_RS46950	peptide ABC transporte	None	0.0	409	3	WP_015243218.1
1,100,723	1,100,723	1	C -> G		104	SNP (transversion)	100.0%		1.209	GCG -> GCC	C770_RS46950	peptide ABC transporte	None	0.0	403	3	WP_015243218.1
1,100,759	1,100,759	1	C -> T		100	SNP (transition)	99.0%		1.173	CAG -> CAA	C770_RS46950	peptide ABC transporte	None	0.0	391	3	WP_015243218.1
1,100,798	1,100,798	1	A -> C		107	SNP (transversion)	100.0%		1.134	CGT -> CGG	C770_RS46950	peptide ABC transporte	None	0.0	378	3	WP_015243218.1
1,100,889	1,100,889	1	T -> C		93	SNP (transition)	100.0%	E -> G	1.043	GAG -> GGG	C770_RS46950	peptide ABC transporte	Substitution	1.0E-279	348	2	WP_015243218.1
1,100,939	1,100,939	1	G -> A		77	SNP (transition)	97.4%		993	GCC -> GCT	C770_RS46950	peptide ABC transporte	None	9.2E-230	331	3	WP_015243218.1
1,100,957	1,100,957	1	A -> G		75	SNP (transition)	100.0%		975	GGT -> GGC	C770_RS46950	peptide ABC transporte	None	1.0E-255	325	3	WP_015243218.1
1,101,059	1,101,059	1	C -> T		88	SNP (transition)	100.0%		873	AAG -> AAA	C770_RS46950	peptide ABC transporte	None	4.0E-291	291	3	WP_015243218.1
1,101,063	1,101,063	1	G -> T		86	SNP (transversion)	100.0%	A -> D	869	GCC -> GAC	C770_RS46950	peptide ABC transporte	Substitution	1.0E-301	290	2	WP_015243218.1
1,101,086	1,101,086	1	C -> T		82	SNP (transition)	100.0%		846	CGG -> CGA	C770_RS46950	peptide ABC transporte	None	1.6E-279	282	3	WP_015243218.1
1,101,137	1,101,137	1	G -> A		83	SNP (transition)	97.6%		795	GTC -> GTT	C770_RS46950	peptide ABC transporte	None	1.7E-264	265	3	WP_015243218.1
1,101,173	1,101,173	1	G -> A		86	SNP (transition)	100.0%		759	CGC -> CGT	C770_RS46950	peptide ABC transporte	None	6.3E-276	253	3	WP_015243218.1
1,101,308	1,101,308	1	C -> A		111	SNP (transversion)	100.0%		624	CTG -> CTT	C770_RS46950	peptide ABC transporte	None	0.0	208	3	WP_015243218.1
1,101,311	1,101,311	1	C -> T		111	SNP (transition)	100.0%		621	AGG -> AGA	C770_RS46950	peptide ABC transporte	None	0.0	207	3	WP_015243218.1
1,101,365	1,101,365	1	G -> C		107	SNP (transversion)	100.0%		567	CCC -> CCG	C770_RS46950	peptide ABC transporte	None	0.0	189	3	WP_015243218.1
1,101,371	1,101,371	1	A -> G		106	SNP (transition)	100.0%		561	CAT -> CAC	C770_RS46950	peptide ABC transporte	None	0.0	187	3	WP_015243218.1
1,101,374	1,101,374	1	C -> A		106	SNP (transversion)	98.1%		558	GGG -> GGT	C770_RS46950	peptide ABC transporte	None	0.0	186	3	WP_015243218.1
1,101,412	1,101,412	1	A -> C		106	SNP (transversion)	100.0%	S -> A	520	TCT -> GCT	C770_RS46950	peptide ABC transporte	Substitution	0.0	174	1	WP_015243218.1
1,101,418	1,101,418	1	G -> C		104	SNP (transversion)	99.0%	R -> G	514	CGG -> GGG	C770_RS46950	peptide ABC transporte	Substitution	0.0	172	1	WP_015243218.1
1,101,433	1,101,433	1	T -> G		105	SNP (transversion)	100.0%	I -> L	499	ATC -> CTC	C770_RS46950	peptide ABC transporte	Substitution	0.0	167	1	WP_015243218.1
1,101,467	1,101,468	2	GC -> CG	108 -> 109		Substitution	98.1% -> 98.2%	G -> A	464	GGC -> GCG	C770_RS46950	peptide ABC transporte	Substitution	0.0	155	2	WP_015243218.1
1,101,482	1,101,482	1	C -> G		110	SNP (transversion)	99.1%		450	CTG -> CTC	C770_RS46950	peptide ABC transporte	None	0.0	150	3	WP_015243218.1
1,101,524	1,101,524	1	G -> A		112	SNP (transition)	100.0%		408	AGC -> AGT	C770_RS46950	peptide ABC transporte	None	0.0	136	3	WP_015243218.1
1,101,589	1,101,589	1	A -> G		94	SNP (transition)	100.0%	Y -> H	343	TAC -> CAC	C770_RS46950	peptide ABC transporte	Substitution	6.3E-311	115	1	WP_015243218.1
1,101,599	1,101,599	1	G -> A		90	SNP (transition)	97.8%		333	GAC -> GAT	C770_RS46950	peptide ABC transporte	None	2.5E-296	111	3	WP_015243218.1
1,101,710	1,101,710	1	T -> G		98	SNP (transversion)	100.0%		222	CTA -> CTC	C770_RS46950	peptide ABC transporte	None	0.0	74	3	WP_015243218.1
1,101,716	1,101,716	1	G -> C		96	SNP (transversion)	99.0%	D -> E	216	GAC -> GAG	C770_RS46950	peptide ABC transporte	Substitution	0.0	72	3	WP_015243218.1
1,101,764	1,101,764	1	G -> A		91	SNP (transition)	100.0%		168	AAC -> AAT	C770_RS46950	peptide ABC transporte	None	3.2E-319	56	3	WP_015243218.1
1,101,770	1,101,770	1	G -> A		88	SNP (transition)	100.0%		162	AAC -> AAT	C770_RS46950	peptide ABC transporte	None	6.3E-300	54	3	WP_015243218.1
1,101,794	1,101,794	1	C -> G		88	SNP (transversion)	100.0%		138	CTG -> CTC	C770_RS46950	peptide ABC transporte	None	6.3E-300	46	3	WP_015243218.1

1,101,812	1,101,812	1	C -> G		92	SNP (transversion)	100.0%		120	TCG -> TCC	C770_RS46950	peptide ABC transporte	None	2.5E-304	40	3	WP_015243218.1
1,101,815	1,101,815	1	T -> C		92	SNP (transition)	100.0%		117	ACA -> ACG	C770_RS46950	peptide ABC transporte	None	1.6E-313	39	3	WP_015243218.1
1,101,848	1,101,849	2	CG -> AA	84 -> 85		Substitution	97.6%	P -> L	83	CCG -> CTT	C770_RS46950	peptide ABC transporte	Substitution	6.3E-278	28	2	WP_015243218.1
1,101,883	1,101,883	1	A -> T		75	SNP (transversion)	100.0%	F -> I	49	TTC -> ATC	C770_RS46950	peptide ABC transporte	Substitution	3.2E-248	17	1	WP_015243218.1
1,101,934	1,101,933	0	(T)3 -> (T)4		61	Insertion (tandem repeat)	98.4%							6.1E-203			
1,101,949	1,101,948	0	(T)4 -> (T)5		55	Insertion (tandem repeat)	100.0%							3.2E-182			
1,101,955	1,101,955	1	(T)5 -> (T)4		52	Deletion (tandem repeat)	100.0%							1.0E-78			
1,101,966	1,101,966	1	G -> A		50	SNP (transition)	100.0%							1.0E-160			
1,101,975	1,101,975	1	A -> C		48	SNP (transversion)	100.0%							2.5E-154			
1,101,981	1,101,981	1	A		43	Deletion	100.0%							1.6E-69			
1,101,987	1,101,987	1	T		41	Deletion	100.0%							2.5E-66			
1,102,028	1,102,030	3	CGG -> TT		36	Deletion	97.2%							1.0E-54			
1,102,038	1,102,038	1	A -> C		33	SNP (transversion)	100.0%							2.5E-106			
1,102,041	1,102,041	1	T -> A		33	SNP (transversion)	100.0%							5.0E-103			
1,102,045	1,102,045	1	T -> G		33	SNP (transversion)	100.0%							5.0E-103			
1,102,047	1,102,047	1	G -> A		30	SNP (transition)	96.7%							4.8E-92			
1,102,049	1,102,049	1	T -> A		30	SNP (transversion)	96.7%							6.0E-95			
1,102,052	1,102,053	2	GC -> AG		27	Substitution	100.0%							2.0E-84			
1,102,058	1,102,058	1	C -> G		25	SNP (transversion)	96.0%							9.9E-74			
1,102,060	1,102,060	1	C -> G		25	SNP (transversion)	96.0%							4.0E-76			
1,102,062	1,102,062	1	G -> C		24	SNP (transversion)	100.0%							1.0E-72			
1,102,065	1,102,065	1	G -> A		21	SNP (transition)	100.0%							4.0E-72			
1,102,069	1,102,069	1	A -> C		21	SNP (transversion)	95.2%							2.1E-65			
1,102,072	1,102,072	1	T -> C		20	SNP (transition)	100.0%							1.0E-62			
1,102,075	1,102,075	1	C -> A		20	SNP (transversion)	100.0%							1.0E-62			
1,102,078	1,102,082	5	GGTGA -> CTGC(	17 -> 19		Substitution	100.0%							4.0E-55			
1,102,086	1,102,086	1	A -> G		15	SNP (transition)	100.0%							1.0E-48			
1,102,088	1,102,089	2	TC		14	Deletion	100.0%							4.0E-23			
1,102,092	1,102,092	1	A -> G		13	SNP (transition)	100.0%							3.2E-46			
1,102,097	1,102,098	2	AT -> GC		12	Substitution	100.0%	D -> G	545	GAT -> GGC	C770_RS46955	hypothetical protein	Substitution	1.0E-42	182	2	WP_015243219.1
1,102,102	1,102,102	1	C -> T		11	SNP (transition)	100.0%	G -> S	541	GGC -> AGC	C770_RS46955	hypothetical protein	Substitution	3.2E-39	181	1	WP_015243219.1
1,102,108	1,102,108	1	G -> A		7	SNP (transition)	100.0%	L -> F	535	CTT -> TTT	C770_RS46955	hypothetical protein	Substitution	3.2E-25	179	1	WP_015243219.1
1,102,114	1,102,114	1	A -> G		6	SNP (transition)	100.0%	S -> P	529	TCC -> CCC	C770_RS46955	hypothetical protein	Substitution	4.0E-21	177	1	WP_015243219.1
1,103,751	1,103,752	2	CC -> GT	6 -> 7		Substitution	100.0%							2.5E-22			
1,103,754	1,103,755	2	CG -> TA		7	Substitution	100.0%							7.9E-24			
1,103,757	1,103,758	2	TC -> CA		8	Substitution	100.0%							1.0E-28			
1,103,761	1,103,761	1	C -> T		9	SNP (transition)	100.0%							2.5E-31			
1,103,763	1,103,762	0	G		10	Insertion	100.0%							1.0E-32			
1,103,768	1,103,769	2	CA -> GT		11	Substitution	100.0%							7.9E-35			
1,103,771	1,103,771	1	A -> C		11	SNP (transversion)	100.0%							3.2E-39			
1,103,788	1,103,787	0	T		15	Insertion	100.0%							1.0E-51			
1,103,801	1,103,801	1	T -> C		17	SNP (transition)	100.0%							4.0E-55			
1,103,824	1,103,824	1	G -> A		24	SNP (transition)	95.8%							6.0E-73			
1,103,830	1,103,829	0	G		25	Insertion	100.0%							3.2E-73			
1,103,860	1,103,860	1	(A)3 -> (A)2		35	Deletion (tandem repeat)	100.0%							1.0E-49			
1,103,866	1,103,866	1	T -> C		35	SNP (transition)	100.0%							3.2E-109			
1,103,871	1,103,871	1	A -> G		36	SNP (transition)	100.0%							6.3E-116			
1,103,889	1,103,889	1	T -> A		41	SNP (transversion)	100.0%							4.0E-140			
1,103,909	1,103,909	1	G		43	Deletion	100.0%							1.6E-69			
1,103,945	1,103,945	1	A -> G		54	SNP (transition)	96.3%							2.3E-174			
1,103,950	1,103,950	1	C -> T		55	SNP (transition)	98.2%							3.5E-177			
1,103,997	1,103,997	1	G -> C		66	SNP (transversion)	100.0%							4.0E-192			
1,104,008	1,104,008	1	A -> T		68	SNP (transversion)	100.0%							4.0E-225			
1,104,021	1,104,021	1	A -> T		74	SNP (transversion)	98.6%							7.4E-218			
1,104,057	1,104,057	1	C -> A		77	SNP (transversion)	98.7%							4.9E-242			
1,104,064	1,104,064	1	A -> G		77	SNP (transition)	98.7%							4.9E-242			
1,104,066	1,104,066	1	A -> G		77	SNP (transition)	100.0%							2.0E-239			
1,104,085	1,104,085	1	A -> G		75	SNP (transition)	100.0%							1.0E-240			
1,104,087	1,104,087	1	C		75	Deletion	100.0%							3.2E-113			
1,104,097	1,104,098	2	TT -> GG		75	Substitution	100.0%							1.0E-240			
1,104,105	1,104,106	2	CT -> TC		72	Substitution	97.2%							4.5E-226			
1,104,120	1,104,120	1	C -> T		75	SNP (transition)	100.0%							1.0E-240			
1,104,128	1,104,128	1	G -> A		73	SNP (transition)	100.0%							2.5E-234			
1,104,147	1,104,147	1	G -> A		68	SNP (transition)	98.5%							2.7E-213			
1,104,157	1,104,158	2	AG -> GA		69	Substitution	100.0%							1.3E-214			
1,104,163	1,104,163	1	(G)3 -> (G)2		68	Deletion (tandem repeat)	100.0%							6.3E-96			
1,104,182	1,104,182	1	G -> T		71	SNP (transversion)	100.0%							5.0E-235			
1,104,191	1,104,191	1	C -> G		69	SNP (transversion)	98.6%							2.7E-223			
1,104,194	1,104,194	1	T -> G		71	SNP (transversion)	98.6%							7.1E-223			
1,104,210	1,104,210	1	T -> G		75	SNP (transversion)	98.7%							4.7E-243			
1,104,213	1,104,213	1	C -> G		78	SNP (transversion)	98.7%							3.1E-245			
1,104,240	1,104,239	0	CT	84 -> 85		Insertion	96.4% -> 96.5%							4.8E-263			
1,104,242	1,104,242	1	T -> G		85	SNP (transversion)	98.8%							3.4E-259			
1,104,252	1,104,252	1	C -> G		83	SNP (transversion)	98.8%							1.3E-277			
1,104,279	1,104,280	2	AT -> GC	76 -> 77		Substitution	96.1%							9.2E-245			
1,104,288	1,104,287	0	C		77	Insertion	100.0%							4.0E-247			
1,104,338	1,104,338	1	G -> A		78	SNP (transition)	98.7%							1.6E-237			
1,104,342	1,104,342	1	C -> A		78	SNP (transversion)	100.0%							4.0E-258			
1,104,385	1,104,385	1	G -> A		80	SNP (transition)	98.8%							1.0E-243			
1,104,483	1,104,483	1	G -> C		84	SNP (transversion)	98.8%							1.1E-272			
1,104,488	1,104,489	2	AC -> TT		87	Substitution	97.7%							8.7E-257			
1,104,522	1,104,522	1	C -> T		77	SNP (transition)	100.0%							4.0E-247			
1,104,552	1,104,552	1	C -> G		76	SNP (transversion)	98.7%							2.4E-246			
1,104,567	1,104,567	1	G -> C		77	SNP (transversion)	100.0%							7.9E-255			
1,104,576	1,104,576	1	G -> C		80	SNP (transversion)	100.0%							1.0E-264			
1,104,642	1,104,642	1	C -> T		79	SNP (transition)	98.7%							2.0E-248			
1,104,651	1,104,651	1	A -> T		78	SNP (transversion)	96.2%							7.6E-251			
1,104,663	1,104,663	1	A -> G		76	SNP (transition)	98.7%							2.4E-231			
1,104,702	1,104,702	1	G -> A		78	SNP (transition)	98.7%							3.9E-222			
1,104,772	1,104,772	1	A -> G		82	SNP (transition)	100.0%							2.5E-271			
1,104,837	1,104,837	1	C -> A		81	SNP (transversion)	100.0%							4.0E-276			
1,104,903	1,104,903	1	C -> T		87	SNP (transition)	98.9%							1.4E-282			
1,104,936	1,104,936	1	C -> T		90	SNP (transition)	97.8%							6.3E-270			
1,104,981	1,104,981	1	A -> G		99	SNP (transition)	99.0%							0.0			
1,105,011	1,105,011	1	A -> C		96	SNP (transversion)	100.0%							2.5E-298			
1,105,020	1,105,020	1	T -> C		100	SNP (transition)	100.0%							0.0			
1,105,029	1,105,029	1	G -> C		102	SNP (transversion)	98.0%							0.0			
1,105,061	1,105,061	1	A -> G		104	SNP (transition)	98.1%							0.0			
1,105,073	1,105,073	1	A -> G		104	SNP (transition)	99.0%							0.0			
1,105,170	1,105,170	1	T -> C		101	SNP (transition)	100.0%	M -> T	56	ATG -> ACG	C770_RS46965	hypothetical protein	Substitution	0.0	19	2	WP_015243221.1
1,105,198	1,105,198	1	G -> A		107	SNP (transition)	100.0%		84	AAG -> AAA	C770_RS46965	hypothetical protein	None	0.0	28	3	WP_015243221.1
1,105,303	1,105,303	1	C -> G		93	SNP (transversion)	98.9%		189	TCC -> TCG	C770_RS46965	hypothetical protein	None	2.3E-302	63	3	WP_015243221.1
1,105,312	1,105,312	1	A -> G		91	SNP (transition)	98.9%		198	ACA -> ACG	C770_RS46965	hypothetical protein	None	9.1E-305	66	3	WP_015243221.1
1,105,336	1,105,336	1	G -> A		89	SNP (transition)	97.8%		222	GCG -> GCA	C770_RS46965	hypothetical protein	None	1.6E-275	74	3	WP_015243221.1
1,105,345	1,105,345	1	G -> C		87	SNP (transversion)	97.7%		231	GGG -> GGC	C770_RS46965	hypothetical protein	None	3.7E-286	77	3	WP_015243221.1
1,105,416	1,105,416	1	C -> G		87	SNP (transversion)	97.7%	T -> S	302	ACC -> AGC	C770_RS46965	hypothetical protein	Substitution	3.7E-286	101	2	WP_015243221.1
1,105,429	1,105,429	1	T -> C		81	SNP (transition)	100.0%		315	CCT -> CCC	C770_RS46965	hypothetical protein	None	1.0E-243	105	3	WP_015243221.1
1,105,471	1,105,471	1	A -> C		85	SNP (transversion)	98.8%		357	GCA -> GCC	C770_RS46965	hypothetical protein	None	3.4E-259	119	3	WP_015243221.1
1,105,546	1,105,546	1	T -> G		74	SNP (transversion)	100.0%	S -> A	58	TCA -> GCA	C770_RS46970	hypothetical protein	Substitution	2.5E-252	20	1	WP_041169889.1
1,105,553	1,105,553	1	T -> C		76	SNP (transition)	98.7%	V -> A	65	GTT -> GCT	C770_RS46970	hypothetical protein	Substitution	7.6E-254	22	2	WP_041169889.1
1,105,596	1,105,596	1	A -> C		73	SNP (transversion)	97.3%		108	GGA -> GGC	C770_RS46970	hypothetical protein	None	1.3E-231	36	3	WP_041169889.1
1,105,713	1,105,713	1	T -> C		88	SNP (transition)	98.9%		225	AGT -> AGC	C770_RS46970	hypothetical protein	None	1.8E-268	75	3	WP_041169889.1
1,105,758	1,105,758	1	G -> C		85	SNP (transversion)	98.8%	K -> N	270	AAG -> AAC	C770_RS46970	hypothetical protein	Substitution	5.4E-276	90	3	WP_041169889.1
1,105,782	1,105,782	1	G -> A		85	SNP (transition)	98.8%		294	GGG -> GGA	C770_RS46970	hypothetical protein	None	5.4E-276	98	3	WP_041169889.1
1,105,784	1,105,785	2	AG -> GA		85	Substitution	97.6%	Q -> R	296	CAG -> CGA	C770_RS46970	hypothetical protein	Substitution	2.3E-279	99	2	WP_041169889.1
1,105,800	1,105,801	2	GA -> CG		89	Substitution	96.6%	AM -> AV	312	GCG,ATG -> GCC	C770_RS46970	hypothetical protein	Substitution	7.8E-267	104	3	WP_041169889.1
1,105,845	1,105,845	1	T -> C		74	SNP (transition)	100.0%		357	GTT -> GTC	C770_RS46970	hypothetical protein	None	1.0E-259	119	3	WP_041169889.1
1,105,869	1,105,869	1	C -> G		67	SNP (transversion)	98.5%		381	CGC -> CGG	C770_RS46970	hypothetical protein	None	2.7E-223	127	3	WP_041169889.1
1,105,884	1,105,884	1	C -> T		67	SNP (transition)	100.0%		396	TCC -> TCT	C770_RS46970	hypothetical protein	None	1.6E-228	132	3	WP_041169889.1
1,105,899	1,105,899	1	A -> G		63	SNP (transition)	98.4%		411	GAA -> GAG	C770_RS46970	hypothetical protein	None	6.3E-216	137	3	WP_041169889.1
1,105,924	1,105,924	1	A -> G		55	SNP (transition)	100.0%	T -> A	436	ACA -> GCA	C770_RS46970	hypothetical protein	Substitution	1.0E-187	146	1	WP_041169889.1
1,105,932	1,105,932	1	C -> T		55	SNP (transition)	100.0%		444	ATC -> ATT	C770_RS46970	hypothetical protein	None	1.0E-187	148	3	WP_041169889.1
1,105,947	1,105,947	1	C -> A		48	SNP (transversion)	100.0%		459	CCC -> CCA	C770_RS46970	hypothetical protein	None	6.3E-164	153	3	WP_041169889.1
1,105,978	1,105,978	1	C -> T		42	SNP (transition)	100.0%							1.6E-143			
1,105,988	1,105,988	1	T -> C		38	SNP (transition)	97.4%							3.0E-121			
1,105,991	1,105,996	6	GTCAAC -> AATC	32 -> 35		Substitution	100.0%							1.6E-109			
1,105,998	1,105,998	1	A -> C		30	SNP (transversion)	100.0%							1.0E-102			
1,106,000	1,106,001	2	GG -> CAACG	27 -> 29		Insertion	100.0%							1.6E-92			
1,106,002	1,106,001	0	TGGCAAGGG	24 -> 25		Insertion	100.0%							1.6E-77			
1,106,004	1,106,004	1	T -> C		24	SNP (transition)	100.0%							6.3E-68			
1,106,014	1,106,014	1	T -> C		23	SNP (transition)	100.0%							2.0E-67			
1,106,043	1,106,043	1	C -> T		17	SNP (transition)	100.0%							1.0E-51			
1,106,045	1,106,045	1	A -> G		16	SNP (transition)	100.0%							4.0E-47			
1,106,048	1,106,048	1	A -> C		13	SNP (transversion)	100.0%							7.9E-36			
1,106,052	1,106,051	0	T		12	Insertion	100.0%							1.6E-35			
1,106,056	1,106,056	1	C -> T		11	SNP (transition)	100.0%							4.0E-27			
1,106,064	1,106,065	2	GT -> CC		10	Substitution	100.0%							1.0E-25			
1,106,067	1,106,070	4	GCTA -> AACG	9 -> 10		Substitution	100.0%							1.3E-19			
1,106,075	1,106,077	3	CTT -> AGC		7	Substitution	100.0%							7.9E-17			
1,106,079	1,106,079	1	G -> C		6	SNP (transversion)	100.0%							6.3E-14			
1,106,239	1,106,240	2	AT -> CC		6	Substitution	100.0%							4.0E-18			
1,106,243	1,106,243	1	G -> A		9	SNP (transition)	100.0%							5.0E-25			
1,106,252	1,106,257	6	CCACAA		9	Deletion	100.0%							3.2E-14			
1,106,269	1,106,269	1	C -> A		16	SNP (transversion)	100.0%							6.3E-52			
1,106,288	1,106,288	1	G -> A		18	SNP (transition)	100.0%							1.0E-63			
1,106,301	1,106,301	1	G -> T		25	SNP (transversion)	100.0%							3.2E-83			
1,106,307	1,106,307	1	T -> A		26	SNP (transversion)	100.0%							4.0E-89			
1,106,311	1,106,311	1	G -> T		27	SNP (transversion)	100.0%							4.0E-87			
1,106,322	1,106,322	1	A -> C		30	SNP (transversion)	100.0%							1.0E-108			
1,106,330	1,106,329	0	T		30	Insertion	100.0%							1.0E-105			
1,106,354	1,106,354	1	T -> C		39	SNP (transition)	97.4%							6.2E-117			
1,106,368	1,106,368	1	T -> G		40	SNP (transversion)	100.0%							1.0E-144			
1,106,380	1,106,381	2	GG -> AT		43	Substitution	97.7%							6.8E-142			
1,106,386	1,106,387	2	TG -> CA	46 -> 47		Substitution	100.0%							6.3E-148			
1,106,394	1,106,394	1	C -> AT	49 -> 50		Insertion	100.0%							2.5E-167			
1,106,412	1,106,413	2	TT		57	Deletion	100.0%							6.3E-92			
1,106,417	1,106,417	1	G -> A		57	SNP (transition)	100.0%							7.9E-189			
1,106,445	1,106,445	1	C -> T		69	SNP (transition)	98.6%							6.9E-237			
1,106,449	1,106,449	1	T -> C		70	SNP (transition)	100.0%							1.0E-252			
1,106,451	1,106,451	1	G -> A		71	SNP (transition)	100.0%							2.5E-256			

1,106,456	1,106,456	1	T -> C		71	SNP (transition)	100.0%							4.0E-242			
1,106,459	1,106,458	0	(C)2 -> (C)3		72	Insertion (tandem repeat)	100.0%							2.5E-238			
1,106,460	1,106,460	1	G -> A		72	SNP (transition)	95.8%							1.5E-230			
1,106,492	1,106,492	1	A -> G		84	SNP (transition)	100.0%							1.0E-294			
1,106,517	1,106,517	1	A -> T		96	SNP (transversion)	99.0%							3.0E-312			
1,106,547	1,106,547	1	C -> A		91	SNP (transversion)	98.9%							9.1E-287			
1,106,556	1,106,556	1	T -> C		94	SNP (transition)	98.9%							5.9E-315			
1,106,566	1,106,566	1	G		91	Deletion	100.0%							2.5E-146			
1,106,588	1,106,587	0	GGC	92 -> 93		Insertion	98.9%							5.8E-290			
1,106,594	1,106,594	1	C -> G		90	SNP (transversion)	100.0%							1.0E-306			
1,106,604	1,106,604	1	C -> T		91	SNP (transition)	98.9%							9.1E-296			
1,106,616	1,106,616	1	T -> G		91	SNP (transversion)	98.9%							9.1E-287			
1,106,618	1,106,618	1	T -> C		91	SNP (transition)	98.9%							9.1E-287			
1,106,625	1,106,625	1	T -> A		91	SNP (transversion)	100.0%							5.0E-301			
1,106,631	1,106,630	0	C		91	Insertion	100.0%							4.0E-310			
1,106,635	1,106,634	0	T		89	Insertion	98.9%							3.5E-289			
1,106,662	1,106,662	1	G -> A		93	SNP (transition)	97.8%							2.1E-297			
1,106,668	1,106,668	1	A -> G		92	SNP (transition)	96.7%							2.0E-280			
1,106,670	1,106,670	1	A -> T		94	SNP (transversion)	100.0%							1.0E-282			
1,106,683	1,106,683	1	C -> T		90	SNP (transition)	100.0%							1.0E-288			
1,106,752	1,106,752	1	A -> G		81	SNP (transition)	100.0%							5.0E-268			
1,106,870	1,106,870	1	T -> C		66	SNP (transition)	100.0%							1.0E-231			
1,106,877	1,106,877	1	C -> A		65	SNP (transversion)	100.0%							3.2E-228			
1,106,892	1,106,892	1	G -> A		63	SNP (transition)	100.0%							6.3E-215			
1,106,906	1,106,906	1	T -> C		55	SNP (transition)	100.0%							1.0E-198			
1,106,909	1,106,909	1	T -> G		55	SNP (transversion)	100.0%							3.2E-193			
1,106,912	1,106,912	1	C -> G		54	SNP (transversion)	98.1%							6.8E-174			
1,106,919	1,106,919	1	C -> T		53	SNP (transition)	100.0%							1.3E-175			
1,106,933	1,106,934	2	GC -> CT		52	Substitution	100.0%							1.0E-182			
1,106,944	1,106,944	1	T -> A		47	SNP (transversion)	100.0%							1.3E-174			
1,106,947	1,106,947	1	C -> T		46	SNP (transition)	100.0%							1.0E-161			
1,106,950	1,106,950	1	G -> C		46	SNP (transversion)	100.0%							4.0E-157			
1,106,954	1,106,954	1	G -> T		45	SNP (transversion)	100.0%							1.0E-153			
1,106,964	1,106,964	1	G -> A		41	SNP (transition)	100.0%							7.9E-128			
1,106,974	1,106,973	0	(T)2 -> (T)3		39	Insertion (tandem repeat)	100.0%							2.0E-129			
1,106,975	1,106,975	1	A -> G		38	SNP (transition)	100.0%							2.5E-122			
1,106,980	1,106,980	1	T -> C		38	SNP (transition)	100.0%							1.6E-118			
1,106,982	1,106,983	2	GA -> AG	37 -> 38		Substitution	100.0%							2.5E-122			
1,106,985	1,106,986	2	AC -> GA		36	Substitution	100.0%							1.6E-119			
1,106,996	1,106,996	1	G -> T		35	SNP (transversion)	100.0%							1.0E-112			
1,107,005	1,107,005	1	G -> T		30	SNP (transversion)	100.0%							1.0E-96			
1,107,013	1,107,013	1	T -> G		26	SNP (transversion)	100.0%							4.0E-76			
1,107,020	1,107,020	1	C -> A		25	SNP (transversion)	100.0%							1.0E-75			
1,107,036	1,107,036	1	G -> C		24	SNP (transversion)	100.0%							2.5E-70			
1,107,056	1,107,056	1	C -> T		20	SNP (transition)	100.0%							1.0E-56			
1,107,068	1,107,068	1	T -> C		18	SNP (transition)	100.0%							1.0E-45			
1,107,074	1,107,076	3	ATT -> CGC	15 -> 16		Substitution	100.0%							1.0E-42			
1,107,078	1,107,078	1	G -> C		15	SNP (transversion)	100.0%							3.2E-41			
1,107,081	1,107,085	5	TGCTC -> CTTGA	11 -> 14		Substitution	100.0%							1.3E-32			
1,107,087	1,107,088	2	CG -> TA		11	Substitution	100.0%							1.3E-32			
1,107,093	1,107,093	1	A -> G		10	SNP (transition)	100.0%							1.0E-27			
1,107,111	1,107,111	1	G -> C		7	SNP (transversion)	100.0%							4.0E-23			
1,107,136	1,107,136	1	T -> C		6	SNP (transition)	100.0%							4.0E-21			
1,107,605	1,107,606	2	CC -> GA		7	Substitution	100.0%	G -> S	199	GGG -> TCG	C770_RS46980	hypothetical protein	Substitution	1.6E-17	67	1	WP_015243224.1
1,107,609	1,107,609	1	C -> G		10	SNP (transversion)	100.0%	E -> Q	196	GAG -> CAG	C770_RS46980	hypothetical protein	Substitution	1.0E-30	66	1	WP_015243224.1
1,107,611	1,107,611	1	G -> T		10	SNP (transversion)	100.0%		194		C770_RS46980	hypothetical protein	Truncation	1.0E-30	65	2	WP_015243224.1
1,107,613	1,107,615	2	CGC -> TGG	10 -> 11		Substitution	100.0%	A -> P	190	GCG -> CCA	C770_RS46980	hypothetical protein	Substitution	1.6E-31	64	1	WP_015243224.1
1,107,618	1,107,618	1	A -> T		15	SNP (transversion)	100.0%	Y -> N	187	TAT -> AAT	C770_RS46980	hypothetical protein	Substitution	3.2E-41	63	1	WP_015243224.1
1,107,625	1,107,626	2	AT -> GG	17 -> 18		Substitution	100.0%	D -> A	179	GAT -> GCC	C770_RS46980	hypothetical protein	Substitution	6.3E-45	60	2	WP_015243224.1
1,107,630	1,107,631	2	CT -> GA		18	Substitution	100.0%	SA -> SP	174	TCA,GCC -> TCT,(	C770_RS46980	hypothetical protein	Substitution	2.5E-49	58	3	WP_015243224.1
1,107,634	1,107,643	9	CTTGCCTTTC -> 1	20 -> 24		Substitution	90.9% -> 95.2%	AKGK -> ASLQ	162	GCG,AAA,GGC,A,	C770_RS46980	hypothetical protein	Substitution	7.9E-56	54	3	WP_015243224.1
1,107,657	1,107,657	1	C -> T		28	SNP (transition)	100.0%	A -> T	148	GCG -> ACG	C770_RS46980	hypothetical protein	Substitution	1.6E-87	50	1	WP_015243224.1
1,107,676	1,107,676	1	C -> G		33	SNP (transversion)	100.0%		129	CTG -> CTC	C770_RS46980	hypothetical protein	None	2.0E-96	43	3	WP_015243224.1
1,107,685	1,107,685	1	G -> C		37	SNP (transversion)	100.0%		120		C770_RS46980	hypothetical protein	Truncation	1.0E-111	40	3	WP_015243224.1
1,107,714	1,107,714	1	G -> A		52	SNP (transition)	100.0%	L -> F	91	CTC -> TTC	C770_RS46980	hypothetical protein	Substitution	1.6E-151	31	1	WP_015243224.1
1,107,722	1,107,722	1	T -> C		55	SNP (transition)	100.0%	D -> G	83	GAC -> GGC	C770_RS46980	hypothetical protein	Substitution	1.0E-165	28	2	WP_015243224.1
1,107,734	1,107,734	1	C -> G		59	SNP (transversion)	96.6%	W -> S	71	TGG -> TCG	C770_RS46980	hypothetical protein	Substitution	3.4E-174	24	2	WP_015243224.1
1,107,754	1,107,754	1	A -> G		62	SNP (transition)	100.0%		51	GAT -> GAC	C770_RS46980	hypothetical protein	None	4.0E-199	17	3	WP_015243224.1
1,107,764	1,107,764	1	T -> C		63	SNP (transition)	100.0%	E -> G	41	GAG -> GGG	C770_RS46980	hypothetical protein	Substitution	5.0E-196	14	2	WP_015243224.1
1,107,766	1,107,766	1	A -> G		64	SNP (transition)	100.0%		39	CCT -> CCC	C770_RS46980	hypothetical protein	None	4.0E-199	13	3	WP_015243224.1
1,107,769	1,107,769	1	A -> G		64	SNP (transition)	100.0%		36	GGT -> GGC	C770_RS46980	hypothetical protein	None	4.0E-199	12	3	WP_015243224.1
1,107,829	1,107,829	1	C		91	Deletion	100.0%							2.5E-146			
1,107,842	1,107,842	1	T -> A		97	SNP (transversion)	100.0%							4.0E-311			
1,107,911	1,107,911	1	A -> G		103	SNP (transition)	99.0%	N -> D	49	AAC -> GAC	C770_RS46985	hypothetical protein	Substitution	0.0	17	1	WP_013850850.1
1,107,914	1,107,914	1	T -> G		100	SNP (transversion)	97.0%	S -> A	52	TCT -> GCT	C770_RS46985	hypothetical protein	Substitution	0.0	18	1	WP_013850850.1
1,107,921	1,107,921	1	C -> T		98	SNP (transition)	99.0%	T -> M	59	ACG -> ATG	C770_RS46985	hypothetical protein	Substitution	0.0	20	2	WP_013850850.1
1,107,968	1,107,968	1	T -> C		93	SNP (transition)	98.9%	F -> L	106	TTC -> CTC	C770_RS46985	hypothetical protein	Substitution	5.9E-284	36	1	WP_013850850.1
1,107,998	1,107,998	1	C -> T		90	SNP (transition)	100.0%	L -> F	136	CTT -> TTT	C770_RS46985	hypothetical protein	Substitution	1.0E-270	46	1	WP_013850850.1
1,108,045	1,108,045	1	A -> T		91	SNP (transversion)	98.9%	E -> D	183	GAA -> GAT	C770_RS46985	hypothetical protein	Substitution	9.1E-296	61	3	WP_013850850.1
1,108,053	1,108,053	1	G -> A		91	SNP (transition)	100.0%		191	TGA -> TAA	C770_RS46985	hypothetical protein	None	5.0E-301	64	2	WP_013850850.1
1,108,066	1,108,066	1	C -> A		95	SNP (transversion)	100.0%							3.2E-314			
1,108,078	1,108,078	1	T -> C		94	SNP (transition)	100.0%							6.3E-311			
1,108,091	1,108,090	0	(C)2 -> (C)3		87	Insertion (tandem repeat)	100.0%							4.0E-279			
1,108,118	1,108,119	2	AA -> TG	78 -> 79		Substitution	100.0%							2.5E-250			
1,108,126	1,108,126	1	T -> G		75	SNP (transversion)	100.0%							1.0E-255			
1,108,134	1,108,134	1	A -> G		73	SNP (transition)	100.0%							1.3E-241			
1,108,139	1,108,139	1	T		72	Deletion	100.0%							6.3E-116			
1,108,147	1,108,147	1	T -> G		70	SNP (transversion)	100.0%							1.0E-231			
1,108,220	1,108,220	1	C -> T		77	SNP (transition)	100.0%							6.3E-278			
1,108,225	1,108,225	1	T -> C		77	SNP (transition)	100.0%							1.6E-262			
1,108,238	1,108,238	1	C -> T		80	SNP (transition)	100.0%							1.0E-264			
1,108,263	1,108,263	1	T -> C		80	SNP (transition)	100.0%							1.0E-272			
1,108,273	1,108,273	1	G -> A		83	SNP (transition)	100.0%							2.5E-266			
1,108,298	1,108,298	1	A -> C		76	SNP (transversion)	100.0%							1.6E-251			
1,108,311	1,108,311	1	T -> C		73	SNP (transition)	98.6%							1.2E-243			
1,108,344	1,108,344	1	A -> C		72	SNP (transversion)	100.0%							2.5E-238			
1,108,453	1,108,453	1	G -> C		81	SNP (transversion)	100.0%							1.0E-243			
1,108,462	1,108,462	1	G -> A		80	SNP (transition)	100.0%							1.0E-272			
1,108,464	1,108,464	1	C -> T		80	SNP (transition)	98.8%							2.5E-275			
1,108,514	1,108,514	1	C -> T		79	SNP (transition)	100.0%							1.6E-253			
1,108,518	1,108,521	4	CGTG -> TCGT	77 -> 78		Substitution	96.1% -> 96.2%							6.2E-253			
1,108,523	1,108,523	1	T -> G		78	SNP (transversion)	100.0%							4.0E-258			
1,108,525	1,108,525	1	A -> T		78	SNP (transversion)	98.7%							1.2E-260			
1,108,537	1,108,537	1	T -> G		81	SNP (transversion)	100.0%							4.0E-276			
1,108,554	1,108,554	1	A -> G		84	SNP (transition)	100.0%							1.6E-269			
1,108,617	1,108,617	1	C -> A		78	SNP (transversion)	100.0%	L -> I	13	CTA -> ATA	C770_RS46990	hypothetical protein	Substitution	6.3E-266	5	1	WP_015243225.1
1,108,622	1,108,622	1	G -> T		76	SNP (transversion)	100.0%	K -> N	18	AAG -> AAT	C770_RS46990	hypothetical protein	Substitution	1.0E-266	6	3	WP_015243225.1
1,108,802	1,108,802	1	T -> C		68	SNP (transition)	98.5%		198	ATT -> ATC	C770_RS46990	hypothetical protein	None	1.1E-226	66	3	WP_015243225.1
1,108,850	1,108,850	1	G -> T		72	SNP (transversion)	98.6%		246	CCG -> CCT	C770_RS46990	hypothetical protein	None	3.6E-233	82	3	WP_015243225.1
1,108,965	1,108,965	1	A -> G		62	SNP (transition)	100.0%	R -> G	361	AGG -> GGG	C770_RS46990	hypothetical protein	Substitution	1.0E-217	121	1	WP_015243225.1
1,108,980	1,108,980	1	G -> A		56	SNP (transition)	100.0%	D -> N	376	GAC -> AAC	C770_RS46990	hypothetical protein	Substitution	1.0E-196	126	1	WP_015243225.1
1,109,032	1,109,032	1	(C)5 -> (C)4		43	Deletion (tandem repeat)	100.0%							1.6E-69			
1,109,045	1,109,045	1	C -> T		43	SNP (transition)	100.0%							3.2E-151			
1,109,087	1,109,086	0	TAAGACAC	31 -> 32		Insertion	100.0%							6.3E-100			
1,109,104	1,109,104	1	G -> T		29	SNP (transversion)	100.0%							1.3E-90			
1,109,106	1,109,106	1	C -> G		26	SNP (transversion)	100.0%							1.0E-91			
1,109,115	1,109,115	1	T -> C		25	SNP (transition)	96.0%							2.5E-71			
1,109,122	1,109,122	1	G -> T		25	SNP (transversion)	100.0%							1.0E-75			
1,109,129	1,109,129	1	C -> G		20	SNP (transversion)	100.0%							1.0E-58			
1,109,141	1,109,141	1	G -> A		19	SNP (transition)	100.0%							5.0E-52			
1,109,148	1,109,148	1	T -> G		16	SNP (transversion)	100.0%							4.0E-47			
1,109,159	1,109,159	1	C -> A		13	SNP (transversion)	100.0%							6.3E-32			
1,109,182	1,109,182	1	G -> A		6	SNP (transition)	100.0%							4.0E-15			
1,109,545	1,109,545	1	G -> A		50	SNP (transition)	96.0%		144	CAC -> CAT	C770_RS46995	antibiotic biosynthesis n	None	7.7E-161	48	3	WP_010975476.1
1,109,611	1,109,611	1	G -> A		70	SNP (transition)	95.7%		78	TAC -> TAT	C770_RS46995	antibiotic biosynthesis n	None	1.7E-230	26	3	WP_010975476.1
1,109,650	1,109,650	1	C -> G		72	SNP (transversion)	98.6%		39	GGG -> GGC	C770_RS46995	antibiotic biosynthesis n	None	4.5E-226	13	3	WP_010975476.1
1,109,692	1,109,692	1	T -> G		72	SNP (transversion)	98.6%							2.9E-240			
1,109,728	1,109,728	1	C -> A		78	SNP (transversion)	98.7%							1.2E-260			
1,109,792	1,109,792	1	C -> G		81	SNP (transversion)	98.8%		1.5	GCG -> GCC	C770_RS47000	Sugar (pentulose and he	None	8.1E-271	500	3	WP_015243226.1
1,109,842	1,109,842	1	C -> G		88	SNP (transversion)	100.0%	D -> H	1.45	GAC -> CAC	C770_RS47000	Sugar (pentulose and he	Substitution	1.6E-273	484	1	WP_015243226.1
1,109,939	1,109,939	1	C -> A		92	SNP (transversion)	96.7%		1.353	GCG -> GCT	C770_RS47000	Sugar (pentulose and he	None	2.0E-280	451	3	WP_015243226.1
1,109,963	1,109,963	1	T -> C		92	SNP (transition)	100.0%		1.329	AAA -> AAG	C770_RS47000	Sugar (pentulose and he	None	6.3E-286	443	3	WP_015243226.1
1,109,978	1,109,978	1	G -> A		87	SNP (transition)	98.9%		1.314	GTC -> GTT	C770_RS47000	Sugar (pentulose and he	None	2.2E-265	438	3	WP_015243226.1
1,110,044	1,110,044	1	C -> A		76	SNP (transversion)	100.0%		1.248	GCG -> GCT	C770_RS47000	Sugar (pentulose and he	None	4.0E-259	416	3	WP_015243226.1
1,110,059	1,110,059	1	A -> G		77	SNP (transition)	98.7%		1.233	GAT -> GAC	C770_RS47000	Sugar (pentulose and he	None	3.1E-257	411	3	WP_015243226.1
1,110,257	1,110,257	1	G -> A		66	SNP (transition)	100.0%		1.035	GCC -> GCT	C770_RS47000	Sugar (pentulose and he	None	6.3E-212	345	3	WP_015243226.1
1,110,263	1,110,263	1	G -> C		68	SNP (transversion)	98.5%		1.029	GTC -> GTG	C770_RS47000	Sugar (pentulose and he	None	2.7E-213	343	3	WP_015243226.1
1,110,266	1,110,266	1	T -> C		67	SNP (transition)	100.0%		1.026	CCA -> CCG	C770_RS47000	Sugar (pentulose and he	None	1.6E-228	342	3	WP_015243226.1
1,110,272	1,110,274	2	GCA -> CCC	66 -> 67		Substitution	100.0%	C -> G	1.018	TGC -> GGG	C770_RS47000	Sugar (pentulose and he	Substitution	7.9E-222	340	1	WP_015243226.1
1,110,281	1,110,281	1	C -> T		66	SNP (transition)	98.5%		1.011	AAG -> AAA	C770_RS47000	Sugar (pentulose and he	None	6.6E-207	337	3	WP_015243226.1
1,110,286	1,110,286	1	G -> C		67	SNP (transversion)	100.0%	Q -> E	1.006	CAG -> GAG	C770_RS47000	Sugar (pentulose and he	Substitution	7.9E-222	336	1	WP_015243226.1
1,110,290	1,110,290	1	C -> G		66	SNP (transversion)	100.0%		1.002	CTG -> CTC	C770_RS47000	Sugar (pentulose and he	None	4.0E-225	334	3	WP_015243226.1
1,110,347	1,110,347	1	G -> A		72	SNP (transition)	97.2%		945	AGC -> AGT	C770_RS47000	Sugar (pentulose and he	None	2.6E-228	315	3	WP_015243226.1
1,110,362	1,110,362	1	G -> A		74	SNP (transition)	98.6%		930	CGC -> CGT	C770_RS47000	Sugar (pentulose and he	None	1.9E-232	310	3	WP_015243226.1
1,110,371	1,110,371	1	G -> C		75	SNP (transversion)	100.0%	I -> M	921	ATC -> ATG	C770_RS47000	Sugar (pentulose and he	Substitution	3.2E-233	307	3	WP_015243226.1
1,110,410	1,110,410	1	C -> G		75	SNP (transversion)	98.7%		882	GGG -> GGC	C770_RS47000	Sugar (pentulose and he	None	1.2E-235	294	3	WP_015243226.1
1,110,413	1,110,414	2	AC -> GT		74	Substitution	95.9%	S -> N	878	AGT -> AAC	C770_RS47000	Sugar (pentulose and he	Substitution	2.0E-244	293	2	WP_015243226.1
1,110,569	1,110,569	1	G -> A		93	SNP (transition)	96.8%		723	GGC -> GGT	C770_RS47000	Sugar (pentulose and he	None	1.3E-292	241	3	WP_015243226.1
1,110,578	1,110,578	1	C -> T		94	SNP (transition)	100.0%		714	GTG -> GTA	C770_RS47000	Sugar (pentulose and he	None	1.6E-301	238	3	WP_015243226.1
1,110,622	1,110,622	1	T -> C		92	SNP (transition)	100.0%	K -> E	670	AAG -> GAG	C770_RS47000	Sugar (pentulose and he	Substitution	1.6E-313	224	1	WP_015243226.1
1,110,626	1,110,626	1	A -> G		91	SNP (transition)	100.0%		666	GTT -> GTC	C770_RS47000	Sugar (pentulose and he	None	6.3E-292	222	3	WP_015243226.1
1,110,661	1,110,661	1	G -> C		96	SNP (transversion)	99.0%	Q -> E	631	CAA -> GAA	C770_RS47000	Sugar (pentulose and he	Substitution	9.6E-303	211	1	WP_015243226.1
1,110,746	1,110,746	1	G -> C		88	SNP (transversion)	100.0%		546	GGC -> GGG	C770_RS47000	Sugar (pentulose and he	None	4.0E-291	182	3	WP_015243226.1
1,110,794	1,110,794	1	A -> G		86	SNP (transition)	98.8%		498	GTT -> GTC	C770_RS47000	Sugar (pentulose and he	None	2.7E-279	166	3	WP_015243226.1
1,110,800	1,110,800	1	G -> A		87	SNP (transition)	97.7%		492	GAC -> GAT	C770_RS47000	Sugar (pentulose and he	None	3.7E-269	164	3	WP_015243226.1
1,110,819	1,110,819	1	C -> T		84	SNP (transition)	97.6%	R -> K	473	AGG -> AAG	C770_RS47000	Sugar (pentulose and he	Substitution	5.5E-276	158	2	WP_015243226.1
1,110,827	1,110,827	1	G -> C		86	SNP (transversion)	100.0%		465	CGC -> CGG	C770_RS47000	Sugar (pentulose and he	None	4.0E-293	155	3	WP_015243226.1
1,110,839	1,110,839	1	T -> G		80	SNP (transversion)	100.0%	E -> D	453	GAA -> GAC	C770_RS47000	Sugar (pentulose and he	Substitution	1.0E-280	151	3	WP_015243226.1
1,110,965	1,110,965	1	C -> A		73	SNP (transversion)	100.0%		327	GTG -> GTT	C770_RS47000	Sugar (pentulose and he	None	2.5E-234	109	3	WP_015243226.1
1,110,968	1,110,968	1	G -> A		71	SNP (transition)	100.0%		324	GCC -> GCT	C770_RS47000	Sugar (pentulose and he	None	4.0E-242	108	3	WP_015243226.1

1,110,974	1,110,974	1	A -> G		70	SNP (transition)	100.0%		318	AGT -> AGC	C770_RS47000	Sugar (pentulose and he	None	1.0E-224	106	3	WP_015243226.1
1,110,998	1,110,998	1	A -> G		71	SNP (transition)	100.0%		294	ATT -> ATC	C770_RS47000	Sugar (pentulose and he	None	4.0E-242	98	3	WP_015243226.1
1,111,115	1,111,115	1	C -> G		65	SNP (transversion)	98.5%		177	TCG -> TCC	C770_RS47000	Sugar (pentulose and he	None	1.6E-216	59	3	WP_015243226.1
1,111,142	1,111,142	1	A -> T		65	SNP (transversion)	100.0%	D -> E	150	GAT -> GAA	C770_RS47000	Sugar (pentulose and he	Substitution	1.0E-195	50	3	WP_015243226.1
1,111,229	1,111,229	1	A -> G		64	SNP (transition)	98.4%		63	TTT -> TTC	C770_RS47000	Sugar (pentulose and he	None	1.6E-200	21	3	WP_015243226.1
1,111,262	1,111,262	1	G -> A		60	SNP (transition)	96.7%		30	GAC -> GAT	C770_RS47000	Sugar (pentulose and he	None	7.0E-189	10	3	WP_015243226.1
1,111,301	1,111,300	0	T		65	Insertion	100.0%		944		C770_RS47005	DeoR family transcriptio	Frame Shift	1.0E-208	315	2	WP_013850854.1
1,111,395	1,111,395	1	A -> G		72	SNP (transition)	100.0%		849	CAT -> CAC	C770_RS47005	DeoR family transcriptio	None	1.0E-252	283	3	WP_013850854.1
1,111,548	1,111,548	1	C -> T		79	SNP (transition)	98.7%		696	TTG -> TTA	C770_RS47005	DeoR family transcriptio	None	7.9E-272	232	3	WP_013850854.1
1,111,698	1,111,698	1	G -> A		78	SNP (transition)	100.0%		546	GTC -> GTT	C770_RS47005	DeoR family transcriptio	None	4.0E-258	182	3	WP_013850854.1
1,111,782	1,111,782	1	C -> A		81	SNP (transversion)	100.0%		462	ACG -> ACT	C770_RS47005	DeoR family transcriptio	None	4.0E-276	154	3	WP_013850854.1
1,111,815	1,111,815	1	G -> A		87	SNP (transition)	100.0%		429	ATC -> ATT	C770_RS47005	DeoR family transcriptio	None	4.0E-279	143	3	WP_013850854.1
1,111,830	1,111,830	1	G -> T		90	SNP (transversion)	97.8%		414	GCC -> GCA	C770_RS47005	DeoR family transcriptio	None	1.0E-278	138	3	WP_013850854.1
1,111,842	1,111,842	1	A -> G		87	SNP (transition)	100.0%		402	CTT -> CTC	C770_RS47005	DeoR family transcriptio	None	1.6E-296	134	3	WP_013850854.1
1,111,858	1,111,858	1	T -> C		92	SNP (transition)	100.0%	N -> S	386	AAC -> AGC	C770_RS47005	DeoR family transcriptio	Substitution	1.6E-313	129	2	WP_013850854.1
1,111,898	1,111,898	1	C -> T		91	SNP (transition)	100.0%	D -> N	346	GAC -> AAC	C770_RS47005	DeoR family transcriptio	Substitution	6.3E-292	116	1	WP_013850854.1
1,111,926	1,111,926	1	C -> T		77	SNP (transition)	100.0%		318	GCG -> GCA	C770_RS47005	DeoR family transcriptio	None	1.6E-262	106	3	WP_013850854.1
1,111,994	1,111,994	1	A -> G		85	SNP (transition)	97.6%		250	TTG -> CTG	C770_RS47005	DeoR family transcriptio	None	1.1E-287	84	1	WP_013850854.1
1,112,028	1,112,028	1	T -> C		86	SNP (transition)	98.8%		216	GCA -> GCG	C770_RS47005	DeoR family transcriptio	None	2.7E-279	72	3	WP_013850854.1
1,112,037	1,112,037	1	A -> G		85	SNP (transition)	98.8%		207	GGT -> GGC	C770_RS47005	DeoR family transcriptio	None	2.1E-284	69	3	WP_013850854.1
1,112,247	1,112,247	1	T -> A		84	SNP (transversion)	95.2%							1.9E-266			
1,112,251	1,112,251	1	G -> A		82	SNP (transition)	96.3%							2.2E-264			
1,112,310	1,112,310	1	G -> A		83	SNP (transition)	97.6%							1.7E-264			
1,112,359	1,112,359	1	T -> G		81	SNP (transversion)	96.3%							2.1E-245			
1,112,361	1,112,362	2	AT -> CA		81	Substitution	95.1%							6.6E-241			
1,112,371	1,112,371	1	A -> T		81	SNP (transversion)	97.5%							4.1E-242			
1,112,377	1,112,377	1	G -> A		79	SNP (transition)	96.2%							1.3E-246			
1,112,380	1,112,381	2	GC -> AG		79	Substitution	96.2%							3.1E-228			
1,112,388	1,112,388	1	G -> A		73	SNP (transition)	95.9%							6.2E-227			
1,112,393	1,112,393	1	C -> T		73	SNP (transition)	98.6%							1.8E-236			
1,112,399	1,112,399	1	T -> C		75	SNP (transition)	96.0%							4.3E-219			
1,112,411	1,112,412	2	TC -> CG		72	Substitution	98.6%							4.5E-226			
1,112,430	1,112,430	1	A -> C		73	SNP (transversion)	98.6%							1.8E-236			
1,112,439	1,112,439	1	A -> C		75	SNP (transversion)	97.3%							1.7E-245			
1,112,441	1,112,441	1	G -> A		75	SNP (transition)	98.7%							1.9E-250			
1,112,482	1,112,482	1	G -> A		75	SNP (transition)	98.7%							3.0E-228			
1,112,487	1,112,487	1	G -> A		71	SNP (transition)	97.2%							3.9E-218			
1,112,509	1,112,509	1	G -> A		72	SNP (transition)	98.6%							4.5E-226			
1,112,543	1,112,543	1	C -> T		69	SNP (transition)	98.6%							4.4E-230			
1,112,546	1,112,546	1	T -> G		69	SNP (transversion)	100.0%							1.6E-221			
1,112,567	1,112,567	1	T -> C		69	SNP (transition)	98.6%							6.9E-237			
1,112,584	1,112,584	1	G -> A		66	SNP (transition)	100.0%							1.6E-218			
1,112,807	1,112,807	1	C -> T		73	SNP (transition)	100.0%							1.0E-219			
1,112,843	1,112,843	1	T -> C		78	SNP (transition)	100.0%							1.0E-234			
1,112,945	1,112,945	1	C -> T		78	SNP (transition)	96.2%							7.6E-236			
1,112,951	1,112,951	1	G -> A		76	SNP (transition)	97.4%							4.5E-234			
1,113,002	1,113,002	1	G -> C		76	SNP (transversion)	97.4%							4.5E-234			
1,113,041	1,113,041	1	T -> C		79	SNP (transition)	98.7%							5.0E-264			
1,113,047	1,113,047	1	C -> T		78	SNP (transition)	97.4%							1.2E-255			
1,113,053	1,113,053	1	C -> T		78	SNP (transition)	98.7%							1.2E-260			
1,113,062	1,113,062	1	A -> C		79	SNP (transversion)	100.0%							2.5E-269			
1,113,080	1,113,081	2	AT -> GC		80	Substitution	97.5%							2.0E-262			
1,113,089	1,113,089	1	A -> G		77	SNP (transition)	98.7%							1.2E-249			
1,113,110	1,113,110	1	G -> A		79	SNP (transition)	97.5%							2.4E-251			
1,113,132	1,113,132	1	G -> T		81	SNP (transversion)	100.0%							5.0E-268			
1,113,245	1,113,245	1	T -> G		88	SNP (transversion)	100.0%							6.3E-300			
1,113,303	1,113,303	1	G -> C		86	SNP (transversion)	100.0%							6.3E-276			
1,113,399	1,113,398	0	C		79	Insertion	100.0%							2.5E-269			
1,113,411	1,113,411	1	C -> G		80	SNP (transversion)	100.0%							1.0E-264			
1,113,424	1,113,424	1	C -> G		77	SNP (transversion)	100.0%							7.9E-255			
1,113,454	1,113,454	1	A -> G		74	SNP (transition)	100.0%							6.3E-245			
1,113,526	1,113,526	1	A -> G		63	SNP (transition)	100.0%							1.3E-208			
1,113,529	1,113,529	1	A -> G		62	SNP (transition)	100.0%							2.5E-205			
1,113,616	1,113,616	1	A -> G		62	SNP (transition)	100.0%							2.5E-205			
1,113,742	1,113,742	1	C -> T		65	SNP (transition)	100.0%							3.2E-215			
1,113,751	1,113,751	1	G -> C		67	SNP (transversion)	98.5%							4.2E-210			
1,113,769	1,113,769	1	G -> C		71	SNP (transversion)	98.6%							7.1E-230			
1,113,875	1,113,875	1	G -> C		74	SNP (transversion)	100.0%							1.6E-237			
1,113,971	1,113,971	1	G -> A		70	SNP (transition)	97.1%							6.1E-215			
1,114,019	1,114,019	1	G -> C		73	SNP (transversion)	100.0%	M -> I	42	ATG -> ATC	C770_RS47015	sugar ABC transporter p	Substitution	2.5E-234	14	3	WP_015243227.1
1,114,022	1,114,022	1	A -> G		72	SNP (transition)	100.0%		45	CTA -> CTG	C770_RS47015	sugar ABC transporter p	None	6.3E-224	15	3	WP_015243227.1
1,114,087	1,114,087	1	C -> T		81	SNP (transition)	98.8%	A -> V	110	GCC -> GTC	C770_RS47015	sugar ABC transporter p	Substitution	8.1E-263	37	2	WP_015243227.1
1,114,286	1,114,286	1	G -> A		100	SNP (transition)	98.0%		309	GGG -> GGA	C770_RS47015	sugar ABC transporter p	None	7.8E-301	103	3	WP_015243227.1
1,114,349	1,114,349	1	A -> G		90	SNP (transition)	98.9%		372	ACA -> ACG	C770_RS47015	sugar ABC transporter p	None	2.3E-301	124	3	WP_015243227.1
1,114,364	1,114,364	1	C -> T		91	SNP (transition)	100.0%		387	CGC -> CGT	C770_RS47015	sugar ABC transporter p	None	6.3E-292	129	3	WP_015243227.1
1,114,436	1,114,436	1	G -> C		88	SNP (transversion)	100.0%		459	GCG -> GCC	C770_RS47015	sugar ABC transporter p	None	4.0E-291	153	3	WP_015243227.1
1,114,501	1,114,501	1	C -> T		86	SNP (transition)	100.0%	T -> I	524	ACC -> ATC	C770_RS47015	sugar ABC transporter p	Substitution	6.3E-276	175	2	WP_015243227.1
1,114,513	1,114,513	1	C -> G		84	SNP (transversion)	98.8%	T -> R	536	ACG -> AGG	C770_RS47015	sugar ABC transporter p	Substitution	4.2E-256	179	2	WP_015243227.1
1,114,526	1,114,526	1	T -> C		83	SNP (transition)	98.8%		549	GGT -> GGC	C770_RS47015	sugar ABC transporter p	None	8.3E-245	183	3	WP_015243227.1
1,114,553	1,114,553	1	T -> C		86	SNP (transition)	100.0%		576	AAT -> AAC	C770_RS47015	sugar ABC transporter p	None	6.3E-276	192	3	WP_015243227.1
1,114,586	1,114,586	1	T -> G		78	SNP (transversion)	100.0%		609	GTT -> GTG	C770_RS47015	sugar ABC transporter p	None	6.3E-266	203	3	WP_015243227.1
1,114,635	1,114,635	1	T -> C		82	SNP (transition)	100.0%	F -> L	658	TTC -> CTC	C770_RS47015	sugar ABC transporter p	Substitution	4.0E-263	220	1	WP_015243227.1
1,114,703	1,114,703	1	A -> G		81	SNP (transition)	100.0%		726	CTA -> CTG	C770_RS47015	sugar ABC transporter p	None	4.0E-276	242	3	WP_015243227.1
1,115,001	1,115,002	2	GT -> AG		80	Substitution	98.8%	V -> S	1.024	GTC -> AGC	C770_RS47015	sugar ABC transporter p	Substitution	1.3E-251	342	1	WP_015243227.1
1,115,031	1,115,031	1	(G)4 -> (G)3		81	Deletion (tandem repeat)	100.0%							4.0E-114			
1,115,136	1,115,136	1	A -> G		81	SNP (transition)	98.8%	I -> V	88	ATT -> GTT	C770_RS47020	Ribose/xylose/arabinos	Substitution	8.1E-287	30	1	WP_015243228.1
1,115,177	1,115,177	1	C -> A		79	SNP (transversion)	100.0%		129	ATC -> ATA	C770_RS47020	Ribose/xylose/arabinos	None	2.5E-269	43	3	WP_015243228.1
1,115,180	1,115,180	1	C -> T		79	SNP (transition)	100.0%		132	ATC -> ATT	C770_RS47020	Ribose/xylose/arabinos	None	2.5E-269	44	3	WP_015243228.1
1,115,192	1,115,192	1	C -> T		77	SNP (transition)	100.0%		144	ATC -> ATT	C770_RS47020	Ribose/xylose/arabinos	None	1.6E-262	48	3	WP_015243228.1
1,115,198	1,115,198	1	C -> G		78	SNP (transversion)	100.0%		150	GTC -> GTG	C770_RS47020	Ribose/xylose/arabinos	None	6.3E-266	50	3	WP_015243228.1
1,115,213	1,115,213	1	G -> A		83	SNP (transition)	100.0%		165	GTG -> GTA	C770_RS47020	Ribose/xylose/arabinos	None	1.3E-274	55	3	WP_015243228.1
1,115,360	1,115,360	1	C -> A		80	SNP (transversion)	100.0%		312	GTC -> GTA	C770_RS47020	Ribose/xylose/arabinos	None	1.0E-264	104	3	WP_015243228.1
1,115,375	1,115,375	1	T -> C		75	SNP (transition)	100.0%		327	AGT -> AGC	C770_RS47020	Ribose/xylose/arabinos	None	1.0E-255	109	3	WP_015243228.1
1,115,449	1,115,449	1	A -> G		91	SNP (transition)	100.0%	N -> S	401	AAC -> AGC	C770_RS47020	Ribose/xylose/arabinos	Substitution	3.2E-319	134	2	WP_015243228.1
1,115,470	1,115,471	2	AA -> CC	88 -> 89		Substitution	100.0%	E -> A	422	GAA -> GCC	C770_RS47020	Ribose/xylose/arabinos	Substitution	6.3E-300	141	2	WP_015243228.1
1,115,501	1,115,501	1	A -> G		86	SNP (transition)	100.0%		453	CCA -> CCG	C770_RS47020	Ribose/xylose/arabinos	None	6.3E-276	151	3	WP_015243228.1
1,115,528	1,115,528	1	C -> T		82	SNP (transition)	98.8%		480	CTC -> CTT	C770_RS47020	Ribose/xylose/arabinos	None	2.6E-282	160	3	WP_015243228.1
1,115,555	1,115,555	1	T -> C		85	SNP (transition)	100.0%		507	CAT -> CAC	C770_RS47020	Ribose/xylose/arabinos	None	3.2E-298	169	3	WP_015243228.1
1,115,611	1,115,611	1	A -> G		88	SNP (transition)	98.9%	H -> R	563	CAT -> CGT	C770_RS47020	Ribose/xylose/arabinos	Substitution	1.4E-294	188	2	WP_015243228.1
1,115,693	1,115,693	1	C -> G		91	SNP (transversion)	95.6%		645	GTC -> GTG	C770_RS47020	Ribose/xylose/arabinos	None	4.2E-290	215	3	WP_015243228.1
1,115,741	1,115,741	1	G -> C		92	SNP (transversion)	100.0%		693	ACG -> ACC	C770_RS47020	Ribose/xylose/arabinos	None	1.6E-313	231	3	WP_015243228.1
1,115,762	1,115,762	1	G -> A		89	SNP (transition)	100.0%		714	GCG -> GCA	C770_RS47020	Ribose/xylose/arabinos	None	2.0E-294	238	3	WP_015243228.1
1,115,768	1,115,768	1	A -> G		90	SNP (transition)	98.9%		720	GTA -> GTG	C770_RS47020	Ribose/xylose/arabinos	None	1.8E-292	240	3	WP_015243228.1
1,115,855	1,115,855	1	C -> T		90	SNP (transition)	100.0%		807	GGC -> GGT	C770_RS47020	Ribose/xylose/arabinos	None	1.0E-288	269	3	WP_015243228.1
1,115,948	1,115,948	1	G -> T		92	SNP (transversion)	100.0%	M -> I	900	ATG -> ATT	C770_RS47020	Ribose/xylose/arabinos	Substitution	6.3E-286	300	3	WP_015243228.1
1,115,966	1,115,966	1	G -> A		92	SNP (transition)	100.0%		918	GCG -> GCA	C770_RS47020	Ribose/xylose/arabinos	None	2.5E-304	306	3	WP_015243228.1
1,115,995	1,115,995	1	T -> C		94	SNP (transition)	96.8%	V -> A	947	GTG -> GCG	C770_RS47020	Ribose/xylose/arabinos	Substitution	6.7E-296	316	2	WP_015243228.1
1,116,004	1,116,004	1	C -> T		91	SNP (transition)	98.9%	T -> I	956	ACT -> ATT	C770_RS47020	Ribose/xylose/arabinos	Substitution	9.1E-278	319	2	WP_015243228.1
1,116,046	1,116,049	3	GAGC -> CCGT	82 -> 83		Substitution	100.0%	SS -> SR	24	TCG,AGC -> TCC,	C770_RS47025	autoinducer	Substitution	2.5E-266	8	3	WP_013850858.1
1,116,056	1,116,056	1	G -> A		81	SNP (transition)	98.8%	A -> T	34	GCC -> ACC	C770_RS47025	autoinducer	Substitution	8.1E-247	12	1	WP_013850858.1
1,116,106	1,116,106	1	A -> G		83	SNP (transition)	98.8%		84	GCA -> GCG	C770_RS47025	autoinducer	None	5.2E-253	28	3	WP_013850858.1
1,116,142	1,116,142	1	T -> C		90	SNP (transition)	100.0%		120	GGT -> GGC	C770_RS47025	autoinducer	None	1.0E-297	40	3	WP_013850858.1
1,116,202	1,116,202	1	G -> C		86	SNP (transversion)	100.0%		180	GCG -> GCC	C770_RS47025	autoinducer	None	6.3E-276	60	3	WP_013850858.1
1,116,211	1,116,211	1	C -> A		86	SNP (transversion)	98.8%		189	ACC -> ACA	C770_RS47025	autoinducer	None	8.6E-271	63	3	WP_013850858.1
1,116,265	1,116,265	1	C -> T		80	SNP (transition)	100.0%		243	AAC -> AAT	C770_RS47025	autoinducer	None	1.0E-256	81	3	WP_013850858.1
1,116,292	1,116,292	1	C -> T		83	SNP (transition)	100.0%		270	CTC -> CTT	C770_RS47025	autoinducer	None	2.5E-266	90	3	WP_013850858.1
1,116,406	1,116,406	1	T -> C		98	SNP (transition)	100.0%		384	TAT -> TAC	C770_RS47025	autoinducer	None	0.0	128	3	WP_013850858.1
1,116,466	1,116,466	1	G -> A		92	SNP (transition)	97.8%		444	GAG -> GAA	C770_RS47025	autoinducer	None	4.2E-285	148	3	WP_013850858.1
1,116,474	1,116,474	1	A -> C		92	SNP (transversion)	97.8%	K -> T	452	AAG -> ACG	C770_RS47025	autoinducer	Substitution	4.2E-312	151	2	WP_013850858.1
1,116,496	1,116,496	1	C -> G		90	SNP (transversion)	98.9%		474	GCC -> GCG	C770_RS47025	autoinducer	None	2.8E-310	158	3	WP_013850858.1
1,116,559	1,116,559	1	A -> G		99	SNP (transition)	100.0%		537	AAA -> AAG	C770_RS47025	autoinducer	None	0.0	179	3	WP_013850858.1
1,116,592	1,116,592	1	T -> G		94	SNP (transversion)	98.9%		570	GTT -> GTG	C770_RS47025	autoinducer	None	5.9E-315	190	3	WP_013850858.1
1,116,601	1,116,601	1	G -> A		89	SNP (transition)	100.0%		579	CAG -> CAA	C770_RS47025	autoinducer	None	2.0E-294	193	3	WP_013850858.1
1,116,604	1,116,604	1	T -> C		90	SNP (transition)	100.0%		582	TAT -> TAC	C770_RS47025	autoinducer	None	1.0E-315	194	3	WP_013850858.1
1,116,808	1,116,808	1	G -> C		81	SNP (transversion)	98.8%		786	ACG -> ACC	C770_RS47025	autoinducer	None	8.1E-263	262	3	WP_013850858.1
1,116,812	1,116,812	1	C -> A		81	SNP (transversion)	98.8%	Q -> K	790	CAG -> AAG	C770_RS47025	autoinducer	Substitution	8.1E-247	264	1	WP_013850858.1
1,116,931	1,116,931	1	A -> C		79	SNP (transversion)	100.0%		909	GGA -> GGC	C770_RS47025	autoinducer	None	2.0E-261	303	3	WP_013850858.1
1,117,033	1,117,033	1	T -> C		76	SNP (transition)	100.0%		1.011	AAT -> AAC	C770_RS47025	autoinducer	None	4.0E-259	337	3	WP_013850858.1
1,117,053	1,117,053	1	G -> A		80	SNP (transition)	100.0%		1.031	TGA -> TAA	C770_RS47025	autoinducer	None	1.0E-248	344	2	WP_013850858.1
1,117,062	1,117,062	1	T -> C		76	SNP (transition)	100.0%							1.6E-251			
1,117,066	1,117,066	1	C -> G		76	SNP (transversion)	100.0%							1.0E-228			
1,117,082	1,117,082	1	C -> T		77	SNP (transition)	100.0%							2.0E-239			
1,117,087	1,117,087	1	T -> C		77	SNP (transition)	100.0%							2.0E-239			
1,117,099	1,117,099	1	T -> A		78	SNP (transversion)	100.0%							4.0E-258			
1,117,104	1,117,104	1	G -> A		79	SNP (transition)	98.7%							5.0E-264			
1,117,206	1,117,206	1	T -> C		73	SNP (transition)	98.6%		87	CGT -> CGC	C770_RS47030	hypothetical protein	None	1.2E-243	29	3	WP_015243229.1
1,117,239	1,117,239	1	A -> G		82	SNP (transition)	100.0%		120	CCA -> CCG	C770_RS47030	hypothetical protein	None	6.3E-255	40	3	WP_015243229.1
1,117,248	1,117,248	1	C -> G		80	SNP (transversion)	98.8%		129	GTC -> GTG	C770_RS47030	hypothetical protein	None	1.6E-259	43	3	WP_015243229.1
1,117,362	1,117,362	1	G -> C		89	SNP (transversion)	100.0%		243	CCG -> CCC	C770_RS47030	hypothetical protein	None	1.6E-285	81	3	WP_015243229.1
1,117,460	1,117,461	2	AC -> GG		95	Substitution	97.9%	D -> G	341	GAC -> GGG	C770_RS47030	hypothetical protein	Substitution	4.5E-276	114	2	WP_015243229.1
1,117,515	1,117,515	1	T -> C		92	SNP (transition)	100.0%		396	TAT -> TAC	C770_RS47030	hypothetical protein	None	2.5E-304	132	3	WP_015243229.1
1,117,565	1,117,565	1	A -> G		87	SNP (transition)	98.9%	N -> S	446	AAC -> AGC	C770_RS47030	hypothetical protein	Substitution	5.5E-274	149	2	WP_015243229.1
1,117,617	1,117,617	1	T -> C		87	SNP (transition)	98.9%		498	CCT -> CCC	C770_RS47030	hypothetical protein	None	2.2E-265	166	3	WP_015243229.1
1,117,663	1,117,663	1	T -> A		78	SNP (transversion)	100.0%	Y -> N	544	TAT -> AAT	C770_RS47030	hypothetical protein	Substitution	4.0E-258	182	1	WP_015243229.1
1,117,698	1,117,698	1	G -> C		79	SNP (transversion)	97.5%		579	CTG -> CTC	C770_RS47030	hypothetical protein	None	1.2E-243	193	3	WP_015243229.1
1,117,815	1,117,815	1	T -> C		94	SNP (transition)	98.9%		696	GGT -> GGC	C770_RS47030	hypothetical protein	None	4.7E-287	232	3	WP_015243229.1
1,117,869	1,117,869	1	C -> A		96	SNP (transversion)	99.0%	N -> K	750	AAC -> AAA	C770_RS47030	hypothetical protein	Substitution	9.6E-284	250	3	WP_015243229.1
1,117,873	1,117,873	1	C -> A		98	SNP (transversion)	99.0%	L -> M	754	CTG -> ATG	C770_RS47030	hypothetical protein	Substitution	3.9E-309	252	1	WP_015243229.1
1,117,887	1,117,887	1	C -> G		100	SNP (transversion)	99.0%		768	GGC -> GGG	C770_RS47030	hypothetical protein	None	7.9E-286	256	3	WP_015243229.1
1,117,893	1,117,893	1	C -> T		103	SNP (transition)	98.1%		774	CCC -> CCT	C770_RS47030	hypothetical protein	None	6.6E-290	258	3	WP_015243229.1
1,117,908	1,117,908	1	T -> C		106	SNP (transition)	99.1%		789	GAT -> GAC	C770_RS47030	hypothetical protein	None	0.0	263	3	WP_015243229.1
1,117,922	1,117,922	1	C -> A		107	SNP (transversion)	98.1%	A -> E	803	GCA -> GAA	C770_RS47030	hypothetical protein	Substitution	5.7E-312	268	2	WP_015243229.1
1,117,932	1,117,932	1	C -> T		107	SNP (transition)	98.1%		813	CGC -> CGT	C770_RS47030	hypothetical protein	None	0.0	271	3	WP_015243229.1
1,117,980	1,117,980	1	A -> G		114	SNP (transition)	99.1%		861	ACA -> ACG	C770_RS47030	hypothetical protein	None	0.0	287	3	WP_015243229.1
1,117,989	1,117,989	1	A -> C		112	SNP (transversion)	98.2%		870	CCA -> CCC	C770_RS47030	hypothetical protein	None	0.0	290	3	WP_015243229.1
1,118,001	1,118,001	1	T -> C		108	SNP (transition)	100.0%		882	ATT -> ATC	C770_RS47030	hypothetical protein	None	0.0	294	3	WP_015243229.1
1,118,004	1,118,004	1	T -> C		105	SNP (transition)	98.1%		885	CGT -> CGC	C770_RS47030	hypothetical protein	None	0.0	295	3	WP_015243229.1
1,118,034	1,118,034	1	C -> G		107	SNP (transversion)	100.0%		915	GCC -> GCG	C770_RS47030	hypothetical protein	None	0.0	305	3	WP_015243229.1
1,118,040	1,118,040	1	G -> C		105	SNP (transversion)	99.0%		921	GCG -> GCC	C770_RS47030	hypothetical protein	None	4.2E-321	307	3	WP_015243229.1
1,118,073	1,118,073	1	C -> T		107	SNP (transition)	99.1%		954	GCC -> GCT	C770_RS47030	hypothetical protein	None	1.1E-316	318	3	WP_015243229.1

1,118,104	1,118,105	2	AC -> CG	104 -> 105	Substitution	97.1%	T -> R	985	ACA -> CGA	C770_RS47030	hypothetical protein	Substitution	0.0	329	1	WP_015243229.1
1,118,109	1,118,109	1	C -> T	102	SNP (transition)	99.0%		990	AAC -> AAT	C770_RS47030	hypothetical protein	None	0.0	330	3	WP_015243229.1
1,118,112	1,118,112	1	G -> A	100	SNP (transition)	100.0%		993	GCG -> GCA	C770_RS47030	hypothetical protein	None	1.0E-320	331	3	WP_015243229.1
1,118,129	1,118,129	1	C -> A	100	SNP (transversion)	100.0%	P -> Q	1.01	CCG -> CAG	C770_RS47030	hypothetical protein	Substitution	0.0	337	2	WP_015243229.1
1,118,149	1,118,151	2	TAC -> CAT	103	Substitution	98.1%	Y -> H	1.03	TAC -> CAT	C770_RS47030	hypothetical protein	Substitution	6.4E-322	344	1	WP_015243229.1
1,118,220	1,118,220	1	T -> C	87	SNP (transition)	100.0%		1.101	ATT -> ATC	C770_RS47030	hypothetical protein	None	3.2E-305	367	3	WP_015243229.1
1,118,232	1,118,232	1	C -> G	88	SNP (transversion)	98.9%		1.113	CTC -> CTG	C770_RS47030	hypothetical protein	None	1.4E-294	371	3	WP_015243229.1
1,118,247	1,118,247	1	C -> G	86	SNP (transversion)	100.0%		1.128	GCC -> GCG	C770_RS47030	hypothetical protein	None	4.0E-293	376	3	WP_015243229.1
1,118,252	1,118,252	1	C -> T	86	SNP (transition)	98.8%	A -> V	1.133	GCA -> GTA	C770_RS47030	hypothetical protein	Substitution	8.6E-288	378	2	WP_015243229.1
1,118,286	1,118,286	1	T -> C	87	SNP (transition)	98.9%		1.167	ACT -> ACC	C770_RS47030	hypothetical protein	None	5.5E-274	389	3	WP_015243229.1
1,118,304	1,118,304	1	T -> A	89	SNP (transversion)	98.9%	D -> E	1.185	GAT -> GAA	C770_RS47030	hypothetical protein	Substitution	3.5E-289	395	3	WP_015243229.1
1,118,322	1,118,322	1	T -> C	93	SNP (transition)	98.9%		1.203	TAT -> TAC	C770_RS47030	hypothetical protein	None	2.3E-302	401	3	WP_015243229.1
1,118,340	1,118,340	1	G -> C	89	SNP (transversion)	98.9%		1.221	GCG -> GCC	C770_RS47030	hypothetical protein	None	2.2E-280	407	3	WP_015243229.1
1,118,350	1,118,349	0	(A)3 -> (A)4	87	Insertion (tandem repeat)	96.6%		1.231		C770_RS47030	hypothetical protein	Frame Shift	6.7E-273	411	1	WP_015243229.1
1,118,371	1,118,371	1	C -> T	75	SNP (transition)	97.3%							8.8E-253			
1,118,402	1,118,402	1	G -> A	65	SNP (transition)	98.5%							4.1E-210			
1,118,419	1,118,419	1	A -> C	65	SNP (transversion)	96.9%							5.2E-199			
1,118,456	1,118,456	1	C	51	Deletion	98.0%							5.0E-84			
1,118,461	1,118,461	1	A -> G	51	SNP (transition)	98.0%							5.1E-169			
1,118,475	1,118,475	1	A -> G	50	SNP (transition)	98.0%							1.6E-170			
1,118,480	1,118,480	1	A -> G	50	SNP (transition)	98.0%							7.9E-156			
1,118,489	1,118,489	1	C -> T	48	SNP (transition)	97.9%							7.6E-159			
1,118,495	1,118,495	1	A -> G	46	SNP (transition)	97.8%							1.5E-138			
1,118,498	1,118,498	1	C -> T	45	SNP (transition)	97.8%							2.8E-144			
1,118,505	1,118,505	1	G -> C	44	SNP (transversion)	97.7%							5.5E-141			
1,118,509	1,118,509	1	T -> C	42	SNP (transition)	95.2%							8.6E-134			
1,118,513	1,118,513	1	A -> G	42	SNP (transition)	97.6%							3.3E-126			
1,118,517	1,118,517	1	C -> T	41	SNP (transition)	97.6%							4.1E-119			
1,118,520	1,118,521	2	TG -> AC	38 -> 39	Substitution	97.4%							1.6E-124			
1,118,526	1,118,529	4	AAGT -> CGTG	36	Substitution	97.2%							3.6E-104			
1,118,533	1,118,534	2	GG	34	Deletion	97.1%							1.0E-48			
1,118,539	1,118,539	1	A -> G	33	SNP (transition)	97.0%							2.1E-98			
1,118,547	1,118,547	1	G -> C	30	SNP (transversion)	96.7%							4.8E-92			
1,118,558	1,118,558	1	A -> T	28	SNP (transversion)	96.4%							2.8E-80			
1,118,563	1,118,563	1	C -> G	26	SNP (transversion)	96.2%							2.6E-79			
1,118,568	1,118,568	1	A -> C	24	SNP (transversion)	95.8%							2.4E-68			
1,118,575	1,118,576	2	AG -> CA	20 -> 21	Substitution	95.0% -> 95.2%							2.1E-61			
1,135,557	1,135,557	1	C -> T	21	SNP (transition)	95.2%		273	GTC -> GTT	C770_RS47095	polysaccharide ABC trar	None	2.1E-59	91	3	WP_015243240.1
1,135,560	1,135,560	1	C -> A	22	SNP (transversion)	95.5%		276	GCC -> GCA	C770_RS47095	polysaccharide ABC trar	None	1.7E-64	92	3	WP_015243240.1
1,135,563	1,135,563	1	A -> T	24	SNP (transversion)	95.8%		279	CGA -> CGT	C770_RS47095	polysaccharide ABC trar	None	3.0E-75	93	3	WP_015243240.1
1,135,641	1,135,641	1	A -> G	52	SNP (transition)	100.0%		357	CCA -> CCG	C770_RS47095	polysaccharide ABC trar	None	1.0E-156	119	3	WP_015243240.1
1,135,674	1,135,674	1	C -> T	63	SNP (transition)	100.0%		390	CGC -> CGT	C770_RS47095	polysaccharide ABC trar	None	6.3E-215	130	3	WP_015243240.1
1,135,713	1,135,713	1	T -> C	77	SNP (transition)	100.0%		429	TAT -> TAC	C770_RS47095	polysaccharide ABC trar	None	3.2E-270	143	3	WP_015243240.1
1,135,737	1,135,737	1	C -> T	81	SNP (transition)	100.0%		453	GCC -> GCT	C770_RS47095	polysaccharide ABC trar	None	5.0E-268	151	3	WP_015243240.1
1,135,743	1,135,743	1	T -> C	83	SNP (transition)	100.0%		459	GGT -> GGC	C770_RS47095	polysaccharide ABC trar	None	1.3E-274	153	3	WP_015243240.1
1,135,749	1,135,749	1	A -> G	84	SNP (transition)	100.0%		465	TCA -> TCG	C770_RS47095	polysaccharide ABC trar	None	1.0E-294	155	3	WP_015243240.1
1,135,757	1,135,757	1	G -> A	90	SNP (transition)	100.0%	R -> K	473	AGG -> AAG	C770_RS47095	polysaccharide ABC trar	Substitution	1.0E-297	158	2	WP_015243240.1
1,135,764	1,135,764	1	G -> A	93	SNP (transition)	100.0%		480	AAG -> AAA	C770_RS47095	polysaccharide ABC trar	None	1.3E-307	160	3	WP_015243240.1
1,135,782	1,135,782	1	C -> G	96	SNP (transversion)	100.0%	D -> E	498	GAC -> GAG	C770_RS47095	polysaccharide ABC trar	Substitution	0.0	166	3	WP_015243240.1
1,135,827	1,135,827	1	T -> C	87	SNP (transition)	100.0%		543	ACT -> ACC	C770_RS47095	polysaccharide ABC trar	None	7.9E-288	181	3	WP_015243240.1
1,135,840	1,135,840	1	G -> A	84	SNP (transition)	97.6%	D -> N	556	GAC -> AAC	C770_RS47095	polysaccharide ABC trar	Substitution	5.5E-276	186	1	WP_015243240.1
1,135,857	1,135,857	1	G -> C	76	SNP (transversion)	100.0%		573	GGG -> GGC	C770_RS47095	polysaccharide ABC trar	None	4.0E-259	191	3	WP_015243240.1
1,135,879	1,135,879	1	T -> A	66	SNP (transversion)	100.0%	L -> M	595	TTG -> ATG	C770_RS47095	polysaccharide ABC trar	Substitution	4.0E-225	199	1	WP_015243240.1
1,135,893	1,135,893	1	C -> G	60	SNP (transversion)	98.3%	D -> E	609	GAC -> GAG	C770_RS47095	polysaccharide ABC trar	Substitution	1.9E-205	203	3	WP_015243240.1
1,135,905	1,135,905	1	G -> C	55	SNP (transversion)	100.0%	E -> D	621	GAG -> GAC	C770_RS47095	polysaccharide ABC trar	Substitution	3.2E-193	207	3	WP_015243240.1
1,135,912	1,135,912	1	A -> C	54	SNP (transversion)	98.1%	S -> R	628	AGC -> CGC	C770_RS47095	polysaccharide ABC trar	Substitution	6.8E-174	210	1	WP_015243240.1
1,135,934	1,135,934	1	G -> A	45	SNP (transition)	100.0%	R -> K	650	AGG -> AAG	C770_RS47095	polysaccharide ABC trar	Substitution	1.0E-144	217	2	WP_015243240.1
1,135,940	1,135,941	2	AT -> GC	44	Substitution	97.7%	N -> S	656	AAT -> AGC	C770_RS47095	polysaccharide ABC trar	Substitution	1.1E-136	219	2	WP_015243240.1
1,135,944	1,135,943	0	G	41	Insertion	100.0%		660		C770_RS47095	polysaccharide ABC trar	Frame Shift	4.0E-140	220	3	WP_015243240.1
1,135,951	1,135,952	2	TC -> CA	39	Substitution	97.4%	V -> A	8	GTC -> GCA	C770_RS47100	capsule polysaccharide	Substitution	2.5E-128	3	2	WP_015243241.1
1,135,955	1,135,955	1	G -> C	36	SNP (transversion)	100.0%	R -> S	12	AGG -> AGC	C770_RS47100	capsule polysaccharide	Substitution	1.6E-119	4	3	WP_015243241.1
1,135,958	1,135,958	1	C -> G	34	SNP (transversion)	100.0%	N -> K	15	AAC -> AAG	C770_RS47100	capsule polysaccharide	Substitution	6.3E-113	5	3	WP_015243241.1
1,135,961	1,135,961	1	C -> A	33	SNP (transversion)	100.0%	D -> E	18	GAC -> GAA	C770_RS47100	capsule polysaccharide	Substitution	6.3E-113	6	3	WP_015243241.1
1,135,967	1,135,967	1	G -> A	33	SNP (transition)	100.0%		24	GCG -> GCA	C770_RS47100	capsule polysaccharide	None	3.2E-116	8	3	WP_015243241.1
1,135,971	1,135,971	1	G -> A	33	SNP (transition)	100.0%	D -> N	28	GAC -> AAC	C770_RS47100	capsule polysaccharide	Substitution	3.2E-116	10	1	WP_015243241.1
1,135,975	1,135,975	1	G -> A	33	SNP (transition)	100.0%	R -> K	32	AGG -> AAG	C770_RS47100	capsule polysaccharide	Substitution	1.3E-109	11	2	WP_015243241.1
1,135,977	1,135,977	1	T -> C	32	SNP (transition)	100.0%	S -> P	34	TCT -> CCT	C770_RS47100	capsule polysaccharide	Substitution	6.3E-100	12	1	WP_015243241.1
1,135,981	1,135,983	3	GTT -> ACC	28 -> 29	Substitution	100.0%	GS -> DP	38	GGT,TCC -> GAC,	C770_RS47100	capsule polysaccharide	Substitution	1.6E-93	13	2	WP_015243241.1
1,135,987	1,135,987	1	T -> C	26	SNP (transition)	100.0%	V -> A	44	GTG -> GCG	C770_RS47100	capsule polysaccharide	Substitution	4.0E-89	15	2	WP_015243241.1
1,135,991	1,135,990	0	GGT	24	Insertion	100.0%	H -> QV	48	CAC -> CAG,GTC	C770_RS47100	capsule polysaccharide	Insertion	1.6E-77	16	3	WP_015243241.1
1,135,999	1,136,003	5	GAAAC -> AGCCC	22 -> 24	Substitution	100.0%	RN -> KP	56	AGA,AAC -> AAG	C770_RS47100	capsule polysaccharide	Substitution	1.3E-76	19	2	WP_015243241.1
1,136,005	1,136,009	5	CTGCG -> AC	20 -> 21	Deletion	95.0% -> 95.2%	TA -> N	62	ACT,GCG -> AAC	C770_RS47100	capsule polysaccharide	Deletion	1.0E-32	21	2	WP_015243241.1
1,136,012	1,136,012	1	T -> C	19	SNP (transition)	100.0%		69	ACT -> ACC	C770_RS47100	capsule polysaccharide	None	1.0E-57	23	3	WP_015243241.1
1,136,018	1,136,018	1	(A)3 -> (A)2	17	Deletion (tandem repeat)	100.0%		75		C770_RS47100	capsule polysaccharide	Frame Shift	1.6E-24	25	3	WP_015243241.1
1,136,022	1,136,024	3	GAT -> CACG	16 -> 17	Insertion	100.0%		79		C770_RS47100	capsule polysaccharide	Frame Shift	2.5E-50	27	1	WP_015243241.1
1,136,027	1,136,027	1	C -> G	16	SNP (transversion)	100.0%		84	GCC -> GCG	C770_RS47100	capsule polysaccharide	None	1.6E-53	28	3	WP_015243241.1
1,136,030	1,136,030	1	G -> A	16	SNP (transition)	100.0%		87	AAG -> AAA	C770_RS47100	capsule polysaccharide	None	2.5E-50	29	3	WP_015243241.1
1,136,036	1,136,037	2	GA -> AG	15	Substitution	100.0%	KS -> KG	93	AAG,AGC -> AAA	C770_RS47100	capsule polysaccharide	Substitution	3.2E-47	31	3	WP_015243241.1
1,136,042	1,136,042	1	T -> C	14	SNP (transition)	100.0%		99	ATT -> ATC	C770_RS47100	capsule polysaccharide	None	4.0E-44	33	3	WP_015243241.1
1,136,045	1,136,048	3	CATA -> GGTC	12	Substitution	100.0%	AI -> AV	102	GCC,ATA -> GCG,	C770_RS47100	capsule polysaccharide	Substitution	6.3E-32	34	3	WP_015243241.1
1,136,051	1,136,051	1	G -> C	10	SNP (transversion)	100.0%		108	CTG -> CTC	C770_RS47100	capsule polysaccharide	None	1.0E-28	36	3	WP_015243241.1
1,136,054	1,136,054	1	A -> G	7	SNP (transition)	100.0%		111	GAA -> GAG	C770_RS47100	capsule polysaccharide	None	6.3E-19	37	3	WP_015243241.1
1,136,057	1,136,058	2	TT -> AC	6	Substitution	100.0%		114	GGT,TTG -> GGA	C770_RS47100	capsule polysaccharide	None	2.5E-19	38	3	WP_015243241.1
1,138,244	1,138,244	1	T -> G	6	SNP (transversion)	100.0%							2.5E-19			
1,138,247	1,138,248	2	CG -> AA	6 -> 7	Substitution	100.0%							6.3E-17			
1,138,250	1,138,250	1	A -> T	7	SNP (transversion)	100.0%							1.0E-21			
1,138,252	1,138,253	2	GA -> CT	8	Substitution	100.0%							1.0E-24			
1,138,257	1,138,257	1	T -> C	12	SNP (transition)	100.0%							4.0E-33			
1,138,259	1,138,259	1	T -> G	14	SNP (transversion)	100.0%							6.3E-40			
1,138,261	1,138,262	2	GA -> AT	16 -> 17	Substitution	100.0%							4.0E-47			
1,138,265	1,138,265	1	C -> G	18	SNP (transversion)	100.0%							4.0E-51			
1,138,268	1,138,268	1	T -> A	18	SNP (transversion)	100.0%							1.6E-56			
1,138,270	1,138,272	3	CGC -> AA	18	Deletion	100.0%							6.3E-26			
1,138,275	1,138,276	2	TG -> CT	18	Substitution	100.0%							2.5E-49			
1,138,278	1,138,278	1	C -> T	18	SNP (transition)	100.0%							1.0E-45			
1,138,283	1,138,285	3	CTG -> GA	19 -> 20	Deletion	100.0%							2.5E-27			
1,138,304	1,138,304	1	A -> G	27	SNP (transition)	96.3%							2.7E-77			
1,138,308	1,138,308	1	A -> G	29	SNP (transition)	100.0%							7.9E-85			
1,138,326	1,138,326	1	T -> A	57	SNP (transversion)	96.5%							1.6E-162			
1,138,330	1,138,330	1	G -> T	59	SNP (transversion)	96.6%							6.8E-180			
1,141,148	1,141,148	1	T -> A	55	SNP (transversion)	96.4%							3.0E-151			
1,141,152	1,141,152	1	G -> T	49	SNP (transversion)	98.0%							4.9E-143			
1,141,158	1,141,158	1	A -> G	42	SNP (transition)	100.0%							1.6E-122			
1,141,160	1,141,160	1	A	38	Deletion	97.4%							5.8E-51			
1,141,167	1,141,169	3	GAA -> CTT	31 -> 32	Substitution	96.8% -> 96.9%							3.1E-83			
1,141,173	1,141,172	0	C	25	Insertion	96.0%							1.6E-66			
1,141,174	1,141,177	4	TCGA -> ATCC	20 -> 25	Substitution	92.0% -> 95.7%							2.8E-60			
1,141,179	1,141,179	1	A -> G	20	SNP (transition)	100.0%							1.0E-60			
1,141,183	1,141,183	1	G -> A	18	SNP (transition)	100.0%							6.3E-53			
1,141,185	1,141,186	2	AT -> GC	17	Substitution	100.0%							1.0E-51			
1,141,188	1,141,189	2	CC -> TT	17	Substitution	100.0%							1.0E-51			
1,141,193	1,141,196	4	GGGC -> CTTG	16	Substitution	100.0%							6.3E-44			
1,141,198	1,141,198	1	A -> G	13	SNP (transition)	100.0%							1.0E-39			
1,141,200	1,141,200	1	C -> G	13	SNP (transversion)	100.0%							2.0E-38			
1,141,202	1,141,204	3	CGC -> ATG	12	Substitution	100.0%							6.3E-38			
1,141,206	1,141,210	5	GGCGA -> CCTTT	8 -> 11	Substitution	100.0%							2.5E-22			
1,141,214	1,141,216	3	CTC -> AAA	6 -> 7	Substitution	100.0%							4.0E-18			
1,150,059	1,150,060	2	GG -> AA	10 -> 11	Substitution	100.0%	GP -> GS	9	GGC,CCG -> GGT	C770_RS47165	capsule polysaccharide	Substitution	1.0E-30	3	3	WP_015243251.1
1,150,100	1,150,100	1	T -> C	25	SNP (transition)	100.0%	T -> A	742	ACC -> GCC	C770_RS47170	polysaccharide ABC trar	Substitution	1.0E-75	248	1	WP_015243252.1
1,150,107	1,150,107	1	C -> A	28	SNP (transversion)	100.0%		735	CTG -> CTT	C770_RS47170	polysaccharide ABC trar	None	1.0E-84	245	3	WP_015243252.1
1,150,125	1,150,125	1	G -> A	31	SNP (transition)	100.0%		717	TTC -> TTT	C770_RS47170	polysaccharide ABC trar	None	7.9E-97	239	3	WP_015243252.1
1,150,140	1,150,142	2	GAC -> AAA	34 -> 35	Substitution	100.0%	V -> F	700	GTC -> TTT	C770_RS47170	polysaccharide ABC trar	Substitution	1.0E-119	234	1	WP_015243252.1
1,150,151	1,150,151	1	C -> G	38	SNP (transversion)	100.0%	V -> L	691	GTG -> CTG	C770_RS47170	polysaccharide ABC trar	Substitution	2.5E-122	231	1	WP_015243252.1
1,150,164	1,150,164	1	C -> G	41	SNP (transversion)	100.0%		678	CTG -> CTC	C770_RS47170	polysaccharide ABC trar	None	4.0E-140	226	3	WP_015243252.1
1,150,168	1,150,171	4	AAAT -> CCGG	41 -> 42	Substitution	97.6%	DL -> AG	671	GAT,TTG -> GCC,	C770_RS47170	polysaccharide ABC trar	Substitution	6.3E-132	224	2	WP_015243252.1
1,150,173	1,150,175	2	GGT -> TGC	42 -> 45	Substitution	97.6% -> 97.8%	T -> A	667	ACC -> GCA	C770_RS47170	polysaccharide ABC trar	Substitution	3.2E-144	223	1	WP_015243252.1
1,150,179	1,150,179	1	G -> A	49	SNP (transition)	100.0%		663	TAC -> TAT	C770_RS47170	polysaccharide ABC trar	None	2.0E-162	221	3	WP_015243252.1
1,150,191	1,150,191	1	G -> A	54	SNP (transition)	100.0%		651	ATC -> ATT	C770_RS47170	polysaccharide ABC trar	None	6.3E-179	217	3	WP_015243252.1
1,150,198	1,150,200	3	TGC -> GCG	55 -> 57	Substitution	100.0%	RQ -> RA	642	CGG,CAA -> CGC	C770_RS47170	polysaccharide ABC trar	Substitution	3.2E-182	214	3	WP_015243252.1
1,150,218	1,150,218	1	A -> G	63	SNP (transition)	100.0%		624	CAT -> CAC	C770_RS47170	polysaccharide ABC trar	None	6.3E-215	208	3	WP_015243252.1
1,150,224	1,150,224	1	A -> C	65	SNP (transversion)	100.0%		618	CTT -> CTG	C770_RS47170	polysaccharide ABC trar	None	1.0E-208	206	3	WP_015243252.1
1,150,254	1,150,254	1	A -> G	73	SNP (transition)	98.6%		588	CCT -> CCC	C770_RS47170	polysaccharide ABC trar	None	1.2E-243	196	3	WP_015243252.1
1,150,305	1,150,305	1	T -> C	84	SNP (transition)	100.0%		537	CCA -> CCG	C770_RS47170	polysaccharide ABC trar	None	1.0E-294	179	3	WP_015243252.1
1,150,308	1,150,308	1	G -> C	84	SNP (transversion)	98.8%		534	CGC -> CGG	C770_RS47170	polysaccharide ABC trar	None	2.1E-264	178	3	WP_015243252.1
1,150,316	1,150,316	1	C -> T	88	SNP (transition)	98.9%	V -> I	526	GTC -> ATC	C770_RS47170	polysaccharide ABC trar	Substitution	1.4E-294	176	1	WP_015243252.1
1,150,322	1,150,322	1	T -> C	89	SNP (transition)	98.9%	S -> G	520	AGC -> GGC	C770_RS47170	polysaccharide ABC trar	Substitution	2.2E-280	174	1	WP_015243252.1
1,150,326	1,150,326	1	T -> G	89	SNP (transversion)	98.9%		516	ATA -> ATC	C770_RS47170	polysaccharide ABC trar	None	3.5E-289	172	3	WP_015243252.1
1,150,335	1,150,335	1	G -> A	91	SNP (transition)	98.9%		507	TAC -> TAT	C770_RS47170	polysaccharide ABC trar	None	9.1E-296	169	3	WP_015243252.1
1,150,359	1,150,359	1	G -> C	93	SNP (transversion)	100.0%		483	GTC -> GTG	C770_RS47170	polysaccharide ABC trar	None	1.3E-307	161	3	WP_015243252.1
1,150,368	1,150,368	1	A -> G	89	SNP (transition)	100.0%		474	ATT -> ATC	C770_RS47170	polysaccharide ABC trar	None	2.5E-303	158	3	WP_015243252.1
1,150,386	1,150,386	1	A -> G	87	SNP (transition)	100.0%		456	GGT -> GGC	C770_RS47170	polysaccharide ABC trar	None	7.9E-288	152	3	WP_015243252.1
1,150,389	1,150,389	1	C -> G	86	SNP (transversion)	100.0%		453	CTG -> CTC	C770_RS47170	polysaccharide ABC trar	None	1.6E-284	151	3	WP_015243252.1
1,150,395	1,150,395	1	G -> C	87	SNP (transversion)	100.0%		447	GCC -> GCG	C770_RS47170	polysaccharide ABC trar	None	7.9E-288	149	3	WP_015243252.1
1,150,420	1,150,420	1	G -> A	88	SNP (transition)	98.9%	T -> M	422	ACG -> ATG	C770_RS47170	polysaccharide ABC trar	Substitution	7.0E-286	141	2	WP_015243252.1
1,150,424	1,150,424	1	C -> T	87	SNP (transition)	100.0%	G -> S	418	GGC -> AGC	C770_RS47170	polysaccharide ABC trar	Substitution	7.9E-288	140	1	WP_015243252.1
1,150,430	1,150,430	1	T -> C	87	SNP (transition)	98.9%	I -> V	412	ATC -> GTC	C770_RS47170	polysaccharide ABC trar	Substitution	1.4E-282	138	1	WP_015243252.1
1,150,452	1,150,452	1	C -> G	88	SNP (transversion)	100.0%		390	GGG -> GGC	C770_RS47170	polysaccharide ABC trar	None	6.3E-300	130	3	WP_015243252.1
1,150,463	1,150,463	1	T -> G	90	SNP (transversion)	98.9%	I -> L	379	ATC -> CTC	C770_RS47170	polysaccharide ABC trar	Substitution	1.8E-292	127	1	WP_015243252.1
1,150,472	1,150,472	1	G -> T	89	SNP (transversion)	100.0%	L -> M	370	CTG -> ATG	C770_RS47170	polysaccharide ABC trar	Substitution	2.0E-294	124	1	WP_015243252.1
1,150,491	1,150,491	1	C -> G	80	SNP (transversion)	98.8%		351	CTG -> CTC	C770_RS47170	polysaccharide ABC trar	None	1.6E-259	117	3	WP_015243252.1
1,150,508	1,150,509	2	AT -> GC	80	Substitution	100.0%		333	CAA,TTG -> CAG,	C770_RS47170	polysaccharide ABC trar	None	1.0E-280	111	3	WP_015243252.1
1,150,545	1,150,545	1	G -> A	80	SNP (transition)	96.3%		297	ACC -> ACT	C770_RS47170	polysaccharide ABC trar	None	6.5E-250	99	3	WP_015243252.1
1,150,554	1,150,554	1	G -> A	85	SNP (transition)	98.8%		288	CCC -> CCT	C770_RS47170	polysaccharide ABC trar	None	5.4E-276	96	3	WP_015243252.1
1,150,569	1,150,569	1	C -> G	87	SNP (transversion)	98.9%		273	GCG -> GCC	C770_RS47170	polysaccharide ABC trar	None	1.4E-282	91	3	WP_015243252.1
1,150,582	1,150,582	1	C -> T	88	SNP (transition)	98.9%	R -> K	260	AGG -> AAG	C770_RS47170	polysaccharide ABC trar	Substitution	3.5E-277	87	2	WP_015243252.1
1,150,587	1,150,587	1	G -> C	86	SNP (transversion)	100.0%		255	ACC -> ACG	C770_RS47170	polysaccharide ABC trar	None	6.3E-276	85	3	WP_015243252.1
1,150,602	1,150,604	3	CGA -> GCC	89 -> 90	Substitution	97.8%	S -> G	238	TCG -> GGC	C770_RS47170	polysaccharide ABC trar	Substitution	1.3E-276	80	1	WP_015243252.1
1,150,717	1,150,717	1	G -> A	80	SNP (transition)	98.8%	A -> V	125	GCG -> GTG	C770_RS47170	polysaccharide ABC trar	Substitution	1.6E-259	42	2	WP_015243252.1
1,150,719	1,150,719	1	G -> A	79	SNP (transition)	100.0%		123	GTC -> GTT	C770_RS47170	polysaccharide ABC trar	None	1.6E-253	41	3	WP_015243252.1
1,150,782	1,150,782	1	G -> C	64	SNP (transversion)	100.0%		60	TCC -> TCG	C770_RS47170	polysaccharide ABC trar	None	2.5E-218	20	3	WP_015243252.1

1,150,850	1,150,851	2	CT -> TC		41	Substitution	95.1%							2.1E-130			
1,150,857	1,150,857	1	A -> T		38	SNP (transversion)	97.4%							6.0E-125			
1,150,864	1,150,864	1	G -> C		33	SNP (transversion)	97.0%							8.3E-105			
1,150,866	1,150,866	1	T -> G		33	SNP (transversion)	97.0%							8.3E-105			
1,150,871	1,150,871	1	G		32	Deletion	96.9%							9.8E-46			
1,150,878	1,150,879	2	CA -> TG		30	Substitution	96.7%							7.5E-98			
1,150,912	1,150,914	3	TCG -> CGC		24	Substitution	95.8%							3.0E-75			
1,150,916	1,150,916	1	G -> T		23	SNP (transversion)	95.7%							3.6E-74			
1,150,919	1,150,919	1	C -> G		21	SNP (transversion)	95.2%							2.1E-67			
1,150,922	1,150,922	1	G -> C		21	SNP (transversion)	95.2%							2.1E-61			
1,150,940	1,150,940	1	A -> C		23	SNP (transversion)	95.7%							1.5E-67			
1,150,948	1,150,948	1	A -> C		21	SNP (transversion)	95.2%							2.1E-59			
1,151,004	1,151,004	1	T -> C		24	SNP (transition)	95.8%							1.2E-70			
1,151,027	1,151,027	1	T -> C		31	SNP (transition)	96.8%							3.1E-92			
1,151,071	1,151,071	1	G -> A		46	SNP (transition)	95.7%							2.6E-125			
1,151,073	1,151,072	0	A		46	Insertion	95.7%							1.6E-138			
1,151,132	1,151,132	1	T -> C		66	SNP (transition)	97.0%							3.4E-202			
1,151,145	1,151,145	1	C -> G		68	SNP (transversion)	95.6%		792	GGG -> GGC	C770_RS47175	Ketopantoate hydroxyn	None	1.6E-210	264	3	WP_015243253.1
1,151,226	1,151,226	1	A -> G		78	SNP (transition)	98.7%		711	CGT -> CGC	C770_RS47175	Ketopantoate hydroxyn	None	1.2E-260	237	3	WP_015243253.1
1,151,268	1,151,268	1	T -> C		80	SNP (transition)	98.8%		669	CAA -> CAG	C770_RS47175	Ketopantoate hydroxyn	None	1.6E-259	223	3	WP_015243253.1
1,151,280	1,151,280	1	G -> A		81	SNP (transition)	98.8%		657	GAC -> GAT	C770_RS47175	Ketopantoate hydroxyn	None	8.1E-271	219	3	WP_015243253.1
1,151,283	1,151,283	1	A -> G		80	SNP (transition)	98.8%		654	TAT -> TAC	C770_RS47175	Ketopantoate hydroxyn	None	3.2E-283	218	3	WP_015243253.1
1,151,304	1,151,304	1	A -> G		82	SNP (transition)	98.8%		633	TAT -> TAC	C770_RS47175	Ketopantoate hydroxyn	None	3.3E-274	211	3	WP_015243253.1
1,151,325	1,151,325	1	G -> A		77	SNP (transition)	98.7%		612	TAC -> TAT	C770_RS47175	Ketopantoate hydroxyn	None	3.1E-257	204	3	WP_015243253.1
1,151,340	1,151,340	1	G -> A		73	SNP (transition)	98.6%		597	GCC -> GCT	C770_RS47175	Ketopantoate hydroxyn	None	1.2E-243	199	3	WP_015243253.1
1,151,388	1,151,388	1	C -> T		70	SNP (transition)	100.0%		549	GGG -> GGA	C770_RS47175	Ketopantoate hydroxyn	None	1.0E-231	183	3	WP_015243253.1
1,151,400	1,151,400	1	C -> A		64	SNP (transversion)	100.0%		537	TCG -> TCT	C770_RS47175	Ketopantoate hydroxyn	None	2.5E-218	179	3	WP_015243253.1
1,151,403	1,151,403	1	C -> A		63	SNP (transversion)	100.0%		534	CTG -> CTT	C770_RS47175	Ketopantoate hydroxyn	None	6.3E-215	178	3	WP_015243253.1
1,151,419	1,151,419	1	C -> G		63	SNP (transversion)	100.0%	S -> T	518	AGC -> ACC	C770_RS47175	Ketopantoate hydroxyn	Substitution	2.5E-202	173	2	WP_015243253.1
1,151,451	1,151,451	1	G -> C		70	SNP (transversion)	98.6%		486	CCC -> CCG	C770_RS47175	Ketopantoate hydroxyn	None	8.8E-213	162	3	WP_015243253.1
1,151,457	1,151,457	1	C -> G		75	SNP (transversion)	98.7%		480	GTG -> GTC	C770_RS47175	Ketopantoate hydroxyn	None	1.2E-235	160	3	WP_015243253.1
1,151,500	1,151,500	1	T -> C		81	SNP (transition)	100.0%	K -> R	437	AAG -> AGG	C770_RS47175	Ketopantoate hydroxyn	Substitution	1.0E-243	146	2	WP_015243253.1
1,151,502	1,151,502	1	G -> C		82	SNP (transversion)	100.0%		435	ACC -> ACG	C770_RS47175	Ketopantoate hydroxyn	None	4.0E-263	145	3	WP_015243253.1
1,151,516	1,151,516	1	C -> T		83	SNP (transition)	96.4%	V -> I	421	GTC -> ATC	C770_RS47175	Ketopantoate hydroxyn	Substitution	9.2E-244	141	1	WP_015243253.1
1,151,544	1,151,544	1	A -> G		76	SNP (transition)	97.4%		393	GTT -> GTC	C770_RS47175	Ketopantoate hydroxyn	None	1.8E-241	131	3	WP_015243253.1
1,151,619	1,151,619	1	G -> C		74	SNP (transversion)	100.0%	D -> E	318	GAC -> GAG	C770_RS47175	Ketopantoate hydroxyn	Substitution	4.0E-230	106	3	WP_015243253.1
1,151,633	1,151,634	2	GC -> TG	75 -> 76		Substitution	98.7%		303	GTG,CGG -> GTC	C770_RS47175	Ketopantoate hydroxyn	None	4.7E-243	101	3	WP_015243253.1
1,151,643	1,151,643	1	A -> G		78	SNP (transition)	100.0%		294	CTT -> CTC	C770_RS47175	Ketopantoate hydroxyn	None	4.0E-258	98	3	WP_015243253.1
1,151,664	1,151,664	1	C -> G		75	SNP (transversion)	98.7%		273	GCG -> GCC	C770_RS47175	Ketopantoate hydroxyn	None	1.2E-235	91	3	WP_015243253.1
1,151,667	1,151,667	1	A -> G		74	SNP (transition)	98.6%		270	GAT -> GAC	C770_RS47175	Ketopantoate hydroxyn	None	1.9E-232	90	3	WP_015243253.1
1,151,736	1,151,736	1	C -> T		80	SNP (transition)	96.3%		201	GAG -> GAA	C770_RS47175	Ketopantoate hydroxyn	None	1.6E-234	67	3	WP_015243253.1
1,151,775	1,151,775	1	C -> G		67	SNP (transversion)	97.0%		162	CGG -> CGC	C770_RS47175	Ketopantoate hydroxyn	None	7.0E-212	54	3	WP_015243253.1
1,151,784	1,151,784	1	G -> C		70	SNP (transversion)	100.0%		153	CCC -> CCG	C770_RS47175	Ketopantoate hydroxyn	None	1.0E-224	51	3	WP_015243253.1
1,151,808	1,151,808	1	A -> G		64	SNP (transition)	100.0%		129	TCT -> TCC	C770_RS47175	Ketopantoate hydroxyn	None	6.3E-212	43	3	WP_015243253.1
1,151,823	1,151,823	1	G -> C		66	SNP (transversion)	100.0%		114	CGC -> CGG	C770_RS47175	Ketopantoate hydroxyn	None	4.0E-225	38	3	WP_015243253.1
1,151,838	1,151,838	1	G -> A		63	SNP (transition)	96.8%		99	GCC -> GCT	C770_RS47175	Ketopantoate hydroxyn	None	1.2E-192	33	3	WP_015243253.1
1,151,853	1,151,853	1	C -> G		65	SNP (transversion)	100.0%		84	CTG -> CTC	C770_RS47175	Ketopantoate hydroxyn	None	1.0E-208	28	3	WP_015243253.1
1,151,856	1,151,856	1	C -> T		66	SNP (transition)	95.5%		81	ACG -> ACA	C770_RS47175	Ketopantoate hydroxyn	None	2.9E-210	27	3	WP_015243253.1
1,151,861	1,151,862	2	CC -> TG	66 -> 67		Substitution	98.5%	VV -> VM	75	GTG,GTG -> GTC	C770_RS47175	Ketopantoate hydroxyn	Substitution	2.1E-213	25	3	WP_015243253.1
1,151,938	1,151,938	1	G -> T		72	SNP (transversion)	97.2%							2.6E-228			
1,151,940	1,151,941	2	TC -> AA		71	Substitution	100.0%							6.3E-228			
1,151,947	1,151,948	2	AG -> GA		72	Substitution	100.0%							4.0E-231			
1,151,958	1,151,957	0	T		74	Insertion	100.0%							4.0E-230			
1,151,962	1,151,962	1	C -> G		74	SNP (transversion)	100.0%							4.0E-230			
1,151,965	1,151,965	1	G -> A		74	SNP (transition)	98.6%							1.9E-232			
1,151,969	1,151,969	1	G -> A		70	SNP (transition)	100.0%							1.0E-217			
1,151,977	1,151,977	1	C -> G		67	SNP (transversion)	97.0%							7.0E-212			
1,151,982	1,151,982	1	G -> C		67	SNP (transversion)	100.0%							1.6E-228			
1,151,984	1,151,984	1	C -> G		67	SNP (transversion)	98.5%							2.7E-223			
1,151,987	1,151,987	1	G -> C		69	SNP (transversion)	100.0%							1.6E-221			
1,151,989	1,151,989	1	A -> C		69	SNP (transversion)	98.6%							4.4E-230			
1,151,991	1,151,991	1	C -> A		69	SNP (transversion)	98.6%							1.1E-209			
1,151,996	1,151,996	1	G -> A		70	SNP (transition)	100.0%							1.0E-224			
1,152,011	1,152,010	0	AAC		74	Insertion	98.6%							1.0E-222			
1,152,014	1,152,014	1	T -> C		73	SNP (transition)	98.6%							1.8E-236			
1,152,042	1,152,043	2	CG -> TT		76	Substitution	98.7%	R -> K	1.201	CGG -> AAG	C770_RS47180	NAD(P)H-nitrite reducta	Substitution	6.3E-244	401	1	WP_015243254.1
1,152,071	1,152,071	1	C -> A		75	SNP (transversion)	98.7%	E -> D	1.173	GAG -> GAT	C770_RS47180	NAD(P)H-nitrite reducta	Substitution	1.2E-235	391	3	WP_015243254.1
1,152,161	1,152,161	1	C -> T		85	SNP (transition)	98.8%		1.083	CTG -> CTA	C770_RS47180	NAD(P)H-nitrite reducta	None	1.3E-267	361	3	WP_015243254.1
1,152,167	1,152,167	1	T -> G		86	SNP (transversion)	98.8%		1.077	GGA -> GGC	C770_RS47180	NAD(P)H-nitrite reducta	None	2.7E-262	359	3	WP_015243254.1
1,152,170	1,152,171	2	CT -> TC	86 -> 87		Substitution	98.8% -> 98.9%	E -> G	1.073	GAG -> GGA	C770_RS47180	NAD(P)H-nitrite reducta	Substitution	2.0E-270	358	2	WP_015243254.1
1,152,175	1,152,175	1	C -> T		86	SNP (transition)	100.0%	G -> S	1.069	GGC -> AGC	C770_RS47180	NAD(P)H-nitrite reducta	Substitution	6.3E-276	357	1	WP_015243254.1
1,152,185	1,152,185	1	A -> G		79	SNP (transition)	100.0%		1.059	TTT -> TTC	C770_RS47180	NAD(P)H-nitrite reducta	None	2.0E-261	353	3	WP_015243254.1
1,152,200	1,152,200	1	T -> C		81	SNP (transition)	98.8%		1.044	GGA -> GGG	C770_RS47180	NAD(P)H-nitrite reducta	None	8.1E-263	348	3	WP_015243254.1
1,152,207	1,152,207	1	T -> C		85	SNP (transition)	100.0%	E -> G	1.037	GAG -> GGG	C770_RS47180	NAD(P)H-nitrite reducta	Substitution	1.0E-289	346	2	WP_015243254.1
1,152,221	1,152,221	1	C -> G		84	SNP (transversion)	100.0%		1.023	GTG -> GTC	C770_RS47180	NAD(P)H-nitrite reducta	None	4.0E-261	341	3	WP_015243254.1
1,152,233	1,152,233	1	T -> C		85	SNP (transition)	100.0%		1.011	GCA -> GCG	C770_RS47180	NAD(P)H-nitrite reducta	None	3.2E-264	337	3	WP_015243254.1
1,152,300	1,152,300	1	C -> T		75	SNP (transition)	100.0%	S -> N	944	AGC -> AAC	C770_RS47180	NAD(P)H-nitrite reducta	Substitution	1.0E-240	315	2	WP_015243254.1
1,152,302	1,152,303	2	GA -> AG		70	Substitution	100.0%	V -> A	941	GTC -> GCT	C770_RS47180	NAD(P)H-nitrite reducta	Substitution	1.0E-238	314	2	WP_015243254.1
1,152,308	1,152,308	1	G -> A		70	SNP (transition)	98.6%		936	ACC -> ACT	C770_RS47180	NAD(P)H-nitrite reducta	None	1.4E-226	312	3	WP_015243254.1
1,152,320	1,152,320	1	A -> G		69	SNP (transition)	97.1%		924	GGT -> GGC	C770_RS47180	NAD(P)H-nitrite reducta	None	3.7E-225	308	3	WP_015243254.1
1,152,326	1,152,326	1	C -> A		66	SNP (transversion)	98.5%		918	CTG -> CTT	C770_RS47180	NAD(P)H-nitrite reducta	None	6.6E-207	306	3	WP_015243254.1
1,152,329	1,152,329	1	A -> G		66	SNP (transition)	100.0%		915	AAT -> AAC	C770_RS47180	NAD(P)H-nitrite reducta	None	6.3E-212	305	3	WP_015243254.1
1,152,338	1,152,338	1	G -> A		64	SNP (transition)	95.3%		906	GCC -> GCT	C770_RS47180	NAD(P)H-nitrite reducta	None	1.7E-203	302	3	WP_015243254.1
1,152,341	1,152,341	1	A -> G		63	SNP (transition)	100.0%		903	CTT -> CTC	C770_RS47180	NAD(P)H-nitrite reducta	None	6.3E-215	301	3	WP_015243254.1
1,152,371	1,152,371	1	G -> A		55	SNP (transition)	96.4%		873	GCC -> GCT	C770_RS47180	NAD(P)H-nitrite reducta	None	1.9E-172	291	3	WP_015243254.1
1,152,374	1,152,374	1	T -> C		55	SNP (transition)	100.0%		870	GAA -> GAG	C770_RS47180	NAD(P)H-nitrite reducta	None	1.0E-187	290	3	WP_015243254.1
1,152,404	1,152,404	1	G -> C		45	SNP (transversion)	97.8%		840	CCC -> CCG	C770_RS47180	NAD(P)H-nitrite reducta	None	1.1E-148	280	3	WP_015243254.1
1,152,437	1,152,437	1	A -> G		37	SNP (transition)	100.0%		807	TAT -> TAC	C770_RS47180	NAD(P)H-nitrite reducta	None	7.9E-123	269	3	WP_015243254.1
1,152,464	1,152,464	1	T -> C		30	SNP (transition)	100.0%		780	AGA -> AGG	C770_RS47180	NAD(P)H-nitrite reducta	None	1.0E-93	260	3	WP_015243254.1
1,152,470	1,152,470	1	G -> A		28	SNP (transition)	100.0%		774	GAC -> GAT	C770_RS47180	NAD(P)H-nitrite reducta	None	1.6E-87	258	3	WP_015243254.1
1,152,485	1,152,485	1	G -> A		25	SNP (transition)	100.0%		759	AAC -> AAT	C770_RS47180	NAD(P)H-nitrite reducta	None	3.2E-78	253	3	WP_015243254.1
1,152,497	1,152,497	1	A -> G		22	SNP (transition)	95.5%		747	CTT -> CTC	C770_RS47180	NAD(P)H-nitrite reducta	None	2.8E-60	249	3	WP_015243254.1
1,152,500	1,152,500	1	T -> C		22	SNP (transition)	100.0%		744	GGA -> GGG	C770_RS47180	NAD(P)H-nitrite reducta	None	1.0E-66	248	3	WP_015243254.1
1,152,518	1,152,518	1	T -> C		11	SNP (transition)	100.0%		726	GAA -> GAG	C770_RS47180	NAD(P)H-nitrite reducta	None	4.0E-38	242	3	WP_015243254.1
1,152,521	1,152,521	1	G -> C		10	SNP (transversion)	100.0%		723	ACC -> ACG	C770_RS47180	NAD(P)H-nitrite reducta	None	1.0E-31	241	3	WP_015243254.1
1,153,306	1,153,307	2	TT -> GA	6 -> 7		Substitution	100.0%							6.3E-17			
1,153,309	1,153,309	1	C -> T		7	SNP (transition)	100.0%							3.2E-18			
1,153,313	1,153,315	3	ACC -> GGA	9 -> 11		Substitution	100.0%							1.3E-19			
1,153,318	1,153,320	3	GCC -> AAT		12	Substitution	100.0%							2.5E-28			
1,153,322	1,153,324	3	CTC -> G		12	Deletion	100.0%							2.5E-16			
1,153,326	1,153,329	4	TCCG -> CTGC	12 -> 13		Substitution	100.0%							1.6E-35			
1,153,332	1,153,332	1	G -> A		14	SNP (transition)	100.0%							6.3E-40			
1,153,339	1,153,339	1	T -> C		18	SNP (transition)	100.0%							6.3E-53			
1,153,341	1,153,341	1	C -> A		18	SNP (transversion)	100.0%							2.5E-49			
1,153,348	1,153,348	1	C -> G		20	SNP (transversion)	100.0%							1.0E-56			
1,153,352	1,153,352	1	C -> T		21	SNP (transition)	95.2%							2.1E-59			
1,153,353	1,153,352	0	A		22	Insertion	95.5%							2.2E-62			
1,153,354	1,153,354	1	T -> C		22	SNP (transition)	100.0%		318	TAA -> TAG	C770_RS47185	Rieske family ferredoxin	None	2.5E-62	106	3	WP_015243255.1
1,153,357	1,153,357	1	A -> G		23	SNP (transition)	100.0%		315	TCT -> TCC	C770_RS47185	Rieske family ferredoxin	None	1.0E-69	105	3	WP_015243255.1
1,153,405	1,153,405	1	C -> T		39	SNP (transition)	100.0%		267	AGG -> AGA	C770_RS47185	Rieske family ferredoxin	None	1.3E-121	89	3	WP_015243255.1
1,153,414	1,153,414	1	A -> G		39	SNP (transition)	100.0%		258	GTT -> GTC	C770_RS47185	Rieske family ferredoxin	None	2.0E-129	86	3	WP_015243255.1
1,153,438	1,153,438	1	C -> T		42	SNP (transition)	100.0%		234	CAG -> CAA	C770_RS47185	Rieske family ferredoxin	None	4.0E-135	78	3	WP_015243255.1
1,153,459	1,153,459	1	A -> G		48	SNP (transition)	97.9%		213	CGT -> CGC	C770_RS47185	Rieske family ferredoxin	None	3.8E-154	71	3	WP_015243255.1
1,153,462	1,153,462	1	A -> G		48	SNP (transition)	100.0%		210	GGT -> GGC	C770_RS47185	Rieske family ferredoxin	None	1.6E-149	70	3	WP_015243255.1
1,153,501	1,153,501	1	T -> C		65	SNP (transition)	100.0%		171	CTA -> CTG	C770_RS47185	Rieske family ferredoxin	None	1.0E-221	57	3	WP_015243255.1
1,153,559	1,153,559	1	T -> C		78	SNP (transition)	100.0%	E -> G	113	GAA -> GGA	C770_RS47185	Rieske family ferredoxin	Substitution	6.3E-266	38	2	WP_015243255.1
1,153,570	1,153,570	1	G -> A		78	SNP (transition)	98.7%		102	AGC -> AGT	C770_RS47185	Rieske family ferredoxin	None	1.2E-260	34	3	WP_015243255.1
1,153,579	1,153,579	1	A -> G		80	SNP (transition)	98.8%		93	GTT -> GTC	C770_RS47185	Rieske family ferredoxin	None	1.6E-259	31	3	WP_015243255.1
1,153,594	1,153,594	1	C -> T		83	SNP (transition)	98.8%		78	GGG -> GGA	C770_RS47185	Rieske family ferredoxin	None	1.3E-277	26	3	WP_015243255.1
1,153,618	1,153,618	1	A -> G		78	SNP (transition)	98.7%		54	GAT -> GAC	C770_RS47185	Rieske family ferredoxin	None	1.2E-260	18	3	WP_015243255.1
1,153,858	1,153,858	1	C -> A		85	SNP (transversion)	100.0%	R -> L	950	CGG -> CTG	C770_RS47190	fatty acid desaturase	Substitution	1.0E-289	317	2	WP_015243256.1
1,154,143	1,154,143	1	C -> T		84	SNP (transition)	100.0%	C -> Y	665	TGT -> TAT	C770_RS47190	fatty acid desaturase	Substitution	6.3E-278	222	2	WP_015243256.1
1,154,961	1,154,961	1	A -> T		66	SNP (transversion)	100.0%							1.6E-218			
1,154,972	1,154,972	1	G -> A		62	SNP (transition)	100.0%							1.0E-217			
1,155,000	1,155,000	1	T -> G		69	SNP (transversion)	100.0%							2.5E-235			
1,155,028	1,155,028	1	G -> C		69	SNP (transversion)	100.0%		15	CCG -> CCC	C770_RS47195	sugar ABC transporter s	None	1.0E-207	5	3	WP_015243257.1
1,155,397	1,155,397	1	A -> G		99	SNP (transition)	100.0%		384	GCA -> GCG	C770_RS47195	sugar ABC transporter s	None	0.0	128	3	WP_015243257.1
1,155,721	1,155,721	1	T -> C		88	SNP (transition)	98.9%		708	ATT -> ATC	C770_RS47195	sugar ABC transporter s	None	1.4E-294	236	3	WP_015243257.1
1,156,075	1,156,075	1	C -> T		81	SNP (transition)	100.0%							5.0E-268			
1,156,080	1,156,081	2	AA -> TT	83 -> 84		Substitution	98.8%							2.5E-266			
1,156,198	1,156,198	1	G -> A		76	SNP (transition)	100.0%		69	CCG -> CCA	C770_RS47200	outer membrane bioger	None	1.6E-251	23	3	WP_015243258.1
1,156,239	1,156,239	1	A -> G		78	SNP (transition)	100.0%	Q -> R	110	CAG -> CGG	C770_RS47200	outer membrane bioger	Substitution	2.5E-250	37	2	WP_015243258.1
1,156,374	1,156,374	1	C -> A		87	SNP (transversion)	98.9%	A -> D	245	GCT -> GAT	C770_RS47200	outer membrane bioger	Substitution	2.2E-265	82	2	WP_015243258.1
1,156,466	1,156,466	1	G -> A		84	SNP (transition)	100.0%	E -> K	337	GAA -> AAA	C770_RS47200	outer membrane bioger	Substitution	6.3E-278	113	1	WP_015243258.1
1,156,512	1,156,512	1	A -> T		95	SNP (transversion)	100.0%	N -> I	383	AAT -> ATT	C770_RS47200	outer membrane bioger	Substitution	3.2E-314	128	2	WP_015243258.1
1,156,673	1,156,673	1	A -> G		101	SNP (transition)	100.0%	I -> V	544	ATC -> GTC	C770_RS47200	outer membrane bioger	Substitution	0.0	182	1	WP_015243258.1
1,156,683	1,156,683	1	A -> C		101	SNP (transversion)	99.0%	E -> A	554	GAG -> GCG	C770_RS47200	outer membrane bioger	Substitution	0.0	185	2	WP_015243258.1
1,156,709	1,156,709	1	A -> G		98	SNP (transition)	100.0%	M -> V	580	ATG -> GTG	C770_RS47200	outer membrane bioger	Substitution	4.9E-324	194	1	WP_015243258.1
1,156,727	1,156,727	1	G -> A		96	SNP (transition)	99.0%	E -> K	598	GAG -> AAG	C770_RS47200	outer membrane bioger	Substitution	3.0E-293	200	1	WP_015243258.1
1,156,738	1,156,738	1	C -> T		98	SNP (transition)	100.0%		609	TTC -> TTT	C770_RS47200	outer membrane bioger	None	2.5E-314	203	3	WP_015243258.1
1,156,747	1,156,747	1	G -> A		98	SNP (transition)	100.0%		618	TCG -> TCA	C770_RS47200	outer membrane bioger	None	2.5E-314	206	3	WP_015243258.1
1,156,793	1,156,793	1	G -> A		90	SNP (transition)	98.9%	A -> T	664	GCC -> ACC	C770_RS47200	outer membrane bioger	Substitution	1.8E-292	222	1	WP_015243258.1
1,156,797	1,156,797	1	T -> C		91	SNP (transition)	100.0%	V -> A	668	GTG -> GCG	C770_RS47200	outer membrane bioger	Substitution	4.0E-310	223	2	WP_015243258.1
1,156,810	1,156,810	1	C -> G		90	SNP (transversion)	97.8%		681	CCC -> CCG	C770_RS47200	outer membrane bioger	None	1.6E-287	227	3	WP_015243258.1
1,156,825	1,156,826	2	GA -> CC		90	Substitution	98.9%	GM -> GL	696	GGG,ATG -> GG(	C770_RS47200	outer membrane bioger	Substitution	1.8E-292	232	3	WP_015243258.1
1,156,829	1,156,829	1	T -> A		90	SNP (transversion)	97.8%	S -> T	700	TCC -> ACC	C770_RS47200	outer membrane bioger	Substitution	1.6E-287	234	1	WP_015243258.1
1,156,833	1,156,833	1	G -> A		90	SNP (transition)	97.8%	S -> N	704	AGC -> AAC	C770_RS47200	outer membrane bioger	Substitution	1.6E-287	235	2	WP_015243258.1
1,156,835	1,156,837	2	CTG -> TTC	89 -> 90		Substitution	97.8%	L -> F	706	CTG -> TTC	C770_RS47200	outer membrane bioger	Substitution	2.0E-249	236	1	WP_015243258.1
1,156,839	1,156,839	1	C -> A		89	SNP (transversion)	97.8%	A -> E	710	GCG -> GAG	C770_RS47200	outer membrane bioger	Substitution	3.1E-284	237	2	WP_015243258.1
1,156,870	1,156,870	1	C -> T		85	SNP (transition)	100.0%		741	AAC -> AAT	C770_RS47200	outer membrane bioger	None	1.0E-272	247	3	WP_015243258.1
1,156,897	1,156,897	1	A -> G		81	SNP (transition)	100.0%		768	GAA -> GAG	C770_RS47200	outer membrane bioger	None	4.0E-276	256	3	WP_015243258.1
1,156,922	1,156,922	1	G -> A		73	SNP (transition)	97.3%	G -> S	793	GGC -> AGC	C770_RS47200	outer membrane bioger	Substitution	1.3E-231	265	1	WP_015243258.1
1,156,930	1,156,930	1	C -> T		75	SNP (transition)	100.0%		801	AAC -> AAT	C770_RS47200	outer membrane bioger	None	1.0E-255	267	3	WP_015243258.1
1,156,933	1,156,933	1	G -> C		75	SNP (transversion)	100.0%		804	GCG -> GCC	C770_RS47200	outer membrane bioger	None	1.0E-255	268	3	WP_015243258.1
1,156,954	1,156,954	1	T -> G		71	SNP (transversion)	98.6%		825	CGT -> CGG	C770_RS47200	outer membrane bioger	None	7.1E-237	275	3	WP_015243258.1
1,156,960	1,156,960	1	C -> G		71	SNP (transversion)	100.0%		831	CGC -> CGG	C770_RS47200	outer membrane bioger	None	6.3E-228	277	3	WP_015243258.1
1,157,013	1,157,013	1	C -> T		68	SNP (transition)	100.0%	A -> V	884	GCC -> GTC	C770_RS47200	outer membrane bioger	Substitution	4.0E-225	295	2	WP_015243258.1
1,157,015	1,157,015	1	C -> G		69	SNP (transversion)	100.0%	P -> A	886	CCG -> GCG	C770_RS47200	outer membrane bioger	Substitution	2.5E-235	296	1	WP_015243258.1
1,157,033	1,157,033	1	T -> A		64	SNP (transversion)	100.0%	S -> T	904	TCG -> ACG	C770_RS47200	outer membrane bioger	Substitution	1.6E-205	302	1	WP_015243258.1
1,157,037	1,157,037	1	G -> A		64	SNP (transition)	100.0%	G -> E	908	GGA -> GAA	C770_RS47200	outer membrane bioger	Substitution	2.5E-186	303	2	WP_015243258.1

1,157,067	1,157,067	1	A -> C	65	SNP (transversion)	100.0%	E -> A	938	GAG -> GCG	C770_RS47200	outer membrane bioger	Substitution	3.2E-215	313	2	WP_015243258.1
1,157,095	1,157,095	1	C -> T	68	SNP (transition)	100.0%		966	GAC -> GAT	C770_RS47200	outer membrane bioger	None	6.3E-232	322	3	WP_015243258.1
1,157,123	1,157,123	1	C -> T	66	SNP (transition)	100.0%		994	CTG -> TTG	C770_RS47200	outer membrane bioger	None	1.6E-218	332	1	WP_015243258.1
1,157,126	1,157,127	2	GT -> AC	66	Substitution	98.5%	V -> T	997	GTC -> ACC	C770_RS47200	outer membrane bioger	Substitution	2.5E-205	333	1	WP_015243258.1
1,157,131	1,157,131	1	C -> T	66	SNP (transition)	100.0%		1.002	CTC -> CTT	C770_RS47200	outer membrane bioger	None	6.3E-212	334	3	WP_015243258.1
1,157,155	1,157,155	1	T -> G	66	SNP (transversion)	100.0%		1.026	GTT -> GTG	C770_RS47200	outer membrane bioger	None	6.3E-212	342	3	WP_015243258.1
1,157,158	1,157,158	1	C -> T	66	SNP (transition)	100.0%		1.029	TCC -> TCT	C770_RS47200	outer membrane bioger	None	6.3E-212	343	3	WP_015243258.1
1,157,161	1,157,161	1	T -> C	67	SNP (transition)	100.0%		1.032	TTT -> TTC	C770_RS47200	outer membrane bioger	None	1.6E-228	344	3	WP_015243258.1
1,157,166	1,157,167	2	CC -> TG	67 -> 68	Substitution	98.5%	A -> V	1.037	GCC -> GTG	C770_RS47200	outer membrane bioger	Substitution	6.8E-200	346	2	WP_015243258.1
1,157,169	1,157,170	2	CA -> GG	69	Substitution	100.0%	A -> G	1.04	GCA -> GGG	C770_RS47200	outer membrane bioger	Substitution	1.0E-207	347	2	WP_015243258.1
1,157,173	1,157,173	1	C -> G	69	SNP (transversion)	100.0%		1.044	CTC -> CTG	C770_RS47200	outer membrane bioger	None	2.0E-228	348	3	WP_015243258.1
1,157,179	1,157,179	1	C -> T	70	SNP (transition)	100.0%		1.05	GAC -> GAT	C770_RS47200	outer membrane bioger	None	1.0E-231	350	3	WP_015243258.1
1,157,182	1,157,183	2	GG -> AA	69	Substitution	98.6%	GE -> GK	1.053	GGG,GAG -> GG/	C770_RS47200	outer membrane bioger	Substitution	2.7E-223	351	3	WP_015243258.1
1,157,189	1,157,191	2	GCT -> ACA	67 -> 68	Substitution	97.0% -> 97.1%	A -> T	1.06	GCT -> ACA	C770_RS47200	outer membrane bioger	Substitution	1.0E-221	354	1	WP_015243258.1
1,157,194	1,157,194	1	C -> T	69	SNP (transition)	97.1%		1.065	GCC -> GCT	C770_RS47200	outer membrane bioger	None	1.5E-238	355	3	WP_015243258.1
1,157,216	1,157,216	1	A -> G	71	SNP (transition)	100.0%	N -> D	1.087	AAT -> GAT	C770_RS47200	outer membrane bioger	Substitution	5.0E-235	363	1	WP_015243258.1
1,157,221	1,157,221	1	C -> G	73	SNP (transversion)	100.0%		1.092	GCC -> GCG	C770_RS47200	outer membrane bioger	None	6.3E-249	364	3	WP_015243258.1
1,157,224	1,157,224	1	C -> A	73	SNP (transversion)	100.0%		1.095	GGC -> GGA	C770_RS47200	outer membrane bioger	None	1.3E-241	365	3	WP_015243258.1
1,157,228	1,157,228	1	C -> G	71	SNP (transversion)	98.6%	L -> V	1.099	CTT -> GTT	C770_RS47200	outer membrane bioger	Substitution	7.1E-237	367	1	WP_015243258.1
1,157,266	1,157,266	1	C -> T	73	SNP (transition)	97.3%		1.137	GAC -> GAT	C770_RS47200	outer membrane bioger	None	2.1E-217	379	3	WP_015243258.1
1,157,271	1,157,271	1	T -> C	73	SNP (transition)	98.6%	V -> A	1.142	GTC -> GCC	C770_RS47200	outer membrane bioger	Substitution	4.6E-222	381	2	WP_015243258.1
1,157,308	1,157,308	1	C -> A	73	SNP (transversion)	100.0%		1.179	ATC -> ATA	C770_RS47200	outer membrane bioger	None	6.3E-249	393	3	WP_015243258.1
1,157,311	1,157,311	1	G -> C	74	SNP (transversion)	100.0%		1.182	GCG -> GCC	C770_RS47200	outer membrane bioger	None	4.0E-230	394	3	WP_015243258.1
1,157,318	1,157,318	1	G -> T	75	SNP (transversion)	100.0%	A -> S	1.189	GCA -> TCA	C770_RS47200	outer membrane bioger	Substitution	1.0E-225	397	1	WP_015243258.1
1,157,336	1,157,336	1	A -> T	77	SNP (transversion)	100.0%	T -> S	1.207	ACC -> TCC	C770_RS47200	outer membrane bioger	Substitution	1.0E-231	403	1	WP_015243258.1
1,157,347	1,157,347	1	T -> G	77	SNP (transversion)	100.0%		1.218	CTT -> CTG	C770_RS47200	outer membrane bioger	None	4.0E-247	406	3	WP_015243258.1
1,157,350	1,157,350	1	G -> C	77	SNP (transversion)	100.0%		1.221	TCG -> TCC	C770_RS47200	outer membrane bioger	None	2.0E-239	407	3	WP_015243258.1
1,157,356	1,157,357	2	AT -> CC	79	Substitution	100.0%		1.227	GCA,TTG -> GCC,	C770_RS47200	outer membrane bioger	None	1.3E-245	409	3	WP_015243258.1
1,157,362	1,157,362	1	A -> G	78	SNP (transition)	98.7%		1.233	ACA -> ACG	C770_RS47200	outer membrane bioger	None	3.1E-245	411	3	WP_015243258.1
1,157,377	1,157,377	1	C -> T	76	SNP (transition)	97.4%		1.248	TTC -> TTT	C770_RS47200	outer membrane bioger	None	1.1E-226	416	3	WP_015243258.1
1,157,384	1,157,384	1	G -> C	77	SNP (transversion)	97.4%	G -> R	1.255	GGC -> CGC	C770_RS47200	outer membrane bioger	Substitution	9.2E-245	419	1	WP_015243258.1
1,157,393	1,157,393	1	G -> A	76	SNP (transition)	97.4%	D -> N	1.264	GAC -> AAC	C770_RS47200	outer membrane bioger	Substitution	7.1E-212	422	1	WP_015243258.1
1,157,400	1,157,400	1	T -> A	76	SNP (transversion)	100.0%	V -> E	1.271	GTA -> GAA	C770_RS47200	outer membrane bioger	Substitution	1.0E-228	424	2	WP_015243258.1
1,157,447	1,157,447	1	G -> A	83	SNP (transition)	97.6%	D -> N	1.318	GAT -> AAT	C770_RS47200	outer membrane bioger	Substitution	3.4E-240	440	1	WP_015243258.1
1,157,453	1,157,453	1	A -> G	83	SNP (transition)	100.0%	T -> A	1.324	ACC -> GCC	C770_RS47200	outer membrane bioger	Substitution	2.0E-241	442	1	WP_015243258.1
1,157,494	1,157,494	1	A -> G	80	SNP (transition)	100.0%		1.365	GAA -> GAG	C770_RS47200	outer membrane bioger	None	1.0E-272	455	3	WP_015243258.1
1,157,515	1,157,515	1	G -> C	81	SNP (transversion)	100.0%		1.386	ACG -> ACC	C770_RS47200	outer membrane bioger	None	5.0E-268	462	3	WP_015243258.1
1,157,600	1,157,600	1	A -> C	77	SNP (transversion)	100.0%	I -> L	1.471	ATC -> CTC	C770_RS47200	outer membrane bioger	Substitution	3.2E-270	491	1	WP_015243258.1
1,157,633	1,157,633	1	C -> T	78	SNP (transition)	98.7%	L -> F	1.504	CTC -> TTC	C770_RS47200	outer membrane bioger	Substitution	1.2E-260	502	1	WP_015243258.1
1,157,714	1,157,714	1	G -> T	91	SNP (transversion)	98.9%	A -> S	1.585	GCG -> TCG	C770_RS47200	outer membrane bioger	Substitution	9.1E-305	529	1	WP_015243258.1
1,157,824	1,157,824	1	G -> T	95	SNP (transversion)	96.8%		1.695	GGG -> GGT	C770_RS47200	outer membrane bioger	None	2.2E-308	565	3	WP_015243258.1
1,157,842	1,157,842	1	G -> C	98	SNP (transversion)	98.0%		1.713	GCG -> GCC	C770_RS47200	outer membrane bioger	None	1.2E-294	571	3	WP_015243258.1
1,157,861	1,157,863	2	AAT -> GAG	98	Substitution	99.0%	N -> E	1.732	AAT -> GAG	C770_RS47200	outer membrane bioger	Substitution	2.0E-299	578	1	WP_015243258.1
1,157,882	1,157,882	1	G -> A	104	SNP (transition)	98.1%	G -> S	1.753	GGC -> AGC	C770_RS47200	outer membrane bioger	Substitution	0.0	585	1	WP_015243258.1
1,157,896	1,157,896	1	A -> G	107	SNP (transition)	99.1%		1.767	GCA -> GCG	C770_RS47200	outer membrane bioger	None	0.0	589	3	WP_015243258.1
1,157,915	1,157,915	1	C -> T	105	SNP (transition)	98.1%	L -> F	1.786	CTC -> TTC	C770_RS47200	outer membrane bioger	Substitution	5.4E-306	596	1	WP_015243258.1
1,157,918	1,157,918	1	G -> A	105	SNP (transition)	98.1%	A -> T	1.789	GCG -> ACG	C770_RS47200	outer membrane bioger	Substitution	0.0	597	1	WP_015243258.1
1,157,923	1,157,923	1	G -> A	104	SNP (transition)	97.1%		1.794	ACG -> ACA	C770_RS47200	outer membrane bioger	None	0.0	598	3	WP_015243258.1
1,157,939	1,157,939	1	G -> A	111	SNP (transition)	97.3%	A -> T	1.81	GCC -> ACC	C770_RS47200	outer membrane bioger	Substitution	0.0	604	1	WP_015243258.1
1,157,973	1,157,973	1	G -> A	110	SNP (transition)	95.5%	R -> Q	1.844	CGG -> CAG	C770_RS47200	outer membrane bioger	Substitution	0.0	615	2	WP_015243258.1
1,157,975	1,157,975	1	C -> T	109	SNP (transition)	98.2%	L -> F	1.846	CTT -> TTT	C770_RS47200	outer membrane bioger	Substitution	0.0	616	1	WP_015243258.1
1,157,980	1,157,980	1	G -> C	108	SNP (transversion)	98.1%		1.851	GCG -> GCC	C770_RS47200	outer membrane bioger	None	0.0	617	3	WP_015243258.1
1,157,988	1,157,988	1	G -> A	109	SNP (transition)	97.2%	R -> Q	1.859	CGA -> CAA	C770_RS47200	outer membrane bioger	Substitution	0.0	620	2	WP_015243258.1
1,157,994	1,157,995	2	GC -> CG	107	Substitution	98.1%	R -> P	1.865	CGC -> CCG	C770_RS47200	outer membrane bioger	Substitution	0.0	622	2	WP_015243258.1
1,158,259	1,158,259	1	G -> A	83	SNP (transition)	98.8%		2.13	CTG -> CTA	C770_RS47200	outer membrane bioger	None	3.3E-261	710	3	WP_015243258.1
1,158,310	1,158,310	1	G -> C	85	SNP (transversion)	100.0%		2.181	GCG -> GCC	C770_RS47200	outer membrane bioger	None	3.2E-281	727	3	WP_015243258.1
1,158,373	1,158,373	1	A -> G	81	SNP (transition)	97.5%		2.244	ACA -> ACG	C770_RS47200	outer membrane bioger	None	8.1E-266	748	3	WP_015243258.1
1,158,478	1,158,479	2	TT -> CC	83	Substitution	100.0%		2.349	AGT,TTG -> AGC,	C770_RS47200	outer membrane bioger	None	1.3E-274	783	3	WP_015243258.1
1,158,661	1,158,661	1	T -> C	88	SNP (transition)	100.0%		2.532	CTT -> CTC	C770_RS47200	outer membrane bioger	None	2.5E-282	844	3	WP_015243258.1
1,158,790	1,158,790	1	G -> A	89	SNP (transition)	100.0%		2.661	CTG -> CTA	C770_RS47200	outer membrane bioger	None	2.0E-294	887	3	WP_015243258.1
1,158,808	1,158,809	2	AT -> GC	92	Substitution	95.7%		2.679	GCA,TTA -> GCG,	C770_RS47200	outer membrane bioger	None	2.5E-289	893	3	WP_015243258.1
1,158,861	1,158,861	1	A -> G	97	SNP (transition)	100.0%	D -> G	2.732	GAC -> GGC	C770_RS47200	outer membrane bioger	Substitution	2.0E-301	911	2	WP_015243258.1
1,158,873	1,158,873	1	A -> G	93	SNP (transition)	97.8%	E -> G	2.744	GAA -> GGA	C770_RS47200	outer membrane bioger	Substitution	2.7E-288	915	2	WP_015243258.1
1,159,002	1,159,002	1	C -> T	84	SNP (transition)	98.8%	P -> L	2.873	CCG -> CTG	C770_RS47200	outer membrane bioger	Substitution	5.3E-281	958	2	WP_015243258.1
1,159,066	1,159,066	1	A -> T	79	SNP (transversion)	98.7%		2.937	CCA -> CCT	C770_RS47200	outer membrane bioger	None	3.1E-256	979	3	WP_015243258.1
1,159,153	1,159,153	1	A -> G	69	SNP (transition)	98.6%		3.024	TAA -> TAG	C770_RS47200	outer membrane bioger	None	4.4E-230	1.008	3	WP_015243258.1
1,159,154	1,159,154	1	T -> C	69	SNP (transition)	100.0%							2.5E-235			
1,159,165	1,159,165	1	(C)3 -> (C)2	73	Deletion (tandem repeat)	100.0%							3.2E-110			
1,159,177	1,159,177	1	G	73	Deletion	100.0%							6.3E-103			
1,159,184	1,159,184	1	G -> T	73	SNP (transversion)	98.6%							2.9E-229			
1,159,187	1,159,187	1	C -> A	72	SNP (transversion)	100.0%							1.6E-245			
1,159,195	1,159,195	1	G -> A	75	SNP (transition)	100.0%							3.2E-248			
1,159,229	1,159,229	1	G -> A	72	SNP (transition)	95.8%		717	CAC -> CAT	C770_RS47205	hypothetical protein	None	1.5E-230	239	3	WP_010975434.1
1,159,252	1,159,252	1	G -> A	74	SNP (transition)	100.0%		694	CTG -> TTG	C770_RS47205	hypothetical protein	None	2.5E-252	232	1	WP_010975434.1
1,159,268	1,159,268	1	C -> G	74	SNP (transversion)	100.0%		678	CGG -> CGC	C770_RS47205	hypothetical protein	None	6.3E-245	226	3	WP_010975434.1
1,159,271	1,159,271	1	G -> A	73	SNP (transition)	98.6%		675	GAC -> GAT	C770_RS47205	hypothetical protein	None	1.8E-236	225	3	WP_010975434.1
1,159,277	1,159,277	1	G -> T	77	SNP (transversion)	98.7%		669	CCC -> CCA	C770_RS47205	hypothetical protein	None	3.1E-257	223	3	WP_010975434.1
1,159,301	1,159,301	1	C -> T	84	SNP (transition)	100.0%		645	CCG -> CCA	C770_RS47205	hypothetical protein	None	1.0E-294	215	3	WP_010975434.1
1,159,307	1,159,308	2	CC -> TG	85	Substitution	98.8%	R -> T	638	AGG -> ACA	C770_RS47205	hypothetical protein	Substitution	1.3E-267	213	2	WP_010975434.1
1,159,319	1,159,319	1	C -> T	87	SNP (transition)	100.0%		627	AAG -> AAA	C770_RS47205	hypothetical protein	None	7.9E-288	209	3	WP_010975434.1
1,159,329	1,159,329	1	G -> A	88	SNP (transition)	98.9%	T -> I	617	ACC -> ATC	C770_RS47205	hypothetical protein	Substitution	1.4E-294	206	2	WP_010975434.1
1,159,334	1,159,334	1	G -> A	89	SNP (transition)	100.0%		612	TTC -> TTT	C770_RS47205	hypothetical protein	None	2.0E-294	204	3	WP_010975434.1
1,159,340	1,159,340	1	T -> C	93	SNP (transition)	98.9%		606	GAA -> GAG	C770_RS47205	hypothetical protein	None	1.5E-311	202	3	WP_010975434.1
1,159,349	1,159,349	1	A -> G	91	SNP (transition)	100.0%		597	CCT -> CCC	C770_RS47205	hypothetical protein	None	5.0E-301	199	3	WP_010975434.1
1,159,355	1,159,355	1	C -> T	92	SNP (transition)	98.9%		591	GGG -> GGA	C770_RS47205	hypothetical protein	None	3.7E-308	197	3	WP_010975434.1
1,159,361	1,159,361	1	C -> T	93	SNP (transition)	100.0%		585	AGG -> AGA	C770_RS47205	hypothetical protein	None	6.3E-317	195	3	WP_010975434.1
1,159,364	1,159,364	1	G -> A	92	SNP (transition)	100.0%		582	GAC -> GAT	C770_RS47205	hypothetical protein	None	1.0E-322	194	3	WP_010975434.1
1,159,374	1,159,374	1	T -> C	93	SNP (transition)	100.0%	N -> S	572	AAC -> AGC	C770_RS47205	hypothetical protein	Substitution	6.3E-317	191	2	WP_010975434.1
1,159,376	1,159,376	1	G -> T	93	SNP (transversion)	100.0%		570	CGC -> CGA	C770_RS47205	hypothetical protein	None	1.3E-307	190	3	WP_010975434.1
1,159,385	1,159,385	1	T -> C	92	SNP (transition)	100.0%		561	GAA -> GAG	C770_RS47205	hypothetical protein	None	2.5E-304	187	3	WP_010975434.1
1,159,409	1,159,411	2	TAC -> GAT	93 -> 94	Substitution	100.0%	V -> I	535	GTA -> ATC	C770_RS47205	hypothetical protein	Substitution	2.5E-298	179	1	WP_010975434.1
1,159,415	1,159,415	1	A -> G	96	SNP (transition)	100.0%		531	GAT -> GAC	C770_RS47205	hypothetical protein	None	0.0	177	3	WP_010975434.1
1,159,426	1,159,426	1	C -> T	100	SNP (transition)	100.0%	V -> I	520	GTT -> ATT	C770_RS47205	hypothetical protein	Substitution	1.0E-300	174	1	WP_010975434.1
1,159,433	1,159,433	1	G -> A	101	SNP (transition)	100.0%		513	AAC -> AAT	C770_RS47205	hypothetical protein	None	0.0	171	3	WP_010975434.1
1,159,454	1,159,454	1	C -> G	103	SNP (transversion)	100.0%		492	TCG -> TCC	C770_RS47205	hypothetical protein	None	5.0E-320	164	3	WP_010975434.1
1,159,459	1,159,459	1	T -> C	104	SNP (transition)	100.0%	K -> E	487	AAG -> GAG	C770_RS47205	hypothetical protein	Substitution	4.0E-323	163	1	WP_010975434.1
1,159,463	1,159,463	1	G -> A	101	SNP (transition)	99.0%		483	AAC -> AAT	C770_RS47205	hypothetical protein	None	1.0E-298	161	3	WP_010975434.1
1,159,480	1,159,480	1	C -> T	108	SNP (transition)	100.0%	E -> K	466	GAG -> AAG	C770_RS47205	hypothetical protein	Substitution	0.0	156	1	WP_010975434.1
1,159,502	1,159,502	1	A -> G	106	SNP (transition)	100.0%		444	TGT -> TGC	C770_RS47205	hypothetical protein	None	0.0	148	3	WP_010975434.1
1,159,520	1,159,521	2	GC -> AG	111 -> 112	Substitution	98.2%	S -> T	425	AGC -> ACT	C770_RS47205	hypothetical protein	Substitution	1.4E-320	142	2	WP_010975434.1
1,159,532	1,159,532	1	A -> G	110	SNP (transition)	100.0%		414	GAT -> GAC	C770_RS47205	hypothetical protein	None	0.0	138	3	WP_010975434.1
1,159,535	1,159,535	1	G -> C	110	SNP (transversion)	99.1%		411	GCC -> GCG	C770_RS47205	hypothetical protein	None	0.0	137	3	WP_010975434.1
1,159,553	1,159,553	1	A -> G	107	SNP (transition)	100.0%		393	AAT -> AAC	C770_RS47205	hypothetical protein	None	0.0	131	3	WP_010975434.1
1,159,565	1,159,565	1	A -> C	106	SNP (transversion)	99.1%		381	GTT -> GTG	C770_RS47205	hypothetical protein	None	0.0	127	3	WP_010975434.1
1,159,568	1,159,568	1	A -> C	103	SNP (transversion)	96.1%		378	GCT -> GCG	C770_RS47205	hypothetical protein	None	5.5E-301	126	3	WP_010975434.1
1,159,611	1,159,611	1	T	90	Deletion	100.0%		335		C770_RS47205	hypothetical protein	Frame Shift	1.0E-144	112	2	WP_010975434.1
1,159,616	1,159,615	0	(T)3 -> (T)4	87	Insertion (tandem repeat)	96.6%		331		C770_RS47205	hypothetical protein	Frame Shift	6.7E-273	111	1	WP_010975434.1
1,159,631	1,159,631	1	G -> A	84	SNP (transition)	100.0%		315	TTC -> TTT	C770_RS47205	hypothetical protein	None	2.5E-286	105	3	WP_010975434.1
1,159,636	1,159,636	1	A -> C	83	SNP (transversion)	100.0%	S -> A	310	TCC -> GCC	C770_RS47205	hypothetical protein	Substitution	1.3E-274	104	1	WP_010975434.1
1,159,640	1,159,640	1	G -> A	78	SNP (transition)	100.0%		306	ATC -> ATT	C770_RS47205	hypothetical protein	None	4.0E-258	102	3	WP_010975434.1
1,159,655	1,159,655	1	C -> T	74	SNP (transition)	100.0%		291	TCG -> TCA	C770_RS47205	hypothetical protein	None	2.5E-252	97	3	WP_010975434.1
1,159,661	1,159,661	1	A -> G	72	SNP (transition)	100.0%		285	GAT -> GAC	C770_RS47205	hypothetical protein	None	1.6E-245	95	3	WP_010975434.1
1,159,666	1,159,666	1	C -> T	72	SNP (transition)	100.0%	A -> T	280	GCG -> ACG	C770_RS47205	hypothetical protein	Substitution	1.0E-252	94	1	WP_010975434.1
1,159,671	1,159,671	1	C -> T	70	SNP (transition)	100.0%	R -> K	275	AGG -> AAG	C770_RS47205	hypothetical protein	Substitution	1.0E-252	92	2	WP_010975434.1
1,159,682	1,159,682	1	A -> G	70	SNP (transition)	100.0%		264	CGT -> CGC	C770_RS47205	hypothetical protein	None	1.0E-252	88	3	WP_010975434.1
1,159,698	1,159,698	1	A -> G	67	SNP (transition)	98.5%	V -> A	248	GTC -> GCC	C770_RS47205	hypothetical protein	Substitution	1.1E-216	83	2	WP_010975434.1
1,159,700	1,159,701	2	AC -> CA	66 -> 67	Substitution	100.0%	G -> V	245	GGT -> GTG	C770_RS47205	hypothetical protein	Substitution	7.9E-222	82	2	WP_010975434.1
1,159,715	1,159,715	1	G -> A	60	SNP (transition)	100.0%		231	GTC -> GTT	C770_RS47205	hypothetical protein	None	1.0E-210	77	3	WP_010975434.1
1,159,730	1,159,730	1	A -> G	54	SNP (transition)	100.0%		216	ATT -> ATC	C770_RS47205	hypothetical protein	None	2.5E-184	72	3	WP_010975434.1
1,159,763	1,159,763	1	G -> A	40	SNP (transition)	100.0%		183	CAC -> CAT	C770_RS47205	hypothetical protein	None	1.0E-140	61	3	WP_010975434.1
1,159,766	1,159,766	1	G -> A	40	SNP (transition)	97.5%		180	TGC -> TGT	C770_RS47205	hypothetical protein	None	1.6E-139	60	3	WP_010975434.1
1,159,775	1,159,775	1	A -> G	34	SNP (transition)	100.0%		171	GCT -> GCC	C770_RS47205	hypothetical protein	None	6.3E-113	57	3	WP_010975434.1
1,159,784	1,159,784	1	C -> T	30	SNP (transition)	100.0%		162	GCG -> GCA	C770_RS47205	hypothetical protein	None	1.0E-102	54	3	WP_010975434.1
1,159,787	1,159,787	1	C -> T	28	SNP (transition)	100.0%		159	CTG -> CTA	C770_RS47205	hypothetical protein	None	4.0E-93	53	3	WP_010975434.1
1,159,832	1,159,832	1	T -> C	12	SNP (transition)	100.0%		114	GGA -> GGG	C770_RS47205	hypothetical protein	None	1.6E-35	38	3	WP_010975434.1
1,159,838	1,159,838	1	C -> T	11	SNP (transition)	100.0%		108	CAG -> CAA	C770_RS47205	hypothetical protein	None	4.0E-38	36	3	WP_010975434.1
1,159,841	1,159,843	2	TAC -> CAG	9	Substitution	100.0%	V -> L	103	GTA -> CTG	C770_RS47205	hypothetical protein	Substitution	1.0E-27	35	1	WP_010975434.1
1,159,850	1,159,850	1	A -> T	9	SNP (transversion)	100.0%		96	CCT -> CCA	C770_RS47205	hypothetical protein	None	1.6E-29	32	3	WP_010975434.1
1,159,857	1,159,857	1	A -> G	8	SNP (transition)	100.0%	F -> S	89	TTC -> TCC	C770_RS47205	hypothetical protein	Substitution	4.0E-23	30	2	WP_010975434.1
1,159,868	1,159,868	1	G -> A	6	SNP (transition)	100.0%		78	CTC -> CTT	C770_RS47205	hypothetical protein	None	6.3E-17	26	3	WP_010975434.1
1,160,134	1,160,135	2	GT -> CC	8	Substitution	100.0%							2.5E-22			
1,160,142	1,160,142	1	C -> T	13	SNP (transition)	100.0%							5.0E-41			
1,160,148	1,160,148	1	G -> A	14	SNP (transition)	100.0%							1.0E-35			
1,160,155	1,160,154	0	G	16	Insertion	100.0%							1.0E-48			
1,160,166	1,160,166	1	T -> A	17	SNP (transversion)	100.0%							5.0E-50			
1,160,169	1,160,174	6	ATCAAG	18	Deletion	100.0%							4.0E-24			
1,160,184	1,160,184	1	A -> C	21	SNP (transversion)	100.0%							5.0E-70			
1,160,189	1,160,190	2	CG -> TC	23	Substitution	100.0%							1.0E-69			
1,160,195	1,160,195	1	G -> A	24	SNP (transition)	100.0%							6.3E-80			
1,160,208	1,160,208	1	A -> G	27	SNP (transition)	100.0%							4.0E-87			
1,160,219	1,160,219	1	A -> G	33	SNP (transition)	100.0%							1.3E-109			
1,160,221	1,160,221	1	C -> G	33	SNP (transversion)	97.0%							8.3E-105			
1,160,233	1,160,233	1	T -> C	37	SNP (transition)	100.0%							2.0E-115			
1,160,235	1,160,234	0	AA	37	Insertion	100.0%							1.6E-126			
1,160,241	1,160,241	1	T -> C	39	SNP (transition)	100.0%							1.6E-125			
1,160,246	1,160,247	2	CA -> AT	40	Substitution	100.0%							1.0E-132			
1,160,252	1,160,258	7	CCTTGCT -> AAC/	42 -> 43	Substitution	97.6% -> 97.7%							2.5E-139			
1,160,264	1,160,264	1	T -> G	43	SNP (transversion)	100.0%							3.2E-151			
1,160,266	1,160,266	1	C -> A	46	SNP (transversion)	100.0%							6.3E-148			
1,160,277	1,160,277	1	C -> A	50	SNP (transversion)	98.0%							1.3E-165			
1,160,292	1,160,293	2	CG -> AT	53	Substitution	100.0%	A -> D	1.796	GCG -> GAT	C770_RS47210	multidrug ABC transpor	Substitution	3.2E-186	599	2	WP_015243259.1
1,160,295	1,160,295	1	C -> G	53	SNP (transversion)	100.0%		1.794	GCG -> GCC	C770_RS47210	multidrug ABC transpor	None	1.3E-175	598	3	WP_015243259.1
1,160,301	1,160,301	1	T -> G	56	SNP (transversion)	100.0%		1.788	GTA -> GTC	C770_RS47210	multidrug ABC transpor	None	1.0E-196	596	3	WP_015243259.1
1,160,315	1,160,315	1	G -> A	62	SNP (transition)	98.4%		1.774	CTG -> TTG	C770_RS47210	multidrug ABC transpor	None	3.1E-200	592	1	WP_015243259.1
1,160,321	1,160,321	1	C -> T	63	SNP (transition)	100.0%	G -> S	1.768	GGC -> AGC	C770_RS47210	multidrug ABC transpor	Substitution	3.2E-221	590	1	WP_015243259.1
1,160,349	1,160,349	1	G -> A	67	SNP (transition)	100.0%		1.74	GTC -> GTT	C770_RS47210	multidrug ABC transpor	None	1.6E-228	580	3	WP_015243259.1
1,160,378	1,160,378	1	C -> G	86	SNP (transversion)	97.7%	E -> Q	1.711	GAG -> CAG	C770_RS47210	multidrug ABC transpor	Substitution	5.8E-266	571	1	WP_015243259.1
1,160,394	1,160,394	1	C -> T	88	SNP (transition)	98.9%		1.695	GTG -> GTA	C770_RS47210	multidrug ABC transpor	None	7.0E-286	565	3	WP_015243259.1
1,160,409	1,160,409	1	G -> C	88	SNP (transversion)	100.0%	N -> K	1.68	AAC -> AAG	C770_RS47210	multidrug ABC transpor	Substitution	4.0E-291	560	3	WP_015243259.1
1,160,430	1,160,430	1	G -> A	96	SNP (transition)	97.9%		1.659	GCC -> GCT	C770_RS47210	multidrug ABC transpor	None	1.1E-316	553	3	WP_015243259.1

1,160,436	1,160,436	1	A -> G		94	SNP (transition)	95.7%		1.653	GTT -> GTC	C770_RS47210	multidrug ABC transpor	None	3.0E-300	551	3	WP_015243259.1
1,160,514	1,160,514	1	T -> C		102	SNP (transition)	99.0%		1.575	GCA -> GCG	C770_RS47210	multidrug ABC transpor	None	0.0	525	3	WP_015243259.1
1,160,523	1,160,523	1	C -> T		104	SNP (transition)	99.0%		1.566	GAG -> GAA	C770_RS47210	multidrug ABC transpor	None	0.0	522	3	WP_015243259.1
1,160,532	1,160,532	1	A -> G		106	SNP (transition)	97.2%		1.557	CTT -> CTC	C770_RS47210	multidrug ABC transpor	None	0.0	519	3	WP_015243259.1
1,160,535	1,160,535	1	A -> C		106	SNP (transversion)	99.1%		1.554	GCT -> GCG	C770_RS47210	multidrug ABC transpor	None	0.0	518	3	WP_015243259.1
1,160,565	1,160,565	1	A -> G		109	SNP (transition)	100.0%		1.524	CCT -> CCC	C770_RS47210	multidrug ABC transpor	None	0.0	508	3	WP_015243259.1
1,160,592	1,160,592	1	G -> A		104	SNP (transition)	99.0%		1.497	ATC -> ATT	C770_RS47210	multidrug ABC transpor	None	0.0	499	3	WP_015243259.1
1,160,598	1,160,598	1	G -> A		104	SNP (transition)	98.1%		1.491	CTC -> CTT	C770_RS47210	multidrug ABC transpor	None	0.0	497	3	WP_015243259.1
1,160,601	1,160,601	1	G -> A		104	SNP (transition)	96.2%		1.488	CGC -> CGT	C770_RS47210	multidrug ABC transpor	None	4.9E-324	496	3	WP_015243259.1
1,160,624	1,160,624	1	A -> G		102	SNP (transition)	99.0%		1.465	TTA -> CTA	C770_RS47210	multidrug ABC transpor	None	0.0	489	1	WP_015243259.1
1,160,631	1,160,631	1	G -> A		102	SNP (transition)	97.1%		1.458	GGC -> GGT	C770_RS47210	multidrug ABC transpor	None	0.0	486	3	WP_015243259.1
1,160,634	1,160,634	1	T -> C		102	SNP (transition)	98.0%		1.455	CAA -> CAG	C770_RS47210	multidrug ABC transpor	None	0.0	485	3	WP_015243259.1
1,160,640	1,160,640	1	A -> G		108	SNP (transition)	100.0%		1.449	GGT -> GGC	C770_RS47210	multidrug ABC transpor	None	0.0	483	3	WP_015243259.1
1,160,655	1,160,655	1	G -> A		107	SNP (transition)	99.1%		1.434	TAC -> TAT	C770_RS47210	multidrug ABC transpor	None	0.0	478	3	WP_015243259.1
1,160,658	1,160,658	1	C -> G		106	SNP (transversion)	98.1%		1.431	GGG -> GGC	C770_RS47210	multidrug ABC transpor	None	0.0	477	3	WP_015243259.1
1,160,664	1,160,664	1	T -> C		104	SNP (transition)	99.0%		1.425	CCA -> CCG	C770_RS47210	multidrug ABC transpor	None	0.0	475	3	WP_015243259.1
1,160,691	1,160,691	1	A -> G		100	SNP (transition)	98.0%		1.398	TAT -> TAC	C770_RS47210	multidrug ABC transpor	None	0.0	466	3	WP_015243259.1
1,160,715	1,160,715	1	T -> G		103	SNP (transversion)	98.1%		1.374	GTA -> GTC	C770_RS47210	multidrug ABC transpor	None	0.0	458	3	WP_015243259.1
1,160,739	1,160,739	1	C -> T		107	SNP (transition)	100.0%		1.35	CGG -> CGA	C770_RS47210	multidrug ABC transpor	None	0.0	450	3	WP_015243259.1
1,160,766	1,160,766	1	G -> A		96	SNP (transition)	97.9%		1.323	TCC -> TCT	C770_RS47210	multidrug ABC transpor	None	1.1E-316	441	3	WP_015243259.1
1,160,769	1,160,769	1	A -> G		95	SNP (transition)	100.0%		1.32	GGT -> GGC	C770_RS47210	multidrug ABC transpor	None	3.2E-314	440	3	WP_015243259.1
1,160,775	1,160,775	1	A -> G		92	SNP (transition)	100.0%		1.314	TTT -> TTC	C770_RS47210	multidrug ABC transpor	None	1.0E-322	438	3	WP_015243259.1
1,160,781	1,160,781	1	G -> A		91	SNP (transition)	98.9%		1.308	TAC -> TAT	C770_RS47210	multidrug ABC transpor	None	9.1E-305	436	3	WP_015243259.1
1,160,784	1,160,786	2	AGC -> GGG		89	Substitution	100.0%	A -> P	1.303	GCT -> CCC	C770_RS47210	multidrug ABC transpor	Substitution	2.0E-294	435	1	WP_015243259.1
1,160,790	1,160,790	1	T -> C		85	SNP (transition)	98.8%		1.299	CAA -> CAG	C770_RS47210	multidrug ABC transpor	None	2.1E-284	433	3	WP_015243259.1
1,160,793	1,160,793	1	T -> C		84	SNP (transition)	97.6%		1.296	TCA -> TCG	C770_RS47210	multidrug ABC transpor	None	5.5E-276	432	3	WP_015243259.1
1,160,808	1,160,808	1	C -> G		78	SNP (transversion)	100.0%		1.281	GGG -> GGC	C770_RS47210	multidrug ABC transpor	None	6.3E-266	427	3	WP_015243259.1
1,160,811	1,160,814	4	GTTG -> AGCA		78	Substitution	96.2%	RN -> RA	1.275	CGC,AAC -> CGT,	C770_RS47210	multidrug ABC transpor	Substitution	1.2E-260	425	3	WP_015243259.1
1,160,817	1,160,817	1	C -> A		76	SNP (transversion)	100.0%		1.272	CTG -> CTT	C770_RS47210	multidrug ABC transpor	None	6.3E-244	424	3	WP_015243259.1
1,160,820	1,160,820	1	A -> C		75	SNP (transversion)	98.7%		1.269	TCT -> TCG	C770_RS47210	multidrug ABC transpor	None	1.9E-250	423	3	WP_015243259.1
1,160,825	1,160,825	1	G -> T		75	SNP (transversion)	100.0%	Q -> K	1.264	CAG -> AAG	C770_RS47210	multidrug ABC transpor	Substitution	1.0E-240	422	1	WP_015243259.1
1,160,832	1,160,832	1	A -> G		71	SNP (transition)	100.0%		1.257	GTT -> GTC	C770_RS47210	multidrug ABC transpor	None	5.0E-235	419	3	WP_015243259.1
1,160,835	1,160,836	2	AG -> GC		69	Substitution	98.6%	A -> G	1.253	GCT -> GGC	C770_RS47210	multidrug ABC transpor	Substitution	2.7E-223	418	2	WP_015243259.1
1,160,841	1,160,841	1	T -> G		68	SNP (transversion)	100.0%		1.248	ATA -> ATC	C770_RS47210	multidrug ABC transpor	None	1.0E-238	416	3	WP_015243259.1
1,160,844	1,160,847	3	GTCT -> ATTC		68	Substitution	100.0%	QD -> QN	1.242	CAA,GAC -> CAG,	C770_RS47210	multidrug ABC transpor	Substitution	4.0E-225	414	3	WP_015243259.1
1,160,850	1,160,850	1	C -> G		64	SNP (transversion)	100.0%		1.239	GGG -> GGC	C770_RS47210	multidrug ABC transpor	None	6.3E-212	413	3	WP_015243259.1
1,160,866	1,160,866	1	A -> G		60	SNP (transition)	100.0%	V -> A	1.223	GTG -> GCG	C770_RS47210	multidrug ABC transpor	Substitution	1.0E-198	408	2	WP_015243259.1
1,160,871	1,160,871	1	C -> G		59	SNP (transversion)	100.0%		1.218	TCG -> TCC	C770_RS47210	multidrug ABC transpor	None	2.0E-195	406	3	WP_015243259.1
1,160,892	1,160,892	1	T -> C		55	SNP (transition)	100.0%		1.197	CCA -> CCG	C770_RS47210	multidrug ABC transpor	None	1.0E-176	399	3	WP_015243259.1
1,160,895	1,160,895	1	A -> G		55	SNP (transition)	100.0%		1.194	ATT -> ATC	C770_RS47210	multidrug ABC transpor	None	3.2E-182	398	3	WP_015243259.1
1,160,910	1,160,910	1	C -> G		51	SNP (transversion)	100.0%		1.179	ACG -> ACC	C770_RS47210	multidrug ABC transpor	None	5.0E-169	393	3	WP_015243259.1
1,160,913	1,160,913	1	C -> G		51	SNP (transversion)	100.0%		1.176	TCG -> TCC	C770_RS47210	multidrug ABC transpor	None	7.9E-159	392	3	WP_015243259.1
1,160,916	1,160,916	1	T -> C		51	SNP (transition)	100.0%		1.173	AAA -> AAG	C770_RS47210	multidrug ABC transpor	None	5.0E-169	391	3	WP_015243259.1
1,160,925	1,160,925	1	T -> G		47	SNP (transversion)	97.9%		1.164	GGA -> GGC	C770_RS47210	multidrug ABC transpor	None	1.9E-155	388	3	WP_015243259.1
1,160,934	1,160,934	1	G -> C		46	SNP (transversion)	97.8%		1.155	GGC -> GGG	C770_RS47210	multidrug ABC transpor	None	1.5E-138	385	3	WP_015243259.1
1,160,937	1,160,937	1	A -> G		43	SNP (transition)	97.7%		1.152	GTT -> GTC	C770_RS47210	multidrug ABC transpor	None	6.8E-142	384	3	WP_015243259.1
1,160,946	1,160,946	1	G -> C		39	SNP (transversion)	100.0%		1.143	ACC -> ACG	C770_RS47210	multidrug ABC transpor	None	1.6E-125	381	3	WP_015243259.1
1,160,949	1,160,949	1	A -> C		37	SNP (transversion)	100.0%		1.14	ACT -> ACG	C770_RS47210	multidrug ABC transpor	None	7.9E-123	380	3	WP_015243259.1
1,160,961	1,160,961	1	T -> G		35	SNP (transversion)	97.1%	E -> D	1.128	GAA -> GAC	C770_RS47210	multidrug ABC transpor	Substitution	8.8E-115	376	3	WP_015243259.1
1,160,964	1,160,964	1	T -> C		33	SNP (transition)	100.0%		1.125	GCA -> GCG	C770_RS47210	multidrug ABC transpor	None	1.3E-109	375	3	WP_015243259.1
1,160,974	1,160,974	1	C -> G		30	SNP (transversion)	100.0%	S -> T	1.115	AGT -> ACT	C770_RS47210	multidrug ABC transpor	Substitution	1.0E-96	372	2	WP_015243259.1
1,160,976	1,160,976	1	G -> A		30	SNP (transition)	100.0%		1.113	GTC -> GTT	C770_RS47210	multidrug ABC transpor	None	1.0E-99	371	3	WP_015243259.1
1,160,979	1,160,979	1	A -> G		28	SNP (transition)	100.0%		1.11	GGT -> GGC	C770_RS47210	multidrug ABC transpor	None	4.0E-93	370	3	WP_015243259.1
1,160,992	1,160,992	1	T -> G		23	SNP (transversion)	95.7%	E -> A	1.097	GAG -> GCG	C770_RS47210	multidrug ABC transpor	Substitution	3.6E-74	366	2	WP_015243259.1
1,160,997	1,160,997	1	G -> A		21	SNP (transition)	100.0%		1.092	GGC -> GGT	C770_RS47210	multidrug ABC transpor	None	4.0E-72	364	3	WP_015243259.1
1,163,802	1,163,803	2	AA -> GT		6	Substitution	100.0%							1.6E-14			
1,163,805	1,163,805	1	T -> A		7	SNP (transversion)	100.0%							1.6E-17			
1,163,807	1,163,807	1	C -> G		8	SNP (transversion)	100.0%							6.3E-24			
1,163,811	1,163,811	1	G -> A		9	SNP (transition)	100.0%							2.0E-21			
1,163,815	1,163,816	2	CG -> GA		11	Substitution	100.0%							5.0E-26			
1,163,820	1,163,821	2	TC -> CG	11 -> 12		Substitution	100.0%							3.2E-28			
1,163,823	1,163,823	1	G -> T		12	SNP (transversion)	100.0%							1.6E-29			
1,163,825	1,163,825	1	A -> G		12	SNP (transition)	100.0%							6.3E-32			
1,163,829	1,163,829	1	T -> G		14	SNP (transversion)	100.0%		294	CGA -> CGC	C770_RS47220	hypothetical protein	None	2.5E-41	98	3	WP_013850942.1
1,163,874	1,163,874	1	T -> C		28	SNP (transition)	100.0%		249	CCA -> CCG	C770_RS47220	hypothetical protein	None	1.0E-84	83	3	WP_013850942.1
1,163,898	1,163,898	1	C -> A		34	SNP (transversion)	100.0%	E -> D	225	GAG -> GAT	C770_RS47220	hypothetical protein	Substitution	6.3E-113	75	3	WP_013850942.1
1,163,916	1,163,916	1	G -> A		40	SNP (transition)	100.0%		207	ATC -> ATT	C770_RS47220	hypothetical protein	None	1.0E-120	69	3	WP_013850942.1
1,163,926	1,163,926	1	G -> A		44	SNP (transition)	97.7%	A -> V	197	GCA -> GTA	C770_RS47220	hypothetical protein	Substitution	2.2E-132	66	2	WP_013850942.1
1,164,009	1,164,009	1	G -> A		62	SNP (transition)	100.0%		114	CTC -> CTT	C770_RS47220	hypothetical protein	None	1.0E-217	38	3	WP_013850942.1
1,164,147	1,164,147	1	G -> C		78	SNP (transversion)	100.0%							4.0E-258			
1,164,225	1,164,225	1	A -> G		72	SNP (transition)	98.6%							2.9E-240			
1,164,230	1,164,230	1	A -> T		72	SNP (transversion)	98.6%							4.5E-226			
1,164,236	1,164,235	0	C		71	Insertion	100.0%							4.0E-242			
1,164,244	1,164,244	1	A -> G		70	SNP (transition)	100.0%							1.0E-224			
1,164,268	1,164,268	1	(G)3 -> (G)2		74	Deletion (tandem repeat)	100.0%							1.0E-111			
1,164,276	1,164,276	1	A -> C		79	SNP (transversion)	100.0%							2.0E-261			
1,164,295	1,164,295	1	C -> T		86	SNP (transition)	100.0%	R -> H	2.03	CGC -> CAC	C770_RS47225	acyltransferase	Substitution	4.0E-293	677	2	WP_015243261.1
1,164,303	1,164,303	1	A -> C		87	SNP (transversion)	100.0%		2.022	GCT -> GCG	C770_RS47225	acyltransferase	None	2.0E-270	674	3	WP_015243261.1
1,164,324	1,164,324	1	C -> T		87	SNP (transition)	100.0%	M -> I	2.001	ATG -> ATA	C770_RS47225	acyltransferase	Substitution	2.0E-270	667	3	WP_015243261.1
1,164,340	1,164,340	1	C -> T		89	SNP (transition)	100.0%	R -> H	1.985	CGT -> CAT	C770_RS47225	acyltransferase	Substitution	2.0E-294	662	2	WP_015243261.1
1,164,349	1,164,349	1	G -> A		93	SNP (transition)	100.0%	A -> V	1.976	GCA -> GTA	C770_RS47225	acyltransferase	Substitution	6.3E-317	659	2	WP_015243261.1
1,164,387	1,164,387	1	C -> T		89	SNP (transition)	98.9%		1.938	GGG -> GGA	C770_RS47225	acyltransferase	None	3.5E-289	646	3	WP_015243261.1
1,164,408	1,164,408	1	G -> A		87	SNP (transition)	100.0%		1.917	GGC -> GGT	C770_RS47225	acyltransferase	None	1.6E-296	639	3	WP_015243261.1
1,164,421	1,164,421	1	G -> A		82	SNP (transition)	100.0%	A -> V	1.904	GCT -> GTT	C770_RS47225	acyltransferase	Substitution	2.5E-271	635	2	WP_015243261.1
1,164,435	1,164,435	1	G -> A		85	SNP (transition)	100.0%		1.89	GCC -> GCT	C770_RS47225	acyltransferase	None	1.0E-272	630	3	WP_015243261.1
1,164,448	1,164,448	1	G -> T		87	SNP (transversion)	96.6%	A -> E	1.877	GCA -> GAA	C770_RS47225	acyltransferase	Substitution	1.7E-264	626	2	WP_015243261.1
1,164,531	1,164,531	1	G -> C		104	SNP (transversion)	99.0%		1.794	ACC -> ACG	C770_RS47225	acyltransferase	None	0.0	598	3	WP_015243261.1
1,164,621	1,164,621	1	C -> A		97	SNP (transversion)	99.0%		1.704	GTG -> GTT	C770_RS47225	acyltransferase	None	1.5E-315	568	3	WP_015243261.1
1,164,630	1,164,630	1	A -> C		95	SNP (transversion)	97.9%		1.695	GTT -> GTG	C770_RS47225	acyltransferase	None	2.8E-313	565	3	WP_015243261.1
1,164,660	1,164,660	1	T -> G		97	SNP (transversion)	97.9%		1.665	CTA -> CTC	C770_RS47225	acyltransferase	None	1.5E-310	555	3	WP_015243261.1
1,164,753	1,164,753	1	A -> C		99	SNP (transversion)	97.0%		1.572	GGT -> GGG	C770_RS47225	acyltransferase	None	2.5E-312	524	3	WP_015243261.1
1,164,765	1,164,765	1	A -> T		96	SNP (transversion)	97.9%	D -> E	1.56	GAT -> GAA	C770_RS47225	acyltransferase	Substitution	7.2E-298	520	3	WP_015243261.1
1,164,816	1,164,816	1	C -> G		100	SNP (transversion)	99.0%	K -> N	1.509	AAG -> AAC	C770_RS47225	acyltransferase	Substitution	0.0	503	3	WP_015243261.1
1,165,096	1,165,096	1	T -> A		80	SNP (transversion)	100.0%	N -> I	1.229	AAT -> ATT	C770_RS47225	acyltransferase	Substitution	1.0E-256	410	2	WP_015243261.1
1,165,125	1,165,125	1	G -> A		81	SNP (transition)	100.0%		1.2	TTC -> TTT	C770_RS47225	acyltransferase	None	6.3E-260	400	3	WP_015243261.1
1,165,149	1,165,149	1	T -> C		81	SNP (transition)	98.8%		1.176	CAA -> CAG	C770_RS47225	acyltransferase	None	8.1E-255	392	3	WP_015243261.1
1,165,179	1,165,179	1	C -> T		79	SNP (transition)	100.0%		1.146	AGG -> AGA	C770_RS47225	acyltransferase	None	2.5E-269	382	3	WP_015243261.1
1,165,191	1,165,191	1	A -> G		75	SNP (transition)	100.0%		1.134	GGT -> GGC	C770_RS47225	acyltransferase	None	1.0E-255	378	3	WP_015243261.1
1,165,224	1,165,224	1	G -> A		85	SNP (transition)	98.8%		1.101	GTC -> GTT	C770_RS47225	acyltransferase	None	2.1E-284	367	3	WP_015243261.1
1,165,259	1,165,259	1	T -> C		88	SNP (transition)	100.0%	I -> V	1.066	ATC -> GTC	C770_RS47225	acyltransferase	Substitution	4.0E-291	356	1	WP_015243261.1
1,165,386	1,165,386	1	G -> A		87	SNP (transition)	97.7%		939	GGC -> GGT	C770_RS47225	acyltransferase	None	3.7E-286	313	3	WP_015243261.1
1,165,401	1,165,403	2	CAA -> AAG		87	Substitution	100.0%		922	TTG -> CTT	C770_RS47225	acyltransferase	None	2.0E-270	308	1	WP_015243261.1
1,165,448	1,165,448	1	T -> C		90	SNP (transition)	98.9%	I -> V	877	ATC -> GTC	C770_RS47225	acyltransferase	Substitution	1.8E-292	293	1	WP_015243261.1
1,165,476	1,165,476	1	A -> G		96	SNP (transition)	99.0%		849	GGT -> GGC	C770_RS47225	acyltransferase	None	9.6E-303	283	3	WP_015243261.1
1,165,523	1,165,523	1	C -> T		90	SNP (transition)	96.7%	V -> M	802	GTG -> ATG	C770_RS47225	acyltransferase	Substitution	9.3E-283	268	1	WP_015243261.1
1,165,542	1,165,542	1	A -> G		85	SNP (transition)	98.8%		783	CCT -> CCC	C770_RS47225	acyltransferase	None	1.3E-267	261	3	WP_015243261.1
1,165,613	1,165,614	2	AT -> CC		79	Substitution	98.7%	LL -> LV	711	CTA,TTG -> CTG,	C770_RS47225	acyltransferase	Substitution	1.6E-253	237	3	WP_015243261.1
1,165,617	1,165,617	1	C -> T		79	SNP (transition)	97.5%		708	GGG -> GGA	C770_RS47225	acyltransferase	None	2.4E-251	236	3	WP_015243261.1
1,165,710	1,165,710	1	G -> A		79	SNP (transition)	100.0%		615	TTC -> TTT	C770_RS47225	acyltransferase	None	1.3E-245	205	3	WP_015243261.1
1,165,721	1,165,721	1	G -> A		79	SNP (transition)	98.7%		604	CTG -> TTG	C770_RS47225	acyltransferase	None	5.0E-264	202	1	WP_015243261.1
1,165,749	1,165,749	1	A -> T		76	SNP (transversion)	100.0%		576	ATT -> ATA	C770_RS47225	acyltransferase	None	4.0E-259	192	3	WP_015243261.1
1,165,806	1,165,806	1	G -> A		79	SNP (transition)	100.0%		519	ATC -> ATT	C770_RS47225	acyltransferase	None	2.0E-261	173	3	WP_015243261.1
1,165,878	1,165,878	1	C -> A		92	SNP (transversion)	98.9%		447	TCG -> TCT	C770_RS47225	acyltransferase	None	2.9E-317	149	3	WP_015243261.1
1,165,923	1,165,923	1	G -> A		94	SNP (transition)	98.9%		402	GAC -> GAT	C770_RS47225	acyltransferase	None	5.9E-315	134	3	WP_015243261.1
1,165,962	1,165,962	1	A -> T		97	SNP (transversion)	97.9%		363	ACT -> ACA	C770_RS47225	acyltransferase	None	4.7E-320	121	3	WP_015243261.1
1,165,965	1,165,965	1	G -> A		96	SNP (transition)	99.0%		360	TTC -> TTT	C770_RS47225	acyltransferase	None	3.0E-312	120	3	WP_015243261.1
1,165,968	1,165,968	1	C -> T		96	SNP (transition)	96.9%		357	TTG -> TTA	C770_RS47225	acyltransferase	None	9.0E-312	119	3	WP_015243261.1
1,166,196	1,166,196	1	A -> G		78	SNP (transition)	100.0%		129	TTT -> TTC	C770_RS47225	acyltransferase	None	1.0E-273	43	3	WP_015243261.1
1,166,214	1,166,214	1	G -> A		75	SNP (transition)	98.7%		111	AGC -> AGT	C770_RS47225	acyltransferase	None	1.9E-250	37	3	WP_015243261.1
1,166,361	1,166,360	0	(T)4 -> (T)5		78	Insertion (tandem repeat)	98.7%							7.8E-230			
1,166,424	1,166,424	1	A -> G		83	SNP (transition)	97.6%							2.1E-256			
1,166,437	1,166,437	1	A -> T		80	SNP (transversion)	100.0%							1.0E-272			
1,166,569	1,166,569	1	G -> A		90	SNP (transition)	100.0%		858	CCC -> CCT	C770_RS47230	arabinose-5-phosphate	None	1.0E-297	286	3	WP_010975429.1
1,166,627	1,166,627	1	C -> T		82	SNP (transition)	97.6%	S -> N	800	AGT -> AAT	C770_RS47230	arabinose-5-phosphate	Substitution	3.3E-237	267	2	WP_010975429.1
1,166,659	1,166,659	1	G -> T		81	SNP (transversion)	97.5%		768	ACC -> ACA	C770_RS47230	arabinose-5-phosphate	None	6.5E-258	256	3	WP_010975429.1
1,166,869	1,166,869	1	C -> A		68	SNP (transversion)	97.1%		558	GGG -> GGT	C770_RS47230	arabinose-5-phosphate	None	5.7E-202	186	3	WP_010975429.1
1,166,898	1,166,898	1	G -> A		75	SNP (transition)	96.0%	L -> F	529	CTT -> TTT	C770_RS47230	arabinose-5-phosphate	Substitution	1.7E-233	177	1	WP_010975429.1
1,166,911	1,166,911	1	T -> C		73	SNP (transition)	98.6%		516	GCA -> GCG	C770_RS47230	arabinose-5-phosphate	None	2.9E-229	172	3	WP_010975429.1
1,166,944	1,166,944	1	T -> A		75	SNP (transversion)	98.7%		483	CTA -> CTT	C770_RS47230	arabinose-5-phosphate	None	3.0E-228	161	3	WP_010975429.1
1,166,950	1,166,950	1	A -> G		77	SNP (transition)	96.1%		477	CAT -> CAC	C770_RS47230	arabinose-5-phosphate	None	1.8E-247	159	3	WP_010975429.1
1,166,986	1,166,986	1	G -> C		74	SNP (transversion)	98.6%		441	CCC -> CCG	C770_RS47230	arabinose-5-phosphate	None	4.7E-247	147	3	WP_010975429.1
1,166,998	1,166,998	1	A -> G		75	SNP (transition)	98.7%		429	AAT -> AAC	C770_RS47230	arabinose-5-phosphate	None	1.9E-250	143	3	WP_010975429.1
1,167,118	1,167,118	1	A -> G		96	SNP (transition)	99.0%		309	CTT -> CTC	C770_RS47230	arabinose-5-phosphate	None	3.0E-312	103	3	WP_010975429.1
1,167,123	1,167,123	1	G -> A		97	SNP (transition)	100.0%		304	CTG -> TTG	C770_RS47230	arabinose-5-phosphate	None	0.0	102	1	WP_010975429.1
1,167,327	1,167,327	1	T -> C		105	SNP (transition)	100.0%	N -> D	100	AAT -> GAT	C770_RS47230	arabinose-5-phosphate	Substitution	0.0	34	1	WP_010975429.1
1,167,457	1,167,457	1	T -> G		108	SNP (transversion)	98.1%							0.0			
1,167,459	1,167,459	1	T -> GC		108	Insertion	99.1%							0.0			
1,167,521	1,167,521	1	T -> C		109	SNP (transition)	100.0%							0.0			
1,167,547	1,167,547	1	C -> G		110	SNP (transversion)	98.2%							0.0			
1,167,658	1,167,658	1	A -> C		103	SNP (transversion)	98.1%							4.2E-310			
1,167,660	1,167,660	1	T -> G		103	SNP (transversion)	99.0%							0.0			
1,167,667	1,167,667	1	A -> C		105	SNP (transversion)	100.0%							0.0			
1,167,675	1,167,675	1	C -> T		110	SNP (transition)	99.1%							0.0			
1,167,681	1,167,681	1	C -> A		109	SNP (transversion)	100.0%							0.0			
1,167,699	1,167,700	2	GA -> AC		111	Substitution	99.1%							0.0			
1,167,769	1,167,769	1	G -> A		110	SNP (transition)	99.1%	G -> D	68	GGC -> GAC	C770_RS47235	hypothetical protein	Substitution	0.0	23	2	WP_014527380.1
1,167,840	1,167,841	2	CA -> TG	103 -> 104		Substitution	98.1%	H -> C	139	CAT -> TGT	C770_RS47235	hypothetical protein	Substitution	0.0	47	1	WP_014527380.1
1,167,926	1,167,926	1	C -> G		103	SNP (transversion)	99.0%		225	CGC -> CGG	C770_RS47235	hypothetical protein	None	0.0	75	3	WP_014527380.1
1,167,935	1,167,935	1	G -> C		100	SNP (transversion)	99.0%		234	TCG -> TCC	C770_RS47235	hypothetical protein	None	1.6E-315	78	3	WP_014527380.1
1,167,968	1,167,968	1	C -> T		97	SNP (transition)	100.0%		267	GGC -> GGT	C770_RS47235	hypothetical protein	None	7.9E-321	89	3	WP_014527380.1
1,167,990	1,167,990	1	T -> C		97	SNP (transition)	100.0%	S -> P	289	TCT -> CCT	C770_RS47235	hypothetical protein	Substitution	2.0E-301	97	1	WP_014527380.1
1,168,013	1,168,013	1	A -> G		93	SNP (transition)	97.8%		312	AAA -> AAG	C770_RS47235	hypothetical protein	None	2.1E-297	104	3	WP_014527380.1
1,168,173	1,168,173	1	A -> G		82	SNP (transition)	100.0%	M -> V	472	ATG -> GTG	C770_RS47235	hypothetical protein	Substitution	1.6E-279	158	1	WP_014527380.1
1,168,199	1,168,199	1	C -> G		85	SNP (transversion)	98.8%		498	GCC -> GCG	C770_RS47235	hypothetical protein	None	3.4E-259	166	3	WP_014527380.1
1,168,202	1,168,202	1	G -> C		85	SNP (transversion)	98.8%		501	GGG -> GGC	C770_RS47235	hypothetical protein	None	1.3E-267	167	3	WP_014527380.1
1,168,212	1,168,214	2	ACC -> GCG	82 -> 84		Substitution	98.8% -> 100.0%	T -> A	511	ACC -> GCG	C770_RS47235	hypothetical protein	Substitution	2.5E-271	171	1	WP_014527380.1
1,168,238	1,168,238	1	G -> T		84	SNP (transversion)	100.0%	E -> D	537	GAG -> GAT	C770_RS47235	hypothetical protein	Substitution	1.0E-252	179	3	WP_014527380.1
1,168,244	1,168,244	1	C -> G		84	SNP (transversion)	98.8%		543	GGC -> GGG	C770_RS47235	hypothetical protein	None	1.1E-272	181	3	WP_014527380.1
1,168,246	1,168,246	1	G -> A		84	SNP (transition)	100.0%	R -> K	545	AGA -> AAA	C770_RS47235	hypothetical protein	Substitution	1.6E-269	182	2	WP_014527380.1
1,168,253	1,168,253	1	C -> G		83	SNP (transversion)	98.8%		552	CGC -> CGG	C770_RS47235	hypothetical protein	None	2.1E-269	184	3	WP_014527380.1

1,168,265	1,168,265	1	G -> T	80	SNP (transversion)	98.8%		564	GCG -> GCT	C770_RS47235	hypothetical protein	None	2.0E-267	188	3	WP_014527380.1
1,168,283	1,168,283	1	C -> G	78	SNP (transversion)	98.7%		582	CTC -> CTG	C770_RS47235	hypothetical protein	None	3.1E-245	194	3	WP_014527380.1
1,168,295	1,168,295	1	A -> C	76	SNP (transversion)	100.0%	R -> S	594	AGA -> AGC	C770_RS47235	hypothetical protein	Substitution	1.6E-251	198	3	WP_014527380.1
1,168,308	1,168,308	1	G -> T	76	SNP (transversion)	100.0%	V -> F	607	GTC -> TTC	C770_RS47235	hypothetical protein	Substitution	4.0E-259	203	1	WP_014527380.1
1,168,356	1,168,356	1	G -> A	81	SNP (transition)	100.0%	V -> I	655	GTT -> ATT	C770_RS47235	hypothetical protein	Substitution	5.0E-268	219	1	WP_014527380.1
1,168,373	1,168,373	1	C -> T	80	SNP (transition)	100.0%		672	GAC -> GAT	C770_RS47235	hypothetical protein	None	1.0E-256	224	3	WP_014527380.1
1,168,446	1,168,446	1	G -> C	82	SNP (transversion)	100.0%	T -> S	1.607	ACT -> AGT	C770_RS47240	4-amino-4-deoxy-L-arat	Substitution	4.0E-263	536	2	WP_015243263.1
1,168,508	1,168,508	1	C -> G	83	SNP (transversion)	100.0%		1.545	GCG -> GCC	C770_RS47240	4-amino-4-deoxy-L-arat	None	1.0E-249	515	3	WP_015243263.1
1,168,518	1,168,518	1	A -> G	83	SNP (transition)	98.8%	V -> A	1.535	GTC -> GCC	C770_RS47240	4-amino-4-deoxy-L-arat	Substitution	5.2E-253	512	2	WP_015243263.1
1,168,531	1,168,531	1	G -> T	82	SNP (transversion)	97.6%	Q -> K	1.522	CAA -> AAA	C770_RS47240	4-amino-4-deoxy-L-arat	Substitution	3.3E-237	508	1	WP_015243263.1
1,168,539	1,168,539	1	C -> G	83	SNP (transversion)	100.0%	G -> A	1.514	GGC -> GCC	C770_RS47240	4-amino-4-deoxy-L-arat	Substitution	5.0E-258	505	2	WP_015243263.1
1,168,558	1,168,558	1	C -> T	86	SNP (transition)	98.8%	V -> I	1.495	GTT -> ATT	C770_RS47240	4-amino-4-deoxy-L-arat	Substitution	8.6E-271	499	1	WP_015243263.1
1,168,580	1,168,580	1	A -> T	88	SNP (transversion)	98.9%		1.473	GCT -> GCA	C770_RS47240	4-amino-4-deoxy-L-arat	None	7.0E-286	491	3	WP_015243263.1
1,168,607	1,168,607	1	C -> T	81	SNP (transition)	98.8%		1.446	CCG -> CCA	C770_RS47240	4-amino-4-deoxy-L-arat	None	8.1E-263	482	3	WP_015243263.1
1,168,610	1,168,610	1	C -> G	82	SNP (transversion)	98.8%		1.443	CCG -> CCC	C770_RS47240	4-amino-4-deoxy-L-arat	None	2.6E-282	481	3	WP_015243263.1
1,168,620	1,168,620	1	T -> C	79	SNP (transition)	100.0%	K -> R	1.433	AAA -> AGA	C770_RS47240	4-amino-4-deoxy-L-arat	Substitution	2.0E-261	478	2	WP_015243263.1
1,168,679	1,168,679	1	G -> A	73	SNP (transition)	100.0%		1.374	CTC -> CTT	C770_RS47240	4-amino-4-deoxy-L-arat	None	2.5E-234	458	3	WP_015243263.1
1,168,718	1,168,718	1	G -> C	80	SNP (transversion)	100.0%		1.335	GTC -> GTG	C770_RS47240	4-amino-4-deoxy-L-arat	None	1.0E-256	445	3	WP_015243263.1
1,168,826	1,168,826	1	A -> C	79	SNP (transversion)	100.0%		1.227	CTT -> CTG	C770_RS47240	4-amino-4-deoxy-L-arat	None	3.2E-277	409	3	WP_015243263.1
1,168,902	1,168,902	1	T -> C	87	SNP (transition)	98.9%	D -> G	1.151	GAC -> GGC	C770_RS47240	4-amino-4-deoxy-L-arat	Substitution	5.5E-274	384	2	WP_015243263.1
1,168,988	1,168,988	1	G -> C	94	SNP (transversion)	98.9%	I -> M	1.065	ATC -> ATG	C770_RS47240	4-amino-4-deoxy-L-arat	Substitution	4.7E-287	355	3	WP_015243263.1
1,169,027	1,169,027	1	G -> A	101	SNP (transition)	100.0%		1.026	GTC -> GTT	C770_RS47240	4-amino-4-deoxy-L-arat	None	4.9E-324	342	3	WP_015243263.1
1,169,054	1,169,054	1	C -> T	97	SNP (transition)	97.9%		999	GGG -> GGA	C770_RS47240	4-amino-4-deoxy-L-arat	None	1.5E-310	333	3	WP_015243263.1
1,169,138	1,169,138	1	C -> T	106	SNP (transition)	99.1%		915	CTG -> CTA	C770_RS47240	4-amino-4-deoxy-L-arat	None	0.0	305	3	WP_015243263.1
1,169,146	1,169,146	1	A -> C	105	SNP (transversion)	100.0%	S -> A	907	TCG -> GCG	C770_RS47240	4-amino-4-deoxy-L-arat	Substitution	0.0	303	1	WP_015243263.1
1,169,330	1,169,330	1	A -> T	98	SNP (transversion)	98.0%	N -> K	723	AAT -> AAA	C770_RS47240	4-amino-4-deoxy-L-arat	Substitution	1.2E-294	241	3	WP_015243263.1
1,169,373	1,169,373	1	A -> G	100	SNP (transition)	98.0%	V -> A	680	GTG -> GCG	C770_RS47240	4-amino-4-deoxy-L-arat	Substitution	2.0E-320	227	2	WP_015243263.1
1,169,396	1,169,396	1	T -> C	100	SNP (transition)	97.0%		657	AAA -> AAG	C770_RS47240	4-amino-4-deoxy-L-arat	None	1.3E-315	219	3	WP_015243263.1
1,169,399	1,169,399	1	C -> G	98	SNP (transversion)	96.9%		654	TCG -> TCC	C770_RS47240	4-amino-4-deoxy-L-arat	None	1.5E-299	218	3	WP_015243263.1
1,169,446	1,169,446	1	T -> C	96	SNP (transition)	99.0%	S -> G	607	AGC -> GGC	C770_RS47240	4-amino-4-deoxy-L-arat	Substitution	3.0E-293	203	1	WP_015243263.1
1,169,477	1,169,477	1	A -> G	96	SNP (transition)	100.0%		576	TTT -> TTC	C770_RS47240	4-amino-4-deoxy-L-arat	None	1.6E-317	192	3	WP_015243263.1
1,169,595	1,169,595	1	G -> A	91	SNP (transition)	100.0%	A -> V	458	GCT -> GTT	C770_RS47240	4-amino-4-deoxy-L-arat	Substitution	7.9E-283	153	2	WP_015243263.1
1,169,630	1,169,630	1	C -> G	89	SNP (transversion)	98.9%		423	GGG -> GGC	C770_RS47240	4-amino-4-deoxy-L-arat	None	8.9E-263	141	3	WP_015243263.1
1,169,690	1,169,690	1	G -> A	86	SNP (transition)	100.0%		363	TCC -> TCT	C770_RS47240	4-amino-4-deoxy-L-arat	None	1.0E-301	121	3	WP_015243263.1
1,169,837	1,169,837	1	A -> G	95	SNP (transition)	100.0%		216	ATT -> ATC	C770_RS47240	4-amino-4-deoxy-L-arat	None	0.0	72	3	WP_015243263.1
1,169,920	1,169,920	1	C -> T	98	SNP (transition)	100.0%	D -> N	133	GAT -> AAT	C770_RS47240	4-amino-4-deoxy-L-arat	Substitution	2.5E-314	45	1	WP_015243263.1
1,169,922	1,169,922	1	T -> C	99	SNP (transition)	100.0%	D -> G	131	GAC -> GGC	C770_RS47240	4-amino-4-deoxy-L-arat	Substitution	1.6E-317	44	2	WP_015243263.1
1,169,943	1,169,943	1	A -> C	98	SNP (transversion)	100.0%	I -> S	110	ATT -> AGT	C770_RS47240	4-amino-4-deoxy-L-arat	Substitution	4.9E-324	37	2	WP_015243263.1
1,169,968	1,169,968	1	C -> G	104	SNP (transversion)	100.0%	V -> L	85	GTA -> CTA	C770_RS47240	4-amino-4-deoxy-L-arat	Substitution	0.0	29	1	WP_015243263.1
1,170,027	1,170,027	1	T -> C	112	SNP (transition)	98.2%	Y -> C	26	TAT -> TGT	C770_RS47240	4-amino-4-deoxy-L-arat	Substitution	0.0	9	2	WP_015243263.1
1,170,042	1,170,042	1	C -> G	110	SNP (transversion)	100.0%	G -> A	11	GGG -> GCG	C770_RS47240	4-amino-4-deoxy-L-arat	Substitution	0.0	4	2	WP_015243263.1
1,170,058	1,170,058	1	C -> G	112	SNP (transversion)	99.1%							0.0			
1,170,080	1,170,085	6	TATCGG	108	Deletion	100.0%							6.3E-152			
1,170,319	1,170,319	1	G -> A	87	SNP (transition)	98.9%		135	GAG -> GAA	C770_RS47245	hypothetical protein	None	5.5E-274	45	3	WP_010975426.1
1,170,454	1,170,454	1	C -> T	72	SNP (transition)	98.6%		270	ATC -> ATT	C770_RS47245	hypothetical protein	None	4.5E-226	90	3	WP_010975426.1
1,170,963	1,170,963	1	G -> A	83	SNP (transition)	97.6%							1.7E-264			
1,171,090	1,171,090	1	C -> A	88	SNP (transversion)	100.0%	S -> I	818	AGC -> ATC	C770_RS47250	hypothetical protein	Substitution	4.0E-291	273	2	WP_015243264.1
1,171,315	1,171,315	1	C -> T	99	SNP (transition)	98.0%	G -> E	593	GGA -> GAA	C770_RS47250	hypothetical protein	Substitution	2.4E-278	198	2	WP_015243264.1
1,171,358	1,171,358	1	G -> C	91	SNP (transversion)	100.0%	P -> A	550	CCG -> GCG	C770_RS47250	hypothetical protein	Substitution	5.0E-301	184	1	WP_015243264.1
1,171,365	1,171,365	1	T -> A	97	SNP (transversion)	96.9%		543	CGA -> CGT	C770_RS47250	hypothetical protein	None	9.3E-306	181	3	WP_015243264.1
1,171,368	1,171,368	1	T -> C	97	SNP (transition)	100.0%		540	CTA -> CTG	C770_RS47250	hypothetical protein	None	0.0	180	3	WP_015243264.1
1,171,431	1,171,432	2	TT -> CG	80 -> 81	Substitution	96.3%	Q -> P	476	CAA -> CCG	C770_RS47250	hypothetical protein	Substitution	8.1E-266	159	2	WP_015243264.1
1,171,434	1,171,434	1	T -> C	81	SNP (transition)	100.0%		474	AGA -> AGG	C770_RS47250	hypothetical protein	None	5.0E-268	158	3	WP_015243264.1
1,171,479	1,171,479	1	G -> C	81	SNP (transversion)	100.0%		429	GTC -> GTG	C770_RS47250	hypothetical protein	None	4.0E-276	143	3	WP_015243264.1
1,171,488	1,171,488	1	G -> A	81	SNP (transition)	100.0%		420	TCC -> TCT	C770_RS47250	hypothetical protein	None	5.0E-268	140	3	WP_015243264.1
1,171,502	1,171,503	2	TA -> CG	83 -> 84	Substitution	98.8%	CI -> CV	405	TGT,ATC -> TGC,	C770_RS47250	hypothetical protein	Substitution	2.7E-289	135	3	WP_015243264.1
1,171,563	1,171,563	1	G -> A	78	SNP (transition)	97.4%		345	ATC -> ATT	C770_RS47250	hypothetical protein	None	4.8E-248	115	3	WP_015243264.1
1,171,608	1,171,608	1	C -> T	88	SNP (transition)	98.9%		300	CTG -> CTA	C770_RS47250	hypothetical protein	None	3.5E-277	100	3	WP_015243264.1
1,171,642	1,171,642	1	G -> T	95	SNP (transversion)	96.8%	A -> D	266	GCC -> GAC	C770_RS47250	hypothetical protein	Substitution	5.5E-290	89	2	WP_015243264.1
1,171,698	1,171,698	1	G -> A	98	SNP (transition)	98.0%		210	AAC -> AAT	C770_RS47250	hypothetical protein	None	7.5E-314	70	3	WP_015243264.1
1,171,702	1,171,702	1	G -> C	101	SNP (transversion)	99.0%	A -> G	206	GCG -> GGG	C770_RS47250	hypothetical protein	Substitution	1.0E-308	69	2	WP_015243264.1
1,171,725	1,171,725	1	T -> C	101	SNP (transition)	100.0%		183	TTA -> TTG	C770_RS47250	hypothetical protein	None	0.0	61	3	WP_015243264.1
1,171,764	1,171,764	1	C -> T	106	SNP (transition)	100.0%		144	CAG -> CAA	C770_RS47250	hypothetical protein	None	0.0	48	3	WP_015243264.1
1,171,904	1,171,904	1	T -> C	110	SNP (transition)	99.1%	I -> V	4	ATT -> GTT	C770_RS47250	hypothetical protein	Substitution	0.0	2	1	WP_015243264.1
1,171,964	1,171,964	1	C -> A	101	SNP (transversion)	99.0%							0.0			
1,172,011	1,172,012	2	TT -> CC	96 -> 97	Substitution	100.0%	SK -> SE	690	TCA,AAA -> TCG,	C770_RS47255	hypothetical protein	Substitution	0.0	230	3	WP_010975424.1
1,172,165	1,172,165	1	A -> G	70	SNP (transition)	100.0%		537	CCT -> CCC	C770_RS47255	hypothetical protein	None	1.0E-217	179	3	WP_010975424.1
1,172,230	1,172,230	1	C -> T	66	SNP (transition)	100.0%	D -> N	472	GAC -> AAC	C770_RS47255	hypothetical protein	Substitution	6.3E-212	158	1	WP_010975424.1
1,172,294	1,172,294	1	G -> A	68	SNP (transition)	100.0%		408	ATC -> ATT	C770_RS47255	hypothetical protein	None	4.0E-225	136	3	WP_010975424.1
1,172,310	1,172,310	1	A -> T	66	SNP (transversion)	97.0%	F -> Y	392	TTC -> TAC	C770_RS47255	hypothetical protein	Substitution	2.1E-221	131	2	WP_010975424.1
1,172,393	1,172,393	1	G -> A	72	SNP (transition)	100.0%		309	CGC -> CGT	C770_RS47255	hypothetical protein	None	1.6E-245	103	3	WP_010975424.1
1,172,399	1,172,399	1	C -> G	71	SNP (transversion)	100.0%		303	GGG -> GGC	C770_RS47255	hypothetical protein	None	5.0E-235	101	3	WP_010975424.1
1,172,860	1,172,860	1	A -> G	92	SNP (transition)	97.8%		715	TTG -> CTG	C770_RS47260	hypothetical protein	None	4.2E-294	239	1	WP_015243265.1
1,173,077	1,173,077	1	C -> T	99	SNP (transition)	97.0%		498	CTG -> CTA	C770_RS47260	hypothetical protein	None	3.9E-293	166	3	WP_015243265.1
1,173,110	1,173,110	1	T -> G	96	SNP (transversion)	99.0%		465	CTA -> CTC	C770_RS47260	hypothetical protein	None	3.0E-293	155	3	WP_015243265.1
1,173,116	1,173,116	1	T -> C	93	SNP (transition)	97.8%		459	CTA -> CTG	C770_RS47260	hypothetical protein	None	2.1E-297	153	3	WP_015243265.1
1,173,122	1,173,122	1	G -> C	90	SNP (transversion)	100.0%	I -> M	453	ATC -> ATG	C770_RS47260	hypothetical protein	Substitution	1.0E-279	151	3	WP_015243265.1
1,173,211	1,173,211	1	G -> A	99	SNP (transition)	99.0%		364	CTG -> TTG	C770_RS47260	hypothetical protein	None	1.6E-302	122	1	WP_015243265.1
1,173,215	1,173,215	1	A -> G	99	SNP (transition)	99.0%		360	GAT -> GAC	C770_RS47260	hypothetical protein	None	0.0	120	3	WP_015243265.1
1,173,298	1,173,298	1	C -> T	94	SNP (transition)	100.0%	V -> I	277	GTC -> ATC	C770_RS47260	hypothetical protein	Substitution	2.5E-320	93	1	WP_015243265.1
1,173,332	1,173,332	1	T -> G	91	SNP (transversion)	100.0%		243	GCA -> GCC	C770_RS47260	hypothetical protein	None	4.0E-310	81	3	WP_015243265.1
1,173,341	1,173,341	1	C -> G	91	SNP (transversion)	100.0%		234	CCG -> CCC	C770_RS47260	hypothetical protein	None	6.3E-292	78	3	WP_015243265.1
1,173,348	1,173,348	1	G -> C	90	SNP (transversion)	100.0%	A -> G	227	GCT -> GGT	C770_RS47260	hypothetical protein	Substitution	1.0E-297	76	2	WP_015243265.1
1,173,394	1,173,394	1	T -> C	86	SNP (transition)	100.0%	I -> V	181	ATT -> GTT	C770_RS47260	hypothetical protein	Substitution	6.3E-276	61	1	WP_015243265.1
1,173,444	1,173,444	1	G -> C	92	SNP (transversion)	97.8%	A -> G	131	GCC -> GGC	C770_RS47260	hypothetical protein	Substitution	4.2E-294	44	2	WP_015243265.1
1,173,482	1,173,482	1	T -> C	82	SNP (transition)	98.8%		93	GTA -> GTG	C770_RS47260	hypothetical protein	None	3.3E-274	31	3	WP_015243265.1
1,173,514	1,173,514	1	A -> G	70	SNP (transition)	98.6%		61	TTG -> CTG	C770_RS47260	hypothetical protein	None	2.2E-240	21	1	WP_015243265.1
1,173,520	1,173,520	1	A -> G	69	SNP (transition)	98.6%	C -> R	55	TGT -> CGT	C770_RS47260	hypothetical protein	Substitution	6.9E-237	19	1	WP_015243265.1
1,173,576	1,173,576	1	T -> G	56	SNP (transversion)	98.2%							1.8E-180			
1,173,605	1,173,605	1	G -> A	48	SNP (transition)	95.8%							1.1E-158			
1,174,820	1,174,820	1	G -> A	9	SNP (transition)	100.0%	S -> N	44	AGC -> AAC	C770_RS47270	nucleoside-diphosphate	Substitution	5.0E-25	15	2	WP_015243267.1
1,174,823	1,174,823	1	G -> A	11	SNP (transition)	100.0%	C -> Y	47	TGC -> TAC	C770_RS47270	nucleoside-diphosphate	Substitution	6.3E-36	16	2	WP_015243267.1
1,174,869	1,174,869	1	T -> C	23	SNP (transition)	100.0%		93	TGT -> TGC	C770_RS47270	nucleoside-diphosphate	None	1.6E-83	31	3	WP_015243267.1
1,174,878	1,174,878	1	T -> G	25	SNP (transversion)	100.0%		102	GTT -> GTG	C770_RS47270	nucleoside-diphosphate	None	3.2E-78	34	3	WP_015243267.1
1,174,881	1,174,881	1	A -> T	25	SNP (transversion)	100.0%		105	GGA -> GGT	C770_RS47270	nucleoside-diphosphate	None	1.0E-90	35	3	WP_015243267.1
1,174,887	1,174,887	1	G -> A	26	SNP (transition)	100.0%		111	CAG -> CAA	C770_RS47270	nucleoside-diphosphate	None	4.0E-89	37	3	WP_015243267.1
1,174,893	1,174,893	1	C -> A	27	SNP (transversion)	100.0%	H -> Q	117	CAC -> CAA	C770_RS47270	nucleoside-diphosphate	Substitution	3.2E-95	39	3	WP_015243267.1
1,174,896	1,174,896	1	G -> T	28	SNP (transversion)	100.0%		120	CGG -> CGT	C770_RS47270	nucleoside-diphosphate	None	4.0E-93	40	3	WP_015243267.1
1,174,902	1,174,902	1	G -> A	29	SNP (transition)	100.0%		126	CCG -> CCA	C770_RS47270	nucleoside-diphosphate	None	2.5E-99	42	3	WP_015243267.1
1,174,908	1,174,908	1	A -> C	29	SNP (transversion)	100.0%		132	GTA -> GTC	C770_RS47270	nucleoside-diphosphate	None	2.5E-99	44	3	WP_015243267.1
1,174,912	1,174,914	2	CTG -> TTA	30 -> 33	Substitution	100.0%		136	CTG -> TTA	C770_RS47270	nucleoside-diphosphate	None	1.0E-96	46	1	WP_015243267.1
1,174,941	1,174,941	1	A -> G	39	SNP (transition)	100.0%		165	AAA -> AAG	C770_RS47270	nucleoside-diphosphate	None	3.2E-137	55	3	WP_015243267.1
1,174,957	1,174,957	1	C -> T	41	SNP (transition)	100.0%		181	CTG -> TTG	C770_RS47270	nucleoside-diphosphate	None	4.0E-140	61	1	WP_015243267.1
1,174,971	1,174,971	1	A -> G	44	SNP (transition)	100.0%		195	AGA -> AGG	C770_RS47270	nucleoside-diphosphate	None	2.5E-150	65	3	WP_015243267.1
1,174,983	1,174,983	1	G -> C	45	SNP (transversion)	100.0%		207	CCG -> CCC	C770_RS47270	nucleoside-diphosphate	None	1.0E-153	69	3	WP_015243267.1
1,174,986	1,174,986	1	A -> G	45	SNP (transition)	100.0%		210	GAA -> GAG	C770_RS47270	nucleoside-diphosphate	None	1.0E-153	70	3	WP_015243267.1
1,174,998	1,174,998	1	G -> C	48	SNP (transversion)	100.0%		222	TCG -> TCC	C770_RS47270	nucleoside-diphosphate	None	1.0E-168	74	3	WP_015243267.1
1,175,019	1,175,019	1	T -> G	56	SNP (transversion)	98.2%		243	CCT -> CCG	C770_RS47270	nucleoside-diphosphate	None	1.8E-180	81	3	WP_015243267.1
1,175,022	1,175,022	1	G -> A	56	SNP (transition)	100.0%		246	GCG -> GCA	C770_RS47270	nucleoside-diphosphate	None	1.0E-196	82	3	WP_015243267.1
1,175,034	1,175,034	1	C -> A	61	SNP (transversion)	98.4%		258	ATC -> ATA	C770_RS47270	nucleoside-diphosphate	None	6.1E-197	86	3	WP_015243267.1
1,175,049	1,175,049	1	C -> A	67	SNP (transversion)	100.0%		273	GGC -> GGA	C770_RS47270	nucleoside-diphosphate	None	7.9E-222	91	3	WP_015243267.1
1,175,052	1,175,052	1	T -> C	68	SNP (transition)	100.0%		276	GGT -> GGC	C770_RS47270	nucleoside-diphosphate	None	1.0E-238	92	3	WP_015243267.1
1,175,058	1,175,061	3	TCTC -> CTTA	72 -> 73	Substitution	100.0%		282	CCT,CTC -> CCC,T	C770_RS47270	nucleoside-diphosphate	None	5.0E-227	94	3	WP_015243267.1
1,175,070	1,175,070	1	C -> T	76	SNP (transition)	97.4%		294	CAC -> CAT	C770_RS47270	nucleoside-diphosphate	None	7.2E-249	98	3	WP_015243267.1
1,175,103	1,175,103	1	T -> C	82	SNP (transition)	100.0%		327	CTT -> CTC	C770_RS47270	nucleoside-diphosphate	None	4.0E-263	109	3	WP_015243267.1
1,175,140	1,175,140	1	G -> A	79	SNP (transition)	98.7%	D -> N	364	GAC -> AAC	C770_RS47270	nucleoside-diphosphate	Substitution	2.0E-248	122	1	WP_015243267.1
1,175,144	1,175,144	1	A -> G	79	SNP (transition)	100.0%	Q -> R	368	CAG -> CGG	C770_RS47270	nucleoside-diphosphate	Substitution	1.0E-237	123	2	WP_015243267.1
1,175,160	1,175,160	1	C -> T	80	SNP (transition)	100.0%		384	CAC -> CAT	C770_RS47270	nucleoside-diphosphate	None	1.0E-256	128	3	WP_015243267.1
1,175,192	1,175,192	1	C -> G	81	SNP (transversion)	98.8%	S -> W	416	TCG -> TGG	C770_RS47270	nucleoside-diphosphate	Substitution	8.1E-255	139	2	WP_015243267.1
1,175,225	1,175,225	1	G -> T	77	SNP (transversion)	98.7%	R -> L	449	CGC -> CTC	C770_RS47270	nucleoside-diphosphate	Substitution	1.2E-249	150	2	WP_015243267.1
1,175,229	1,175,229	1	G -> A	79	SNP (transition)	98.7%		453	AGG -> AGA	C770_RS47270	nucleoside-diphosphate	None	5.0E-264	151	3	WP_015243267.1
1,175,238	1,175,238	1	G -> A	83	SNP (transition)	98.8%		462	TCG -> TCA	C770_RS47270	nucleoside-diphosphate	None	1.3E-277	154	3	WP_015243267.1
1,175,244	1,175,244	1	A -> C	84	SNP (transversion)	97.6%	E -> D	468	GAA -> GAC	C770_RS47270	nucleoside-diphosphate	Substitution	8.7E-268	156	3	WP_015243267.1
1,175,247	1,175,247	1	C -> G	84	SNP (transversion)	100.0%		471	GGC -> GGG	C770_RS47270	nucleoside-diphosphate	None	4.0E-261	157	3	WP_015243267.1
1,175,253	1,175,253	1	G -> A	85	SNP (transition)	97.6%		477	CCG -> CCA	C770_RS47270	nucleoside-diphosphate	None	9.0E-263	159	3	WP_015243267.1
1,175,280	1,175,280	1	A -> G	89	SNP (transition)	100.0%		504	TCA -> TCG	C770_RS47270	nucleoside-diphosphate	None	1.6E-285	168	3	WP_015243267.1
1,175,286	1,175,286	1	C -> A	90	SNP (transversion)	98.9%		510	GGC -> GGA	C770_RS47270	nucleoside-diphosphate	None	1.8E-292	170	3	WP_015243267.1
1,175,289	1,175,289	1	C -> A	91	SNP (transversion)	100.0%		513	GCC -> GCA	C770_RS47270	nucleoside-diphosphate	None	4.0E-310	171	3	WP_015243267.1
1,175,298	1,175,298	1	T -> C	94	SNP (transition)	100.0%		522	TAT -> TAC	C770_RS47270	nucleoside-diphosphate	None	6.3E-311	174	3	WP_015243267.1
1,175,306	1,175,306	1	A -> G	91	SNP (transition)	100.0%	K -> R	530	AAA -> AGA	C770_RS47270	nucleoside-diphosphate	Substitution	4.0E-310	177	2	WP_015243267.1
1,175,315	1,175,316	2	AG -> GA	90	Substitution	97.8%	Q -> R	539	CAG -> CGA	C770_RS47270	nucleoside-diphosphate	Substitution	1.1E-274	180	2	WP_015243267.1
1,175,326	1,175,326	1	T -> G	90	SNP (transversion)	98.9%	C -> G	550	TGC -> GGC	C770_RS47270	nucleoside-diphosphate	Substitution	1.4E-283	184	1	WP_015243267.1
1,175,331	1,175,332	2	TG -> AA	89 -> 91	Substitution	100.0%	AV -> AI	555	GCT,GTC -> GCA,	C770_RS47270	nucleoside-diphosphate	Substitution	1.6E-285	185	3	WP_015243267.1
1,175,337	1,175,337	1	A -> G	90	SNP (transition)	100.0%		561	GAA -> GAG	C770_RS47270	nucleoside-diphosphate	None	1.0E-297	187	3	WP_015243267.1
1,175,351	1,175,351	1	C -> A	88	SNP (transversion)	95.5%	A -> E	575	GCG -> GAG	C770_RS47270	nucleoside-diphosphate	Substitution	3.7E-263	192	2	WP_015243267.1
1,175,355	1,175,355	1	T -> C	85	SNP (transition)	100.0%		579	GGT -> GGC	C770_RS47270	nucleoside-diphosphate	None	1.0E-272	193	3	WP_015243267.1
1,175,370	1,175,370	1	G -> A	85	SNP (transition)	100.0%		594	GTG -> GTA	C770_RS47270	nucleoside-diphosphate	None	1.0E-289	198	3	WP_015243267.1
1,175,382	1,175,382	1	G -> T	85	SNP (transversion)	98.8%		606	CGG -> CGT	C770_RS47270	nucleoside-diphosphate	None	2.1E-284	202	3	WP_015243267.1
1,175,400	1,175,400	1	T -> A	89	SNP (transversion)	97.8%		624	ACT -> ACA	C770_RS47270	nucleoside-diphosphate	None	3.1E-284	208	3	WP_015243267.1
1,175,405	1,175,407	3	AAA -> GGG	89	Substitution	97.8%	QT -> RA	629	CAA,ACC -> CGG,	C770_RS47270	nucleoside-diphosphate	Substitution	1.4E-271	210	2	WP_015243267.1
1,175,418	1,175,418	1	G -> A	86	SNP (transition)	98.8%		642	ACG -> ACA	C770_RS47270	nucleoside-diphosphate	None	2.7E-296	214	3	WP_015243267.1
1,175,433	1,175,433	1	C -> G	84	SNP (transversion)	97.6%	S -> R	657	AGC -> AGG	C770_RS47270	nucleoside-diphosphate	Substitution	1.4E-259	219	3	WP_015243267.1
1,175,436	1,175,436	1	C -> T	84	SNP (transition)	98.8%		660	ATC -> ATT	C770_RS47270	nucleoside-diphosphate	None	2.1E-264	220	3	WP_015243267.1
1,175,452	1,175,452	1	A -> C	85	SNP (transversion)	98.8%		676	AGA -> CGA	C770_RS47270	nucleoside-diphosphate	None	2.1E-284	226	1	WP_015243267.1
1,175,490	1,175,490	1	C -> T	88	SNP (transition)	100.0%		714	GCC -> GCT	C770_RS47270	nucleoside-diphosphate	None	6.3E-300	238	3	WP_015243267.1
1,175,508	1,175,508	1	G -> A	91	SNP (transition)	96.7%		732	GCG -> GCA	C770_RS47270	nucleoside-diphosphate	None	3.0E-277	244	3	WP_015243267.1
1,175,529	1,175,529	1	G -> C	91	SNP (transversion)	100.0%		753	CCG -> CCC	C770_RS47270	nucleoside-diphosphate	None	1.0E-273	251	3	WP_015243267.1
1,175,572	1,175,572	1	C -> T	93	SNP (transition)	98.9%		796	CTA -> TTA	C770_RS47270	nucleoside-diphosphate	None	3.7E-293	266	1	WP_015243267.1
1,175,625	1,175,625	1	C -> T	82	SNP (transition)	97.6%		849	GAC -> GAT	C770_RS47270	nucleoside-diphosphate	None	3.3E-261	283	3	WP_015243267.1
1,175,637	1,175,637	1	C -> T	82	SNP (transition)	100.0%		861	ACC -> ACT	C770_RS47270	nucleoside-diphosphate	None	4.0E-263	287	3	WP_015243267.1
1,175,649	1,175,649	1	G -> C	87	SNP (transversion)	100.0%		873	ACG -> ACC	C770_RS47270	nucleoside-diphosphate	None	7.9E-288	291	3	WP_015243267.1
1,175,664	1,175,664	1	C -> A	85	SNP (transversion)	98.8%		888	GGC -> GGA	C770_RS47270	nucleoside-diphosphate	None	1.3E-267	296	3	WP_015243267.1
1,175,667	1,175,667	1	A -> G	84	SNP (transition)	98.8%		891	AAA -> AAG	C770_RS47270	nucleoside-diphosphate	None	5.3E-281	297	3	WP_015243267.1
1,175,675	1,175,675	1	C -> G	85	SNP (transversion)	100.0%	P -> R	899	CCC -> CGC	C770_RS47270	nucleoside-diphosphate	Substitution	3.2E-281	300	2	WP_015243267.1
1,175,706	1,175,706	1	G -> C	86	SNP (transversion)	98.8%		930	CGG -> CGC	C770_RS47270	nucleoside-diphosphate	None	2.7E-262	310	3	WP_015243267.1
1,175,730	1,175,730	1	A -> C	89	SNP (transversion)	98.9%		954	TCA -> TCC	C770_RS47270	nucleoside-diphosphate	None	2.2E-280	318	3	WP_015243267.1
1,175,750	1,175,750	1	G -> A	89	SNP (transition)	96.6%							7.2E-271			
1,175,756	1,175,756	1	A	88	Deletion	98.9%							5.4E-138			
1,175,801	1,175,801	1	G -> C	86	SNP (transversion)	96.5%		816	ACC -> ACG	C770_RS47275	hypothetical protein	None	1.3E-269	272	3	WP_015243268.1
1,175,853	1,175,853	1	T -> C	82	SNP (transition)	97.6%	Q -> R	764	CAA -> CGA	C770_RS47275	hypothetical protein	Substitution	3.3E-261	255	2	WP_015243268.1

1,176,002	1,176,002	1	G -> A		83	SNP (transition)	96.4%		615	TAC -> TAT	C770_RS47275	hypothetical protein	None	9.2E-244	205	3	WP_015243268.1
1,176,191	1,176,191	1	G -> A		81	SNP (transition)	98.8%		426	TTC -> TTT	C770_RS47275	hypothetical protein	None	8.1E-255	142	3	WP_015243268.1
1,176,221	1,176,221	1	C -> T		87	SNP (transition)	98.9%		396	CCG -> CCA	C770_RS47275	hypothetical protein	None	3.5E-291	132	3	WP_015243268.1
1,176,250	1,176,250	1	C -> T		90	SNP (transition)	97.8%	V -> I	367	GTA -> ATA	C770_RS47275	hypothetical protein	Substitution	1.6E-287	123	1	WP_015243268.1
1,176,279	1,176,279	1	A -> G		91	SNP (transition)	97.8%	V -> A	338	GTT -> GCT	C770_RS47275	hypothetical protein	Substitution	6.5E-282	113	2	WP_015243268.1
1,176,329	1,176,329	1	A -> G		85	SNP (transition)	96.5%		288	GGT -> GGC	C770_RS47275	hypothetical protein	None	6.2E-250	96	3	WP_015243268.1
1,176,365	1,176,365	1	C -> G		86	SNP (transversion)	98.8%		252	GCG -> GCC	C770_RS47275	hypothetical protein	None	8.6E-288	84	3	WP_015243268.1
1,176,443	1,176,443	1	C -> G		96	SNP (transversion)	100.0%		174	CCG -> CCC	C770_RS47275	hypothetical protein	None	2.5E-298	58	3	WP_015243268.1
1,176,802	1,176,802	1	(T)3 -> (T)2		113	Deletion (tandem repeat)	100.0%							3.2E-170			
1,176,815	1,176,815	1	G -> A		114	SNP (transition)	98.2%							0.0			
1,176,825	1,176,825	1	G -> T		109	SNP (transversion)	99.1%							0.0			
1,176,830	1,176,830	1	G -> A		107	SNP (transition)	100.0%							0.0			
1,176,880	1,176,880	1	A -> C		101	SNP (transversion)	100.0%							0.0			
1,176,885	1,176,885	1	G -> C		101	SNP (transversion)	100.0%							0.0			
1,176,908	1,176,908	1	C -> G		103	SNP (transversion)	99.0%							0.0			
1,176,928	1,176,928	1	T -> C		100	SNP (transition)	98.0%							2.0E-320			
1,176,937	1,176,937	1	A -> C		104	SNP (transversion)	100.0%							0.0			
1,176,942	1,176,942	1	C -> G		102	SNP (transversion)	100.0%							6.3E-317			
1,176,954	1,176,954	1	A -> G		103	SNP (transition)	100.0%							0.0			
1,177,028	1,177,028	1	T -> C		98	SNP (transition)	100.0%							2.5E-314			
1,177,098	1,177,098	1	C -> A		100	SNP (transversion)	100.0%	Q -> K	67	CAA -> AAA	C770_RS47280	hypothetical protein	Substitution	1.0E-310	23	1	WP_015243269.1
1,177,123	1,177,123	1	A -> T		100	SNP (transversion)	99.0%	D -> V	92	GAT -> GTT	C770_RS47280	hypothetical protein	Substitution	1.6E-315	31	2	WP_015243269.1
1,177,146	1,177,146	1	A -> T		102	SNP (transversion)	99.0%	T -> S	115	ACC -> TCC	C770_RS47280	hypothetical protein	Substitution	0.0	39	1	WP_015243269.1
1,177,166	1,177,166	1	C -> G		92	SNP (transversion)	98.9%	D -> E	135	GAC -> GAG	C770_RS47280	hypothetical protein	Substitution	2.9E-317	45	3	WP_015243269.1
1,177,181	1,177,181	1	T -> A		91	SNP (transversion)	98.9%		150	GTT -> GTA	C770_RS47280	hypothetical protein	None	9.1E-296	50	3	WP_015243269.1
1,177,193	1,177,193	1	G -> T		95	SNP (transversion)	98.9%		162	GCG -> GCT	C770_RS47280	hypothetical protein	None	2.4E-318	54	3	WP_015243269.1
1,177,211	1,177,211	1	T -> C		93	SNP (transition)	100.0%		180	TAT -> TAC	C770_RS47280	hypothetical protein	None	2.5E-298	60	3	WP_015243269.1
1,177,250	1,177,250	1	T -> C		90	SNP (transition)	100.0%		219	GAT -> GAC	C770_RS47280	hypothetical protein	None	1.0E-297	73	3	WP_015243269.1
1,177,255	1,177,256	2	AT -> TC		90	Substitution	100.0%							1.0E-288			
1,177,260	1,177,260	1	G -> A		93	SNP (transition)	97.8%							1.7E-306			
1,177,264	1,177,264	1	A -> T		93	SNP (transversion)	100.0%							1.3E-307			
1,177,271	1,177,271	1	(A)3 -> (A)2		94	Deletion (tandem repeat)	100.0%							4.0E-151			
1,177,293	1,177,293	1	G -> T		96	SNP (transversion)	99.0%							9.6E-322			
1,177,300	1,177,299	0	(GC)2 -> (GC)3		96	Insertion (tandem repeat)	99.0%							1.6E-317			
1,177,321	1,177,322	2	TA -> CT		96	Substitution	100.0%							1.6E-317			
1,177,340	1,177,340	1	G -> A		96	SNP (transition)	100.0%							0.0			
1,177,373	1,177,373	1	C -> A		85	SNP (transversion)	97.6%							1.1E-287			
1,177,451	1,177,451	1	G -> C		82	SNP (transversion)	100.0%	L -> V	526	CTT -> GTT	C770_RS47285	hypothetical protein	Substitution	1.6E-279	176	1	WP_041169896.1
1,177,512	1,177,512	1	A -> C		87	SNP (transversion)	100.0%		465	GCT -> GCG	C770_RS47285	hypothetical protein	None	1.6E-296	155	3	WP_041169896.1
1,177,529	1,177,529	1	T -> C		88	SNP (transition)	97.7%	K -> E	448	AAG -> GAG	C770_RS47285	hypothetical protein	Substitution	6.1E-281	150	1	WP_041169896.1
1,177,593	1,177,593	1	C -> T		77	SNP (transition)	100.0%		384	GAG -> GAA	C770_RS47285	hypothetical protein	None	7.9E-255	128	3	WP_041169896.1
1,177,597	1,177,597	1	A -> C		78	SNP (transversion)	100.0%	I -> S	380	ATC -> AGC	C770_RS47285	hypothetical protein	Substitution	6.3E-266	127	2	WP_041169896.1
1,177,615	1,177,615	1	A -> G		71	SNP (transition)	100.0%	M -> T	362	ATG -> ACG	C770_RS47285	hypothetical protein	Substitution	4.0E-242	121	2	WP_041169896.1
1,177,619	1,177,619	1	T -> C		71	SNP (transition)	100.0%	K -> E	358	AAG -> GAG	C770_RS47285	hypothetical protein	Substitution	5.0E-235	120	1	WP_041169896.1
1,177,654	1,177,654	1	G -> A		70	SNP (transition)	98.6%	A -> V	323	GCC -> GTC	C770_RS47285	hypothetical protein	Substitution	1.8E-233	108	2	WP_041169896.1
1,177,675	1,177,675	1	C -> T		66	SNP (transition)	100.0%	R -> K	302	AGG -> AAG	C770_RS47285	hypothetical protein	Substitution	4.0E-225	101	2	WP_041169896.1
1,177,710	1,177,710	1	A -> G		72	SNP (transition)	98.6%		267	AAT -> AAC	C770_RS47285	hypothetical protein	None	2.3E-247	89	3	WP_041169896.1
1,177,788	1,177,788	1	G -> C		69	SNP (transversion)	100.0%	I -> M	189	ATC -> ATG	C770_RS47285	hypothetical protein	Substitution	3.2E-242	63	3	WP_041169896.1
1,177,840	1,177,840	1	G -> T		68	SNP (transversion)	100.0%	A -> E	137	GCG -> GAG	C770_RS47285	hypothetical protein	Substitution	2.5E-218	46	2	WP_041169896.1
1,177,854	1,177,854	1	C -> A		68	SNP (transversion)	95.6%		123	CTG -> CTT	C770_RS47285	hypothetical protein	None	1.6E-210	41	3	WP_041169896.1
1,177,862	1,177,862	1	A -> G		67	SNP (transition)	97.0%		115	TTG -> CTG	C770_RS47285	hypothetical protein	None	7.0E-212	39	1	WP_041169896.1
1,177,872	1,177,872	1	C -> A		74	SNP (transversion)	100.0%	M -> I	105	ATG -> ATT	C770_RS47285	hypothetical protein	Substitution	2.5E-252	35	3	WP_041169896.1
1,177,879	1,177,879	1	G -> A		76	SNP (transition)	97.4%	T -> M	98	ACG -> ATG	C770_RS47285	hypothetical protein	Substitution	7.2E-249	33	2	WP_041169896.1
1,177,894	1,177,894	1	T -> C		78	SNP (transition)	98.7%	K -> R	83	AAA -> AGA	C770_RS47285	hypothetical protein	Substitution	1.2E-260	28	2	WP_041169896.1
1,177,900	1,177,900	1	G -> A		79	SNP (transition)	100.0%	P -> L	77	CCC -> CTC	C770_RS47285	hypothetical protein	Substitution	3.2E-277	26	2	WP_041169896.1
1,177,924	1,177,924	1	A -> G		81	SNP (transition)	100.0%	L -> P	53	CTC -> CCC	C770_RS47285	hypothetical protein	Substitution	3.2E-284	18	2	WP_041169896.1
1,177,929	1,177,929	1	G -> A		80	SNP (transition)	100.0%		48	CAC -> CAT	C770_RS47285	hypothetical protein	None	1.0E-272	16	3	WP_041169896.1
1,177,996	1,177,996	1	T -> G		82	SNP (transversion)	100.0%	F -> V	34	TTT -> GTT	C770_RS47290	cAMP-binding protein	Substitution	1.6E-279	12	1	WP_041169930.1
1,178,002	1,178,002	1	C -> A		82	SNP (transversion)	100.0%		40	CGG -> AGG	C770_RS47290	cAMP-binding protein	None	6.3E-296	14	1	WP_041169930.1
1,178,006	1,178,006	1	C -> G		81	SNP (transversion)	98.8%	P -> R	44	CCG -> CGG	C770_RS47290	cAMP-binding protein	Substitution	8.1E-263	15	2	WP_041169930.1
1,178,073	1,178,073	1	A -> G		80	SNP (transition)	100.0%		111	CGA -> CGG	C770_RS47290	cAMP-binding protein	None	1.0E-272	37	3	WP_041169930.1
1,178,199	1,178,199	1	C -> A		83	SNP (transversion)	97.6%	H -> Q	237	CAC -> CAA	C770_RS47290	cAMP-binding protein	Substitution	1.7E-264	79	3	WP_041169930.1
1,178,242	1,178,242	1	C -> T		87	SNP (transition)	100.0%		280	CTG -> TTG	C770_RS47290	cAMP-binding protein	None	4.0E-279	94	1	WP_041169930.1
1,178,284	1,178,284	1	C		90	Deletion	100.0%		322		C770_RS47290	cAMP-binding protein	Frame Shift	1.0E-135	108	1	WP_041169930.1
1,178,310	1,178,310	1	T -> C		94	SNP (transition)	100.0%		348	CGT -> CGC	C770_RS47290	cAMP-binding protein	None	2.5E-320	116	3	WP_041169930.1
1,178,319	1,178,319	1	G -> A		98	SNP (transition)	99.0%		357	TCG -> TCA	C770_RS47290	cAMP-binding protein	None	3.9E-309	119	3	WP_041169930.1
1,178,334	1,178,334	1	T -> C		95	SNP (transition)	100.0%		372	CAT -> CAC	C770_RS47290	cAMP-binding protein	None	3.2E-314	124	3	WP_041169930.1
1,178,352	1,178,352	1	A -> G		96	SNP (transition)	99.0%		390	GAA -> GAG	C770_RS47290	cAMP-binding protein	None	9.6E-322	130	3	WP_041169930.1
1,178,373	1,178,373	1	C -> T		95	SNP (transition)	97.9%		411	GTC -> GTT	C770_RS47290	cAMP-binding protein	None	2.8E-313	137	3	WP_041169930.1
1,178,390	1,178,390	1	G -> C		97	SNP (transversion)	99.0%	G -> A	428	GGC -> GCC	C770_RS47290	cAMP-binding protein	Substitution	1.5E-315	143	2	WP_041169930.1
1,178,422	1,178,424	2	CCA -> GCG	90 -> 91		Substitution	100.0%	P -> A	460	CCA -> GCG	C770_RS47290	cAMP-binding protein	Substitution	4.0E-310	154	1	WP_041169930.1
1,178,448	1,178,448	1	C -> G		93	SNP (transversion)	98.9%		486	GCC -> GCG	C770_RS47290	cAMP-binding protein	None	9.3E-321	162	3	WP_041169930.1
1,178,504	1,178,504	1	A -> G		88	SNP (transition)	100.0%	D -> G	542	GAC -> GGC	C770_RS47290	cAMP-binding protein	Substitution	1.6E-273	181	2	WP_041169930.1
1,178,517	1,178,517	1	A -> G		86	SNP (transition)	100.0%							2.5E-267			
1,178,530	1,178,530	1	G -> A		85	SNP (transition)	100.0%							1.0E-255			
1,178,542	1,178,542	1	C -> A		85	SNP (transversion)	100.0%							1.0E-272			
1,178,550	1,178,551	2	GA -> CG		84	Substitution	97.6%							8.7E-268			
1,178,560	1,178,560	1	C -> A		88	SNP (transversion)	95.5%							1.5E-271			
1,178,568	1,178,568	1	A -> G		85	SNP (transition)	98.8%							2.1E-284			
1,178,584	1,178,584	1	A -> T		86	SNP (transversion)	97.7%							9.2E-283			
1,178,589	1,178,589	1	T -> G		88	SNP (transversion)	100.0%							1.0E-308			
1,178,620	1,178,620	1	T -> C		81	SNP (transition)	97.5%							5.1E-250			
1,178,644	1,178,645	2	CC -> TT	88 -> 89		Substitution	98.9%							2.2E-280			
1,178,647	1,178,646	0	G		89	Insertion	100.0%							2.0E-294			
1,178,652	1,178,652	1	G -> T		91	SNP (transversion)	98.9%							9.1E-296			
1,178,659	1,178,659	1	C -> A		92	SNP (transversion)	98.9%							5.8E-290			
1,178,669	1,178,669	1	G -> A		93	SNP (transition)	98.9%							2.3E-302			
1,178,707	1,178,707	1	C -> T		97	SNP (transition)	99.0%							1.5E-315			
1,178,740	1,178,740	1	A -> C		96	SNP (transversion)	100.0%							1.6E-317			
1,178,744	1,178,744	1	(G)3 -> (G)2		94	Deletion (tandem repeat)	100.0%							4.0E-151			
1,178,750	1,178,750	1	C -> T		98	SNP (transition)	100.0%							4.9E-324			
1,178,753	1,178,753	1	G -> A		99	SNP (transition)	100.0%							0.0			
1,178,766	1,178,766	1	T -> C		98	SNP (transition)	100.0%							0.0			
1,178,769	1,178,769	1	C -> T		98	SNP (transition)	100.0%							0.0			
1,178,772	1,178,772	1	A -> G		100	SNP (transition)	100.0%							0.0			
1,178,780	1,178,780	1	A -> G		97	SNP (transition)	99.0%							6.1E-306			
1,178,789	1,178,790	2	CG -> AAC	100 -> 101		Insertion	99.0%							0.0			
1,178,814	1,178,814	1	T -> A		103	SNP (transversion)	98.1%							3.3E-320			
1,178,881	1,178,881	1	T -> C		94	SNP (transition)	100.0%	C -> R	28	TGC -> CGC	C770_RS47295	hypothetical protein	Substitution	6.3E-311	10	1	WP_015243272.1
1,178,946	1,178,946	1	C -> T		84	SNP (transition)	98.8%		93	GTC -> GTT	C770_RS47295	hypothetical protein	None	8.4E-248	31	3	WP_015243272.1
1,178,952	1,178,952	1	T -> C		83	SNP (transition)	98.8%		99	GAT -> GAC	C770_RS47295	hypothetical protein	None	1.3E-236	33	3	WP_015243272.1
1,178,973	1,178,973	1	C -> T		82	SNP (transition)	97.6%		120	TAC -> TAT	C770_RS47295	hypothetical protein	None	3.3E-237	40	3	WP_015243272.1
1,178,979	1,178,979	1	T -> C		83	SNP (transition)	100.0%		126	GCT -> GCC	C770_RS47295	hypothetical protein	None	2.0E-241	42	3	WP_015243272.1
1,178,983	1,178,983	1	G -> A		85	SNP (transition)	96.5%	A -> T	130	GCC -> ACC	C770_RS47295	hypothetical protein	Substitution	1.6E-233	44	1	WP_015243272.1
1,179,012	1,179,012	1	T -> C		87	SNP (transition)	97.7%		159	GAT -> GAC	C770_RS47295	hypothetical protein	None	1.2E-243	53	3	WP_015243272.1
1,179,017	1,179,017	1	T -> C		87	SNP (transition)	96.6%	V -> A	164	GTA -> GCA	C770_RS47295	hypothetical protein	Substitution	6.7E-231	55	2	WP_015243272.1
1,179,043	1,179,043	1	A -> G		78	SNP (transition)	100.0%	R -> G	190	AGG -> GGG	C770_RS47295	hypothetical protein	Substitution	6.3E-227	64	1	WP_015243272.1
1,179,092	1,179,092	1	G -> A		69	SNP (transition)	100.0%							3.2E-242			
1,179,107	1,179,107	1	T -> G		67	SNP (transversion)	100.0%							6.3E-242			
1,179,157	1,179,157	1	C -> G		69	SNP (transversion)	100.0%							2.5E-235			
1,179,167	1,179,166	0	G		72	Insertion	100.0%							1.6E-245			
1,179,176	1,179,176	1	G -> A		73	SNP (transition)	98.6%							1.8E-236			
1,179,198	1,179,198	1	A -> G		76	SNP (transition)	98.7%	I -> M	15	ATA -> ATG	C770_RS47300	hypothetical protein	Substitution	7.6E-254	5	3	WP_015243273.1
1,179,299	1,179,299	1	C -> T		64	SNP (transition)	96.9%	A -> V	116	GCA -> GTA	C770_RS47300	hypothetical protein	Substitution	3.2E-208	39	2	WP_015243273.1
1,179,369	1,179,369	1	A -> T		75	SNP (transversion)	97.3%		186	CCA -> CCT	C770_RS47300	hypothetical protein	None	7.0E-231	62	3	WP_015243273.1
1,179,393	1,179,393	1	A -> G		76	SNP (transition)	96.1%		210	GGA -> GGG	C770_RS47300	hypothetical protein	None	3.5E-222	70	3	WP_015243273.1
1,179,438	1,179,438	1	C -> T		74	SNP (transition)	95.9%		255	AGC -> AGT	C770_RS47300	hypothetical protein	None	4.1E-223	85	3	WP_015243273.1
1,179,444	1,179,444	1	G -> A		75	SNP (transition)	96.0%		261	GCG -> GCA	C770_RS47300	hypothetical protein	None	6.7E-248	87	3	WP_015243273.1
1,179,447	1,179,447	1	T -> C		74	SNP (transition)	95.9%		264	CTT -> CTC	C770_RS47300	hypothetical protein	None	3.2E-230	88	3	WP_015243273.1
1,179,504	1,179,504	1	A -> T		65	SNP (transversion)	96.9%		321	GGA -> GGT	C770_RS47300	hypothetical protein	None	1.3E-211	107	3	WP_015243273.1
1,179,515	1,179,515	1	T -> C		68	SNP (transition)	98.5%	I -> T	332	ATC -> ACC	C770_RS47300	hypothetical protein	Substitution	2.7E-213	111	2	WP_015243273.1
1,179,561	1,179,561	1	A -> G		73	SNP (transition)	100.0%		378	CCA -> CCG	C770_RS47300	hypothetical protein	None	1.3E-241	126	3	WP_015243273.1
1,179,576	1,179,578	3	TCA -> C		75	Deletion	100.0%							3.2E-113			
1,179,586	1,179,587	2	GC		75	Deletion	100.0%							1.0E-105			
1,179,591	1,179,591	1	C -> T		75	SNP (transition)	98.7%							1.2E-235			
1,179,607	1,179,607	1	G -> C		76	SNP (transversion)	100.0%							6.3E-244			
1,179,627	1,179,627	1	G -> C		77	SNP (transversion)	97.4%							9.2E-245			
1,179,635	1,179,635	1	A -> G		78	SNP (transition)	100.0%							2.5E-250			
1,179,652	1,179,652	1	A -> C		77	SNP (transversion)	100.0%							4.0E-247			
1,179,654	1,179,654	1	T -> C		76	SNP (transition)	98.7%							7.6E-254			
1,179,657	1,179,658	2	GA -> AG		76	Substitution	98.7%							7.6E-239			
1,179,674	1,179,674	1	A -> C		76	SNP (transversion)	98.7%	N -> K	126	AAT -> AAG	C770_RS47305	membrane protein	Substitution	7.6E-254	42	3	WP_014527390.1
1,179,743	1,179,743	1	C -> T		84	SNP (transition)	97.6%		57	TCG -> TCA	C770_RS47305	membrane protein	None	3.5E-284	19	3	WP_014527390.1
1,179,816	1,179,817	2	AC		85	Deletion	100.0%							1.0E-136			
1,179,852	1,179,852	1	C -> A		86	SNP (transversion)	98.8%							2.7E-279			
1,179,942	1,179,942	1	A -> G		80	SNP (transition)	100.0%							1.0E-264			
1,179,979	1,179,979	1	G -> A		85	SNP (transition)	96.5%							2.5E-266			
1,180,029	1,180,029	1	C -> T		82	SNP (transition)	98.8%							4.1E-266			
1,180,033	1,180,033	1	T -> G		81	SNP (transversion)	98.8%							8.1E-255			
1,180,047	1,180,048	2	CC -> AA		80	Substitution	100.0%							1.0E-256			
1,180,053	1,180,053	1	C -> T		81	SNP (transition)	98.8%							8.1E-247			
1,180,072	1,180,072	1	G -> A		79	SNP (transition)	100.0%							2.5E-269			
1,180,084	1,180,084	1	G -> C		78	SNP (transversion)	100.0%							6.3E-266			
1,180,127	1,180,127	1	G -> C		73	SNP (transversion)	98.6%							1.2E-243			
1,180,174	1,180,174	1	T -> C		76	SNP (transition)	98.7%							7.6E-239			
1,180,259	1,180,259	1	C -> G		72	SNP (transversion)	98.6%							3.6E-233			
1,180,434	1,180,434	1	T -> C		80	SNP (transition)	97.5%							7.9E-247			
1,180,561	1,180,561	1	T -> C		75	SNP (transition)	98.7%							1.9E-250			
1,180,583	1,180,583	1	A -> G		75	SNP (transition)	98.7%							1.9E-250			
1,180,715	1,180,715	1	C -> A		73	SNP (transversion)	98.6%		582	CTG -> CTT	C770_RS47315	hypothetical protein	None	1.2E-243	194	3	WP_015243274.1
1,181,074	1,181,074	1	C -> T		88	SNP (transition)	98.9%	V -> I	223	GTC -> ATC	C770_RS47315	hypothetical protein	Substitution	1.4E-294	75	1	WP_015243274.1
1,181,259	1,181,259	1	G -> A		82	SNP (transition)	97.6%	T -> I	38	ACT -> ATT	C770_RS47315	hypothetical protein	Substitution	3.3E-245	13	2	WP_015243274.1
1,181,545	1,181,545	1	C -> T		101	SNP (transition)	99.0%							0.0			
1,181,924	1,181,924	1	C -> T		85	SNP (transition)	98.8%	H -> Y	172	CAC -> TAC	C770_RS47320	hypothetical protein	Substitution	1.3E-267	58	1	WP_010975414.1
1,182,309	1,182,309	1	T -> C		76	SNP (transition)	100.0%	V -> A	332	GTA -> GCA	C770_RS47325	hypothetical protein	Substitution	6.3E-244	111	2	WP_015243275.1
1,182,492	1,182,492	1	C -> T		108	SNP (transition)	98.1%		9	ACC -> ACT	C770_RS47330	hypothetical protein	None	0.0	3	3	WP_015243276.1
1,182,496	1,182,496	1	C -> G		109	SNP (transversion)	98.2%	P -> A	13	CCC -> GCC	C770_RS47330	hypothetical protein	Substitution	0.0	5	1	WP_015243276.1
1,182,626	1,182,626	1	C -> G		117	SNP (transversion)	98.3%	P -> R	143	CCG -> CGG	C770_RS47330	hypothetical protein	Substitution	0.0	48	2	WP_015243276.1
1,182,692	1,182,692	1	T -> G		113	SNP (transversion)	97.3%							0.0			
1,182,720	1,182,725	6	TTATAG		110	Deletion	98.2%							9.0E-170			

1,182,758	1,182,758	1	A -> C	104	SNP (transversion)	98.1%							0.0			
1,182,794	1,182,794	1	G -> A	101	SNP (transition)	99.0%							0.0			
1,182,815	1,182,815	1	T	95	Deletion	98.9%							9.2E-140			
1,182,835	1,182,835	1	A -> C	94	SNP (transversion)	98.9%							5.9E-315			
1,182,863	1,182,863	1	G -> C	94	SNP (transversion)	98.9%		177	GGC -> GGG	C770_RS47335	hypothetical protein	None	2.4E-296	59	3	WP_010975412.1
1,182,881	1,182,881	1	(C)3 -> (C)2	98	Deletion (tandem repeat)	99.0%		159		C770_RS47335	hypothetical protein	Frame Shift	6.0E-154	53	3	WP_010975412.1
1,182,896	1,182,896	1	C -> A	96	SNP (transversion)	99.0%	E -> D	144	GAG -> GAT	C770_RS47335	hypothetical protein	Substitution	9.6E-322	48	3	WP_010975412.1
1,183,186	1,183,186	1	C -> G	75	SNP (transversion)	100.0%		606	CTG -> CTC	C770_RS47340	phospholipase	None	1.0E-255	202	3	WP_010975411.1
1,183,354	1,183,354	1	T -> C	90	SNP (transition)	97.8%		438	GAA -> GAG	C770_RS47340	phospholipase	None	2.5E-296	146	3	WP_010975411.1
1,183,621	1,183,621	1	G -> A	85	SNP (transition)	100.0%		171	CCC -> CCT	C770_RS47340	phospholipase	None	1.0E-289	57	3	WP_010975411.1
1,183,628	1,183,628	1	C -> A	87	SNP (transversion)	100.0%	S -> I	164	AGT -> ATT	C770_RS47340	phospholipase	Substitution	7.9E-288	55	2	WP_010975411.1
1,183,693	1,183,693	1	C -> G	80	SNP (transversion)	100.0%		99	GTG -> GTC	C770_RS47340	phospholipase	None	1.0E-272	33	3	WP_010975411.1
1,183,777	1,183,777	1	A -> T	61	SNP (transversion)	100.0%		15	ATT -> ATA	C770_RS47340	phospholipase	None	3.2E-214	5	3	WP_010975411.1
1,183,782	1,183,782	1	C -> T	61	SNP (transition)	100.0%	G -> R	10	GGA -> AGA	C770_RS47340	phospholipase	Substitution	4.0E-208	4	1	WP_010975411.1
1,183,793	1,183,793	1	A -> C	60	SNP (transversion)	100.0%							1.0E-192			
1,183,922	1,183,922	1	C -> G	27	SNP (transversion)	96.3%							6.8E-80			
1,183,933	1,183,933	1	(T)3 -> (T)2	21	Deletion (tandem repeat)	100.0%							3.2E-32			
1,183,939	1,183,939	1	G -> C	20	SNP (transversion)	100.0%							1.0E-64			
1,183,944	1,183,946	3	TAA -> CGG	18 -> 20	Substitution	100.0%							6.3E-53			
1,183,949	1,183,949	1	G -> C	18	SNP (transversion)	100.0%							6.3E-53			
1,183,951	1,183,951	1	C -> G	18	SNP (transversion)	100.0%							4.0E-51			
1,183,953	1,183,954	2	TC -> GA	17 -> 18	Substitution	100.0%							5.0E-50			
1,183,956	1,183,961	6	CCGCCC -> ATCT/	13 -> 15	Substitution	100.0%							2.5E-42			
1,183,963	1,183,968	6	ATTGGA -> CGGT	11 -> 12	Substitution	100.0%							7.9E-35			
1,183,970	1,183,971	2	AG -> TC	11	Substitution	100.0%							7.9E-35			
1,183,973	1,183,974	2	CT -> TG	11	Substitution	100.0%							6.3E-36			
1,183,977	1,183,978	2	AT -> TA	9 -> 10	Substitution	100.0%							1.3E-28			
1,183,980	1,183,981	2	TT -> GA	9	Substitution	100.0%							2.0E-30			
1,183,983	1,183,982	0	CC	8	Insertion	100.0%							1.6E-25			
1,183,987	1,183,987	1	T	7	Deletion	100.0%							1.6E-10			
1,183,992	1,183,994	3	GGG -> TAC	7	Substitution	100.0%							3.2E-18			
1,183,996	1,183,996	1	G -> C	6	SNP (transversion)	100.0%							1.0E-18			
1,184,829	1,184,829	1	A -> G	69	SNP (transition)	98.6%							6.9E-237			
1,184,832	1,184,832	1	T	68	Deletion	100.0%							1.6E-109			
1,184,838	1,184,838	1	G -> C	69	SNP (transversion)	98.6%							4.4E-230			
1,184,855	1,184,855	1	G -> A	71	SNP (transition)	100.0%							3.2E-249			
1,184,886	1,184,886	1	A -> G	90	SNP (transition)	100.0%							1.0E-288			
1,186,619	1,186,619	1	T -> A	21	SNP (transversion)	100.0%							5.0E-28			
1,186,624	1,186,624	1	G -> A	18	SNP (transition)	100.0%							1.6E-20			
1,186,671	1,186,671	1	G -> T	10	SNP (transversion)	100.0%							1.0E-28			
1,186,675	1,186,675	1	T -> C	14	SNP (transition)	100.0%							4.0E-37			
1,186,686	1,186,686	1	T -> G	18	SNP (transversion)	100.0%							2.5E-49			
1,186,727	1,186,727	1	A -> C	24	SNP (transversion)	95.8%							1.2E-70			
1,186,736	1,186,736	1	T -> G	26	SNP (transversion)	96.2%							2.6E-79			
1,186,738	1,186,738	1	A -> G	27	SNP (transition)	100.0%							5.0E-79			
1,186,744	1,186,744	1	T -> C	27	SNP (transition)	96.3%							4.3E-72			
1,186,750	1,186,750	1	A -> C	30	SNP (transversion)	96.7%							3.8E-89			
1,186,759	1,186,759	1	G -> A	30	SNP (transition)	100.0%							1.0E-102			
1,186,966	1,186,966	1	G -> C	70	SNP (transversion)	100.0%							1.0E-238			
1,186,983	1,186,983	1	T -> C	69	SNP (transition)	100.0%							3.2E-242			
1,187,001	1,187,001	1	G -> C	64	SNP (transversion)	100.0%							6.3E-212			
1,187,044	1,187,044	1	G -> AC	58	Insertion	100.0%							6.3E-198			
1,187,053	1,187,052	0	T	55	Insertion	98.2%							1.4E-182			
1,187,066	1,187,066	1	T -> A	51	SNP (transversion)	96.1%							2.0E-154			
1,187,069	1,187,069	1	C -> T	48	SNP (transition)	100.0%							4.0E-159			
1,187,072	1,187,073	2	AG -> CA	48	Substitution	100.0%							1.6E-149			
1,187,085	1,187,085	1	G -> A	47	SNP (transition)	97.9%							7.4E-151			
1,187,088	1,187,088	1	T -> C	47	SNP (transition)	97.9%							3.0E-146			
1,187,101	1,187,102	2	TG -> CA	39 -> 40	Substitution	97.4% -> 97.5%							6.3E-124			
1,187,115	1,187,114	0	(A)2 -> (A)3	34	Insertion (tandem repeat)	100.0%							6.3E-113			
1,187,122	1,187,122	1	T -> C	33	SNP (transition)	100.0%							2.5E-106			
1,187,144	1,187,144	1	T -> A	29	SNP (transversion)	100.0%							2.0E-96			
1,187,146	1,187,145	0	C	29	Insertion	96.6%							1.8E-94			
1,187,149	1,187,149	1	C -> T	29	SNP (transition)	96.6%							4.6E-86			
1,187,162	1,187,162	1	C -> G	26	SNP (transversion)	100.0%							6.3E-84			
1,187,171	1,187,171	1	A -> G	24	SNP (transition)	100.0%							2.5E-70			
1,187,178	1,187,178	1	C -> T	23	SNP (transition)	95.7%							5.8E-61			
1,187,212	1,187,212	1	A -> G	16	SNP (transition)	100.0%							1.6E-45			
1,187,285	1,187,287	3	ACG -> CTT	10 -> 11	Substitution	100.0%							1.0E-28			
1,187,289	1,187,289	1	A -> C	11	SNP (transversion)	100.0%							5.0E-37			
1,187,313	1,187,313	1	T -> C	14	SNP (transition)	100.0%							1.0E-49			
1,187,449	1,187,449	1	T -> C	60	SNP (transition)	100.0%		51	GAT -> GAC	C770_RS47360	DUF982 domain-contair	None	1.0E-198	17	3	WP_015243280.1
1,187,456	1,187,456	1	C -> A	65	SNP (transversion)	100.0%		58	CGA -> AGA	C770_RS47360	DUF982 domain-contair	None	3.2E-228	20	1	WP_015243280.1
1,187,499	1,187,500	2	GG -> AA	76	Substitution	100.0%	G -> E	101	GGG -> GAA	C770_RS47360	DUF982 domain-contair	Substitution	4.0E-259	34	2	WP_015243280.1
1,187,512	1,187,512	1	C -> A	78	SNP (transversion)	100.0%		114	CGC -> CGA	C770_RS47360	DUF982 domain-contair	None	1.0E-273	38	3	WP_015243280.1
1,187,540	1,187,540	1	C -> T	87	SNP (transition)	100.0%		142	CTG -> TTG	C770_RS47360	DUF982 domain-contair	None	1.6E-296	48	1	WP_015243280.1
1,187,548	1,187,548	1	C -> T	88	SNP (transition)	98.9%		150	ACC -> ACT	C770_RS47360	DUF982 domain-contair	None	1.4E-294	50	3	WP_015243280.1
1,187,587	1,187,587	1	T -> G	89	SNP (transversion)	100.0%		189	GTT -> GTG	C770_RS47360	DUF982 domain-contair	None	1.6E-285	63	3	WP_015243280.1
1,187,601	1,187,601	1	T -> G	98	SNP (transversion)	100.0%	I -> S	203	ATC -> AGC	C770_RS47360	DUF982 domain-contair	Substitution	4.9E-324	68	2	WP_015243280.1
1,187,605	1,187,605	1	A -> G	100	SNP (transition)	100.0%		207	CGA -> CGG	C770_RS47360	DUF982 domain-contair	None	1.0E-320	69	3	WP_015243280.1
1,187,608	1,187,608	1	C -> T	101	SNP (transition)	100.0%		210	GCC -> GCT	C770_RS47360	DUF982 domain-contair	None	1.0E-303	70	3	WP_015243280.1
1,187,619	1,187,619	1	A -> G	103	SNP (transition)	100.0%	K -> R	221	AAG -> AGG	C770_RS47360	DUF982 domain-contair	Substitution	0.0	74	2	WP_015243280.1
1,187,632	1,187,632	1	G -> T	102	SNP (transversion)	99.0%		234	CTG -> CTT	C770_RS47360	DUF982 domain-contair	None	0.0	78	3	WP_015243280.1
1,187,652	1,187,652	1	T -> G	100	SNP (transversion)	100.0%	L -> R	254	CTC -> CGC	C770_RS47360	DUF982 domain-contair	Substitution	0.0	85	2	WP_015243280.1
1,187,655	1,187,655	1	C -> T	101	SNP (transition)	97.0%	P -> L	257	CCG -> CTG	C770_RS47360	DUF982 domain-contair	Substitution	4.2E-309	86	2	WP_015243280.1
1,187,686	1,187,686	1	A -> G	109	SNP (transition)	98.2%		288	GGA -> GGG	C770_RS47360	DUF982 domain-contair	None	0.0	96	3	WP_015243280.1
1,187,702	1,187,703	2	GC -> CG	107 -> 108	Substitution	99.1%							0.0			
1,187,711	1,187,711	1	C -> T	107	SNP (transition)	98.1%							1.8E-322			
1,187,716	1,187,716	1	G -> A	107	SNP (transition)	98.1%							0.0			
1,187,729	1,187,729	1	A -> C	107	SNP (transversion)	99.1%							0.0			
1,187,736	1,187,737	2	CC -> TT	103	Substitution	98.1%							0.0			
1,187,753	1,187,753	1	C -> A	97	SNP (transversion)	99.0%							0.0			
1,187,757	1,187,757	1	G -> A	97	SNP (transition)	99.0%							0.0			
1,187,761	1,187,761	1	A -> T	97	SNP (transversion)	99.0%							0.0			
1,187,763	1,187,763	1	G -> C	98	SNP (transversion)	99.0%							0.0			
1,187,768	1,187,768	1	G -> A	97	SNP (transition)	97.9%							0.0			
1,187,771	1,187,771	1	G -> A	96	SNP (transition)	99.0%							3.0E-312			
1,187,776	1,187,776	1	C -> T	97	SNP (transition)	100.0%							7.9E-321			
1,187,789	1,187,789	1	G -> A	97	SNP (transition)	97.9%							4.7E-320			
1,187,796	1,187,796	1	T -> C	96	SNP (transition)	99.0%							9.6E-322			
1,187,799	1,187,799	1	T -> G	96	SNP (transversion)	100.0%							1.6E-317			
1,187,802	1,187,802	1	T -> C	96	SNP (transition)	100.0%							1.6E-317			
1,187,806	1,187,806	1	G -> T	96	SNP (transversion)	97.9%							1.1E-316			
1,187,815	1,187,815	1	G -> A	98	SNP (transition)	99.0%							0.0			
1,187,835	1,187,835	1	G -> C	89	SNP (transversion)	98.9%							5.6E-298			
1,187,849	1,187,849	1	A -> G	86	SNP (transition)	100.0%							1.0E-301			
1,187,851	1,187,851	1	T -> C	86	SNP (transition)	100.0%							2.5E-310			
1,187,861	1,187,861	1	G -> A	86	SNP (transition)	98.8%							8.6E-271			
1,187,866	1,187,866	1	A -> G	86	SNP (transition)	98.8%							2.7E-279			
1,187,871	1,187,871	1	(T)4 -> (T)3	85	Deletion (tandem repeat)	100.0%							1.0E-136			
1,187,875	1,187,875	1	A -> G	83	SNP (transition)	98.8%	M -> V	1	ATG -> GTG	C770_RS47365	hypothetical protein	Substitution	5.2E-294	1	1	WP_010975409.1
1,187,882	1,187,882	1	C -> T	78	SNP (transition)	100.0%	T -> I	8	ACC -> ATC	C770_RS47365	hypothetical protein	Substitution	1.0E-273	3	2	WP_010975409.1
1,187,885	1,187,885	1	T -> C	78	SNP (transition)	100.0%	V -> A	11	GTT -> GCT	C770_RS47365	hypothetical protein	Substitution	1.0E-273	4	2	WP_010975409.1
1,187,893	1,187,893	1	G -> A	75	SNP (transition)	100.0%	E -> K	19	GAG -> AAG	C770_RS47365	hypothetical protein	Substitution	1.0E-240	7	1	WP_010975409.1
1,187,905	1,187,907	2	CTC -> ATT	63 -> 64	Substitution	100.0%	L -> I	31	CTC -> ATT	C770_RS47365	hypothetical protein	Substitution	3.2E-221	11	1	WP_010975409.1
1,187,922	1,187,922	1	G -> C	57	SNP (transversion)	100.0%		48	CTG -> CTC	C770_RS47365	hypothetical protein	None	6.3E-206	16	3	WP_010975409.1
1,187,925	1,187,925	1	T -> C	55	SNP (transition)	100.0%		51	GAT -> GAC	C770_RS47365	hypothetical protein	None	3.2E-193	17	3	WP_010975409.1
1,187,946	1,187,946	1	T -> C	50	SNP (transition)	98.0%		72	CAT -> CAC	C770_RS47365	hypothetical protein	None	1.0E-160	24	3	WP_010975409.1
1,187,952	1,187,952	1	C -> T	49	SNP (transition)	100.0%		78	GCC -> GCT	C770_RS47365	hypothetical protein	None	2.0E-162	26	3	WP_010975409.1
1,187,963	1,187,963	1	G -> A	43	SNP (transition)	97.7%	R -> H	89	CGC -> CAC	C770_RS47365	hypothetical protein	Substitution	6.8E-142	30	2	WP_010975409.1
1,187,967	1,187,967	1	G -> A	42	SNP (transition)	97.6%		93	TTG -> TTA	C770_RS47365	hypothetical protein	None	1.7E-138	31	3	WP_010975409.1
1,187,985	1,187,985	1	C -> G	34	SNP (transversion)	100.0%		111	CCC -> CCG	C770_RS47365	hypothetical protein	None	1.6E-109	37	3	WP_010975409.1
1,188,010	1,188,010	1	A -> G	26	SNP (transition)	96.2%	T -> A	136	ACG -> GCG	C770_RS47365	hypothetical protein	Substitution	8.2E-87	46	1	WP_010975409.1
1,188,015	1,188,015	1	C -> G	24	SNP (transversion)	100.0%		141	GCC -> GCG	C770_RS47365	hypothetical protein	None	6.3E-80	47	3	WP_010975409.1
1,188,037	1,188,037	1	A -> G	17	SNP (transition)	100.0%	M -> V	163	ATG -> GTG	C770_RS47365	hypothetical protein	Substitution	3.2E-60	55	1	WP_010975409.1
1,188,045	1,188,045	1	C -> G	15	SNP (transversion)	100.0%		171	GGC -> GGG	C770_RS47365	hypothetical protein	None	3.2E-53	57	3	WP_010975409.1
1,188,047	1,188,047	1	T -> C	15	SNP (transition)	100.0%	I -> T	173	ATA -> ACA	C770_RS47365	hypothetical protein	Substitution	3.2E-53	58	2	WP_010975409.1
1,188,049	1,188,049	1	T -> C	15	SNP (transition)	100.0%	C -> R	175	TGT -> CGT	C770_RS47365	hypothetical protein	Substitution	1.0E-54	59	1	WP_010975409.1
1,188,057	1,188,057	1	G -> T	15	SNP (transversion)	100.0%		183	GGG -> GGT	C770_RS47365	hypothetical protein	None	1.0E-54	61	3	WP_010975409.1
1,188,062	1,188,062	1	G -> A	12	SNP (transition)	100.0%	R -> H	188	CGT -> CAT	C770_RS47365	hypothetical protein	Substitution	1.0E-42	63	2	WP_010975409.1
1,188,259	1,188,259	1	A -> G	6	SNP (transition)	100.0%							4.0E-21			
1,188,270	1,188,270	1	A -> G	9	SNP (transition)	100.0%							2.0E-30			
1,188,281	1,188,282	2	GC -> AA	10	Substitution	100.0%							1.0E-35			
1,188,285	1,188,285	1	A -> G	10	SNP (transition)	100.0%							1.0E-34			
1,188,291	1,188,291	1	G -> C	11	SNP (transversion)	100.0%							3.2E-39			
1,188,309	1,188,309	1	G -> C	16	SNP (transversion)	100.0%							1.0E-48			
1,188,312	1,188,312	1	G -> C	17	SNP (transversion)	100.0%							1.6E-58			
1,188,318	1,188,318	1	A -> G	19	SNP (transition)	100.0%							2.5E-65			
1,188,321	1,188,321	1	G -> A	19	SNP (transition)	100.0%							2.0E-63			
1,188,323	1,188,324	2	AC -> GG	19	Substitution	100.0%							2.0E-63			
1,188,327	1,188,327	1	A -> G	19	SNP (transition)	100.0%							2.0E-63			
1,188,336	1,188,336	1	G -> A	20	SNP (transition)	100.0%							1.0E-68			
1,188,345	1,188,345	1	G -> T	21	SNP (transversion)	100.0%							2.5E-76			
1,188,348	1,188,348	1	A -> G	22	SNP (transition)	100.0%							1.0E-77			
1,188,357	1,188,357	1	G -> A	26	SNP (transition)	100.0%							1.6E-86			
1,188,359	1,188,359	1	T -> A	27	SNP (transversion)	100.0%							2.0E-84			
1,188,366	1,188,366	1	G -> C	30	SNP (transversion)	100.0%							1.0E-93			
1,188,369	1,188,369	1	G -> A	32	SNP (transition)	100.0%							6.3E-100			
1,188,372	1,188,372	1	C -> T	32	SNP (transition)	96.9%							2.5E-95			
1,188,375	1,188,375	1	G -> T	32	SNP (transversion)	100.0%							6.3E-100			
1,188,383	1,188,384	2	CA -> GC	32	Substitution	100.0%							4.0E-103			
1,188,437	1,188,437	1	G -> A	43	SNP (transition)	100.0%							1.3E-142			
1,188,449	1,188,449	1	T -> C	47	SNP (transition)	100.0%							3.2E-165			
1,188,455	1,188,455	1	C -> G	48	SNP (transversion)	100.0%							2.5E-154			
1,188,459	1,188,459	1	A -> T	48	SNP (transversion)	97.9%							3.8E-154			
1,188,476	1,188,476	1	G -> A	51	SNP (transition)	100.0%							3.2E-179			
1,188,486	1,188,486	1	C -> G	54	SNP (transversion)	96.3%							1.4E-179			
1,188,509	1,188,508	0	(T)2 -> (T)3	60	Insertion (tandem repeat)	100.0%							1.0E-198			
1,188,517	1,188,517	1	G -> A	64	SNP (transition)	98.4%							8.1E-207			
1,188,545	1,188,545	1	T -> C	68	SNP (transition)	100.0%							1.0E-238			

1,188,568	1,188,568	1	T -> GAC	64 -> 65		Insertion	100.0%							3.2E-215			
1,188,574	1,188,574	1	A -> G		64	SNP (transition)	100.0%							2.5E-218			
1,188,597	1,188,597	1	T -> C		67	SNP (transition)	100.0%							7.9E-222			
1,188,608	1,188,608	1	G -> T		67	SNP (transversion)	97.0%							7.0E-225			
1,188,616	1,188,616	1	G -> C		64	SNP (transversion)	100.0%							2.5E-218			
1,188,622	1,188,622	1	A -> C		67	SNP (transversion)	100.0%							1.6E-228			
1,188,635	1,188,635	1	T -> G		71	SNP (transversion)	100.0%							4.0E-242			
1,188,637	1,188,636	0	AGC	71 -> 72		Insertion	97.2% -> 98.6%							7.1E-244			
1,188,640	1,188,640	1	G -> A		71	SNP (transition)	100.0%							4.0E-242			
1,188,644	1,188,644	1	A -> G		69	SNP (transition)	98.6%							6.9E-237			
1,188,651	1,188,651	1	A -> G		69	SNP (transition)	98.6%							4.4E-230			
1,188,662	1,188,662	1	C -> G		67	SNP (transversion)	98.5%							1.1E-216			
1,188,664	1,188,664	1	C		65	Deletion	100.0%							3.2E-98			
1,188,673	1,188,675	3	CAA -> GGC		66	Substitution	98.5%							6.6E-207			
1,188,686	1,188,686	1	G -> T		68	SNP (transversion)	100.0%							2.5E-218			
1,188,716	1,188,716	1	G -> T		76	SNP (transversion)	100.0%							6.3E-244			
1,188,719	1,188,718	0	A		75	Insertion	100.0%							1.0E-255			
1,188,731	1,188,731	1	C -> T		74	SNP (transition)	100.0%							2.5E-252			
1,188,739	1,188,739	1	G -> C		75	SNP (transversion)	100.0%	A -> P	4	GCT -> CCT	C770_RS47375	hypothetical protein	Substitution	1.0E-240	2	1	WP_015243282.1
1,188,765	1,188,765	1	C -> T		80	SNP (transition)	98.8%		30	AAC -> AAT	C770_RS47375	hypothetical protein	None	1.6E-259	10	3	WP_015243282.1
1,188,856	1,188,856	1	A -> G		65	SNP (transition)	100.0%	N -> D	121	AAC -> GAC	C770_RS47375	hypothetical protein	Substitution	3.2E-215	41	1	WP_015243282.1
1,188,862	1,188,862	1	C -> G		64	SNP (transversion)	100.0%	P -> A	127	CCA -> GCA	C770_RS47375	hypothetical protein	Substitution	4.0E-199	43	1	WP_015243282.1
1,188,871	1,188,871	1	A -> G		64	SNP (transition)	100.0%	I -> V	136	ATC -> GTC	C770_RS47375	hypothetical protein	Substitution	6.3E-212	46	1	WP_015243282.1
1,188,902	1,188,902	1	A -> G		55	SNP (transition)	100.0%	Q -> R	167	CAG -> CGG	C770_RS47375	hypothetical protein	Substitution	1.0E-176	56	2	WP_015243282.1
1,188,935	1,188,935	1	(C)3 -> (C)2		46	Deletion (tandem repeat)	100.0%							4.0E-65			
1,188,940	1,188,940	1	A -> T		44	SNP (transversion)	100.0%							2.5E-150			
1,188,961	1,188,961	1	C -> T		41	SNP (transition)	100.0%							7.9E-128			
1,188,973	1,188,973	1	C -> T		39	SNP (transition)	97.4%							6.2E-117			
1,188,980	1,188,980	1	C -> T		36	SNP (transition)	100.0%							2.5E-112			
1,188,982	1,188,982	1	G -> T		35	SNP (transversion)	97.1%							5.5E-108			
1,188,993	1,188,993	1	G -> C		29	SNP (transversion)	96.6%							7.3E-89			
1,188,996	1,188,996	1	G -> A		29	SNP (transition)	100.0%							6.3E-82			
1,189,004	1,189,004	1	T -> A		25	SNP (transversion)	100.0%							1.0E-75			
1,189,024	1,189,024	1	G -> T		20	SNP (transversion)	100.0%							1.0E-60			
1,189,026	1,189,027	2	TT -> AG		20	Substitution	100.0%							1.0E-56			
1,189,032	1,189,032	1	A -> C		20	SNP (transversion)	100.0%							1.0E-52			
1,189,035	1,189,035	1	A -> C		18	SNP (transversion)	100.0%							1.0E-45			
1,189,038	1,189,038	1	C -> G		18	SNP (transversion)	100.0%							1.0E-54			
1,189,041	1,189,043	3	TTT -> GGC		15	Substitution	100.0%							1.0E-36			
1,189,045	1,189,047	3	TAA -> ACC	14 -> 15		Substitution	100.0%							3.2E-44			
1,189,050	1,189,050	1	G -> C		12	SNP (transversion)	100.0%							1.6E-35			
1,189,052	1,189,052	1	G -> C		11	SNP (transversion)	100.0%							1.6E-31			
1,189,054	1,189,054	1	C -> A		10	SNP (transversion)	100.0%							1.0E-29			
1,189,056	1,189,057	2	GA -> CT		10	Substitution	100.0%							1.0E-26			
1,189,059	1,189,059	1	C -> G		10	SNP (transversion)	100.0%							1.0E-29			
1,189,061	1,189,069	9	AGCTATGCG -> C	6 -> 10		Substitution	100.0%							1.0E-15			
1,189,845	1,189,846	2	CA -> GG		6	Substitution	100.0%							1.0E-15			
1,189,849	1,189,850	2	AG -> CC		7	Substitution	100.0%							3.2E-18			
1,189,855	1,189,854	0	T		7	Insertion	100.0%							4.0E-16			
1,189,866	1,189,866	1	G -> C		12	SNP (transversion)	100.0%							1.6E-29			
1,189,885	1,189,885	1	A -> T		19	SNP (transversion)	100.0%							7.9E-56			
1,189,903	1,189,903	1	T -> C		22	SNP (transition)	100.0%							2.5E-62			
1,189,946	1,189,946	1	C -> T		36	SNP (transition)	100.0%							4.0E-123			
1,190,004	1,190,004	1	A -> G		53	SNP (transition)	98.1%							3.3E-186			
1,190,023	1,190,023	1	A -> C		57	SNP (transversion)	100.0%							3.2E-200			
1,190,029	1,190,029	1	A -> T		58	SNP (transversion)	100.0%							4.0E-192			
1,190,048	1,190,048	1	T -> A		64	SNP (transversion)	100.0%							2.5E-218			
1,190,068	1,190,068	1	A -> G		68	SNP (transition)	100.0%							1.0E-238			
1,190,073	1,190,073	1	T -> G		70	SNP (transversion)	100.0%							1.0E-231			
1,190,081	1,190,081	1	A -> G		76	SNP (transition)	100.0%							4.0E-259			
1,190,093	1,190,093	1	C -> G		82	SNP (transversion)	100.0%							1.0E-287			
1,190,099	1,190,099	1	C -> A		85	SNP (transversion)	98.8%							2.1E-284			
1,190,120	1,190,120	1	C -> G		89	SNP (transversion)	100.0%							2.5E-303			
1,190,123	1,190,123	1	T -> C		89	SNP (transition)	98.9%							3.5E-289			
1,190,129	1,190,130	2	AC -> GT		94	Substitution	100.0%							2.5E-320			
1,190,157	1,190,157	1	C		99	Deletion	100.0%							3.2E-149			
1,190,181	1,190,181	1	T -> G		97	SNP (transversion)	100.0%							4.0E-311			
1,190,202	1,190,202	1	C -> T		96	SNP (transition)	100.0%							1.6E-317			
1,190,223	1,190,224	2	TC -> GT		98	Substitution	100.0%							4.9E-324			
1,190,239	1,190,239	1	G -> A		96	SNP (transition)	100.0%							1.6E-317			
1,190,257	1,190,257	1	C -> T		94	SNP (transition)	100.0%							0.0			
1,190,268	1,190,268	1	(G)3 -> (G)2		93	Deletion (tandem repeat)	100.0%							3.2E-140			
1,190,297	1,190,297	1	G -> C		104	SNP (transversion)	100.0%							0.0			
1,190,315	1,190,315	1	G -> C		105	SNP (transversion)	99.0%							0.0			
1,190,357	1,190,357	1	C -> G		106	SNP (transversion)	100.0%							0.0			
1,190,379	1,190,379	1	A -> G		107	SNP (transition)	99.1%							1.1E-316			
1,190,386	1,190,386	1	G -> T		110	SNP (transversion)	98.2%							0.0			
1,190,408	1,190,408	1	T -> C		103	SNP (transition)	100.0%							0.0			
1,190,450	1,190,451	2	GG -> AA	96 -> 97		Substitution	97.9%							6.1E-306			
1,190,460	1,190,460	1	G -> C		95	SNP (transversion)	100.0%							1.0E-323			
1,190,463	1,190,464	2	AC -> TG	95 -> 96		Substitution	100.0%							3.2E-314			
1,190,466	1,190,466	1	(C)3 -> (C)2		96	Deletion (tandem repeat)	100.0%							2.5E-154			
1,190,479	1,190,479	1	A -> G		99	SNP (transition)	100.0%							0.0			
1,190,488	1,190,488	1	G -> A		97	SNP (transition)	100.0%							0.0			
1,190,494	1,190,494	1	G -> A		97	SNP (transition)	100.0%							0.0			
1,190,561	1,190,561	1	C -> T		95	SNP (transition)	98.9%							6.0E-309			
1,190,570	1,190,570	1	C -> G		98	SNP (transversion)	100.0%							4.9E-324			
1,190,595	1,190,595	1	G -> A		92	SNP (transition)	100.0%							1.6E-313			
1,190,605	1,190,605	1	G -> C		91	SNP (transversion)	98.9%							9.1E-305			
1,190,689	1,190,689	1	G -> A		82	SNP (transition)	98.8%							3.3E-274			
1,190,695	1,190,695	1	C -> T		84	SNP (transition)	100.0%							6.3E-278			
1,190,723	1,190,724	2	GG		88	Deletion	100.0%							1.0E-132			
1,190,737	1,190,737	1	A -> G		87	SNP (transition)	100.0%							2.0E-270			
1,190,757	1,190,756	0	(C)2 -> (C)3		90	Insertion (tandem repeat)	98.9%							1.4E-283			
1,190,777	1,190,777	1	A -> C		88	SNP (transversion)	100.0%							4.0E-291			
1,190,811	1,190,811	1	A -> T		88	SNP (transversion)	100.0%	F -> I	208	TTT -> ATT	C770_RS47390	GcrA cell cycle regulator	Substitution	6.3E-300	70	1	WP_015243285.1
1,190,838	1,190,838	1	C -> G		84	SNP (transversion)	100.0%	E -> Q	181	GAA -> CAA	C770_RS47390	GcrA cell cycle regulator	Substitution	2.5E-286	61	1	WP_015243285.1
1,190,862	1,190,862	1	G -> C		85	SNP (transversion)	98.8%	P -> A	157	CCC -> GCC	C770_RS47390	GcrA cell cycle regulator	Substitution	8.5E-293	53	1	WP_015243285.1
1,190,881	1,190,881	1	T -> A		83	SNP (transversion)	100.0%		138	CTA -> CTT	C770_RS47390	GcrA cell cycle regulator	None	1.3E-274	46	3	WP_015243285.1
1,190,914	1,190,914	1	A -> G		83	SNP (transition)	97.6%		105	TGT -> TGC	C770_RS47390	GcrA cell cycle regulator	None	1.4E-272	35	3	WP_015243285.1
1,190,992	1,190,992	1	T -> C		90	SNP (transition)	100.0%		27	GTA -> GTG	C770_RS47390	GcrA cell cycle regulator	None	1.0E-306	9	3	WP_015243285.1
1,191,032	1,191,032	1	T -> C		85	SNP (transition)	100.0%							3.2E-264			
1,191,133	1,191,133	1	A -> G		96	SNP (transition)	99.0%							9.6E-322			
1,191,196	1,191,196	1	T -> G		89	SNP (transversion)	100.0%							2.0E-294			
1,191,205	1,191,205	1	G -> A		89	SNP (transition)	96.6%							4.5E-288			
1,191,227	1,191,227	1	T -> C		89	SNP (transition)	98.9%							2.2E-280			
1,191,249	1,191,249	1	C -> T		90	SNP (transition)	97.8%							1.0E-278			
1,191,261	1,191,261	1	T -> C		88	SNP (transition)	98.9%							7.0E-286			
1,191,263	1,191,263	1	T -> C		89	SNP (transition)	100.0%							2.5E-303			
1,191,311	1,191,311	1	C -> T		82	SNP (transition)	100.0%							2.5E-271			
1,191,342	1,191,342	1	A -> G		74	SNP (transition)	100.0%							1.6E-237			
1,191,348	1,191,348	1	G -> T		72	SNP (transversion)	97.2%							2.6E-228			
1,191,373	1,191,373	1	T -> C		61	SNP (transition)	100.0%							5.0E-202			
1,191,401	1,191,401	1	T -> G		51	SNP (transversion)	100.0%							5.0E-169			
1,191,442	1,191,442	1	A -> G		34	SNP (transition)	100.0%							2.5E-116			
1,191,508	1,191,508	1	A -> G		24	SNP (transition)	100.0%							2.5E-70			
1,191,522	1,191,522	1	T		20	Deletion	100.0%							1.0E-26			
1,191,611	1,191,611	1	C -> T		29	SNP (transition)	100.0%							3.2E-102			
1,191,615	1,191,615	1	C -> AAA		30	Insertion	100.0%							1.0E-96			
1,191,657	1,191,657	1	(C)4 -> (C)3		42	Deletion (tandem repeat)	100.0%							1.0E-63			
1,191,660	1,191,660	1	A -> G		43	SNP (transition)	100.0%							1.3E-142			
1,191,674	1,191,674	1	G -> C		47	SNP (transversion)	100.0%							1.6E-160			
1,191,676	1,191,676	1	T -> G		47	SNP (transversion)	100.0%							4.0E-151			
1,191,678	1,191,678	1	A -> G		47	SNP (transition)	100.0%							4.0E-151			
1,191,699	1,191,700	2	GC -> AA		54	Substitution	100.0%							2.5E-184			
1,191,708	1,191,708	1	C -> G		55	SNP (transversion)	98.2%							5.5E-188			
1,191,873	1,191,873	1	T -> C		79	SNP (transition)	98.7%		37	TTG -> CTG	C770_RS47400	hypothetical protein	None	7.9E-272	13	1	WP_015243286.1
1,192,247	1,192,247	1	T -> C		37	SNP (transition)	97.3%							1.5E-103			
1,192,437	1,192,437	1	T -> C		88	SNP (transition)	100.0%	F -> L	172	TTC -> CTC	C770_RS47405	hypothetical protein	Substitution	6.3E-300	58	1	WP_041169899.1
1,192,500	1,192,500	1	G -> A		100	SNP (transition)	99.0%	V -> I	235	GTT -> ATT	C770_RS47405	hypothetical protein	Substitution	0.0	79	1	WP_041169899.1
1,192,518	1,192,518	1	C -> G		102	SNP (transversion)	100.0%							0.0			
1,192,530	1,192,530	1	C -> A		101	SNP (transversion)	99.0%							1.0E-308			
1,192,533	1,192,532	0	CT		101	Insertion	98.0%							6.3E-304			
1,192,554	1,192,554	1	A -> G		96	SNP (transition)	99.0%							9.6E-303			
1,192,564	1,192,564	1	G -> A		94	SNP (transition)	98.9%							1.2E-305			
1,192,585	1,192,585	1	G		95	Deletion	100.0%							3.2E-143			
1,192,599	1,192,599	1	T		94	Deletion	100.0%							1.0E-141			
1,192,604	1,192,604	1	C -> G		93	SNP (transversion)	100.0%							1.3E-307			
1,192,644	1,192,644	1	G -> A		94	SNP (transition)	100.0%							6.3E-311			
1,192,648	1,192,649	2	AA -> C		93	Deletion	98.9%							3.2E-140			
1,192,684	1,192,684	1	C -> G		95	SNP (transversion)	98.9%							1.5E-299			
1,192,709	1,192,710	2	TA -> CG	90 -> 91		Substitution	98.9%							9.1E-314			
1,192,745	1,192,745	1	C -> G		89	SNP (transversion)	100.0%							3.2E-312			
1,192,752	1,192,752	1	G		84	Deletion	100.0%							1.6E-143			
1,192,758	1,192,758	1	A -> C		81	SNP (transversion)	100.0%							4.0E-276			
1,192,767	1,192,767	1	A -> T		79	SNP (transversion)	100.0%							3.2E-277			
1,192,772	1,192,772	1	C -> G		78	SNP (transversion)	100.0%							6.3E-266			
1,192,863	1,192,863	1	T -> C		86	SNP (transition)	100.0%							6.3E-276			
1,192,953	1,192,953	1	C -> G		90	SNP (transversion)	98.9%							1.1E-274			
1,192,965	1,192,965	1	(A)3 -> (A)2		87	Deletion (tandem repeat)	100.0%							3.2E-131			
1,192,989	1,192,989	1	G -> C		90	SNP (transversion)	100.0%	E -> Q	13	GAA -> CAA	C770_RS47410	hypothetical protein	Substitution	1.0E-279	5	1	WP_010975404.1
1,193,005	1,193,005	1	C -> G		96	SNP (transversion)	100.0%	A -> G	29	GCC -> GGC	C770_RS47410	hypothetical protein	Substitution	6.3E-308	10	2	WP_010975404.1
1,193,188	1,193,188	1	A -> G		91	SNP (transition)	97.8%	D -> G	212	GAC -> GGC	C770_RS47410	hypothetical protein	Substitution	4.1E-264	71	2	WP_010975404.1
1,193,226	1,193,226	1	T -> G		88	SNP (transversion)	97.7%							2.4E-272			
1,193,247	1,193,247	1	G -> A		84	SNP (transition)	96.4%							6.0E-255			
1,193,275	1,193,275	1	C -> G		81	SNP (transversion)	98.8%							8.1E-263			
1,193,282	1,193,282	1	G -> T		80	SNP (transversion)	98.8%							2.0E-267			
1,193,353	1,193,352	0	(C)2 -> (C)3		69	Insertion (tandem repeat)	95.7%							8.3E-214			
1,193,492	1,193,492	1	C -> T		31	SNP (transition)	96.8%	G -> S	340	GGC -> AGC	C770_RS47415	putative addiction modu	Substitution	3.1E-86	114	1	WP_015243288.1
1,193,502	1,193,502	1	T -> C		30	SNP (transition)	96.7%		330	CGA -> CGG	C770_RS47415	putative addiction modu	None	3.0E-86	110	3	WP_015243288.1

1,193,661	1,193,661	1	T -> G	34	SNP (transversion)	97.1%		171	GGA -> GGC	C770_RS47415	putative addiction modu	None	4.3E-108	57	3 WP_015243288.1
1,193,712	1,193,712	1	T -> C	48	SNP (transition)	100.0%		120	CTA -> CTG	C770_RS47415	putative addiction modu	None	2.5E-154	40	3 WP_015243288.1
1,193,757	1,193,757	1	T -> G	50	SNP (transversion)	100.0%	E -> D	75	GAA -> GAC	C770_RS47415	putative addiction modu	Substitution	1.0E-175	25	3 WP_015243288.1
1,193,818	1,193,818	1	A -> G	50	SNP (transition)	100.0%	M -> T	14	ATG -> ACG	C770_RS47415	putative addiction modu	Substitution	1.0E-180	5	2 WP_015243288.1
1,193,828	1,193,828	1	A -> C	50	SNP (transversion)	100.0%	S -> A	4	TCT -> GCT	C770_RS47415	putative addiction modu	Substitution	1.0E-165	2	1 WP_015243288.1
1,193,880	1,193,880	1	A -> G	48	SNP (transition)	100.0%							1.0E-144		
1,193,894	1,193,894	1	G -> C	46	SNP (transversion)	100.0%							2.5E-143		
1,193,933	1,193,933	1	G -> A	29	SNP (transition)	96.6%							4.6E-86		
1,193,970	1,193,969	0	T	14	Insertion	100.0%							4.0E-44		
1,194,005	1,194,005	1	A -> T	7	SNP (transversion)	100.0%							3.2E-18		
1,194,010	1,194,009	0	C	7	Insertion	100.0%							1.0E-21		
1,194,078	1,194,078	1	G -> T	31	SNP (transversion)	100.0%							4.0E-106		
1,194,083	1,194,083	1	C -> G	32	SNP (transversion)	100.0%							1.6E-109		
1,194,166	1,194,166	1	T -> C	51	SNP (transition)	100.0%							7.9E-159		
1,194,272	1,194,272	1	C -> G	86	SNP (transversion)	98.8%							2.7E-279		
1,194,291	1,194,291	1	(C)3 -> (C)2	92	Deletion (tandem repeat)	100.0%							1.0E-138		
1,194,361	1,194,361	1	C -> T	87	SNP (transition)	97.7%							1.2E-277		
1,194,391	1,194,391	1	G -> C	92	SNP (transversion)	98.9%							4.6E-299		
1,194,460	1,194,460	1	T -> C	94	SNP (transition)	100.0%							0.0		
1,194,463	1,194,463	1	G	94	Deletion	100.0%							4.0E-151		
1,194,534	1,194,534	1	C -> T	90	SNP (transition)	100.0%							1.0E-297		
1,194,550	1,194,550	1	T -> C	86	SNP (transition)	98.8%							2.7E-279		
1,194,558	1,194,558	1	A -> G	87	SNP (transition)	100.0%							3.2E-305		
1,194,585	1,194,585	1	G -> A	88	SNP (transition)	100.0%							4.0E-291		
1,194,598	1,194,604	7	AGGACAA	86	Deletion	100.0%							1.0E-129		
1,194,625	1,194,625	1	C -> T	90	SNP (transition)	100.0%							1.0E-306		
1,194,650	1,194,656	7	TCTCGCT	88	Deletion	100.0%							1.0E-132		
1,194,679	1,194,679	1	A -> C	90	SNP (transversion)	98.9%							2.3E-301		
1,194,969	1,194,969	1	T -> G	34	SNP (transversion)	97.1%							3.4E-98		
1,195,370	1,195,370	1	C -> T	87	SNP (transition)	97.7%		1.761	GAG -> GAA	C770_RS47430	adenylate cyclase	None	3.7E-269	587	3 WP_049589739.1
1,195,406	1,195,406	1	A -> T	83	SNP (transversion)	98.8%	D -> E	1.725	GAT -> GAA	C770_RS47430	adenylate cyclase	Substitution	2.1E-269	575	3 WP_049589739.1
1,195,481	1,195,481	1	C -> A	80	SNP (transversion)	97.5%		1.65	GCG -> GCT	C770_RS47430	adenylate cyclase	None	5.0E-239	550	3 WP_049589739.1
1,195,595	1,195,595	1	T -> G	81	SNP (transversion)	98.8%		1.536	CGA -> CGC	C770_RS47430	adenylate cyclase	None	8.1E-279	512	3 WP_049589739.1
1,195,858	1,195,858	1	G -> A	82	SNP (transition)	98.8%		1.273	CTG -> TTG	C770_RS47430	adenylate cyclase	None	3.3E-274	425	1 WP_049589739.1
1,195,943	1,195,943	1	C -> T	89	SNP (transition)	100.0%		1.188	TCG -> TCA	C770_RS47430	adenylate cyclase	None	1.6E-285	396	3 WP_049589739.1
1,196,120	1,196,120	1	C -> G	91	SNP (transversion)	98.9%		1.011	GCG -> GCC	C770_RS47430	adenylate cyclase	None	9.1E-278	337	3 WP_049589739.1
1,196,129	1,196,129	1	A -> G	88	SNP (transition)	98.9%		1.002	AAT -> AAC	C770_RS47430	adenylate cyclase	None	1.4E-294	334	3 WP_049589739.1
1,196,138	1,196,138	1	G -> T	88	SNP (transversion)	100.0%		993	CCC -> CCA	C770_RS47430	adenylate cyclase	None	4.0E-291	331	3 WP_049589739.1
1,196,468	1,196,468	1	A -> G	77	SNP (transition)	98.7%		663	ATT -> ATC	C770_RS47430	adenylate cyclase	None	3.1E-257	221	3 WP_049589739.1
1,196,537	1,196,537	1	C -> G	89	SNP (transversion)	97.8%		594	GTG -> GTC	C770_RS47430	adenylate cyclase	None	1.6E-275	198	3 WP_049589739.1
1,196,558	1,196,558	1	G -> A	90	SNP (transition)	100.0%		573	ATC -> ATT	C770_RS47430	adenylate cyclase	None	1.0E-288	191	3 WP_049589739.1
1,196,567	1,196,567	1	A -> G	91	SNP (transition)	100.0%		564	CCT -> CCC	C770_RS47430	adenylate cyclase	None	1.0E-273	188	3 WP_049589739.1
1,196,573	1,196,573	1	G -> T	94	SNP (transversion)	97.9%		558	CGC -> CGA	C770_RS47430	adenylate cyclase	None	1.1E-300	186	3 WP_049589739.1
1,196,609	1,196,609	1	T -> C	96	SNP (transition)	100.0%		522	GCA -> GCG	C770_RS47430	adenylate cyclase	None	2.5E-298	174	3 WP_049589739.1
1,196,639	1,196,639	1	A -> T	95	SNP (transversion)	98.9%		492	CGT -> CGA	C770_RS47430	adenylate cyclase	None	6.0E-309	164	3 WP_049589739.1
1,196,714	1,196,714	1	A -> G	98	SNP (transition)	99.0%		417	AAT -> AAC	C770_RS47430	adenylate cyclase	None	0.0	139	3 WP_049589739.1
1,196,861	1,196,861	1	G -> A	97	SNP (transition)	99.0%		270	AGC -> AGT	C770_RS47430	adenylate cyclase	None	6.1E-306	90	3 WP_049589739.1
1,196,873	1,196,873	1	T -> C	100	SNP (transition)	100.0%		258	GTA -> GTG	C770_RS47430	adenylate cyclase	None	0.0	86	3 WP_049589739.1
1,196,897	1,196,897	1	A -> T	97	SNP (transversion)	97.9%		234	GGT -> GGA	C770_RS47430	adenylate cyclase	None	4.7E-301	78	3 WP_049589739.1
1,196,912	1,196,912	1	G -> C	97	SNP (transversion)	100.0%		219	GCC -> GCG	C770_RS47430	adenylate cyclase	None	0.0	73	3 WP_049589739.1
1,196,957	1,196,957	1	G -> A	98	SNP (transition)	99.0%		174	TTC -> TTT	C770_RS47430	adenylate cyclase	None	2.0E-299	58	3 WP_049589739.1
1,196,966	1,196,966	1	C -> G	101	SNP (transversion)	99.0%		165	GGG -> GGC	C770_RS47430	adenylate cyclase	None	0.0	55	3 WP_049589739.1
1,196,971	1,196,971	1	C -> T	103	SNP (transition)	99.0%	A -> T	160	GCC -> ACC	C770_RS47430	adenylate cyclase	Substitution	0.0	54	1 WP_049589739.1
1,196,984	1,196,984	1	A -> G	104	SNP (transition)	99.0%		147	CGT -> CGC	C770_RS47430	adenylate cyclase	None	0.0	49	3 WP_049589739.1
1,197,000	1,197,001	2	GT -> AC	101 -> 102	Substitution	98.0%	T -> V	130	ACC -> GTC	C770_RS47430	adenylate cyclase	Substitution	5.1E-317	44	1 WP_049589739.1
1,197,017	1,197,017	1	T -> C	98	SNP (transition)	100.0%		114	CTA -> CTG	C770_RS47430	adenylate cyclase	None	2.5E-314	38	3 WP_049589739.1
1,197,023	1,197,024	2	CC -> TT	99	Substitution	97.0%	R -> K	107	AGG -> AAA	C770_RS47430	adenylate cyclase	Substitution	1.9E-307	36	2 WP_049589739.1
1,197,026	1,197,026	1	G -> T	100	SNP (transversion)	99.0%		105	CGC -> CGA	C770_RS47430	adenylate cyclase	None	1.6E-315	35	3 WP_049589739.1
1,197,047	1,197,047	1	A -> G	100	SNP (transition)	99.0%		84	CAT -> CAC	C770_RS47430	adenylate cyclase	None	1.6E-315	28	3 WP_049589739.1
1,197,130	1,197,130	1	C -> T	97	SNP (transition)	99.0%	V -> M	1	GTG -> ATG	C770_RS47430	adenylate cyclase	Substitution	1.5E-315	1	1 WP_049589739.1
1,197,159	1,197,159	1	C -> T	92	SNP (transition)	98.9%							7.3E-281		
1,197,201	1,197,203	3	AGG -> CC	93 -> 94	Deletion	98.9%							3.2E-140		
1,197,208	1,197,209	2	TG -> AA	95	Substitution	97.9%							1.5E-299		
1,197,236	1,197,237	2	CT -> GG	93	Substitution	100.0%							5.0E-289		
1,197,239	1,197,239	1	A -> T	93	SNP (transversion)	100.0%							2.5E-298		
1,197,243	1,197,244	2	AA -> GT	89	Substitution	97.8%							3.1E-284		
1,197,248	1,197,248	1	G -> C	90	SNP (transversion)	97.8%							1.0E-278		
1,197,254	1,197,254	1	C -> G	91	SNP (transversion)	100.0%							7.9E-283		
1,197,256	1,197,256	1	T -> C	91	SNP (transition)	100.0%							7.9E-283		
1,197,258	1,197,258	1	G -> A	90	SNP (transition)	100.0%							1.0E-297		
1,197,280	1,197,282	3	TGG -> CAA	86 -> 88	Substitution	97.7%							7.0E-286		
1,197,288	1,197,288	1	A	87	Deletion	100.0%							6.3E-140		
1,197,293	1,197,293	1	C -> G	87	SNP (transversion)	100.0%							1.0E-261		
1,197,296	1,197,297	2	GT -> AC	86	Substitution	98.8%							1.6E-284		
1,197,323	1,197,323	1	G -> A	87	SNP (transition)	100.0%							7.9E-288		
1,197,328	1,197,328	1	A -> G	87	SNP (transition)	100.0%							1.6E-296		
1,197,340	1,197,340	1	G -> A	91	SNP (transition)	100.0%							3.2E-319		
1,197,348	1,197,348	1	G -> T	87	SNP (transversion)	100.0%							3.2E-305		
1,197,396	1,197,396	1	A -> G	86	SNP (transition)	100.0%							4.0E-293		
1,197,493	1,197,493	1	A -> G	89	SNP (transition)	97.8%		72	GTA -> GTG	C770_RS47435	hypothetical protein	None	3.1E-284	24	3 WP_014530658.1
1,197,556	1,197,556	1	C -> G	94	SNP (transversion)	96.8%		135	CTC -> CTG	C770_RS47435	hypothetical protein	None	1.1E-277	45	3 WP_014530658.1
1,197,574	1,197,574	1	T -> C	89	SNP (transition)	100.0%		153	TTT -> TTC	C770_RS47435	hypothetical protein	None	1.3E-276	51	3 WP_014530658.1
1,197,628	1,197,628	1	C -> G	73	SNP (transversion)	100.0%	H -> Q	207	CAC -> CAG	C770_RS47435	hypothetical protein	Substitution	5.0E-227	69	3 WP_014530658.1
1,197,646	1,197,646	1	T	67	Deletion	100.0%							1.6E-94		
1,197,736	1,197,736	1	G -> A	42	SNP (transition)	97.6%							2.1E-134		
1,197,778	1,197,778	1	A -> G	29	SNP (transition)	100.0%							2.0E-96		
1,197,975	1,197,975	1	T -> A	38	SNP (transversion)	100.0%							2.5E-122		
1,198,163	1,198,163	1	A -> C	75	SNP (transversion)	100.0%							3.2E-248		
1,198,188	1,198,188	1	T -> C	83	SNP (transition)	98.8%							2.1E-269		
1,198,228	1,198,227	0	AAT	71 -> 72	Insertion	100.0%							4.0E-242		
1,198,337	1,198,337	1	A -> G	76	SNP (transition)	100.0%		1.203	GAT -> GAC	C770_RS47440	sugar ABC transporter s	None	1.6E-251	401	3 WP_015243290.1
1,198,346	1,198,346	1	A -> G	79	SNP (transition)	100.0%		1.194	CCT -> CCC	C770_RS47440	sugar ABC transporter s	None	1.6E-253	398	3 WP_015243290.1
1,198,613	1,198,613	1	G -> A	87	SNP (transition)	100.0%		927	ATC -> ATT	C770_RS47440	sugar ABC transporter s	None	3.2E-305	309	3 WP_015243290.1
1,198,625	1,198,625	1	A -> G	81	SNP (transition)	100.0%		915	CGT -> CGC	C770_RS47440	sugar ABC transporter s	None	6.3E-260	305	3 WP_015243290.1
1,198,655	1,198,655	1	C -> T	86	SNP (transition)	98.8%		885	GAG -> GAA	C770_RS47440	sugar ABC transporter s	None	8.6E-288	295	3 WP_015243290.1
1,198,667	1,198,667	1	T -> C	86	SNP (transition)	100.0%		873	CTA -> CTG	C770_RS47440	sugar ABC transporter s	None	1.6E-284	291	3 WP_015243290.1
1,198,703	1,198,703	1	T -> C	85	SNP (transition)	98.8%		837	CAA -> CAG	C770_RS47440	sugar ABC transporter s	None	2.1E-284	279	3 WP_015243290.1
1,198,724	1,198,724	1	C -> G	91	SNP (transversion)	98.9%		816	TCG -> TCC	C770_RS47440	sugar ABC transporter s	None	9.1E-287	272	3 WP_015243290.1
1,198,766	1,198,766	1	G -> A	86	SNP (transition)	100.0%		774	AAC -> AAT	C770_RS47440	sugar ABC transporter s	None	1.0E-258	258	3 WP_015243290.1
1,198,778	1,198,778	1	G -> C	84	SNP (transversion)	100.0%		762	ACC -> ACG	C770_RS47440	sugar ABC transporter s	None	6.3E-278	254	3 WP_015243290.1
1,198,787	1,198,787	1	A -> G	89	SNP (transition)	100.0%		753	GAT -> GAC	C770_RS47440	sugar ABC transporter s	None	1.0E-267	251	3 WP_015243290.1
1,198,799	1,198,799	1	A -> T	87	SNP (transversion)	97.7%		741	ATT -> ATA	C770_RS47440	sugar ABC transporter s	None	3.7E-286	247	3 WP_015243290.1
1,198,820	1,198,820	1	G -> A	95	SNP (transition)	97.9%		720	GGC -> GGT	C770_RS47440	sugar ABC transporter s	None	1.1E-294	240	3 WP_015243290.1
1,198,874	1,198,876	2	CCG -> GCT	97	Substitution	97.9%	R -> S	664	CGG -> AGC	C770_RS47440	sugar ABC transporter s	Substitution	3.0E-312	222	1 WP_015243290.1
1,198,928	1,198,928	1	G -> A	97	SNP (transition)	100.0%		612	GCC -> GCT	C770_RS47440	sugar ABC transporter s	None	4.0E-311	204	3 WP_015243290.1
1,198,931	1,198,931	1	A -> G	96	SNP (transition)	99.0%		609	GAT -> GAC	C770_RS47440	sugar ABC transporter s	None	3.0E-312	203	3 WP_015243290.1
1,198,967	1,198,967	1	G -> A	94	SNP (transition)	98.9%		573	GGC -> GGT	C770_RS47440	sugar ABC transporter s	None	5.9E-315	191	3 WP_015243290.1
1,198,979	1,198,979	1	C -> T	95	SNP (transition)	100.0%		561	AAG -> AAA	C770_RS47440	sugar ABC transporter s	None	3.2E-314	187	3 WP_015243290.1
1,199,028	1,199,028	1	T -> G	88	SNP (transversion)	98.9%	E -> A	512	GAG -> GCG	C770_RS47440	sugar ABC transporter s	Substitution	7.0E-286	171	2 WP_015243290.1
1,199,035	1,199,035	1	C -> T	87	SNP (transition)	98.9%	A -> T	505	GCC -> ACC	C770_RS47440	sugar ABC transporter s	Substitution	1.4E-282	169	1 WP_015243290.1
1,199,045	1,199,045	1	C -> T	85	SNP (transition)	98.8%		495	GGG -> GGA	C770_RS47440	sugar ABC transporter s	None	1.3E-267	165	3 WP_015243290.1
1,199,087	1,199,087	1	G -> A	86	SNP (transition)	98.8%		453	TAC -> TAT	C770_RS47440	sugar ABC transporter s	None	8.6E-288	151	3 WP_015243290.1
1,199,095	1,199,095	1	G -> T	85	SNP (transversion)	97.6%	L -> M	445	CTG -> ATG	C770_RS47440	sugar ABC transporter s	Substitution	4.5E-271	149	1 WP_015243290.1
1,199,129	1,199,129	1	G -> A	86	SNP (transition)	100.0%		411	CTC -> CTT	C770_RS47440	sugar ABC transporter s	None	6.3E-276	137	3 WP_015243290.1
1,199,141	1,199,141	1	C -> G	87	SNP (transversion)	100.0%		399	GTG -> GTC	C770_RS47440	sugar ABC transporter s	None	7.9E-288	133	3 WP_015243290.1
1,199,144	1,199,144	1	C -> T	85	SNP (transition)	98.8%		396	ACG -> ACA	C770_RS47440	sugar ABC transporter s	None	1.3E-267	132	3 WP_015243290.1
1,199,152	1,199,153	2	AA -> GG	85	Substitution	98.8%		387	AAT,TTG -> AAC,	C770_RS47440	sugar ABC transporter s	None	5.4E-276	129	3 WP_015243290.1
1,199,185	1,199,185	1	G -> C	80	SNP (transversion)	100.0%	H -> D	355	CAC -> GAC	C770_RS47440	sugar ABC transporter s	Substitution	1.0E-280	119	1 WP_015243290.1
1,199,225	1,199,225	1	G -> A	77	SNP (transition)	98.7%		315	GGC -> GGT	C770_RS47440	sugar ABC transporter s	None	7.7E-265	105	3 WP_015243290.1
1,199,231	1,199,231	1	T -> C	77	SNP (transition)	98.7%		309	AAA -> AAG	C770_RS47440	sugar ABC transporter s	None	3.1E-257	103	3 WP_015243290.1
1,199,270	1,199,270	1	G -> A	73	SNP (transition)	100.0%		270	ATC -> ATT	C770_RS47440	sugar ABC transporter s	None	6.3E-249	90	3 WP_015243290.1
1,199,348	1,199,348	1	T -> G	87	SNP (transversion)	98.9%		192	ATA -> ATC	C770_RS47440	sugar ABC transporter s	None	2.2E-265	64	3 WP_015243290.1
1,199,375	1,199,375	1	G -> A	85	SNP (transition)	100.0%		165	TTC -> TTT	C770_RS47440	sugar ABC transporter s	None	1.0E-255	55	3 WP_015243290.1
1,199,417	1,199,417	1	T -> G	75	SNP (transversion)	100.0%		123	TCA -> TCC	C770_RS47440	sugar ABC transporter s	None	3.2E-233	41	3 WP_015243290.1
1,199,450	1,199,450	1	T -> C	71	SNP (transition)	100.0%		90	GAA -> GAG	C770_RS47440	sugar ABC transporter s	None	5.0E-235	30	3 WP_015243290.1
1,199,463	1,199,463	1	G -> C	71	SNP (transversion)	100.0%	A -> G	77	GCC -> GGC	C770_RS47440	sugar ABC transporter s	Substitution	6.3E-228	26	2 WP_015243290.1
1,199,465	1,199,465	1	C -> T	71	SNP (transition)	100.0%		75	TCG -> TCA	C770_RS47440	sugar ABC transporter s	None	4.0E-242	25	3 WP_015243290.1
1,199,468	1,199,468	1	G -> C	72	SNP (transversion)	100.0%		72	CGC -> CGG	C770_RS47440	sugar ABC transporter s	None	6.3E-224	24	3 WP_015243290.1
1,199,477	1,199,477	1	G -> A	73	SNP (transition)	100.0%		63	CTC -> CTT	C770_RS47440	sugar ABC transporter s	None	2.5E-234	21	3 WP_015243290.1
1,199,512	1,199,512	1	T -> C	82	SNP (transition)	98.8%	I -> V	28	ATC -> GTC	C770_RS47440	sugar ABC transporter s	Substitution	4.1E-266	10	1 WP_015243290.1
1,199,540	1,199,540	1	G -> T	80	SNP (transversion)	98.8%							1.6E-259		
1,199,563	1,199,563	1	T -> C	83	SNP (transition)	100.0%							1.3E-274		
1,199,602	1,199,602	1	G -> A	86	SNP (transition)	100.0%							2.5E-267		
1,199,624	1,199,624	1	T -> C	86	SNP (transition)	97.7%							3.7E-291		
1,199,628	1,199,628	1	G -> A	85	SNP (transition)	100.0%							3.2E-298		
1,199,638	1,199,640	3	ATC -> GCA	84 -> 85	Substitution	100.0%							1.0E-294		
1,199,649	1,199,649	1	C -> A	82	SNP (transversion)	98.8%							2.6E-282		
1,199,685	1,199,685	1	C -> T	91	SNP (transition)	100.0%							3.2E-319		
1,199,810	1,199,810	1	C -> G	101	SNP (transversion)	100.0%	Q -> E	124	CAG -> GAG	C770_RS47445	transcriptional regulato	Substitution	0.0	42	1 WP_015243291.1
1,199,815	1,199,815	1	C -> T	99	SNP (transition)	100.0%		129	ATC -> ATT	C770_RS47445	transcriptional regulato	None	1.3E-307	43	3 WP_015243291.1
1,199,842	1,199,842	1	C -> T	99	SNP (transition)	99.0%		156	TAC -> TAT	C770_RS47445	transcriptional regulato	None	4.0E-322	52	3 WP_015243291.1
1,199,881	1,199,881	1	C -> G	103	SNP (transversion)	100.0%		195	CTC -> CTG	C770_RS47445	transcriptional regulato	None	1.0E-309	65	3 WP_015243291.1
1,199,929	1,199,929	1	T -> C	96	SNP (transition)	99.0%		243	TAT -> TAC	C770_RS47445	transcriptional regulato	None	9.6E-303	81	3 WP_015243291.1
1,200,050	1,200,050	1	A -> G	85	SNP (transition)	100.0%	S -> G	364	AGT -> GGT	C770_RS47445	transcriptional regulato	Substitution	3.2E-298	122	1 WP_015243291.1
1,200,107	1,200,107	1	T -> C	85	SNP (transition)	98.8%	F -> L	421	TTC -> CTC	C770_RS47445	transcriptional regulato	Substitution	2.1E-284	141	1 WP_015243291.1
1,200,121	1,200,121	1	C -> T	85	SNP (transition)	98.8%		435	CGC -> CGT	C770_RS47445	transcriptional regulato	None	2.1E-284	145	3 WP_015243291.1
1,200,136	1,200,136	1	C -> T	81	SNP (transition)	100.0%		450	CTC -> CTT	C770_RS47445	transcriptional regulato	None	5.0E-268	150	3 WP_015243291.1
1,200,158	1,200,160	2	TTA -> ATG	78 -> 79	Substitution	98.7%	L -> M	472	TTA -> ATG	C770_RS47445	transcriptional regulato	Substitution	6.2E-253	158	1 WP_015243291.1
1,200,184	1,200,184	1	A -> G	76	SNP (transition)	100.0%		498	CCA -> CCG	C770_RS47445	transcriptional regulato	None	1.0E-266	166	3 WP_015243291.1
1,200,232	1,200,232	1	T -> C	80	SNP (transition)	98.8%		546	GGT -> GGC	C770_RS47445	transcriptional regulato	None	1.6E-259	182	3 WP_015243291.1
1,200,250	1,200,250	1	C -> T	85	SNP (transition)	98.8%		564	CAC -> CAT	C770_RS47445	transcriptional regulato	None	5.4E-276	188	3 WP_015243291.1
1,200,307	1,200,307	1	T -> G	88	SNP (transversion)	97.7%		621	GCT -> GCG	C770_RS47445	transcriptional regulato	None	2.4E-272	207	3 WP_015243291.1
1,200,323	1,200,323	1	A -> G	88	SNP (transition)	100.0%	S -> G	637	AGC -> GGC	C770_RS47445	transcriptional regulato	Substitution	2.5E-282	213	1 WP_015243291.1
1,200,361	1,200,361	1	T -> C	91	SNP (transition)	100.0%		675	AAT -> AAC	C770_RS47445	transcriptional regulato	None	4.0E-310	225	3 WP_015243291.1
1,200,391	1,200,391	1	G -> C	91	SNP (transversion)	100.0%		705	ACG -> ACC	C770_RS47445	transcriptional regulato	None	4.0E-310	235	3 WP_015243291.1
1,200,395	1,200,397	2	GCG -> ACC	89	Substitution	97.8%	A -> T	709	GCG -> ACC	C770_RS47445	transcriptional regulato	Substitution	7.8E-267	237	1 WP_015243291.1
1,200,414	1,200,415	2	AG -> TC	90 -> 91	Substitution	98.9%	E -> V	728	GAG -> GTC	C770_RS47445	transcriptional regulato	Substitution	5.0E-301	243	2 WP_015243291.1
1,200,688	1,200,688	1	G -> C	85	SNP (transversion)	100.0%		1.002	GGG -> GGC	C770_RS47445	transcriptional regulato	None	1.0E-289	334	3 WP_015243291.1
1,200,710	1,200,710	1	C -> T	85	SNP (transition)	100.0%	P -> S	1.024	CCA -> TCA	C770_RS47445	transcriptional regulato	Substitution	1.0E-272	342	1 WP_015243291.1
1,200,768	1,200,769	2	TG -> CA	87	Substitution	97.7%	RG -> RS	57	CGT,GGC -> CGC	C770_RS47450	hypothetical protein	Substitution	3.7E-269	19	3 WP_015243292.1

1,200,818	1,200,818	1	C -> A	90	SNP (transversion)	97.8%	S -> Y	107	TCT -> TAT	C770_RS47450	hypothetical protein	Substitution	1.6E-287	36	2	WP_015243292.1
1,200,843	1,200,843	1	T -> C	84	SNP (transition)	100.0%		132	GTT -> GTC	C770_RS47450	hypothetical protein	None	4.0E-261	44	3	WP_015243292.1
1,200,994	1,200,994	1	A -> G	93	SNP (transition)	100.0%	N -> D	283	AAC -> GAC	C770_RS47450	hypothetical protein	Substitution	1.3E-307	95	1	WP_015243292.1
1,201,001	1,201,001	1	A -> G	92	SNP (transition)	100.0%	E -> G	290	GAA -> GGA	C770_RS47450	hypothetical protein	Substitution	2.5E-304	97	2	WP_015243292.1
1,201,083	1,201,083	1	G -> A	90	SNP (transition)	97.8%		372	CTG -> CTA	C770_RS47450	hypothetical protein	None	2.5E-296	124	3	WP_015243292.1
1,201,179	1,201,179	1	T -> C	96	SNP (transition)	99.0%		468	AAT -> AAC	C770_RS47450	hypothetical protein	None	3.0E-312	156	3	WP_015243292.1
1,201,182	1,201,182	1	T -> C	95	SNP (transition)	98.9%		471	AAT -> AAC	C770_RS47450	hypothetical protein	None	6.0E-309	157	3	WP_015243292.1
1,201,317	1,201,317	1	C -> T	85	SNP (transition)	97.6%		606	AAC -> AAT	C770_RS47450	hypothetical protein	None	2.3E-279	202	3	WP_015243292.1
1,201,359	1,201,359	1	T -> C	88	SNP (transition)	98.9%		648	TTT -> TTC	C770_RS47450	hypothetical protein	None	7.0E-286	216	3	WP_015243292.1
1,201,593	1,201,593	1	T -> C	83	SNP (transition)	98.8%		882	CAT -> CAC	C770_RS47450	hypothetical protein	None	1.3E-277	294	3	WP_015243292.1
1,201,977	1,201,977	1	T -> C	73	SNP (transition)	100.0%		1.266	GGT -> GGC	C770_RS47450	hypothetical protein	None	6.3E-249	422	3	WP_015243292.1
1,202,012	1,202,012	1	A -> G	74	SNP (transition)	98.6%	D -> G	1.301	GAC -> GGC	C770_RS47450	hypothetical protein	Substitution	9.3E-240	434	2	WP_015243292.1
1,202,022	1,202,022	1	C -> T	72	SNP (transition)	95.8%		1.311	TAC -> TAT	C770_RS47450	hypothetical protein	None	1.5E-230	437	3	WP_015243292.1
1,202,025	1,202,025	1	A -> G	71	SNP (transition)	100.0%		1.314	CGA -> CGG	C770_RS47450	hypothetical protein	None	4.0E-242	438	3	WP_015243292.1
1,202,085	1,202,085	1	C -> G	73	SNP (transversion)	100.0%		1.374	TCC -> TCG	C770_RS47450	hypothetical protein	None	6.3E-249	458	3	WP_015243292.1
1,202,210	1,202,210	1	A -> G	81	SNP (transition)	100.0%	Q -> R	1.499	CAG -> CGG	C770_RS47450	hypothetical protein	Substitution	6.3E-260	500	2	WP_015243292.1
1,202,217	1,202,217	1	C -> A	80	SNP (transversion)	98.8%	F -> L	1.506	TTC -> TTA	C770_RS47450	hypothetical protein	Substitution	1.3E-251	502	3	WP_015243292.1
1,202,250	1,202,250	1	G -> T	81	SNP (transversion)	97.5%		1.539	ACG -> ACT	C770_RS47450	hypothetical protein	None	6.5E-258	513	3	WP_015243292.1
1,202,284	1,202,284	1	A -> C	76	SNP (transversion)	100.0%	I -> L	1.573	ATC -> CTC	C770_RS47450	hypothetical protein	Substitution	1.0E-266	525	1	WP_015243292.1
1,202,298	1,202,298	1	T -> G	81	SNP (transversion)	100.0%		1.587	GGT -> GGG	C770_RS47450	hypothetical protein	None	7.9E-252	529	3	WP_015243292.1
1,202,337	1,202,337	1	G -> C	93	SNP (transversion)	98.9%		1.626	CCG -> CCC	C770_RS47450	hypothetical protein	None	3.7E-293	542	3	WP_015243292.1
1,202,390	1,202,390	1	A -> C	88	SNP (transversion)	95.5%	K -> T	1.679	AAA -> ACA	C770_RS47450	hypothetical protein	Substitution	5.8E-280	560	2	WP_015243292.1
1,202,392	1,202,392	1	G -> A	86	SNP (transition)	98.8%	V -> I	1.681	GTC -> ATC	C770_RS47450	hypothetical protein	Substitution	2.7E-262	561	1	WP_015243292.1
1,202,472	1,202,472	1	T -> C	80	SNP (transition)	98.8%		1.761	AAT -> AAC	C770_RS47450	hypothetical protein	None	2.0E-267	587	3	WP_015243292.1
1,202,557	1,202,557	1	G -> A	82	SNP (transition)	95.1%	A -> T	1.846	GCG -> ACG	C770_RS47450	hypothetical protein	Substitution	6.9E-252	616	1	WP_015243292.1
1,202,699	1,202,699	1	G -> C	72	SNP (transversion)	95.8%		138	CGG -> CGC	C770_RS47455	phosphoglycerate dehy	None	9.4E-217	46	3	WP_015243293.1
1,202,715	1,202,715	1	A -> G	75	SNP (transition)	98.7%	T -> A	154	ACC -> GCC	C770_RS47455	phosphoglycerate dehy	Substitution	3.0E-228	52	1	WP_015243293.1
1,202,863	1,202,864	2	GG -> AA	72	Substitution	98.6%	R -> Q	302	CGG -> CAA	C770_RS47455	phosphoglycerate dehy	Substitution	3.6E-233	101	2	WP_015243293.1
1,202,888	1,202,888	1	C -> T	69	SNP (transition)	98.6%		327	AAC -> AAT	C770_RS47455	phosphoglycerate dehy	None	4.4E-230	109	3	WP_015243293.1
1,202,967	1,202,967	1	A -> G	75	SNP (transition)	100.0%	T -> A	406	ACG -> GCG	C770_RS47455	phosphoglycerate dehy	Substitution	1.0E-255	136	1	WP_015243293.1
1,203,173	1,203,173	1	C -> T	84	SNP (transition)	97.6%		612	CTC -> CTT	C770_RS47455	phosphoglycerate dehy	None	8.7E-268	204	3	WP_015243293.1
1,203,191	1,203,191	1	G -> C	88	SNP (transversion)	100.0%		630	CGG -> CGC	C770_RS47455	phosphoglycerate dehy	None	2.5E-282	210	3	WP_015243293.1
1,203,236	1,203,236	1	A -> G	89	SNP (transition)	100.0%		675	CGA -> CGG	C770_RS47455	phosphoglycerate dehy	None	1.3E-276	225	3	WP_015243293.1
1,203,247	1,203,247	1	G -> A	85	SNP (transition)	98.8%	G -> D	686	GGT -> GAT	C770_RS47455	phosphoglycerate dehy	Substitution	1.3E-267	229	2	WP_015243293.1
1,203,256	1,203,256	1	A -> G	85	SNP (transition)	98.8%	Q -> R	695	CAG -> CGG	C770_RS47455	phosphoglycerate dehy	Substitution	1.3E-267	232	2	WP_015243293.1
1,203,263	1,203,263	1	T -> C	87	SNP (transition)	100.0%		702	GCT -> GCC	C770_RS47455	phosphoglycerate dehy	None	7.9E-288	234	3	WP_015243293.1
1,203,296	1,203,296	1	A -> G	89	SNP (transition)	100.0%		735	ACA -> ACG	C770_RS47455	phosphoglycerate dehy	None	1.3E-276	245	3	WP_015243293.1
1,203,305	1,203,305	1	G -> C	89	SNP (transversion)	100.0%		744	GGG -> GGC	C770_RS47455	phosphoglycerate dehy	None	1.6E-285	248	3	WP_015243293.1
1,203,308	1,203,308	1	A -> G	89	SNP (transition)	100.0%		747	GCA -> GCG	C770_RS47455	phosphoglycerate dehy	None	2.0E-294	249	3	WP_015243293.1
1,203,337	1,203,337	1	G -> A	100	SNP (transition)	100.0%	R -> K	776	AGA -> AAA	C770_RS47455	phosphoglycerate dehy	Substitution	1.0E-290	259	2	WP_015243293.1
1,203,401	1,203,401	1	T -> C	100	SNP (transition)	100.0%		840	AAT -> AAC	C770_RS47455	phosphoglycerate dehy	None	0.0	280	3	WP_015243293.1
1,203,584	1,203,584	1	C -> G	98	SNP (transversion)	99.0%	P -> A	16	CCG -> GCG	C770_RS47460	Sugar phosphate isome	Substitution	0.0	6	1	WP_015243294.1
1,203,601	1,203,601	1	C -> A	97	SNP (transversion)	100.0%		33	GTC -> GTA	C770_RS47460	Sugar phosphate isome	None	4.0E-311	11	3	WP_015243294.1
1,203,617	1,203,617	1	G -> C	98	SNP (transversion)	100.0%	A -> P	49	GCC -> CCC	C770_RS47460	Sugar phosphate isome	Substitution	2.5E-314	17	1	WP_015243294.1
1,203,631	1,203,631	1	C -> T	90	SNP (transition)	98.9%		63	ATC -> ATT	C770_RS47460	Sugar phosphate isome	None	1.4E-283	21	3	WP_015243294.1
1,203,640	1,203,640	1	G -> A	92	SNP (transition)	96.7%		72	CTG -> CTA	C770_RS47460	Sugar phosphate isome	None	3.2E-298	24	3	WP_015243294.1
1,203,664	1,203,664	1	T -> G	97	SNP (transversion)	97.9%		96	GCT -> GCG	C770_RS47460	Sugar phosphate isome	None	1.5E-310	32	3	WP_015243294.1
1,203,697	1,203,697	1	G -> C	93	SNP (transversion)	100.0%		129	CCG -> CCC	C770_RS47460	Sugar phosphate isome	None	5.0E-289	43	3	WP_015243294.1
1,203,721	1,203,721	1	T -> G	91	SNP (transversion)	100.0%		153	ACT -> ACG	C770_RS47460	Sugar phosphate isome	None	7.9E-283	51	3	WP_015243294.1
1,203,751	1,203,751	1	G -> C	87	SNP (transversion)	97.7%		183	CTG -> CTC	C770_RS47460	Sugar phosphate isome	None	1.2E-277	61	3	WP_015243294.1
1,203,757	1,203,757	1	C -> T	87	SNP (transition)	97.7%		189	ACC -> ACT	C770_RS47460	Sugar phosphate isome	None	1.2E-260	63	3	WP_015243294.1
1,203,820	1,203,820	1	G -> A	94	SNP (transition)	96.8%		252	GCG -> GCA	C770_RS47460	Sugar phosphate isome	None	1.1E-277	84	3	WP_015243294.1
1,203,823	1,203,823	1	T -> C	94	SNP (transition)	98.9%		255	ACT -> ACC	C770_RS47460	Sugar phosphate isome	None	2.4E-296	85	3	WP_015243294.1
1,203,905	1,203,905	1	T -> C	92	SNP (transition)	98.9%	W -> R	337	TGG -> CGG	C770_RS47460	Sugar phosphate isome	Substitution	2.9E-317	113	1	WP_015243294.1
1,203,934	1,203,934	1	T -> C	92	SNP (transition)	98.9%		366	GAT -> GAC	C770_RS47460	Sugar phosphate isome	None	3.7E-308	122	3	WP_015243294.1
1,203,940	1,203,940	1	T -> C	90	SNP (transition)	98.9%		372	GGT -> GGC	C770_RS47460	Sugar phosphate isome	None	2.3E-301	124	3	WP_015243294.1
1,203,952	1,203,952	1	C -> T	80	SNP (transition)	98.8%		384	GCC -> GCT	C770_RS47460	Sugar phosphate isome	None	1.6E-259	128	3	WP_015243294.1
1,204,024	1,204,024	1	T -> C	107	SNP (transition)	98.1%		456	CGT -> CGC	C770_RS47460	Sugar phosphate isome	None	0.0	152	3	WP_015243294.1
1,204,030	1,204,030	1	A -> C	107	SNP (transversion)	98.1%		462	ATA -> ATC	C770_RS47460	Sugar phosphate isome	None	0.0	154	3	WP_015243294.1
1,204,033	1,204,033	1	A -> G	106	SNP (transition)	98.1%		465	GAA -> GAG	C770_RS47460	Sugar phosphate isome	None	2.2E-319	155	3	WP_015243294.1
1,204,045	1,204,045	1	C -> T	106	SNP (transition)	96.2%		477	CAC -> CAT	C770_RS47460	Sugar phosphate isome	None	3.1E-310	159	3	WP_015243294.1
1,204,057	1,204,057	1	C -> T	103	SNP (transition)	96.1%		489	TAC -> TAT	C770_RS47460	Sugar phosphate isome	None	5.5E-301	163	3	WP_015243294.1
1,204,099	1,204,099	1	G -> A	92	SNP (transition)	97.8%		531	GCG -> GCA	C770_RS47460	Sugar phosphate isome	None	4.2E-294	177	3	WP_015243294.1
1,204,161	1,204,161	1	G -> A	92	SNP (transition)	97.8%	R -> H	593	CGC -> CAC	C770_RS47460	Sugar phosphate isome	Substitution	4.2E-303	198	2	WP_015243294.1
1,204,208	1,204,208	1	G -> A	104	SNP (transition)	98.1%	A -> T	640	GCG -> ACG	C770_RS47460	Sugar phosphate isome	Substitution	3.4E-313	214	1	WP_015243294.1
1,204,373	1,204,373	1	G -> A	88	SNP (transition)	100.0%	D -> N	805	GAT -> AAT	C770_RS47460	Sugar phosphate isome	Substitution	6.3E-300	269	1	WP_015243294.1
1,204,397	1,204,397	1	A -> T	93	SNP (transversion)	100.0%							2.5E-298			
1,204,424	1,204,424	1	T -> C	95	SNP (transition)	97.9%							1.4E-322			
1,204,430	1,204,430	1	C -> A	96	SNP (transversion)	100.0%							1.6E-317			
1,204,443	1,204,443	1	C -> T	94	SNP (transition)	98.9%							5.9E-315			
1,204,454	1,204,454	1	T -> C	98	SNP (transition)	100.0%							4.9E-324			
1,204,503	1,204,503	1	A -> G	92	SNP (transition)	96.7%							3.2E-298			
1,204,585	1,204,585	1	A -> G	101	SNP (transition)	100.0%							0.0			
1,204,601	1,204,601	1	G	103	Deletion	100.0%							1.6E-165			
1,204,613	1,204,613	1	A -> G	103	SNP (transition)	99.0%							0.0			
1,204,631	1,204,631	1	G -> A	109	SNP (transition)	97.2%							0.0			
1,204,694	1,204,694	1	A -> G	105	SNP (transition)	100.0%							0.0			
1,204,743	1,204,744	2	TC -> GT	104 -> 105	Substitution	100.0%	S -> V	40	TCG -> GTG	C770_RS47465	aminoglycoside adenyly	Substitution	0.0	14	1	WP_049589732.1
1,204,754	1,204,754	1	C -> G	103	SNP (transversion)	100.0%		51	CTC -> CTG	C770_RS47465	aminoglycoside adenyly	None	0.0	17	3	WP_049589732.1
1,204,804	1,204,805	2	AC -> GT	97 -> 98	Substitution	99.0%	D -> G	101	GAC -> GGT	C770_RS47465	aminoglycoside adenyly	Substitution	2.5E-314	34	2	WP_049589732.1
1,204,886	1,204,886	1	C -> T	97	SNP (transition)	99.0%		183	TTC -> TTT	C770_RS47465	aminoglycoside adenyly	None	6.1E-306	61	3	WP_049589732.1
1,204,937	1,204,937	1	C -> T	91	SNP (transition)	100.0%		234	GCC -> GCT	C770_RS47465	aminoglycoside adenyly	None	4.0E-310	78	3	WP_049589732.1
1,204,940	1,204,940	1	T -> G	92	SNP (transversion)	97.8%	N -> K	237	AAT -> AAG	C770_RS47465	aminoglycoside adenyly	Substitution	4.2E-303	79	3	WP_049589732.1
1,204,948	1,204,948	1	A -> G	91	SNP (transition)	98.9%	K -> R	245	AAG -> AGG	C770_RS47465	aminoglycoside adenyly	Substitution	9.1E-287	82	2	WP_049589732.1
1,205,010	1,205,010	1	A -> T	82	SNP (transversion)	100.0%	T -> S	307	ACA -> TCA	C770_RS47465	aminoglycoside adenyly	Substitution	2.5E-271	103	1	WP_049589732.1
1,205,105	1,205,105	1	C -> T	85	SNP (transition)	98.8%		402	GCC -> GCT	C770_RS47465	aminoglycoside adenyly	None	3.4E-301	134	3	WP_049589732.1
1,205,146	1,205,146	1	T -> A	81	SNP (transversion)	100.0%	L -> Q	443	CTA -> CAA	C770_RS47465	aminoglycoside adenyly	Substitution	4.0E-276	148	2	WP_049589732.1
1,205,206	1,205,206	1	A -> T	76	SNP (transversion)	100.0%							4.0E-259			
1,205,209	1,205,209	1	T -> C	74	SNP (transition)	100.0%							1.6E-274			
1,205,234	1,205,234	1	A -> CC	68	Insertion	100.0%							6.3E-232			
1,205,241	1,205,241	1	A -> G	65	SNP (transition)	100.0%							1.0E-221			
1,205,281	1,205,281	1	C -> G	57	SNP (transversion)	100.0%							1.6E-194			
1,205,297	1,205,297	1	G -> C	51	SNP (transversion)	100.0%							5.0E-169			
1,205,300	1,205,300	1	G -> A	51	SNP (transition)	98.0%							5.1E-169			
1,205,326	1,205,326	1	T -> G	46	SNP (transversion)	100.0%							6.3E-148			
1,205,327	1,205,326	0	G	44	Insertion	100.0%							1.6E-141			
1,205,348	1,205,348	1	G -> A	38	SNP (transition)	100.0%							2.5E-122			
1,205,350	1,205,350	1	C -> G	38	SNP (transversion)	97.4%							1.5E-117			
1,205,382	1,205,382	1	C -> G	29	SNP (transversion)	96.6%							1.8E-80			
1,205,427	1,205,429	3	CAT -> TGC	12	Substitution	100.0%							4.0E-33			
1,205,431	1,205,430	0	AT	12	Insertion	100.0%							2.5E-34			
1,205,435	1,205,435	1	G -> T	11	SNP (transversion)	100.0%							7.9E-35			
1,205,437	1,205,437	1	C -> A	11	SNP (transversion)	100.0%							7.9E-35			
1,205,441	1,205,444	4	AAAC -> CGGG	9 -> 11	Substitution	100.0%							2.5E-29			
1,205,446	1,205,449	4	CCAA -> GATC	7	Substitution	100.0%							3.2E-18			
1,205,453	1,205,456	4	GCCA -> AATC	7	Substitution	100.0%							5.0E-21			
1,205,459	1,205,459	1	A -> G	7	SNP (transition)	100.0%							4.0E-23			
1,205,462	1,205,462	1	A -> T	6	SNP (transversion)	100.0%							4.0E-15			
1,205,595	1,205,597	3	TGA -> GTC	6	Substitution	100.0%							1.0E-12			
1,205,599	1,205,600	2	TT -> CG	7 -> 8	Substitution	100.0%							6.3E-19			
1,205,603	1,205,603	1	G -> T	9	SNP (transversion)	100.0%							7.9E-27			
1,205,607	1,205,612	6	GGGCGA -> CCT	9 -> 11	Substitution	100.0%							7.9E-27			
1,205,614	1,205,614	1	C -> G	12	SNP (transversion)	100.0%							1.0E-30			
1,205,616	1,205,616	1	A -> G	12	SNP (transition)	100.0%							2.5E-40			
1,205,618	1,205,621	4	AATC -> TGAT	12	Substitution	100.0%							2.5E-34			
1,205,626	1,205,626	1	A -> G	15	SNP (transition)	100.0%							3.2E-41			
1,205,628	1,205,632	5	GAAAT -> CCGG	15 -> 16	Substitution	100.0%							1.0E-42			
1,205,637	1,205,636	0	C	17	Insertion	100.0%							1.6E-58			
1,205,638	1,205,639	2	CT -> AA	18	Substitution	100.0%							1.0E-54			
1,205,643	1,205,643	1	A -> C	18	SNP (transversion)	100.0%							2.5E-58			
1,205,645	1,205,657	13	GTTGAGACGCG	18 -> 23	Substitution	100.0%							1.6E-56			
1,205,659	1,205,661	3	CGC -> TAA	23 -> 25	Substitution	100.0%							1.0E-72			
1,205,663	1,205,664	2	GG -> TT	25	Substitution	100.0%							1.0E-80			
1,205,666	1,205,666	1	G -> A	28	SNP (transition)	100.0%							6.3E-96			
1,205,717	1,205,717	1	G -> C	44	SNP (transversion)	100.0%							2.5E-150			
1,205,724	1,205,728	5	AACTC	46	Deletion	100.0%							6.3E-79			
1,205,732	1,205,732	1	T -> C	46	SNP (transition)	100.0%							4.0E-157			
1,205,753	1,205,753	1	G -> T	52	SNP (transversion)	98.1%							3.3E-162			
1,205,772	1,205,771	0	(C)2 -> (C)3	59	Insertion (tandem repeat)	100.0%							1.6E-189			
1,205,789	1,205,789	1	A -> T	65	SNP (transversion)	100.0%		627	GTT -> GTA	C770_RS47470	hypothetical protein	None	1.0E-208	209	3	WP_015243296.1
1,205,909	1,205,909	1	G -> A	88	SNP (transition)	97.7%		507	GGC -> GGT	C770_RS47470	hypothetical protein	None	6.1E-281	169	3	WP_015243296.1
1,205,930	1,205,930	1	A -> C	84	SNP (transversion)	100.0%		486	GCT -> GCG	C770_RS47470	hypothetical protein	None	1.0E-252	162	3	WP_015243296.1
1,205,950	1,205,950	1	G -> A	87	SNP (transition)	100.0%	P -> S	466	CCC -> TCC	C770_RS47470	hypothetical protein	Substitution	2.0E-270	156	1	WP_015243296.1
1,205,977	1,205,977	1	T -> C	83	SNP (transition)	96.4%	T -> A	439	ACG -> GCG	C770_RS47470	hypothetical protein	Substitution	9.2E-252	147	1	WP_015243296.1
1,206,024	1,206,024	1	C -> G	79	SNP (transversion)	100.0%	S -> T	392	AGC -> ACC	C770_RS47470	hypothetical protein	Substitution	2.0E-261	131	2	WP_015243296.1
1,206,038	1,206,038	1	G -> A	81	SNP (transition)	98.8%		378	AGC -> AGT	C770_RS47470	hypothetical protein	None	8.1E-271	126	3	WP_015243296.1
1,206,097	1,206,097	1	T -> C	70	SNP (transition)	100.0%	T -> A	319	ACC -> GCC	C770_RS47470	hypothetical protein	Substitution	1.0E-245	107	1	WP_015243296.1
1,206,107	1,206,107	1	T -> C	71	SNP (transition)	100.0%	I -> M	309	ATA -> ATG	C770_RS47470	hypothetical protein	Substitution	4.0E-242	103	3	WP_015243296.1
1,206,122	1,206,122	1	C -> T	74	SNP (transition)	98.6%		294	CCG -> CCA	C770_RS47470	hypothetical protein	None	9.3E-240	98	3	WP_015243296.1
1,206,152	1,206,152	1	G -> A	74	SNP (transition)	100.0%		264	CGC -> CGT	C770_RS47470	hypothetical protein	None	1.0E-259	88	3	WP_015243296.1
1,206,155	1,206,155	1	T -> C	74	SNP (transition)	98.6%		261	CCA -> CCG	C770_RS47470	hypothetical protein	None	4.7E-247	87	3	WP_015243296.1
1,206,312	1,206,312	1	A -> G	74	SNP (transition)	97.3%	V -> A	104	GTC -> GCC	C770_RS47470	hypothetical protein	Substitution	4.3E-242	35	2	WP_015243296.1
1,206,513	1,206,513	1	A -> G	89	SNP (transition)	100.0%		441	GCT -> GCC	C770_RS47475	DNA-directed RNA poly	None	1.6E-285	147	3	WP_015243297.1
1,206,555	1,206,555	1	A -> G	89	SNP (transition)	100.0%		399	CGT -> CGC	C770_RS47475	DNA-directed RNA poly	None	1.6E-285	133	3	WP_015243297.1
1,206,602	1,206,602	1	A -> C	87	SNP (transversion)	100.0%	Y -> D	352	TAC -> GAC	C770_RS47475	DNA-directed RNA poly	Substitution	1.6E-296	118	1	WP_015243297.1
1,206,666	1,206,666	1	G -> A	94	SNP (transition)	100.0%		288	CAC -> CAT	C770_RS47475	DNA-directed RNA poly	None	2.5E-320	96	3	WP_015243297.1
1,206,735	1,206,735	1	C -> G	93	SNP (transversion)	100.0%		219	CCG -> CCC	C770_RS47475	DNA-directed RNA poly	None	1.0E-279	73	3	WP_015243297.1
1,206,738	1,206,738	1	C -> T	93	SNP (transition)	100.0%		216	GGG -> GGA	C770_RS47475	DNA-directed RNA poly	None	2.5E-298	72	3	WP_015243297.1
1,206,854	1,206,854	1	G -> C	84	SNP (transversion)	100.0%	L -> V	100	CTT -> GTT	C770_RS47475	DNA-directed RNA poly	Substitution	1.0E-294	34	1	WP_015243297.1
1,206,864	1,206,864	1	C -> A	81	SNP (transversion)	100.0%	R -> S	90	AGG -> AGT	C770_RS47475	DNA-directed RNA poly	Substitution	5.0E-268	30	3	WP_015243297.1
1,206,896	1,206,896	1	C -> T	86	SNP (transition)	98.8%	D -> N	58	GAC -> AAC	C770_RS47475	DNA-directed RNA poly	Substitution	2.7E-279	20	1	WP_015243297.1
1,207,002	1,207,002	1	G -> C	92	SNP (transversion)	98.9%							3.7E-308			
1,207,055	1,207,055	1	T -> C	87	SNP (transition)	100.0%							7.9E-288			
1,207,346	1,207,346	1	A -> C	88	SNP (transversion)	95.5%		144	GTA -> GTC	C770_RS47480	membrane protein	None	1.5E-271	48	3	WP_013845873.1
1,207,448	1,207,448	1	G -> C	79	SNP (transversion)	98.7%		246	GGG -> GGC	C770_RS47480	membrane protein	None	7.9E-272	82	3	WP_013845873.1
1,207,515	1,207,515	1	G -> C	74	SNP (transversion)	100.0%	V -> L	313	GTG -> CTG	C770_RS47480	membrane protein	Substitution	4.0E-230	105	1	WP_013845873.1
1,207,536	1,207,537	2	TG -> CC	69	Substitution	100.0%							1.6E-221			
1,207,573	1,207,573	1	T -> A	68	SNP (transversion)	97.1%		15	ACT -> ACA	C770_RS47485	hypothetical protein	None	5.7E-202	5	3	WP_015243298.1
1,207,582	1,207,582	1	A -> G	67	SNP (transition)	100.0%		24	GCA -> GCG	C770_RS47485	hypothetical protein	None	1.0E-201	8	3	WP_015243298.1
1,207,585	1,207,585	1	C -> T	68	SNP (transition)	98.5%		27	GAC -> GAT	C770_RS47485	hypothetical protein	None	2.7E-213	9	3	WP_015243298.1

1,207,587	1,207,587	1	C -> G		69	SNP (transversion)	100.0%	P -> R	29	CCC -> CGC	C770_RS47485	hypothetical protein	Substitution	1.6E-221	10	2	WP_015243298.1
1,207,594	1,207,594	1	A -> G		68	SNP (transition)	98.5%		36	CAA -> CAG	C770_RS47485	hypothetical protein	None	5.4E-220	12	3	WP_015243298.1
1,207,596	1,207,596	1	C -> T		70	SNP (transition)	100.0%	P -> L	38	CCG -> CTG	C770_RS47485	hypothetical protein	Substitution	1.0E-231	13	2	WP_015243298.1
1,207,648	1,207,648	1	T -> C		78	SNP (transition)	100.0%		90	AGT -> AGC	C770_RS47485	hypothetical protein	None	4.0E-258	30	3	WP_015243298.1
1,207,651	1,207,651	1	C -> T		77	SNP (transition)	100.0%		93	TTC -> TTT	C770_RS47485	hypothetical protein	None	4.0E-247	31	3	WP_015243298.1
1,207,684	1,207,684	1	C -> A		84	SNP (transversion)	100.0%	D -> E	126	GAC -> GAA	C770_RS47485	hypothetical protein	Substitution	1.6E-269	42	3	WP_015243298.1
1,207,813	1,207,813	1	T -> C		84	SNP (transition)	98.8%		255	TCT -> TCC	C770_RS47485	hypothetical protein	None	2.1E-264	85	3	WP_015243298.1
1,207,945	1,207,945	1	G -> C		84	SNP (transversion)	100.0%		387	CTG -> CTC	C770_RS47485	hypothetical protein	None	6.3E-278	129	3	WP_015243298.1
1,208,245	1,208,245	1	G -> A		100	SNP (transition)	100.0%		687	ACG -> ACA	C770_RS47485	hypothetical protein	None	0.0	229	3	WP_015243298.1
1,208,339	1,208,339	1	G -> T		86	SNP (transversion)	100.0%	A -> S	781	GCC -> TCC	C770_RS47485	hypothetical protein	Substitution	1.6E-284	261	1	WP_015243298.1
1,208,354	1,208,354	1	A -> G		88	SNP (transition)	97.7%	I -> V	796	ATT -> GTT	C770_RS47485	hypothetical protein	Substitution	6.1E-281	266	1	WP_015243298.1
1,208,359	1,208,359	1	G -> T		89	SNP (transversion)	100.0%		801	CCG -> CCT	C770_RS47485	hypothetical protein	None	2.0E-294	267	3	WP_015243298.1
1,208,507	1,208,507	1	G -> A		84	SNP (transition)	97.6%		33	GCG -> GCA	C770_RS47490	hypothetical protein	None	5.5E-276	11	3	WP_010975387.1
1,208,532	1,208,532	1	C -> G		84	SNP (transversion)	100.0%	P -> A	58	CCG -> GCG	C770_RS47490	hypothetical protein	Substitution	6.3E-278	20	1	WP_010975387.1
1,208,543	1,208,543	1	C -> G		82	SNP (transversion)	100.0%		69	GCC -> GCG	C770_RS47490	hypothetical protein	None	4.0E-263	23	3	WP_010975387.1
1,208,585	1,208,585	1	G -> A		82	SNP (transition)	98.8%		111	ACG -> ACA	C770_RS47490	hypothetical protein	None	3.3E-274	37	3	WP_010975387.1
1,208,605	1,208,605	1	C -> G		89	SNP (transversion)	100.0%	A -> G	131	GCC -> GGC	C770_RS47490	hypothetical protein	Substitution	1.6E-285	44	2	WP_010975387.1
1,208,619	1,208,620	2	GC -> AA		88	Substitution	97.7%	A -> N	145	GCT -> AAT	C770_RS47490	hypothetical protein	Substitution	6.1E-281	49	1	WP_010975387.1
1,208,652	1,208,652	1	A -> G		100	SNP (transition)	99.0%	I -> V	178	ATC -> GTC	C770_RS47490	hypothetical protein	Substitution	1.6E-315	60	1	WP_010975387.1
1,208,726	1,208,726	1	G -> C		94	SNP (transversion)	97.9%		252	CGG -> CGC	C770_RS47490	hypothetical protein	None	1.7E-291	84	3	WP_010975387.1
1,208,742	1,208,742	1	G -> A		93	SNP (transition)	100.0%	V -> M	268	GTG -> ATG	C770_RS47490	hypothetical protein	Substitution	5.0E-289	90	1	WP_010975387.1
1,208,792	1,208,792	1	C -> T		90	SNP (transition)	98.9%		318	GAC -> GAT	C770_RS47490	hypothetical protein	None	1.4E-283	106	3	WP_010975387.1
1,208,834	1,208,834	1	A -> G		96	SNP (transition)	100.0%		360	CTA -> CTG	C770_RS47490	hypothetical protein	None	2.5E-298	120	3	WP_010975387.1
1,208,870	1,208,870	1	C -> G		93	SNP (transversion)	100.0%	D -> E	396	GAC -> GAG	C770_RS47490	hypothetical protein	Substitution	5.0E-289	132	3	WP_010975387.1
1,208,885	1,208,885	1	C -> A		98	SNP (transversion)	100.0%		411	GCC -> GCA	C770_RS47490	hypothetical protein	None	4.9E-324	137	3	WP_010975387.1
1,208,894	1,208,894	1	C -> A		100	SNP (transversion)	98.0%		420	GCC -> GCA	C770_RS47490	hypothetical protein	None	1.2E-310	140	3	WP_010975387.1
1,208,975	1,208,975	1	T -> C		95	SNP (transition)	100.0%		501	GGT -> GGC	C770_RS47490	hypothetical protein	None	1.0E-304	167	3	WP_010975387.1
1,209,123	1,209,123	1	A -> G		117	SNP (transition)	99.1%	I -> V	649	ATC -> GTC	C770_RS47490	hypothetical protein	Substitution	0.0	217	1	WP_010975387.1
1,209,162	1,209,162	1	T -> C		121	SNP (transition)	99.2%	F -> L	688	TTC -> CTC	C770_RS47490	hypothetical protein	Substitution	0.0	230	1	WP_010975387.1
1,209,191	1,209,191	1	A -> T		114	SNP (transversion)	98.2%		717	GCA -> GCT	C770_RS47490	hypothetical protein	None	0.0	239	3	WP_010975387.1
1,209,202	1,209,202	1	A -> C		111	SNP (transversion)	100.0%	N -> T	728	AAT -> ACT	C770_RS47490	hypothetical protein	Substitution	0.0	243	2	WP_010975387.1
1,209,321	1,209,321	1	C -> G		107	SNP (transversion)	100.0%							0.0			
1,209,331	1,209,331	1	G -> C		108	SNP (transversion)	98.1%	G -> R	7	GGT -> CGT	C770_RS47495	membrane protein	Substitution	0.0	3	1	WP_049589740.1
1,209,346	1,209,346	1	G -> A		106	SNP (transition)	98.1%	V -> I	22	GTC -> ATC	C770_RS47495	membrane protein	Substitution	0.0	8	1	WP_049589740.1
1,209,372	1,209,372	1	G -> C		98	SNP (transversion)	99.0%		48	GCG -> GCC	C770_RS47495	membrane protein	None	7.8E-319	16	3	WP_049589740.1
1,209,477	1,209,477	1	T -> C		90	SNP (transition)	97.8%		153	GCT -> GCC	C770_RS47495	membrane protein	None	1.6E-287	51	3	WP_049589740.1
1,209,569	1,209,569	1	T -> C		95	SNP (transition)	96.8%	F -> S	245	TTC -> TCC	C770_RS47495	membrane protein	Substitution	5.5E-290	82	2	WP_049589740.1
1,209,672	1,209,672	1	G -> C		104	SNP (transversion)	98.1%		348	CCG -> CCC	C770_RS47495	membrane protein	None	3.4E-313	116	3	WP_049589740.1
1,209,772	1,209,772	1	A -> G		102	SNP (transition)	99.0%	S -> G	448	AGT -> GGT	C770_RS47495	membrane protein	Substitution	0.0	150	1	WP_049589740.1
1,209,804	1,209,803	0	A		100	Insertion	100.0%							0.0			
1,209,829	1,209,829	1	C -> G		104	SNP (transversion)	99.0%							0.0			
1,209,847	1,209,847	1	T -> G		104	SNP (transversion)	100.0%							0.0			
1,209,883	1,209,883	1	C		103	Deletion	100.0%							3.2E-155			
1,209,902	1,209,902	1	T -> G		100	SNP (transversion)	100.0%							0.0			
1,209,948	1,209,948	1	A -> C		93	SNP (transversion)	100.0%							6.3E-317			
1,209,978	1,209,978	1	A -> T		89	SNP (transversion)	98.9%							8.9E-307			
1,209,988	1,209,988	1	A -> C		91	SNP (transversion)	100.0%							4.0E-310			
1,209,997	1,209,997	1	C -> T		89	SNP (transition)	100.0%							2.5E-303			
1,210,013	1,210,013	1	G -> A		93	SNP (transition)	100.0%							1.3E-307			
1,210,051	1,210,052	2	AA -> GC		93	Substitution	98.9%							2.3E-302			
1,210,081	1,210,081	1	A -> G		92	SNP (transition)	100.0%							1.6E-313			
1,210,138	1,210,138	1	C -> T		80	SNP (transition)	97.5%	A -> T	259	GCT -> ACT	C770_RS47500	hypothetical protein	Substitution	2.0E-262	87	1	WP_015243300.1
1,210,265	1,210,265	1	G -> C		81	SNP (transversion)	100.0%		132	GCC -> GCG	C770_RS47500	hypothetical protein	None	1.0E-243	44	3	WP_015243300.1
1,210,310	1,210,310	1	G -> T		79	SNP (transversion)	100.0%		87	GGC -> GGA	C770_RS47500	hypothetical protein	None	1.3E-245	29	3	WP_015243300.1
1,210,359	1,210,359	1	A -> G		76	SNP (transition)	98.7%	I -> T	38	ATC -> ACC	C770_RS47500	hypothetical protein	Substitution	2.4E-246	13	2	WP_015243300.1
1,210,465	1,210,465	1	G -> T		82	SNP (transversion)	100.0%	N -> K	522	AAC -> AAA	C770_RS47505	transcriptional regulato	Substitution	4.0E-263	174	3	WP_015243301.1
1,210,488	1,210,488	1	T -> C		78	SNP (transition)	100.0%	K -> E	499	AAG -> GAG	C770_RS47505	transcriptional regulato	Substitution	1.0E-273	167	1	WP_015243301.1
1,210,525	1,210,525	1	G -> A		72	SNP (transition)	100.0%		462	GCC -> GCT	C770_RS47505	transcriptional regulato	None	2.5E-238	154	3	WP_015243301.1
1,210,530	1,210,530	1	G -> A		74	SNP (transition)	100.0%		457	CTA -> TTA	C770_RS47505	transcriptional regulato	None	1.6E-237	153	1	WP_015243301.1
1,210,537	1,210,537	1	T -> C		73	SNP (transition)	100.0%		450	CTA -> CTG	C770_RS47505	transcriptional regulato	None	6.3E-249	150	3	WP_015243301.1
1,210,578	1,210,578	1	T -> G		79	SNP (transversion)	100.0%	S -> R	409	AGT -> CGT	C770_RS47505	transcriptional regulato	Substitution	1.3E-245	137	1	WP_015243301.1
1,210,634	1,210,634	1	T -> C		80	SNP (transition)	98.8%	Q -> R	353	CAG -> CGG	C770_RS47505	transcriptional regulato	Substitution	1.3E-251	118	2	WP_015243301.1
1,210,636	1,210,636	1	T -> C		80	SNP (transition)	97.5%		351	GCA -> GCG	C770_RS47505	transcriptional regulato	None	7.9E-247	117	3	WP_015243301.1
1,210,647	1,210,647	1	C -> T		80	SNP (transition)	98.8%	D -> N	340	GAC -> AAC	C770_RS47505	transcriptional regulato	Substitution	1.3E-251	114	1	WP_015243301.1
1,210,651	1,210,651	1	T -> C		78	SNP (transition)	98.7%		336	AAA -> AAG	C770_RS47505	transcriptional regulato	None	1.2E-260	112	3	WP_015243301.1
1,210,802	1,210,802	1	C -> T		78	SNP (transition)	97.4%	G -> D	185	GGC -> GAC	C770_RS47505	transcriptional regulato	Substitution	4.8E-248	62	2	WP_015243301.1
1,210,805	1,210,805	1	A -> C		79	SNP (transversion)	100.0%	M -> R	182	ATG -> AGG	C770_RS47505	transcriptional regulato	Substitution	1.6E-253	61	2	WP_015243301.1
1,210,834	1,210,834	1	G -> C		85	SNP (transversion)	98.8%	D -> E	153	GAC -> GAG	C770_RS47505	transcriptional regulato	Substitution	5.4E-276	51	3	WP_015243301.1
1,210,882	1,210,882	1	T -> C		82	SNP (transition)	98.8%		105	GTA -> GTG	C770_RS47505	transcriptional regulato	None	1.3E-225	35	3	WP_015243301.1
1,210,885	1,210,885	1	A -> G		81	SNP (transition)	100.0%		102	GCT -> GCC	C770_RS47505	transcriptional regulato	None	7.9E-252	34	3	WP_015243301.1
1,210,990	1,210,990	1	T -> C		67	SNP (transition)	98.5%							4.2E-210			
1,210,993	1,210,993	1	C -> T		66	SNP (transition)	100.0%							4.0E-225			
1,211,007	1,211,007	1	G -> A		61	SNP (transition)	98.4%							6.1E-209			
1,211,028	1,211,028	1	C -> T		57	SNP (transition)	100.0%							4.0E-183			
1,211,049	1,211,049	1	C -> A		53	SNP (transversion)	98.1%							5.3E-155			
1,211,060	1,211,060	1	A -> T		49	SNP (transversion)	98.0%							1.2E-152			
1,211,067	1,211,067	1	C -> T		44	SNP (transition)	97.7%							7.0E-154			
1,211,069	1,211,069	1	C -> T		44	SNP (transition)	100.0%							2.5E-150			
1,211,085	1,211,085	1	T -> C		42	SNP (transition)	100.0%							1.6E-143			
1,211,087	1,211,087	1	G -> A		42	SNP (transition)	97.6%							1.3E-142			
1,211,097	1,211,097	1	T -> C		42	SNP (transition)	100.0%							2.5E-139			
1,211,099	1,211,100	2	TT -> GC		39	Substitution	100.0%							2.5E-133			
1,211,102	1,211,102	1	C -> G		39	SNP (transversion)	100.0%							2.0E-129			
1,211,107	1,211,107	1	G -> T		37	SNP (transversion)	97.3%							2.3E-114			
1,211,129	1,211,129	1	T -> A		25	SNP (transversion)	100.0%							3.2E-83			
1,211,135	1,211,135	1	G -> C		23	SNP (transversion)	100.0%							2.5E-74			
1,211,139	1,211,138	0	TGGTCAT	21 -> 22		Insertion	95.2% -> 95.5%							5.0E-70			
1,211,147	1,211,148	2	GC -> CA		19	Substitution	100.0%							1.3E-59			
1,211,196	1,211,196	1	C -> TTCGAT	7 -> 8		Insertion	100.0%							7.9E-24			
1,211,197	1,211,196	0	G		8	Insertion	100.0%							1.0E-24			
1,211,220	1,211,220	1	C -> G		11	SNP (transversion)	100.0%							1.6E-31			
1,211,224	1,211,224	1	A -> G		10	SNP (transition)	100.0%							1.0E-28			
1,211,228	1,211,231	4	TTCG -> GGAA		11	Substitution	100.0%							2.5E-29			
1,211,233	1,211,233	1	A -> G		11	SNP (transition)	100.0%							5.0E-26			
1,211,237	1,211,239	3	GAA -> ATT		11	Substitution	100.0%							3.2E-28			
1,211,241	1,211,242	2	AA -> GC	13 -> 14		Substitution	100.0%							1.0E-39			
1,211,245	1,211,245	1	G -> A		15	SNP (transition)	100.0%							3.2E-50			
1,211,248	1,211,248	1	A -> T		17	SNP (transversion)	100.0%							2.0E-53			
1,211,251	1,211,253	3	CAA -> ATT		18	Substitution	100.0%							2.5E-49			
1,211,256	1,211,257	2	AA -> C	18 -> 20		Deletion	100.0%							6.3E-26			
1,211,270	1,211,270	1	G -> A		24	SNP (transition)	100.0%							1.0E-72			
1,211,277	1,211,277	1	C -> G		26	SNP (transversion)	100.0%							6.3E-84			
1,211,289	1,211,289	1	T -> C		30	SNP (transition)	100.0%							1.0E-102			
1,211,292	1,211,293	2	AA -> TC		33	Substitution	100.0%							1.3E-109			
1,211,300	1,211,300	1	T -> C		33	SNP (transition)	100.0%							1.0E-99			
1,211,316	1,211,316	1	C -> T		39	SNP (transition)	97.4%							2.5E-128			
1,211,383	1,211,383	1	A -> T		62	SNP (transversion)	100.0%							1.0E-217			
1,211,395	1,211,395	1	T -> CGC		67	Insertion	98.5%							2.7E-223			
1,211,443	1,211,443	1	G -> A		81	SNP (transition)	100.0%							4.0E-276			
1,211,501	1,211,501	1	A -> G		93	SNP (transition)	100.0%							6.3E-317			
1,211,513	1,211,513	1	G -> C		95	SNP (transversion)	100.0%		12	GCG -> GCC	C770_RS47510	diguanylate cyclase	None	3.2E-314	4	3	WP_010975384.1
1,211,560	1,211,560	1	T -> C		91	SNP (transition)	98.9%	V -> A	59	GTT -> GCT	C770_RS47510	diguanylate cyclase	Substitution	9.1E-296	20	2	WP_010975384.1
1,211,699	1,211,699	1	T -> C		81	SNP (transition)	100.0%		198	GTT -> GTC	C770_RS47510	diguanylate cyclase	None	6.3E-260	66	3	WP_010975384.1
1,211,853	1,211,853	1	C -> T		64	SNP (transition)	98.4%		352	CTG -> TTG	C770_RS47510	diguanylate cyclase	None	4.0E-213	118	1	WP_010975384.1
1,211,906	1,211,906	1	A -> C		69	SNP (transversion)	100.0%		405	TCA -> TCC	C770_RS47510	diguanylate cyclase	None	1.6E-221	135	3	WP_010975384.1
1,211,918	1,211,918	1	C -> T		72	SNP (transition)	100.0%		417	ATC -> ATT	C770_RS47510	diguanylate cyclase	None	6.3E-224	139	3	WP_010975384.1
1,211,939	1,211,939	1	C -> T		69	SNP (transition)	100.0%		438	TCC -> TCT	C770_RS47510	diguanylate cyclase	None	1.0E-207	146	3	WP_010975384.1
1,212,128	1,212,129	2	TG -> CA		77	Substitution	100.0%	VV -> VM	627	GTT,GTG -> GTC,	C770_RS47510	diguanylate cyclase	Substitution	1.6E-262	209	3	WP_010975384.1
1,212,453	1,212,453	1	G -> A		91	SNP (transition)	100.0%	V -> I	952	GTA -> ATA	C770_RS47510	diguanylate cyclase	Substitution	5.0E-301	318	1	WP_010975384.1
1,212,569	1,212,569	1	G -> A		83	SNP (transition)	97.6%		1.068	TTG -> TTA	C770_RS47510	diguanylate cyclase	None	4.3E-232	356	3	WP_010975384.1
1,212,716	1,212,716	1	A -> G		83	SNP (transition)	98.8%							2.1E-269			
1,212,788	1,212,788	1	A -> G		72	SNP (transition)	100.0%							4.0E-231			
1,212,863	1,212,863	1	C -> G		73	SNP (transversion)	98.6%							1.8E-236			
1,212,895	1,212,895	1	T -> A		76	SNP (transversion)	98.7%							7.6E-254			
1,212,911	1,212,911	1	C -> A		77	SNP (transversion)	100.0%							7.9E-255			
1,213,065	1,213,065	1	C -> G		85	SNP (transversion)	100.0%							1.0E-289			
1,213,353	1,213,353	1	G -> T		98	SNP (transversion)	99.0%		201	CCC -> CCA	C770_RS47515	photosystem reaction c	None	7.8E-319	67	3	WP_010975383.1
1,213,383	1,213,383	1	G -> A		95	SNP (transition)	100.0%		171	CTC -> CTT	C770_RS47515	photosystem reaction c	None	3.2E-314	57	3	WP_010975383.1
1,213,446	1,213,446	1	T -> C		85	SNP (transition)	100.0%		108	CGA -> CGG	C770_RS47515	photosystem reaction c	None	1.0E-272	36	3	WP_010975383.1
1,213,503	1,213,504	2	TC -> CT		83	Substitution	100.0%	R -> K	50	AGA -> AAG	C770_RS47515	photosystem reaction c	Substitution	1.3E-274	17	2	WP_010975383.1
1,213,582	1,213,582	1	C -> AGAGCAGG	76 -> 79		Insertion	97.4% -> 97.5%							4.0E-221			
1,213,592	1,213,592	1	G -> T		73	SNP (transversion)	100.0%							1.0E-219			
1,213,622	1,213,622	1	C -> T		75	SNP (transition)	98.7%							4.7E-243			
1,213,629	1,213,629	1	T -> C		71	SNP (transition)	98.6%							7.1E-216			
1,213,635	1,213,635	1	A -> G		69	SNP (transition)	100.0%							2.0E-228			
1,213,642	1,213,642	1	G -> T		71	SNP (transversion)	100.0%							5.0E-235			
1,213,650	1,213,649	0	(G)5 -> (G)6		68	Insertion (tandem repeat)	98.5%							1.4E-206			
1,213,670	1,213,671	2	GT -> CC	71 -> 72		Substitution	100.0%							7.9E-221			
1,213,678	1,213,678	1	A -> G		69	SNP (transition)	100.0%							2.5E-235			
1,213,730	1,213,730	1	A -> G		75	SNP (transition)	100.0%							1.0E-240			
1,213,765	1,213,765	1	G -> A		73	SNP (transition)	100.0%							6.3E-249			
1,213,849	1,213,849	1	C -> T		71	SNP (transition)	100.0%		1.023	GCG -> GCA	C770_RS47520	FMN reductase	None	4.0E-242	341	3	WP_041169900.1
1,213,975	1,213,975	1	A -> G		86	SNP (transition)	97.7%		897	AGT -> AGC	C770_RS47520	FMN reductase	None	2.3E-274	299	3	WP_041169900.1
1,214,096	1,214,096	1	C -> T		99	SNP (transition)	98.0%	G -> E	776	GGG -> GAG	C770_RS47520	FMN reductase	Substitution	9.7E-298	259	2	WP_041169900.1
1,214,179	1,214,179	1	G -> A		96	SNP (transition)	97.9%		693	GCC -> GCT	C770_RS47520	FMN reductase	None	2.9E-307	231	3	WP_041169900.1
1,214,185	1,214,185	1	C -> T		96	SNP (transition)	99.0%		687	AAG -> AAA	C770_RS47520	FMN reductase	None	3.0E-293	229	3	WP_041169900.1
1,214,221	1,214,221	1	A -> G		95	SNP (transition)	100.0%		651	AAT -> AAC	C770_RS47520	FMN reductase	None	1.0E-285	217	3	WP_041169900.1
1,214,233	1,214,233	1	G -> A		91	SNP (transition)	96.7%		639	GCC -> GCT	C770_RS47520	FMN reductase	None	7.7E-295	213	3	WP_041169900.1
1,214,269	1,214,269	1	C -> G		90	SNP (transversion)	100.0%		603	TCG -> TCC	C770_RS47520	FMN reductase	None	1.0E-270	201	3	WP_041169900.1
1,214,332	1,214,332	1	G -> C		85	SNP (transversion)	100.0%		540	CCC -> CCG	C770_RS47520	FMN reductase	None	1.0E-289	180	3	WP_041169900.1
1,214,386	1,214,386	1	A -> G		76	SNP (transition)	100.0%		486	TGT -> TGC	C770_RS47520	FMN reductase	None	1.0E-266	162	3	WP_041169900.1
1,214,511	1,214,511	1	T -> C		80	SNP (transition)	100.0%	T -> A	361	ACG -> GCG	C770_RS47520	FMN reductase	Substitution	1.0E-272	121	1	WP_041169900.1
1,214,535	1,214,535	1	T -> C		83	SNP (transition)	100.0%	I -> V	337	ATC -> GTC	C770_RS47520	FMN reductase	Substitution	5.0E-258	113	1	WP_041169900.1
1,214,807	1,214,808	2	CA -> AG		95	Substitution	98.9%	C -> L	64	TGT -> CTT	C770_RS47520	FMN reductase	Substitution	6.0E-309	22	1	WP_041169900.1
1,214,836	1,214,836	1	G -> A		87	SNP (transition)	98.9%		36	AAC -> AAT	C770_RS47520	FMN reductase	None	1.4E-282	12	3	WP_041169900.1
1,214,913	1,214,913	1	A -> G		60	SNP (transition)	100.0%							1.0E-192			
1,214,993	1,214,993	1	A -> C		36	SNP (transversion)	97.2%							1.1E-107			
1,215,012	1,215,015	4	GGGG -> TTTT	26 -> 28		Substitution	96.2% -> 96.4%							4.0E-76			
1,215,017	1,215,020	4	GAAC -> CGTG	25 -> 26		Substitution	96.0% -> 96.2%							3.2E-68			
1,215,022	1,215,027	6	GATGAT -> CCCC	22 -> 24		Substitution	95.5% -> 95.8%							1.7E-64			

1,215,030	1,215,029	0	G	22	Insertion	100.0%							2.5E-62			
1,215,034	1,215,036	3	GAG -> TGC	17 -> 19	Substitution	100.0%							5.0E-50			
1,215,039	1,215,039	1	C -> A	16	SNP (transversion)	100.0%							6.3E-44			
1,215,041	1,215,041	1	C -> G	14	SNP (transversion)	100.0%							1.6E-38			
1,215,047	1,215,048	2	TT -> GC	11	Substitution	100.0%							1.6E-31			
1,215,051	1,215,054	4	TCGT -> CAAG	9	Substitution	100.0%							1.3E-28			
1,215,058	1,215,058	1	A -> G	8	SNP (transition)	100.0%							2.5E-26			
1,215,061	1,215,063	3	CGC -> GCG	8	Substitution	100.0%							6.3E-24			
1,215,065	1,215,065	1	G -> T	8	SNP (transversion)	100.0%							4.0E-23			
1,215,070	1,215,070	1	G -> C	7	SNP (transversion)	100.0%							1.3E-19			
1,215,072	1,215,072	1	A -> G	7	SNP (transition)	100.0%							3.2E-18			
1,219,750	1,219,750	1	T -> G	6	SNP (transversion)	100.0%							6.3E-20			
1,219,754	1,219,754	1	A -> C	8	SNP (transversion)	100.0%							1.0E-24			
1,219,756	1,219,756	1	C -> G	8	SNP (transversion)	100.0%							1.6E-25			
1,219,759	1,219,758	0	C	8	Insertion	100.0%							1.0E-24			
1,219,762	1,219,762	1	T -> A	10	SNP (transversion)	100.0%							1.0E-30			
1,219,764	1,219,764	1	C -> G	12	SNP (transversion)	100.0%							2.5E-34			
1,219,767	1,219,768	2	TA -> GC	13	Substitution	100.0%							7.9E-36			
1,219,770	1,219,771	2	AG -> GT	14	Substitution	100.0%							6.3E-40			
1,219,778	1,219,779	2	AG -> TT	15	Substitution	100.0%							3.2E-41			
1,219,782	1,219,782	1	T -> A	15	SNP (transversion)	100.0%							1.0E-39			
1,219,786	1,219,786	1	G -> C	16	SNP (transversion)	100.0%							6.3E-44			
1,219,789	1,219,789	1	G -> C	16	SNP (transversion)	100.0%							2.5E-42			
1,219,791	1,219,791	1	T -> C	18	SNP (transition)	100.0%							1.6E-47			
1,219,800	1,219,800	1	G -> A	23	SNP (transition)	95.7%							5.8E-61			
1,219,805	1,219,804	0	T	27	Insertion	100.0%							1.3E-73			
1,219,809	1,219,809	1	G -> C	28	SNP (transversion)	96.4%							7.0E-75			
1,219,811	1,219,811	1	C -> A	28	SNP (transversion)	96.4%							1.8E-69			
1,219,821	1,219,821	1	G -> A	29	SNP (transition)	100.0%							5.0E-79			
1,219,865	1,219,865	1	C -> G	49	SNP (transversion)	100.0%							1.3E-152			
1,219,896	1,219,896	1	(G)4 -> (G)3	59	Deletion (tandem repeat)	100.0%							2.5E-83			
1,219,898	1,219,898	1	T -> C	59	SNP (transition)	100.0%							1.0E-177			
1,219,901	1,219,901	1	A -> T	59	SNP (transversion)	100.0%							2.0E-195			
1,219,914	1,219,914	1	G -> T	60	SNP (transversion)	100.0%							1.0E-198			
1,219,919	1,219,919	1	T -> C	64	SNP (transition)	100.0%							4.0E-199			
1,219,938	1,219,938	1	G -> A	70	SNP (transition)	98.6%							1.4E-226			
1,219,970	1,219,970	1	A -> T	82	SNP (transversion)	98.8%							6.5E-250			
1,219,983	1,219,983	1	G -> T	82	SNP (transversion)	100.0%							1.6E-279			
1,219,990	1,219,990	1	T -> C	84	SNP (transition)	100.0%							2.5E-286			
1,220,004	1,220,004	1	C -> T	87	SNP (transition)	97.7%							1.2E-277			
1,220,053	1,220,053	1	A -> G	98	SNP (transition)	99.0%							3.9E-309			
1,220,074	1,220,074	1	T -> G	96	SNP (transversion)	100.0%							6.3E-308			
1,220,080	1,220,080	1	C -> T	97	SNP (transition)	100.0%							0.0			
1,220,133	1,220,133	1	T -> C	99	SNP (transition)	99.0%							2.5E-312			
1,220,150	1,220,150	1	A -> C	93	SNP (transversion)	98.9%							9.3E-321			
1,220,240	1,220,240	1	A -> C	85	SNP (transversion)	97.6%							4.5E-271			
1,220,255	1,220,255	1	G -> A	81	SNP (transition)	100.0%							6.3E-260			
1,220,257	1,220,256	0	A	82	Insertion	96.3%							1.4E-248			
1,220,269	1,220,269	1	G -> A	81	SNP (transition)	100.0%							6.3E-260			
1,220,281	1,220,281	1	T -> C	84	SNP (transition)	100.0%							1.6E-269			
1,220,284	1,220,284	1	T -> A	86	SNP (transversion)	98.8%							8.6E-288			
1,220,286	1,220,286	1	C -> G	86	SNP (transversion)	97.7%							5.8E-266			
1,220,291	1,220,291	1	T -> G	84	SNP (transversion)	100.0%							2.5E-286			
1,220,297	1,220,298	2	TC -> CG	85	Substitution	100.0%							1.0E-272			
1,220,300	1,220,300	1	G -> T	85	SNP (transversion)	97.6%							1.8E-254			
1,220,314	1,220,314	1	A -> G	89	SNP (transition)	100.0%							2.5E-303			
1,220,325	1,220,327	3	ACT -> C	88 -> 89	Deletion	98.9%							6.3E-124			
1,220,338	1,220,339	2	GG -> AA	86 -> 87	Substitution	100.0%							2.5E-267			
1,220,374	1,220,374	1	A -> G	81	SNP (transition)	100.0%							4.0E-276			
1,220,379	1,220,379	1	T -> A	81	SNP (transversion)	100.0%							5.0E-268			
1,220,396	1,220,396	1	C -> G	80	SNP (transversion)	100.0%							1.0E-272			
1,220,399	1,220,399	1	T -> G	79	SNP (transversion)	98.7%							3.1E-256			
1,220,405	1,220,405	1	T -> C	79	SNP (transition)	100.0%							1.6E-253			
1,220,407	1,220,407	1	T -> G	78	SNP (transversion)	100.0%							4.0E-258			
1,220,437	1,220,437	1	T -> C	71	SNP (transition)	100.0%							4.0E-242			
1,220,442	1,220,442	1	G -> A	73	SNP (transition)	100.0%							1.3E-241			
1,220,450	1,220,450	1	A -> G	72	SNP (transition)	100.0%							4.0E-231			
1,220,458	1,220,458	1	G -> A	70	SNP (transition)	98.6%	S -> L	407	TCG -> TTG	C770_RS47550	chemotaxis protein Che	Substitution	1.4E-226	136	2	WP_015243308.1
1,220,474	1,220,475	2	GA -> TG	69 -> 70	Substitution	97.1%	NL -> NM	390	AAT,CTG -> AAC,	C770_RS47550	chemotaxis protein Che	Substitution	1.8E-233	130	3	WP_015243308.1
1,220,481	1,220,481	1	T -> G	69	SNP (transversion)	100.0%		384	GCA -> GCC	C770_RS47550	chemotaxis protein Che	None	2.5E-235	128	3	WP_015243308.1
1,220,484	1,220,485	2	CG -> TA	70	Substitution	97.1%	T -> I	380	ACG -> ATA	C770_RS47550	chemotaxis protein Che	Substitution	2.4E-235	127	2	WP_015243308.1
1,220,571	1,220,571	1	C -> G	63	SNP (transversion)	100.0%	E -> D	294	GAG -> GAC	C770_RS47550	chemotaxis protein Che	Substitution	6.3E-215	98	3	WP_015243308.1
1,220,618	1,220,618	1	G -> T	66	SNP (transversion)	100.0%		247	CGG -> AGG	C770_RS47550	chemotaxis protein Che	None	6.3E-212	83	1	WP_015243308.1
1,220,621	1,220,621	1	G -> C	66	SNP (transversion)	100.0%	Q -> E	244	CAG -> GAG	C770_RS47550	chemotaxis protein Che	Substitution	4.0E-225	82	1	WP_015243308.1
1,220,637	1,220,637	1	A -> G	67	SNP (transition)	95.5%		228	GTT -> GTC	C770_RS47550	chemotaxis protein Che	None	1.2E-213	76	3	WP_015243308.1
1,220,654	1,220,654	1	C -> T	70	SNP (transition)	97.1%	A -> T	211	GCA -> ACA	C770_RS47550	chemotaxis protein Che	Substitution	6.1E-215	71	1	WP_015243308.1
1,220,676	1,220,676	1	A -> C	75	SNP (transversion)	97.3%		189	GTT -> GTG	C770_RS47550	chemotaxis protein Che	None	1.4E-223	63	3	WP_015243308.1
1,220,688	1,220,688	1	A -> C	77	SNP (transversion)	97.4%		177	CTT -> CTG	C770_RS47550	chemotaxis protein Che	None	2.9E-237	59	3	WP_015243308.1
1,220,709	1,220,710	2	TG -> CA	79	Substitution	96.2%	A -> V	155	GCA -> GTG	C770_RS47550	chemotaxis protein Che	Substitution	2.4E-251	52	2	WP_015243308.1
1,220,715	1,220,715	1	C -> T	78	SNP (transition)	100.0%		150	GCG -> GCA	C770_RS47550	chemotaxis protein Che	None	6.3E-266	50	3	WP_015243308.1
1,220,718	1,220,718	1	C -> G	79	SNP (transversion)	98.7%		147	GCG -> GCC	C770_RS47550	chemotaxis protein Che	None	1.3E-240	49	3	WP_015243308.1
1,220,731	1,220,731	1	C -> G	79	SNP (transversion)	100.0%	G -> A	134	GGG -> GCG	C770_RS47550	chemotaxis protein Che	Substitution	2.5E-269	45	2	WP_015243308.1
1,220,745	1,220,745	1	G -> C	77	SNP (transversion)	98.7%		120	GTC -> GTG	C770_RS47550	chemotaxis protein Che	None	4.9E-242	40	3	WP_015243308.1
1,220,765	1,220,765	1	G -> A	83	SNP (transition)	100.0%	L -> F	100	CTC -> TTC	C770_RS47550	chemotaxis protein Che	Substitution	2.5E-266	34	1	WP_015243308.1
1,220,811	1,220,811	1	T -> C	92	SNP (transition)	97.8%		54	GTA -> GTG	C770_RS47550	chemotaxis protein Che	None	4.2E-276	18	3	WP_015243308.1
1,220,814	1,220,814	1	C -> G	92	SNP (transversion)	97.8%		51	CTG -> CTC	C770_RS47550	chemotaxis protein Che	None	4.2E-285	17	3	WP_015243308.1
1,220,817	1,220,817	1	A -> G	92	SNP (transition)	100.0%		48	GTT -> GTC	C770_RS47550	chemotaxis protein Che	None	1.6E-267	16	3	WP_015243308.1
1,220,820	1,220,820	1	T -> C	94	SNP (transition)	97.9%		45	AGA -> AGG	C770_RS47550	chemotaxis protein Che	None	1.1E-300	15	3	WP_015243308.1
1,220,824	1,220,824	1	A -> G	94	SNP (transition)	100.0%	V -> A	41	GTC -> GCC	C770_RS47550	chemotaxis protein Che	Substitution	1.0E-282	14	2	WP_015243308.1
1,220,847	1,220,847	1	G -> T	101	SNP (transversion)	99.0%	D -> E	18	GAC -> GAA	C770_RS47550	chemotaxis protein Che	Substitution	1.0E-318	6	3	WP_015243308.1
1,220,851	1,220,851	1	G -> C	102	SNP (transversion)	99.0%	T -> S	14	ACT -> AGT	C770_RS47550	chemotaxis protein Che	Substitution	0.0	5	2	WP_015243308.1
1,220,851	1,220,851	1	G -> C	102	SNP (transversion)	99.0%	N -> K	3.483	AAC -> AAG	C770_RS47555	sensor histidine kinase	Substitution	0.0	1.161	3	WP_015243309.1
1,220,870	1,220,870	1	C -> G	100	SNP (transversion)	99.0%	R -> P	3.464	CGG -> CCG	C770_RS47555	sensor histidine kinase	Substitution	1.0E-295	1.155	2	WP_015243309.1
1,220,977	1,220,977	1	A -> C	88	SNP (transversion)	100.0%		3.357	GGT -> GGG	C770_RS47555	sensor histidine kinase	None	2.5E-282	1.119	3	WP_015243309.1
1,221,037	1,221,037	1	C -> A	96	SNP (transversion)	99.0%	R -> S	3.297	AGG -> AGT	C770_RS47555	sensor histidine kinase	Substitution	3.0E-312	1.099	3	WP_015243309.1
1,221,064	1,221,064	1	G -> A	93	SNP (transition)	97.8%		3.27	AGC -> AGT	C770_RS47555	sensor histidine kinase	None	2.1E-297	1.09	3	WP_015243309.1
1,221,070	1,221,070	1	C -> T	93	SNP (transition)	98.9%		3.264	AGG -> AGA	C770_RS47555	sensor histidine kinase	None	1.5E-311	1.088	3	WP_015243309.1
1,221,103	1,221,103	1	G -> A	91	SNP (transition)	100.0%		3.231	TAC -> TAT	C770_RS47555	sensor histidine kinase	None	4.0E-310	1.077	3	WP_015243309.1
1,221,112	1,221,112	1	A -> G	92	SNP (transition)	100.0%		3.222	GCT -> GCC	C770_RS47555	sensor histidine kinase	None	2.5E-304	1.074	3	WP_015243309.1
1,221,124	1,221,124	1	G -> A	95	SNP (transition)	98.9%		3.21	CTC -> CTT	C770_RS47555	sensor histidine kinase	None	6.0E-309	1.07	3	WP_015243309.1
1,221,145	1,221,145	1	A -> G	90	SNP (transition)	100.0%		3.189	TTT -> TTC	C770_RS47555	sensor histidine kinase	None	1.0E-315	1.063	3	WP_015243309.1
1,221,151	1,221,151	1	T -> C	88	SNP (transition)	98.9%		3.183	ACA -> ACG	C770_RS47555	sensor histidine kinase	None	1.4E-294	1.061	3	WP_015243309.1
1,221,154	1,221,154	1	A -> C	88	SNP (transversion)	100.0%		3.18	GCT -> GCG	C770_RS47555	sensor histidine kinase	None	1.0E-308	1.06	3	WP_015243309.1
1,221,181	1,221,181	1	T -> C	92	SNP (transition)	97.8%		3.153	GAA -> GAG	C770_RS47555	sensor histidine kinase	None	4.2E-303	1.051	3	WP_015243309.1
1,221,205	1,221,205	1	C -> T	91	SNP (transition)	100.0%		3.129	CGG -> CGA	C770_RS47555	sensor histidine kinase	None	5.0E-301	1.043	3	WP_015243309.1
1,221,227	1,221,227	1	T -> G	99	SNP (transversion)	100.0%	D -> A	3.107	GAT -> GCT	C770_RS47555	sensor histidine kinase	Substitution	0.0	1.036	2	WP_015243309.1
1,221,229	1,221,229	1	A -> G	100	SNP (transition)	100.0%		3.105	CTT -> CTC	C770_RS47555	sensor histidine kinase	None	0.0	1.035	3	WP_015243309.1
1,221,246	1,221,246	1	A -> G	101	SNP (transition)	100.0%		3.088	TTG -> CTG	C770_RS47555	sensor histidine kinase	None	0.0	1.03	1	WP_015243309.1
1,221,316	1,221,316	1	G -> C	106	SNP (transversion)	99.1%		3.018	CGC -> CGG	C770_RS47555	sensor histidine kinase	None	0.0	1.006	3	WP_015243309.1
1,221,328	1,221,328	1	G -> A	102	SNP (transition)	100.0%		3.006	CGC -> CGT	C770_RS47555	sensor histidine kinase	None	0.0	1.002	3	WP_015243309.1
1,221,331	1,221,331	1	T -> C	102	SNP (transition)	99.0%		3.003	GAA -> GAG	C770_RS47555	sensor histidine kinase	None	0.0	1.001	3	WP_015243309.1
1,221,370	1,221,370	1	T -> C	102	SNP (transition)	99.0%		2.964	CAA -> CAG	C770_RS47555	sensor histidine kinase	None	0.0	988	3	WP_015243309.1
1,221,373	1,221,373	1	G -> T	100	SNP (transversion)	98.0%		2.961	CGC -> CGA	C770_RS47555	sensor histidine kinase	None	2.0E-320	987	3	WP_015243309.1
1,221,376	1,221,376	1	C -> T	99	SNP (transition)	98.0%		2.958	GCG -> GCA	C770_RS47555	sensor histidine kinase	None	1.9E-307	986	3	WP_015243309.1
1,221,415	1,221,415	1	G -> A	96	SNP (transition)	97.9%		2.919	CAC -> CAT	C770_RS47555	sensor histidine kinase	None	1.1E-316	973	3	WP_015243309.1
1,221,430	1,221,432	2	TAG -> CAA	95 -> 96	Substitution	97.9%		2.902	CTA -> TTG	C770_RS47555	sensor histidine kinase	None	5.9E-315	968	1	WP_015243309.1
1,221,436	1,221,436	1	A -> G	95	SNP (transition)	98.9%		2.898	CTT -> CTC	C770_RS47555	sensor histidine kinase	None	6.0E-309	966	3	WP_015243309.1
1,221,457	1,221,457	1	C -> T	103	SNP (transition)	97.1%		2.877	CGG -> CGA	C770_RS47555	sensor histidine kinase	None	0.0	959	3	WP_015243309.1
1,221,466	1,221,466	1	G -> T	105	SNP (transversion)	99.0%	D -> E	2.868	GAC -> GAA	C770_RS47555	sensor histidine kinase	Substitution	0.0	956	3	WP_015243309.1
1,221,472	1,221,472	1	G -> A	105	SNP (transition)	99.0%		2.862	ATC -> ATT	C770_RS47555	sensor histidine kinase	None	0.0	954	3	WP_015243309.1
1,221,478	1,221,478	1	G -> A	102	SNP (transition)	96.1%		2.856	CGC -> CGT	C770_RS47555	sensor histidine kinase	None	0.0	952	3	WP_015243309.1
1,221,514	1,221,514	1	C -> T	97	SNP (transition)	99.0%		2.82	CCG -> CCA	C770_RS47555	sensor histidine kinase	None	0.0	940	3	WP_015243309.1
1,221,529	1,221,529	1	C -> T	91	SNP (transition)	98.9%		2.805	CAG -> CAA	C770_RS47555	sensor histidine kinase	None	9.1E-305	935	3	WP_015243309.1
1,221,550	1,221,550	1	T -> G	89	SNP (transversion)	97.8%		2.784	CGA -> CGC	C770_RS47555	sensor histidine kinase	None	6.2E-293	928	3	WP_015243309.1
1,221,565	1,221,565	1	G -> C	87	SNP (transversion)	95.4%		2.769	ACC -> ACG	C770_RS47555	sensor histidine kinase	None	2.8E-268	923	3	WP_015243309.1
1,221,600	1,221,601	2	CG -> GA	83	Substitution	95.2%	SE -> SQ	2.733	AGC,GAA -> AGT	C770_RS47555	sensor histidine kinase	Substitution	4.6E-263	911	3	WP_015243309.1
1,221,620	1,221,620	1	C -> T	82	SNP (transition)	97.6%	R -> K	2.714	AGG -> AAG	C770_RS47555	sensor histidine kinase	Substitution	3.3E-245	905	2	WP_015243309.1
1,221,631	1,221,631	1	G -> C	82	SNP (transversion)	97.6%		2.703	TCC -> TCG	C770_RS47555	sensor histidine kinase	None	3.3E-261	901	3	WP_015243309.1
1,221,678	1,221,678	1	T -> A	84	SNP (transversion)	96.4%	I -> F	2.656	ATT -> TTT	C770_RS47555	sensor histidine kinase	Substitution	1.2E-230	886	1	WP_015243309.1
1,221,754	1,221,754	1	T -> C	80	SNP (transition)	97.5%		2.58	GTA -> GTG	C770_RS47555	sensor histidine kinase	None	1.3E-254	860	3	WP_015243309.1
1,221,809	1,221,809	1	C -> T	84	SNP (transition)	98.8%	R -> Q	2.525	CGA -> CAA	C770_RS47555	sensor histidine kinase	Substitution	4.2E-256	842	2	WP_015243309.1
1,221,874	1,221,874	1	A -> G	86	SNP (transition)	100.0%		2.46	GTT -> GTC	C770_RS47555	sensor histidine kinase	None	6.3E-276	820	3	WP_015243309.1
1,221,880	1,221,880	1	G -> A	87	SNP (transition)	100.0%		2.454	GAC -> GAT	C770_RS47555	sensor histidine kinase	None	7.9E-288	818	3	WP_015243309.1
1,221,897	1,221,898	2	CT -> TC	87	Substitution	98.9%	TV -> TM	2.436	ACA,GTG -> ACG	C770_RS47555	sensor histidine kinase	Substitution	5.5E-274	812	3	WP_015243309.1
1,221,925	1,221,925	1	A -> G	98	SNP (transition)	98.0%		2.409	TAT -> TAC	C770_RS47555	sensor histidine kinase	None	1.2E-294	803	3	WP_015243309.1
1,221,934	1,221,934	1	G -> A	96	SNP (transition)	100.0%		2.4	GAC -> GAT	C770_RS47555	sensor histidine kinase	None	0.0	800	3	WP_015243309.1
1,221,958	1,221,958	1	C -> T	92	SNP (transition)	98.9%		2.376	GAG -> GAA	C770_RS47555	sensor histidine kinase	None	5.8E-290	792	3	WP_015243309.1
1,221,963	1,221,963	1	G -> C	90	SNP (transversion)	100.0%	L -> V	2.371	CTC -> GTC	C770_RS47555	sensor histidine kinase	Substitution	1.0E-297	791	1	WP_015243309.1
1,221,988	1,221,988	1	G -> C	91	SNP (transversion)	100.0%		2.346	CGC -> CGG	C770_RS47555	sensor histidine kinase	None	1.0E-273	782	3	WP_015243309.1
1,221,991	1,221,991	1	T -> C	91	SNP (transition)	98.9%		2.343	GCA -> GCG	C770_RS47555	sensor histidine kinase	None	9.1E-287	781	3	WP_015243309.1
1,222,002	1,222,002	1	A -> C	91	SNP (transversion)	98.9%	S -> A	2.332	TCT -> GCT	C770_RS47555	sensor histidine kinase	Substitution	9.1E-287	778	1	WP_015243309.1
1,222,012	1,222,012	1	A -> G	90	SNP (transition)	98.9%		2.322	TAT -> TAC	C770_RS47555	sensor histidine kinase	None	1.4E-283	774	3	WP_015243309.1
1,222,017	1,222,018	2	TA -> GG	87	Substitution	100.0%	LK -> LQ	2.316	CTT,AAA -> CTC,(	C770_RS47555	sensor histidine kinase	Substitution	2.0E-270	772	3	WP_015243309.1
1,222,021	1,222,023	2	ATA -> GTG	90 -> 91	Substitution	97.8%	Y -> H	2.311	TAT -> CAC	C770_RS47555	sensor histidine kinase	Substitution	1.0E-264	771	1	WP_015243309.1
1,222,048	1,222,048	1	A -> G	87	SNP (transition)	100.0%		2.286	GGT -> GGC	C770_RS47555	sensor histidine kinase	None	2.0E-270	762	3	WP_015243309.1
1,222,058	1,222,058	1	C -> T	89	SNP (transition)	100.0%	S -> N	2.276	AGC -> AAC	C770_RS47555	sensor histidine kinase	Substitution	1.3E-276	759	2	WP_015243309.1
1,222,063	1,222,063	1	C -> A	92	SNP (transversion)	98.9%		2.271	ACG -> ACT	C770_RS47555	sensor histidine kinase	None	4.6E-299	757	3	WP_015243309.1
1,222,078	1,222,078	1	C -> T	92	SNP (transition)	98.9%		2.256	GAG -> GAA	C770_RS47555	sensor histidine kinase	None	3.7E-308	752	3	WP_015243309.1
1,222,086	1,222,086	1	G -> A	92	SNP (transition)	97.8%		2.248	CTG -> TTG	C770_RS47555	sensor histidine kinase	None	4.2E-285	750	1	WP_015243309.1
1,222,099	1,222,099	1	A -> C	88	SNP (transversion)	98.9%		2.235	CGT -> CGG	C770_RS47555	sensor histidine kinase	None	3.5E-277	745	3	WP_015243309.1
1,222,118	1,222,118	1	G -> T	86	SNP (transversion)	98.8%	P -> Q	2.216	CCG -> CAG	C770_RS47555	sensor histidine kinase	Substitution	2.7E-296	739	2	WP_015243309.1
1,222,123	1,222,123	1	G -> A	89	SNP (transition)	100.0%		2.211	CTC -> CTT	C770_RS47555	sensor histidine kinase	None	2.5E-303	737	3	WP_015243309.1
1,222,126	1,222,126	1	A -> G	88	SNP (transition)	100.0%		2.208	TTT -> TTC	C770_RS47555	sensor histidine kinase	None	4.0E-291	736	3	WP_015243309.1
1,222,141	1,222,141	1	G -> A	89	SNP (transition)	100.0%		2.193	GAC -> GAT	C770_RS47555	sensor histidine kinase	None	2.5E-303	731	3	WP_015243309.1
1,222,147	1,222,147	1	C -> T	86	SNP (transition)	100.0%		2.187	GCG -> GCA	C770_RS47555	sensor histidine kinase	None	1.6E-284	729	3	WP_015243309.1
1,222,165	1,222,167	2	CAA -> TAG	83 -> 84	Substitution	100.0%		2.167	TTG -> CTA	C770_RS47555	sensor histidine kinase	None	2.5E-266	723	1	WP_015243309.1
1,222,195	1,222,195	1	C -> G	85	SNP (transversion)	98.8%		2.139	CTG -> CTC	C770_RS47555	sensor histidine kinase	None	5.4E-276	713	3	WP_015243309.1
1,222,204	1,222,204	1	T -> C	81	SNP (transition)	97.5%		2.13	AAA -> AAG	C770_RS47555	sensor histidine kinase	None	8.1E-266	710	3	WP_015243309.1
1,222,222	1,222,222	1	G -> A	75	SNP (transition)	100.0%		2.112	ACC -> ACT	C770_RS47555	sensor histidine kinase	None	1.0E-255	704	3	WP_015243309.1
1,222,237	1,222,237	1	A -> G	78	SNP (transition)	100.0%		2.097	AAT -> AAC	C770_RS47555	sensor histidine kinase	None	1.0E-273	699	3	WP_015243309.1

1,222,249	1,222,249	1	G -> T	79	SNP (transversion)	98.7%		2.085	CTC -> CTA	C770_RS47555	sensor histidine kinase	None	1.3E-279	695	3	WP_015243309.1
1,222,312	1,222,312	1	T -> C	69	SNP (transition)	100.0%		2.022	CTA -> CTG	C770_RS47555	sensor histidine kinase	None	3.2E-242	674	3	WP_015243309.1
1,222,335	1,222,336	2	GT -> AC	59	Substitution	100.0%		1.998	GAA,CTG -> GAG	C770_RS47555	sensor histidine kinase	None	2.5E-201	666	3	WP_015243309.1
1,222,342	1,222,342	1	G -> A	59	SNP (transition)	100.0%		1.992	ACC -> ACT	C770_RS47555	sensor histidine kinase	None	1.6E-189	664	3	WP_015243309.1
1,222,345	1,222,345	1	G -> A	59	SNP (transition)	98.3%		1.989	GCC -> GCT	C770_RS47555	sensor histidine kinase	None	5.9E-202	663	3	WP_015243309.1
1,222,348	1,222,348	1	G -> A	59	SNP (transition)	98.3%		1.986	ACC -> ACT	C770_RS47555	sensor histidine kinase	None	5.9E-202	662	3	WP_015243309.1
1,222,352	1,222,352	1	G -> T	59	SNP (transversion)	100.0%	A -> D	1.982	GCC -> GAC	C770_RS47555	sensor histidine kinase	Substitution	2.5E-201	661	2	WP_015243309.1
1,222,363	1,222,366	3	ACTG -> GCCC	51 -> 53	Substitution	98.0% -> 98.1%	TS -> TG	1.968	ACC,AGT -> ACG,	C770_RS47555	sensor histidine kinase	Substitution	1.0E-170	656	3	WP_015243309.1
1,222,372	1,222,372	1	T -> G	48	SNP (transversion)	100.0%		1.962	CGA -> CGC	C770_RS47555	sensor histidine kinase	None	4.0E-159	654	3	WP_015243309.1
1,222,378	1,222,378	1	A -> C	45	SNP (transversion)	100.0%		1.956	CGT -> CGG	C770_RS47555	sensor histidine kinase	None	1.0E-153	652	3	WP_015243309.1
1,222,393	1,222,393	1	T -> C	42	SNP (transition)	97.6%		1.941	CAA -> CAG	C770_RS47555	sensor histidine kinase	None	1.3E-142	647	3	WP_015243309.1
1,222,398	1,222,399	2	GC -> AT	42	Substitution	100.0%		1.935	GAG,CTG -> GAA	C770_RS47555	sensor histidine kinase	None	6.3E-131	645	3	WP_015243309.1
1,222,411	1,222,411	1	G -> T	37	SNP (transversion)	100.0%		1.923	CGC -> CGA	C770_RS47555	sensor histidine kinase	None	1.6E-126	641	3	WP_015243309.1
1,222,420	1,222,420	1	C -> T	37	SNP (transition)	100.0%		1.914	GCG -> GCA	C770_RS47555	sensor histidine kinase	None	2.0E-115	638	3	WP_015243309.1
1,222,450	1,222,450	1	A -> C	31	SNP (transversion)	100.0%		1.884	ACT -> ACG	C770_RS47555	sensor histidine kinase	None	5.0E-103	628	3	WP_015243309.1
1,222,789	1,222,789	1	A -> C	29	SNP (transversion)	96.6%		1.545	GCT -> GCG	C770_RS47555	sensor histidine kinase	None	1.2E-91	515	3	WP_015243309.1
1,222,801	1,222,801	1	C -> G	29	SNP (transversion)	100.0%		1.533	CTG -> CTC	C770_RS47555	sensor histidine kinase	None	2.0E-96	511	3	WP_015243309.1
1,222,845	1,222,849	4	CTGGT -> GCGAC	44 -> 45	Substitution	95.5% -> 95.6%	TPA -> TSP	1.485	ACA,CCA,GCG ->	C770_RS47555	sensor histidine kinase	Substitution	4.0E-135	495	3	WP_015243309.1
1,222,856	1,222,856	1	A -> C	47	SNP (transversion)	100.0%	I -> S	1.478	ATT -> AGT	C770_RS47555	sensor histidine kinase	Substitution	7.9E-156	493	2	WP_015243309.1
1,222,882	1,222,882	1	T -> A	50	SNP (transversion)	100.0%	Q -> H	1.452	CAA -> CAT	C770_RS47555	sensor histidine kinase	Substitution	1.0E-165	484	3	WP_015243309.1
1,222,909	1,222,909	1	C -> T	56	SNP (transition)	96.4%	M -> I	1.425	ATG -> ATA	C770_RS47555	sensor histidine kinase	Substitution	3.9E-181	475	3	WP_015243309.1
1,222,912	1,222,912	1	T -> A	56	SNP (transversion)	98.2%		1.422	CCA -> CCT	C770_RS47555	sensor histidine kinase	None	1.8E-180	474	3	WP_015243309.1
1,222,918	1,222,920	3	TCG	57	Deletion	100.0%	R ->	1.414	CGA ->	C770_RS47555	sensor histidine kinase	Deletion	3.2E-86	472	1	WP_015243309.1
1,222,936	1,222,936	1	G -> T	59	SNP (transversion)	96.6%		1.398	GTC -> GTA	C770_RS47555	sensor histidine kinase	None	2.7E-191	466	3	WP_015243309.1
1,222,971	1,222,971	1	T -> G	64	SNP (transversion)	98.4%	T -> P	1.363	ACT -> CCT	C770_RS47555	sensor histidine kinase	Substitution	4.0E-213	455	1	WP_015243309.1
1,222,973	1,222,973	1	A -> C	65	SNP (transversion)	100.0%	L -> R	1.361	CTC -> CGC	C770_RS47555	sensor histidine kinase	Substitution	1.0E-208	454	2	WP_015243309.1
1,222,984	1,222,984	1	G -> C	68	SNP (transversion)	98.5%		1.35	CCC -> CCG	C770_RS47555	sensor histidine kinase	None	3.4E-193	450	3	WP_015243309.1
1,222,989	1,222,989	1	G -> A	69	SNP (transition)	98.6%	H -> Y	1.345	CAC -> TAC	C770_RS47555	sensor histidine kinase	Substitution	1.7E-216	449	1	WP_015243309.1
1,222,993	1,222,993	1	G -> A	69	SNP (transition)	100.0%		1.341	GCC -> GCT	C770_RS47555	sensor histidine kinase	None	2.5E-235	447	3	WP_015243309.1
1,223,044	1,223,044	1	A -> G	75	SNP (transition)	97.3%		1.29	CAT -> CAC	C770_RS47555	sensor histidine kinase	None	3.5E-238	430	3	WP_015243309.1
1,223,065	1,223,065	1	C -> T	81	SNP (transition)	100.0%		1.269	CAG -> CAA	C770_RS47555	sensor histidine kinase	None	6.3E-260	423	3	WP_015243309.1
1,223,086	1,223,086	1	A -> G	71	SNP (transition)	100.0%		1.248	TAT -> TAC	C770_RS47555	sensor histidine kinase	None	5.0E-235	416	3	WP_015243309.1
1,223,131	1,223,131	1	G -> A	72	SNP (transition)	100.0%		1.203	CCC -> CCT	C770_RS47555	sensor histidine kinase	None	2.5E-238	401	3	WP_015243309.1
1,223,167	1,223,167	1	C -> A	71	SNP (transversion)	100.0%		1.167	GTG -> GTT	C770_RS47555	sensor histidine kinase	None	3.2E-249	389	3	WP_015243309.1
1,223,194	1,223,194	1	C -> T	71	SNP (transition)	100.0%		1.14	CGG -> CGA	C770_RS47555	sensor histidine kinase	None	5.0E-235	380	3	WP_015243309.1
1,223,208	1,223,208	1	G -> C	76	SNP (transversion)	98.7%	R -> G	1.126	CGA -> GGA	C770_RS47555	sensor histidine kinase	Substitution	2.4E-246	376	1	WP_015243309.1
1,223,253	1,223,253	1	T -> C	79	SNP (transition)	98.7%	S -> G	1.081	AGC -> GGC	C770_RS47555	sensor histidine kinase	Substitution	2.0E-248	361	1	WP_015243309.1
1,223,260	1,223,260	1	A -> C	80	SNP (transversion)	98.8%		1.074	ACT -> ACG	C770_RS47555	sensor histidine kinase	None	2.5E-275	358	3	WP_015243309.1
1,223,266	1,223,266	1	T -> C	79	SNP (transition)	100.0%		1.068	CCA -> CCG	C770_RS47555	sensor histidine kinase	None	2.5E-269	356	3	WP_015243309.1
1,223,272	1,223,272	1	T -> A	81	SNP (transversion)	98.8%		1.062	CGA -> CGT	C770_RS47555	sensor histidine kinase	None	8.1E-263	354	3	WP_015243309.1
1,223,302	1,223,303	2	TT -> AG	74	Substitution	98.6%	E -> A	1.031	GAA -> GCT	C770_RS47555	sensor histidine kinase	Substitution	9.3E-240	344	2	WP_015243309.1
1,223,329	1,223,329	1	G -> A	76	SNP (transition)	97.4%		1.005	ATC -> ATT	C770_RS47555	sensor histidine kinase	None	4.5E-234	335	3	WP_015243309.1
1,223,341	1,223,341	1	A -> G	71	SNP (transition)	97.2%		993	CTT -> CTC	C770_RS47555	sensor histidine kinase	None	5.0E-225	331	3	WP_015243309.1
1,223,344	1,223,344	1	G -> A	73	SNP (transition)	100.0%		990	GAC -> GAT	C770_RS47555	sensor histidine kinase	None	1.3E-241	330	3	WP_015243309.1
1,223,350	1,223,350	1	C -> G	72	SNP (transversion)	100.0%		984	CGG -> CGC	C770_RS47555	sensor histidine kinase	None	2.5E-238	328	3	WP_015243309.1
1,223,374	1,223,374	1	C -> G	69	SNP (transversion)	100.0%	M -> I	960	ATG -> ATC	C770_RS47555	sensor histidine kinase	Substitution	2.5E-235	320	3	WP_015243309.1
1,223,380	1,223,380	1	C -> A	67	SNP (transversion)	100.0%	M -> I	954	ATG -> ATT	C770_RS47555	sensor histidine kinase	Substitution	7.9E-222	318	3	WP_015243309.1
1,223,383	1,223,383	1	C -> T	69	SNP (transition)	97.1%		951	ACG -> ACA	C770_RS47555	sensor histidine kinase	None	1.9E-218	317	3	WP_015243309.1
1,223,392	1,223,392	1	T -> C	71	SNP (transition)	98.6%		942	GAA -> GAG	C770_RS47555	sensor histidine kinase	None	7.1E-237	314	3	WP_015243309.1
1,223,404	1,223,404	1	G -> A	71	SNP (transition)	98.6%		930	GCC -> GCT	C770_RS47555	sensor histidine kinase	None	7.1E-230	310	3	WP_015243309.1
1,223,422	1,223,422	1	G -> A	68	SNP (transition)	97.1%		912	GTC -> GTT	C770_RS47555	sensor histidine kinase	None	3.6E-215	304	3	WP_015243309.1
1,223,431	1,223,433	2	CGC -> AGT	69 -> 70	Substitution	100.0%	A -> T	901	GCG -> ACT	C770_RS47555	sensor histidine kinase	Substitution	2.0E-228	301	1	WP_015243309.1
1,223,460	1,223,461	2	AT -> GG	69 -> 70	Substitution	98.6%	QL -> HL	873	CAA,TTG -> CAC,	C770_RS47555	sensor histidine kinase	Substitution	4.4E-230	291	3	WP_015243309.1
1,223,464	1,223,464	1	C -> T	70	SNP (transition)	100.0%		870	CCG -> CCA	C770_RS47555	sensor histidine kinase	None	1.0E-231	290	3	WP_015243309.1
1,223,470	1,223,470	1	A -> G	72	SNP (transition)	100.0%		864	GTT -> GTC	C770_RS47555	sensor histidine kinase	None	1.6E-245	288	3	WP_015243309.1
1,223,475	1,223,475	1	T -> C	70	SNP (transition)	100.0%	R -> G	859	AGA -> GGA	C770_RS47555	sensor histidine kinase	Substitution	1.0E-231	287	1	WP_015243309.1
1,223,482	1,223,482	1	A -> C	71	SNP (transversion)	100.0%		852	CTT -> CTG	C770_RS47555	sensor histidine kinase	None	5.0E-235	284	3	WP_015243309.1
1,223,486	1,223,486	1	G -> A	69	SNP (transition)	100.0%	A -> V	848	GCT -> GTT	C770_RS47555	sensor histidine kinase	Substitution	2.0E-228	283	2	WP_015243309.1
1,223,494	1,223,494	1	G -> A	68	SNP (transition)	100.0%		840	GCC -> GCT	C770_RS47555	sensor histidine kinase	None	2.5E-218	280	3	WP_015243309.1
1,223,500	1,223,500	1	C -> A	70	SNP (transversion)	100.0%		834	CCG -> CCT	C770_RS47555	sensor histidine kinase	None	1.0E-224	278	3	WP_015243309.1
1,223,530	1,223,530	1	C -> T	68	SNP (transition)	100.0%		804	TTG -> TTA	C770_RS47555	sensor histidine kinase	None	2.5E-218	268	3	WP_015243309.1
1,223,563	1,223,563	1	A -> G	65	SNP (transition)	100.0%		771	CCT -> CCC	C770_RS47555	sensor histidine kinase	None	1.0E-221	257	3	WP_015243309.1
1,223,616	1,223,617	2	TT -> CC	70	Substitution	100.0%	QI -> QV	717	CAA,ATT -> CAG,	C770_RS47555	sensor histidine kinase	Substitution	1.0E-245	239	3	WP_015243309.1
1,223,643	1,223,644	2	AA -> GG	75	Substitution	100.0%		690	ATT,TTG -> ATC,(	C770_RS47555	sensor histidine kinase	None	1.0E-240	230	3	WP_015243309.1
1,223,665	1,223,665	1	T -> G	76	SNP (transversion)	100.0%		669	TCA -> TCC	C770_RS47555	sensor histidine kinase	None	1.6E-251	223	3	WP_015243309.1
1,223,764	1,223,764	1	C -> G	84	SNP (transversion)	100.0%		570	CGG -> CGC	C770_RS47555	sensor histidine kinase	None	4.0E-261	190	3	WP_015243309.1
1,223,773	1,223,773	1	C -> T	83	SNP (transition)	100.0%		561	TTG -> TTA	C770_RS47555	sensor histidine kinase	None	1.3E-274	187	3	WP_015243309.1
1,223,788	1,223,788	1	G -> A	84	SNP (transition)	100.0%		546	CAC -> CAT	C770_RS47555	sensor histidine kinase	None	1.0E-252	182	3	WP_015243309.1
1,223,800	1,223,800	1	G -> T	85	SNP (transversion)	98.8%		534	CGC -> CGA	C770_RS47555	sensor histidine kinase	None	3.4E-259	178	3	WP_015243309.1
1,223,803	1,223,803	1	T -> G	86	SNP (transversion)	100.0%		531	ATA -> ATC	C770_RS47555	sensor histidine kinase	None	1.0E-258	177	3	WP_015243309.1
1,223,821	1,223,821	1	G -> T	87	SNP (transversion)	100.0%		513	GCC -> GCA	C770_RS47555	sensor histidine kinase	None	7.9E-288	171	3	WP_015243309.1
1,223,829	1,223,829	1	C -> G	90	SNP (transversion)	100.0%	E -> Q	505	GAG -> CAG	C770_RS47555	sensor histidine kinase	Substitution	1.0E-306	169	1	WP_015243309.1
1,223,872	1,223,872	1	C -> T	87	SNP (transition)	98.9%		462	GAG -> GAA	C770_RS47555	sensor histidine kinase	None	5.5E-274	154	3	WP_015243309.1
1,223,890	1,223,890	1	C -> T	88	SNP (transition)	97.7%		444	GTG -> GTA	C770_RS47555	sensor histidine kinase	None	1.5E-289	148	3	WP_015243309.1
1,223,911	1,223,913	2	CAC -> GAT	89	Substitution	97.8%	V -> I	421	GTG -> ATC	C770_RS47555	sensor histidine kinase	Substitution	3.1E-284	141	1	WP_015243309.1
1,223,923	1,223,923	1	C -> A	91	SNP (transversion)	98.9%		411	GGG -> GGT	C770_RS47555	sensor histidine kinase	None	9.1E-296	137	3	WP_015243309.1
1,223,983	1,223,983	1	C -> T	94	SNP (transition)	100.0%		351	GAG -> GAA	C770_RS47555	sensor histidine kinase	None	4.0E-292	117	3	WP_015243309.1
1,224,016	1,224,016	1	T -> C	92	SNP (transition)	98.9%		318	GCA -> GCG	C770_RS47555	sensor histidine kinase	None	5.8E-290	106	3	WP_015243309.1
1,224,064	1,224,064	1	A -> G	89	SNP (transition)	100.0%		270	GAT -> GAC	C770_RS47555	sensor histidine kinase	None	1.6E-285	90	3	WP_015243309.1
1,224,067	1,224,067	1	A -> G	91	SNP (transition)	100.0%		267	GCT -> GCC	C770_RS47555	sensor histidine kinase	None	7.9E-283	89	3	WP_015243309.1
1,224,072	1,224,072	1	C -> T	90	SNP (transition)	100.0%	V -> I	262	GTT -> ATT	C770_RS47555	sensor histidine kinase	Substitution	1.0E-279	88	1	WP_015243309.1
1,224,079	1,224,079	1	C -> T	89	SNP (transition)	100.0%		255	CGG -> CGA	C770_RS47555	sensor histidine kinase	None	1.6E-285	85	3	WP_015243309.1
1,224,132	1,224,132	1	C -> T	84	SNP (transition)	100.0%	D -> N	202	GAC -> AAC	C770_RS47555	sensor histidine kinase	Substitution	1.6E-269	68	1	WP_015243309.1
1,224,136	1,224,136	1	G -> C	84	SNP (transversion)	100.0%		198	GTC -> GTG	C770_RS47555	sensor histidine kinase	None	1.6E-269	66	3	WP_015243309.1
1,224,142	1,224,142	1	T -> C	84	SNP (transition)	98.8%		192	ACA -> ACG	C770_RS47555	sensor histidine kinase	None	5.3E-281	64	3	WP_015243309.1
1,224,148	1,224,150	2	CGG -> GGA	82 -> 84	Substitution	96.3% -> 96.4%	P -> S	184	CCG -> TCC	C770_RS47555	sensor histidine kinase	Substitution	6.5E-250	62	1	WP_015243309.1
1,224,205	1,224,205	1	G -> A	82	SNP (transition)	100.0%		129	CTC -> CTT	C770_RS47555	sensor histidine kinase	None	2.5E-271	43	3	WP_015243309.1
1,224,256	1,224,256	1	A -> G	89	SNP (transition)	100.0%		78	TTT -> TTC	C770_RS47555	sensor histidine kinase	None	2.0E-294	26	3	WP_015243309.1
1,224,265	1,224,265	1	T -> C	84	SNP (transition)	100.0%		69	CAA -> CAG	C770_RS47555	sensor histidine kinase	None	6.3E-278	23	3	WP_015243309.1
1,224,307	1,224,307	1	G -> A	78	SNP (transition)	100.0%		27	ATC -> ATT	C770_RS47555	sensor histidine kinase	None	2.5E-250	9	3	WP_015243309.1
1,224,339	1,224,339	1	T -> A	79	SNP (transversion)	96.2%							1.3E-246			
1,224,347	1,224,347	1	C -> G	79	SNP (transversion)	98.7%							2.0E-248			
1,224,352	1,224,352	1	G -> T	78	SNP (transversion)	98.7%							1.2E-260			
1,224,359	1,224,359	1	C -> A	78	SNP (transversion)	98.7%							1.6E-237			
1,224,387	1,224,387	1	C -> G	77	SNP (transversion)	100.0%							7.9E-255			
1,224,396	1,224,396	1	G -> A	78	SNP (transition)	98.7%							1.6E-237			
1,224,431	1,224,431	1	T -> C	79	SNP (transition)	100.0%							1.6E-253			
1,224,441	1,224,441	1	A -> T	82	SNP (transversion)	98.8%							6.5E-250			
1,224,455	1,224,455	1	T	85	Deletion	100.0%							3.2E-128			
1,224,473	1,224,473	1	A -> G	80	SNP (transition)	98.8%							1.3E-251			
1,224,489	1,224,489	1	C -> T	78	SNP (transition)	100.0%							2.5E-250			
1,224,492	1,224,492	1	G	79	Deletion	100.0%							3.2E-119			
1,224,514	1,224,513	0	(C)3 -> (C)4	75	Insertion (tandem repeat)	98.7%							1.2E-235			
1,224,515	1,224,515	1	A -> G	75	SNP (transition)	97.3%							1.4E-223			
1,224,517	1,224,517	1	T -> C	75	SNP (transition)	100.0%							3.2E-233			
1,224,519	1,224,519	1	A -> T	75	SNP (transversion)	100.0%							1.0E-240			
1,224,525	1,224,525	1	T -> C	76	SNP (transition)	100.0%							1.6E-251			
1,224,629	1,224,629	1	A -> G	88	SNP (transition)	100.0%		51	GCA -> GCG	C770_RS47560	chemotaxis protein Che	None	4.0E-291	17	3	WP_015243310.1
1,224,661	1,224,662	2	GC -> CA	89	Substitution	98.9%	G -> A	83	GGC -> GCA	C770_RS47560	chemotaxis protein Che	Substitution	8.9E-307	28	2	WP_015243310.1
1,224,686	1,224,686	1	G -> C	89	SNP (transversion)	100.0%		108	GTG -> GTC	C770_RS47560	chemotaxis protein Che	None	3.2E-312	36	3	WP_015243310.1
1,224,716	1,224,716	1	T -> C	87	SNP (transition)	100.0%		138	GAT -> GAC	C770_RS47560	chemotaxis protein Che	None	1.6E-296	46	3	WP_015243310.1
1,224,732	1,224,732	1	C -> T	87	SNP (transition)	100.0%	L -> F	154	CTC -> TTC	C770_RS47560	chemotaxis protein Che	Substitution	3.2E-305	52	1	WP_015243310.1
1,224,740	1,224,740	1	C -> G	90	SNP (transversion)	100.0%		162	GCC -> GCG	C770_RS47560	chemotaxis protein Che	None	1.0E-315	54	3	WP_015243310.1
1,224,755	1,224,755	1	T -> C	94	SNP (transition)	100.0%		177	CCT -> CCC	C770_RS47560	chemotaxis protein Che	None	6.3E-311	59	3	WP_015243310.1
1,224,773	1,224,773	1	C -> T	97	SNP (transition)	100.0%		195	GAC -> GAT	C770_RS47560	chemotaxis protein Che	None	0.0	65	3	WP_015243310.1
1,224,776	1,224,776	1	T -> G	98	SNP (transversion)	100.0%		198	GGT -> GGG	C770_RS47560	chemotaxis protein Che	None	0.0	66	3	WP_015243310.1
1,224,781	1,224,781	1	C -> T	98	SNP (transition)	100.0%	A -> V	203	GCT -> GTT	C770_RS47560	chemotaxis protein Che	Substitution	4.9E-324	68	2	WP_015243310.1
1,224,785	1,224,785	1	T -> A	102	SNP (transversion)	100.0%		207	GTT -> GTA	C770_RS47560	chemotaxis protein Che	None	0.0	69	3	WP_015243310.1
1,224,789	1,224,789	1	C -> A	103	SNP (transversion)	97.1%	R -> S	211	CGC -> AGC	C770_RS47560	chemotaxis protein Che	Substitution	0.0	71	1	WP_015243310.1
1,224,863	1,224,863	1	C -> G	108	SNP (transversion)	99.1%		285	GGC -> GGG	C770_RS47560	chemotaxis protein Che	None	0.0	95	3	WP_015243310.1
1,224,893	1,224,893	1	T -> C	97	SNP (transition)	100.0%		315	GTT -> GTC	C770_RS47560	chemotaxis protein Che	None	4.0E-311	105	3	WP_015243310.1
1,224,912	1,224,912	1	G -> A	100	SNP (transition)	100.0%	V -> I	334	GTC -> ATC	C770_RS47560	chemotaxis protein Che	Substitution	1.0E-290	112	1	WP_015243310.1
1,224,936	1,224,938	2	GCA -> TCG	97 -> 98	Substitution	95.9%	A -> S	358	GCA -> TCG	C770_RS47560	chemotaxis protein Che	Substitution	1.8E-288	120	1	WP_015243310.1
1,224,945	1,224,947	2	ATT -> GTC	99	Substitution	98.0%	I -> V	367	ATT -> GTC	C770_RS47560	chemotaxis protein Che	Substitution	9.8E-290	123	1	WP_015243310.1
1,225,037	1,225,037	1	A -> C	101	SNP (transversion)	100.0%	E -> D	459	GAA -> GAC	C770_RS47560	chemotaxis protein Che	Substitution	4.9E-324	153	3	WP_015243310.1
1,225,052	1,225,052	1	C -> T	101	SNP (transition)	100.0%		474	GAC -> GAT	C770_RS47560	chemotaxis protein Che	None	7.9E-314	158	3	WP_015243310.1
1,225,068	1,225,070	2	CTG -> TTA	95	Substitution	97.9%		490	CTG -> TTA	C770_RS47560	chemotaxis protein Che	None	2.5E-298	164	1	WP_015243310.1
1,225,097	1,225,097	1	G -> T	92	SNP (transversion)	98.9%		519	GCG -> GCT	C770_RS47560	chemotaxis protein Che	None	5.8E-290	173	3	WP_015243310.1
1,225,106	1,225,106	1	A -> C	89	SNP (transversion)	98.9%	E -> D	528	GAA -> GAC	C770_RS47560	chemotaxis protein Che	Substitution	3.5E-289	176	3	WP_015243310.1
1,225,108	1,225,108	1	A -> G	90	SNP (transition)	98.9%	D -> G	530	GAT -> GGT	C770_RS47560	chemotaxis protein Che	Substitution	1.1E-274	177	2	WP_015243310.1
1,225,162	1,225,162	1	T -> C	87	SNP (transition)	98.9%	L -> P	584	CTC -> CCC	C770_RS47560	chemotaxis protein Che	Substitution	1.4E-282	195	2	WP_015243310.1
1,225,184	1,225,184	1	T -> C	92	SNP (transition)	98.9%		606	CTT -> CTC	C770_RS47560	chemotaxis protein Che	None	3.7E-308	202	3	WP_015243310.1
1,225,190	1,225,190	1	T -> C	93	SNP (transition)	100.0%		612	GTT -> GTC	C770_RS47560	chemotaxis protein Che	None	2.5E-298	204	3	WP_015243310.1
1,225,200	1,225,202	2	CTT -> TTC	87 -> 89	Substitution	97.7% -> 97.8%	L -> F	622	CTT -> TTC	C770_RS47560	chemotaxis protein Che	Substitution	7.0E-286	208	1	WP_015243310.1
1,225,207	1,225,207	1	T -> G	85	SNP (transversion)	97.6%	L -> R	629	CTA -> CGA	C770_RS47560	chemotaxis protein Che	Substitution	1.1E-287	210	2	WP_015243310.1
1,225,313	1,225,313	1	G -> A	81	SNP (transition)	98.8%		735	CGG -> CGA	C770_RS47560	chemotaxis protein Che	None	8.1E-263	245	3	WP_015243310.1
1,225,363	1,225,363	1	C -> A	86	SNP (transversion)	97.7%	A -> E	785	GCG -> GAG	C770_RS47560	chemotaxis protein Che	Substitution	9.2E-283	262	2	WP_015243310.1
1,225,406	1,225,406	1	C -> G	84	SNP (transversion)	98.8%		828	CGC -> CGG	C770_RS47560	chemotaxis protein Che	None	2.1E-264	276	3	WP_015243310.1
1,225,421	1,225,421	1	A -> G	86	SNP (transition)	100.0%		843	AGA -> AGG	C770_RS47560	chemotaxis protein Che	None	4.0E-293	281	3	WP_015243310.1
1,225,460	1,225,460	1	A -> T	86	SNP (transversion)	97.7%		882	CGA -> CGT	C770_RS47560	chemotaxis protein Che	None	2.3E-274	294	3	WP_015243310.1
1,225,466	1,225,466	1	A -> G	89	SNP (transition)	97.8%		888	GGA -> GGG	C770_RS47560	chemotaxis protein Che	None	7.8E-267	296	3	WP_015243310.1
1,225,492	1,225,492	1	G -> A	87	SNP (transition)	100.0%	R -> K	914	AGA -> AAA	C770_RS47560	chemotaxis protein Che	Substitution	7.9E-288	305	2	WP_015243310.1
1,225,530	1,225,532	2	TTG -> CTA	91	Substitution	97.8% -> 98.9%		952	TTG -> CTA	C770_RS47560	chemotaxis protein Che	None	1.0E-297	318	1	WP_015243310.1
1,225,555	1,225,557	3	GGA -> CAC	93 -> 95	Substitution	94.7% -> 96.8%	GT -> AP	977	GGG,ACG -> GCA	C770_RS47560	chemotaxis protein Che	Substitution	2.0E-285	326	2	WP_015243310.1
1,225,562	1,225,562	1	C -> A	94	SNP (transversion)	96.8%	D -> E	984	GAC -> GAA	C770_RS47560	chemotaxis protein Che	Substitution	1.1E-277	328	3	WP_015243310.1
1,225,575	1,225,575	1	G -> A	91	SNP (transition)	97.8%	E -> K	997	GAA -> AAA	C770_RS47560	chemotaxis protein Che	Substitution	5.1E-273	333	1	WP_015243310.1
1,225,591	1,225,591	1	A -> G	85	SNP (transition)	97.6%	D -> G	1.013	GAT -> GGT	C770_RS47560	chemotaxis protein Che	Substitution	9.0E-263	338	2	WP_015243310.1
1,225,604	1,225,604	1	T -> C	83	SNP (transition)	97.6%		1.026	GCT -> GCC	C770_RS47560	chemotaxis protein Che	None	1.7E-264	342	3	WP_015243310.1
1,225,617	1,225,617	1	A -> C	82	SNP (transversion)	97.6%	T -> P	1.039	ACA -> CCA	C770_RS47560	chemotaxis protein Che	Substitution	3.3E-253	347	1	WP_015243310.1
1,225,621	1,225,621	1	T -> C	80	SNP (transition)	97.5%	I -> T	1.043	ATC -> ACC	C770_RS47560	chemotaxis protein Che	Substitution	2.0E-262	348	2	WP_015243310.1
1,225,654	1,225,654	1	G -> TC	67	Insertion	97.0%							2.2E-218			
1,225,675	1,225,675	1	G -> C	61	SNP (transversion)	96.7%							5.8E-204			
1,225,679	1,225,679	1	A -> G	60	SNP (transition)	96.7%							1.8E-200			
1,225,683	1,225,686	4	AAAT -> G	59 -> 60	Deletion	96.6% -> 96.7%							1.0E-88			
1,225,710	1,225,710	1	T -> C	57	SNP (transition)	96.5%	D -> G	143	GAC -> GGC	C770_RS47565	histidine kinase	Substitution	5.0E-179	48	2	WP_041169932.1
1,225,712	1,225,712	1	C -> G	57	SNP (transversion)	96.5%		141	ACG -> ACC	C770_RS47565	histidine kinase	None	5.0E-179	47	3	WP_041169932.1
1,225,719	1,225,719	1	C -> G	54	SNP (transversion)	96.3%	R -> P	134	CGT -> CCT	C770_RS47565	histidine kinase	Substitution	5.7E-164	45	2	WP_041169932.1
1,225,721	1,225,721	1	C	54	Deletion	96.3%		132		C770_RS47565	histidine kinase	Frame Shift	1.3E-75	44	3	WP_041169932.1
1,225,733	1,225,733	1	G	52	Deletion	96.2%		120		C770_RS47565	histidine kinase	Frame Shift	1.2E-72	40	3	WP_041169932.1
1,225,860	1,225,860	1	G -> C	6	SNP (transversion)	100.0%							1.6E-17			

1,226,254	1,226,257	4	TCTT -> GTCC		6	Substitution	100.0%							4.0E-18			
1,226,259	1,226,259	1	T -> C		7	SNP (transition)	100.0%							1.0E-21			
1,226,261	1,226,266	6	GGTGCC -> CTGA	7 -> 9		Substitution	100.0%							7.9E-24			
1,226,268	1,226,271	4	TTGG -> AAAC		10	Substitution	100.0%							1.0E-31			
1,226,273	1,226,274	2	CG -> AC		10	Substitution	100.0%							1.0E-30			
1,226,276	1,226,279	4	TTTT -> GCCA	10 -> 11		Substitution	100.0%							1.0E-34			
1,226,281	1,226,281	1	C -> T		11	SNP (transition)	100.0%							3.2E-39			
1,226,283	1,226,284	2	GA -> CC		11	Substitution	100.0%							4.0E-38			
1,226,286	1,226,286	1	C -> T		11	SNP (transition)	100.0%							5.0E-37			
1,226,288	1,226,289	2	TA -> CC	12 -> 13		Substitution	100.0%							2.5E-40			
1,226,295	1,226,295	1	A -> G		14	SNP (transition)	100.0%							1.6E-45			
1,226,303	1,226,303	1	C -> T		19	SNP (transition)	100.0%							1.3E-59			
1,226,338	1,226,338	1	C -> G		25	SNP (transversion)	100.0%							1.0E-75			
1,226,342	1,226,342	1	T -> C		26	SNP (transition)	100.0%							2.5E-81			
1,226,352	1,226,352	1	G -> A		30	SNP (transition)	96.7%							6.0E-95			
1,226,360	1,226,360	1	C -> T		31	SNP (transition)	96.8%							3.1E-86			
1,226,384	1,226,384	1	C -> T		37	SNP (transition)	100.0%							4.0E-119			
1,226,396	1,226,396	1	G -> A		41	SNP (transition)	100.0%							7.9E-128			
1,226,400	1,226,401	2	CT		41	Deletion	100.0%							4.0E-58			
1,226,405	1,226,406	2	AT -> GA	41 -> 42		Substitution	100.0%							1.0E-123			
1,226,416	1,226,417	2	CC -> GT		44	Substitution	97.7%							2.2E-132			
1,226,436	1,226,436	1	(T)4 -> (T)3		56	Deletion (tandem repeat)	100.0%							1.0E-84			
1,226,439	1,226,439	1	C -> G		56	SNP (transversion)	100.0%							6.3E-180			
1,226,446	1,226,446	1	G -> A		58	SNP (transition)	100.0%							2.5E-186			
1,226,450	1,226,450	1	A -> G		58	SNP (transition)	100.0%							2.5E-186			
1,226,463	1,226,463	1	C -> G		62	SNP (transversion)	98.4%							3.1E-200			
1,226,468	1,226,467	0	C		62	Insertion	100.0%							4.0E-199			
1,226,476	1,226,478	2	ACA -> GCG	65 -> 66		Substitution	98.5%	C -> R	922	TGT -> CGC	C770_RS47570	hypothetical protein	Substitution	2.5E-218	308	1	WP_015243312.1
1,226,484	1,226,484	1	A -> G		72	SNP (transition)	100.0%	W -> R	916	TGG -> CGG	C770_RS47570	hypothetical protein	Substitution	1.0E-252	306	1	WP_015243312.1
1,226,488	1,226,488	1	C -> A		73	SNP (transversion)	100.0%		912	GCG -> GCT	C770_RS47570	hypothetical protein	None	2.5E-234	304	3	WP_015243312.1
1,226,504	1,226,504	1	T -> A		78	SNP (transversion)	100.0%	Q -> L	896	CAG -> CTG	C770_RS47570	hypothetical protein	Substitution	1.0E-273	299	2	WP_015243312.1
1,226,509	1,226,509	1	C -> T		78	SNP (transition)	100.0%		891	ACG -> ACA	C770_RS47570	hypothetical protein	None	1.0E-273	297	3	WP_015243312.1
1,226,521	1,226,521	1	T -> C		79	SNP (transition)	98.7%		879	CGA -> CGG	C770_RS47570	hypothetical protein	None	2.0E-248	293	3	WP_015243312.1
1,226,593	1,226,593	1	A -> C		87	SNP (transversion)	100.0%		807	GCT -> GCG	C770_RS47570	hypothetical protein	None	7.9E-288	269	3	WP_015243312.1
1,226,596	1,226,596	1	C -> T		89	SNP (transition)	100.0%		804	CAG -> CAA	C770_RS47570	hypothetical protein	None	2.5E-303	268	3	WP_015243312.1
1,226,622	1,226,623	2	AC -> CT	90 -> 91		Substitution	100.0%	TY -> TD	777	ACG,TAC -> ACA,	C770_RS47570	hypothetical protein	Substitution	1.0E-306	259	3	WP_015243312.1
1,226,665	1,226,665	1	C -> T		98	SNP (transition)	98.0%		735	CGG -> CGA	C770_RS47570	hypothetical protein	None	2.0E-323	245	3	WP_015243312.1
1,226,673	1,226,673	1	C -> T		97	SNP (transition)	99.0%	A -> T	727	GCT -> ACT	C770_RS47570	hypothetical protein	Substitution	0.0	243	1	WP_015243312.1
1,226,795	1,226,795	1	T -> C		92	SNP (transition)	100.0%	Q -> R	605	CAA -> CGA	C770_RS47570	hypothetical protein	Substitution	1.6E-313	202	2	WP_015243312.1
1,226,914	1,226,914	1	G -> A		88	SNP (transition)	98.9%		486	GAC -> GAT	C770_RS47570	hypothetical protein	None	1.8E-268	162	3	WP_015243312.1
1,226,969	1,226,969	1	C -> A		76	SNP (transversion)	100.0%	C -> F	431	TGC -> TTC	C770_RS47570	hypothetical protein	Substitution	1.6E-251	144	2	WP_015243312.1
1,227,015	1,227,015	1	C -> T		80	SNP (transition)	98.8%	V -> I	385	GTC -> ATC	C770_RS47570	hypothetical protein	Substitution	2.5E-275	129	1	WP_015243312.1
1,227,018	1,227,018	1	T -> C		77	SNP (transition)	100.0%	S -> G	382	AGC -> GGC	C770_RS47570	hypothetical protein	Substitution	3.2E-270	128	1	WP_015243312.1
1,227,030	1,227,032	3	GAT -> CGA	73 -> 74		Substitution	98.6%	YL -> FV	368	TAT,CTC -> TTC,C	C770_RS47570	hypothetical protein	Substitution	9.3E-240	123	2	WP_015243312.1
1,227,067	1,227,067	1	C -> T		74	SNP (transition)	100.0%		333	CAG -> CAA	C770_RS47570	hypothetical protein	None	2.5E-252	111	3	WP_015243312.1
1,227,162	1,227,162	1	G -> C		78	SNP (transversion)	100.0%	R -> G	238	CGG -> GGG	C770_RS47570	hypothetical protein	Substitution	1.0E-234	80	1	WP_015243312.1
1,227,190	1,227,190	1	C -> A		81	SNP (transversion)	100.0%	M -> I	210	ATG -> ATT	C770_RS47570	hypothetical protein	Substitution	7.9E-252	70	3	WP_015243312.1
1,227,228	1,227,228	1	T -> A		83	SNP (transversion)	98.8%	T -> S	172	ACG -> TCG	C770_RS47570	hypothetical protein	Substitution	2.1E-269	58	1	WP_015243312.1
1,227,290	1,227,290	1	G -> C		75	SNP (transversion)	100.0%	A -> G	110	GCT -> GGT	C770_RS47570	hypothetical protein	Substitution	3.2E-248	37	2	WP_015243312.1
1,227,304	1,227,304	1	C -> G		76	SNP (transversion)	100.0%	M -> I	96	ATG -> ATC	C770_RS47570	hypothetical protein	Substitution	4.0E-259	32	3	WP_015243312.1
1,227,334	1,227,334	1	C -> G		77	SNP (transversion)	100.0%	L -> F	66	TTG -> TTC	C770_RS47570	hypothetical protein	Substitution	3.2E-270	22	3	WP_015243312.1
1,227,471	1,227,471	1	G -> T		83	SNP (transversion)	97.6%							2.1E-256			
1,227,473	1,227,473	1	C -> T		83	SNP (transition)	100.0%							5.0E-258			
1,227,477	1,227,476	0	(T)3 -> (T)4		83	Insertion (tandem repeat)	100.0%							2.5E-266			
1,227,482	1,227,482	1	C -> A		83	SNP (transversion)	100.0%							1.3E-274			
1,227,503	1,227,503	1	G -> A		83	SNP (transition)	100.0%							1.3E-274			
1,227,517	1,227,517	1	G -> C		88	SNP (transversion)	100.0%							4.0E-291			
1,227,532	1,227,531	0	GGTTT		85	Insertion	98.8%							1.0E-272			
1,227,602	1,227,602	1	G -> A		90	SNP (transition)	98.9%							2.8E-310			
1,227,622	1,227,622	1	C -> G		94	SNP (transversion)	97.9%							6.9E-310			
1,227,655	1,227,655	1	C -> T		94	SNP (transition)	98.9%							1.2E-305			
1,227,762	1,227,762	1	T -> A		94	SNP (transversion)	98.9%							1.2E-305			
1,227,805	1,227,805	1	C -> G		89	SNP (transversion)	97.8%							3.1E-284			
1,227,865	1,227,865	1	C -> T		81	SNP (transition)	100.0%							3.2E-284			
1,227,887	1,227,887	1	G -> C		85	SNP (transversion)	100.0%							1.0E-306			
1,227,907	1,227,907	1	G -> T		85	SNP (transversion)	98.8%							2.1E-284			
1,227,915	1,227,915	1	A -> G		88	SNP (transition)	98.9%							1.4E-294			
1,227,953	1,227,953	1	A -> T		93	SNP (transversion)	100.0%							6.3E-317			
1,227,955	1,227,955	1	C -> A		93	SNP (transversion)	100.0%							1.3E-307			
1,227,977	1,227,977	1	T -> A		96	SNP (transversion)	99.0%							3.0E-312			
1,228,029	1,228,029	1	C -> G		92	SNP (transversion)	100.0%							2.5E-304			
1,228,050	1,228,050	1	C -> G		88	SNP (transversion)	100.0%							6.3E-300			
1,228,061	1,228,061	1	C -> T		86	SNP (transition)	100.0%							2.5E-267			
1,228,084	1,228,084	1	G -> A		91	SNP (transition)	98.9%							9.1E-287			
1,228,127	1,228,127	1	T -> C		98	SNP (transition)	98.0%		744	GTA -> GTG	C770_RS47575	dehydrogenase	None	7.5E-314	248	3	WP_015243313.1
1,228,283	1,228,283	1	A -> G		89	SNP (transition)	100.0%		588	GAT -> GAC	C770_RS47575	dehydrogenase	None	3.2E-312	196	3	WP_015243313.1
1,228,376	1,228,376	1	G -> A		83	SNP (transition)	100.0%		495	GGC -> GGT	C770_RS47575	dehydrogenase	None	6.3E-283	165	3	WP_015243313.1
1,228,400	1,228,400	1	T -> C		77	SNP (transition)	100.0%		471	CCA -> CCG	C770_RS47575	dehydrogenase	None	1.6E-262	157	3	WP_015243313.1
1,228,433	1,228,433	1	G -> T		78	SNP (transversion)	100.0%		438	GCC -> GCA	C770_RS47575	dehydrogenase	None	4.0E-258	146	3	WP_015243313.1
1,228,595	1,228,595	1	G -> T		87	SNP (transversion)	98.9%		276	GCC -> GCA	C770_RS47575	dehydrogenase	None	3.5E-291	92	3	WP_015243313.1
1,228,640	1,228,640	1	C -> T		96	SNP (transition)	97.9%		231	GCG -> GCA	C770_RS47575	dehydrogenase	None	2.9E-307	77	3	WP_015243313.1
1,228,720	1,228,720	1	G -> A		96	SNP (transition)	97.9%		151	CTG -> TTG	C770_RS47575	dehydrogenase	None	7.2E-298	51	1	WP_015243313.1
1,228,736	1,228,736	1	T -> C		97	SNP (transition)	97.9%		135	GAA -> GAG	C770_RS47575	dehydrogenase	None	4.7E-320	45	3	WP_015243313.1
1,228,778	1,228,780	2	CCG -> TCC		85	Substitution	96.5%	R -> G	91	CGG -> GGA	C770_RS47575	dehydrogenase	Substitution	2.5E-266	31	1	WP_015243313.1
1,228,794	1,228,794	1	G -> A		89	SNP (transition)	97.8%	A -> V	77	GCG -> GTG	C770_RS47575	dehydrogenase	Substitution	1.6E-275	26	2	WP_015243313.1
1,228,826	1,228,826	1	A -> C		92	SNP (transversion)	98.9%		45	GCT -> GCG	C770_RS47575	dehydrogenase	None	7.3E-281	15	3	WP_015243313.1
1,228,835	1,228,835	1	T -> C		93	SNP (transition)	96.8%		36	GTA -> GTG	C770_RS47575	dehydrogenase	None	1.3E-301	12	3	WP_015243313.1
1,228,855	1,228,856	2	CA -> TT	94 -> 95		Substitution	96.8% -> 97.9%	LE -> LK	15	CTT,GAG -> CTA,	C770_RS47575	dehydrogenase	Substitution	5.5E-290	5	3	WP_015243313.1
1,228,871	1,228,871	1	T -> C		94	SNP (transition)	98.9%							2.4E-296			
1,228,879	1,228,878	0	TC		96	Insertion	99.0%							3.0E-293			
1,228,942	1,228,942	1	A -> G		84	SNP (transition)	98.8%		1.107	AAT -> AAC	C770_RS47580	L-alanine-DL-glutamate	None	1.1E-272	369	3	WP_015243314.1
1,228,990	1,228,990	1	G -> A		71	SNP (transition)	100.0%		1.059	GAC -> GAT	C770_RS47580	L-alanine-DL-glutamate	None	3.2E-249	353	3	WP_015243314.1
1,229,014	1,229,014	1	C -> G		81	SNP (transversion)	100.0%		1.035	GGG -> GGC	C770_RS47580	L-alanine-DL-glutamate	None	5.0E-268	345	3	WP_015243314.1
1,229,041	1,229,041	1	T -> G		83	SNP (transversion)	100.0%	E -> D	1.008	GAA -> GAC	C770_RS47580	L-alanine-DL-glutamate	Substitution	3.2E-291	336	3	WP_015243314.1
1,229,335	1,229,335	1	G -> A		95	SNP (transition)	100.0%		714	TAC -> TAT	C770_RS47580	L-alanine-DL-glutamate	None	1.0E-304	238	3	WP_015243314.1
1,229,347	1,229,347	1	T -> G		92	SNP (transversion)	98.9%		702	GGA -> GGC	C770_RS47580	L-alanine-DL-glutamate	None	4.6E-299	234	3	WP_015243314.1
1,229,544	1,229,544	1	G -> T		96	SNP (transversion)	100.0%	L -> I	505	CTC -> ATC	C770_RS47580	L-alanine-DL-glutamate	Substitution	0.0	169	1	WP_015243314.1
1,229,722	1,229,722	1	C -> T		103	SNP (transition)	99.0%		327	CAG -> CAA	C770_RS47580	L-alanine-DL-glutamate	None	6.5E-315	109	3	WP_015243314.1
1,229,731	1,229,731	1	G -> C		106	SNP (transversion)	100.0%		318	CTC -> CTG	C770_RS47580	L-alanine-DL-glutamate	None	1.0E-318	106	3	WP_015243314.1
1,229,736	1,229,736	1	A -> G		105	SNP (transition)	100.0%		313	TTG -> CTG	C770_RS47580	L-alanine-DL-glutamate	None	0.0	105	1	WP_015243314.1
1,229,947	1,229,947	1	C -> G		95	SNP (transversion)	100.0%		102	GTG -> GTC	C770_RS47580	L-alanine-DL-glutamate	None	1.0E-323	34	3	WP_015243314.1
1,230,077	1,230,077	1	G -> A		78	SNP (transition)	100.0%							1.0E-273			
1,230,080	1,230,080	1	A -> T		82	SNP (transversion)	100.0%							2.5E-271			
1,230,117	1,230,117	1	C -> T		82	SNP (transition)	100.0%	R -> K	746	AGG -> AAG	C770_RS47585	GntR family transcriptio	Substitution	6.3E-255	249	2	WP_017276119.1
1,230,242	1,230,242	1	A -> G		85	SNP (transition)	98.8%		621	TGT -> TGC	C770_RS47585	GntR family transcriptio	None	1.3E-267	207	3	WP_017276119.1
1,230,353	1,230,353	1	G -> A		89	SNP (transition)	96.6%		510	CAC -> CAT	C770_RS47585	GntR family transcriptio	None	4.5E-288	170	3	WP_017276119.1
1,230,961	1,230,961	1	A -> C		98	SNP (transversion)	100.0%							0.0			
1,231,204	1,231,204	1	A -> G		95	SNP (transition)	98.9%	K -> E	154	AAG -> GAG	C770_RS47590	sugar ABC transporter s	Substitution	2.4E-318	52	1	WP_015243316.1
1,231,306	1,231,306	1	G -> A		91	SNP (transition)	98.9%	V -> I	256	GTC -> ATC	C770_RS47590	sugar ABC transporter s	Substitution	9.1E-305	86	1	WP_015243316.1
1,231,317	1,231,317	1	T -> C		87	SNP (transition)	100.0%		267	CCT -> CCC	C770_RS47590	sugar ABC transporter s	None	7.9E-288	89	3	WP_015243316.1
1,231,386	1,231,386	1	T -> C		76	SNP (transition)	98.7%		336	GAT -> GAC	C770_RS47590	sugar ABC transporter s	None	7.6E-254	112	3	WP_015243316.1
1,231,584	1,231,584	1	T -> G		85	SNP (transversion)	96.5%		534	GCT -> GCG	C770_RS47590	sugar ABC transporter s	None	2.5E-266	178	3	WP_015243316.1
1,231,875	1,231,875	1	A -> C		115	SNP (transversion)	100.0%		825	ACA -> ACC	C770_RS47590	sugar ABC transporter s	None	0.0	275	3	WP_015243316.1
1,231,914	1,231,914	1	T -> C		108	SNP (transition)	99.1%		864	GCT -> GCC	C770_RS47590	sugar ABC transporter s	None	0.0	288	3	WP_015243316.1
1,231,983	1,231,983	1	C -> T		103	SNP (transition)	97.1%		933	CTC -> CTT	C770_RS47590	sugar ABC transporter s	None	0.0	311	3	WP_015243316.1
1,231,987	1,231,987	1	C -> T		104	SNP (transition)	98.1%		937	CTG -> TTG	C770_RS47590	sugar ABC transporter s	None	3.4E-313	313	1	WP_015243316.1
1,232,051	1,232,051	1	C -> T		104	SNP (transition)	99.0%							0.0			
1,232,066	1,232,066	1	C -> A		106	SNP (transversion)	99.1%							0.0			
1,232,086	1,232,086	1	C -> T		94	SNP (transition)	96.8%							6.7E-296			
1,232,149	1,232,149	1	T -> C		91	SNP (transition)	100.0%		51	GTT -> GTC	C770_RS47595	sugar ABC transporter A	None	6.3E-292	17	3	WP_015243317.1
1,232,164	1,232,164	1	A -> G		91	SNP (transition)	100.0%		66	GAA -> GAG	C770_RS47595	sugar ABC transporter A	None	6.3E-292	22	3	WP_015243317.1
1,232,287	1,232,288	2	CG -> AA	88 -> 89		Substitution	98.9%	ID -> IN	189	ATC,GAC -> ATA,	C770_RS47595	sugar ABC transporter A	Substitution	1.6E-273	63	3	WP_015243317.1
1,232,362	1,232,362	1	T -> G		92	SNP (transversion)	98.9%		264	CTT -> CTG	C770_RS47595	sugar ABC transporter A	None	4.6E-299	88	3	WP_015243317.1
1,232,474	1,232,474	1	G -> T		89	SNP (transversion)	96.6%	A -> S	376	GCC -> TCC	C770_RS47595	sugar ABC transporter A	Substitution	4.5E-288	126	1	WP_015243317.1
1,232,506	1,232,506	1	G -> A		85	SNP (transition)	100.0%		408	GCG -> GCA	C770_RS47595	sugar ABC transporter A	None	1.0E-272	136	3	WP_015243317.1
1,232,713	1,232,714	2	CT -> TC		81	Substitution	97.5%		615	CGC,TTG -> CGT,	C770_RS47595	sugar ABC transporter A	None	1.0E-273	205	3	WP_015243317.1
1,232,720	1,232,720	1	G -> A		82	SNP (transition)	100.0%	D -> N	622	GAC -> AAC	C770_RS47595	sugar ABC transporter A	Substitution	2.5E-271	208	1	WP_015243317.1
1,232,743	1,232,743	1	T -> C		81	SNP (transition)	98.8%		645	GAT -> GAC	C770_RS47595	sugar ABC transporter A	None	8.1E-279	215	3	WP_015243317.1
1,232,776	1,232,776	1	G -> A		79	SNP (transition)	100.0%		678	TCG -> TCA	C770_RS47595	sugar ABC transporter A	None	2.0E-261	226	3	WP_015243317.1
1,232,825	1,232,825	1	A -> T		82	SNP (transversion)	98.8%	T -> S	727	ACG -> TCG	C770_RS47595	sugar ABC transporter A	Substitution	8.2E-242	243	1	WP_015243317.1
1,232,839	1,232,839	1	C -> T		81	SNP (transition)	100.0%		741	GGC -> GGT	C770_RS47595	sugar ABC transporter A	None	6.3E-260	247	3	WP_015243317.1
1,232,852	1,232,855	3	CCCA -> ACGC		89	Substitution	97.8%	PI -> TL	754	CCC,ATC -> ACG,	C770_RS47595	sugar ABC transporter A	Substitution	3.1E-284	252	1	WP_015243317.1
1,232,878	1,232,878	1	T -> C		86	SNP (transition)	100.0%		780	GTT -> GTC	C770_RS47595	sugar ABC transporter A	None	4.0E-293	260	3	WP_015243317.1
1,232,882	1,232,882	1	G -> C		88	SNP (transversion)	100.0%	G -> R	784	GGC -> CGC	C770_RS47595	sugar ABC transporter A	Substitution	6.3E-300	262	1	WP_015243317.1
1,232,898	1,232,898	1	C -> A		94	SNP (transversion)	97.9%	T -> N	800	ACC -> AAC	C770_RS47595	sugar ABC transporter A	Substitution	2.8E-282	267	2	WP_015243317.1
1,232,908	1,232,908	1	A -> G		95	SNP (transition)	98.9%		810	CAA -> CAG	C770_RS47595	sugar ABC transporter A	None	2.4E-318	270	3	WP_015243317.1
1,232,928	1,232,928	1	A -> G		94	SNP (transition)	100.0%	H -> R	830	CAC -> CGC	C770_RS47595	sugar ABC transporter A	Substitution	2.5E-320	277	2	WP_015243317.1
1,232,930	1,232,932	2	ACT -> GCC	95 -> 96		Substitution	97.9%	T -> A	832	ACT -> GCC	C770_RS47595	sugar ABC transporter A	Substitution	5.6E-304	278	1	WP_015243317.1
1,232,959	1,232,959	1	G -> C		97	SNP (transversion)	97.9%		861	CTG -> CTC	C770_RS47595	sugar ABC transporter A	None	1.5E-310	287	3	WP_015243317.1
1,233,028	1,233,028	1	C -> T		100	SNP (transition)	100.0%		930	GTC -> GTT	C770_RS47595	sugar ABC transporter A	None	1.0E-310	310	3	WP_015243317.1
1,233,229	1,233,229	1	T -> C		97	SNP (transition)	95.9%		1.131	CGT -> CGC	C770_RS47595	sugar ABC transporter A	None	4.4E-301	377	3	WP_015243317.1
1,233,238	1,233,238	1	C -> G		94	SNP (transversion)	98.9%		1.14	GCC -> GCG	C770_RS47595	sugar ABC transporter A	None	1.2E-305	380	3	WP_015243317.1
1,233,265	1,233,265	1	C -> T		87	SNP (transition)	100.0%		1.167	CGC -> CGT	C770_RS47595	sugar ABC transporter A	None	1.6E-296	389	3	WP_015243317.1
1,233,340	1,233,340	1	T -> C		83	SNP (transition)	100.0%		1.242	GGT -> GGC	C770_RS47595	sugar ABC transporter A	None	3.2E-291	414	3	WP_015243317.1
1,233,972	1,233,972	1	C -> T		74	SNP (transition)	100.0%		345	TTC -> TTT	C770_RS47600	sugar ABC transporter p	None	1.6E-237	115	3	WP_015243318.1
1,233,999	1,233,999	1	G -> C		75	SNP (transversion)	97.3%		372	CTG -> CTC	C770_RS47600	sugar ABC transporter p	None	1.7E-245	124	3	WP_015243318.1
1,234,062	1,234,062	1	T -> C		89	SNP (transition)	97.8%		435	ATT -> ATC	C770_RS47600	sugar ABC transporter p	None	3.1E-284	145	3	WP_015243318.1
1,234,073	1,234,073	1	C -> T		88	SNP (transition)	100.0%	A -> V	446	GCA -> GTA	C770_RS47600	sugar ABC transporter p	Substitution	2.5E-282	149	2	WP_015243318.1
1,234,132	1,234,132	1	G -> A		81	SNP (transition)	100.0%	A -> T	505	GCT -> ACT	C770_RS47600	sugar ABC transporter p	Substitution	4.0E-276	169	1	WP_015243318.1
1,234,221	1,234,221	1	C -> A		97	SNP (transversion)	97.9%		594	TCC -> TCA	C770_RS47600	sugar ABC transporter p	None	4.7E-301	198	3	WP_015243318.1
1,234,332	1,234,332	1	A -> G		81	SNP (transition)	100.0%		705	CAA -> CAG	C770_RS47600	sugar ABC transporter p	None	3.2E-284	235	3	WP_015243318.1
1,234,383	1,234,383	1	C -> A		93	SNP (transversion)	95.7%		756	CTC -> CTA	C770_RS47600	sugar ABC transporter p	None	7.3E-297	252	3	WP_015243318.1
1,234,563	1,234,563	1	C -> T		83	SNP (transition)	100.0%		936	GCC -> GCT	C770_RS47600	sugar ABC transporter p	None	2.5E-266	312	3	WP_015243318.1
1,234,584	1,234,584	1	A -> G		88	SNP (transition)	100.0%							4.0E-291			
1,234,599	1,234,599	1	C -> A		92	SNP (transversion)	98.9%							4.6E-299			
1,234,601	1,234,601	1	C -> T		92	SNP (transition)	98.9%							3.7E-308			
1,234,667	1,234,667	1	C -> T		79	SNP (transition)	98.7%		867		C770_RS47605	aminoglycoside phosph	Truncation	2.0E-248	289	3	WP_015243319.1
1,234,676	1,234,676	1	A -> G		82	SNP (transition)	98.8%		858	AGT -> AGC	C770_RS47605	aminoglycoside phosph	None	4.1E-266	286	3	WP_015243319.1
1,234,697	1,234,697	1	G -> C		82	SNP (transversion)	100.0%		837	GGC -> GGG	C770_RS47605	aminoglycoside phosph	None	4.0E-263	279	3	WP_015243319.1
1,234,713	1,234,713	1	G -> A		77	SNP (transition)	100.0%	A -> V	821	GCT -> GTT	C770_RS47605	aminoglycoside phosph	Substitution	2.0E-239	274	2	WP_015243319.1

1,234,840	1,234,840	1	T -> A	79	SNP (transversion)	100.0%	I -> F	694	ATC -> TTC	C770_RS47605	aminoglycoside phosph	Substitution	2.0E-261	232	1	WP_015243319.1
1,234,886	1,234,886	1	C -> T	77	SNP (transition)	100.0%		648	AGG -> AGA	C770_RS47605	aminoglycoside phosph	None	7.9E-255	216	3	WP_015243319.1
1,235,051	1,235,051	1	G -> T	82	SNP (transversion)	98.8%	D -> E	483	GAC -> GAA	C770_RS47605	aminoglycoside phosph	Substitution	3.3E-274	161	3	WP_015243319.1
1,235,197	1,235,197	1	G -> C	93	SNP (transversion)	100.0%	L -> V	337	CTC -> GTC	C770_RS47605	aminoglycoside phosph	Substitution	2.5E-298	113	1	WP_015243319.1
1,235,304	1,235,308	5	GCATG	97	Deletion	100.0%		226		C770_RS47605	aminoglycoside phosph	Frame Shift	3.2E-146	76	1	WP_015243319.1
1,235,366	1,235,366	1	A -> G	96	SNP (transition)	100.0%		168	GCT -> GCC	C770_RS47605	aminoglycoside phosph	None	2.5E-298	56	3	WP_015243319.1
1,235,380	1,235,379	0	(TC)4 -> (TC)5	94	Insertion (tandem repeat)	95.7% -> 96.8%		155		C770_RS47605	aminoglycoside phosph	Frame Shift	8.4E-287	52	2	WP_015243319.1
1,235,402	1,235,402	1	G -> A	95	SNP (transition)	98.9%		132	GAC -> GAT	C770_RS47605	aminoglycoside phosph	None	2.4E-318	44	3	WP_015243319.1
1,235,493	1,235,493	1	A -> C	101	SNP (transversion)	99.0%	L -> R	41	CTG -> CGG	C770_RS47605	aminoglycoside phosph	Substitution	0.0	14	2	WP_015243319.1
1,235,537	1,235,537	1	G -> C	93	SNP (transversion)	98.9%							9.3E-321			
1,235,556	1,235,556	1	C -> G	95	SNP (transversion)	97.9%							5.6E-304			
1,235,723	1,235,723	1	G -> A	86	SNP (transition)	96.5%	A -> T	67	GCC -> ACC	C770_RS47610	sugar ABC transporter s	Substitution	2.6E-261	23	1	WP_015243320.1
1,235,836	1,235,836	1	C -> A	66	SNP (transversion)	100.0%		180	GCC -> GCA	C770_RS47610	sugar ABC transporter s	None	1.0E-231	60	3	WP_015243320.1
1,235,845	1,235,845	1	G -> A	69	SNP (transition)	98.6%		189	CAG -> CAA	C770_RS47610	sugar ABC transporter s	None	2.7E-223	63	3	WP_015243320.1
1,235,956	1,235,956	1	G -> C	67	SNP (transversion)	98.5%		300	CCG -> CCC	C770_RS47610	sugar ABC transporter s	None	2.7E-223	100	3	WP_015243320.1
1,236,040	1,236,040	1	C -> G	60	SNP (transversion)	98.3%		384	CCC -> CCG	C770_RS47610	sugar ABC transporter s	None	1.5E-199	128	3	WP_015243320.1
1,236,379	1,236,379	1	T -> C	82	SNP (transition)	98.8%		723	GGT -> GGC	C770_RS47610	sugar ABC transporter s	None	4.1E-266	241	3	WP_015243320.1
1,236,457	1,236,457	1	A -> G	87	SNP (transition)	98.9%		801	TCA -> TCG	C770_RS47610	sugar ABC transporter s	None	1.4E-282	267	3	WP_015243320.1
1,236,829	1,236,829	1	C -> T	81	SNP (transition)	98.8%	L -> F	79	CTT -> TTT	C770_RS47615	sugar ABC transporter A	Substitution	8.1E-255	27	1	WP_015243321.1
1,237,556	1,237,555	0	CCG	106	Insertion	99.1%	G -> AG	806	GGC -> GCC,GGC	C770_RS47615	sugar ABC transporter A	Insertion	0.0	269	2	WP_015243321.1
1,237,591	1,237,591	1	T -> C	105	SNP (transition)	100.0%	Y -> H	841	TAC -> CAC	C770_RS47615	sugar ABC transporter A	Substitution	0.0	281	1	WP_015243321.1
1,237,699	1,237,699	1	A -> G	100	SNP (transition)	100.0%	N -> D	949	AAC -> GAC	C770_RS47615	sugar ABC transporter A	Substitution	0.0	317	1	WP_015243321.1
1,238,086	1,238,086	1	C -> G	99	SNP (transversion)	100.0%	R -> G	1.336	CGT -> GGT	C770_RS47615	sugar ABC transporter A	Substitution	0.0	446	1	WP_015243321.1
1,238,318	1,238,318	1	G -> A	90	SNP (transition)	98.9%	D -> N	10	GAT -> AAT	C770_RS47620	sugar ABC transporter p	Substitution	2.3E-301	4	1	WP_015243322.1
1,238,371	1,238,371	1	T -> C	95	SNP (transition)	98.9%		63	GGT -> GGC	C770_RS47620	sugar ABC transporter p	None	2.4E-318	21	3	WP_015243322.1
1,238,443	1,238,443	1	C -> T	83	SNP (transition)	100.0%		135	CAC -> CAT	C770_RS47620	sugar ABC transporter p	None	1.3E-274	45	3	WP_015243322.1
1,238,446	1,238,446	1	C -> T	82	SNP (transition)	100.0%		138	TTC -> TTT	C770_RS47620	sugar ABC transporter p	None	4.0E-263	46	3	WP_015243322.1
1,238,498	1,238,498	1	G -> A	84	SNP (transition)	100.0%	V -> I	190	GTC -> ATC	C770_RS47620	sugar ABC transporter p	Substitution	6.3E-278	64	1	WP_015243322.1
1,238,593	1,238,593	1	C -> G	88	SNP (transversion)	100.0%		285	TCC -> TCG	C770_RS47620	sugar ABC transporter p	None	1.0E-308	95	3	WP_015243322.1
1,238,710	1,238,710	1	T -> C	89	SNP (transition)	98.9%		402	ATT -> ATC	C770_RS47620	sugar ABC transporter p	None	3.5E-289	134	3	WP_015243322.1
1,238,753	1,238,753	1	G -> A	87	SNP (transition)	96.6%	V -> I	445	GTC -> ATC	C770_RS47620	sugar ABC transporter p	Substitution	2.7E-281	149	1	WP_015243322.1
1,238,798	1,238,798	1	G -> A	90	SNP (transition)	100.0%	A -> T	490	GCA -> ACA	C770_RS47620	sugar ABC transporter p	Substitution	1.0E-306	164	1	WP_015243322.1
1,238,871	1,238,871	1	G -> C	97	SNP (transversion)	100.0%	G -> A	563	GGC -> GCC	C770_RS47620	sugar ABC transporter p	Substitution	4.0E-311	188	2	WP_015243322.1
1,238,923	1,238,923	1	T -> C	90	SNP (transition)	100.0%		615	TAT -> TAC	C770_RS47620	sugar ABC transporter p	None	1.0E-315	205	3	WP_015243322.1
1,238,959	1,238,959	1	C -> T	84	SNP (transition)	100.0%		651	GGC -> GGT	C770_RS47620	sugar ABC transporter p	None	1.0E-294	217	3	WP_015243322.1
1,238,995	1,238,995	1	G -> A	86	SNP (transition)	98.8%		687	GTG -> GTA	C770_RS47620	sugar ABC transporter p	None	8.6E-288	229	3	WP_015243322.1
1,239,250	1,239,250	1	G -> T	93	SNP (transversion)	97.8%		942	CTG -> CTT	C770_RS47620	sugar ABC transporter p	None	2.7E-288	314	3	WP_015243322.1
1,239,497	1,239,497	1	T -> C	93	SNP (transition)	96.8%		195	ATT -> ATC	C770_RS47625	Crp/Fnr family transcrip	None	1.3E-301	65	3	WP_015008513.1
1,239,653	1,239,653	1	G -> A	92	SNP (transition)	98.9%		351	GAG -> GAA	C770_RS47625	Crp/Fnr family transcrip	None	5.8E-290	117	3	WP_015008513.1
1,239,959	1,239,959	1	C -> G	84	SNP (transversion)	100.0%		657	GTC -> GTG	C770_RS47625	Crp/Fnr family transcrip	None	1.6E-269	219	3	WP_015008513.1
1,240,076	1,240,076	1	A -> G	98	SNP (transition)	100.0%		774	CTA -> CTG	C770_RS47625	Crp/Fnr family transcrip	None	4.9E-324	258	3	WP_015008513.1
1,240,160	1,240,160	1	C -> G	96	SNP (transversion)	100.0%		858	CCC -> CCG	C770_RS47625	Crp/Fnr family transcrip	None	0.0	286	3	WP_015008513.1
1,240,735	1,240,735	1	G -> A	78	SNP (transition)	98.7%		432	ACG -> ACA	C770_RS47630	oxidoreductase (related	None	2.5E-268	144	3	WP_015243323.1
1,240,796	1,240,796	1	G -> A	79	SNP (transition)	100.0%	V -> I	493	GTC -> ATC	C770_RS47630	oxidoreductase (related	Substitution	2.0E-261	165	1	WP_015243323.1
1,240,841	1,240,841	1	A -> C	82	SNP (transversion)	100.0%	K -> Q	538	AAG -> CAG	C770_RS47630	oxidoreductase (related	Substitution	2.5E-271	180	1	WP_015243323.1
1,240,893	1,240,893	1	G -> A	82	SNP (transition)	96.3%	G -> E	590	GGG -> GAG	C770_RS47630	oxidoreductase (related	Substitution	1.8E-256	197	2	WP_015243323.1
1,240,991	1,240,991	1	G -> C	81	SNP (transversion)	97.5%	A -> P	688	GCC -> CCC	C770_RS47630	oxidoreductase (related	Substitution	6.5E-258	230	1	WP_015243323.1
1,241,104	1,241,105	2	GC -> AT	93	Substitution	97.8%	EH -> EY	801	GAG,CAC -> GAA	C770_RS47630	oxidoreductase (related	Substitution	9.3E-275	267	3	WP_015243323.1
1,241,283	1,241,283	1	T -> C	91	SNP (transition)	96.7%	V -> A	980	GTA -> GCA	C770_RS47630	oxidoreductase (related	Substitution	7.7E-295	327	2	WP_015243323.1
1,241,448	1,241,448	1	A -> G	80	SNP (transition)	97.5%		138	AAA -> AAG	C770_RS47635	glycerol-3-phosphate de	None	2.0E-262	46	3	WP_015243324.1
1,241,553	1,241,553	1	G -> T	83	SNP (transversion)	96.4%		243	GTG -> GTT	C770_RS47635	glycerol-3-phosphate de	None	9.2E-252	81	3	WP_015243324.1
1,241,568	1,241,568	1	A -> G	82	SNP (transition)	96.3%		258	CTA -> CTG	C770_RS47635	glycerol-3-phosphate de	None	1.8E-256	86	3	WP_015243324.1
1,241,622	1,241,622	1	T -> A	72	SNP (transversion)	97.2%	F -> L	312	TTT -> TTA	C770_RS47635	glycerol-3-phosphate de	Substitution	2.6E-228	104	3	WP_015243324.1
1,241,682	1,241,682	1	A -> G	73	SNP (transition)	98.6%		372	CCA -> CCG	C770_RS47635	glycerol-3-phosphate de	None	1.8E-236	124	3	WP_015243324.1
1,241,703	1,241,703	1	A -> G	76	SNP (transition)	98.7%		393	GCA -> GCG	C770_RS47635	glycerol-3-phosphate de	None	2.4E-246	131	3	WP_015243324.1
1,241,709	1,241,709	1	A -> G	76	SNP (transition)	100.0%		399	AAA -> AAG	C770_RS47635	glycerol-3-phosphate de	None	6.3E-244	133	3	WP_015243324.1
1,241,724	1,241,724	1	T -> C	72	SNP (transition)	100.0%		414	ATT -> ATC	C770_RS47635	glycerol-3-phosphate de	None	1.6E-245	138	3	WP_015243324.1
1,241,911	1,241,911	1	C -> A	81	SNP (transversion)	98.8%	P -> T	601	CCC -> ACC	C770_RS47635	glycerol-3-phosphate de	Substitution	8.1E-263	201	1	WP_015243324.1
1,242,156	1,242,156	1	T -> C	94	SNP (transition)	100.0%		846	CTT -> CTC	C770_RS47635	glycerol-3-phosphate de	None	2.5E-320	282	3	WP_015243324.1
1,242,159	1,242,159	1	T -> C	93	SNP (transition)	100.0%		849	GGT -> GGC	C770_RS47635	glycerol-3-phosphate de	None	0.0	283	3	WP_015243324.1
1,242,189	1,242,189	1	T -> C	96	SNP (transition)	99.0%		879	GAT -> GAC	C770_RS47635	glycerol-3-phosphate de	None	9.6E-322	293	3	WP_015243324.1
1,242,207	1,242,207	1	C -> T	92	SNP (transition)	100.0%		897	CTC -> CTT	C770_RS47635	glycerol-3-phosphate de	None	2.5E-304	299	3	WP_015243324.1
1,242,288	1,242,288	1	G -> A	82	SNP (transition)	100.0%		978	GAG -> GAA	C770_RS47635	glycerol-3-phosphate de	None	2.5E-271	326	3	WP_015243324.1
1,242,333	1,242,333	1	T -> C	80	SNP (transition)	97.5%		1.023	GGT -> GGC	C770_RS47635	glycerol-3-phosphate de	None	3.2E-270	341	3	WP_015243324.1
1,242,347	1,242,347	1	A -> C	78	SNP (transversion)	100.0%	Q -> P	1.037	CAG -> CCG	C770_RS47635	glycerol-3-phosphate de	Substitution	4.0E-258	346	2	WP_015243324.1
1,242,351	1,242,351	1	C -> G	79	SNP (transversion)	100.0%	D -> E	1.041	GAC -> GAG	C770_RS47635	glycerol-3-phosphate de	Substitution	2.0E-261	347	3	WP_015243324.1
1,242,362	1,242,362	1	T -> A	80	SNP (transversion)	100.0%	F -> Y	1.052	TTC -> TAC	C770_RS47635	glycerol-3-phosphate de	Substitution	1.0E-272	351	2	WP_015243324.1
1,242,411	1,242,411	1	T -> A	87	SNP (transversion)	100.0%		1.101	ACT -> ACA	C770_RS47635	glycerol-3-phosphate de	None	1.6E-296	367	3	WP_015243324.1
1,242,414	1,242,414	1	T -> C	88	SNP (transition)	100.0%		1.104	GGT -> GGC	C770_RS47635	glycerol-3-phosphate de	None	4.0E-291	368	3	WP_015243324.1
1,242,443	1,242,443	1	C -> T	83	SNP (transition)	98.8%	T -> M	1.133	ACG -> ATG	C770_RS47635	glycerol-3-phosphate de	Substitution	1.3E-277	378	2	WP_015243324.1
1,242,471	1,242,471	1	A -> T	81	SNP (transversion)	98.8%		1.161	CTA -> CTT	C770_RS47635	glycerol-3-phosphate de	None	8.1E-279	387	3	WP_015243324.1
1,242,531	1,242,531	1	G -> T	71	SNP (transversion)	100.0%		1.221	ACG -> ACT	C770_RS47635	glycerol-3-phosphate de	None	5.0E-235	407	3	WP_015243324.1
1,242,533	1,242,533	1	C -> T	71	SNP (transition)	100.0%	A -> V	1.223	GCG -> GTG	C770_RS47635	glycerol-3-phosphate de	Substitution	4.0E-242	408	2	WP_015243324.1
1,242,553	1,242,553	1	T -> C	74	SNP (transition)	100.0%	S -> P	1.243	TCA -> CCA	C770_RS47635	glycerol-3-phosphate de	Substitution	6.3E-245	415	1	WP_015243324.1
1,242,563	1,242,563	1	T -> C	75	SNP (transition)	100.0%	V -> A	1.253	GTC -> GCC	C770_RS47635	glycerol-3-phosphate de	Substitution	1.0E-240	418	2	WP_015243324.1
1,242,611	1,242,611	1	C -> A	72	SNP (transversion)	97.2%	A -> D	1.301	GCC -> GAC	C770_RS47635	glycerol-3-phosphate de	Substitution	2.6E-221	434	2	WP_015243324.1
1,242,633	1,242,633	1	G -> A	77	SNP (transition)	97.4%		1.323	GCG -> GCA	C770_RS47635	glycerol-3-phosphate de	None	9.2E-215	441	3	WP_015243324.1
1,242,699	1,242,699	1	G -> A	74	SNP (transition)	98.6%		1.389	GAG -> GAA	C770_RS47635	glycerol-3-phosphate de	None	3.7E-225	463	3	WP_015243324.1
1,242,822	1,242,822	1	A -> G	87	SNP (transition)	97.7%		1.512	CTA -> CTG	C770_RS47635	glycerol-3-phosphate de	None	3.7E-269	504	3	WP_015243324.1
1,242,902	1,242,902	1	A -> G	84	SNP (transition)	98.8%	E -> G	1.592	GAG -> GGG	C770_RS47635	glycerol-3-phosphate de	Substitution	5.3E-281	531	2	WP_015243324.1
1,242,956	1,242,956	1	C -> A	74	SNP (transversion)	97.3%	A -> E	1.646	GCG -> GAG	C770_RS47635	glycerol-3-phosphate de	Substitution	6.8E-235	549	2	WP_015243324.1
1,243,011	1,243,011	1	G -> A	75	SNP (transition)	98.7%		1.701	CCG -> CCA	C770_RS47635	glycerol-3-phosphate de	None	1.9E-250	567	3	WP_015243324.1
1,243,061	1,243,061	1	T -> C	71	SNP (transition)	100.0%							5.0E-235			
1,243,063	1,243,063	1	C -> T	71	SNP (transition)	100.0%							5.0E-235			
1,243,070	1,243,072	3	GAA -> AGT	69 -> 70	Substitution	98.6%							6.9E-237			
1,243,077	1,243,077	1	T -> G	71	SNP (transversion)	98.6%							7.1E-230			
1,243,106	1,243,106	1	A -> CT	67	Insertion	97.0%							2.7E-223			
1,243,152	1,243,152	1	G -> C	58	SNP (transversion)	96.6%	G -> R	43	GGC -> CGC	C770_RS47640	fructose-bisphosphate a	Substitution	2.6E-182	15	1	WP_015243325.1
1,243,172	1,243,172	1	T -> C	56	SNP (transition)	98.2%		63	GTT -> GTC	C770_RS47640	fructose-bisphosphate a	None	5.6E-186	21	3	WP_015243325.1
1,243,259	1,243,259	1	G -> T	65	SNP (transversion)	96.9%		150	GCG -> GCT	C770_RS47640	fructose-bisphosphate a	None	5.2E-199	50	3	WP_015243325.1
1,243,347	1,243,347	1	A -> G	69	SNP (transition)	100.0%	I -> V	238	ATC -> GTC	C770_RS47640	fructose-bisphosphate a	Substitution	3.2E-242	80	1	WP_015243325.1
1,243,560	1,243,560	1	T -> C	94	SNP (transition)	100.0%		451	TTG -> CTG	C770_RS47640	fructose-bisphosphate a	None	1.6E-301	151	1	WP_015243325.1
1,243,691	1,243,691	1	T -> C	85	SNP (transition)	97.6%		582	GAT -> GAC	C770_RS47640	fructose-bisphosphate a	None	7.1E-238	194	3	WP_015243325.1
1,243,699	1,243,699	1	C -> A	85	SNP (transversion)	97.6%	T -> N	590	ACC -> AAC	C770_RS47640	fructose-bisphosphate a	Substitution	1.8E-254	197	2	WP_015243325.1
1,244,194	1,244,194	1	T -> G	98	SNP (transversion)	99.0%	V -> G	212	GTC -> GGC	C770_RS47645	Sugar (pentulose and he	Substitution	9.8E-290	71	2	WP_015243326.1
1,244,219	1,244,219	1	A -> C	102	SNP (transversion)	100.0%		237	GCA -> GCC	C770_RS47645	Sugar (pentulose and he	None	1.0E-306	79	3	WP_015243326.1
1,244,273	1,244,273	1	G -> C	91	SNP (transversion)	100.0%		291	GGG -> GGC	C770_RS47645	Sugar (pentulose and he	None	3.2E-319	97	3	WP_015243326.1
1,244,397	1,244,397	1	G -> T	80	SNP (transversion)	97.5%	A -> S	415	GCC -> TCC	C770_RS47645	Sugar (pentulose and he	Substitution	1.3E-254	139	1	WP_015243326.1
1,244,820	1,244,820	1	C -> T	86	SNP (transition)	100.0%	P -> S	838	CCG -> TCG	C770_RS47645	Sugar (pentulose and he	Substitution	6.3E-276	280	1	WP_015243326.1
1,245,140	1,245,140	1	C -> G	81	SNP (transversion)	98.8%	H -> Q	1.158	CAC -> CAG	C770_RS47645	Sugar (pentulose and he	Substitution	8.1E-279	386	3	WP_015243326.1
1,245,164	1,245,164	1	C -> A	77	SNP (transversion)	97.4%		1.182	GCC -> GCA	C770_RS47645	Sugar (pentulose and he	None	9.2E-245	394	3	WP_015243326.1
1,245,260	1,245,260	1	C -> T	77	SNP (transition)	96.1%		1.278	TCC -> TCT	C770_RS47645	Sugar (pentulose and he	None	1.8E-247	426	3	WP_015243326.1
1,245,310	1,245,310	1	G -> C	75	SNP (transversion)	97.3%	G -> A	1.328	GGC -> GCC	C770_RS47645	Sugar (pentulose and he	Substitution	1.7E-245	443	2	WP_015243326.1
1,245,489	1,245,489	1	G -> A	85	SNP (transition)	100.0%	V -> I	1.507	GTC -> ATC	C770_RS47645	Sugar (pentulose and he	Substitution	1.0E-289	503	1	WP_015243326.1
1,245,616	1,245,616	1	T -> C	95	SNP (transition)	100.0%		96	GAT -> GAC	C770_RS47650	choline dehydrogenase	None	1.0E-304	32	3	WP_041169903.1
1,245,634	1,245,635	2	CT -> GC	95	Substitution	100.0%		114	CTC,TTG -> CTG,(	C770_RS47650	choline dehydrogenase	None	3.2E-314	38	3	WP_041169903.1
1,245,669	1,245,669	1	T -> G	86	SNP (transversion)	100.0%	L -> W	149	TTG -> TGG	C770_RS47650	choline dehydrogenase	Substitution	6.3E-276	50	2	WP_041169903.1
1,246,253	1,246,253	1	T -> C	80	SNP (transition)	97.5%	C -> R	733	TGC -> CGC	C770_RS47650	choline dehydrogenase	Substitution	5.0E-239	245	1	WP_041169903.1
1,246,264	1,246,264	1	C -> G	81	SNP (transversion)	100.0%		744	GTC -> GTG	C770_RS47650	choline dehydrogenase	None	6.3E-260	248	3	WP_041169903.1
1,246,286	1,246,286	1	G -> A	83	SNP (transition)	100.0%	V -> I	766	GTC -> ATC	C770_RS47650	choline dehydrogenase	Substitution	6.3E-283	256	1	WP_041169903.1
1,246,435	1,246,435	1	G -> C	76	SNP (transversion)	100.0%		915	ACG -> ACC	C770_RS47650	choline dehydrogenase	None	4.0E-259	305	3	WP_041169903.1
1,246,590	1,246,590	1	C -> T	82	SNP (transition)	100.0%	A -> V	1.07	GCC -> GTC	C770_RS47650	choline dehydrogenase	Substitution	1.6E-279	357	2	WP_041169903.1
1,246,600	1,246,600	1	C -> T	84	SNP (transition)	98.8%		1.08	GCC -> GCT	C770_RS47650	choline dehydrogenase	None	5.3E-281	360	3	WP_041169903.1
1,246,603	1,246,603	1	C -> T	82	SNP (transition)	98.8%		1.083	GAC -> GAT	C770_RS47650	choline dehydrogenase	None	4.1E-266	361	3	WP_041169903.1
1,246,786	1,246,786	1	C -> T	93	SNP (transition)	98.9%		1.266	GAC -> GAT	C770_RS47650	choline dehydrogenase	None	5.9E-284	422	3	WP_041169903.1
1,246,858	1,246,858	1	A -> G	87	SNP (transition)	98.9%		1.338	AGA -> AGG	C770_RS47650	choline dehydrogenase	None	1.4E-282	446	3	WP_041169903.1
1,247,054	1,247,054	1	A -> C	93	SNP (transversion)	100.0%	S -> R	1.534	AGC -> CGC	C770_RS47650	choline dehydrogenase	Substitution	2.5E-298	512	1	WP_041169903.1
1,247,134	1,247,135	2	CC -> TA	90 -> 91	Substitution	98.9%							7.9E-283			
1,247,240	1,247,240	1	C -> T	82	SNP (transition)	98.8%		9	CGC -> CGT	C770_RS47655	hypothetical protein	None	3.3E-274	3	3	WP_041169904.1
1,247,260	1,247,260	1	G -> T	85	SNP (transversion)	100.0%	R -> L	29	CGT -> CTT	C770_RS47655	hypothetical protein	Substitution	1.0E-272	10	2	WP_041169904.1
1,247,282	1,247,282	1	G -> C	83	SNP (transversion)	100.0%		51	ACG -> ACC	C770_RS47655	hypothetical protein	None	3.2E-291	17	3	WP_041169904.1
1,247,284	1,247,283	0	A	82	Insertion	100.0%		53		C770_RS47655	hypothetical protein	Frame Shift	1.0E-287	18	2	WP_041169904.1
1,247,469	1,247,469	1	T -> G	85	SNP (transversion)	98.8%							1.3E-267			
1,247,480	1,247,480	1	G -> C	85	SNP (transversion)	98.8%							5.4E-276			
1,247,503	1,247,502	0	TT	83	Insertion	100.0%							2.5E-266			
1,247,645	1,247,645	1	C -> T	86	SNP (transition)	97.7%		1.641	GAG -> GAA	C770_RS47660	hypothetical protein	None	2.3E-274	547	3	WP_015243328.1
1,247,681	1,247,681	1	A -> G	93	SNP (transition)	98.9%		1.605	TTT -> TTC	C770_RS47660	hypothetical protein	None	1.5E-311	535	3	WP_015243328.1
1,247,743	1,247,743	1	T -> C	95	SNP (transition)	98.9%	R -> G	1.543	AGG -> GGG	C770_RS47660	hypothetical protein	Substitution	3.8E-290	515	1	WP_015243328.1
1,247,854	1,247,854	1	C -> A	101	SNP (transversion)	97.0%	A -> S	1.432	GCG -> TCG	C770_RS47660	hypothetical protein	Substitution	4.2E-309	478	1	WP_015243328.1
1,247,981	1,247,981	1	T -> G	87	SNP (transversion)	100.0%		1.305	TCA -> TCC	C770_RS47660	hypothetical protein	None	4.0E-279	435	3	WP_015243328.1
1,248,005	1,248,005	1	A -> C	93	SNP (transversion)	100.0%		1.281	GCT -> GCG	C770_RS47660	hypothetical protein	None	1.3E-307	427	3	WP_015243328.1
1,248,032	1,248,032	1	C -> G	91	SNP (transversion)	98.9%	E -> D	1.254	GAG -> GAC	C770_RS47660	hypothetical protein	Substitution	9.1E-305	418	3	WP_015243328.1
1,248,043	1,248,043	1	G -> C	87	SNP (transversion)	100.0%	R -> G	1.243	CGG -> GGG	C770_RS47660	hypothetical protein	Substitution	4.0E-279	415	1	WP_015243328.1
1,248,220	1,248,220	1	T -> C	84	SNP (transition)	100.0%	I -> V	1.066	ATT -> GTT	C770_RS47660	hypothetical protein	Substitution	2.5E-286	356	1	WP_015243328.1
1,248,609	1,248,609	1	G -> C	89	SNP (transversion)	100.0%	A -> G	677	GCG -> GGG	C770_RS47660	hypothetical protein	Substitution	1.6E-285	226	2	WP_015243328.1
1,248,697	1,248,697	1	G -> T	94	SNP (transversion)	98.9%	L -> I	589	CTC -> ATC	C770_RS47660	hypothetical protein	Substitution	1.2E-305	197	1	WP_015243328.1
1,248,739	1,248,739	1	T -> C	91	SNP (transition)	98.9%	T -> A	547	ACC -> GCC	C770_RS47660	hypothetical protein	Substitution	9.1E-287	183	1	WP_015243328.1
1,248,803	1,248,803	1	T -> G	87	SNP (transversion)	100.0%		483	GCA -> GCC	C770_RS47660	hypothetical protein	None	7.9E-288	161	3	WP_015243328.1
1,248,833	1,248,833	1	G -> A	89	SNP (transition)	100.0%		453	AAC -> AAT	C770_RS47660	hypothetical protein	None	2.5E-303	151	3	WP_015243328.1
1,248,970	1,248,970	1	C -> T	96	SNP (transition)	99.0%	E -> K	316	GAG -> AAG	C770_RS47660	hypothetical protein	Substitution	3.0E-312	106	1	WP_015243328.1
1,248,989	1,248,989	1	G -> C	96	SNP (transversion)	100.0%		297	CGC -> CGG	C770_RS47660	hypothetical protein	None	2.5E-298	99	3	WP_015243328.1
1,248,992	1,248,992	1	G -> C	94	SNP (transversion)	98.9%		294	GCC -> GCG	C770_RS47660	hypothetical protein	None	1.2E-305	98	3	WP_015243328.1
1,249,013	1,249,013	1	A -> C	96	SNP (transversion)	99.0%		273	GGT -> GGG	C770_RS47660	hypothetical protein	None	9.6E-303	91	3	WP_015243328.1
1,249,034	1,249,034	1	T -> C	97	SNP (transition)	100.0%		252	CAA -> CAG	C770_RS47660	hypothetical protein	None	2.0E-301	84	3	WP_015243328.1
1,249,055	1,249,055	1	T -> A	93	SNP (transversion)	98.9%	E -> D	231	GAA -> GAT	C770_RS47660	hypothetical protein	Substitution	1.5E-311	77	3	WP_015243328.1
1,249,058	1,249,058	1	C -> T	93	SNP (transition)	100.0%		228	AAG -> AAA	C770_RS47660	hypothetical protein	None	1.3E-307	76	3	WP_015243328.1
1,249,077	1,249,078	2	AA -> GC	95	Substitution	100.0%	F -> A	208	TTC -> GCC	C770_RS47660	hypothetical protein	Substitution	3.2E-295	70	1	WP_015243328.1
1,249,082	1,249,082	1	A -> G	95	SNP (transition)	100.0%		204	CGT -> CGC	C770_RS47660	hypothetical protein	None	3.2E-314	68	3	WP_015243328.1
1,249,236	1,249,236	1	C -> G	80	SNP (transversion)	97.5%	G -> A	50	GGG -> GCG	C770_RS47660	hypothetical protein	Substitution	3.2E-270	17	2	WP_015243328.1
1,249,246	1,249,246	1	G -> T	83	SNP (transversion)	98.8%	L -> M	40	CTG -> ATG	C770_RS47660	hypothetical protein	Substitution	5.2E-294	14	1	WP_015243328.1
1,249,300	1,249,300	1	G -> T	85	SNP (transversion)	100.0%							3.2E-281			
1,249,620	1,249,620	1	A -> C	89	SNP (transversion)	100.0%							2.0E-294			
1,249,689	1,249,689	1	C -> T	87	SNP (transition)	98.9%							1.4E-282			
1,249,701	1,249,700	0	C	84	Insertion	98.8%							2.1E-264			
1,249,795	1,249,795	1	C -> T	83	SNP (transition)	100.0%							6.3E-283			
1,249,808	1,249,808	1	G -> C	82	SNP (transversion)	100.0%							1.6E-279			
1,249,819	1,249,819	1	G -> A	83	SNP (transition)	98.8%							2.1E-269			
1,249,860	1,249,859	0	C	77	Insertion	98.7%							1.2E-249			
1,249,892	1,249,892	1	C -> A	78	SNP (transversion)	96.2%							2.4E-258			
1,249,951	1,249,951	1	C -> T	85	SNP (transition)	100.0%							3.2E-281			

1,249,985	1,249,985	1	C -> T		85	SNP (transition)	100.0%							3.2E-281			
1,249,999	1,249,999	1	C -> A		89	SNP (transversion)	100.0%							2.0E-294			
1,250,128	1,250,128	1	C		78	Deletion	100.0%							1.0E-117			
1,250,538	1,250,538	1	G -> A		95	SNP (transition)	96.8%		243	AGC -> AGT	C770_RS47670	transcriptional regulato	None	3.5E-299	81	3	WP_041169933.1
1,250,574	1,250,574	1	C -> T		90	SNP (transition)	98.9%		207	CGG -> CGA	C770_RS47670	transcriptional regulato	None	1.1E-274	69	3	WP_041169933.1
1,250,708	1,250,708	1	T -> G		48	SNP (transversion)	100.0%	K -> Q	73	AAG -> CAG	C770_RS47670	transcriptional regulato	Substitution	6.3E-164	25	1	WP_041169933.1
1,250,780	1,250,780	1	A		18	Deletion	100.0%		1		C770_RS47670	transcriptional regulato	Frame Shift	6.3E-26	1	1	WP_041169933.1
1,250,781	1,250,781	1	G		18	Deletion	100.0%							6.3E-26			
1,250,785	1,250,786	2	GT -> CG	16 -> 17		Substitution	100.0%							6.3E-52			
1,250,791	1,250,791	1	T -> A		14	SNP (transversion)	100.0%							1.0E-42			
1,250,794	1,250,795	2	AA -> CG	12 -> 13		Substitution	100.0%							1.0E-36			
1,250,800	1,250,802	3	AAT -> CGG	9 -> 11		Substitution	100.0%							1.3E-28			
1,250,805	1,250,805	1	G -> T		9	SNP (transversion)	100.0%							6.3E-26			
1,250,807	1,250,807	1	G -> T		9	SNP (transversion)	100.0%							7.9E-27			
1,250,809	1,250,811	3	GAG -> CCA		9	Substitution	100.0%							7.9E-27			
1,250,815	1,250,819	5	TCCTT -> CGGCG		9	Substitution	100.0%							2.5E-22			
1,250,822	1,250,822	1	C -> G		8	SNP (transversion)	100.0%							1.6E-25			
1,250,825	1,250,827	3	AGC -> TCT		7	Substitution	100.0%							2.0E-22			
1,251,880	1,251,880	1	C -> T		23	SNP (transition)	100.0%	V -> I	208	GTC -> ATC	C770_RS47685	transcriptional regulato	Substitution	6.3E-79	70	1	WP_015243333.1
1,252,088	1,252,088	1	G -> A		107	SNP (transition)	99.1%							0.0			
1,252,142	1,252,143	2	CT -> TC	116 -> 117		Substitution	98.3% -> 99.1%							0.0			
1,252,281	1,252,281	1	A -> G		115	SNP (transition)	100.0%		72	CTA -> CTG	C770_RS47690	3-ketoacyl-ACP reducta:	None	0.0	24	3	WP_010975354.1
1,252,409	1,252,409	1	C -> T		98	SNP (transition)	99.0%	A -> V	200	GCG -> GTG	C770_RS47690	3-ketoacyl-ACP reducta:	Substitution	0.0	67	2	WP_010975354.1
1,252,428	1,252,428	1	T -> C		100	SNP (transition)	99.0%		219	TTT -> TTC	C770_RS47690	3-ketoacyl-ACP reducta:	None	0.0	73	3	WP_010975354.1
1,252,455	1,252,455	1	A -> T		98	SNP (transversion)	99.0%		246	GGA -> GGT	C770_RS47690	3-ketoacyl-ACP reducta:	None	7.8E-319	82	3	WP_010975354.1
1,252,468	1,252,468	1	G -> A		99	SNP (transition)	98.0%	V -> I	259	GTC -> ATC	C770_RS47690	3-ketoacyl-ACP reducta:	Substitution	3.8E-317	87	1	WP_010975354.1
1,252,523	1,252,523	1	T -> C		87	SNP (transition)	100.0%	V -> A	314	GTC -> GCC	C770_RS47690	3-ketoacyl-ACP reducta:	Substitution	7.9E-288	105	2	WP_010975354.1
1,252,539	1,252,539	1	A -> C		87	SNP (transversion)	100.0%		330	ATA -> ATC	C770_RS47690	3-ketoacyl-ACP reducta:	None	1.6E-296	110	3	WP_010975354.1
1,252,674	1,252,674	1	T -> G		97	SNP (transversion)	100.0%		465	GCT -> GCG	C770_RS47690	3-ketoacyl-ACP reducta:	None	0.0	155	3	WP_010975354.1
1,252,827	1,252,827	1	G -> C		104	SNP (transversion)	99.0%		618	GCG -> GCC	C770_RS47690	3-ketoacyl-ACP reducta:	None	0.0	206	3	WP_010975354.1
1,252,867	1,252,867	1	G -> A		98	SNP (transition)	100.0%	A -> T	658	GCC -> ACC	C770_RS47690	3-ketoacyl-ACP reducta:	Substitution	0.0	220	1	WP_010975354.1
1,252,881	1,252,881	1	T -> C		100	SNP (transition)	100.0%		672	CTT -> CTC	C770_RS47690	3-ketoacyl-ACP reducta:	None	0.0	224	3	WP_010975354.1
1,252,993	1,252,992	0	(G)2 -> (G)3		97	Insertion (tandem repeat)	100.0%							2.0E-301			
1,253,008	1,253,008	1	A -> G		95	SNP (transition)	100.0%							3.2E-314			
1,253,135	1,253,135	1	T -> C		95	SNP (transition)	100.0%		78	GCT -> GCC	C770_RS47695	oxidoreductase	None	1.0E-304	26	3	WP_010975353.1
1,253,214	1,253,214	1	A -> G		87	SNP (transition)	98.9%	R -> G	157	AGG -> GGG	C770_RS47695	oxidoreductase	Substitution	5.5E-274	53	1	WP_010975353.1
1,253,525	1,253,525	1	G -> C		112	SNP (transversion)	100.0%		468	GCG -> GCC	C770_RS47695	oxidoreductase	None	0.0	156	3	WP_010975353.1
1,253,876	1,253,876	1	T -> A		83	SNP (transversion)	100.0%		819	GGT -> GGA	C770_RS47695	oxidoreductase	None	1.3E-274	273	3	WP_010975353.1
1,253,936	1,253,936	1	T -> A		79	SNP (transversion)	100.0%		879	CTT -> CTA	C770_RS47695	oxidoreductase	None	1.6E-253	293	3	WP_010975353.1
1,254,012	1,254,012	1	C -> G		75	SNP (transversion)	100.0%							1.0E-240			
1,254,028	1,254,028	1	G -> C		75	SNP (transversion)	100.0%							1.0E-240			
1,255,062	1,255,062	1	T -> C		12	SNP (transition)	100.0%							4.0E-27			
1,255,115	1,255,115	1	T -> C		22	SNP (transition)	100.0%							2.5E-73			
1,255,200	1,255,200	1	T -> C		45	SNP (transition)	100.0%		27	AGT -> AGC	C770_RS47705	hypothetical protein	None	3.2E-158	9	3	WP_015243335.1
1,255,475	1,255,475	1	A -> C		90	SNP (transversion)	100.0%	E -> A	302	GAA -> GCA	C770_RS47705	hypothetical protein	Substitution	1.0E-306	101	2	WP_015243335.1
1,255,524	1,255,524	1	G -> C		96	SNP (transversion)	99.0%		351	CTG -> CTC	C770_RS47705	hypothetical protein	None	9.6E-322	117	3	WP_015243335.1
1,255,575	1,255,575	1	T -> C		90	SNP (transition)	98.9%		402	ATT -> ATC	C770_RS47705	hypothetical protein	None	1.8E-292	134	3	WP_015243335.1
1,255,878	1,255,878	1	T -> G		101	SNP (transversion)	100.0%		705	CCT -> CCG	C770_RS47705	hypothetical protein	None	0.0	235	3	WP_015243335.1
1,255,958	1,255,958	1	G -> C		94	SNP (transversion)	98.9%							2.4E-296			
1,255,961	1,255,961	1	A -> G		94	SNP (transition)	100.0%							2.5E-320			
1,255,967	1,255,968	2	GT -> AG		93	Substitution	98.9%							3.7E-293			
1,255,972	1,255,972	1	C -> A		94	SNP (transversion)	95.7%							3.0E-291			
1,255,997	1,255,997	1	C -> G		95	SNP (transversion)	100.0%							3.2E-295			
1,256,011	1,256,010	0	(C)4 -> (C)5		95	Insertion (tandem repeat)	100.0%							3.2E-295			
1,256,283	1,256,283	1	G -> C		69	SNP (transversion)	100.0%	L -> V	436	CTC -> GTC	C770_RS47710	Ribulose-5-phosphate 4	Substitution	2.5E-235	146	1	WP_015243336.1
1,256,341	1,256,341	1	T -> C		64	SNP (transition)	95.3%		378	ACA -> ACG	C770_RS47710	Ribulose-5-phosphate 4	None	2.1E-197	126	3	WP_015243336.1
1,256,549	1,256,549	1	G -> T		55	SNP (transversion)	98.2%	P -> Q	170	CCG -> CAG	C770_RS47710	Ribulose-5-phosphate 4	Substitution	1.4E-182	57	2	WP_015243336.1
1,256,656	1,256,656	1	C -> A		76	SNP (transversion)	97.4%		63	CGG -> CGT	C770_RS47710	Ribulose-5-phosphate 4	None	7.2E-249	21	3	WP_015243336.1
1,256,712	1,256,712	1	C -> T		71	SNP (transition)	97.2%	E -> K	7	GAG -> AAG	C770_RS47710	Ribulose-5-phosphate 4	Substitution	7.9E-239	3	1	WP_015243336.1
1,256,712	1,256,712	1	C -> T		71	SNP (transition)	97.2%		1.472	TGA -> TAA	C770_RS47715	Sugar (pentulose and he	None	7.9E-239	491	2	WP_015243337.1
1,256,715	1,256,715	1	C -> A		72	SNP (transversion)	97.2%	A -> S	4	GCT -> TCT	C770_RS47710	Ribulose-5-phosphate 4	Substitution	2.6E-242	2	1	WP_015243336.1
1,256,715	1,256,715	1	C -> A		72	SNP (transversion)	97.2%	G -> V	1.469	GGC -> GTC	C770_RS47715	Sugar (pentulose and he	Substitution	2.6E-242	490	2	WP_015243337.1
1,256,987	1,256,987	1	T -> C		92	SNP (transition)	98.9%		1.197	CTA -> CTG	C770_RS47715	Sugar (pentulose and he	None	3.7E-308	399	3	WP_015243337.1
1,257,105	1,257,105	1	T -> G		87	SNP (transversion)	100.0%	D -> A	1.079	GAT -> GCT	C770_RS47715	Sugar (pentulose and he	Substitution	3.2E-305	360	2	WP_015243337.1
1,257,197	1,257,197	1	T -> C		91	SNP (transition)	100.0%		987	CTA -> CTG	C770_RS47715	Sugar (pentulose and he	None	5.0E-301	329	3	WP_015243337.1
1,257,332	1,257,332	1	C -> A		103	SNP (transversion)	97.1%		852	CGG -> CGT	C770_RS47715	Sugar (pentulose and he	None	0.0	284	3	WP_015243337.1
1,257,347	1,257,347	1	A -> G		106	SNP (transition)	100.0%		837	CGT -> CGC	C770_RS47715	Sugar (pentulose and he	None	0.0	279	3	WP_015243337.1
1,257,356	1,257,356	1	T -> C		105	SNP (transition)	100.0%		828	CCA -> CCG	C770_RS47715	Sugar (pentulose and he	None	0.0	276	3	WP_015243337.1
1,257,525	1,257,527	3	CAT -> GGA	113 -> 114		Substitution	97.3% -> 97.4%	GW -> GP	657	GGA,TGG -> GGT	C770_RS47715	Sugar (pentulose and he	Substitution	0.0	219	3	WP_015243337.1
1,257,545	1,257,545	1	C -> T		103	SNP (transition)	99.0%		639	AAG -> AAA	C770_RS47715	Sugar (pentulose and he	None	6.5E-315	213	3	WP_015243337.1
1,257,550	1,257,551	2	AG -> CA		103	Substitution	100.0%	FS -> FA	633	TTC,TCG -> TTT,(	C770_RS47715	Sugar (pentulose and he	Substitution	1.0E-309	211	3	WP_015243337.1
1,257,554	1,257,554	1	T -> G		105	SNP (transversion)	99.0%		630	CCA -> CCC	C770_RS47715	Sugar (pentulose and he	None	4.2E-321	210	3	WP_015243337.1
1,257,575	1,257,575	1	G -> A		106	SNP (transition)	100.0%		609	GGC -> GGT	C770_RS47715	Sugar (pentulose and he	None	1.0E-318	203	3	WP_015243337.1
1,257,593	1,257,593	1	C -> G		102	SNP (transversion)	100.0%		591	CCG -> CCC	C770_RS47715	Sugar (pentulose and he	None	6.3E-317	197	3	WP_015243337.1
1,257,659	1,257,659	1	C -> G		101	SNP (transversion)	100.0%		525	GCG -> GCC	C770_RS47715	Sugar (pentulose and he	None	4.9E-324	175	3	WP_015243337.1
1,257,668	1,257,668	1	G -> A		98	SNP (transition)	98.0%		516	GGC -> GGT	C770_RS47715	Sugar (pentulose and he	None	2.0E-323	172	3	WP_015243337.1
1,257,677	1,257,677	1	C -> G		96	SNP (transversion)	100.0%		507	CGG -> CGC	C770_RS47715	Sugar (pentulose and he	None	1.6E-317	169	3	WP_015243337.1
1,257,683	1,257,683	1	G -> A		97	SNP (transition)	100.0%		501	GCC -> GCT	C770_RS47715	Sugar (pentulose and he	None	7.9E-321	167	3	WP_015243337.1
1,257,716	1,257,718	2	CGC -> GGA		98	Substitution	98.0%	A -> S	466	GCG -> TCC	C770_RS47715	Sugar (pentulose and he	Substitution	1.6E-317	156	1	WP_015243337.1
1,257,725	1,257,725	1	G -> A		97	SNP (transition)	97.9%		459	TTC -> TTT	C770_RS47715	Sugar (pentulose and he	None	4.7E-301	153	3	WP_015243337.1
1,257,755	1,257,755	1	A -> G		88	SNP (transition)	100.0%		429	TAT -> TAC	C770_RS47715	Sugar (pentulose and he	None	6.3E-300	143	3	WP_015243337.1
1,257,761	1,257,761	1	T -> C		88	SNP (transition)	100.0%		423	CAA -> CAG	C770_RS47715	Sugar (pentulose and he	None	4.0E-291	141	3	WP_015243337.1
1,257,854	1,257,854	1	A -> T		94	SNP (transversion)	96.8%		330	CCT -> CCA	C770_RS47715	Sugar (pentulose and he	None	8.4E-287	110	3	WP_015243337.1
1,257,890	1,257,890	1	G -> A		92	SNP (transition)	97.8%		294	GCC -> GCT	C770_RS47715	Sugar (pentulose and he	None	4.2E-276	98	3	WP_015243337.1
1,257,980	1,257,980	1	C -> T		84	SNP (transition)	100.0%		204	TCG -> TCA	C770_RS47715	Sugar (pentulose and he	None	6.3E-278	68	3	WP_015243337.1
1,257,987	1,257,987	1	A -> G		84	SNP (transition)	100.0%	V -> A	197	GTT -> GCT	C770_RS47715	Sugar (pentulose and he	Substitution	6.3E-278	66	2	WP_015243337.1
1,257,997	1,257,997	1	T -> C		85	SNP (transition)	100.0%	T -> A	187	ACG -> GCG	C770_RS47715	Sugar (pentulose and he	Substitution	3.2E-281	63	1	WP_015243337.1
1,258,011	1,258,011	1	C -> T		85	SNP (transition)	97.6%	G -> E	173	GGA -> GAA	C770_RS47715	Sugar (pentulose and he	Substitution	1.8E-254	58	2	WP_015243337.1
1,258,015	1,258,016	2	CC -> TT	83 -> 84		Substitution	98.8%	LG -> LS	168	CTG,GGC -> CTA,	C770_RS47715	Sugar (pentulose and he	Substitution	2.1E-264	56	3	WP_015243337.1
1,258,028	1,258,028	1	G -> C		87	SNP (transversion)	100.0%	D -> E	156	GAC -> GAG	C770_RS47715	Sugar (pentulose and he	Substitution	1.6E-296	52	3	WP_015243337.1
1,258,062	1,258,062	1	C -> G		102	SNP (transversion)	100.0%	R -> P	122	CGC -> CCC	C770_RS47715	Sugar (pentulose and he	Substitution	0.0	41	2	WP_015243337.1
1,258,149	1,258,149	1	G -> C		87	SNP (transversion)	100.0%	A -> G	35	GCG -> GGG	C770_RS47715	Sugar (pentulose and he	Substitution	7.9E-288	12	2	WP_015243337.1
1,258,151	1,258,151	1	G -> T		87	SNP (transversion)	100.0%		33	GTC -> GTA	C770_RS47715	Sugar (pentulose and he	None	1.6E-296	11	3	WP_015243337.1
1,258,159	1,258,160	2	CC -> TT	83 -> 84		Substitution	100.0%	AA -> AT	24	GCG,GCT -> GCA	C770_RS47715	Sugar (pentulose and he	Substitution	2.5E-266	8	3	WP_015243337.1
1,258,163	1,258,163	1	A -> C		84	SNP (transversion)	100.0%		21	GGT -> GGG	C770_RS47715	Sugar (pentulose and he	None	6.3E-278	7	3	WP_015243337.1
1,258,188	1,258,188	1	A -> T		79	SNP (transversion)	97.5%							4.9E-259			
1,258,207	1,258,207	1	A -> G		79	SNP (transition)	100.0%							2.5E-269			
1,258,363	1,258,363	1	T -> C		74	SNP (transition)	100.0%		714	GAA -> GAG	C770_RS47720	Sugar phosphate isome	None	6.3E-245	238	3	WP_015243338.1
1,258,369	1,258,369	1	G -> C		74	SNP (transversion)	97.3%		708	GCC -> GCG	C770_RS47720	Sugar phosphate isome	None	4.3E-242	236	3	WP_015243338.1
1,258,372	1,258,372	1	G -> A		74	SNP (transition)	98.6%		705	CTC -> CTT	C770_RS47720	Sugar phosphate isome	None	9.3E-240	235	3	WP_015243338.1
1,258,382	1,258,382	1	T -> C		76	SNP (transition)	100.0%	K -> R	695	AAG -> AGG	C770_RS47720	Sugar phosphate isome	Substitution	2.5E-236	232	2	WP_015243338.1
1,258,396	1,258,396	1	C -> T		76	SNP (transition)	100.0%		681	GCG -> GCA	C770_RS47720	Sugar phosphate isome	None	1.6E-251	227	3	WP_015243338.1
1,259,805	1,259,805	1	G -> A		93	SNP (transition)	98.9%		273	AAC -> AAT	C770_RS47725	sugar ABC transporter p	None	2.3E-302	91	3	WP_010975347.1
1,259,844	1,259,844	1	G -> A		93	SNP (transition)	100.0%		234	AAC -> AAT	C770_RS47725	sugar ABC transporter p	None	2.5E-298	78	3	WP_010975347.1
1,259,880	1,259,880	1	C -> T		95	SNP (transition)	96.8%		198	ACG -> ACA	C770_RS47725	sugar ABC transporter p	None	3.5E-299	66	3	WP_010975347.1
1,259,937	1,259,937	1	G -> C		101	SNP (transversion)	99.0%		141	GCC -> GCG	C770_RS47725	sugar ABC transporter p	None	0.0	47	3	WP_010975347.1
1,260,374	1,260,374	1	G -> A		83	SNP (transition)	97.6%		702	CTC -> CTT	C770_RS47730	sugar ABC transporter p	None	2.7E-248	234	3	WP_014527450.1
1,260,418	1,260,418	1	T -> C		88	SNP (transition)	98.9%	I -> V	658	ATT -> GTT	C770_RS47730	sugar ABC transporter p	Substitution	1.4E-294	220	1	WP_014527450.1
1,260,443	1,260,443	1	C -> G		86	SNP (transversion)	98.8%		633	GGG -> GGC	C770_RS47730	sugar ABC transporter p	None	8.6E-288	211	3	WP_014527450.1
1,260,710	1,260,710	1	A -> G		87	SNP (transition)	97.7%		366	GTT -> GTC	C770_RS47730	sugar ABC transporter p	None	3.7E-286	122	3	WP_014527450.1
1,260,775	1,260,775	1	A -> G		92	SNP (transition)	98.9%		301	TTG -> CTG	C770_RS47730	sugar ABC transporter p	None	5.8E-290	101	1	WP_014527450.1
1,261,080	1,261,080	1	T -> G		88	SNP (transversion)	96.6%		1.518	GCA -> GCC	C770_RS47735	ABC-type sugar transpo	None	1.1E-267	506	3	WP_015243339.1
1,261,215	1,261,215	1	C -> G		102	SNP (transversion)	96.1%		1.383	GGG -> GGC	C770_RS47735	ABC-type sugar transpo	None	6.7E-298	461	3	WP_015243339.1
1,261,380	1,261,380	1	A -> G		84	SNP (transition)	97.6%		1.218	GCT -> GCC	C770_RS47735	ABC-type sugar transpo	None	1.4E-259	406	3	WP_015243339.1
1,261,419	1,261,419	1	C -> G		91	SNP (transversion)	97.8%		1.179	CGG -> CGC	C770_RS47735	ABC-type sugar transpo	None	8.2E-291	393	3	WP_015243339.1
1,261,436	1,261,436	1	G -> T		86	SNP (transversion)	98.8%		1.162	CGA -> AGA	C770_RS47735	ABC-type sugar transpo	None	2.7E-262	388	1	WP_015243339.1
1,261,458	1,261,458	1	A -> C		82	SNP (transversion)	100.0%		1.14	GGT -> GGG	C770_RS47735	ABC-type sugar transpo	None	4.0E-263	380	3	WP_015243339.1
1,261,464	1,261,464	1	T -> G		80	SNP (transversion)	100.0%		1.134	TCA -> TCC	C770_RS47735	ABC-type sugar transpo	None	1.0E-248	378	3	WP_015243339.1
1,261,476	1,261,476	1	G -> T		78	SNP (transversion)	97.4%		1.122	CGC -> CGA	C770_RS47735	ABC-type sugar transpo	None	7.5E-233	374	3	WP_015243339.1
1,261,494	1,261,494	1	G -> C		75	SNP (transversion)	98.7%		1.104	CTC -> CTG	C770_RS47735	ABC-type sugar transpo	None	3.0E-228	368	3	WP_015243339.1
1,261,527	1,261,527	1	T -> C		68	SNP (transition)	100.0%		1.071	GGA -> GGG	C770_RS47735	ABC-type sugar transpo	None	4.0E-225	357	3	WP_015243339.1
1,261,557	1,261,557	1	T -> G		64	SNP (transversion)	98.4%		1.041	GCA -> GCC	C770_RS47735	ABC-type sugar transpo	None	2.0E-219	347	3	WP_015243339.1
1,261,590	1,261,590	1	G -> A		69	SNP (transition)	98.6%		1.008	GGC -> GGT	C770_RS47735	ABC-type sugar transpo	None	1.1E-209	336	3	WP_015243339.1
1,261,616	1,261,616	1	T -> G		68	SNP (transversion)	98.5%	I -> L	982	ATC -> CTC	C770_RS47735	ABC-type sugar transpo	Substitution	1.1E-226	328	1	WP_015243339.1
1,261,629	1,261,631	2	GGT -> CGA	71 -> 73		Substitution	95.8% -> 95.9%	T -> S	967	ACC -> TCG	C770_RS47735	ABC-type sugar transpo	Substitution	2.5E-218	323	1	WP_015243339.1
1,261,667	1,261,668	2	CT -> GC		71	Substitution	100.0%	EV -> EL	930	GAA,GTG -> GAG	C770_RS47735	ABC-type sugar transpo	Substitution	6.3E-228	310	3	WP_015243339.1
1,261,728	1,261,730	2	AAA -> CAG		72	Substitution	97.2%	F -> L	868	TTT -> CTG	C770_RS47735	ABC-type sugar transpo	Substitution	2.6E-214	290	1	WP_015243339.1
1,261,740	1,261,740	1	C -> T		71	SNP (transition)	100.0%		858	GAG -> GAA	C770_RS47735	ABC-type sugar transpo	None	1.0E-213	286	3	WP_015243339.1
1,261,743	1,261,743	1	A -> G		70	SNP (transition)	98.6%		855	TTT -> TTC	C770_RS47735	ABC-type sugar transpo	None	1.1E-219	285	3	WP_015243339.1
1,261,746	1,261,748	2	CTG -> GTC		69	Substitution	100.0%	Q -> D	850	CAG -> GAC	C770_RS47735	ABC-type sugar transpo	Substitution	1.3E-214	284	1	WP_015243339.1
1,261,752	1,261,752	1	T -> C		69	SNP (transition)	98.6%		846	CCA -> CCG	C770_RS47735	ABC-type sugar transpo	None	1.7E-216	282	3	WP_015243339.1
1,261,758	1,261,758	1	G -> C		71	SNP (transversion)	100.0%		840	ACC -> ACG	C770_RS47735	ABC-type sugar transpo	None	5.0E-235	280	3	WP_015243339.1
1,261,761	1,261,761	1	G -> C		72	SNP (transversion)	100.0%		837	CTC -> CTG	C770_RS47735	ABC-type sugar transpo	None	4.0E-231	279	3	WP_015243339.1
1,261,764	1,261,765	2	CG -> GC	72 -> 73		Substitution	100.0%	A -> G	833	GCG -> GGC	C770_RS47735	ABC-type sugar transpo	Substitution	6.3E-224	278	2	WP_015243339.1
1,261,770	1,261,770	1	C -> A		77	SNP (transversion)	98.7%		828	GTG -> GTT	C770_RS47735	ABC-type sugar transpo	None	7.7E-227	276	3	WP_015243339.1
1,261,773	1,261,773	1	G -> C		78	SNP (transversion)	100.0%	D -> E	825	GAC -> GAG	C770_RS47735	ABC-type sugar transpo	Substitution	2.5E-250	275	3	WP_015243339.1
1,261,779	1,261,779	1	C -> A		80	SNP (transversion)	98.8%		819	GTG -> GTT	C770_RS47735	ABC-type sugar transpo	None	8.0E-236	273	3	WP_015243339.1
1,261,783	1,261,783	1	A -> G		82	SNP (transition)	100.0%	V -> A	815	GTC -> GCC	C770_RS47735	ABC-type sugar transpo	Substitution	4.0E-263	272	2	WP_015243339.1
1,261,791	1,261,791	1	A -> G		84	SNP (transition)	98.8%		807	GAT -> GAC	C770_RS47735	ABC-type sugar transpo	None	8.4E-248	269	3	WP_015243339.1
1,261,794	1,261,796	3	GTC -> CGT		84	Substitution	95.2%	D -> T	802	GAC -> ACG	C770_RS47735	ABC-type sugar transpo	Substitution	6.0E-255	268	1	WP_015243339.1
1,261,798	1,261,798	1	C -> T		84	SNP (transition)	97.6%	R -> K	800	AGG -> AAG	C770_RS47735	ABC-type sugar transpo	Substitution	3.5E-243	267	2	WP_015243339.1
1,261,800	1,261,800	1	G -> C		84	SNP (transversion)	98.8%		798	CCC -> CCG	C770_RS47735	ABC-type sugar transpo	None	5.3E-281	266	3	WP_015243339.1
1,261,803	1,261,803	1	A -> G		84	SNP (transition)	100.0%		795	CGT -> CGC	C770_RS47735	ABC-type sugar transpo	None	4.0E-261	265	3	WP_015243339.1
1,261,806	1,261,806	1	T -> C		84	SNP (transition)	100.0%		792	GCA -> GCG	C770_RS47735	ABC-type sugar transpo	None	1.6E-269	264	3	WP_015243339.1
1,261,812	1,261,812	1	A -> G		86	SNP (transition)	98.8%		786	GTT -> GTC	C770_RS47735	ABC-type sugar transpo	None	8.6E-271	262	3	WP_015243339.1
1,261,848	1,261,848	1	A -> G		87	SNP (transition)	98.9%		750	ACT -> ACC	C770_RS47735	ABC-type sugar transpo	None	1.4E-282	250	3	WP_015243339.1
1,261,875	1,261,875	1	G -> T		87	SNP (transversion)	100.0%		723	GCC -> GCA	C770_RS47735	ABC-type sugar transpo	None	1.6E-296	241	3	WP_015243339.1
1,261,878	1,261,878	1	C -> G		87	SNP (transversion)	100.0%		720	CCG -> CCC	C770_RS47735	ABC-type sugar transpo	None	7.9E-288	240	3	WP_015243339.1
1,261,884	1,261,884	1	A -> G		84	SNP (transition)	100.0%		714	GTT -> GTC	C770_RS47735	ABC-type sugar transpo	None	2.5E-286	238	3	WP_015243339.1
1,261,914	1,261,914	1	T -> C		92	SNP (transition)	100.0%		684	ACA -> ACG	C770_RS47735	ABC-type sugar transpo	None	4.0E-295	228	3	WP_015243339.1
1,261,980	1,261,980	1	G -> C		103	SNP (transversion)	99.0%		618	TCC -> TCG	C770_RS47735	ABC-type sugar transpo	None	6.5E-315	206	3	WP_015243339.1
1,261,989	1,261,989	1	G -> C		103	SNP (transversion)	100.0%		609	CTC -> CTG	C770_RS47735	ABC-type sugar transpo	None	0.0	203	3	WP_015243339.1
1,261,995	1,261,995	1	G -> A		103	SNP (transition)	98.1%		603	CGC -> CGT	C770_RS47735	ABC-type sugar transpo	None	0.0	201	3	WP_015243339.1
1,262,112	1,262,112	1	C -> T		97	SNP (transition)	97.9%		486	CAG -> CAA	C770_RS47735	ABC-type sugar transpo	None	4.7E-301	162	3	WP_015243339.1
1,262,136	1,262,136	1	C -> T		100	SNP (transition)	98.0%		462	CCG -> CCA	C770_RS47735	ABC-type sugar transpo	None	0.0	154	3	WP_015243339.1
1,262,139	1,262,139	1	T -> C		99	SNP (transition)	99.0%		459	CTA -> CTG	C770_RS47735	ABC-type sugar transpo	None	4.0E-322	153	3	WP_015243339.1
1,262,147	1,262,147	1	C -> G		98	SNP (transversion)	100.0%	V -> L	451	GTC -> CTC	C770_RS47735	ABC-type sugar transpo	Substitution	2.5E-314	151	1	WP_015243339.1
1,262,151	1,262,153	2	CAA -> GAG	97 -> 98		Substitution	97.9% -> 98.0%		445	TTG -> CTC	C770_RS47735	ABC-type sugar transpo	None	2.0E-323	149	1	WP_015243339.1
1,263,591	1,263,591	1	A -> G		82	SNP (transition)	98.8%		105	ATT -> ATC	C770_RS47740	LacI family transcription	None	4.1E-266	35	3	WP_033045584.1
1,263,600	1,263,600	1	C -> G		85	SNP (transversion)	98.8%		96	GTG -> GTC	C770_RS47740	LacI family transcription	None	5.4E-276	32	3	WP_033045584.1

1,263,722	1,263,722	1	A -> G	93	SNP (transition)	97.8%							2.1E-297			
1,263,919	1,263,918	0	G	88	Insertion	97.7%							6.1E-281			
1,263,974	1,263,974	1	G -> A	90	SNP (transition)	100.0%	A -> T	4	GCG -> ACG	C770_RS47745	transcriptional regulatoı	Substitution	1.0E-297	2	1	WP_015243340.1
1,264,130	1,264,131	2	TC -> CA	85	Substitution	100.0%	S -> Q	160	TCG -> CAG	C770_RS47745	transcriptional regulatoı	Substitution	1.0E-272	54	1	WP_015243340.1
1,582,010	1,582,010	1	G -> A	87	SNP (transition)	100.0%		915	AGC -> AGT	C770_RS49260	Na+/phosphate sympor	None	1.6E-296	305	3	WP_015243496.1

G6 pSymB

Minimum	Maximum	Length	Change	Coverage	Polymorphism Type	Variant Frequency	Amino Acid Change	CDS Position	Codon Change	locus_tag	product	Protein Effect	Variant P-Value (approximate)	CDS Codon Number	CDS Position Within Codon	protein_id
28.796	28.795	0	ACC	90	Insertion	98.9%							1.4E-283			
61.021	61.021	1	T -> C	72	SNP (transition)	100.0%							2.5E-238			
499.166	499.166	1	C -> G	76	SNP (transversion)	100.0%							1.6E-251			
693.997	693.997	1	T -> C	85	SNP (transition)	100.0%	S -> P	691	TCG -> CCG	C770_RS45205	sugar ABC transporter s	Substitution	3.2E-281	231	1	WP_010975799.1
748.330	748.331	2	GA -> TC	84	Substitution	96.4%	S -> E	1.885	TCG -> GAG	C770_RS45480	glycosyltransferase	Substitution	5.5E-276	629	1	WP_015243054.1
768.557	768.557	1	G -> A	75	SNP (transition)	100.0%	V -> I	508	GTC -> ATC	C770_RS45550	peptide ABC transporte	Substitution	3.2E-263	170	1	WP_015243061.1
790.565	790.565	1	C -> G	95	SNP (transversion)	100.0%		516	CGG -> CGC	C770_RS45645	ABC transporter	None	3.2E-314	172	3	WP_003529067.1
800.349	800.349	1	C -> T	89	SNP (transition)	100.0%		567	CTG -> CTA	C770_RS45680	LacI family transcription	None	3.2E-312	189	3	WP_003529089.1
801.077	801.077	1	G -> A	52	SNP (transition)	100.0%							2.5E-172			
801.928	801.928	1	T -> C	85	SNP (transition)	100.0%		801	CTT -> CTC	C770_RS45685	4-aminobutyrate amino	None	1.0E-289	267	3	WP_010975708.1
802.670	802.670	1	A -> G	60	SNP (transition)	100.0%		159	GGA -> GGG	C770_RS45690	succinate-semialdehyde	None	1.0E-180	53	3	WP_010975707.1
802.997	802.997	1	C -> T	71	SNP (transition)	98.6%		486	CCC -> CCT	C770_RS45690	succinate-semialdehyde	None	7.1E-230	162	3	WP_010975707.1
804.285	804.285	1	C -> G	67	SNP (transversion)	98.5%		918	CTG -> CTC	C770_RS45695	acetate kinase	None	1.1E-216	306	3	WP_014530439.1
804.786	804.786	1	C -> G	76	SNP (transversion)	98.7%		417	CCG -> CCC	C770_RS45695	acetate kinase	None	2.4E-261	139	3	WP_014530439.1
804.795	804.795	1	G -> T	74	SNP (transversion)	98.6%	D -> E	408	GAC -> GAA	C770_RS45695	acetate kinase	Substitution	4.7E-247	136	3	WP_014530439.1
804.866	804.866	1	A -> G	80	SNP (transition)	97.5%		337	TTG -> CTG	C770_RS45695	acetate kinase	None	2.0E-262	113	1	WP_014530439.1
805.000	805.000	1	T -> G	88	SNP (transversion)	97.7%	E -> A	203	GAG -> GCG	C770_RS45695	acetate kinase	Substitution	3.8E-298	68	2	WP_014530439.1
805.054	805.054	1	C -> G	84	SNP (transversion)	98.8%	G -> A	149	GGC -> GCC	C770_RS45695	acetate kinase	Substitution	2.1E-264	50	2	WP_014530439.1
807.127	807.127	1	T -> C	93	SNP (transition)	100.0%		1.821	GAT -> GAC	C770_RS45700	heat-shock protein Hsp!	None	6.3E-317	607	3	WP_015008300.1
807.352	807.352	1	T -> C	83	SNP (transition)	100.0%							1.0E-249			
807.465	807.465	1	A -> G	74	SNP (transition)	100.0%	C -> R	1.108	TGT -> CGT	C770_RS45705	acyl-CoA transferase/ca	Substitution	1.0E-222	370	1	WP_015243071.1
807.529	807.529	1	G -> C	67	SNP (transversion)	100.0%		1.044	GCC -> GCG	C770_RS45705	acyl-CoA transferase/ca	None	1.6E-228	348	3	WP_015243071.1
808.716	808.716	1	G -> A	80	SNP (transition)	100.0%		1.056	AAC -> AAT	C770_RS45710	acyl-CoA dehydrogenas	None	1.0E-280	352	3	WP_013850703.1
808.814	808.814	1	C -> A	76	SNP (transversion)	100.0%	G -> C	958	GGC -> TGC	C770_RS45710	acyl-CoA dehydrogenas	Substitution	6.3E-244	320	1	WP_013850703.1
808.878	808.878	1	A -> G	70	SNP (transition)	98.6%		894	GAT -> GAC	C770_RS45710	acyl-CoA dehydrogenas	None	1.4E-226	298	3	WP_013850703.1
809.520	809.520	1	A -> G	75	SNP (transition)	98.7%		252	GGT -> GGC	C770_RS45710	acyl-CoA dehydrogenas	None	4.7E-243	84	3	WP_013850703.1
809.574	809.574	1	G -> A	71	SNP (transition)	100.0%		198	GGC -> GGT	C770_RS45710	acyl-CoA dehydrogenas	None	3.2E-249	66	3	WP_013850703.1
810.060	810.060	1	A -> G	75	SNP (transition)	98.7%		102	GCA -> GCG	C770_RS45715	transcriptional regulatoı	None	1.9E-250	34	3	WP_015243072.1
810.132	810.132	1	A -> G	73	SNP (transition)	100.0%		174	AAA -> AAG	C770_RS45715	transcriptional regulatoı	None	3.2E-256	58	3	WP_015243072.1
810.336	810.336	1	T -> C	69	SNP (transition)	98.6%		378	TTT -> TTC	C770_RS45715	transcriptional regulatoı	None	2.7E-223	126	3	WP_015243072.1
810.531	810.531	1	C -> T	87	SNP (transition)	98.9%		573	GGC -> GGT	C770_RS45715	transcriptional regulatoı	None	3.5E-291	191	3	WP_015243072.1
810.536	810.536	1	A -> T	88	SNP (transversion)	100.0%	D -> V	578	GAT -> GTT	C770_RS45715	transcriptional regulatoı	Substitution	1.6E-273	193	2	WP_015243072.1
810.645	810.645	1	A -> G	85	SNP (transition)	98.8%		687	CAA -> CAG	C770_RS45715	transcriptional regulatoı	None	2.1E-284	229	3	WP_015243072.1
810.708	810.708	1	G -> T	89	SNP (transversion)	100.0%		750	GGG -> GGT	C770_RS45715	transcriptional regulatoı	None	3.2E-312	250	3	WP_015243072.1
810.786	810.786	1	T -> C	75	SNP (transition)	100.0%		828	GAT -> GAC	C770_RS45715	transcriptional regulatoı	None	1.0E-255	276	3	WP_015243072.1
810.798	810.798	1	A -> C	74	SNP (transversion)	100.0%		840	ACA -> ACC	C770_RS45715	transcriptional regulatoı	None	6.3E-245	280	3	WP_015243072.1
810.924	810.924	1	T -> G	78	SNP (transversion)	97.4%							1.2E-255			
811.002	811.002	1	C -> T	61	SNP (transition)	98.4%		12	TAC -> TAT	C770_RS45720	glycine/D-amino acid ox	None	6.1E-203	4	3	WP_015243073.1
811.326	811.326	1	A -> G	61	SNP (transition)	98.4%		336	GAA -> GAG	C770_RS45720	glycine/D-amino acid ox	None	6.1E-203	112	3	WP_015243073.1
811.358	811.358	1	C -> G	62	SNP (transversion)	98.4%	A -> G	368	GCC -> GGC	C770_RS45720	glycine/D-amino acid ox	Substitution	2.5E-206	123	2	WP_015243073.1
811.398	811.398	1	C -> G	59	SNP (transversion)	98.3%		408	GCC -> GCG	C770_RS45720	glycine/D-amino acid ox	None	5.9E-202	136	3	WP_015243073.1
811.969	811.969	1	C -> A	69	SNP (transversion)	98.6%	L -> M	979	CTG -> ATG	C770_RS45720	glycine/D-amino acid ox	Substitution	1.1E-209	327	1	WP_015243073.1
812.035	812.035	1	C -> T	67	SNP (transition)	100.0%		1.045	CTG -> TTG	C770_RS45720	glycine/D-amino acid ox	None	2.0E-208	349	1	WP_015243073.1
812.283	812.283	1	C -> T	61	SNP (transition)	98.4%		63	ATC -> ATT	C770_RS45725	Trimethylamine:corrino	None	6.1E-197	21	3	WP_015243074.1
812.695	812.695	1	G -> C	75	SNP (transversion)	100.0%	V -> L	475	GTG -> CTG	C770_RS45725	Trimethylamine:corrino	Substitution	3.2E-248	159	1	WP_015243074.1
812.796	812.796	1	G -> A	72	SNP (transition)	98.6%		576	CAG -> CAA	C770_RS45725	Trimethylamine:corrino	None	4.5E-226	192	3	WP_015243074.1
812.863	812.863	1	C -> T	68	SNP (transition)	100.0%		643	CTG -> TTG	C770_RS45725	Trimethylamine:corrino	None	6.3E-232	215	1	WP_015243074.1
812.868	812.868	1	T -> C	67	SNP (transition)	100.0%		648	ATT -> ATC	C770_RS45725	Trimethylamine:corrino	None	3.2E-235	216	3	WP_015243074.1
813.338	813.339	2	CA -> GC	83 -> 85	Substitution	100.0%	S -> C	1.118	TCA -> TGC	C770_RS45725	Trimethylamine:corrino	Substitution	6.3E-283	373	2	WP_015243074.1
813.695	813.695	1	T -> A	73	SNP (transversion)	97.3%	S -> T	55	TCG -> ACG	C770_RS45730	phosphonates import A	Substitution	8.3E-246	19	1	WP_015008301.1
814.584	814.584	1	A -> G	85	SNP (transition)	100.0%	T -> A	13	ACT -> GCT	C770_RS45735	phosphonate ABC trans	Substitution	3.2E-281	5	1	WP_015243075.1
814.951	814.951	1	A -> G	72	SNP (transition)	100.0%	D -> G	380	GAC -> GGC	C770_RS45735	phosphonate ABC trans	Substitution	1.6E-245	127	2	WP_015243075.1
815.366	815.366	1	C -> T	73	SNP (transition)	100.0%		795	GAC -> GAT	C770_RS45735	phosphonate ABC trans	None	1.3E-241	265	3	WP_015243075.1
815.865	815.865	1	A -> G	85	SNP (transition)	98.8%	M -> V	217	ATG -> GTG	C770_RS45740	phosphonate ABC trans	Substitution	8.5E-293	73	1	WP_010975697.1
815.882	815.882	1	A -> C	82	SNP (transversion)	100.0%		234	ATA -> ATC	C770_RS45740	phosphonate ABC trans	None	1.6E-279	78	3	WP_010975697.1
817.718	817.718	1	T -> C	60	SNP (transition)	98.3%		1.098	TTT -> TTC	C770_RS45745	phosphonate ABC trans	None	1.5E-199	366	3	WP_014530446.1
818.31	818.31	1	G -> C	66	SNP (transversion)	100.0%							4.0E-225			
819.201	819.201	1	G -> A	61	SNP (transition)	100.0%		21	TTG -> TTA	C770_RS45755	phosphonate metabolis	None	5.0E-202	7	3	WP_015243076.1
819.888	819.888	1	C -> A	81	SNP (transversion)	100.0%		708	GTC -> GTA	C770_RS45755	phosphonate metabolis	None	4.0E-276	236	3	WP_015243076.1
819.89	819.89	1	C -> A	80	SNP (transversion)	98.8%	A -> E	710	GCG -> GAG	C770_RS45755	phosphonate metabolis	Substitution	2.0E-267	237	2	WP_015243076.1
819.942	819.942	1	G -> A	79	SNP (transition)	100.0%		762	ACG -> ACA	C770_RS45755	phosphonate metabolis	None	1.6E-253	254	3	WP_015243076.1
820.241	820.241	1	C -> G	73	SNP (transversion)	100.0%	A -> G	1.061	GCC -> GGC	C770_RS45755	phosphonate metabolis	Substitution	2.0E-212	354	2	WP_015243076.1
820.442	820.442	1	C -> A	59	SNP (transversion)	100.0%		126	GTC -> GTA	C770_RS45760	phosphonate metabolis	None	2.5E-201	42	3	WP_015243077.1
820.613	820.613	1	A -> G	52	SNP (transition)	100.0%	I -> M	297	ATA -> ATG	C770_RS45760	phosphonate metabolis	Substitution	1.6E-177	99	3	WP_015243077.1
821.215	821.215	1	C -> T	90	SNP (transition)	100.0%		81	GCG -> GCA	C770_RS45765	ArsR family transcriptioı	None	1.0E-279	27	3	WP_013850711.1
821.243	821.243	1	A -> G	88	SNP (transition)	100.0%	V -> A	53	GTG -> GCG	C770_RS45765	ArsR family transcriptioı	Substitution	6.3E-300	18	2	WP_013850711.1
821.361	821.361	1	C -> A	81	SNP (transversion)	100.0%							4.0E-276			
821.819	821.819	1	C -> T	65	SNP (transition)	96.9%		435	CCC -> CCT	C770_RS45770	MFS transporter	None	2.6E-205	145	3	WP_003534319.1
821.912	821.912	1	G -> C	68	SNP (transversion)	100.0%		528	CTG -> CTC	C770_RS45770	MFS transporter	None	2.5E-218	176	3	WP_003534319.1
822.624	822.624	1	A -> G	81	SNP (transition)	100.0%							4.0E-276			
822.649	822.649	1	T -> C	80	SNP (transition)	100.0%							1.0E-264			
822.834	822.834	1	C -> A	82	SNP (transversion)	100.0%							1.0E-205			
822.869	822.869	1	G -> T	76	SNP (transversion)	98.7%							2.4E-171			
822.965	822.965	1	A -> G	80	SNP (transition)	100.0%							1.0E-104			
822.98	822.98	1	C -> T	81	SNP (transition)	100.0%							6.3E-98			
823.086	823.086	1	G -> A	71	SNP (transition)	98.6%	G -> R	55	GGG -> AGG	C770_RS45775	DNA polymerase	Substitution	6.0E-55	19	1	WP_041169873.1
823.293	823.293	1	G -> T	92	SNP (transversion)	98.9%	A -> S	262	GCG -> TCG	C770_RS45775	DNA polymerase	Substitution	5.4E-108	88	1	WP_041169873.1
823.303	823.303	1	A -> G	95	SNP (transition)	100.0%	D -> G	272	GAC -> GGC	C770_RS45775	DNA polymerase	Substitution	3.2E-124	91	2	WP_041169873.1
823.496	823.496	1	G -> T	80	SNP (transversion)	98.8%		465	CGG -> CGT	C770_RS45775	DNA polymerase	None	6.3E-228	155	3	WP_041169873.1
823.547	823.547	1	G -> A	75	SNP (transition)	100.0%		516	CAG -> CAA	C770_RS45775	DNA polymerase	None	1.0E-240	172	3	WP_041169873.1
823.559	823.559	1	G -> A	78	SNP (transition)	100.0%		528	CGG -> CGA	C770_RS45775	DNA polymerase	None	1.0E-234	176	3	WP_041169873.1
823.562	823.562	1	C -> T	76	SNP (transition)	100.0%		531	ATC -> ATT	C770_RS45775	DNA polymerase	None	2.5E-236	177	3	WP_041169873.1
823.648	823.648	1	G -> A	79	SNP (transition)	100.0%	R -> K	617	AGG -> AAG	C770_RS45775	DNA polymerase	Substitution	5.0E-214	206	2	WP_041169873.1
823.661	823.661	1	T -> C	81	SNP (transition)	100.0%		630	CGT -> CGC	C770_RS45775	DNA polymerase	None	3.2E-203	210	3	WP_041169873.1
824.331	824.332	2	GG -> TT	6	Substitution	100.0%	G -> F	1.3	GGC -> TTC	C770_RS45775	DNA polymerase	Substitution	6.3E-14	434	1	WP_041169873.1
824.335	824.341	7	TGTTGCT -> GCG	6 -> 8	Substitution	100.0%	MLL -> SGG	1.304	ATG,TTG,CTC ->	C770_RS45775	DNA polymerase	Substitution	4.0E-16	435	2	WP_041169873.1
824.343	824.344	2	AA -> TT	9	Substitution	100.0%	N -> F	1.312	AAT -> TTT	C770_RS45775	DNA polymerase	Substitution	1.6E-20	438	1	WP_041169873.1
824.346	824.346	1	T -> A	9	SNP (transversion)	100.0%	S -> T	1.315	TCG -> ACG	C770_RS45775	DNA polymerase	Substitution	6.3E-26	439	1	WP_041169873.1
824.35	824.35	1	T -> A	10	SNP (transversion)	100.0%	V -> D	1.319	GTC -> GAC	C770_RS45775	DNA polymerase	Substitution	1.0E-27	440	2	WP_041169873.1
824.352	824.354	3	CTA -> GGC	10 -> 11	Substitution	100.0%	L -> G	1.321	CTA -> GGC	C770_RS45775	DNA polymerase	Substitution	1.0E-24	441	1	WP_041169873.1
824.356	824.356	1	C -> G	12	SNP (transversion)	100.0%	T -> S	1.325	ACC -> AGC	C770_RS45775	DNA polymerase	Substitution	6.3E-38	442	2	WP_041169873.1
824.358	824.359	2	CT -> AC	14	Substitution	100.0%	L -> T	1.327	CTC -> ACC	C770_RS45775	DNA polymerase	Substitution	6.3E-40	443	1	WP_041169873.1
824.361	824.374	12	AGGTGGATGGA	14 -> 17	Substitution	100.0%		1.33		C770_RS45775	DNA polymerase	Truncation	1.0E-42	444	1	WP_041169873.1
824.376	824.384	8	CTCCGTATC -> G	18 -> 20	Substitution	100.0%	LRI -> ARA	1.345	CTC,CGT,ATC ->	C770_RS45775	DNA polymerase	Substitution	1.3E-59	449	1	WP_041169873.1
824.386	824.39	5	CCCGC -> GGTTA	20	Substitution	100.0%	PR -> RL	1.355	CCC,CGC -> CGG,	C770_RS45775	DNA polymerase	Substitution	1.0E-64	452	2	WP_041169873.1
824.392	824.393	2	TC -> CA	21	Substitution	95.2%	L -> P	1.361	CTC -> CCA	C770_RS45775	DNA polymerase	Substitution	2.1E-65	454	2	WP_041169873.1
824.395	824.396	2	CC -> TG	21	Substitution	100.0%	A -> V	1.364	GCC -> GTG	C770_RS45775	DNA polymerase	Substitution	5.0E-70	455	2	WP_041169873.1
824.723	824.723	1	A -> G	89	SNP (transition)	98.9%	H -> R	122	CAC -> CGC	C770_RS45780	imidazolonepropionase	Substitution	8.9E-307	41	2	WP_015243079.1
825.069	825.069	1	T -> C	78	SNP (transition)	100.0%		468	GTT -> GTC	C770_RS45780	imidazolonepropionase	None	1.0E-273	156	3	WP_015243079.1
825.084	825.084	1	C -> G	79	SNP (transversion)	98.7%	N -> K	483	AAC -> AAG	C770_RS45780	imidazolonepropionase	Substitution	5.0E-264	161	3	WP_015243079.1
825.474	825.474	1	G -> C	68	SNP (transversion)	100.0%		873	GTG -> GTC	C770_RS45780	imidazolonepropionase	None	4.0E-225	291	3	WP_015243079.1
825.74	825.74	1	T -> C	69	SNP (transition)	100.0%	F -> S	1.139	TTT -> TCT	C770_RS45780	imidazolonepropionase	Substitution	2.0E-228	380	2	WP_015243079.1
825.909	825.909	1	G -> C	72	SNP (transversion)	98.6%	G -> R	64	GGC -> CGC	C770_RS45785	histidine ammonia-lyase	Substitution	3.6E-233	22	1	WP_015243080.1
826.61	826.61	1	T -> C	90	SNP (transition)	100.0%		765	GCT -> GCC	C770_RS45785	histidine ammonia-lyase	None	1.0E-306	255	3	WP_015243080.1
826.997	826.997	1	C -> T	69	SNP (transition)	100.0%		1.152	GCC -> GCT	C770_RS45785	histidine ammonia-lyase	None	3.2E-242	384	3	WP_015243080.1
827.31	827.31	1	A -> G	74	SNP (transition)	100.0%	S -> G	1.465	AGC -> GGC	C770_RS45785	histidine ammonia-lyase	Substitution	2.5E-252	489	1	WP_015243080.1
827.333	827.333	1	G -> A	74	SNP (transition)	100.0%		1.488	CGG -> CGA	C770_RS45785	histidine ammonia-lyase	None	2.5E-252	496	3	WP_015243080.1
827.557	827.557	1	A -> G	59	SNP (transition)	100.0%	T -> A	175	ACG -> GCG	C770_RS45790	N-formylglutamate amic	Substitution	3.2E-207	59	1	WP_015008305.1
828.125	828.125	1	G -> T	77	SNP (transversion)	96.1%	R -> L	743	CGT -> CTT	C770_RS45790	N-formylglutamate amic	Substitution	4.6E-240	248	2	WP_015008305.1
828.669	828.669	1	A -> C	75	SNP (transversion)	100.0%	S -> R	469	AGT -> CGT	C770_RS45795	urocanate hydratase	Substitution	3.2E-248	157	1	WP_041169921.1
829.124	829.124	1	G -> C	73	SNP (transversion)	100.0%		924	GCG -> GCC	C770_RS45795	urocanate hydratase	None	1.3E-241	308	3	WP_041169921.1
829.321	829.321	1	A -> G	73	SNP (transition)	100.0%	K -> R	1.121	AAG -> AGG	C770_RS45795	urocanate hydratase	Substitution	6.3E-249	374	2	WP_041169921.1
829.757	829.757	1	C -> G	84	SNP (transversion)	100.0%		1.557	CGC -> CGG	C770_RS45795	urocanate hydratase	None	1.0E-294	519	3	WP_041169921.1
829.813	829.813	1	T -> A	85	SNP (transversion)	98.8%	V -> D	1.613	GTC -> GAC	C770_RS45795	urocanate hydratase	Substitution	2.1E-284	538	2	WP_041169921.1
829.844	829.844	1	C -> T	86	SNP (transition)	98.8%		1.644	GGC -> GGT	C770_RS45795	urocanate hydratase	None	8.6E-288	548	3	WP_041169921.1
829.847	829.847	1	G -> T	85	SNP (transversion)	100.0%		1.647	CTG -> CTT	C770_RS45795	urocanate hydratase	None	1.0E-272	549	3	WP_041169921.1
829.853	829.853	1	G -> A	81	SNP (transition)	98.8%		1.653	CTG -> CTA	C770_RS45795	urocanate hydratase	None	8.1E-279	551	3	WP_041169921.1
829.868	829.868	1	G -> C	78	SNP (transversion)	97.4%		1.668	GGG -> GGC	C770_RS45795	urocanate hydratase	None	1.2E-255	556	3	WP_041169921.1
829.902	829.902	1	G -> T	76	SNP (transversion)	100.0%							4.0E-259			
829.907	829.907	1	T -> C	77	SNP (transition)	100.0%							1.6E-262			
829.912	829.911	0	(C)3 -> (C)4	77	Insertion (tandem repeat)	98.7%							1.2E-249			
829.917	829.917	1	T -> C	77	SNP (transition)	100.0%							1.6E-262			
829.919	829.92	2	TC -> CA	75	Substitution	100.0%							3.2E-233			
829.942	829.941	0	(G)3 -> (G)4	79	Insertion (tandem repeat)	100.0%							2.0E-261			
829.945	829.945	1	A -> G	77	SNP (transition)	100.0%							3.2E-270			
829.95	829.95	1	G -> A	76	SNP (transition)	100.0%							2.5E-274			
829.966	829.966	1	C -> T	75	SNP (transition)	97.3%							4.4E-260			
829.974	829.974	1	G -> A	76	SNP (transition)	100.0%							1.0E-266			
830.054	830.054	1	G -> A	73	SNP (transition)	100.0%							1.3E-241			
830.06	830.06	1	C -> A	73	SNP (transversion)	100.0%							3.2E-256			
830.118	830.118	1	G -> A	70	SNP (transition)	100.0%		1.168	CTG -> TTG	C770_RS45800	Arabinose efflux perme	None	1.0E-238	390	1	WP_015243082.1
830.134	830.134	1	G -> A	73	SNP (transition)	100.0%		1.152	ATC -> ATT	C770_RS45800	Arabinose efflux perme	None	5.0E-227	384	3	WP_015243082.1
830.146	830.146	1	G -> C	75	SNP (transversion)	98.7%		1.14	TCC -> TCG	C770_RS45800	Arabinose efflux perme	None	4.7E-243	380	3	WP_015243082.1
830.163	830.163	1	C -> T	73	SNP (transition)	100.0%	A -> T	1.123	GCC -> ACC	C770_RS45800	Arabinose efflux perme	Substitution	1.3E-241	375	1	WP_015243082.1
830.197	830.197	1	A -> G	76	SNP (transition)	100.0%		1.089	TTT -> TTC	C770_RS45800	Arabinose efflux perme	None	1.6E-251	363	3	WP_015243082.1
830.218	830.218	1	T -> C	76	SNP (transition)	98.7%		1.068	GCA -> GCG	C770_RS45800	Arabinose efflux perme	None	2.4E-246	356	3	WP_015243082.1
830.5	830.5	1	A -> G	79	SNP (transition)	100.0%		786	GGT -> GGC	C770_RS45800	Arabinose efflux perme	None	3.2E-277	262	3	WP_015243082.1
830.618	830.618	1	A -> G	80	SNP (transition)	100.0%	V -> A	668	GTA -> GCA	C770_RS45800	Arabinose efflux perme	Substitution	1.0E-280	223	2	WP_015243082.1
830.691	830.691	1	C -> A	79	SNP (transversion)	100.0%	V -> L	595	GTG -> TTG	C770_RS45800	Arabinose efflux perme	Substitution	2.5E-269	199	1	WP_015243082.1
830.977	830.977	1	A -> C	85	SNP (transversion)	98.8%		309	GGT -> GGG	C770_RS45800	Arabinose efflux perme	None	5.4E-276	103	3	WP_015243082.1
831.651	831.651	1	G -> A	81	SNP (transition)	97.5%	A -> V	1.142	GCC -> GTC	C770_RS45805	hypothetical protein	Substitution	6.5E-258	381	2	WP_015243083.1
832.034	832.034	1	G -> A	82	SNP (transition)	98.8%		759	GCC -> GCT	C770_RS45805	hypothetical protein	None	2.6E-282	253	3	WP_015243083.1
832.573	832.573	1	C -> G	68	SNP (transversion)	100.0%	E -> Q	220	GAG -> CAG	C770_RS45805	hypothetical protein	Substitution	6.3E-232	74	1	WP_015243083.1
833.034	833.034	1	T -> C	78	SNP (transition)	100.0%	T -> A	1.687	ACG -> GCG	C770_RS45810	N-acetyl-beta-hexosami	Substitution	4.0E-258	563	1	WP_015243084.1
833.398	833.398	1	T -> C	90	SNP (transition)	100.0%		1.323	CGA -> CGG	C770_RS45810	N-acetyl-beta-hexosami	None	1.0E-297	441	3	WP_015243084.1
833.46	833.46	1	A -> G	75	SNP (transition)	100.0%	S -> P	1.261	TCG -> CCG	C770_RS45810	N-acetyl-beta-hexosami	Substitution	3.2E-263	421	1	WP_015243084.1
833.5	833.5	1	T -> C	82	SNP (transition)	100.0%		1.221	TCA -> TCG	C770_RS45810	N-acetyl-beta-hexosami	None	4.0E-263	407	3	WP_015243084.1
833.602	833.602	1	T -> G	102	SNP (transversion)	100.0%		1.119	ATA -> ATC	C770_RS45810	N-acetyl-beta-hexosami	None	0.0	373	3	WP_015243084.1
833.626	833.626	1	C -> G	89	SNP (transversion)	98.9%		1.095	GTG -> GTC	C770_RS45810	N-acetyl-beta-hexosami	None	8.9E-307	365	3	WP_015243084.1
833.686	833.686	1	C -> G	90	SNP (transversion)	98.9%		1.035	CTG -> CTC	C770_RS45810	N-acetyl-beta-hexosami	None	3.6E-319	345	3	WP_015243084.1
833.721	833.721	1	G -> T	94	SNP (transversion)	100.0%	L -> M	1	CTG -> ATG	C770_RS45810	N-acetyl-beta-hexosami	Substitution	2.5E-320	334	1	WP_015243084.1
833.881	833.881	1	A -> G	77	SNP (transition)	97.4%		840	CAT -> CAC	C770_RS45810	N-acetyl-beta-hexosami	None	2.9E-252	280	3	WP_015243084.1
833.917	833.917	1	A -> G	81	SNP (transition)	100.0%		804	ATT -> ATC	C770_RS45810	N-acetyl-beta-hexosami	None	4.0E-276	268	3	WP_015243084.1

833.921	833.921	1	G -> T		80	SNP (transversion)	100.0%	A -> E	800	GCA -> GAA	C770_RS45810	N-acetyl-beta-hexosami	Substitution	1.0E-272	267	2	WP_015243084.1
833.928	833.928	1	T -> A		80	SNP (transversion)	98.8%	T -> S	793	ACC -> TCC	C770_RS45810	N-acetyl-beta-hexosami	Substitution	2.0E-267	265	1	WP_015243084.1
833.944	833.944	1	T -> A		81	SNP (transversion)	100.0%	E -> D	777	GAA -> GAT	C770_RS45810	N-acetyl-beta-hexosami	Substitution	5.0E-268	259	3	WP_015243084.1
834.898	834.898	1	A -> C		77	SNP (transversion)	100.0%		624	CGT -> CGG	C770_RS45815	short-chain dehydroger	None	2.0E-239	208	3	WP_010975681.1
835.66	835.66	1	C -> T		76	SNP (transition)	100.0%	V -> I	745	GTC -> ATC	C770_RS45820	RpiR family transcriptio	Substitution	1.0E-266	249	1	WP_014530457.1
836.668	836.668	1	G -> A		69	SNP (transition)	98.6%		147	GTC -> GTT	C770_RS45825	endoribonuclease L-PSF	None	2.7E-223	49	3	WP_010975679.1
836.869	836.869	1	A -> C		64	SNP (transversion)	98.4%							1.0E-225			
836.907	836.907	1	T -> G		66	SNP (transversion)	100.0%							4.0E-225			
836.922	836.922	1	T -> GC	62 -> 65		Insertion	100.0%							2.5E-205			
836.948	836.948	1	C -> G		63	SNP (transversion)	100.0%	H -> Q	18	CAC -> CAG	C770_RS45830	hypothetical protein	Substitution	6.3E-215	6	3	WP_015243086.1
838.886	838.886	1	G -> C		66	SNP (transversion)	98.5%	S -> T	1.442	AGC -> ACC	C770_RS45835	hypothetical protein	Substitution	6.6E-220	481	2	WP_015243087.1
839.241	839.241	1	C -> G		92	SNP (transversion)	97.8%		141	CGG -> CGC	C770_RS45840	glyoxalase	None	4.2E-285	47	3	WP_014530460.1
839.694	839.694	1	G -> C		75	SNP (transversion)	100.0%	D -> E	339	GAC -> GAG	C770_RS45845	Sortase and related acyl	Substitution	1.0E-240	113	3	WP_015243088.1
839.807	839.807	1	G -> T		88	SNP (transversion)	100.0%	R -> S	226	CGC -> AGC	C770_RS45845	Sortase and related acyl	Substitution	6.3E-300	76	1	WP_015243088.1
840.346	840.346	1	C -> T		86	SNP (transition)	98.8%	G -> S	823	GGC -> AGC	C770_RS45850	sugar ABC transporter A	Substitution	2.7E-296	275	1	WP_015243089.1
840.56	840.56	1	G -> C		70	SNP (transversion)	97.1%		609	CGC -> CGG	C770_RS45850	sugar ABC transporter A	None	1.5E-228	203	3	WP_015243089.1
840.948	840.948	1	T -> C		56	SNP (transition)	100.0%	K -> R	221	AAG -> AGG	C770_RS45850	sugar ABC transporter A	Substitution	4.0E-191	74	2	WP_015243089.1
841.335	841.335	1	C -> G		77	SNP (transversion)	100.0%		1.152	GTG -> GTC	C770_RS45855	sugar ABC transporter s	None	7.9E-255	384	3	WP_015243090.1
841.841	841.841	1	C -> T		67	SNP (transition)	100.0%	V -> I	646	GTC -> ATC	C770_RS45855	sugar ABC transporter s	Substitution	1.6E-228	216	1	WP_015243090.1
843.23	843.23	1	A -> G		65	SNP (transition)	100.0%		144	TTT -> TTC	C770_RS45860	ABC transporter perme	None	3.2E-228	48	3	WP_014530463.1
843.45	843.45	1	A -> G		80	SNP (transition)	100.0%		805	TTG -> CTG	C770_RS45865	ABC transporter perme	None	1.0E-280	269	1	WP_010975671.1
844.582	844.582	1	C -> G		79	SNP (transversion)	100.0%		846	TCG -> TCC	C770_RS45870	oxidoreductase	None	3.2E-277	282	3	WP_014527226.1
845.271	845.271	1	A -> G		73	SNP (transition)	98.6%	Y -> H	157	TAC -> CAC	C770_RS45870	oxidoreductase	Substitution	2.9E-229	53	1	WP_014527226.1
845.479	845.479	1	G -> C		54	SNP (transversion)	100.0%							4.0E-195			
845.523	845.523	1	C -> G		58	SNP (transversion)	100.0%							1.6E-209			
846.222	846.222	1	C -> G		75	SNP (transversion)	100.0%	Q -> H	135	CAG -> CAC	C770_RS45875	ABC transporter	Substitution	1.0E-255	45	3	WP_033044297.1
848.251	848.251	1	C -> G		91	SNP (transversion)	97.8%		237	ACG -> ACC	C770_RS45885	ABC transporter perme	None	1.0E-299	79	3	WP_014527228.1
848.263	848.263	1	C -> G		93	SNP (transversion)	97.8%		225	GCG -> GCC	C770_RS45885	ABC transporter perme	None	1.7E-306	75	3	WP_014527228.1
913.819	913.819	1	C -> G		55	SNP (transversion)	100.0%	E -> D	552	GAG -> GAC	C770_RS46145	polysaccharide deacetyl	Substitution	1.0E-187	184	3	WP_015243112.1
921.29	921.29	1	C -> A		69	SNP (transversion)	100.0%	A -> S	289	GCC -> TCC	C770_RS46175	pyridoxal phosphate-de	Substitution	1.6E-221	97	1	WP_015243117.1
1,013,670	1,013,670	1	T -> G		91	SNP (transversion)	98.9%							9.1E-305			
1,059,297	1,059,297	1	A -> G		75	SNP (transition)	100.0%							3.2E-263			
1,078,126	1,078,126	1	T -> C		78	SNP (transition)	100.0%	V -> A	77	GTA -> GCA	C770_RS46845	hypothetical protein	Substitution	6.3E-266	26	2	WP_015243200.1
1,094,103	1,094,103	1	C -> T		99	SNP (transition)	99.0%							2.5E-312			
1,098,191	1,098,191	1	T -> C		80	SNP (transition)	100.0%		276	ACA -> ACG	C770_RS46935	ABC transporter ATPase	None	1.0E-272	92	3	WP_015243216.1
1,098,196	1,098,196	1	C -> G		80	SNP (transversion)	100.0%	E -> Q	271	GAG -> CAG	C770_RS46935	ABC transporter ATPase	Substitution	1.0E-264	91	1	WP_015243216.1
1,098,215	1,098,215	1	C -> G		71	SNP (transversion)	98.6%		252	GGG -> GGC	C770_RS46935	ABC transporter ATPase	None	7.1E-244	84	3	WP_015243216.1
1,098,254	1,098,254	1	T -> G		80	SNP (transversion)	100.0%		213	ACA -> ACC	C770_RS46935	ABC transporter ATPase	None	1.0E-264	71	3	WP_015243216.1
1,098,265	1,098,265	1	A -> G		79	SNP (transition)	100.0%	F -> L	202	TTC -> CTC	C770_RS46935	ABC transporter ATPase	Substitution	2.5E-269	68	1	WP_015243216.1
1,098,293	1,098,293	1	G -> A		79	SNP (transition)	98.7%		174	AAC -> AAT	C770_RS46935	ABC transporter ATPase	None	3.1E-256	58	3	WP_015243216.1
1,098,302	1,098,302	1	A -> G		82	SNP (transition)	100.0%		165	ACT -> ACC	C770_RS46935	ABC transporter ATPase	None	2.5E-271	55	3	WP_015243216.1
1,098,736	1,098,736	1	A -> C		92	SNP (transversion)	98.9%		603	CTT -> CTG	C770_RS46940	peptide ABC transporte	None	2.9E-317	201	3	WP_015243217.1
1,098,772	1,098,772	1	T -> C		94	SNP (transition)	100.0%		567	CGA -> CGG	C770_RS46940	peptide ABC transporte	None	6.3E-311	189	3	WP_015243217.1
1,098,859	1,098,859	1	A -> C		90	SNP (transversion)	100.0%		480	GCT -> GCG	C770_RS46940	peptide ABC transporte	None	1.0E-315	160	3	WP_015243217.1
1,099,260	1,099,260	1	T -> C		86	SNP (transition)	100.0%	S -> G	79	AGC -> GGC	C770_RS46940	peptide ABC transporte	Substitution	1.6E-284	27	1	WP_015243217.1
1,099,288	1,099,288	1	G -> C		85	SNP (transversion)	100.0%		51	GTC -> GTG	C770_RS46940	peptide ABC transporte	None	1.0E-289	17	3	WP_015243217.1
1,099,433	1,099,433	1	A -> G		93	SNP (transition)	100.0%		897	ATT -> ATC	C770_RS46945	ABC transporter perme	None	1.3E-307	299	3	WP_010975490.1
1,099,438	1,099,438	1	C -> A		94	SNP (transversion)	100.0%	V -> F	892	GTC -> TTC	C770_RS46945	ABC transporter perme	Substitution	6.3E-311	298	1	WP_010975490.1
1,099,445	1,099,445	1	G -> C		94	SNP (transversion)	100.0%		885	ACC -> ACG	C770_RS46945	ABC transporter perme	None	6.3E-311	295	3	WP_010975490.1
1,099,517	1,099,517	1	G -> A		96	SNP (transition)	100.0%		813	TTC -> TTT	C770_RS46945	ABC transporter perme	None	6.3E-308	271	3	WP_010975490.1
1,099,633	1,099,633	1	C -> A		98	SNP (transversion)	100.0%	A -> S	697	GCG -> TCG	C770_RS46945	ABC transporter perme	Substitution	0.0	233	1	WP_010975490.1
1,099,665	1,099,665	1	T -> A		97	SNP (transversion)	100.0%	Y -> F	665	TAC -> TTC	C770_RS46945	ABC transporter perme	Substitution	7.9E-321	222	2	WP_010975490.1
1,099,691	1,099,691	1	C -> T		92	SNP (transition)	98.9%		639	GGG -> GGA	C770_RS46945	ABC transporter perme	None	5.8E-290	213	3	WP_010975490.1
1,099,742	1,099,742	1	T -> C		97	SNP (transition)	100.0%		588	TTA -> TTG	C770_RS46945	ABC transporter perme	None	7.9E-321	196	3	WP_010975490.1
1,099,819	1,099,819	1	G -> A		92	SNP (transition)	98.9%		511	CTG -> TTG	C770_RS46945	ABC transporter perme	None	4.6E-299	171	1	WP_010975490.1
1,099,970	1,099,970	1	A -> G		101	SNP (transition)	100.0%		360	CTT -> CTC	C770_RS46945	ABC transporter perme	None	0.0	120	3	WP_010975490.1
1,099,973	1,099,973	1	T -> A		100	SNP (transversion)	100.0%		357	GGA -> GGT	C770_RS46945	ABC transporter perme	None	0.0	119	3	WP_010975490.1
1,100,183	1,100,183	1	G -> A		80	SNP (transition)	100.0%		147	GAC -> GAT	C770_RS46945	ABC transporter perme	None	1.0E-272	49	3	WP_010975490.1
1,100,243	1,100,243	1	G -> A		75	SNP (transition)	100.0%		87	ATC -> ATT	C770_RS46945	ABC transporter perme	None	1.0E-255	29	3	WP_010975490.1
1,100,348	1,100,348	1	C -> T		74	SNP (transition)	100.0%		1.584	CCG -> CCA	C770_RS46950	peptide ABC transporte	None	1.0E-259	528	3	WP_015243218.1
1,100,352	1,100,352	1	C -> A		71	SNP (transversion)	98.6%	R -> L	1.58	CGT -> CTT	C770_RS46950	peptide ABC transporte	Substitution	7.1E-230	527	2	WP_015243218.1
1,100,495	1,100,495	1	G -> C		76	SNP (transversion)	100.0%		1.437	GCC -> GCG	C770_RS46950	peptide ABC transporte	None	1.6E-251	479	3	WP_015243218.1
1,100,581	1,100,581	1	A -> G		83	SNP (transition)	100.0%		1.351	TTG -> CTG	C770_RS46950	peptide ABC transporte	None	3.2E-291	451	1	WP_015243218.1
1,100,592	1,100,592	1	T -> C		89	SNP (transition)	100.0%	E -> G	1.34	GAG -> GGG	C770_RS46950	peptide ABC transporte	Substitution	2.5E-303	447	2	WP_015243218.1
1,100,681	1,100,681	1	C -> G		88	SNP (transversion)	100.0%		1.251	GGG -> GGC	C770_RS46950	peptide ABC transporte	None	6.3E-300	417	3	WP_015243218.1
1,100,690	1,100,690	1	G -> C		87	SNP (transversion)	98.9%		1.242	CGC -> CGG	C770_RS46950	peptide ABC transporte	None	3.5E-291	414	3	WP_015243218.1
1,100,705	1,100,705	1	G -> T		82	SNP (transversion)	100.0%		1.227	GTC -> GTA	C770_RS46950	peptide ABC transporte	None	2.5E-271	409	3	WP_015243218.1
1,100,723	1,100,723	1	C -> T		85	SNP (transition)	100.0%		1.209	GCG -> GCA	C770_RS46950	peptide ABC transporte	None	3.2E-281	403	3	WP_015243218.1
1,100,759	1,100,759	1	C -> T		88	SNP (transition)	100.0%		1.173	CAG -> CAA	C770_RS46950	peptide ABC transporte	None	2.5E-282	391	3	WP_015243218.1
1,100,810	1,100,810	1	C -> G		91	SNP (transversion)	98.9%		1.122	GGG -> GGC	C770_RS46950	peptide ABC transporte	None	9.1E-287	374	3	WP_015243218.1
1,100,844	1,100,844	1	C -> T		83	SNP (transition)	100.0%	G -> D	1.088	GGT -> GAT	C770_RS46950	peptide ABC transporte	Substitution	6.3E-283	363	2	WP_015243218.1
1,100,899	1,100,900	2	AG -> GT		85	Substitution	100.0%	NL -> KL	1.032	AAC,TTG -> AAA,	C770_RS46950	peptide ABC transporte	Substitution	1.0E-289	344	3	WP_015243218.1
1,100,939	1,100,939	1	G -> A		80	SNP (transition)	100.0%		993	GCC -> GCT	C770_RS46950	peptide ABC transporte	None	1.0E-272	331	3	WP_015243218.1
1,100,960	1,100,960	1	G -> T		86	SNP (transversion)	100.0%		972	GCC -> GCA	C770_RS46950	peptide ABC transporte	None	1.0E-301	324	3	WP_015243218.1
1,101,063	1,101,063	1	G -> T		91	SNP (transversion)	98.9%	A -> D	869	GCC -> GAC	C770_RS46950	peptide ABC transporte	Substitution	9.1E-305	290	2	WP_015243218.1
1,101,074	1,101,074	1	T -> C		93	SNP (transition)	98.9%		858	CCA -> CCG	C770_RS46950	peptide ABC transporte	None	3.7E-293	286	3	WP_015243218.1
1,101,086	1,101,086	1	C -> T		89	SNP (transition)	98.9%		846	CGG -> CGA	C770_RS46950	peptide ABC transporte	None	3.5E-289	282	3	WP_015243218.1
1,101,248	1,101,248	1	A -> C		101	SNP (transversion)	100.0%		684	GTT -> GTG	C770_RS46950	peptide ABC transporte	None	0.0	228	3	WP_015243218.1
1,101,308	1,101,308	1	C -> A		87	SNP (transversion)	100.0%		624	CTG -> CTT	C770_RS46950	peptide ABC transporte	None	4.0E-279	208	3	WP_015243218.1
1,101,311	1,101,311	1	C -> T		87	SNP (transition)	98.9%		621	AGG -> AGA	C770_RS46950	peptide ABC transporte	None	3.5E-291	207	3	WP_015243218.1
1,101,371	1,101,371	1	A -> G		84	SNP (transition)	100.0%		561	CAT -> CAC	C770_RS46950	peptide ABC transporte	None	2.5E-286	187	3	WP_015243218.1
1,101,374	1,101,374	1	C -> A		85	SNP (transversion)	100.0%		558	GGG -> GGT	C770_RS46950	peptide ABC transporte	None	1.0E-289	186	3	WP_015243218.1
1,101,426	1,101,426	1	A -> C		84	SNP (transversion)	98.8%	V -> G	506	GTG -> GGG	C770_RS46950	peptide ABC transporte	Substitution	5.3E-281	169	2	WP_015243218.1
1,101,485	1,101,485	1	C -> T		82	SNP (transition)	98.8%		447	GTG -> GTA	C770_RS46950	peptide ABC transporte	None	4.1E-266	149	3	WP_015243218.1
1,101,497	1,101,497	1	A -> G		83	SNP (transition)	100.0%		435	GAT -> GAC	C770_RS46950	peptide ABC transporte	None	1.3E-274	145	3	WP_015243218.1
1,101,654	1,101,654	1	G -> T		72	SNP (transversion)	98.6%	A -> E	278	GCG -> GAG	C770_RS46950	peptide ABC transporte	Substitution	2.3E-247	93	2	WP_015243218.1
1,101,710	1,101,710	1	T -> G		73	SNP (transversion)	97.3%		222	CTA -> CTC	C770_RS46950	peptide ABC transporte	None	8.3E-246	74	3	WP_015243218.1
1,101,716	1,101,716	1	G -> C		71	SNP (transversion)	100.0%	D -> E	216	GAC -> GAG	C770_RS46950	peptide ABC transporte	Substitution	3.2E-249	72	3	WP_015243218.1
1,101,764	1,101,764	1	G -> A		71	SNP (transition)	100.0%		168	AAC -> AAT	C770_RS46950	peptide ABC transporte	None	3.2E-249	56	3	WP_015243218.1
1,101,782	1,101,782	1	A -> G		74	SNP (transition)	100.0%		150	TAT -> TAC	C770_RS46950	peptide ABC transporte	None	1.0E-259	50	3	WP_015243218.1
1,101,794	1,101,794	1	C -> G		68	SNP (transversion)	98.5%		138	CTG -> CTC	C770_RS46950	peptide ABC transporte	None	2.1E-233	46	3	WP_015243218.1
1,101,812	1,101,812	1	C -> G		74	SNP (transversion)	100.0%		120	TCG -> TCC	C770_RS46950	peptide ABC transporte	None	6.3E-245	40	3	WP_015243218.1
1,101,815	1,101,815	1	T -> C		73	SNP (transition)	100.0%		117	ACA -> ACG	C770_RS46950	peptide ABC transporte	None	6.3E-249	39	3	WP_015243218.1
1,101,866	1,101,866	1	C -> T		79	SNP (transition)	100.0%		66	GGG -> GGA	C770_RS46950	peptide ABC transporte	None	2.0E-261	22	3	WP_015243218.1
1,101,883	1,101,883	1	A -> T		74	SNP (transversion)	100.0%	F -> I	49	TTC -> ATC	C770_RS46950	peptide ABC transporte	Substitution	6.3E-245	17	1	WP_015243218.1
1,101,955	1,101,955	1	(T)5 -> (T)4		54	Deletion (tandem repeat)	100.0%							1.0E-81			
1,101,975	1,101,975	1	A -> G		49	SNP (transition)	100.0%							2.0E-162			
1,101,981	1,101,981	1	A		49	Deletion	100.0%							3.2E-74			
1,101,998	1,101,998	1	T -> G		44	SNP (transversion)	100.0%							4.0E-137			
1,102,005	1,102,005	1	A -> G		43	SNP (transition)	100.0%							1.3E-142			
1,102,026	1,102,026	1	C -> G		35	SNP (transversion)	97.1%							5.5E-108			
1,102,030	1,102,030	1	G -> T		34	SNP (transversion)	100.0%							1.0E-102			
1,102,032	1,102,031	0	T		33	Insertion	100.0%							1.0E-99			
1,102,038	1,102,039	2	AG -> GT		29	Substitution	100.0%							1.6E-93			
1,102,041	1,102,041	1	T -> A		29	SNP (transversion)	100.0%							2.5E-99			
1,102,045	1,102,045	1	T -> G		26	SNP (transversion)	100.0%							2.5E-94			
1,102,047	1,102,047	1	G -> A		25	SNP (transition)	100.0%							3.2E-88			
1,102,052	1,102,053	2	GC -> AA		22	Substitution	95.5%							7.0E-73			
1,102,058	1,102,057	0	A		21	Insertion	100.0%							3.2E-74			
1,102,064	1,102,065	2	GG -> A		20	Deletion	100.0%							1.0E-32			
1,102,069	1,102,069	1	A -> C		20	SNP (transversion)	100.0%							1.0E-66			
1,102,072	1,102,072	1	T -> C		20	SNP (transition)	100.0%							1.0E-72			
1,102,075	1,102,075	1	C -> G		20	SNP (transversion)	100.0%							1.0E-68			
1,102,078	1,102,084	7	GGTGAAC -> CTC	16 -> 19		Substitution	100.0%							2.0E-53			
1,102,086	1,102,088	3	ATT -> G	15 -> 16		Deletion	100.0%							1.0E-21			
1,102,092	1,102,092	1	A -> G		15	SNP (transition)	100.0%							3.2E-47			
1,102,097	1,102,099	3	ATC -> GCT	14 -> 15		Substitution	100.0%	D -> S	544	GAT -> AGC	C770_RS46955	hypothetical protein	Substitution	6.3E-47	182	1	WP_015243219.1
1,102,102	1,102,102	1	C -> T		14	SNP (transition)	100.0%	G -> S	541	GGC -> AGC	C770_RS46955	hypothetical protein	Substitution	6.3E-47	181	1	WP_015243219.1
1,102,108	1,102,108	1	G -> A		13	SNP (transition)	100.0%	L -> F	535	CTT -> TTT	C770_RS46955	hypothetical protein	Substitution	4.0E-37	179	1	WP_015243219.1
1,102,113	1,102,114	2	GA -> AG	11 -> 12		Substitution	100.0%	S -> L	529	TCC -> CTC	C770_RS46955	hypothetical protein	Substitution	6.3E-36	177	1	WP_015243219.1
1,102,116	1,102,116	1	T -> C		11	SNP (transition)	100.0%	Q -> R	527	CAA -> CGA	C770_RS46955	hypothetical protein	Substitution	6.3E-36	176	2	WP_015243219.1
1,102,119	1,102,119	1	C -> A		9	SNP (transversion)	100.0%	R -> L	524	CGC -> CTC	C770_RS46955	hypothetical protein	Substitution	1.3E-28	175	2	WP_015243219.1
1,102,121	1,102,122	2	TC -> GT		8	Substitution	100.0%	G -> D	521	GGA -> GAC	C770_RS46955	hypothetical protein	Substitution	2.5E-26	174	2	WP_015243219.1
1,102,126	1,102,126	1	G -> C		7	SNP (transversion)	100.0%	P -> A	517	CCC -> GCC	C770_RS46955	hypothetical protein	Substitution	5.0E-21	173	1	WP_015243219.1
1,102,128	1,102,128	1	A -> G		7	SNP (transition)	100.0%	M -> T	515	ATG -> ACG	C770_RS46955	hypothetical protein	Substitution	4.0E-23	172	2	WP_015243219.1
1,102,130	1,102,130	1	A -> C		6	SNP (transversion)	100.0%		513	TCT -> TCG	C770_RS46955	hypothetical protein	None	1.6E-20	171	3	WP_015243219.1
1,103,720	1,103,723	4	GTCA -> CATT	6 -> 7		Substitution	100.0%							2.5E-16			
1,103,725	1,103,727	3	GCA -> TTT		10	Substitution	100.0%							1.0E-34			
1,103,730	1,103,730	1	A -> T		12	SNP (transversion)	100.0%							4.0E-39			
1,103,732	1,103,737	6	GCTTGC -> TTCA	13 -> 14		Substitution	100.0%							3.2E-46			
1,103,741	1,103,742	2	AA -> TC		15	Substitution	100.0%							1.0E-48			
1,103,744	1,103,746	3	TCC -> GAA		15	Substitution	100.0%							3.2E-47			
1,103,748	1,103,748	1	G -> A		15	SNP (transition)	100.0%							1.0E-45			
1,103,750	1,103,753	4	CCCT -> AGTC	16 -> 17		Substitution	100.0%							6.3E-52			
1,103,757	1,103,757	1	T -> C		19	SNP (transition)	100.0%							1.6E-61			
1,103,761	1,103,761	1	C -> T		19	SNP (transition)	100.0%							2.0E-63			
1,103,763	1,103,762	0	G		19	Insertion	100.0%							1.6E-61			
1,103,768	1,103,771	4	CACA -> GTTC	21 -> 23		Substitution	95.2% -> 95.7%							2.1E-61			
1,103,788	1,103,787	0	T		29	Insertion	100.0%							2.0E-96			
1,103,801	1,103,801	1	T -> C		31	SNP (transition)	100.0%							7.9E-97			
1,103,824	1,103,824	1	G -> A		37	SNP (transition)	100.0%							3.2E-130			
1,103,830	1,103,829	0	G		39	Insertion	100.0%							2.5E-133			
1,103,866	1,103,866	1	T -> C		47	SNP (transition)	100.0%							1.6E-160			
1,103,871	1,103,871	1	A -> G		48	SNP (transition)	100.0%							2.5E-154			
1,103,889	1,103,889	1	T -> A		54	SNP (transversion)	100.0%							1.0E-189			
1,103,909	1,103,909	1	G		61	Deletion	100.0%							2.5E-98			
1,103,945	1,103,945	1	A -> G		78	SNP (transition)	100.0%							4.0E-258			
1,103,987	1,103,987	1	G -> C		80	SNP (transversion)	98.8%							2.0E-267			
1,103,991	1,103,991	1	A -> C		77	SNP (transversion)	97.4%							9.2E-260			
1,104,008	1,104,008	1	A -> T		77	SNP (transversion)	100.0%							1.6E-262			
1,104,017	1,104,017	1	G -> A		74	SNP (transition)	100.0%							1.6E-237			
1,104,021	1,104,021	1	A -> T		77	SNP (transversion)	97.4%							9.2E-245			
1,104,064	1,104,064	1	A -> G		73	SNP (transition)	100.0%							1.3E-241			
1,104,066	1,104,066	1	A -> G		72	SNP (transition)	98.6%							3.6E-233			
1,104,087	1,104,087	1	-C		76	Deletion	100.0%							6.3E-130			
1,104,097	1,104,098	2	TT -> GG	79 -> 80		Substitution	100.0%							2.5E-269			
1,104,105	1,104,106	2	CT -> TC	81 -> 82		Substitution	97.5% -> 97.6%							4.1E-266			
1,104,115	1,104,115	1	A -> C		85	SNP (transversion)	100.0%							3.2E-298			
1,104,120	1,104,120	1	C -> T		86	SNP (transition)	100.0%							1.6E-284			
1,104,128	1,104,128	1	G -> A		85	SNP (transition)	98.8%							5.4E-276			

1,104,147	1,104,147	1	G -> A	81	SNP (transition)	100.0%								7.9E-252			
1,104,149	1,104,149	1	A -> T	82	SNP (transversion)	98.8%								6.5E-250			
1,104,157	1,104,157	1	A -> G	82	SNP (transition)	100.0%								1.6E-279			
1,104,163	1,104,163	1	(G)3 -> (G)2	85	Deletion (tandem repeat)	100.0%								1.0E-136			
1,104,191	1,104,191	1	C -> A	82	SNP (transversion)	98.8%								3.3E-274			
1,104,194	1,104,194	1	T -> G	81	SNP (transversion)	100.0%								6.3E-260			
1,104,210	1,104,210	1	T -> G	76	SNP (transversion)	98.7%								2.4E-261			
1,104,240	1,104,240	1	G	77	Deletion	100.0%								6.3E-124			
1,104,279	1,104,280	2	AT -> GC	82	Substitution	100.0%								2.5E-271			
1,104,288	1,104,287	0	C	83	Insertion	100.0%								6.3E-283			
1,104,297	1,104,297	1	G -> C	79	SNP (transversion)	97.5%								2.4E-251			
1,104,319	1,104,319	1	G -> C	78	SNP (transversion)	100.0%								2.5E-250			
1,104,414	1,104,414	1	C -> G	74	SNP (transversion)	100.0%								1.0E-259			
1,104,488	1,104,489	2	AC -> TT	70	Substitution	100.0%								1.0E-224			
1,104,576	1,104,576	1	G -> C	68	SNP (transversion)	100.0%								1.0E-238			
1,104,627	1,104,627	1	G -> A	67	SNP (transition)	100.0%								1.6E-228			
1,104,642	1,104,642	1	C -> T	69	SNP (transition)	100.0%								3.2E-242			
1,104,651	1,104,651	1	A -> T	69	SNP (transversion)	100.0%								3.2E-242			
1,104,657	1,104,657	1	A -> G	68	SNP (transition)	100.0%								4.0E-225			
1,104,663	1,104,663	1	A -> G	67	SNP (transition)	100.0%								7.9E-222			
1,104,702	1,104,702	1	G -> A	61	SNP (transition)	100.0%								6.3E-196			
1,104,772	1,104,772	1	A -> G	79	SNP (transition)	100.0%								2.0E-261			
1,104,837	1,104,837	1	C -> A	77	SNP (transversion)	97.4%								9.2E-245			
1,104,903	1,104,903	1	C -> T	81	SNP (transition)	100.0%								4.0E-276			
1,104,981	1,104,981	1	A -> G	90	SNP (transition)	100.0%								1.0E-306			
1,104,990	1,104,990	1	G -> A	89	SNP (transition)	97.8%								6.2E-293			
1,105,011	1,105,011	1	A -> C	87	SNP (transversion)	100.0%								7.9E-288			
1,105,061	1,105,061	1	A -> G	88	SNP (transition)	98.9%								3.5E-277			
1,105,073	1,105,073	1	A -> G	86	SNP (transition)	98.8%								8.6E-271			
1,105,198	1,105,198	1	G -> A	94	SNP (transition)	100.0%			84	AAG -> AAA	C770_RS46965	hypothetical protein	None	6.3E-311	28	3	WP_015243221.1
1,105,210	1,105,210	1	C -> T	93	SNP (transition)	100.0%			96	CTC -> CTT	C770_RS46965	hypothetical protein	None	1.3E-307	32	3	WP_015243221.1
1,105,267	1,105,267	1	T -> A	95	SNP (transversion)	100.0%			153	GTT -> GTA	C770_RS46965	hypothetical protein	None	3.2E-314	51	3	WP_015243221.1
1,105,285	1,105,285	1	T -> A	99	SNP (transversion)	99.0%			171	GTT -> GTA	C770_RS46965	hypothetical protein	None	4.0E-322	57	3	WP_015243221.1
1,105,312	1,105,312	1	A -> G	92	SNP (transition)	98.9%			198	ACA -> ACG	C770_RS46965	hypothetical protein	None	4.6E-299	66	3	WP_015243221.1
1,105,331	1,105,331	1	A -> T	89	SNP (transversion)	98.9%		T -> S	217	ACG -> TCG	C770_RS46965	hypothetical protein	Substitution	2.2E-280	73	1	WP_015243221.1
1,105,336	1,105,336	1	G -> A	88	SNP (transition)	100.0%			222	GCG -> GCA	C770_RS46965	hypothetical protein	None	2.5E-282	74	3	WP_015243221.1
1,105,345	1,105,345	1	G -> C	89	SNP (transversion)	100.0%			231	GGG -> GGC	C770_RS46965	hypothetical protein	None	2.0E-294	77	3	WP_015243221.1
1,105,392	1,105,392	1	A -> G	84	SNP (transition)	100.0%		D -> G	278	GAT -> GGT	C770_RS46965	hypothetical protein	Substitution	2.5E-286	93	2	WP_015243221.1
1,105,429	1,105,429	1	T -> C	80	SNP (transition)	98.8%			315	CCT -> CCC	C770_RS46965	hypothetical protein	None	1.3E-251	105	3	WP_015243221.1
1,105,489	1,105,489	1	A -> G	67	SNP (transition)	100.0%			375	AAA -> AAG	C770_RS46965	hypothetical protein	None	1.6E-228	125	3	WP_015243221.1
1,105,489	1,105,489	1	A -> G	67	SNP (transition)	100.0%		M -> V	1	ATG -> GTG	C770_RS46970	hypothetical protein	Substitution	1.6E-228	1	1	WP_041169889.1
1,105,545	1,105,546	2	AT -> GG	63 -> 64	Substitution	100.0%		SS -> SA	57	TCA,TCA -> TCG,(	C770_RS46970	hypothetical protein	Substitution	2.5E-202	19	3	WP_041169889.1
1,105,553	1,105,554	2	TT -> CC	65	Substitution	98.5%		V -> A	65	GTT -> GCC	C770_RS46970	hypothetical protein	Substitution	1.0E-203	22	2	WP_041169889.1
1,105,559	1,105,559	1	C -> T	65	SNP (transition)	100.0%		S -> F	71	TCC -> TTC	C770_RS46970	hypothetical protein	Substitution	3.2E-202	24	2	WP_041169889.1
1,105,569	1,105,569	1	G -> A	66	SNP (transition)	100.0%			81	GCG -> GCA	C770_RS46970	hypothetical protein	None	1.6E-218	27	3	WP_041169889.1
1,105,572	1,105,572	1	G -> C	62	SNP (transversion)	100.0%		E -> D	84	GAG -> GAC	C770_RS46970	hypothetical protein	Substitution	1.6E-211	28	3	WP_041169889.1
1,105,578	1,105,578	1	A -> G	62	SNP (transition)	98.4%			90	CAA -> CAG	C770_RS46970	hypothetical protein	None	2.5E-206	30	3	WP_041169889.1
1,105,586	1,105,586	1	A -> G	63	SNP (transition)	100.0%		D -> G	98	GAT -> GGT	C770_RS46970	hypothetical protein	Substitution	6.3E-215	33	2	WP_041169889.1
1,105,593	1,105,593	1	G -> C	64	SNP (transversion)	100.0%			105	GCG -> GCC	C770_RS46970	hypothetical protein	None	6.3E-212	35	3	WP_041169889.1
1,105,599	1,105,599	1	G -> C	63	SNP (transversion)	100.0%			111	ACG -> ACC	C770_RS46970	hypothetical protein	None	1.3E-208	37	3	WP_041169889.1
1,105,629	1,105,629	1	T -> A	69	SNP (transversion)	97.1%			141	GGT -> GGA	C770_RS46970	hypothetical protein	None	3.7E-225	47	3	WP_041169889.1
1,105,638	1,105,638	1	G -> C	68	SNP (transversion)	98.5%			150	TCG -> TCC	C770_RS46970	hypothetical protein	None	5.4E-220	50	3	WP_041169889.1
1,105,671	1,105,671	1	G -> A	67	SNP (transition)	98.5%			183	AGG -> AGA	C770_RS46970	hypothetical protein	None	1.7E-203	61	3	WP_041169889.1
1,105,677	1,105,677	1	C -> T	66	SNP (transition)	100.0%			189	GAC -> GAT	C770_RS46970	hypothetical protein	None	1.6E-218	63	3	WP_041169889.1
1,105,692	1,105,692	1	C -> A	65	SNP (transversion)	100.0%			204	TCC -> TCA	C770_RS46970	hypothetical protein	None	1.0E-208	68	3	WP_041169889.1
1,105,703	1,105,703	1	T -> C	67	SNP (transition)	100.0%		V -> A	215	GTA -> GCA	C770_RS46970	hypothetical protein	Substitution	4.0E-215	72	2	WP_041169889.1
1,105,713	1,105,713	1	T -> C	68	SNP (transition)	95.6%			225	AGT -> AGC	C770_RS46970	hypothetical protein	None	1.6E-210	75	3	WP_041169889.1
1,105,737	1,105,737	1	A -> G	76	SNP (transition)	100.0%			249	GCA -> GCG	C770_RS46970	hypothetical protein	None	1.6E-251	83	3	WP_041169889.1
1,105,752	1,105,752	1	A -> G	81	SNP (transition)	98.8%			264	AAA -> AAG	C770_RS46970	hypothetical protein	None	8.1E-263	88	3	WP_041169889.1
1,105,758	1,105,758	1	G -> T	79	SNP (transversion)	98.7%		K -> N	270	AAG -> AAT	C770_RS46970	hypothetical protein	Substitution	2.0E-248	90	3	WP_041169889.1
1,105,761	1,105,761	1	C -> G	80	SNP (transversion)	100.0%		D -> E	273	GAC -> GAG	C770_RS46970	hypothetical protein	Substitution	1.0E-272	91	3	WP_041169889.1
1,105,767	1,105,767	1	C -> A	79	SNP (transversion)	98.7%			279	CCC -> CCA	C770_RS46970	hypothetical protein	None	3.1E-256	93	3	WP_041169889.1
1,105,782	1,105,782	1	G -> A	78	SNP (transition)	100.0%			294	GGG -> GGA	C770_RS46970	hypothetical protein	None	6.3E-266	98	3	WP_041169889.1
1,105,784	1,105,785	2	AG -> GA	78	Substitution	100.0%		Q -> R	296	CAG -> CGA	C770_RS46970	hypothetical protein	Substitution	4.0E-258	99	2	WP_041169889.1
1,105,800	1,105,800	1	G -> C	71	SNP (transversion)	100.0%			312	GCG -> GCC	C770_RS46970	hypothetical protein	None	7.9E-221	104	3	WP_041169889.1
1,105,827	1,105,827	1	G -> A	65	SNP (transition)	100.0%			339	TCG -> TCA	C770_RS46970	hypothetical protein	None	3.2E-228	113	3	WP_041169889.1
1,105,869	1,105,869	1	C -> G	55	SNP (transversion)	100.0%			381	CGC -> CGG	C770_RS46970	hypothetical protein	None	1.0E-176	127	3	WP_041169889.1
1,105,899	1,105,899	1	A -> G	46	SNP (transition)	100.0%			411	GAA -> GAG	C770_RS46970	hypothetical protein	None	1.0E-161	137	3	WP_041169889.1
1,105,924	1,105,924	1	A -> G	42	SNP (transition)	100.0%		T -> A	436	ACA -> GCA	C770_RS46970	hypothetical protein	Substitution	1.6E-143	146	1	WP_041169889.1
1,105,932	1,105,932	1	C -> T	41	SNP (transition)	100.0%			444	ATC -> ATT	C770_RS46970	hypothetical protein	None	5.0E-136	148	3	WP_041169889.1
1,105,947	1,105,947	1	C -> A	38	SNP (transversion)	100.0%			459	CCC -> CCA	C770_RS46970	hypothetical protein	None	2.5E-122	153	3	WP_041169889.1
1,105,979	1,105,979	1	T -> C	27	SNP (transition)	100.0%								2.5E-76			
1,105,988	1,105,988	1	T -> C	21	SNP (transition)	100.0%								5.0E-70			
1,105,994	1,105,995	2	AA -> CT	18 -> 19	Substitution	100.0%								1.0E-54			
1,105,998	1,105,998	1	A -> C	16	SNP (transversion)	100.0%								4.0E-55			
1,106,001	1,106,002	2	GT -> AA	16	Substitution	100.0%								4.0E-47			
1,106,004	1,106,006	3	TTA -> GGT	12 -> 14	Substitution	100.0%								2.5E-40			
1,106,011	1,106,011	1	G -> A	8	SNP (transition)	100.0%								1.0E-20			
1,106,014	1,106,015	2	TG -> GT	7	Substitution	100.0%								6.3E-19			
1,106,018	1,106,018	1	G -> T	7	SNP (transversion)	100.0%								5.0E-21			
1,106,269	1,106,269	1	C -> A	6	SNP (transversion)	100.0%								2.5E-22			
1,106,288	1,106,288	1	G -> A	8	SNP (transition)	100.0%								1.0E-28			
1,106,307	1,106,307	1	T -> A	13	SNP (transversion)	100.0%								5.0E-41			
1,106,311	1,106,311	1	G -> T	13	SNP (transversion)	100.0%								2.5E-42			
1,106,332	1,106,332	1	T -> G	17	SNP (transversion)	100.0%								1.6E-58			
1,106,352	1,106,352	1	G -> C	26	SNP (transversion)	100.0%								1.6E-86			
1,106,354	1,106,354	1	T -> C	28	SNP (transition)	100.0%								1.0E-84			
1,106,363	1,106,363	1	T -> G	31	SNP (transversion)	100.0%								1.0E-93			
1,106,368	1,106,368	1	T -> G	32	SNP (transversion)	100.0%								4.0E-103			
1,106,380	1,106,381	2	GG -> AT	34	Substitution	97.1%								4.3E-108			
1,106,386	1,106,387	2	TG -> CA	34	Substitution	100.0%								1.6E-109			
1,106,394	1,106,394	1	C -> AT	37	Insertion	100.0%								7.9E-123			
1,106,404	1,106,404	1	G -> T	43	SNP (transversion)	100.0%								1.3E-142			
1,106,412	1,106,413	2	TT	46	Deletion	100.0%								1.0E-69			
1,106,417	1,106,417	1	G -> A	47	SNP (transition)	100.0%								4.0E-151			
1,106,422	1,106,421	0	(C)2 -> (C)3	47	Insertion (tandem repeat)	100.0%								4.0E-151			
1,106,445	1,106,445	1	C -> T	54	SNP (transition)	100.0%								2.5E-184			
1,106,449	1,106,449	1	T -> C	55	SNP (transition)	98.2%								5.5E-188			
1,106,451	1,106,451	1	G -> A	55	SNP (transition)	98.2%								3.5E-177			
1,106,456	1,106,456	1	T -> C	58	SNP (transition)	100.0%								4.0E-192			
1,106,459	1,106,458	0	(C)2 -> (C)3	58	Insertion (tandem repeat)	100.0%								4.0E-192			
1,106,481	1,106,481	1	C -> T	61	SNP (transition)	100.0%								3.2E-214			
1,106,492	1,106,492	1	A -> G	63	SNP (transition)	98.4%								1.6E-203			
1,106,503	1,106,503	1	A -> T	69	SNP (transversion)	100.0%								3.2E-242			
1,106,515	1,106,515	1	T -> A	73	SNP (transversion)	98.6%								7.3E-251			
1,106,517	1,106,517	1	A -> T	73	SNP (transversion)	100.0%								6.3E-249			
1,106,547	1,106,547	1	C -> A	77	SNP (transversion)	98.7%								1.2E-249			
1,106,556	1,106,556	1	T -> C	80	SNP (transition)	100.0%								1.0E-272			
1,106,566	1,106,566	1	G	82	Deletion	100.0%								6.3E-132			
1,106,579	1,106,580	2	GC -> AT	85	Substitution	98.8%								5.4E-276			
1,106,588	1,106,587	0	GGC	80 -> 81	Insertion	96.3%								8.1E-263			
1,106,594	1,106,594	1	C -> G	86	SNP (transversion)	100.0%								1.6E-284			
1,106,616	1,106,616	1	T -> G	85	SNP (transversion)	100.0%								1.0E-272			
1,106,618	1,106,618	1	T -> C	86	SNP (transition)	100.0%								6.3E-276			
1,106,621	1,106,621	1	C -> T	87	SNP (transition)	100.0%								7.9E-288			
1,106,625	1,106,625	1	T -> A	85	SNP (transversion)	100.0%								1.0E-289			
1,106,631	1,106,630	0	C	82	Insertion	98.8%								3.3E-274			
1,106,635	1,106,634	0	T	82	Insertion	100.0%								2.5E-271			
1,106,656	1,106,656	1	C -> A	86	SNP (transversion)	98.8%								2.7E-262			
1,106,661	1,106,662	2	GG -> CA	88	Substitution	100.0%								4.0E-291			
1,106,668	1,106,668	1	A -> G	83	SNP (transition)	98.8%								2.1E-269			
1,106,670	1,106,670	1	A -> T	84	SNP (transversion)	98.8%								4.2E-256			
1,106,702	1,106,702	1	A -> C	79	SNP (transversion)	100.0%								2.0E-261			
1,106,752	1,106,752	1	A -> G	86	SNP (transition)	100.0%								2.5E-267			
1,106,766	1,106,766	1	T -> C	84	SNP (transition)	100.0%								4.0E-261			
1,106,870	1,106,870	1	T -> C	73	SNP (transition)	100.0%								2.5E-234			
1,106,877	1,106,877	1	C -> A	74	SNP (transversion)	98.6%								9.3E-240			
1,106,882	1,106,882	1	C -> T	74	SNP (transition)	100.0%								1.6E-237			
1,106,892	1,106,892	1	G -> A	76	SNP (transition)	100.0%								6.3E-244			
1,106,944	1,106,944	1	T -> G	77	SNP (transversion)	98.7%								1.2E-249			
1,106,955	1,106,954	0	(GG)3 -> (GG)4	78 -> 79	Insertion (tandem repeat)	98.7%								2.0E-248			
1,106,962	1,106,962	1	G -> C	80	SNP (transversion)	98.8%								1.3E-251			
1,106,987	1,106,987	1	C -> G	82	SNP (transversion)	100.0%								1.6E-279			
1,107,031	1,107,031	1	C -> T	82	SNP (transition)	100.0%								4.0E-263			
1,107,036	1,107,036	1	G -> C	84	SNP (transversion)	100.0%								4.0E-261			
1,107,061	1,107,061	1	A -> G	84	SNP (transition)	98.8%								1.1E-272			
1,107,099	1,107,099	1	A -> G	84	SNP (transition)	96.4%								7.6E-247			
1,107,124	1,107,124	1	C -> A	79	SNP (transversion)	98.7%								3.1E-256			
1,107,239	1,107,239	1	G -> C	88	SNP (transversion)	98.9%								3.5E-277			
1,107,260	1,107,260	1	G -> C	86	SNP (transversion)	98.8%		M -> I	3	ATG -> ATC	C770_RS46975	hypothetical protein	Substitution	8.6E-288	1	3	WP_041169890.1
1,107,293	1,107,293	1	G	86	Deletion	98.8%			36		C770_RS46975	hypothetical protein	Frame Shift	8.3E-118	12	3	WP_041169890.1
1,107,316	1,107,317	2	GA -> TC	84 -> 85	Substitution	96.4% -> 96.5%		R -> L	59	CGA -> CTC	C770_RS46975	hypothetical protein	Substitution	1.3E-267	20	2	WP_041169890.1
1,107,321	1,107,320	0	(C)2 -> (C)3	86	Insertion (tandem repeat)	98.8%			64		C770_RS46975	hypothetical protein	Frame Shift	2.7E-262	22	1	WP_041169890.1
1,107,369	1,107,369	1	G -> T	86	SNP (transversion)	100.0%		V -> F	112	GTT -> TTT	C770_RS46975	hypothetical protein	Substitution	2.5E-267	38	1	WP_041169890.1
1,107,388	1,107,387	0	G	93	Insertion	97.8%			131		C770_RS46975	hypothetical protein	Frame Shift	1.7E-306	44	2	WP_041169890.1
1,107,476	1,107,476	1	G -> C	82	SNP (transversion)	100.0%								2.5E-271			
1,107,505	1,107,505	1	C -> G	83	SNP (transversion)	100.0%								5.0E-258			
1,107,520	1,107,520	1	C -> G	90	SNP (transversion)	98.9%								1.8E-292			
1,107,557	1,107,557	1	G -> C	94	SNP (transversion)	100.0%								1.6E-301			
1,107,607	1,107,607	1	(C)4 -> (C)3	93	Deletion (tandem repeat)	100.0%			198		C770_RS46980	hypothetical protein	Frame Shift	6.3E-131	66	3	WP_015243224.1
1,107,623	1,107,624	2	TC	92	Deletion	100.0%			181		C770_RS46980	hypothetical protein	Frame Shift	1.6E-129	61	1	WP_015243224.1
1,107,627	1,107,627	1	C -> G	91	SNP (transversion)	97.8%		D -> H	178	GAT -> CAT	C770_RS46980	hypothetical protein	Substitution	6.5E-282	60	1	WP_015243224.1
1,107,722	1,107,722	1	T -> C	85	SNP (transition)	100.0%		D -> G	83	GAC -> GGC	C770_RS46980	hypothetical protein	Substitution	3.2E-281	28	2	WP_015243224.1
1,107,740	1,107,740	1	T -> C	93	SNP (transition)	100.0%		N -> S	65	AAC -> AGC	C770_RS46980	hypothetical protein	Substitution	6.3E-317	22	2	WP_015243224.1
1,107,759	1,107,759	1	C -> T	89	SNP (transition)	98.9%		G -> R	46	GGG -> AGG	C770_RS46980	hypothetical protein	Substitution	1.4E-271	16	1	WP_015243224.1
1,107,769	1,107,769	1	A -> G	95	SNP (transition)	100.0%			36	GGT -> GGC	C770_RS46980	hypothetical protein	None	3.2E-295	12	3	WP_015243224.1
1,107,805	1,107,805	1	T -> C	89	SNP (transition)	100.0%								2.0E-294			
1,107,964	1,107,964	1	C -> T	83	SNP (transition)	100.0%			102	GTC -> GTT	C770_RS46985	hypothetical protein	None	6.3E-283	34	3	WP_013850850.1
1,108,066	1,108,066	1	C -> A	77	SNP (transversion)	100.0%								1.6E-262			
1,108,091	1,108,090	0	(C)2 -> (C)3	78	Insertion (tandem repeat)	100.0%								2.5E-250			

1,108,159	1,108,159	1	G -> C		71	SNP (transversion)	100.0%							4.0E-242			
1,108,614	1,108,614	1	T -> A		80	SNP (transversion)	98.8%	F -> I	10	TTT -> ATT	C770_RS46990	hypothetical protein	Substitution	1.6E-259	4	1	WP_015243225.1
1,108,802	1,108,802	1	T -> C		81	SNP (transition)	98.8%		198	ATT -> ATC	C770_RS46990	hypothetical protein	None	8.1E-271	66	3	WP_015243225.1
1,108,980	1,108,980	1	G -> A		80	SNP (transition)	98.8%	D -> N	376	GAC -> AAC	C770_RS46990	hypothetical protein	Substitution	2.5E-275	126	1	WP_015243225.1
1,108,988	1,108,988	1	G -> A		83	SNP (transition)	97.6%	M -> I	384	ATG -> ATA	C770_RS46990	hypothetical protein	Substitution	1.7E-264	128	3	WP_015243225.1
1,109,104	1,109,104	1	G -> T		89	SNP (transversion)	100.0%							1.3E-276			
1,109,129	1,109,129	1	C -> G		88	SNP (transversion)	97.7%							6.1E-281			
1,109,152	1,109,151	0	(G)2 -> (G)3		90	Insertion (tandem repeat)	100.0%							1.0E-297			
1,109,284	1,109,284	1	C -> T		88	SNP (transition)	100.0%							1.6E-273			
1,109,287	1,109,287	1	A -> T		85	SNP (transversion)	100.0%							3.2E-264			
1,109,306	1,109,306	1	G -> T		77	SNP (transversion)	100.0%							7.9E-255			
1,109,320	1,109,320	1	A -> C		75	SNP (transversion)	100.0%							3.2E-248			
1,109,331	1,109,331	1	A -> G		78	SNP (transition)	98.7%							6.2E-253			
1,109,343	1,109,343	1	C -> T		77	SNP (transition)	100.0%							1.6E-262			
1,109,363	1,109,363	1	T -> A		73	SNP (transversion)	100.0%							3.2E-256			
1,109,367	1,109,366	0	C		70	Insertion	100.0%							1.0E-238			
1,109,385	1,109,385	1	C -> T		68	SNP (transition)	100.0%	G -> S	304	GGC -> AGC	C770_RS46995	antibiotic biosynthesis r	Substitution	4.0E-225	102	1	WP_010975476.1
1,109,650	1,109,650	1	C -> G		73	SNP (transversion)	100.0%		39	GGG -> GGC	C770_RS46995	antibiotic biosynthesis r	None	2.5E-234	13	3	WP_010975476.1
1,109,842	1,109,842	1	C -> G		75	SNP (transversion)	100.0%	D -> H	1.45	GAC -> CAC	C770_RS47000	Sugar (pentulose and he	Substitution	1.0E-255	484	1	WP_015243226.1
1,109,912	1,109,912	1	C -> T		75	SNP (transition)	100.0%		1.38	CTG -> CTA	C770_RS47000	Sugar (pentulose and he	None	1.0E-255	460	3	WP_015243226.1
1,109,921	1,109,921	1	G -> C		74	SNP (transversion)	100.0%		1.371	GGC -> GGG	C770_RS47000	Sugar (pentulose and he	None	6.3E-245	457	3	WP_015243226.1
1,109,939	1,109,939	1	C -> G		81	SNP (transversion)	100.0%		1.353	GCG -> GCC	C770_RS47000	Sugar (pentulose and he	None	6.3E-260	451	3	WP_015243226.1
1,109,963	1,109,963	1	T -> C		80	SNP (transition)	100.0%		1.329	AAA -> AAG	C770_RS47000	Sugar (pentulose and he	None	1.0E-280	443	3	WP_015243226.1
1,109,978	1,109,978	1	G -> T		78	SNP (transversion)	100.0%		1.314	GTC -> GTA	C770_RS47000	Sugar (pentulose and he	None	4.0E-258	438	3	WP_015243226.1
1,110,001	1,110,001	1	T -> C		77	SNP (transition)	100.0%	N -> D	1.291	AAT -> GAT	C770_RS47000	Sugar (pentulose and he	Substitution	1.6E-262	431	1	WP_015243226.1
1,110,044	1,110,044	1	C -> A		77	SNP (transversion)	98.7%		1.248	GCG -> GCT	C770_RS47000	Sugar (pentulose and he	None	3.1E-257	416	3	WP_015243226.1
1,110,059	1,110,059	1	A -> G		80	SNP (transition)	98.8%		1.233	GAT -> GAC	C770_RS47000	Sugar (pentulose and he	None	3.2E-283	411	3	WP_015243226.1
1,110,122	1,110,122	1	G -> A		76	SNP (transition)	98.7%		1.17	AAC -> AAT	C770_RS47000	Sugar (pentulose and he	None	7.6E-254	390	3	WP_015243226.1
1,110,170	1,110,170	1	G -> T		77	SNP (transversion)	100.0%	D -> E	1.122	GAC -> GAA	C770_RS47000	Sugar (pentulose and he	Substitution	7.9E-255	374	3	WP_015243226.1
1,110,176	1,110,176	1	A -> G		76	SNP (transition)	100.0%		1.116	TCT -> TCC	C770_RS47000	Sugar (pentulose and he	None	6.3E-244	372	3	WP_015243226.1
1,110,341	1,110,341	1	G -> A		63	SNP (transition)	100.0%		951	GGC -> GGT	C770_RS47000	Sugar (pentulose and he	None	6.3E-215	317	3	WP_015243226.1
1,110,622	1,110,622	1	T -> C		69	SNP (transition)	97.1%	K -> E	670	AAG -> GAG	C770_RS47000	Sugar (pentulose and he	Substitution	3.7E-225	224	1	WP_015243226.1
1,110,626	1,110,626	1	A -> G		68	SNP (transition)	97.1%		666	GTT -> GTC	C770_RS47000	Sugar (pentulose and he	None	9.1E-222	222	3	WP_015243226.1
1,110,661	1,110,661	1	G -> C		70	SNP (transversion)	98.6%	Q -> E	631	CAA -> GAA	C770_RS47000	Sugar (pentulose and he	Substitution	1.8E-233	211	1	WP_015243226.1
1,110,746	1,110,746	1	G -> C		70	SNP (transversion)	97.1%		546	GGC -> GGG	C770_RS47000	Sugar (pentulose and he	None	9.6E-222	182	3	WP_015243226.1
1,110,773	1,110,773	1	A -> G		74	SNP (transition)	98.6%		519	GTT -> GTC	C770_RS47000	Sugar (pentulose and he	None	2.3E-254	173	3	WP_015243226.1
1,110,819	1,110,819	1	C -> T		77	SNP (transition)	97.4%	R -> K	473	AGG -> AAG	C770_RS47000	Sugar (pentulose and he	Substitution	9.2E-245	158	2	WP_015243226.1
1,110,824	1,110,824	1	G -> A		74	SNP (transition)	98.6%		468	ATC -> ATT	C770_RS47000	Sugar (pentulose and he	None	9.3E-240	156	3	WP_015243226.1
1,110,827	1,110,827	1	G -> C		76	SNP (transversion)	98.7%		465	CGC -> CGG	C770_RS47000	Sugar (pentulose and he	None	2.4E-246	155	3	WP_015243226.1
1,110,839	1,110,839	1	T -> G		76	SNP (transversion)	98.7%	E -> D	453	GAA -> GAC	C770_RS47000	Sugar (pentulose and he	Substitution	2.4E-261	151	3	WP_015243226.1
1,110,941	1,110,941	1	G -> T		85	SNP (transversion)	96.5%		351	CGC -> CGA	C770_RS47000	Sugar (pentulose and he	None	2.5E-266	117	3	WP_015243226.1
1,110,946	1,110,946	1	G -> T		88	SNP (transversion)	98.9%	Q -> K	346	CAG -> AAG	C770_RS47000	Sugar (pentulose and he	Substitution	7.0E-286	116	1	WP_015243226.1
1,110,974	1,110,974	1	A -> G		85	SNP (transition)	100.0%		318	AGT -> AGC	C770_RS47000	Sugar (pentulose and he	None	3.2E-281	106	3	WP_015243226.1
1,110,998	1,110,998	1	A -> G		86	SNP (transition)	100.0%		294	ATT -> ATC	C770_RS47000	Sugar (pentulose and he	None	2.5E-310	98	3	WP_015243226.1
1,111,014	1,111,014	1	C -> T		82	SNP (transition)	100.0%	R -> H	278	CGC -> CAC	C770_RS47000	Sugar (pentulose and he	Substitution	1.6E-279	93	2	WP_015243226.1
1,111,037	1,111,037	1	A -> G		82	SNP (transition)	100.0%		255	CGT -> CGC	C770_RS47000	Sugar (pentulose and he	None	1.6E-279	85	3	WP_015243226.1
1,111,136	1,111,136	1	C -> T		73	SNP (transition)	98.6%		156	GAG -> GAA	C770_RS47000	Sugar (pentulose and he	None	2.9E-229	52	3	WP_015243226.1
1,111,214	1,111,214	1	G -> A		76	SNP (transition)	98.7%		78	AAC -> AAT	C770_RS47000	Sugar (pentulose and he	None	2.4E-246	26	3	WP_015243226.1
1,111,229	1,111,229	1	A -> G		75	SNP (transition)	100.0%		63	TTT -> TTC	C770_RS47000	Sugar (pentulose and he	None	1.0E-255	21	3	WP_015243226.1
1,111,262	1,111,262	1	G -> A		73	SNP (transition)	97.3%		30	GAC -> GAT	C770_RS47000	Sugar (pentulose and he	None	1.0E-238	10	3	WP_015243226.1
1,111,283	1,111,284	2	AG -> GA	71 -> 72		Substitution	100.0%	T -> I	8	ACT -> ATC	C770_RS47000	Sugar (pentulose and he	Substitution	5.0E-235	3	2	WP_015243226.1
1,111,363	1,111,363	1	C -> G		69	SNP (transversion)	100.0%	G -> A	881	GGA -> GCA	C770_RS47005	DeoR family transcriptio	Substitution	3.2E-242	294	2	WP_013850854.1
1,111,716	1,111,716	1	G -> A		69	SNP (transition)	100.0%		528	GTC -> GTT	C770_RS47005	DeoR family transcriptio	None	2.5E-235	176	3	WP_013850854.1
1,111,755	1,111,755	1	G -> A		67	SNP (transition)	100.0%		489	AAC -> AAT	C770_RS47005	DeoR family transcriptio	None	7.9E-222	163	3	WP_013850854.1
1,111,761	1,111,761	1	C -> G		72	SNP (transversion)	100.0%		483	ACG -> ACC	C770_RS47005	DeoR family transcriptio	None	1.6E-245	161	3	WP_013850854.1
1,111,782	1,111,782	1	C -> G		71	SNP (transversion)	100.0%		462	ACG -> ACC	C770_RS47005	DeoR family transcriptio	None	4.0E-242	154	3	WP_013850854.1
1,111,815	1,111,815	1	G -> A		76	SNP (transition)	100.0%		429	ATC -> ATT	C770_RS47005	DeoR family transcriptio	None	1.0E-266	143	3	WP_013850854.1
1,112,028	1,112,028	1	T -> C		85	SNP (transition)	100.0%		216	GCA -> GCG	C770_RS47005	DeoR family transcriptio	None	1.0E-289	72	3	WP_013850854.1
1,112,037	1,112,037	1	A -> G		83	SNP (transition)	100.0%		207	GGT -> GGC	C770_RS47005	DeoR family transcriptio	None	1.3E-274	69	3	WP_013850854.1
1,112,247	1,112,247	1	T -> A		77	SNP (transversion)	97.4%							2.9E-237			
1,112,359	1,112,359	1	T -> G		86	SNP (transversion)	100.0%							1.6E-284			
1,112,367	1,112,369	3	TCG -> CGA	86 -> 87		Substitution	97.7%							1.2E-277			
1,112,371	1,112,371	1	A -> T		87	SNP (transversion)	97.7%							1.2E-277			
1,112,373	1,112,376	4	CCTG -> GAGA		86	Substitution	100.0%							1.6E-284			
1,112,384	1,112,384	1	G -> A		86	SNP (transition)	100.0%							4.0E-293			
1,112,395	1,112,395	1	A -> G		87	SNP (transition)	100.0%							3.2E-305			
1,112,412	1,112,412	1	C -> G		82	SNP (transversion)	97.6%							3.3E-253			
1,112,439	1,112,439	1	A -> C		68	SNP (transversion)	100.0%							1.0E-238			
1,112,453	1,112,453	1	C -> A		71	SNP (transversion)	98.6%							7.1E-237			
1,112,584	1,112,584	1	G -> A		76	SNP (transition)	98.7%							2.4E-246			
1,112,807	1,112,807	1	C -> T		57	SNP (transition)	100.0%							1.0E-171			
1,112,835	1,112,835	1	A -> C		65	SNP (transversion)	96.9%							6.6E-218			
1,112,945	1,112,945	1	C -> T		72	SNP (transition)	100.0%							1.6E-245			
1,112,951	1,112,951	1	G -> A		72	SNP (transition)	100.0%							1.0E-252			
1,113,002	1,113,002	1	G -> C		85	SNP (transversion)	100.0%							1.0E-272			
1,113,041	1,113,041	1	T -> C		90	SNP (transition)	98.9%							1.8E-292			
1,113,047	1,113,047	1	C -> T		92	SNP (transition)	97.8%							4.2E-303			
1,113,053	1,113,053	1	C -> T		92	SNP (transition)	97.8%							4.2E-303			
1,113,062	1,113,062	1	A -> C		92	SNP (transversion)	98.9%							2.9E-317			
1,113,080	1,113,081	2	AT -> GC		90	Substitution	100.0%							1.0E-315			
1,113,089	1,113,089	1	A -> G		92	SNP (transition)	100.0%							1.6E-313			
1,113,101	1,113,101	1	C -> T		91	SNP (transition)	100.0%							4.0E-310			
1,113,110	1,113,110	1	G -> A		87	SNP (transition)	100.0%							4.0E-279			
1,113,399	1,113,398	0	C		83	Insertion	100.0%							3.2E-291			
1,113,445	1,113,445	1	A -> G		76	SNP (transition)	100.0%							1.0E-266			
1,113,454	1,113,454	1	A -> G		74	SNP (transition)	100.0%							1.0E-259			
1,113,463	1,113,463	1	A -> G		75	SNP (transition)	100.0%							3.2E-263			
1,113,526	1,113,526	1	A -> G		78	SNP (transition)	98.7%							3.1E-245			
1,113,529	1,113,529	1	A -> C		78	SNP (transversion)	98.7%							6.2E-253			
1,113,568	1,113,568	1	C -> G		73	SNP (transversion)	98.6%							1.8E-236			
1,113,616	1,113,616	1	A -> G		72	SNP (transition)	98.6%							3.6E-233			
1,113,769	1,113,769	1	G -> C		73	SNP (transversion)	98.6%							7.3E-251			
1,113,875	1,113,875	1	G -> C		73	SNP (transversion)	98.6%							1.2E-243			
1,113,913	1,113,913	1	G -> A		71	SNP (transition)	97.2%							3.9E-218			
1,113,931	1,113,931	1	G -> A		70	SNP (transition)	98.6%							1.8E-233			
1,114,019	1,114,019	1	G -> C		82	SNP (transversion)	100.0%	M -> I	42	ATG -> ATC	C770_RS47015	sugar ABC transporter p	Substitution	1.6E-279	14	3	WP_015243227.1
1,114,062	1,114,062	1	C -> T		84	SNP (transition)	100.0%	P -> S	85	CCC -> TCC	C770_RS47015	sugar ABC transporter p	Substitution	2.5E-286	29	1	WP_015243227.1
1,114,085	1,114,085	1	C -> G		86	SNP (transversion)	98.8%		108	ACC -> ACG	C770_RS47015	sugar ABC transporter p	None	2.7E-279	36	3	WP_015243227.1
1,114,097	1,114,097	1	A -> T		86	SNP (transversion)	100.0%		120	GTA -> GTT	C770_RS47015	sugar ABC transporter p	None	2.5E-267	40	3	WP_015243227.1
1,114,175	1,114,175	1	G -> C		89	SNP (transversion)	100.0%		198	TCG -> TCC	C770_RS47015	sugar ABC transporter p	None	1.6E-285	66	3	WP_015243227.1
1,114,204	1,114,204	1	C -> T		93	SNP (transition)	98.9%	A -> V	227	GCG -> GTG	C770_RS47015	sugar ABC transporter p	Substitution	2.3E-302	76	2	WP_015243227.1
1,114,299	1,114,299	1	C -> T		78	SNP (transition)	98.7%		322	CTG -> TTG	C770_RS47015	sugar ABC transporter p	None	2.5E-268	108	1	WP_015243227.1
1,114,349	1,114,349	1	A -> G		80	SNP (transition)	98.8%		372	ACA -> ACG	C770_RS47015	sugar ABC transporter p	None	1.6E-259	124	3	WP_015243227.1
1,114,355	1,114,355	1	T -> A		81	SNP (transversion)	98.8%		378	GGT -> GGA	C770_RS47015	sugar ABC transporter p	None	8.1E-263	126	3	WP_015243227.1
1,114,358	1,114,358	1	A -> T		81	SNP (transversion)	98.8%		381	CTA -> CTT	C770_RS47015	sugar ABC transporter p	None	8.1E-271	127	3	WP_015243227.1
1,114,382	1,114,382	1	C -> T		79	SNP (transition)	98.7%		405	CTC -> CTT	C770_RS47015	sugar ABC transporter p	None	5.0E-264	135	3	WP_015243227.1
1,114,388	1,114,388	1	T -> C		80	SNP (transition)	98.8%		411	GGT -> GGC	C770_RS47015	sugar ABC transporter p	None	2.5E-275	137	3	WP_015243227.1
1,114,418	1,114,418	1	A -> T		79	SNP (transversion)	98.7%		441	GGA -> GGT	C770_RS47015	sugar ABC transporter p	None	7.9E-272	147	3	WP_015243227.1
1,114,436	1,114,436	1	G -> C		73	SNP (transversion)	98.6%		459	GCG -> GCC	C770_RS47015	sugar ABC transporter p	None	7.3E-251	153	3	WP_015243227.1
1,114,451	1,114,451	1	T -> C		71	SNP (transition)	98.6%		474	GGT -> GGC	C770_RS47015	sugar ABC transporter p	None	7.1E-244	158	3	WP_015243227.1
1,114,472	1,114,472	1	C -> T		66	SNP (transition)	98.5%		495	GTC -> GTT	C770_RS47015	sugar ABC transporter p	None	6.6E-220	165	3	WP_015243227.1
1,114,501	1,114,501	1	C -> T		64	SNP (transition)	98.4%	T -> I	524	ACC -> ATC	C770_RS47015	sugar ABC transporter p	Substitution	4.0E-213	175	2	WP_015243227.1
1,114,513	1,114,513	1	C -> A		64	SNP (transversion)	95.3%	T -> K	536	ACG -> AAG	C770_RS47015	sugar ABC transporter p	Substitution	2.6E-191	179	2	WP_015243227.1
1,114,520	1,114,520	1	T -> C		67	SNP (transition)	98.5%		543	CGT -> CGC	C770_RS47015	sugar ABC transporter p	None	6.7E-230	181	3	WP_015243227.1
1,114,526	1,114,526	1	T -> C		71	SNP (transition)	98.6%		549	GGT -> GGC	C770_RS47015	sugar ABC transporter p	None	7.1E-223	183	3	WP_015243227.1
1,114,531	1,114,531	1	G -> A		73	SNP (transition)	98.6%	S -> N	554	AGT -> AAT	C770_RS47015	sugar ABC transporter p	Substitution	7.3E-215	185	2	WP_015243227.1
1,114,553	1,114,553	1	T -> C		78	SNP (transition)	100.0%		576	AAT -> AAC	C770_RS47015	sugar ABC transporter p	None	4.0E-258	192	3	WP_015243227.1
1,114,562	1,114,562	1	C -> T		77	SNP (transition)	100.0%		585	GCC -> GCT	C770_RS47015	sugar ABC transporter p	None	7.9E-255	195	3	WP_015243227.1
1,114,579	1,114,580	2	TA -> CG		69	Substitution	97.1%	V -> A	602	GTA -> GCG	C770_RS47015	sugar ABC transporter p	Substitution	3.7E-225	201	2	WP_015243227.1
1,114,603	1,114,603	1	C -> T		69	SNP (transition)	100.0%	S -> F	626	TCC -> TTC	C770_RS47015	sugar ABC transporter p	Substitution	1.3E-214	209	2	WP_015243227.1
1,114,607	1,114,607	1	G -> T		68	SNP (transversion)	100.0%		630	GCG -> GCT	C770_RS47015	sugar ABC transporter p	None	1.6E-211	210	3	WP_015243227.1
1,114,635	1,114,635	1	T -> C		69	SNP (transition)	98.6%	F -> L	658	TTC -> CTC	C770_RS47015	sugar ABC transporter p	Substitution	2.7E-223	220	1	WP_015243227.1
1,114,703	1,114,703	1	A -> G		70	SNP (transition)	100.0%		726	CTA -> CTG	C770_RS47015	sugar ABC transporter p	None	1.0E-238	242	3	WP_015243227.1
1,114,712	1,114,712	1	C -> T		71	SNP (transition)	98.6%		735	ATC -> ATT	C770_RS47015	sugar ABC transporter p	None	7.1E-237	245	3	WP_015243227.1
1,114,715	1,114,715	1	C -> G		72	SNP (transversion)	98.6%		738	GCC -> GCG	C770_RS47015	sugar ABC transporter p	None	3.6E-233	246	3	WP_015243227.1
1,114,724	1,114,724	1	G -> A		69	SNP (transition)	100.0%		747	GTG -> GTA	C770_RS47015	sugar ABC transporter p	None	3.2E-242	249	3	WP_015243227.1
1,114,733	1,114,733	1	C -> T		69	SNP (transition)	100.0%		756	GGC -> GGT	C770_RS47015	sugar ABC transporter p	None	3.2E-242	252	3	WP_015243227.1
1,114,754	1,114,754	1	G -> C		67	SNP (transversion)	100.0%		777	ACG -> ACC	C770_RS47015	sugar ABC transporter p	None	7.9E-222	259	3	WP_015243227.1
1,114,766	1,114,766	1	T -> A		67	SNP (transversion)	100.0%		789	GCT -> GCA	C770_RS47015	sugar ABC transporter p	None	3.2E-235	263	3	WP_015243227.1
1,114,856	1,114,856	1	A -> G		63	SNP (transition)	100.0%		879	GCA -> GCG	C770_RS47015	sugar ABC transporter p	None	2.0E-183	293	3	WP_015243227.1
1,114,871	1,114,871	1	C -> T		67	SNP (transition)	100.0%		894	CTC -> CTT	C770_RS47015	sugar ABC transporter p	None	4.0E-215	298	3	WP_015243227.1
1,114,898	1,114,898	1	C -> A		65	SNP (transversion)	98.5%		921	ATC -> ATA	C770_RS47015	sugar ABC transporter p	None	1.0E-203	307	3	WP_015243227.1
1,114,965	1,114,965	1	T -> C		68	SNP (transition)	98.5%	S -> P	988	TCC -> CCC	C770_RS47015	sugar ABC transporter p	Substitution	5.4E-220	330	1	WP_015243227.1
1,114,982	1,114,982	1	G -> A		68	SNP (transition)	98.5%		1.005	ACG -> ACA	C770_RS47015	sugar ABC transporter p	None	1.1E-226	335	3	WP_015243227.1
1,115,001	1,115,002	2	GT -> AG	67 -> 68		Substitution	100.0%	V -> S	1.024	GTC -> AGC	C770_RS47015	sugar ABC transporter p	Substitution	7.9E-222	342	1	WP_015243227.1
1,115,005	1,115,005	1	G -> T		69	SNP (transversion)	100.0%	R -> L	1.028	CGA -> CTA	C770_RS47015	sugar ABC transporter p	Substitution	3.2E-242	343	2	WP_015243227.1
1,115,011	1,115,011	1	A -> G		70	SNP (transition)	100.0%							1.0E-245			
1,115,031	1,115,031	1	(G)4 -> (G)3		71	Deletion (tandem repeat)	100.0%							2.5E-114			
1,115,067	1,115,067	1	C -> G		73	SNP (transversion)	100.0%	L -> V	19	CTC -> GTC	C770_RS47020	Ribose/xylose/arabinos	Substitution	3.2E-256	7	1	WP_015243228.1
1,115,123	1,115,123	1	C -> G		79	SNP (transversion)	97.5%		75	GGC -> GGG	C770_RS47020	Ribose/xylose/arabinos	None	1.2E-243	25	3	WP_015243228.1
1,115,136	1,115,136	1	A -> G		81	SNP (transition)	100.0%	I -> V	88	ATT -> GTT	C770_RS47020	Ribose/xylose/arabinos	Substitution	3.2E-284	30	1	WP_015243228.1
1,115,180	1,115,180	1	C -> T		79	SNP (transition)	100.0%		132	ATC -> ATT	C770_RS47020	Ribose/xylose/arabinos	None	2.5E-269	44	3	WP_015243228.1
1,115,192	1,115,192	1	C -> T		75	SNP (transition)	100.0%		144	ATC -> ATT	C770_RS47020	Ribose/xylose/arabinos	None	3.2E-248	48	3	WP_015243228.1
1,115,198	1,115,198	1	C -> G		77	SNP (transversion)	98.7%		150	GTC -> GTG	C770_RS47020	Ribose/xylose/arabinos	None	3.1E-257	50	3	WP_015243228.1
1,115,213	1,115,213	1	G -> A		83	SNP (transition)	100.0%		165	GTG -> GTA	C770_RS47020	Ribose/xylose/arabinos	None	1.3E-274	55	3	WP_015243228.1
1,115,219	1,115,219	1	C -> A		83	SNP (transversion)	97.6%		171	GGC -> GGA	C770_RS47020	Ribose/xylose/arabinos	None	1.7E-264	57	3	WP_015243228.1
1,115,223	1,115,223	1	A -> G		83	SNP (transition)	97.6%	T -> A	175	ACT -> GCT	C770_RS47020	Ribose/xylose/arabinos	Substitution	1.4E-272	59	1	WP_015243228.1
1,115,288	1,115,289	2	TC -> CT		88	Substitution	100.0%		240	GCT,CTG -> GCC,	C770_RS47020	Ribose/xylose/arabinos	None	4.0E-291	80	3	WP_015243228.1
1,115,294	1,115,294	1	T -> C		90	SNP (transition)	98.9%		246	GTT -> GTC	C770_RS47020	Ribose/xylose/arabinos	None	2.3E-301	82	3	WP_015243228.1
1,115,330	1,115,330	1	G -> C		82	SNP (transversion)	100.0%		282	GTG -> GTC	C770_RS47020	Ribose/xylose/arabinos	None	1.0E-287	94	3	WP_015243228.1
1,115,375	1,115,375	1	T -> C		84	SNP (transition)	100.0%		327	AGT -> AGC	C770_RS47020	Ribose/xylose/arabinos	None	6.3E-278	109	3	WP_015243228.1
1,115,384	1,115,384	1	T -> C		89	SNP (transition)	98.9%		336	CTT -> CTC	C770_RS47020	Ribose/xylose/arabinos	None	5.6E-298	112	3	WP_015243228.1
1,115,435	1,115,435	1	C -> T		92	SNP (transition)	98.9%		387	TAC -> TAT	C770_RS47020	Ribose/xylose/arabinos	None	2.9E-317	129	3	WP_015243228.1
1,115,470	1,115,471	2	AA -> CC	90 -> 91		Substitution	98.9%	E -> A	422	GAA -> GCC	C770_RS47020	Ribose/xylose/arabinos	Substitution	2.3E-301	141	2	WP_015243228.1
1,115,501	1,115,501	1	A -> G		86	SNP (transition)	98.8%		453	CCA -> CCG	C770_RS47020	Ribose/xylose/arabinos	None	8.6E-288	151	3	WP_015243228.1
1,115,528	1,115,528	1	C -> T		87	SNP (transition)	98.9%		480	CTC -> CTT	C770_RS47020	Ribose/xylose/arabinos	None	2.2E-308	160	3	WP_015243228.1
1,115,611	1,115,611	1	A -> G		97	SNP (transition)	97.9%	H -> R	563	CAT -> CGT	C770_RS47020	Ribose/xylose/arabinos	Substitution	0.0	188	2	WP_015243228.1
1,115,693	1,115,693	1	C -> G		85	SNP (transversion)	98.8%		645	GTC -> GTG	C770_RS47020	Ribose/xylose/arabinos	None	2.1E-284	215	3	WP_015243228.1
1,115,816	1,115,816	1	C -> T		89	SNP (transition)	97.8%		768	ACC -> ACT	C770_RS47020	Ribose/xylose/arabinos	None	3.1E-284	256	3	WP_015243228.1
1,115,831	1,115,832	2	CG -> TA		87	Substitution	96.6%	FV -> FI	783	TTC,GTC -> TTT,/	C770_RS47020	Ribose/xylose/arabinos	Substitution	5.5E-274	261	3	WP_015243228.1

1,115,855	1,115,855	1	C -> T	87	SNP (transition)	97.7%		807	GGC -> GGT	C770_RS47020	Ribose/xylose/arabinos	None	3.7E-286	269	3	WP_015243228.1
1,115,882	1,115,882	1	G -> A	82	SNP (transition)	98.8%		834	CAG -> CAA	C770_RS47020	Ribose/xylose/arabinos	None	2.6E-282	278	3	WP_015243228.1
1,115,915	1,115,915	1	C -> T	81	SNP (transition)	100.0%		867	GTC -> GTT	C770_RS47020	Ribose/xylose/arabinos	None	4.0E-276	289	3	WP_015243228.1
1,115,983	1,115,983	1	G -> A	74	SNP (transition)	100.0%	R -> K	935	AGA -> AAA	C770_RS47020	Ribose/xylose/arabinos	Substitution	1.0E-259	312	2	WP_015243228.1
1,115,986	1,115,986	1	A -> T	74	SNP (transversion)	100.0%	Q -> L	938	CAG -> CTG	C770_RS47020	Ribose/xylose/arabinos	Substitution	1.0E-259	313	2	WP_015243228.1
1,116,001	1,116,001	1	T -> C	69	SNP (transition)	100.0%	V -> A	953	GTC -> GCC	C770_RS47020	Ribose/xylose/arabinos	Substitution	2.5E-235	318	2	WP_015243228.1
1,116,046	1,116,047	2	GA -> CG	57 -> 58	Substitution	100.0%	SS -> SG	24	TCG,AGC -> TCC,	C770_RS47025	autoinducer	Substitution	6.3E-198	8	3	WP_013850858.1
1,116,106	1,116,106	1	A -> G	59	SNP (transition)	100.0%		84	GCA -> GCG	C770_RS47025	autoinducer	None	3.2E-207	28	3	WP_013850858.1
1,116,160	1,116,160	1	C -> T	74	SNP (transition)	100.0%		138	TCC -> TCT	C770_RS47025	autoinducer	None	2.5E-252	46	3	WP_013850858.1
1,116,172	1,116,172	1	C -> G	74	SNP (transversion)	98.6%		150	GGC -> GGG	C770_RS47025	autoinducer	None	9.3E-240	50	3	WP_013850858.1
1,116,190	1,116,190	1	G -> A	70	SNP (transition)	97.1%		168	GAG -> GAA	C770_RS47025	autoinducer	None	9.6E-222	56	3	WP_013850858.1
1,116,265	1,116,265	1	C -> T	76	SNP (transition)	100.0%		243	AAC -> AAT	C770_RS47025	autoinducer	None	1.0E-228	81	3	WP_013850858.1
1,116,307	1,116,307	1	C -> T	83	SNP (transition)	98.8%		285	GTC -> GTT	C770_RS47025	autoinducer	None	2.1E-269	95	3	WP_013850858.1
1,116,554	1,116,554	1	G -> A	86	SNP (transition)	100.0%	A -> T	532	GCG -> ACG	C770_RS47025	autoinducer	Substitution	1.0E-301	178	1	WP_013850858.1
1,116,559	1,116,559	1	A -> G	85	SNP (transition)	100.0%		537	AAA -> AAG	C770_RS47025	autoinducer	None	1.0E-289	179	3	WP_013850858.1
1,116,727	1,116,727	1	A -> T	100	SNP (transversion)	99.0%	E -> D	705	GAA -> GAT	C770_RS47025	autoinducer	Substitution	1.3E-305	235	3	WP_013850858.1
1,116,808	1,116,808	1	G -> C	106	SNP (transversion)	100.0%		786	ACG -> ACC	C770_RS47025	autoinducer	None	0.0	262	3	WP_013850858.1
1,116,883	1,116,883	1	G -> A	98	SNP (transition)	100.0%		861	AAG -> AAA	C770_RS47025	autoinducer	None	2.5E-314	287	3	WP_013850858.1
1,116,931	1,116,931	1	A -> C	93	SNP (transversion)	100.0%		909	GGA -> GGC	C770_RS47025	autoinducer	None	1.3E-307	303	3	WP_013850858.1
1,116,956	1,116,956	1	G -> A	91	SNP (transition)	100.0%	V -> I	934	GTA -> ATA	C770_RS47025	autoinducer	Substitution	4.0E-310	312	1	WP_013850858.1
1,116,961	1,116,961	1	G -> A	93	SNP (transition)	100.0%		939	CAG -> CAA	C770_RS47025	autoinducer	None	1.3E-307	313	3	WP_013850858.1
1,117,033	1,117,033	1	T -> C	91	SNP (transition)	98.9%		1.011	AAT -> AAC	C770_RS47025	autoinducer	None	9.1E-314	337	3	WP_013850858.1
1,117,053	1,117,053	1	G -> A	82	SNP (transition)	100.0%		1.031	TGA -> TAA	C770_RS47025	autoinducer	None	1.0E-287	344	2	WP_013850858.1
1,117,062	1,117,062	1	T -> C	78	SNP (transition)	100.0%							1.0E-273			
1,117,082	1,117,082	1	C -> T	78	SNP (transition)	100.0%							6.3E-266			
1,117,087	1,117,088	2	TG -> CA	78 -> 79	Substitution	98.7%							1.2E-260			
1,117,099	1,117,099	1	T -> A	76	SNP (transversion)	98.7%							7.6E-254			
1,117,104	1,117,104	1	G -> A	76	SNP (transition)	100.0%							1.0E-266			
1,117,131	1,117,131	1	C -> A	73	SNP (transversion)	98.6%		12	CGC -> CGA	C770_RS47030	hypothetical protein	None	7.3E-251	4	3	WP_015243229.1
1,117,239	1,117,239	1	A -> G	85	SNP (transition)	100.0%		120	CCA -> CCG	C770_RS47030	hypothetical protein	None	3.2E-281	40	3	WP_015243229.1
1,117,248	1,117,248	1	C -> G	78	SNP (transversion)	100.0%		129	GTC -> GTG	C770_RS47030	hypothetical protein	None	4.0E-258	43	3	WP_015243229.1
1,117,362	1,117,362	1	G -> C	85	SNP (transversion)	100.0%		243	CCG -> CCC	C770_RS47030	hypothetical protein	None	1.0E-272	81	3	WP_015243229.1
1,117,515	1,117,515	1	T -> C	81	SNP (transition)	100.0%		396	TAT -> TAC	C770_RS47030	hypothetical protein	None	3.2E-284	132	3	WP_015243229.1
1,117,607	1,117,607	1	C -> T	83	SNP (transition)	98.8%	A -> V	488	GCC -> GTC	C770_RS47030	hypothetical protein	Substitution	2.1E-269	163	2	WP_015243229.1
1,117,671	1,117,671	1	T -> C	79	SNP (transition)	98.7%		552	AAT -> AAC	C770_RS47030	hypothetical protein	None	3.1E-256	184	3	WP_015243229.1
1,117,722	1,117,722	1	C -> T	72	SNP (transition)	100.0%		603	TTC -> TTT	C770_RS47030	hypothetical protein	None	2.5E-238	201	3	WP_015243229.1
1,117,728	1,117,728	1	C -> T	72	SNP (transition)	100.0%		609	AAC -> AAT	C770_RS47030	hypothetical protein	None	1.6E-245	203	3	WP_015243229.1
1,117,732	1,117,732	1	G -> A	73	SNP (transition)	100.0%	A -> T	613	GCC -> ACC	C770_RS47030	hypothetical protein	Substitution	1.3E-241	205	1	WP_015243229.1
1,117,812	1,117,812	1	G -> T	73	SNP (transversion)	98.6%		693	ACG -> ACT	C770_RS47030	hypothetical protein	None	7.3E-251	231	3	WP_015243229.1
1,117,815	1,117,815	1	T -> C	75	SNP (transition)	98.7%		696	GGT -> GGC	C770_RS47030	hypothetical protein	None	4.7E-243	232	3	WP_015243229.1
1,117,868	1,117,869	2	AC -> GA	67	Substitution	98.5%	N -> R	749	AAC -> AGA	C770_RS47030	hypothetical protein	Substitution	6.7E-230	250	2	WP_015243229.1
1,117,887	1,117,887	1	C -> G	57	SNP (transversion)	100.0%		768	GGC -> GGG	C770_RS47030	hypothetical protein	None	2.0E-177	256	3	WP_015243229.1
1,117,897	1,117,899	1	GTC -> GCAAAG(	55 -> 56	Insertion	98.2%	V -> AKGV	778	GTC -> GCA,AAG	C770_RS47030	hypothetical protein	Insertion	3.2E-182	260	1	WP_015243229.1
1,118,004	1,118,004	1	T -> C	67	SNP (transition)	98.5%		885	CGT -> CGC	C770_RS47030	hypothetical protein	None	1.1E-216	295	3	WP_015243229.1
1,118,034	1,118,034	1	C -> A	71	SNP (transversion)	100.0%		915	GCC -> GCA	C770_RS47030	hypothetical protein	None	3.2E-249	305	3	WP_015243229.1
1,118,040	1,118,040	1	G -> T	70	SNP (transversion)	100.0%		921	GCG -> GCT	C770_RS47030	hypothetical protein	None	1.0E-245	307	3	WP_015243229.1
1,118,048	1,118,048	1	C -> G	67	SNP (transversion)	98.5%	P -> R	929	CCC -> CGC	C770_RS47030	hypothetical protein	Substitution	6.7E-230	310	2	WP_015243229.1
1,118,064	1,118,064	1	G -> A	69	SNP (transition)	98.6%		945	GCG -> GCA	C770_RS47030	hypothetical protein	None	6.9E-237	315	3	WP_015243229.1
1,118,105	1,118,106	2	CA -> AG	75	Substitution	100.0%	T -> K	986	ACA -> AAG	C770_RS47030	hypothetical protein	Substitution	1.0E-255	329	2	WP_015243229.1
1,118,151	1,118,151	1	C -> T	88	SNP (transition)	98.9%		1.032	TAC -> TAT	C770_RS47030	hypothetical protein	None	1.4E-294	344	3	WP_015243229.1
1,118,175	1,118,175	1	C -> G	92	SNP (transversion)	100.0%		1.056	GGC -> GGG	C770_RS47030	hypothetical protein	None	1.6E-313	352	3	WP_015243229.1
1,118,208	1,118,208	1	G -> C	91	SNP (transversion)	100.0%	K -> N	1.089	AAG -> AAC	C770_RS47030	hypothetical protein	Substitution	3.2E-319	363	3	WP_015243229.1
1,118,220	1,118,220	1	T -> C	88	SNP (transition)	100.0%		1.101	ATT -> ATC	C770_RS47030	hypothetical protein	None	6.3E-300	367	3	WP_015243229.1
1,118,246	1,118,246	1	C -> T	92	SNP (transition)	100.0%	A -> V	1.127	GCC -> GTC	C770_RS47030	hypothetical protein	Substitution	1.6E-313	376	2	WP_015243229.1
1,118,252	1,118,252	1	C -> T	91	SNP (transition)	100.0%	A -> V	1.133	GCA -> GTA	C770_RS47030	hypothetical protein	Substitution	4.0E-310	378	2	WP_015243229.1
1,118,286	1,118,286	1	T -> C	93	SNP (transition)	98.9%		1.167	ACT -> ACC	C770_RS47030	hypothetical protein	None	1.5E-311	389	3	WP_015243229.1
1,118,322	1,118,322	1	T -> C	99	SNP (transition)	100.0%		1.203	TAT -> TAC	C770_RS47030	hypothetical protein	None	0.0	401	3	WP_015243229.1
1,118,340	1,118,340	1	G -> C	99	SNP (transversion)	99.0%		1.221	GCG -> GCC	C770_RS47030	hypothetical protein	None	4.0E-322	407	3	WP_015243229.1
1,118,350	1,118,349	0	(A)3 -> (A)4	103	Insertion (tandem repeat)	100.0%		1.231		C770_RS47030	hypothetical protein	Frame Shift	0.0	411	1	WP_015243229.1
1,118,379	1,118,379	1	T -> G	103	SNP (transversion)	100.0%							0.0			
1,118,391	1,118,391	1	G -> A	103	SNP (transition)	100.0%							0.0			
1,118,417	1,118,417	1	C -> T	94	SNP (transition)	97.9%							6.9E-310			
1,118,419	1,118,419	1	A -> T	93	SNP (transversion)	100.0%							0.0			
1,118,428	1,118,428	1	A	94	Deletion	100.0%							1.0E-141			
1,118,445	1,118,446	2	CC -> TT	90	Substitution	100.0%							1.0E-297			
1,118,450	1,118,450	1	G -> A	88	SNP (transition)	100.0%							6.3E-300			
1,118,461	1,118,461	1	A -> G	82	SNP (transition)	98.8%							2.6E-282			
1,118,465	1,118,465	1	A -> G	82	SNP (transition)	100.0%							1.0E-287			
1,118,483	1,118,483	1	T -> C	78	SNP (transition)	100.0%							6.3E-266			
1,118,488	1,118,488	1	G -> C	76	SNP (transversion)	100.0%							1.6E-251			
1,118,490	1,118,491	2	CT -> TG	76	Substitution	98.7%							2.4E-261			
1,118,493	1,118,493	1	C -> G	76	SNP (transversion)	100.0%							4.0E-259			
1,118,496	1,118,496	1	A -> T	76	SNP (transversion)	97.4%							7.2E-249			
1,118,506	1,118,506	1	T -> C	75	SNP (transition)	100.0%							1.0E-255			
1,118,508	1,118,508	1	G -> A	74	SNP (transition)	100.0%							1.0E-259			
1,118,520	1,118,522	3	TGA -> CCC	63 -> 65	Substitution	98.4% -> 98.5%							1.3E-208			
1,118,530	1,118,530	1	G -> A	61	SNP (transition)	100.0%							3.2E-214			
1,118,534	1,118,534	1	G -> A	61	SNP (transition)	100.0%							4.0E-208			
1,118,537	1,118,537	1	C -> G	61	SNP (transversion)	98.4%							6.1E-203			
1,118,541	1,118,541	1	T -> C	60	SNP (transition)	100.0%							1.0E-192			
1,118,547	1,118,547	1	G -> C	54	SNP (transversion)	100.0%							4.0E-195			
1,118,558	1,118,558	1	A -> C	49	SNP (transversion)	100.0%							2.5E-167			
1,118,562	1,118,563	2	CC -> TG	47	Substitution	100.0%							7.9E-156			
1,118,583	1,118,583	1	C -> A	42	SNP (transversion)	97.6%							2.6E-130			
1,118,594	1,118,594	1	T -> C	37	SNP (transition)	100.0%							3.2E-130			
1,118,600	1,118,601	2	CG -> TC	35	Substitution	100.0%							3.2E-116			
1,118,607	1,118,607	1	G	33	Deletion	100.0%							3.2E-50			
1,118,617	1,118,617	1	C -> A	28	SNP (transversion)	100.0%							6.3E-96			
1,118,628	1,118,628	1	G -> C	23	SNP (transversion)	100.0%							1.3E-76			
1,118,653	1,118,653	1	A -> T	15	SNP (transversion)	100.0%							1.0E-48			
1,118,661	1,118,661	1	T -> C	11	SNP (transition)	100.0%							1.0E-33			
1,118,664	1,118,664	1	G -> A	10	SNP (transition)	100.0%							1.0E-31			
1,118,668	1,118,668	1	C -> T	8	SNP (transition)	100.0%							4.0E-27			
1,134,425	1,134,425	1	C -> T	9	SNP (transition)	100.0%		1.212	GAC -> GAT	C770_RS47085	capsule polysaccharide	None	1.6E-29	404	3	WP_015243238.1
1,134,429	1,134,429	1	A -> C	10	SNP (transversion)	100.0%		1.216	AGG -> CGG	C770_RS47085	capsule polysaccharide	None	1.0E-36	406	1	WP_015243238.1
1,134,437	1,134,437	1	C -> T	10	SNP (transition)	100.0%		1.224	GAC -> GAT	C770_RS47085	capsule polysaccharide	None	1.0E-35	408	3	WP_015243238.1
1,134,455	1,134,455	1	G -> C	13	SNP (transversion)	100.0%		1.242	CGG -> CGC	C770_RS47085	capsule polysaccharide	None	7.9E-49	414	3	WP_015243238.1
1,134,467	1,134,467	1	G -> T	15	SNP (transversion)	100.0%	E -> D	1.254	GAG -> GAT	C770_RS47085	capsule polysaccharide	Substitution	3.2E-56	418	3	WP_015243238.1
1,134,470	1,134,470	1	G -> A	16	SNP (transition)	100.0%		1.257	CGG -> CGA	C770_RS47085	capsule polysaccharide	None	2.5E-58	419	3	WP_015243238.1
1,134,494	1,134,494	1	C -> A	22	SNP (transversion)	100.0%		1.281	ATC -> ATA	C770_RS47085	capsule polysaccharide	None	4.0E-82	427	3	WP_015243238.1
1,134,497	1,134,497	1	T -> C	24	SNP (transition)	100.0%		1.284	CTT -> CTC	C770_RS47085	capsule polysaccharide	None	1.0E-84	428	3	WP_015243238.1
1,134,503	1,134,503	1	A -> G	25	SNP (transition)	100.0%		1.29	CCA -> CCG	C770_RS47085	capsule polysaccharide	None	3.2E-88	430	3	WP_015243238.1
1,134,509	1,134,509	1	T -> G	27	SNP (transversion)	96.3%		1.296	GGT -> GGG	C770_RS47085	capsule polysaccharide	None	1.1E-87	432	3	WP_015243238.1
1,134,524	1,134,524	1	C -> T	32	SNP (transition)	100.0%	H -> Y	7	CAT -> TAT	C770_RS47090	polysaccharide ABC trar	Substitution	6.3E-116	3	1	WP_015243239.1
1,134,529	1,134,529	1	C -> G	33	SNP (transversion)	100.0%		12	CTC -> CTG	C770_RS47090	polysaccharide ABC trar	None	1.6E-119	4	3	WP_015243239.1
1,134,531	1,134,531	1	T -> C	34	SNP (transition)	100.0%	I -> T	14	ATC -> ACC	C770_RS47090	polysaccharide ABC trar	Substitution	4.0E-123	5	2	WP_015243239.1
1,134,550	1,134,550	1	C -> T	40	SNP (transition)	100.0%		33	GTC -> GTT	C770_RS47090	polysaccharide ABC trar	None	1.0E-136	11	3	WP_015243239.1
1,134,553	1,134,553	1	G -> C	40	SNP (transversion)	100.0%		36	GGG -> GGC	C770_RS47090	polysaccharide ABC trar	None	1.0E-144	12	3	WP_015243239.1
1,134,592	1,134,592	1	C -> G	52	SNP (transversion)	100.0%		75	TCC -> TCG	C770_RS47090	polysaccharide ABC trar	None	1.6E-177	25	3	WP_015243239.1
1,134,595	1,134,595	1	A -> G	55	SNP (transition)	100.0%		78	AAA -> AAG	C770_RS47090	polysaccharide ABC trar	None	3.2E-193	26	3	WP_015243239.1
1,134,640	1,134,640	1	A -> C	71	SNP (transversion)	100.0%		123	ATA -> ATC	C770_RS47090	polysaccharide ABC trar	None	5.0E-235	41	3	WP_015243239.1
1,134,644	1,134,646	2	TTA -> CTG	71	Substitution	100.0%		127	TTA -> CTG	C770_RS47090	polysaccharide ABC trar	None	3.2E-249	43	1	WP_015243239.1
1,134,655	1,134,655	1	G -> A	73	SNP (transition)	100.0%		138	GTG -> GTA	C770_RS47090	polysaccharide ABC trar	None	1.3E-241	46	3	WP_015243239.1
1,134,679	1,134,679	1	C -> G	72	SNP (transversion)	100.0%		162	GCC -> GCG	C770_RS47090	polysaccharide ABC trar	None	1.0E-252	54	3	WP_015243239.1
1,134,691	1,134,691	1	C -> T	72	SNP (transition)	100.0%		174	GGC -> GGT	C770_RS47090	polysaccharide ABC trar	None	1.0E-252	58	3	WP_015243239.1
1,134,715	1,134,715	1	G -> C	72	SNP (transversion)	100.0%		198	GCG -> GCC	C770_RS47090	polysaccharide ABC trar	None	4.0E-231	66	3	WP_015243239.1
1,134,718	1,134,718	1	A -> C	72	SNP (transversion)	100.0%		201	ACA -> ACC	C770_RS47090	polysaccharide ABC trar	None	2.5E-238	67	3	WP_015243239.1
1,134,760	1,134,761	2	CC -> TT	76 -> 77	Substitution	100.0%		243	TAC,CTG -> TAT,'	C770_RS47090	polysaccharide ABC trar	None	1.6E-251	81	3	WP_015243239.1
1,134,769	1,134,769	1	C -> A	77	SNP (transversion)	98.7%		252	GCC -> GCA	C770_RS47090	polysaccharide ABC trar	None	3.1E-257	84	3	WP_015243239.1
1,134,808	1,134,808	1	C -> T	73	SNP (transition)	98.6%		291	AAC -> AAT	C770_RS47090	polysaccharide ABC trar	None	1.2E-243	97	3	WP_015243239.1
1,134,826	1,134,826	1	G -> C	75	SNP (transversion)	100.0%		309	ACG -> ACC	C770_RS47090	polysaccharide ABC trar	None	1.0E-255	103	3	WP_015243239.1
1,134,829	1,134,829	1	C -> T	76	SNP (transition)	98.7%		312	ATC -> ATT	C770_RS47090	polysaccharide ABC trar	None	2.4E-246	104	3	WP_015243239.1
1,134,847	1,134,847	1	G -> A	77	SNP (transition)	100.0%		330	CTG -> CTA	C770_RS47090	polysaccharide ABC trar	None	6.3E-278	110	3	WP_015243239.1
1,134,865	1,134,865	1	A -> G	71	SNP (transition)	100.0%		348	TCA -> TCG	C770_RS47090	polysaccharide ABC trar	None	4.0E-242	116	3	WP_015243239.1
1,134,874	1,134,874	1	G -> C	69	SNP (transversion)	100.0%		357	GCG -> GCC	C770_RS47090	polysaccharide ABC trar	None	2.5E-235	119	3	WP_015243239.1
1,134,877	1,134,877	1	C -> T	69	SNP (transition)	100.0%		360	TTC -> TTT	C770_RS47090	polysaccharide ABC trar	None	3.2E-242	120	3	WP_015243239.1
1,134,895	1,134,895	1	T -> C	65	SNP (transition)	100.0%		378	ATT -> ATC	C770_RS47090	polysaccharide ABC trar	None	3.2E-228	126	3	WP_015243239.1
1,134,915	1,134,915	1	A -> G	71	SNP (transition)	100.0%	D -> G	398	GAC -> GGC	C770_RS47090	polysaccharide ABC trar	Substitution	5.0E-235	133	2	WP_015243239.1
1,134,920	1,134,922	2	AAC -> GAT	70	Substitution	98.6%	N -> D	403	AAC -> GAT	C770_RS47090	polysaccharide ABC trar	Substitution	2.0E-228	135	1	WP_015243239.1
1,134,925	1,134,925	1	T -> C	68	SNP (transition)	98.5%		408	ATT -> ATC	C770_RS47090	polysaccharide ABC trar	None	1.1E-226	136	3	WP_015243239.1
1,134,940	1,134,940	1	C -> T	67	SNP (transition)	100.0%		423	ATC -> ATT	C770_RS47090	polysaccharide ABC trar	None	1.6E-228	141	3	WP_015243239.1
1,134,952	1,134,952	1	G -> C	71	SNP (transversion)	100.0%		435	GTG -> GTC	C770_RS47090	polysaccharide ABC trar	None	3.2E-249	145	3	WP_015243239.1
1,134,976	1,134,976	1	C -> T	78	SNP (transition)	100.0%		459	CTC -> CTT	C770_RS47090	polysaccharide ABC trar	None	6.3E-266	153	3	WP_015243239.1
1,134,979	1,134,979	1	C -> T	77	SNP (transition)	100.0%		462	GGC -> GGT	C770_RS47090	polysaccharide ABC trar	None	3.2E-270	154	3	WP_015243239.1
1,134,985	1,134,985	1	A -> C	76	SNP (transversion)	100.0%		468	GCA -> GCC	C770_RS47090	polysaccharide ABC trar	None	1.6E-251	156	3	WP_015243239.1
1,135,003	1,135,003	1	G -> C	75	SNP (transversion)	100.0%		486	CTG -> CTC	C770_RS47090	polysaccharide ABC trar	None	1.0E-255	162	3	WP_015243239.1
1,135,009	1,135,009	1	C -> T	75	SNP (transition)	100.0%		492	CTC -> CTT	C770_RS47090	polysaccharide ABC trar	None	1.0E-255	164	3	WP_015243239.1
1,135,011	1,135,011	1	G -> A	74	SNP (transition)	100.0%	R -> K	494	AGA -> AAA	C770_RS47090	polysaccharide ABC trar	Substitution	2.5E-252	165	2	WP_015243239.1
1,135,031	1,135,031	1	A -> G	74	SNP (transition)	100.0%	I -> V	514	ATC -> GTC	C770_RS47090	polysaccharide ABC trar	Substitution	6.3E-245	172	1	WP_015243239.1
1,135,036	1,135,038	3	CAC -> TGG	76	Substitution	98.7%	FT -> FG	519	TTC,ACC -> TTT,(	C770_RS47090	polysaccharide ABC trar	Substitution	1.6E-251	173	3	WP_015243239.1
1,135,066	1,135,066	1	C -> A	82	SNP (transversion)	100.0%		549	ATC -> ATA	C770_RS47090	polysaccharide ABC trar	None	1.0E-287	183	3	WP_015243239.1
1,135,069	1,135,069	1	T -> A	83	SNP (transversion)	98.8%		552	TCT -> TCA	C770_RS47090	polysaccharide ABC trar	None	1.3E-277	184	3	WP_015243239.1
1,135,138	1,135,138	1	C -> T	89	SNP (transition)	100.0%		621	GTC -> GTT	C770_RS47090	polysaccharide ABC trar	None	1.6E-285	207	3	WP_015243239.1
1,135,142	1,135,142	1	G -> A	88	SNP (transition)	98.9%	V -> I	625	GTC -> ATC	C770_RS47090	polysaccharide ABC trar	Substitution	7.0E-286	209	1	WP_015243239.1
1,135,147	1,135,147	1	C -> T	89	SNP (transition)	100.0%		630	ATC -> ATT	C770_RS47090	polysaccharide ABC trar	None	1.6E-285	210	3	WP_015243239.1
1,135,165	1,135,165	1	T -> C	88	SNP (transition)	100.0%		648	GGT -> GGC	C770_RS47090	polysaccharide ABC trar	None	1.0E-308	216	3	WP_015243239.1
1,135,183	1,135,183	1	G -> T	87	SNP (transversion)	98.9%		666	CGG -> CGT	C770_RS47090	polysaccharide ABC trar	None	1.4E-282	222	3	WP_015243239.1
1,135,202	1,135,203	2	AC -> GA	87	Substitution	98.9%	T -> D	685	ACC -> GAC	C770_RS47090	polysaccharide ABC trar	Substitution	7.9E-288	229	1	WP_015243239.1
1,135,213	1,135,213	1	T -> C	87	SNP (transition)	100.0%		696	TAT -> TAC	C770_RS47090	polysaccharide ABC trar	None	3.2E-305	232	3	WP_015243239.1
1,135,247	1,135,247	1	T -> C	89	SNP (transition)	100.0%		730	TTG -> CTG	C770_RS47090	polysaccharide ABC trar	None	2.0E-294	244	1	WP_015243239.1
1,135,290	1,135,290	1	A -> C	93	SNP (transversion)	100.0%		6	ATA -> ATC	C770_RS47095	polysaccharide ABC trar	None	6.3E-317	2	3	WP_015243240.1
1,135,314	1,135,314	1	T -> C	85	SNP (transition)	100.0%		30	TAT -> TAC	C770_RS47095	polysaccharide ABC trar	None	3.2E-298	10	3	WP_015243240.1
1,135,323	1,135,323	1	C -> T	88	SNP (transition)	100.0%		39	ACC -> ACT	C770_RS47095	polysaccharide ABC trar	None	4.0E-291	13	3	WP_015243240.1
1,135,344	1,135,344	1	T -> C	81	SNP (transition)	100.0%		60	ATT -> ATC	C770_RS47095	polysaccharide ABC trar	None	4.0E-276	20	3	WP_015243240.1
1,135,347	1,135,347	1	T -> C	81	SNP (transition)	100.0%		63	ATT -> ATC	C770_RS47095	polysaccharide ABC trar	None	3.2E-284	21	3	WP_015243240.1
1,135,353	1,135,353	1	C -> T	81	SNP (transition)	100.0%		69	GAC -> GAT	C770_RS47095	polysaccharide ABC trar	None	3.2E-284	23	3	WP_015243240.1
1,135,356	1,135,356	1	A -> T	81	SNP (transversion)	100.0%		72	GCA -> GCT	C770_RS47095	polysaccharide ABC trar	None	5.0E-268	24	3	WP_015243240.1
1,135,368	1,135,368	1	G -> C	83	SNP (transversion)	100.0%		84	CTG -> CTC	C770_RS47095	polysaccharide ABC trar	None	1.3E-274	28	3	WP_015243240.1
1,135,392	1,135,392	1	G -> A	82	SNP (transition)	100.0%		108	CTG -> CTA	C770_RS47095	polysaccharide ABC trar	None	2.5E-271	36	3	WP_015243240.1
1,135,422	1,135,425	3	GCTG -> CTTA	71	Substitution	97.2%		138	ACG,CTG -> ACC,	C770_RS47095	polysaccharide ABC trar	None	1.4E-226	46	3	WP_015243240.1

1,135,428	1,135,428	1	G -> T	71	SNP (transversion)	100.0%		144	CTG -> CTT	C770_RS47095	polysaccharide ABC trar	None	6.3E-228	48	3	WP_015243240.1
1,135,434	1,135,435	2	CA -> GG	72 -> 73	Substitution	98.6%	LI -> LV	150	CTC,ATC -> CTG,(	C770_RS47095	polysaccharide ABC trar	Substitution	1.8E-236	50	3	WP_015243240.1
1,135,446	1,135,446	1	A -> C	77	SNP (transversion)	97.4%		162	GCA -> GCC	C770_RS47095	polysaccharide ABC trar	None	9.2E-245	54	3	WP_015243240.1
1,135,488	1,135,488	1	T -> C	76	SNP (transition)	100.0%		204	ATT -> ATC	C770_RS47095	polysaccharide ABC trar	None	4.0E-259	68	3	WP_015243240.1
1,135,503	1,135,503	1	C -> A	72	SNP (transversion)	98.6%		219	GGC -> GGA	C770_RS47095	polysaccharide ABC trar	None	1.8E-254	73	3	WP_015243240.1
1,135,507	1,135,509	3	TCA -> CAG	70 -> 71	Substitution	100.0%	S -> Q	223	TCA -> CAG	C770_RS47095	polysaccharide ABC trar	Substitution	4.0E-242	75	1	WP_015243240.1
1,135,518	1,135,518	1	T -> C	70	SNP (transition)	98.6%		234	TTT -> TTC	C770_RS47095	polysaccharide ABC trar	None	2.2E-240	78	3	WP_015243240.1
1,135,521	1,135,524	4	GAGC -> ACCA	70 -> 71	Substitution	97.1% -> 97.2%	QS -> QP	237	CAG,AGC -> CAA,	C770_RS47095	polysaccharide ABC trar	Substitution	6.2E-232	79	3	WP_015243240.1
1,135,533	1,135,533	1	G -> A	69	SNP (transition)	100.0%		249	ACG -> ACA	C770_RS47095	polysaccharide ABC trar	None	3.2E-242	83	3	WP_015243240.1
1,135,536	1,135,536	1	C -> A	69	SNP (transversion)	100.0%		252	GGC -> GGA	C770_RS47095	polysaccharide ABC trar	None	2.5E-235	84	3	WP_015243240.1
1,135,539	1,135,539	1	G -> A	70	SNP (transition)	98.6%		255	GAG -> GAA	C770_RS47095	polysaccharide ABC trar	None	2.2E-240	85	3	WP_015243240.1
1,135,557	1,135,557	1	C -> T	65	SNP (transition)	100.0%		273	GTC -> GTT	C770_RS47095	polysaccharide ABC trar	None	1.0E-221	91	3	WP_015243240.1
1,135,560	1,135,560	1	C -> A	65	SNP (transversion)	100.0%		276	GCC -> GCA	C770_RS47095	polysaccharide ABC trar	None	3.2E-228	92	3	WP_015243240.1
1,135,563	1,135,563	1	A -> T	65	SNP (transversion)	100.0%		279	CGA -> CGT	C770_RS47095	polysaccharide ABC trar	None	3.2E-228	93	3	WP_015243240.1
1,135,641	1,135,641	1	A -> G	62	SNP (transition)	98.4%		357	CCA -> CCG	C770_RS47095	polysaccharide ABC trar	None	3.1E-200	119	3	WP_015243240.1
1,135,674	1,135,674	1	C -> T	59	SNP (transition)	100.0%		390	CGC -> CGT	C770_RS47095	polysaccharide ABC trar	None	2.0E-195	130	3	WP_015243240.1
1,135,713	1,135,713	1	T -> C	61	SNP (transition)	100.0%		429	TAT -> TAC	C770_RS47095	polysaccharide ABC trar	None	3.2E-214	143	3	WP_015243240.1
1,135,737	1,135,737	1	C -> T	63	SNP (transition)	100.0%		453	GCC -> GCT	C770_RS47095	polysaccharide ABC trar	None	1.3E-208	151	3	WP_015243240.1
1,135,743	1,135,743	1	T -> C	65	SNP (transition)	100.0%		459	GGT -> GGC	C770_RS47095	polysaccharide ABC trar	None	3.2E-215	153	3	WP_015243240.1
1,135,749	1,135,749	1	A -> G	65	SNP (transition)	100.0%		465	TCA -> TCG	C770_RS47095	polysaccharide ABC trar	None	1.0E-221	155	3	WP_015243240.1
1,135,757	1,135,757	1	G -> A	66	SNP (transition)	100.0%	R -> K	473	AGG -> AAG	C770_RS47095	polysaccharide ABC trar	Substitution	6.3E-212	158	2	WP_015243240.1
1,135,764	1,135,764	1	G -> A	66	SNP (transition)	100.0%		480	AAG -> AAA	C770_RS47095	polysaccharide ABC trar	None	6.3E-212	160	3	WP_015243240.1
1,135,782	1,135,782	1	C -> G	66	SNP (transversion)	100.0%	D -> E	498	GAC -> GAG	C770_RS47095	polysaccharide ABC trar	Substitution	6.3E-212	166	3	WP_015243240.1
1,135,827	1,135,827	1	T -> C	56	SNP (transition)	100.0%		543	ACT -> ACC	C770_RS47095	polysaccharide ABC trar	None	1.6E-185	181	3	WP_015243240.1
1,135,840	1,135,840	1	G -> A	54	SNP (transition)	100.0%	D -> N	556	GAC -> AAC	C770_RS47095	polysaccharide ABC trar	Substitution	1.0E-189	186	1	WP_015243240.1
1,135,857	1,135,857	1	G -> C	48	SNP (transversion)	100.0%		573	GGG -> GGC	C770_RS47095	polysaccharide ABC trar	None	1.6E-173	191	3	WP_015243240.1
1,135,879	1,135,879	1	T -> A	45	SNP (transversion)	100.0%	L -> M	595	TTG -> ATG	C770_RS47095	polysaccharide ABC trar	Substitution	1.0E-153	199	1	WP_015243240.1
1,135,893	1,135,893	1	C -> G	43	SNP (transversion)	100.0%	D -> E	609	GAC -> GAG	C770_RS47095	polysaccharide ABC trar	Substitution	1.6E-155	203	3	WP_015243240.1
1,135,905	1,135,905	1	G -> C	42	SNP (transversion)	100.0%	E -> D	621	GAG -> GAC	C770_RS47095	polysaccharide ABC trar	Substitution	1.6E-143	207	3	WP_015243240.1
1,135,912	1,135,912	1	A -> C	37	SNP (transversion)	100.0%	S -> R	628	AGC -> CGC	C770_RS47095	polysaccharide ABC trar	Substitution	4.0E-119	210	1	WP_015243240.1
1,135,934	1,135,934	1	G -> A	34	SNP (transition)	100.0%	R -> K	650	AGG -> AAG	C770_RS47095	polysaccharide ABC trar	Substitution	1.6E-109	217	2	WP_015243240.1
1,135,940	1,135,941	2	AT -> GC	33 -> 34	Substitution	100.0%	N -> S	656	AAT -> AGC	C770_RS47095	polysaccharide ABC trar	Substitution	6.3E-113	219	2	WP_015243240.1
1,135,944	1,135,943	0	G	32	Insertion	100.0%		660		C770_RS47095	polysaccharide ABC trar	Frame Shift	4.0E-103	220	3	WP_015243240.1
1,135,951	1,135,952	2	TC -> CA	31	Substitution	100.0%	V -> A	8	GTC -> GCA	C770_RS47100	capsule polysaccharide	Substitution	4.0E-106	3	2	WP_015243241.1
1,135,955	1,135,955	1	G -> C	29	SNP (transversion)	100.0%	R -> S	12	AGG -> AGC	C770_RS47100	capsule polysaccharide	Substitution	3.2E-102	4	3	WP_015243241.1
1,135,958	1,135,958	1	C -> G	28	SNP (transversion)	100.0%	N -> K	15	AAC -> AAG	C770_RS47100	capsule polysaccharide	Substitution	4.0E-93	5	3	WP_015243241.1
1,135,961	1,135,961	1	C -> A	26	SNP (transversion)	100.0%	D -> E	18	GAC -> GAA	C770_RS47100	capsule polysaccharide	Substitution	6.3E-84	6	3	WP_015243241.1
1,135,967	1,135,967	1	G -> A	24	SNP (transition)	100.0%		24	GCG -> GCA	C770_RS47100	capsule polysaccharide	None	4.0E-87	8	3	WP_015243241.1
1,135,971	1,135,971	1	G -> A	24	SNP (transition)	95.8%	D -> N	28	GAC -> AAC	C770_RS47100	capsule polysaccharide	Substitution	6.0E-73	10	1	WP_015243241.1
1,135,975	1,135,975	1	G -> A	23	SNP (transition)	100.0%	R -> K	32	AGG -> AAG	C770_RS47100	capsule polysaccharide	Substitution	1.3E-76	11	2	WP_015243241.1
1,135,977	1,135,977	1	T -> C	23	SNP (transition)	100.0%	S -> P	34	TCT -> CCT	C770_RS47100	capsule polysaccharide	Substitution	5.0E-72	12	1	WP_015243241.1
1,135,981	1,135,983	3	GTT -> ACC	21 -> 22	Substitution	95.2% -> 95.5%	GS -> DP	38	GGT,TCC -> GAC,	C770_RS47100	capsule polysaccharide	Substitution	1.0E-63	13	2	WP_015243241.1
1,135,987	1,135,987	1	T -> C	20	SNP (transition)	100.0%	V -> A	44	GTG -> GCG	C770_RS47100	capsule polysaccharide	Substitution	1.0E-60	15	2	WP_015243241.1
1,135,991	1,135,990	0	GGT	19	Insertion	100.0%	H -> QV	48	CAC -> CAG,GTC	C770_RS47100	capsule polysaccharide	Insertion	1.3E-59	16	3	WP_015243241.1
1,135,999	1,136,000	2	GA -> AGCG	13 -> 16	Insertion	100.0%		56		C770_RS47100	capsule polysaccharide	Frame Shift	5.0E-41	19	2	WP_015243241.1
1,136,006	1,136,006	1	T -> C	12	SNP (transition)	100.0%		63	ACT -> ACC	C770_RS47100	capsule polysaccharide	None	4.0E-39	21	3	WP_015243241.1
1,136,009	1,136,009	1	G -> C	10	SNP (transversion)	100.0%		66	GCG -> GCC	C770_RS47100	capsule polysaccharide	None	1.0E-27	22	3	WP_015243241.1
1,136,011	1,136,012	2	CT -> AG	10	Substitution	100.0%	T -> K	68	ACT -> AAG	C770_RS47100	capsule polysaccharide	Substitution	1.0E-24	23	2	WP_015243241.1
1,136,017	1,136,018	2	AA -> C	9	Deletion	100.0%		74		C770_RS47100	capsule polysaccharide	Frame Shift	1.6E-11	25	2	WP_015243241.1
1,136,024	1,136,027	2	TGCC -> AAAGCA	7 -> 8	Insertion	100.0%		81		C770_RS47100	capsule polysaccharide	Frame Shift	4.0E-15	27	3	WP_015243241.1
1,138,279	1,138,290	12	TGTTCTGAGATT	7 -> 10	Substitution	100.0%							2.5E-20			
1,138,292	1,138,293	2	TC -> CA	10	Substitution	100.0%							1.0E-31			
1,138,295	1,138,296	2	TG -> CC	11	Substitution	100.0%							1.6E-31			
1,138,299	1,138,299	1	A -> C	12	SNP (transversion)	100.0%							2.5E-40			
1,138,301	1,138,303	3	TGC -> GCA	12 -> 13	Substitution	100.0%							6.3E-38			
1,138,305	1,138,305	1	G -> A	13	SNP (transition)	100.0%							2.5E-42			
1,138,307	1,138,308	2	GA -> AC	14	Substitution	100.0%							2.5E-41			
1,138,311	1,138,311	1	G -> C	17	SNP (transversion)	100.0%							1.0E-51			
1,138,326	1,138,326	1	T -> A	33	SNP (transversion)	100.0%							2.5E-106			
1,138,328	1,138,328	1	A -> G	35	SNP (transition)	100.0%							3.2E-102			
1,138,330	1,138,330	1	G -> T	36	SNP (transversion)	100.0%							1.6E-119			
1,141,148	1,141,148	1	T -> A	51	SNP (transversion)	98.0%							5.1E-154			
1,141,152	1,141,152	1	G -> T	45	SNP (transversion)	100.0%							1.0E-144			
1,141,158	1,141,158	1	A -> G	37	SNP (transition)	97.3%							1.5E-121			
1,141,160	1,141,160	1	A	36	Deletion	100.0%							2.5E-58			
1,141,167	1,141,169	3	GAA -> CTT	22 -> 25	Substitution	95.5% -> 96.0%							2.5E-73			
1,141,171	1,141,177	7	TAGTCGA -> CCC	21 -> 22	Substitution	100.0%							6.3E-69			
1,141,179	1,141,179	1	A	19	Deletion	100.0%							4.0E-31			
1,141,182	1,141,182	1	A -> C	19	SNP (transversion)	100.0%							2.5E-65			
1,141,184	1,141,184	1	C -> A	19	SNP (transversion)	100.0%							1.3E-59			
1,141,189	1,141,189	1	-C	17	Deletion	100.0%							6.3E-28			
1,141,191	1,141,191	1	C -> T	17	SNP (transition)	100.0%							1.6E-58			
1,141,194	1,141,195	2	GG -> AT	15	Substitution	100.0%							1.0E-51			
1,141,197	1,141,201	5	GACCT -> TTGAG	13 -> 14	Substitution	100.0%							6.3E-47			
1,141,204	1,141,206	3	CCG -> AAT	10	Substitution	100.0%							1.0E-35			
1,141,209	1,141,218	10	GACAGCTCGG ->	6 -> 9	Substitution	100.0%							1.6E-17			
1,149,000	1,149,001	2	AA	7	Deletion	100.0%		1.068		C770_RS47165	capsule polysaccharide	Frame Shift	6.3E-12	356	3	WP_015243251.1
1,149,005	1,149,006	2	TC -> CA	7	Substitution	100.0%	D -> C	1.063	GAC -> TGC	C770_RS47165	capsule polysaccharide	Substitution	7.9E-24	355	1	WP_015243251.1
1,149,009	1,149,009	1	C -> G	7	SNP (transversion)	100.0%	A -> P	1.06	GCG -> CCG	C770_RS47165	capsule polysaccharide	Substitution	6.3E-26	354	1	WP_015243251.1
1,149,014	1,149,020	7	TTGACGC -> GAA	6	Substitution	100.0%	RVK -> PTS	1.049	CGC,GTC,AAA ->	C770_RS47165	capsule polysaccharide	Substitution	2.5E-19	350	2	WP_015243251.1
1,150,045	1,150,047	3	TGC -> ATG	7	Substitution	100.0%	A -> H	22	GCA -> CAT	C770_RS47165	capsule polysaccharide	Substitution	7.9E-17	8	1	WP_015243251.1
1,150,059	1,150,059	1	G -> A	8	SNP (transition)	100.0%	P -> S	10	CCG -> TCG	C770_RS47165	capsule polysaccharide	Substitution	4.0E-23	4	1	WP_015243251.1
1,150,100	1,150,100	1	T -> C	19	SNP (transition)	100.0%	T -> A	742	ACC -> GCC	C770_RS47170	polysaccharide ABC trar	Substitution	1.3E-59	248	1	WP_015243252.1
1,150,107	1,150,107	1	C -> A	22	SNP (transversion)	100.0%		735	CTG -> CTT	C770_RS47170	polysaccharide ABC trar	None	6.3E-69	245	3	WP_015243252.1
1,150,125	1,150,125	1	G -> A	32	SNP (transition)	100.0%		717	TTC -> TTT	C770_RS47170	polysaccharide ABC trar	None	4.0E-103	239	3	WP_015243252.1
1,150,140	1,150,142	2	GAC -> AAA	36 -> 37	Substitution	100.0%	V -> F	700	GTC -> TTT	C770_RS47170	polysaccharide ABC trar	Substitution	1.0E-108	234	1	WP_015243252.1
1,150,151	1,150,151	1	C -> G	42	SNP (transversion)	97.6%	V -> L	691	GTG -> CTG	C770_RS47170	polysaccharide ABC trar	Substitution	1.3E-142	231	1	WP_015243252.1
1,150,164	1,150,164	1	C -> G	45	SNP (transversion)	100.0%		678	CTG -> CTC	C770_RS47170	polysaccharide ABC trar	None	1.0E-153	226	3	WP_015243252.1
1,150,168	1,150,171	4	AAAT -> CCGG	47 -> 49	Substitution	100.0%	DL -> AG	671	GAT,TTG -> GCC,	C770_RS47170	polysaccharide ABC trar	Substitution	2.0E-146	224	2	WP_015243252.1
1,150,173	1,150,175	2	GGT -> TGC	51 -> 52	Substitution	100.0%	T -> A	667	ACC -> GCA	C770_RS47170	polysaccharide ABC trar	Substitution	6.3E-164	223	1	WP_015243252.1
1,150,179	1,150,179	1	G -> A	52	SNP (transition)	100.0%		663	TAC -> TAT	C770_RS47170	polysaccharide ABC trar	None	4.0E-167	221	3	WP_015243252.1
1,150,191	1,150,191	1	G -> A	56	SNP (transition)	100.0%		651	ATC -> ATT	C770_RS47170	polysaccharide ABC trar	None	4.0E-191	217	3	WP_015243252.1
1,150,198	1,150,200	3	TGC -> GCG	56	Substitution	100.0%	RQ -> RA	642	CGG,CAA -> CGC	C770_RS47170	polysaccharide ABC trar	Substitution	1.6E-185	214	3	WP_015243252.1
1,150,218	1,150,218	1	A -> G	61	SNP (transition)	98.4%		624	CAT -> CAC	C770_RS47170	polysaccharide ABC trar	None	6.1E-215	208	3	WP_015243252.1
1,150,224	1,150,224	1	A -> C	63	SNP (transversion)	100.0%		618	CTT -> CTG	C770_RS47170	polysaccharide ABC trar	None	2.5E-202	206	3	WP_015243252.1
1,150,254	1,150,254	1	A -> G	69	SNP (transition)	100.0%		588	CCT -> CCC	C770_RS47170	polysaccharide ABC trar	None	3.2E-242	196	3	WP_015243252.1
1,150,305	1,150,305	1	T -> C	79	SNP (transition)	100.0%		537	CCA -> CCG	C770_RS47170	polysaccharide ABC trar	None	3.2E-277	179	3	WP_015243252.1
1,150,308	1,150,308	1	G -> C	80	SNP (transversion)	100.0%		534	CGC -> CGG	C770_RS47170	polysaccharide ABC trar	None	1.0E-264	178	3	WP_015243252.1
1,150,316	1,150,316	1	C -> T	82	SNP (transition)	100.0%	V -> I	526	GTC -> ATC	C770_RS47170	polysaccharide ABC trar	Substitution	1.6E-279	176	1	WP_015243252.1
1,150,322	1,150,322	1	T -> C	82	SNP (transition)	100.0%	S -> G	520	AGC -> GGC	C770_RS47170	polysaccharide ABC trar	Substitution	4.0E-263	174	1	WP_015243252.1
1,150,326	1,150,326	1	T -> G	82	SNP (transversion)	100.0%		516	ATA -> ATC	C770_RS47170	polysaccharide ABC trar	None	1.6E-279	172	3	WP_015243252.1
1,150,335	1,150,335	1	G -> A	81	SNP (transition)	100.0%		507	TAC -> TAT	C770_RS47170	polysaccharide ABC trar	None	3.2E-284	169	3	WP_015243252.1
1,150,359	1,150,359	1	G -> C	80	SNP (transversion)	100.0%		483	GTC -> GTG	C770_RS47170	polysaccharide ABC trar	None	1.0E-264	161	3	WP_015243252.1
1,150,368	1,150,368	1	A -> G	80	SNP (transition)	100.0%		474	ATT -> ATC	C770_RS47170	polysaccharide ABC trar	None	1.0E-264	158	3	WP_015243252.1
1,150,386	1,150,386	1	A -> G	82	SNP (transition)	100.0%		456	GGT -> GGC	C770_RS47170	polysaccharide ABC trar	None	2.5E-271	152	3	WP_015243252.1
1,150,389	1,150,389	1	C -> G	80	SNP (transversion)	100.0%		453	CTG -> CTC	C770_RS47170	polysaccharide ABC trar	None	1.0E-272	151	3	WP_015243252.1
1,150,395	1,150,395	1	G -> C	81	SNP (transversion)	100.0%		447	GCC -> GCG	C770_RS47170	polysaccharide ABC trar	None	4.0E-276	149	3	WP_015243252.1
1,150,420	1,150,420	1	G -> A	74	SNP (transition)	98.6%	T -> M	422	ACG -> ATG	C770_RS47170	polysaccharide ABC trar	Substitution	2.3E-254	141	2	WP_015243252.1
1,150,424	1,150,424	1	C -> T	72	SNP (transition)	100.0%	G -> S	418	GGC -> AGC	C770_RS47170	polysaccharide ABC trar	Substitution	6.3E-260	140	1	WP_015243252.1
1,150,430	1,150,430	1	T -> C	73	SNP (transition)	100.0%	I -> V	412	ATC -> GTC	C770_RS47170	polysaccharide ABC trar	Substitution	6.3E-249	138	1	WP_015243252.1
1,150,452	1,150,452	1	C -> G	74	SNP (transversion)	98.6%		390	GGG -> GGC	C770_RS47170	polysaccharide ABC trar	None	4.7E-247	130	3	WP_015243252.1
1,150,463	1,150,463	1	T -> G	76	SNP (transversion)	100.0%	I -> L	379	ATC -> CTC	C770_RS47170	polysaccharide ABC trar	Substitution	1.6E-251	127	1	WP_015243252.1
1,150,472	1,150,472	1	G -> T	73	SNP (transversion)	97.3%	L -> M	370	CTG -> ATG	C770_RS47170	polysaccharide ABC trar	Substitution	8.3E-246	124	1	WP_015243252.1
1,150,491	1,150,491	1	C -> G	74	SNP (transversion)	100.0%		351	CTG -> CTC	C770_RS47170	polysaccharide ABC trar	None	2.5E-252	117	3	WP_015243252.1
1,150,508	1,150,509	2	AT -> GC	70 -> 72	Substitution	100.0%		333	CAA,TTG -> CAG,	C770_RS47170	polysaccharide ABC trar	None	1.0E-238	111	3	WP_015243252.1
1,150,545	1,150,545	1	G -> A	78	SNP (transition)	100.0%		297	ACC -> ACT	C770_RS47170	polysaccharide ABC trar	None	4.0E-258	99	3	WP_015243252.1
1,150,554	1,150,554	1	G -> A	78	SNP (transition)	100.0%		288	CCC -> CCT	C770_RS47170	polysaccharide ABC trar	None	4.0E-258	96	3	WP_015243252.1
1,150,569	1,150,569	1	C -> G	80	SNP (transversion)	100.0%		273	GCG -> GCC	C770_RS47170	polysaccharide ABC trar	None	1.0E-280	91	3	WP_015243252.1
1,150,582	1,150,582	1	C -> T	84	SNP (transition)	100.0%	R -> K	260	AGG -> AAG	C770_RS47170	polysaccharide ABC trar	Substitution	6.3E-278	87	2	WP_015243252.1
1,150,587	1,150,587	1	G -> C	82	SNP (transversion)	100.0%		255	ACC -> ACG	C770_RS47170	polysaccharide ABC trar	None	1.6E-279	85	3	WP_015243252.1
1,150,602	1,150,604	3	CGA -> GCC	79	Substitution	98.7%	S -> G	238	TCG -> GGC	C770_RS47170	polysaccharide ABC trar	Substitution	2.0E-248	80	1	WP_015243252.1
1,150,716	1,150,716	1	C -> T	77	SNP (transition)	100.0%		126	GCG -> GCA	C770_RS47170	polysaccharide ABC trar	None	1.6E-262	42	3	WP_015243252.1
1,150,755	1,150,755	1	G -> A	76	SNP (transition)	100.0%		87	GGC -> GGT	C770_RS47170	polysaccharide ABC trar	None	1.6E-251	29	3	WP_015243252.1
1,150,761	1,150,761	1	C -> A	75	SNP (transversion)	100.0%		81	CCG -> CCT	C770_RS47170	polysaccharide ABC trar	None	1.0E-255	27	3	WP_015243252.1
1,150,794	1,150,794	1	C -> T	74	SNP (transition)	98.6%		48	GTG -> GTA	C770_RS47170	polysaccharide ABC trar	None	4.7E-247	16	3	WP_015243252.1
1,150,821	1,150,821	1	A -> G	68	SNP (transition)	100.0%		21	CAT -> CAC	C770_RS47170	polysaccharide ABC trar	None	6.3E-232	7	3	WP_015243252.1
1,150,833	1,150,833	1	G -> T	67	SNP (transversion)	100.0%		9	CTC -> CTA	C770_RS47170	polysaccharide ABC trar	None	7.9E-222	3	3	WP_015243252.1
1,150,871	1,150,871	1	G -> A	65	SNP (transition)	100.0%							1.0E-221			
1,150,874	1,150,874	1	T -> A	63	SNP (transversion)	100.0%							6.3E-215			
1,150,879	1,150,879	1	A -> G	65	SNP (transition)	100.0%							3.2E-228			
1,150,906	1,150,906	1	T -> C	69	SNP (transition)	100.0%							1.6E-221			
1,150,911	1,150,912	2	CT -> GC	70	Substitution	98.6%							1.8E-233			
1,150,928	1,150,928	1	T -> G	67	SNP (transversion)	100.0%							7.9E-222			
1,150,935	1,150,935	1	G -> T	66	SNP (transversion)	100.0%							4.0E-225			
1,150,961	1,150,961	1	C -> T	70	SNP (transition)	98.6%							1.8E-233			
1,150,966	1,150,966	1	G -> A	70	SNP (transition)	100.0%							1.0E-231			
1,150,976	1,150,976	1	G -> A	72	SNP (transition)	98.6%							2.9E-240			
1,150,978	1,150,978	1	C -> T	72	SNP (transition)	100.0%							2.5E-238			
1,150,982	1,150,982	1	G -> A	72	SNP (transition)	100.0%							1.6E-245			
1,150,995	1,150,995	1	C -> T	70	SNP (transition)	98.6%							1.4E-226			
1,151,004	1,151,004	1	T -> C	72	SNP (transition)	100.0%							1.6E-245			
1,151,007	1,151,007	1	C -> T	72	SNP (transition)	100.0%							1.6E-245			
1,151,027	1,151,027	1	T -> A	70	SNP (transversion)	100.0%							1.0E-238			
1,151,029	1,151,030	2	TT -> CG	69 -> 70	Substitution	100.0%							2.5E-235			
1,151,041	1,151,041	1	C -> A	66	SNP (transversion)	100.0%							4.0E-225			
1,151,050	1,151,052	3	TCT -> AGC	65	Substitution	100.0%							1.0E-221			
1,151,054	1,151,056	3	CCT -> GGG	65	Substitution	100.0%							3.2E-215			
1,151,058	1,151,061	4	ACCA -> G	65 -> 66	Deletion	100.0%							3.2E-111			
1,151,063	1,151,065	3	ACA -> CAC	68	Substitution	98.5%							5.4E-220			
1,151,082	1,151,082	1	T -> C	75	SNP (transition)	98.7%							1.9E-250			
1,151,085	1,151,085	1	T -> G	76	SNP (transversion)	100.0%							2.5E-274			
1,151,091	1,151,091	1	G -> A	77	SNP (transition)	100.0%							1.6E-262			
1,151,094	1,151,094	1	G -> A	77	SNP (transition)	100.0%							4.0E-247			
1,151,133	1,151,133	1	T -> C	74	SNP (transition)	98.6%							4.7E-247			
1,151,143	1,151,143	1	C -> T	72	SNP (transition)	100.0%	G -> E	794	GGG -> GAG	C770_RS47175	Ketopantoate hydroxyn	Substitution	1.0E-252	265	2	WP_015243253.1
1,151,145	1,151,145	1	C -> G	72	SNP (transversion)	100.0%		792	GGG -> GGC	C770_RS47175	Ketopantoate hydroxyn	None	1.0E-252	264	3	WP_015243253.1
1,151,203	1,151,204	2	CC -> TT	76	Substitution	98.7%	G -> N	733	GGC -> AAC	C770_RS47175	Ketopantoate hydroxyn	Substitution	2.4E-246	245	1	WP_015243253.1
1,151,226	1,151,226	1	A -> G	76	SNP (transition)	100.0%		711	CGT -> CGC	C770_RS47175	Ketopantoate hydroxyn	None	4.0E-259	237	3	WP_015243253.1
1,151,247	1,151,247	1	G -> C	79	SNP (transversion)	100.0%		690	TCC -> TCG	C770_RS47175	Ketopantoate hydroxyn	None	2.0E-261	230	3	WP_015243253.1
1,151,268	1,151,268	1	T -> C	75	SNP (transition)	98.7%		669	CAA -> CAG	C770_RS47175	Ketopantoate hydroxyn	None	1.2E-235	223	3	WP_015243253.1

1,151,280	1,151,280	1	G -> A	78	SNP (transition)	100.0%		657	GAC -> GAT	C770_RS47175	Ketopantoate hydroxym	None	6.3E-266	219	3	WP_015243253.1
1,151,283	1,151,283	1	A -> G	79	SNP (transition)	98.7%		654	TAT -> TAC	C770_RS47175	Ketopantoate hydroxym	None	1.3E-279	218	3	WP_015243253.1
1,151,304	1,151,304	1	A -> G	79	SNP (transition)	100.0%		633	TAT -> TAC	C770_RS47175	Ketopantoate hydroxym	None	3.2E-277	211	3	WP_015243253.1
1,151,307	1,151,307	1	G -> A	78	SNP (transition)	100.0%		630	ACC -> ACT	C770_RS47175	Ketopantoate hydroxym	None	4.0E-258	210	3	WP_015243253.1
1,151,325	1,151,325	1	G -> A	73	SNP (transition)	100.0%		612	TAC -> TAT	C770_RS47175	Ketopantoate hydroxym	None	6.3E-249	204	3	WP_015243253.1
1,151,391	1,151,391	1	C -> T	74	SNP (transition)	100.0%		546	GCG -> GCA	C770_RS47175	Ketopantoate hydroxym	None	2.5E-252	182	3	WP_015243253.1
1,151,394	1,151,394	1	C -> T	74	SNP (transition)	95.9%		543	GGG -> GGA	C770_RS47175	Ketopantoate hydroxym	None	4.1E-223	181	3	WP_015243253.1
1,151,400	1,151,400	1	C -> A	74	SNP (transversion)	100.0%		537	TCG -> TCT	C770_RS47175	Ketopantoate hydroxym	None	6.3E-245	179	3	WP_015243253.1
1,151,403	1,151,403	1	C -> A	73	SNP (transversion)	100.0%		534	CTG -> CTT	C770_RS47175	Ketopantoate hydroxym	None	6.3E-249	178	3	WP_015243253.1
1,151,419	1,151,419	1	C -> G	73	SNP (transversion)	97.3%	S -> T	518	AGC -> ACC	C770_RS47175	Ketopantoate hydroxym	Substitution	1.7E-224	173	2	WP_015243253.1
1,151,451	1,151,451	1	G -> C	80	SNP (transversion)	100.0%		486	CCC -> CCG	C770_RS47175	Ketopantoate hydroxym	None	1.0E-264	162	3	WP_015243253.1
1,151,457	1,151,457	1	C -> G	80	SNP (transversion)	100.0%		480	GTG -> GTC	C770_RS47175	Ketopantoate hydroxym	None	1.0E-280	160	3	WP_015243253.1
1,151,495	1,151,495	1	G -> A	76	SNP (transition)	98.7%		442	CTG -> TTG	C770_RS47175	Ketopantoate hydroxym	None	2.4E-246	148	1	WP_015243253.1
1,151,499	1,151,499	1	C -> T	76	SNP (transition)	100.0%		438	AAG -> AAA	C770_RS47175	Ketopantoate hydroxym	None	6.3E-244	146	3	WP_015243253.1
1,151,502	1,151,502	1	G -> C	76	SNP (transversion)	100.0%		435	ACC -> ACG	C770_RS47175	Ketopantoate hydroxym	None	2.5E-274	145	3	WP_015243253.1
1,151,516	1,151,516	1	C -> T	74	SNP (transition)	100.0%	V -> I	421	GTC -> ATC	C770_RS47175	Ketopantoate hydroxym	Substitution	2.5E-252	141	1	WP_015243253.1
1,151,544	1,151,544	1	A -> G	74	SNP (transition)	100.0%		393	GTT -> GTC	C770_RS47175	Ketopantoate hydroxym	None	2.5E-252	131	3	WP_015243253.1
1,151,586	1,151,586	1	G -> C	80	SNP (transversion)	100.0%		351	CTC -> CTG	C770_RS47175	Ketopantoate hydroxym	None	1.0E-272	117	3	WP_015243253.1
1,151,619	1,151,619	1	G -> C	84	SNP (transversion)	100.0%	D -> E	318	GAC -> GAG	C770_RS47175	Ketopantoate hydroxym	Substitution	6.3E-278	106	3	WP_015243253.1
1,151,664	1,151,664	1	C -> G	77	SNP (transversion)	98.7%		273	GCG -> GCC	C770_RS47175	Ketopantoate hydroxym	None	3.1E-257	91	3	WP_015243253.1
1,151,667	1,151,667	1	A -> G	78	SNP (transition)	100.0%		270	GAT -> GAC	C770_RS47175	Ketopantoate hydroxym	None	6.3E-266	90	3	WP_015243253.1
1,151,775	1,151,775	1	C -> G	82	SNP (transversion)	100.0%		162	CGG -> CGC	C770_RS47175	Ketopantoate hydroxym	None	1.6E-279	54	3	WP_015243253.1
1,151,781	1,151,781	1	T -> C	83	SNP (transition)	100.0%		156	GAA -> GAG	C770_RS47175	Ketopantoate hydroxym	None	3.2E-291	52	3	WP_015243253.1
1,151,786	1,151,786	1	G -> C	82	SNP (transversion)	100.0%	P -> A	151	CCC -> GCC	C770_RS47175	Ketopantoate hydroxym	Substitution	1.0E-287	51	1	WP_015243253.1
1,151,817	1,151,817	1	G -> A	88	SNP (transition)	98.9%		120	GAC -> GAT	C770_RS47175	Ketopantoate hydroxym	None	7.0E-286	40	3	WP_015243253.1
1,151,838	1,151,838	1	G -> A	85	SNP (transition)	100.0%		99	GCC -> GCT	C770_RS47175	Ketopantoate hydroxym	None	1.0E-289	33	3	WP_015243253.1
1,151,861	1,151,862	2	CC -> TG	82	Substitution	100.0%	VV -> VM	75	GTG,GTG -> GTC	C770_RS47175	Ketopantoate hydroxym	Substitution	1.6E-279	25	3	WP_015243253.1
1,151,889	1,151,889	1	T -> C	83	SNP (transition)	98.8%		48	GGA -> GGG	C770_RS47175	Ketopantoate hydroxym	None	3.3E-261	16	3	WP_015243253.1
1,151,930	1,151,930	1	G -> T	83	SNP (transversion)	100.0%	H -> N	7	CAC -> AAC	C770_RS47175	Ketopantoate hydroxym	Substitution	6.3E-283	3	1	WP_015243253.1
1,151,938	1,151,938	1	G -> T	83	SNP (transversion)	98.8%							5.2E-294			
1,151,940	1,151,939	0	A	83	Insertion	98.8%							1.3E-277			
1,151,941	1,151,941	1	C -> A	84	SNP (transversion)	100.0%							2.5E-286			
1,151,947	1,151,947	1	A -> G	83	SNP (transition)	97.6%							1.4E-272			
1,151,958	1,151,958	1	C -> T	88	SNP (transition)	98.9%							3.5E-277			
1,151,960	1,151,963	4	AGCA -> CAGG	85	Substitution	98.8%							3.2E-281			
1,151,965	1,151,965	1	G -> A	85	SNP (transition)	100.0%							1.0E-289			
1,151,968	1,151,968	1	C -> T	85	SNP (transition)	98.8%							5.4E-276			
1,151,975	1,151,975	1	C -> T	88	SNP (transition)	100.0%							6.3E-300			
1,151,977	1,151,977	1	C -> G	88	SNP (transversion)	100.0%							6.3E-300			
1,151,982	1,151,982	1	G -> C	87	SNP (transversion)	98.9%							1.4E-282			
1,151,984	1,151,984	1	C -> G	87	SNP (transversion)	100.0%							1.6E-296			
1,151,987	1,151,987	1	G -> C	89	SNP (transversion)	98.9%							3.5E-289			
1,151,989	1,151,989	1	A -> C	89	SNP (transversion)	98.9%							5.6E-298			
1,151,991	1,151,991	1	C -> A	90	SNP (transversion)	100.0%							1.0E-288			
1,152,006	1,152,006	1	A -> T	88	SNP (transversion)	100.0%							4.0E-291			
1,152,011	1,152,011	1	G -> A	84	SNP (transition)	100.0%							6.3E-278			
1,152,015	1,152,014	0	C	83	Insertion	100.0%							6.3E-283			
1,152,041	1,152,044	3	CCGG -> GCTC	84 -> 86	Substitution	97.6% -> 97.7%	TR -> TS	1.2	ACC,CGG -> ACG	C770_RS47180	NAD(P)H-nitrite reducta	Substitution	1.1E-272	400	3	WP_015243254.1
1,152,071	1,152,071	1	C -> A	81	SNP (transversion)	100.0%	E -> D	1.173	GAG -> GAT	C770_RS47180	NAD(P)H-nitrite reducta	Substitution	5.0E-268	391	3	WP_015243254.1
1,152,116	1,152,116	1	A -> G	84	SNP (transition)	100.0%		1.128	GAT -> GAC	C770_RS47180	NAD(P)H-nitrite reducta	None	2.5E-286	376	3	WP_015243254.1
1,152,125	1,152,125	1	G -> A	85	SNP (transition)	100.0%		1.119	GTC -> GTT	C770_RS47180	NAD(P)H-nitrite reducta	None	3.2E-281	373	3	WP_015243254.1
1,152,128	1,152,128	1	T -> G	86	SNP (transversion)	98.8%		1.116	GCA -> GCC	C770_RS47180	NAD(P)H-nitrite reducta	None	2.7E-279	372	3	WP_015243254.1
1,152,152	1,152,152	1	T -> G	93	SNP (transversion)	100.0%		1.092	GCA -> GCC	C770_RS47180	NAD(P)H-nitrite reducta	None	5.0E-289	364	3	WP_015243254.1
1,152,160	1,152,160	1	T -> G	89	SNP (transversion)	98.9%	I -> L	1.084	ATC -> CTC	C770_RS47180	NAD(P)H-nitrite reducta	Substitution	3.5E-289	362	1	WP_015243254.1
1,152,167	1,152,167	1	T -> G	87	SNP (transversion)	100.0%		1.077	GGA -> GGC	C770_RS47180	NAD(P)H-nitrite reducta	None	4.0E-279	359	3	WP_015243254.1
1,152,170	1,152,170	1	C -> T	87	SNP (transition)	100.0%		1.074	GAG -> GAA	C770_RS47180	NAD(P)H-nitrite reducta	None	7.9E-288	358	3	WP_015243254.1
1,152,173	1,152,173	1	G -> A	84	SNP (transition)	100.0%		1.071	GGC -> GGT	C770_RS47180	NAD(P)H-nitrite reducta	None	6.3E-278	357	3	WP_015243254.1
1,152,185	1,152,185	1	A -> G	84	SNP (transition)	100.0%		1.059	TTT -> TTC	C770_RS47180	NAD(P)H-nitrite reducta	None	2.5E-286	353	3	WP_015243254.1
1,152,194	1,152,194	1	G -> A	85	SNP (transition)	100.0%		1.05	TTC -> TTT	C770_RS47180	NAD(P)H-nitrite reducta	None	1.0E-272	350	3	WP_015243254.1
1,152,200	1,152,200	1	T -> C	81	SNP (transition)	100.0%		1.044	GGA -> GGG	C770_RS47180	NAD(P)H-nitrite reducta	None	6.3E-260	348	3	WP_015243254.1
1,152,203	1,152,203	1	C -> T	83	SNP (transition)	97.6%		1.041	GAG -> GAA	C770_RS47180	NAD(P)H-nitrite reducta	None	2.1E-256	347	3	WP_015243254.1
1,152,221	1,152,221	1	C -> G	82	SNP (transversion)	98.8%		1.023	GTG -> GTC	C770_RS47180	NAD(P)H-nitrite reducta	None	4.1E-266	341	3	WP_015243254.1
1,152,229	1,152,229	1	T -> C	81	SNP (transition)	98.8%	T -> A	1.015	ACG -> GCG	C770_RS47180	NAD(P)H-nitrite reducta	Substitution	8.1E-271	339	1	WP_015243254.1
1,152,232	1,152,233	2	CT -> GC	79	Substitution	100.0%	AE -> AQ	1.011	GCA,GAA -> GCG	C770_RS47180	NAD(P)H-nitrite reducta	Substitution	2.5E-269	337	3	WP_015243254.1
1,152,299	1,152,299	1	G -> A	68	SNP (transition)	100.0%		945	AGC -> AGT	C770_RS47180	NAD(P)H-nitrite reducta	None	1.0E-238	315	3	WP_015243254.1
1,152,303	1,152,303	1	A -> G	68	SNP (transition)	100.0%	V -> A	941	GTC -> GCC	C770_RS47180	NAD(P)H-nitrite reducta	Substitution	6.3E-232	314	2	WP_015243254.1
1,152,313	1,152,313	1	C -> T	67	SNP (transition)	100.0%	E -> K	931	GAA -> AAA	C770_RS47180	NAD(P)H-nitrite reducta	Substitution	3.2E-235	311	1	WP_015243254.1
1,152,316	1,152,316	1	C -> T	66	SNP (transition)	100.0%	G -> R	928	GGA -> AGA	C770_RS47180	NAD(P)H-nitrite reducta	Substitution	1.6E-218	310	1	WP_015243254.1
1,152,320	1,152,320	1	A -> G	65	SNP (transition)	100.0%		924	GGT -> GGC	C770_RS47180	NAD(P)H-nitrite reducta	None	1.0E-221	308	3	WP_015243254.1
1,152,324	1,152,324	1	A -> G	63	SNP (transition)	100.0%	M -> T	920	ATG -> ACG	C770_RS47180	NAD(P)H-nitrite reducta	Substitution	7.9E-234	307	2	WP_015243254.1
1,152,338	1,152,338	1	G -> A	61	SNP (transition)	98.4%		906	GCC -> GCT	C770_RS47180	NAD(P)H-nitrite reducta	None	6.1E-209	302	3	WP_015243254.1
1,152,341	1,152,341	1	A -> G	58	SNP (transition)	100.0%		903	CTT -> CTC	C770_RS47180	NAD(P)H-nitrite reducta	None	1.0E-203	301	3	WP_015243254.1
1,152,345	1,152,345	1	G -> A	58	SNP (transition)	100.0%	A -> V	899	GCG -> GTG	C770_RS47180	NAD(P)H-nitrite reducta	Substitution	6.3E-198	300	2	WP_015243254.1
1,152,371	1,152,371	1	G -> A	50	SNP (transition)	100.0%		873	GCC -> GCT	C770_RS47180	NAD(P)H-nitrite reducta	None	1.0E-180	291	3	WP_015243254.1
1,152,374	1,152,374	1	T -> C	50	SNP (transition)	100.0%		870	GAA -> GAG	C770_RS47180	NAD(P)H-nitrite reducta	None	1.0E-175	290	3	WP_015243254.1
1,152,382	1,152,382	1	G -> T	49	SNP (transversion)	100.0%		862	CGG -> AGG	C770_RS47180	NAD(P)H-nitrite reducta	None	3.2E-172	288	1	WP_015243254.1
1,152,389	1,152,389	1	C -> A	48	SNP (transversion)	100.0%		855	CGG -> CGT	C770_RS47180	NAD(P)H-nitrite reducta	None	1.0E-168	285	3	WP_015243254.1
1,152,404	1,152,404	1	G -> C	40	SNP (transversion)	100.0%		840	CCC -> CCG	C770_RS47180	NAD(P)H-nitrite reducta	None	1.0E-140	280	3	WP_015243254.1
1,152,437	1,152,437	1	A -> G	28	SNP (transition)	100.0%		807	TAT -> TAC	C770_RS47180	NAD(P)H-nitrite reducta	None	1.6E-101	269	3	WP_015243254.1
1,152,464	1,152,465	2	TC -> CA	21	Substitution	100.0%	R -> M	779	AGA -> ATG	C770_RS47180	NAD(P)H-nitrite reducta	Substitution	3.2E-74	260	2	WP_015243254.1
1,152,476	1,152,476	1	G -> T	20	SNP (transversion)	100.0%		768	GCC -> GCA	C770_RS47180	NAD(P)H-nitrite reducta	None	1.0E-66	256	3	WP_015243254.1
1,152,479	1,152,479	1	A -> G	20	SNP (transition)	100.0%		765	ATT -> ATC	C770_RS47180	NAD(P)H-nitrite reducta	None	1.0E-72	255	3	WP_015243254.1
1,153,309	1,153,310	2	CG -> GA	7	Substitution	100.0%							2.0E-22			
1,153,313	1,153,313	1	A -> G	8	SNP (transition)	100.0%							1.6E-25			
1,153,315	1,153,322	8	CATGCCCC -> AG	9 -> 12	Substitution	100.0%							1.0E-27			
1,153,328	1,153,328	1	C -> T	16	SNP (transition)	100.0%							1.6E-45			
1,153,331	1,153,331	1	T -> G	18	SNP (transversion)	100.0%							2.5E-49			
1,153,335	1,153,335	1	A -> C	19	SNP (transversion)	100.0%							7.9E-56			
1,153,339	1,153,339	1	T -> C	20	SNP (transition)	100.0%							1.0E-58			
1,153,341	1,153,341	1	C -> A	22	SNP (transversion)	100.0%							6.3E-58			
1,153,353	1,153,352	0	GA	24	Insertion	95.8%							1.2E-70			
1,153,354	1,153,354	1	T -> C	24	SNP (transition)	100.0%		318	TAA -> TAG	C770_RS47185	Rieske family ferredoxin	None	1.6E-77	106	3	WP_015243255.1
1,153,357	1,153,357	1	A -> G	25	SNP (transition)	96.0%		315	TCT -> TCC	C770_RS47185	Rieske family ferredoxin	None	4.0E-76	105	3	WP_015243255.1
1,153,376	1,153,376	1	C -> T	34	SNP (transition)	97.1%	S -> N	296	AGC -> AAC	C770_RS47185	Rieske family ferredoxin	Substitution	1.7E-101	99	2	WP_015243255.1
1,153,405	1,153,405	1	C -> T	43	SNP (transition)	100.0%		267	AGG -> AGA	C770_RS47185	Rieske family ferredoxin	None	1.3E-142	89	3	WP_015243255.1
1,153,414	1,153,414	1	A -> G	45	SNP (transition)	100.0%		258	GTT -> GTC	C770_RS47185	Rieske family ferredoxin	None	3.2E-158	86	3	WP_015243255.1
1,153,438	1,153,438	1	C -> T	53	SNP (transition)	100.0%		234	CAG -> CAA	C770_RS47185	Rieske family ferredoxin	None	6.3E-181	78	3	WP_015243255.1
1,153,447	1,153,447	1	C -> T	55	SNP (transition)	100.0%		225	AAG -> AAA	C770_RS47185	Rieske family ferredoxin	None	3.2E-182	75	3	WP_015243255.1
1,153,459	1,153,459	1	A -> G	61	SNP (transition)	100.0%		213	CGT -> CGC	C770_RS47185	Rieske family ferredoxin	None	4.0E-208	71	3	WP_015243255.1
1,153,462	1,153,462	1	A -> G	62	SNP (transition)	100.0%		210	GGT -> GGC	C770_RS47185	Rieske family ferredoxin	None	2.5E-205	70	3	WP_015243255.1
1,153,501	1,153,501	1	T -> C	77	SNP (transition)	100.0%		171	CTA -> CTG	C770_RS47185	Rieske family ferredoxin	None	1.6E-262	57	3	WP_015243255.1
1,153,559	1,153,559	1	T -> C	85	SNP (transition)	100.0%	E -> G	113	GAA -> GGA	C770_RS47185	Rieske family ferredoxin	Substitution	1.0E-289	38	2	WP_015243255.1
1,153,570	1,153,570	1	G -> A	85	SNP (transition)	100.0%		102	AGC -> AGT	C770_RS47185	Rieske family ferredoxin	None	1.0E-289	34	3	WP_015243255.1
1,153,579	1,153,579	1	A -> G	89	SNP (transition)	100.0%		93	GTT -> GTC	C770_RS47185	Rieske family ferredoxin	None	3.2E-312	31	3	WP_015243255.1
1,153,594	1,153,594	1	C -> T	88	SNP (transition)	100.0%		78	GGG -> GGA	C770_RS47185	Rieske family ferredoxin	None	4.0E-291	26	3	WP_015243255.1
1,153,618	1,153,618	1	A -> G	82	SNP (transition)	97.6%		54	GAT -> GAC	C770_RS47185	Rieske family ferredoxin	None	3.3E-261	18	3	WP_015243255.1
1,153,858	1,153,858	1	C -> A	81	SNP (transversion)	97.5%	R -> L	950	CGG -> CTG	C770_RS47190	fatty acid desaturase	Substitution	1.0E-273	317	2	WP_015243256.1
1,154,143	1,154,143	1	C -> T	94	SNP (transition)	100.0%	C -> Y	665	TGT -> TAT	C770_RS47190	fatty acid desaturase	Substitution	2.5E-320	222	2	WP_015243256.1
1,154,961	1,154,961	1	A -> T	76	SNP (transversion)	100.0%							1.6E-251			
1,154,972	1,154,972	1	G -> A	76	SNP (transition)	100.0%							2.5E-274			
1,154,977	1,154,977	1	A -> T	76	SNP (transversion)	98.7%							7.6E-254			
1,155,000	1,155,000	1	T -> G	78	SNP (transversion)	100.0%							6.3E-266			
1,155,028	1,155,028	1	G -> C	76	SNP (transversion)	97.4%		15	CCG -> CCC	C770_RS47195	sugar ABC transporter s	None	4.5E-234	5	3	WP_015243257.1
1,155,397	1,155,397	1	A -> G	92	SNP (transition)	100.0%		384	GCA -> GCG	C770_RS47195	sugar ABC transporter s	None	4.0E-295	128	3	WP_015243257.1
1,155,721	1,155,721	1	T -> C	94	SNP (transition)	100.0%		708	ATT -> ATC	C770_RS47195	sugar ABC transporter s	None	0.0	236	3	WP_015243257.1
1,156,075	1,156,075	1	C -> T	66	SNP (transition)	100.0%							4.0E-225			
1,156,080	1,156,081	2	AA -> TT	68	Substitution	98.5%							4.0E-225			
1,156,198	1,156,198	1	G -> A	63	SNP (transition)	98.4%		69	CCG -> CCA	C770_RS47200	outer membrane biogen	None	2.5E-197	23	3	WP_015243258.1
1,156,239	1,156,239	1	A -> G	67	SNP (transition)	97.0%	Q -> R	110	CAG -> CGG	C770_RS47200	outer membrane biogen	Substitution	2.2E-205	37	2	WP_015243258.1
1,156,466	1,156,466	1	G -> A	60	SNP (transition)	100.0%	E -> K	337	GAA -> AAA	C770_RS47200	outer membrane biogen	Substitution	1.0E-192	113	1	WP_015243258.1
1,156,512	1,156,512	1	A -> T	63	SNP (transversion)	100.0%	N -> I	383	AAT -> ATT	C770_RS47200	outer membrane biogen	Substitution	1.3E-208	128	2	WP_015243258.1
1,156,673	1,156,673	1	A -> G	59	SNP (transition)	100.0%	I -> V	544	ATC -> GTC	C770_RS47200	outer membrane biogen	Substitution	2.5E-201	182	1	WP_015243258.1
1,156,683	1,156,683	1	A -> C	61	SNP (transversion)	100.0%	E -> A	554	GAG -> GCG	C770_RS47200	outer membrane biogen	Substitution	2.5E-220	185	2	WP_015243258.1
1,156,709	1,156,709	1	A -> G	62	SNP (transition)	98.4%	M -> V	580	ATG -> GTG	C770_RS47200	outer membrane biogen	Substitution	1.6E-218	194	1	WP_015243258.1
1,156,727	1,156,727	1	G -> A	57	SNP (transition)	100.0%	E -> K	598	GAG -> AAG	C770_RS47200	outer membrane biogen	Substitution	4.0E-183	200	1	WP_015243258.1
1,156,738	1,156,738	1	C -> T	55	SNP (transition)	100.0%		609	TTC -> TTT	C770_RS47200	outer membrane biogen	None	1.0E-176	203	3	WP_015243258.1
1,156,747	1,156,747	1	G -> A	55	SNP (transition)	100.0%		618	TCG -> TCA	C770_RS47200	outer membrane biogen	None	1.0E-165	206	3	WP_015243258.1
1,156,793	1,156,793	1	G -> A	58	SNP (transition)	100.0%	A -> T	664	GCC -> ACC	C770_RS47200	outer membrane biogen	Substitution	4.0E-192	222	1	WP_015243258.1
1,156,797	1,156,797	1	T -> C	59	SNP (transition)	100.0%	V -> A	668	GTG -> GCG	C770_RS47200	outer membrane biogen	Substitution	2.0E-195	223	2	WP_015243258.1
1,156,810	1,156,810	1	C -> G	60	SNP (transversion)	100.0%		681	CCC -> CCG	C770_RS47200	outer membrane biogen	None	1.0E-192	227	3	WP_015243258.1
1,156,825	1,156,826	2	GA -> CC	61 -> 62	Substitution	100.0%	GM -> GL	696	GGG,ATG -> GG	C770_RS47200	outer membrane biogen	Substitution	4.0E-208	232	3	WP_015243258.1
1,156,829	1,156,829	1	T -> A	60	SNP (transversion)	100.0%	S -> T	700	TCC -> ACC	C770_RS47200	outer membrane biogen	Substitution	1.0E-204	234	1	WP_015243258.1
1,156,833	1,156,833	1	G -> A	57	SNP (transition)	100.0%	S -> N	704	AGC -> AAC	C770_RS47200	outer membrane biogen	Substitution	7.9E-189	235	2	WP_015243258.1
1,156,835	1,156,837	2	CTG -> TTC	58	Substitution	100.0%	L -> F	706	CTG -> TTC	C770_RS47200	outer membrane biogen	Substitution	1.6E-180	236	1	WP_015243258.1
1,156,839	1,156,839	1	C -> A	58	SNP (transversion)	98.3%	A -> E	710	GCG -> GAG	C770_RS47200	outer membrane biogen	Substitution	9.2E-193	237	2	WP_015243258.1
1,156,870	1,156,870	1	C -> T	58	SNP (transition)	100.0%		741	AAC -> AAT	C770_RS47200	outer membrane biogen	None	1.0E-203	247	3	WP_015243258.1
1,156,897	1,156,897	1	A -> G	62	SNP (transition)	100.0%		768	GAA -> GAG	C770_RS47200	outer membrane biogen	None	4.0E-199	256	3	WP_015243258.1
1,156,922	1,156,922	1	G -> A	61	SNP (transition)	98.4%	G -> S	793	GGC -> AGC	C770_RS47200	outer membrane biogen	Substitution	6.1E-203	265	1	WP_015243258.1
1,156,930	1,156,930	1	C -> T	61	SNP (transition)	100.0%		801	AAC -> AAT	C770_RS47200	outer membrane biogen	None	3.2E-214	267	3	WP_015243258.1
1,156,933	1,156,933	1	G -> C	61	SNP (transversion)	100.0%		804	GCG -> GCC	C770_RS47200	outer membrane biogen	None	4.0E-208	268	3	WP_015243258.1
1,156,954	1,156,954	1	T -> G	59	SNP (transversion)	100.0%		825	CGT -> CGG	C770_RS47200	outer membrane biogen	None	2.5E-201	275	3	WP_015243258.1
1,156,960	1,156,960	1	C -> G	60	SNP (transversion)	100.0%		831	CGC -> CGG	C770_RS47200	outer membrane biogen	None	1.0E-204	277	3	WP_015243258.1
1,157,013	1,157,013	1	C -> T	67	SNP (transition)	100.0%	A -> V	884	GCC -> GTC	C770_RS47200	outer membrane biogen	Substitution	1.6E-228	295	2	WP_015243258.1
1,157,015	1,157,015	1	C -> G	67	SNP (transversion)	100.0%	P -> A	886	CCG -> GCG	C770_RS47200	outer membrane biogen	Substitution	3.2E-235	296	1	WP_015243258.1
1,157,033	1,157,033	1	T -> A	69	SNP (transversion)	98.6%	S -> T	904	TCG -> ACG	C770_RS47200	outer membrane biogen	Substitution	2.7E-223	302	1	WP_015243258.1
1,157,037	1,157,037	1	G -> A	69	SNP (transition)	100.0%	G -> E	908	GGA -> GAA	C770_RS47200	outer membrane biogen	Substitution	1.6E-221	303	2	WP_015243258.1
1,157,067	1,157,067	1	A -> C	66	SNP (transversion)	100.0%	E -> A	938	GAG -> GCG	C770_RS47200	outer membrane biogen	Substitution	4.0E-225	313	2	WP_015243258.1
1,157,095	1,157,095	1	C -> T	61	SNP (transition)	98.4%		966	GAC -> GAT	C770_RS47200	outer membrane biogen	None	6.1E-215	322	3	WP_015243258.1
1,157,123	1,157,123	1	C -> T	67	SNP (transition)	100.0%		994	CTG -> TTG	C770_RS47200	outer membrane biogen	None	1.6E-228	332	1	WP_015243258.1
1,157,126	1,157,127	2	GT -> AC	66	Substitution	98.5%	V -> T	997	GTC -> ACC	C770_RS47200	outer membrane biogen	Substitution	6.6E-207	333	1	WP_015243258.1
1,157,131	1,157,131	1	C -> T	67	SNP (transition)	100.0%		1.002	CTC -> CTT	C770_RS47200	outer membrane biogen	None	4.0E-215	334	3	WP_015243258.1
1,157,185	1,157,185	1	G -> A	74	SNP (transition)	100.0%		1.056	GAG -> GAA	C770_RS47200	outer membrane biogen	None	6.3E-245	352	3	WP_015243258.1
1,157,266	1,157,266	1	C -> A	80	SNP (transversion)	100.0%	D -> E	1.137	GAC -> GAA	C770_RS47200	outer membrane biogen	Substitution	1.0E-256	379	3	WP_015243258.1
1,157,284	1,157,284	1	T -> C	73	SNP (transition)	100.0%		1.155	CGT -> CGC	C770_RS47200	outer membrane biogen	None	1.3E-241	385	3	WP_015243258.1
1,157,296	1,157,296	1	G -> A	74	SNP (transition)	98.6%		1.167	AAG -> AAA	C770_RS47200	outer membrane biogen	None	3.7E-225	389	3	WP_015243258.1
1,157,311	1,157,311	1	G -> C	70	SNP (transversion)	100.0%		1.182	GCG -> GCC	C770_RS47200	outer membrane biogen	None	1.0E-238	394	3	WP_015243258.1
1,157,318	1,157,318	1	G -> T	71	SNP (transversion)	97.2%	A -> S	1.189	GCA -> TCA	C770_RS47200	outer membrane biogen	Substitution	3.9E-218	397	1	WP_015243258.1
1,157,362	1,157,362	1	A -> G	77	SNP (transition)	100.0%		1.233	ACA -> ACG	C770_RS47200	outer membrane biogen	None	1.6E-262	411	3	WP_015243258.1
1,157,384	1,157,386	2	GGC -> CGT	78 -> 79	Substitution	100.0%	G -> R	1.255	GGC -> CGT	C770_RS47200	outer membrane biogen	Substitution	6.3E-266	419	1	WP_015243258.1
1,157,400	1,157,400	1	T -> A	85	SNP (transversion)	100.0%	V -> E	1.271	GTA -> GAA	C770_RS47200	outer membrane biogen	Substitution	3.2E-281	424	2	WP_015243258.1

1,157,422	1,157,422	1	C -> T		80	SNP (transition)	97.5%		1.293	GAC -> GAT	C770_RS47200	outer membrane bioger	None	7.9E-247	431	3	WP_015243258.1
1,157,453	1,157,453	1	A -> G		85	SNP (transition)	100.0%	T -> A	1.324	ACC -> GCC	C770_RS47200	outer membrane bioger	Substitution	1.0E-272	442	1	WP_015243258.1
1,157,473	1,157,473	1	T -> C		85	SNP (transition)	98.8%		1.344	ATT -> ATC	C770_RS47200	outer membrane bioger	None	5.4E-276	448	3	WP_015243258.1
1,157,494	1,157,494	1	A -> G		82	SNP (transition)	100.0%		1.365	GAA -> GAG	C770_RS47200	outer membrane bioger	None	1.6E-279	455	3	WP_015243258.1
1,157,560	1,157,560	1	C -> A		84	SNP (transversion)	100.0%		1.431	CGC -> CGA	C770_RS47200	outer membrane bioger	None	2.5E-286	477	3	WP_015243258.1
1,157,610	1,157,610	1	C -> T		86	SNP (transition)	98.8%	A -> V	1.481	GCC -> GTC	C770_RS47200	outer membrane bioger	Substitution	2.7E-279	494	2	WP_015243258.1
1,157,618	1,157,618	1	A -> G		86	SNP (transition)	100.0%	I -> V	1.489	ATT -> GTT	C770_RS47200	outer membrane bioger	Substitution	4.0E-293	497	1	WP_015243258.1
1,157,638	1,157,638	1	T -> C		89	SNP (transition)	100.0%		1.509	TCT -> TCC	C770_RS47200	outer membrane bioger	None	2.5E-303	503	3	WP_015243258.1
1,157,677	1,157,677	1	C -> G		89	SNP (transversion)	100.0%		1.548	CCC -> CCG	C770_RS47200	outer membrane bioger	None	2.5E-303	516	3	WP_015243258.1
1,157,721	1,157,721	1	C -> G		95	SNP (transversion)	100.0%	T -> S	1.592	ACC -> AGC	C770_RS47200	outer membrane bioger	Substitution	0.0	531	2	WP_015243258.1
1,157,738	1,157,738	1	G -> A		92	SNP (transition)	100.0%	G -> S	1.609	GGT -> AGT	C770_RS47200	outer membrane bioger	Substitution	0.0	537	1	WP_015243258.1
1,157,743	1,157,743	1	T -> C		94	SNP (transition)	100.0%		1.614	TTT -> TTC	C770_RS47200	outer membrane bioger	None	0.0	538	3	WP_015243258.1
1,157,863	1,157,863	1	T -> A		83	SNP (transversion)	98.8%	N -> K	1.734	AAT -> AAA	C770_RS47200	outer membrane bioger	Substitution	1.3E-277	578	3	WP_015243258.1
1,157,935	1,157,937	3	CAT -> TTC		87	Substitution	97.7%	IM -> IS	1.806	ATC,ATG -> ATT,'	C770_RS47200	outer membrane bioger	Substitution	2.0E-270	602	3	WP_015243258.1
1,157,939	1,157,941	2	GCC -> ACG		86	Substitution	100.0%	A -> T	1.81	GCC -> ACG	C770_RS47200	outer membrane bioger	Substitution	1.6E-284	604	1	WP_015243258.1
1,157,947	1,157,948	2	GA -> TG		83	Substitution	98.8%	LN -> LD	1.818	CTG,AAC -> CTT,(	C770_RS47200	outer membrane bioger	Substitution	1.3E-274	606	3	WP_015243258.1
1,157,959	1,157,959	1	A -> G		78	SNP (transition)	100.0%		1.83	GAA -> GAG	C770_RS47200	outer membrane bioger	None	6.3E-266	610	3	WP_015243258.1
1,158,166	1,158,166	1	A -> G		80	SNP (transition)	100.0%		2.037	GCA -> GCG	C770_RS47200	outer membrane bioger	None	1.0E-272	679	3	WP_015243258.1
1,158,170	1,158,170	1	T -> C		81	SNP (transition)	100.0%		2.041	TTG -> CTG	C770_RS47200	outer membrane bioger	None	4.0E-276	681	1	WP_015243258.1
1,158,181	1,158,181	1	C -> G		82	SNP (transversion)	98.8%		2.052	CCC -> CCG	C770_RS47200	outer membrane bioger	None	3.3E-274	684	3	WP_015243258.1
1,158,301	1,158,301	1	C -> G		78	SNP (transversion)	100.0%		2.172	ACC -> ACG	C770_RS47200	outer membrane bioger	None	4.0E-258	724	3	WP_015243258.1
1,158,346	1,158,347	2	AT -> GC	76 -> 77		Substitution	100.0%	EF -> EL	2.217	GAA,TTC -> GAG,	C770_RS47200	outer membrane bioger	Substitution	4.0E-247	739	3	WP_015243258.1
1,158,412	1,158,412	1	T -> A		80	SNP (transversion)	100.0%	N -> K	2.283	AAT -> AAA	C770_RS47200	outer membrane bioger	Substitution	1.0E-256	761	3	WP_015243258.1
1,158,478	1,158,478	1	T -> C		75	SNP (transition)	100.0%		2.349	AGT -> AGC	C770_RS47200	outer membrane bioger	None	3.2E-248	783	3	WP_015243258.1
1,158,547	1,158,547	1	T -> C		70	SNP (transition)	100.0%		2.418	TTT -> TTC	C770_RS47200	outer membrane bioger	None	1.0E-238	806	3	WP_015243258.1
1,158,618	1,158,618	1	A -> G		78	SNP (transition)	100.0%	N -> S	2.489	AAC -> AGC	C770_RS47200	outer membrane bioger	Substitution	4.0E-258	830	2	WP_015243258.1
1,158,661	1,158,661	1	T -> C		68	SNP (transition)	100.0%		2.532	CTT -> CTC	C770_RS47200	outer membrane bioger	None	4.0E-225	844	3	WP_015243258.1
1,158,682	1,158,682	1	C -> T		67	SNP (transition)	98.5%		2.553	TCC -> TCT	C770_RS47200	outer membrane bioger	None	6.7E-197	851	3	WP_015243258.1
1,158,794	1,158,794	1	A -> G		79	SNP (transition)	100.0%	N -> D	2.665	AAT -> GAT	C770_RS47200	outer membrane bioger	Substitution	2.5E-269	889	1	WP_015243258.1
1,158,811	1,158,811	1	A -> G		79	SNP (transition)	100.0%		2.682	TTA -> TTG	C770_RS47200	outer membrane bioger	None	2.5E-269	894	3	WP_015243258.1
1,158,825	1,158,825	1	A -> C		76	SNP (transversion)	100.0%	D -> A	2.696	GAT -> GCT	C770_RS47200	outer membrane bioger	Substitution	1.0E-266	899	2	WP_015243258.1
1,158,873	1,158,873	1	A -> G		68	SNP (transition)	100.0%	E -> G	2.744	GAA -> GGA	C770_RS47200	outer membrane bioger	Substitution	6.3E-232	915	2	WP_015243258.1
1,158,877	1,158,877	1	C -> T		70	SNP (transition)	100.0%		2.748	GAC -> GAT	C770_RS47200	outer membrane bioger	None	1.0E-245	916	3	WP_015243258.1
1,158,883	1,158,883	1	C -> G		71	SNP (transversion)	100.0%		2.754	GCC -> GCG	C770_RS47200	outer membrane bioger	None	5.0E-235	918	3	WP_015243258.1
1,158,890	1,158,890	1	G -> T		73	SNP (transversion)	100.0%	A -> S	2.761	GCC -> TCC	C770_RS47200	outer membrane bioger	Substitution	6.3E-249	921	1	WP_015243258.1
1,159,074	1,159,074	1	A -> T		79	SNP (transversion)	100.0%	Q -> L	2.945	CAA -> CTA	C770_RS47200	outer membrane bioger	Substitution	1.6E-253	982	2	WP_015243258.1
1,159,121	1,159,121	1	A -> G		75	SNP (transition)	100.0%	M -> V	2.992	ATG -> GTG	C770_RS47200	outer membrane bioger	Substitution	3.2E-263	998	1	WP_015243258.1
1,159,153	1,159,153	1	A -> G		69	SNP (transition)	100.0%		3.024	TAA -> TAG	C770_RS47200	outer membrane bioger	None	3.2E-242	1.008	3	WP_015243258.1
1,159,154	1,159,154	1	T -> C		69	SNP (transition)	100.0%							3.2E-242			
1,159,165	1,159,165	1	(C)3 -> (C)2		70	Deletion (tandem repeat)	100.0%							1.0E-105			
1,159,177	1,159,177	1	G		70	Deletion	100.0%							1.0E-105			
1,159,184	1,159,184	1	G -> T		72	SNP (transversion)	100.0%							1.6E-245			
1,159,187	1,159,187	1	C -> A		72	SNP (transversion)	100.0%							6.3E-260			
1,159,195	1,159,195	1	G -> A		70	SNP (transition)	100.0%							1.0E-238			
1,159,229	1,159,229	1	G -> A		63	SNP (transition)	100.0%		717	CAC -> CAT	C770_RS47205	hypothetical protein	None	3.2E-221	239	3	WP_010975434.1
1,159,252	1,159,252	1	G -> A		64	SNP (transition)	100.0%		694	CTG -> TTG	C770_RS47205	hypothetical protein	None	1.0E-224	232	1	WP_010975434.1
1,159,268	1,159,268	1	C -> G		63	SNP (transversion)	100.0%		678	CGG -> CGC	C770_RS47205	hypothetical protein	None	3.2E-221	226	3	WP_010975434.1
1,159,271	1,159,271	1	G -> A		62	SNP (transition)	100.0%		675	GAC -> GAT	C770_RS47205	hypothetical protein	None	1.0E-217	225	3	WP_010975434.1
1,159,277	1,159,277	1	G -> T		61	SNP (transversion)	100.0%		669	CCC -> CCA	C770_RS47205	hypothetical protein	None	3.2E-214	223	3	WP_010975434.1
1,159,301	1,159,301	1	C -> T		60	SNP (transition)	100.0%		645	CCG -> CCA	C770_RS47205	hypothetical protein	None	1.0E-210	215	3	WP_010975434.1
1,159,307	1,159,308	2	CC -> TG	62 -> 63		Substitution	100.0%	R -> T	638	AGG -> ACA	C770_RS47205	hypothetical protein	Substitution	1.3E-208	213	2	WP_010975434.1
1,159,319	1,159,319	1	C -> T		65	SNP (transition)	100.0%		627	AAG -> AAA	C770_RS47205	hypothetical protein	None	1.0E-208	209	3	WP_010975434.1
1,159,329	1,159,329	1	G -> A		66	SNP (transition)	100.0%	T -> I	617	ACC -> ATC	C770_RS47205	hypothetical protein	Substitution	1.0E-231	206	2	WP_010975434.1
1,159,334	1,159,334	1	G -> A		68	SNP (transition)	100.0%		612	TTC -> TTT	C770_RS47205	hypothetical protein	None	6.3E-232	204	3	WP_010975434.1
1,159,340	1,159,340	1	T -> C		65	SNP (transition)	100.0%		606	GAA -> GAG	C770_RS47205	hypothetical protein	None	3.2E-228	202	3	WP_010975434.1
1,159,349	1,159,349	1	A -> G		65	SNP (transition)	98.5%		597	CCT -> CCC	C770_RS47205	hypothetical protein	None	4.1E-210	199	3	WP_010975434.1
1,159,355	1,159,355	1	C -> T		66	SNP (transition)	100.0%		591	GGG -> GGA	C770_RS47205	hypothetical protein	None	4.0E-225	197	3	WP_010975434.1
1,159,361	1,159,361	1	C -> T		65	SNP (transition)	98.5%		585	AGG -> AGA	C770_RS47205	hypothetical protein	None	2.6E-229	195	3	WP_010975434.1
1,159,364	1,159,364	1	G -> A		66	SNP (transition)	100.0%		582	GAC -> GAT	C770_RS47205	hypothetical protein	None	1.0E-231	194	3	WP_010975434.1
1,159,374	1,159,374	1	T -> C		67	SNP (transition)	100.0%	N -> S	572	AAC -> AGC	C770_RS47205	hypothetical protein	Substitution	3.2E-235	191	2	WP_010975434.1
1,159,376	1,159,376	1	G -> T		68	SNP (transversion)	100.0%		570	CGC -> CGA	C770_RS47205	hypothetical protein	None	6.3E-232	190	3	WP_010975434.1
1,159,385	1,159,385	1	T -> C		67	SNP (transition)	100.0%		561	GAA -> GAG	C770_RS47205	hypothetical protein	None	4.0E-215	187	3	WP_010975434.1
1,159,409	1,159,411	2	TAC -> GAT		68	Substitution	98.5%	V -> I	535	GTA -> ATC	C770_RS47205	hypothetical protein	Substitution	7.9E-222	179	1	WP_010975434.1
1,159,415	1,159,415	1	A -> G		68	SNP (transition)	98.5%		531	GAT -> GAC	C770_RS47205	hypothetical protein	None	5.4E-220	177	3	WP_010975434.1
1,159,426	1,159,426	1	C -> T		66	SNP (transition)	100.0%	V -> I	520	GTT -> ATT	C770_RS47205	hypothetical protein	Substitution	6.3E-212	174	1	WP_010975434.1
1,159,433	1,159,433	1	G -> A		66	SNP (transition)	100.0%		513	AAC -> AAT	C770_RS47205	hypothetical protein	None	4.0E-225	171	3	WP_010975434.1
1,159,454	1,159,454	1	C -> G		62	SNP (transversion)	100.0%		492	TCG -> TCC	C770_RS47205	hypothetical protein	None	1.0E-186	164	3	WP_010975434.1
1,159,459	1,159,459	1	T -> C		60	SNP (transition)	100.0%	K -> E	487	AAG -> GAG	C770_RS47205	hypothetical protein	Substitution	1.0E-186	163	1	WP_010975434.1
1,159,463	1,159,463	1	G -> A		60	SNP (transition)	96.7%		483	AAC -> AAT	C770_RS47205	hypothetical protein	None	4.4E-183	161	3	WP_010975434.1
1,159,480	1,159,480	1	C -> T		61	SNP (transition)	100.0%	E -> K	466	GAG -> AAG	C770_RS47205	hypothetical protein	Substitution	5.0E-202	156	1	WP_010975434.1
1,159,502	1,159,502	1	A -> G		67	SNP (transition)	97.0%		444	TGT -> TGC	C770_RS47205	hypothetical protein	None	7.0E-212	148	3	WP_010975434.1
1,159,520	1,159,521	2	GC -> AG		66	Substitution	100.0%	S -> T	425	AGC -> ACT	C770_RS47205	hypothetical protein	Substitution	6.3E-212	142	2	WP_010975434.1
1,159,532	1,159,532	1	A -> G		66	SNP (transition)	100.0%		414	GAT -> GAC	C770_RS47205	hypothetical protein	None	1.6E-218	138	3	WP_010975434.1
1,159,535	1,159,535	1	G -> C		69	SNP (transversion)	100.0%		411	GCC -> GCG	C770_RS47205	hypothetical protein	None	1.6E-221	137	3	WP_010975434.1
1,159,553	1,159,553	1	A -> G		66	SNP (transition)	100.0%		393	AAT -> AAC	C770_RS47205	hypothetical protein	None	2.5E-238	131	3	WP_010975434.1
1,159,565	1,159,565	1	A -> C		62	SNP (transversion)	96.8%		381	GTT -> GTG	C770_RS47205	hypothetical protein	None	1.9E-201	127	3	WP_010975434.1
1,159,568	1,159,568	1	A -> C		62	SNP (transversion)	98.4%		378	GCT -> GCG	C770_RS47205	hypothetical protein	None	3.1E-200	126	3	WP_010975434.1
1,159,611	1,159,612	2	TG -> GT	48 -> 49		Substitution	100.0%	Q -> T	334	CAG -> ACG	C770_RS47205	hypothetical protein	Substitution	6.3E-164	112	1	WP_010975434.1
1,159,631	1,159,631	1	G -> A		45	SNP (transition)	100.0%		315	TTC -> TTT	C770_RS47205	hypothetical protein	None	1.0E-153	105	3	WP_010975434.1
1,159,636	1,159,636	1	A -> C		44	SNP (transversion)	100.0%	S -> A	310	TCC -> GCC	C770_RS47205	hypothetical protein	Substitution	2.5E-150	104	1	WP_010975434.1
1,159,640	1,159,640	1	G -> A		44	SNP (transition)	100.0%		306	ATC -> ATT	C770_RS47205	hypothetical protein	None	2.5E-150	102	3	WP_010975434.1
1,159,655	1,159,655	1	C -> T		39	SNP (transition)	100.0%		291	TCG -> TCA	C770_RS47205	hypothetical protein	None	2.5E-133	97	3	WP_010975434.1
1,159,661	1,159,661	1	A -> G		39	SNP (transition)	100.0%		285	GAT -> GAC	C770_RS47205	hypothetical protein	None	3.2E-137	95	3	WP_010975434.1
1,159,666	1,159,666	1	C -> T		36	SNP (transition)	100.0%	A -> T	280	GCG -> ACG	C770_RS47205	hypothetical protein	Substitution	1.0E-126	94	1	WP_010975434.1
1,159,671	1,159,671	1	C -> T		36	SNP (transition)	100.0%	R -> K	275	AGG -> AAG	C770_RS47205	hypothetical protein	Substitution	2.5E-130	92	2	WP_010975434.1
1,159,682	1,159,682	1	A -> G		34	SNP (transition)	100.0%		264	CGT -> CGC	C770_RS47205	hypothetical protein	None	4.0E-123	88	3	WP_010975434.1
1,159,698	1,159,698	1	A -> G		29	SNP (transition)	100.0%	V -> A	248	GTC -> GCC	C770_RS47205	hypothetical protein	Substitution	3.2E-102	83	2	WP_010975434.1
1,159,700	1,159,701	2	AC -> CA		29	Substitution	100.0%	G -> V	245	GGT -> GTG	C770_RS47205	hypothetical protein	Substitution	3.2E-102	82	2	WP_010975434.1
1,159,715	1,159,715	1	G -> A		27	SNP (transition)	100.0%		231	GTC -> GTT	C770_RS47205	hypothetical protein	None	1.6E-92	77	3	WP_010975434.1
1,159,730	1,159,730	1	A -> G		25	SNP (transition)	100.0%		216	ATT -> ATC	C770_RS47205	hypothetical protein	None	3.2E-88	72	3	WP_010975434.1
1,159,763	1,159,763	1	G -> A		18	SNP (transition)	100.0%		183	CAC -> CAT	C770_RS47205	hypothetical protein	None	4.0E-60	61	3	WP_010975434.1
1,159,766	1,159,766	1	G -> A		16	SNP (transition)	100.0%		180	TGC -> TGT	C770_RS47205	hypothetical protein	None	2.5E-58	60	3	WP_010975434.1
1,159,775	1,159,775	1	A -> G		11	SNP (transition)	100.0%		171	GCT -> GCC	C770_RS47205	hypothetical protein	None	2.5E-40	57	3	WP_010975434.1
1,159,784	1,159,784	1	C -> T		8	SNP (transition)	100.0%		162	GCG -> GCA	C770_RS47205	hypothetical protein	None	1.6E-29	54	3	WP_010975434.1
1,159,787	1,159,787	1	C -> T		8	SNP (transition)	100.0%		159	CTG -> CTA	C770_RS47205	hypothetical protein	None	2.5E-30	53	3	WP_010975434.1
1,160,114	1,160,114	1	A -> G		7	SNP (transition)	100.0%							1.3E-19			
1,160,121	1,160,121	1	C -> T		7	SNP (transition)	100.0%							4.0E-23			
1,160,124	1,160,124	1	A -> C		7	SNP (transversion)	100.0%							3.2E-18			
1,160,134	1,160,134	1	G -> C		11	SNP (transversion)	100.0%							1.0E-33			
1,160,148	1,160,148	1	G -> A		16	SNP (transition)	100.0%							2.5E-50			
1,160,155	1,160,154	0	CG		16	Insertion	100.0%							2.5E-50			
1,160,166	1,160,166	1	T -> A		16	SNP (transversion)	100.0%							4.0E-55			
1,160,169	1,160,172	4	ATCA		16	Deletion	100.0%							2.5E-26			
1,160,184	1,160,184	1	A -> C		19	SNP (transversion)	100.0%							2.5E-65			
1,160,189	1,160,190	2	CG -> TC		21	Substitution	100.0%							1.0E-63			
1,160,195	1,160,195	1	G -> A		23	SNP (transition)	100.0%							3.2E-81			
1,160,208	1,160,208	1	A -> G		29	SNP (transition)	100.0%							1.6E-93			
1,160,219	1,160,219	1	A -> G		33	SNP (transition)	100.0%							5.0E-103			
1,160,221	1,160,221	1	C -> G		33	SNP (transversion)	100.0%							2.5E-106			
1,160,233	1,160,234	2	TG -> CTAA		35	Insertion	97.1%							1.0E-112			
1,160,241	1,160,241	1	T -> C		38	SNP (transition)	100.0%							6.3E-130			
1,160,246	1,160,247	2	CA -> AT		41	Substitution	100.0%							4.0E-140			
1,160,251	1,160,250	0	A		44	Insertion	100.0%							1.6E-141			
1,160,252	1,160,252	1	C -> A		46	SNP (transversion)	100.0%							6.3E-148			
1,160,254	1,160,258	5	TTGCT -> AATTG	46 -> 50		Substitution	95.7% -> 96.0%							1.5E-147			
1,160,264	1,160,264	1	T -> G		52	SNP (transversion)	100.0%							1.0E-182			
1,160,266	1,160,266	1	C -> A		52	SNP (transversion)	100.0%							2.5E-172			
1,160,277	1,160,277	1	C -> A		53	SNP (transversion)	100.0%							2.5E-170			
1,160,292	1,160,293	2	CG -> AT		55	Substitution	100.0%	A -> D	1.796	GCG -> GAT	C770_RS47210	multidrug ABC transpor	Substitution	3.2E-182	599	2	WP_015243259.1
1,160,295	1,160,295	1	C -> G		56	SNP (transversion)	100.0%		1.794	GCG -> GCC	C770_RS47210	multidrug ABC transpor	None	1.6E-185	598	3	WP_015243259.1
1,160,301	1,160,301	1	T -> G		58	SNP (transversion)	100.0%		1.788	GTA -> GTC	C770_RS47210	multidrug ABC transpor	None	1.0E-203	596	3	WP_015243259.1
1,160,315	1,160,315	1	G -> A		67	SNP (transition)	97.0%		1.774	CTG -> TTG	C770_RS47210	multidrug ABC transpor	None	7.0E-212	592	1	WP_015243259.1
1,160,321	1,160,321	1	C -> T		68	SNP (transition)	98.5%	G -> S	1.768	GGC -> AGC	C770_RS47210	multidrug ABC transpor	Substitution	1.1E-226	590	1	WP_015243259.1
1,160,349	1,160,349	1	G -> A		70	SNP (transition)	100.0%		1.74	GTC -> GTT	C770_RS47210	multidrug ABC transpor	None	1.0E-231	580	3	WP_015243259.1
1,160,378	1,160,378	1	C -> G		76	SNP (transversion)	100.0%	E -> Q	1.711	GAG -> CAG	C770_RS47210	multidrug ABC transpor	Substitution	1.6E-251	571	1	WP_015243259.1
1,160,394	1,160,394	1	C -> T		75	SNP (transition)	100.0%		1.695	GTG -> GTA	C770_RS47210	multidrug ABC transpor	None	1.0E-255	565	3	WP_015243259.1
1,160,409	1,160,409	1	G -> C		78	SNP (transversion)	100.0%	N -> K	1.68	AAC -> AAG	C770_RS47210	multidrug ABC transpor	Substitution	6.3E-266	560	3	WP_015243259.1
1,160,430	1,160,430	1	G -> A		76	SNP (transition)	98.7%		1.659	GCC -> GCT	C770_RS47210	multidrug ABC transpor	None	2.4E-261	553	3	WP_015243259.1
1,160,436	1,160,436	1	A -> G		78	SNP (transition)	98.7%		1.653	GTT -> GTC	C770_RS47210	multidrug ABC transpor	None	1.2E-260	551	3	WP_015243259.1
1,160,514	1,160,514	1	T -> C		92	SNP (transition)	100.0%		1.575	GCA -> GCG	C770_RS47210	multidrug ABC transpor	None	2.5E-304	525	3	WP_015243259.1
1,160,523	1,160,523	1	C -> T		91	SNP (transition)	100.0%		1.566	GAG -> GAA	C770_RS47210	multidrug ABC transpor	None	6.3E-292	522	3	WP_015243259.1
1,160,532	1,160,532	1	A -> G		89	SNP (transition)	98.9%		1.557	CTT -> CTC	C770_RS47210	multidrug ABC transpor	None	3.5E-289	519	3	WP_015243259.1
1,160,535	1,160,535	1	A -> C		87	SNP (transversion)	98.9%		1.554	GCT -> GCG	C770_RS47210	multidrug ABC transpor	None	3.5E-291	518	3	WP_015243259.1
1,160,565	1,160,565	1	A -> G		88	SNP (transition)	96.6%		1.524	CCT -> CCC	C770_RS47210	multidrug ABC transpor	None	1.1E-284	508	3	WP_015243259.1
1,160,592	1,160,592	1	G -> A		77	SNP (transition)	100.0%		1.497	ATC -> ATT	C770_RS47210	multidrug ABC transpor	None	1.6E-262	499	3	WP_015243259.1
1,160,598	1,160,598	1	G -> A		81	SNP (transition)	100.0%		1.491	CTC -> CTT	C770_RS47210	multidrug ABC transpor	None	4.0E-276	497	3	WP_015243259.1
1,160,601	1,160,601	1	G -> A		81	SNP (transition)	100.0%		1.488	CGC -> CGT	C770_RS47210	multidrug ABC transpor	None	4.0E-276	496	3	WP_015243259.1
1,160,624	1,160,624	1	A -> G		91	SNP (transition)	98.9%		1.465	TTA -> CTA	C770_RS47210	multidrug ABC transpor	None	9.1E-314	489	1	WP_015243259.1
1,160,631	1,160,631	1	G -> A		94	SNP (transition)	100.0%		1.458	GGC -> GGT	C770_RS47210	multidrug ABC transpor	None	0.0	486	3	WP_015243259.1
1,160,634	1,160,634	1	T -> C		97	SNP (transition)	100.0%		1.455	CAA -> CAG	C770_RS47210	multidrug ABC transpor	None	0.0	485	3	WP_015243259.1
1,160,640	1,160,640	1	A -> G		98	SNP (transition)	99.0%		1.449	GGT -> GGC	C770_RS47210	multidrug ABC transpor	None	0.0	483	3	WP_015243259.1
1,160,655	1,160,655	1	G -> A		101	SNP (transition)	100.0%		1.434	TAC -> TAT	C770_RS47210	multidrug ABC transpor	None	0.0	478	3	WP_015243259.1
1,160,658	1,160,658	1	C -> G		101	SNP (transversion)	100.0%		1.431	GGG -> GGC	C770_RS47210	multidrug ABC transpor	None	0.0	477	3	WP_015243259.1
1,160,664	1,160,664	1	T -> C		100	SNP (transition)	100.0%		1.425	CCA -> CCG	C770_RS47210	multidrug ABC transpor	None	0.0	475	3	WP_015243259.1
1,160,691	1,160,691	1	A -> G		101	SNP (transition)	100.0%		1.398	TAT -> TAC	C770_RS47210	multidrug ABC transpor	None	0.0	466	3	WP_015243259.1
1,160,703	1,160,703	1	G -> A		98	SNP (transition)	96.9%		1.386	GCC -> GCT	C770_RS47210	multidrug ABC transpor	None	1.5E-299	462	3	WP_015243259.1
1,160,715	1,160,715	1	T -> G		97	SNP (transversion)	100.0%		1.374	GTA -> GTC	C770_RS47210	multidrug ABC transpor	None	0.0	458	3	WP_015243259.1
1,160,718	1,160,718	1	T -> G		96	SNP (transversion)	100.0%	E -> D	1.371	GAA -> GAC	C770_RS47210	multidrug ABC transpor	Substitution	0.0	457	3	WP_015243259.1
1,160,739	1,160,739	1	C -> T		86	SNP (transition)	100.0%		1.35	CGG -> CGA	C770_RS47210	multidrug ABC transpor	None	1.0E-301	450	3	WP_015243259.1
1,160,766	1,160,766	1	G -> A		76	SNP (transition)	100.0%		1.323	TCC -> TCT	C770_RS47210	multidrug ABC transpor	None	4.0E-259	441	3	WP_015243259.1
1,160,769	1,160,769	1	A -> G		74	SNP (transition)	100.0%		1.32	GGT -> GGC	C770_RS47210	multidrug ABC transpor	None	1.0E-259	440	3	WP_015243259.1
1,160,775	1,160,775	1	A -> G		67	SNP (transition)	100.0%		1.314	TTT -> TTC	C770_RS47210	multidrug ABC transpor	None	1.6E-228	438	3	WP_015243259.1
1,160,781	1,160,781	1	G -> A		64	SNP (transition)	100.0%		1.308	TAC -> TAT	C770_RS47210	multidrug ABC transpor	None	1.0E-224	436	3	WP_015243259.1
1,160,784	1,160,786	2	AGC -> GGG	63 -> 64		Substitution	100.0%	A -> P	1.303	GCT -> CCC	C770_RS47210	multidrug ABC transpor	Substitution	3.2E-221	435	1	WP_015243259.1
1,160,790	1,160,790	1	T -> C		63	SNP (transition)	100.0%		1.299	CAA -> CAG	C770_RS47210	multidrug ABC transpor	None	1.6E-227	433	3	WP_015243259.1
1,160,793	1,160,793	1	T -> C		62	SNP (transition)	96.8%		1.296	TCA -> TCG	C770_RS47210	multidrug ABC transpor	None	1.9E-213	432	3	WP_015243259.1
1,160,808	1,160,808	1	C -> G		58	SNP (transversion)	100.0%		1.281	GGG -> GGC	C770_RS47210	multidrug ABC transpor	None	1.0E-203	427	3	WP_015243259.1
1,160,811	1,160,814	4	GTTG -> AGCA		58	Substitution	100.0%	RN -> RA	1.275	CGC,AAC -> CGT,	C770_RS47210	multidrug ABC transpor	Substitution	6.3E-198	425	3	WP_015243259.1
1,160,817	1,160,817	1	C -> A		58	SNP (transversion)	100.0%		1.272	CTG -> CTT	C770_RS47210	multidrug ABC transpor	None	4.0E-192	424	3	WP_015243259.1
1,160,820	1,160,820	1	A -> C		57	SNP (transversion)	98.2%		1.269	TCT -> TCG	C770_RS47210	multidrug ABC transpor	None	9.0E-184	423	3	WP_015243259.1

1,160,825	1,160,825	1	G -> T		55	SNP (transversion)	98.2%	Q -> K	1.264	CAG -> AAG	C770_RS47210	multidrug ABC transpor	Substitution	8.7E-172	422	1	WP_015243259.1
1,160,832	1,160,832	1	A -> G		53	SNP (transition)	100.0%		1.257	GTT -> GTC	C770_RS47210	multidrug ABC transpor	None	3.2E-186	419	3	WP_015243259.1
1,160,835	1,160,836	2	AG -> GC		53	Substitution	100.0%	A -> G	1.253	GCT -> GGC	C770_RS47210	multidrug ABC transpor	Substitution	6.3E-181	418	2	WP_015243259.1
1,160,841	1,160,841	1	T -> G		51	SNP (transversion)	100.0%		1.248	ATA -> ATC	C770_RS47210	multidrug ABC transpor	None	3.2E-179	416	3	WP_015243259.1
1,160,844	1,160,847	3	GTCT -> ATTC	49 -> 50		Substitution	98.0%	QD -> QN	1.242	CAA,GAC -> CAG,	C770_RS47210	multidrug ABC transpor	Substitution	1.6E-157	414	3	WP_015243259.1
1,160,850	1,160,850	1	C -> G		49	SNP (transversion)	100.0%		1.239	GGG -> GGC	C770_RS47210	multidrug ABC transpor	None	2.5E-167	413	3	WP_015243259.1
1,160,866	1,160,866	1	A -> G		44	SNP (transition)	100.0%	V -> A	1.223	GTG -> GCG	C770_RS47210	multidrug ABC transpor	Substitution	2.5E-150	408	2	WP_015243259.1
1,160,871	1,160,871	1	C -> G		43	SNP (transversion)	100.0%		1.218	TCG -> TCC	C770_RS47210	multidrug ABC transpor	None	6.3E-147	406	3	WP_015243259.1
1,160,892	1,160,892	1	T -> C		37	SNP (transition)	100.0%		1.197	CCA -> CCG	C770_RS47210	multidrug ABC transpor	None	7.9E-123	399	3	WP_015243259.1
1,160,895	1,160,895	1	A -> G		34	SNP (transition)	100.0%		1.194	ATT -> ATC	C770_RS47210	multidrug ABC transpor	None	1.6E-109	398	3	WP_015243259.1
1,160,910	1,160,910	1	C -> G		28	SNP (transversion)	100.0%		1.179	ACG -> ACC	C770_RS47210	multidrug ABC transpor	None	2.5E-90	393	3	WP_015243259.1
1,160,913	1,160,913	1	C -> G		27	SNP (transversion)	100.0%		1.176	TCG -> TCC	C770_RS47210	multidrug ABC transpor	None	4.0E-87	392	3	WP_015243259.1
1,160,916	1,160,916	1	T -> C		26	SNP (transition)	100.0%		1.173	AAA -> AAG	C770_RS47210	multidrug ABC transpor	None	1.0E-78	391	3	WP_015243259.1
1,160,925	1,160,925	1	T -> G		22	SNP (transversion)	100.0%		1.164	GGA -> GGC	C770_RS47210	multidrug ABC transpor	None	2.5E-73	388	3	WP_015243259.1
1,160,934	1,160,934	1	G -> C		15	SNP (transversion)	100.0%		1.155	GGC -> GGG	C770_RS47210	multidrug ABC transpor	None	3.2E-50	385	3	WP_015243259.1
1,160,937	1,160,937	1	A -> G		14	SNP (transition)	100.0%		1.152	GTT -> GTC	C770_RS47210	multidrug ABC transpor	None	2.5E-48	384	3	WP_015243259.1
1,160,946	1,160,946	1	G -> C		12	SNP (transversion)	100.0%		1.143	ACC -> ACG	C770_RS47210	multidrug ABC transpor	None	1.6E-41	381	3	WP_015243259.1
1,160,949	1,160,949	1	A -> C		11	SNP (transversion)	100.0%		1.14	ACT -> ACG	C770_RS47210	multidrug ABC transpor	None	4.0E-38	380	3	WP_015243259.1
1,160,961	1,160,961	1	T -> G		9	SNP (transversion)	100.0%	E -> D	1.128	GAA -> GAC	C770_RS47210	multidrug ABC transpor	Substitution	4.0E-33	376	3	WP_015243259.1
1,160,964	1,160,964	1	T -> C		9	SNP (transition)	100.0%		1.125	GCA -> GCG	C770_RS47210	multidrug ABC transpor	None	2.5E-31	375	3	WP_015243259.1
1,163,776	1,163,777	2	AC -> GT		6	Substitution	100.0%							6.3E-14			
1,163,779	1,163,780	2	AG -> CT		6	Substitution	100.0%							1.0E-15			
1,163,782	1,163,787	6	CGCTGC -> AAGA		6	Substitution	100.0%							1.6E-14			
1,163,789	1,163,789	1	G -> C		6	SNP (transversion)	100.0%							4.0E-18			
1,163,791	1,163,791	1	G -> T		8	SNP (transversion)	100.0%							4.0E-27			
1,163,794	1,163,799	6	GAGCGG -> TCA/	8 -> 10		Substitution	100.0%							1.0E-27			
1,163,801	1,163,805	5	CAATT -> TGTCA	10 -> 11		Substitution	100.0%							1.0E-30			
1,163,807	1,163,813	7	CAGCGGC -> GG'	11 -> 13		Substitution	100.0%							7.9E-35			
1,163,815	1,163,821	7	CGCGTTC -> GAC	15 -> 19		Substitution	100.0%							3.2E-44			
1,163,823	1,163,825	3	GAA -> TTG		19	Substitution	100.0%							1.6E-61			
1,163,829	1,163,829	1	T -> G		20	SNP (transversion)	100.0%		294	CGA -> CGC	C770_RS47220	hypothetical protein	None	1.0E-68	98	3	WP_013850942.1
1,163,874	1,163,874	1	T -> C		26	SNP (transition)	100.0%		249	CCA -> CCG	C770_RS47220	hypothetical protein	None	1.0E-91	83	3	WP_013850942.1
1,163,898	1,163,898	1	C -> A		30	SNP (transversion)	100.0%	E -> D	225	GAG -> GAT	C770_RS47220	hypothetical protein	Substitution	1.0E-108	75	3	WP_013850942.1
1,163,916	1,163,916	1	G -> A		35	SNP (transition)	100.0%		207	ATC -> ATT	C770_RS47220	hypothetical protein	None	1.0E-119	69	3	WP_013850942.1
1,163,926	1,163,926	1	G -> A		38	SNP (transition)	97.4%	A -> V	197	GCA -> GTA	C770_RS47220	hypothetical protein	Substitution	6.0E-125	66	2	WP_013850942.1
1,164,009	1,164,009	1	G -> A		58	SNP (transition)	100.0%		114	CTC -> CTT	C770_RS47220	hypothetical protein	None	1.0E-203	38	3	WP_013850942.1
1,164,147	1,164,147	1	G -> C		75	SNP (transversion)	100.0%							1.0E-225			
1,164,225	1,164,225	1	A -> G		73	SNP (transition)	98.6%							1.8E-236			
1,164,230	1,164,230	1	A -> T		72	SNP (transversion)	98.6%							4.5E-226			
1,164,236	1,164,235	0	(A)2 -> (A)3		73	Insertion (tandem repeat)	100.0%							2.5E-234			
1,164,244	1,164,244	1	A -> G		75	SNP (transition)	100.0%							3.2E-233			
1,164,268	1,164,268	1	(G)3 -> (G)2		75	Deletion (tandem repeat)	100.0%							3.2E-113			
1,164,276	1,164,276	1	A -> C		77	SNP (transversion)	100.0%							7.9E-255			
1,164,295	1,164,295	1	C -> T		78	SNP (transition)	98.7%	R -> H	2.03	CGC -> CAC	C770_RS47225	acyltransferase	Substitution	6.2E-253	677	2	WP_015243261.1
1,164,303	1,164,303	1	A -> C		78	SNP (transversion)	98.7%		2.022	GCT -> GCG	C770_RS47225	acyltransferase	None	1.6E-237	674	3	WP_015243261.1
1,164,324	1,164,324	1	C -> T		79	SNP (transition)	100.0%	M -> I	2.001	ATG -> ATA	C770_RS47225	acyltransferase	Substitution	1.6E-253	667	3	WP_015243261.1
1,164,340	1,164,340	1	C -> T		86	SNP (transition)	98.8%	R -> H	1.985	CGT -> CAT	C770_RS47225	acyltransferase	Substitution	2.7E-279	662	2	WP_015243261.1
1,164,349	1,164,349	1	G -> A		89	SNP (transition)	100.0%	A -> V	1.976	GCA -> GTA	C770_RS47225	acyltransferase	Substitution	2.5E-303	659	2	WP_015243261.1
1,164,387	1,164,387	1	C -> T		84	SNP (transition)	100.0%		1.938	GGG -> GGA	C770_RS47225	acyltransferase	None	1.6E-269	646	3	WP_015243261.1
1,164,408	1,164,408	1	G -> A		85	SNP (transition)	98.8%		1.917	GGC -> GGT	C770_RS47225	acyltransferase	None	5.4E-276	639	3	WP_015243261.1
1,164,421	1,164,421	1	G -> A		81	SNP (transition)	98.8%	A -> V	1.904	GCT -> GTT	C770_RS47225	acyltransferase	Substitution	8.1E-255	635	2	WP_015243261.1
1,164,435	1,164,435	1	G -> A		76	SNP (transition)	98.7%		1.89	GCC -> GCT	C770_RS47225	acyltransferase	None	7.6E-224	630	3	WP_015243261.1
1,164,448	1,164,448	1	G -> T		80	SNP (transversion)	100.0%	A -> E	1.877	GCA -> GAA	C770_RS47225	acyltransferase	Substitution	1.0E-240	626	2	WP_015243261.1
1,164,531	1,164,531	1	G -> C		67	SNP (transversion)	100.0%		1.794	ACC -> ACG	C770_RS47225	acyltransferase	None	6.3E-242	598	3	WP_015243261.1
1,164,621	1,164,621	1	C -> A		60	SNP (transversion)	100.0%		1.704	GTG -> GTT	C770_RS47225	acyltransferase	None	1.0E-204	568	3	WP_015243261.1
1,164,630	1,164,630	1	A -> C		59	SNP (transversion)	100.0%		1.695	GTT -> GTG	C770_RS47225	acyltransferase	None	2.5E-201	565	3	WP_015243261.1
1,164,660	1,164,660	1	T -> G		58	SNP (transversion)	100.0%		1.665	CTA -> CTC	C770_RS47225	acyltransferase	None	1.6E-209	555	3	WP_015243261.1
1,164,753	1,164,753	1	A -> C		66	SNP (transversion)	100.0%		1.572	GGT -> GGG	C770_RS47225	acyltransferase	None	1.6E-218	524	3	WP_015243261.1
1,164,765	1,164,765	1	A -> T		68	SNP (transversion)	100.0%	D -> E	1.56	GAT -> GAA	C770_RS47225	acyltransferase	Substitution	2.5E-218	520	3	WP_015243261.1
1,164,816	1,164,816	1	C -> G		69	SNP (transversion)	100.0%	K -> N	1.509	AAG -> AAC	C770_RS47225	acyltransferase	Substitution	2.5E-235	503	3	WP_015243261.1
1,165,096	1,165,096	1	T -> A		95	SNP (transversion)	98.9%	N -> I	1.229	AAT -> ATT	C770_RS47225	acyltransferase	Substitution	1.5E-299	410	2	WP_015243261.1
1,165,125	1,165,125	1	G -> A		94	SNP (transition)	100.0%		1.2	TTC -> TTT	C770_RS47225	acyltransferase	None	1.6E-301	400	3	WP_015243261.1
1,165,149	1,165,149	1	T -> C		97	SNP (transition)	99.0%		1.176	CAA -> CAG	C770_RS47225	acyltransferase	None	1.5E-315	392	3	WP_015243261.1
1,165,179	1,165,179	1	C -> T		94	SNP (transition)	100.0%		1.146	AGG -> AGA	C770_RS47225	acyltransferase	None	2.5E-320	382	3	WP_015243261.1
1,165,191	1,165,191	1	A -> G		92	SNP (transition)	100.0%		1.134	GGT -> GGC	C770_RS47225	acyltransferase	None	1.0E-322	378	3	WP_015243261.1
1,165,224	1,165,224	1	G -> A		97	SNP (transition)	100.0%		1.101	GTC -> GTT	C770_RS47225	acyltransferase	None	0.0	367	3	WP_015243261.1
1,165,259	1,165,259	1	T -> C		88	SNP (transition)	100.0%	I -> V	1.066	ATC -> GTC	C770_RS47225	acyltransferase	Substitution	6.3E-300	356	1	WP_015243261.1
1,165,386	1,165,386	1	G -> A		93	SNP (transition)	98.9%		939	GGC -> GGT	C770_RS47225	acyltransferase	None	9.3E-321	313	3	WP_015243261.1
1,165,401	1,165,403	2	CAA -> AAG	91 -> 92		Substitution	100.0%		922	TTG -> CTT	C770_RS47225	acyltransferase	None	4.0E-295	308	1	WP_015243261.1
1,165,448	1,165,448	1	T -> C		88	SNP (transition)	100.0%	I -> V	877	ATC -> GTC	C770_RS47225	acyltransferase	Substitution	2.5E-282	293	1	WP_015243261.1
1,165,476	1,165,476	1	A -> G		85	SNP (transition)	100.0%		849	GGT -> GGC	C770_RS47225	acyltransferase	None	3.2E-281	283	3	WP_015243261.1
1,165,542	1,165,542	1	A -> G		83	SNP (transition)	100.0%		783	CCT -> CCC	C770_RS47225	acyltransferase	None	1.0E-249	261	3	WP_015243261.1
1,165,613	1,165,614	2	AT -> CC		82	Substitution	98.8%	LL -> LV	711	CTA,TTG -> CTG,(	C770_RS47225	acyltransferase	Substitution	5.2E-258	237	3	WP_015243261.1
1,165,617	1,165,617	1	C -> T		82	SNP (transition)	98.8%		708	GGG -> GGA	C770_RS47225	acyltransferase	None	4.1E-266	236	3	WP_015243261.1
1,165,710	1,165,710	1	G -> A		80	SNP (transition)	100.0%		615	TTC -> TTT	C770_RS47225	acyltransferase	None	1.0E-272	205	3	WP_015243261.1
1,165,721	1,165,721	1	G -> A		81	SNP (transition)	100.0%		604	CTG -> TTG	C770_RS47225	acyltransferase	None	4.0E-276	202	1	WP_015243261.1
1,165,749	1,165,749	1	A -> T		84	SNP (transversion)	100.0%		576	ATT -> ATA	C770_RS47225	acyltransferase	None	1.0E-294	192	3	WP_015243261.1
1,165,806	1,165,806	1	G -> A		89	SNP (transition)	100.0%		519	ATC -> ATT	C770_RS47225	acyltransferase	None	2.5E-303	173	3	WP_015243261.1
1,165,878	1,165,878	1	C -> A		90	SNP (transversion)	100.0%		447	TCG -> TCT	C770_RS47225	acyltransferase	None	1.0E-306	149	3	WP_015243261.1
1,165,923	1,165,923	1	G -> A		84	SNP (transition)	97.6%		402	GAC -> GAT	C770_RS47225	acyltransferase	None	8.7E-268	134	3	WP_015243261.1
1,165,962	1,165,962	1	A -> T		89	SNP (transversion)	98.9%		363	ACT -> ACA	C770_RS47225	acyltransferase	None	8.9E-307	121	3	WP_015243261.1
1,165,965	1,165,965	1	G -> A		89	SNP (transition)	100.0%		360	TTC -> TTT	C770_RS47225	acyltransferase	None	1.6E-285	120	3	WP_015243261.1
1,165,968	1,165,968	1	C -> T		91	SNP (transition)	100.0%		357	TTG -> TTA	C770_RS47225	acyltransferase	None	5.0E-301	119	3	WP_015243261.1
1,166,069	1,166,069	1	C -> T		79	SNP (transition)	98.7%	A -> T	256	GCA -> ACA	C770_RS47225	acyltransferase	Substitution	5.0E-264	86	1	WP_015243261.1
1,166,196	1,166,196	1	A -> G		70	SNP (transition)	100.0%		129	TTT -> TTC	C770_RS47225	acyltransferase	None	1.0E-238	43	3	WP_015243261.1
1,166,214	1,166,214	1	G -> A		71	SNP (transition)	100.0%		111	AGC -> AGT	C770_RS47225	acyltransferase	None	6.3E-228	37	3	WP_015243261.1
1,166,361	1,166,360	0	(T)4 -> (T)5		68	Insertion (tandem repeat)	97.1%							1.4E-208			
1,166,424	1,166,424	1	A -> G		70	SNP (transition)	98.6%							1.8E-233			
1,166,437	1,166,437	1	A -> T		71	SNP (transversion)	97.2%							6.2E-232			
1,166,569	1,166,569	1	G -> A		73	SNP (transition)	100.0%		858	CCC -> CCT	C770_RS47230	arabinose-5-phosphate	None	1.6E-263	286	3	WP_010975429.1
1,166,627	1,166,627	1	C -> T		79	SNP (transition)	100.0%	S -> N	800	AGT -> AAT	C770_RS47230	arabinose-5-phosphate	Substitution	1.6E-253	267	2	WP_010975429.1
1,166,659	1,166,659	1	G -> T		71	SNP (transversion)	100.0%		768	ACC -> ACA	C770_RS47230	arabinose-5-phosphate	None	5.0E-235	256	3	WP_010975429.1
1,166,869	1,166,869	1	C -> A		72	SNP (transversion)	98.6%		558	GGG -> GGT	C770_RS47230	arabinose-5-phosphate	None	4.5E-226	186	3	WP_010975429.1
1,166,898	1,166,898	1	G -> A		75	SNP (transition)	98.7%	L -> F	529	CTT -> TTT	C770_RS47230	arabinose-5-phosphate	Substitution	4.7E-243	177	1	WP_010975429.1
1,166,911	1,166,911	1	T -> C		76	SNP (transition)	100.0%		516	GCA -> GCG	C770_RS47230	arabinose-5-phosphate	None	1.6E-251	172	3	WP_010975429.1
1,166,944	1,166,944	1	T -> A		80	SNP (transversion)	98.8%		483	CTA -> CTT	C770_RS47230	arabinose-5-phosphate	None	1.6E-259	161	3	WP_010975429.1
1,166,950	1,166,950	1	A -> G		78	SNP (transition)	100.0%		477	CAT -> CAC	C770_RS47230	arabinose-5-phosphate	None	6.3E-266	159	3	WP_010975429.1
1,166,986	1,166,986	1	G -> C		77	SNP (transversion)	98.7%		441	CCC -> CCG	C770_RS47230	arabinose-5-phosphate	None	1.2E-249	147	3	WP_010975429.1
1,166,998	1,166,998	1	A -> G		75	SNP (transition)	100.0%		429	AAT -> AAC	C770_RS47230	arabinose-5-phosphate	None	1.0E-255	143	3	WP_010975429.1
1,167,118	1,167,118	1	A -> G		75	SNP (transition)	100.0%		309	CTT -> CTC	C770_RS47230	arabinose-5-phosphate	None	3.2E-248	103	3	WP_010975429.1
1,167,123	1,167,123	1	G -> A		77	SNP (transition)	100.0%		304	CTG -> TTG	C770_RS47230	arabinose-5-phosphate	None	1.6E-262	102	1	WP_010975429.1
1,167,268	1,167,268	1	G -> C		88	SNP (transversion)	100.0%		159	GGC -> GGG	C770_RS47230	arabinose-5-phosphate	None	4.0E-291	53	3	WP_010975429.1
1,167,327	1,167,327	1	T -> C		83	SNP (transition)	100.0%	N -> D	100	AAT -> GAT	C770_RS47230	arabinose-5-phosphate	Substitution	1.6E-299	34	1	WP_010975429.1
1,167,457	1,167,457	1	T -> G		88	SNP (transversion)	100.0%							4.0E-291			
1,167,459	1,167,459	1	T -> GC		88	Insertion	97.7%							1.4E-294			
1,167,521	1,167,521	1	T -> C		73	SNP (transition)	98.6%							1.8E-236			
1,167,547	1,167,547	1	C -> G		71	SNP (transversion)	100.0%							4.0E-242			
1,167,658	1,167,658	1	A -> C		71	SNP (transversion)	97.2%							3.9E-218			
1,167,660	1,167,660	1	T -> G		70	SNP (transversion)	100.0%							1.0E-231			
1,167,667	1,167,667	1	A -> C		71	SNP (transversion)	100.0%							4.0E-242			
1,167,675	1,167,675	1	C -> T		73	SNP (transition)	100.0%							6.3E-249			
1,167,681	1,167,681	1	C -> A		74	SNP (transversion)	100.0%							6.3E-245			
1,167,699	1,167,700	2	GA -> AC		73	Substitution	98.6%							7.3E-251			
1,167,769	1,167,769	1	G -> A		69	SNP (transition)	100.0%	G -> D	68	GGC -> GAC	C770_RS47235	hypothetical protein	Substitution	1.6E-221	23	2	WP_014527380.1
1,167,840	1,167,841	2	CA -> TG	64 -> 65		Substitution	100.0%	H -> C	139	CAT -> TGT	C770_RS47235	hypothetical protein	Substitution	3.2E-202	47	1	WP_014527380.1
1,167,926	1,167,926	1	C -> G		74	SNP (transversion)	100.0%		225	CGC -> CGG	C770_RS47235	hypothetical protein	None	1.6E-237	75	3	WP_014527380.1
1,167,935	1,167,935	1	G -> C		74	SNP (transversion)	100.0%		234	TCG -> TCC	C770_RS47235	hypothetical protein	None	1.6E-237	78	3	WP_014527380.1
1,167,968	1,167,968	1	C -> T		78	SNP (transition)	98.7%		267	GGC -> GGT	C770_RS47235	hypothetical protein	None	1.2E-260	89	3	WP_014527380.1
1,167,990	1,167,990	1	T -> C		85	SNP (transition)	100.0%	S -> P	289	TCT -> CCT	C770_RS47235	hypothetical protein	Substitution	3.2E-281	97	1	WP_014527380.1
1,168,013	1,168,013	1	A -> G		79	SNP (transition)	98.7%		312	AAA -> AAG	C770_RS47235	hypothetical protein	None	7.9E-272	104	3	WP_014527380.1
1,168,173	1,168,173	1	A -> G		90	SNP (transition)	97.8%	M -> V	472	ATG -> GTG	C770_RS47235	hypothetical protein	Substitution	2.5E-296	158	1	WP_014527380.1
1,168,199	1,168,199	1	C -> G		85	SNP (transversion)	100.0%		498	GCC -> GCG	C770_RS47235	hypothetical protein	None	1.0E-255	166	3	WP_014527380.1
1,168,202	1,168,202	1	G -> C		85	SNP (transversion)	98.8%		501	GGG -> GGC	C770_RS47235	hypothetical protein	None	3.4E-259	167	3	WP_014527380.1
1,168,212	1,168,214	2	ACC -> GCG	87 -> 88		Substitution	98.9%	T -> A	511	ACC -> GCG	C770_RS47235	hypothetical protein	Substitution	4.0E-279	171	1	WP_014527380.1
1,168,238	1,168,238	1	G -> T		87	SNP (transversion)	98.9%	E -> D	537	GAG -> GAT	C770_RS47235	hypothetical protein	Substitution	5.5E-274	179	3	WP_014527380.1
1,168,244	1,168,244	1	C -> G		87	SNP (transversion)	100.0%		543	GGC -> GGG	C770_RS47235	hypothetical protein	None	4.0E-279	181	3	WP_014527380.1
1,168,246	1,168,246	1	G -> A		87	SNP (transition)	100.0%	R -> K	545	AGA -> AAA	C770_RS47235	hypothetical protein	Substitution	4.0E-279	182	2	WP_014527380.1
1,168,253	1,168,253	1	C -> G		86	SNP (transversion)	100.0%		552	CGC -> CGG	C770_RS47235	hypothetical protein	None	6.3E-276	184	3	WP_014527380.1
1,168,265	1,168,265	1	G -> T		85	SNP (transversion)	98.8%		564	GCG -> GCT	C770_RS47235	hypothetical protein	None	5.4E-276	188	3	WP_014527380.1
1,168,283	1,168,283	1	C -> G		87	SNP (transversion)	100.0%		582	CTC -> CTG	C770_RS47235	hypothetical protein	None	7.9E-288	194	3	WP_014527380.1
1,168,295	1,168,295	1	A -> C		89	SNP (transversion)	100.0%	R -> S	594	AGA -> AGC	C770_RS47235	hypothetical protein	Substitution	2.0E-294	198	3	WP_014527380.1
1,168,308	1,168,308	1	G -> T		91	SNP (transversion)	98.9%	V -> F	607	GTC -> TTC	C770_RS47235	hypothetical protein	Substitution	9.1E-287	203	1	WP_014527380.1
1,168,356	1,168,356	1	G -> A		82	SNP (transition)	98.8%	V -> I	655	GTT -> ATT	C770_RS47235	hypothetical protein	Substitution	4.1E-266	219	1	WP_014527380.1
1,168,373	1,168,373	1	C -> T		82	SNP (transition)	100.0%		672	GAC -> GAT	C770_RS47235	hypothetical protein	None	1.6E-279	224	3	WP_014527380.1
1,168,446	1,168,446	1	G -> C		73	SNP (transversion)	100.0%	T -> S	1.607	ACT -> AGT	C770_RS47240	4-amino-4-deoxy-L-arat	Substitution	6.3E-249	536	2	WP_015243263.1
1,168,508	1,168,508	1	C -> G		78	SNP (transversion)	100.0%		1.545	GCG -> GCC	C770_RS47240	4-amino-4-deoxy-L-arat	None	2.5E-250	515	3	WP_015243263.1
1,168,518	1,168,518	1	A -> G		72	SNP (transition)	98.6%	V -> A	1.535	GTC -> GCC	C770_RS47240	4-amino-4-deoxy-L-arat	Substitution	4.5E-226	512	2	WP_015243263.1
1,168,531	1,168,531	1	G -> T		69	SNP (transversion)	100.0%	Q -> K	1.522	CAA -> AAA	C770_RS47240	4-amino-4-deoxy-L-arat	Substitution	1.3E-214	508	1	WP_015243263.1
1,168,539	1,168,539	1	C -> G		67	SNP (transversion)	100.0%	G -> A	1.514	GGC -> GCC	C770_RS47240	4-amino-4-deoxy-L-arat	Substitution	2.0E-208	505	2	WP_015243263.1
1,168,558	1,168,558	1	C -> T		69	SNP (transition)	100.0%	V -> I	1.495	GTT -> ATT	C770_RS47240	4-amino-4-deoxy-L-arat	Substitution	2.0E-228	499	1	WP_015243263.1
1,168,580	1,168,580	1	A -> T		68	SNP (transversion)	98.5%		1.473	GCT -> GCA	C770_RS47240	4-amino-4-deoxy-L-arat	None	1.1E-226	491	3	WP_015243263.1
1,168,607	1,168,607	1	C -> T		70	SNP (transition)	97.1%		1.446	CCG -> CCA	C770_RS47240	4-amino-4-deoxy-L-arat	None	1.5E-228	482	3	WP_015243263.1
1,168,610	1,168,610	1	C -> G		71	SNP (transversion)	98.6%		1.443	CCG -> CCC	C770_RS47240	4-amino-4-deoxy-L-arat	None	7.1E-230	481	3	WP_015243263.1
1,168,620	1,168,620	1	T -> C		71	SNP (transition)	100.0%	K -> R	1.433	AAA -> AGA	C770_RS47240	4-amino-4-deoxy-L-arat	Substitution	4.0E-242	478	2	WP_015243263.1
1,168,634	1,168,634	1	T -> A		72	SNP (transversion)	97.2%		1.419	TCA -> TCT	C770_RS47240	4-amino-4-deoxy-L-arat	None	2.6E-221	473	3	WP_015243263.1
1,168,679	1,168,679	1	G -> A		77	SNP (transition)	100.0%		1.374	CTC -> CTT	C770_RS47240	4-amino-4-deoxy-L-arat	None	2.0E-239	458	3	WP_015243263.1
1,168,718	1,168,718	1	G -> C		75	SNP (transversion)	100.0%		1.335	GTC -> GTG	C770_RS47240	4-amino-4-deoxy-L-arat	None	3.2E-248	445	3	WP_015243263.1
1,168,826	1,168,826	1	A -> C		76	SNP (transversion)	100.0%		1.227	CTT -> CTG	C770_RS47240	4-amino-4-deoxy-L-arat	None	1.0E-266	409	3	WP_015243263.1
1,168,902	1,168,902	1	T -> C		79	SNP (transition)	100.0%	D -> G	1.151	GAC -> GGC	C770_RS47240	4-amino-4-deoxy-L-arat	Substitution	2.0E-261	384	2	WP_015243263.1
1,168,988	1,168,988	1	G -> C		73	SNP (transversion)	100.0%	I -> M	1.065	ATC -> ATG	C770_RS47240	4-amino-4-deoxy-L-arat	Substitution	1.3E-241	355	3	WP_015243263.1
1,169,138	1,169,138	1	C -> T		85	SNP (transition)	100.0%		915	CTG -> CTA	C770_RS47240	4-amino-4-deoxy-L-arat	None	1.0E-272	305	3	WP_015243263.1
1,169,146	1,169,146	1	A -> C		80	SNP (transversion)	98.8%	S -> A	907	TCG -> GCG	C770_RS47240	4-amino-4-deoxy-L-arat	Substitution	1.6E-259	303	1	WP_015243263.1
1,169,165	1,169,165	1	G -> C		85	SNP (transversion)	98.8%		888	GGC -> GGG	C770_RS47240	4-amino-4-deoxy-L-arat	None	5.4E-276	296	3	WP_015243263.1
1,169,222	1,169,222	1	A -> G		93	SNP (transition)	98.9%		831	ACT -> ACC	C770_RS47240	4-amino-4-deoxy-L-arat	None	2.3E-302	277	3	WP_015243263.1
1,169,330	1,169,330	1	A -> T		76	SNP (transversion)	98.7%	N -> K	723	AAT -> AAA	C770_RS47240	4-amino-4-deoxy-L-arat	Substitution	2.4E-246	241	3	WP_015243263.1
1,169,396	1,169,396	1	T -> C		80	SNP (transition)	100.0%		657	AAA -> AAG	C770_RS47240	4-amino-4-deoxy-L-arat	None	1.0E-272	219	3	WP_015243263.1
1,169,399	1,169,399	1	C -> G		80	SNP (transversion)	100.0%		654	TCG -> TCC	C770_RS47240	4-amino-4-deoxy-L-arat	None	1.0E-256	218	3	WP_015243263.1
1,169,446	1,169,446	1	T -> C		78	SNP (transition)	98.7%	S -> G	607	AGC -> GGC	C770_RS47240	4-amino-4-deoxy-L-arat	Substitution	3.1E-245	203	1	WP_015243263.1
1,169,477	1,169,477	1	A -> G		69	SNP (transition)	100.0%		576	TTT -> TTC	C770_RS47240	4-amino-4-deoxy-L-arat	None	2.5E-235	192	3	WP_015243263.1
1,169,618	1,169,618	1	G -> A		71	SNP (transition)	98.6%		435	CGC -> CGT	C770_RS47240	4-amino-4-deoxy-L-arat	None	7.1E-230	145	3	WP_015243263.1

1,169,621	1,169,621	1	C -> G		72	SNP (transversion)	100.0%		432	GCG -> GCC	C770_RS47240	4-amino-4-deoxy-L-arat	None	1.6E-245	144	3	WP_015243263.1
1,169,627	1,169,627	1	G -> A		72	SNP (transition)	98.6%		426	CTC -> CTT	C770_RS47240	4-amino-4-deoxy-L-arat	None	4.5E-226	142	3	WP_015243263.1
1,169,650	1,169,650	1	G -> A		73	SNP (transition)	100.0%	L -> F	403	CTC -> TTC	C770_RS47240	4-amino-4-deoxy-L-arat	Substitution	6.3E-249	135	1	WP_015243263.1
1,169,684	1,169,684	1	C -> T		71	SNP (transition)	98.6%		369	GCG -> GCA	C770_RS47240	4-amino-4-deoxy-L-arat	None	7.1E-251	123	3	WP_015243263.1
1,169,741	1,169,741	1	T -> C		82	SNP (transition)	100.0%		312	CCA -> CCG	C770_RS47240	4-amino-4-deoxy-L-arat	None	1.0E-287	104	3	WP_015243263.1
1,169,837	1,169,837	1	A -> G		59	SNP (transition)	100.0%		216	ATT -> ATC	C770_RS47240	4-amino-4-deoxy-L-arat	None	4.0E-213	72	3	WP_015243263.1
1,169,922	1,169,922	1	T -> C		31	SNP (transition)	96.8%	D -> G	131	GAC -> GGC	C770_RS47240	4-amino-4-deoxy-L-arat	Substitution	3.1E-98	44	2	WP_015243263.1
1,169,943	1,169,943	1	A -> G		25	SNP (transition)	100.0%	I -> T	110	ATT -> ACT	C770_RS47240	4-amino-4-deoxy-L-arat	Substitution	3.2E-88	37	2	WP_015243263.1
1,170,002	1,170,002	1	C -> T		9	SNP (transition)	100.0%		51	AGG -> AGA	C770_RS47240	4-amino-4-deoxy-L-arat	None	1.3E-28	17	3	WP_015243263.1
1,170,015	1,170,015	1	G -> T		11	SNP (transversion)	100.0%	S -> Y	38	TCC -> TAC	C770_RS47240	4-amino-4-deoxy-L-arat	Substitution	5.0E-37	13	2	WP_015243263.1
1,170,054	1,170,054	1	C -> G		10	SNP (transversion)	100.0%							1.0E-32			
1,170,070	1,170,070	1	T -> C		14	SNP (transition)	100.0%							1.6E-52			
1,170,085	1,170,085	1	G -> A		17	SNP (transition)	100.0%							3.2E-60			
1,170,963	1,170,963	1	G -> A		86	SNP (transition)	100.0%							1.0E-301			
1,170,989	1,170,990	2	CC -> TT		90	Substitution	100.0%							1.0E-297			
1,171,116	1,171,116	1	C -> G		82	SNP (transversion)	100.0%		792	GTG -> GTC	C770_RS47250	hypothetical protein	None	1.6E-279	264	3	WP_015243264.1
1,171,217	1,171,217	1	T -> C		86	SNP (transition)	100.0%	I -> V	691	ATC -> GTC	C770_RS47250	hypothetical protein	Substitution	1.6E-284	231	1	WP_015243264.1
1,171,226	1,171,226	1	A -> G		88	SNP (transition)	98.9%	F -> L	682	TTC -> CTC	C770_RS47250	hypothetical protein	Substitution	3.5E-277	228	1	WP_015243264.1
1,171,302	1,171,302	1	G -> T		94	SNP (transversion)	98.9%		606	GCC -> GCA	C770_RS47250	hypothetical protein	None	4.9E-324	202	3	WP_015243264.1
1,171,315	1,171,315	1	C -> T		91	SNP (transition)	100.0%	G -> E	593	GGA -> GAA	C770_RS47250	hypothetical protein	Substitution	5.0E-301	198	2	WP_015243264.1
1,171,431	1,171,432	2	TT -> CG		96	Substitution	97.9%	Q -> P	476	CAA -> CCG	C770_RS47250	hypothetical protein	Substitution	2.9E-307	159	2	WP_015243264.1
1,171,488	1,171,488	1	G -> A		93	SNP (transition)	98.9%		420	TCC -> TCT	C770_RS47250	hypothetical protein	None	9.3E-321	140	3	WP_015243264.1
1,171,503	1,171,503	1	A -> G		93	SNP (transition)	100.0%		405	TGT -> TGC	C770_RS47250	hypothetical protein	None	6.3E-317	135	3	WP_015243264.1
1,171,535	1,171,535	1	A -> T		91	SNP (transversion)	98.9%	F -> I	373	TTC -> ATC	C770_RS47250	hypothetical protein	Substitution	9.1E-305	125	1	WP_015243264.1
1,171,632	1,171,632	1	G -> C		99	SNP (transversion)	100.0%		276	TCC -> TCG	C770_RS47250	hypothetical protein	None	0.0	92	3	WP_015243264.1
1,171,698	1,171,698	1	G -> A		93	SNP (transition)	100.0%		210	AAC -> AAT	C770_RS47250	hypothetical protein	None	6.3E-317	70	3	WP_015243264.1
1,171,836	1,171,836	1	C -> G		96	SNP (transversion)	100.0%	R -> S	72	AGG -> AGC	C770_RS47250	hypothetical protein	Substitution	0.0	24	3	WP_015243264.1
1,171,926	1,171,926	1	-C		71	Deletion	100.0%							2.5E-114			
1,171,962	1,171,962	1	G -> A		74	SNP (transition)	100.0%							1.0E-259			
1,171,964	1,171,964	1	C -> A		74	SNP (transversion)	100.0%							1.6E-237			
1,172,011	1,172,012	2	TT -> CC		69	Substitution	100.0%	SK -> SE	690	TCA,AAA -> TCG,	C770_RS47255	hypothetical protein	Substitution	2.5E-235	230	3	WP_010975424.1
1,172,165	1,172,165	1	A -> G		68	SNP (transition)	100.0%		537	CCT -> CCC	C770_RS47255	hypothetical protein	None	4.0E-225	179	3	WP_010975424.1
1,172,261	1,172,261	1	A -> G		66	SNP (transition)	100.0%		441	GAT -> GAC	C770_RS47255	hypothetical protein	None	1.0E-231	147	3	WP_010975424.1
1,172,310	1,172,310	1	A -> T		74	SNP (transversion)	100.0%	F -> Y	392	TTC -> TAC	C770_RS47255	hypothetical protein	Substitution	2.5E-252	131	2	WP_010975424.1
1,172,393	1,172,393	1	G -> A		83	SNP (transition)	98.8%		309	CGC -> CGT	C770_RS47255	hypothetical protein	None	8.3E-286	103	3	WP_010975424.1
1,172,549	1,172,549	1	C -> G		93	SNP (transversion)	100.0%	L -> F	153	TTG -> TTC	C770_RS47255	hypothetical protein	Substitution	1.3E-307	51	3	WP_010975424.1
1,172,604	1,172,604	1	T -> C		88	SNP (transition)	100.0%	N -> S	98	AAT -> AGT	C770_RS47255	hypothetical protein	Substitution	6.3E-300	33	2	WP_010975424.1
1,172,860	1,172,860	1	A -> G		65	SNP (transition)	98.5%		715	TTG -> CTG	C770_RS47260	hypothetical protein	None	2.6E-229	239	1	WP_015243265.1
1,173,077	1,173,077	1	C -> T		80	SNP (transition)	100.0%		498	CTG -> CTA	C770_RS47260	hypothetical protein	None	1.0E-272	166	3	WP_015243265.1
1,173,110	1,173,110	1	T -> G		77	SNP (transversion)	100.0%		465	CTA -> CTC	C770_RS47260	hypothetical protein	None	1.6E-262	155	3	WP_015243265.1
1,173,116	1,173,116	1	T -> C		80	SNP (transition)	100.0%		459	CTA -> CTG	C770_RS47260	hypothetical protein	None	1.0E-280	153	3	WP_015243265.1
1,173,122	1,173,122	1	G -> C		83	SNP (transversion)	98.8%	I -> M	453	ATC -> ATG	C770_RS47260	hypothetical protein	Substitution	3.3E-261	151	3	WP_015243265.1
1,173,149	1,173,149	1	G -> T		82	SNP (transversion)	100.0%		426	GCC -> GCA	C770_RS47260	hypothetical protein	None	4.0E-263	142	3	WP_015243265.1
1,173,211	1,173,211	1	G -> A		82	SNP (transition)	100.0%		364	CTG -> TTG	C770_RS47260	hypothetical protein	None	4.0E-263	122	1	WP_015243265.1
1,173,215	1,173,215	1	A -> G		80	SNP (transition)	100.0%		360	GAT -> GAC	C770_RS47260	hypothetical protein	None	1.0E-280	120	3	WP_015243265.1
1,173,239	1,173,239	1	G -> C		78	SNP (transversion)	100.0%	S -> R	336	AGC -> AGG	C770_RS47260	hypothetical protein	Substitution	1.0E-273	112	3	WP_015243265.1
1,173,298	1,173,298	1	C -> T		79	SNP (transition)	100.0%	V -> I	277	GTC -> ATC	C770_RS47260	hypothetical protein	Substitution	4.0E-285	93	1	WP_015243265.1
1,173,332	1,173,332	1	T -> G		76	SNP (transversion)	100.0%		243	GCA -> GCC	C770_RS47260	hypothetical protein	None	4.0E-259	81	3	WP_015243265.1
1,173,341	1,173,341	1	C -> G		75	SNP (transversion)	100.0%		234	CCG -> CCC	C770_RS47260	hypothetical protein	None	3.2E-248	78	3	WP_015243265.1
1,173,348	1,173,348	1	G -> C		76	SNP (transversion)	100.0%	A -> G	227	GCT -> GGT	C770_RS47260	hypothetical protein	Substitution	1.6E-251	76	2	WP_015243265.1
1,173,394	1,173,394	1	T -> C		85	SNP (transition)	100.0%	I -> V	181	ATT -> GTT	C770_RS47260	hypothetical protein	Substitution	3.2E-281	61	1	WP_015243265.1
1,173,444	1,173,444	1	G -> C		91	SNP (transversion)	98.9%	A -> G	131	GCC -> GGC	C770_RS47260	hypothetical protein	Substitution	9.1E-287	44	2	WP_015243265.1
1,173,482	1,173,482	1	T -> C		84	SNP (transition)	100.0%		93	GTA -> GTG	C770_RS47260	hypothetical protein	None	6.3E-278	31	3	WP_015243265.1
1,173,514	1,173,514	1	A -> G		88	SNP (transition)	98.9%		61	TTG -> CTG	C770_RS47260	hypothetical protein	None	2.8E-303	21	1	WP_015243265.1
1,173,520	1,173,520	1	A -> G		85	SNP (transition)	100.0%	C -> R	55	TGT -> CGT	C770_RS47260	hypothetical protein	Substitution	1.0E-289	19	1	WP_015243265.1
1,173,576	1,173,576	1	T -> G		85	SNP (transversion)	100.0%							3.2E-281			
1,173,651	1,173,651	1	C -> T		68	SNP (transition)	100.0%							2.5E-218			
1,173,667	1,173,667	1	A -> T		60	SNP (transversion)	100.0%							1.0E-198			
1,173,670	1,173,669	0	(G)2 -> (G)3		58	Insertion (tandem repeat)	100.0%							1.6E-180			
1,173,675	1,173,676	2	TA -> GT		55	Substitution	100.0%							1.0E-176			
1,173,691	1,173,691	1	C -> A		48	SNP (transversion)	95.8%							2.8E-163			
1,173,697	1,173,697	1	G -> A		44	SNP (transition)	97.7%							1.4E-149			
1,173,699	1,173,699	1	T -> A		44	SNP (transversion)	100.0%							1.6E-141			
1,173,701	1,173,701	1	G -> A		44	SNP (transition)	100.0%							1.6E-141			
1,173,710	1,173,710	1	G -> T		43	SNP (transversion)	100.0%							3.2E-151			
1,173,712	1,173,712	1	A -> G		42	SNP (transition)	100.0%							1.0E-147			
1,173,715	1,173,715	1	T -> C		38	SNP (transition)	100.0%							6.3E-130			
1,173,717	1,173,717	1	G -> T		38	SNP (transversion)	100.0%							4.0E-126			
1,173,719	1,173,719	1	C -> T		37	SNP (transition)	97.3%							5.9E-118			
1,173,725	1,173,728	4	TTTG -> CGCT	35 -> 36		Substitution	97.1% -> 97.2%							8.8E-115			
1,173,736	1,173,735	0	TACAC		33	Insertion	100.0%							1.3E-109			
1,173,741	1,173,741	1	T -> C		32	SNP (transition)	96.9%							1.6E-101			
1,173,745	1,173,745	1	T -> C		32	SNP (transition)	100.0%							4.0E-103			
1,173,749	1,173,749	1	G -> C		30	SNP (transversion)	100.0%							1.0E-102			
1,173,752	1,173,751	0	AC	29 -> 30		Insertion	96.6% -> 96.7%							3.2E-102			
1,173,753	1,173,753	1	G -> T		29	SNP (transversion)	100.0%							2.5E-99			
1,173,758	1,173,759	2	AG		28	Deletion	100.0%							1.6E-45			
1,173,762	1,173,762	1	C -> A		28	SNP (transversion)	100.0%							6.3E-96			
1,173,777	1,173,777	1	T -> AC		23	Insertion	100.0%							3.2E-81			
1,173,780	1,173,780	1	G -> A		22	SNP (transition)	100.0%							1.0E-77			
1,173,789	1,173,790	2	AG -> TA		20	Substitution	100.0%							1.0E-64			
1,173,792	1,173,792	1	C -> A		20	SNP (transversion)	100.0%							1.0E-64			
1,173,820	1,173,822	2	GCT -> ACC	14 -> 15		Substitution	100.0%	A -> T	16	GCT -> ACC	C770_RS47265	glycosyltransferase invo	Substitution	2.5E-48	6	1	WP_015243266.1
1,173,825	1,173,826	2	AC -> GG		13	Substitution	100.0%	KR -> KG	21	AAA,CGG -> AAG	C770_RS47265	glycosyltransferase invo	Substitution	3.2E-46	7	3	WP_015243266.1
1,173,837	1,173,838	2	GA -> AC	7 -> 8		Substitution	100.0%		33	ACG,AGA -> ACA	C770_RS47265	glycosyltransferase invo	None	1.6E-24	11	3	WP_015243266.1
1,174,902	1,174,902	1	G -> A		8	SNP (transition)	100.0%		126	CCG -> CCA	C770_RS47270	nucleoside-diphosphate	None	4.0E-27	42	3	WP_015243267.1
1,174,908	1,174,908	1	A -> C		10	SNP (transversion)	100.0%		132	GTA -> GTC	C770_RS47270	nucleoside-diphosphate	None	1.0E-31	44	3	WP_015243267.1
1,174,912	1,174,914	2	CTG -> TTA	10 -> 11		Substitution	100.0%		136	CTG -> TTA	C770_RS47270	nucleoside-diphosphate	None	1.0E-29	46	1	WP_015243267.1
1,174,941	1,174,941	1	A -> G		20	SNP (transition)	100.0%		165	AAA -> AAG	C770_RS47270	nucleoside-diphosphate	None	1.0E-68	55	3	WP_015243267.1
1,174,957	1,174,957	1	C -> T		27	SNP (transition)	100.0%		181	CTG -> TTG	C770_RS47270	nucleoside-diphosphate	None	1.0E-81	61	1	WP_015243267.1
1,174,971	1,174,971	1	A -> G		31	SNP (transition)	100.0%		195	AGA -> AGG	C770_RS47270	nucleoside-diphosphate	None	4.0E-106	65	3	WP_015243267.1
1,174,983	1,174,983	1	G -> C		31	SNP (transversion)	100.0%		207	CCG -> CCC	C770_RS47270	nucleoside-diphosphate	None	4.0E-106	69	3	WP_015243267.1
1,174,986	1,174,986	1	A -> G		31	SNP (transition)	100.0%		210	GAA -> GAG	C770_RS47270	nucleoside-diphosphate	None	3.2E-109	70	3	WP_015243267.1
1,174,998	1,174,998	1	G -> C		35	SNP (transversion)	100.0%		222	TCG -> TCC	C770_RS47270	nucleoside-diphosphate	None	3.2E-116	74	3	WP_015243267.1
1,175,019	1,175,019	1	T -> G		41	SNP (transversion)	100.0%		243	CCT -> CCG	C770_RS47270	nucleoside-diphosphate	None	4.0E-140	81	3	WP_015243267.1
1,175,022	1,175,022	1	G -> A		44	SNP (transition)	100.0%		246	GCG -> GCA	C770_RS47270	nucleoside-diphosphate	None	1.0E-154	82	3	WP_015243267.1
1,175,034	1,175,034	1	C -> A		50	SNP (transversion)	100.0%		258	ATC -> ATA	C770_RS47270	nucleoside-diphosphate	None	1.0E-160	86	3	WP_015243267.1
1,175,049	1,175,049	1	C -> A		57	SNP (transversion)	98.2%		273	GGC -> GGA	C770_RS47270	nucleoside-diphosphate	None	5.7E-195	91	3	WP_015243267.1
1,175,052	1,175,052	1	T -> C		60	SNP (transition)	100.0%		276	GGT -> GGC	C770_RS47270	nucleoside-diphosphate	None	1.0E-204	92	3	WP_015243267.1
1,175,058	1,175,061	3	TCTC -> CTTA	60 -> 61		Substitution	100.0%		282	CCT,CTC -> CCC,T	C770_RS47270	nucleoside-diphosphate	None	1.0E-192	94	3	WP_015243267.1
1,175,070	1,175,070	1	C -> T		62	SNP (transition)	100.0%		294	CAC -> CAT	C770_RS47270	nucleoside-diphosphate	None	1.6E-211	98	3	WP_015243267.1
1,175,103	1,175,103	1	T -> C		75	SNP (transition)	100.0%		327	CTT -> CTC	C770_RS47270	nucleoside-diphosphate	None	3.2E-248	109	3	WP_015243267.1
1,175,140	1,175,140	1	G -> A		87	SNP (transition)	100.0%	D -> N	364	GAC -> AAC	C770_RS47270	nucleoside-diphosphate	Substitution	2.0E-270	122	1	WP_015243267.1
1,175,144	1,175,144	1	A -> G		89	SNP (transition)	98.9%	Q -> R	368	CAG -> CGG	C770_RS47270	nucleoside-diphosphate	Substitution	2.2E-280	123	2	WP_015243267.1
1,175,160	1,175,160	1	C -> T		90	SNP (transition)	98.9%		384	CAC -> CAT	C770_RS47270	nucleoside-diphosphate	None	1.8E-292	128	3	WP_015243267.1
1,175,192	1,175,192	1	C -> G		89	SNP (transversion)	97.8%	S -> W	416	TCG -> TGG	C770_RS47270	nucleoside-diphosphate	Substitution	1.6E-275	139	2	WP_015243267.1
1,175,225	1,175,225	1	G -> T		84	SNP (transversion)	100.0%	R -> L	449	CGC -> CTC	C770_RS47270	nucleoside-diphosphate	Substitution	6.3E-278	150	2	WP_015243267.1
1,175,229	1,175,229	1	G -> A		82	SNP (transition)	98.8%		453	AGG -> AGA	C770_RS47270	nucleoside-diphosphate	None	3.3E-274	151	3	WP_015243267.1
1,175,238	1,175,238	1	G -> A		78	SNP (transition)	97.4%		462	TCG -> TCA	C770_RS47270	nucleoside-diphosphate	None	1.2E-255	154	3	WP_015243267.1
1,175,244	1,175,244	1	A -> C		77	SNP (transversion)	100.0%	E -> D	468	GAA -> GAC	C770_RS47270	nucleoside-diphosphate	Substitution	7.9E-255	156	3	WP_015243267.1
1,175,247	1,175,247	1	C -> G		77	SNP (transversion)	100.0%		471	GGC -> GGG	C770_RS47270	nucleoside-diphosphate	None	1.0E-231	157	3	WP_015243267.1
1,175,253	1,175,253	1	G -> A		78	SNP (transition)	100.0%		477	CCG -> CCA	C770_RS47270	nucleoside-diphosphate	None	4.0E-258	159	3	WP_015243267.1
1,175,280	1,175,280	1	A -> G		83	SNP (transition)	98.8%		504	TCA -> TCG	C770_RS47270	nucleoside-diphosphate	None	1.3E-277	168	3	WP_015243267.1
1,175,286	1,175,286	1	C -> A		83	SNP (transversion)	100.0%		510	GGC -> GGA	C770_RS47270	nucleoside-diphosphate	None	2.5E-266	170	3	WP_015243267.1
1,175,289	1,175,289	1	C -> A		82	SNP (transversion)	98.8%		513	GCC -> GCA	C770_RS47270	nucleoside-diphosphate	None	4.1E-266	171	3	WP_015243267.1
1,175,298	1,175,298	1	T -> C		83	SNP (transition)	100.0%		522	TAT -> TAC	C770_RS47270	nucleoside-diphosphate	None	2.5E-266	174	3	WP_015243267.1
1,175,306	1,175,306	1	A -> G		83	SNP (transition)	100.0%	K -> R	530	AAA -> AGA	C770_RS47270	nucleoside-diphosphate	Substitution	2.5E-266	177	2	WP_015243267.1
1,175,315	1,175,316	2	AG -> GA	82 -> 85		Substitution	100.0%	Q -> R	539	CAG -> CGA	C770_RS47270	nucleoside-diphosphate	Substitution	1.6E-279	180	2	WP_015243267.1
1,175,326	1,175,326	1	T -> G		85	SNP (transversion)	100.0%	C -> G	550	TGC -> GGC	C770_RS47270	nucleoside-diphosphate	Substitution	1.0E-289	184	1	WP_015243267.1
1,175,331	1,175,332	2	TG -> AA		88	Substitution	100.0%	AV -> AI	555	GCT,GTC -> GCA,	C770_RS47270	nucleoside-diphosphate	Substitution	4.0E-291	185	3	WP_015243267.1
1,175,337	1,175,337	1	A -> G		85	SNP (transition)	100.0%		561	GAA -> GAG	C770_RS47270	nucleoside-diphosphate	None	1.0E-289	187	3	WP_015243267.1
1,175,351	1,175,351	1	C -> A		86	SNP (transversion)	100.0%	A -> E	575	GCG -> GAG	C770_RS47270	nucleoside-diphosphate	Substitution	1.6E-284	192	2	WP_015243267.1
1,175,355	1,175,355	1	T -> C		85	SNP (transition)	100.0%		579	GGT -> GGC	C770_RS47270	nucleoside-diphosphate	None	3.2E-298	193	3	WP_015243267.1
1,175,370	1,175,370	1	G -> A		84	SNP (transition)	100.0%		594	GTG -> GTA	C770_RS47270	nucleoside-diphosphate	None	1.0E-294	198	3	WP_015243267.1
1,175,382	1,175,382	1	G -> T		83	SNP (transversion)	100.0%		606	CGG -> CGT	C770_RS47270	nucleoside-diphosphate	None	3.2E-291	202	3	WP_015243267.1
1,175,400	1,175,400	1	T -> A		79	SNP (transversion)	98.7%		624	ACT -> ACA	C770_RS47270	nucleoside-diphosphate	None	7.9E-272	208	3	WP_015243267.1
1,175,405	1,175,407	3	AAA -> GGG	78 -> 79		Substitution	98.7%	QT -> RA	629	CAA,ACC -> CGG,	C770_RS47270	nucleoside-diphosphate	Substitution	2.5E-250	210	2	WP_015243267.1
1,175,418	1,175,418	1	G -> A		80	SNP (transition)	100.0%		642	ACG -> ACA	C770_RS47270	nucleoside-diphosphate	None	1.0E-280	214	3	WP_015243267.1
1,175,433	1,175,433	1	C -> G		77	SNP (transversion)	100.0%	S -> R	657	AGC -> AGG	C770_RS47270	nucleoside-diphosphate	Substitution	4.0E-247	219	3	WP_015243267.1
1,175,436	1,175,436	1	C -> T		77	SNP (transition)	98.7%		660	ATC -> ATT	C770_RS47270	nucleoside-diphosphate	None	1.2E-249	220	3	WP_015243267.1
1,175,452	1,175,452	1	A -> C		76	SNP (transversion)	100.0%		676	AGA -> CGA	C770_RS47270	nucleoside-diphosphate	None	4.0E-259	226	1	WP_015243267.1
1,175,490	1,175,490	1	C -> T		78	SNP (transition)	100.0%		714	GCC -> GCT	C770_RS47270	nucleoside-diphosphate	None	1.0E-273	238	3	WP_015243267.1
1,175,508	1,175,508	1	G -> A		77	SNP (transition)	100.0%		732	GCG -> GCA	C770_RS47270	nucleoside-diphosphate	None	7.9E-255	244	3	WP_015243267.1
1,175,529	1,175,529	1	G -> C		73	SNP (transversion)	100.0%		753	CCG -> CCC	C770_RS47270	nucleoside-diphosphate	None	1.3E-241	251	3	WP_015243267.1
1,175,572	1,175,572	1	C -> T		63	SNP (transition)	100.0%		796	CTA -> TTA	C770_RS47270	nucleoside-diphosphate	None	2.5E-202	266	1	WP_015243267.1
1,175,625	1,175,625	1	C -> T		40	SNP (transition)	100.0%		849	GAC -> GAT	C770_RS47270	nucleoside-diphosphate	None	1.0E-132	283	3	WP_015243267.1
1,175,637	1,175,637	1	C -> T		39	SNP (transition)	100.0%		861	ACC -> ACT	C770_RS47270	nucleoside-diphosphate	None	1.6E-125	287	3	WP_015243267.1
1,175,649	1,175,649	1	G -> C		35	SNP (transversion)	100.0%		873	ACG -> ACC	C770_RS47270	nucleoside-diphosphate	None	3.2E-116	291	3	WP_015243267.1
1,175,664	1,175,664	1	C -> A		29	SNP (transversion)	100.0%		888	GGC -> GGA	C770_RS47270	nucleoside-diphosphate	None	1.0E-87	296	3	WP_015243267.1
1,175,667	1,175,667	1	A -> G		29	SNP (transition)	100.0%		891	AAA -> AAG	C770_RS47270	nucleoside-diphosphate	None	1.6E-93	297	3	WP_015243267.1
1,175,706	1,175,706	1	G -> C		19	SNP (transversion)	100.0%		930	CGG -> CGC	C770_RS47270	nucleoside-diphosphate	None	1.3E-59	310	3	WP_015243267.1
1,175,730	1,175,730	1	A -> C		15	SNP (transversion)	100.0%		954	TCA -> TCC	C770_RS47270	nucleoside-diphosphate	None	1.0E-45	318	3	WP_015243267.1
1,175,756	1,175,756	1	A		14	Deletion	100.0%							6.3E-19			
1,175,801	1,175,801	1	G -> C		21	SNP (transversion)	95.2%		816	ACC -> ACG	C770_RS47275	hypothetical protein	None	2.1E-63	272	3	WP_015243268.1
1,175,853	1,175,853	1	T -> C		35	SNP (transition)	100.0%	Q -> R	764	CAA -> CGA	C770_RS47275	hypothetical protein	Substitution	3.2E-123	255	2	WP_015243268.1
1,176,002	1,176,002	1	G -> A		63	SNP (transition)	100.0%		615	TAC -> TAT	C770_RS47275	hypothetical protein	None	1.3E-208	205	3	WP_015243268.1
1,176,191	1,176,191	1	G -> A		54	SNP (transition)	100.0%		426	TTC -> TTT	C770_RS47275	hypothetical protein	None	6.3E-179	142	3	WP_015243268.1
1,176,221	1,176,221	1	C -> T		58	SNP (transition)	100.0%		396	CCG -> CCA	C770_RS47275	hypothetical protein	None	6.3E-198	132	3	WP_015243268.1
1,176,250	1,176,250	1	C -> T		64	SNP (transition)	100.0%	V -> I	367	GTA -> ATA	C770_RS47275	hypothetical protein	Substitution	2.5E-218	123	1	WP_015243268.1
1,176,329	1,176,329	1	A -> G		76	SNP (transition)	100.0%		288	GGT -> GGC	C770_RS47275	hypothetical protein	None	1.0E-228	96	3	WP_015243268.1
1,176,365	1,176,365	1	C -> G		75	SNP (transversion)	98.7%		252	GCG -> GCC	C770_RS47275	hypothetical protein	None	1.9E-250	84	3	WP_015243268.1
1,176,443	1,176,443	1	C -> G		78	SNP (transversion)	100.0%		174	CCG -> CCC	C770_RS47275	hypothetical protein	None	2.5E-250	58	3	WP_015243268.1
1,176,802	1,176,802	1	(T)3 -> (T)2		83	Deletion (tandem repeat)	100.0%							1.6E-133			
1,176,815	1,176,815	1	G -> A		84	SNP (transition)	98.8%							5.3E-281			
1,176,825	1,176,825	1	G -> T		86	SNP (transversion)	100.0%							1.0E-301			
1,176,830	1,176,830	1	G -> A		90	SNP (transition)	100.0%							1.0E-306			
1,176,880	1,176,880	1	A -> C		84	SNP (transversion)	100.0%							1.0E-294			
1,176,885	1,176,885	1	G -> C		86	SNP (transversion)	100.0%							4.0E-293			
1,176,908	1,176,908	1	C -> G		85	SNP (transversion)	100.0%							3.2E-298			
1,176,928	1,176,928	1	T -> C		89	SNP (transition)	100.0%							2.0E-294			

1,176,937	1,176,937	1	A -> C	87	SNP (transversion)	100.0%							3.2E-305			
1,176,942	1,176,942	1	C -> G	85	SNP (transversion)	100.0%							3.2E-281			
1,176,954	1,176,954	1	A -> G	84	SNP (transition)	100.0%							6.3E-278			
1,177,028	1,177,028	1	T -> C	70	SNP (transition)	98.6%							2.2E-240			
1,177,098	1,177,098	1	C -> A	53	SNP (transversion)	100.0%	Q -> K	67	CAA -> AAA	C770_RS47280	hypothetical protein	Substitution	3.2E-186	23	1	WP_015243269.1
1,177,123	1,177,123	1	A -> T	46	SNP (transversion)	100.0%	D -> V	92	GAT -> GTT	C770_RS47280	hypothetical protein	Substitution	1.0E-161	31	2	WP_015243269.1
1,177,146	1,177,146	1	A -> T	39	SNP (transversion)	100.0%	T -> S	115	ACC -> TCC	C770_RS47280	hypothetical protein	Substitution	4.0E-141	39	1	WP_015243269.1
1,177,166	1,177,166	1	C -> G	36	SNP (transversion)	100.0%	D -> E	135	GAC -> GAG	C770_RS47280	hypothetical protein	Substitution	4.0E-123	45	3	WP_015243269.1
1,177,181	1,177,181	1	T -> A	34	SNP (transversion)	100.0%		150	GTT -> GTA	C770_RS47280	hypothetical protein	None	1.0E-119	50	3	WP_015243269.1
1,177,193	1,177,193	1	G -> T	27	SNP (transversion)	100.0%		162	GCG -> GCT	C770_RS47280	hypothetical protein	None	6.3E-98	54	3	WP_015243269.1
1,177,211	1,177,211	1	T -> C	21	SNP (transition)	100.0%		180	TAT -> TAC	C770_RS47280	hypothetical protein	None	2.5E-76	60	3	WP_015243269.1
1,177,250	1,177,250	1	T -> C	16	SNP (transition)	100.0%		219	GAT -> GAC	C770_RS47280	hypothetical protein	None	1.6E-53	73	3	WP_015243269.1
1,177,255	1,177,257	3	ATC -> GGG	15	Substitution	100.0%							1.0E-45			
1,177,259	1,177,263	5	CGGGT -> AA	12 -> 14	Deletion	100.0%							1.6E-17			
1,177,268	1,177,268	1	G -> A	11	SNP (transition)	100.0%							2.0E-30			
1,177,271	1,177,271	1	A -> G	11	SNP (transition)	100.0%							1.3E-32			
1,177,274	1,177,275	2	AT -> GG	10	Substitution	100.0%							1.0E-30			
1,177,277	1,177,279	3	ACA -> TGG	7 -> 9	Substitution	100.0%							1.0E-21			
1,177,283	1,177,287	5	GCCGC -> TGGTT	8	Substitution	100.0%							2.5E-22			
1,177,299	1,177,299	1	G -> C	6	SNP (transversion)	100.0%							4.0E-18			
1,177,305	1,177,305	1	G -> A	11	SNP (transition)	100.0%							5.0E-37			
1,177,309	1,177,309	1	A -> G	11	SNP (transition)	100.0%							5.0E-37			
1,177,312	1,177,314	3	AAT -> CTG	12 -> 13	Substitution	100.0%							2.5E-40			
1,177,316	1,177,318	3	GAA -> TGC	15 -> 16	Substitution	100.0%							1.0E-48			
1,177,320	1,177,322	3	CTA -> GCC	17	Substitution	100.0%							5.0E-50			
1,177,324	1,177,327	4	ACGA -> CGTC	17 -> 18	Substitution	100.0%							4.0E-55			
1,177,329	1,177,329	1	C -> A	18	SNP (transversion)	100.0%							6.3E-62			
1,177,331	1,177,335	5	GTTCC -> TGATG	18 -> 20	Substitution	100.0%							1.0E-62			
1,177,340	1,177,340	1	G -> A	20	SNP (transition)	100.0%							1.0E-66			
1,177,373	1,177,373	1	C -> A	25	SNP (transversion)	100.0%							1.0E-90			
1,177,451	1,177,451	1	G -> C	49	SNP (transversion)	100.0%	L -> V	526	CTT -> GTT	C770_RS47285	hypothetical protein	Substitution	4.0E-177	176	1	WP_041169896.1
1,177,512	1,177,512	1	A -> C	69	SNP (transversion)	100.0%		465	GCT -> GCG	C770_RS47285	hypothetical protein	None	3.2E-242	155	3	WP_041169896.1
1,177,529	1,177,529	1	T -> C	73	SNP (transition)	100.0%	K -> E	448	AAG -> GAG	C770_RS47285	hypothetical protein	Substitution	3.2E-256	150	1	WP_041169896.1
1,177,593	1,177,593	1	C -> T	72	SNP (transition)	98.6%		384	GAG -> GAA	C770_RS47285	hypothetical protein	None	2.9E-240	128	3	WP_041169896.1
1,177,597	1,177,597	1	A -> C	70	SNP (transversion)	100.0%	I -> S	380	ATC -> AGC	C770_RS47285	hypothetical protein	Substitution	1.0E-238	127	2	WP_041169896.1
1,177,615	1,177,615	1	A -> G	71	SNP (transition)	100.0%	M -> T	362	ATG -> ACG	C770_RS47285	hypothetical protein	Substitution	4.0E-242	121	2	WP_041169896.1
1,177,619	1,177,619	1	T -> C	71	SNP (transition)	100.0%	K -> E	358	AAG -> GAG	C770_RS47285	hypothetical protein	Substitution	7.9E-221	120	1	WP_041169896.1
1,177,654	1,177,654	1	G -> A	75	SNP (transition)	98.7%	A -> V	323	GCC -> GTC	C770_RS47285	hypothetical protein	Substitution	1.2E-235	108	2	WP_041169896.1
1,177,675	1,177,675	1	C -> T	76	SNP (transition)	100.0%	R -> K	302	AGG -> AAG	C770_RS47285	hypothetical protein	Substitution	1.6E-251	101	2	WP_041169896.1
1,177,710	1,177,710	1	A -> G	70	SNP (transition)	100.0%		267	AAT -> AAC	C770_RS47285	hypothetical protein	None	1.0E-245	89	3	WP_041169896.1
1,177,788	1,177,788	1	G -> C	56	SNP (transversion)	98.2%	I -> M	189	ATC -> ATG	C770_RS47285	hypothetical protein	Substitution	1.8E-191	63	3	WP_041169896.1
1,177,840	1,177,840	1	G -> T	66	SNP (transversion)	98.5%	A -> E	137	GCG -> GAG	C770_RS47285	hypothetical protein	Substitution	6.6E-220	46	2	WP_041169896.1
1,177,854	1,177,854	1	C -> A	66	SNP (transversion)	97.0%		123	CTG -> CTT	C770_RS47285	hypothetical protein	None	8.5E-196	41	3	WP_041169896.1
1,177,862	1,177,862	1	A -> G	66	SNP (transition)	100.0%		115	TTG -> CTG	C770_RS47285	hypothetical protein	None	1.6E-218	39	1	WP_041169896.1
1,177,872	1,177,872	1	C -> A	67	SNP (transversion)	100.0%	M -> I	105	ATG -> ATT	C770_RS47285	hypothetical protein	Substitution	1.6E-228	35	3	WP_041169896.1
1,177,879	1,177,879	1	G -> A	68	SNP (transition)	98.5%	T -> M	98	ACG -> ATG	C770_RS47285	hypothetical protein	Substitution	2.1E-233	33	2	WP_041169896.1
1,177,894	1,177,894	1	T -> C	68	SNP (transition)	98.5%	K -> R	83	AAA -> AGA	C770_RS47285	hypothetical protein	Substitution	5.4E-220	28	2	WP_041169896.1
1,177,900	1,177,900	1	G -> A	67	SNP (transition)	100.0%	P -> L	77	CCC -> CTC	C770_RS47285	hypothetical protein	Substitution	3.2E-235	26	2	WP_041169896.1
1,177,924	1,177,924	1	A -> G	71	SNP (transition)	98.6%	L -> P	53	CTC -> CCC	C770_RS47285	hypothetical protein	Substitution	7.1E-244	18	2	WP_041169896.1
1,177,929	1,177,929	1	G -> A	70	SNP (transition)	100.0%		48	CAC -> CAT	C770_RS47285	hypothetical protein	None	1.0E-231	16	3	WP_041169896.1
1,177,978	1,177,978	1	T -> G	65	SNP (transversion)	100.0%	S -> A	16	TCG -> GCG	C770_RS47290	cAMP-binding protein	Substitution	3.2E-215	6	1	WP_041169930.1
1,177,996	1,177,996	1	T -> G	67	SNP (transversion)	98.5%	F -> V	34	TTT -> GTT	C770_RS47290	cAMP-binding protein	Substitution	2.7E-223	12	1	WP_041169930.1
1,178,002	1,178,002	1	C -> A	69	SNP (transversion)	100.0%		40	CGG -> AGG	C770_RS47290	cAMP-binding protein	None	3.2E-242	14	1	WP_041169930.1
1,178,006	1,178,006	1	C -> G	68	SNP (transversion)	100.0%	P -> R	44	CCG -> CGG	C770_RS47290	cAMP-binding protein	Substitution	4.0E-225	15	2	WP_041169930.1
1,178,073	1,178,073	1	A -> G	71	SNP (transition)	98.6%		111	CGA -> CGG	C770_RS47290	cAMP-binding protein	None	7.1E-237	37	3	WP_041169930.1
1,178,199	1,178,199	1	C -> A	68	SNP (transversion)	100.0%	H -> Q	237	CAC -> CAA	C770_RS47290	cAMP-binding protein	Substitution	6.3E-232	79	3	WP_041169930.1
1,178,224	1,178,224	1	A -> G	74	SNP (transition)	100.0%	M -> V	262	ATG -> GTG	C770_RS47290	cAMP-binding protein	Substitution	6.3E-245	88	1	WP_041169930.1
1,178,242	1,178,242	1	C -> T	75	SNP (transition)	100.0%		280	CTG -> TTG	C770_RS47290	cAMP-binding protein	None	1.0E-225	94	1	WP_041169930.1
1,178,310	1,178,310	1	T -> C	78	SNP (transition)	98.7%		348	CGT -> CGC	C770_RS47290	cAMP-binding protein	None	3.1E-245	116	3	WP_041169930.1
1,178,319	1,178,319	1	G -> A	79	SNP (transition)	98.7%		357	TCG -> TCA	C770_RS47290	cAMP-binding protein	None	2.0E-248	119	3	WP_041169930.1
1,178,334	1,178,334	1	T -> C	77	SNP (transition)	100.0%		372	CAT -> CAC	C770_RS47290	cAMP-binding protein	None	3.2E-270	124	3	WP_041169930.1
1,178,349	1,178,349	1	C -> G	75	SNP (transversion)	100.0%		387	GCC -> GCG	C770_RS47290	cAMP-binding protein	None	3.2E-248	129	3	WP_041169930.1
1,178,390	1,178,390	1	G -> C	82	SNP (transversion)	100.0%	G -> A	428	GGC -> GCC	C770_RS47290	cAMP-binding protein	Substitution	6.3E-255	143	2	WP_041169930.1
1,178,422	1,178,424	2	CCA -> GCG	92 -> 93	Substitution	100.0%	P -> A	460	CCA -> GCG	C770_RS47290	cAMP-binding protein	Substitution	1.6E-313	154	1	WP_041169930.1
1,178,448	1,178,448	1	C -> G	90	SNP (transversion)	100.0%		486	GCC -> GCG	C770_RS47290	cAMP-binding protein	None	1.0E-297	162	3	WP_041169930.1
1,178,475	1,178,475	1	A -> T	85	SNP (transversion)	100.0%		513	GCA -> GCT	C770_RS47290	cAMP-binding protein	None	3.2E-281	171	3	WP_041169930.1
1,178,504	1,178,504	1	A -> G	81	SNP (transition)	100.0%	D -> G	542	GAC -> GGC	C770_RS47290	cAMP-binding protein	Substitution	5.0E-268	181	2	WP_041169930.1
1,178,508	1,178,508	1	A -> G	81	SNP (transition)	100.0%		546	TAA -> TAG	C770_RS47290	cAMP-binding protein	None	4.0E-276	182	3	WP_041169930.1
1,178,515	1,178,515	1	A -> C	85	SNP (transversion)	98.8%							8.5E-293			
1,178,520	1,178,520	1	C -> G	85	SNP (transversion)	100.0%							1.0E-289			
1,178,525	1,178,524	0	TT	84	Insertion	98.8%							1.6E-269			
1,178,550	1,178,550	1	G -> C	88	SNP (transversion)	100.0%							4.0E-291			
1,178,554	1,178,554	1	G -> A	88	SNP (transition)	100.0%							4.0E-291			
1,178,559	1,178,560	2	GC -> T	88 -> 89	Deletion	100.0%							1.0E-132			
1,178,576	1,178,576	1	G -> A	90	SNP (transition)	100.0%							1.0E-306			
1,178,584	1,178,584	1	A -> T	91	SNP (transversion)	97.8%							1.0E-299			
1,178,589	1,178,589	1	T -> G	90	SNP (transversion)	100.0%							1.0E-315			
1,178,616	1,178,617	2	TT -> AC	92	Substitution	97.8%							4.2E-285			
1,178,620	1,178,620	1	T -> C	90	SNP (transition)	98.9%							1.1E-274			
1,178,624	1,178,624	1	G -> A	91	SNP (transition)	100.0%							4.0E-310			
1,178,626	1,178,626	1	C -> T	91	SNP (transition)	100.0%							5.0E-301			
1,178,644	1,178,645	2	CC -> TG	98 -> 99	Substitution	98.0%							7.8E-319			
1,178,652	1,178,652	1	G -> T	97	SNP (transversion)	100.0%							4.0E-311			
1,178,659	1,178,659	1	C -> A	94	SNP (transversion)	98.9%							2.4E-296			
1,178,669	1,178,669	1	G -> A	93	SNP (transition)	100.0%							1.3E-307			
1,178,707	1,178,707	1	C -> T	84	SNP (transition)	100.0%							1.6E-269			
1,178,740	1,178,740	1	A -> C	90	SNP (transversion)	98.9%							1.1E-274			
1,178,744	1,178,744	1	(G)3 -> (G)2	90	Deletion (tandem repeat)	100.0%							1.0E-135			
1,178,750	1,178,750	1	C -> T	91	SNP (transition)	100.0%							6.3E-292			
1,178,753	1,178,753	1	G -> A	90	SNP (transition)	100.0%							1.0E-306			
1,178,766	1,178,766	1	T -> C	89	SNP (transition)	100.0%							2.0E-294			
1,178,769	1,178,769	1	C -> T	90	SNP (transition)	96.7%							2.3E-265			
1,178,772	1,178,772	1	A -> G	92	SNP (transition)	100.0%							4.0E-295			
1,178,780	1,178,780	1	A -> G	91	SNP (transition)	100.0%							7.9E-283			
1,178,789	1,178,790	2	CG -> AAC	88 -> 90	Insertion	96.6% -> 96.7%							1.8E-279			
1,178,814	1,178,814	1	T -> A	90	SNP (transversion)	98.9%							1.1E-274			
1,178,865	1,178,865	1	C -> T	84	SNP (transition)	97.6%		12	CTC -> CTT	C770_RS47295	hypothetical protein	None	8.7E-268	4	3	WP_015243272.1
1,178,881	1,178,881	1	T -> C	84	SNP (transition)	98.8%	C -> R	28	TGC -> CGC	C770_RS47295	hypothetical protein	Substitution	5.3E-281	10	1	WP_015243272.1
1,178,913	1,178,913	1	G -> A	81	SNP (transition)	98.8%		60	GCG -> GCA	C770_RS47295	hypothetical protein	None	8.1E-263	20	3	WP_015243272.1
1,178,946	1,178,946	1	C -> T	77	SNP (transition)	98.7%		93	GTC -> GTT	C770_RS47295	hypothetical protein	None	1.9E-234	31	3	WP_015243272.1
1,178,952	1,178,952	1	T -> C	78	SNP (transition)	100.0%		99	GAT -> GAC	C770_RS47295	hypothetical protein	None	1.6E-242	33	3	WP_015243272.1
1,178,973	1,178,973	1	C -> T	76	SNP (transition)	97.4%		120	TAC -> TAT	C770_RS47295	hypothetical protein	None	1.8E-241	40	3	WP_015243272.1
1,178,977	1,178,977	1	G -> T	74	SNP (transversion)	98.6%	A -> S	124	GCT -> TCT	C770_RS47295	hypothetical protein	Substitution	9.3E-240	42	1	WP_015243272.1
1,178,983	1,178,983	1	G -> A	73	SNP (transition)	98.6%	A -> T	130	GCC -> ACC	C770_RS47295	hypothetical protein	Substitution	4.6E-222	44	1	WP_015243272.1
1,179,012	1,179,012	1	T -> C	66	SNP (transition)	98.5%		159	GAT -> GAC	C770_RS47295	hypothetical protein	None	6.6E-194	53	3	WP_015243272.1
1,179,017	1,179,017	1	T -> C	64	SNP (transition)	96.9%	V -> A	164	GTA -> GCA	C770_RS47295	hypothetical protein	Substitution	2.0E-183	55	2	WP_015243272.1
1,179,043	1,179,043	1	A -> G	66	SNP (transition)	100.0%	R -> G	190	AGG -> GGG	C770_RS47295	hypothetical protein	Substitution	1.0E-198	64	1	WP_015243272.1
1,179,092	1,179,092	1	G -> A	65	SNP (transition)	96.9%							1.3E-211			
1,179,107	1,179,107	1	T -> A	66	SNP (transversion)	100.0%							4.0E-225			
1,179,167	1,179,166	0	G	72	Insertion	98.6%							3.6E-233			
1,179,171	1,179,170	0	TGACC	73 -> 74	Insertion	95.9%							1.8E-236			
1,179,198	1,179,198	1	A -> G	72	SNP (transition)	100.0%	I -> M	15	ATA -> ATG	C770_RS47300	hypothetical protein	Substitution	1.6E-245	5	3	WP_015243273.1
1,179,291	1,179,291	1	G -> T	71	SNP (transversion)	98.6%		108	CTG -> CTT	C770_RS47300	hypothetical protein	None	7.1E-230	36	3	WP_015243273.1
1,179,297	1,179,297	1	A -> G	72	SNP (transition)	100.0%		114	GCA -> GCG	C770_RS47300	hypothetical protein	None	2.5E-238	38	3	WP_015243273.1
1,179,299	1,179,299	1	C -> T	72	SNP (transition)	100.0%	A -> V	116	GCA -> GTA	C770_RS47300	hypothetical protein	Substitution	1.0E-252	39	2	WP_015243273.1
1,179,327	1,179,327	1	C -> T	75	SNP (transition)	100.0%		144	CGC -> CGT	C770_RS47300	hypothetical protein	None	1.0E-255	48	3	WP_015243273.1
1,179,389	1,179,389	1	G -> A	73	SNP (transition)	100.0%	G -> E	206	GGA -> GAA	C770_RS47300	hypothetical protein	Substitution	5.0E-227	69	2	WP_015243273.1
1,179,399	1,179,399	1	A -> C	69	SNP (transversion)	100.0%		216	ATA -> ATC	C770_RS47300	hypothetical protein	None	2.5E-235	72	3	WP_015243273.1
1,179,414	1,179,414	1	G -> A	70	SNP (transition)	100.0%		231	AAG -> AAA	C770_RS47300	hypothetical protein	None	1.0E-224	77	3	WP_015243273.1
1,179,438	1,179,438	1	C -> T	67	SNP (transition)	100.0%		255	AGC -> AGT	C770_RS47300	hypothetical protein	None	4.0E-215	85	3	WP_015243273.1
1,179,444	1,179,444	1	G -> A	68	SNP (transition)	98.5%		261	GCG -> GCA	C770_RS47300	hypothetical protein	None	1.1E-226	87	3	WP_015243273.1
1,179,447	1,179,447	1	T -> C	67	SNP (transition)	100.0%		264	CTT -> CTC	C770_RS47300	hypothetical protein	None	7.9E-222	88	3	WP_015243273.1
1,179,468	1,179,468	1	T -> C	70	SNP (transition)	100.0%		285	TTT -> TTC	C770_RS47300	hypothetical protein	None	1.0E-238	95	3	WP_015243273.1
1,179,501	1,179,501	1	G -> C	63	SNP (transversion)	100.0%		318	ACG -> ACC	C770_RS47300	hypothetical protein	None	1.3E-208	106	3	WP_015243273.1
1,179,504	1,179,504	1	A -> T	62	SNP (transversion)	100.0%		321	GGA -> GGT	C770_RS47300	hypothetical protein	None	1.0E-217	107	3	WP_015243273.1
1,179,515	1,179,515	1	T -> C	60	SNP (transition)	100.0%	I -> T	332	ATC -> ACC	C770_RS47300	hypothetical protein	Substitution	1.0E-198	111	2	WP_015243273.1
1,179,576	1,179,578	3	TCA -> CGC	49 -> 51	Substitution	98.0%							2.0E-162			
1,179,586	1,179,587	2	GC	46	Deletion	100.0%							2.5E-74			
1,179,591	1,179,591	1	C -> T	45	SNP (transition)	100.0%							1.0E-153			
1,179,607	1,179,608	2	GT -> CC	39	Substitution	97.4%							2.5E-128			
1,179,627	1,179,627	1	G -> C	36	SNP (transversion)	97.2%							3.6E-125			
1,179,635	1,179,635	1	A -> G	33	SNP (transition)	97.0%							5.2E-108			
1,179,654	1,179,654	1	T -> C	29	SNP (transition)	100.0%							2.5E-99			
1,179,657	1,179,658	2	GA -> AG	27	Substitution	100.0%							4.0E-87			
1,179,692	1,179,692	1	T -> C	17	SNP (transition)	100.0%		108	CAA -> CAG	C770_RS47305	membrane protein	None	2.0E-53	36	3	WP_014527390.1
1,179,713	1,179,713	1	C -> T	13	SNP (transition)	100.0%		87	CCG -> CCA	C770_RS47305	membrane protein	None	1.3E-43	29	3	WP_014527390.1
1,179,754	1,179,754	1	G -> C	10	SNP (transversion)	100.0%	L -> V	46	CTC -> GTC	C770_RS47305	membrane protein	Substitution	1.0E-32	16	1	WP_014527390.1
1,179,820	1,179,821	2	GC	34	Deletion	100.0%							2.5E-48			
1,179,869	1,179,869	1	T -> C	46	SNP (transition)	100.0%							4.0E-157			
1,179,883	1,179,883	1	C -> A	51	SNP (transversion)	100.0%							5.0E-169			
1,179,929	1,179,929	1	A	61	Deletion	100.0%							2.5E-98			
1,179,938	1,179,939	2	AC -> CG	61	Substitution	100.0%							6.3E-196			
1,179,963	1,179,963	1	(T)6 -> (T)5	65	Deletion (tandem repeat)	100.0%							3.2E-98			
1,179,970	1,179,972	3	GAC	65	Deletion	100.0%							3.2E-98			
1,180,029	1,180,029	1	C -> T	75	SNP (transition)	100.0%							1.0E-255			
1,180,043	1,180,043	1	A -> G	78	SNP (transition)	100.0%							4.0E-258			
1,180,072	1,180,072	1	G -> T	71	SNP (transversion)	100.0%							7.9E-221			
1,180,084	1,180,084	1	G -> C	70	SNP (transversion)	100.0%							1.0E-238			
1,180,089	1,180,089	1	G -> A	69	SNP (transition)	100.0%							1.3E-214			
1,180,114	1,180,114	1	A -> C	69	SNP (transversion)	98.6%							4.4E-230			
1,180,122	1,180,122	1	G -> C	71	SNP (transversion)	98.6%							7.1E-237			
1,180,156	1,180,155	0	G	67	Insertion	100.0%							1.6E-228			
1,180,248	1,180,247	0	T	78	Insertion	100.0%							4.0E-258			
1,180,250	1,180,250	1	G -> A	78	SNP (transition)	100.0%							1.0E-273			
1,180,257	1,180,257	1	G -> C	77	SNP (transversion)	100.0%							3.2E-270			
1,180,259	1,180,259	1	C -> G	75	SNP (transversion)	100.0%							1.0E-240			
1,180,285	1,180,285	1	T -> C	74	SNP (transition)	100.0%							1.6E-237			
1,180,310	1,180,310	1	G -> A	77	SNP (transition)	100.0%							2.0E-239			
1,180,337	1,180,337	1	A -> G	76	SNP (transition)	100.0%							4.0E-259			
1,180,346	1,180,346	1	C -> T	76	SNP (transition)	100.0%							4.0E-259			

1,180,368	1,180,368	1	C -> T	81	SNP (transition)	98.8%							8.1E-271			
1,180,417	1,180,417	1	A -> C	86	SNP (transversion)	100.0%							4.0E-293			
1,180,427	1,180,427	1	G -> T	87	SNP (transversion)	100.0%							3.2E-305			
1,180,450	1,180,450	1	C -> T	86	SNP (transition)	100.0%							1.0E-301			
1,180,515	1,180,515	1	T -> C	76	SNP (transition)	100.0%							4.0E-259			
1,180,561	1,180,561	1	T -> C	82	SNP (transition)	98.8%							3.3E-274			
1,180,583	1,180,583	1	A -> G	88	SNP (transition)	100.0%							6.3E-300			
1,180,585	1,180,585	1	G -> C	86	SNP (transversion)	100.0%							6.3E-276			
1,180,600	1,180,600	1	C -> A	83	SNP (transversion)	100.0%							2.5E-266			
1,180,649	1,180,649	1	T -> G	84	SNP (transversion)	98.8%		648	CCA -> CCC	C770_RS47315	hypothetical protein	None	2.1E-264	216	3	WP_015243274.1
1,180,733	1,180,733	1	A -> G	67	SNP (transition)	100.0%		564	TAT -> TAC	C770_RS47315	hypothetical protein	None	6.3E-242	188	3	WP_015243274.1
1,180,866	1,180,866	1	G -> C	66	SNP (transversion)	100.0%	T -> R	431	ACA -> AGA	C770_RS47315	hypothetical protein	Substitution	1.0E-231	144	2	WP_015243274.1
1,180,952	1,180,952	1	G -> A	55	SNP (transition)	100.0%		345	AAC -> AAT	C770_RS47315	hypothetical protein	None	3.2E-193	115	3	WP_015243274.1
1,181,017	1,181,017	1	A -> C	64	SNP (transversion)	100.0%	S -> A	280	TCG -> GCG	C770_RS47315	hypothetical protein	Substitution	2.5E-218	94	1	WP_015243274.1
1,181,057	1,181,057	1	C -> A	65	SNP (transversion)	100.0%		240	CTG -> CTT	C770_RS47315	hypothetical protein	None	3.2E-215	80	3	WP_015243274.1
1,181,069	1,181,069	1	C -> G	63	SNP (transversion)	100.0%		228	CTG -> CTC	C770_RS47315	hypothetical protein	None	3.2E-221	76	3	WP_015243274.1
1,181,072	1,181,074	2	GAC -> AAT	65 -> 66	Substitution	100.0%	V -> I	223	GTC -> ATT	C770_RS47315	hypothetical protein	Substitution	1.6E-218	75	1	WP_015243274.1
1,181,084	1,181,084	1	T -> C	64	SNP (transition)	98.4%		213	GAA -> GAG	C770_RS47315	hypothetical protein	None	1.6E-200	71	3	WP_015243274.1
1,181,149	1,181,149	1	C -> A	66	SNP (transversion)	100.0%	V -> L	148	GTG -> TTG	C770_RS47315	hypothetical protein	Substitution	1.0E-231	50	1	WP_015243274.1
1,181,259	1,181,259	1	G -> A	80	SNP (transition)	100.0%	T -> I	38	ACT -> ATT	C770_RS47315	hypothetical protein	Substitution	1.0E-264	13	2	WP_015243274.1
1,181,415	1,181,415	1	T -> C	91	SNP (transition)	100.0%							4.0E-310			
1,181,550	1,181,550	1	T -> A	73	SNP (transversion)	100.0%							6.3E-249			
1,181,572	1,181,572	1	G -> A	65	SNP (transition)	100.0%							1.0E-221			
1,181,591	1,181,591	1	C -> T	57	SNP (transition)	100.0%							7.9E-189			
1,181,674	1,181,674	1	T -> A	29	SNP (transversion)	100.0%							2.5E-99			
1,181,678	1,181,678	1	A -> G	28	SNP (transition)	100.0%							4.0E-93			
1,181,690	1,181,694	5	AACCA -> GTTGC	26	Substitution	100.0%							1.6E-86			
1,181,696	1,181,696	1	A -> C	25	SNP (transversion)	96.0%							2.5E-83			
1,181,698	1,181,700	3	GCC -> AGA	23	Substitution	100.0%							2.5E-74			
1,181,702	1,181,702	1	A -> C	23	SNP (transversion)	100.0%							1.6E-83			
1,181,704	1,181,706	3	GCC -> CAT	22 -> 23	Substitution	100.0%							2.5E-73			
1,181,712	1,181,716	5	GTATT -> AACAA	20 -> 22	Substitution	95.0% -> 95.5%							4.0E-62			
1,181,718	1,181,719	2	TG -> AT	20	Substitution	100.0%							1.0E-68			
1,181,722	1,181,723	2	CT -> GA	19 -> 20	Substitution	100.0%							3.2E-67			
1,181,725	1,181,728	4	GTTG -> AGAC	15 -> 19	Substitution	100.0%							1.0E-51			
1,181,730	1,181,731	2	GC -> AG	12 -> 14	Substitution	100.0%							1.6E-41			
1,181,733	1,181,733	1	A -> G	10	SNP (transition)	100.0%							1.0E-34			
1,181,738	1,181,738	1	C -> A	8	SNP (transversion)	100.0%							2.5E-26			
1,181,740	1,181,748	9	ATCTTCAAA -> G	6 -> 8	Substitution	100.0%							2.5E-22			
1,181,811	1,181,812	2	TG -> AA	6	Substitution	100.0%		59		C770_RS47320	hypothetical protein	Truncation	1.0E-21	20	2	WP_010975414.1
1,181,814	1,181,816	3	TTG -> ACC	6 -> 7	Substitution	100.0%	VD -> DH	62	GTT,GAT -> GAC,	C770_RS47320	hypothetical protein	Substitution	6.3E-17	21	2	WP_010975414.1
1,181,819	1,181,820	2	CC -> GG	9	Substitution	100.0%	P -> G	67	CCT -> GGT	C770_RS47320	hypothetical protein	Substitution	5.0E-25	23	1	WP_010975414.1
1,181,823	1,181,826	4	CGCG -> GAAC	9 -> 11	Substitution	100.0%	PR -> RT	71	CCG,CGA -> CGA	C770_RS47320	hypothetical protein	Substitution	1.0E-30	24	2	WP_010975414.1
1,181,828	1,181,828	1	A -> C	11	SNP (transversion)	100.0%	M -> L	76	ATG -> CTG	C770_RS47320	hypothetical protein	Substitution	1.0E-33	26	1	WP_010975414.1
1,181,831	1,181,831	1	A -> G	12	SNP (transition)	100.0%	T -> A	79	ACG -> GCG	C770_RS47320	hypothetical protein	Substitution	1.0E-36	27	1	WP_010975414.1
1,181,834	1,181,839	6	CAAAGG -> GCTT	12 -> 16	Substitution	100.0%	QR -> AS	82	CAA,AGG -> GCT	C770_RS47320	hypothetical protein	Substitution	2.5E-40	28	1	WP_010975414.1
1,181,841	1,181,842	2	CG -> TT	16	Substitution	100.0%	P -> L	89	CCG -> CTT	C770_RS47320	hypothetical protein	Substitution	1.6E-53	30	2	WP_010975414.1
1,181,851	1,181,851	1	G -> T	18	SNP (transversion)	100.0%		99	GCG -> GCT	C770_RS47320	hypothetical protein	None	1.6E-65	33	3	WP_010975414.1
1,181,904	1,181,904	1	A	28	Deletion	100.0%		152		C770_RS47320	hypothetical protein	Frame Shift	1.0E-42	51	2	WP_010975414.1
1,181,938	1,181,938	1	G -> C	39	SNP (transversion)	100.0%		186	GCG -> GCC	C770_RS47320	hypothetical protein	None	1.3E-121	62	3	WP_010975414.1
1,181,955	1,181,955	1	T -> C	44	SNP (transition)	100.0%	V -> A	203	GTC -> GCC	C770_RS47320	hypothetical protein	Substitution	4.0E-137	68	2	WP_010975414.1
1,182,017	1,182,017	1	G -> T	68	SNP (transversion)	100.0%	A -> S	40	GCT -> TCT	C770_RS47325	hypothetical protein	Substitution	1.0E-238	14	1	WP_015243275.1
1,182,046	1,182,046	1	C -> T	75	SNP (transition)	98.7%		69	GCC -> GCT	C770_RS47325	hypothetical protein	None	1.2E-235	23	3	WP_015243275.1
1,182,112	1,182,112	1	C -> T	74	SNP (transition)	100.0%		135	AAC -> AAT	C770_RS47325	hypothetical protein	None	6.3E-245	45	3	WP_015243275.1
1,182,132	1,182,132	1	G -> T	74	SNP (transversion)	100.0%	C -> F	155	TGC -> TTC	C770_RS47325	hypothetical protein	Substitution	1.6E-237	52	2	WP_015243275.1
1,182,238	1,182,238	1	G -> A	72	SNP (transition)	100.0%		261	AAG -> AAA	C770_RS47325	hypothetical protein	None	4.0E-231	87	3	WP_015243275.1
1,182,282	1,182,282	1	G -> A	74	SNP (transition)	98.6%	R -> K	305	AGG -> AAG	C770_RS47325	hypothetical protein	Substitution	4.7E-247	102	2	WP_015243275.1
1,182,309	1,182,309	1	T -> C	71	SNP (transition)	100.0%	V -> A	332	GTA -> GCA	C770_RS47325	hypothetical protein	Substitution	6.3E-228	111	2	WP_015243275.1
1,182,325	1,182,325	1	T -> C	73	SNP (transition)	100.0%		348	TGT -> TGC	C770_RS47325	hypothetical protein	None	3.2E-256	116	3	WP_015243275.1
1,182,345	1,182,345	1	C -> T	77	SNP (transition)	100.0%	T -> I	368	ACA -> ATA	C770_RS47325	hypothetical protein	Substitution	3.2E-270	123	2	WP_015243275.1
1,182,376	1,182,377	2	GA -> CG	87	Substitution	100.0%	ER -> DG	399	GAG,AGG -> GAC	C770_RS47325	hypothetical protein	Substitution	1.6E-296	133	3	WP_015243275.1
1,182,601	1,182,601	1	C -> T	108	SNP (transition)	100.0%		118	CTG -> TTG	C770_RS47330	hypothetical protein	None	0.0	40	1	WP_015243276.1
1,182,626	1,182,626	1	C -> G	100	SNP (transversion)	100.0%	P -> R	143	CCG -> CGG	C770_RS47330	hypothetical protein	Substitution	1.0E-310	48	2	WP_015243276.1
1,182,663	1,182,665	3	GGC	99	Deletion	100.0%	PA -> P	180	CCG,GCC -> CCC	C770_RS47330	hypothetical protein	Deletion	4.0E-159	60	3	WP_015243276.1
1,182,692	1,182,692	1	T -> G	100	SNP (transversion)	100.0%							1.0E-320			
1,182,726	1,182,725	0	CG	100	Insertion	98.0%							2.0E-320			
1,182,758	1,182,758	1	A -> G	102	SNP (transition)	99.0%							6.4E-322			
1,182,835	1,182,835	1	A -> C	96	SNP (transversion)	100.0%							0.0			
1,182,881	1,182,881	1	(C)3 -> (C)2	93	Deletion (tandem repeat)	100.0%		159		C770_RS47335	hypothetical protein	Frame Shift	3.2E-140	53	3	WP_010975412.1
1,182,896	1,182,896	1	C -> A	92	SNP (transversion)	100.0%	E -> D	144	GAG -> GAT	C770_RS47335	hypothetical protein	Substitution	1.0E-322	48	3	WP_010975412.1
1,183,394	1,183,393	0	(A)4 -> (A)5	74	Insertion (tandem repeat)	100.0%		399		C770_RS47340	phospholipase	Frame Shift	4.0E-230	133	3	WP_010975411.1
1,183,417	1,183,417	1	G -> A	75	SNP (transition)	98.7%		375	GAC -> GAT	C770_RS47340	phospholipase	None	4.7E-243	125	3	WP_010975411.1
1,183,621	1,183,621	1	G -> A	72	SNP (transition)	100.0%		171	CCC -> CCT	C770_RS47340	phospholipase	None	1.6E-245	57	3	WP_010975411.1
1,183,782	1,183,782	1	C -> T	77	SNP (transition)	100.0%	G -> R	10	GGA -> AGA	C770_RS47340	phospholipase	Substitution	1.6E-262	4	1	WP_010975411.1
1,183,922	1,183,922	1	C -> G	41	SNP (transversion)	100.0%							6.3E-132			
1,183,933	1,183,933	1	(T)3 -> (T)2	38	Deletion (tandem repeat)	100.0%							1.0E-57			
1,183,939	1,183,939	1	G -> C	36	SNP (transversion)	100.0%							4.0E-123			
1,183,944	1,183,946	3	TAA -> CGG	35 -> 36	Substitution	100.0%							1.6E-101			
1,183,949	1,183,949	1	G -> C	33	SNP (transversion)	100.0%							5.0E-103			
1,183,951	1,183,951	1	C -> G	33	SNP (transversion)	100.0%							1.0E-99			
1,183,953	1,183,961	9	TCTCCGCCC -> G	31 -> 32	Substitution	100.0%							4.0E-87			
1,183,963	1,183,968	6	ATTGGA -> CGGT	29 -> 30	Substitution	96.6% -> 96.7%							4.0E-76			
1,183,970	1,183,971	2	AG -> TC	25 -> 27	Substitution	100.0%							6.3E-71			
1,183,973	1,183,974	2	CT -> TG	23 -> 25	Substitution	100.0%							4.0E-65			
1,183,977	1,183,978	2	AT	21	Deletion	100.0%							5.0E-28			
1,183,980	1,183,980	1	T -> A	21	SNP (transversion)	95.2%							2.1E-59			
1,183,985	1,183,985	1	G -> C	20	SNP (transversion)	100.0%							1.0E-58			
1,183,987	1,183,987	1	T -> A	18	SNP (transversion)	100.0%							2.5E-58			
1,183,991	1,183,991	1	T -> G	18	SNP (transversion)	100.0%							6.3E-53			
1,183,993	1,183,998	6	GGCGTG -> CATA	16 -> 18	Substitution	100.0%							4.0E-47			
1,184,001	1,184,009	9	AGAAGGTTT -> T	13	Substitution	100.0%							1.6E-34			
1,184,011	1,184,013	3	CAG -> TGC	7 -> 8	Substitution	100.0%							7.9E-24			
1,184,015	1,184,015	1	A -> G	6	SNP (transition)	100.0%							2.5E-16			
1,186,619	1,186,619	1	T -> A	10	SNP (transversion)	100.0%							1.0E-19			
1,186,624	1,186,624	1	G -> A	10	SNP (transition)	100.0%							1.0E-19			
1,186,675	1,186,675	1	T -> C	13	SNP (transition)	100.0%							4.0E-37			
1,186,704	1,186,704	1	T -> A	22	SNP (transversion)	100.0%							6.3E-69			
1,186,717	1,186,717	1	T -> C	23	SNP (transition)	100.0%							5.0E-72			
1,186,727	1,186,727	1	A -> C	25	SNP (transversion)	100.0%							3.2E-83			
1,186,736	1,186,736	1	T -> G	25	SNP (transversion)	100.0%							3.2E-78			
1,186,738	1,186,738	1	A -> G	25	SNP (transition)	100.0%							3.2E-73			
1,186,744	1,186,744	1	T -> C	25	SNP (transition)	100.0%							1.0E-70			
1,186,750	1,186,750	1	A -> C	25	SNP (transversion)	100.0%							3.2E-83			
1,186,759	1,186,759	1	G -> A	25	SNP (transition)	100.0%							3.2E-88			
1,186,922	1,186,923	2	AT -> GC	14	Substitution	100.0%							2.5E-48			
1,186,925	1,186,925	1	C -> G	14	SNP (transversion)	100.0%							1.0E-49			
1,186,927	1,186,929	3	TGG -> CTC	13	Substitution	100.0%							2.5E-42			
1,186,931	1,186,941	11	ATATAGGGGAC -	10 -> 13	Substitution	100.0%							1.0E-34			
1,186,944	1,186,946	3	ATG -> CGA	9 -> 10	Substitution	100.0%							3.2E-32			
1,186,949	1,186,953	5	TTCGA -> GCGCT	8	Substitution	100.0%							4.0E-27			
1,186,955	1,186,959	5	ACAAG -> CGCGA	8	Substitution	100.0%							2.5E-26			
1,186,961	1,186,962	2	GC -> AA	7 -> 8	Substitution	100.0%							3.2E-25			
1,186,964	1,186,965	2	CA -> TC	7	Substitution	100.0%							1.6E-24			
1,187,048	1,187,048	1	G -> C	6	SNP (transversion)	100.0%							1.0E-21			
1,187,050	1,187,053	4	CGCA -> ATGG	6 -> 7	Substitution	100.0%							1.6E-20			
1,187,057	1,187,057	1	G -> T	8	SNP (transversion)	100.0%							4.0E-27			
1,187,059	1,187,059	1	G -> C	8	SNP (transversion)	100.0%							1.0E-28			
1,187,063	1,187,063	1	-C	8	Deletion	100.0%							1.0E-12			
1,187,066	1,187,066	1	T -> A	8	SNP (transversion)	100.0%							2.5E-26			
1,187,069	1,187,069	1	C -> T	8	SNP (transition)	100.0%							4.0E-27			
1,187,072	1,187,074	3	AGA -> CAG	8	Substitution	100.0%							2.5E-26			
1,187,085	1,187,085	1	G -> A	9	SNP (transition)	100.0%							1.6E-29			
1,187,101	1,187,101	1	T -> C	15	SNP (transition)	100.0%							1.0E-45			
1,187,115	1,187,114	0	(A)2 -> (A)3	20	Insertion (tandem repeat)	100.0%							1.0E-62			
1,187,116	1,187,116	1	C -> T	20	SNP (transition)	100.0%							1.0E-66			
1,187,144	1,187,144	1	T -> C	34	SNP (transition)	100.0%							6.3E-113			
1,187,153	1,187,153	1	A -> T	35	SNP (transversion)	100.0%							3.2E-116			
1,187,171	1,187,171	1	A -> G	43	SNP (transition)	100.0%							6.3E-147			
1,187,178	1,187,178	1	C -> T	48	SNP (transition)	100.0%							1.6E-149			
1,187,181	1,187,180	0	A	50	Insertion	100.0%							1.0E-160			
1,187,193	1,187,193	1	T -> C	58	SNP (transition)	100.0%							6.3E-198			
1,187,204	1,187,205	2	GT -> CC	60 -> 61	Substitution	100.0%							1.0E-192			
1,187,212	1,187,213	2	AA -> GT	62	Substitution	98.4%							3.1E-200			
1,187,217	1,187,217	1	G -> C	62	SNP (transversion)	100.0%							2.5E-205			
1,187,236	1,187,236	1	C -> G	68	SNP (transversion)	98.5%							5.4E-220			
1,187,252	1,187,252	1	C -> A	69	SNP (transversion)	100.0%							2.5E-235			
1,187,256	1,187,256	1	T -> G	70	SNP (transversion)	100.0%							1.0E-224			
1,187,264	1,187,265	2	GC -> AT	75	Substitution	100.0%							1.0E-255			
1,187,273	1,187,273	1	G -> C	76	SNP (transversion)	100.0%							6.3E-244			
1,187,296	1,187,296	1	T -> A	77	SNP (transversion)	96.1%							4.6E-240			
1,187,313	1,187,313	1	T -> C	80	SNP (transition)	100.0%							1.0E-272			
1,187,332	1,187,332	1	A -> G	85	SNP (transition)	100.0%							3.2E-281			
1,187,338	1,187,338	1	C -> T	84	SNP (transition)	98.8%							2.1E-264			
1,187,343	1,187,343	1	G -> A	86	SNP (transition)	98.8%							8.6E-254			
1,187,446	1,187,446	1	G -> A	92	SNP (transition)	100.0%		48	AAG -> AAA	C770_RS47360	DUF982 domain-contair	None	4.0E-295	16	3	WP_015243280.1
1,187,449	1,187,449	1	T -> C	89	SNP (transition)	100.0%		51	GAT -> GAC	C770_RS47360	DUF982 domain-contair	None	2.0E-294	17	3	WP_015243280.1
1,187,456	1,187,456	1	C -> A	89	SNP (transversion)	100.0%		58	CGA -> AGA	C770_RS47360	DUF982 domain-contair	None	2.5E-303	20	1	WP_015243280.1
1,187,494	1,187,494	1	T -> C	84	SNP (transition)	100.0%		96	CTT -> CTC	C770_RS47360	DUF982 domain-contair	None	2.5E-286	32	3	WP_015243280.1
1,187,499	1,187,499	1	G -> A	84	SNP (transition)	100.0%	G -> E	101	GGG -> GAG	C770_RS47360	DUF982 domain-contair	Substitution	6.3E-278	34	2	WP_015243280.1
1,187,548	1,187,548	1	C -> T	78	SNP (transition)	97.4%		150	ACC -> ACT	C770_RS47360	DUF982 domain-contair	None	1.2E-255	50	3	WP_015243280.1
1,187,605	1,187,605	1	A -> G	88	SNP (transition)	98.9%		207	CGA -> CGG	C770_RS47360	DUF982 domain-contair	None	2.8E-303	69	3	WP_015243280.1
1,187,632	1,187,632	1	G -> T	92	SNP (transversion)	98.9%		234	CTG -> CTT	C770_RS47360	DUF982 domain-contair	None	4.6E-299	78	3	WP_015243280.1
1,187,652	1,187,652	1	T -> G	96	SNP (transversion)	100.0%	L -> R	254	CTC -> CGC	C770_RS47360	DUF982 domain-contair	Substitution	1.6E-317	85	2	WP_015243280.1
1,187,702	1,187,703	2	GC -> CG	85	Substitution	97.6%							5.4E-276			
1,187,711	1,187,711	1	C -> T	82	SNP (transition)	98.8%							8.2E-242			
1,187,716	1,187,716	1	G -> A	79	SNP (transition)	98.7%							7.9E-272			
1,187,736	1,187,737	2	CC -> TT	81	Substitution	100.0%							5.0E-268			
1,187,753	1,187,753	1	C -> A	84	SNP (transversion)	100.0%							1.0E-294			
1,187,757	1,187,757	1	G -> A	84	SNP (transition)	100.0%							2.5E-286			
1,187,761	1,187,761	1	A -> T	83	SNP (transversion)	100.0%							3.2E-291			

1,187,763	1,187,763	1	G -> C		84	SNP (transversion)	100.0%							1.0E-294			
1,187,768	1,187,768	1	G -> A		85	SNP (transition)	100.0%							1.0E-289			
1,187,771	1,187,771	1	G -> A		83	SNP (transition)	100.0%							1.3E-274			
1,187,776	1,187,776	1	C -> T		84	SNP (transition)	100.0%							1.0E-294			
1,187,789	1,187,789	1	G -> A		86	SNP (transition)	100.0%							1.6E-284			
1,187,796	1,187,796	1	T -> C		91	SNP (transition)	100.0%							5.0E-301			
1,187,799	1,187,799	1	T -> G		92	SNP (transversion)	98.9%							5.8E-290			
1,187,802	1,187,802	1	T -> C		92	SNP (transition)	100.0%							2.5E-304			
1,187,806	1,187,806	1	G -> T		93	SNP (transversion)	100.0%							1.3E-307			
1,187,815	1,187,815	1	G -> A		94	SNP (transition)	97.9%							1.1E-300			
1,187,835	1,187,835	1	G -> C		95	SNP (transversion)	100.0%							3.2E-314			
1,187,849	1,187,849	1	A -> G		91	SNP (transition)	100.0%							0.0			
1,187,851	1,187,851	1	T -> C		92	SNP (transition)	100.0%							1.0E-322			
1,187,861	1,187,861	1	G -> A		91	SNP (transition)	100.0%							6.3E-292			
1,187,866	1,187,866	1	A -> G		91	SNP (transition)	100.0%							5.0E-301			
1,187,869	1,187,869	1	T -> C		91	SNP (transition)	100.0%							3.2E-319			
1,187,871	1,187,871	1	(T)4 -> (T)3		91	Deletion (tandem repeat)	100.0%							2.0E-155			
1,187,882	1,187,882	1	C -> T		86	SNP (transition)	100.0%	T -> I	8	ACC -> ATC	C770_RS47365	hypothetical protein	Substitution	4.0E-293	3	2	WP_010975409.1
1,187,885	1,187,885	1	T -> C		84	SNP (transition)	100.0%	V -> A	11	GTT -> GCT	C770_RS47365	hypothetical protein	Substitution	1.0E-294	4	2	WP_010975409.1
1,187,893	1,187,893	1	G -> A		82	SNP (transition)	100.0%	E -> K	19	GAG -> AAG	C770_RS47365	hypothetical protein	Substitution	2.5E-271	7	1	WP_010975409.1
1,187,905	1,187,907	2	CTC -> ATT	70 -> 73		Substitution	100.0%	L -> I	31	CTC -> ATT	C770_RS47365	hypothetical protein	Substitution	1.0E-245	11	1	WP_010975409.1
1,187,922	1,187,922	1	G -> C		64	SNP (transversion)	98.4%		48	CTG -> CTC	C770_RS47365	hypothetical protein	None	4.0E-213	16	3	WP_010975409.1
1,187,925	1,187,925	1	T -> C		63	SNP (transition)	100.0%		51	GAT -> GAC	C770_RS47365	hypothetical protein	None	3.2E-221	17	3	WP_010975409.1
1,187,946	1,187,946	1	T -> C		60	SNP (transition)	100.0%		72	CAT -> CAC	C770_RS47365	hypothetical protein	None	1.0E-204	24	3	WP_010975409.1
1,187,952	1,187,952	1	C -> T		60	SNP (transition)	100.0%		78	GCC -> GCT	C770_RS47365	hypothetical protein	None	1.0E-210	26	3	WP_010975409.1
1,187,954	1,187,954	1	A -> G		60	SNP (transition)	100.0%	Y -> C	80	TAC -> TGC	C770_RS47365	hypothetical protein	Substitution	1.0E-216	27	2	WP_010975409.1
1,187,963	1,187,963	1	G -> A		59	SNP (transition)	100.0%	R -> H	89	CGC -> CAC	C770_RS47365	hypothetical protein	Substitution	4.0E-213	30	2	WP_010975409.1
1,187,967	1,187,967	1	G -> A		57	SNP (transition)	100.0%		93	TTG -> TTA	C770_RS47365	hypothetical protein	None	6.3E-206	31	3	WP_010975409.1
1,187,985	1,187,985	1	C -> G		52	SNP (transversion)	100.0%		111	CCC -> CCG	C770_RS47365	hypothetical protein	None	1.0E-182	37	3	WP_010975409.1
1,187,987	1,187,986	0	G		52	Insertion	100.0%		113		C770_RS47365	hypothetical protein	Frame Shift	1.0E-182	38	2	WP_010975409.1
1,188,000	1,188,000	1	T -> C		51	SNP (transition)	100.0%		126	CGT -> CGC	C770_RS47365	hypothetical protein	None	4.0E-174	42	3	WP_010975409.1
1,188,010	1,188,010	1	A -> G		47	SNP (transition)	100.0%	T -> A	136	ACG -> GCG	C770_RS47365	hypothetical protein	Substitution	6.3E-170	46	1	WP_010975409.1
1,188,015	1,188,015	1	C -> G		43	SNP (transversion)	100.0%		141	GCC -> GCG	C770_RS47365	hypothetical protein	None	3.2E-151	47	3	WP_010975409.1
1,188,037	1,188,037	1	A -> G		39	SNP (transition)	100.0%	M -> V	163	ATG -> GTG	C770_RS47365	hypothetical protein	Substitution	4.0E-141	55	1	WP_010975409.1
1,188,047	1,188,047	1	T -> C		37	SNP (transition)	100.0%	I -> T	173	ATA -> ACA	C770_RS47365	hypothetical protein	Substitution	3.2E-130	58	2	WP_010975409.1
1,188,049	1,188,049	1	T -> C		37	SNP (transition)	100.0%	C -> R	175	TGT -> CGT	C770_RS47365	hypothetical protein	Substitution	6.3E-134	59	1	WP_010975409.1
1,188,057	1,188,057	1	G -> T		36	SNP (transversion)	100.0%		183	GGG -> GGT	C770_RS47365	hypothetical protein	None	1.0E-126	61	3	WP_010975409.1
1,188,062	1,188,062	1	G -> A		35	SNP (transition)	100.0%	R -> H	188	CGT -> CAT	C770_RS47365	hypothetical protein	Substitution	1.0E-126	63	2	WP_010975409.1
1,188,100	1,188,100	1	A -> G		17	SNP (transition)	100.0%	K -> E	226	AAG -> GAG	C770_RS47365	hypothetical protein	Substitution	2.0E-53	76	1	WP_010975409.1
1,188,103	1,188,105	2	ATT -> GTC	15 -> 16		Substitution	100.0%	I -> V	229	ATT -> GTC	C770_RS47365	hypothetical protein	Substitution	3.2E-44	77	1	WP_010975409.1
1,188,108	1,188,109	2	TT -> AA	14 -> 15		Substitution	100.0%	DL -> EM	234	GAT,TTG -> GAA,	C770_RS47365	hypothetical protein	Substitution	2.5E-41	78	3	WP_010975409.1
1,188,112	1,188,114	2	CTC -> TTG	13 -> 14		Substitution	100.0%		238	CTC -> TTG	C770_RS47365	hypothetical protein	None	7.9E-36	80	1	WP_010975409.1
1,188,126	1,188,127	2	AT -> TG		10	Substitution	100.0%							1.0E-31			
1,188,138	1,188,138	1	C -> T		8	SNP (transition)	100.0%							4.0E-27			
1,188,232	1,188,232	1	T -> G		6	SNP (transversion)	100.0%							1.6E-20			
1,188,234	1,188,234	1	G -> T		7	SNP (transversion)	100.0%							7.9E-24			
1,188,259	1,188,259	1	A -> G		11	SNP (transition)	100.0%							7.9E-35			
1,188,270	1,188,270	1	A -> G		14	SNP (transition)	100.0%							6.3E-47			
1,188,281	1,188,282	2	GC -> AA	18 -> 19		Substitution	100.0%							1.0E-54			
1,188,285	1,188,285	1	A -> G		20	SNP (transition)	100.0%							1.0E-54			
1,188,291	1,188,291	1	G -> C		20	SNP (transversion)	100.0%							1.0E-62			
1,188,309	1,188,309	1	G -> C		24	SNP (transversion)	100.0%							6.3E-68			
1,188,312	1,188,312	1	G -> C		25	SNP (transversion)	100.0%							1.0E-80			
1,188,318	1,188,318	1	A -> G		28	SNP (transition)	100.0%							2.5E-90			
1,188,321	1,188,324	4	GAAC -> AGGG		30	Substitution	100.0%							1.0E-87			
1,188,327	1,188,327	1	A -> G		33	SNP (transition)	100.0%							2.5E-106			
1,188,336	1,188,336	1	G -> A		36	SNP (transition)	100.0%							1.6E-119			
1,188,345	1,188,345	1	G -> T		40	SNP (transversion)	100.0%							1.0E-128			
1,188,348	1,188,348	1	A -> G		44	SNP (transition)	100.0%							6.3E-146			
1,188,357	1,188,357	1	G -> A		45	SNP (transition)	100.0%							3.2E-149			
1,188,359	1,188,359	1	T -> A		45	SNP (transversion)	97.8%							4.5E-131			
1,188,366	1,188,366	1	G -> C		47	SNP (transversion)	100.0%							2.0E-146			
1,188,369	1,188,369	1	G -> A		47	SNP (transition)	97.9%							1.9E-132			
1,188,372	1,188,372	1	C -> T		48	SNP (transition)	95.8%							2.8E-140			
1,188,375	1,188,375	1	G -> C		48	SNP (transversion)	100.0%							4.0E-159			
1,188,384	1,188,384	1	A -> C		51	SNP (transversion)	100.0%							6.3E-164			
1,188,437	1,188,437	1	G -> A		71	SNP (transition)	100.0%							4.0E-242			
1,188,449	1,188,449	1	T -> C		73	SNP (transition)	100.0%							3.2E-256			
1,188,455	1,188,455	1	C -> G		74	SNP (transversion)	98.6%							2.3E-254			
1,188,459	1,188,459	1	A -> T		75	SNP (transversion)	100.0%							1.0E-240			
1,188,469	1,188,469	1	G -> A		75	SNP (transition)	98.7%							1.9E-250			
1,188,476	1,188,476	1	G -> A		76	SNP (transition)	100.0%							1.0E-266			
1,188,486	1,188,486	1	C -> G		76	SNP (transversion)	100.0%							1.0E-266			
1,188,493	1,188,493	1	T -> C		77	SNP (transition)	100.0%							1.6E-262			
1,188,564	1,188,564	1	T -> A		76	SNP (transversion)	100.0%							4.0E-259			
1,188,568	1,188,568	1	T -> GAC	73 -> 74		Insertion	100.0%							6.3E-249			
1,188,593	1,188,593	1	(A)3 -> (A)2		75	Deletion (tandem repeat)	100.0%							3.2E-113			
1,188,597	1,188,597	1	T -> C		78	SNP (transition)	100.0%							1.6E-281			
1,188,608	1,188,608	1	G -> T		75	SNP (transversion)	100.0%							3.2E-263			
1,188,622	1,188,622	1	A -> C		80	SNP (transversion)	100.0%							1.0E-264			
1,188,635	1,188,635	1	T -> G		76	SNP (transversion)	100.0%							4.0E-259			
1,188,637	1,188,636	0	AGC		76	Insertion	100.0%							6.3E-244			
1,188,640	1,188,640	1	G -> A		78	SNP (transition)	100.0%							6.3E-266			
1,188,644	1,188,644	1	A -> G		77	SNP (transition)	100.0%							3.2E-270			
1,188,651	1,188,651	1	A -> G		79	SNP (transition)	100.0%							3.2E-277			
1,188,664	1,188,664	1	C -> G		77	SNP (transversion)	100.0%							7.9E-255			
1,188,673	1,188,675	3	CAA -> GGC	74 -> 75		Substitution	100.0%							1.6E-237			
1,188,716	1,188,716	1	G -> T		64	SNP (transversion)	100.0%							6.3E-212			
1,188,719	1,188,718	0	A		64	Insertion	100.0%							1.0E-224			
1,188,731	1,188,731	1	C -> T		67	SNP (transition)	97.0%							7.0E-212			
1,188,739	1,188,739	1	G -> C		67	SNP (transversion)	100.0%	A -> P	4	GCT -> CCT	C770_RS47375	hypothetical protein	Substitution	4.0E-215	2	1	WP_015243282.1
1,188,765	1,188,765	1	C -> T		74	SNP (transition)	98.6%		30	AAC -> AAT	C770_RS47375	hypothetical protein	None	1.9E-232	10	3	WP_015243282.1
1,188,856	1,188,856	1	A -> G		67	SNP (transition)	100.0%	N -> D	121	AAC -> GAC	C770_RS47375	hypothetical protein	Substitution	7.9E-222	41	1	WP_015243282.1
1,188,862	1,188,862	1	C -> G		65	SNP (transversion)	100.0%	P -> A	127	CCA -> GCA	C770_RS47375	hypothetical protein	Substitution	3.2E-215	43	1	WP_015243282.1
1,188,871	1,188,871	1	A -> G		61	SNP (transition)	100.0%	I -> V	136	ATC -> GTC	C770_RS47375	hypothetical protein	Substitution	3.2E-214	46	1	WP_015243282.1
1,188,902	1,188,902	1	A -> G		50	SNP (transition)	100.0%	Q -> R	167	CAG -> CGG	C770_RS47375	hypothetical protein	Substitution	1.0E-175	56	2	WP_015243282.1
1,188,935	1,188,935	1	(C)3 -> (C)2		44	Deletion (tandem repeat)	100.0%							1.0E-66			
1,188,940	1,188,940	1	A -> T		44	SNP (transversion)	100.0%							2.5E-150			
1,188,949	1,188,949	1	C -> G		43	SNP (transversion)	100.0%							3.2E-151			
1,188,961	1,188,961	1	C -> T		41	SNP (transition)	100.0%							5.0E-136			
1,188,973	1,188,973	1	C -> T		40	SNP (transition)	100.0%							1.0E-132			
1,188,980	1,188,980	1	C -> T		37	SNP (transition)	100.0%							2.0E-115			
1,188,982	1,188,982	1	G -> T		37	SNP (transversion)	100.0%							2.0E-115			
1,188,996	1,188,996	1	G -> A		34	SNP (transition)	100.0%							1.6E-109			
1,189,004	1,189,004	1	T -> A		33	SNP (transversion)	97.0%							3.3E-111			
1,189,018	1,189,021	4	ATCT -> TAGC	25 -> 26		Substitution	100.0%							1.0E-75			
1,189,024	1,189,024	1	G -> T		20	SNP (transversion)	100.0%							1.0E-66			
1,189,030	1,189,030	1	G -> C		18	SNP (transversion)	100.0%							1.6E-56			
1,189,032	1,189,032	1	A -> C		18	SNP (transversion)	100.0%							6.3E-53			
1,189,034	1,189,038	5	GAGGC -> ACTCC	15 -> 16		Substitution	100.0%							4.0E-39			
1,189,041	1,189,043	3	TTT -> GGC	11 -> 13		Substitution	100.0%							5.0E-37			
1,189,045	1,189,047	3	TAA -> ACC		11	Substitution	100.0%							1.0E-33			
1,189,050	1,189,059	10	GCGGCCGATC ->	9 -> 10		Substitution	100.0%							6.3E-26			
1,189,061	1,189,064	4	AGCT -> CTTC	6 -> 8		Substitution	100.0%							1.0E-18			
1,189,842	1,189,847	6	TTCCAA -> GGTG	6 -> 9		Substitution	100.0%							6.3E-23			
1,189,849	1,189,849	1	A -> C		10	SNP (transversion)	100.0%							1.0E-31			
1,189,855	1,189,854	0	T		10	Insertion	100.0%							1.0E-32			
1,189,866	1,189,866	1	G -> C		14	SNP (transversion)	100.0%							4.0E-44			
1,189,885	1,189,885	1	A -> T		21	SNP (transversion)	100.0%							5.0E-70			
1,189,903	1,189,903	1	T -> C		25	SNP (transition)	100.0%							1.0E-80			
1,189,946	1,189,946	1	C -> T		40	SNP (transition)	97.5%							1.3E-135			
1,190,004	1,190,004	1	A -> G		61	SNP (transition)	100.0%							2.5E-220			
1,190,023	1,190,023	1	A -> C		65	SNP (transversion)	100.0%							3.2E-228			
1,190,029	1,190,029	1	A -> T		69	SNP (transversion)	98.6%							2.7E-223			
1,190,048	1,190,048	1	T -> A		74	SNP (transversion)	100.0%							2.5E-252			
1,190,093	1,190,093	1	C -> G		85	SNP (transversion)	100.0%							1.0E-289			
1,190,101	1,190,101	1	C -> T		87	SNP (transition)	98.9%							3.5E-291			
1,190,120	1,190,120	1	C -> G		85	SNP (transversion)	100.0%							1.0E-289			
1,190,123	1,190,123	1	T -> C		85	SNP (transition)	100.0%							1.0E-272			
1,190,157	1,190,157	1	-C		85	Deletion	100.0%							3.2E-128			
1,190,180	1,190,181	2	CT -> GG	84 -> 85		Substitution	100.0%							1.6E-269			
1,190,190	1,190,190	1	C -> G		86	SNP (transversion)	100.0%							6.3E-276			
1,190,193	1,190,193	1	A -> C		87	SNP (transversion)	100.0%							3.2E-305			
1,190,220	1,190,220	1	G -> A		90	SNP (transition)	98.9%							2.3E-301			
1,190,223	1,190,224	2	TC -> GT	88 -> 89		Substitution	98.9%							3.5E-277			
1,190,230	1,190,230	1	C -> T		85	SNP (transition)	100.0%							1.0E-289			
1,190,239	1,190,239	1	G -> A		89	SNP (transition)	100.0%							1.6E-285			
1,190,242	1,190,242	1	G -> T		90	SNP (transversion)	98.9%							1.4E-283			
1,190,250	1,190,250	1	T -> C		85	SNP (transition)	100.0%							3.2E-281			
1,190,257	1,190,257	1	C -> T		83	SNP (transition)	98.8%							8.3E-286			
1,190,262	1,190,262	1	C -> T		80	SNP (transition)	100.0%							1.0E-264			
1,190,275	1,190,275	1	A -> G		80	SNP (transition)	100.0%							1.0E-264			
1,190,286	1,190,286	1	A -> C		77	SNP (transversion)	100.0%							7.9E-255			
1,190,292	1,190,292	1	G -> C		81	SNP (transversion)	100.0%							5.0E-268			
1,190,295	1,190,295	1	A -> C		82	SNP (transversion)	100.0%							1.6E-279			
1,190,316	1,190,316	1	C -> A		83	SNP (transversion)	97.6%							1.7E-264			
1,190,324	1,190,325	2	GA -> TG	82 -> 83		Substitution	98.8%							2.1E-290			
1,190,331	1,190,331	1	T -> C		84	SNP (transition)	100.0%							4.0E-303			
1,190,343	1,190,343	1	C -> G		86	SNP (transversion)	100.0%							4.0E-293			
1,190,346	1,190,346	1	G -> A		87	SNP (transition)	100.0%							7.9E-288			
1,190,351	1,190,351	1	G -> A		88	SNP (transition)	100.0%							1.0E-308			
1,190,357	1,190,358	2	CC -> GA		85	Substitution	100.0%							3.2E-281			
1,190,360	1,190,360	1	A -> G		85	SNP (transition)	100.0%							3.2E-281			
1,190,369	1,190,369	1	T -> C		84	SNP (transition)	98.8%							5.3E-281			
1,190,373	1,190,373	1	C -> T		83	SNP (transition)	100.0%							6.3E-283			
1,190,376	1,190,376	1	G -> A		83	SNP (transition)	98.8%							8.3E-286			
1,190,379	1,190,379	1	A -> G		83	SNP (transition)	97.6%							1.4E-272			
1,190,386	1,190,386	1	G -> T		87	SNP (transversion)	100.0%							1.6E-296			
1,190,408	1,190,408	1	T -> C		83	SNP (transition)	100.0%							3.2E-291			
1,190,427	1,190,427	1	T -> A		84	SNP (transversion)	100.0%							1.0E-294			

1,190,433	1,190,433	1	G -> A		81	SNP (transition)	98.8%								8.1E-271			
1,190,436	1,190,436	1	A -> C		80	SNP (transversion)	100.0%								1.0E-280			
1,190,451	1,190,451	1	G -> A		80	SNP (transition)	98.8%								2.0E-267			
1,190,453	1,190,453	1	C -> A		82	SNP (transversion)	100.0%								6.3E-296			
1,190,460	1,190,460	1	G -> C		80	SNP (transversion)	100.0%								1.0E-280			
1,190,463	1,190,463	1	A		79	Deletion	100.0%								4.0E-127			
1,190,465	1,190,465	1	C -> A		79	SNP (transversion)	98.7%								7.9E-272			
1,190,478	1,190,479	2	CA -> TG		77	Substitution	100.0%								1.6E-262			
1,190,488	1,190,488	1	G -> A		77	SNP (transition)	100.0%								6.3E-278			
1,190,497	1,190,497	1	A -> G		79	SNP (transition)	98.7%								7.9E-272			
1,190,503	1,190,503	1	C -> G		80	SNP (transversion)	100.0%								1.0E-272			
1,190,509	1,190,509	1	G -> A		80	SNP (transition)	100.0%								1.0E-256			
1,190,515	1,190,515	1	G -> A		82	SNP (transition)	100.0%								6.3E-255			
1,190,518	1,190,518	1	G -> C		81	SNP (transversion)	100.0%								5.0E-268			
1,190,525	1,190,525	1	A -> G		79	SNP (transition)	100.0%								2.0E-261			
1,190,529	1,190,529	1	G -> T		79	SNP (transversion)	100.0%								3.2E-277			
1,190,545	1,190,545	1	A -> G		81	SNP (transition)	100.0%								5.0E-268			
1,190,550	1,190,550	1	G -> A		83	SNP (transition)	100.0%								2.5E-266			
1,190,559	1,190,561	3	AAC -> GGT	82 -> 84		Substitution	96.3% -> 96.4%								1.8E-256			
1,190,570	1,190,570	1	C -> G		80	SNP (transversion)	98.8%								1.3E-251			
1,190,584	1,190,584	1	G -> A		79	SNP (transition)	100.0%								1.3E-245			
1,190,591	1,190,591	1	A -> C		82	SNP (transversion)	98.8%								2.6E-282			
1,190,593	1,190,593	1	C -> A		81	SNP (transversion)	100.0%								4.0E-276			
1,190,596	1,190,596	1	T -> C		82	SNP (transition)	100.0%								1.0E-287			
1,190,602	1,190,602	1	T -> G		83	SNP (transversion)	98.8%								1.3E-277			
1,190,644	1,190,644	1	G -> C		74	SNP (transversion)	100.0%								1.0E-259			
1,190,647	1,190,647	1	C -> G		72	SNP (transversion)	100.0%								1.6E-245			
1,190,676	1,190,676	1	G -> C		75	SNP (transversion)	100.0%								1.0E-255			
1,190,714	1,190,714	1	T -> C		78	SNP (transition)	100.0%								6.3E-266			
1,190,719	1,190,719	1	C -> A		79	SNP (transversion)	98.7%								3.1E-256			
1,190,723	1,190,724	2	GG		80	Deletion	100.0%								1.0E-128			
1,190,737	1,190,737	1	A -> G		76	SNP (transition)	100.0%								1.6E-251			
1,190,740	1,190,740	1	G -> A		76	SNP (transition)	96.1%								4.4E-244			
1,190,742	1,190,742	1	-C		75	Deletion	100.0%								1.0E-120			
1,190,749	1,190,755	7	TGATAGC		78	Deletion	100.0%								1.6E-125			
1,190,761	1,190,762	2	CA -> GG	78 -> 80		Substitution	98.7% -> 98.8%								1.2E-260			
1,190,777	1,190,777	1	A -> C		76	SNP (transversion)	100.0%								1.6E-251			
1,190,779	1,190,779	1	A -> G		77	SNP (transition)	100.0%								3.2E-270			
1,190,811	1,190,811	1	A -> T		80	SNP (transversion)	98.8%		F -> I	208	TTT -> ATT	C770_RS47390	GcrA cell cycle regulator	Substitution	2.5E-275	70	1	WP_015243285.1
1,190,838	1,190,838	1	C -> G		81	SNP (transversion)	100.0%		E -> Q	181	GAA -> CAA	C770_RS47390	GcrA cell cycle regulator	Substitution	5.0E-268	61	1	WP_015243285.1
1,190,843	1,190,843	1	T -> C		80	SNP (transition)	100.0%		H -> R	176	CAC -> CGC	C770_RS47390	GcrA cell cycle regulator	Substitution	1.0E-256	59	2	WP_015243285.1
1,190,854	1,190,854	1	C -> T		82	SNP (transition)	100.0%			165	AAG -> AAA	C770_RS47390	GcrA cell cycle regulator	None	2.5E-271	55	3	WP_015243285.1
1,190,862	1,190,863	2	GG -> CA	82 -> 83		Substitution	100.0%		TP -> TA	156	ACC,CCC -> ACT,(	C770_RS47390	GcrA cell cycle regulator	Substitution	1.6E-279	52	3	WP_015243285.1
1,190,869	1,190,869	1	T -> G		81	SNP (transversion)	100.0%			150	GCA -> GCC	C770_RS47390	GcrA cell cycle regulator	None	6.3E-260	50	3	WP_015243285.1
1,190,881	1,190,881	1	T -> C		75	SNP (transition)	100.0%			138	CTA -> CTG	C770_RS47390	GcrA cell cycle regulator	None	1.0E-255	46	3	WP_015243285.1
1,190,914	1,190,914	1	A -> G		74	SNP (transition)	100.0%			105	TGT -> TGC	C770_RS47390	GcrA cell cycle regulator	None	6.3E-245	35	3	WP_015243285.1
1,190,937	1,190,937	1	A -> C		76	SNP (transversion)	100.0%		L -> V	82	TTA -> GTA	C770_RS47390	GcrA cell cycle regulator	Substitution	4.0E-259	28	1	WP_015243285.1
1,190,944	1,190,944	1	T -> C		73	SNP (transition)	100.0%			75	AAA -> AAG	C770_RS47390	GcrA cell cycle regulator	None	1.3E-241	25	3	WP_015243285.1
1,190,995	1,190,995	1	G -> A		74	SNP (transition)	100.0%			24	ACC -> ACT	C770_RS47390	GcrA cell cycle regulator	None	2.5E-252	8	3	WP_015243285.1
1,191,027	1,191,028	2	CG -> TA		75	Substitution	98.7%								4.7E-243			
1,191,048	1,191,049	2	(CT)4 -> (CT)3		73	Deletion (tandem repeat)	100.0%								1.6E-117			
1,191,129	1,191,129	1	C -> T		72	SNP (transition)	98.6%								2.9E-240			
1,191,133	1,191,133	1	A -> G		72	SNP (transition)	98.6%								2.3E-247			
1,191,144	1,191,144	1	C -> A		76	SNP (transversion)	100.0%								4.0E-259			
1,191,160	1,191,160	1	C -> A		75	SNP (transversion)	97.3%								3.5E-238			
1,191,196	1,191,196	1	T -> G		76	SNP (transversion)	100.0%								6.3E-244			
1,191,205	1,191,205	1	G -> C		77	SNP (transversion)	100.0%								7.9E-255			
1,191,210	1,191,210	1	A -> T		77	SNP (transversion)	98.7%								1.2E-249			
1,191,261	1,191,261	1	T -> C		64	SNP (transition)	100.0%								2.5E-218			
1,191,263	1,191,263	1	T -> C		65	SNP (transition)	100.0%								3.2E-215			
1,191,288	1,191,288	1	T -> GG		70	Insertion	97.1%								6.1E-215			
1,191,292	1,191,292	1	C -> T		70	SNP (transition)	98.6%								1.8E-233			
1,191,342	1,191,342	1	A -> G		56	SNP (transition)	100.0%								2.5E-174			
1,191,348	1,191,348	1	G -> T		54	SNP (transversion)	98.1%								2.7E-163			
1,191,373	1,191,373	1	T -> C		50	SNP (transition)	100.0%								1.0E-170			
1,191,401	1,191,401	1	T -> G		44	SNP (transversion)	100.0%								6.3E-146			
1,191,442	1,191,442	1	A -> G		30	SNP (transition)	100.0%								1.0E-102			
1,191,611	1,191,611	1	C -> T		24	SNP (transition)	95.8%								3.0E-75			
1,191,615	1,191,615	1	C -> AAA		26	Insertion	96.2%								2.6E-69			
1,191,657	1,191,657	1	(C)4 -> (C)3		41	Deletion (tandem repeat)	97.6%								4.0E-59			
1,191,660	1,191,660	1	A -> G		42	SNP (transition)	97.6%								2.1E-134			
1,191,674	1,191,674	1	G -> C		45	SNP (transversion)	97.8%								7.1E-140			
1,191,676	1,191,676	1	T -> G		46	SNP (transversion)	97.8%								4.6E-143			
1,191,678	1,191,678	1	A -> G		47	SNP (transition)	97.9%								3.0E-146			
1,191,699	1,191,700	2	GC -> AA	58 -> 59		Substitution	96.6%								6.6E-188			
1,191,708	1,191,708	1	C -> G		62	SNP (transversion)	96.8%								1.9E-201			
1,191,873	1,191,873	1	T -> C		82	SNP (transition)	98.8%			37	TTG -> CTG	C770_RS47400	hypothetical protein	None	2.6E-282	13	1	WP_015243286.1
1,192,006	1,192,006	1	T -> G		46	SNP (transversion)	100.0%		V -> G	170	GTG -> GGG	C770_RS47400	hypothetical protein	Substitution	1.6E-152	57	2	WP_015243286.1
1,192,071	1,192,071	1	A -> G		34	SNP (transition)	100.0%		T -> A	235	ACG -> GCG	C770_RS47400	hypothetical protein	Substitution	1.0E-119	79	1	WP_015243286.1
1,192,108	1,192,108	1	T -> C		23	SNP (transition)	100.0%		L -> P	272	CTT -> CCT	C770_RS47400	hypothetical protein	Substitution	1.3E-76	91	2	WP_015243286.1
1,192,126	1,192,126	1	T -> G		18	SNP (transversion)	100.0%								1.0E-63			
1,192,152	1,192,152	1	T -> A		17	SNP (transversion)	100.0%								2.5E-48			
1,192,247	1,192,247	1	T -> C		31	SNP (transition)	100.0%								5.0E-103			
1,192,437	1,192,437	1	T -> C		77	SNP (transition)	100.0%		F -> L	172	TTC -> CTC	C770_RS47405	hypothetical protein	Substitution	1.6E-262	58	1	WP_041169899.1
1,192,500	1,192,500	1	G -> A		79	SNP (transition)	97.5%		V -> I	235	GTT -> ATT	C770_RS47405	hypothetical protein	Substitution	2.4E-251	79	1	WP_041169899.1
1,192,518	1,192,518	1	C -> G		85	SNP (transversion)	100.0%								1.0E-289			
1,192,530	1,192,530	1	C -> A		89	SNP (transversion)	100.0%								2.5E-303			
1,192,533	1,192,532	0	CT		90	Insertion	95.6%								1.0E-286			
1,192,554	1,192,554	1	A -> G		89	SNP (transition)	100.0%								1.6E-285			
1,192,564	1,192,564	1	G -> A		89	SNP (transition)	100.0%								1.6E-285			
1,192,585	1,192,585	1	G		84	Deletion	100.0%								1.0E-126			
1,192,599	1,192,599	1	T		84	Deletion	100.0%								1.0E-126			
1,192,604	1,192,604	1	C -> G		85	SNP (transversion)	100.0%								3.2E-281			
1,192,644	1,192,644	1	G -> A		92	SNP (transition)	100.0%								4.0E-295			
1,192,648	1,192,649	2	AA -> C	89 -> 90		Deletion	100.0%								4.0E-143			
1,192,684	1,192,684	1	C -> G		92	SNP (transversion)	100.0%								2.5E-304			
1,192,709	1,192,710	2	TA -> CG	90 -> 91		Substitution	100.0%								4.0E-310			
1,192,745	1,192,745	1	C -> G		92	SNP (transversion)	100.0%								0.0			
1,192,752	1,192,752	1	G		91	Deletion	100.0%								2.0E-155			
1,192,758	1,192,758	1	A -> C		91	SNP (transversion)	100.0%								0.0			
1,192,767	1,192,767	1	A -> T		89	SNP (transversion)	100.0%								3.2E-312			
1,192,772	1,192,772	1	C -> G		89	SNP (transversion)	100.0%								3.2E-312			
1,192,863	1,192,863	1	T -> C		91	SNP (transition)	100.0%								7.9E-283			
1,192,953	1,192,953	1	C -> G		85	SNP (transversion)	100.0%								1.0E-289			
1,192,965	1,192,965	1	(A)3 -> (A)2		83	Deletion (tandem repeat)	100.0%								3.2E-125			
1,192,989	1,192,989	1	G -> C		89	SNP (transversion)	98.9%		E -> Q	13	GAA -> CAA	C770_RS47410	hypothetical protein	Substitution	3.5E-289	5	1	WP_010975404.1
1,193,005	1,193,005	1	C -> G		97	SNP (transversion)	100.0%		A -> G	29	GCC -> GGC	C770_RS47410	hypothetical protein	Substitution	4.0E-311	10	2	WP_010975404.1
1,193,188	1,193,188	1	A -> G		90	SNP (transition)	100.0%		D -> G	212	GAC -> GGC	C770_RS47410	hypothetical protein	Substitution	1.0E-297	71	2	WP_010975404.1
1,193,226	1,193,226	1	T -> G		90	SNP (transversion)	100.0%								1.0E-297			
1,193,247	1,193,247	1	G -> A		91	SNP (transition)	98.9%								9.1E-305			
1,193,275	1,193,275	1	C -> G		83	SNP (transversion)	100.0%								1.3E-274			
1,193,282	1,193,282	1	G -> T		80	SNP (transversion)	100.0%								1.0E-272			
1,193,353	1,193,352	0	(C)2 -> (C)3		77	Insertion (tandem repeat)	100.0%								7.9E-255			
1,193,421	1,193,421	1	A -> T		57	SNP (transversion)	100.0%								1.6E-194			
1,193,492	1,193,492	1	C -> T		37	SNP (transition)	100.0%		G -> S	340	GGC -> AGC	C770_RS47415	putative addiction modu	Substitution	1.0E-111	114	1	WP_015243288.1
1,193,502	1,193,502	1	T -> C		33	SNP (transition)	100.0%			330	CGA -> CGG	C770_RS47415	putative addiction modu	None	1.0E-99	110	3	WP_015243288.1
1,193,548	1,193,548	1	T -> C		19	SNP (transition)	100.0%		E -> G	284	GAG -> GGG	C770_RS47415	putative addiction modu	Substitution	1.6E-61	95	2	WP_015243288.1
1,193,582	1,193,582	1	T -> C		12	SNP (transition)	100.0%		N -> D	250	AAC -> GAC	C770_RS47415	putative addiction modu	Substitution	1.0E-42	84	1	WP_015243288.1
1,193,661	1,193,661	1	T -> G		24	SNP (transversion)	100.0%			171	GGA -> GGC	C770_RS47415	putative addiction modu	None	1.0E-84	57	3	WP_015243288.1
1,193,712	1,193,712	1	T -> C		43	SNP (transition)	100.0%			120	CTA -> CTG	C770_RS47415	putative addiction modu	None	1.3E-142	40	3	WP_015243288.1
1,193,757	1,193,757	1	T -> G		55	SNP (transversion)	100.0%		E -> D	75	GAA -> GAC	C770_RS47415	putative addiction modu	Substitution	3.2E-193	25	3	WP_015243288.1
1,193,818	1,193,818	1	A -> G		55	SNP (transition)	98.2%		M -> T	14	ATG -> ACG	C770_RS47415	putative addiction modu	Substitution	5.5E-188	5	2	WP_015243288.1
1,193,828	1,193,828	1	A -> C		54	SNP (transversion)	100.0%		S -> A	4	TCT -> GCT	C770_RS47415	putative addiction modu	Substitution	2.5E-184	2	1	WP_015243288.1
1,193,880	1,193,880	1	A -> G		44	SNP (transition)	100.0%								1.6E-141			
1,193,894	1,193,894	1	G -> C		41	SNP (transversion)	100.0%								6.3E-132			
1,193,933	1,193,933	1	G -> A		28	SNP (transition)	100.0%								4.0E-93			
1,193,970	1,193,969	0	T		17	Insertion	100.0%								1.6E-58			
1,194,007	1,194,009	3	TCG -> CGC	10 -> 11		Substitution	100.0%								1.0E-34			
1,194,166	1,194,166	1	T -> C		55	SNP (transition)	100.0%								3.2E-182			
1,194,272	1,194,272	1	C -> G		83	SNP (transversion)	100.0%								1.3E-274			
1,194,281	1,194,282	2	CC -> TT		81	Substitution	98.8%								6.3E-260			
1,194,291	1,194,291	1	(C)3 -> (C)2		80	Deletion (tandem repeat)	100.0%								1.0E-128			
1,194,361	1,194,361	1	C -> T		79	SNP (transition)	100.0%								3.2E-277			
1,194,387	1,194,387	1	G -> C		79	SNP (transversion)	100.0%								4.0E-285			
1,194,391	1,194,391	1	G -> C		81	SNP (transversion)	100.0%								3.2E-284			
1,194,421	1,194,421	1	T -> C		85	SNP (transition)	97.6%								1.1E-287			
1,194,460	1,194,460	1	T -> C		81	SNP (transition)	100.0%								3.2E-284			
1,194,534	1,194,534	1	C -> T		82	SNP (transition)	100.0%								4.0E-263			
1,194,550	1,194,550	1	T -> C		83	SNP (transition)	100.0%								6.3E-283			
1,194,558	1,194,558	1	A -> G		82	SNP (transition)	97.6%								3.3E-277			
1,194,603	1,194,609	7	AAAGGAC		80	Deletion	100.0%								1.0E-128			
1,194,625	1,194,625	1	C -> T		83	SNP (transition)	100.0%								3.2E-291			
1,194,666	1,194,666	1	C -> T		83	SNP (transition)	100.0%								1.3E-274			
1,194,679	1,194,679	1	A -> C		80	SNP (transversion)	100.0%								1.0E-272			
1,195,065	1,195,065	1	G -> A		8	SNP (transition)	100.0%								6.3E-28			
1,195,120	1,195,121	2	TC -> CT	21 -> 22		Substitution	100.0%								3.2E-74			
1,195,209	1,195,209	1	A		45	Deletion	100.0%								1.0E-63			
1,195,406	1,195,406	1	A -> T		82	SNP (transversion)	100.0%		D -> E	1.725	GAT -> GAA	C770_RS47430	adenylate cyclase	Substitution	4.0E-263	575	3	WP_049589739.1
1,195,427	1,195,427	1	G -> A		87	SNP (transition)	98.9%			1.704	TAC -> TAT	C770_RS47430	adenylate cyclase	None	5.5E-274	568	3	WP_049589739.1
1,195,481	1,195,481	1	C -> A		86	SNP (transversion)	100.0%			1.65	GCG -> GCT	C770_RS47430	adenylate cyclase	None	6.3E-276	550	3	WP_049589739.1
1,195,595	1,195,595	1	T -> G		100	SNP (transversion)	100.0%			1.536	CGA -> CGC	C770_RS47430	adenylate cyclase	None	0.0	512	3	WP_049589739.1
1,195,713	1,195,713	1	T -> C		98	SNP (transition)	100.0%		Q -> R	1.418	CAG -> CGG	C770_RS47430	adenylate cyclase	Substitution	2.5E-314	473	2	WP_049589739.1
1,195,858	1,195,858	1	G -> A		95	SNP (transition)	100.0%			1.273	CTG -> TTG	C770_RS47430	adenylate cyclase	None	0.0	425	1	WP_049589739.1
1,195,943	1,195,943	1	C -> T		83	SNP (transition)	100.0%			1.188	TCG -> TCA	C770_RS47430	adenylate cyclase	None	6.3E-283	396	3	WP_049589739.1
1,196,114	1,196,114	1	C -> G		80	SNP (transversion)	100.0%			1.017	GCG -> GCC	C770_RS47430	adenylate cyclase	None	1.0E-264	339	3	WP_049589739.1
1,196,267	1,196,267	1	G -> C		84	SNP (transversion)	100.0%			864	CTC -> CTG	C770_RS47430	adenylate cyclase	None	2.5E-286	288	3	WP_049589739.1
1,196,270	1,196,271	2	AG -> GT	83 -> 84		Substitution	100.0%		P -> H	860	CCT -> CAC	C770_RS47430	adenylate cyclase	Substitution	2.5E-286	287	2	WP_049589739.1
1,196,300	1,196,300	1	C -> T		77	SNP (transition)	100.0%			831	GCG -> GCA	C770_RS47430	adenylate cyclase	None	3.2E-270	277	3	WP_049589739.1
1,196,468	1,196,468	1	A -> G		84	SNP (transition)	98.8%			663	ATT -> ATC	C770_RS47430	adenylate cyclase	None	2.7E-289	221	3	WP_049589739.1
1,196,528	1,196,528	1	G -> A		83	SNP (transition)	100.0%			603	TTC -> TTT	C770_RS47430	adenylate cyclase	None	1.3E-274	201	3	WP_049589739.1

1,196,537	1,196,537	1	C -> G	84	SNP (transversion)	100.0%		594	GTG -> GTC	C770_RS47430	adenylate cyclase	None	1.6E-269	198	3	WP_049589739.1
1,196,558	1,196,558	1	G -> A	91	SNP (transition)	100.0%		573	ATC -> ATT	C770_RS47430	adenylate cyclase	None	6.3E-292	191	3	WP_049589739.1
1,196,573	1,196,573	1	G -> T	90	SNP (transversion)	98.9%		558	CGC -> CGA	C770_RS47430	adenylate cyclase	None	2.3E-301	186	3	WP_049589739.1
1,196,609	1,196,609	1	T -> C	84	SNP (transition)	98.8%		522	GCA -> GCG	C770_RS47430	adenylate cyclase	None	5.3E-281	174	3	WP_049589739.1
1,196,714	1,196,714	1	A -> G	77	SNP (transition)	98.7%		417	AAT -> AAC	C770_RS47430	adenylate cyclase	None	7.7E-265	139	3	WP_049589739.1
1,196,761	1,196,761	1	T -> G	75	SNP (transversion)	98.7%	I -> L	370	ATA -> CTA	C770_RS47430	adenylate cyclase	Substitution	1.9E-250	124	1	WP_049589739.1
1,196,822	1,196,822	1	C -> G	67	SNP (transversion)	98.5%		309	GTG -> GTC	C770_RS47430	adenylate cyclase	None	6.7E-230	103	3	WP_049589739.1
1,196,861	1,196,861	1	G -> A	60	SNP (transition)	98.3%		270	AGC -> AGT	C770_RS47430	adenylate cyclase	None	1.2E-193	90	3	WP_049589739.1
1,196,873	1,196,873	1	T -> C	60	SNP (transition)	100.0%		258	GTA -> GTG	C770_RS47430	adenylate cyclase	None	1.0E-204	86	3	WP_049589739.1
1,196,897	1,196,897	1	A -> T	65	SNP (transversion)	98.5%		234	GGT -> GGA	C770_RS47430	adenylate cyclase	None	4.1E-210	78	3	WP_049589739.1
1,196,912	1,196,912	1	G -> C	66	SNP (transversion)	100.0%		219	GCC -> GCG	C770_RS47430	adenylate cyclase	None	4.0E-225	73	3	WP_049589739.1
1,196,936	1,196,936	1	G -> A	65	SNP (transition)	100.0%		195	GTC -> GTT	C770_RS47430	adenylate cyclase	None	1.0E-221	65	3	WP_049589739.1
1,196,966	1,196,966	1	C -> G	66	SNP (transversion)	100.0%		165	GGG -> GGC	C770_RS47430	adenylate cyclase	None	2.5E-238	55	3	WP_049589739.1
1,197,014	1,197,014	1	G -> A	66	SNP (transition)	100.0%		117	ATC -> ATT	C770_RS47430	adenylate cyclase	None	1.6E-218	39	3	WP_049589739.1
1,197,159	1,197,159	1	C -> T	73	SNP (transition)	100.0%							5.0E-227			
1,197,209	1,197,209	1	G -> A	73	SNP (transition)	97.3%							6.6E-253			
1,197,218	1,197,218	1	C -> T	74	SNP (transition)	98.6%							9.3E-240			
1,197,224	1,197,224	1	G -> A	71	SNP (transition)	100.0%							4.0E-242			
1,197,236	1,197,237	2	CT -> GC	73 -> 75	Substitution	98.6% -> 98.7%							6.3E-249			
1,197,243	1,197,243	1	A -> G	76	SNP (transition)	100.0%							6.3E-244			
1,197,280	1,197,281	2	TG -> CA	80 -> 81	Substitution	100.0%							7.9E-252			
1,197,340	1,197,340	1	G -> A	73	SNP (transition)	100.0%							3.2E-256			
1,197,348	1,197,348	1	G -> T	70	SNP (transversion)	100.0%							1.0E-245			
1,197,396	1,197,396	1	A -> G	75	SNP (transition)	100.0%							3.2E-263			
1,197,398	1,197,398	1	-C	74	Deletion	100.0%							4.0E-119			
1,197,421	1,197,421	1	A -> C	75	SNP (transversion)	98.7%							1.2E-235			
1,197,450	1,197,450	1	G -> A	78	SNP (transition)	100.0%	R -> K	29	AGA -> AAA	C770_RS47435	hypothetical protein	Substitution	2.5E-250	10	2	WP_014530658.1
1,197,466	1,197,466	1	T -> C	77	SNP (transition)	98.7%		45	TCT -> TCC	C770_RS47435	hypothetical protein	None	4.9E-242	15	3	WP_014530658.1
1,197,514	1,197,514	1	T -> C	83	SNP (transition)	100.0%		93	TAT -> TAC	C770_RS47435	hypothetical protein	None	3.2E-291	31	3	WP_014530658.1
1,197,574	1,197,574	1	T -> C	70	SNP (transition)	100.0%		153	TTT -> TTC	C770_RS47435	hypothetical protein	None	1.0E-231	51	3	WP_014530658.1
1,197,598	1,197,598	1	G -> A	66	SNP (transition)	100.0%		177	CTG -> CTA	C770_RS47435	hypothetical protein	None	4.0E-225	59	3	WP_014530658.1
1,197,619	1,197,619	1	G -> C	66	SNP (transversion)	98.5%		198	GCG -> GCC	C770_RS47435	hypothetical protein	None	2.1E-213	66	3	WP_014530658.1
1,197,628	1,197,628	1	C -> G	66	SNP (transversion)	98.5%	H -> Q	207	CAC -> CAG	C770_RS47435	hypothetical protein	Substitution	2.1E-200	69	3	WP_014530658.1
1,197,638	1,197,638	1	A -> G	62	SNP (transition)	100.0%							6.3E-193			
1,197,717	1,197,717	1	C -> G	34	SNP (transversion)	100.0%							2.5E-99			
1,197,733	1,197,733	1	C -> G	29	SNP (transversion)	100.0%							1.3E-90			
1,197,735	1,197,734	0	C	28	Insertion	100.0%							6.3E-96			
1,197,740	1,197,740	1	T -> C	24	SNP (transition)	100.0%							1.6E-77			
1,197,742	1,197,744	3	TGC -> CTT	23	Substitution	100.0%							1.0E-69			
1,197,778	1,197,778	1	A -> G	10	SNP (transition)	100.0%							1.0E-33			
1,197,788	1,197,789	2	CT -> TC	8	Substitution	100.0%							2.5E-26			
1,197,936	1,197,936	1	T -> C	37	SNP (transition)	100.0%							7.9E-123			
1,197,965	1,197,965	1	G -> A	46	SNP (transition)	100.0%							4.0E-157			
1,197,975	1,197,975	1	T -> A	48	SNP (transversion)	100.0%							4.0E-159			
1,198,017	1,198,017	1	A -> G	56	SNP (transition)	100.0%							1.6E-185			
1,198,031	1,198,031	1	G -> A	61	SNP (transition)	100.0%							5.0E-202			
1,198,066	1,198,066	1	T -> C	70	SNP (transition)	100.0%							1.0E-231			
1,198,113	1,198,113	1	G -> C	84	SNP (transversion)	100.0%							6.3E-278			
1,198,157	1,198,157	1	(T)4 -> (T)3	83	Deletion (tandem repeat)	100.0%							3.2E-125			
1,198,159	1,198,159	1	G -> A	83	SNP (transition)	97.6%							1.7E-264			
1,198,163	1,198,163	1	A -> C	81	SNP (transversion)	100.0%							4.0E-276			
1,198,228	1,198,227	0	AAT	78 -> 79	Insertion	96.2%							3.1E-254			
1,198,230	1,198,230	1	G -> A	79	SNP (transition)	100.0%							2.0E-261			
1,198,235	1,198,235	1	C -> G	78	SNP (transversion)	100.0%							6.3E-266			
1,198,370	1,198,370	1	C -> T	76	SNP (transition)	100.0%		1.17	GAG -> GAA	C770_RS47440	sugar ABC transporter s	None	1.6E-251	390	3	WP_015243290.1
1,198,426	1,198,427	2	CG -> AA	69 -> 70	Substitution	100.0%	NA -> NS	1.113	AAC,GCC -> AAT,	C770_RS47440	sugar ABC transporter s	Substitution	2.0E-228	371	3	WP_015243290.1
1,198,625	1,198,625	1	A -> G	63	SNP (transition)	100.0%		915	CGT -> CGC	C770_RS47440	sugar ABC transporter s	None	6.3E-215	305	3	WP_015243290.1
1,198,724	1,198,724	1	C -> G	70	SNP (transversion)	100.0%		816	TCG -> TCC	C770_RS47440	sugar ABC transporter s	None	1.0E-231	272	3	WP_015243290.1
1,198,772	1,198,772	1	G -> A	80	SNP (transition)	98.8%		768	GAC -> GAT	C770_RS47440	sugar ABC transporter s	None	2.0E-267	256	3	WP_015243290.1
1,198,876	1,198,876	1	G -> T	69	SNP (transversion)	100.0%		664	CGG -> AGG	C770_RS47440	sugar ABC transporter s	None	3.2E-242	222	1	WP_015243290.1
1,199,028	1,199,028	1	T -> G	68	SNP (transversion)	98.5%	E -> A	512	GAG -> GCG	C770_RS47440	sugar ABC transporter s	Substitution	2.1E-233	171	2	WP_015243290.1
1,199,035	1,199,035	1	C -> T	70	SNP (transition)	100.0%	A -> T	505	GCC -> ACC	C770_RS47440	sugar ABC transporter s	Substitution	1.0E-231	169	1	WP_015243290.1
1,199,045	1,199,045	1	C -> T	65	SNP (transition)	96.9%		495	GGG -> GGA	C770_RS47440	sugar ABC transporter s	None	1.3E-211	165	3	WP_015243290.1
1,199,087	1,199,087	1	G -> A	61	SNP (transition)	100.0%		453	TAC -> TAT	C770_RS47440	sugar ABC transporter s	None	2.5E-220	151	3	WP_015243290.1
1,199,095	1,199,095	1	G -> T	60	SNP (transversion)	98.3%	L -> M	445	CTG -> ATG	C770_RS47440	sugar ABC transporter s	Substitution	1.5E-199	149	1	WP_015243290.1
1,199,129	1,199,129	1	G -> A	68	SNP (transition)	98.5%		411	CTC -> CTT	C770_RS47440	sugar ABC transporter s	None	2.1E-233	137	3	WP_015243290.1
1,199,141	1,199,141	1	C -> G	68	SNP (transversion)	98.5%		399	GTG -> GTC	C770_RS47440	sugar ABC transporter s	None	2.1E-233	133	3	WP_015243290.1
1,199,144	1,199,144	1	C -> T	69	SNP (transition)	100.0%		396	ACG -> ACA	C770_RS47440	sugar ABC transporter s	None	4.0E-249	132	3	WP_015243290.1
1,199,152	1,199,153	2	AA -> GG	72	Substitution	100.0%		387	AAT,TTG -> AAC,	C770_RS47440	sugar ABC transporter s	None	1.0E-252	129	3	WP_015243290.1
1,199,185	1,199,185	1	G -> C	79	SNP (transversion)	100.0%	H -> D	355	CAC -> GAC	C770_RS47440	sugar ABC transporter s	Substitution	2.5E-269	119	1	WP_015243290.1
1,199,225	1,199,225	1	G -> A	87	SNP (transition)	100.0%		315	GGC -> GGT	C770_RS47440	sugar ABC transporter s	None	3.2E-305	105	3	WP_015243290.1
1,199,231	1,199,231	1	T -> C	82	SNP (transition)	100.0%		309	AAA -> AAG	C770_RS47440	sugar ABC transporter s	None	1.0E-287	103	3	WP_015243290.1
1,199,270	1,199,270	1	G -> A	85	SNP (transition)	100.0%		270	ATC -> ATT	C770_RS47440	sugar ABC transporter s	None	1.0E-272	90	3	WP_015243290.1
1,199,348	1,199,348	1	T -> G	89	SNP (transversion)	100.0%		192	ATA -> ATC	C770_RS47440	sugar ABC transporter s	None	2.0E-294	64	3	WP_015243290.1
1,199,447	1,199,447	1	G -> A	93	SNP (transition)	98.9%		93	AGC -> AGT	C770_RS47440	sugar ABC transporter s	None	3.7E-293	31	3	WP_015243290.1
1,199,461	1,199,461	1	G -> A	94	SNP (transition)	98.9%	L -> F	79	CTT -> TTT	C770_RS47440	sugar ABC transporter s	Substitution	4.7E-287	27	1	WP_015243290.1
1,199,566	1,199,566	1	G -> C	93	SNP (transversion)	98.9%							9.3E-321			
1,199,596	1,199,597	2	CA -> AT	91	Substitution	100.0%							0.0			
1,199,601	1,199,602	2	AG -> GA	91	Substitution	100.0%							0.0			
1,199,624	1,199,624	1	T -> C	92	SNP (transition)	98.9%							0.0			
1,199,628	1,199,629	2	GT -> AA	93 -> 94	Substitution	100.0%							6.3E-317			
1,199,640	1,199,640	1	C -> T	91	SNP (transition)	100.0%							0.0			
1,199,649	1,199,649	1	C -> A	89	SNP (transversion)	98.9%							8.9E-307			
1,199,734	1,199,734	1	A -> G	103	SNP (transition)	100.0%		48	GCA -> GCG	C770_RS47445	transcriptional regulato	None	0.0	16	3	WP_015243291.1
1,199,755	1,199,755	1	T -> C	99	SNP (transition)	99.0%		69	TCT -> TCC	C770_RS47445	transcriptional regulato	None	4.0E-322	23	3	WP_015243291.1
1,199,810	1,199,810	1	C -> G	96	SNP (transversion)	100.0%	Q -> E	124	CAG -> GAG	C770_RS47445	transcriptional regulato	Substitution	0.0	42	1	WP_015243291.1
1,199,815	1,199,815	1	C -> T	96	SNP (transition)	100.0%		129	ATC -> ATT	C770_RS47445	transcriptional regulato	None	6.3E-308	43	3	WP_015243291.1
1,199,881	1,199,881	1	C -> G	99	SNP (transversion)	100.0%		195	CTC -> CTG	C770_RS47445	transcriptional regulato	None	1.6E-317	65	3	WP_015243291.1
1,199,914	1,199,914	1	T -> C	97	SNP (transition)	100.0%		228	AGT -> AGC	C770_RS47445	transcriptional regulato	None	4.0E-311	76	3	WP_015243291.1
1,199,929	1,199,929	1	T -> C	99	SNP (transition)	99.0%		243	TAT -> TAC	C770_RS47445	transcriptional regulato	None	4.0E-322	81	3	WP_015243291.1
1,200,050	1,200,050	1	A -> G	81	SNP (transition)	100.0%	S -> G	364	AGT -> GGT	C770_RS47445	transcriptional regulato	Substitution	5.0E-268	122	1	WP_015243291.1
1,200,107	1,200,107	1	T -> C	71	SNP (transition)	100.0%	F -> L	421	TTC -> CTC	C770_RS47445	transcriptional regulato	Substitution	3.2E-249	141	1	WP_015243291.1
1,200,211	1,200,211	1	G -> C	70	SNP (transversion)	98.6%		525	ACG -> ACC	C770_RS47445	transcriptional regulato	None	2.2E-240	175	3	WP_015243291.1
1,200,232	1,200,232	1	T -> C	76	SNP (transition)	100.0%		546	GGT -> GGC	C770_RS47445	transcriptional regulato	None	6.3E-244	182	3	WP_015243291.1
1,200,307	1,200,307	1	T -> G	76	SNP (transversion)	100.0%		621	GCT -> GCG	C770_RS47445	transcriptional regulato	None	2.5E-236	207	3	WP_015243291.1
1,200,313	1,200,313	1	C -> T	76	SNP (transition)	97.4%		627	GCC -> GCT	C770_RS47445	transcriptional regulato	None	1.8E-241	209	3	WP_015243291.1
1,200,323	1,200,323	1	A -> C	81	SNP (transversion)	100.0%	S -> R	637	AGC -> CGC	C770_RS47445	transcriptional regulato	Substitution	4.0E-276	213	1	WP_015243291.1
1,200,361	1,200,361	1	T -> C	86	SNP (transition)	100.0%		675	AAT -> AAC	C770_RS47445	transcriptional regulato	None	1.0E-301	225	3	WP_015243291.1
1,200,395	1,200,397	2	GCG -> ACC	90	Substitution	100.0%	A -> T	709	GCG -> ACC	C770_RS47445	transcriptional regulato	Substitution	1.0E-306	237	1	WP_015243291.1
1,200,414	1,200,415	2	AG -> TC	87 -> 88	Substitution	98.9%	E -> V	728	GAG -> GTC	C770_RS47445	transcriptional regulato	Substitution	3.5E-291	243	2	WP_015243291.1
1,200,639	1,200,639	1	A -> G	86	SNP (transition)	100.0%	D -> G	953	GAC -> GGC	C770_RS47445	transcriptional regulato	Substitution	1.6E-284	318	2	WP_015243291.1
1,200,688	1,200,688	1	G -> C	80	SNP (transversion)	100.0%		1.002	GGG -> GGC	C770_RS47445	transcriptional regulato	None	1.0E-280	334	3	WP_015243291.1
1,200,768	1,200,769	2	TG -> CA	79	Substitution	98.7%	RG -> RS	57	CGT,GGC -> CGC	C770_RS47450	hypothetical protein	Substitution	5.0E-264	19	3	WP_015243292.1
1,200,817	1,200,818	2	TC -> AA	84	Substitution	98.8%	S -> N	106	TCT -> AAT	C770_RS47450	hypothetical protein	Substitution	4.2E-256	36	1	WP_015243292.1
1,200,843	1,200,843	1	T -> C	74	SNP (transition)	100.0%		132	GTT -> GTC	C770_RS47450	hypothetical protein	None	6.3E-245	44	3	WP_015243292.1
1,200,994	1,200,994	1	A -> G	81	SNP (transition)	97.5%	N -> D	283	AAC -> GAC	C770_RS47450	hypothetical protein	Substitution	1.0E-273	95	1	WP_015243292.1
1,201,001	1,201,001	1	A -> G	81	SNP (transition)	100.0%	E -> G	290	GAA -> GGA	C770_RS47450	hypothetical protein	Substitution	4.0E-276	97	2	WP_015243292.1
1,201,182	1,201,182	1	T -> C	79	SNP (transition)	98.7%		471	AAT -> AAC	C770_RS47450	hypothetical protein	None	5.0E-264	157	3	WP_015243292.1
1,201,317	1,201,317	1	C -> T	76	SNP (transition)	98.7%		606	AAC -> AAT	C770_RS47450	hypothetical protein	None	2.4E-261	202	3	WP_015243292.1
1,201,359	1,201,359	1	T -> C	73	SNP (transition)	98.6%		648	TTT -> TTC	C770_RS47450	hypothetical protein	None	7.3E-251	216	3	WP_015243292.1
1,201,425	1,201,425	1	G -> C	81	SNP (transversion)	97.5%		714	GTG -> GTC	C770_RS47450	hypothetical protein	None	8.1E-266	238	3	WP_015243292.1
1,201,530	1,201,530	1	A -> G	69	SNP (transition)	98.6%		819	GCA -> GCG	C770_RS47450	hypothetical protein	None	4.4E-230	273	3	WP_015243292.1
1,201,593	1,201,593	1	T -> C	75	SNP (transition)	97.3%		882	CAT -> CAC	C770_RS47450	hypothetical protein	None	1.7E-245	294	3	WP_015243292.1
1,201,669	1,201,671	2	CTT -> TTG	74 -> 75	Substitution	97.3%		958	CTT -> TTG	C770_RS47450	hypothetical protein	None	6.8E-235	320	1	WP_015243292.1
1,201,803	1,201,803	1	A -> T	81	SNP (transversion)	100.0%		1.092	GTA -> GTT	C770_RS47450	hypothetical protein	None	5.0E-268	364	3	WP_015243292.1
1,201,805	1,201,805	1	T -> C	81	SNP (transition)	100.0%	V -> A	1.094	GTC -> GCC	C770_RS47450	hypothetical protein	Substitution	6.3E-260	365	2	WP_015243292.1
1,201,812	1,201,812	1	C -> A	86	SNP (transversion)	98.8%		1.101	CGC -> CGA	C770_RS47450	hypothetical protein	None	8.6E-288	367	3	WP_015243292.1
1,201,815	1,201,815	1	C -> T	87	SNP (transition)	100.0%		1.104	CAC -> CAT	C770_RS47450	hypothetical protein	None	7.9E-288	368	3	WP_015243292.1
1,201,833	1,201,833	1	A -> G	87	SNP (transition)	98.9%	I -> M	1.122	ATA -> ATG	C770_RS47450	hypothetical protein	Substitution	3.5E-291	374	3	WP_015243292.1
1,201,860	1,201,860	1	C -> T	88	SNP (transition)	98.9%		1.149	CTC -> CTT	C770_RS47450	hypothetical protein	None	7.0E-286	383	3	WP_015243292.1
1,201,899	1,201,899	1	T -> C	80	SNP (transition)	100.0%		1.188	TCT -> TCC	C770_RS47450	hypothetical protein	None	1.0E-264	396	3	WP_015243292.1
1,202,012	1,202,012	1	A -> G	101	SNP (transition)	100.0%	D -> G	1.301	GAC -> GGC	C770_RS47450	hypothetical protein	Substitution	4.9E-324	434	2	WP_015243292.1
1,202,025	1,202,025	1	A -> G	98	SNP (transition)	99.0%		1.314	CGA -> CGG	C770_RS47450	hypothetical protein	None	0.0	438	3	WP_015243292.1
1,202,084	1,202,084	1	C -> T	90	SNP (transition)	100.0%	S -> F	1.373	TCC -> TTC	C770_RS47450	hypothetical protein	Substitution	1.0E-297	458	2	WP_015243292.1
1,202,130	1,202,130	1	G -> C	82	SNP (transversion)	100.0%		1.419	TCG -> TCC	C770_RS47450	hypothetical protein	None	2.5E-271	473	3	WP_015243292.1
1,202,210	1,202,210	1	A -> G	76	SNP (transition)	100.0%	Q -> R	1.499	CAG -> CGG	C770_RS47450	hypothetical protein	Substitution	6.3E-244	500	2	WP_015243292.1
1,202,250	1,202,250	1	G -> T	78	SNP (transversion)	96.2%		1.539	ACG -> ACT	C770_RS47450	hypothetical protein	None	2.4E-258	513	3	WP_015243292.1
1,202,337	1,202,337	1	G -> C	82	SNP (transversion)	100.0%		1.626	CCG -> CCC	C770_RS47450	hypothetical protein	None	2.5E-271	542	3	WP_015243292.1
1,202,392	1,202,392	1	G -> A	77	SNP (transition)	97.4%	V -> I	1.681	GTC -> ATC	C770_RS47450	hypothetical protein	Substitution	9.2E-245	561	1	WP_015243292.1
1,202,424	1,202,424	1	T -> C	82	SNP (transition)	100.0%		1.713	GCT -> GCC	C770_RS47450	hypothetical protein	None	1.0E-287	571	3	WP_015243292.1
1,202,472	1,202,472	1	T -> C	86	SNP (transition)	100.0%		1.761	AAT -> AAC	C770_RS47450	hypothetical protein	None	4.0E-293	587	3	WP_015243292.1
1,202,642	1,202,642	1	A -> T	95	SNP (transversion)	100.0%		81	CTA -> CTT	C770_RS47455	phosphoglycerate dehy	None	3.2E-314	27	3	WP_015243293.1
1,202,683	1,202,683	1	A -> C	98	SNP (transversion)	100.0%	E -> A	122	GAG -> GCG	C770_RS47455	phosphoglycerate dehy	Substitution	0.0	41	2	WP_015243293.1
1,202,732	1,202,732	1	G -> A	87	SNP (transition)	100.0%		171	TTG -> TTA	C770_RS47455	phosphoglycerate dehy	None	1.6E-296	57	3	WP_015243293.1
1,203,005	1,203,005	1	G -> A	84	SNP (transition)	98.8%		444	CAG -> CAA	C770_RS47455	phosphoglycerate dehy	None	1.1E-272	148	3	WP_015243293.1
1,203,401	1,203,401	1	T -> C	70	SNP (transition)	100.0%		840	AAT -> AAC	C770_RS47455	phosphoglycerate dehy	None	1.0E-238	280	3	WP_015243293.1
1,203,601	1,203,601	1	C -> A	74	SNP (transversion)	100.0%		33	GTC -> GTA	C770_RS47460	Sugar phosphate isome	None	6.3E-245	11	3	WP_015243294.1
1,203,617	1,203,617	1	G -> C	74	SNP (transversion)	98.6%	A -> P	49	GCC -> CCC	C770_RS47460	Sugar phosphate isome	Substitution	1.9E-232	17	1	WP_015243294.1
1,203,631	1,203,631	1	C -> T	76	SNP (transition)	100.0%		63	ATC -> ATT	C770_RS47460	Sugar phosphate isome	None	1.6E-251	21	3	WP_015243294.1
1,203,640	1,203,640	1	G -> A	76	SNP (transition)	100.0%		72	CTG -> CTA	C770_RS47460	Sugar phosphate isome	None	4.0E-259	24	3	WP_015243294.1
1,203,661	1,203,661	1	C -> G	77	SNP (transversion)	100.0%		93	GCC -> GCG	C770_RS47460	Sugar phosphate isome	None	1.6E-262	31	3	WP_015243294.1
1,203,664	1,203,664	1	T -> G	78	SNP (transversion)	100.0%		96	GCT -> GCG	C770_RS47460	Sugar phosphate isome	None	1.0E-273	32	3	WP_015243294.1
1,203,697	1,203,697	1	G -> C	78	SNP (transversion)	100.0%		129	CCG -> CCC	C770_RS47460	Sugar phosphate isome	None	2.5E-250	43	3	WP_015243294.1
1,203,721	1,203,721	1	T -> G	76	SNP (transversion)	100.0%		153	ACT -> ACG	C770_RS47460	Sugar phosphate isome	None	1.6E-251	51	3	WP_015243294.1
1,203,727	1,203,727	1	G -> A	79	SNP (transition)	98.7%		159	GGG -> GGA	C770_RS47460	Sugar phosphate isome	None	5.0E-264	53	3	WP_015243294.1
1,203,751	1,203,751	1	G -> C	76	SNP (transversion)	100.0%		183	CTG -> CTC	C770_RS47460	Sugar phosphate isome	None	4.0E-259	61	3	WP_015243294.1
1,203,757	1,203,757	1	C -> T	74	SNP (transition)	98.6%		189	ACC -> ACT	C770_RS47460	Sugar phosphate isome	None	1.9E-232	63	3	WP_015243294.1
1,203,820	1,203,820	1	G -> A	77	SNP (transition)	98.7%		252	GCG -> GCA	C770_RS47460	Sugar phosphate isome	None	1.9E-234	84	3	WP_015243294.1
1,203,823	1,203,823	1	T -> C	79	SNP (transition)	100.0%		255	ACT -> ACC	C770_RS47460	Sugar phosphate isome	None	1.6E-253	85	3	WP_015243294.1
1,203,888	1,203,888	1	A -> G	88	SNP (transition)	100.0%	D -> G	320	GAT -> GGT	C770_RS47460	Sugar phosphate isome	Substitution	6.3E-300	107	2	WP_015243294.1
1,203,905	1,203,905	1	T -> C	89	SNP (transition)	100.0%	W -> R	337	TGG -> CGG	C770_RS47460	Sugar phosphate isome	Substitution	3.2E-312	113	1	WP_015243294.1
1,203,934	1,203,934	1	T -> C	81	SNP (transition)	98.8%		366	GAT -> GAC	C770_RS47460	Sugar phosphate isome	None	8.1E-279	122	3	WP_015243294.1
1,203,940	1,203,940	1	T -> C	82	SNP (transition)	100.0%		372	GGT -> GGC	C770_RS47460	Sugar phosphate isome	None	1.0E-287	124	3	WP_015243294.1
1,203,952	1,203,952	1	C -> T	79	SNP (transition)	98.7%		384	GCC -> GCT	C770_RS47460	Sugar phosphate isome	None	2.0E-248	128	3	WP_015243294.1
1,204,024	1,204,024	1	T -> C	94	SNP (transition)	100.0%		456	CGT -> CGC	C770_RS47460	Sugar phosphate isome	None	2.5E-320	152	3	WP_015243294.1
1,204,030	1,204,030	1	A -> C	90	SNP (transversion)	100.0%		462	ATA -> ATC	C770_RS47460	Sugar phosphate isome	None	1.0E-306	154	3	WP_015243294.1
1,204,033	1,204,033	1	A -> G	91	SNP (transition)	100.0%		465	GAA -> GAG	C770_RS47460	Sugar phosphate isome	None	5.0E-301	155	3	WP_015243294.1
1,204,045	1,204,045	1	C -> T	83	SNP (transition)	100.0%		477	CAC -> CAT	C770_RS47460	Sugar phosphate isome	None	6.3E-283	159	3	WP_015243294.1
1,204,057	1,204,057	1	C -> T	88	SNP (transition)	100.0%		489	TAC -> TAT	C770_RS47460	Sugar phosphate isome	None	4.0E-291	163	3	WP_015243294.1
1,204,099	1,204,099	1	G -> A	90	SNP (transition)	100.0%		531	GCG -> GCA	C770_RS47460	Sugar phosphate isome	None	1.0E-306	177	3	WP_015243294.1

1,204,161	1,204,161	1	G -> A	85	SNP (transition)	98.8%	R -> H	593	CGC -> CAC	C770_RS47460	Sugar phosphate isome	Substitution	5.4E-276	198	2	WP_015243294.1
1,204,172	1,204,172	1	C -> T	82	SNP (transition)	100.0%	R -> C	604	CGC -> TGC	C770_RS47460	Sugar phosphate isome	Substitution	2.5E-271	202	1	WP_015243294.1
1,204,208	1,204,208	1	G -> A	89	SNP (transition)	100.0%	A -> T	640	GCG -> ACG	C770_RS47460	Sugar phosphate isome	Substitution	1.6E-285	214	1	WP_015243294.1
1,204,279	1,204,279	1	A -> C	90	SNP (transversion)	100.0%		711	GGA -> GGC	C770_RS47460	Sugar phosphate isome	None	1.0E-306	237	3	WP_015243294.1
1,204,373	1,204,373	1	G -> A	79	SNP (transition)	100.0%	D -> N	805	GAT -> AAT	C770_RS47460	Sugar phosphate isome	Substitution	3.2E-277	269	1	WP_015243294.1
1,204,397	1,204,397	1	A -> T	87	SNP (transversion)	97.7%							3.7E-286			
1,204,413	1,204,413	1	A -> G	86	SNP (transition)	100.0%							4.0E-293			
1,204,424	1,204,424	1	T -> C	83	SNP (transition)	100.0%							3.2E-291			
1,204,430	1,204,430	1	C -> A	82	SNP (transversion)	100.0%							4.0E-263			
1,204,454	1,204,454	1	T -> C	85	SNP (transition)	100.0%							1.0E-272			
1,204,503	1,204,503	1	A -> G	78	SNP (transition)	100.0%							4.0E-258			
1,204,550	1,204,550	1	G -> A	70	SNP (transition)	100.0%							1.0E-231			
1,204,585	1,204,585	1	A -> G	75	SNP (transition)	98.7%							4.7E-243			
1,204,613	1,204,613	1	A -> G	72	SNP (transition)	100.0%							2.5E-238			
1,204,631	1,204,631	1	G -> A	76	SNP (transition)	100.0%							1.6E-251			
1,204,649	1,204,649	1	T -> A	73	SNP (transversion)	97.3%							1.0E-238			
1,204,691	1,204,691	1	C -> T	64	SNP (transition)	100.0%							1.6E-205			
1,204,694	1,204,694	1	A -> G	65	SNP (transition)	100.0%							1.0E-208			
1,204,744	1,204,744	1	C -> T	65	SNP (transition)	98.5%	S -> L	41	TCG -> TTG	C770_RS47465	aminoglycoside adenyl	Substitution	4.1E-210	14	2	WP_049589732.1
1,204,784	1,204,784	1	G -> A	65	SNP (transition)	100.0%		81	CCG -> CCA	C770_RS47465	aminoglycoside adenyl	None	3.2E-228	27	3	WP_049589732.1
1,204,886	1,204,886	1	C -> T	68	SNP (transition)	100.0%		183	TTC -> TTT	C770_RS47465	aminoglycoside adenyl	None	4.0E-225	61	3	WP_049589732.1
1,204,928	1,204,928	1	C -> T	72	SNP (transition)	100.0%		225	CAC -> CAT	C770_RS47465	aminoglycoside adenyl	None	1.6E-245	75	3	WP_049589732.1
1,204,937	1,204,937	1	C -> T	72	SNP (transition)	100.0%		234	GCC -> GCT	C770_RS47465	aminoglycoside adenyl	None	1.0E-252	78	3	WP_049589732.1
1,204,940	1,204,940	1	T -> G	73	SNP (transversion)	100.0%	N -> K	237	AAT -> AAG	C770_RS47465	aminoglycoside adenyl	Substitution	3.2E-256	79	3	WP_049589732.1
1,204,948	1,204,948	1	A -> G	72	SNP (transition)	100.0%	K -> R	245	AAG -> AGG	C770_RS47465	aminoglycoside adenyl	Substitution	6.3E-224	82	2	WP_049589732.1
1,205,009	1,205,010	2	AA -> GT	74 -> 75	Substitution	98.6% -> 98.7%	GT -> GS	306	GGA,ACA -> GGC	C770_RS47465	aminoglycoside adenyl	Substitution	3.2E-248	102	3	WP_049589732.1
1,205,105	1,205,105	1	C -> T	86	SNP (transition)	100.0%		402	GCC -> GCT	C770_RS47465	aminoglycoside adenyl	None	1.0E-301	134	3	WP_049589732.1
1,205,108	1,205,108	1	C -> G	87	SNP (transversion)	100.0%		405	GTC -> GTG	C770_RS47465	aminoglycoside adenyl	None	1.6E-296	135	3	WP_049589732.1
1,205,165	1,205,165	1	G -> A	84	SNP (transition)	100.0%		462	TTG -> TTA	C770_RS47465	aminoglycoside adenyl	None	1.6E-269	154	3	WP_049589732.1
1,205,209	1,205,209	1	T -> C	83	SNP (transition)	100.0%							6.3E-283			
1,205,234	1,205,234	1	A -> CC	85 -> 86	Insertion	100.0%							3.2E-281			
1,205,239	1,205,239	1	G -> A	88	SNP (transition)	100.0%							1.0E-308			
1,205,241	1,205,241	1	A -> G	89	SNP (transition)	96.6%							1.1E-296			
1,205,297	1,205,297	1	G -> T	86	SNP (transversion)	97.7%							2.3E-274			
1,205,300	1,205,300	1	G -> A	86	SNP (transition)	100.0%							4.0E-293			
1,205,326	1,205,326	1	T -> GCG	80 -> 81	Insertion	98.8%							1.6E-259			
1,205,340	1,205,340	1	A -> G	82	SNP (transition)	100.0%							1.6E-279			
1,205,350	1,205,350	1	C -> G	83	SNP (transversion)	100.0%							3.2E-291			
1,205,382	1,205,382	1	C -> G	82	SNP (transversion)	100.0%							2.5E-271			
1,205,424	1,205,424	1	C -> G	73	SNP (transversion)	100.0%							3.2E-256			
1,205,436	1,205,436	1	G -> C	73	SNP (transversion)	98.6%							1.8E-236			
1,205,486	1,205,486	1	C -> T	52	SNP (transition)	100.0%							1.6E-177			
1,205,488	1,205,488	1	(C)3 -> (C)2	52	Deletion (tandem repeat)	100.0%							6.3E-84			
1,205,501	1,205,501	1	C -> T	47	SNP (transition)	100.0%							3.2E-165			
1,205,539	1,205,539	1	G -> C	36	SNP (transversion)	100.0%							1.0E-126			
1,205,601	1,205,601	1	(T)4 -> (T)3	19	Deletion (tandem repeat)	100.0%							4.0E-31			
1,205,610	1,205,610	1	-C	15	Deletion	100.0%							1.0E-21			
1,205,655	1,205,655	1	G -> C	10	SNP (transversion)	100.0%							1.0E-33			
1,205,663	1,205,664	2	GG -> TT	9	Substitution	100.0%							3.2E-32			
1,205,666	1,205,666	1	G -> A	11	SNP (transition)	100.0%							7.9E-35			
1,205,688	1,205,688	1	T -> G	19	SNP (transversion)	100.0%							4.0E-69			
1,205,717	1,205,717	1	G -> C	26	SNP (transversion)	100.0%							2.5E-81			
1,205,719	1,205,719	1	G -> A	26	SNP (transition)	100.0%							1.6E-86			
1,205,753	1,205,753	1	G -> T	36	SNP (transversion)	97.2%							3.6E-111			
1,205,772	1,205,771	0	(C)2 -> (C)3	40	Insertion (tandem repeat)	100.0%							1.0E-132			
1,205,789	1,205,789	1	A -> T	45	SNP (transversion)	97.8%		627	GTT -> GTA	C770_RS47470	hypothetical protein	None	1.1E-148	209	3	WP_015243296.1
1,205,807	1,205,807	1	T -> G	52	SNP (transversion)	100.0%		609	GCA -> GCC	C770_RS47470	hypothetical protein	None	1.6E-177	203	3	WP_015243296.1
1,205,950	1,205,950	1	G -> A	89	SNP (transition)	100.0%	P -> S	466	CCC -> TCC	C770_RS47470	hypothetical protein	Substitution	2.0E-294	156	1	WP_015243296.1
1,205,977	1,205,977	1	T -> C	91	SNP (transition)	98.9%	T -> A	439	ACG -> GCG	C770_RS47470	hypothetical protein	Substitution	9.1E-305	147	1	WP_015243296.1
1,206,024	1,206,024	1	C -> G	96	SNP (transversion)	100.0%	S -> T	392	AGC -> ACC	C770_RS47470	hypothetical protein	Substitution	0.0	131	2	WP_015243296.1
1,206,038	1,206,038	1	G -> A	94	SNP (transition)	100.0%		378	AGC -> AGT	C770_RS47470	hypothetical protein	None	2.5E-320	126	3	WP_015243296.1
1,206,097	1,206,097	1	T -> C	83	SNP (transition)	100.0%	T -> A	319	ACC -> GCC	C770_RS47470	hypothetical protein	Substitution	3.2E-291	107	1	WP_015243296.1
1,206,107	1,206,107	1	T -> C	84	SNP (transition)	100.0%	I -> M	309	ATA -> ATG	C770_RS47470	hypothetical protein	Substitution	2.5E-286	103	3	WP_015243296.1
1,206,155	1,206,155	1	T -> C	86	SNP (transition)	100.0%		261	CCA -> CCG	C770_RS47470	hypothetical protein	None	4.0E-293	87	3	WP_015243296.1
1,206,313	1,206,313	1	C -> G	81	SNP (transversion)	98.8%	V -> L	103	GTC -> CTC	C770_RS47470	hypothetical protein	Substitution	8.1E-279	35	1	WP_015243296.1
1,206,362	1,206,362	1	T -> C	85	SNP (transition)	100.0%		54	GGA -> GGG	C770_RS47470	hypothetical protein	None	1.0E-289	18	3	WP_015243296.1
1,206,373	1,206,375	3	GGT	87	Deletion	100.0%	NQ -> K	41	AAC,CAG -> AAG	C770_RS47470	hypothetical protein	Deletion	1.3E-148	14	2	WP_015243296.1
1,206,449	1,206,449	1	C -> T	89	SNP (transition)	98.9%	A -> T	505	GCT -> ACT	C770_RS47475	DNA-directed RNA poly	Substitution	5.6E-298	169	1	WP_015243297.1
1,206,513	1,206,513	1	A -> G	82	SNP (transition)	98.8%		441	GCT -> GCC	C770_RS47475	DNA-directed RNA poly	None	3.3E-274	147	3	WP_015243297.1
1,206,555	1,206,555	1	A -> G	84	SNP (transition)	100.0%		399	CGT -> CGC	C770_RS47475	DNA-directed RNA poly	None	1.0E-294	133	3	WP_015243297.1
1,206,602	1,206,603	2	AC -> CT	88 -> 89	Substitution	96.6%	RY -> RD	351	CGG,TAC -> CGA	C770_RS47475	DNA-directed RNA poly	Substitution	3.5E-276	117	3	WP_015243297.1
1,206,639	1,206,639	1	C -> T	89	SNP (transition)	100.0%		315	GAG -> GAA	C770_RS47475	DNA-directed RNA poly	None	2.0E-294	105	3	WP_015243297.1
1,206,648	1,206,648	1	A -> G	88	SNP (transition)	100.0%		306	AGT -> AGC	C770_RS47475	DNA-directed RNA poly	None	6.3E-300	102	3	WP_015243297.1
1,206,665	1,206,666	2	CG -> AA	83 -> 84	Substitution	96.4%	HA -> HS	288	CAC,GCT -> CAT,	C770_RS47475	DNA-directed RNA poly	Substitution	9.2E-252	96	3	WP_015243297.1
1,206,738	1,206,738	1	C -> T	85	SNP (transition)	100.0%		216	GGG -> GGA	C770_RS47475	DNA-directed RNA poly	None	3.2E-281	72	3	WP_015243297.1
1,206,864	1,206,864	1	C -> A	88	SNP (transversion)	97.7%	R -> S	90	AGG -> AGT	C770_RS47475	DNA-directed RNA poly	Substitution	1.5E-289	30	3	WP_015243297.1
1,206,896	1,206,896	1	C -> T	88	SNP (transition)	100.0%	D -> N	58	GAC -> AAC	C770_RS47475	DNA-directed RNA poly	Substitution	6.3E-300	20	1	WP_015243297.1
1,207,002	1,207,002	1	G -> C	95	SNP (transversion)	98.9%							0.0			
1,207,136	1,207,136	1	G -> A	88	SNP (transition)	100.0%							1.0E-308			
1,207,346	1,207,346	1	A -> C	74	SNP (transversion)	100.0%		144	GTA -> GTC	C770_RS47480	membrane protein	None	6.3E-245	48	3	WP_013845873.1
1,207,358	1,207,358	1	C -> T	73	SNP (transition)	98.6%		156	GGC -> GGT	C770_RS47480	membrane protein	None	1.8E-236	52	3	WP_013845873.1
1,207,448	1,207,448	1	G -> C	71	SNP (transversion)	100.0%		246	GGG -> GGC	C770_RS47480	membrane protein	None	4.0E-242	82	3	WP_013845873.1
1,207,515	1,207,515	1	G -> C	65	SNP (transversion)	100.0%	V -> L	313	GTG -> CTG	C770_RS47480	membrane protein	Substitution	1.0E-221	105	1	WP_013845873.1
1,207,536	1,207,536	1	T -> C	67	SNP (transition)	100.0%							3.2E-235			
1,207,573	1,207,573	1	T -> A	71	SNP (transversion)	100.0%		15	ACT -> ACA	C770_RS47485	hypothetical protein	None	4.0E-242	5	3	WP_015243298.1
1,207,582	1,207,582	1	A -> G	70	SNP (transition)	100.0%		24	GCA -> GCG	C770_RS47485	hypothetical protein	None	1.0E-224	8	3	WP_015243298.1
1,207,585	1,207,585	1	C -> T	70	SNP (transition)	100.0%		27	GAC -> GAT	C770_RS47485	hypothetical protein	None	1.0E-224	9	3	WP_015243298.1
1,207,587	1,207,587	1	C -> G	71	SNP (transversion)	100.0%	P -> R	29	CCC -> CGC	C770_RS47485	hypothetical protein	Substitution	6.3E-228	10	2	WP_015243298.1
1,207,594	1,207,594	1	A -> G	73	SNP (transition)	100.0%		36	CAA -> CAG	C770_RS47485	hypothetical protein	None	1.3E-241	12	3	WP_015243298.1
1,207,596	1,207,596	1	C -> T	73	SNP (transition)	98.6%	P -> L	38	CCG -> CTG	C770_RS47485	hypothetical protein	Substitution	1.8E-236	13	2	WP_015243298.1
1,207,628	1,207,628	1	G -> A	77	SNP (transition)	100.0%	D -> N	70	GAT -> AAT	C770_RS47485	hypothetical protein	Substitution	1.0E-231	24	1	WP_015243298.1
1,207,648	1,207,648	1	T -> C	75	SNP (transition)	100.0%		90	AGT -> AGC	C770_RS47485	hypothetical protein	None	3.2E-248	30	3	WP_015243298.1
1,207,651	1,207,651	1	C -> T	75	SNP (transition)	97.3%		93	TTC -> TTT	C770_RS47485	hypothetical protein	None	1.4E-223	31	3	WP_015243298.1
1,207,684	1,207,684	1	C -> A	81	SNP (transversion)	100.0%	D -> E	126	GAC -> GAA	C770_RS47485	hypothetical protein	Substitution	5.0E-268	42	3	WP_015243298.1
1,207,786	1,207,786	1	A -> G	89	SNP (transition)	100.0%		228	CCA -> CCG	C770_RS47485	hypothetical protein	None	2.0E-294	76	3	WP_015243298.1
1,207,831	1,207,831	1	A -> G	92	SNP (transition)	100.0%		273	TCA -> TCG	C770_RS47485	hypothetical protein	None	1.6E-313	91	3	WP_015243298.1
1,207,842	1,207,842	1	G -> A	86	SNP (transition)	100.0%	G -> D	284	GGT -> GAT	C770_RS47485	hypothetical protein	Substitution	6.3E-276	95	2	WP_015243298.1
1,207,945	1,207,945	1	G -> C	95	SNP (transversion)	97.9%		387	CTG -> CTC	C770_RS47485	hypothetical protein	None	1.4E-322	129	3	WP_015243298.1
1,208,049	1,208,049	1	G -> C	83	SNP (transversion)	100.0%	S -> T	491	AGC -> ACC	C770_RS47485	hypothetical protein	Substitution	1.3E-274	164	2	WP_015243298.1
1,208,064	1,208,064	1	A -> G	80	SNP (transition)	100.0%	K -> R	506	AAG -> AGG	C770_RS47485	hypothetical protein	Substitution	1.0E-264	169	2	WP_015243298.1
1,208,162	1,208,162	1	A -> G	89	SNP (transition)	100.0%	T -> A	604	ACC -> GCC	C770_RS47485	hypothetical protein	Substitution	3.2E-312	202	1	WP_015243298.1
1,208,245	1,208,245	1	G -> A	91	SNP (transition)	100.0%		687	GCG -> GCA	C770_RS47485	hypothetical protein	None	0.0	229	3	WP_015243298.1
1,208,359	1,208,359	1	G -> T	102	SNP (transversion)	100.0%		801	CCG -> CCT	C770_RS47485	hypothetical protein	None	0.0	267	3	WP_015243298.1
1,208,584	1,208,584	1	C -> A	91	SNP (transversion)	98.9%	T -> K	110	ACG -> AAG	C770_RS47490	hypothetical protein	Substitution	9.1E-278	37	2	WP_010975387.1
1,208,612	1,208,612	1	G -> T	87	SNP (transversion)	100.0%	R -> S	138	AGG -> AGT	C770_RS47490	hypothetical protein	Substitution	7.9E-288	46	3	WP_010975387.1
1,208,619	1,208,620	2	GC -> AA	86 -> 87	Substitution	98.8% -> 98.9%	A -> N	145	GCT -> AAT	C770_RS47490	hypothetical protein	Substitution	2.7E-262	49	1	WP_010975387.1
1,208,742	1,208,742	1	G -> A	78	SNP (transition)	100.0%	V -> M	268	GTG -> ATG	C770_RS47490	hypothetical protein	Substitution	2.5E-250	90	1	WP_010975387.1
1,208,792	1,208,792	1	C -> T	79	SNP (transition)	98.7%		318	GAC -> GAT	C770_RS47490	hypothetical protein	None	2.0E-248	106	3	WP_010975387.1
1,208,834	1,208,834	1	A -> G	86	SNP (transition)	100.0%		360	CTA -> CTG	C770_RS47490	hypothetical protein	None	4.0E-293	120	3	WP_010975387.1
1,208,885	1,208,885	1	C -> A	88	SNP (transversion)	98.9%		411	GCC -> GCA	C770_RS47490	hypothetical protein	None	1.4E-294	137	3	WP_010975387.1
1,208,931	1,208,931	1	A -> G	87	SNP (transition)	100.0%	T -> A	457	ACT -> GCT	C770_RS47490	hypothetical protein	Substitution	3.2E-305	153	1	WP_010975387.1
1,208,975	1,208,975	1	T -> C	90	SNP (transition)	98.9%		501	GGT -> GGC	C770_RS47490	hypothetical protein	None	2.8E-310	167	3	WP_010975387.1
1,209,035	1,209,035	1	C -> A	91	SNP (transversion)	98.9%		561	CCC -> CCA	C770_RS47490	hypothetical protein	None	9.1E-305	187	3	WP_010975387.1
1,209,058	1,209,058	1	T -> C	95	SNP (transition)	98.9%	V -> A	584	GTG -> GCG	C770_RS47490	hypothetical protein	Substitution	0.0	195	2	WP_010975387.1
1,209,123	1,209,123	1	A -> G	87	SNP (transition)	100.0%	I -> V	649	ATC -> GTC	C770_RS47490	hypothetical protein	Substitution	7.9E-288	217	1	WP_010975387.1
1,209,191	1,209,191	1	A -> T	88	SNP (transversion)	98.9%		717	GCA -> GCT	C770_RS47490	hypothetical protein	None	1.4E-294	239	3	WP_010975387.1
1,209,321	1,209,321	1	C -> G	79	SNP (transversion)	100.0%							1.6E-253			
1,209,331	1,209,331	1	G -> C	74	SNP (transversion)	100.0%	G -> R	7	GGT -> CGT	C770_RS47495	membrane protein	Substitution	2.5E-252	3	1	WP_049589740.1
1,209,346	1,209,346	1	G -> A	71	SNP (transition)	100.0%	V -> I	22	GTC -> ATC	C770_RS47495	membrane protein	Substitution	4.0E-242	8	1	WP_049589740.1
1,209,372	1,209,372	1	G -> C	72	SNP (transversion)	98.6%		48	GCG -> GCC	C770_RS47495	membrane protein	None	3.6E-233	16	3	WP_049589740.1
1,209,477	1,209,477	1	T -> C	70	SNP (transition)	100.0%		153	GCT -> GCC	C770_RS47495	membrane protein	None	1.0E-231	51	3	WP_049589740.1
1,209,545	1,209,545	1	C -> T	72	SNP (transition)	100.0%	A -> V	221	GCG -> GTG	C770_RS47495	membrane protein	Substitution	1.6E-245	74	2	WP_049589740.1
1,209,569	1,209,569	1	T -> C	71	SNP (transition)	100.0%	F -> S	245	TTC -> TCC	C770_RS47495	membrane protein	Substitution	5.0E-235	82	2	WP_049589740.1
1,209,772	1,209,772	1	A -> G	72	SNP (transition)	100.0%	S -> G	448	AGT -> GGT	C770_RS47495	membrane protein	Substitution	1.6E-245	150	1	WP_049589740.1
1,209,796	1,209,796	1	G -> A	64	SNP (transition)	98.4%							2.0E-219			
1,209,804	1,209,803	0	A	64	Insertion	100.0%							6.3E-212			
1,209,829	1,209,829	1	C -> G	57	SNP (transversion)	100.0%							1.6E-194			
1,209,832	1,209,832	1	G -> A	57	SNP (transition)	98.2%							9.0E-184			
1,209,912	1,209,912	1	G -> A	35	SNP (transition)	97.1%							8.8E-115			
1,209,948	1,209,948	1	A -> C	23	SNP (transversion)	100.0%							1.6E-83			
1,209,978	1,209,978	1	A -> T	18	SNP (transversion)	100.0%							2.5E-58			
1,209,997	1,209,997	1	C -> G	15	SNP (transversion)	100.0%							1.0E-45			
1,210,006	1,210,008	3	GAC -> CCG	10 -> 12	Substitution	100.0%							1.0E-26			
1,210,012	1,210,012	1	G -> A	11	SNP (transition)	100.0%							6.3E-36			
1,210,015	1,210,015	1	G -> C	17	SNP (transversion)	100.0%							5.0E-50			
1,210,022	1,210,023	2	TT -> CG	16 -> 17	Substitution	100.0%							6.3E-52			
1,210,025	1,210,025	1	C -> T	17	SNP (transition)	100.0%							7.9E-57			
1,210,029	1,210,029	1	T -> C	18	SNP (transition)	100.0%							2.5E-58			
1,210,031	1,210,031	1	G -> C	19	SNP (transversion)	100.0%							2.0E-63			
1,210,034	1,210,035	2	AG -> GC	19	Substitution	100.0%							1.3E-59			
1,210,041	1,210,041	1	G -> C	15	SNP (transversion)	100.0%							1.0E-45			
1,210,043	1,210,043	1	A -> T	15	SNP (transversion)	100.0%							3.2E-41			
1,210,049	1,210,049	1	G -> A	15	SNP (transition)	100.0%							1.0E-45			
1,210,052	1,210,060	9	AAACGGTGC -> (	15 -> 17	Substitution	100.0%							3.2E-50			
1,210,062	1,210,063	2	CC -> AT	18	Substitution	100.0%							1.6E-56			
1,210,066	1,210,067	2	CG -> AC	18	Substitution	100.0%							4.0E-60			
1,210,069	1,210,069	1	C -> T	18	SNP (transition)	100.0%							2.5E-58			
1,210,071	1,210,071	1	A -> G	20	SNP (transition)	100.0%							1.0E-62			
1,210,073	1,210,073	1	G -> C	21	SNP (transversion)	100.0%							5.0E-70			
1,210,081	1,210,081	1	A -> G	21	SNP (transition)	100.0%							3.2E-74			
1,210,132	1,210,132	1	C -> T	38	SNP (transition)	97.4%	V -> I	265	GTC -> ATC	C770_RS47500	hypothetical protein	Substitution	3.0E-121	89	1	WP_015243300.1
1,210,138	1,210,138	1	C -> T	39	SNP (transition)	100.0%	A -> T	259	GCT -> ACT	C770_RS47500	hypothetical protein	Substitution	1.6E-125	87	1	WP_015243300.1
1,210,265	1,210,265	1	G -> C	77	SNP (transversion)	98.7%		132	GCC -> GCG	C770_RS47500	hypothetical protein	None	3.1E-257	44	3	WP_015243300.1
1,210,359	1,210,359	1	A -> G	74	SNP (transition)	100.0%	I -> T	38	ATC -> ACC	C770_RS47500	hypothetical protein	Substitution	2.5E-252	13	2	WP_015243300.1
1,210,414	1,210,414	1	A -> T	76	SNP (transversion)	100.0%	N -> K	573	AAT -> AAA	C770_RS47505	transcriptional regulato	Substitution	6.3E-244	191	3	WP_015243301.1
1,210,530	1,210,530	1	G -> A	79	SNP (transition)	98.7%		457	CTA -> TTA	C770_RS47505	transcriptional regulato	None	3.1E-256	153	1	WP_015243301.1
1,210,537	1,210,537	1	T -> C	79	SNP (transition)	100.0%		450	CTA -> CTG	C770_RS47505	transcriptional regulato	None	3.2E-277	150	3	WP_015243301.1
1,210,578	1,210,578	1	T -> G	76	SNP (transversion)	100.0%	S -> R	409	AGT -> CGT	C770_RS47505	transcriptional regulato	Substitution	4.0E-259	137	1	WP_015243301.1
1,210,608	1,210,608	1	G -> C	80	SNP (transversion)	100.0%	L -> V	379	CTT -> GTT	C770_RS47505	transcriptional regulato	Substitution	1.0E-280	127	1	WP_015243301.1
1,210,612	1,210,612	1	G -> A	78	SNP (transition)	100.0%		375	TTC -> TTT	C770_RS47505	transcriptional regulato	None	4.0E-258	125	3	WP_015243301.1

1,210,651	1,210,651	1	T -> C		79	SNP (transition)	100.0%		336	AAA -> AAG	C770_RS47505	transcriptional regulato	None	2.5E-269	112	3	WP_015243301.1
1,210,695	1,210,695	1	C -> T		77	SNP (transition)	97.4%	D -> N	292	GAT -> AAT	C770_RS47505	transcriptional regulato	Substitution	2.9E-237	98	1	WP_015243301.1
1,210,705	1,210,705	1	G -> T		72	SNP (transversion)	100.0%		282	GCC -> GCA	C770_RS47505	transcriptional regulato	None	1.0E-252	94	3	WP_015243301.1
1,210,769	1,210,769	1	G -> A		75	SNP (transition)	100.0%	T -> M	218	ACG -> ATG	C770_RS47505	transcriptional regulato	Substitution	3.2E-263	73	2	WP_015243301.1
1,210,802	1,210,802	1	C -> T		83	SNP (transition)	100.0%	G -> D	185	GGC -> GAC	C770_RS47505	transcriptional regulato	Substitution	1.3E-274	62	2	WP_015243301.1
1,210,816	1,210,816	1	A -> G		81	SNP (transition)	100.0%		171	ATT -> ATC	C770_RS47505	transcriptional regulato	None	3.2E-284	57	3	WP_015243301.1
1,210,830	1,210,830	1	G -> A		82	SNP (transition)	97.6%		157	CTG -> TTG	C770_RS47505	transcriptional regulato	None	3.3E-261	53	1	WP_015243301.1
1,210,882	1,210,882	1	T -> C		84	SNP (transition)	97.6%		105	GTA -> GTG	C770_RS47505	transcriptional regulato	None	2.2E-251	35	3	WP_015243301.1
1,210,885	1,210,885	1	A -> G		85	SNP (transition)	100.0%		102	GCT -> GCC	C770_RS47505	transcriptional regulato	None	3.2E-281	34	3	WP_015243301.1
1,210,912	1,210,912	1	G -> T		83	SNP (transversion)	100.0%		75	GCC -> GCA	C770_RS47505	transcriptional regulato	None	1.3E-274	25	3	WP_015243301.1
1,210,924	1,210,924	1	G -> A		83	SNP (transition)	100.0%		63	AGC -> AGT	C770_RS47505	transcriptional regulato	None	1.3E-274	21	3	WP_015243301.1
1,210,932	1,210,932	1	A -> G		88	SNP (transition)	100.0%	Y -> H	55	TAC -> CAC	C770_RS47505	transcriptional regulato	Substitution	4.0E-291	19	1	WP_015243301.1
1,210,950	1,210,950	1	C -> T		88	SNP (transition)	100.0%	E -> K	37	GAG -> AAG	C770_RS47505	transcriptional regulato	Substitution	4.0E-291	13	1	WP_015243301.1
1,210,965	1,210,965	1	T -> C		94	SNP (transition)	100.0%	N -> D	22	AAC -> GAC	C770_RS47505	transcriptional regulato	Substitution	6.3E-311	8	1	WP_015243301.1
1,211,007	1,211,007	1	G -> A		92	SNP (transition)	100.0%							1.6E-313			
1,211,017	1,211,017	1	C -> A		86	SNP (transversion)	97.7%							9.2E-283			
1,211,028	1,211,028	1	C -> T		83	SNP (transition)	100.0%							2.5E-266			
1,211,036	1,211,036	1	G -> T		81	SNP (transversion)	100.0%							5.0E-268			
1,211,039	1,211,039	1	T -> C		80	SNP (transition)	98.8%							2.5E-275			
1,211,060	1,211,060	1	A -> T		72	SNP (transversion)	98.6%							3.6E-233			
1,211,069	1,211,069	1	C -> T		66	SNP (transition)	100.0%							1.6E-218			
1,211,087	1,211,087	1	G -> A		64	SNP (transition)	98.4%							2.0E-219			
1,211,097	1,211,097	1	T -> C		63	SNP (transition)	100.0%							2.5E-202			
1,211,099	1,211,100	2	TT -> GC		64	Substitution	100.0%							2.5E-218			
1,211,102	1,211,102	1	C -> G		63	SNP (transversion)	100.0%							6.3E-215			
1,211,107	1,211,107	1	G -> C		61	SNP (transversion)	100.0%							5.0E-202			
1,211,129	1,211,129	1	T -> A		48	SNP (transversion)	100.0%							4.0E-159			
1,211,135	1,211,135	1	G -> C		46	SNP (transversion)	100.0%							4.0E-157			
1,211,147	1,211,148	2	GC -> CA		42	Substitution	100.0%							6.3E-131			
1,211,169	1,211,169	1	G -> A		35	SNP (transition)	100.0%							3.2E-109			
1,211,196	1,211,195	0	TTCGATAGG	21 -> 23		Insertion	95.2% -> 95.7%							7.9E-66			
1,211,221	1,211,221	1	C -> G		12	SNP (transversion)	100.0%							4.0E-39			
1,211,228	1,211,228	1	T -> A		9	SNP (transversion)	100.0%							1.6E-29			
1,211,233	1,211,233	1	A -> G		7	SNP (transition)	100.0%							2.5E-20			
1,211,237	1,211,238	2	GA -> AT	9 -> 10		Substitution	100.0%							1.0E-26			
1,211,242	1,211,242	1	A -> C		11	SNP (transversion)	100.0%							6.3E-36			
1,211,245	1,211,245	1	G -> A		12	SNP (transition)	100.0%							4.0E-39			
1,211,251	1,211,251	1	-C		13	Deletion	100.0%							5.0E-15			
1,211,253	1,211,253	1	A -> T		13	SNP (transversion)	100.0%							5.0E-41			
1,211,255	1,211,257	3	AAA -> GGC		14	Substitution	100.0%							6.3E-40			
1,211,270	1,211,270	1	G -> A		18	SNP (transition)	100.0%							4.0E-51			
1,211,292	1,211,293	2	AA -> TC		22	Substitution	100.0%							4.0E-71			
1,211,300	1,211,300	1	T -> C		23	SNP (transition)	100.0%							5.0E-72			
1,211,316	1,211,316	1	C -> T		30	SNP (transition)	100.0%							1.0E-96			
1,211,360	1,211,360	1	T -> A		37	SNP (transversion)	100.0%							1.6E-126			
1,211,381	1,211,381	1	C -> A		41	SNP (transversion)	100.0%							2.5E-148			
1,211,383	1,211,383	1	A -> T		41	SNP (transversion)	100.0%							2.5E-148			
1,211,395	1,211,395	1	T -> CGC	43 -> 45		Insertion	100.0%							6.3E-147			
1,211,443	1,211,443	1	G -> A		60	SNP (transition)	100.0%							1.0E-198			
1,211,476	1,211,476	1	T -> A		67	SNP (transversion)	100.0%							1.6E-228			
1,211,485	1,211,485	1	T -> A		66	SNP (transversion)	100.0%							6.3E-212			
1,211,501	1,211,501	1	A -> G		71	SNP (transition)	100.0%							4.0E-242			
1,211,560	1,211,560	1	T -> C		76	SNP (transition)	100.0%	V -> A	59	GTT -> GCT	C770_RS47510	diguanylate cyclase	Substitution	4.0E-259	20	2	WP_010975384.1
1,211,853	1,211,853	1	C -> T		78	SNP (transition)	100.0%		352	CTG -> TTG	C770_RS47510	diguanylate cyclase	None	1.0E-273	118	1	WP_010975384.1
1,211,906	1,211,906	1	A -> C		80	SNP (transversion)	100.0%		405	TCA -> TCC	C770_RS47510	diguanylate cyclase	None	1.0E-264	135	3	WP_010975384.1
1,212,053	1,212,053	1	A -> G		79	SNP (transition)	100.0%		552	CAA -> CAG	C770_RS47510	diguanylate cyclase	None	2.0E-261	184	3	WP_010975384.1
1,212,128	1,212,129	2	TG -> CA		82	Substitution	100.0%	VV -> VM	627	GTT,GTG -> GTC,	C770_RS47510	diguanylate cyclase	Substitution	1.6E-279	209	3	WP_010975384.1
1,212,158	1,212,158	1	C -> A		83	SNP (transversion)	98.8%		657	CGC -> CGA	C770_RS47510	diguanylate cyclase	None	1.3E-277	219	3	WP_010975384.1
1,212,429	1,212,429	1	C -> T		65	SNP (transition)	100.0%	R -> C	928	CGC -> TGC	C770_RS47510	diguanylate cyclase	Substitution	3.2E-215	310	1	WP_010975384.1
1,212,434	1,212,434	1	G -> A		66	SNP (transition)	100.0%		933	GTG -> GTA	C770_RS47510	diguanylate cyclase	None	1.0E-231	311	3	WP_010975384.1
1,212,453	1,212,453	1	G -> A		70	SNP (transition)	100.0%	V -> I	952	GTA -> ATA	C770_RS47510	diguanylate cyclase	Substitution	1.0E-245	318	1	WP_010975384.1
1,212,564	1,212,564	1	T -> C		71	SNP (transition)	100.0%		1.063	TTA -> CTA	C770_RS47510	diguanylate cyclase	None	1.0E-213	355	1	WP_010975384.1
1,212,663	1,212,663	1	A -> C		76	SNP (transversion)	100.0%	I -> L	1.162	ATC -> CTC	C770_RS47510	diguanylate cyclase	Substitution	1.0E-228	388	1	WP_010975384.1
1,212,668	1,212,668	1	G -> T		74	SNP (transversion)	98.6%		1.167	GCG -> GCT	C770_RS47510	diguanylate cyclase	None	3.7E-225	389	3	WP_010975384.1
1,212,716	1,212,716	1	A -> G		78	SNP (transition)	100.0%							4.0E-258			
1,212,736	1,212,736	1	C -> T		87	SNP (transition)	100.0%							7.9E-288			
1,212,760	1,212,760	1	C -> G		87	SNP (transversion)	100.0%							1.6E-296			
1,212,774	1,212,774	1	T -> C		86	SNP (transition)	100.0%							4.0E-293			
1,212,788	1,212,788	1	A -> G		83	SNP (transition)	100.0%							6.3E-283			
1,212,799	1,212,805	7	GTGGCGC		83	Deletion	100.0%							7.9E-142			
1,212,851	1,212,851	1	C -> T		94	SNP (transition)	98.9%							5.9E-315			
1,212,854	1,212,854	1	A -> G		94	SNP (transition)	100.0%							2.5E-320			
1,212,895	1,212,895	1	T -> A		100	SNP (transversion)	100.0%							0.0			
1,212,908	1,212,908	1	C -> G		100	SNP (transversion)	99.0%							0.0			
1,212,911	1,212,911	1	C -> A		97	SNP (transversion)	100.0%							0.0			
1,212,922	1,212,922	1	C -> G		96	SNP (transversion)	100.0%							0.0			
1,212,955	1,212,955	1	G -> A		102	SNP (transition)	100.0%							0.0			
1,213,100	1,213,099	0	C		105	Insertion	100.0%							0.0			
1,213,117	1,213,117	1	G -> A		103	SNP (transition)	99.0%							0.0			
1,213,148	1,213,148	1	G -> A		104	SNP (transition)	99.0%							0.0			
1,213,203	1,213,203	1	T -> A		104	SNP (transversion)	99.0%		351	ATA -> ATT	C770_RS47515	photosystem reaction c	None	0.0	117	3	WP_010975383.1
1,213,221	1,213,221	1	A -> G		85	SNP (transition)	100.0%		333	GTT -> GTC	C770_RS47515	photosystem reaction c	None	1.0E-289	111	3	WP_010975383.1
1,213,239	1,213,239	1	A -> G		87	SNP (transition)	100.0%		315	CCT -> CCC	C770_RS47515	photosystem reaction c	None	4.0E-279	105	3	WP_010975383.1
1,213,323	1,213,323	1	G -> A		90	SNP (transition)	98.9%		231	CTC -> CTT	C770_RS47515	photosystem reaction c	None	2.3E-301	77	3	WP_010975383.1
1,213,327	1,213,327	1	T -> C		90	SNP (transition)	100.0%	D -> G	227	GAC -> GGC	C770_RS47515	photosystem reaction c	Substitution	1.0E-306	76	2	WP_010975383.1
1,213,329	1,213,329	1	G -> C		90	SNP (transversion)	100.0%		225	ACC -> ACG	C770_RS47515	photosystem reaction c	None	1.0E-306	75	3	WP_010975383.1
1,213,374	1,213,374	1	T -> C		83	SNP (transition)	100.0%		180	GGA -> GGG	C770_RS47515	photosystem reaction c	None	6.3E-283	60	3	WP_010975383.1
1,213,380	1,213,380	1	A -> G		84	SNP (transition)	100.0%		174	GGT -> GGC	C770_RS47515	photosystem reaction c	None	1.0E-294	58	3	WP_010975383.1
1,213,428	1,213,428	1	G -> A		81	SNP (transition)	98.8%		126	CGC -> CGT	C770_RS47515	photosystem reaction c	None	8.1E-279	42	3	WP_010975383.1
1,213,437	1,213,437	1	C -> G		78	SNP (transversion)	100.0%		117	CTG -> CTC	C770_RS47515	photosystem reaction c	None	6.3E-266	39	3	WP_010975383.1
1,213,443	1,213,443	1	G -> A		78	SNP (transition)	100.0%		111	ATC -> ATT	C770_RS47515	photosystem reaction c	None	4.0E-258	37	3	WP_010975383.1
1,213,446	1,213,446	1	T -> C		75	SNP (transition)	100.0%		108	CGA -> CGG	C770_RS47515	photosystem reaction c	None	1.0E-255	36	3	WP_010975383.1
1,213,455	1,213,455	1	A -> G		74	SNP (transition)	100.0%		99	TCT -> TCC	C770_RS47515	photosystem reaction c	None	2.5E-252	33	3	WP_010975383.1
1,213,470	1,213,470	1	C -> G		72	SNP (transversion)	98.6%		84	GGG -> GGC	C770_RS47515	photosystem reaction c	None	2.3E-247	28	3	WP_010975383.1
1,213,491	1,213,491	1	G -> T		65	SNP (transversion)	100.0%		63	ACC -> ACA	C770_RS47515	photosystem reaction c	None	1.0E-221	21	3	WP_010975383.1
1,213,494	1,213,494	1	G -> C		64	SNP (transversion)	98.4%		60	GGC -> GGG	C770_RS47515	photosystem reaction c	None	8.1E-207	20	3	WP_010975383.1
1,213,503	1,213,504	2	TC -> CT		63	Substitution	100.0%	R -> K	50	AGA -> AAG	C770_RS47515	photosystem reaction c	Substitution	6.3E-215	17	2	WP_010975383.1
1,213,506	1,213,506	1	A -> G		62	SNP (transition)	100.0%		48	GAT -> GAC	C770_RS47515	photosystem reaction c	None	1.0E-217	16	3	WP_010975383.1
1,213,527	1,213,527	1	T -> G		53	SNP (transversion)	100.0%		27	TCA -> TCC	C770_RS47515	photosystem reaction c	None	3.2E-186	9	3	WP_010975383.1
1,213,558	1,213,558	1	G -> T		40	SNP (transversion)	100.0%							1.0E-136			
1,213,575	1,213,575	1	G -> A		31	SNP (transition)	100.0%							3.2E-109			
1,213,582	1,213,585	4	CTAA -> AGGG	29 -> 30		Substitution	100.0%							1.3E-90			
1,213,587	1,213,587	1	G -> A		28	SNP (transition)	100.0%							1.0E-98			
1,213,589	1,213,589	1	G -> T		28	SNP (transversion)	100.0%							1.0E-98			
1,213,592	1,213,592	1	G -> C		26	SNP (transversion)	100.0%							1.6E-86			
1,213,594	1,213,594	1	C -> A		26	SNP (transversion)	100.0%							4.0E-89			
1,213,616	1,213,616	1	G -> T		18	SNP (transversion)	100.0%							1.6E-65			
1,213,622	1,213,622	1	C -> T		18	SNP (transition)	100.0%							4.0E-60			
1,213,635	1,213,635	1	A -> G		17	SNP (transition)	100.0%							1.6E-58			
1,213,697	1,213,697	1	A -> G		13	SNP (transition)	100.0%							4.0E-37			
1,213,702	1,213,702	1	T -> G		13	SNP (transversion)	100.0%							2.0E-38			
1,213,730	1,213,730	1	A -> G		19	SNP (transition)	100.0%							1.6E-61			
1,213,811	1,213,811	1	C -> G		41	SNP (transversion)	97.6%							4.1E-127			
1,213,975	1,213,975	1	A -> G		76	SNP (transition)	100.0%		897	AGT -> AGC	C770_RS47520	FMN reductase	None	1.6E-251	299	3	WP_041169900.1
1,214,096	1,214,096	1	C -> T		73	SNP (transition)	95.9%	G -> E	776	GGG -> GAG	C770_RS47520	FMN reductase	Substitution	6.2E-220	259	2	WP_041169900.1
1,214,221	1,214,221	1	A -> G		75	SNP (transition)	100.0%		651	AAT -> AAC	C770_RS47520	FMN reductase	None	1.0E-240	217	3	WP_041169900.1
1,214,233	1,214,233	1	G -> A		74	SNP (transition)	98.6%		639	GCC -> GCT	C770_RS47520	FMN reductase	None	2.3E-254	213	3	WP_041169900.1
1,214,269	1,214,269	1	C -> G		67	SNP (transversion)	100.0%		603	TCG -> TCC	C770_RS47520	FMN reductase	None	7.9E-222	201	3	WP_041169900.1
1,214,332	1,214,332	1	G -> C		62	SNP (transversion)	100.0%		540	CCC -> CCG	C770_RS47520	FMN reductase	None	1.6E-211	180	3	WP_041169900.1
1,214,386	1,214,386	1	A -> G		67	SNP (transition)	100.0%		486	TGT -> TGC	C770_RS47520	FMN reductase	None	3.2E-235	162	3	WP_041169900.1
1,214,443	1,214,443	1	A -> G		66	SNP (transition)	100.0%		429	CAT -> CAC	C770_RS47520	FMN reductase	None	1.6E-218	143	3	WP_041169900.1
1,214,511	1,214,511	1	T -> C		65	SNP (transition)	100.0%	T -> A	361	ACG -> GCG	C770_RS47520	FMN reductase	Substitution	1.0E-221	121	1	WP_041169900.1
1,214,535	1,214,535	1	T -> C		67	SNP (transition)	100.0%	I -> V	337	ATC -> GTC	C770_RS47520	FMN reductase	Substitution	7.9E-222	113	1	WP_041169900.1
1,214,807	1,214,808	2	CA -> AG	66 -> 67		Substitution	100.0%	C -> L	64	TGT -> CTT	C770_RS47520	FMN reductase	Substitution	2.0E-208	22	1	WP_041169900.1
1,214,837	1,214,837	1	T -> C		59	SNP (transition)	98.3%	N -> S	35	AAC -> AGC	C770_RS47520	FMN reductase	Substitution	3.7E-196	12	2	WP_041169900.1
1,214,840	1,214,840	1	A -> T		57	SNP (transversion)	100.0%	L -> H	32	CTC -> CAC	C770_RS47520	FMN reductase	Substitution	1.6E-194	11	2	WP_041169900.1
1,214,848	1,214,848	1	A -> G		57	SNP (transition)	100.0%		24	CGT -> CGC	C770_RS47520	FMN reductase	None	4.0E-183	8	3	WP_041169900.1
1,214,924	1,214,924	1	A -> G		35	SNP (transition)	100.0%							1.0E-112			
1,214,964	1,214,964	1	G -> T		23	SNP (transversion)	100.0%							5.0E-72			
1,214,976	1,214,976	1	G -> T		22	SNP (transversion)	100.0%							1.6E-75			
1,214,993	1,214,993	1	A -> C		18	SNP (transversion)	100.0%							4.0E-60			
1,215,012	1,215,015	4	GGGG -> TTTT		11	Substitution	100.0%							1.0E-33			
1,215,017	1,215,027	11	GAACTGATGAT -	9 -> 11		Substitution	100.0%							6.3E-26			
1,215,029	1,215,029	1	T -> AG	8 -> 9		Insertion	100.0%							2.5E-22			
1,215,034	1,215,035	2	GA -> T		8	Deletion	100.0%							1.0E-12			
1,215,039	1,215,039	1	C -> A		8	SNP (transversion)	100.0%							1.0E-24			
1,215,041	1,215,042	2	CA -> GT		6	Substitution	100.0%							2.5E-22			
1,219,757	1,219,759	3	CGT -> TCC		6	Substitution	100.0%							1.0E-15			
1,219,773	1,219,773	1	C -> A		12	SNP (transversion)	100.0%							1.0E-42			
1,219,800	1,219,800	1	G -> A		20	SNP (transition)	100.0%							1.0E-62			
1,219,805	1,219,804	0	T		21	Insertion	100.0%							6.3E-68			
1,219,821	1,219,821	1	G -> A		27	SNP (transition)	100.0%							1.6E-92			
1,219,854	1,219,854	1	T -> G		36	SNP (transversion)	97.2%							3.6E-111			
1,219,860	1,219,860	1	(T)3 -> (T)2		37	Deletion (tandem repeat)	100.0%							1.6E-52			
1,219,864	1,219,865	2	TC -> GT		39	Substitution	100.0%							2.0E-129			
1,219,873	1,219,873	1	G -> C		42	SNP (transversion)	100.0%							2.5E-139			
1,219,882	1,219,882	1	C -> T		42	SNP (transition)	100.0%							4.0E-135			
1,219,884	1,219,883	0	A		43	Insertion	100.0%							3.2E-151			
1,219,896	1,219,899	4	GATC -> A		46	Deletion	100.0%							2.5E-74			
1,219,905	1,219,905	1	C -> A		48	SNP (transversion)	100.0%							6.3E-164			
1,219,910	1,219,909	0	(G)2 -> (G)3		48	Insertion (tandem repeat)	100.0%							1.6E-149			
1,219,911	1,219,911	1	A -> T		49	SNP (transversion)	98.0%							1.2E-152			
1,219,914	1,219,914	1	G -> T		52	SNP (transversion)	98.1%							1.6E-177			
1,219,917	1,219,917	1	C -> T		52	SNP (transition)	98.1%							2.6E-167			
1,219,919	1,219,919	1	T -> C		53	SNP (transition)	100.0%							2.5E-170			
1,219,926	1,219,926	1	C -> T		55	SNP (transition)	100.0%							1.0E-165			
1,219,964	1,219,965	2	GG -> AA		71	Substitution	98.6%							1.0E-213			
1,219,970	1,219,970	1	A -> T		73	SNP (transversion)	98.6%							7.3E-215			
1,219,990	1,219,990	1	T -> C		77	SNP (transition)	100.0%							1.6E-262			
1,219,994	1,219,995	2	AA -> GG		79	Substitution	100.0%							1.3E-245			

1,219,999	1,219,998	0	A		79	Insertion	98.7%							3.1E-256			
1,220,004	1,220,004	1	C -> T		81	SNP (transition)	98.8%							8.1E-271			
1,220,008	1,220,008	1	G -> T		80	SNP (transversion)	100.0%							1.0E-264			
1,220,017	1,220,017	1	T -> C		82	SNP (transition)	98.8%							3.3E-274			
1,220,027	1,220,027	1	A -> G		83	SNP (transition)	100.0%							1.3E-274			
1,220,037	1,220,037	1	C -> T		81	SNP (transition)	100.0%							5.0E-268			
1,220,039	1,220,039	1	T -> C		81	SNP (transition)	100.0%							5.0E-268			
1,220,053	1,220,053	1	A -> G		88	SNP (transition)	100.0%							2.5E-282			
1,220,067	1,220,067	1	T -> C		83	SNP (transition)	100.0%							1.3E-274			
1,220,074	1,220,074	1	T -> G		80	SNP (transversion)	97.5%							7.9E-247			
1,220,091	1,220,091	1	C -> G		77	SNP (transversion)	98.7%							7.7E-265			
1,220,150	1,220,150	1	A -> C		79	SNP (transversion)	98.7%							7.9E-272			
1,220,255	1,220,255	1	G -> A		79	SNP (transition)	100.0%							2.0E-261			
1,220,257	1,220,256	0	A		79	Insertion	98.7%							3.1E-256			
1,220,300	1,220,300	1	G -> TC		81	Insertion	97.5%							8.1E-266			
1,220,304	1,220,306	3	TCG		81	Deletion	100.0%							2.5E-130			
1,220,318	1,220,318	1	C -> T		80	SNP (transition)	100.0%							1.0E-272			
1,220,326	1,220,326	1	C -> T		79	SNP (transition)	100.0%							1.6E-253			
1,220,332	1,220,331	0	(G)2 -> (G)3		74	Insertion (tandem repeat)	100.0%							1.6E-237			
1,220,339	1,220,339	1	G -> A		72	SNP (transition)	100.0%							2.5E-238			
1,220,354	1,220,354	1	C -> A		76	SNP (transversion)	100.0%							1.0E-266			
1,220,362	1,220,362	1	-C		72	Deletion	100.0%							6.3E-116			
1,220,371	1,220,371	1	C -> T		73	SNP (transition)	100.0%							6.3E-249			
1,220,374	1,220,374	1	A -> G		73	SNP (transition)	100.0%							3.2E-256			
1,220,396	1,220,396	1	C -> G		74	SNP (transversion)	100.0%							4.0E-267			
1,220,400	1,220,400	1	C -> G		74	SNP (transversion)	100.0%							1.0E-259			
1,220,407	1,220,407	1	T -> G		71	SNP (transversion)	100.0%							3.2E-249			
1,220,417	1,220,417	1	A -> G		70	SNP (transition)	100.0%							1.0E-245			
1,220,437	1,220,437	1	T -> C		67	SNP (transition)	100.0%							3.2E-235			
1,220,442	1,220,442	1	G -> A		67	SNP (transition)	100.0%							1.6E-228			
1,220,450	1,220,450	1	A -> G		66	SNP (transition)	100.0%							6.3E-212			
1,220,458	1,220,458	1	G -> A		64	SNP (transition)	98.4%	S -> L	407	TCG -> TTG	C770_RS47550	chemotaxis protein Che	Substitution	2.0E-219	136	2	WP_015243308.1
1,220,466	1,220,466	1	C -> A		63	SNP (transversion)	100.0%		399	CCG -> CCT	C770_RS47550	chemotaxis protein Che	None	1.3E-208	133	3	WP_015243308.1
1,220,475	1,220,475	1	A -> G		62	SNP (transition)	100.0%		390	AAT -> AAC	C770_RS47550	chemotaxis protein Che	None	2.5E-205	130	3	WP_015243308.1
1,220,481	1,220,481	1	T -> G		63	SNP (transversion)	100.0%		384	GCA -> GCC	C770_RS47550	chemotaxis protein Che	None	1.3E-208	128	3	WP_015243308.1
1,220,484	1,220,485	2	CG -> TA		63	Substitution	100.0%	T -> I	380	ACG -> ATA	C770_RS47550	chemotaxis protein Che	Substitution	3.2E-221	127	2	WP_015243308.1
1,220,571	1,220,571	1	C -> G		45	SNP (transversion)	100.0%	E -> D	294	GAG -> GAC	C770_RS47550	chemotaxis protein Che	Substitution	3.2E-158	98	3	WP_015243308.1
1,220,618	1,220,618	1	G -> T		46	SNP (transversion)	100.0%		247	CGG -> AGG	C770_RS47550	chemotaxis protein Che	None	1.6E-152	83	1	WP_015243308.1
1,220,621	1,220,621	1	G -> C		46	SNP (transversion)	100.0%	Q -> E	244	CAG -> GAG	C770_RS47550	chemotaxis protein Che	Substitution	2.5E-166	82	1	WP_015243308.1
1,220,637	1,220,637	1	A -> G		47	SNP (transition)	100.0%		228	GTT -> GTC	C770_RS47550	chemotaxis protein Che	None	6.3E-170	76	3	WP_015243308.1
1,220,654	1,220,654	1	C -> T		46	SNP (transition)	97.8%	A -> T	211	GCA -> ACA	C770_RS47550	chemotaxis protein Che	Substitution	1.5E-156	71	1	WP_015243308.1
1,220,676	1,220,676	1	A -> C		41	SNP (transversion)	97.6%		189	GTT -> GTG	C770_RS47550	chemotaxis protein Che	None	4.1E-135	63	3	WP_015243308.1
1,220,688	1,220,688	1	A -> C		37	SNP (transversion)	100.0%		177	CTT -> CTG	C770_RS47550	chemotaxis protein Che	None	1.6E-126	59	3	WP_015243308.1
1,220,709	1,220,710	2	TG -> CA		37	Substitution	100.0%	A -> V	155	GCA -> GTG	C770_RS47550	chemotaxis protein Che	Substitution	6.3E-134	52	2	WP_015243308.1
1,220,715	1,220,715	1	C -> T		37	SNP (transition)	100.0%		150	GCG -> GCA	C770_RS47550	chemotaxis protein Che	None	6.3E-134	50	3	WP_015243308.1
1,220,718	1,220,718	1	C -> G		37	SNP (transversion)	100.0%		147	GCG -> GCC	C770_RS47550	chemotaxis protein Che	None	3.2E-130	49	3	WP_015243308.1
1,220,721	1,220,721	1	C -> T		35	SNP (transition)	100.0%		144	GAG -> GAA	C770_RS47550	chemotaxis protein Che	None	3.2E-123	48	3	WP_015243308.1
1,220,731	1,220,731	1	C -> G		37	SNP (transversion)	100.0%	G -> A	134	GGG -> GCG	C770_RS47550	chemotaxis protein Che	Substitution	6.3E-134	45	2	WP_015243308.1
1,220,739	1,220,739	1	T -> C		38	SNP (transition)	100.0%		126	GGA -> GGG	C770_RS47550	chemotaxis protein Che	None	4.0E-126	42	3	WP_015243308.1
1,220,745	1,220,745	1	G -> C		41	SNP (transversion)	97.6%		120	GTC -> GTG	C770_RS47550	chemotaxis protein Che	None	4.1E-131	40	3	WP_015243308.1
1,220,811	1,220,811	1	T -> C		51	SNP (transition)	100.0%		54	GTA -> GTG	C770_RS47550	chemotaxis protein Che	None	4.0E-174	18	3	WP_015243308.1
1,220,814	1,220,814	1	C -> G		51	SNP (transversion)	98.0%		51	CTG -> CTC	C770_RS47550	chemotaxis protein Che	None	5.1E-174	17	3	WP_015243308.1
1,220,817	1,220,817	1	A -> G		52	SNP (transition)	100.0%		48	GTT -> GTC	C770_RS47550	chemotaxis protein Che	None	4.0E-167	16	3	WP_015243308.1
1,220,820	1,220,820	1	T -> C		52	SNP (transition)	100.0%		45	AGA -> AGG	C770_RS47550	chemotaxis protein Che	None	1.0E-182	15	3	WP_015243308.1
1,220,823	1,220,824	2	GA -> AG	55 -> 56		Substitution	100.0%	V -> A	41	GTC -> GCT	C770_RS47550	chemotaxis protein Che	Substitution	3.2E-193	14	2	WP_015243308.1
1,220,845	1,220,845	1	T -> A		59	SNP (transversion)	100.0%	D -> V	20	GAC -> GTC	C770_RS47550	chemotaxis protein Che	Substitution	2.5E-201	7	2	WP_015243308.1
1,220,847	1,220,847	1	G -> T		59	SNP (transversion)	100.0%	D -> E	18	GAC -> GAA	C770_RS47550	chemotaxis protein Che	Substitution	2.5E-201	6	3	WP_015243308.1
1,220,851	1,220,851	1	G -> C		60	SNP (transversion)	100.0%	T -> S	14	ACT -> AGT	C770_RS47550	chemotaxis protein Che	Substitution	1.0E-210	5	2	WP_015243308.1
1,220,851	1,220,851	1	G -> C		60	SNP (transversion)	100.0%	N -> K	3.483	AAC -> AAG	C770_RS47555	sensor histidine kinase	Substitution	1.0E-210	1.161	3	WP_015243309.1
1,220,870	1,220,870	1	C -> G		61	SNP (transversion)	98.4%	R -> P	3.464	CGG -> CCG	C770_RS47555	sensor histidine kinase	Substitution	6.1E-191	1.155	2	WP_015243309.1
1,220,893	1,220,893	1	G -> A		66	SNP (transition)	100.0%		3.441	ATC -> ATT	C770_RS47555	sensor histidine kinase	None	1.6E-218	1.147	3	WP_015243309.1
1,221,031	1,221,032	2	CC -> TT		84	Substitution	100.0%	G -> E	3.302	GGG -> GAA	C770_RS47555	sensor histidine kinase	Substitution	4.0E-261	1.101	2	WP_015243309.1
1,221,037	1,221,038	2	CC -> GT	82 -> 83		Substitution	98.8%	R -> N	3.296	AGG -> AAC	C770_RS47555	sensor histidine kinase	Substitution	3.3E-261	1.099	2	WP_015243309.1
1,221,040	1,221,040	1	T -> C		81	SNP (transition)	100.0%		3.294	CGA -> CGG	C770_RS47555	sensor histidine kinase	None	6.3E-260	1.098	3	WP_015243309.1
1,221,064	1,221,064	1	G -> A		82	SNP (transition)	100.0%		3.27	AGC -> AGT	C770_RS47555	sensor histidine kinase	None	2.5E-271	1.09	3	WP_015243309.1
1,221,075	1,221,075	1	T -> C		80	SNP (transition)	100.0%	I -> V	3.259	ATC -> GTC	C770_RS47555	sensor histidine kinase	Substitution	1.0E-264	1.087	1	WP_015243309.1
1,221,083	1,221,083	1	T -> A		77	SNP (transversion)	100.0%	N -> I	3.251	AAC -> ATC	C770_RS47555	sensor histidine kinase	Substitution	7.9E-255	1.084	2	WP_015243309.1
1,221,103	1,221,103	1	G -> A		77	SNP (transition)	98.7%		3.231	TAC -> TAT	C770_RS47555	sensor histidine kinase	None	1.2E-249	1.077	3	WP_015243309.1
1,221,112	1,221,112	1	A -> G		78	SNP (transition)	100.0%		3.222	GCT -> GCC	C770_RS47555	sensor histidine kinase	None	6.3E-266	1.074	3	WP_015243309.1
1,221,145	1,221,145	1	A -> G		85	SNP (transition)	100.0%		3.189	TTT -> TTC	C770_RS47555	sensor histidine kinase	None	1.0E-289	1.063	3	WP_015243309.1
1,221,148	1,221,148	1	G -> C		86	SNP (transversion)	98.8%		3.186	CCC -> CCG	C770_RS47555	sensor histidine kinase	None	8.6E-288	1.062	3	WP_015243309.1
1,221,163	1,221,163	1	C -> T		86	SNP (transition)	100.0%		3.171	CCG -> CCA	C770_RS47555	sensor histidine kinase	None	1.6E-284	1.057	3	WP_015243309.1
1,221,181	1,221,181	1	T -> C		80	SNP (transition)	98.8%		3.153	GAA -> GAG	C770_RS47555	sensor histidine kinase	None	2.5E-275	1.051	3	WP_015243309.1
1,221,184	1,221,184	1	G -> C		80	SNP (transversion)	100.0%		3.15	GGC -> GGG	C770_RS47555	sensor histidine kinase	None	1.0E-256	1.05	3	WP_015243309.1
1,221,203	1,221,203	1	C -> T		81	SNP (transition)	97.5%	R -> H	3.131	CGC -> CAC	C770_RS47555	sensor histidine kinase	Substitution	8.1E-266	1.044	2	WP_015243309.1
1,221,229	1,221,229	1	A -> G		83	SNP (transition)	100.0%		3.105	CTT -> CTC	C770_RS47555	sensor histidine kinase	None	6.3E-283	1.035	3	WP_015243309.1
1,221,246	1,221,246	1	A -> G		80	SNP (transition)	100.0%		3.088	TTG -> CTG	C770_RS47555	sensor histidine kinase	None	1.0E-272	1.03	1	WP_015243309.1
1,221,313	1,221,313	1	G -> A		96	SNP (transition)	100.0%		3.021	CTC -> CTT	C770_RS47555	sensor histidine kinase	None	6.3E-308	1.007	3	WP_015243309.1
1,221,331	1,221,331	1	T -> C		95	SNP (transition)	100.0%		3.003	GAA -> GAG	C770_RS47555	sensor histidine kinase	None	1.0E-323	1.001	3	WP_015243309.1
1,221,370	1,221,370	1	T -> C		97	SNP (transition)	100.0%		2.964	CAA -> CAG	C770_RS47555	sensor histidine kinase	None	0.0	988	3	WP_015243309.1
1,221,412	1,221,412	1	G -> C		95	SNP (transversion)	100.0%		2.922	CGC -> CGG	C770_RS47555	sensor histidine kinase	None	3.2E-314	974	3	WP_015243309.1
1,221,415	1,221,415	1	G -> A		95	SNP (transition)	98.9%		2.919	CAC -> CAT	C770_RS47555	sensor histidine kinase	None	1.5E-299	973	3	WP_015243309.1
1,221,430	1,221,430	1	T -> C		85	SNP (transition)	100.0%		2.904	CTA -> CTG	C770_RS47555	sensor histidine kinase	None	3.2E-298	968	3	WP_015243309.1
1,221,436	1,221,436	1	A -> G		84	SNP (transition)	98.8%		2.898	CTT -> CTC	C770_RS47555	sensor histidine kinase	None	5.3E-281	966	3	WP_015243309.1
1,221,457	1,221,457	1	C -> G		91	SNP (transversion)	100.0%		2.877	CGG -> CGC	C770_RS47555	sensor histidine kinase	None	4.0E-310	959	3	WP_015243309.1
1,221,471	1,221,472	2	TG -> AA	91 -> 93		Substitution	100.0%	IT -> IS	2.862	ATC,ACC -> ATT,'	C770_RS47555	sensor histidine kinase	Substitution	7.9E-283	954	3	WP_015243309.1
1,221,499	1,221,499	1	G -> C		95	SNP (transversion)	100.0%		2.835	CGC -> CGG	C770_RS47555	sensor histidine kinase	None	1.0E-304	945	3	WP_015243309.1
1,221,600	1,221,600	1	C -> T		86	SNP (transition)	100.0%	E -> K	2.734	GAA -> AAA	C770_RS47555	sensor histidine kinase	Substitution	1.0E-301	912	1	WP_015243309.1
1,221,620	1,221,620	1	C -> T		84	SNP (transition)	100.0%	R -> K	2.714	AGG -> AAG	C770_RS47555	sensor histidine kinase	Substitution	1.0E-294	905	2	WP_015243309.1
1,221,631	1,221,631	1	G -> C		87	SNP (transversion)	100.0%		2.703	TCC -> TCG	C770_RS47555	sensor histidine kinase	None	1.6E-296	901	3	WP_015243309.1
1,221,643	1,221,643	1	G -> T		89	SNP (transversion)	100.0%		2.691	GCC -> GCA	C770_RS47555	sensor histidine kinase	None	4.0E-321	897	3	WP_015243309.1
1,221,678	1,221,678	1	T -> G		93	SNP (transversion)	100.0%	I -> L	2.656	ATT -> CTT	C770_RS47555	sensor histidine kinase	Substitution	6.3E-317	886	1	WP_015243309.1
1,221,688	1,221,688	1	G -> A		91	SNP (transition)	97.8%		2.646	CGC -> CGT	C770_RS47555	sensor histidine kinase	None	8.2E-291	882	3	WP_015243309.1
1,221,721	1,221,721	1	A -> G		102	SNP (transition)	99.0%		2.613	AAT -> AAC	C770_RS47555	sensor histidine kinase	None	0.0	871	3	WP_015243309.1
1,221,754	1,221,754	1	T -> C		98	SNP (transition)	99.0%		2.58	GTA -> GTG	C770_RS47555	sensor histidine kinase	None	0.0	860	3	WP_015243309.1
1,221,809	1,221,809	1	C -> T		101	SNP (transition)	99.0%	R -> Q	2.525	CGA -> CAA	C770_RS47555	sensor histidine kinase	Substitution	0.0	842	2	WP_015243309.1
1,221,874	1,221,874	1	A -> G		99	SNP (transition)	100.0%		2.46	GTT -> GTC	C770_RS47555	sensor histidine kinase	None	0.0	820	3	WP_015243309.1
1,221,880	1,221,880	1	G -> A		101	SNP (transition)	100.0%		2.454	GAC -> GAT	C770_RS47555	sensor histidine kinase	None	0.0	818	3	WP_015243309.1
1,221,897	1,221,898	2	CT -> TC	105 -> 106		Substitution	99.0% -> 99.1%	TV -> TM	2.436	ACA,GTG -> ACG	C770_RS47555	sensor histidine kinase	Substitution	0.0	812	3	WP_015243309.1
1,221,925	1,221,925	1	A -> G		111	SNP (transition)	100.0%		2.409	TAT -> TAC	C770_RS47555	sensor histidine kinase	None	0.0	803	3	WP_015243309.1
1,221,934	1,221,934	1	G -> A		114	SNP (transition)	99.1%		2.4	GAC -> GAT	C770_RS47555	sensor histidine kinase	None	0.0	800	3	WP_015243309.1
1,221,958	1,221,958	1	C -> T		114	SNP (transition)	100.0%		2.376	GAG -> GAA	C770_RS47555	sensor histidine kinase	None	0.0	792	3	WP_015243309.1
1,221,963	1,221,963	1	G -> C		113	SNP (transversion)	100.0%	L -> V	2.371	CTC -> GTC	C770_RS47555	sensor histidine kinase	Substitution	0.0	791	1	WP_015243309.1
1,221,982	1,221,982	1	C -> T		110	SNP (transition)	100.0%		2.352	GTG -> GTA	C770_RS47555	sensor histidine kinase	None	0.0	784	3	WP_015243309.1
1,221,997	1,221,997	1	A -> C		105	SNP (transversion)	100.0%	D -> E	2.337	GAT -> GAG	C770_RS47555	sensor histidine kinase	Substitution	0.0	779	3	WP_015243309.1
1,222,023	1,222,023	1	A -> G		100	SNP (transition)	99.0%	Y -> H	2.311	TAT -> CAT	C770_RS47555	sensor histidine kinase	Substitution	0.0	771	1	WP_015243309.1
1,222,048	1,222,048	1	A -> G		96	SNP (transition)	100.0%		2.286	GGT -> GGC	C770_RS47555	sensor histidine kinase	None	0.0	762	3	WP_015243309.1
1,222,132	1,222,132	1	C -> T		89	SNP (transition)	98.9%		2.202	ACG -> ACA	C770_RS47555	sensor histidine kinase	None	8.9E-307	734	3	WP_015243309.1
1,222,312	1,222,312	1	T -> C		80	SNP (transition)	100.0%		2.022	CTA -> CTG	C770_RS47555	sensor histidine kinase	None	1.0E-272	674	3	WP_015243309.1
1,222,335	1,222,336	2	GT -> AC		85	Substitution	98.8%		1.998	GAA,CTG -> GAG	C770_RS47555	sensor histidine kinase	None	8.5E-293	666	3	WP_015243309.1
1,222,342	1,222,342	1	G -> A		85	SNP (transition)	97.6%		1.992	ACC -> ACT	C770_RS47555	sensor histidine kinase	None	1.1E-287	664	3	WP_015243309.1
1,222,345	1,222,345	1	G -> A		84	SNP (transition)	100.0%		1.989	GCC -> GCT	C770_RS47555	sensor histidine kinase	None	2.5E-286	663	3	WP_015243309.1
1,222,348	1,222,348	1	G -> A		82	SNP (transition)	98.8%		1.986	ACC -> ACT	C770_RS47555	sensor histidine kinase	None	2.6E-282	662	3	WP_015243309.1
1,222,362	1,222,363	2	CA -> GG	82 -> 83		Substitution	100.0%	SE -> SQ	1.971	AGT,GAG -> AGC	C770_RS47555	sensor histidine kinase	Substitution	6.3E-283	657	3	WP_015243309.1
1,222,366	1,222,366	1	G -> C		83	SNP (transversion)	100.0%		1.968	ACC -> ACG	C770_RS47555	sensor histidine kinase	None	1.6E-299	656	3	WP_015243309.1
1,222,372	1,222,372	1	T -> G		84	SNP (transversion)	100.0%		1.962	CGA -> CGC	C770_RS47555	sensor histidine kinase	None	2.5E-286	654	3	WP_015243309.1
1,222,378	1,222,378	1	A -> C		81	SNP (transversion)	100.0%		1.956	CGT -> CGG	C770_RS47555	sensor histidine kinase	None	4.0E-276	652	3	WP_015243309.1
1,222,393	1,222,393	1	T -> C		82	SNP (transition)	100.0%		1.941	CAA -> CAG	C770_RS47555	sensor histidine kinase	None	1.0E-287	647	3	WP_015243309.1
1,222,420	1,222,420	1	C -> T		83	SNP (transition)	100.0%		1.914	GCG -> GCA	C770_RS47555	sensor histidine kinase	None	1.3E-274	638	3	WP_015243309.1
1,222,435	1,222,435	1	C -> T		83	SNP (transition)	100.0%		1.899	CCG -> CCA	C770_RS47555	sensor histidine kinase	None	1.3E-274	633	3	WP_015243309.1
1,222,445	1,222,445	1	T -> C		86	SNP (transition)	100.0%	D -> G	1.889	GAC -> GGC	C770_RS47555	sensor histidine kinase	Substitution	1.6E-284	630	2	WP_015243309.1
1,222,450	1,222,450	1	A -> C		85	SNP (transversion)	100.0%		1.884	ACT -> ACG	C770_RS47555	sensor histidine kinase	None	1.0E-289	628	3	WP_015243309.1
1,222,462	1,222,462	1	T -> G		88	SNP (transversion)	100.0%		1.872	GTA -> GTC	C770_RS47555	sensor histidine kinase	None	4.0E-291	624	3	WP_015243309.1
1,222,483	1,222,483	1	T -> G		85	SNP (transversion)	98.8%		1.851	GCA -> GCC	C770_RS47555	sensor histidine kinase	None	5.4E-276	617	3	WP_015243309.1
1,222,486	1,222,486	1	C -> T		84	SNP (transition)	100.0%		1.848	GAG -> GAA	C770_RS47555	sensor histidine kinase	None	1.6E-269	616	3	WP_015243309.1
1,222,498	1,222,498	1	A -> G		82	SNP (transition)	100.0%		1.836	TTT -> TTC	C770_RS47555	sensor histidine kinase	None	1.6E-279	612	3	WP_015243309.1
1,222,523	1,222,523	1	C -> G		81	SNP (transversion)	100.0%	R -> P	1.811	CGC -> CCC	C770_RS47555	sensor histidine kinase	Substitution	4.0E-276	604	2	WP_015243309.1
1,222,531	1,222,531	1	T -> C		79	SNP (transition)	100.0%		1.803	GGA -> GGG	C770_RS47555	sensor histidine kinase	None	2.5E-269	601	3	WP_015243309.1
1,222,576	1,222,576	1	C -> T		85	SNP (transition)	100.0%		1.758	GGG -> GGA	C770_RS47555	sensor histidine kinase	None	1.0E-289	586	3	WP_015243309.1
1,222,582	1,222,582	1	A -> C		85	SNP (transversion)	100.0%		1.752	CTT -> CTG	C770_RS47555	sensor histidine kinase	None	3.2E-281	584	3	WP_015243309.1
1,222,609	1,222,609	1	A -> C		84	SNP (transversion)	100.0%		1.725	ACT -> ACG	C770_RS47555	sensor histidine kinase	None	1.0E-294	575	3	WP_015243309.1
1,222,618	1,222,620	2	ATC -> GTT	83 -> 85		Substitution	100.0%	D -> N	1.714	GAT -> AAC	C770_RS47555	sensor histidine kinase	Substitution	1.0E-289	572	1	WP_015243309.1
1,222,623	1,222,623	1	T -> G		87	SNP (transversion)	100.0%		1.711	AGG -> CGG	C770_RS47555	sensor histidine kinase	None	1.6E-296	571	1	WP_015243309.1
1,222,653	1,222,653	1	C -> G		83	SNP (transversion)	100.0%	V -> L	1.681	GTA -> CTA	C770_RS47555	sensor histidine kinase	Substitution	3.2E-291	561	1	WP_015243309.1
1,222,684	1,222,684	1	C -> G		77	SNP (transversion)	100.0%		1.65	ACG -> ACC	C770_RS47555	sensor histidine kinase	None	1.6E-262	550	3	WP_015243309.1
1,222,693	1,222,693	1	G -> C		81	SNP (transversion)	100.0%		1.641	ACC -> ACG	C770_RS47555	sensor histidine kinase	None	4.0E-276	547	3	WP_015243309.1
1,222,718	1,222,718	1	C -> T		81	SNP (transition)	100.0%	R -> Q	1.616	CGA -> CAA	C770_RS47555	sensor histidine kinase	Substitution	3.2E-284	539	2	WP_015243309.1
1,222,732	1,222,732	1	G -> A		83	SNP (transition)	100.0%		1.602	GCC -> GCT	C770_RS47555	sensor histidine kinase	None	1.6E-299	534	3	WP_015243309.1
1,222,735	1,222,735	1	G -> A		82	SNP (transition)	98.8%		1.599	AAC -> AAT	C770_RS47555	sensor histidine kinase	None	2.6E-282	533	3	WP_015243309.1
1,222,750	1,222,750	1	A -> C		81	SNP (transversion)	100.0%	I -> M	1.584	ATT -> ATG	C770_RS47555	sensor histidine kinase	Substitution	3.2E-284	528	3	WP_015243309.1
1,222,783	1,222,783	1	T -> G		84	SNP (transversion)	100.0%		1.551	CCA -> CCC	C770_RS47555	sensor histidine kinase	None	6.3E-278	517	3	WP_015243309.1
1,222,789	1,222,789	1	A -> C		85	SNP (transversion)	100.0%		1.545	GCT -> GCG	C770_RS47555	sensor histidine kinase	None	3.2E-298	515	3	WP_015243309.1
1,222,801	1,222,801	1	C -> G		82	SNP (transversion)	100.0%		1.533	CTG -> CTC	C770_RS47555	sensor histidine kinase	None	1.0E-287	511	3	WP_015243309.1
1,222,822	1,222,822	1	T -> C		81	SNP (transition)	100.0%		1.512	GCA -> GCG	C770_RS47555	sensor histidine kinase	None	5.0E-268	504	3	WP_015243309.1
1,222,846	1,222,846	1	T -> C		74	SNP (transition)	100.0%		1.488	CCA -> CCG	C770_RS47555	sensor histidine kinase	None	2.5E-252	496	3	WP_015243309.1
1,222,849	1,222,849	1	T -> C		75	SNP (transition)	100.0%		1.485	ACA -> ACG	C770_RS47555	sensor histidine kinase	None	1.0E-255	495	3	WP_015243309.1
1,222,879	1,222,884	5	CTTTTG -> GCCA'	76 -> 77		Substitution	96.1%	QK -> NG	1.45	CAA,AAG -> AAT,	C770_RS47555	sensor histidine kinase	Substitution	1.6E-237	484	1	WP_015243309.1
1,222,909	1,222,912	3	CATT -> TACA	74 -> 75		Substitution	100.0%	PM -> PV	1.422	CCA,ATG -> CCT,	C770_RS47555	sensor histidine kinase	Substitution	1.0E-240	474	3	WP_015243309.1
1,222,918	1,222,920	3	TCG		77	Deletion	100.0%	R ->	1.414	CGA ->	C770_RS47555	sensor histidine kinase	Deletion	3.2E-116	472	1	WP_015243309.1
1,222,933	1,222,933	1	C -> A		76	SNP (transversion)	100.0%		1.401	GCG -> GCT	C770_RS47555	sensor histidine kinase	None	4.0E-259	467	3	WP_015243309.1
1,222,938	1,222,938	1	C -> T		80	SNP (transition)	98.8%	V -> I	1.396	GTC -> ATC	C770_RS47555	sensor histidine kinase	Substitution	1.0E-243	466	1	WP_015243309.1
1,222,981	1,222,981	1	C -> T		84	SNP (transition)	100.0%		1.353	GGG -> GGA	C770_RS47555	sensor histidine kinase	None	6.3E-278	451	3	WP_015243309.1
1,222,989	1,222,989	1	G -> A		84	SNP (transition)	100.0%	H -> Y	1.345	CAC -> TAC	C770_RS47555	sensor histidine kinase	Substitution	6.3E-278	449	1	WP_015243309.1
1,222,993	1,222,993	1	G -> A		84	SNP (transition)	100.0%		1.341	GCC -> GCT	C770_RS47555	sensor histidine kinase	None	2.5E-286	447	3	WP_015243309.1
1,223,035	1,223,035	1	G -> C		82	SNP (transversion)	100.0%		1.299	CTC -> CTG	C770_RS47555	sensor histidine kinase	None	2.5E-271	433	3	WP_015243309.1
1,223,054	1,223,054	1	T -> C		81	SNP (transition)	100.0%	N -> S	1.28	AAC -> AGC	C770_RS47555	sensor histidine kinase	Substitution	3.2E-284	427	2	WP_015243309.1
1,223,068	1,223,068	1	C -> T		78	SNP (transition)	100.0%		1.266	CAG -> CAA	C770_RS47555	sensor histidine kinase	None	6.3E-266	422	3	WP_015243309.1
1,223,071	1,223,071	1	C -> A		80	SNP (transversion)	98.8%		1.263	CTG -> CTT	C770_RS47555	sensor histidine kinase	None	2.0E-267	421	3	WP_015243309.1
1,223,089	1,223,089	1	G -> A		77	SNP (transition)	100.0%		1.245	ATC -> ATT	C770_RS47555	sensor histidine kinase	None	6.3E-278	415	3	WP_015243309.1
1,223,101	1,223,101	1	T -> G		83	SNP (transversion)	100.0%		1.233	CGA -> CGC	C770_RS47555	sensor histidine kinase	None	1.6E-299	411	3	WP_015243309.1

1,223,131	1,223,131	1	G -> C		82	SNP (transversion)	100.0%		1.203	CCC -> CCG	C770_RS47555	sensor histidine kinase	None	1.0E-287	401	3	WP_015243309.1
1,223,146	1,223,146	1	C -> T		83	SNP (transition)	98.8%		1.188	TTG -> TTA	C770_RS47555	sensor histidine kinase	None	2.1E-269	396	3	WP_015243309.1
1,223,167	1,223,167	1	C -> A		82	SNP (transversion)	97.6%		1.167	GTG -> GTT	C770_RS47555	sensor histidine kinase	None	3.3E-269	389	3	WP_015243309.1
1,223,194	1,223,194	1	C -> T		83	SNP (transition)	97.6%		1.14	CGG -> CGA	C770_RS47555	sensor histidine kinase	None	2.1E-256	380	3	WP_015243309.1
1,223,208	1,223,208	1	G -> C		85	SNP (transversion)	98.8%	R -> G	1.126	CGA -> GGA	C770_RS47555	sensor histidine kinase	Substitution	5.4E-276	376	1	WP_015243309.1
1,223,253	1,223,253	1	T -> C		82	SNP (transition)	100.0%	S -> G	1.081	AGC -> GGC	C770_RS47555	sensor histidine kinase	Substitution	2.5E-271	361	1	WP_015243309.1
1,223,260	1,223,260	1	A -> T		79	SNP (transversion)	100.0%		1.074	ACT -> ACA	C770_RS47555	sensor histidine kinase	None	2.0E-261	358	3	WP_015243309.1
1,223,300	1,223,301	2	TG -> GC		83	Substitution	100.0%	Q -> A	1.033	CAG -> GCG	C770_RS47555	sensor histidine kinase	Substitution	6.3E-283	345	1	WP_015243309.1
1,223,303	1,223,303	1	T -> G		83	SNP (transversion)	100.0%	E -> A	1.031	GAA -> GCA	C770_RS47555	sensor histidine kinase	Substitution	6.3E-283	344	2	WP_015243309.1
1,223,341	1,223,341	1	A -> T		82	SNP (transversion)	100.0%		993	CTT -> CTA	C770_RS47555	sensor histidine kinase	None	2.5E-271	331	3	WP_015243309.1
1,223,350	1,223,350	1	C -> G		82	SNP (transversion)	100.0%		984	CGG -> CGC	C770_RS47555	sensor histidine kinase	None	2.5E-271	328	3	WP_015243309.1
1,223,362	1,223,362	1	C -> T		82	SNP (transition)	98.8%		972	GAG -> GAA	C770_RS47555	sensor histidine kinase	None	4.1E-266	324	3	WP_015243309.1
1,223,439	1,223,439	1	C -> T		86	SNP (transition)	100.0%	A -> T	895	GCC -> ACC	C770_RS47555	sensor histidine kinase	Substitution	4.0E-293	299	1	WP_015243309.1
1,223,470	1,223,470	1	A -> G		83	SNP (transition)	100.0%		864	GTT -> GTC	C770_RS47555	sensor histidine kinase	None	3.2E-291	288	3	WP_015243309.1
1,223,494	1,223,494	1	G -> A		79	SNP (transition)	100.0%		840	GCC -> GCT	C770_RS47555	sensor histidine kinase	None	1.6E-253	280	3	WP_015243309.1
1,223,506	1,223,506	1	G -> C		78	SNP (transversion)	100.0%		828	CGC -> CGG	C770_RS47555	sensor histidine kinase	None	4.0E-258	276	3	WP_015243309.1
1,223,518	1,223,518	1	T -> C		78	SNP (transition)	98.7%		816	ACA -> ACG	C770_RS47555	sensor histidine kinase	None	2.5E-268	272	3	WP_015243309.1
1,223,563	1,223,563	1	A -> G		78	SNP (transition)	98.7%		771	CCT -> CCC	C770_RS47555	sensor histidine kinase	None	3.1E-245	257	3	WP_015243309.1
1,223,605	1,223,605	1	C -> G		85	SNP (transversion)	100.0%	R -> S	729	AGG -> AGC	C770_RS47555	sensor histidine kinase	Substitution	1.0E-289	243	3	WP_015243309.1
1,223,612	1,223,612	1	G -> A		86	SNP (transition)	97.7%	A -> V	722	GCC -> GTC	C770_RS47555	sensor histidine kinase	Substitution	2.3E-274	241	2	WP_015243309.1
1,223,616	1,223,617	2	TT -> CC		87	Substitution	100.0%	QI -> QV	717	CAA,ATT -> CAG,	C770_RS47555	sensor histidine kinase	Substitution	4.0E-279	239	3	WP_015243309.1
1,223,623	1,223,623	1	T -> C		85	SNP (transition)	100.0%		711	CGA -> CGG	C770_RS47555	sensor histidine kinase	None	1.0E-289	237	3	WP_015243309.1
1,223,643	1,223,643	1	A -> G		86	SNP (transition)	100.0%		691	TTG -> CTG	C770_RS47555	sensor histidine kinase	None	1.0E-301	231	1	WP_015243309.1
1,223,652	1,223,652	1	C -> T		87	SNP (transition)	100.0%	A -> T	682	GCT -> ACT	C770_RS47555	sensor histidine kinase	Substitution	7.9E-288	228	1	WP_015243309.1
1,223,665	1,223,665	1	T -> G		90	SNP (transversion)	100.0%		669	TCA -> TCC	C770_RS47555	sensor histidine kinase	None	1.0E-315	223	3	WP_015243309.1
1,223,686	1,223,686	1	G -> A		88	SNP (transition)	100.0%		648	GGC -> GGT	C770_RS47555	sensor histidine kinase	None	6.3E-300	216	3	WP_015243309.1
1,223,764	1,223,764	1	C -> G		100	SNP (transversion)	99.0%		570	CGG -> CGC	C770_RS47555	sensor histidine kinase	None	0.0	190	3	WP_015243309.1
1,223,773	1,223,773	1	C -> T		100	SNP (transition)	100.0%		561	TTG -> TTA	C770_RS47555	sensor histidine kinase	None	0.0	187	3	WP_015243309.1
1,223,788	1,223,788	1	G -> A		102	SNP (transition)	98.0%		546	CAC -> CAT	C770_RS47555	sensor histidine kinase	None	0.0	182	3	WP_015243309.1
1,223,800	1,223,800	1	G -> T		100	SNP (transversion)	100.0%		534	CGC -> CGA	C770_RS47555	sensor histidine kinase	None	1.0E-320	178	3	WP_015243309.1
1,223,803	1,223,803	1	T -> G		100	SNP (transversion)	100.0%		531	ATA -> ATC	C770_RS47555	sensor histidine kinase	None	1.0E-320	177	3	WP_015243309.1
1,223,821	1,223,821	1	G -> T		100	SNP (transversion)	99.0%		513	GCC -> GCA	C770_RS47555	sensor histidine kinase	None	0.0	171	3	WP_015243309.1
1,223,863	1,223,863	1	T -> C		98	SNP (transition)	100.0%		471	GAA -> GAG	C770_RS47555	sensor histidine kinase	None	0.0	157	3	WP_015243309.1
1,223,866	1,223,866	1	C -> T		97	SNP (transition)	97.9%		468	GCG -> GCA	C770_RS47555	sensor histidine kinase	None	0.0	156	3	WP_015243309.1
1,223,872	1,223,872	1	C -> T		95	SNP (transition)	100.0%		462	GAG -> GAA	C770_RS47555	sensor histidine kinase	None	1.0E-304	154	3	WP_015243309.1
1,223,875	1,223,875	1	G -> C		94	SNP (transversion)	100.0%		459	CCC -> CCG	C770_RS47555	sensor histidine kinase	None	6.3E-311	153	3	WP_015243309.1
1,223,911	1,223,913	2	CAC -> AAT		99	Substitution	100.0%	V -> I	421	GTG -> ATT	C770_RS47555	sensor histidine kinase	Substitution	1.3E-307	141	1	WP_015243309.1
1,223,923	1,223,923	1	C -> A		102	SNP (transversion)	100.0%		411	GGG -> GGT	C770_RS47555	sensor histidine kinase	None	0.0	137	3	WP_015243309.1
1,223,968	1,223,968	1	T -> C		97	SNP (transition)	100.0%		366	GAA -> GAG	C770_RS47555	sensor histidine kinase	None	0.0	122	3	WP_015243309.1
1,223,992	1,223,992	1	C -> G		90	SNP (transversion)	98.9%		342	TCG -> TCC	C770_RS47555	sensor histidine kinase	None	1.4E-283	114	3	WP_015243309.1
1,224,010	1,224,010	1	A -> G		79	SNP (transition)	100.0%		324	ATT -> ATC	C770_RS47555	sensor histidine kinase	None	2.0E-261	108	3	WP_015243309.1
1,224,016	1,224,016	1	T -> C		83	SNP (transition)	98.8%		318	GCA -> GCG	C770_RS47555	sensor histidine kinase	None	1.3E-277	106	3	WP_015243309.1
1,224,045	1,224,045	1	G -> C		86	SNP (transversion)	100.0%	P -> A	289	CCC -> GCC	C770_RS47555	sensor histidine kinase	Substitution	1.6E-284	97	1	WP_015243309.1
1,224,064	1,224,064	1	A -> G		87	SNP (transition)	98.9%		270	GAT -> GAC	C770_RS47555	sensor histidine kinase	None	3.5E-291	90	3	WP_015243309.1
1,224,067	1,224,069	2	AGC -> GGT	85 -> 87		Substitution	97.6% -> 97.7%	A -> T	265	GCT -> ACC	C770_RS47555	sensor histidine kinase	Substitution	9.0E-263	89	1	WP_015243309.1
1,224,072	1,224,072	1	C -> T		86	SNP (transition)	100.0%	V -> I	262	GTT -> ATT	C770_RS47555	sensor histidine kinase	Substitution	4.0E-293	88	1	WP_015243309.1
1,224,101	1,224,101	1	T -> A		82	SNP (transversion)	100.0%	Y -> F	233	TAC -> TTC	C770_RS47555	sensor histidine kinase	Substitution	6.3E-255	78	2	WP_015243309.1
1,224,118	1,224,118	1	A -> G		81	SNP (transition)	100.0%		216	CTT -> CTC	C770_RS47555	sensor histidine kinase	None	4.0E-276	72	3	WP_015243309.1
1,224,121	1,224,121	1	G -> A		79	SNP (transition)	98.7%		213	CGC -> CGT	C770_RS47555	sensor histidine kinase	None	5.0E-264	71	3	WP_015243309.1
1,224,142	1,224,142	1	T -> C		73	SNP (transition)	98.6%		192	ACA -> ACG	C770_RS47555	sensor histidine kinase	None	1.2E-243	64	3	WP_015243309.1
1,224,145	1,224,145	1	A -> T		74	SNP (transversion)	100.0%		189	GTT -> GTA	C770_RS47555	sensor histidine kinase	None	1.6E-237	63	3	WP_015243309.1
1,224,217	1,224,217	1	G -> T		62	SNP (transversion)	100.0%		117	GTC -> GTA	C770_RS47555	sensor histidine kinase	None	4.0E-199	39	3	WP_015243309.1
1,224,242	1,224,242	1	T -> C		70	SNP (transition)	100.0%	N -> S	92	AAT -> AGT	C770_RS47555	sensor histidine kinase	Substitution	1.0E-252	31	2	WP_015243309.1
1,224,244	1,224,244	1	C -> G		69	SNP (transversion)	100.0%		90	CCG -> CCC	C770_RS47555	sensor histidine kinase	None	2.5E-235	30	3	WP_015243309.1
1,224,265	1,224,265	1	T -> C		70	SNP (transition)	98.6%		69	CAA -> CAG	C770_RS47555	sensor histidine kinase	None	2.2E-240	23	3	WP_015243309.1
1,224,292	1,224,292	1	G -> A		78	SNP (transition)	97.4%		42	GCC -> GCT	C770_RS47555	sensor histidine kinase	None	4.8E-248	14	3	WP_015243309.1
1,224,298	1,224,298	1	G -> A		76	SNP (transition)	98.7%		36	ATC -> ATT	C770_RS47555	sensor histidine kinase	None	7.6E-254	12	3	WP_015243309.1
1,224,307	1,224,307	1	G -> A		75	SNP (transition)	98.7%		27	ATC -> ATT	C770_RS47555	sensor histidine kinase	None	1.9E-250	9	3	WP_015243309.1
1,224,319	1,224,319	1	C -> T		79	SNP (transition)	98.7%		15	TTG -> TTA	C770_RS47555	sensor histidine kinase	None	5.0E-264	5	3	WP_015243309.1
1,224,347	1,224,347	1	C -> G		75	SNP (transversion)	98.7%							4.7E-243			
1,224,359	1,224,360	2	CA -> AG		76	Substitution	97.4%							4.5E-234			
1,224,364	1,224,364	1	G		77	Deletion	98.7%							7.5E-113			
1,224,372	1,224,376	5	TCCGG		76	Deletion	98.7%							2.4E-126			
1,224,431	1,224,431	1	T -> C		79	SNP (transition)	98.7%							5.0E-264			
1,224,441	1,224,441	1	A -> T		77	SNP (transversion)	98.7%							1.2E-249			
1,224,450	1,224,450	1	G -> A		77	SNP (transition)	98.7%							4.9E-242			
1,224,479	1,224,479	1	G -> C		79	SNP (transversion)	98.7%							7.9E-233			
1,224,488	1,224,489	2	GC -> AT	82 -> 83		Substitution	98.8%							3.3E-261			
1,224,512	1,224,511	0	T		82	Insertion	96.3%							1.4E-248			
1,224,517	1,224,517	1	T -> C		81	SNP (transition)	96.3%							1.3E-237			
1,224,525	1,224,525	1	T -> C		77	SNP (transition)	97.4%							9.2E-245			
1,224,674	1,224,674	1	T -> C		75	SNP (transition)	98.7%		96	GCT -> GCC	C770_RS47560	chemotaxis protein Che	None	4.7E-243	32	3	WP_015243310.1
1,224,680	1,224,680	1	G -> A		73	SNP (transition)	98.6%		102	GTG -> GTA	C770_RS47560	chemotaxis protein Che	None	1.2E-243	34	3	WP_015243310.1
1,224,686	1,224,686	1	G -> C		72	SNP (transversion)	98.6%		108	GTG -> GTC	C770_RS47560	chemotaxis protein Che	None	2.3E-247	36	3	WP_015243310.1
1,224,698	1,224,698	1	A -> C		72	SNP (transversion)	98.6%		120	GTA -> GTC	C770_RS47560	chemotaxis protein Che	None	2.3E-247	40	3	WP_015243310.1
1,224,732	1,224,732	1	C -> T		72	SNP (transition)	98.6%	L -> F	154	CTC -> TTC	C770_RS47560	chemotaxis protein Che	Substitution	2.3E-247	52	1	WP_015243310.1
1,224,740	1,224,740	1	C -> G		71	SNP (transversion)	98.6%		162	GCC -> GCG	C770_RS47560	chemotaxis protein Che	None	7.1E-244	54	3	WP_015243310.1
1,224,776	1,224,776	1	T -> G		71	SNP (transversion)	100.0%		198	GGT -> GGG	C770_RS47560	chemotaxis protein Che	None	5.0E-235	66	3	WP_015243310.1
1,224,785	1,224,785	1	T -> A		75	SNP (transversion)	100.0%		207	GTT -> GTA	C770_RS47560	chemotaxis protein Che	None	3.2E-263	69	3	WP_015243310.1
1,224,809	1,224,809	1	G -> T		73	SNP (transversion)	98.6%		231	GCG -> GCT	C770_RS47560	chemotaxis protein Che	None	1.2E-243	77	3	WP_015243310.1
1,224,863	1,224,863	1	C -> A		69	SNP (transversion)	98.6%		285	GGC -> GGA	C770_RS47560	chemotaxis protein Che	None	2.7E-223	95	3	WP_015243310.1
1,224,912	1,224,912	1	G -> A		68	SNP (transition)	100.0%	V -> I	334	GTC -> ATC	C770_RS47560	chemotaxis protein Che	Substitution	1.6E-211	112	1	WP_015243310.1
1,224,917	1,224,917	1	C -> T		69	SNP (transition)	98.6%		339	GGC -> GGT	C770_RS47560	chemotaxis protein Che	None	4.4E-230	113	3	WP_015243310.1
1,224,936	1,224,936	1	G -> T		74	SNP (transversion)	100.0%	A -> S	358	GCA -> TCA	C770_RS47560	chemotaxis protein Che	Substitution	1.0E-259	120	1	WP_015243310.1
1,224,945	1,224,947	2	ATT -> GTC		78	Substitution	98.7%	I -> V	367	ATT -> GTC	C770_RS47560	chemotaxis protein Che	Substitution	7.8E-230	123	1	WP_015243310.1
1,225,028	1,225,028	1	C -> T		71	SNP (transition)	95.8%		450	AAC -> AAT	C770_RS47560	chemotaxis protein Che	None	2.3E-220	150	3	WP_015243310.1
1,225,037	1,225,037	1	A -> C		73	SNP (transversion)	98.6%	E -> D	459	GAA -> GAC	C770_RS47560	chemotaxis protein Che	Substitution	1.2E-243	153	3	WP_015243310.1
1,225,070	1,225,070	1	G -> A		77	SNP (transition)	97.4%		492	CTG -> CTA	C770_RS47560	chemotaxis protein Che	None	9.2E-245	164	3	WP_015243310.1
1,225,097	1,225,097	1	G -> T		76	SNP (transversion)	97.4%		519	GCG -> GCT	C770_RS47560	chemotaxis protein Che	None	1.8E-241	173	3	WP_015243310.1
1,225,108	1,225,108	1	A -> G		76	SNP (transition)	98.7%	D -> G	530	GAT -> GGT	C770_RS47560	chemotaxis protein Che	Substitution	7.6E-254	177	2	WP_015243310.1
1,225,121	1,225,121	1	C -> T		77	SNP (transition)	98.7%		543	CTC -> CTT	C770_RS47560	chemotaxis protein Che	None	3.1E-257	181	3	WP_015243310.1
1,225,162	1,225,162	1	T -> C		82	SNP (transition)	98.8%	L -> P	584	CTC -> CCC	C770_RS47560	chemotaxis protein Che	Substitution	2.6E-282	195	2	WP_015243310.1
1,225,184	1,225,184	1	T -> C		78	SNP (transition)	98.7%		606	CTT -> CTC	C770_RS47560	chemotaxis protein Che	None	1.2E-260	202	3	WP_015243310.1
1,225,190	1,225,190	1	T -> C		78	SNP (transition)	97.4%		612	GTT -> GTC	C770_RS47560	chemotaxis protein Che	None	1.2E-255	204	3	WP_015243310.1
1,225,200	1,225,202	2	CTT -> TTC	74 -> 76		Substitution	98.6% -> 98.7%	L -> F	622	CTT -> TTC	C770_RS47560	chemotaxis protein Che	Substitution	4.7E-247	208	1	WP_015243310.1
1,225,207	1,225,207	1	T -> G		76	SNP (transversion)	98.7%	L -> R	629	CTA -> CGA	C770_RS47560	chemotaxis protein Che	Substitution	2.4E-261	210	2	WP_015243310.1
1,225,242	1,225,242	1	A -> G		79	SNP (transition)	96.2%	N -> D	664	AAC -> GAC	C770_RS47560	chemotaxis protein Che	Substitution	5.0E-239	222	1	WP_015243310.1
1,225,363	1,225,363	1	C -> A		68	SNP (transversion)	95.6%	A -> E	785	GCG -> GAG	C770_RS47560	chemotaxis protein Che	Substitution	5.0E-217	262	2	WP_015243310.1
1,225,388	1,225,388	1	T -> C		73	SNP (transition)	97.3%		810	GCT -> GCC	C770_RS47560	chemotaxis protein Che	None	1.0E-238	270	3	WP_015243310.1
1,225,406	1,225,406	1	C -> G		72	SNP (transversion)	98.6%		828	CGC -> CGG	C770_RS47560	chemotaxis protein Che	None	3.6E-233	276	3	WP_015243310.1
1,225,421	1,225,421	1	A -> G		72	SNP (transition)	97.2%		843	AGA -> AGG	C770_RS47560	chemotaxis protein Che	None	2.6E-242	281	3	WP_015243310.1
1,225,457	1,225,457	1	T -> G		76	SNP (transversion)	98.7%	H -> Q	879	CAT -> CAG	C770_RS47560	chemotaxis protein Che	Substitution	2.4E-246	293	3	WP_015243310.1
1,225,460	1,225,460	1	A -> C		75	SNP (transversion)	98.7%		882	CGA -> CGC	C770_RS47560	chemotaxis protein Che	None	4.7E-243	294	3	WP_015243310.1
1,225,466	1,225,466	1	A -> G		76	SNP (transition)	98.7%		888	GGA -> GGG	C770_RS47560	chemotaxis protein Che	None	2.4E-246	296	3	WP_015243310.1
1,225,530	1,225,530	1	T -> C		70	SNP (transition)	100.0%		952	TTG -> CTG	C770_RS47560	chemotaxis protein Che	None	1.0E-238	318	1	WP_015243310.1
1,225,555	1,225,555	1	G -> C		74	SNP (transversion)	100.0%	G -> A	977	GGG -> GCG	C770_RS47560	chemotaxis protein Che	Substitution	2.5E-252	326	2	WP_015243310.1
1,225,557	1,225,557	1	A -> C		74	SNP (transversion)	100.0%	T -> P	979	ACG -> CCG	C770_RS47560	chemotaxis protein Che	Substitution	1.6E-237	327	1	WP_015243310.1
1,225,563	1,225,563	1	G -> A		72	SNP (transition)	100.0%	A -> T	985	GCA -> ACA	C770_RS47560	chemotaxis protein Che	Substitution	1.6E-245	329	1	WP_015243310.1
1,225,575	1,225,575	1	G -> A		72	SNP (transition)	98.6%	E -> K	997	GAA -> AAA	C770_RS47560	chemotaxis protein Che	Substitution	3.6E-233	333	1	WP_015243310.1
1,225,604	1,225,604	1	T -> C		68	SNP (transition)	100.0%		1.026	GCT -> GCC	C770_RS47560	chemotaxis protein Che	None	6.3E-232	342	3	WP_015243310.1
1,225,616	1,225,617	2	CA -> TC		70	Substitution	100.0%	DT -> DP	1.038	GAC,ACA -> GAT,	C770_RS47560	chemotaxis protein Che	Substitution	1.0E-224	346	3	WP_015243310.1
1,225,646	1,225,646	1	A -> C		74	SNP (transversion)	100.0%							6.3E-245			
1,225,654	1,225,654	1	G -> TC		73	Insertion	98.6%							1.8E-236			
1,225,679	1,225,679	1	A -> G		72	SNP (transition)	100.0%							1.0E-252			
1,225,682	1,225,682	1	G -> C		73	SNP (transversion)	98.6%							1.2E-243			
1,225,712	1,225,712	1	C -> G		71	SNP (transversion)	98.6%		141	ACG -> ACC	C770_RS47565	histidine kinase	None	7.1E-230	47	3	WP_041169932.1
1,225,716	1,225,716	1	G -> A		70	SNP (transition)	100.0%	S -> L	137	TCG -> TTG	C770_RS47565	histidine kinase	Substitution	1.0E-224	46	2	WP_041169932.1
1,225,721	1,225,721	1	C -> G		71	SNP (transversion)	98.6%	E -> D	132	GAG -> GAC	C770_RS47565	histidine kinase	Substitution	7.1E-230	44	3	WP_041169932.1
1,225,733	1,225,733	1	G -> C		75	SNP (transversion)	98.7%	H -> Q	120	CAC -> CAG	C770_RS47565	histidine kinase	Substitution	7.5E-258	40	3	WP_041169932.1
1,225,750	1,225,750	1	T -> C		75	SNP (transition)	98.7%	I -> V	103	ATA -> GTA	C770_RS47565	histidine kinase	Substitution	4.7E-243	35	1	WP_041169932.1
1,225,784	1,225,784	1	T -> C		72	SNP (transition)	100.0%	I -> M	69	ATA -> ATG	C770_RS47565	histidine kinase	Substitution	1.0E-252	23	3	WP_041169932.1
1,225,798	1,225,798	1	A -> C		73	SNP (transversion)	100.0%	F -> V	55	TTC -> GTC	C770_RS47565	histidine kinase	Substitution	6.3E-249	19	1	WP_041169932.1
1,225,845	1,225,845	1	A -> C		75	SNP (transversion)	100.0%	V -> G	8	GTC -> GGC	C770_RS47565	histidine kinase	Substitution	1.0E-255	3	2	WP_041169932.1
1,225,872	1,225,872	1	C -> G		78	SNP (transversion)	98.7%							1.2E-260			
1,225,887	1,225,887	1	T		80	Deletion	100.0%							1.0E-120			
1,225,895	1,225,895	1	C -> G		81	SNP (transversion)	100.0%							3.2E-284			
1,225,925	1,225,924	0	GGTGAC	78 -> 81		Insertion	98.7% -> 98.8%							8.1E-263			
1,225,938	1,225,938	1	G -> C		77	SNP (transversion)	100.0%							4.0E-247			
1,225,940	1,225,943	4	CCAG		77	Deletion	100.0%							6.3E-124			
1,225,950	1,225,950	1	A -> T		76	SNP (transversion)	100.0%							1.6E-251			
1,225,957	1,225,957	1	G		76	Deletion	100.0%							2.5E-122			
1,225,964	1,225,964	1	G -> A		77	SNP (transition)	100.0%							1.6E-262			
1,225,968	1,225,968	1	T -> G		77	SNP (transversion)	98.7%							1.9E-272			
1,225,971	1,225,971	1	G -> T		76	SNP (transversion)	98.7%							7.6E-254			
1,225,988	1,225,988	1	G -> A		75	SNP (transition)	98.7%							4.7E-243			
1,226,011	1,226,011	1	T -> C		76	SNP (transition)	100.0%							1.6E-251			
1,226,040	1,226,040	1	G -> A		71	SNP (transition)	100.0%							3.2E-249			
1,226,051	1,226,050	0	(C)2 -> (C)3		70	Insertion (tandem repeat)	100.0%							1.0E-245			
1,226,076	1,226,076	1	G -> A		72	SNP (transition)	100.0%							1.0E-252			
1,226,100	1,226,100	1	A -> G		68	SNP (transition)	100.0%							6.3E-232			
1,226,113	1,226,115	3	CGT -> GTG	69 -> 70		Substitution	100.0%							1.0E-231			
1,226,140	1,226,140	1	C -> G		68	SNP (transversion)	100.0%							1.0E-238			
1,226,142	1,226,142	1	A -> G		67	SNP (transition)	100.0%							3.2E-235			
1,226,144	1,226,144	1	G -> A		67	SNP (transition)	98.5%							1.7E-236			
1,226,147	1,226,147	1	T -> G		68	SNP (transversion)	100.0%							1.6E-245			
1,226,167	1,226,167	1	C -> T		61	SNP (transition)	100.0%							3.2E-214			
1,226,182	1,226,184	3	TGC -> CCT	63 -> 64		Substitution	100.0%							1.0E-224			
1,226,192	1,226,192	1	G -> A		64	SNP (transition)	100.0%							2.5E-218			
1,226,197	1,226,199	3	TTA -> GGC	65 -> 66		Substitution	96.9% -> 97.0%							2.6E-205			
1,226,202	1,226,202	1	T -> C		65	SNP (transition)	100.0%							1.0E-234			
1,226,237	1,226,237	1	C -> T		72	SNP (transition)	100.0%							4.0E-231			
1,226,248	1,226,248	1	-C		71	Deletion	100.0%							3.2E-107			
1,226,254	1,226,254	1	T -> C		76	SNP (transition)	100.0%							1.6E-251			
1,226,256	1,226,256	1	T -> C		76	SNP (transition)	100.0%							1.6E-251			
1,226,260	1,226,261	2	GG -> CC	76 -> 77		Substitution	98.7%							1.2E-249			
1,226,263	1,226,264	2	TG -> CC		78	Substitution	100.0%							2.5E-250			
1,226,269	1,226,269	1	T		77	Deletion	97.4%							8.7E-110			
1,226,280	1,226,281	2	TC -> AT		77	Substitution	100.0%							7.9E-255			
1,226,288	1,226,288	1	T -> C		77	SNP (transition)	100.0%							1.6E-262			
1,226,292	1,226,291	0	A		80	Insertion	100.0%							1.0E-264			

1,226,295	1,226,295	1	A -> G	80	SNP (transition)	98.8%							1.6E-259			
1,226,299	1,226,299	1	C -> T	82	SNP (transition)	98.8%							3.3E-274			
1,226,303	1,226,303	1	C -> T	83	SNP (transition)	98.8%							1.3E-277			
1,226,338	1,226,338	1	C -> G	90	SNP (transversion)	98.9%							1.8E-292			
1,226,342	1,226,342	1	T -> C	88	SNP (transition)	100.0%							6.3E-300			
1,226,352	1,226,352	1	G -> A	89	SNP (transition)	100.0%							3.2E-312			
1,226,360	1,226,360	1	C -> T	94	SNP (transition)	100.0%							1.6E-301			
1,226,384	1,226,384	1	C -> T	99	SNP (transition)	100.0%							0.0			
1,226,391	1,226,391	1	G -> A	96	SNP (transition)	99.0%							0.0			
1,226,396	1,226,396	1	G -> A	94	SNP (transition)	100.0%							6.3E-311			
1,226,400	1,226,401	2	CT -> GG	97	Substitution	100.0%							0.0			
1,226,405	1,226,408	4	ATTT	97	Deletion	100.0%							6.3E-156			
1,226,417	1,226,417	1	C -> T	97	SNP (transition)	100.0%							4.0E-311			
1,226,436	1,226,436	1	(T)4 -> (T)3	99	Deletion (tandem repeat)	100.0%							4.0E-159			
1,226,439	1,226,439	1	C -> G	99	SNP (transversion)	100.0%							0.0			
1,226,449	1,226,450	2	TA -> CG	101	Substitution	100.0%							0.0			
1,226,463	1,226,463	1	C -> G	98	SNP (transversion)	99.0%							0.0			
1,226,468	1,226,467	0	C	98	Insertion	100.0%							0.0			
1,226,472	1,226,472	1	A -> T	100	SNP (transversion)	100.0%							0.0			
1,226,476	1,226,476	1	A -> G	99	SNP (transition)	100.0%		924	TGT -> TGC	C770_RS47570	hypothetical protein	None	0.0	308	3	WP_015243312.1
1,226,482	1,226,484	2	CCA -> GCG	98 -> 99	Substitution	98.0%	W -> R	916	TGG -> CGC	C770_RS47570	hypothetical protein	Substitution	0.0	306	1	WP_015243312.1
1,226,488	1,226,488	1	C -> A	98	SNP (transversion)	99.0%		912	GCG -> GCT	C770_RS47570	hypothetical protein	None	7.8E-319	304	3	WP_015243312.1
1,226,509	1,226,509	1	C -> T	95	SNP (transition)	100.0%		891	ACG -> ACA	C770_RS47570	hypothetical protein	None	0.0	297	3	WP_015243312.1
1,226,521	1,226,521	1	T -> C	93	SNP (transition)	100.0%		879	CGA -> CGG	C770_RS47570	hypothetical protein	None	1.3E-307	293	3	WP_015243312.1
1,226,593	1,226,593	1	A -> C	92	SNP (transversion)	100.0%		807	GCT -> GCG	C770_RS47570	hypothetical protein	None	2.5E-304	269	3	WP_015243312.1
1,226,596	1,226,596	1	C -> T	93	SNP (transition)	100.0%		804	CAG -> CAA	C770_RS47570	hypothetical protein	None	1.3E-307	268	3	WP_015243312.1
1,226,622	1,226,623	2	AC -> CT	82 -> 84	Substitution	100.0%	TY -> TD	777	ACG,TAC -> ACA,	C770_RS47570	hypothetical protein	Substitution	2.5E-286	259	3	WP_015243312.1
1,226,673	1,226,673	1	C -> T	80	SNP (transition)	100.0%	A -> T	727	GCT -> ACT	C770_RS47570	hypothetical protein	Substitution	1.0E-280	243	1	WP_015243312.1
1,226,795	1,226,795	1	T -> C	84	SNP (transition)	98.8%	Q -> R	605	CAA -> CGA	C770_RS47570	hypothetical protein	Substitution	2.7E-289	202	2	WP_015243312.1
1,226,969	1,226,969	1	C -> A	87	SNP (transversion)	100.0%	C -> F	431	TGC -> TTC	C770_RS47570	hypothetical protein	Substitution	7.9E-288	144	2	WP_015243312.1
1,227,003	1,227,003	1	G -> T	85	SNP (transversion)	100.0%		397	CGG -> AGG	C770_RS47570	hypothetical protein	None	3.2E-298	133	1	WP_015243312.1
1,227,015	1,227,015	1	C -> T	85	SNP (transition)	100.0%	V -> I	385	GTC -> ATC	C770_RS47570	hypothetical protein	Substitution	1.0E-289	129	1	WP_015243312.1
1,227,018	1,227,018	1	T -> C	85	SNP (transition)	100.0%	S -> G	382	AGC -> GGC	C770_RS47570	hypothetical protein	Substitution	1.0E-289	128	1	WP_015243312.1
1,227,030	1,227,032	3	GAT -> CGA	86 -> 88	Substitution	98.8% -> 98.9%	YL -> FV	368	TAT,CTC -> TTC,(	C770_RS47570	hypothetical protein	Substitution	4.0E-291	123	2	WP_015243312.1
1,227,067	1,227,067	1	C -> T	93	SNP (transition)	98.9%		333	CAG -> CAA	C770_RS47570	hypothetical protein	None	2.3E-302	111	3	WP_015243312.1
1,227,117	1,227,117	1	G -> A	90	SNP (transition)	100.0%	H -> Y	283	CAC -> TAC	C770_RS47570	hypothetical protein	Substitution	1.0E-306	95	1	WP_015243312.1
1,227,125	1,227,125	1	T -> C	91	SNP (transition)	100.0%	Q -> R	275	CAA -> CGA	C770_RS47570	hypothetical protein	Substitution	4.0E-310	92	2	WP_015243312.1
1,227,153	1,227,153	1	C -> T	91	SNP (transition)	100.0%	G -> R	247	GGA -> AGA	C770_RS47570	hypothetical protein	Substitution	4.0E-310	83	1	WP_015243312.1
1,227,162	1,227,162	1	G -> C	90	SNP (transversion)	100.0%	R -> G	238	CGG -> GGG	C770_RS47570	hypothetical protein	Substitution	1.0E-288	80	1	WP_015243312.1
1,227,210	1,227,210	1	T -> C	97	SNP (transition)	100.0%	N -> D	190	AAT -> GAT	C770_RS47570	hypothetical protein	Substitution	7.9E-321	64	1	WP_015243312.1
1,227,290	1,227,290	1	G -> C	96	SNP (transversion)	100.0%	A -> G	110	GCT -> GGT	C770_RS47570	hypothetical protein	Substitution	1.6E-317	37	2	WP_015243312.1
1,227,304	1,227,304	1	C -> G	96	SNP (transversion)	99.0%	M -> I	96	ATG -> ATC	C770_RS47570	hypothetical protein	Substitution	0.0	32	3	WP_015243312.1
1,227,334	1,227,334	1	C -> A	98	SNP (transversion)	100.0%	L -> F	66	TTG -> TTT	C770_RS47570	hypothetical protein	Substitution	4.9E-324	22	3	WP_015243312.1
1,227,471	1,227,471	1	G -> T	94	SNP (transversion)	100.0%							1.6E-301			
1,227,473	1,227,473	1	C -> T	94	SNP (transition)	98.9%							1.2E-305			
1,227,477	1,227,476	0	(T)3 -> (T)4	92	Insertion (tandem repeat)	100.0%							4.0E-295			
1,227,503	1,227,503	1	G -> A	91	SNP (transition)	100.0%							3.2E-319			
1,227,517	1,227,517	1	G -> C	95	SNP (transversion)	97.9%							2.8E-313			
1,227,532	1,227,531	0	GCTTT	100 -> 101	Insertion	98.0%							1.6E-315			
1,227,545	1,227,545	1	G -> T	99	SNP (transversion)	100.0%							0.0			
1,227,562	1,227,563	2	AA -> GG	94	Substitution	100.0%							6.3E-311			
1,227,655	1,227,655	1	C -> T	88	SNP (transition)	100.0%							6.3E-300			
1,227,701	1,227,701	1	C -> G	86	SNP (transversion)	100.0%							2.5E-267			
1,227,728	1,227,728	1	G -> A	90	SNP (transition)	97.8%							1.6E-287			
1,227,755	1,227,755	1	G -> A	91	SNP (transition)	100.0%							5.0E-301			
1,227,884	1,227,884	1	C -> A	72	SNP (transversion)	98.6%							2.9E-240			
1,227,887	1,227,887	1	G -> C	73	SNP (transversion)	100.0%							3.2E-256			
1,227,916	1,227,916	1	A -> G	79	SNP (transition)	100.0%							1.6E-253			
1,227,953	1,227,953	1	A -> T	82	SNP (transversion)	98.8%							3.3E-274			
1,227,977	1,227,977	1	T -> A	86	SNP (transversion)	100.0%							4.0E-293			
1,228,024	1,228,026	3	TTA	77	Deletion	98.7%							1.9E-120			
1,228,035	1,228,035	1	A -> C	78	SNP (transversion)	98.7%							2.5E-268			
1,228,050	1,228,050	1	C -> G	75	SNP (transversion)	98.7%							7.5E-258			
1,228,097	1,228,097	1	C -> T	75	SNP (transition)	98.7%		774	CCG -> CCA	C770_RS47575	dehydrogenase	None	7.5E-258	258	3	WP_015243313.1
1,228,127	1,228,127	1	T -> C	86	SNP (transition)	98.8%		744	GTA -> GTG	C770_RS47575	dehydrogenase	None	2.7E-279	248	3	WP_015243313.1
1,228,262	1,228,262	1	G -> A	85	SNP (transition)	100.0%		609	GAC -> GAT	C770_RS47575	dehydrogenase	None	1.0E-289	203	3	WP_015243313.1
1,228,265	1,228,265	1	A -> C	85	SNP (transversion)	100.0%		606	CCT -> CCG	C770_RS47575	dehydrogenase	None	3.2E-281	202	3	WP_015243313.1
1,228,283	1,228,283	1	A -> G	92	SNP (transition)	100.0%		588	GAT -> GAC	C770_RS47575	dehydrogenase	None	1.6E-313	196	3	WP_015243313.1
1,228,322	1,228,322	1	C -> T	88	SNP (transition)	100.0%		549	GCG -> GCA	C770_RS47575	dehydrogenase	None	6.3E-300	183	3	WP_015243313.1
1,228,336	1,228,336	1	C -> G	88	SNP (transversion)	100.0%	V -> L	535	GTC -> CTC	C770_RS47575	dehydrogenase	Substitution	1.0E-308	179	1	WP_015243313.1
1,228,409	1,228,409	1	G -> A	84	SNP (transition)	100.0%		462	TTC -> TTT	C770_RS47575	dehydrogenase	None	4.0E-261	154	3	WP_015243313.1
1,228,481	1,228,481	1	A -> C	76	SNP (transversion)	98.7%		390	CTT -> CTG	C770_RS47575	dehydrogenase	None	7.6E-269	130	3	WP_015243313.1
1,228,632	1,228,632	1	G -> A	77	SNP (transition)	100.0%	S -> L	239	TCG -> TTG	C770_RS47575	dehydrogenase	Substitution	7.9E-255	80	2	WP_015243313.1
1,228,645	1,228,645	1	A -> C	78	SNP (transversion)	100.0%	S -> A	226	TCG -> GCG	C770_RS47575	dehydrogenase	Substitution	6.3E-266	76	1	WP_015243313.1
1,228,685	1,228,685	1	C -> T	71	SNP (transition)	100.0%		186	GAG -> GAA	C770_RS47575	dehydrogenase	None	6.3E-228	62	3	WP_015243313.1
1,228,688	1,228,688	1	G -> A	70	SNP (transition)	98.6%		183	GCC -> GCT	C770_RS47575	dehydrogenase	None	2.2E-240	61	3	WP_015243313.1
1,228,733	1,228,733	1	C -> T	69	SNP (transition)	100.0%		138	AAG -> AAA	C770_RS47575	dehydrogenase	None	1.6E-221	46	3	WP_015243313.1
1,228,778	1,228,780	2	CCG -> TCC	71 -> 73	Substitution	100.0%	R -> G	91	CGG -> GGA	C770_RS47575	dehydrogenase	Substitution	1.3E-241	31	1	WP_015243313.1
1,228,794	1,228,794	1	G -> A	74	SNP (transition)	100.0%	A -> V	77	GCG -> GTG	C770_RS47575	dehydrogenase	Substitution	6.3E-245	26	2	WP_015243313.1
1,228,826	1,228,826	1	A -> C	72	SNP (transversion)	100.0%		45	GCT -> GCG	C770_RS47575	dehydrogenase	None	4.0E-231	15	3	WP_015243313.1
1,228,835	1,228,835	1	T -> C	74	SNP (transition)	100.0%		36	GTA -> GTG	C770_RS47575	dehydrogenase	None	1.0E-259	12	3	WP_015243313.1
1,228,838	1,228,838	1	C -> T	73	SNP (transition)	100.0%	M -> I	33	ATG -> ATA	C770_RS47575	dehydrogenase	Substitution	6.3E-249	11	3	WP_015243313.1
1,228,855	1,228,856	2	CA -> TT	72 -> 73	Substitution	97.2% -> 97.3%	LE -> LK	15	CTT,GAG -> CTA,	C770_RS47575	dehydrogenase	Substitution	4.6E-222	5	3	WP_015243313.1
1,228,871	1,228,871	1	T -> C	69	SNP (transition)	98.6%							4.4E-230			
1,228,879	1,228,878	0	TC	68	Insertion	100.0%							2.5E-218			
1,228,942	1,228,942	1	A -> G	69	SNP (transition)	100.0%		1.107	AAT -> AAC	C770_RS47580	L-alanine-DL-glutamate	None	2.5E-235	369	3	WP_015243314.1
1,229,290	1,229,290	1	G -> A	69	SNP (transition)	100.0%		759	GAC -> GAT	C770_RS47580	L-alanine-DL-glutamate	None	2.0E-228	253	3	WP_015243314.1
1,229,296	1,229,296	1	G -> A	70	SNP (transition)	100.0%		753	TTC -> TTT	C770_RS47580	L-alanine-DL-glutamate	None	1.0E-231	251	3	WP_015243314.1
1,229,335	1,229,335	1	G -> A	69	SNP (transition)	98.6%		714	TAC -> TAT	C770_RS47580	L-alanine-DL-glutamate	None	2.7E-223	238	3	WP_015243314.1
1,229,347	1,229,347	1	T -> G	65	SNP (transversion)	100.0%		702	GGA -> GGC	C770_RS47580	L-alanine-DL-glutamate	None	1.0E-221	234	3	WP_015243314.1
1,229,524	1,229,524	1	G -> A	57	SNP (transition)	100.0%		525	GGC -> GGT	C770_RS47580	L-alanine-DL-glutamate	None	1.6E-194	175	3	WP_015243314.1
1,229,736	1,229,736	1	A -> G	74	SNP (transition)	98.6%		313	TTG -> CTG	C770_RS47580	L-alanine-DL-glutamate	None	4.7E-247	105	1	WP_015243314.1
1,229,836	1,229,836	1	C -> G	67	SNP (transversion)	100.0%		213	GGG -> GGC	C770_RS47580	L-alanine-DL-glutamate	None	7.9E-222	71	3	WP_015243314.1
1,229,890	1,229,890	1	C -> A	69	SNP (transversion)	100.0%		159	GTG -> GTT	C770_RS47580	L-alanine-DL-glutamate	None	1.6E-221	53	3	WP_015243314.1
1,229,947	1,229,947	1	C -> G	64	SNP (transversion)	98.4%		102	GTG -> GTC	C770_RS47580	L-alanine-DL-glutamate	None	8.1E-207	34	3	WP_015243314.1
1,229,965	1,229,965	1	G -> C	69	SNP (transversion)	100.0%		84	GGC -> GGG	C770_RS47580	L-alanine-DL-glutamate	None	1.6E-221	28	3	WP_015243314.1
1,229,968	1,229,968	1	G -> C	68	SNP (transversion)	100.0%		81	GTC -> GTG	C770_RS47580	L-alanine-DL-glutamate	None	2.5E-218	27	3	WP_015243314.1
1,229,973	1,229,973	1	T -> C	66	SNP (transition)	100.0%	I -> V	76	ATC -> GTC	C770_RS47580	L-alanine-DL-glutamate	Substitution	4.0E-225	26	1	WP_015243314.1
1,229,986	1,229,986	1	C -> T	68	SNP (transition)	100.0%		63	GAG -> GAA	C770_RS47580	L-alanine-DL-glutamate	None	6.3E-232	21	3	WP_015243314.1
1,229,993	1,229,993	1	T -> C	66	SNP (transition)	100.0%	K -> R	56	AAG -> AGG	C770_RS47580	L-alanine-DL-glutamate	Substitution	1.6E-218	19	2	WP_015243314.1
1,230,022	1,230,022	1	G -> A	65	SNP (transition)	100.0%		27	TAC -> TAT	C770_RS47580	L-alanine-DL-glutamate	None	3.2E-215	9	3	WP_015243314.1
1,230,037	1,230,037	1	G -> T	71	SNP (transversion)	100.0%		12	ACC -> ACA	C770_RS47580	L-alanine-DL-glutamate	None	6.3E-228	4	3	WP_015243314.1
1,230,050	1,230,050	1	A -> C	77	SNP (transversion)	100.0%							7.9E-255			
1,230,061	1,230,061	1	G -> A	80	SNP (transition)	100.0%							1.0E-248			
1,230,063	1,230,063	1	T -> C	80	SNP (transition)	100.0%							1.0E-280			
1,230,065	1,230,070	6	GTCAGA -> AAG1	80 -> 82	Substitution	97.5% -> 97.6%							1.0E-256			
1,230,073	1,230,073	1	G -> A	79	SNP (transition)	100.0%							1.0E-237			
1,230,075	1,230,075	1	G -> A	79	SNP (transition)	98.7%							5.0E-264			
1,230,080	1,230,080	1	A -> T	79	SNP (transversion)	100.0%							2.5E-269			
1,230,083	1,230,082	0	CCGAAGGGCGT	78 -> 81	Insertion	96.2% -> 96.3%							1.3E-251			
1,230,085	1,230,085	1	G -> A	76	SNP (transition)	100.0%							1.0E-266			
1,230,087	1,230,088	2	TT -> AC	76	Substitution	98.7%							2.4E-261			
1,230,090	1,230,090	1	C -> G	78	SNP (transversion)	100.0%							1.0E-273			
1,230,099	1,230,099	1	G -> C	78	SNP (transversion)	98.7%	A -> G	764	GCC -> GGC	C770_RS47585	GntR family transcriptio	Substitution	6.2E-253	255	2	WP_017276119.1
1,230,101	1,230,102	2	GG -> TT	80 -> 81	Substitution	97.5%	P -> Q	761	CCC -> CAA	C770_RS47585	GntR family transcriptio	Substitution	8.1E-266	254	2	WP_017276119.1
1,230,110	1,230,110	1	G -> A	80	SNP (transition)	100.0%		753	CAC -> CAT	C770_RS47585	GntR family transcriptio	None	1.0E-256	251	3	WP_017276119.1
1,230,117	1,230,119	3	CTT -> TCG	78	Substitution	100.0%	VR -> VE	744	GTA,AGG -> GTC	C770_RS47585	GntR family transcriptio	Substitution	6.3E-266	248	3	WP_017276119.1
1,230,137	1,230,137	1	G -> A	78	SNP (transition)	100.0%		726	CGC -> CGT	C770_RS47585	GntR family transcriptio	None	1.0E-273	242	3	WP_017276119.1
1,230,140	1,230,140	1	G -> T	81	SNP (transversion)	96.3%		723	GCC -> GCA	C770_RS47585	GntR family transcriptio	None	5.4E-261	241	3	WP_017276119.1
1,230,146	1,230,146	1	G -> A	81	SNP (transition)	100.0%		717	CTC -> CTT	C770_RS47585	GntR family transcriptio	None	5.0E-268	239	3	WP_017276119.1
1,230,155	1,230,155	1	C -> G	81	SNP (transversion)	100.0%		708	CTG -> CTC	C770_RS47585	GntR family transcriptio	None	5.0E-268	236	3	WP_017276119.1
1,230,161	1,230,162	2	GC -> AT	83	Substitution	98.8%	C -> Y	701	TGC -> TAT	C770_RS47585	GntR family transcriptio	Substitution	6.3E-283	234	2	WP_017276119.1
1,230,171	1,230,172	2	GC -> CT	81	Substitution	100.0%	A -> S	691	GCC -> AGC	C770_RS47585	GntR family transcriptio	Substitution	4.0E-276	231	1	WP_017276119.1
1,230,176	1,230,176	1	T -> C	81	SNP (transition)	100.0%		687	GCA -> GCG	C770_RS47585	GntR family transcriptio	None	4.0E-276	229	3	WP_017276119.1
1,230,182	1,230,182	1	T -> A	83	SNP (transversion)	98.8%		681	GCA -> GCT	C770_RS47585	GntR family transcriptio	None	8.3E-286	227	3	WP_017276119.1
1,230,203	1,230,203	1	C -> T	83	SNP (transition)	100.0%		660	GCG -> GCA	C770_RS47585	GntR family transcriptio	None	1.3E-274	220	3	WP_017276119.1
1,230,338	1,230,338	1	C -> T	84	SNP (transition)	98.8%		525	AAG -> AAA	C770_RS47585	GntR family transcriptio	None	2.1E-264	175	3	WP_017276119.1
1,230,401	1,230,401	1	G -> A	77	SNP (transition)	98.7%		462	GAC -> GAT	C770_RS47585	GntR family transcriptio	None	7.7E-265	154	3	WP_017276119.1
1,230,428	1,230,428	1	A -> G	76	SNP (transition)	100.0%		435	ATT -> ATC	C770_RS47585	GntR family transcriptio	None	1.0E-266	145	3	WP_017276119.1
1,230,455	1,230,455	1	A -> G	75	SNP (transition)	100.0%		408	CGT -> CGC	C770_RS47585	GntR family transcriptio	None	1.0E-270	136	3	WP_017276119.1
1,230,476	1,230,476	1	A -> C	78	SNP (transversion)	98.7%		387	GCT -> GCG	C770_RS47585	GntR family transcriptio	None	1.2E-260	129	3	WP_017276119.1
1,230,482	1,230,482	1	C -> T	76	SNP (transition)	100.0%		381	CCG -> CCA	C770_RS47585	GntR family transcriptio	None	1.0E-266	127	3	WP_017276119.1
1,230,826	1,230,826	1	T -> C	79	SNP (transition)	100.0%	T -> A	37	ACG -> GCG	C770_RS47585	GntR family transcriptio	Substitution	2.0E-261	13	1	WP_017276119.1
1,230,891	1,230,891	1	G -> A	78	SNP (transition)	100.0%							4.0E-258			
1,230,899	1,230,899	1	T -> A	78	SNP (transversion)	100.0%							1.0E-273			
1,230,961	1,230,961	1	A -> C	96	SNP (transversion)	100.0%							0.0			
1,230,976	1,230,976	1	A -> C	97	SNP (transversion)	100.0%							0.0			
1,231,072	1,231,072	1	G -> A	90	SNP (transition)	98.9%	A -> T	22	GCT -> ACT	C770_RS47590	sugar ABC transporter s	Substitution	1.8E-292	8	1	WP_015243316.1
1,231,306	1,231,306	1	G -> A	76	SNP (transition)	98.7%	V -> I	256	GTC -> ATC	C770_RS47590	sugar ABC transporter s	Substitution	7.6E-254	86	1	WP_015243316.1
1,231,353	1,231,354	2	GG -> CA	80 -> 81	Substitution	97.5%	AA -> AT	303	GCG,GCA -> GCC	C770_RS47590	sugar ABC transporter s	Substitution	8.1E-255	101	3	WP_015243316.1
1,231,386	1,231,386	1	T -> C	79	SNP (transition)	100.0%		336	GAT -> GAC	C770_RS47590	sugar ABC transporter s	None	2.5E-269	112	3	WP_015243316.1
1,231,467	1,231,467	1	G -> C	68	SNP (transversion)	100.0%		417	GCG -> GCC	C770_RS47590	sugar ABC transporter s	None	2.5E-218	139	3	WP_015243316.1
1,231,584	1,231,584	1	T -> A	85	SNP (transversion)	100.0%		534	GCT -> GCA	C770_RS47590	sugar ABC transporter s	None	1.0E-289	178	3	WP_015243316.1
1,231,701	1,231,701	1	C -> G	86	SNP (transversion)	100.0%		651	GCC -> GCG	C770_RS47590	sugar ABC transporter s	None	6.3E-276	217	3	WP_015243316.1
1,231,875	1,231,875	1	A -> C	86	SNP (transversion)	100.0%		825	ACA -> ACC	C770_RS47590	sugar ABC transporter s	None	6.3E-276	275	3	WP_015243316.1
1,231,914	1,231,915	2	TC -> CG	79	Substitution	98.7%	AL -> AV	864	GCT,CTC -> GCC,	C770_RS47590	sugar ABC transporter s	Substitution	2.0E-248	288	3	WP_015243316.1
1,232,063	1,232,063	1	T -> C	65	SNP (transition)	98.5%							1.6E-216			
1,232,071	1,232,071	1	-C	63	Deletion	100.0%							7.9E-108			
1,232,107	1,232,107	1	C -> T	58	SNP (transition)	100.0%		9	GAC -> GAT	C770_RS47595	sugar ABC transporter A	None	2.5E-186	3	3	WP_015243317.1
1,232,149	1,232,149	1	T -> C	59	SNP (transition)	100.0%		51	GTT -> GTC	C770_RS47595	sugar ABC transporter A	None	2.5E-201	17	3	WP_015243317.1
1,232,162	1,232,162	1	G -> A	63	SNP (transition)	100.0%	E -> K	64	GAA -> AAA	C770_RS47595	sugar ABC transporter A	Substitution	6.3E-215	22	1	WP_015243317.1
1,232,170	1,232,170	1	C -> A	64	SNP (transversion)	100.0%		72	TCC -> TCA	C770_RS47595	sugar ABC transporter A	None	2.5E-218	24	3	WP_015243317.1
1,232,287	1,232,288	2	CG -> AA	72	Substitution	97.2%	ID -> IN	189	ATC,GAC -> ATA,	C770_RS47595	sugar ABC transporter A	Substitution	4.5E-226	63	3	WP_015243317.1
1,232,362	1,232,362	1	T -> G	77	SNP (transversion)	100.0%		264	CTT -> CTG	C770_RS47595	sugar ABC transporter A	None	3.2E-270	88	3	WP_015243317.1
1,232,710	1,232,710	1	C -> T	70	SNP (transition)	100.0%		612	TTC -> TTT	C770_RS47595	sugar ABC transporter A	None	1.0E-238	204	3	WP_015243317.1
1,232,713	1,232,714	2	CT -> TC	69 -> 70	Substitution	100.0%		615	CGC,TTG -> CGT,	C770_RS47595	sugar ABC transporter A	None	1.0E-245	205	3	WP_015243317.1
1,232,720	1,232,720	1	G -> A	72	SNP (transition)	100.0%	D -> N	622	GAC -> AAC	C770_RS47595	sugar ABC transporter A	Substitution	1.0E-252	208	1	WP_015243317.1
1,232,743	1,232,743	1	T -> C	70	SNP (transition)	98.6%		645	GAT -> GAC	C770_RS47595	sugar ABC transporter A	None	2.2E-240	215	3	WP_015243317.1
1,232,852	1,232,855	3	CCCA -> ACGC	67	Substitution	98.5%	PI -> TL	754	CCC,ATC -> ACG,	C770_RS47595	sugar ABC transporter A	Substitution	1.1E-216	252	1	WP_015243317.1
1,232,862	1,232,862	1	C -> T	70	SNP (transition)	100.0%	A -> V	764	GCC -> GTC	C770_RS47595	sugar ABC transporter A	Substitution	1.0E-231	255	2	WP_015243317.1

1,232,878	1,232,878	1	T -> C		76	SNP (transition)	98.7%		780	GTT -> GTC	C770_RS47595	sugar ABC transporter A	None	2.4E-246	260	3	WP_015243317.1
1,232,882	1,232,882	1	G -> C		76	SNP (transversion)	98.7%	G -> R	784	GGC -> CGC	C770_RS47595	sugar ABC transporter A	Substitution	7.6E-254	262	1	WP_015243317.1
1,232,898	1,232,898	1	C -> A		75	SNP (transversion)	100.0%	T -> N	800	ACC -> AAC	C770_RS47595	sugar ABC transporter A	Substitution	3.2E-233	267	2	WP_015243317.1
1,232,908	1,232,908	1	A -> G		76	SNP (transition)	98.7%		810	CAA -> CAG	C770_RS47595	sugar ABC transporter A	None	2.4E-231	270	3	WP_015243317.1
1,232,928	1,232,928	1	A -> G		81	SNP (transition)	98.8%	H -> R	830	CAC -> CGC	C770_RS47595	sugar ABC transporter A	Substitution	8.1E-263	277	2	WP_015243317.1
1,232,930	1,232,932	2	ACT -> GCC		79	Substitution	98.7%	T -> A	832	ACT -> GCC	C770_RS47595	sugar ABC transporter A	Substitution	3.1E-256	278	1	WP_015243317.1
1,232,959	1,232,959	1	G -> C		84	SNP (transversion)	100.0%		861	CTG -> CTC	C770_RS47595	sugar ABC transporter A	None	6.3E-278	287	3	WP_015243317.1
1,233,238	1,233,238	1	C -> G		88	SNP (transversion)	100.0%		1.14	GCC -> GCG	C770_RS47595	sugar ABC transporter A	None	6.3E-300	380	3	WP_015243317.1
1,233,469	1,233,469	1	G -> C		85	SNP (transversion)	100.0%		1.371	CCG -> CCC	C770_RS47595	sugar ABC transporter A	None	1.0E-272	457	3	WP_015243317.1
1,233,514	1,233,514	1	C -> T		87	SNP (transition)	100.0%		1.416	GGC -> GGT	C770_RS47595	sugar ABC transporter A	None	3.2E-305	472	3	WP_015243317.1
1,233,753	1,233,753	1	G -> A		94	SNP (transition)	96.8%		126	CTG -> CTA	C770_RS47600	sugar ABC transporter p	None	4.2E-314	42	3	WP_015243318.1
1,233,807	1,233,807	1	G -> C		87	SNP (transversion)	100.0%		180	CTG -> CTC	C770_RS47600	sugar ABC transporter p	None	1.6E-296	60	3	WP_015243318.1
1,233,897	1,233,897	1	A -> C		86	SNP (transversion)	100.0%		270	ATA -> ATC	C770_RS47600	sugar ABC transporter p	None	1.6E-284	90	3	WP_015243318.1
1,234,176	1,234,176	1	C -> T		65	SNP (transition)	100.0%		549	GTC -> GTT	C770_RS47600	sugar ABC transporter p	None	3.2E-215	183	3	WP_015243318.1
1,234,182	1,234,182	1	C -> A		65	SNP (transversion)	98.5%		555	GGC -> GGA	C770_RS47600	sugar ABC transporter p	None	2.6E-197	185	3	WP_015243318.1
1,234,236	1,234,236	1	G -> C		65	SNP (transversion)	100.0%		609	GTG -> GTC	C770_RS47600	sugar ABC transporter p	None	1.0E-208	203	3	WP_015243318.1
1,234,272	1,234,272	1	C -> G		73	SNP (transversion)	100.0%		645	CTC -> CTG	C770_RS47600	sugar ABC transporter p	None	1.3E-241	215	3	WP_015243318.1
1,234,287	1,234,287	1	C -> T		72	SNP (transition)	98.6%		660	TGC -> TGT	C770_RS47600	sugar ABC transporter p	None	3.6E-233	220	3	WP_015243318.1
1,234,296	1,234,296	1	A -> T		72	SNP (transversion)	100.0%		669	GCA -> GCT	C770_RS47600	sugar ABC transporter p	None	4.0E-231	223	3	WP_015243318.1
1,234,302	1,234,302	1	G -> C		70	SNP (transversion)	100.0%		675	GTG -> GTC	C770_RS47600	sugar ABC transporter p	None	1.0E-231	225	3	WP_015243318.1
1,234,326	1,234,326	1	G -> A		72	SNP (transition)	100.0%		699	TCG -> TCA	C770_RS47600	sugar ABC transporter p	None	1.6E-245	233	3	WP_015243318.1
1,234,332	1,234,332	1	A -> G		70	SNP (transition)	100.0%		705	CAA -> CAG	C770_RS47600	sugar ABC transporter p	None	1.0E-231	235	3	WP_015243318.1
1,234,350	1,234,350	1	T -> C		70	SNP (transition)	100.0%		723	GGT -> GGC	C770_RS47600	sugar ABC transporter p	None	1.0E-224	241	3	WP_015243318.1
1,234,425	1,234,425	1	C -> A		69	SNP (transversion)	98.6%		798	GGC -> GGA	C770_RS47600	sugar ABC transporter p	None	6.9E-237	266	3	WP_015243318.1
1,234,434	1,234,434	1	A -> C		69	SNP (transversion)	100.0%		807	GTA -> GTC	C770_RS47600	sugar ABC transporter p	None	2.5E-235	269	3	WP_015243318.1
1,234,476	1,234,476	1	G -> T		66	SNP (transversion)	100.0%		849	CTG -> CTT	C770_RS47600	sugar ABC transporter p	None	1.0E-231	283	3	WP_015243318.1
1,234,515	1,234,515	1	C -> T		61	SNP (transition)	98.4%		888	GGC -> GGT	C770_RS47600	sugar ABC transporter p	None	6.1E-209	296	3	WP_015243318.1
1,234,548	1,234,548	1	T -> C		59	SNP (transition)	100.0%		921	AAT -> AAC	C770_RS47600	sugar ABC transporter p	None	4.0E-213	307	3	WP_015243318.1
1,234,566	1,234,566	1	C -> T		60	SNP (transition)	100.0%		939	GGC -> GGT	C770_RS47600	sugar ABC transporter p	None	1.0E-210	313	3	WP_015243318.1
1,234,582	1,234,581	0	A		57	Insertion	98.2%							3.6E-178			
1,234,593	1,234,593	1	G -> T		55	SNP (transversion)	100.0%							1.0E-187			
1,234,639	1,234,639	1	G -> A		60	SNP (transition)	100.0%	R -> C	895	CGC -> TGC	C770_RS47605	aminoglycoside phosph	Substitution	1.0E-204	299	1	WP_015243319.1
1,234,664	1,234,664	1	C -> T		60	SNP (transition)	98.3%		870	TTG -> TTA	C770_RS47605	aminoglycoside phosph	None	1.5E-199	290	3	WP_015243319.1
1,234,676	1,234,676	1	A -> G		58	SNP (transition)	100.0%		858	AGT -> AGC	C770_RS47605	aminoglycoside phosph	None	2.5E-186	286	3	WP_015243319.1
1,234,840	1,234,840	1	T -> A		69	SNP (transversion)	100.0%	I -> F	694	ATC -> TTC	C770_RS47605	aminoglycoside phosph	Substitution	2.5E-235	232	1	WP_015243319.1
1,234,872	1,234,872	1	T -> A		73	SNP (transversion)	100.0%	D -> V	662	GAT -> GTT	C770_RS47605	aminoglycoside phosph	Substitution	2.5E-234	221	2	WP_015243319.1
1,234,886	1,234,888	2	CCT -> TCC	74 -> 75		Substitution	100.0%	R -> G	646	AGG -> GGA	C770_RS47605	aminoglycoside phosph	Substitution	4.0E-230	216	1	WP_015243319.1
1,234,896	1,234,896	1	A -> G		77	SNP (transition)	100.0%	V -> A	638	GTT -> GCT	C770_RS47605	aminoglycoside phosph	Substitution	1.6E-262	213	2	WP_015243319.1
1,234,942	1,234,942	1	A -> G		79	SNP (transition)	100.0%		592	TTG -> CTG	C770_RS47605	aminoglycoside phosph	None	2.5E-269	198	1	WP_015243319.1
1,235,020	1,235,020	1	T -> C		88	SNP (transition)	100.0%	N -> D	514	AAT -> GAT	C770_RS47605	aminoglycoside phosph	Substitution	2.5E-282	172	1	WP_015243319.1
1,235,047	1,235,047	1	T -> C		92	SNP (transition)	100.0%	I -> V	487	ATC -> GTC	C770_RS47605	aminoglycoside phosph	Substitution	1.6E-313	163	1	WP_015243319.1
1,235,102	1,235,102	1	T -> A		90	SNP (transversion)	97.8%		432	CCA -> CCT	C770_RS47605	aminoglycoside phosph	None	2.5E-296	144	3	WP_015243319.1
1,235,120	1,235,121	2	AG -> GA	93 -> 94		Substitution	98.9%	A -> V	413	GCT -> GTC	C770_RS47605	aminoglycoside phosph	Substitution	5.9E-315	138	2	WP_015243319.1
1,235,194	1,235,194	1	T -> C		92	SNP (transition)	100.0%	I -> V	340	ATC -> GTC	C770_RS47605	aminoglycoside phosph	Substitution	1.0E-322	114	1	WP_015243319.1
1,235,197	1,235,197	1	G -> C		91	SNP (transversion)	100.0%	L -> V	337	CTC -> GTC	C770_RS47605	aminoglycoside phosph	Substitution	4.0E-310	113	1	WP_015243319.1
1,235,381	1,235,381	1	G -> C		91	SNP (transversion)	100.0%		153	GGC -> GGG	C770_RS47605	aminoglycoside phosph	None	4.0E-310	51	3	WP_015243319.1
1,235,386	1,235,386	1	T -> C		89	SNP (transition)	100.0%	T -> A	148	ACC -> GCC	C770_RS47605	aminoglycoside phosph	Substitution	2.0E-294	50	1	WP_015243319.1
1,235,437	1,235,437	1	C -> G		83	SNP (transversion)	100.0%	A -> P	97	GCC -> CCC	C770_RS47605	aminoglycoside phosph	Substitution	1.3E-274	33	1	WP_015243319.1
1,235,444	1,235,446	1	AGG -> TGGG	85 -> 88		Insertion	100.0%		88		C770_RS47605	aminoglycoside phosph	Frame Shift	3.2E-281	30	1	WP_015243319.1
1,235,466	1,235,466	1	G -> T		86	SNP (transversion)	98.8%	A -> D	68	GCC -> GAC	C770_RS47605	aminoglycoside phosph	Substitution	8.6E-288	23	2	WP_015243319.1
1,235,474	1,235,476	2	CCC -> GCT	82 -> 83		Substitution	100.0%	G -> S	58	GGG -> AGC	C770_RS47605	aminoglycoside phosph	Substitution	5.0E-258	20	1	WP_015243319.1
1,235,494	1,235,494	1	G -> T		82	SNP (transversion)	98.8%	L -> M	40	CTG -> ATG	C770_RS47605	aminoglycoside phosph	Substitution	5.2E-258	14	1	WP_015243319.1
1,235,537	1,235,537	1	G -> C		76	SNP (transversion)	100.0%							4.0E-259			
1,235,607	1,235,607	1	T -> C		77	SNP (transition)	100.0%							1.6E-262			
1,235,681	1,235,681	1	G -> A		75	SNP (transition)	100.0%	A -> T	25	GCC -> ACC	C770_RS47610	sugar ABC transporter s	Substitution	1.0E-255	9	1	WP_015243320.1
1,235,956	1,235,956	1	G -> C		96	SNP (transversion)	100.0%		300	CCG -> CCC	C770_RS47610	sugar ABC transporter s	None	0.0	100	3	WP_015243320.1
1,236,004	1,236,004	1	G -> T		96	SNP (transversion)	100.0%	Q -> H	348	CAG -> CAT	C770_RS47610	sugar ABC transporter s	Substitution	0.0	116	3	WP_015243320.1
1,236,379	1,236,379	1	T -> C		87	SNP (transition)	98.9%		723	GGT -> GGC	C770_RS47610	sugar ABC transporter s	None	3.5E-291	241	3	WP_015243320.1
1,236,457	1,236,457	1	A -> G		88	SNP (transition)	100.0%		801	TCA -> TCG	C770_RS47610	sugar ABC transporter s	None	6.3E-300	267	3	WP_015243320.1
1,236,463	1,236,463	1	T -> C		88	SNP (transition)	100.0%		807	AAT -> AAC	C770_RS47610	sugar ABC transporter s	None	2.5E-282	269	3	WP_015243320.1
1,236,466	1,236,466	1	T -> C		87	SNP (transition)	100.0%		810	CCT -> CCC	C770_RS47610	sugar ABC transporter s	None	4.0E-279	270	3	WP_015243320.1
1,236,538	1,236,538	1	A -> T		80	SNP (transversion)	98.8%		882	CCA -> CCT	C770_RS47610	sugar ABC transporter s	None	1.6E-259	294	3	WP_015243320.1
1,236,583	1,236,583	1	C -> G		73	SNP (transversion)	100.0%		927	CCC -> CCG	C770_RS47610	sugar ABC transporter s	None	1.3E-241	309	3	WP_015243320.1
1,236,736	1,236,736	1	G -> A		69	SNP (transition)	100.0%							2.0E-228			
1,236,829	1,236,829	1	C -> T		85	SNP (transition)	100.0%	L -> F	79	CTT -> TTT	C770_RS47615	sugar ABC transporter A	Substitution	3.2E-281	27	1	WP_015243321.1
1,236,837	1,236,837	1	C -> G		83	SNP (transversion)	98.8%		87	CTC -> CTG	C770_RS47615	sugar ABC transporter A	None	2.1E-269	29	3	WP_015243321.1
1,237,023	1,237,023	1	A -> G		84	SNP (transition)	100.0%		273	GCA -> GCG	C770_RS47615	sugar ABC transporter A	None	2.5E-286	91	3	WP_015243321.1
1,237,275	1,237,275	1	G -> A		72	SNP (transition)	100.0%		525	GAG -> GAA	C770_RS47615	sugar ABC transporter A	None	1.0E-252	175	3	WP_015243321.1
1,237,336	1,237,336	1	A -> G		83	SNP (transition)	100.0%	I -> V	586	ATC -> GTC	C770_RS47615	sugar ABC transporter A	Substitution	3.2E-291	196	1	WP_015243321.1
1,237,556	1,237,555	0	CCG	93 -> 96		Insertion	100.0%	G -> AG	806	GGC -> GCC,GGC	C770_RS47615	sugar ABC transporter A	Insertion	1.3E-307	269	2	WP_015243321.1
1,237,591	1,237,591	1	T -> C		92	SNP (transition)	100.0%	Y -> H	841	TAC -> CAC	C770_RS47615	sugar ABC transporter A	Substitution	2.5E-304	281	1	WP_015243321.1
1,237,602	1,237,602	1	T -> C		94	SNP (transition)	100.0%		852	ATT -> ATC	C770_RS47615	sugar ABC transporter A	None	6.3E-311	284	3	WP_015243321.1
1,237,699	1,237,699	1	A -> G		92	SNP (transition)	100.0%	N -> D	949	AAC -> GAC	C770_RS47615	sugar ABC transporter A	Substitution	1.0E-322	317	1	WP_015243321.1
1,238,371	1,238,371	1	T -> C		67	SNP (transition)	100.0%		63	GGT -> GGC	C770_RS47620	sugar ABC transporter p	None	3.2E-235	21	3	WP_015243322.1
1,238,498	1,238,498	1	G -> A		69	SNP (transition)	100.0%	V -> I	190	GTC -> ATC	C770_RS47620	sugar ABC transporter p	Substitution	2.5E-235	64	1	WP_015243322.1
1,238,593	1,238,593	1	C -> G		77	SNP (transversion)	100.0%		285	TCC -> TCG	C770_RS47620	sugar ABC transporter p	None	1.6E-262	95	3	WP_015243322.1
1,238,871	1,238,871	1	G -> C		86	SNP (transversion)	100.0%	G -> A	563	GGC -> GCC	C770_RS47620	sugar ABC transporter p	Substitution	1.6E-284	188	2	WP_015243322.1
1,238,905	1,238,905	1	C -> A		85	SNP (transversion)	98.8%		597	GCC -> GCA	C770_RS47620	sugar ABC transporter p	None	3.4E-301	199	3	WP_015243322.1
1,238,923	1,238,923	1	T -> C		86	SNP (transition)	100.0%		615	TAT -> TAC	C770_RS47620	sugar ABC transporter p	None	4.0E-293	205	3	WP_015243322.1
1,239,001	1,239,001	1	C -> T		85	SNP (transition)	100.0%		693	TGC -> TGT	C770_RS47620	sugar ABC transporter p	None	1.0E-289	231	3	WP_015243322.1
1,239,022	1,239,022	1	T -> G		88	SNP (transversion)	100.0%		714	GCT -> GCG	C770_RS47620	sugar ABC transporter p	None	2.5E-282	238	3	WP_015243322.1
1,239,118	1,239,118	1	T -> C		93	SNP (transition)	100.0%		810	GCT -> GCC	C770_RS47620	sugar ABC transporter p	None	2.5E-298	270	3	WP_015243322.1
1,239,211	1,239,211	1	C -> T		83	SNP (transition)	100.0%		903	GTC -> GTT	C770_RS47620	sugar ABC transporter p	None	3.2E-291	301	3	WP_015243322.1
1,239,302	1,239,302	1	T -> C		94	SNP (transition)	100.0%							2.5E-320			
1,239,497	1,239,497	1	T -> C		85	SNP (transition)	100.0%		195	ATT -> ATC	C770_RS47625	Crp/Fnr family transcrip	None	3.2E-298	65	3	WP_015008513.1
1,239,653	1,239,653	1	G -> A		75	SNP (transition)	100.0%		351	GAG -> GAA	C770_RS47625	Crp/Fnr family transcrip	None	1.0E-240	117	3	WP_015008513.1
1,239,674	1,239,674	1	G -> T		76	SNP (transversion)	100.0%		372	ACG -> ACT	C770_RS47625	Crp/Fnr family transcrip	None	4.0E-259	124	3	WP_015008513.1
1,239,740	1,239,740	1	C -> T		70	SNP (transition)	100.0%		438	TTC -> TTT	C770_RS47625	Crp/Fnr family transcrip	None	1.0E-224	146	3	WP_015008513.1
1,239,959	1,239,959	1	C -> G		86	SNP (transversion)	100.0%		657	GTC -> GTG	C770_RS47625	Crp/Fnr family transcrip	None	6.3E-276	219	3	WP_015008513.1
1,239,989	1,239,989	1	C -> A		90	SNP (transversion)	100.0%		687	TCC -> TCA	C770_RS47625	Crp/Fnr family transcrip	None	1.0E-288	229	3	WP_015008513.1
1,240,076	1,240,076	1	A -> G		102	SNP (transition)	100.0%		774	CTA -> CTG	C770_RS47625	Crp/Fnr family transcrip	None	0.0	258	3	WP_015008513.1
1,240,160	1,240,160	1	C -> G		109	SNP (transversion)	100.0%		858	CCC -> CCG	C770_RS47625	Crp/Fnr family transcrip	None	0.0	286	3	WP_015008513.1
1,240,611	1,240,611	1	A -> G		79	SNP (transition)	100.0%	D -> G	308	GAC -> GGC	C770_RS47630	oxidoreductase (related	Substitution	2.5E-269	103	2	WP_015243323.1
1,240,796	1,240,796	1	G -> A		83	SNP (transition)	96.4%	V -> I	493	GTC -> ATC	C770_RS47630	oxidoreductase (related	Substitution	9.2E-260	165	1	WP_015243323.1
1,240,819	1,240,819	1	G -> C		79	SNP (transversion)	100.0%		516	CTG -> CTC	C770_RS47630	oxidoreductase (related	None	2.5E-269	172	3	WP_015243323.1
1,240,829	1,240,831	2	CAG -> GAA	79 -> 80		Substitution	100.0%	Q -> E	526	CAG -> GAA	C770_RS47630	oxidoreductase (related	Substitution	2.0E-261	176	1	WP_015243323.1
1,240,864	1,240,864	1	C -> A		80	SNP (transversion)	98.8%		561	ATC -> ATA	C770_RS47630	oxidoreductase (related	None	2.5E-275	187	3	WP_015243323.1
1,240,893	1,240,893	1	G -> A		77	SNP (transition)	100.0%	G -> E	590	GGG -> GAG	C770_RS47630	oxidoreductase (related	Substitution	4.0E-247	197	2	WP_015243323.1
1,240,991	1,240,991	1	G -> C		84	SNP (transversion)	100.0%	A -> P	688	GCC -> CCC	C770_RS47630	oxidoreductase (related	Substitution	1.6E-269	230	1	WP_015243323.1
1,241,065	1,241,065	1	C -> G		78	SNP (transversion)	100.0%		762	GTC -> GTG	C770_RS47630	oxidoreductase (related	None	4.0E-258	254	3	WP_015243323.1
1,241,104	1,241,105	2	GC -> AT	82 -> 83		Substitution	98.8%	EH -> EY	801	GAG,CAC -> GAA	C770_RS47630	oxidoreductase (related	Substitution	5.2E-258	267	3	WP_015243323.1
1,241,169	1,241,169	1	C -> T		83	SNP (transition)	98.8%	A -> V	866	GCG -> GTG	C770_RS47630	oxidoreductase (related	Substitution	1.3E-277	289	2	WP_015243323.1
1,241,283	1,241,283	1	T -> C		72	SNP (transition)	100.0%	V -> A	980	GTA -> GCA	C770_RS47630	oxidoreductase (related	Substitution	1.0E-252	327	2	WP_015243323.1
1,241,345	1,241,345	1	A -> G		67	SNP (transition)	98.5%	Q -> R	35	CAG -> CGG	C770_RS47635	glycerol-3-phosphate de	Substitution	1.1E-216	12	2	WP_015243324.1
1,241,394	1,241,394	1	C -> T		67	SNP (transition)	100.0%		84	CTC -> CTT	C770_RS47635	glycerol-3-phosphate de	None	4.0E-215	28	3	WP_015243324.1
1,241,448	1,241,448	1	A -> G		65	SNP (transition)	98.5%		138	AAA -> AAG	C770_RS47635	glycerol-3-phosphate de	None	1.6E-216	46	3	WP_015243324.1
1,241,703	1,241,703	1	A -> G		96	SNP (transition)	97.9%		393	GCA -> GCG	C770_RS47635	glycerol-3-phosphate de	None	2.9E-307	131	3	WP_015243324.1
1,241,724	1,241,724	1	T -> C		99	SNP (transition)	100.0%		414	ATT -> ATC	C770_RS47635	glycerol-3-phosphate de	None	0.0	138	3	WP_015243324.1
1,241,742	1,241,742	1	C -> A		99	SNP (transversion)	100.0%		432	CGC -> CGA	C770_RS47635	glycerol-3-phosphate de	None	1.3E-307	144	3	WP_015243324.1
1,241,898	1,241,898	1	A -> G		96	SNP (transition)	100.0%		588	GGA -> GGG	C770_RS47635	glycerol-3-phosphate de	None	6.3E-308	196	3	WP_015243324.1
1,241,911	1,241,911	1	C -> A		94	SNP (transversion)	100.0%	P -> T	601	CCC -> ACC	C770_RS47635	glycerol-3-phosphate de	Substitution	6.3E-311	201	1	WP_015243324.1
1,242,036	1,242,036	1	T -> C		82	SNP (transition)	100.0%		726	GGT -> GGC	C770_RS47635	glycerol-3-phosphate de	None	1.0E-287	242	3	WP_015243324.1
1,242,156	1,242,156	1	T -> C		89	SNP (transition)	100.0%		846	CTT -> CTC	C770_RS47635	glycerol-3-phosphate de	None	2.5E-303	282	3	WP_015243324.1
1,242,159	1,242,159	1	T -> C		89	SNP (transition)	100.0%		849	GGT -> GGC	C770_RS47635	glycerol-3-phosphate de	None	3.2E-312	283	3	WP_015243324.1
1,242,189	1,242,189	1	T -> C		87	SNP (transition)	98.9%		879	GAT -> GAC	C770_RS47635	glycerol-3-phosphate de	None	3.5E-291	293	3	WP_015243324.1
1,242,351	1,242,351	1	C -> G		77	SNP (transversion)	100.0%	D -> E	1.041	GAC -> GAG	C770_RS47635	glycerol-3-phosphate de	Substitution	7.9E-255	347	3	WP_015243324.1
1,242,471	1,242,471	1	A -> T		82	SNP (transversion)	100.0%		1.161	CTA -> CTT	C770_RS47635	glycerol-3-phosphate de	None	1.0E-287	387	3	WP_015243324.1
1,242,504	1,242,504	1	C -> T		80	SNP (transition)	100.0%		1.194	CGC -> CGT	C770_RS47635	glycerol-3-phosphate de	None	1.0E-264	398	3	WP_015243324.1
1,242,533	1,242,533	1	C -> T		81	SNP (transition)	96.3%	A -> V	1.223	GCG -> GTG	C770_RS47635	glycerol-3-phosphate de	Substitution	8.5E-269	408	2	WP_015243324.1
1,242,563	1,242,563	1	T -> C		83	SNP (transition)	100.0%	V -> A	1.253	GTC -> GCC	C770_RS47635	glycerol-3-phosphate de	Substitution	1.3E-274	418	2	WP_015243324.1
1,242,611	1,242,611	1	C -> A		82	SNP (transversion)	100.0%	A -> D	1.301	GCC -> GAC	C770_RS47635	glycerol-3-phosphate de	Substitution	2.5E-230	434	2	WP_015243324.1
1,242,699	1,242,699	1	G -> A		82	SNP (transition)	100.0%		1.389	GAG -> GAA	C770_RS47635	glycerol-3-phosphate de	None	2.5E-271	463	3	WP_015243324.1
1,242,744	1,242,744	1	G -> A		88	SNP (transition)	98.9%		1.434	GCG -> GCA	C770_RS47635	glycerol-3-phosphate de	None	3.5E-277	478	3	WP_015243324.1
1,242,749	1,242,749	1	C -> T		88	SNP (transition)	98.9%	P -> L	1.439	CCT -> CTT	C770_RS47635	glycerol-3-phosphate de	Substitution	7.0E-286	480	2	WP_015243324.1
1,242,822	1,242,822	1	A -> G		91	SNP (transition)	100.0%		1.512	CTA -> CTG	C770_RS47635	glycerol-3-phosphate de	None	0.0	504	3	WP_015243324.1
1,242,924	1,242,924	1	G -> A		100	SNP (transition)	99.0%		1.614	GCG -> GCA	C770_RS47635	glycerol-3-phosphate de	None	0.0	538	3	WP_015243324.1
1,242,931	1,242,931	1	T -> C		98	SNP (transition)	99.0%		1.621	TTG -> CTG	C770_RS47635	glycerol-3-phosphate de	None	7.8E-319	541	1	WP_015243324.1
1,243,010	1,243,010	1	C -> G		94	SNP (transversion)	100.0%	P -> R	1.7	CCG -> CGG	C770_RS47635	glycerol-3-phosphate de	Substitution	2.5E-320	567	2	WP_015243324.1
1,243,061	1,243,061	1	T -> C		98	SNP (transition)	100.0%							0.0			
1,243,063	1,243,063	1	C -> T		99	SNP (transition)	100.0%							0.0			
1,243,070	1,243,070	1	G -> A		98	SNP (transition)	100.0%							4.9E-324			
1,243,072	1,243,072	1	A -> T		99	SNP (transversion)	100.0%							0.0			
1,243,077	1,243,077	1	T -> G		101	SNP (transversion)	100.0%							0.0			
1,243,106	1,243,106	1	A -> CT	94 -> 95		Insertion	98.9%							2.4E-318			
1,243,117	1,243,117	1	C -> T		94	SNP (transition)	100.0%	T -> I	8	ACC -> ATC	C770_RS47640	fructose-bisphosphate a	Substitution	2.5E-320	3	2	WP_015243325.1
1,243,152	1,243,152	1	G -> C		87	SNP (transversion)	100.0%	G -> R	43	GGC -> CGC	C770_RS47640	fructose-bisphosphate a	Substitution	3.2E-305	15	1	WP_015243325.1
1,243,172	1,243,172	1	T -> C		83	SNP (transition)	100.0%		63	GTT -> GTC	C770_RS47640	fructose-bisphosphate a	None	1.6E-299	21	3	WP_015243325.1
1,243,560	1,243,560	1	T -> C		114	SNP (transition)	100.0%		451	TTG -> CTG	C770_RS47640	fructose-bisphosphate a	None	0.0	151	1	WP_015243325.1
1,243,691	1,243,691	1	T -> C		93	SNP (transition)	100.0%		582	GAT -> GAC	C770_RS47640	fructose-bisphosphate a	None	2.5E-298	194	3	WP_015243325.1
1,243,960	1,243,960	1	A -> C		62	SNP (transversion)	100.0%							4.0E-199			
1,244,194	1,244,194	1	T -> G		67	SNP (transversion)	98.5%	V -> G	212	GTC -> GGC	C770_RS47645	Sugar (pentulose and he	Substitution	1.7E-203	71	2	WP_015243326.1
1,244,219	1,244,219	1	A -> C		66	SNP (transversion)	100.0%		237	GCA -> GCC	C770_RS47645	Sugar (pentulose and he	None	2.5E-205	79	3	WP_015243326.1
1,244,273	1,244,273	1	G -> C		66	SNP (transversion)	100.0%		291	GGG -> GGC	C770_RS47645	Sugar (pentulose and he	None	4.0E-225	97	3	WP_015243326.1
1,244,413	1,244,413	1	A -> T		70	SNP (transversion)	98.6%	Y -> F	431	TAC -> TTC	C770_RS47645	Sugar (pentulose and he	Substitution	1.1E-219	144	2	WP_015243326.1
1,244,607	1,244,607	1	C -> T		70	SNP (transition)	95.7%	P -> S	625	CCG -> TCG	C770_RS47645	Sugar (pentulose and he	Substitution	4.3E-217	209	1	WP_015243326.1
1,244,615	1,244,615	1	G -> A		71	SNP (transition)	98.6%		633	GTG -> GTA	C770_RS47645	Sugar (pentulose and he	None	7.1E-237	211	3	WP_015243326.1
1,244,663	1,244,663	1	C -> G		65	SNP (transversion)	100.0%		681	GGC -> GGG	C770_RS47645	Sugar (pentulose and he	None	3.2E-215	227	3	WP_015243326.1
1,244,934	1,244,934	1	T -> G		77	SNP (transversion)	100.0%	S -> A	952	TCC -> GCC	C770_RS47645	Sugar (pentulose and he	Substitution	7.9E-255	318	1	WP_015243326.1
1,244,995	1,244,995	1	C -> T		80	SNP (transition)	100.0%	S -> F	1.013	TCC -> TTC	C770_RS47645	Sugar (pentulose and he	Substitution	1.0E-264	338	2	WP_015243326.1
1,245,041	1,245,041	1	T -> C		90	SNP (transition)	100.0%		1.059	GAT -> GAC	C770_RS47645	Sugar (pentulose and he	None	1.0E-315	353	3	WP_015243326.1
1,245,050	1,245,050	1	T -> G		93	SNP (transversion)	98.9%		1.068	GCT -> GCG	C770_RS47645	Sugar (pentulose and he	None	1.5E-311	356	3	WP_015243326.1
1,245,140	1,245,140	1	C -> G		108	SNP (transversion)	99.1%	H -> Q	1.158	CAC -> CAG	C770_RS47645	Sugar (pentulose and he	Substitution	0.0	386	3	WP_015243326.1
1,245,164	1,245,164	1	C -> A		105	SNP (transversion)	98.1%		1.182	GCC -> GCA	C770_RS47645	Sugar (pentulose and he	None	0.0	394	3	WP_015243326.1
1,245,380	1,245,380	1	C -> T		88	SNP (transition)	100.0%		1.398	TAC -> TAT	C770_RS47645	Sugar (pentulose and he	None	6.3E-300	466	3	WP_015243326.1
1,245,402	1,245,402	1	G -> C		85	SNP (transversion)	100.0%	A -> P	1.42	GCG -> CCG	C770_RS47645	Sugar (pentulose and he	Substitution	3.2E-281	474	1	WP_015243326.1
1,245,489	1,245,489	1	G -> A		84	SNP (transition)	98.8%	V -> I	1.507	GTC -> ATC	C770_RS47645	Sugar (pentulose and he	Substitution	5.3E-281	503	1	WP_015243326.1
1,245,508	1,245,508	1	C -> T		78	SNP (transition)	100.0%	A -> V	1.526	GCG -> GTG	C770_RS47645	Sugar (pentulose and he	Substitution	1.0E-273	509	2	WP_015243326.1
1,245,559	1,245,559	1	C -> T		81	SNP (transition)	98.8%		39	TTC -> TTT	C770_RS47650	choline dehydrogenase	None	8.1E-263	13	3	WP_041169903.1
1,245,616	1,245,616	1	T -> C		80	SNP (transition)	100.0%		96	GAT -> GAC	C770_RS47650	choline dehydrogenase	None	1.0E-272	32	3	WP_041169903.1
1,245,634	1,245,635	2	CT -> GC	84 -> 85		Substitution	98.8%		114	CTC,TTG -> CTG,(	C770_RS47650	choline dehydrogenase	None	8.5E-293	38	3	WP_041169903.1

1,245,669	1,245,669	1	T -> G		82	SNP (transversion)	100.0%	L -> W	149	TTG -> TGG	C770_RS47650	choline dehydrogenase	Substitution	2.5E-271	50	2	WP_041169903.1
1,245,832	1,245,832	1	T -> C		76	SNP (transition)	100.0%		312	CGT -> CGC	C770_RS47650	choline dehydrogenase	None	1.0E-266	104	3	WP_041169903.1
1,245,835	1,245,835	1	G -> C		76	SNP (transversion)	100.0%		315	GGG -> GGC	C770_RS47650	choline dehydrogenase	None	4.0E-259	105	3	WP_041169903.1
1,246,030	1,246,030	1	A -> G		73	SNP (transition)	100.0%		510	GAA -> GAG	C770_RS47650	choline dehydrogenase	None	1.0E-219	170	3	WP_041169903.1
1,246,114	1,246,114	1	C -> T		82	SNP (transition)	96.3%		594	GGC -> GGT	C770_RS47650	choline dehydrogenase	None	1.4E-248	198	3	WP_041169903.1
1,246,154	1,246,154	1	A -> G		89	SNP (transition)	100.0%	M -> V	634	ATG -> GTG	C770_RS47650	choline dehydrogenase	Substitution	2.0E-294	212	1	WP_041169903.1
1,246,253	1,246,253	1	T -> C		97	SNP (transition)	97.9%	C -> R	733	TGC -> CGC	C770_RS47650	choline dehydrogenase	Substitution	4.7E-320	245	1	WP_041169903.1
1,246,264	1,246,264	1	C -> G		93	SNP (transversion)	98.9%		744	GTC -> GTG	C770_RS47650	choline dehydrogenase	None	2.3E-302	248	3	WP_041169903.1
1,246,435	1,246,435	1	G -> C		80	SNP (transversion)	100.0%		915	ACG -> ACC	C770_RS47650	choline dehydrogenase	None	1.0E-272	305	3	WP_041169903.1
1,246,476	1,246,476	1	A -> T		87	SNP (transversion)	100.0%	Y -> F	956	TAT -> TTT	C770_RS47650	choline dehydrogenase	Substitution	7.9E-288	319	2	WP_041169903.1
1,246,590	1,246,590	1	C -> T		89	SNP (transition)	100.0%	A -> V	1.07	GCC -> GTC	C770_RS47650	choline dehydrogenase	Substitution	3.2E-312	357	2	WP_041169903.1
1,246,858	1,246,858	1	A -> G		104	SNP (transition)	100.0%		1.338	AGA -> AGG	C770_RS47650	choline dehydrogenase	None	0.0	446	3	WP_041169903.1
1,246,882	1,246,882	1	G -> A		102	SNP (transition)	100.0%		1.362	TCG -> TCA	C770_RS47650	choline dehydrogenase	None	0.0	454	3	WP_041169903.1
1,246,978	1,246,978	1	G -> A		93	SNP (transition)	100.0%		1.458	CCG -> CCA	C770_RS47650	choline dehydrogenase	None	1.3E-307	486	3	WP_041169903.1
1,246,999	1,246,999	1	C -> T		89	SNP (transition)	98.9%		1.479	TCC -> TCT	C770_RS47650	choline dehydrogenase	None	5.6E-298	493	3	WP_041169903.1
1,247,135	1,247,135	1	C -> A		81	SNP (transversion)	100.0%							3.2E-284			
1,247,221	1,247,221	1	T -> G		67	SNP (transversion)	100.0%							1.6E-228			
1,247,240	1,247,240	1	C -> T		66	SNP (transition)	100.0%		9	CGC -> CGT	C770_RS47655	hypothetical protein	None	2.5E-238	3	3	WP_041169904.1
1,247,260	1,247,260	1	G -> T		68	SNP (transversion)	100.0%	R -> L	29	CGT -> CTT	C770_RS47655	hypothetical protein	Substitution	6.3E-232	10	2	WP_041169904.1
1,247,282	1,247,282	1	G -> C		73	SNP (transversion)	100.0%		51	ACG -> ACC	C770_RS47655	hypothetical protein	None	6.3E-249	17	3	WP_041169904.1
1,247,284	1,247,283	0	A		72	Insertion	98.6%		53		C770_RS47655	hypothetical protein	Frame Shift	2.3E-247	18	2	WP_041169904.1
1,247,332	1,247,332	1	C -> T		81	SNP (transition)	100.0%	A -> V	101	GCG -> GTG	C770_RS47655	hypothetical protein	Substitution	4.0E-276	34	2	WP_041169904.1
1,247,339	1,247,339	1	T -> C		77	SNP (transition)	100.0%		108	CAT -> CAC	C770_RS47655	hypothetical protein	None	3.2E-270	36	3	WP_041169904.1
1,247,359	1,247,359	1	T		77	Deletion	100.0%		128		C770_RS47655	hypothetical protein	Frame Shift	6.3E-124	43	2	WP_041169904.1
1,247,480	1,247,480	1	G -> C		85	SNP (transversion)	98.8%							5.4E-276			
1,247,572	1,247,572	1	G -> A		89	SNP (transition)	98.9%	R -> W	1.714	CGG -> TGG	C770_RS47660	hypothetical protein	Substitution	3.5E-289	572	1	WP_015243328.1
1,247,591	1,247,591	1	C -> A		84	SNP (transversion)	100.0%		1.695	ACG -> ACT	C770_RS47660	hypothetical protein	None	1.6E-269	565	3	WP_015243328.1
1,247,681	1,247,681	1	A -> G		98	SNP (transition)	99.0%		1.605	TTT -> TTC	C770_RS47660	hypothetical protein	None	0.0	535	3	WP_015243328.1
1,247,743	1,247,743	1	T -> C		86	SNP (transition)	100.0%	R -> G	1.543	AGG -> GGG	C770_RS47660	hypothetical protein	Substitution	1.6E-284	515	1	WP_015243328.1
1,248,005	1,248,005	1	A -> C		63	SNP (transversion)	100.0%		1.281	GCT -> GCG	C770_RS47660	hypothetical protein	None	6.3E-215	427	3	WP_015243328.1
1,248,239	1,248,239	1	C -> T		63	SNP (transition)	98.4%		1.047	GCG -> GCA	C770_RS47660	hypothetical protein	None	6.3E-216	349	3	WP_015243328.1
1,248,697	1,248,697	1	G -> T		105	SNP (transversion)	100.0%	L -> I	589	CTC -> ATC	C770_RS47660	hypothetical protein	Substitution	0.0	197	1	WP_015243328.1
1,248,739	1,248,739	1	T -> C		109	SNP (transition)	100.0%	T -> A	547	ACC -> GCC	C770_RS47660	hypothetical protein	Substitution	0.0	183	1	WP_015243328.1
1,249,116	1,249,116	1	T -> G		78	SNP (transversion)	100.0%	Q -> P	170	CAG -> CCG	C770_RS47660	hypothetical protein	Substitution	4.0E-258	57	2	WP_015243328.1
1,249,236	1,249,236	1	C -> G		73	SNP (transversion)	98.6%	G -> A	50	GGG -> GCG	C770_RS47660	hypothetical protein	Substitution	7.3E-251	17	2	WP_015243328.1
1,249,701	1,249,700	0	C		78	Insertion	100.0%							4.0E-258			
1,249,808	1,249,808	1	G -> C		87	SNP (transversion)	100.0%							1.6E-296			
1,249,819	1,249,819	1	G -> A		86	SNP (transition)	96.5%							1.3E-269			
1,249,892	1,249,892	1	C -> A		96	SNP (transversion)	99.0%							9.6E-322			
1,249,918	1,249,918	1	T -> C		99	SNP (transition)	99.0%							4.0E-322			
1,249,924	1,249,924	1	C -> A		99	SNP (transversion)	99.0%							0.0			
1,249,999	1,249,999	1	C -> A		100	SNP (transversion)	100.0%							0.0			
1,250,131	1,250,131	1	G -> A		106	SNP (transition)	99.1%							0.0			
1,250,418	1,250,418	1	G -> A		94	SNP (transition)	100.0%		363	GTC -> GTT	C770_RS47670	transcriptional regulato	None	2.5E-320	121	3	WP_041169933.1
1,250,574	1,250,574	1	C -> T		92	SNP (transition)	98.9%		207	CGG -> CGA	C770_RS47670	transcriptional regulato	None	5.8E-290	69	3	WP_041169933.1
1,250,766	1,250,766	1	G -> A		28	SNP (transition)	100.0%		15	GGC -> GGT	C770_RS47670	transcriptional regulato	None	1.6E-101	5	3	WP_041169933.1
1,250,780	1,250,780	1	A		26	Deletion	100.0%		1		C770_RS47670	transcriptional regulato	Frame Shift	2.5E-42	1	1	WP_041169933.1
1,250,781	1,250,781	1	G		26	Deletion	100.0%							2.5E-42			
1,250,785	1,250,786	2	GT -> CG		25	Substitution	100.0%							1.0E-80			
1,250,791	1,250,791	1	T -> A		25	SNP (transversion)	100.0%							3.2E-78			
1,250,793	1,250,795	3	AAA -> CCG	23 -> 24		Substitution	100.0%							1.0E-72			
1,250,798	1,250,801	4	GGAA -> C		22	Deletion	100.0%							6.3E-36			
1,250,809	1,250,822	14	GAGGATTCCTTT	15 -> 20		Substitution	100.0%							1.0E-51			
1,250,824	1,250,829	6	GAGCGT -> TGA(	12 -> 15		Substitution	100.0%							2.5E-40			
1,250,831	1,250,835	5	CTGTG -> GACG(	10 -> 12		Substitution	100.0%							7.9E-35			
1,250,837	1,250,843	7	TTCCCTT -> GCG	7 -> 10		Substitution	100.0%							1.0E-21			
1,250,845	1,250,845	1	T -> A		7	SNP (transversion)	100.0%							2.5E-20			
1,250,847	1,250,847	1	C -> T		7	SNP (transition)	100.0%							2.0E-22			
1,251,812	1,251,813	2	TT -> CA	15 -> 16		Substitution	100.0%		275		C770_RS47685	transcriptional regulato	Extension	4.0E-47	92	2	WP_015243333.1
1,251,815	1,251,815	1	A -> C		17	SNP (transversion)	100.0%		273	CTT -> CTG	C770_RS47685	transcriptional regulato	None	2.0E-53	91	3	WP_015243333.1
1,251,819	1,251,827	8	GGACGTGCT -> (	18 -> 21		Substitution	100.0%	RARP -> SPSG	261	AGA,GCA,CGT,C(	C770_RS47685	transcriptional regulato	Substitution	1.0E-54	87	3	WP_015243333.1
1,251,843	1,251,843	1	A -> C		25	SNP (transversion)	100.0%	V -> G	245	GTC -> GGC	C770_RS47685	transcriptional regulato	Substitution	3.2E-78	82	2	WP_015243333.1
1,252,100	1,252,100	1	A -> G		86	SNP (transition)	97.7%							2.3E-274			
1,252,167	1,252,167	1	C -> T		79	SNP (transition)	100.0%							2.5E-269			
1,252,281	1,252,281	1	A -> G		100	SNP (transition)	100.0%		72	CTA -> CTG	C770_RS47690	3-ketoacyl-ACP reducta:	None	0.0	24	3	WP_010975354.1
1,252,539	1,252,539	1	A -> C		81	SNP (transversion)	100.0%		330	ATA -> ATC	C770_RS47690	3-ketoacyl-ACP reducta:	None	2.5E-292	110	3	WP_010975354.1
1,252,674	1,252,674	1	T -> G		90	SNP (transversion)	100.0%		465	GCT -> GCG	C770_RS47690	3-ketoacyl-ACP reducta:	None	1.0E-315	155	3	WP_010975354.1
1,252,827	1,252,827	1	G -> C		107	SNP (transversion)	98.1%		618	GCG -> GCC	C770_RS47690	3-ketoacyl-ACP reducta:	None	0.0	206	3	WP_010975354.1
1,252,867	1,252,867	1	G -> A		98	SNP (transition)	100.0%	A -> T	658	GCC -> ACC	C770_RS47690	3-ketoacyl-ACP reducta:	Substitution	0.0	220	1	WP_010975354.1
1,252,881	1,252,881	1	T -> C		94	SNP (transition)	100.0%		672	CTT -> CTC	C770_RS47690	3-ketoacyl-ACP reducta:	None	0.0	224	3	WP_010975354.1
1,252,896	1,252,896	1	T -> C		95	SNP (transition)	98.9%		687	GGT -> GGC	C770_RS47690	3-ketoacyl-ACP reducta:	None	2.4E-318	229	3	WP_010975354.1
1,252,929	1,252,929	1	T -> C		100	SNP (transition)	100.0%		720	AAT -> AAC	C770_RS47690	3-ketoacyl-ACP reducta:	None	0.0	240	3	WP_010975354.1
1,252,950	1,252,950	1	C -> T		100	SNP (transition)	100.0%							0.0			
1,252,952	1,252,952	1	T -> C		101	SNP (transition)	100.0%							0.0			
1,252,968	1,252,968	1	C -> G		102	SNP (transversion)	99.0%							0.0			
1,252,993	1,252,992	0	(G)2 -> (G)3		107	Insertion (tandem repeat)	99.1%							0.0			
1,253,008	1,253,008	1	A -> G		104	SNP (transition)	100.0%							0.0			
1,253,042	1,253,047	6	AAGTGA		98	Deletion	100.0%							1.6E-157			
1,253,051	1,253,051	1	C -> A		99	SNP (transversion)	100.0%							0.0			
1,253,135	1,253,135	1	T -> C		96	SNP (transition)	100.0%		78	GCT -> GCC	C770_RS47695	oxidoreductase	None	0.0	26	3	WP_010975353.1
1,253,276	1,253,276	1	T -> C		93	SNP (transition)	100.0%		219	GAT -> GAC	C770_RS47695	oxidoreductase	None	6.3E-317	73	3	WP_010975353.1
1,253,322	1,253,322	1	C -> T		90	SNP (transition)	100.0%		265	CTG -> TTG	C770_RS47695	oxidoreductase	None	1.0E-279	89	1	WP_010975353.1
1,253,444	1,253,444	1	G -> A		91	SNP (transition)	98.9%		387	CCG -> CCA	C770_RS47695	oxidoreductase	None	9.1E-305	129	3	WP_010975353.1
1,253,525	1,253,525	1	G -> C		88	SNP (transversion)	98.9%		468	GCG -> GCC	C770_RS47695	oxidoreductase	None	3.5E-277	156	3	WP_010975353.1
1,253,561	1,253,561	1	C -> T		90	SNP (transition)	98.9%		504	ATC -> ATT	C770_RS47695	oxidoreductase	None	1.8E-292	168	3	WP_010975353.1
1,253,648	1,253,648	1	C -> T		84	SNP (transition)	97.6%		591	CAC -> CAT	C770_RS47695	oxidoreductase	None	2.2E-251	197	3	WP_010975353.1
1,253,829	1,253,829	1	C -> G		66	SNP (transversion)	100.0%	P -> A	772	CCG -> GCG	C770_RS47695	oxidoreductase	Substitution	1.0E-231	258	1	WP_010975353.1
1,253,852	1,253,852	1	G -> A		63	SNP (transition)	100.0%		795	CCG -> CCA	C770_RS47695	oxidoreductase	None	3.2E-221	265	3	WP_010975353.1
1,253,856	1,253,856	1	G -> A		65	SNP (transition)	100.0%	E -> K	799	GAA -> AAA	C770_RS47695	oxidoreductase	Substitution	1.0E-208	267	1	WP_010975353.1
1,253,876	1,253,876	1	T -> A		62	SNP (transversion)	98.4%		819	GGT -> GGA	C770_RS47695	oxidoreductase	None	2.5E-206	273	3	WP_010975353.1
1,253,936	1,253,936	1	T -> A		61	SNP (transversion)	100.0%		879	CTT -> CTA	C770_RS47695	oxidoreductase	None	3.2E-214	293	3	WP_010975353.1
1,253,978	1,253,978	1	C -> T		59	SNP (transition)	100.0%							1.6E-189			
1,254,011	1,254,012	2	GC -> CG		59	Substitution	100.0%							2.0E-195			
1,254,028	1,254,028	1	G -> C		61	SNP (transversion)	100.0%							5.0E-202			
1,254,037	1,254,038	2	GG -> AC	60 -> 61		Substitution	100.0%							1.0E-204			
1,254,092	1,254,092	1	C -> G		60	SNP (transversion)	100.0%							1.0E-198			
1,254,122	1,254,122	1	G -> A		63	SNP (transition)	100.0%							2.5E-202			
1,254,128	1,254,128	1	G -> A		64	SNP (transition)	98.4%							4.0E-213			
1,254,177	1,254,177	1	A -> G		61	SNP (transition)	100.0%							4.0E-208			
1,254,183	1,254,183	1	C -> T		62	SNP (transition)	98.4%							2.5E-206			
1,254,196	1,254,196	1	A -> G		65	SNP (transition)	96.9%							2.6E-205			
1,254,228	1,254,228	1	T -> A		70	SNP (transversion)	97.1%							1.5E-228			
1,254,231	1,254,231	1	-C		72	Deletion	98.6%							1.8E-112			
1,254,249	1,254,249	1	G -> A		73	SNP (transition)	97.3%							8.3E-246			
1,254,263	1,254,263	1	T -> C		73	SNP (transition)	97.3%							8.3E-246			
1,254,312	1,254,312	1	G -> A		79	SNP (transition)	98.7%	V -> I	31	GTA -> ATA	C770_RS47700	cysteine hydrolase	Substitution	5.0E-264	11	1	WP_010975352.1
1,254,389	1,254,389	1	T -> C		96	SNP (transition)	96.9%		108	GTT -> GTC	C770_RS47700	cysteine hydrolase	None	9.0E-312	36	3	WP_010975352.1
1,254,554	1,254,554	1	G -> A		106	SNP (transition)	98.1%		273	AAG -> AAA	C770_RS47700	cysteine hydrolase	None	5.6E-309	91	3	WP_010975352.1
1,254,942	1,254,942	1	T -> C		80	SNP (transition)	100.0%							1.0E-272			
1,254,952	1,254,952	1	G -> C		80	SNP (transversion)	100.0%							1.0E-264			
1,255,064	1,255,064	1	T -> A		83	SNP (transversion)	100.0%							1.3E-274			
1,255,077	1,255,077	1	T -> C		85	SNP (transition)	100.0%							1.0E-289			
1,255,106	1,255,106	1	T -> C		80	SNP (transition)	100.0%							1.0E-256			
1,255,131	1,255,131	1	A -> C		76	SNP (transversion)	100.0%							4.0E-259			
1,255,475	1,255,475	1	A -> C		88	SNP (transversion)	100.0%	E -> A	302	GAA -> GCA	C770_RS47705	hypothetical protein	Substitution	4.0E-291	101	2	WP_015243335.1
1,255,524	1,255,524	1	G -> C		82	SNP (transversion)	100.0%		351	CTG -> CTC	C770_RS47705	hypothetical protein	None	4.0E-263	117	3	WP_015243335.1
1,255,549	1,255,549	1	C -> T		78	SNP (transition)	100.0%	H -> Y	376	CAC -> TAC	C770_RS47705	hypothetical protein	Substitution	4.0E-258	126	1	WP_015243335.1
1,255,575	1,255,575	1	T -> C		79	SNP (transition)	100.0%		402	ATT -> ATC	C770_RS47705	hypothetical protein	None	2.5E-269	134	3	WP_015243335.1
1,255,614	1,255,614	1	C -> G		74	SNP (transversion)	100.0%		441	GCC -> GCG	C770_RS47705	hypothetical protein	None	1.0E-259	147	3	WP_015243335.1
1,255,632	1,255,632	1	C -> T		72	SNP (transition)	100.0%		459	GGC -> GGT	C770_RS47705	hypothetical protein	None	4.0E-231	153	3	WP_015243335.1
1,255,878	1,255,878	1	T -> G		69	SNP (transversion)	98.6%		705	CCT -> CCG	C770_RS47705	hypothetical protein	None	1.1E-243	235	3	WP_015243335.1
1,255,915	1,255,915	1	G -> A		76	SNP (transition)	98.7%							2.4E-246			
1,255,955	1,255,955	1	A -> G		73	SNP (transition)	98.6%							7.3E-251			
1,255,972	1,255,972	1	C -> T		75	SNP (transition)	96.0%							1.1E-240			
1,255,980	1,255,979	0	ACCC		77	Insertion	98.7%							3.1E-257			
1,255,997	1,255,997	1	C -> G		81	SNP (transversion)	97.5%							6.5E-258			
1,256,011	1,256,010	0	(C)4 -> (C)5		83	Insertion (tandem repeat)	97.6%							2.1E-256			
1,256,239	1,256,239	1	G -> A		98	SNP (transition)	99.0%		480	GGC -> GGT	C770_RS47710	Ribulose-5-phosphate 4	None	7.8E-319	160	3	WP_015243336.1
1,256,283	1,256,283	1	G -> C		94	SNP (transversion)	98.9%	L -> V	436	CTC -> GTC	C770_RS47710	Ribulose-5-phosphate 4	Substitution	4.9E-324	146	1	WP_015243336.1
1,256,341	1,256,341	1	T -> C		94	SNP (transition)	100.0%		378	ACA -> ACG	C770_RS47710	Ribulose-5-phosphate 4	None	2.5E-320	126	3	WP_015243336.1
1,256,731	1,256,731	1	C -> T		77	SNP (transition)	98.7%	E -> K	1.453	GAA -> AAA	C770_RS47715	Sugar (pentulose and he	Substitution	4.9E-242	485	1	WP_015243337.1
1,256,836	1,256,836	1	A -> G		73	SNP (transition)	100.0%	S -> P	1.348	TCC -> CCC	C770_RS47715	Sugar (pentulose and he	Substitution	6.3E-249	450	1	WP_015243337.1
1,256,987	1,256,987	1	T -> C		81	SNP (transition)	98.8%		1.197	CTA -> CTG	C770_RS47715	Sugar (pentulose and he	None	8.1E-279	399	3	WP_015243337.1
1,257,105	1,257,105	1	T -> G		78	SNP (transversion)	100.0%	D -> A	1.079	GAT -> GCT	C770_RS47715	Sugar (pentulose and he	Substitution	6.3E-266	360	2	WP_015243337.1
1,257,197	1,257,197	1	T -> C		88	SNP (transition)	100.0%		987	CTA -> CTG	C770_RS47715	Sugar (pentulose and he	None	6.3E-300	329	3	WP_015243337.1
1,257,332	1,257,332	1	C -> A		79	SNP (transversion)	100.0%		852	CGG -> CGT	C770_RS47715	Sugar (pentulose and he	None	4.0E-285	284	3	WP_015243337.1
1,257,347	1,257,347	1	A -> G		80	SNP (transition)	100.0%		837	CGT -> CGC	C770_RS47715	Sugar (pentulose and he	None	1.0E-272	279	3	WP_015243337.1
1,257,356	1,257,356	1	T -> C		83	SNP (transition)	100.0%		828	CCA -> CCG	C770_RS47715	Sugar (pentulose and he	None	3.2E-291	276	3	WP_015243337.1
1,257,510	1,257,510	1	A -> C		71	SNP (transversion)	100.0%	L -> R	674	CTC -> CGC	C770_RS47715	Sugar (pentulose and he	Substitution	4.0E-242	225	2	WP_015243337.1
1,257,525	1,257,527	3	CAT -> GGA	68 -> 69		Substitution	100.0%	GW -> GP	657	GGA,TGG -> GGT	C770_RS47715	Sugar (pentulose and he	Substitution	6.3E-232	219	3	WP_015243337.1
1,257,545	1,257,545	1	C -> T		71	SNP (transition)	100.0%		639	AAG -> AAA	C770_RS47715	Sugar (pentulose and he	None	6.3E-228	213	3	WP_015243337.1
1,257,550	1,257,551	2	AG -> CA	70 -> 71		Substitution	100.0%	FS -> FA	633	TTC,TCG -> TTT,(	C770_RS47715	Sugar (pentulose and he	Substitution	1.0E-231	211	3	WP_015243337.1
1,257,554	1,257,554	1	T -> G		69	SNP (transversion)	100.0%		630	CCA -> CCC	C770_RS47715	Sugar (pentulose and he	None	2.0E-228	210	3	WP_015243337.1
1,257,563	1,257,563	1	C -> T		67	SNP (transition)	100.0%		621	CTG -> CTA	C770_RS47715	Sugar (pentulose and he	None	7.9E-222	207	3	WP_015243337.1
1,257,659	1,257,659	1	C -> G		69	SNP (transversion)	100.0%		525	GCG -> GCC	C770_RS47715	Sugar (pentulose and he	None	2.0E-228	175	3	WP_015243337.1
1,257,668	1,257,668	1	G -> A		70	SNP (transition)	98.6%		516	GGC -> GGT	C770_RS47715	Sugar (pentulose and he	None	1.4E-226	172	3	WP_015243337.1
1,257,677	1,257,677	1	C -> G		70	SNP (transversion)	100.0%		507	CGG -> CGC	C770_RS47715	Sugar (pentulose and he	None	1.0E-231	169	3	WP_015243337.1
1,257,683	1,257,683	1	G -> A		69	SNP (transition)	100.0%		501	GCC -> GCT	C770_RS47715	Sugar (pentulose and he	None	2.0E-228	167	3	WP_015243337.1
1,257,716	1,257,716	1	C -> G		71	SNP (transversion)	100.0%		468	GCG -> GCC	C770_RS47715	Sugar (pentulose and he	None	4.0E-242	156	3	WP_015243337.1
1,257,730	1,257,730	1	C -> T		67	SNP (transition)	98.5%	A -> T	454	GCT -> ACT	C770_RS47715	Sugar (pentulose and he	Substitution	6.7E-230	152	1	WP_015243337.1
1,257,755	1,257,755	1	A -> G		70	SNP (transition)	98.6%		429	TAT -> TAC	C770_RS47715	Sugar (pentulose and he	None	1.8E-233	143	3	WP_015243337.1
1,257,761	1,257,761	1	T -> C		72	SNP (transition)	100.0%		423	CAA -> CAG	C770_RS47715	Sugar (pentulose and he	None	2.5E-238	141	3	WP_015243337.1
1,257,854	1,257,854	1	A -> T		66	SNP (transversion)	100.0%		330	CCT -> CCA	C770_RS47715	Sugar (pentulose and he	None	4.0E-225	110	3	WP_015243337.1
1,257,902	1,257,904	2	GAC -> CAG	71 -> 72		Substitution	100.0%	V -> L	280	GTC -> CTG	C770_RS47715	Sugar (pentulose and he	Substitution	4.0E-242	94	1	WP_015243337.1
1,257,925	1,257,925	1	A -> G		77	SNP (transition)	100.0%		259	TTG -> CTG	C770_RS47715	Sugar (pentulose and he	None	1.6E-262	87	1	WP_015243337.1
1,257,929	1,257,929	1	G -> A		78	SNP (transition)	100.0%		255	GGC -> GGT	C770_RS47715	Sugar (pentulose and he	None	4.0E-258	85	3	WP_015243337.1
1,257,938	1,257,938	1	G -> A		74	SNP (transition)	98.6%		246	GGC -> GGT	C770_RS47715	Sugar (pentulose and he	None	7.4E-218	82	3	WP_015243337.1
1,257,947	1,257,947	1	C -> G		75	SNP (transversion)	98.7%		237	GCG -> GCC	C770_RS47715	Sugar (pentulose and he	None	4.7E-243	79	3	WP_015243337.1
1,257,950	1,257,950	1	C -> T		74	SNP (transition)	100.0%		234	GCG -> GCA	C770_RS47715	Sugar (pentulose and he	None	1.6E-237	78	3	WP_015243337.1
1,257,953	1,257,953	1	T -> G		75	SNP (transversion)	100.0%		231	GTA -> GTC	C770_RS47715	Sugar (pentulose and he	None	1.0E-255	77	3	WP_015243337.1

1,257,962	1,257,964	2	AGC -> GGT	74 -> 76	Substitution	98.6% -> 98.7%	A -> T	220	GCT -> ACC	C770_RS47715	Sugar (pentulose and he	Substitution	7.6E-239	74	1	WP_015243337.1
1,257,968	1,257,969	2	AC -> TT	75	Substitution	98.7%	G -> E	215	GGT -> GAA	C770_RS47715	Sugar (pentulose and he	Substitution	4.7E-243	72	2	WP_015243337.1
1,257,971	1,257,971	1	G -> A	75	SNP (transition)	98.7%		213	CAC -> CAT	C770_RS47715	Sugar (pentulose and he	None	4.7E-243	71	3	WP_015243337.1
1,257,987	1,257,987	1	A -> G	76	SNP (transition)	100.0%	V -> A	197	GTT -> GCT	C770_RS47715	Sugar (pentulose and he	Substitution	1.6E-251	66	2	WP_015243337.1
1,258,011	1,258,011	1	C -> T	73	SNP (transition)	97.3%	G -> E	173	GGA -> GAA	C770_RS47715	Sugar (pentulose and he	Substitution	1.7E-224	58	2	WP_015243337.1
1,258,013	1,258,013	1	G -> A	73	SNP (transition)	100.0%		171	GGC -> GGT	C770_RS47715	Sugar (pentulose and he	None	6.3E-249	57	3	WP_015243337.1
1,258,025	1,258,025	1	G -> T	74	SNP (transversion)	98.6%		159	CTC -> CTA	C770_RS47715	Sugar (pentulose and he	None	9.3E-240	53	3	WP_015243337.1
1,258,046	1,258,046	1	C -> A	72	SNP (transversion)	100.0%		138	CCG -> CCT	C770_RS47715	Sugar (pentulose and he	None	2.5E-238	46	3	WP_015243337.1
1,258,049	1,258,049	1	T -> C	72	SNP (transition)	100.0%		135	GGA -> GGG	C770_RS47715	Sugar (pentulose and he	None	1.6E-245	45	3	WP_015243337.1
1,258,062	1,258,062	1	C -> G	71	SNP (transversion)	98.6%	R -> P	122	CGC -> CCC	C770_RS47715	Sugar (pentulose and he	Substitution	7.1E-237	41	2	WP_015243337.1
1,258,067	1,258,067	1	T -> A	71	SNP (transversion)	100.0%		117	CCA -> CCT	C770_RS47715	Sugar (pentulose and he	None	4.0E-242	39	3	WP_015243337.1
1,258,082	1,258,082	1	T -> C	73	SNP (transition)	100.0%		102	GAA -> GAG	C770_RS47715	Sugar (pentulose and he	None	1.3E-241	34	3	WP_015243337.1
1,258,085	1,258,086	2	GA -> CG	72	Substitution	98.6%	V -> A	98	GTC -> GCG	C770_RS47715	Sugar (pentulose and he	Substitution	3.6E-233	33	2	WP_015243337.1
1,258,090	1,258,090	1	G -> C	72	SNP (transversion)	98.6%	L -> V	94	CTG -> GTG	C770_RS47715	Sugar (pentulose and he	Substitution	3.6E-233	32	1	WP_015243337.1
1,258,103	1,258,104	2	TG -> CC	75 -> 76	Substitution	100.0%	A -> G	80	GCA -> GGG	C770_RS47715	Sugar (pentulose and he	Substitution	1.0E-240	27	2	WP_015243337.1
1,258,106	1,258,106	1	A -> G	75	SNP (transition)	100.0%		78	GTT -> GTC	C770_RS47715	Sugar (pentulose and he	None	1.0E-255	26	3	WP_015243337.1
1,258,124	1,258,124	1	A -> G	81	SNP (transition)	100.0%		60	AAT -> AAC	C770_RS47715	Sugar (pentulose and he	None	3.2E-284	20	3	WP_015243337.1
1,258,136	1,258,136	1	A -> G	82	SNP (transition)	98.8%		48	GTT -> GTC	C770_RS47715	Sugar (pentulose and he	None	2.6E-282	16	3	WP_015243337.1
1,258,139	1,258,139	1	A -> G	80	SNP (transition)	100.0%		45	GAT -> GAC	C770_RS47715	Sugar (pentulose and he	None	1.0E-280	15	3	WP_015243337.1
1,258,145	1,258,145	1	G -> A	79	SNP (transition)	98.7%		39	GTC -> GTT	C770_RS47715	Sugar (pentulose and he	None	2.0E-248	13	3	WP_015243337.1
1,258,207	1,258,207	1	A -> G	76	SNP (transition)	100.0%							1.6E-251			
1,258,405	1,258,405	1	C -> G	90	SNP (transversion)	100.0%		672	TCG -> TCC	C770_RS47720	Sugar phosphate isomei	None	1.0E-306	224	3	WP_015243338.1
1,258,516	1,258,516	1	G -> A	97	SNP (transition)	100.0%		561	GGC -> GGT	C770_RS47720	Sugar phosphate isomei	None	0.0	187	3	WP_015243338.1
1,258,909	1,258,909	1	T -> C	83	SNP (transition)	98.8%		168	AAA -> AAG	C770_RS47720	Sugar phosphate isomei	None	1.3E-277	56	3	WP_015243338.1
1,258,922	1,258,922	1	C -> G	84	SNP (transversion)	100.0%	G -> A	155	GGC -> GCC	C770_RS47720	Sugar phosphate isomei	Substitution	4.0E-261	52	2	WP_015243338.1
1,259,007	1,259,007	1	C -> T	80	SNP (transition)	100.0%	V -> I	70	GTA -> ATA	C770_RS47720	Sugar phosphate isomei	Substitution	1.0E-272	24	1	WP_015243338.1
1,259,214	1,259,214	1	A -> G	84	SNP (transition)	100.0%		864	CTT -> CTC	C770_RS47725	sugar ABC transporter p	None	2.5E-286	288	3	WP_010975347.1
1,259,334	1,259,334	1	C -> G	104	SNP (transversion)	100.0%		744	GGG -> GGC	C770_RS47725	sugar ABC transporter p	None	0.0	248	3	WP_010975347.1
1,259,379	1,259,379	1	G -> T	116	SNP (transversion)	100.0%		699	ATC -> ATA	C770_RS47725	sugar ABC transporter p	None	0.0	233	3	WP_010975347.1
1,259,384	1,259,384	1	A -> C	116	SNP (transversion)	100.0%	S -> A	694	TCG -> GCG	C770_RS47725	sugar ABC transporter p	Substitution	0.0	232	1	WP_010975347.1
1,259,559	1,259,559	1	T -> G	120	SNP (transversion)	100.0%		519	CCA -> CCC	C770_RS47725	sugar ABC transporter p	None	0.0	173	3	WP_010975347.1
1,259,637	1,259,637	1	G -> A	98	SNP (transition)	100.0%		441	TTC -> TTT	C770_RS47725	sugar ABC transporter p	None	2.5E-314	147	3	WP_010975347.1
1,259,675	1,259,675	1	A -> G	86	SNP (transition)	100.0%		403	TTG -> CTG	C770_RS47725	sugar ABC transporter p	None	4.0E-293	135	1	WP_010975347.1
1,259,844	1,259,844	1	G -> A	74	SNP (transition)	98.6%		234	AAC -> AAT	C770_RS47725	sugar ABC transporter p	None	9.3E-240	78	3	WP_010975347.1
1,259,913	1,259,913	1	G -> C	80	SNP (transversion)	100.0%		165	CTC -> CTG	C770_RS47725	sugar ABC transporter p	None	1.0E-280	55	3	WP_010975347.1
1,259,916	1,259,916	1	T -> C	80	SNP (transition)	98.8%		162	GGA -> GGG	C770_RS47725	sugar ABC transporter p	None	2.0E-267	54	3	WP_010975347.1
1,260,191	1,260,191	1	C -> T	83	SNP (transition)	98.8%		885	GTG -> GTA	C770_RS47730	sugar ABC transporter p	None	1.3E-277	295	3	WP_014527450.1
1,260,293	1,260,293	1	G -> C	88	SNP (transversion)	100.0%		783	CTC -> CTG	C770_RS47730	sugar ABC transporter p	None	4.0E-291	261	3	WP_014527450.1
1,260,443	1,260,443	1	C -> G	99	SNP (transversion)	100.0%		633	GGG -> GGC	C770_RS47730	sugar ABC transporter p	None	0.0	211	3	WP_014527450.1
1,260,449	1,260,449	1	G -> A	100	SNP (transition)	99.0%		627	CGC -> CGT	C770_RS47730	sugar ABC transporter p	None	1.3E-305	209	3	WP_014527450.1
1,260,689	1,260,689	1	A -> G	81	SNP (transition)	100.0%		387	AAT -> AAC	C770_RS47730	sugar ABC transporter p	None	3.2E-284	129	3	WP_014527450.1
1,260,701	1,260,701	1	G -> A	82	SNP (transition)	100.0%		375	ATC -> ATT	C770_RS47730	sugar ABC transporter p	None	1.6E-279	125	3	WP_014527450.1
1,260,710	1,260,710	1	A -> G	76	SNP (transition)	100.0%		366	GTT -> GTC	C770_RS47730	sugar ABC transporter p	None	1.0E-266	122	3	WP_014527450.1
1,260,779	1,260,779	1	G -> A	78	SNP (transition)	100.0%		297	GGC -> GGT	C770_RS47730	sugar ABC transporter p	None	1.6E-242	99	3	WP_014527450.1
1,260,844	1,260,844	1	C -> A	72	SNP (transversion)	100.0%	G -> C	232	GGC -> TGC	C770_RS47730	sugar ABC transporter p	Substitution	6.3E-260	78	1	WP_014527450.1
1,260,863	1,260,863	1	G -> T	72	SNP (transversion)	97.2%		213	GCC -> GCA	C770_RS47730	sugar ABC transporter p	None	2.6E-228	71	3	WP_014527450.1
1,260,962	1,260,962	1	A -> G	74	SNP (transition)	100.0%		114	GCT -> GCC	C770_RS47730	sugar ABC transporter p	None	2.5E-252	38	3	WP_014527450.1
1,260,980	1,260,980	1	A -> G	75	SNP (transition)	100.0%		96	TTT -> TTC	C770_RS47730	sugar ABC transporter p	None	3.2E-263	32	3	WP_014527450.1
1,261,001	1,261,001	1	A -> G	77	SNP (transition)	100.0%		75	CTT -> CTC	C770_RS47730	sugar ABC transporter p	None	1.6E-262	25	3	WP_014527450.1
1,261,007	1,261,009	2	TGT -> GGC	81	Substitution	98.8%	T -> A	67	ACA -> GCC	C770_RS47730	sugar ABC transporter p	Substitution	8.1E-255	23	1	WP_014527450.1
1,261,025	1,261,025	1	T -> C	82	SNP (transition)	97.6%		51	GTA -> GTG	C770_RS47730	sugar ABC transporter p	None	3.3E-269	17	3	WP_014527450.1
1,261,031	1,261,031	1	C -> T	82	SNP (transition)	98.8%		45	CTG -> CTA	C770_RS47730	sugar ABC transporter p	None	2.6E-282	15	3	WP_014527450.1
1,261,080	1,261,080	1	T -> G	85	SNP (transversion)	97.6%		1.518	GCA -> GCC	C770_RS47735	ABC-type sugar transpo	None	9.0E-263	506	3	WP_015243339.1
1,261,494	1,261,494	1	G -> C	71	SNP (transversion)	98.6%		1.104	CTC -> CTG	C770_RS47735	ABC-type sugar transpo	None	7.1E-223	368	3	WP_015243339.1
1,261,590	1,261,590	1	G -> A	68	SNP (transition)	100.0%		1.008	GGC -> GGT	C770_RS47735	ABC-type sugar transpo	None	2.5E-218	336	3	WP_015243339.1
1,261,616	1,261,616	1	T -> G	72	SNP (transversion)	100.0%	I -> L	982	ATC -> CTC	C770_RS47735	ABC-type sugar transpo	Substitution	1.6E-245	328	1	WP_015243339.1
1,261,631	1,261,631	1	T -> A	73	SNP (transversion)	100.0%	T -> S	967	ACC -> TCC	C770_RS47735	ABC-type sugar transpo	Substitution	6.3E-249	323	1	WP_015243339.1
1,261,667	1,261,668	2	CT -> GC	78	Substitution	98.7%	EV -> EL	930	GAA,GTG -> GAG	C770_RS47735	ABC-type sugar transpo	Substitution	2.5E-268	310	3	WP_015243339.1
1,261,692	1,261,692	1	G -> A	79	SNP (transition)	100.0%		906	GGC -> GGT	C770_RS47735	ABC-type sugar transpo	None	3.2E-277	302	3	WP_015243339.1
1,261,737	1,261,737	1	A -> T	77	SNP (transversion)	100.0%	D -> E	861	GAT -> GAA	C770_RS47735	ABC-type sugar transpo	Substitution	1.6E-262	287	3	WP_015243339.1
1,261,743	1,261,743	1	A -> G	79	SNP (transition)	100.0%		855	TTT -> TTC	C770_RS47735	ABC-type sugar transpo	None	2.0E-261	285	3	WP_015243339.1
1,261,765	1,261,765	1	G -> C	79	SNP (transversion)	100.0%	A -> G	833	GCG -> GGG	C770_RS47735	ABC-type sugar transpo	Substitution	3.2E-277	278	2	WP_015243339.1
1,261,770	1,261,770	1	C -> A	81	SNP (transversion)	98.8%		828	GTG -> GTT	C770_RS47735	ABC-type sugar transpo	None	8.1E-263	276	3	WP_015243339.1
1,261,803	1,261,803	1	A -> G	87	SNP (transition)	97.7%		795	CGT -> CGC	C770_RS47735	ABC-type sugar transpo	None	1.2E-277	265	3	WP_015243339.1
1,261,848	1,261,848	1	A -> G	92	SNP (transition)	100.0%		750	ACT -> ACC	C770_RS47735	ABC-type sugar transpo	None	2.5E-304	250	3	WP_015243339.1
1,261,875	1,261,875	1	G -> A	96	SNP (transition)	99.0%		723	GCC -> GCT	C770_RS47735	ABC-type sugar transpo	None	3.0E-312	241	3	WP_015243339.1
1,261,878	1,261,878	1	C -> G	96	SNP (transversion)	100.0%		720	CCG -> CCC	C770_RS47735	ABC-type sugar transpo	None	6.3E-308	240	3	WP_015243339.1
1,261,884	1,261,884	1	A -> G	97	SNP (transition)	100.0%		714	GTT -> GTC	C770_RS47735	ABC-type sugar transpo	None	4.0E-311	238	3	WP_015243339.1
1,261,914	1,261,914	1	T -> C	101	SNP (transition)	100.0%		684	ACA -> ACG	C770_RS47735	ABC-type sugar transpo	None	0.0	228	3	WP_015243339.1
1,261,953	1,261,953	1	A -> G	103	SNP (transition)	98.1%		645	CAT -> CAC	C770_RS47735	ABC-type sugar transpo	None	0.0	215	3	WP_015243339.1
1,261,989	1,261,989	1	G -> C	104	SNP (transversion)	100.0%		609	CTC -> CTG	C770_RS47735	ABC-type sugar transpo	None	0.0	203	3	WP_015243339.1
1,262,043	1,262,043	1	G -> C	102	SNP (transversion)	100.0%		555	ACC -> ACG	C770_RS47735	ABC-type sugar transpo	None	0.0	185	3	WP_015243339.1
1,262,112	1,262,112	1	C -> T	100	SNP (transition)	98.0%		486	CAG -> CAA	C770_RS47735	ABC-type sugar transpo	None	2.0E-320	162	3	WP_015243339.1
1,262,136	1,262,136	1	C -> T	98	SNP (transition)	100.0%		462	CCG -> CCA	C770_RS47735	ABC-type sugar transpo	None	0.0	154	3	WP_015243339.1
1,262,139	1,262,139	1	T -> C	98	SNP (transition)	96.9%		459	CTA -> CTG	C770_RS47735	ABC-type sugar transpo	None	0.0	153	3	WP_015243339.1
1,262,151	1,262,153	2	CAA -> GAG	99 -> 100	Substitution	99.0%		445	TTG -> CTC	C770_RS47735	ABC-type sugar transpo	None	0.0	149	1	WP_015243339.1
1,262,181	1,262,181	1	C -> G	98	SNP (transversion)	100.0%		417	GTG -> GTC	C770_RS47735	ABC-type sugar transpo	None	4.9E-324	139	3	WP_015243339.1
1,262,193	1,262,193	1	A -> G	93	SNP (transition)	100.0%		405	CGT -> CGC	C770_RS47735	ABC-type sugar transpo	None	6.3E-317	135	3	WP_015243339.1
1,262,199	1,262,199	1	A -> G	95	SNP (transition)	100.0%		399	CGT -> CGC	C770_RS47735	ABC-type sugar transpo	None	1.0E-304	133	3	WP_015243339.1
1,262,266	1,262,266	1	G -> C	91	SNP (transversion)	98.9%	T -> S	332	ACC -> AGC	C770_RS47735	ABC-type sugar transpo	Substitution	9.1E-314	111	2	WP_015243339.1
1,262,286	1,262,286	1	A -> G	88	SNP (transition)	100.0%		312	CTT -> CTC	C770_RS47735	ABC-type sugar transpo	None	6.3E-300	104	3	WP_015243339.1
1,262,436	1,262,436	1	G -> A	82	SNP (transition)	100.0%		162	AAC -> AAT	C770_RS47735	ABC-type sugar transpo	None	1.6E-279	54	3	WP_015243339.1
1,262,469	1,262,470	2	CT -> GC	87	Substitution	98.9%	Q -> R	128	CAG -> CGC	C770_RS47735	ABC-type sugar transpo	Substitution	1.4E-282	43	2	WP_015243339.1
1,262,502	1,262,502	1	A -> G	89	SNP (transition)	100.0%		96	GTT -> GTC	C770_RS47735	ABC-type sugar transpo	None	1.6E-285	32	3	WP_015243339.1
1,262,686	1,262,686	1	C -> T	81	SNP (transition)	100.0%							6.3E-260			
1,262,727	1,262,727	1	A -> G	77	SNP (transition)	100.0%		969	GAT -> GAC	C770_RS47740	LacI family transcription	None	1.6E-262	323	3	WP_033045584.1
1,262,775	1,262,775	1	G -> A	79	SNP (transition)	98.7%		921	AAC -> AAT	C770_RS47740	LacI family transcription	None	5.0E-264	307	3	WP_033045584.1
1,262,778	1,262,778	1	A -> T	79	SNP (transversion)	100.0%		918	CGT -> CGA	C770_RS47740	LacI family transcription	None	1.6E-253	306	3	WP_033045584.1
1,262,946	1,262,946	1	G -> A	88	SNP (transition)	100.0%		750	GGC -> GGT	C770_RS47740	LacI family transcription	None	2.5E-282	250	3	WP_033045584.1
1,262,982	1,262,982	1	T -> C	81	SNP (transition)	98.8%		714	GAA -> GAG	C770_RS47740	LacI family transcription	None	8.1E-263	238	3	WP_033045584.1
1,263,183	1,263,183	1	T -> A	82	SNP (transversion)	100.0%		513	GCA -> GCT	C770_RS47740	LacI family transcription	None	1.0E-287	171	3	WP_033045584.1
1,263,300	1,263,300	1	G -> C	90	SNP (transversion)	100.0%		396	ACC -> ACG	C770_RS47740	LacI family transcription	None	1.0E-297	132	3	WP_033045584.1
1,263,435	1,263,435	1	G -> A	92	SNP (transition)	98.9%		261	ATC -> ATT	C770_RS47740	LacI family transcription	None	4.6E-299	87	3	WP_033045584.1
1,263,582	1,263,582	1	T -> A	93	SNP (transversion)	98.9%		114	ATA -> ATT	C770_RS47740	LacI family transcription	None	3.7E-293	38	3	WP_033045584.1
1,263,591	1,263,591	1	A -> G	88	SNP (transition)	100.0%		105	ATT -> ATC	C770_RS47740	LacI family transcription	None	4.0E-291	35	3	WP_033045584.1
1,263,600	1,263,600	1	C -> G	90	SNP (transversion)	98.9%		96	GTG -> GTC	C770_RS47740	LacI family transcription	None	1.8E-292	32	3	WP_033045584.1
1,263,722	1,263,722	1	A -> G	78	SNP (transition)	100.0%							6.3E-266			
1,263,824	1,263,824	1	A -> T	73	SNP (transversion)	95.9%							6.2E-241			
1,263,827	1,263,827	1	A -> G	70	SNP (transition)	100.0%							1.0E-238			
1,263,829	1,263,829	1	C -> T	70	SNP (transition)	100.0%							1.0E-231			
1,263,919	1,263,918	0	G	77	Insertion	100.0%							1.6E-262			
1,263,974	1,263,974	1	G -> A	83	SNP (transition)	100.0%	A -> T	4	GCG -> ACG	C770_RS47745	transcriptional regulatoı	Substitution	1.3E-274	2	1	WP_015243340.1
1,264,039	1,264,039	1	T -> C	81	SNP (transition)	100.0%		69	TCT -> TCC	C770_RS47745	transcriptional regulatoı	None	4.0E-276	23	3	WP_015243340.1
1,264,195	1,264,195	1	C -> G	74	SNP (transversion)	100.0%		225	CTC -> CTG	C770_RS47745	transcriptional regulatoı	None	1.0E-259	75	3	WP_015243340.1
1,264,304	1,264,304	1	A -> G	86	SNP (transition)	100.0%	K -> E	334	AAG -> GAG	C770_RS47745	transcriptional regulatoı	Substitution	1.0E-301	112	1	WP_015243340.1
1,264,453	1,264,453	1	A -> G	98	SNP (transition)	100.0%		483	GTA -> GTG	C770_RS47745	transcriptional regulatoı	None	4.9E-324	161	3	WP_015243340.1
1,264,567	1,264,567	1	G -> C	102	SNP (transversion)	100.0%		597	GGG -> GGC	C770_RS47745	transcriptional regulatoı	None	0.0	199	3	WP_015243340.1
1,264,597	1,264,597	1	C -> A	109	SNP (transversion)	100.0%		627	ATC -> ATA	C770_RS47745	transcriptional regulatoı	None	0.0	209	3	WP_015243340.1
1,264,603	1,264,603	1	A -> C	110	SNP (transversion)	99.1%		633	GGA -> GGC	C770_RS47745	transcriptional regulatoı	None	0.0	211	3	WP_015243340.1
1,264,606	1,264,606	1	T -> C	111	SNP (transition)	100.0%		636	TTT -> TTC	C770_RS47745	transcriptional regulatoı	None	0.0	212	3	WP_015243340.1
1,264,609	1,264,609	1	G -> C	114	SNP (transversion)	100.0%		639	CGG -> CGC	C770_RS47745	transcriptional regulatoı	None	0.0	213	3	WP_015243340.1
1,264,612	1,264,612	1	G -> C	115	SNP (transversion)	100.0%		642	GCG -> GCC	C770_RS47745	transcriptional regulatoı	None	0.0	214	3	WP_015243340.1
1,264,618	1,264,619	2	GC -> AG	114 -> 116	Substitution	100.0%	KQ -> KE	648	AAG,CAG -> AAA	C770_RS47745	transcriptional regulatoı	Substitution	0.0	216	3	WP_015243340.1
1,264,625	1,264,625	1	C -> T	118	SNP (transition)	99.2%		655	CTG -> TTG	C770_RS47745	transcriptional regulatoı	None	0.0	219	1	WP_015243340.1
1,264,634	1,264,635	2	AG -> GC	120	Substitution	98.3%	S -> A	664	AGC -> GCC	C770_RS47745	transcriptional regulatoı	Substitution	0.0	222	1	WP_015243340.1
1,264,645	1,264,645	1	C -> T	125	SNP (transition)	97.6%		675	GAC -> GAT	C770_RS47745	transcriptional regulatoı	None	0.0	225	3	WP_015243340.1
1,264,648	1,264,648	1	G -> A	126	SNP (transition)	99.2%		678	GAG -> GAA	C770_RS47745	transcriptional regulatoı	None	0.0	226	3	WP_015243340.1
1,264,681	1,264,681	1	C -> T	123	SNP (transition)	99.2%		711	AAC -> AAT	C770_RS47745	transcriptional regulatoı	None	0.0	237	3	WP_015243340.1
1,264,689	1,264,689	1	A -> T	121	SNP (transversion)	100.0%	K -> M	719	AAG -> ATG	C770_RS47745	transcriptional regulatoı	Substitution	0.0	240	2	WP_015243340.1
1,264,702	1,264,702	1	C -> T	119	SNP (transition)	99.2%		732	GCC -> GCT	C770_RS47745	transcriptional regulatoı	None	0.0	244	3	WP_015243340.1
1,264,729	1,264,729	1	G -> A	112	SNP (transition)	99.1%		759	CCG -> CCA	C770_RS47745	transcriptional regulatoı	None	0.0	253	3	WP_015243340.1
1,264,756	1,264,756	1	C -> A	115	SNP (transversion)	98.3%		786	ATC -> ATA	C770_RS47745	transcriptional regulatoı	None	0.0	262	3	WP_015243340.1
1,264,825	1,264,825	1	C -> A	106	SNP (transversion)	100.0%		855	GGC -> GGA	C770_RS47745	transcriptional regulatoı	None	0.0	285	3	WP_015243340.1
1,264,834	1,264,834	1	T -> C	109	SNP (transition)	100.0%		864	GAT -> GAC	C770_RS47745	transcriptional regulatoı	None	0.0	288	3	WP_015243340.1
1,265,088	1,265,088	1	G -> A	92	SNP (transition)	96.7%		1.749	TTC -> TTT	C770_RS47750	GNAT family acetyltrans	None	1.6E-271	583	3	WP_015243341.1
1,265,202	1,265,202	1	C -> T	75	SNP (transition)	98.7%		1.635	GTG -> GTA	C770_RS47750	GNAT family acetyltrans	None	1.9E-250	545	3	WP_015243341.1
1,265,352	1,265,352	1	A -> G	69	SNP (transition)	100.0%		1.485	CTT -> CTC	C770_RS47750	GNAT family acetyltrans	None	2.5E-235	495	3	WP_015243341.1
1,265,387	1,265,387	1	T -> C	69	SNP (transition)	100.0%	T -> A	1.45	ACG -> GCG	C770_RS47750	GNAT family acetyltrans	Substitution	2.0E-228	484	1	WP_015243341.1
1,265,487	1,265,487	1	A -> G	70	SNP (transition)	100.0%		1.35	ATT -> ATC	C770_RS47750	GNAT family acetyltrans	None	1.0E-238	450	3	WP_015243341.1
1,265,527	1,265,527	1	G -> A	70	SNP (transition)	98.6%	T -> M	1.31	ACG -> ATG	C770_RS47750	GNAT family acetyltrans	Substitution	1.8E-233	437	2	WP_015243341.1
1,265,640	1,265,640	1	C -> T	75	SNP (transition)	98.7%		1.197	ACG -> ACA	C770_RS47750	GNAT family acetyltrans	None	3.0E-228	399	3	WP_015243341.1
1,265,661	1,265,661	1	G -> C	71	SNP (transversion)	98.6%		1.176	CGC -> CGG	C770_RS47750	GNAT family acetyltrans	None	7.1E-223	392	3	WP_015243341.1
1,265,706	1,265,706	1	-C	78	Deletion	98.7%		1.131		C770_RS47750	GNAT family acetyltrans	Frame Shift	2.4E-114	377	3	WP_015243341.1
1,265,712	1,265,712	1	T -> CG	77	Insertion	98.7%		1.125		C770_RS47750	GNAT family acetyltrans	Frame Shift	1.2E-249	375	3	WP_015243341.1
1,265,715	1,265,715	1	G -> A	76	SNP (transition)	97.4%		1.122	TTC -> TTT	C770_RS47750	GNAT family acetyltrans	None	1.1E-226	374	3	WP_015243341.1
1,265,719	1,265,719	1	T -> G	77	SNP (transversion)	96.1%	D -> A	1.118	GAC -> GCC	C770_RS47750	GNAT family acetyltrans	Substitution	1.2E-232	373	2	WP_015243341.1
1,265,721	1,265,722	2	CT -> GC	77 -> 78	Substitution	97.4%	Q -> R	1.115	CAG -> CGC	C770_RS47750	GNAT family acetyltrans	Substitution	9.2E-245	372	2	WP_015243341.1
1,265,726	1,265,726	1	G -> C	75	SNP (transversion)	97.3%	L -> V	1.111	CTT -> GTT	C770_RS47750	GNAT family acetyltrans	Substitution	3.5E-238	371	1	WP_015243341.1
1,265,732	1,265,735	3	TGCC -> CCCG	75	Substitution	97.3%	GK -> RE	1.102	GGC,AAG -> CGC	C770_RS47750	GNAT family acetyltrans	Substitution	3.7E-225	368	1	WP_015243341.1
1,265,738	1,265,739	2	TT -> CC	73 -> 74	Substitution	97.3%	EN -> ED	1.098	GAA,AAC -> GAG	C770_RS47750	GNAT family acetyltrans	Substitution	1.7E-224	366	3	WP_015243341.1
1,265,742	1,265,743	2	AT -> CC	74	Substitution	98.6%	D -> G	1.094	GAT -> GGG	C770_RS47750	GNAT family acetyltrans	Substitution	3.7E-225	365	2	WP_015243341.1
1,265,751	1,265,753	2	GAT -> CAC	72 -> 73	Substitution	98.6%	I -> V	1.084	ATC -> GTG	C770_RS47750	GNAT family acetyltrans	Substitution	4.5E-226	362	1	WP_015243341.1
1,265,769	1,265,769	1	C -> A	74	SNP (transversion)	98.6%		1.068	CTG -> CTT	C770_RS47750	GNAT family acetyltrans	None	7.4E-218	356	3	WP_015243341.1
1,265,772	1,265,772	1	C -> G	74	SNP (transversion)	98.6%		1.065	GGG -> GGC	C770_RS47750	GNAT family acetyltrans	None	1.9E-232	355	3	WP_015243341.1
1,265,775	1,265,775	1	T -> C	74	SNP (transition)	95.9%		1.062	GCA -> GCG	C770_RS47750	GNAT family acetyltrans	None	5.1E-216	354	3	WP_015243341.1
1,265,898	1,265,898	1	A -> G	70	SNP (transition)	98.6%		939	TTT -> TTC	C770_RS47750	GNAT family acetyltrans	None	1.8E-233	313	3	WP_015243341.1
1,265,910	1,265,910	1	C -> T	74	SNP (transition)	100.0%		927	GCG -> GCA	C770_RS47750	GNAT family acetyltrans	None	2.5E-252	309	3	WP_015243341.1
1,265,913	1,265,913	1	A -> G	70	SNP (transition)	100.0%		924	GAT -> GAC	C770_RS47750	GNAT family acetyltrans	None	1.0E-224	308	3	WP_015243341.1
1,265,922	1,265,922	1	T -> C	69	SNP (transition)	100.0%		915	GAA -> GAG	C770_RS47750	GNAT family acetyltrans	None	2.5E-235	305	3	WP_015243341.1
1,265,952	1,265,952	1	A -> G	70	SNP (transition)	100.0%		885	GAT -> GAC	C770_RS47750	GNAT family acetyltrans	None	1.0E-245	295	3	WP_015243341.1
1,265,955	1,265,955	1	C -> G	71	SNP (transversion)	100.0%		882	GTG -> GTC	C770_RS47750	GNAT family acetyltrans	None	3.2E-249	294	3	WP_015243341.1
1,266,060	1,266,060	1	G -> T	89	SNP (transversion)	100.0%		777	CGC -> CGA	C770_RS47750	GNAT family acetyltrans	None	2.0E-294	259	3	WP_015243341.1
1,266,114	1,266,114	1	C -> G	92	SNP (transversion)	100.0%		723	GTG -> GTC	C770_RS47750	GNAT family acetyltrans	None	1.6E-313	241	3	WP_015243341.1
1,266,156	1,266,156	1	C -> T	91	SNP (transition)	100.0%		681	GAG -> GAA	C770_RS47750	GNAT family acetyltrans	None	6.3E-292	227	3	WP_015243341.1
1,266,297	1,266,297	1	C -> A	87	SNP (transversion)	98.9%	E -> D	540	GAG -> GAT	C770_RS47750	GNAT family acetyltrans	Substitution	3.5E-291	180	3	WP_015243341.1
1,266,306	1,266,306	1	G -> A	85	SNP (transition)	100.0%		531	GCC -> GCT	C770_RS47750	GNAT family acetyltrans	None	1.0E-289	177	3	WP_015243341.1
1,266,312	1,266,312	1	G -> A	85	SNP (transition)	97.6%		525	GAC -> GAT	C770_RS47750	GNAT family acetyltrans	None	2.3E-279	175	3	WP_015243341.1
1,266,339	1,266,339	1	C -> A	91	SNP (transversion)	100.0%		498	CGG -> CGT	C770_RS47750	GNAT family acetyltrans	None	6.3E-292	166	3	WP_015243341.1
1,266,413	1,266,413	1	C -> T	82	SNP (transition)	100.0%	V -> I	424	GTC -> ATC	C770_RS47750	GNAT family acetyltrans	Substitution	1.6E-279	142	1	WP_015243341.1
1,266,426	1,266,426	1	A -> G	79	SNP (transition)	98.7%		411	GCT -> GCC	C770_RS47750	GNAT family acetyltrans	None	3.1E-256	137	3	WP_015243341.1

1,266,435	1,266,435	1	A -> C		77	SNP (transversion)	98.7%		402	GCT -> GCG	C770_RS47750	GNAT family acetyltrans	None	7.7E-265	134	3	WP_015243341.1
1,266,516	1,266,516	1	G -> C		70	SNP (transversion)	100.0%		321	CCC -> CCG	C770_RS47750	GNAT family acetyltrans	None	1.0E-238	107	3	WP_015243341.1
1,266,537	1,266,537	1	T -> A		75	SNP (transversion)	100.0%		300	CCA -> CCT	C770_RS47750	GNAT family acetyltrans	None	3.2E-248	100	3	WP_015243341.1
1,266,573	1,266,573	1	G -> A		73	SNP (transition)	100.0%		264	TAC -> TAT	C770_RS47750	GNAT family acetyltrans	None	6.3E-249	88	3	WP_015243341.1
1,266,582	1,266,582	1	A -> G		73	SNP (transition)	100.0%		255	ATT -> ATC	C770_RS47750	GNAT family acetyltrans	None	3.2E-256	85	3	WP_015243341.1
1,266,603	1,266,603	1	T -> C		70	SNP (transition)	100.0%		234	GAA -> GAG	C770_RS47750	GNAT family acetyltrans	None	1.0E-231	78	3	WP_015243341.1
1,266,623	1,266,623	1	A -> G		69	SNP (transition)	100.0%		214	TTG -> CTG	C770_RS47750	GNAT family acetyltrans	None	2.0E-228	72	1	WP_015243341.1
1,266,648	1,266,648	1	C -> T		65	SNP (transition)	100.0%		189	CAG -> CAA	C770_RS47750	GNAT family acetyltrans	None	3.2E-215	63	3	WP_015243341.1
1,266,651	1,266,651	1	C -> G		65	SNP (transversion)	98.5%		186	GCG -> GCC	C770_RS47750	GNAT family acetyltrans	None	6.5E-223	62	3	WP_015243341.1
1,266,690	1,266,690	1	G -> A		73	SNP (transition)	100.0%		147	GAC -> GAT	C770_RS47750	GNAT family acetyltrans	None	6.3E-249	49	3	WP_015243341.1
1,266,729	1,266,729	1	A -> G		83	SNP (transition)	100.0%		108	GAT -> GAC	C770_RS47750	GNAT family acetyltrans	None	1.3E-274	36	3	WP_015243341.1
1,266,738	1,266,738	1	C -> G		85	SNP (transversion)	100.0%		99	CCG -> CCC	C770_RS47750	GNAT family acetyltrans	None	1.0E-272	33	3	WP_015243341.1
1,266,758	1,266,758	1	A -> G		83	SNP (transition)	100.0%		79	TTG -> CTG	C770_RS47750	GNAT family acetyltrans	None	6.3E-283	27	1	WP_015243341.1
1,266,767	1,266,768	2	GC -> AA		81	Substitution	98.8%		69	CGG,CTG -> CGT	C770_RS47750	GNAT family acetyltrans	None	5.0E-268	23	3	WP_015243341.1
1,266,780	1,266,780	1	C -> T		84	SNP (transition)	100.0%		57	GCG -> GCA	C770_RS47750	GNAT family acetyltrans	None	1.0E-294	19	3	WP_015243341.1
1,266,827	1,266,827	1	A -> T		74	SNP (transversion)	100.0%	S -> T	10	TCG -> ACG	C770_RS47750	GNAT family acetyltrans	Substitution	2.5E-252	4	1	WP_015243341.1
1,266,831	1,266,831	1	C -> T		78	SNP (transition)	100.0%		6	AAG -> AAA	C770_RS47750	GNAT family acetyltrans	None	6.3E-266	2	3	WP_015243341.1
1,266,838	1,266,838	1	C -> T		82	SNP (transition)	98.8%		1.776	TAG -> TAA	C770_RS47755	asparagine synthase (gl	None	3.3E-274	592	3	WP_015243342.1
1,266,841	1,266,841	1	T -> C		80	SNP (transition)	100.0%		1.773	GTA -> GTG	C770_RS47755	asparagine synthase (gl	None	1.0E-280	591	3	WP_015243342.1
1,266,901	1,266,901	1	C -> T		87	SNP (transition)	98.9%		1.713	CTG -> CTA	C770_RS47755	asparagine synthase (gl	None	3.5E-291	571	3	WP_015243342.1
1,266,926	1,266,930	4	TCCTG -> CGTTC	85 -> 88		Substitution	97.6% -> 97.7%	QE -> ER	1.684	CAG,GAG -> GAA	C770_RS47755	asparagine synthase (gl	Substitution	5.4E-276	562	1	WP_015243342.1
1,266,943	1,266,943	1	A -> G		90	SNP (transition)	100.0%		1.671	CTT -> CTC	C770_RS47755	asparagine synthase (gl	None	1.0E-315	557	3	WP_015243342.1
1,266,950	1,266,950	1	G -> A		91	SNP (transition)	100.0%	A -> V	1.664	GCT -> GTT	C770_RS47755	asparagine synthase (gl	Substitution	4.0E-310	555	2	WP_015243342.1
1,266,952	1,266,952	1	A -> G		91	SNP (transition)	98.9%		1.662	GAT -> GAC	C770_RS47755	asparagine synthase (gl	None	9.1E-314	554	3	WP_015243342.1
1,266,957	1,266,957	1	G -> T		91	SNP (transversion)	100.0%	Q -> K	1.657	CAA -> AAA	C770_RS47755	asparagine synthase (gl	Substitution	6.3E-292	553	1	WP_015243342.1
1,266,963	1,266,963	1	G -> T		88	SNP (transversion)	100.0%	L -> I	1.651	CTC -> ATC	C770_RS47755	asparagine synthase (gl	Substitution	1.0E-308	551	1	WP_015243342.1
1,266,970	1,266,970	1	C -> G		88	SNP (transversion)	100.0%	E -> D	1.644	GAG -> GAC	C770_RS47755	asparagine synthase (gl	Substitution	4.0E-291	548	3	WP_015243342.1
1,266,985	1,266,985	1	C -> A		86	SNP (transversion)	100.0%		1.629	TCG -> TCT	C770_RS47755	asparagine synthase (gl	None	4.0E-293	543	3	WP_015243342.1
1,266,997	1,266,997	1	A -> G		83	SNP (transition)	98.8%		1.617	GAT -> GAC	C770_RS47755	asparagine synthase (gl	None	8.3E-286	539	3	WP_015243342.1
1,267,009	1,267,009	1	A -> G		86	SNP (transition)	98.8%		1.605	GAT -> GAC	C770_RS47755	asparagine synthase (gl	None	8.6E-288	535	3	WP_015243342.1
1,267,060	1,267,060	1	T -> C		90	SNP (transition)	100.0%		1.554	AAA -> AAG	C770_RS47755	asparagine synthase (gl	None	1.0E-297	518	3	WP_015243342.1
1,267,066	1,267,066	1	G -> A		91	SNP (transition)	98.9%		1.548	CGC -> CGT	C770_RS47755	asparagine synthase (gl	None	9.1E-305	516	3	WP_015243342.1
1,267,069	1,267,069	1	G -> A		91	SNP (transition)	98.9%		1.545	GAC -> GAT	C770_RS47755	asparagine synthase (gl	None	9.1E-296	515	3	WP_015243342.1
1,267,141	1,267,141	1	C -> T		92	SNP (transition)	100.0%		1.473	GAG -> GAA	C770_RS47755	asparagine synthase (gl	None	2.5E-304	491	3	WP_015243342.1
1,267,176	1,267,176	1	G -> A		88	SNP (transition)	100.0%		1.438	CTG -> TTG	C770_RS47755	asparagine synthase (gl	None	1.6E-273	480	1	WP_015243342.1
1,267,198	1,267,198	1	G -> A		89	SNP (transition)	100.0%		1.416	CGC -> CGT	C770_RS47755	asparagine synthase (gl	None	2.0E-294	472	3	WP_015243342.1
1,267,207	1,267,207	1	G -> C		91	SNP (transversion)	100.0%		1.407	CTC -> CTG	C770_RS47755	asparagine synthase (gl	None	5.0E-301	469	3	WP_015243342.1
1,267,249	1,267,249	1	G -> A		87	SNP (transition)	100.0%		1.365	GAC -> GAT	C770_RS47755	asparagine synthase (gl	None	1.6E-296	455	3	WP_015243342.1
1,267,264	1,267,264	1	A -> G		89	SNP (transition)	98.9%		1.35	GTT -> GTC	C770_RS47755	asparagine synthase (gl	None	5.6E-298	450	3	WP_015243342.1
1,267,297	1,267,297	1	G -> A		84	SNP (transition)	100.0%		1.317	CCC -> CCT	C770_RS47755	asparagine synthase (gl	None	2.5E-286	439	3	WP_015243342.1
1,267,309	1,267,309	1	A -> G		86	SNP (transition)	97.7%		1.305	GGT -> GGC	C770_RS47755	asparagine synthase (gl	None	9.2E-283	435	3	WP_015243342.1
1,267,314	1,267,314	1	C -> T		85	SNP (transition)	100.0%	E -> K	1.3	GAG -> AAG	C770_RS47755	asparagine synthase (gl	Substitution	3.2E-281	434	1	WP_015243342.1
1,267,324	1,267,324	1	G -> A		81	SNP (transition)	100.0%		1.29	CAC -> CAT	C770_RS47755	asparagine synthase (gl	None	4.0E-276	430	3	WP_015243342.1
1,267,339	1,267,339	1	G -> A		80	SNP (transition)	100.0%		1.275	GAC -> GAT	C770_RS47755	asparagine synthase (gl	None	1.0E-272	425	3	WP_015243342.1
1,267,345	1,267,345	1	A -> G		80	SNP (transition)	100.0%		1.269	AGT -> AGC	C770_RS47755	asparagine synthase (gl	None	1.0E-264	423	3	WP_015243342.1
1,267,348	1,267,348	1	C -> T		80	SNP (transition)	100.0%		1.266	GTG -> GTA	C770_RS47755	asparagine synthase (gl	None	1.0E-256	422	3	WP_015243342.1
1,267,356	1,267,356	1	G -> C		81	SNP (transversion)	100.0%	H -> D	1.258	CAC -> GAC	C770_RS47755	asparagine synthase (gl	Substitution	4.0E-276	420	1	WP_015243342.1
1,267,389	1,267,389	1	T -> A		71	SNP (transversion)	100.0%	T -> S	1.225	ACC -> TCC	C770_RS47755	asparagine synthase (gl	Substitution	3.2E-249	409	1	WP_015243342.1
1,267,392	1,267,392	1	G -> A		71	SNP (transition)	100.0%		1.222	CTG -> TTG	C770_RS47755	asparagine synthase (gl	None	4.0E-242	408	1	WP_015243342.1
1,267,465	1,267,465	1	G -> A		71	SNP (transition)	98.6%		1.149	TAC -> TAT	C770_RS47755	asparagine synthase (gl	None	7.1E-230	383	3	WP_015243342.1
1,267,480	1,267,480	1	T -> G		66	SNP (transversion)	100.0%		1.134	GGA -> GGC	C770_RS47755	asparagine synthase (gl	None	1.0E-231	378	3	WP_015243342.1
1,267,529	1,267,529	1	T -> C		67	SNP (transition)	100.0%	K -> R	1.085	AAG -> AGG	C770_RS47755	asparagine synthase (gl	Substitution	4.0E-215	362	2	WP_015243342.1
1,268,112	1,268,112	1	C -> A		88	SNP (transversion)	100.0%	A -> S	502	GCG -> TCG	C770_RS47755	asparagine synthase (gl	Substitution	6.3E-300	168	1	WP_015243342.1
1,268,146	1,268,146	1	T -> C		82	SNP (transition)	100.0%		468	TTA -> TTG	C770_RS47755	asparagine synthase (gl	None	1.0E-287	156	3	WP_015243342.1
1,268,301	1,268,301	1	G -> C		87	SNP (transversion)	100.0%	L -> V	313	CTG -> GTG	C770_RS47755	asparagine synthase (gl	Substitution	3.2E-305	105	1	WP_015243342.1
1,268,304	1,268,304	1	T -> G		83	SNP (transversion)	98.8%	I -> L	310	ATC -> CTC	C770_RS47755	asparagine synthase (gl	Substitution	2.1E-269	104	1	WP_015243342.1
1,268,357	1,268,357	1	A -> G		85	SNP (transition)	98.8%	L -> S	257	TTG -> TCG	C770_RS47755	asparagine synthase (gl	Substitution	2.1E-284	86	2	WP_015243342.1
1,268,566	1,268,566	1	C -> T		78	SNP (transition)	100.0%		48	TCG -> TCA	C770_RS47755	asparagine synthase (gl	None	6.3E-266	16	3	WP_015243342.1
1,268,656	1,268,656	1	C -> T		70	SNP (transition)	100.0%							1.0E-231			
1,268,687	1,268,687	1	G -> T		68	SNP (transversion)	100.0%							4.0E-225			
1,269,094	1,269,094	1	T -> C		84	SNP (transition)	100.0%		420	GAA -> GAG	C770_RS47760	transcriptional regulato	None	6.3E-278	140	3	WP_015243343.1
1,269,318	1,269,318	1	C -> T		87	SNP (transition)	98.9%	V -> I	196	GTA -> ATA	C770_RS47760	transcriptional regulato	Substitution	3.5E-291	66	1	WP_015243343.1
1,269,469	1,269,469	1	A -> C		82	SNP (transversion)	100.0%		45	CGT -> CGG	C770_RS47760	transcriptional regulato	None	1.0E-287	15	3	WP_015243343.1
1,269,474	1,269,473	0	GGCCAT	82 -> 83		Insertion	100.0%	E -> DGQ	41	GAG -> GAT,GGC	C770_RS47760	transcriptional regulato	Insertion	1.6E-279	14	2	WP_015243343.1
1,269,490	1,269,490	1	T -> C		83	SNP (transition)	100.0%		24	AAA -> AAG	C770_RS47760	transcriptional regulato	None	1.6E-299	8	3	WP_015243343.1
1,269,796	1,269,796	1	G -> T		74	SNP (transversion)	98.6%		522	GCC -> GCA	C770_RS47765	ABC transporter ATPase	None	9.3E-240	174	3	WP_015243344.1
1,269,883	1,269,883	1	G -> C		76	SNP (transversion)	100.0%		435	CCC -> CCG	C770_RS47765	ABC transporter ATPase	None	1.0E-266	145	3	WP_015243344.1
1,269,934	1,269,934	1	A -> G		82	SNP (transition)	100.0%		384	GCT -> GCC	C770_RS47765	ABC transporter ATPase	None	2.5E-271	128	3	WP_015243344.1
1,269,984	1,269,984	1	C -> T		86	SNP (transition)	100.0%	V -> I	334	GTC -> ATC	C770_RS47765	ABC transporter ATPase	Substitution	4.0E-293	112	1	WP_015243344.1
1,269,995	1,269,995	1	G -> C		82	SNP (transversion)	100.0%	P -> R	323	CCG -> CGG	C770_RS47765	ABC transporter ATPase	Substitution	1.6E-279	108	2	WP_015243344.1
1,270,026	1,270,026	1	T -> C		80	SNP (transition)	98.8%	I -> V	292	ATC -> GTC	C770_RS47765	ABC transporter ATPase	Substitution	2.5E-275	98	1	WP_015243344.1
1,270,135	1,270,135	1	A -> C		94	SNP (transversion)	100.0%	I -> M	183	ATT -> ATG	C770_RS47765	ABC transporter ATPase	Substitution	6.3E-311	61	3	WP_015243344.1
1,270,252	1,270,252	1	A -> G		81	SNP (transition)	98.8%		66	TTT -> TTC	C770_RS47765	ABC transporter ATPase	None	8.1E-263	22	3	WP_015243344.1
1,270,282	1,270,283	2	CG -> GC	77 -> 78		Substitution	100.0%	S -> C	35	TCG -> TGC	C770_RS47765	ABC transporter ATPase	Substitution	1.0E-273	12	2	WP_015243344.1
1,270,335	1,270,335	1	G -> C		80	SNP (transversion)	100.0%		1.782	GGC -> GGG	C770_RS47770	peptide ABC transporte	None	1.0E-280	594	3	WP_015243345.1
1,270,743	1,270,743	1	C -> T		100	SNP (transition)	100.0%		1.374	CCG -> CCA	C770_RS47770	peptide ABC transporte	None	0.0	458	3	WP_015243345.1
1,270,771	1,270,771	1	G -> A		90	SNP (transition)	100.0%	A -> V	1.346	GCC -> GTC	C770_RS47770	peptide ABC transporte	Substitution	1.0E-306	449	2	WP_015243345.1
1,270,801	1,270,801	1	A -> G		91	SNP (transition)	100.0%	V -> A	1.316	GTC -> GCC	C770_RS47770	peptide ABC transporte	Substitution	6.3E-292	439	2	WP_015243345.1
1,270,884	1,270,884	1	C -> A		77	SNP (transversion)	100.0%		1.233	GTG -> GTT	C770_RS47770	peptide ABC transporte	None	7.9E-255	411	3	WP_015243345.1
1,270,913	1,270,913	1	G -> C		82	SNP (transversion)	100.0%	P -> A	1.204	CCG -> GCG	C770_RS47770	peptide ABC transporte	Substitution	1.6E-279	402	1	WP_015243345.1
1,270,940	1,270,940	1	A -> G		93	SNP (transition)	100.0%	F -> L	1.177	TTT -> CTT	C770_RS47770	peptide ABC transporte	Substitution	0.0	393	1	WP_015243345.1
1,271,004	1,271,004	1	G -> C		84	SNP (transversion)	100.0%		1.113	GGC -> GGG	C770_RS47770	peptide ABC transporte	None	6.3E-278	371	3	WP_015243345.1
1,271,013	1,271,013	1	C -> G		79	SNP (transversion)	100.0%		1.104	TCG -> TCC	C770_RS47770	peptide ABC transporte	None	2.5E-269	368	3	WP_015243345.1
1,271,262	1,271,262	1	G -> C		78	SNP (transversion)	100.0%		855	GTC -> GTG	C770_RS47770	peptide ABC transporte	None	6.3E-266	285	3	WP_015243345.1
1,271,346	1,271,346	1	A -> G		79	SNP (transition)	98.7%		771	GCT -> GCC	C770_RS47770	peptide ABC transporte	None	3.1E-256	257	3	WP_015243345.1
1,271,799	1,271,799	1	C -> G		73	SNP (transversion)	100.0%		318	GGG -> GGC	C770_RS47770	peptide ABC transporte	None	6.3E-249	106	3	WP_015243345.1
1,271,927	1,271,927	1	G -> T		82	SNP (transversion)	97.6%	Q -> K	190	CAG -> AAG	C770_RS47770	peptide ABC transporte	Substitution	3.3E-253	64	1	WP_015243345.1
1,272,081	1,272,081	1	T -> C		82	SNP (transition)	100.0%		36	AGA -> AGG	C770_RS47770	peptide ABC transporte	None	2.5E-271	12	3	WP_015243345.1
1,272,121	1,272,121	1	C -> G		75	SNP (transversion)	100.0%	G -> R	940	GGC -> CGC	C770_RS47775	peptide ABC transporte	Substitution	3.2E-263	314	1	WP_015243346.1
1,272,140	1,272,140	1	C -> T		74	SNP (transition)	98.6%		921	TCG -> TCA	C770_RS47775	peptide ABC transporte	None	9.3E-240	307	3	WP_015243346.1
1,272,151	1,272,151	1	C -> G		70	SNP (transversion)	100.0%	V -> L	910	GTC -> CTC	C770_RS47775	peptide ABC transporte	Substitution	1.0E-238	304	1	WP_015243346.1
1,272,173	1,272,173	1	G -> C		76	SNP (transversion)	100.0%		888	GTC -> GTG	C770_RS47775	peptide ABC transporte	None	1.0E-266	296	3	WP_015243346.1
1,272,230	1,272,230	1	A -> C		79	SNP (transversion)	98.7%		831	TCT -> TCG	C770_RS47775	peptide ABC transporte	None	2.0E-287	277	3	WP_015243346.1
1,272,546	1,272,546	1	T -> C		93	SNP (transition)	100.0%	D -> G	515	GAC -> GGC	C770_RS47775	peptide ABC transporte	Substitution	1.3E-307	172	2	WP_015243346.1
1,272,701	1,272,701	1	G -> C		86	SNP (transversion)	100.0%		360	TCC -> TCG	C770_RS47775	peptide ABC transporte	None	6.3E-276	120	3	WP_015243346.1
1,272,704	1,272,706	2	GAT -> AAC	85 -> 86		Substitution	100.0%	I -> V	355	ATC -> GTT	C770_RS47775	peptide ABC transporte	Substitution	3.2E-281	119	1	WP_015243346.1
1,272,815	1,272,815	1	C -> A		80	SNP (transversion)	98.8%		246	GCG -> GCT	C770_RS47775	peptide ABC transporte	None	1.6E-259	82	3	WP_015243346.1
1,272,876	1,272,876	1	A -> G		89	SNP (transition)	100.0%	V -> A	185	GTG -> GCG	C770_RS47775	peptide ABC transporte	Substitution	2.5E-303	62	2	WP_015243346.1
1,272,887	1,272,887	1	G -> A		92	SNP (transition)	100.0%		174	GAC -> GAT	C770_RS47775	peptide ABC transporte	None	1.6E-313	58	3	WP_015243346.1
1,273,109	1,273,109	1	G -> C		86	SNP (transversion)	100.0%							1.6E-284			
1,273,198	1,273,198	1	A -> G		75	SNP (transition)	100.0%		1.506	GTT -> GTC	C770_RS47780	peptide ABC transporte	None	3.2E-263	502	3	WP_015243347.1
1,273,243	1,273,243	1	A -> G		85	SNP (transition)	100.0%		1.461	CTT -> CTC	C770_RS47780	peptide ABC transporte	None	3.2E-298	487	3	WP_015243347.1
1,273,369	1,273,369	1	G -> T		71	SNP (transversion)	100.0%		1.335	TCC -> TCA	C770_RS47780	peptide ABC transporte	None	2.5E-256	445	3	WP_015243347.1
1,273,573	1,273,573	1	T -> C		80	SNP (transition)	100.0%		1.131	GCA -> GCG	C770_RS47780	peptide ABC transporte	None	1.0E-264	377	3	WP_015243347.1
1,273,648	1,273,648	1	C -> G		79	SNP (transversion)	98.7%		1.056	GGG -> GGC	C770_RS47780	peptide ABC transporte	None	7.9E-272	352	3	WP_015243347.1
1,273,677	1,273,677	1	G -> T		85	SNP (transversion)	100.0%	Q -> K	1.027	CAG -> AAG	C770_RS47780	peptide ABC transporte	Substitution	3.2E-264	343	1	WP_015243347.1
1,273,792	1,273,792	1	T -> C		76	SNP (transition)	100.0%		912	AAA -> AAG	C770_RS47780	peptide ABC transporte	None	1.0E-266	304	3	WP_015243347.1
1,273,873	1,273,873	1	T -> G		86	SNP (transversion)	100.0%		831	GTA -> GTC	C770_RS47780	peptide ABC transporte	None	1.6E-284	277	3	WP_015243347.1
1,274,080	1,274,080	1	A -> G		70	SNP (transition)	100.0%		624	CGT -> CGC	C770_RS47780	peptide ABC transporte	None	1.0E-238	208	3	WP_015243347.1
1,274,146	1,274,146	1	C -> T		58	SNP (transition)	100.0%		558	AAG -> AAA	C770_RS47780	peptide ABC transporte	None	6.3E-198	186	3	WP_015243347.1
1,274,152	1,274,152	1	A -> G		57	SNP (transition)	100.0%		552	CAT -> CAC	C770_RS47780	peptide ABC transporte	None	3.2E-200	184	3	WP_015243347.1
1,274,156	1,274,156	1	T -> C		54	SNP (transition)	98.1%	K -> R	548	AAA -> AGA	C770_RS47780	peptide ABC transporte	Substitution	6.8E-174	183	2	WP_015243347.1
1,274,168	1,274,168	1	A -> G		52	SNP (transition)	100.0%	V -> A	536	GTC -> GCC	C770_RS47780	peptide ABC transporte	Substitution	6.3E-162	179	2	WP_015243347.1
1,274,269	1,274,269	1	A -> G		55	SNP (transition)	98.2%		435	GTT -> GTC	C770_RS47780	peptide ABC transporte	None	5.5E-188	145	3	WP_015243347.1
1,274,287	1,274,287	1	G -> A		59	SNP (transition)	98.3%		417	TTC -> TTT	C770_RS47780	peptide ABC transporte	None	3.7E-196	139	3	WP_015243347.1
1,274,333	1,274,333	1	C -> T		56	SNP (transition)	100.0%	G -> D	371	GGC -> GAC	C770_RS47780	peptide ABC transporte	Substitution	2.5E-202	124	2	WP_015243347.1
1,274,335	1,274,335	1	T -> C		58	SNP (transition)	98.3%		369	AAA -> AAG	C770_RS47780	peptide ABC transporte	None	9.2E-193	123	3	WP_015243347.1
1,274,383	1,274,386	3	ATTC -> GTCG	56 -> 57		Substitution	98.2%	SN -> SD	318	TCG,AAT -> TCC,(	C770_RS47780	peptide ABC transporte	Substitution	2.3E-189	106	3	WP_015243347.1
1,274,420	1,274,420	1	G -> T		63	SNP (transversion)	100.0%	T -> K	284	ACG -> AAG	C770_RS47780	peptide ABC transporte	Substitution	5.0E-196	95	2	WP_015243347.1
1,274,440	1,274,440	1	A -> G		61	SNP (transition)	100.0%		264	GAT -> GAC	C770_RS47780	peptide ABC transporte	None	4.0E-208	88	3	WP_015243347.1
1,274,443	1,274,443	1	G -> A		64	SNP (transition)	100.0%		261	GAC -> GAT	C770_RS47780	peptide ABC transporte	None	1.0E-224	87	3	WP_015243347.1
1,274,536	1,274,536	1	G -> A		68	SNP (transition)	98.5%		168	CGC -> CGT	C770_RS47780	peptide ABC transporte	None	5.4E-220	56	3	WP_015243347.1
1,274,611	1,274,611	1	A -> G		72	SNP (transition)	98.6%		93	GAT -> GAC	C770_RS47780	peptide ABC transporte	None	2.9E-240	31	3	WP_015243347.1
1,274,743	1,274,742	0	A		71	Insertion	100.0%							3.2E-249			
1,274,774	1,274,774	1	G -> A		80	SNP (transition)	100.0%							1.0E-272			
1,274,818	1,274,818	1	A -> G		85	SNP (transition)	100.0%							1.0E-289			
1,274,831	1,274,831	1	(C)4 -> (C)3		86	Deletion (tandem repeat)	100.0%							1.0E-129			
1,274,917	1,274,917	1	T -> C		83	SNP (transition)	98.8%							1.3E-277			
1,274,924	1,274,924	1	G -> A		85	SNP (transition)	100.0%							1.0E-289			
1,274,973	1,274,973	1	C -> G		89	SNP (transversion)	100.0%							1.0E-267			
1,275,018	1,275,018	1	G -> C		99	SNP (transversion)	100.0%							0.0			
1,275,042	1,275,042	1	A -> G		98	SNP (transition)	100.0%							4.9E-324			
1,275,066	1,275,066	1	A -> G		98	SNP (transition)	99.0%		24	CTA -> CTG	C770_RS47785	hypothetical protein	None	0.0	8	3	WP_015243348.1
1,275,090	1,275,090	1	A -> T		98	SNP (transversion)	100.0%	E -> D	48	GAA -> GAT	C770_RS47785	hypothetical protein	Substitution	0.0	16	3	WP_015243348.1
1,275,443	1,275,443	1	A -> C		89	SNP (transversion)	97.8%	K -> T	401	AAA -> ACA	C770_RS47785	hypothetical protein	Substitution	2.5E-310	134	2	WP_015243348.1
1,275,453	1,275,453	1	C -> T		90	SNP (transition)	100.0%		411	TTC -> TTT	C770_RS47785	hypothetical protein	None	1.0E-297	137	3	WP_015243348.1
1,275,483	1,275,483	1	T -> C		89	SNP (transition)	100.0%		441	GAT -> GAC	C770_RS47785	hypothetical protein	None	2.5E-303	147	3	WP_015243348.1
1,275,584	1,275,584	1	T -> C		92	SNP (transition)	100.0%		60	TAT -> TAC	C770_RS47790	hypothetical protein	None	1.0E-322	20	3	WP_014527459.1
1,275,801	1,275,801	1	A -> G		99	SNP (transition)	99.0%							4.0E-322			
1,275,837	1,275,837	1	C -> A		107	SNP (transversion)	100.0%							0.0			
1,275,877	1,275,876	0	(C)2 -> (C)3		101	Insertion (tandem repeat)	100.0%							0.0			
1,275,932	1,275,932	1	C -> G		85	SNP (transversion)	100.0%							3.2E-298			
1,275,937	1,275,937	1	C -> T		84	SNP (transition)	100.0%							1.0E-294			
1,275,985	1,275,985	1	C -> T		80	SNP (transition)	100.0%							1.0E-280			
1,276,079	1,276,079	1	C -> T		77	SNP (transition)	98.7%							7.7E-265			
1,276,097	1,276,097	1	G -> A		73	SNP (transition)	100.0%							2.5E-234			
1,276,113	1,276,113	1	A -> G		69	SNP (transition)	100.0%							3.2E-242			
1,276,720	1,276,720	1	G -> T		73	SNP (transversion)	100.0%		559	CGG -> AGG	C770_RS47795	peptidase M42	None	3.2E-256	187	1	WP_013849941.1
1,278,028	1,278,028	1	C -> T		94	SNP (transition)	100.0%		576	GAC -> GAT	C770_RS47800	glucokinase	None	2.5E-320	192	3	WP_014527460.1
1,278,031	1,278,031	1	C -> A		95	SNP (transversion)	100.0%		579	ATC -> ATA	C770_RS47800	glucokinase	None	1.0E-323	193	3	WP_014527460.1
1,278,034	1,278,034	1	C -> G		95	SNP (transversion)	100.0%		582	GGC -> GGG	C770_RS47800	glucokinase	None	1.0E-323	194	3	WP_014527460.1
1,278,079	1,278,079	1	C -> T		87	SNP (transition)	100.0%		627	GAC -> GAT	C770_RS47800	glucokinase	None	1.6E-296	209	3	WP_014527460.1
1,278,325	1,278,325	1	G -> A		77	SNP (transition)	100.0%		873	GAG -> GAA	C770_RS47800	glucokinase	None	1.6E-262	291	3	WP_014527460.1
1,278,520	1,278,520	1	C -> T		61	SNP (transition)	100.0%		1.068	AAC -> AAT	C770_RS47800	glucokinase	None	6.3E-196	356	3	WP_014527460.1
1,279,029	1,279,029	1	G -> A		92	SNP (transition)	97.8%							4.2E-294			
1,279,075	1,279,076	2	TT -> CC	88 -> 89		Substitution	98.9%							1.3E-276			
1,279,269	1,279,269	1	T -> C		86	SNP (transition)	100.0%		420	AGA -> AGG	C770_RS47810	hypothetical protein	None	1.6E-284	140	3	WP_014527461.1
1,279,874	1,279,874	1	G -> A		77	SNP (transition)	98.7%		792	GGC -> GGT	C770_RS47815	glycosyl hydrolase famil	None	1.2E-249	264	3	WP_010975322.1
1,279,907	1,279,907	1	T -> C		76	SNP (transition)	100.0%		759	AAA -> AAG	C770_RS47815	glycosyl hydrolase famil	None	4.0E-259	253	3	WP_010975322.1
1,279,910	1,279,910	1	T -> G		75	SNP (transversion)	100.0%		756	GGA -> GGC	C770_RS47815	glycosyl hydrolase famil	None	3.2E-248	252	3	WP_010975322.1

1,280,024	1,280,024	1	G -> A	72	SNP (transition)	98.6%		642	GAC -> GAT	C770_RS47815	glycosyl hydrolase famil	None	2.9E-240	214	3	WP_010975322.1
1,280,189	1,280,189	1	G -> A	78	SNP (transition)	100.0%		477	ACC -> ACT	C770_RS47815	glycosyl hydrolase famil	None	6.3E-266	159	3	WP_010975322.1
1,280,273	1,280,273	1	A -> G	88	SNP (transition)	98.9%		393	GAT -> GAC	C770_RS47815	glycosyl hydrolase famil	None	2.8E-303	131	3	WP_010975322.1
1,280,328	1,280,328	1	T -> A	101	SNP (transversion)	100.0%	E -> V	338	GAG -> GTG	C770_RS47815	glycosyl hydrolase famil	Substitution	0.0	113	2	WP_010975322.1
1,280,567	1,280,567	1	G -> C	109	SNP (transversion)	100.0%		99	CCC -> CCG	C770_RS47815	glycosyl hydrolase famil	None	0.0	33	3	WP_010975322.1
1,280,627	1,280,627	1	G -> A	105	SNP (transition)	99.0%		39	CTC -> CTT	C770_RS47815	glycosyl hydrolase famil	None	0.0	13	3	WP_010975322.1
1,280,709	1,280,709	1	C -> T	99	SNP (transition)	100.0%	G -> S	568	GGC -> AGC	C770_RS47820	DUF995 domain-contair	Substitution	0.0	190	1	WP_015243349.1
1,280,755	1,280,755	1	C -> T	97	SNP (transition)	100.0%		522	GCG -> GCA	C770_RS47820	DUF995 domain-contair	None	0.0	174	3	WP_015243349.1
1,280,810	1,280,811	2	CC -> GT	96	Substitution	97.9%	G -> T	466	GGA -> ACA	C770_RS47820	DUF995 domain-contair	Substitution	1.1E-316	156	1	WP_015243349.1
1,280,843	1,280,843	1	C -> T	90	SNP (transition)	98.9%	G -> D	434	GGC -> GAC	C770_RS47820	DUF995 domain-contair	Substitution	2.8E-310	145	2	WP_015243349.1
1,281,085	1,281,085	1	C -> G	86	SNP (transversion)	97.7%		192	GGG -> GGC	C770_RS47820	DUF995 domain-contair	None	9.2E-283	64	3	WP_015243349.1
1,281,094	1,281,094	1	A -> G	88	SNP (transition)	97.7%		183	ATT -> ATC	C770_RS47820	DUF995 domain-contair	None	1.5E-289	61	3	WP_015243349.1
1,281,106	1,281,106	1	T -> C	88	SNP (transition)	97.7%		171	GAA -> GAG	C770_RS47820	DUF995 domain-contair	None	3.8E-298	57	3	WP_015243349.1
1,281,178	1,281,178	1	T -> A	98	SNP (transversion)	99.0%		99	CTA -> CTT	C770_RS47820	DUF995 domain-contair	None	7.8E-319	33	3	WP_015243349.1
1,281,182	1,281,182	1	G -> A	99	SNP (transition)	98.0%	A -> V	95	GCC -> GTC	C770_RS47820	DUF995 domain-contair	Substitution	3.8E-317	32	2	WP_015243349.1
1,281,186	1,281,186	1	C -> T	100	SNP (transition)	98.0%	A -> T	91	GCC -> ACC	C770_RS47820	DUF995 domain-contair	Substitution	2.0E-320	31	1	WP_015243349.1
1,281,225	1,281,225	1	C -> T	92	SNP (transition)	97.8%	A -> T	52	GCG -> ACG	C770_RS47820	DUF995 domain-contair	Substitution	4.2E-294	18	1	WP_015243349.1
1,281,227	1,281,227	1	C -> T	90	SNP (transition)	96.7%	R -> H	50	CGC -> CAC	C770_RS47820	DUF995 domain-contair	Substitution	9.3E-283	17	2	WP_015243349.1
1,281,248	1,281,248	1	T -> C	83	SNP (transition)	100.0%	K -> R	29	AAA -> AGA	C770_RS47820	DUF995 domain-contair	Substitution	2.5E-266	10	2	WP_015243349.1
1,281,294	1,281,294	1	A -> G	86	SNP (transition)	100.0%	L -> S	1.853	TTA -> TCA	C770_RS47825	glycosyltransferase prol	Substitution	1.6E-284	618	2	WP_015243350.1
1,281,347	1,281,347	1	T -> G	87	SNP (transversion)	100.0%		1.8	ATA -> ATC	C770_RS47825	glycosyltransferase prol	None	7.9E-288	600	3	WP_015243350.1
1,281,452	1,281,452	1	C -> T	77	SNP (transition)	98.7%		1.695	AAG -> AAA	C770_RS47825	glycosyltransferase prol	None	3.1E-257	565	3	WP_015243350.1
1,282,444	1,282,444	1	T -> C	106	SNP (transition)	99.1%	K -> E	703	AAG -> GAG	C770_RS47825	glycosyltransferase prol	Substitution	0.0	235	1	WP_015243350.1
1,282,665	1,282,665	1	G -> T	113	SNP (transversion)	100.0%	A -> E	482	GCA -> GAA	C770_RS47825	glycosyltransferase prol	Substitution	0.0	161	2	WP_015243350.1
1,282,682	1,282,682	1	G -> C	106	SNP (transversion)	100.0%		465	TCC -> TCG	C770_RS47825	glycosyltransferase prol	None	0.0	155	3	WP_015243350.1
1,282,700	1,282,700	1	C -> G	103	SNP (transversion)	100.0%	R -> S	447	AGG -> AGC	C770_RS47825	glycosyltransferase prol	Substitution	0.0	149	3	WP_015243350.1
1,282,715	1,282,717	2	GAT -> TAG	93 -> 95	Substitution	98.9% -> 100.0%	I -> L	430	ATC -> CTA	C770_RS47825	glycosyltransferase prol	Substitution	2.4E-318	144	1	WP_015243350.1
1,282,856	1,282,857	2	GA -> CG	88 -> 89	Substitution	98.9%	V -> A	290	GTC -> GCG	C770_RS47825	glycosyltransferase prol	Substitution	3.5E-289	97	2	WP_015243350.1
1,282,888	1,282,888	1	A -> G	77	SNP (transition)	100.0%		259	TTG -> CTG	C770_RS47825	glycosyltransferase prol	None	3.2E-270	87	1	WP_015243350.1
1,282,904	1,282,904	1	C -> G	77	SNP (transversion)	98.7%		243	GCG -> GCC	C770_RS47825	glycosyltransferase prol	None	1.2E-249	81	3	WP_015243350.1
1,282,919	1,282,919	1	G -> C	78	SNP (transversion)	100.0%		228	CCC -> CCG	C770_RS47825	glycosyltransferase prol	None	6.3E-266	76	3	WP_015243350.1
1,282,964	1,282,964	1	A -> G	74	SNP (transition)	100.0%		183	GGT -> GGC	C770_RS47825	glycosyltransferase prol	None	2.5E-252	61	3	WP_015243350.1
1,283,024	1,283,024	1	C -> T	68	SNP (transition)	100.0%		123	GCG -> GCA	C770_RS47825	glycosyltransferase prol	None	6.3E-232	41	3	WP_015243350.1
1,283,036	1,283,036	1	C -> T	64	SNP (transition)	100.0%		111	GGG -> GGA	C770_RS47825	glycosyltransferase prol	None	2.5E-218	37	3	WP_015243350.1
1,283,152	1,283,152	1	C -> G	49	SNP (transversion)	100.0%		978	GTG -> GTC	C770_RS47830	UDP-glucose-4-epimera	None	1.6E-157	326	3	WP_015243351.1
1,283,242	1,283,242	1	T -> C	58	SNP (transition)	98.3%		888	GGA -> GGG	C770_RS47830	UDP-glucose-4-epimera	None	4.6E-187	296	3	WP_015243351.1
1,283,668	1,283,668	1	C -> G	84	SNP (transversion)	100.0%		462	CTG -> CTC	C770_RS47830	UDP-glucose-4-epimera	None	2.5E-286	154	3	WP_015243351.1
1,284,161	1,284,161	1	G -> A	70	SNP (transition)	100.0%	R -> W	1.015	CGG -> TGG	C770_RS47835	dTDP-glucose 4,6-dehyc	Substitution	1.0E-245	339	1	WP_010975318.1
1,284,879	1,284,879	1	G -> C	85	SNP (transversion)	98.8%		297	CTC -> CTG	C770_RS47835	dTDP-glucose 4,6-dehyc	None	5.4E-276	99	3	WP_010975318.1
1,285,472	1,285,472	1	A -> G	78	SNP (transition)	100.0%							4.0E-258			
1,285,958	1,285,958	1	C -> G	96	SNP (transversion)	99.0%		426	GTC -> GTG	C770_RS47840	AraC family transcriptio	None	3.0E-312	142	3	WP_015243352.1
1,285,971	1,285,971	1	T -> C	96	SNP (transition)	100.0%	S -> P	439	TCG -> CCG	C770_RS47840	AraC family transcriptio	Substitution	6.3E-308	147	1	WP_015243352.1
1,286,322	1,286,322	1	T -> A	87	SNP (transversion)	100.0%	Y -> N	790	TAC -> AAC	C770_RS47840	AraC family transcriptio	Substitution	2.5E-244	264	1	WP_015243352.1
1,287,253	1,287,253	1	C -> T	81	SNP (transition)	100.0%	G -> R	256	GGG -> AGG	C770_RS47845	3-ketoacyl-ACP reducta:	Substitution	3.2E-284	86	1	WP_010975316.1
1,287,678	1,287,678	1	G -> C	70	SNP (transversion)	100.0%		66	GTG -> GTC	C770_RS47850	transcriptional regulato	None	1.0E-245	22	3	WP_015243353.1
1,287,690	1,287,690	1	A -> G	71	SNP (transition)	100.0%		78	AAA -> AAG	C770_RS47850	transcriptional regulato	None	5.0E-235	26	3	WP_015243353.1
1,287,747	1,287,747	1	C -> G	73	SNP (transversion)	98.6%		135	CCC -> CCG	C770_RS47850	transcriptional regulato	None	7.3E-251	45	3	WP_015243353.1
1,287,765	1,287,765	1	C -> T	79	SNP (transition)	98.7%		153	TTC -> TTT	C770_RS47850	transcriptional regulato	None	3.1E-256	51	3	WP_015243353.1
1,288,007	1,288,007	1	C -> A	89	SNP (transversion)	97.8%	T -> K	395	ACG -> AAG	C770_RS47850	transcriptional regulato	Substitution	3.1E-284	132	2	WP_015243353.1
1,288,026	1,288,026	1	G -> A	89	SNP (transition)	100.0%		414	CTG -> CTA	C770_RS47850	transcriptional regulato	None	2.5E-303	138	3	WP_015243353.1
1,288,250	1,288,250	1	(A)3 -> (A)2	75	Deletion (tandem repeat)	100.0%							3.2E-128			
1,288,308	1,288,308	1	C -> A	76	SNP (transversion)	100.0%							1.0E-266			
1,288,321	1,288,321	1	(T)4 -> (T)3	84	Deletion (tandem repeat)	100.0%							4.0E-135			
1,288,403	1,288,403	1	A -> G	86	SNP (transition)	100.0%		324	TAT -> TAC	C770_RS47855	membrane protein	None	1.6E-284	108	3	WP_049589741.1
1,288,424	1,288,424	1	A -> G	76	SNP (transition)	100.0%		303	CCT -> CCC	C770_RS47855	membrane protein	None	6.3E-244	101	3	WP_049589741.1
1,288,430	1,288,430	1	T -> C	76	SNP (transition)	100.0%		297	CCA -> CCG	C770_RS47855	membrane protein	None	4.0E-259	99	3	WP_049589741.1
1,288,436	1,288,436	1	A -> G	74	SNP (transition)	100.0%		291	GTT -> GTC	C770_RS47855	membrane protein	None	2.5E-252	97	3	WP_049589741.1
1,288,469	1,288,469	1	T -> C	76	SNP (transition)	100.0%		258	GTA -> GTG	C770_RS47855	membrane protein	None	4.0E-259	86	3	WP_049589741.1
1,288,574	1,288,574	1	C -> G	86	SNP (transversion)	100.0%		153	GGG -> GGC	C770_RS47855	membrane protein	None	4.0E-293	51	3	WP_049589741.1
1,288,657	1,288,657	1	C -> T	71	SNP (transition)	100.0%	A -> T	70	GCC -> ACC	C770_RS47855	membrane protein	Substitution	3.2E-249	24	1	WP_049589741.1
1,288,804	1,288,804	1	G -> T	76	SNP (transversion)	98.7%							7.6E-254			
1,288,912	1,288,912	1	A -> G	73	SNP (transition)	98.6%							1.2E-243			
1,288,914	1,288,914	1	G -> A	74	SNP (transition)	100.0%							2.5E-252			
1,288,948	1,288,948	1	A -> G	80	SNP (transition)	100.0%							1.0E-272			
1,288,980	1,288,980	1	G -> A	83	SNP (transition)	98.8%							1.3E-277			
1,289,020	1,289,020	1	A -> G	80	SNP (transition)	100.0%							1.0E-272			
1,289,029	1,289,029	1	G -> C	78	SNP (transversion)	100.0%							6.3E-266			
1,289,069	1,289,069	1	G -> A	80	SNP (transition)	98.8%		36	GAG -> GAA	C770_RS47860	gluconolactonase	None	1.6E-259	12	3	WP_015243355.1
1,289,102	1,289,102	1	G -> C	81	SNP (transversion)	100.0%		69	GCG -> GCC	C770_RS47860	gluconolactonase	None	4.0E-276	23	3	WP_015243355.1
1,289,195	1,289,195	1	A -> G	91	SNP (transition)	100.0%		162	CAA -> CAG	C770_RS47860	gluconolactonase	None	3.2E-319	54	3	WP_015243355.1
1,289,250	1,289,250	1	G -> A	90	SNP (transition)	100.0%	D -> N	217	GAT -> AAT	C770_RS47860	gluconolactonase	Substitution	1.0E-279	73	1	WP_015243355.1
1,289,282	1,289,282	1	A -> G	85	SNP (transition)	100.0%		249	GAA -> GAG	C770_RS47860	gluconolactonase	None	1.0E-272	83	3	WP_015243355.1
1,289,365	1,289,365	1	G -> T	85	SNP (transversion)	100.0%	S -> I	332	AGT -> ATT	C770_RS47860	gluconolactonase	Substitution	3.2E-264	111	2	WP_015243355.1
1,289,483	1,289,483	1	G -> A	81	SNP (transition)	100.0%		450	GAG -> GAA	C770_RS47860	gluconolactonase	None	5.0E-268	150	3	WP_015243355.1
1,289,576	1,289,576	1	T -> C	85	SNP (transition)	100.0%		543	CTT -> CTC	C770_RS47860	gluconolactonase	None	3.2E-298	181	3	WP_015243355.1
1,289,600	1,289,600	1	G -> A	82	SNP (transition)	98.8%		567	CGG -> CGA	C770_RS47860	gluconolactonase	None	3.3E-274	189	3	WP_015243355.1
1,289,645	1,289,645	1	G -> C	89	SNP (transversion)	100.0%	R -> S	612	AGG -> AGC	C770_RS47860	gluconolactonase	Substitution	2.5E-303	204	3	WP_015243355.1
1,289,681	1,289,681	1	C -> A	98	SNP (transversion)	100.0%		648	GTC -> GTA	C770_RS47860	gluconolactonase	None	0.0	216	3	WP_015243355.1
1,289,714	1,289,714	1	T -> C	100	SNP (transition)	100.0%		681	GAT -> GAC	C770_RS47860	gluconolactonase	None	0.0	227	3	WP_015243355.1
1,289,720	1,289,720	1	C -> T	100	SNP (transition)	100.0%		687	GGC -> GGT	C770_RS47860	gluconolactonase	None	0.0	229	3	WP_015243355.1
1,289,756	1,289,756	1	T -> C	99	SNP (transition)	100.0%		723	AAT -> AAC	C770_RS47860	gluconolactonase	None	1.6E-317	241	3	WP_015243355.1
1,289,808	1,289,808	1	C -> T	90	SNP (transition)	100.0%		775	CTG -> TTG	C770_RS47860	gluconolactonase	None	1.0E-297	259	1	WP_015243355.1
1,289,813	1,289,813	1	T -> C	91	SNP (transition)	100.0%		780	CTT -> CTC	C770_RS47860	gluconolactonase	None	3.2E-319	260	3	WP_015243355.1
1,289,849	1,289,849	1	C -> A	98	SNP (transversion)	100.0%		816	CTC -> CTA	C770_RS47860	gluconolactonase	None	4.9E-324	272	3	WP_015243355.1
1,289,894	1,289,894	1	C -> G	95	SNP (transversion)	98.9%		861	ACC -> ACG	C770_RS47860	gluconolactonase	None	0.0	287	3	WP_015243355.1
1,289,896	1,289,896	1	G -> A	97	SNP (transition)	100.0%	R -> Q	863	CGG -> CAG	C770_RS47860	gluconolactonase	Substitution	0.0	288	2	WP_015243355.1
1,289,944	1,289,944	1	G -> A	87	SNP (transition)	100.0%		911	TGA -> TAA	C770_RS47860	gluconolactonase	None	3.2E-305	304	2	WP_015243355.1
1,289,990	1,289,990	1	T -> C	74	SNP (transition)	100.0%		330	AGA -> AGG	C770_RS47865	hypothetical protein	None	2.5E-252	110	3	WP_010975312.1
1,290,002	1,290,002	1	G -> A	75	SNP (transition)	98.7%		318	GGC -> GGT	C770_RS47865	hypothetical protein	None	1.9E-250	106	3	WP_010975312.1
1,290,484	1,290,484	1	T -> C	79	SNP (transition)	100.0%		414	TCA -> TCG	C770_RS47870	ATP-binding protein	None	1.3E-245	138	3	WP_003526433.1
1,290,620	1,290,620	1	G -> T	79	SNP (transversion)	98.7%	T -> N	278	ACC -> AAC	C770_RS47870	ATP-binding protein	Substitution	3.1E-256	93	2	WP_003526433.1
1,290,712	1,290,712	1	G -> A	86	SNP (transition)	100.0%		186	CAC -> CAT	C770_RS47870	ATP-binding protein	None	4.0E-293	62	3	WP_003526433.1
1,290,730	1,290,730	1	G -> A	83	SNP (transition)	98.8%		168	CTC -> CTT	C770_RS47870	ATP-binding protein	None	2.1E-269	56	3	WP_003526433.1
1,291,267	1,291,267	1	A -> G	96	SNP (transition)	99.0%		1.996	TTG -> CTG	C770_RS47875	Serine phosphatase Rsb	None	3.0E-312	666	1	WP_015243356.1
1,291,517	1,291,517	1	A -> G	76	SNP (transition)	100.0%		1.746	GGT -> GGC	C770_RS47875	Serine phosphatase Rsb	None	1.6E-251	582	3	WP_015243356.1
1,292,075	1,292,075	1	T -> C	87	SNP (transition)	100.0%		1.188	CCA -> CCG	C770_RS47875	Serine phosphatase Rsb	None	1.6E-296	396	3	WP_015243356.1
1,292,117	1,292,117	1	G -> C	85	SNP (transversion)	100.0%		1.146	GTC -> GTG	C770_RS47875	Serine phosphatase Rsb	None	3.2E-298	382	3	WP_015243356.1
1,293,065	1,293,065	1	G -> A	84	SNP (transition)	100.0%		198	ATC -> ATT	C770_RS47875	Serine phosphatase Rsb	None	4.0E-261	66	3	WP_015243356.1
1,293,439	1,293,439	1	A -> G	67	SNP (transition)	100.0%		999	GCT -> GCC	C770_RS47880	ubiquinone/menaquino	None	1.6E-228	333	3	WP_015243357.1
1,293,658	1,293,658	1	G -> A	71	SNP (transition)	100.0%		780	GTC -> GTT	C770_RS47880	ubiquinone/menaquino	None	4.0E-242	260	3	WP_015243357.1
1,294,030	1,294,030	1	G -> T	96	SNP (transversion)	99.0%		408	ATC -> ATA	C770_RS47880	ubiquinone/menaquino	None	9.6E-303	136	3	WP_015243357.1
1,294,189	1,294,189	1	C -> A	85	SNP (transversion)	98.8%		249	TCG -> TCT	C770_RS47880	ubiquinone/menaquino	None	8.5E-293	83	3	WP_015243357.1
1,294,708	1,294,708	1	G -> A	87	SNP (transition)	100.0%	A -> V	245	GCA -> GTA	C770_RS47885	hypothetical protein	Substitution	1.6E-296	82	2	WP_003525909.1
1,294,755	1,294,757	2	GAG -> TAA	87 -> 88	Substitution	98.9%		196	CTC -> TTA	C770_RS47885	hypothetical protein	None	4.0E-293	66	1	WP_003525909.1
1,295,006	1,295,006	1	A -> G	82	SNP (transition)	100.0%							1.0E-287			
1,295,025	1,295,025	1	G -> A	82	SNP (transition)	100.0%							1.6E-279			
1,295,082	1,295,082	1	T -> G	86	SNP (transversion)	100.0%		1.641	GCA -> GCC	C770_RS47890	diguanylate cyclase	None	2.5E-267	547	3	WP_015243358.1
1,295,133	1,295,133	1	G -> A	80	SNP (transition)	98.8%		1.59	GAC -> GAT	C770_RS47890	diguanylate cyclase	None	2.0E-267	530	3	WP_015243358.1
1,295,193	1,295,193	1	G -> A	100	SNP (transition)	100.0%		1.53	GTC -> GTT	C770_RS47890	diguanylate cyclase	None	0.0	510	3	WP_015243358.1
1,295,268	1,295,268	1	G -> A	99	SNP (transition)	100.0%		1.455	GAC -> GAT	C770_RS47890	diguanylate cyclase	None	0.0	485	3	WP_015243358.1
1,295,577	1,295,577	1	G -> T	80	SNP (transversion)	100.0%		1.146	GCC -> GCA	C770_RS47890	diguanylate cyclase	None	1.0E-264	382	3	WP_015243358.1
1,295,853	1,295,853	1	C -> T	80	SNP (transition)	100.0%		870	CGG -> CGA	C770_RS47890	diguanylate cyclase	None	1.0E-272	290	3	WP_015243358.1
1,296,006	1,296,006	1	A -> G	85	SNP (transition)	100.0%		717	GCT -> GCC	C770_RS47890	diguanylate cyclase	None	1.0E-272	239	3	WP_015243358.1
1,296,234	1,296,234	1	G -> T	66	SNP (transversion)	98.5%		489	GCC -> GCA	C770_RS47890	diguanylate cyclase	None	2.1E-226	163	3	WP_015243358.1
1,296,315	1,296,315	1	C -> G	68	SNP (transversion)	100.0%		408	ACG -> ACC	C770_RS47890	diguanylate cyclase	None	1.0E-238	136	3	WP_015243358.1
1,349,665	1,349,665	1	C -> T	84	SNP (transition)	96.4%	T -> I	248	ACT -> ATT	C770_RS48135	hypothetical protein	Substitution	6.0E-255	83	2	WP_015243388.1
1,496,925	1,496,925	1	C -> T	70	SNP (transition)	100.0%		189	TCC -> TCT	C770_RS48835	glycosyltransferase	None	1.0E-217	63	3	WP_015243456.1

7G9 pRmeGR4b

Minimum	Maximum	Length	Change	Coverage	Polymorphism Type	Variant Frequency	Amino Acid Change	CDS Position	Codon Change	locus_tag	product	Protein Effect	Variant P-Value (approximate)	CDS Codon Number	CDS Position Within Codon	protein_id
12.335	12.335	1	G -> C	43	SNP (transversion)	97.7%							4.3E-125			
78.069	78.069	1	G -> C	198	SNP (transversion)	99.5%		186	GTC -> GTG	C770_RS34675	hypothetical protein	None	0.0	62	3	WP_015241587.1

G6 pRmeGR4b

Minimum	Maximum	Length	Change	Coverage	Polymorphism Type	Variant Frequency	Amino Acid Change	CDS Position	Codon Change	locus_tag	product	Protein Effect	Variant P-Value (approximate)	CDS Codon Number	CDS Position Within Codon	protein_id
6.236	6.238	3	GGT -> ACC	36	Substitution	97.2%							3.6E-111			
6.241	6.241	1	T -> A	35	SNP (transversion)	97.1%							8.8E-115			
12.335	12.335	1	G -> C	36	SNP (transversion)	100.0%							6.3E-116			

Data was obtained using a minimum fraction of reads of ≥0.95 that must contained a base to be called as a variation. The harbored transposases were omitted in the analysis.

Rows in green indicate the SNPs and INDELs shared by G6 and 7G9 in each replicon. Rows in yelow ochre indicate the SNPs and INDELs within the pSymB GID in 7G9 and G6 described in this work. Rows in blue indicate SNPs and INDELs within a specific pSymB island harboured by the isolate G6.

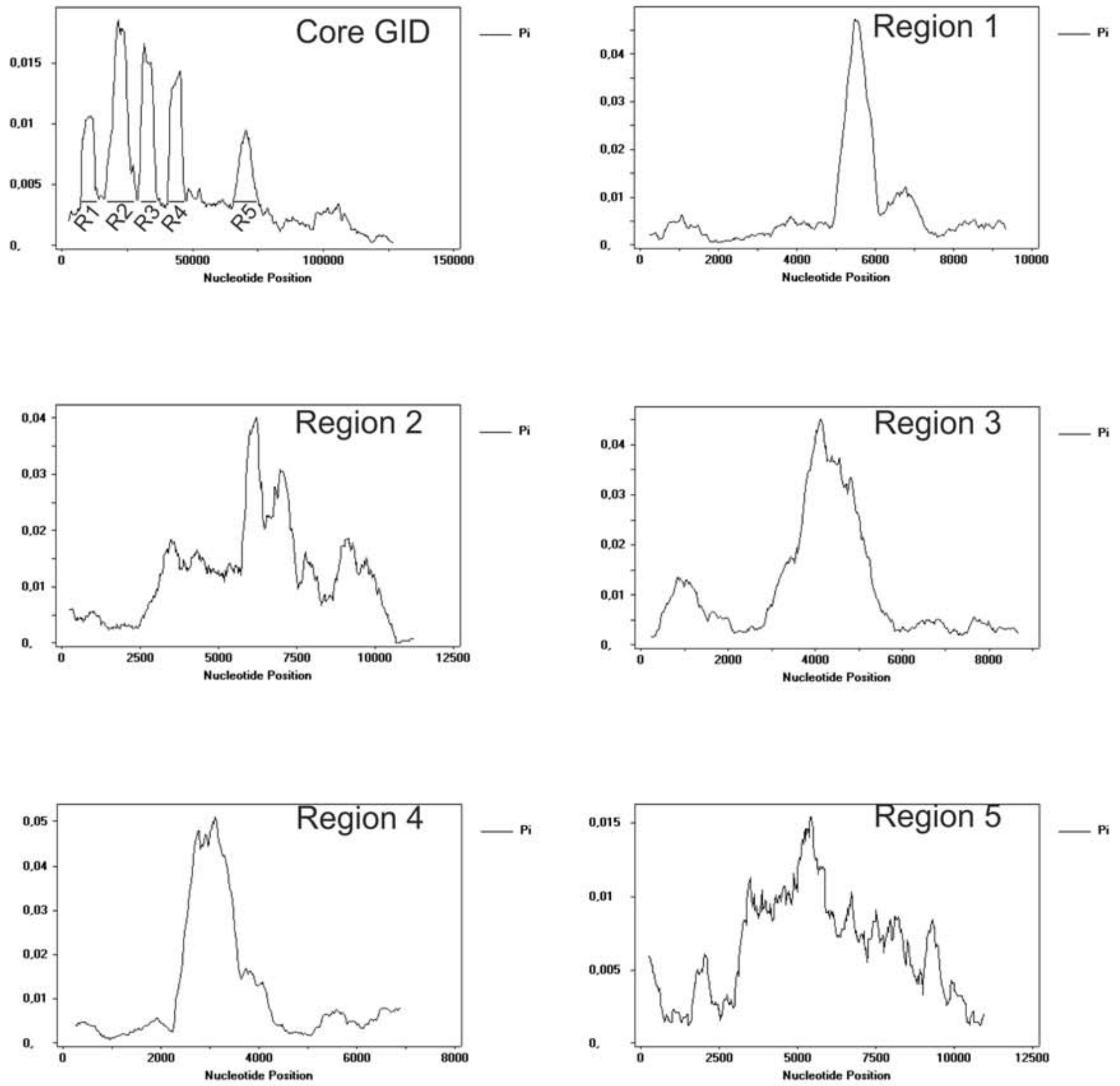

Supplementary Figure S1 Analysis of DNA polymorphisms in the GID. A sliding-window analysis of the core GID (a window length of 5,000 nts and steps of 250 nts) was performed with DnaSP (Librado and Rozas, 2009). This analysis identified five regions (R1 to R5) with higher nucleotide sequence diversity (π). A sliding window length of 500 nts, with steps of 25 nts, was then used to analyze each region, to identify the genes displaying the highest levels of nucleotide sequence diversity.

Supplementary Table S2. Primers used in PCR analyses

Primers	localization	GR4	G6	7G9
**881F** 5´TGCACCTGACGATCAACGAG 3´<br>**1612R** 5´GCTGGTTCTGGCAGGATTTG 3´	pSymB DNA polymerase (C770_RS45775, GI:752481673)	732pb	286pb	732pb
**7G9F** 5´AGCCGGGAATAGTACCACCT3´<br>**7G9R** 5´GCCTCGCTTTCGTTTTTGGT3´	Methyltransferase acquired by G6 and 7G9 in the GID (1 in Fig. 3)	-	974pb	974pb
**7G92F** 5´CCGGCATTCCCTACTACGTC3´<br>**7G92R** 5´TGCGTTGACGTCGACAAAAC3´	Glycosiltransferase acquired by G6 and 7G9 in the GID (8 in Fig. 3)	-	845pb	845pb
**999F** 5´CCATACCGCGATGAAGACGA3´<br>**1877R** 5´AAAGCAAAGTCGTTGGCGTC3´	pSymB Chromosome segregation ATPase-like protein (C770_RS47055, GI:752481725)	878pb	-	-

Supplementary Data 1
>7E2 Concatenation of 3 sequences
ATGGACATCAGCATTGCGCGTACGTTCCTCGAGGTGGTCAAGACGGGAAGCTTCGTGAATGCCGCTGCCAATC
TGAACATCTCCCAGACGGCAGTGAGTGCGCGAATTCGCGTACTCGAGGAACAACTCGATCGGCCGATGTTCGT
TCGGAACAAGGCCGGTGCCAGGCTGACCCCCGCCGGAGAGCAGTTTTTCCGTTTTGCAACGAGCCTAGTGCAA
GTATGGGAGGCGGCGCGAACCACGGTCGCTTTGCCCCCGGGACGCGAGGCCATGGTGACGATCGGTGCTGAGC
TCAGCCTGTGGAATCCGCTGTTGAAGCATTGGCTTTTGTGGATGCGCCGCGAATGCGCCAGCGTCGCCATTCG
AACGACGATAGACAGATCGGAGCGGCTGATGGAACTCGTGCAGGACGGTTCACTTGACGCGGCGGTCCTTTAT
GGAGCGCCAAATCGTCCAGGCACGCTTACGGAGCTGCTTTTCGAAGAAAAGCTCGTTCTCGTTCGCACTACAG
CTTTGTCGCAGCCGCCCGGACCGGAAGACCTCGTTGGCATCGATTGGGGAGAGGATTTTGCCGGCAGCTATAG
GGCGGCCTTTCCGGGCGGCCGGAATCCGGTGGTAGCGATCAGTTATGGGCCGCTTGCTCTCGATTACATTCTG
GAAGTCGGCGGATCCGGATATTTTCGGCAGGGCTTCGTTCGGCCCTATCTTGTGGACGGCCGCCTCGAGCTCG
TTCCCAACAGTCCGGAATTTGCCTACCCCGCTTATCTCGTTTCTTCCGCAAAGGCGGATCGGTCTATGCTGGA
TCGCATTCGGGCAGGATTGAGGGCCGCCGCATCCAGTGCCGGCGTCTGAATGTTCGGCATAGACATCACCGTC
CTGGGATTGGCCGGCCTCGTCGCCCTGGCGGCGGCAGCACTCGCCTATGGTGTGCTCTACCCCCAGATCGAGA
CCGAGAAGAAGGCGGAGGGCCGCCTGCGCCGGGTCAGCGCCTCCGAGACGGACCGCACCAAGATCAAGGCGGC
GCGCGACCGCGTCAACGAGATGTCGAAGCGACGCAAATCCGTTCAGGATTCGCTGAAGGAACTCGAGAAAAAG
CAGCAGGAAAAATCCGCCAATACGGCGCCTTCGATGAAGAAGCGCCTGCTGCAGGCCGGTCTTTCGATATCGC
TCGCGCAGTTCTACCTGTTCAGCGCGCTTTTCGGACTGTTCGCCTTCCTTGTCGTCCTCCTGGCAGGGGCGGG
GCTGCTCATTGCCGCGGGCGTTGCCCTGATAGGCGCGGCCGGCCTGCCGCGATGGATCGTCGGCTCCATGGTC
AAACGCCGTTGCAGAAAGTTTCTCGACGAGTTTCCGAATTCGCTCGATGTCATGGTCCGTTCGATCAAGTCGG
GGCTGCCGCTGAACGACGCCTTGCGGCTGATCGCCAGCGACGGGCAGGAGCCGGTCAGGACGGAATTCCGCCG
TGTCGTCGAGTCCCAGCAGGTGGGCCTCAACGTTCCCGAAGCCTGCGCCCGCATGATTCACAGCATCCCGCTG
CCGGAAGTGAACTTCTTCGCGATCGTCATCGCCATTCAGGCGCAGGCGGGCGGCAATCTGTCCGAAGCGCTCG
GCAATCTCTCCAAGGTGCTGCGCGAACGCAGGAAGATGAAGGCGAAGGTCAGCGCGCTGTCGATGGAGGCCAA
GGCGTCCGCCTGTATCATCGGCGCGCTGCCGTTCATCGTCGCAACCCTCGTCTATCTGACTTCGCCCGATTAC
ATGATGGTTCTCTTCACCGACCCGCGCGGCCACATCATCATGGGCGCTTCCGCCGTCTGGATGAGCATCGGCA
TCTGGGTGATGCGCAACATGATCAACTTCGACATCTGAGTGAATTCATCCGCACCCCCGATCCGCGTGCTCAG
CGCCGGGCTCGGCAATATGGGCCGCAGCCACGCGCTCGCCTATCATCAGAACCCCGGTTTCGAAATCGCCGGG
CTGGTCAACCGCTCGAAGGTGGCGCTGCCCGAGGCTCTTGCAGGATACGAGATTCTGCCGTCCTTTCCGGAGG
CGCTCGCCGAACTGGAGCCGGAGCTCTGCTCGATCAACACCTATTCGGACACGCATGCGGACTACGCCGTGAT
GGCGATGGAGGCGGGAGCCCATGTCTTCGTGGAGAAGCCGCTGGCAACGACGGTCGCCGATGCCGAGCGCGTC
GTCGCCTGTGCGCGGGCCAATGGCCGCAAGCTGGTCGTCGGCTATATCCTGCGCCACCACCCGTCCTGGATGC
GGCTGATCGCGGAAGCCCGCAAGCTCGGAGGGCCTTACGTCTTCCGCATGAACCTGAACCAGCAATCGAGCGG
CCCGACCTGGGCGACGCACAAGTCGCTGATGCAGACGACACCGCCGATCGTCGATTGCGGCGTCCACTATGTC
GACGTCATGTGCCAGATCACCGATGCCAACCCCGTCGAGGTGCGCGGCATGGGCTTGAGGCTCTCGAACGAGA
TAGCCGCGGATATGTACAATTACGGGCATCTGCAGGTGATCTTCGAGGACGGCTCGGTGGGCTGGTACGAGGC
GGGCTGGGGGCCGATGATCTCCGAGACGGCCTTCTTCGTGAAGGACGTGATGTCTCCGAACGGCTCCGTGTCG
ATCGTCATGGACCAGAACGCCAAGTCCGACGACATCGACGTGCACACGAAAACCTCCGTCATCCGCGTGCACA
GCGCCGCTACGGGACCGGACGGCCGGTTCCTGAAATCCGACGAGGACCTGGTGATGGAGGGCGAGCCCGGCCA
CCAGGAGCTTTGCGACCGCGAGCAGGCCTTCGTGCTCAAGGCCATCCGAGAAGACATCGATCTTGGCCGGCAC
ATGGACGATGCGGTGCAGTCGCTGAAGATCTGTCTCGCCGCCGACGACAGTGTGCGCACCGGCAAGCCGGTCA
AGCTTTAAATGACGAAATTTCGGAATTTCGTTGTCAGCTGCGCACTCGCCGCCGCCTCCGCGGTTCCCCCTGT
AACGTCCGTTCAGGCCATGCCGCGCCCGGCGCTCGACATTGCGATCAAAGCGCCGGCCGGCGTCGAGAATGCC
ACGCATCGCGGCCGTTACGGGTGGCGCGGCGGCTATTCTCGCCGCGGCTATTATGGAGGATCCGGCTTCTATC
CGGGCTGGAGCCCCTATTACAGGCGGTCCGGGATCAGCTTCTATTTCGGACCCAGCTACCGTTACCGCCCGTA
TTACGGGAACCGCTATTATTACGGACCGCGCTTTTATGGCCCCGGCTATTACAGCTATCCCGGGCCCGCATAT
TACGGTTATCGCTCCTATAGGCCACGCTACTATGGCGGCGGCTACGTCCGGAGCGGCGGCAACAGCCATGTCA
GCTGGTGCCTGTCGCGCTATCGGTCCTATAACCCTGCCTCCAACCGCTACCTGACCTATGGCGGGGTCTACCG
GGTCTGCTACTCGCCCTATCGTTAGATGACTTCGTTCTTTGATTCCGAATCTCCCTCCAACGTCGCCGAATAT
TCGGTGTCGGAATTGTCCGGCTCGATCAAGCGCACGGTCGAGCAGGCCTTCGAGCATGTCCGGGTGCGGGGCG
AGATTTCCGGCTACCGCGGCCCGCATTCCTCGGGACACGCTTATTTCGCCCTGAAGGACGATCGCGCCCGCAT
CGATGCGGTCGTCTGGAAGACCACCTTCGCGCGGCTGAAGTTCCGGCCGGAGGAGGGGATGGAGGTCATCGCG
ACCGGCAGGGTCACGACCTTCCCCGGCTCGTCCAAATATCAGATCGTCATCGACTCGCTTGAGCCGGCCGGTG
CCGGGGCGCTGATGGCGCTTATCGAGGAACGCAAGCGCAAGCTTGCGGCGGAGGGCCTTTTCGATGCGGGCCG
CAAGCGGCCGCTGCCTTTCATGCCGAGGGTCGTCGGCGTCGTGACCTCGCCGACCGGAGCTGTGATCCGCGAC
ATCCTGCACCGCATTGCCGACCGTTTCCCGGTCCATGTCATCGTCTGGCCGGTGCGCGTGCAGGGCGACGGCG
CCTGCGAGGAGATCGTGGCGGCGATCGAGGGTTTCAACGCGCTGCAGCCGGGCGGCTCCATTCCCCGGCCCGA

CGTGCTGATCGTCGCGCGCGGCGGCGGCAGCCTCGAGGATCTCTGGTGCTTCAACGACGAGGCGATGGTGCGC
GCCGCGGCGGCTTCCGCCATCCCTCTGATCTCGGCCGTCGGTCACGAGACGGACTGGACGCTGATCGACCATG
CGGCCGACCAGCGGGCGCCGACGCCGACCGGTGCGGCCGAAATGGCGGTGCCGGTGAAGGCCGATCTCGAGGC
CCAGCTCGCCAGTCTCGCAGCCCGGCTCAAGGGCGCCGCGGCGCGGCAGATGGACAATCGCCGCCAGACGCTG
CGCTCGCTTGCGCGGGCGCTACCCTCGCTCGACCAATTGCTCGCCTTGCCTCGGCGTCGCTTCGACGAGGCGG
CCGCCGGACTCGGCCGCGGCCTGCAGATGAACACCGCCAACAAGCGGCGAAGCTTCGAACGCAATGCGGCCCA
TCTCCGGCCGGAGCTCCTGACGGCAAGGATCGTCGATCGCCGTCAGCGCGTCTTGGATGCCGTCAACCGGGCC
GAGCGCATCGTCGAGCGTAAGGTTCAACGTGGCGCTCAGCGCATTTCGTCTGCCGATGCCTCGCTCCGCGTTC
TGCCGTCGCGGCTGATCGGCCAGATTCATCGCGCATCGGATCGCGTTTCCAGCCTGGGCAGGCGTGGGGATGC
GGCGATCGCCGCCGATCTGCGCAGGATGAAAAGCGCGCTTGCAGCACAGGACCGCGTGCTGCAGTCGCTCTCC
TATCACAGCGTCCTGCAGCGCGGCTTCGCGCTGGTGCGCGATGCCGCGGGCGAGCCGGTTAAACAGGCGGCTG
CCGTGCATCCCGGGATGGCGCTTTCGCTGGAATTCGCCGATGGCCGCATCGCGGCGGTCGCCGGCGAGGACGG
CACTGCGCCGCAGTCGCCGAAGAAGCGGCCTGCACGCTCCGGCGAGCCGACGAAGCAGGGCAGCCTCTTCTGA
ATGAATTTCTCACGCTTCTTCGTCGACCGCCCGGTCTTCGCGGGCGTTCTTTCCGTGATCATCTTCGTCGCGG
GCCTGATCGGCATGACCGGCCTGCCGATTTCCGAATATCCGGAAGTCGTGCCGCCGCAGATCGTCGTCAGGGC
GCAATATCCGGGCGCCAACCCGGCCGTCATCGCCGAAACCGTCGCCACACCGCTCGAAGAGCAGATCAACGGC
GTCGAGGGCATGCTCTACATGCAGAGCCAGGCGACCGCCGATGGCCTGATGACGCTGACCGTCACCTTCGAGC
TCGGAACCGATCCGGATCAGGCCCAGCAGCTGGTGCAGAACCGCGTCTCGCAGGGCGAGCCGCGGCTGCCGGA
AGAGGTGCGCCGGCTCGGCGTCACCACCGTCAAGAGCTCGCCCGACCTGACGCTCGTCGTGCACCTGATTTCA
CCCAACGGGCAATACGACATCAACTATCTGCGCAACTACGGCGTCCTGAACGTCAAGGACCGGCTGGCGCGCG
TAGATGGTGTCGGCCAGGTGCAGATCTTCGGCGGCGGCGACTATTCCATGCGCGTCTGGATCGACCCCGAAAA
GGCCGCAGAGCGCGGGCTTGCCGCGAGCGACATCGCCAATGCCGTCCGCGGGCAGAACGTCCAGGCAGCCGCC
GGCGTCATCGGCGCTTCGCCGTCGGTTCCCGGCCTGGACCTGCAACTCTCCGTCAACGCTCAGGGCCGCCTCA
AGACACCCGAGGATTTTGCCGACATCGTCGTCAAATCGGGCGAGAGCGGGGAGATCACGCGCCTGGGCGACGT
CGCCCGCGTCGAAATGGGAGCCGCTGACTACTCGCTGCGCTCGCTTCTCGACAACAAGGCCGCCGTCGGCATG
GGCGTGTTCCAGGCGCCCGGCTCGAACGCCATCCAGATCTCCGAAAACGTCCACAAGGTCATGGCCGAGCTGA
AGCAGACCATGCCTGATGGCGTCGATTACGAGATCGTCTACGACACGACGCAGTTCGTCCGGGCGTCGATCGA
ATCGGTCATCCACACGCTGCTCGAAGCCATCGCGCTCGTGGTCGTCGTCGTCATCGTCTTCCTGCAGACCTGG
CGCGCCTCGATCATCCCGCTCGTCGCCGTGCCCGTCTCGATCGTCGGTACCTTCGCCGTGATGTATGTCTTCG
GCTTCTCGATCAACGCGCTCAGCCTCTTCGGTCTGGTGCTGGCGATCGGCATCGTCGTCGACGACGCCATCGT
CGTCGTGGAGAACGTCGAACGCAATATCTCCCAGGGCCTCTCACCGGTGCAGGCGACCTATCGCGCCATGCAG
GAGGTCTCTGGGCCGATCATCGCGATCGCGCTGGTCCTCGTCGCGGTCTTCGTGCCGCTCGCCTTCATCACCG
GGCTCACCGGCCAGTTCTACCGCCAGTTCGCGCTGACCATCGCGATTTCGACGGTGATTTCGGCCCTCAACTC
GCTGACGCTCTCGCCGGCGCTCGCCGCCCTTCTCCTGAAGGACCACCACGCCCCGAAGGACCGGCTGACGCGG
GTCATGGATGGGTTGTTCGGCTGGTTCTTCCGCGGCTTCAACCGCTTCTTCGGAGCGAGTTCGGAGGCCTATG
GCCGCGGCGTCGGCGGCATCCTCACCCGCAAGTCGCTGGTCATGGGCGTCTATGTCGTCCTCCTCGGCGTCAC
CTTCGTGCTCTTCCGCGCGGTGCCCGGCGGCTTCGTGCCGGCGCAGGACAAGCAATACCTCATCGGCTTTGCG
CAGCTGCCGGACGCGGCGACGCTCGATCGCTCGGAAGATGTCATCCGGCGCATGAGCGAGATCGCCCTCAAGC
ACCCGGGCGTCGAACACGCCATCGCCTTCCCGGGCCTGTCGATCAACGGCTTCACCAATTCGTCCAACTCCGG
CATCGTCTTCGTGTCGCTGAAGCCGTTCGAGGAGCGTACGACGCCCGAACTTTCGGGCGGCGCCATCGCCATG
CAGTTGAACCAGGAGTTCGGGGCGATACAGGATGCCTTCATCGCCATGTTCCCGCCACCGCCCGTCCAGGGTC
TCGGCACGACCGGCGGCTTCAAGCTGCAGATCGAGGACAGGAACGGCCTCGGCTACCGGACGCTGGACGATGC
GACCAAGGCCGTCCTCGCCAAGGCGATGGCGGCACCGGAACTCGCCGGGCTCTATTCGAGCTTCCAGATCAAC
GTGCCGCAGCTCTATGCCGACCTCGACCGCACCAAGGCCCGTCAGCTCGGCGTCGCGGTCACGGACGTGTTCG
AGACGCTGCAGATCTATCTTGGCTCGCTCTACGTCAACGACTTCAACGCTTTCGGCCGCACCTACAGCGTGCG
CATCCAGGCCGATGCGAGCTATCGCAGCCACGCCGACGACATCGGCAAGCTGAAGGTGCGTTCGCAGACGGGC
GAGATGATCCCGCTGTCGGCCCTGCTCAACGTCGAGCAGACGGTCGGCGCCGAGCGGGCCATCCGCTACAACG
GCTTCCTCGCCGCCGATATCAATGGCGGACCGGCACCCGGCTTCTCTTCCGGTCAGGCGCAGGCGGCCATCGA
GCGGATTGCGGCCGAGACTCTGCCGCCCGGCATTTCATACGAGTGGACGGACCTGACCTACCAGCAAATCCTC
GCCGGCAATTCTGGCATCCTCGTCTTCCCGCTGGCGCTGCTGCTCGTCTATCTCGTGCTTGCGGCCCAGTATG
AGAGCCTGCTGCTGCCGATCGCGATCATCCTGATCGTGCCGATGGGCATCATGGCGGCGCTGACGGGCGTCTG
GCTGACCGGCGGCGACAACAACGTCTTCACCCAGATCGGCCTAATCGTTCTGGTGGGTCTGTCGGCAAAGAAC
GCTATCCTGATCGTCGAATTCGCCCGCGAACTGGAACTGTCCGGCAGCAATGCCGTCAGTGCGGCGATCGAGG
CGAGCCGGCTCCGGCTCAGGCCGATCCTGATGACCTCGATGGCCTTCATCATGGGTGTCGTGCCGCTCGTCAC
CTCCACCGGCGCCGGCGCGGAAATGCGCTCCGCCATGGGCGTGGCGGTCTTTGCCGGCATGATCGGCGTCACG
GCCTTCGGCATCTTCATGACGCCCGTCTTCTATGTGCTCATCCGCAAGCTTTCCGGCGAAAAGCCGCTGAAGC
ACACCGGAGCGCATATAGAAGCCCCGCATCTCGCCCCCGGCGAGTAGATGTCACTGACAGATGGCCCCTATGC
GGGGCCGCCCCAGCCGGATGTGGTCGACCCGAGCGGCGGGGCCAATCAAACGCGCGTGCGCGCCTATAACGAA

CGGCTGGTCATGTCGCTGGTGCGCCGTCACGGCAGCTTGTCCAAGGCCGAGATCGCGCGCCGATCCGGTCTAT
CGGCGCAGACCGCCTCCGTCATCATGCGGTCGCTCGAAGCCGACGGGCTGCTCGTCCGCGGCGCGCCGGTGCG
CGGCCGGGTTGGACAGCCCTCCATCCCCATGCGGCTCAATCCGGATGCGGTCTATTCCTTCGGCGTGAAGATC
GGCCGGCGCAGCGCCGATCTGGTGCTGATGGATTTTCTCGGCACCATCCGGCTGCACCTGCATCAGATCCATA
CCTATCCGCTGCCCGAGGAGATCGTCAGCTTCATCGTCGGGGGCATCGGCAAGCTCGAGGGACAGCTTTGCCC
CGGCGAGCGCGGCCGCATTGCCGGCGTCGGCATCGCCACGCCGTTCGAACTGTGGAACTGGTCGGAGGAAGTC
GGCGCGCCGCGCGAAGAGATGGACAGGTGGCGCGATTTCGACCTGCAGGCGGCGGTCGCCTCGCGCATCCGGC
ATCCGGTTTTCCTTCAGAACGACGGAACCAGCGCCTGCGGTGCCGAACTCGCCTTTGGTGTGGGCGCCAGTTA
TCCCGATTTCGTCTATTTCTATATCGGCTCCTTTATCGGCGGCGGCGTCGTGATCAACTCCGCCCTCTTTTCC
GGACGAACCGGCACCGCCGGCGCCGTCGGCCCGCTGCCTGTCGCCGGCAAGGACGGCAAGTCGACGCAGTTGC
TGAAAATCGCCTCGGTCTTCGTCCTGGAAAAGCTCCTGCGCGAACGCGGCATCGACCCGGAGCCGCTCTGGTA
CTCCGCCGACGACTGGATCGATTTCGGCGAACCGCTCGAAATATGGATTCAGGATGCGGCGGCAGCGCTTGCG
CAAGCCGTCGTCTCCGCGGTTTCCGTCGTCGATTTTTCGGCGGCCGTGATCGACGGCGGCTTTCCGCCCTGGG
TGCGTGCGCGCCTTCTTGCCGCGACGCGAAAGGCCCTCCAGACGCTCGACCTGCAGGGCGTCACGGTTCCGGA
CCTCGTCGAAGGCACCGTCGGCAGCCACGCCCGCGCGATCGGCGGCGCAAGCCTGCCGCTCTTCTCCCGCTAT
CTGCTGGACACCAATGTCCTCTTCAAGGAGCTTTAATTGAGGAACGAGGATATCGTGTTGGATGGCGCGAAGG
TGTTCTGGCGCACCCTGCTGCCGAGGTTGAAGGCGGAGCGCGGGCTTCTCGGCCTGTCCGTCGTGCTCGCCTT
CCTCGCCGCACTCGCGGGCATCCATCCGCTGGCCGTCCTGCCTTTCTGGATCGTGCTCGTCGCGGCCATCCTC
AACCGGGGAGCGCCGCCCGTCCAGACAGCGCCGGCGAGCGTCGGCGCTGCTGTGGAAACCAAGGCCGACCCGC
TTGCTCCCCTGCTCGACGCGCTCGACATGCCGGTGCTCGTCGTGGCGGCCGACGAGACCGTCGTCTTCCAGAA
CGCCGCCGCAGAGAAGGCCTTCGGAACTATCCCCCAGGACAGCTACCTTTCCGCCCGCGTCCGTTCGCCCGGC
ATTCTCGACATGGTGCGCGAGACGATCGCGACAGGCAAGGTCAACCAGATCGAGCATGCGGAAAGGCTGCCTT
CGGAGGCGGTCTACGTGGTTCGCGTCGCGCCCGTGGACATCGGCTCCGACGTTTCCGGCCGGCGCATCTTTCT
GCTGACCTATCGCGATATCTCGCAGGCTCGCCGCATCGACCGGATGCGCTCCGATTTCGTCGCCAATGCCAGC
CATGAGCTGCGCACGCCGCTCGCATCGCTGCGCGGGTTCATCGAGACGCTGCAAGGCCCTGCCCGCGACGACC
GGAAGGCCCAGGAGAAATTCTTCGGCATCATGCACGAGCAGGTCACGCGCATGAGCCGGCTCGTGGACGACCT
CCTGTCGCTGTCGCGCCTCGAGTTGAAGTCGCACATCGCGCCGGACGATGCCGTCGATCTCGGGCCGCTGCTC
GGACACGTGCGCGACAGCCTGGCGCCGCTTGCGGGCGAACTCGACGTGACGATCGCGCTGGAGGTTCCGGAGA
CTCCGGTTTTCGTCCAGGGGGATCGGGACGAATTGGTGGAGGTCTTCGAAAACCTGATCGAAAACGCCTGCAA
ATACGGGCAGGAAGGCAAGCAGGTGGATGTGCGGCTGACCGGCGGGAATGGTCACCAGGCGGAGGTGACGGTT
ACCGACCACGGTCCGGGAATACCTGCCGAGCATGTCCCGCGCATCACCGAGCGTTTCTACCGGGTCAATGTCG
AGGCGAGCCGGTCGAAGAAAGGCACCGGCCTGGGCCTTGCGATCGTCAAGCATATCCTCACGCGTCACCGCGC
CCGCCTGCTCATCCATTCGGAGGTGGGGAAGGGCACGGTCTTCAAGGTCCGCTTCTGAATGGAGCAGAACCTA
TCCACGATACGCAAAGCCGCGGGTAGCGAGGGAGAGCCGACCATCGCGCTTGCGCTCGGCGGAGGCGGTGCGC
GGGGACTTGCCCACATCCATGTCATCGAGGCGCTCGATGAGATGGGGCTCCGCCCCGCAGTCGTGGCCGGTTC
GTCGATCGGCGCCCTCATGGGCGCGGGCGTGGCCGCTGGCATGAGCGGGAAGGAAATCCGCGAACACGTGCTT
TCGACCGTCGGCCGGCGTGGGGAGGTGCTCAACCGCCTATGGCGACTGAGGCCGACCAGTCTTGCCGAGGCTG
TGGCGAGCGGTTTCCGCGTCGGCCAGTTCAATATAGAGCGCGTGCTGAAGGCCTTCCTGCCCGAGGCGGTGCC
TCTCCGCTTCTCCGATCTGGCACTCCCCCTTAAAATCATCGTCACGGACTATTACGGGCAGACGGAGCGGGTA
TGCGAAAGCGGCGACCTCTACAAGGCGCTCGCTGCCTCCTGCGCCCTGCCGGCGGTCTTCATGCCGGTGAAGA
TCGACGGCCGCGTGATGATCGACGGCGGCATCTATAATCCGATCCCCTTCGATCACCTGCGCGGACTGGCAGA
TATCGTCGTCGCCGTCGATGTCGTCGGCGGGCCGGACGGTGACGGCGAGACGATCCCGAGCCGTATCGACAGC
CTGTTCGGCGCGACCCAGTTGATGATGCAGTCGATCATCACCATGAAGATGCAGGCCGGCGCACCGGATATTC
TCCTGCGTCCCGATGTCGGACGGTTCCGCGTGCTCGATTTCCTGCGCGCCCAGGAACTGCTTGCCGCCACCAG
CGGGACCAAGGACGAGGTGAAGCGCGCGCTGGCAGAGCGCATCGATCGGTGGAGGAAAGGGATATAAATGGTT
TCAGAAGCGCCCCCGTTCTGGTGGACCAAGGCCGACTGGCGCGCCTACGCTCTTTGGCCCTTTTCCTGGGTGT
ACGGCCGGATCGCCGGGATGCGCATGGACCGGGCGCGGCGGGCGACCTCCGCCGTACCGCTGATCTGCATCGG
CAACTTCACCGTCGGCGGAGCCGGAAAGACGCCGACGGCGATCGCGATTGCGCGGGCCGCCAGGGCACGCGGC
CTGAAGCCCGCTTTCCTGAGCCGCGGTTATGGCGGGTCGCTGGACGTCACGACCGTGGTCGACCCCGAGCACC
ATCGCGCGCGCGATGTCGGCGACGAGCCGCTGCTGCTTGCCCGCGAGGCGCTGACCGTGATCTGCCGCCGCAG
AGTGGATGGCGCACGCAAGCTTGCCGCGGAAGGCGCTGACATCATCATCATGGATGACGGATTCCAGAGTGCC
CGGCTCGTCTTCGATTTCGCCCTTCTCGTGGTCGATTCCGGCCGCGGTATCGGCAACGGCCATCTCGTGCCTT
CCGGCCCCGTTCGGGCGCCGATCGGCAATCAGCTGCGCCATGCCAACGCTCTGCTGAAACTCGGCCACGGTTC
CGCCGCCGATCCTCTCGTCCGCCGGGCGGCTCGGGCCGGCAAGCCCGTCTATGTGGCCGAAACGGTGCGCACG
GATGCAGGGTCGCTCGACGGCGTCAAGGTCCTTGCCTGGGCCGGAATTGCCGATCCGGAGAAGTTCTTCAAGA
CCGTTCGCGAGACCGGCGCGGTCATCGAAGAGACCCGGAGCTTCCCGGATCACCATCATTTCTCCGAGGACGA
GATTGCCGACCTAATCGACCGCGCCGCAAGCCGGGGCTACACGCTGGTGACCACGGCCAAGGACATGGTCCGT
CTGGAGCCCGGCCACGGGCGGGCAGGGGAACTGGCGGCAAAGAGCCGCGTGATCGAGATCGAAGTGCGCTTCG

ACGATCCGGCAGCGCCGGGAAAGATCATCGACGCGGCACTTGCCTCCGCCCGTGCCCGCCGATTGCGCGAGCG
TAAGGCGGGCTAGTTGGCAAGAAAAACGTCCCGCAGCATCCGATATGCCGACCTGAACGCGGACGACAATGCG
GAGACGCGTCGGCTGATCGATGCCGGCAGCCGCCTTGCCGAGGAGGTCGCGCGTCTCTTTCCGCCCGAGCCCG
ACATGGCCGACCGCCACTACTGGCTCGAAGCGATGATCAACCACGTTCCCGATTATATCTATGCGAAGGATAC
GGCGGGGCGCTTCCTGATCGCCAACGAGGTGACGGTTGCCGACAACGGCCTCGAAAGCCTGCGCGACCTCGTC
GGCAAGACCGATTTCGATCTGCACCCGCACGAGTTCGCCCAGGCCGTCGCCGATACCGAGCGACGCGTTTTGG
AAACCGGCGAACCGATCTTCGGCATCGAGGAACGCGCCATCGTGACCAAGGACCGCGATCGCTGGCTGATGAC
CTCGAAAGTGCCCTTGCGCAGCAAGAGCGGGAGGATCATCGGTATCGTCGGCATCTCGCGCGATATCAGCGAT
CGCAAGGCCGCGGAGCGCCTGCTCGAAGGTCAGGCCCGGCTGCTGGAAATGATCGCCAACGGCAAGCCCCTTG
GCGAGTTCTTCCACGAGCTGGTGCTGCTGATCGAAGCGCTGCTGCCCGGGATCAAGGGCTCCATCCTGCTCCT
TTCGGAGGATGGCCGGCATCTCCTGCACGGCGCTGCCCCCAGCCTCGACGCGGGCTTTTGCGCTGCCATCCAT
GGCCTCGAGATCGGCCCTGCGGCCGGCTCCTGCGGAACGGCCGCCTGGTGCGGCGAACAGGTGATCGTTTCCG
ACGTCTTCGCCGACCCGCTCTGGGAGGAATACGCGCCTCTCCTGAGGCCTTCCGGCCTCCGTTCCTGCTGGTC
CACGCCCATCCTGTCGCGCGAGCGAAAGGTGCTCGGGACATTCGCGCTCTATGCGCAGGAGGTCGGAACGCCG
AGCGAGCAGCAGCAGGAACTGATTGCCATGGCCGCTCATCTCGCCGGAATAGCAATCGAGCGCAAGCGGGCGG
AGGACCGCATCGGTTTCATGGCCCATCACGATGCCTTGACGGGGCTGCCGAACCGCGTGCTTTTCGAGGAGCA
GGTCGCCGGCATGCTCGAGGAGATCCGCGAGCGGGATCAATGGGCCGTGCTCGCCTTTCTCGACCTCGACGAT
TTCAAACTGATCAATGACAGCCTTGGGCACGCCACCGGTGACGAGCTCCTGAAGATCGTAGCCGGGCGCATGC
GGGCCGCCGTCCGGAGATCCGATTCCGTCGTGCGCGTCGGCGGCGACGAGTTCATCATCCTCCTGAACGGCCT
GCCGAAGGAGCGGGACGTCGTGCTATCGCGGCTGGAGGATATCCGCACTGCAATCGCCATGCCGGTGCAGTTG
CAGGGCCGCTCTCTGCAGGTGAGCTGCAGCATGGGCGTGGCCTGCTTCCCCAACCAGGGCAAGACGGCGGGCG
AACTGCTCGCCAATGCCGACATGGCCATGTACCGCGCCAAGGAGCTCGGCCGCAACAACATCCAGGTCTTCAC
CGAGGAGATGGCCGCCAGAGCCCATGAAAAACTCCAGTCGCAAGAGGAGCTTCGCGAGGCGCTTGCGCGGGAG
GAGTTCCTGCTGCATTTCCAGCCGCAGATGAGCCTCGAGACCGGCCGCATCTTCGCTGCGGAGGCGCTGTTGC
GCTGGCAGCACCCGGTGCGCGGCATCATCTCGCCTGCGGCATTCATCCCGCTCGCCGAGGAAACCGGTCTGAT
CGTGCCGATCGGAGATTGGGTCCTGCGCACCGCCTGCCGCCAGTTGAAGGCCTGGCAGGACGCCGGCCTGCCG
CCGCTGATCGTCAGCGTCAATGTTTCCGCGCGGCAATTCCGCGAGCGCAACTGGGCAGCGCGTGTGGCGGCGG
TTTTGGAGGAAACCTGCCTCGAGGCCCGTTACCTCGAACTCGAGCTCACCGAAAGTCTGATCATGCAGGACGT
GCCGGGAGCGATCGCCACAATGCATGCGCTGGAGGCGATCGGCGTGCACCTTGCCATCGACGATTTCGGGACA
GGCTATTCGAGCCTCAGCGCGCTCAAGCGCTTCCCGGTGCGCCGGTTGAAGATCGATCGGTCCTTCGTGACGG
ACATTCCGCACGATGCCGACGACATGGCGATTACTTCCGCGATCATCTCGCTTGCGCAGAAGCTCGGTCTGCG
CGTCATTGCCGAGGGTGTCGAGACGGAGGCCCAGGTCGAGTTTCTGCAAAAGAGCGGCTGCGGCGAGATCCAG
GGCTACTTCTTCAGCCAGCCCTTGCCGGGCGAGGATTTCGAAGTGCTGCTTTCCTCTGAGCGGCGGTGAATGG
ATGCCGCGGCCCTGACATTCCTGGCCCTCGGTCTTCCCTTTGCCGCGGCCGCCGTCGCACCGGCCCTGACCCG
GCTTCTCGGCGGGAATGCCGCCTGGCTGCTGGCGCTTGCGCCACTCGCCATCTTCATGCATCTCGCAGGTTTC
ATTCCGGAAGTCGCCGCTGGCGAGATCGTTACCGGCGGCTATATCTGGATACCGTCCTTCAACGTCAGCTTCT
CCTGGCTGATCGACGGCCTGTCACTGACCTTCGCGCTTCTGATTTCGGGCATCGGGACACTGATCGTCCTCTA
TTCGGGCGGATATCTGAAGGGGCATCCCGACCAGGGCCGCTTCTTTTCGTTCATCCTGATGTTCATGGGCTCG
ATGCAGGGGCTCGTCGTCTCGGACAGCTTCCTGATGCTCTTCGTCTTCTGGGAGCTGACGTCGATCACTTCGT
TCCTGCTGATCGGCTTCGATCACACCCGCGAGGCGGCGCGCCGCGCGGCTTTGCAGGCCCTGGTCGTGACGGG
GGGAGGCGGGCTTGCCTTGCTCGCCGGGCTCCTCATCCTGTGGAACGTCAGCGGCGTCACGCAATTTTCGCTG
CTCCTGTCCTTCGGCGACGAACTGAAGCAAAGCCCTTTCTATCTCGCGGCGCTGCTCCTCGTCCTCGGCGGCG
CATTCACCAAGTCGGCACAGTTTCCGTTTCACTTCTGGCTGCCGAACGCCATGGAAGCGCCGACGCCCGTTTC
GGCCTATCTGCACTCGGCGACGATGGTGAAGGCGGGGGTCTATCTTCTGATGCGGCTCAACCCGGTGCTCGGC
GGTACGACGGAGTGGCAGGTTCTGCTGCCGCTCTTCGGCGTGACGACGCTCGTCGCCGGAGCCGCCCTGGCAG
TGCGCCAGACGGACCTGAAGATGATGCTGGCCTATACGACCATGTCGTCGCTCGGTCTTCTGGTCATGCTGAC
GGGCCTCGGCCTGCCCTACGCCATCGAAGCCGCAGTGCTTTACTTGGTGGCGCATGCCCTGTTCAAGGGGGCG
CTGTTCATGGTGGCCGGCATCATCGATCATGAAACCGGTACGCGCGATCTGACGAAGCTCGGCGGCCTGCGCA
GGGCGATGCCTCTGACCTTCGCCATAGCTCTTGCGGCGGCCCTGTCGATGGGCGGCCTGCCGCCCTTTTTCGG
GTTCCTTGCAAAAGAGGAAATCTACGCCGCACTCTCCGGTTTCGACCGCCGGTCCATGGTCTTCGCGGCATTG
ACCATCCTCGGCAACGGCCTGATGTTCGCCGTCGGCTTCGCGATCGCTCTAAAACCGTTTCTGGGCCCGAAGG
TCGATACGCCGAGGCATGCCCACGAGGCACCGGTGCTCCTCTGGATCGGCCCGGCAATCCTGGCTGCGAAGGG
CCTTTCCATCGCGCTGATGTCGGGCCTTGTTCATCGGTTCGTGACCTCGCCCATGGCCTCGGCCGTTGCCGGC
GAACCGAGAACCGTGACGATTTCTCTCGCACCGCATATCGGCGTGCCGCTGTTGCTCTCCGTCGTGACGCTGG
CGGTCGGCGTCGTTCTGTTCCTCAATCTGACGCGGCTGCGCGCAGCCATGGCGGTGATCCTTGCCGATATCGG
CTGGGGACCGGATCGAGGCTTCGACCAGTTCATACGCGGCCTCGTGCGGTTTGCGGTCGCCGCGACCCGCCGA
TTGCAGGGCGGCCGTCTCGATAACTACATGACCGTGACCTTCCTGCTCGTCGCCGCCGTCATGCTCGCATTGC
CGCTGCGCTACGGCGAATTCCCGCGCGCACCTTTCTTTCCGGGCGACGTTCCGCTGCACGAATTCGCGATCAT

GGCGATCGCCGTGCTTGGCCTCTTCGCCGTGGTGCTGGCCGCGGACCGGCTGACGGCGATCGTCTCGCTCGGA
ATTCAGGGCTTCGCCGTCGCGATGCTCTTCCTGCTTTACGGCGCTCCGGATCTCGCCTTCACCCAGTTCATGA
TAGAGACGCTGTCCGTCGTCGTGCTGGCACTGGTGATGACCCGGCTGCGGCTGTCTCCTTCGGATCACCGACC
ACTCAGGCAGAAGCTTCCCGACTTCACGATCGCGCTCGCCTGCGGAATCGGATTCGGCCTTTTCCTTCTCCGG
GTTACCGGCGTGCCGTTCGATGCGACGCTGACGGACTTCTTCAACCTCTATTCGAAATCGATCGCGCACGGCG
CGAACGTGGTCAACGTCATCATCGTCGACTTCCGAGGCACGGATACGCTTGGCGAAATTGCCGTCGTGCTGGT
TGCGGGCCTGTCCATCCTTTCCCTCGTGCGGTTGCGGGCGGGATCGTTGAGACGCGGCGAAACGCGCGAGATC
GCGGCCGCTGATGCGAACACGAAGGAGCCGGTATGAGTGGCCCGAGGTCAAGGCAAGGTTATTCCAGTCAGCA
GGGTTCAGCTCGTTCAGCCACAGACCAACGGGAGGATCATACGCATCCTGGTCACAGAGGGGCAGCCGGTGAA
GGCTGGTGACTTGTTAGTCGGCATGGATACCGTCTCGGTTGAGAGCCAGATCAAGCTCAGGCTGACCGAGGTT
CGTCGCCAACGTCGCGGGGCGACGGTTGCCCGAGTAATCATTGCGGCGCTGACAAATGTTGCAGTCGCAAACG
ATGATGTGCTTGCGGCAGGCAGAGCTGCCTTCGAAAGGGAGCGGCATGACCTGGAGGTTATCGACGGAGCGGA
GGCACTTATAGAGGCTGTGCTAACGTCTCTTCGAGATCAGGTTGCGCGGATCAATGCGCAACTTGATCGTGTC
GAACAATCGCGAATAACCCAGCAGGCGCGACTGCAGAAAGCTCGGTCTGATCTGGATATTTCCTCCAGGAAAT
TTTTGTCAGCCGGCAAGTTGAAGGACAAGGGAGCCATCAGCGAAGTAGACTACTTCGAGCGATTGCGTGAGCA
AAGAGCACGCGAACTGGAGGTGCTCATCTCCGAGAGCGAATTGAATATCCTTGCAGCGGAAGCGAACGCGTTG
GCTAGACAGCGGCAGAGCATCATTTCGACCGCAGTCGAGAACTATCAGAAACAACTGAACGAGGCGAGCACGT
CCCTTGCAGGGCTGGAAGCGGAACTGAGCGCGGCTAGGAACCAACTGGCCTTGCTCGCGATTCGCGCACCAGC
AGATGGCAAGGTCGAGAACCTCACGGTATTTACCATCGGCGGTTTCGTGGAAGCAGGTTCAACACTCATGTCC
ATCGTACCTTCCGGCGATGGCATGGAGATTGAAGCTTTCTTCGACAACCGTGACATCGGCTTTCTTGAAAAAG
GGCAAGAGGCATTTGTGAAGTTTGACGCCTTTCCGGCCGAACGTTTCGGTATTGTTCGCGGTCGGGTGACTAG
AGTTGGTGCGGATGCGCGCGGCGACATCGTCCCAGGGAAATGGGTCTACGCAATCCACCTTGTACTCGACCAA
GAGACAATTGGGATAGGCGGGCGCGACATAGGGTTCTCGCCCGGCATGACTGCGACGATCGATGTGATAACGG
GTGAACGGCGGCTCATTAGTTACTTCTTCGAGCCAATAATCAAGGCAATTCAAGACGGATTAGGGGAACGTTG
AATGGCCTATTTTCTGCAGCAGATCGCCAATGCCGTACCCGTGGCCGCGCTTTATGCGGCGCTCGCCTTCGGA
TACGCGATCGCCTTTGCTGTCACACGGCGGGCGGACCTGACCTATGGCGCGCTCTTCGCCTTTGCCGGGCAAA
TCTTCGTGCTCTTCGCGGATGTCGGCTGGAACCGGCTGTGGCTGGTACTGCCGGCGGCGCTCGGGCTCGGGGC
GACAGCGGCGCTCGCATTCGGGCTAGGTGCCGGTCTTATTGCCGGCCGCTACGTCATGCGGCCCCTGGCTTTT
TCCTCCGCCAATGCCGTGGTCGTCGCCTCGCTCGGAAGCCTGCTGGTGCTGATGGAAACGGCCCGGCTCGCCT
CCGACACCAGAAGCCTCTGGCTGCCGCCCTTCCTGAACGACGTGGTGGTGTTCTGGCCGAACCCTGCCTTTCC
GGTCGCGCTGACGGTCGTGCAGCTCGTCAACACCGCGCTCATGATGGCCCTTGTCGCCGGCGGCCATTGGCTG
TTGACCCGTTCCTATGCAGGCCGCTACTGGCGCGCCGTTTCCGAGGATCGCAAGGCCGCCGCGCTTTGCGGCA
TCGATCCGGGAGCGGTCTATATCGCCGCCTATGGCGTGGCGTCGCTGATCGCTGCGTTCTGCGGGATTCTCGC
TGCTTCCTATTACGGCAACATGGATTTCGGCACCGGGCTGACCTTCGGGGTCAAGGTCCTGTTCATCGCCGCC
ATCGGCGGGCAGACTTCGCCGTTATTCGCGGCACTGGGCGCAGCCGGCATCGGCCTCATGGAGACGGTGTGGA
GCGCCTATGGACCCATCCTCTGGCGGGACTTCGCCATATTCGGCTTTCTGGTGATCGTGCTCGTGGTCACGCG
CCGGGAAAAGATCATTCCCTGAATGAACGACAGGACAAATCTTGTGGACAGGATCACCGGACACCGCCGCATG
CGCCGCAATCGCAAGGCGGATTGGACGCGCCGGCTGGTCCGGGAGAACCGCCTGACGGTGGACGACCTGATCT
GGCCGATCTTCGTCGTGCCCGGCAGCGGCATCGTCGAGCCGATCGATGCGATGCCGGGCGTCAACCGCATGAG
CGTCGACAAGGCGGTCGAGGCCGCGAAGGAGGCGGCCGGCCTCGGCATCCCGGCCATCGCCACCTTCCCCAAT
ATCGACATGGCGCTTCGCGACGAGACCGGATCCGACAGCCTCAACGCCGACAATCTGATCAATCAGGCGACGC
GGGCGATCAAGAAGGCCGTACCCGACATCGGGATCATCACCGATGTCGCGCTCGACCCGTTCACCAGCCACGG
GCACGACGGCATCCTGCGGGGCGGCGAGATCGTCAACGACGAGACGGTGGAGGCGATCGCCAGGGCGGCGGTC
ATTCAGGCCGACGCCGGCTCCGACATCATCGCGCCCTCCGACATGATGGACGGGCGGATCGGCGCGATCCGCG
AGGCGCTCGACGCGGCCGGCCACCAGAATGTCGGCATCATGTCCTATGCGACGAAATTCGCCTCCGCCTTCTA
TGGCCCTTATCGAGAGGCGATCGGCACCGGCGGCCTGCTCAAGGGCGACAAGAAGACCTATTACATCGATCCG
TCGAACGGTACGGAGGCGATCCGCGATGCGGCACTCGATGTGGAGGAAGGCGCGGACATGCTGATGGTGAAGC
CCGGCCTGCCCTATCTCGATATCTGCTGGCGGATGAAGGAAGCCTTCGGCCTGCCGGTCTTCGCGTATCAGGT
TTCCGGCGAATACGCGCAGATCAAGGCAGCCGCCGCCAATGGCTGGATCGATGGCGAGCGGGTGATGCTGGAG
ACGCTGCTCGCCTTCAAGCGGGCGGGTTGCGACGGGATCCTCAGCTACTTTGCCGTCGACGTGGCTCGAATTC
TCGCGAAACGCTGAATGTTTACAGGCATCATCACCGACATCGGTACGGTCGAGAAGGTCACACCTCTCGAGGA
AGGCATCAAGCTTCGCGTCGCCACGAATTACGACCCGCAGACGATCGATCTGGGTGCCTCGGTCGCGCATGCC
GGCATCTGCCTGACCGTCACGTCACTTCCGGAAGCCGGCAGCAACGAACGCTGGTTCGAGGTCGAGGCATGGG
AGGAAGCGCTGAGGCTGACGACGATCGCCGACTGGCGTGAGGGCACGCGCATCAATCTTGAGCGCTCGCTGAA
GATCGGCGACGAACTGGGCGGTCACATCGTCTCCGGCCATGTCGACGGCAAAGCGGAGATCCTTGCCGTCGAA
GCGGAGGGGGAAGCCGTGCGCTTCCGCCTCAGGGCACCCGAACATCTCGCGAAATTCATCGCGCCCAAGGGCT
CGGTGGCGCTCGACGGGACGTCGCTCACCGTGAACAAGGTGGATGGCGTCGAATTCGACGTATTGCTGATCCG
ACATTCGCTCGAAGTGACGACCTGGGGCGAACGCAAGTCCGGCGATCTCGTCAATTTCGAGGTGGACACGATG

GCGCGCTACGCCGCGCGGTTGGCGGAATTCCCGGCTCCCAAGCAGGAATAAATGACGCTCAAGATGAAGATGA
TTGCCGCAGCATCCCTGATCGCGCTTGCTGCCGGCGTCGCCCTGCCCGGGCCGGCGGCGGCGCTCGATGCCAA
GCAGAAGGAGGAATTCGGCGCCTTCATCAAGGAGTACCTGCTCGCCAATCCGGAGATCATGCTGGAAGTGCAG
GAAGCGCTCACCTCCAAGCAGCGGGCCAAGCAGCAGGAAACCGCCCAGGCGGCAATCGCCGACAACAAGAAGG
CGATCTTCAACTCTGACTACGACATAGCCCTCGGCAATCCGGATGGCGATGTGACGATCGTCGAGTTCTTCGA
TTACAACTGCGGCTATTGCAAACGGGCCATGTCCGACATGGACGACATCCTCGCCAAGGACAAGAATGTCCGA
TTCGTTCTCAAGGAGCTCCCAATTCTCGGACCGGATTCGCTCGCGGCCCACAAGGTCAGCGCCGCCTTCCGCG
TGATCGCACCGGAGAAATACGGCGATTTCCATCGCGCCCTGCTCGGGGCAGAGGAACGGGCGACGGAGGAAAC
CGCGATCGCCGTTGCGGCCAAGCTCGGGGTGACGGAAGCGCAGTTGCGCGAAAAGATGGATGACGGGCCGTAT
GATGCCTCGGTGCGCGAAGCCTATACGCTCGCCAACGAGCTCGGCATCACGGGAACGCCTTCCTATGTCGTCG
GCAACGAGGCGGTCTTCGGCGCTGTCGGGGCGCCTGAGATCGAACAGAAAGTCGCCAACATGCGTGCGTGCGG
CAAGACGGTCTGCTGAATGTCCGAACACAGCGCGACGACGACACCTGAAGCGCAAATGCGCACGGAGGCGACC
GGAACCAACCGGATGGTTCTCCTGGATGCGCTGCGCGGCCTGGCACTCGTCGCCATGGCCCTCTATCACTTTG
TCTGGGACCTCGAATTCTTCGGCTATGTCGCGGCCGGCACCGCCGGGACCGGCGGCTGGCGGATGTTTGCACG
CTTGATTGCCAGCAGCTTCCTGTTTCTTGCCGGTTACAGCCTCGTGCTCGGCCAGCTCCCGAAACTCCGTCCG
CGCGCCTTCGCCATACGTTTCGCGAAGATCGCCGGCGCAGCCGCGCTGATCAGCATCGGGACCTGGTTCGCCT
TCCCTCAGTCGTTCATCTTCTTCGGCATTCTCCACGCCATTGCGGCGGCAAGCCTTGTCGGCCTCCTGTTCCT
GAAATTGCCGGCCGCCGTTTCCTTTCTTGCCGCTGCCGTGGCCTTCGCGGCTCCCCTCTATCTGCGCTCGTCG
TTTTTCGACATGCCGGCGCTCTGGTGGGTCGGCCTCTCGGAAACGATCCCGCGCTCGAATGACTACGTTCCTC
TGCTTCCCTGGATTGCCCCTTTCCTGCTCGGCCTCGGCACCGCGAAGCTGCTCCACCCGTTATTTTCCGCCCG
CCGCAGCCGAAGCGCCGGGACCCGTAAGCACCCGTGGATGACGCCTCTCGCCTTTGGCGGCCGTCACAGCCTT
CTGATATATCTCATCCACCAGCCGCTGCTTATCGCGGCAGTCTATCTCTTCTCTCTCGCCGTTCCCGCCCCGG
CACCGGATCCGGCACGGGTCTATCTGCTGAACTGCGAACGGGCCTGCAGCAACGAAAATTCCGGGATCTCCTG
CAGCGCCTTTTGCGGTTGCACGCTCGCCGGCCTGAAGGAACAGAACCTGTTCGACGATCTGAACCTGGGCAAG
ATCGACGTGAACACGGATGAACGCATTGCGCGAATCGCCGGTCAGTGCACAATGGATGCACAATCAGGAGAGG
CACAGTCAGGGGACTAGATGAACTTCGAAGCAGCCCGCATCAAGATGGTGGACAATCAGATCCGGACCACGGA
TGTGACCTCCCATTCAGTCCTGTCTGCTTTCCTGTCGGTCCCGCGTGAGGAGTTCGTGCCTGCAAAGATGCGG
GAGCTGGCCTATATCGACACCGATATCGAGCTCGTCGGCGGTTCCCAGCCGCGTTACCTGATGGAGCCGTCGC
CGTTGGCAAAGCTCCTCCAGCTCGCGGAGATCTCCAAATCGGATCTGGTCCTCGAGATCGGCTGCGGGACGGG
TTACGCCTCGGCTCTGCTTTCGCTTCTCGCCGGTTCCGTCGTGGCGCTGGAGAGCGACGAAGCCCTGGCCGCC
ACGGCCACGGCAACGCTGGCACGCCTCGGTTACGACAACGTCGCCGTGGTGAGCGGCGATCTGACCAAGGGAT
ATGCGGCGGAAGCGCCCTATGACGTCATCTTCATCGACGGTGCCGTCGAGATGCTCCCTGCCGAGCTGTTCGA
GCAGCTTCGCGACGGCGGCCGGCTCGTGGCCGTGGAAGGTTTTGGTAACGCATCTCGTGCGAAACTTTACGTG
CGCGAATCGGGCATGACGTCCGAGCGAGCTGACTTCAACACCGCCGTGAAGCCGCTTCCCGGCTTCCGCCGCG
ACCGTTCTTTTGTTTTTTGAATGGTTTCAGATGCGCGTAACGGCGCCGTCGCGATGACGAGGCGCTCGGGTGT
GTCGTTCGCAGCGAACCTGGCATTCGAGCATATCAGCCATCGTTACCATTCCAAGGATACGATCAAGGGCGTA
ACGCTCAAGGCCGAAGCCGGCGAAGTGCTCTGCCTGCTCGGACCGTCCGGCTCCGGCAAGACAACGCTGTTGC
GTATCGCCGCGGGCATCGAGAAGCAGACGGGCGGGCGCCTTCTGCTGAACGGACAGGAAATATCCGGCCCTTC
CATCTTCGTTCCGCCCGAGCAACGCGGCGTCGGCCTGATGTTCCAGGACTTCGCCCTTTTTCCTCATATGACC
ATCCGTGAGAACGTCTGTTTCGGCTTGACCGAATTGCCGAAGAAGAAGGCATTGCTGCAGGCCGATGCCGCGC
TGGAGCGGGTCGGCCTTCTGCATTATGCCGAGCGGTATCCGCATGTGCTCTCCGGCGGCGAGCAGCAGCGCGT
GGCGCTGGCCCGTGCACTTGCGCCGCGGCCGGCGGTGCTGCTGATGGACGAGCCTTTTTCCGGCCTCGATTCC
CGCCTGAAGGATTCTATCCGCGCCGACACGCTGGCCATACTGCGCGAGACGCGCGCGACCGCCATCGTCGTAA
CGCACGACGCCGAGGAGGCGATGCGTATGGCTGACCGGATCGCCCTCCTGAAGGACGGCCGCCTGGTGCAGGT
CGGCACGGCGGACGAGCTCTATCGCCGGCCCTGCGATCTCTTCACTGCCGGCTTCTTCTCCGAAATAAACGTC
TTCGATGCGCGCGTGCGGGACGGTCATGTCGAAACGCCGCTGGGGGTTGTGGCAACCGGGCAATATACGGAGG
GGCAGCCACTGAGCGTAGCCGTGCGTCTTTCCGGCATCCGGCTCGACGAGACCGGTGGAGAAATTCCGGCGCG
CATCGTGTCCCGGCGCTTCCTGGGCGTCGTGGAGCTTTTGGAACTCGCCGTGCCGCAGTCCGAACGCACGGTT
CGCGCCCGCATCCGCGCCGATTTGCTGCCGCAAGGATTGCGTGACGTCACGCTTTCCGTTAACGAGCGCGACA
TATTGGTGTTTGAAAAAGACGCATCAACACCTTAGATGCCGAAGCGCCAAGACATCAAATCGATCCTCATCAT
CGGCGCGGGGCCGATCGTCATCGGTCAGGCATGCGAATTCGACTACTCCGGCACCCAGGCGTGCAAGGCGTTG
AAGGAGGAAGGCTACCGCGTCATCCTGGTCAACTCCAACCCGGCGACGATCATGACCGATCCGGGTCTGGCGG
ACGCGACCTATGTCGAACCGATCACGCCAGAGGTCGTCGCCAAGATCATCGCCAAGGAACGCCCCGACGCGCT
GCTGCCGACCATGGGCGGACAGACGGCGCTCAACACGGCCCTGTCGCTGCGCCGCATGGGCGTGCTCGACCGC
TACAATGTCGAGATGATCGGTGCGAAGCCCGAGGCGATCGACAAGGCGGAAGACCGCGCCCTGTTCCGCGAGG
CCATGGCGCATATCGGCCTCGAGACGCCCAAGTCGCGTCTGGCGAACGCCACGGACATCAAGGACCACGACCG
CAAGTCGCACGAGGCGGAGCGCTCAGCGCTCAAGGCGAAGCTTTCCGGTGACGAACTCGACAAGGCGCTGGAC
GAACTCGAAAACCAGTGGAACCTCGGCGAAGGCGATCGCAAGCAGCGCTATGTGAACCACGCCATGGCGATCG

CCGCACAGGCGCTCGACGATGTGGGCCTCCCCGCGATCATCCGGCCGTCCTTCACGCTCGGCGGCACCGGGGG
CGGCATCGCCTATAACCGTTCGGAGTTCTTCGAGATCGTCGGCAGCGGTCTCGACGCGTCGCCGACGACGGAA
GTCCTGATCGAGGAATCGGTCCTCGGCTGGAAGGAATATGAAATGGAGGTCGTCCGCGACAAGGCGGACAACT
GCATCATCATCTGCTCGATCGAGAACATCGATCCGATGGGCGTCCACACGGGCGATTCGATCACCGTTGCGCC
GGCACTGACCTTGACCGACAAGGAATATCAGATCATGCGCAACGCCTCGATCGCGGTGCTGCGCGAGATCGGC
GTCGAAACGGGCGGCTCGAACGTCCAGTTCGCCGTCAATCCCGAGAATGGCCGCCTCGTCGTCATCGAGATGA
ACCCGCGCGTCTCGCGCTCCTCCGCACTCGCATCGAAGGCCACCGGCTTCCCGATTGCCAAGATCGCCGCCAA
GCTCGCAGTCGGCTATACGCTCGACGAACTCGAAAACGATATCACCGGCGGCGCGACGCCTGCATCCTTCGAG
CCTTCGATCGACTACGTCGTCACCAAGATCCCGCGCTTCGCCTTCGAGAAATTCCCGGGAGCCGAGCCGACGC
TCACGACCGCAATGAAGTCGGTGGGCGAAGTCATGGCGATCGGCCGGACCTTCGCCGAATCGCTGCAGAAGGC
GCTGCGCGGTCTCGAGACCGGACTGACCGGACTTGACGAGATCGAGGTGCCTGATTTCGACGACAATGGCGAC
GGCCGCAATGCCATCCGCGCCGCGCTCGGCACGCCGACGCCGGACCGCCTGCGCATGGTCGCACAGGCGCTGC
GTCTCGGCATGAGCGAAGCCGAAGTGCATGAAGCCTGCAAGATCGACCCCTGGTTCATTGCCCAGTTCAAGGC
GATCGTCGACATGGAGGCGCGCATCCGCGAGCACGGCCTGCCCGCCGACGCCGAGAACCTGCGCATGCTGAAG
GCAATGGGCTTCTCCGACGCCCGGCTTGCGACGCTTACCGGCAAGCGTCCGAAAGAGGTGGCCGAGCTCCGCA
ACGCACTGAACGTGCGCCCCGTCTACAAGCGCATCGACACCTGCGCGGCCGAGTTCGCATCGCCGACCGCTTA
CATGTATTCGACCTATGAGACGCCTTTCGTCGGTGCCGCCCGGTCGGAGGCGCAGGTTTCCGACCGGAAAAAG
GTCGTCATCCTCGGCGGCGGCCCGAACCGGATCGGCCAGGGCATCGAATTCGACTACTGCTGCTGCCATGCCG
CCTTCGCACTGAAGGACGCCGGCTACGAAGCGATCATGATCAACTGCAACCCGGAGACGGTTTCGACCGACTA
CGACACCTCCGACCGGCTCTATTTCGAGCCCCTGACGGCAGAGGATGTGATCGAGATCATGCGTGCCGAACAG
GAAAACGGCACGTTGCATGGCGTCATTGTCCAGTTCGGCGGCCAGACTCCGCTGAAGCTTGCCGAAGCGCTGG
AAAAGAACGGCATCCCGATCCTCGGCACCGCGCCGGACGCGATCGATCTCGCCGAGGACCGTGATCGCTTCCA
GAAGCTCCTGATGAAGCTCGATCTCAACCAGCCGAACAACGGCATTGCCTACTCCGTCGAGCAGGCCCGCCTC
GTTGCCGGCGAAATCGGCTTCCCGCTGGTCGTGCGCCCGTCCTATGTTCTCGGCGGCCGCGCCATGCAGATCA
TCCATTCGGAAAGCATGCTGCAGAGCTATTTGCTCGACACCGTTCCGGGGCTGGTTCCTGAAGATATCAAGCA
GCGCTATCCGAACGACAAGACCGGCCAGATCAACACCTTGCTCGGCAAGAACCCGCTCCTCTTCGACAGCTAC
CTGACGAATGCGATCGAAGTGGACGTCGATTGCCTTTGTGACGGCAAGGATGTCTTCGTTTCGGGCATCATGG
AGCATATCGAGGAGGCCGGCATCCACTCGGGCGACTCCGCCTGCTCGCTGCCGGTGCATTCGCTCGGCACGGA
CATGGTCGACGAACTGGAGCGTCAGACGGGGGCGCTCGCCAGAGCGCTGAACGTCGGCGGCCTGATGAACGTG
CAGTTCGCCATCAAGGACGGCACCATCTATGTGCTCGAAGTAAACCCGCGCGCCTCACGCACCGTTCCCTTCG
TCGCCAAGACGATCGGCGCGCCGATCGCCAAGATCGCCGCCCGCGTCATGGCCGGCGAAATGCTCGACGAGGC
GATCGCCGCCTATGGCAAGAAGCCGAATCCGCGCAACCTGAAACATATCGCCGTCAAGGAGGCCGTCTTCCCC
TTCGCCCGCTTCCCGGGCGTCGATACGCTGCTCGGCCCGGAAATGCGTTCGACCGGCGAGGTCATCGGGCTCG
ACACCGATTATGCGCTGGCCTTTGCCAAGTCGCAGCTCGGCGCCGGCGTCGACCTGCCGCGCGACGGAACGGT
CTTCGTCTCGGTTCGCGACGAGGACAAGGAACGGGTGCTGCCCGCGATCCGCATTCTCTCCGACATCGGCTTC
AAGGTCATGGCGACCGGCGGCACGGCGCGCTTCCTCGGCGAGCAGGGCATCGTTGCAACCAAGATCAACAAGG
TCCTGGAAGGACGGCCACATGTCGAGGACGCCATCCGCAACCGGCAGGTCCAGCTCGTGATCAACACGACCGA
CGGCAACAAGGCGATCTCCGACTCGAAATCGCTTCGCCGCGCGACGCTGATGCAGAAGGTGCCCTATTACACC
ACGATGGCCGGCGCCGAGGCCGCGGCACTCGCCATCAAGGCACTGAAGGCGGGCAACCTCGAAGTGCGTCCGC
TGCAGAGCTACTTCGAAACCTGATTGAACGCGAATCTGATCCGGCATGCTGTCCATCTCGCGGCGATCCTGGG
GCTCTATCTCTCCGGCGCGATGCTGATTCCGGCCCTGGTCGATCTCTATTATGGTCACCCGGACTGGCAGGTC
TTCGCCGTGACCGCCTTCATGTCGGGCGCAATCTCGTCGACGACGTTCATGGCGACCCGCGCAGGCCCCCCGC
CCTTCTCGAAGAAATTCGGCTTCCTGGTTGTGAATGTCCTGTGGATCGTGTTTTCGATCGTCGGCGCCATTCC
ACTCTGGCTGTCCTCGGTAAAGCTGGATTTTGCCCAGGCCCTGTTCGAGTCCGTCTCGGCGGTAACGACGACC
GGCTCGACCGTAATCGTCGGGCTCGATGACGCGCCGCCCGGCCTCTTGCTCTGGCGGTCGCTCCTGAGCTGGT
TCGGCGGCATCGGCATCGTCGTTCTCGGCCTGTTCATCATCCCCTATCTGCGTGTCGGCGGAATGTCCTTCTT
CAAGATGGAATCCTCCGACACCAGCGACAAACCCTTTGCCCGGCTCGCGAGCTTCGGCCGCGCCTTCCTCGTC
GTGTACGTGTCGATCACTCTGCTGTGCGCGATCAGCTATGCCATGCTCGGGATGAACAGGTTCGACGCGATCA
ACCACGCGATGACGACCGTCGCGACGGGCGGCTTTTCTACGCACGACGCGTCATTCGGCTACTTTTCCAGCAT
TCCCCTGCTCTGGGCCAGCACGTTCTTCATGACGGTCTGCAGCCTGCCCTTCTCCATCCTGATCGTGCTCGTC
GTGCGCGGGCGCCTGGACGCCCTTCGCGATCCTCAGATCATCGTATTTCTGGGCTATCTCACCGCATTCTCGG
TTGCGGTCGCGGCCTATCACCGGCTCGCCAACGGCGTCGAGTTCCACCTCGCGCTTACCTATGCCTTCTTCAA
CATGACGTCGATATTGTCGACCACGGGCTATGCGAGCGAAGATTACAGCCTTTGGGGACCGTTCGTGGTCATG
GTCGCGTTCATATGCACGTTCATCGGCGGTTGTTCAGGCTCGACAGCGGGCGGCATGAAGGCCTATCGGTTCG
TCGTGCTCTTCAACGCCATCCGGTCTGCCCTGAACAAGCTCGTCTACCCGAATGCGATCTACGCGGTCCGGTA
CGGGAAGAATACGGTCGACGCGGACGTGCAGCGCGCCATTCTGCTGTTCTTCATCACCTATATCCTGCTCTGG
GTCTTCGGCAGTCTGGTCATGGGTGCCCTCGGCTATGATCTCGTCACGGCCGTGTCGGCCGTCATCACGTGTC
TCTCCAATGTCGGCCCCGGCCTCGGCAGCCTTATCGGCCCTGCGGGCAATTTCTCGACACTCCAGGATCCCGA

GCTCTATCTGCTGTCGCTGATGATGCTGCTCGGGCGTCTGGAGGTCCTGACCGTCCTCGTCGTGCTCACTCCG
ATCTTCTGGAAGCAGTAAATGCCGAGATACCTGCTCCTGCTGGCGGCACTCACGAACGCCACCGTCATCCTCG
CCGACACGCAGGCAGAGGCGGGTCCGCTTGCCGACGCTGCGGCGAAGGCCGAACAGCAGGCGAGCTCCGGCGA
TGCCGCCGGCGCCCAGGAAACCTTGCGCAAGGCCATGAGCGACTTTTCGCAGACCCTTCCCTTCGCCATCGGC
AAGGCGGTCTTCGTCACAGCAGCACCGGCCGGCTTTGCAATGTACGAGCCCCGGCCCGATCCGGTATTCGAAG
CCGGCGAAACGCTCATATCCTATGTCGAGCCCGTGGGACTTACCTGGAAGGAGGCAAGCGACGAGGGTAAGCT
CGAGACACGCTTCACGGTCGACTTCGACATCCTGAATCCGAAGGGAGAGGTCCTCGCCAGCCAGAAGGCGTTC
GGCGATTTCACCTTCAAGGGCTATCAGCGCAACCAGGAAATCTATGCGACGCTGAGGATCGACGTCGCCGGGG
CACCATCAGGGAATTATGTGCTGCGCTACCGCTTCAACGACACCAACAGCGGCAGGAGCGCAAGCGTCGATCA
ACCATTCGAGATCTCCGGCCCTTGAATGCGCATTGAACTCGAAAAGCGCGCGAAGGCCTCGACGCTGTTTTCC
ATCCTTTCGCCGTTCATCGCATTTGCGCTGACCATCGTCTTCGGCGGCGTGATGTTCGCACTTCTCGGCAAGA
ATCCGGTAACCGCGCTCTACAGCTTTTTCGTGGAGCCGCTGAGCGAGGTCTGGTCGCTGCACGAACTCGCGAT
CAAGGCGGCGCCGCTGATCCTGATCGGCGTCGGGCTCTCCGTCTGCTTCCGCTCCAACAACTGGAACATCGGC
GCAGAGGGTCAGTTCATCATGGGCGCAATCGCCGGCTCGGCCCTGCCGGTACTCTTCTACGATTGGCAGTCAC
CGTTGATATTGCCGCTGATGATGCTTCTCGGCATGCTTGGCGGAGCGCTCTTCGCGGCGATACCGGCCTTCCT
GAAGGCGCATATGAATACGAACGAGATATTGACGAGCCTGATGCTGGTCTATGTGGCCCAGCTCTTTCTCGAC
TGGCTCGTACGTGGCCCTTGGCGCAACCCGCAGGGCATGAACTTCCCGGAAACGCGAACGTTCGGCGCGGATG
CGATCCTTCCCGAAATGCTCGCCTCGGGGCGTACGCATTGGGGTTTTGCCTTCGCGATCATCGCGGCGGTGCT
GGTGTGGTTCATGATGCGCTATACGTTGAAGGGCTTCGAAATCACCGTTCTCGGCCAGTCCGAGCGGGCAGGG
CGCTTTGCCGGCTTTTCCTCGCGCAGGATGATCTGGTTCTCGATGCTGTTTTCCGGCGCGCTGGCGGGGCTCG
CCGGTATTTCGGAAGTGAGCGGCGCCATGCAGCAGCTGCGTCCGGTGATCTCGCCCGGTTACGGATTCACGGC
TATCATCGTCGCGTTCCTCGGCCGGCTCAATCCGCTCGGAATCGTGGCCGCCGGTCTCGTCCTGGCGTTGACT
TATCTCGGCGGCGAGGCCGCGCAGCTTTCGATCGGCGTATCCGAAAAGGTGACGCGCGTCTTCCAGGGGCTGC
TGCTGTTCTTCGTGCTGTCTTGCGACGCGCTCATTCACTACCGCATCCGGCTGGTGTGGGACCGCTTCGCAGC
GGTCAGGACGGAGGAGGCGCACTGAATGCGGAGTCTCCGCCAACCTGGTGCACCGTTCACGCCGAGGGTAATC
TCTCTCGGGTGCCACGCCGAGAAGGCGCGATTGACCTTCAAACCGGGCCGGAGCGTGCTCGAAGCCGTCCATG
AGGCGCTCGCGGAAACAGGCTGCGACAGCGCGATGATAGAGGTCAGGGGCGGTGCGTTTGCGCCGCTTGCCTA
TGTCACACCGACTCTCTCGGATGACGGCATTCATGCCGCCTGGTACAGCGACATACATGCGCCTGATGGCCGC
GCACCCATCGAAGATCTGGTCATGACGTTCGGCCGGCGCGACGGGGAGCCGTTCCTGCACGGCCATGGTGTCT
GGCGGCACGAGGACGGGTTGCGCGCAGCCGGGCACGTAATGCCGAAGGACTCGCAATTCGCCGAACTGGTGGA
GGCGGAAGCCTGGCTCATGTCGGGCGCCATCATGGACCAACTGGAGGACAGCGAGACCCGGTTCCGTCTGTTC
ACGCCTGTGCCTCATACCGCCGCACCGCCCATCACGACGCGGCGCGCCGTTCTCTGCCGGTTGAAACCGAACG
AACCGATCCATCCGGCGATCGAGCGCACCGCAGCCGAGCACGGCATCGAGCGGGCGACTTTGCACGGCATCGG
CAGTCTCGTCGGCTGCACTTTCGCCGACGGCCAGGTCATGGAATCGGCCGCTTCGGAATTGCTTATTCGCAAG
GGGACGCTTTCACCCGATCGAAGCGGACAGGCTAAAGCGAAACTCGATATCGCGATCGTCGATACGGAATGCA
CCATATTCGAGGGTGAGATAGCCTACGGCGTTGATGCCGTTTGCATAACGTTCGAGGTGCTGATCGTCGAAGA
GTGAATGGCCTGGCAACCGGCGGAAAACAGATACGAGAAGATGAAGTACAATCGCTGCGGCAGGAGCGGCTTG
AAGCTGCCGGCGATTTCGCTGGGTCTTTGGCACAATTTCGGTGGCGACACGCCGCATGAGCGCAAGGTGGACA
TGTGCCGCACGGCCTTCGATCTCGGCATCACCCATTTCGATCTGGCCAATAATTACGGTCCGCCGCCCGGCTC
GGCCGAAATCGCCTTCGGCGAGATCATGCGCAGCGATTTCGCCGCCCTGCGCGACGAACTCATCATCTCGTCC
AAGGCGGGGTATGACATGTGGCCCGGCCCCTATGGCGAGTGGGGCAGCCGGAAATATCTTGTCGCCTCTTGCG
ATCAGAGCCTCAAACGCATGGGGCTCGACTATGTCGACATCTTCTATTCCCATCGCTTCGACCCGGAAACGCC
CCTCGAAGAGACTTGCGGAGCGCTCGATCACATCGTCCGCTCGGGGCGTGCGCTCTATGTCGGCATCTCCTCG
TATAACTCCCAGCGTACCCGCGAGGCGGCGGTAATCCTGAAGGAGATGGGCACGCCCTGTCTCATCCACCAGC
CGAGCTATTCGATGCTCAACCGTTGGGTCGAGGATGACGGTCTCGTCGACACGCTGGAGGAACTCGGCGTCGG
CTCGATCGTCTTTTCGCCGCTCGCCCAGGGCATGCTGACGACGAAGTACCTGAACGGCATTCCCGAGGACAGC
CGCGCCGCGCAGAACCACTTCCTCAAGAAGGACTTCATCCGTCCGGGGATCATCGACAATATCCGCAAGCTGA
ACGCCATCGCCGAACGGCGCGGCCAGACGCTCGCCCAGATGGCACTCGCCTGGGTGCTGCGCGGCGGCCGCAT
CACCTCGGCGCTGATCGGCGCCAGCCGTTCCGCACAGATCGTCGACTGCGTCAAGGCGCTGGAAAACGACACT
TTCACACCAGAGGAACTGAGCGAGATCGATCTTTACGCCCGCGAGGCCGACGTCAATCTCTGGGCCAAATCCG
CCGAGCTCTGAATGAGCAAGGCTTATAAGGACCACCTCGGCCGTCCGATCGTTGCCGTCACCGGCATGGGCGT
CATCACCTCGCTCGGGCAGGGGCTCGACGACAACTGGGCGGCGCTCACCGGGGGCGTATCGGGCATTCACAAG
ATCACCCGGTTCCCGACCGATGCTCTTTCGACCCGCATCAGCGGCACGGTCGACTTCATCGAGATTCCTGCGG
AGAATGCCGTCGAGCGTTCCTATGCGATGGCGCGCGAAACGACGCTGGAGGCCCTGGCTCAGGCCGGCCTTTC
CGGCGATTTCAACGGCCCGCTCTTCCTGGCGGCCCCGCCGATCGAGCCCGAATGGAATGCCCGTTTCGAGCTT
GCCGACCGCTCGCCGCCGTCGAGCCAGCCGGGCGACGCCTATAATCGGTTCCTCGCGGCAATGCGGCAGAAGG
CGGATCCGACCTTCCACGAGGCCGTGCTATTCGGCTCGATCTCGGAGCGTCTTGCGGACCGCTTCGGTACGCG
CGGCCTTCCGGTGACGCTTTCGACGGCTTGCGCATCCGGGGCGACCGCCATACAGCTCGGCGTCGAAGCCATC

CGCCAGGGCAGGACCGACAGGGCGCTCACGGTTGCGACGGACGGCTCGGTCAGTGCCGAGGCCCTCATCCGCT
TCTCGCTGCTCTCGGCGCTCTCCACACAGAACGAGCCGCCGGAAAAGGCTTCGAAGCCCTTCACCAAGGATCG
CGACGGTTTCGTGATTGCGGAAGGCGCCGCGACGCTGGTGCTCGAATCGCTGGAAGCGGCGGTCGCTCGCGGC
GCCCGCATCTACGGCATTCTCAAGGGCTGCGGCGAAAAGGCCGACCTCTTCCACCGCACCCGTTCCTCCCCCG
ATGGCGGACCGGCGATCGCGACGATCCGCGCGGCACTTGCGGATGCGGGCATCGACGAAGCCGGGATCGGCTA
CATCAATGCCCATGGCACCTCCACGCCCGAAAACGACAAGATGGAGTATCTGTCGATGTCCGCGGTCTTCGGC
GAGCGGTTGCCGTCGATCCCGGTCTCATCCAACAAGTCGATGATCGGTCACACGCTGACGGCAGCCGGTGCGG
TCGAAGCGGTCTTCTCGCTCCAGACGATGCTCAGCGGCACGCTGCCGCCGACGATCAACTACCAGAACCCCGA
CCCGGCGATCGTTCTCGATGTCGTGCCGAACGTGAAGCGCAGCCAGCAGGTGACTGCAGTGCTGTCGAACTCC
TTCGGCTTCGGCGGCCAGAACGCCAGCCTTGTCATGACCGCCGAGCCGGCCTGAATGAGGCGGCTGGCAGCGA
TCATGGACGCGGACGTCGTCGGCTACAGCCGGCTGATGGGACTGGATGAAGCGGGCACGTACCGGGCGGTCAA
ACGGTGCCGTGATGCCTTCATTCTGCCTTTGGTCGCGGCCCATGGCGGAAGGATAGTCAAGCAGGCGGGAGAC
GGCACGCTTGCGGAATTCGCAAGCGTTCTCGACGCGGTCGCCTGCGCGGTCGCGATCCAGCGAACGATGCATG
ACCACGCCGGAGGCGCCGAAGCGGAGCGTCTGGAATTGCGCATCGGCGTCCATCTCGGTGATATCGTCGCTGA
CGATGGCGATATCCATGGCGAAGGCATTGCCGTTGCCGAGCACCTGCAGGAGATGGCGCCGCCCGGGGGCGTC
TGCGTGTCGCAGCAGGTCTATGACCAGATCGGCTTGAAGGTGGATTTCCCCTTGACCGAAATCGGCTGCAGGA
CATTTGCCGATCTCCCCGGTCCGCTGCGCGCCTGGCGCTGGCAGCCGGGCGGCGCGGCAGGCGAAGAGCCGCC
GGCGCCGGCGCAAACCGATCCCCTGCCCGGTAAGAAGCGCCCCTCGATCGCCGTCCTGCCCTTCGTCAATCTT
TCGAGCGTCGAAGAACAGGAACATTTCTCCGACGGCTTTACCGAGGAACTGATCGCCACGCTCGCCCGATGCC
GGTGGTTGCGCGTCGTCGCCCGCAACTCCTCCTTCACCTTCAAAGGGAAGGCAGCCGACGTAAGGAGGGTGGC
GGAAGACCTGGGTGTCAAATACGTGATCGAGGGCAGCATACGTCGCGCGGCGAACCGCATCCGCATCACGGCG
CAATTGCTGAGCGGCGAGACCGGCATGCTGCTCTGGGCAGAACGCTACGACCGCATGCTGGACGATGTTTTCG
TGCTCCAGGACGAGATCGCGGGGCAGATCACCGGCACCGTGGAGCCGGAACTCGGCTTCATCGAATTCGCTGC
GCTCCGCGGCCACACCGCGACGGACATGGATGCCTGGAACATTTATCTGAAGGGGCTATGGCACCTTTACAAG
TTCGATCTGGAGAATCTGAGGATATCCAAAGCGCTCTTCGAGCGGGCGATCGATCTCGAGCCCGCCTTTGCCC
AGGCCCATGCGCGGCTTGCCTATGTCCATATACAGCTCGGCTGGTATGGCCCCCTGGACGAGCGGGCCGAGCG
GATCGCCGACGCGACGGCGCTCGCGGAACGCGCAATCGCGCTCGACGACCGTGAGCCGGCTGCCCATCTTGCG
CTCGGCCGGGCACGGGCGCTCGGCGGCCAGCCGCAGCGCGGCATAGACCACCTGCGCAATGCGCTGAGGCTCG
ATGCAAGCTTCGCCCAGGGCCACTTCGCCCTCGGGCAAGCGCTTTGCTATGTCTGCCGCCCGGAGGAAGGCAT
TGCCGCGATCAACGAAGCCTTCCGGCTGAGCCCCCGCGATCCGCATTTGTGGACGTTCTACAACATGGTGGCC
ATCGCCCACTATCAGTCGGGTCGCTTCGCGCAAGCCGCCGAAGCGGCCCGCGCCTCCCTGCGCCAGGAGAACG
CCACCTTCTGGCCGGCAATGGTCCTGGCAGCCTCCCTCGGTGCCACGGAGCGGTCCGACGAAGCCGGTTCAGC
CGTGGCGAACCTTCTTCGCCGGCGGCCGGACATGACCGTGAAAACGGCCCGCGCCGAATTCTACTTCGGCAGC
GTGCCGGTCATGCCGGAAGATTTCATCGATCGTTTCGTCCGCGATCTGCACCGCGCCGGCCTGCCTGACTGAA
TGGCGCAATTGATCCTCAACCACCTGACCAAGGACTTCGGCGGCAAAGGTGCAGACCGGGTAAGGCCGGCTGT
TTCGGACGTGTCGCTGGCGCTGCGCAAGCAGGGATTTCTCGCACTGCTCGGGCCTTCCGGCTGCGGCAAGACG
ACGGTGCTCCGGATGATCGCCGGCTTCGAGCAGCCGACGGACGGCTCGATCGAGCTCGGCGAGCGCAGGCTTT
CGGATGCAACCCGCGTGCTGCCGCCGGAAAAGCGCAACATGGCGATGGTGTTTCAGTCCTATGCGCTCTGGCC
GCATATGACGGTGGCGGAGAATGTCGGCTACCCGTTGAAGGTCCGCGGCATTTCCGGGGAAGCCTGGCGCAAG
CGGGTAGGCGACGCGCTTTCGCTGGTGAAGCTTACGGATTTCGCCGATCGGCGGCCGGCTGTACTTTCCGGCG
GTCAGCGGCAGCGTGTGGCGCTGGCGCGCTGCCTGGTCACGGAACCGGACGTGGTGCTGCTCGATGAGCCCCT
CGCCAATCTCGATCAGCATTTGAGGAAGTCGATGGAGGAGACCTTCCGCGCCTTTCACGAGCGCTCCGGCGCG
ACGATGATCTATGTGACTCACGACCAGGCCGAGGCGATGGCGCTTGCGACCGATGTCGCGGTGATGTCGGAAG
GCAAGCTTCTTCAGGTGGCGCCGCCGGCGGAAATCTATGCGCGGCCGGAGGGGCGGGTCGTCGGGGGCTTGAT
CGGTCGCGGCGCGATCGTGCGGTTGAATTTGCCGGAGGGAGGGGCGCGTGCGCTCGATTGGCCGCTCCTCGGC
GCCGCGCTTGGTGAGATGCCCGCGGGTGAGGAGCATGCAGGCGGGGGGCCGCTTTCCGACGTGCTCGTCCGTC
CGGAGCAGGTTCATCGGGATAGCGAAGGCATTCCGATGCGGGTTCTCTCCTCGGTCTTCGCGGGCGAACGCTT
CGCTCTCACACTGGCGCTGCCGGACGGGCAGAGCGTCAAAGCCTATAGCAGCGTGCCGCTCTGCGAGGGCGCT
CGCGTTCCTTCGTCGTGAGTGATCATCCTCGAGACCGGGCGGCTCAGGCTCCGCGCCTGGATCGAGACCGATC
GCGAACTCTTTGCCGAGATCAACAGCGATCCCGCGGTCATGGAGTTCTTCCCGTTCCGCCGCACCCGCGCGGA
GGCCGACGCGCTTTTCGACCACAACAACCGCAACATCCGCGAAACCGGCTTCGGCTTCTTCGCGCTCGCGTTC
CGTGAGAACGACGCGCCGATCGGCTTCTGCGGACTTGCACGCACCGACCTCGAACCGCACCTGCCCGACGGCA
CCGTCGAGATCGGCTGGCGCCTGGCTCTCCGCCATTGGGGAAAGGGCTACGTGACGGAGGCAGCGCGGGCGCT
TCTCGATTATGGATTCAGCGAGAGGAAGCTCGGGGAGATCGTTGCCATCGCCGTTCCGGCCAACACTCGCTCC
ACCGCCGTGATGACGCGGATCGGCATGCGTCCTGATTCCAAGCGCGACTTCGATCATTCCGGCGTCCCGGACA
CGCATCCCCATCTCAGACGCCACGTGCTTTACGCAACAACCGCGGAAGAATGGCGGCGGGATGGCTCGGCCGC
GAAGCGGTAGATGAATGAGATGAAGTGCAGGATCCGCGGGGCGCATCTGAAGACTCTTTTGGCGAGCGGAGTT
GCGCTCGCGCCGCTGATGATGAGCGGAATCGCGCTGGCGCAGGAGGGAAACGCCACGCAGCTCGAGCGAATCG

TCGTCGAGGGCGGCAATGCGGCCGGCGCCAGCGCGACCGGACCGGTGGACGGCTACGTCGCAAAGGCGACCGC
GACCGGGTCGAAGACCGCCATGCCGCTCAACGAAATCCCCCAGTCGGTCTCCGTCGTCGGGCGTGAGGAACTG
GACGACCGCGCCGTGGTCAACAAGGTCGATGAAGCATTGCGCTATACGCCGGGCGTCCTGTCGGCACCATTCG
GCACCGACCCGGATACGGACTGGTTCTACATCCGCGGCTTCGATGCGGCACAGACGGGCCTCTTCCTCGACGG
ACTGCCGCTCTTCAGCTTCGGCTTCGGCAATTTCCAGGTCGACCCCTTCATGCTGGAGCGGGTCGAGGTGCTG
AAAGGGCCGGCCTCCGTGCTTTATGGCGGGTCCAATCCGGGCGGCATCATCAACCTGATCAGCAAGCGGCCGC
TTGACGAGCCGCTCTACTACACCGAGGTCGGCATCAACAGCAACGGCAATGCCTTCACCGGCTTCGACGTGAA
CGACAAGCTGAACGATGACGGAACGGTTCGCTACCGCCTCACCGGCAAGGTCGCCGGCGGCGACAACTATTCG
GACTATTCCGAAGACCTGCGCGGCTTCATCCTTCCTCAGGTAACCTATGCGCCTGATGACGCAACCAGCCTGA
CCGTCTTCGGCCTGCTCCAGGGCCTCGATCAGGTCCATGTCGGCAACGGCTTCCTGCCCTATGTCGGAACCGT
CGAAGACGCGCCCTTCGGCAAGATCGACCGGGATGCCTATTACAGCGAGCCTGATATCGACGAGGGCAGCTAT
ACGCAGCAGATGCTCGGCTACGAGTTCAAGCACGACTTCGACAATGGCTGGACCTTCACCCAGAACGCGCGCT
ACGCCAATCTTCACAAACACGAGAAGTACCCGTACACCTATGGCTATGTCGGCGGCGCACCTACCGGCCCGGA
CTACCTCCTGAACCGCATCGGCTTCGAGGCGACGTCGAAAGTCGACAGCTTCTCGATCGACAACCGGACGGAA
ACGGATTTCGATCTCGGCGCCACGACCCACACCTTCCTCGCCGGCCTCGATTACAAATACTACCGGCTCGACC
ATATTCAGGCATGCTGCGGCGCGACCCCGATCAGCGCAACCAATCCGGTTTACGGCACACCTCAGGGTGCAAA
TTTCGTATATCTCGATCAGATCGTCACGCAGCAGCAGATCGGCCTCTACGCACAGGACCAGATTCGTTTTGGC
GACGGCTGGCTGGTTACGCTCAACGGCCGCTACGACTATGTGGACACCAAATCCGACGCCGCGATCGGCACGA
GCTACGAATCGAATGACGGTGCCTTCAGTGGCCGTGCCGGCCTCGCTTACGAGTTCGACAACGGCCTCACGCC
CTATGTAAGCGCTGCGACTTTCTTCAATCCGCTGGTCGGCACGGGCACGAGCGATCCATCGGATCCGACGAAT
CCGGCAAAGATGGTCGCGCTCGAGCCGGAGGAAGGCTATCAATACGAGGCGGGCGTCAAATACGAGCCCTCCT
TTATCGACGGGCTGTTGACTGCATCAGTCTTCCAGATAACCAAGCAGAACGTCTCAATTGCCGTTCCCGGCTT
CTTCGTCAACTCGCAGCTTGGCGAGGTCCGTTCGAGGGGCGTCGAACTCGAGGGCAAGATCAATCTCAATACC
AACTGGAAGATCATAAGCGCCTTCTCCTATACCGATCTCGAGGTCACGGAAGATCTCAACGCCAGTCTCATCG
GCAATACACCGGTTCTCATTCCCGAAACACAGGCGTCGCTGTGGCTCGACTACACGGTTGCGAACGGCACGTT
CGAGGGCGTGAGCCTCGGCGCCGGCGTGCGCTACCAGGGGGAATCCTGGGCCGACGCGGAGAACACGAAGAAA
GTCCCCGCTGCGACACTCGTGGACGCCGCTATCCGCTACGAGAAGAACGACTGGACGGCGTCGCTCAACGTCG
CCAACCTGTTCGACAAGGAATATGTTGCGGGTTGCCAGGGTCTCCAGACCTGCGGCTATGGCGAAAGCCGGAC
CTTCACGCTGAAGCTCAGCAAGAAGTGGTAAATGGCTTCAGTAACGCACTCCAGCCCAAGGGGCAGCAGGTTC
AGGATCAGCAAGAGGGCCTTGCCCTATGTGCTCTCCCTGCCGGCGCTTCTCGTGTGCATCGGTATCCTGATCC
CGTTCTTCACGGCGGTGATCTATTCCTTCCAGCGCTACCGGCTCAGCCAGCCCTGGGCACGCCAGTTCAACTG
GGGTGAGAACTATCTGAACTTCTTCACCGATCCGGGTTTCTGGAACACGCTGAAGATCTCGCTGCTCTATGCC
GGCGTCACCGTGACCCTGGAGCTGCTGCTCGGTCTCGGCATTGCGCTGCTCCTGCAGCGCCGCTCGACGGTCA
ACAACTTCATCTCGATCATGCTGCTCCTGCCGCTGATGACGGCGCCGGCGCTTGCGGCGCTGATGTGGAAGCT
GATGACCAATCCCGGCTTCGGGATCCTGAGCTATCTCGCAAGTCTCGTGGGCCTTGAGAACTTCCGCTGGGCA
TCGTCGCCGGACACGGCGCTGCTGACCGTCGTGCTCGTGGACATCTGGGTCTATACGCCCTTCATCATGATCC
TGCTGCTCGCGGGCCTGCGCTCGCTGCCGACGCAGCCATTCGAAGCGGCCGCGCTCGACGGCGTGCCGCGCAG
CTTCGTCTTTTTCCGCATCACCCTGCCGATGCTCACGCCCTATATCCTGACGGCGACGCTCTTTCGCCTGCTC
GATTCCATCCAGCAGTTCGACATCATCTACGCGATGACGCAGGGCGGGCCCGGAGACACGCTGACGGTCTTCC
AGGTCGAGGCTTACCTCAACTTCTTCCAGTCGACCAATGTGGGGCGTTCCGCGGCGCTGCTGATCATCCTGTG
GGCGATCACCTACGCGCTGTCGAACATCTTTATCAAGAACTGGCTCCGGCTGCGCGAACGCGCCCGCGGCGAA
GCGTAAATGACGCTGGAGGAGCTCTACCAGGCCGGGCGCAAGGTCGTCACCGGCAGCCTGACATTCACCGCGG
AGGACATCGTCCGCTTCGCCCGCGATTTCGACCCGCAACCCTTCCACGTCGATGAGGAGCAGGCGAGACAGTC
GCTTTTCGGCGGCCTTTGCGCCTCCGGCTGGCATACCAGCGCCGGCTGGATGCAGTGCTTCCTGCGGTTCTGG
AAGGAGGAGATCCGCCGCCTTGCAGCCGAGGGATTGCACGCGCCGAAGCTCGGCCCCTCGCCCGGGTTCAAGG
AACTCAGGTGGTTGAAGCCGGTCTATGCCGGCGACACGATCACCTATGCGGTCACTCTACTGGAGGCCCGGAT
CGTCGCCTCGCGACCCGGCTGGCGGATCAACACGATCCTCTGCGAGGGCGAGAACCAGCACGGCGAGCCGGTC
ATCCGCTTCGAGAGCAAGGTCATCGAATTCGCTTGAATGTTCGATCCGATCGTTGCGTTCTTCCAGCGTGTAT
TCACCGCGATCGGCCGTGGCATCGGATTGGCGGTGGCGTGGCTTCTCTGGCCGTTCGTCGCGCTCGGCAACTG
GTATCGGGGACGAAGCTGGATCATCAAGGGGCCGATCGGCCTGATCCTCCTCTGCCTCGTGCTCTTCTACGCC
TACTTTGTCTGGCAGACGCAAGCCTGGACGAATTTCGACCCGGACTATGTCGACCGATACAAGCTCGCCGACC
GCAGGACGCCGGCCGGTTCACCCGTCAGCGGTCCGGGGGCAACAACGGGACAGTCAGGCTCGGCGGCTGACGC
AAACCAGGCCGCCGCGGTCGCTCCTTCCGGTGCGGACAGTACGACCGAAGCCCTTGCCGTGGCCCAGCTTCCG
GCCGGGACGGTCTGCCAGACTTCGGCCATCGTCGACGTGGCGGCCGACCTCATCGACCTCAATGTCAATCAGA
ATGCCTGGATATCGTCGATGCTCGGCTACAAGCTCGGCTTTTTCGGGATTGACTGGGACGACACGCCCTGGCT
CGACAACAAGGCGTCCTTCCAGCGCGGCGTCAACCAGGCGGTGCGCCGCACGTCGGTGGAACTCGTCGATTCG
CTTGGGCGCGTCCGTGGCACGTCGGGGGTCAACAACGAGCTCCAGAACGCGCGCGGAAGCATCCAGTTCGACG
AGGAAACCTGGTATTTCGGCCTGAACCCCTTCGGCCCCAAAACCCCGACCCCGAGCTTCTACCGCTCGGCTAT

GCGCGATCTCAGGAAGTTCAACGTCTCGCTCGGCAAGTGCGAAGCGATCTTCGACGGCCGATCCGACAACATG
GTCGAGTTCCTCGACCGGGTCGCGAACGATCTCGGCAATACCTCGGCGATCATCCGCGAACGTTCGGAGTTCC
ACAATGGCGGCTGGTTCGACACGCGGGCGGACGACCGCTTCTGGTTCGCCTTCGGCCAGCTCTACGGCTATTA
CGGCATTCTCTCGGCCGCCGGCGCCGATTTCGAGAATGTCGTCACCCAACGTGGCCTGGCGCCGATCTATACC
GAAACGATGAAGCAGCTTCGCGCGGCACTCAGGATCCAGCCGCTCATCATCTCGAACGGGCGCGAGGACGGCT
GGATCATGCCGACGCACCTCGCGACGATGGGCTTCTACATCCTCCGGGTTCGATCGAACCTCGTGGAGATGCG
CGATATTCTTGCCCGTTGAATGAACTTCTCCGCCTGGTCCATCCGCAATCCGGTCGCGCCCATCCTGGCGTTT
TTCGTGCTCGTGGTGCTCGGATGGCAGTCGTTCAATTCGCTGCCCATCACGCGCTTCCCGAACATCGACGTGC
CGATCGTTTCGATCGCCGTCACACAGAGCGGTGCGGCGCCCGCCGAACTGGAAACCCAGGTCACCAAGGAGAT
CGAGGACGCGGTCGCCGGCGTCTCCGGCGTCGACCATATCGAGTCGACGATCACCGACGGCATTTCGACGACC
GCCGTCATCTTCCGCATGGAAGTCCCGACGACGCAGGCCGTGCAGGATGTCAAGGATGCCATCGACCGCATCC
GAAGCGACCTGCCGACCTCGATCGAAGAGCCGATCGTTTCCAAGGTCGATGTGGAAGGCCAGGCGATCCAGAC
ATTTTCCGTCTCGTCGCCCGGCATGACGCTGGAAGAGCTGTCCTGGTTCGTCGACGACACGATCAAGCGCGCC
ATCCAGGGCCAAACCGGCATCGGCCGTGTCGACCGCTACGGCGGCTCCGACCGCGAGGTCCGGGTCGAGCTCG
ATGAGGACCGTCTGAATTCCTTTGGCATCAGCGCCGCCGACGTCAACGCCCAGCTTCGCCGAATGAACATGGA
CCTCGGTTCCGGCCGCGGCCAGGTCGGCGGCAGCGAACAGGCGATCCGCACATTGGGCGACACGCGCGACGTA
ACGGCGCTGGCGGGTACGATGATCTCGCTGCCGAACGGCCGCTTCGTCCGTCTGTCGGAACTCGGCAAGGTGA
TCGACACCTATGAGGAGCCGAAGTCGTTCTCCCGCTTCAACGGGCAGCCGGGCGTCACCTTCGCCGTGTTCCG
CGCCAAGGGGGCGAGCGAAGTGTCGGTCGCGGAAACCGTCGGCAAGACGCTCGATGAAATCCGCGCGAAGCAT
CCGGATGTCTCCATCGAGATGGTGGACGATTCGGTCTACTTCACCTACGGCAACTACGAAGCGGCGATTCATA
CGCTGATCGAGGGCGCCGTGCTCGCCGTCATCGTCGTTATGCTGTTCCTGCGCAACTGGCGCGCGACCTTGAT
CTCGGCGGTCGCACTGCCGCTCTCAGCCATCCCCACCTTCTGGGTGATGGAACTGCTCGGCTTCTCGCTGAAC
CTCGTCAGCTTCCTGGCGATGACGCTCGCGACGGGTATTCTCGTCGACGATGCGATCGTGGAGATCGAGAACA
TCGAGCGTCATATCCGCATGGGCAAGTCGCCTTACCGCGCAGCGATCGAGGCTGCGGACGAGATCGGCCTTGC
GGTGATCGCCACCACCTTCACGATCATCGCCGTCTTCGTGCCCGTATCCTTCATGCCTGGCATTCCGGGACAG
TACTTCATCCAGTTCGGCCTGACGGTCGCCGTCTCGGTGTTCTTCTCGCTCCTGGTCGCCCGCCTGATCACGC
CGGTCATGGCCGCCTATCTGATGAAGCCTTCGGATGTCAGCGGGCATCATGACGACGAAGGCTTCATGATGCG
CCAGTACACGCGCCTCGTACGCTTCACCACGAAGCGCTGGTACACGCGCTACTCCACCCTGCTTGCGGCGATC
CTGGTCTCGGTCGGTTCCGTGGTCGCGCTGCTCGTCTTCGTCCCGGGCAGCTTCCTGCCGCCGGAAGACAGCT
CGCGCGTCAGCCTGTCCATCGAACTGCCGCCGGACGCAATGCTCGCGGATACGGACCGGACCACCACCGAGAT
CTACAATCGCGTCAAGGATATCGACGGCGTGGAAAACGTCTTCGTACTCGGCGGCGCCTCGCCCAAGGGCGAC
CTCGAACTGCGACGCGCTGCCGTCACGGTGCTGCTCGAGAAGCTCGATCATTCGCTGATCAACAAGGTCGTCA
ACGACGTGATCGGCCGCACACCGCTGATCGGCCAATACCTGCCGAAGCTGCCCCCGGCCGGTCGGATCAAGCC
GCAATCGGAGATCGAGAAGGAAATCTTCGCCCGATTGCGATCGATTCCCGATGTCCGCGTCACCAAGCTGAAC
GATCGCGGCGAGCGTGATCTGTCGTTCAACCTGCTTTCCAGCAACGAGGCGGATCTCGACAAGGCGGTCGCCA
CCCTGGAAGCGGATCTGCGCCGAGATCCGCTGCTTGCCAACGTTAGCCCCGAGGGAGCGCTGCCCCGACCGGA
ACTGCAGATCCGGCCGCGCGACGAGCAGATGTCGCGCCTTGGCATCACGACGGCGCAGATCTCCGAGGTGATC
CGCATCGCCACGATCGGCGATATCGACGCGCAGTTGACGAAGATTGCGCTCGATGGGCGCCTGATCCCGATCC
GGGTGCAGCTCGACCGTGATTTTCGCACCGACCTGGCGGCGATCCGCAACCTGAAGGTCCAGACGGCATCCGG
AGCGACCGTTCCGCTGTCGAGCGTCGCGGACATCAACTATGCGGAAGGGCCGAGTTCCATCAAGCGCTACGAC
CGCTACCGCGTCGTCAAGCTCGGCGCCGACCTGCCCGCCGGCGTGGCGCTCGACACGGCATCGGCCCGCTTCA
GGGAAATCGCCGCCGACGCCGATCTGCCTGAAACGGTCCAGTTCCTTGAAAGCGGCGACGCCGAGGTGCAGGC
CGAGATGCAGGAGAGCTTCGGCAACGCCATGCTGCTGGGTCTGATGATGGTGCTGGTCGTGCTCATCCTCCTG
TTCAAGGATGTGATACAGCCTTTCACCATCCTGTTCTCGCTGCCGCTCGCGATCGGCGGCGTGGCAGCCGCCT
TGATCCTGACTCAGAACGCGCTGTCGATGCCGGTGCTGATCGGTATCCTGATGCTGATGGGCATCGTCACCAA
GAACGCCATCCTGCTCGTCGACTTCGGTATCGAGATGATGCATCACGGCATGGACCGGACGCTCGCCATGATC
GAGGCCGGACGGAAGCGGGCTCGTCCGATCGTCATGACCTCGATCGCCATGTCCGCGGGCATGCTGCCCTCGG
CGCTGGGCGTCGGCGAAGGCGGTTCCTTCCGTGCACCGATGGCGATCGCCGTCATCGGAGGCATCATCGTTTC
GACGGTCCTGAGCCTCGTCGTGGTTCCGTCCTTCTTCCTGATCATGGACGACCTGTCGCGTCTCCTCGCCTGG
GCTTTCGGCCGCTTCATCGGCAAGAAGGACGAGGAGGCACTGCCGCTCGACCAGGAGGCGCTGAGCAAGCTTG
CCGCCGAACAGGGCAGCACGATCGAGAGCCTCGAAGAGCGCATCAAGGCGCTCGAAGACGAGAAGCGCCGCAA
GTCCGACGGCAAGGTCATCAGCCATCCGGCGCTGGCCGCGGAATAGATGTCGCGTTTAAACAGGTTTCTGATA
TCGGCACTGGCCGCAGCGGCTATTGCCGCGCCGGCGCTGGCCACTTCGGCAAGTGCCGCGACGCTGCGTTGGG
GCAGCCGGGCAGATATCTATTCGCTCGATCCGGATTCCGTTCCCTCGACATCCAATCTGGCGTTCCTGAACCA
CATCTATGAAGGTCTGATCCGTTATGGCCCGAACTTCGAGATCGAGCCGGCGCTCGCCACCGAGTGGAAGCTG
ATCGACAGCAAACACTGGCGTTTCACCCTGCGCAAAGGCGTGAAATTCCACGACGGCGCGGATTTCACCGCCG
ACGACGTGGTCGCCTCGATGAACCGCGTTTCGGACCCGACTTCGCCCCTGCGCGGCAATATTCCGCTCTATGT
CGGCGTCAAGAAGGTCGATGATTTCACGGTCGATATCGAGGTGTCGGCGCCGAGCGCGCTGTTCCTGAACGAC

ATGAGCAATATCTTCATGTTCAGCGCCAAATGGCTGAAGGACAACAACGCGGAAAAGCCGACCGATATTGCGT
CCAACACCGAGAATTACGCCACGCACAACACGAACGGCACAGGCCCGTTCAAGCTGGAAAGCCGCGTTCCGGA
CAGCAAGACCGTTCTCCTCGTCAACGATCAGTGGTGGGATCAGAAGAAGCACAATCTGGACCGGATCGAATAT
ATTCCGATCTCGTCGGCGGCAACGCGTGTAGCGGCGCTTCTTTCCAGCGAAATCGATCTGCTCGATTCCGCCC
CCATTCAGGATCTTCCCCGTCTGGAATCCTCGCCGGGCATCACCGTGAGCAAGCGCACGGAGCTGCGTACGGT
TTTCATCGGCTTCAACGGTAAGGAGAAGCTTGAAGACGGGCGTCCGAACCCGTTCCTGGACCTTCGCGTCCGT
CAGGCCGTGGAAGCAAGCATCGATCGCGATCTCATCAACAAGAAGATCATGCGCGGTCTGGCGCGGCCTTCCG
GCTCGCTCATCGCACCGGAAATCGCCGGCTATGCGAAGTCGCTCGACACTTATCAGCCTGCCGATTCCAAGCT
TGCCCAGAAGCTGCTCGAAGATGCCGGCCAGAAGGATCTCGCCTTCACCTATCTGTGCATGAACGACGAGAGC
ATCAACGAGGAAGACATCTGCTCGGGCATTGCAAACATGCTCAAGCGTGCCGGCTTCCAGCCCACCATCGACA
TGGGCCCCCGCGCCGTGCAGCAGCCGAAGCGCACCAATGGCAAGGCCGACATGTTCAATCTGAGCTGGGCAAA
CGAACCGACACTCGATGCCTATTCGCTGCTGTCTCAGGTTCTCTCCACACGCAGCGGCTCGACGGGCGTGTCC
AACTATGGCGGCTGGTCCTATCCGGAACTCGACGATCTGGTGAAGAAGGCGGCACAGGAACCTGACACCGCCA
AGCGTCTCGCGCTGGAAGAACAGGCGCTGAAAATCGCCAAGGACAAGGCCATCCTGATCCCGCTTCACCAGCA
GCCGATCGCCTGGGGCATGCTGGACAAGGTCAAGAGCGTGGATTTCCGCGCCGACAACAAGCCGCGCCACTGG
CACACGCAAATGGCCGAATAGATGCAGCCGCAGGAGGCGCGCGTGTGGGCAACCCTGGCCGGTGCACTTCTTT
TCATCAGCGGGCTTCTCTACATGGCATGGGAGGCGCTCGGACGGCGACGGCTGAGCGAGCCGCCGCAGCCCGC
GGAACGCAATCCCAAGCAGACGCTTGAGCCGCGCGGGCAAGGCCTGCGCTTTCTGAGCCCGTTGCGCAACTGG
CTGGGCGTCGCGCTCATGCTGGTCGGCGGAATTCTCCTGCTCTTCGGCATGCGCTGAATGAGACACGCGGAAA
TCGGAAAGCTCGACAAATCCCAGGCCAGGCATGTCTGGGACGTGGCGGAGTATTGCAGGCGGACCGGCATCAA
CAAGGCCGAGGAACGCCGCCTGATCAGGGTTCTCGGGCGATTTGCATCCAGCCACGAGCTTCAGATGAACATC
GTGCGTCCTCAGACGCGCATCCGCTGAGTGAACGCCAGGGCCTATTTCTATCTCGGTATTACCGCGCTCTTTT
GGGGCGGCAATTCGGTAGCGGGCAAGATGGCCGTCGGGCACGTCAGCCCGATGATGCTGACGACCTTGCGCTG
GTTGCTCGCGCTTGCGGTCATCCTGGTCCTGATGACGCCGCAGATCCGCCGCGACTGGCAAAAGATCCGCCAG
CACTGGCTGCAGCTGCTTGCCTATGGCGCCGTCGGCTTCACCATGTTCAATGCGTTCCTGTATTCCGCGGTGC
AATACACCAGCGCCATCAATGCCGTCATCCTTCAGGCCGGCATTCCGATGCTGATCTTCATCTTCAATTTCGC
GCTTTTCAAGATGAAGGCGTCGCTCGCCCAGGTCATCGGCTTTACCGTCACATTGGTCGGCGTGCTCATTACC
GCCGCCCATGGCGATCTCGCGAGCCTCATGCGCCTGAGCTTCAATTTCGGCGACGCGCTGATGATCCTCGCCT
GCATCGTCTATGCCGCATATACGGTTGCGCTGCGGTGGAAACCGGCGATGCATTGGCAGAGCTTCATAGCCGC
CCCGGCCTTCGGGGCGCTGCTGAGCGCCATACCGCTCCTCTTGTGGGAGATGGGGAAAGGCACGGCGATCATG
CCGGATGCGACCGGCTGGGCGATCGTGATCTATGCGGCGATCTTCCCCTCGCTGATGTCGCAGGTTCTCTATG
TTCGCGGCGTCGAGATGATCGGCGCCAACCGTGCCGGTCTGTTCATCAACGCCATTCCGGTGTTCGGGACCCT
TCTCTCCGTCCTTCTGGTCGGCGAGATATTTCACCCGTTTCATCTCGCGGCGTTGGCACTCGTCCTCGGCGGC
ATCGCCATCGCCGAACGCGGCCGGCCGAAGCTGCCGCGGTGAATGAGAAATAACCTGCTTCCCGTTGCCGCGC
TGCTGCTCGGTACCCTGTTTCTCTTCCTCGGAAACGGCCTGCAGGGCCTGCTCCTGCCGGTGCGCGGCACCGC
GGAGGGCTATCCGACAACCATCCTCGGCCTGTTCGGTACGCTCTGGGCCACCGGTTTCGTGCTTGGCTGCTTC
TTCGCGCCGAACGTCGTCAAGCGCATAGGGCATGTGCGCGCCTTCAGCGTGTTCACGGCCTTGATCGCCATCG
TTTCCCTGCTGACGGGCATTCTGATCGACCCGATCTGGTGGCTGGCGCTCCGCGCAGTCACGGGTTTCTCCAC
TGCCGGAACGTCGATGATCATCGAAAGCTGGCTGAACGAGCGCGCCACCAATGAGAGCCGCGGCGTGATCTTC
TCGCTCTATATCGCCATCACCCTTTTCGGCGTCGTCGGCGGCCAGATGATGATTCCCTTCGGCGAAACGTCGA
CGACCTTCTTCTTCATGATCTGCGGCATTCTCTACTGCGTGGCCATGCTGCCGACGCTGTTGTCCAGGGCCGC
CTCGCCGCAGCCGCTGAAACAGGTCCGCCTCGATCTGCGCGGCCTCTACCGCAATTCGCCGGTTTCCTTCCTC
GGCATCCTGCTCATCGGCATTGCCAACGGCGCATTCGGCACGCTCGGCGCGGTCTTCGGCCGCCAGGCGGGGC
TGTCGGACAGCACAGTTGCGGCGATGATGAGCGTCGCGATCTTCTCGGGCGCCGTCATGCAACTGCCGGCCGG
CCGCATCTCCGACCGTATCGACCGCCGCTATGTGCTTGCCGCGCTCGCCGGGGTCGGCGCTCTCGCCGGCCTC
CTGATCTTCCTCGTCGAGCCCGGCCAGGTCTGGATCGTTCTGACGTTGATCGCGATCTACGGCGCGGCCGCCA
ATGCGCTCTACCCGATCGCGGTTTCCCACGCCAACGACTTCGCCACGCCCGAGGATTTCGTGAAGGTTTCCGG
CGGGCTCCTGCTGCTCTACGGCATCGGCACGATCATCGGCCCGACGATCGGCGGGCCGATAATGACCGCCAGC
GGGCCTTATGGCCTCTTCATGATCACGGCCTGTGCGCATATGCTGATCACTGCCTATGCAATCGTCCGCAGCC
GCCGGCGCGCACCGGTGCCCGCGGCCGAGCGCGAAAACTTCTCGCCGGTCAATGCCGGCACGGCGACGACGCC
GGAGAGCCTGCAGCTCTCGCCGCGTGCGGCTCCGCTGGAGGAACTGCCGGACGAAGGCGCCGACGATCCCCAG
GAAGAGGAGAGATCGAATGAGCCTGTATGAATGAGACGGAACAGGAAAGTCAAAATCCTCGCGACGCTCGGGC
CGGCATCGGCCGACGAGCAAATGATTCAGAAGCTGCACGAGGCGGGGGCCGATCTGTTCCGTATCAACATGAG
CCATGCAAGCCATGAGGTCATGCGCACGCTGATCGAGCGGATCCGTGTGGTCGAGGCGCGCTGCGGCCGTCCG
ATCGGCATCCTCGCCGATCTGCAGGGGCCGAAGCTGCGCGTCGGAAAGTTCGCCGAAGGCAAGGTCGAGCTCA
AGATCGGCCAGACATTCACGCTCGACAACCGCGACGTGCCCGGCGACAGCCGCCGCGTCTATCTTCCGCATCC
GGAGATCCTCGAAGCGGTGAAGCCCGGCCATCGCCTGCTCATCGACGATGGCAAGCTGCATCTGCGCGCCGAA
AAGACCGATGGCAAGAGCATCGTCACCACGGTCGTTTCCGGCACGCGGATTTCGGATCGCAAGGGCGTCAGCC

TGCCCGATACCCTGCTTGGCGTCGGCGCGCTGACGGACAAGGACCGCGTCGACCTCGACGCCGTGCTGGCGAC
AGGAGAGGTCGATTGGGTGGCGCTTTCCTTCATCCAGCGCCCTGAGGACCTTGCCGAGGTTCGCAAGATCGCC
CGCGGCCGCGTTGGACTGATGTCGAAGATCGAGAAGCCGCAGGCGATCGAGCGGATAGACGAGATCATCGAGC
TTTCGGATGCGCTGATGGTGGCACGCGGCGACCTGGGCGTCGAAATGCCGCTCGAATCCGTACCGGGACTTCA
GAAGCAACTGACGCGTGCCTGCCGGCGAGCCGGCAAGCCGGTCGTCGTCGCCACGCAGATGCTCGAATCGATG
ATCTCCTCGCCTGTGCCGACGCGCGCAGAAGTGTCGGACGTGGCTACCGCCGTGTTCGAGGGTGCCGATGCGG
TGATGCTTTCGGCCGAATCCGCCTCCGGCGAGTACCCCGTCGAGGCGGTTTCTACCATGGCGTCGATCGCCAG
CAACGTCGAGCGCGATCCGCATTATCCGGGGATCATCTACGCTCAGCGCACGCCGCCGGAGGCAACGGGCGCG
GACGCCATTTCGCTTGCCGCTCACCAGATCGCCGAGACGCTCAAGCTTGCCGCAATCGTGACCTACACCTCCT
CTGGCACCACCGGCCTGCGCGCGGCGCGCGAGCGCCCCCAGGTGCCTGTCATCGCGCTTTCGCCGATCGTCCA
GACGGCCCGTCGCCTTTCCGTGGTCTGGGGGCTGCACTGTGTCGTCACAGAGGACGCGACCGACCTCGACGAT
ATGGTCAACCGCGCCTGTCGAATCGTCTCGACGGAAGGCTTCGGCAAGCCGGGAGACCGCATCATCGTTACCG
CCGGCGTGCCGCTCGGTACCCCCGGCGCCACCAACATGCTGCGCATCGCCTATATCGGCTCGGACGGCCAATC
GGGCATCTGAATGACTGCGAAAATCCTCCTTGCCGAAGACGACAACGACATGCGCCGGTTTCTTGTGAAGGCG
CTGGAAAAAGCCGGCTACAAGGTTCTTTCCTATGACAACGGCGCCAGCGCCTATGACCGGCTTCGCGAAGAAC
CCTTTTCGCTTCTCCTGACCGACATCGTCATGCCGGAGATGGACGGCATCGAACTCGCGCGCCGCGCGACCGA
ACTCGACCCCGACCTGAAAGTGATGTTCATCACCGGCTTCGCCGCCGTTGCGCTGAACCCGGATTCCAAGGCG
CCGAAAGACGCCAAGGTCCTTTCCAAGCCCTTCCACCTGCGCGACCTCGTCAACGAGGTCAACAAGATGCTGG
CCGCCTGAATGAACATCCATGAATACCAGGCCAAGGCTCTGTTGAAGAGCTATGGCGCGCCGGTCGCGGAAGG
CGTCGCGATCTTCTCCGCCGACGAGGCCGAGGCGGCCGCGAAGAAGCTGCCGGGGCCGCTTTACGTGGTGAAG
AGCCAGATCCACGCCGGCGGCCGCGGCAAGGGCAAGTTCAAGGAACTCGGCCCCGATGCGAAGGGCGGCGTGC
GCCTGGCCAAGTCGGTGGACGAGGTCGTCGCCAATGCCAAGGACATGCTCGGCAACACGCTGGTGACCAAGCA
GACCGGTCCGGCCGGCAAGCAGGTCAACCGCCTCTATATCGAGGACGGCGCCGATATCGACCGCGAGCTCTAT
CTCTCGATCCTCGTCGATCGCTCCGTCGGCCAGGTGGCCTTCGTCGTTTCGACCGAGGGCGGCATGGATATCG
AAGCGGTCGCCGAACATACGCCGGAGAAGATCGTCACCGTTGCGATCGATCCGGAAAAGGGTGTGACCGCAGA
GAATCTGAAGACGCTGGCCGACGCGCTGAAGCTCGAAGGCGAAGCGCGTGCCGACGCCGAGAAGCTCTTCCCG
ATCCTCTACAAGGCATTCGTCGAAAAGGACATGAGCCTGCTCGAGGTCAATCCGCTGATCGTCATGACGAACG
GCCGGATGCGCGTCCTCGACGCCAAGGTCTCCTTCGACGGCAATGCGCTCTTCCGCCATGAGGACGTGGTCGC
GCTGCGCGACACGACGGAAGAGGACGACAAGGAAATCGAGGCGTCGAAATACGACCTCGCCTATGTCGCCCTG
GACGGCAATATCGGCTGTATGGTCAACGGCGCCGGTCTTGCCATGGCGACGATGGACATCATCAAGCTCTACG
GCGCCGAGCCCGCGAACTTCCTCGATGTCGGCGGCGGCGCCTCGAAGGAAAAGGTCACCCAGGCCTTCAAGAT
CATCACGGCCGATCCGGCGGTGAAGGGCATCCTCGTCAACATCTTCGGCGGCATCATGAAATGCGATGTCATC
GCCGAGGGCGTGCTCGCAGCGGTCAAGGAAGTCGGCCTCAAGGTGCCGCTCGTCGTGCGCCTGGAAGGCACCA
ATGTCGAGCTCGGCAAGAAGATCATCAATGAATCGGGCCTCAACGTGATTTCCGCCGATGATCTGGACGACGC
CGCCCAGAAGATCGTTGCAGCCGTGAAGGGAGCCTGAATGATACGTAGAGCTTCACTTGTCCTGGTCGGGGCG
CTGATGGGCGCGACGGCGATGGGCGTCGTCTACTCGGCAACCATGCCGGCCGTGGCAGCAAATTCGTCGACAT
ACCGCGAACTGGCGATCTTCGGCGACGTCTTCGAGCGCGTGCGCGCGCAATACGTGACGCCGCCGCAGGACGA
CAAGCTGATCGAGAACGCGATCAACGGCATGCTCACGTCTCTCGACCCGCATTCGAGCTACATGAATTCGACC
GACGCCGAAGATATGCGCACCCAGACGCGCGGCGAGTTCGGCGGCCTCGGCATCGAGGTCACCATGGAGGAAG
ATCTCGTCAAGGTGACCAGTCCGATCGACGACACGCCCGCCGCGCGCGCCGGTGTGCTCGCAGGCGATTTCAT
CTCGAAGATCGACGGTCAGGACGTCCGCGGCCTCAAGCTCGAGGAAGCGGTCGACAAGATGCGCGGTGCCGTC
GGCACGCCGATCAAGCTGACGATCCTGCGCAAGGGAGCCGAAAAGCCGATCGAGCTGACGATCGTACGCGACG
TGATCGCAGTACGCGCGGTGAAGGTCCGTGTCGAAGGAGATGTCGGCTATCTCAGGGTCATCTCGTTTACGGA
GAAGACCTTCGAAGACCTGAAGAAGGGCATCGAGAAGATCCAGGCGGAAGTTCCCGCGGACAAGCTCAAGGGC
TATGTTCTCGACCTGCGCCTCAATCCGGGCGGCCTGCTCGACCAGGCGATCAATGTCTCGGACGCATTCCTGG
AGCGTGGCGAGGTCGTTTCGACCCGCGGCCGCAATCCGGATGAAACCCGCCGCTTCAATGCGACCCCGGGCGA
TCTGGCCGGCGGCAAGCCCGTCGTCGTGCTCGTCAACGGCGGTTCGGCATCCGCGTCGGAAATCGTGGCCGGC
GCTCTCCAGGACCTGAAGCGCGCCACCGTTCTCGGCACGCGCTCCTTCGGCAAGGGTTCGGTGCAGACGATCA
TCCCGCTTGGCGACGCCGGCGCGCTGCGTCTGACCACGGCGCTCTATTACACGCCGTCCGGCAAGTCGATCCA
GGGCACCGGCATCACGCCTGACATCAAGGTGGAGCAGCCGCTGCCGCCCGAGCTTCTGGGCAAGGTGGAAGCG
CAGGGCGAATCCGATCTGCGCGGCCACATTCCGGGCCAGGAAGAGACCGAAGAGGGCTCCGGCTCGGTAGCTT
ACGTGCCGCCGGAAACCAAGGACGACATTCAGCTGAACTATGCGTTCGACCTGTTGCGCGGCAAGAAGACGGA
TCCGTCCTTCCCGGCCAATCCGGAACAGGCGCAGCTGAAGGAATAGATGACCATCGCCGCCGGCGCCTTCGCC
CTCGGCGGCATCCTGCTTTTCGTCGGCCTCGCCGGCGCGGCACTGCTTGGACAGTCGGCCGGCAGCGCCGGCG
GTGGTCCGCTTTTCGATGCCTATATCTGGCGCATCACACGCTTCACGCTGCTGCAGGCCAGCCTCTCGACACT
TCTTGCCATCCTATTCGCCATACCGGTTGCCCGGGCACTTGCCCGCCAGGCTTCGTTCCCCGGCCGCATCTGG
GTGCTGCGGCTTCTGGCGCTGCCACTCGGCCTGCCGGCGCTCGTCGCCGCACTCGGGCTCATCGAAATCTGGG
GCCGGCAGGGGCTCTTGAACAGGGTGCTTTCGGCAGCCGGGCTGGAGCAGCCGGTCAGCATCTATGGCCTCTC

CGGCATTCTCATTGCCCATGTCTTCTTCAACATGCCGCTCGCCGCACGCTTGATGCTTGCCGGCATCGAGCGC
ATTCCGGCCGAATACTGGCGCACCAGCGCCAATCTCGGCATGGGATCCTTCGCCACCTTTCGCTTCATCGAGT
GGCCGGTTATCCGCGGATTGCTTCCCGGCATCGCCGGGCTCATCTTCATGCTCTGCGCCACCAGCTTCACGCT
CGTGCTGACGCTTGGCGGCGGGCCGGCCGCCAGCACGATCGAAGTGGCGATCTATCAGGCGCTGCGCTTCGAC
TTCGACCCGCCGCGGGCGATCGCCCTTTCGGCACTTCAGGTCGCCCTTACCGGAGCGCTGCTTCTCGTCCTGA
AATTGATCGCGCCGGCACCGCTGGAGGGAGAGACCAGCGGCAGGGCCATCCGGCGTTTCGACGGCGCAAGCGG
TCTGTCGCGGCTTGCCGACCGGATCTGGCTGATACTCGCGGTCATCCTCGTCGGCCTGCCATTTGCGGCCATC
GCCTATAGCGGATCGAAGGCCGACCTCGTCCGTCTCGTGAGCGAGCCGGCATTCCATCGGGCGCTCGCCATGA
GCGCGACGATCGCCCTTGTCTCCGCCACGATCTCCGTCGCCATCGCGGCGCTGATGATCCGTACCGAACGGCT
CGCGCTGGCGCAGCGGCGCCCCGGAGCGGCGCCTCGCCTCTTTGCAGGCACGATCGGCGCGAGCACCTCCTTC
ATACTGCTCGTGCCTCCCGTCGTGCTCGGCGCCGGATGGTTCCTGCTCCTGAGACCTTTCGGCGACGTCGCCC
GTTTCGCGCCGGCCGTCGTCGTCGCCATCAATGCGCTGATGGCGCTTCCCTTCGTTCACCGGGTGCTCGCGCC
GGCCATGGCGACGCATGCCGCGCGCACCGCGCGCCTTGCCGCGAGCCTTGGCATCGGCGGCTTTCACCGCCTG
CTGTGGATCGACTGGCCGGGCCTGCGCAAGCCTCTCTTTGTCGCCTTTTCCTTTGCCATGGCCCTCTCGCTCG
GCGATCTGGGAGCAGTCGCTCTTTTCGGCTCCGAGGACATGGTAACCCTCCCCTATCTGCTCTATAGCCGGAT
GGGCAGCTATCGCACCGCGGATGCGGCCGGACTGGCGCTGGTTCTCGGCCTTCTCTGCCTTGTCCTGACGGTG
CTCGGCACGGCGGGAGAGGAGAGGCCGCCCGAAGGTAAAAGCACATGAGTGAAAGAGAATAGTTTCGTATCCG
CCGACGATATTCGCTCGGCCTTTTCCGCTGCGATGTCGTTGATGTACCGCGAGGAAGTGCCGGCCTACGGCAC
GCTGATGGAGCTCGTCGCCAGGGTCAACGGCGAAACGCTCTCGGCCGACGCCACCCTGAAGGGGCGCCTGGAA
GCGACCGATTCGCTCGAACGCATTTCCGAGGAGCGTCACGGCGCGATCCGTCTCGGCACGCCGGCGGAGCTTT
CGATGATGCGCCGGGTATTTGCCGTGATGGGCATGTATCCGGTCGGCTACTACGATCTGTCGACCGCCGGGGT
GCCGGTCCACTCCACGGCCTTCCGGCCGGTGGGCGACGCGGCTCTGAAGCACAACCCCTTCCGCGTCTTCACC
TCGCTTCTGAGGCTCGACCTCATTTCCGACGAGGCCCTTCGCGCCGAGGCGGAGGCGATCCTGAAAGAGCGCC
GGATCTTTACATCCGGTGCGGTCGAGCTTACCGAAAAGGCCGAGCGGGACGGCGGCCTCGACAAGGCCGATGC
CGAGCGTTTCGTCGCCGAAGTGCTCGAGACCTTCCGCTGGCACGACGAGGCGAATGTCAGCGCCGGCATGTAC
GAGCGCCTGCACGATGCGCACCGGCTGATCGCCGACGTGGTCTCCTTCAAGGGGCCGCATATCAACCATTTGA
CGCCACGGACGCTCGACATCGACCGCGTCCAGGCGCTGATGCCGGAATATGGCATTGCCCCGAAGGCCGTCGT
CGAAGGACCGCCGACACGCAAATGCCCGGTCCTGCTTCGCCAGACGTCCTTCAAGGCGCTCGAAGAGCCGGTT
TCCTTCCGCGACTCCGACGGCAGCTGGAAGACCGGCTCGCATACCGCCCGCTTCGGCGAGATCGAGCAGCGCG
GCATCGCTTTGACGCCTAAGGGCCGAGGTCTCTACGACCGGCTGCTCGACGAATCGCGCAAGATCGTACGCCC
GGCCGCCGATGGTTCAAACGCCAGGGAATACGAAGCGGCCTTGGCCCAGGTCTTCGAGGCGTTCCCCGACAGC
TGGGCGGAGCTCCGCGAGGCGGGCCTCGGCTATTTCAGCTACTCCCTGACGGATAAGGGCCGGCGGACGAAGC
TGCCCGGCCGGCGTGATCTCAATTCCCTGATCGCCGACGGCCTCGTTCAATTCGATCCGATCGTCTATGAGGA
CTTCCTTCCCGTCAGCGCCGCCGGTATCTTCCAGTCGAACCTCGGCGACGGTGCGCAGCAGGAATTCGAAGCG
AGCCCGAACCAGAAGCGCTTCGAGACCGATCTCGGCGTCGCCGTGCTCAACGAATTCGATCACTATGCCGGCA
TCGAGCAGGCATCGATCGAAGGCTGCCTCAAGGCGCTTACCGCCGCCATGGCAGCGGAGTGAATGATTGACGA
GCACCATATCGACGCATTGACGAAGCTCCTTGGCGACAAGGGCGTCGTCACGCGTCGGGAGGACATGGAGGCT
TACGAGGCCGGCGCACGCTACGACCGCGGCCGCGCGGCGGCCGTCCTGCGTCCGGTAACGACGGAAGAGGTCT
CGGCCGCCGTCGGTTACTGCGTCCGCAGCGGCATCGCCCTCATCCCCCAGTCGGGCAATACGGGCCTCGTCTC
GGGATCGACGCCGGACGAGTCCGGCAGCGAGGTCGTTGTCAGCCTCGACCGGCTGACCCGCCAATTCGCGCTC
GACCTCGACAATCGCTCCGTGCGCGTCGATGCCGGCTTCCGCCTGTCCGAGCTCAATCGGCGCCTGGAGGAAC
ACGGCCTGTTCTTCCCGATCGATCTCGGCGCCGACCCGCGGATCGGCGGCATGATCGCCACCAATACCGGCGG
CTCGCGCTTCCTCAAATATGGCGACGTGCGCCGCAACACGCTGGGTCTGAAAGTGGTCCTCGCCGACGAGGCG
GGCACGGTGCTCGACCTCGGCTCGGACCTGCGCAAGAACAATACCGGCGTCGACTGGAAGCAGATCTTCATCG
GCACCTCGGGCGCCTTCGGCATCGTCACCGAATGCGTGCTCAATCTCGAGCGCCTGCCGAAGCAGACGGCGAC
CGCCTTCCTCGTGCCGGCGAGCGGCGCGCATGTCCTGCCGCTCCTGAAGGCCATGGAAGAGCGGCTCGGCGCC
TATCTCTCGGCCTTCGAGGGAATGTCGAGGAACGCGATCGCCGCGGCCTTCGCCCATGTGCCGGCGCTGAAAA
ACCCCTTCCAGGGCGGCAAGGTCCCCGACTACGTGATCCTCGCCGAAATCTCCCGCACCTCGAGCCCGCGCGA
AGGCGAGCAGCCGCTCGACGCGGTTCTCGAAAGCGTGCTCGCGGAGATATGGGAGATGGAAGAGGTACTGCTC
GCCGATGCACTCGTCGGCCCGCCGCACGAGATCTGGGCGCTGCGCCATGCGCTTTCGGAGGGCGTAAAACACC
TCGGCAAGCTGATCGCCTTCGATCTCTCCTTCCGCAGGGGTGACATCATGGCCTTCTGCGACCATATGAAGAC
GGAAATGCCGGAAAAATTTCCGGGCGTCACGGTCTGCGATTTCGGCCATATCGGCGACGGCGGCGTTCACTTC
AACCTGGTCGTCGCCAAGGATAGTCCGCTACTGGCCGATGCGACCTTCGAGCAGCGACTGCGCGAATGGGTCT
TTGCGGTGGCGGTCGAGCAATATCACGGCAGCTTCAGCGCGGAACATGCGCTCGGCCGCCGCAACCAGGCTTT
TTACGATCTCTACACGCCGGAAAAACTGAAGAACATGGCGGCCGGCCTCAAGACGCTCACCTCGCCGGGCAAG
CTCGGCAGCGTGCGCTTCGGTTAGATGGAGAGCCAGCCGGATATCGTCATCATCGGATCGGGAGTCGGCGGCG
CAACGGTCGCTGCGGGCCTTGCTGCCTCGGGCGCCGAGATACTGATCCTCGAAGCGGGCTCGCATATCGAGGA
CCTGCCGGTCAATCGCGACCAGCGGGCGATCTTCCAGCGCGGCCATTTCCGCCCGAAGGAGACCTGGTACGAG

GCCGGCGGCCCCGGCTTCAATCCCGGCAATTACTACAATGTCGGCGGCAATTCGAAGTTCTACGGCGCGGTGC
TGACCCGCTATCGCCGGGAGGATTTCGAGGAGATGCAGCATCGGGACGGCGTCTCCCCGGCCTGGCCCTTTTC
CTATGAGGAGCTCGAGCCGTGGTATTCCAGGGCAGAGCAGCTTTACCAAGTGCGAGGCAAATTGGGCGAGGAT
CCGACGGAGCCCGCTCATTCGAGCGGCTATCCCCATGGCCCGGTGCCGGACGAGCCTGCGATCGCCATGGTCC
GCGAGCGCCTGGCCGAGATCGGCCTCCATCCCTATTCGCTGCCGCTCGGCGTCGATATCGAGCGCTGGCTCGC
CAAGGGCAAGACGCCGTGGGACGCGCATCCGAACTCGTCCGACGGCAAGATGGATGCCGAAACGGCGGCGCTG
GCGGCGGCGCTGAAATTTCCGAATGTGCGGCTGCAGACCGGCTCGCGAGTGACCCGGCTCGTAACCGGGCCGG
ACGGCAAGGCGATCGAAACCGTGCTCTACGCCAGGGACGGGGCAGAGCACAGTCTTTCGCCGAAGCTCGTCAT
CCTCTGCGCCGGCGCGGTGCAGTCGGCCGTACTGCTCCTGCGCTCGGCCGACGACAGCAATCCGACCGGGCTT
GCCAACCGGTCCGATCAGGTCGGCCGCAATTTCATGAACCACAATTCGAGCGCCGTCATCGCGCTCAGCCCCT
GGTACCGGAACGACTCGGTCTATCAGAAGACCTTCGGCCTCAACGACTTCTATCTCTCCGACGGTGCGGGCGG
GCCGCCGCTCGGCAATGTCCAGCTTCTCGGCCGCATTTCCGGCGCGATACTGAAGGCGAACATGCCGGCAATG
CCGGAATGGCTGCTAAACCGGGTGTCCGCCCGCGGCATCGACTTCTACGCCATGAGCGAGGACCTGCCCTCAC
CGGAAAGCCGCGTCATGGTCGACGGCGAGCGCGTTGTGCTGAAATGGGTGCGCACCAATTGGCAGGCCCATCT
CGACCTCGTCGCCAGGCTCAAGGCGGTGCTGAAGAAAGCCGGCTTCCCGATCGTGCTCGCACGGGCCTTCGAC
AAGCGGACGCCCTCGCACCAGTGCGGCACGGTCCGGATCGGCAATGATCCCGGCCAGGCGCCGCTCGACGTCT
ATTGCCGCGCCTTCGATCATCCGAATCTCTTCGTCGTCGATGCCGGCTTCCTGCCGACCTCCGCCGCCGTCAA
TCCGGCGCTGACCGTCGCGGCACAGGCGCTGCGTGTCGCCGACCATATCGTGAAACAGGATCTGCGGCCATGA
ATGAGCGAGGTCAAGTCGCACGAATTCGATAGTTTCCTGCAGCGATCTGCGGCGATCTACCGGCTATTCCTGC
TCTACGGTCCCGATCGCGGCCTCGTCTCGGAACGCGCGTCCGAGCTCGCCGGCAGCTTCGGCATCCCCCTCGA
TGATCCCTTCGCCGTCGTCAAGCTGGACGCAGCTACCTTGCAGGGCGCCGGTAGCGTGCTTGACGAGGTCAAT
GCCATCGGCCTGTTCGGCGGCGACAAGCTGGTCTGGGTGCGCGGCGCGTCCGCTGAGAAGGCGCTCACCGAAG
CGCTGCAGATCCTCGCGGCCGATCCCCCGGCCGGCGCCCGTCTCATCGTCGAAGCCGGCGACCTCAAGAAGGG
GGCGGCGCTCAGGAAGGTTGGCGAGACTTCCCGCTCCGTCGCTTCGATCGCCTGCTACAGCGACGACATCAGG
GGCCTGCAGGCACTTGTCGACACGGAACTTTCGGAAGCGGGCCTGCGCATCGGTCCCGCCGCGCGCGAACGCC
TCGTCGAGGCGCTCGGGGGCGACCGCATGGCTTCGCGCAACGAGCTTCGCAAACTCGCCCTCTATTGCCGCGG
CAAGGATATGGTCGATGAGGATGACGTCCTCGGTATCGTCGGCGACGCCAGCGCCATTTCGGTCGACGATGCG
GTCGATGCGGTGTTGAAGGGCGACGCCGACGCGCTGCTGCATGCGATGAAGAAGATCACGACATCCAAGACGC
CGCCTTTCCTGGTGCTTCAGGCCTGCCTCAGGCAATTCCAGCAGCTCGACGTGATGCGAGCCGAAATGGATGC
GAGCCGCCAGTCTGCGGGCCAGGTCGTCGCAAGCCTCGGCCGCGGCCTGCACTTCCGCCGCAAGCCGGTTGTC
GAGGCGGCGCTGAAACACTGGACCGGGCCGGCGATTCGACGCGAATTGGGCCGCCTGCAGGCGACGATCTATC
AAAGCCGCAGCCGCCAGAGCCTCGAGGAAAGCCTGGTGATCCAGAATCTCCTGGCGATCACCATCCAGTCGGC
GAGGCGTTGAATGCCGCCCGACACCGCGCTTGAAAGCTTGCTGCGACGGGATCGGGCAATCGTGGCAGCGTCG
CTCGTCGCATTGACGGTGGTCGCCTGGACATACACGCTCTGGCTGGCGGCAGCCATGAACATCGACGGCATGA
GCATGTCTGCCCCCGGCATGGAAGCGGACATGAAGATGGACCCTGCCATGGATATGGATGGCATGGAGATGGG
ATCCCGTGGCGCCCTGTCGCTCGGCACCGTCCTCGGCATCTCGCCGCGTCCCTGGAGTTCCGTGGAAGCCGGC
GTCACCCTGACCATGTGGATCGTGATGATGGTCGGCATGATGCTGCCTTCGGCAACGCCGATGATCCTGCTCT
ACGCCCGGGTCGGCCGGCAAAGCCGGATAGCGGGCAAGCCCTTCGCCGCGACCGGTTTCTTCGCCGGCGGCTA
TCTCCTTGCCTGGGCGGGATTCGCGCTCGTCGCGACGCTTGGACAATGGCTCATGGAGGGAACCCTGCTGACG
CCGGCGCTGGCAAGCGCCAGTCGCATCTTCAGCGGTGTCGTGCTGGTGATCGCGGGTCTCTACCAATGGACGT
CGCTCAAGGATGCCTGCCTCAGCCAATGTCAGACGCCGATCGTCTTCCTGCAGCGGCACGGCGGTTTTCGCCG
CGATCCCGCGGGAGCCGTCGGACTGGGCCTGCGCCACGGGGTCTATTGCATCGGCTGCTGCTGGGCGCTGATG
ACGCTTCTTTTCGTCGGCGGCATCATGAATGTGCTCTGGATTGCGGCAATCGCCATCTTCGTGCTCGCGGAGA
AGGTGATACCGACCGGCCGCGTTCTCTCACGCGTCGCCGGATCTCTGCTCGCGGCGTTCGGCCTTTGGCAGCT
CATTTCGGCTTCGATTTAAATGACTGCGAAGAAGACGATCAGCGGAGCGCGGATTTTCGACGGCATCGACTGG
CACGACGGGGCAGCCCTCGTGATCGAGGCAGGACACGTCAAGGCGATTGCGCCGGCGGGAAGCGTCCCCGCCG
GTGGCGAGACCATCGACGCGCGTGGCCTGCTTCTCGTGCCCGGCTTCATCGATCTGCAGGTGAACGGCGGCGG
CGGCGCGCTTCTGAACGAAAAGCCGTCCCTCGAAAGTATCCGGCAGATCTGCGCGGCGCATGCGCAATTCGGC
ACGACGGCGCTGCTGCCGACGCTGATCACCGACACGCGCGCGGTCAGGACCGCGGCCATCGCCGCAGGCATCG
AGGCGAAAGCCGCGGCGGTTCCCGGCTTCCTAGGCCTGCATCTCGAAGGCCCGCATCTTTCCGTCGCGCGCAA
GGGGGCTCACGATCCGGCGCTGATCCGTCCGATGGGCGATGACGATCTTGCCGAGATACTCGCCTGCGCAAAG
GCGCTCAGCCGTCTGATGCTCACCGTGGCCCCGGAAAACGCCACCAAGGAGCAGGTGCGGGCGCTGGCCGATG
CCGGCGTCGTGGTGAGCCTCGGCCATACCGACGTGGATTACGACACCGCCTGCGCCTATGCCAAAGCGGGTGC
CCGGACCGTCACGCATCTCTTCAACGCCATGAGCGGACTCGGCCATCGCGAGCCTGGCGTGGTCGGCGCGGCG
CTTTCGACCGGCACGCTCCACGCCGGCATGATCGCCGACGGTTTTCACGTTCACCCGGCCTCGATGGGCATCG
CGCTGCGCGGCAAGAAAGGCCCGGGACAGATCTTTCTGGTCACCGACGCCATGTCGCCGATCGGCACCGATCA
GACGAGCTTCTTCCTAAACGGACGGGAAATCCTGCGCCAGGGCGGGCGCCTGACGCTTGCCGACGGCACGCTT
GCCGGCGCCGATATCGACATGCTTTCGTCTGTCCGCTTCGTCCACCAAAAGCTTGGCCTGCCGATCGAGGAGG

CGGTCCGCATGGCCTCCGCCTATCCCGCCGACGCCATGGGGATCGCCTCGCACAAGGGCCGGCTCCTGCCGGG
TACGGATGCGGATTTCGTGCTGCTCACGCCGGAACTCGCAATGAAATCGACCTGGATCGGCGGGGAGACGGTT
TTTGCGGCTTGAATGAGCGACGAAGCGCCCACTTCATCCCCGATTTTACCCGTCGAGAAACCGGCCGCCTACC
GTGTTCTGGCGCGCAAATACCGCCCCAAGGACTTCTCCGACCTGATGGTCGGCCAGGAGCCGATGGTGCGCAC
GCTCACCAACGCCTTCGAAACCGGCCGCATCGCCCAGGCCTACATGCTGACCGGCGTTCGCGGCGTCGGCAAG
ACGACGACGGCGCGTATCCTGGCACGGGCGCTGAACTACAAGACCGCCGAGATCGACAAGCCCACCATCGACC
TGCGCGTCCCCGGCGAACATTGCCAGGCGATCATGGACGGCCGCCATGTCGACGTGATCGAGATGGACGCCGC
CTCGCATACCGGGATCGACGATATCCGCGAGATCATCGAGCAGGTACGCTATCGGCCGGTCTCCGCGCGCTAC
AAGGTCTATATCATCGACGAAGTGCACATGCTCTCGACGCAGGCCTTCAATGGCCTGCTGAAGACGCTCGAAG
AGCCGCCGGAGCATGTGAAGTTCATTTTCGCCACGACCGAAATCCGCAAGGTTCCGATTACCGTCCTGTCGCG
CTGCCAGCGCTTCGATCTCCGGCGCATCAGCGCTTCCGATCTGGTCGGCCTCTTCTCCACCATTCTCGGCAAG
GAGGGCGTGCCCTTCGATCCGGAGGCGCTGGCGATGGTGGCGCGGGCAGCGGAAGGCTCGGCGCGCGACGGCC
TGTCGCTGCTCGATCAGGCGATCGCCCATGGCGGCGGTTCGGTCGAGATCGAAACCGTGCGCTCGATGCTCGG
CCTTGCCGATCGGGCGCGGATCGTCGACCTGTTCGAGCATGTCATCAAGGGCGATGTCGCCGGCGCGCTCGAC
GAGTTCGCCGCGCAATACGAAGCCGGCGCCAATCCGACCGTGGTCCTCACCGACCTTGCCGACTTCACCCACC
TCGTGACAAGGCTGAAATATGTGCCGGACGCGGTCAACGACCAGTCGCTGAGCGAGATCGAGCGCACGCGCGG
GGCCGAATTCGCCGGCAGCGTTGCCGTGACGACGCTTTCGCGCGTCTGGCAGATGTTGCTCAAGGGCATTCCC
GAGGCGGAGAGCTCTGCGCGCCCCGCAGGTGCTGCCGAGATGGTGCTGATCAGGCTCGCCCATGCCGCCCATC
TGCCTTCGCCCGAAGAGGCCGCGCGGCGGCTTCTCGATCTTTCGGGCGGCGAAGGCGGCGGTCGCCCGGCCCC
GAACGGCGGTGGTGGCGGCGGCGGTGCGCAGGCGCCTGCAGGCACCCCCGTCGAGGCTCGCGCCGTGGAGACG
GCAGTCTCGCGTCCAAGCGGTAATGGCGCGACCATGCTGCGCGCCGTGCCGAGCTCCGCGCCGCCCCAGCCGA
TCAGTGTCGGCCGCATCGAAGAGCGCACGGTCGCGAGCGCCGCTGCGAAACCCGAACCGAAGGTTCCCGTCAA
TTCCATCGGCGACATCGCTGATCTCTGCGCCAAGAATCGCGACATCAAGCTGAAGACGATGGTGCGCGGTTTC
CTGAGGCTCGTGCATATCGAACCCGGCCGGCTCGACGTCAATCTGCCCGACGACGCACCGAAGACGCTGCTGA
ACGAACTCGCCGTCAAGCTCAAGGAATGGACGGGTATCCATTGGGTCGTGAGCTACAGTCGGGAGCAGGGCGA
GCCGACCCTTGTCGAGGCAGAGCAGCGCGCGCAGGAGCAGCGCGTCAACGATGCGCGCCAGGACCCGGACGTC
GCTGCCATTCTTGCGCGCTTTCCCGGGGCCAGGATCACCGACGTGCGCATCCGCGCGGCGGAAGAGGAGCTCG
AGAGCCTCGCTCCGGCCGCGGCCGAATCCGAGGACGGCGACATCGTCCCGGACGACGACATCGAGTAGATGAC
CATTACCGAGAGCAGGAATGCGGCCGTTGCGGAGATCGAGGAGGTAATCGGGGACTGGGCGGAGGCGCTGCGC
CGGAAGGATGCCGCCCGCGTCCTCGCCCACGGCACGGAAGATTGCCTCGTCTTTTCGCTGGCGCCCCCCCTCA
AGGCTTCCGACGCAGATGCCGGCGGCCTTGAGCAGTGGTTTTCCACGTGGAAAGGGCCGCTCGGCTTCACCCT
CGAGGAGCTCGACGTTTCGGTGAGCGGCGACGTCGCCTTCGCCACTGCGCTGACGCATCTTTCCGGCGAGAAG
CACGATGGCCAGAAGGCGGCGCTCTGGTATCGCCTGACCTTGGGCCTGACACGCACCGACAGGGGATGGAAGA
TCGCCCATCAGCACGAATCCGTCCCCTTCTACATGGACGGAAGCTTCAAGGCGGCGATCGATCTCGATCCCCG
GTCCGAGGTCGACTGGCAAGCGCCGTCGCGCCCGCCCATGCCCGGCGTCATCGCCTATCTCAACGTGGAGGAT
GCAGATGCGGCGGCGGCCTTCTATGTCCGCGCTTTCGGCGCCAAGGAGGAGGACCGCAAACATGCCCAGGATG
GAAAGCGCCTCATCCACTGCCACCTGACGATCAATGGCGGCGCATTGATGCTGAACGATCCTTTCCCGGAATT
CGGCCATCCCTGGAAGCCGCCGGAGGGCTTCGTGCTGCACCTCGTCGTCGACGACGCTGATTTCTGGTGGAAT
CGGGCGGTGGAGGCCGGCGCAGAGGTGACGATGCCGCTGGAGATCGCATTCTGGGGCGACCATTACGGCCAGC
TCCGGGATCCCTTTGGAGTTACCTGGGGGATCGTCGCACCGGTGAAGGAAGGCGGCTGAATGTTCAAGTTCCA
AGCCAGATCGCTGGCGACAAAGTTGATCGCGGTGACGGGAGGCACGATCGCCCTCGTGCTGCTCGCGTCGAAT
TACGTGCTCATCTCCCAGACGCAGGACCGTGTGGAAACGCTGGTCTTCGACGGCGCGAAGACGGAAGCGCGGG
CGATCGCGTCCGACATCGCGGGAAGCGTCGGCGAACTTGCCGCCGCCGCGCGGACCATGTCGGGCGTTCTGGG
GCGCGGCCATGCCGGGCAGTCCACCGACCGCGCAGGCGCCATCAATCTCCTGAAGGCCAATCTGGAGCAGCAT
GGCTTTGCCTTCGGCAGCTGGTTCGCCGAAGAGCCCAAGGCCTATGACGGAAAGGACGTCATCGACAACACGG
AACGTGGCGGCAATGCCGACGGTGCGTTCACGCCCTATTGGTCCAAGGACCGCAACGGAAATATCCAGCTCTC
GACCTTCAAGGCCGATTATGCGGCCGAGTGGTACGGCCTCGCCGCGAAGAGCGGCAAGGGCGCCATCACCCAG
CCATATCTCGCCGAGGGCACCGACGTCCCGACCACGATGACGTCGATCGCCTACCCCGTCATGTCGAACGGCA
GGATGATCGGCGTCTCGGGTGTCGATATCTCGCTCGCCGCACTCGCCGACCGTCTCTCGGCGGTCAAGCCTTT
CGGCTCCGGCCGGGTCTACCTGCTGTCGCAGAGCGGCAAGTGGCTCGCCGCACCTATCCCCGAACTGCTGATG
AAGGAATACGACGGCGAAGGCGTTGAGTCCGTGAAGGATGCCCTTTCCACCGGCACGCCCCGCATGATCGAGA
ACCTGACTTACGACGGCAACGAGCCTTTCGACCGAGTCGTCTATCCTTTCAGCCTGCCCGACGTGAACGCGCA
GTGGCTGGTTCTGGTCGATGTTCCGCGCTCAGCCATCAATGCGCCGGTTCGCGATCAGACCTATATGATGATC
GTCGGCGGCCTGGTGGTCCTCGGCGCCGTGCTCGCCGGCCTCTATTTCGCCGTTCGCCGCTTCGTCCAGAAGC
CGCTTGCCGGACTCGTGGGCGACGTCGAGACGCTCAGCAACGGAGAATATACCCGGCCGATTTCCGGTCAGGA
ACGCTCCGACGAGACAGGCGCCGTCGCCAGGTCGCTCGAGGGCTTCCGTCACCAGCTTGCCGACAACAGGCGG
CTCGAAAGCGAGGCCCGCCACGAGAGAGAACAGGCCGAACTGGAACGCGGACGTTCGGAAAACGAACGCACCG
AGGCCAGCGCGCTGCAGCGCGACATCGTCGCACGCCTTGGCAAGGCCCTGTCGCATCTCTCCGCCGGTGACCT

CGCCTTCCGCCTTACCGGCGACTTCCCCGGCGAATACGCGCAGCTGAAGCGCGATTTCAACGCGACGATGGAG
AGCCTCGAAGAGACGATCCGCACCGTCAACCACTCCGTCGTCAATATCGGCAGTGGCACCAGCGAGATCTCGG
GCGCCGCCAACGACCTGTCGCATCGGACTGAACAGCAGGCAGCAAGCCTCGAGGAGACCGCGGCCGCCCTTGA
CGAACTGACCTCGCAAGTGAACGCCAGCGCCGAGAACGCCAAGGTCGCCGCGAAGTCCGTCGAGGTCGCAAGC
AGCGACGCGGAACAGTCCGGCGAGGTGGTGCAGAAGGCGATCGCCGCCATGCAGGGTATCGAGCAGTCGTCGC
ATGAAGTCAGCCGGATCATCGGCGTCATCGATGAAATCGCCTTTCAGACCAACCTTCTGGCGCTCAATGCCGG
GGTCGAAGCCGCCCGCGCCGGCGACGCCGGCAAGGGCTTCGCGGTCGTCGCACAGGAAGTCCGCGAACTCGCG
CAGCGCTCCGCCAATGCCGCAAAGGAAATCAAGACGCTGATCAATACATCGGCGGGCCAGGTTCGCGAGGGCG
TCGACCTCGTCGGCAAGGCCGGCGGCGCGCTCGAGAAGATCGCCGAGCAGGTGGTGCAGATCAACGGGCTCAT
CCGCCAGATCTCCAGCTCCGCCTCGGAACAGGCGGTCGGCCTCAAGGAGATCAATTCCGCCGTCAATCAGATG
GACCAGGTGACACAGCAGAACGCCGCCATGGTGGAAGAGACGACTGCCGCCAGCATGGCGCTGAACGACGAGG
CCCGCGCCCTCAGCGCGCTGGTCGCCCGTTTCCAGATCGCCCCGCAGGCTGCCCAGGCCCAAGCCTCGGCCGA
AATGCTGCGCGGTACGGCCGAGCGGATGCGTGCGGCTGCTCCCGCGGAAAATCGTCCGGCACAGGCGCCGCGC
AGTGCCGCCTACTCCAATTCGACTCAAAGAGTGCTTGCCAAGACATCCGGCGCCAATGCGCTCGCGCAGGACA
ATTGGGAAGAATTTTGAATGACAGCAGATACGACCATAGATCCCGCGGCGCCAAGAACCTTCCTGTTCTTGCA
GGGACCGGCGTCGCCGGTCTTCGCCAAGGTCGCCGGGCATCTCGAAGTGCTGGGTCATTGCTGTCTGCGCATC
AACCTCAATCCCGGCGACCAGATATTCTGGCGGCGCCGGGGCGGCCATAACTACCGTGGCTCGTTGGCGCGGT
GGCCGGCCTATGTGGAGGCCTTCATCCGCCGCCACGCCGTCACCGACCTCGTCCTGCTCGGCGAAGAGCGCTC
CTATCACCGGATCGCCACAGCTGCCGCGCGAAAAGCCGGCGTCCGAGTCTTTGTGGTCGAAATGGGTTATCTC
AGGCCGGACTGGTTGACGCTCGAGCGCGGCGGCATGTCGTCCAATTCGCACTTTCCGGTCGATCCCGCGCAAA
TCCTGCGAGCGGCAACGGGATTGCCCGAGCCCGACTGGCAACGGCGCTACCGGCAGAGCTTTCTTGCGGAGGC
GGCATACGACCTCCTCTACAACCTGCCGAACGTGTTTTTCTGCTTCCTCTTTCCCGGCTACCGCCGGCACGGC
ATCTTTCATCCGCTCGCCGAATATGCCGGGTGGATCCGGCGGCTGGCGGCCGGCAAAGGCGAGCAGCGGCGCG
CGGATGCGTTGATCCGCTCGCTTTCGGCGAAAAATGCGCCGTACTTCGTCTATCCGCTTCAACTTGAAACGGA
CTATCAATTGCGTGCGCATTCACCCTTCGGCAGCCAGCGGGAAGCGATCGACGGCATCCTTGCCTCTTTCGCG
CGCCGTGCGCCGGCAGGAACCAGACTCGCCGTCAAGGTGCACCCGCTCGACAACAGCCTGATCTCCTGGCGCA
GGATCGTCGCGGACAAGGCCGCCTCACTCGGTGTCGGCGACAGGGTCCTCTATCTCGACGGCGGCAATCTCGA
TTCGCTCACGGAGCACAGTTGCGGAATGGTAACGGTCAACTCGACCGCCGGACTGCATGCTCTGAAGCAGGGC
AAGCCCGTGAAGGTCCTGGGAAAGGCCGTCTTCGACATTGCCGGTCTCACCGACCAGCAGCCGCTCGACGCGT
TCTGGACGGCGCCCGAGGCCCCGGATCCGGTGCTGAGCGGGGCAATGTTCAGGCTGATGGCCGCTTCGATCCA
GGTGCGCGGGAACTTCTATTCCCGTGCGGGTACGGATGCCGGCGCCAAGGCGATCGCCGAGCGGCTGCACCGC
AACGACGTCAATGAACCGGGCGCCTTCGTCGACCCGCCACCCCGGCAAAAGCCGGTGAGACTCAGCGAAGATC
AGGCGACGCCGGCGCGCAGCAGATCGTGATTGCAGACCGGTCTTTATGTCGCGCTTTCGTCGCAGATGGCGCT
TGAAAAGCGCCTGAACACGCTTGCCGACAATATTGCCAACTCCAACACGGTCGGCTTCCGCGCGACGGAAGTG
AAGTTCAACCAGGTGCTGGGCGACACCAAGCCGACAAAGGTCTCCTATGTGTCGGAGGGCGAGGAATTCCTGA
GCACCAAAACCGGCGCGCTCGCGCGCACCGGCTCCGCACTCGATTTCGCCATCAAGGGTGACGCCTGGTTCTC
GATCGACACGCCCGGCGGCCCGGCGCTGACGCGCGACGGCCGGTTCACCCTGACGGAGACCGGCGAGCTCGTA
ACCATCAAGGGATATCCGGTGCTCGACGCCGGCGGCGCGCCGATCCAGCTGAACGGCGGTGCCGGTGAAATTG
CCGTCGGTGCCGATGGTGCCATTCACCAGAACGGCGTCCAGATCGCCCTGCTCGGGCTTTACGAGGCGGATTT
CAGCAAAGGCTTCATGCGTTACGACAACAGTTCCGTTATGCCGGCCGCCCAGCCGGAACCGGTCGTTGACCGG
TTCGATGTCGGCGTGATACAGGGCTTCCTGGAGGAATCGAACGTGAACGCCATACAGGAAATGTCGCAGCTGA
TCATGATCACCCGCGCCTTCGACAACGTCACCGCACTTATGCGCGACAGTGAGGGATCGCTCGATAAGGCCAT
CGAGACGCTCGGCGGCAGCCGTTGAATGCCGGAGACGATCGGCTTTCTGAAGCCAAGGCAGGCCGTCCTCGAT
CGTCGGCGGGAAATCGTCGCCGATCTTGCCGATCTGTTGCCGGAAGGCGGCCTCATCAGCGACGAGCGCGGCC
TCAAGCCCTTCGAGACGGATGCGTTTATCGCCTATCGGCGGATGCCGCTCGCGGTCGTTCTTCCCGAGACGAC
GGAGCACGTGGCCGCGGTGCTCAAATATTGCAGCCGCTACGGCATTCCGATCGTGCCGCGGGGCGCCGGCACC
TCGCTCTCCGGCGGCGCCATTCCTCAGGAGGACGCGATCGTCGTCGGTCTGTCGAAGATGTCGCGCACCCTGG
ATATCGACCTGTTCAACCGCACGGCGACGGTTCAGGCGGGCGTGACCAATCTCAATATTTCCGATGCGGTTTC
GGCCGACGGCTTCTTCTATGCCCCGGATCCGAGTTCGCAGCTCGCCTGCACCATAGGCGGGAACATCGGGATG
AATTCCGGCGGCGCGCACTGTCTGAAGTACGGGGTTACGACCAACAACCTTCTGGGCGTCAAGATGGTGCTTT
TCGACGGCACCGTGATCGAACTCGGCGGCAAGGCGCTCGACGCGCCGGGATACGACCTGCTTGGGCTCGTCTG
CGGTTCGGAGGGGCAGCTCGGCATCGTCACGGAAGCGACGGTGCGGCTGATTGCGAAGCCCGAAGGTGCGCGG
CCCGTGCTCTTCGGATTTGCCTCTTCCGAGTCCGCCGGCAGCTGCGTCGCCGATATCATTGGATCGGGCATCA
TCCCCGTCGCAATCGAATTCATGGACAGGCCGGCGATCGAGATCTGCGAGGCCTTTGCCCAGGCCGGCTATCC
GCTCGACGTCGAAGCATTGCTGATCGTCGAAGTGGAAGGCTCCGAGGCTGAGATGGATGCGACGCTCGCCGGC
ATCATCGAGATCGCCCGCCGCCACGGCGTGATGACGATCAGGGAATCGCAATCGGCTCTGGAAGCGGCACTGA
TCTGGAAGGGGCGTAAGTCGGCCTTCGGCGCAACCGGCCGGATTGCCGACTATATCTGCATGGACGGAACGGT
GCCGCTGAGCCAGCTCTCCCATGTCTTGCGCCGGACCGGGGAGATCGTCGCCGGCTACGGCCTGCGGGTAGCG

AATGTCTTCCATGCCGGCGATGGCAACATGCATCCGCTGATCCTCTACAACATCAACGATCCGGAGGAAGCTG
CCCGCGCGGAAGCTGCCGGCAACGACATACTGAAGCTTTGCGTCGAGGCGGGCGGCTGCCTGACCGGGGAACA
CGGCGTCGGCATCGAGAAACGCGATCTGATGCTGCACCAGTACAGCAGGGCCGATCTCGGCCAGCAGATGGCG
GCGCGCGCCGCCTTCGATCCGCAATGGCTTATGAACCCGTCGAAGGTTTTTCCGCTCGAAGGACGGCCCGCGG
CATGAATGAAAGAGAGACTTATATCTTCCGTTGCCGCTGGCGCGCTTCTCGTGGGCGTTGCCGTTGCCCCGAT
GAGCTTCGCACAGCAAACCACGACGCCGCCGGCTACGGCACCTGAAACCCAGACCATGGAACCGGCTCCGGCT
CCGGCCCCGGCTACACCGCCGGCCGCCGACGAGCAGGCGCAGACCCCGGATACGGCAGACAAGACGACCGATA
TGGCGGCTGGTGACGCCACCTACCTGACCGAGCAGGCGGAGAATCAAGTTTCCGCCAACACCTATATCGGCCA
GTCCGTCTACAATGCCAGCGATGAAAGCATCGGCGAGATCAACGACCTGATCATCGAGAAGGAAGGCGGTATC
GCTGCAGCTGTCGTCGGAGTTGGCGGCTTCCTCGGTATCGGGGAAAAGAACGTCGCAGTTCCGTTCGACAAGA
TCGCAGTCGCCGACCAGCCCGACTCGGATGACATCAAGCTGACGACCACCGAAGCGGCCGACTCTCTGAAGGC
CGCACCGGAGTTCAAGACGAAATCGCAGCTGATGGCCGAGCGTAACGCCGACCAGCCGGTCGATACGTCGACG
ACTTCCGCAACGGGTACAACCCCGGCACCGGCGCCTGCGGAACCTGCCCCTCAGCAGTAAATGACCCTTCCGA
CAGAAATGACCTACGTCGATCTGCCTGGCCCCGGAGGGCCGGAGAATATGGTGCTGGCGCGAGGGCCGCTGCC
TGAGGTCCGGCCCGGCGACATTCTGATCCGCGTCGAAGCTTTCGGCATCAATCGGCCGGACGTCCTGCAGCGC
AAGGGAGACTACCCGCTGCCCGCTGGGGCGAGCCCCATCCTCGGGCTCGAAGCTGCGGGCGAAGTGGTTGCTC
TCGGCGAGGGAGCGACCGGCTTCTCGACCGGCGACAAGGTCTGCGCTCTTGCCAATGGCGGCGCTTACGCGGA
ATATTGCGCGGTTCCCGCCACGCAGGCCCTTCGCTGGCCCAAGGGCTACGATGCCGCGAAGGCGGCAGCCCTG
CCGGAGACCTTCTTCACCGTCTGGGCCAACGTCTTCGACATGGCCCGCCTGAAGTCCGGCGAAACATTCCTCG
TCCATGGAGGATCGAGCGGTATCGGTACGACCGCGATCCAGCTTGCGAGGGCCTTCGGTGCGGAGGTCTTCGC
GACCGCCGGCAACGCTGCGAAGTGCGAGGCCTGCGAAGCACTCGGGGCGAAACGTGCGATCAATTATCGCGAG
GAGGATTTCAAGGCTGTCGTGCTGCAAGAGACCGGCGGCCGCGGGGTCGACGTCATTCTCGACATGGTGGGCG
GGCGCTACTTCGATCGCAACATTGGCAGCCTCGCCAAGGACGGGCGCCTGTCGATCATCGCTTTCCTTGGCGG
CGCCCGGGTGGACGGCGCCAACATTGCGCCAATTCTCATGAAGCGCCTGCAGGTAATGGGCTCGCTGCTCCGC
CCGCGCACCGCCGCCGAGAAGCAGGCGATCCGCGACGGGCTTGCGGAAAAGGTCTGGCCGCTGCTGGAGGGGG
GCGACGTTGCACCGGTCATCCAGGCGGTCCTGCCCTTCGCCGAGATTGCCGGCGCCCATCGGCTGATGGAGGA
GGGCGATCATATCGGCAAGATCGTGATGCGGATCGGGTAGATGACCGAGCAACAAGAACTCCCCGAACGCGAG
AGCATGGAATTCGACGTCGTGATCGTCGGGGCGGGACCGGCGGGGCTTGCCGCGGCGATCCGGCTGAAGCAGG
TCAGTCCGGAACTCTCGGTCGTGGTGCTGGAGAAGGGCGCCGAAGTGGGCGCACATATCCTCTCCGGCGCCGT
GGTCGACCCGATCGGCATCGACCGGCTCTTGCCGGGCTGGCGCGACGAACCGGACCACCCGTTCAAGACGGAA
GTCACCGACGATCAGTTCCTCTTTCTCGGCCCCGCAGGCTCTGTCCGCCTTCCCAATTTCCTGATGCCGCCGC
TGATGAACAATCACGGCAACTACGTGGTATCGCTGGGCAATGTCTGTCGCTGGCTCGCCACCCAGGCGGAAGC
GCTCGGCGTCGAGATCTATCCGGGTTTTGCCGCAACGGAAGTGCTCTACAATGACGAGGGCGCGGTGATCGGC
GTTGCCACCGGCGACATGGGCATCGAGCGGAACGGCGAGCCGGGGCCGAACTTCGCCCGCGGCATGGAGCTGC
TCGGCAAGTACACCCTCATCGGCGAGGGCGTGCGTGGCTCGCTCGCCAAGCAGCTGATTGCCAAATACAAGCT
CGACGCGGGCTGCGACGTGCCGAAATTCGGCATCGGCCTGAAGGAGCTCTGGGAGGTGAAACCGGAAAATCAT
AAGCCGGGCCTCGTGCAGCATTCCTTCGGCTGGCCGCTCGGCATGAAGACCGGCGGCGGCTCCTTCCTCTATC
ACCTTGAGGACAATCTGGTGGCCGTCGGTTTCGTCGTCCACCTCAACTACAAGAACCCGTATCTCTATCCGTT
CGAGGAATTCCAGCGCTTCAAGACGCATCCGGCGATCCGCGGCACTTTCGAGGGTGGAAAAAGGCTCTCCTAC
GGCGCCCGCGCCATCACCGAAGGCGGCTACCAGTCGGTGCCGAAGCTGTCTTTCCCCGGCGGGGCGCTGATCG
GCTGCTCGGCCGGCTTCGTCAACGTGCCGCGCATCAAGGGCAGCCACAATGCGGTGCTGTCCGGCATTCTCGC
CGCGGAGAAGCTTGCGGCGGCGATCGCCGACGGCCGCGCCAATGACGAGCCGATCGAGATCGAGCGCGGCTGG
CGCGAGAGCGCTATCGGCCAGGACCTGAAGCGGGTCCGGAACGTCAAGCCGCTGTGGTCGAAATTCGGCACCG
CCGTTGGGGTGGCGCTCGGCGGTCTCGACATGTGGACCAACCAGCTCTTCGGCTTTTCCTTCTTCGGGACGCT
GAAGCACGGCAAGACCGATGCGCAATCGCTCGAGCCGGCCGCCAAGCACGAGAGGATCGACTATCCGAAACCG
GACGGGGTGCTGACCTTCGACCGGCTCTCCTCGGTGTTCCTGTCGAACACCAATCACGAGGAGGACCAGCCGG
TGCATCTGCAGGTGAAGGACTGGGACCTGCAGAAGCGCTCGGAATACGAGGTCTATGCCGGCCCATCTTCGCG
CTACTGTCCGGCCGGCGTCTATGAATGGGTGGAGAAGGACGGCCAGCAGACCTTCGTCATCAACGCCCAGAAC
TGCGTCCACTGCAAGACCTGCGACATCAAGGACCCGAACCAGAACATCAACTGGGTGCCGCCACAAGGGGGCG
AAGGGCCGGTCTATCCGAACATGTGAATGAGAACGATGATTGCGATCGCCTGTGCGGCAGGAATGGCGGGTGC
CGCTTCGGCACAACAGTCCTCGCAGACGGCGACGGCCGAGTTCGTGGGCAAGGACGGCACGGACATCGGCCGG
GCCACTTTGACGGCAGGCGGCAAGGGCGTGCTGATCGAGATGGAGATAAGCGGCCTGCCGAAGGATACCTGGG
TGGCTTTTCACGCTCACGAAATGGGGCGCTGCGATCCCAAGGACGGGTTCGAGTCGGCGGGAAAGCATTTTGC
CGGCGAAGATGAGAATGCCGAGCACGGCTTCCTCGCTTCCAATGGCCCGCATGCCGGCGACATGCCGAACCAG
TATGTCGGTGCCGATGGAACCTTGCGCGCCCAGGTCTTCAACAGCTTCGTGTCCCTCGACGACAGGGCTACCG
CCCTTCGCGGCCGGGCCCTGGTGATCCATGTGCATTCGGACGACAATCGCAGCCAGCCCTCCGGCGATGCCGG
CGACCGGCTGGCCTGCGCGGTGGTGAAGTAGATGGCCGCCCCCGTCACCGTGCGCGTCCAGCGCGACGACTTC
GACATCGCCGCCGAAGTCTCGGCGCTTTGCCGTGCGCGGACTGACATCGGTGCGGTGGTCACGTTTTCGGGGC

TGTGCCGGGACGAGGCCGGAGCCCTCAGTGCGCTCGAGCTCGAGCATTATCCCGGCATGGCAGAGGCGGAGAT
CGAACGGATATGCCACGAAGCGGTCGAGCGCTTCGGCCTGCAGGCTGCAACCGCCATCCATCGCTTCGGCCGC
ATGGAGCCGGGAGCGAATATCGTCCTGGTGGTAACCGCCTCGCCGCACCGGCAGGCGGCATTCGATGGCGCGA
ATTTCATCATGGATTTCCTGAAGACCTCGGCCCCCTTCTGGAAGAAGGAACATCACGCCGACGGCAGCGCAGG
CGATTGGGTCAGCGCGAAGGATGCGGACGACGCGGCACGCGATCGCTGGACGCGCCGCTGAATGGTCGAATCT
GAAGCGCAGGGACGTCTGCCCGGTATCCGCCCGGTCTCCGTTGTGGATATCGGCTCCAACTCCATTCGTCTCG
TCGTCTATGAAGGGCTGAGCCGTTCGCCGGCGATGCTTTTCAACGAGAAGGTGATGTGCGGCCTCGGCAAGGG
CATCGATGCGACCGGCCGAATGGAGGAGGAAAGCGTGGCGAGGGCGCTGAGGGCGCTGCACCGCTTTCGTGCG
CTCTCCGACCAGGCGCGCGCGACCGAGATGTATGTGCTCGCGACCGCTGCGGCGCGGGAGGCCACCAACGGAG
CGGCCTTCATCGAAAGGGCGGAAGCCATCCTCGGGCAAAGGGTGAAGATCCTGAGCGGTGAGGAGGAAGCCTA
TTATTCGGCGATGGGCATCGTCAGCAGCTACCACGAGCCGGATGGCGTCGTCGGCGATCTCGGCGGCGGCTCG
CTCGAGCTTGTCGATGTAAACGGCAAGCGGATCGGCAAAGGCATAACGCTGCCGCTGGGCGGCATTCGCCTCT
TCGAACATTCGAGCGGCTCGCTCAGCAAGGCGCGGACCTGGGTACGCAAGTTCATGAAAAATGCCGAGGTCCT
GAAGAAGGGGACCGGACGCACGTTCTACGCCGTGGGCGGAACCTGGCGAGCGATCGCCAAGCTGCACATGGAG
ATGCACAATTACCCGCTGCACATGATGCAGGGTTACGAAATCTCCTATGACGAAGCGCTGGCGATCCTCGCCG
AAGTGATCGAGCCGAAGAATATCAAGCCCTCCGCCTATGCGACGATTTCCAAGAGCCGCCGGAGCCTGCTGCC
CTACGGGGCGGTGACGATGCAGGAGGCAATCTCCATCATGCAACCCGAGCGCATCTCCTTTTCCGCGCTGGGC
GTCCGCGAGGGCTATCTCTTTTCGCTGCTTTCCGAGGAGGAGCGCCGGCAGGACCCGCTGCTGACCGCTGCCA
ACGAACTCGCCATCCTTCGCGCCCGCTCGCCCGAGCATGCGCGCGAGCTCGCCGAATGGACCGGCCGCCTGGT
GCCGTTCTTCGGCGTCGAGGAGACGGAAGAGGAGAGGCGCTACCGTCAGGCCGCCTGTTTGTTGGCGGATATC
AGCTGGCGGGCGCATCCCGACTATCGCGGTCTGCAGGCGCTGAACATCATTGCGCACTCGGCCTTCACGGGTA
TCACCCATGCGGGGCGGGCCTATATCGCGCTCGCGAATTACTATCGCTTCGAAGGGCTCAACGACGACGGCGC
CACGAGCCCGCTCGCCGGCATCACGACGCCGCGCCTGCACGAACTGGCCAAGCTCCTGGGCGGGCTGTTGCGC
GTCGCCTATCTCTTTTCGGCCTCCATGCCGGGTGTGGCGCGCCACCTCACGCTCCGGCGATCTTCGAAGCCCG
ACATCGATCTCGAATTCGTCGTCCCGGCAGCCTATGCCGACTTCGAGGGCGAACGCCTCGACGGCCGGCTCCA
GCAGCTCGGCAAGCTTACCGGCAAGAAGCTCGCCTTCTCCTTCGAGCGCTGAATGATACATACGCGCAAAGTC
CGCCTGATGCTGGCCAGCGCCGCCCTCCTTACCCTCGCCGGACCGGCCATGGCTCTGGACGGCGCGGACATGA
TGCAAAAGCTCAATGCAGCGACGTCGCCCAACGGCACTTCGGTGACCTACGACAAGGCGGAGGCCGACGGCGA
CGTGGTGACGGTCACCGGCATGAAGCTGCAGGTGGTGGCGAAGCCCGGCACGTCCATCGACATAGGCGAAGTG
ACGTTCGAAGGCGTCGAGGAAAAAGGCGGCGGCTACTATGCCGAGACCGTCTCCTTCCCGGATATCGCTGTCA
GCGACAAGGATGCAAGCTTCTCGGTCAAGGACATCTCGATCGGCGGCCTGTCCATTCCGGGCAATGCGAGCGG
TACCACGATCGATGACATCCTGCTTTATGAAACGGCCGGCACCGGCCCCATGACCGTAAACGTCAAAGGCAAG
GACGTCTTCACCGCCGAGAGTACGGAAACCAATCTTACGCGGCAGGAAAACGATGCGGGCTTCGACTTCGACG
CGACGCTTGCCGGAATCAAGGCGGATCTGTCGCAGATCGAGGATGCCAAGGCAAAGGAGGCGGTCGAGAAGCT
TGGGCTGACGACGCTCGACGGCGGAGTCACGATGAAGGGCAGCTGGGAATTGCAAACCGGCAACCTTGCTCTC
GACGAATACGCGCTGGACTTCAAGAATGTCGGCCGACTGAATATCGCGATGGACTTATCCGGCTATACGCTGC
AATTCATGAAGTCGTTGCAGGAGGCAGCCAAAGCGGCTGAGGCAAACCCGAACAGGAAGGAAGCCGACCAGGC
GATGGCGCTGGCGATGCTCGGCCTCATGCAGCAGCTTACCTTCAACAGCGCCTCGATCCGCTTCGACGATGCA
TCGATCACGAAAAAGGCGCTCGACTACGCCGGCAGCCAGCAGGGCGTCACCGGCGAACAGCTGGCGCAATCGC
TGAAGGGGCTCGTGCCGATCATGATGGCGCAGCTCAATCTCCCGGAACTGCAGAACCAGGTCTCCAGCGCCGT
CAACGCCTTTCTCGACAATCCGAAGAGCCTGACGATCAGCGCTGAGCCGGAAAAACCCGTGCCGTTCCCGATG
ATCATGGGTGCTGCGATGGGGGCTCCCAACACCGTCCCGTCCGTGCTGGGCGTCAAGGTGACGGCGAACGACT
GAATGAACAGCATTGCGAAAATCTCCGTCATCGCCACCCTCTCCGCCCTTACCTTCGCCGGAATCTCCCAAGC
GGCGAGCGTCGAAATGAACTGGCCGGACCGGATCGACCAGACCGTGCGCACCTTCAAGGGCGACAATTTCCGC
GTTGTCGATGTCGATACGTTGAGCCGCCAGAACCAGACCCGTATGCGAGTAGACAAGGCAACACCGGGGCAGC
GTGCCGCATTGCAAGCCGCTGTCGAAGCCAACAGACCGCTGCTCGCTTCGCTTAGGGCACGCAATGTCGAACT
GGACAACGTCGCCGCGGCGAAGCAGGCGGCCGATGGCGGACTGACGATCTACCTCCGCTGAATGATCCGTTCT
GTCGTGCGCGGCTTCGGGGCAGCACTGCCGAAGCGAGTGATGACCAATAAGGAAATCGAATCCAGGGTCGACA
CGTCCGACGAATGGATCGTGCAGCGGACCGGCATCCGCCAGCGCTATATCGCAGGCGAGGGCGAGACGAGCGC
CTCTCTCGGCGAGGCGGCGGCGCGGGCGGCGCTCGAGAGAGCCGGGCTGACGCCGGACGACGTCGACCTGATC
ATCGTCGCGACCTCCACTCCGGACAACACCTTCCCTGCAACCGCAGTCAACATCCAGAACCGCCTCGGCATGC
GGCACGGTGCGGCATTCGACATGCAGGCGGTCTGTTCCGGCTTCGTCTACGCGGTGGCGACGGCCGACGCCTA
TATTCGCGGCGGCCTCTCCAAACGCGCTCTTGTCATCGGCGCAGAGACCTTCTCGCGGCTTCTCGACTGGACC
GACCGGACGACGTGTGTGCTGTTCGGCGACGGCGCCGGCGCAATCGTTCTGGAGGCGCAGGAGGCGGCCGGGA
CCAAGGCTGATCGGGGCGTTCTGACGGCGCAATTGCGCTCCGACGGCGCCCACCGCGACAAGCTCTATGTCGA
CGGCGGGCCGTCGACGACCGGCACCGTCGGCCATCTCCGGATGGAAGGGCGCGAAGTCTTCAAGCACGCGGTC
GGCATGATCACCGACGTGATCGAGGCGGCTTTCGAGGCAACCGGAACGACCGCGGACGACATCGATTGGCTGG
TTCCGCACCAGGCCAATCGCCGCATTATTGACGGCTCGGCCAAGAAGCTGGGCATCCCTCTCGAAAAGGTTGT

GGTTACCGTCGACCTGCATGGCAACACCTCCGCGGCATCGATTCCGCTTGCCCTCGATGCCGCCGCCTCTGAC
GGCCGCATCAAGAAGGGTGATCTCGTAATGCTCGAAGCGATGGGCGGGGGCTTCACCTGGGGCTCCGTTCTTC
TTCGCTGGTAAATGACCACGAGTCGCGAGCCGCCCGACGCCCCTCTGCGCCTGCTCGAGGTACTGGAACCCGG
CGGCGGAGGGTCCGGACGCCACTTTCTCGATCTTTGCCGCGGCATGCATGCGCGCGGCCACCATGTCGAAGCG
ATCTATTCTCCCGTTCGGGCCGAAGACGGCTTCGTGCGCGAACTCAAGGCGCTCGGCCTTCCCGCCATTCATG
CGGTCGGCATGCGCCGGGCGCCCGGCCCCTCCGACTGGTCCTGCTTACGCGCCATAAATCGCATCATCCGCAC
TGCCGGGCCGTTCGACGTCATTCACGGCCACAGTTCCAAGGCCGGAGCCCTGACGCGCCTGCGCCTCCCGGGA
AGGCACGTGCCGCGCGTCTACACGCCGCATGCCTTCAGGACGATGGACCCGACGCTCGGGCGCGGCGGCCGAC
TGATTTACGGGGCGATCGAAACACTTCTCGCCCGCTTCTTCACCGACCATCTCGTCACGGTCTCCGGTGACGA
ATTCGCCCATGCGCTGTCGATCGGCATTTCGGGAAAGGGCATGTCCGTTATCGTCAACGGCGTCGACACCCCT
TCGCCCGATATGGCGCAGACGGTGCGCGCCAGCTTCGGCATTCCGGCGGACGCCTTCGTTTTCGGCTTCATCG
GCCGGCTCTCGGCACAAAAGGCGCCCGAGCGTCTCCTCAATGCCTTCGGCAAGGCCGCGTCGGCGGTCAGGAA
CAGTCACGTCGTCATGATCGGATCGGGAGAATTGGAGGAAGAGGTCCGCGCCGCGATCGCCGCAAGCGGCTTG
CAGAACCGCATCCATCTGACCTCGGCCTTTACGGGACCTCAGGCGGTACCCGCATTCGATCTCCTCGTCATGC
CGAGCCGCTACGAGGCAATGTCCTATGTGATGCTCGAAGCGGCAGCCGCCGGCCGGCCGATCATCATTTCCGA
CGTCGGCGGAGCCGGAACGGCCGTCGATCACGGAGAGAACGGCTACATCGTTCCGAACAGCGAGGACGTCTCA
CCGCTTGCAAGAGCGATGATAGCGGCGGCCGACCCGGATATGTTTCGGCACCTTGCCGGCGCCGCCGCGGCGC
GCCGGGACCGTTTTACACTGAAACGTATGCTCGACGAGACGGAAGAGGTTTACCGACGAATGGCAGCGCAGCG
CCGCCGACAGCAGTCAGTAAGATCGAATCAAGAATACTGGAAACCGACGGAAATGATGAAAGCCGCTCAATCA
CTGCATGGGGGCTAAATGAACCACGACGCCTACCCGGACTATTATCTTGCCGACATCCTGCGCGAGACGAAGC
CCATCGCGCTCGTCGGCGCCTCGCCGAAGCCGGAACGGCCGAGCTATCGCGTCATGGCCTTCCTTCTCCGCAA
GGGATACCGCGTCATTCCGGTCAATCCCGGCCATGCGGGCGGCACGATCCTCGACCAGCCGGTGGTAGCGCGC
CTCGCGGATATAGGGGAGCCGATCGACATGGTGGACGTCTTCCGTGCGGCTTCGGCCTTGCCCGCGCTCGTCG
ACGAGATCCTGGCGCTGCCGGCCCTGCCCAAGGTCATCTGGGGACAACTGTCCGTCCGCGACGACGCAGCTGC
CGCCAAGGCTGAGGCCGCAGGCATCAAGGTCGTCATGGACCGTTGCCCGGCAATCGAATATCCGCGACTGGTC
GGATGAATGTCCATCGCCCCCGGTATCACCATCGACGGCCCCGCCCGGCGCGTCTCCCTTGACGTCCGCAACC
CCCGTTTCTTCCGCAATCCGCTGCCGGCCTACGCGGCGCTCCATGCGCAATGTCCGGCCTTTTTCTGGGAAGA
GCCGCAGCAGTGGTTCTTTGCGGGCTACGAGCAGGTGAACAGCCTGTTGCGCGACCGTCGCTTCGGCCGACAG
ATCCTGCATGTCGCGACCCGGGAAGAACTCGGCATGCCGGAGCCGAAACCGCATCTGAAAGATTTCGATGCAC
TCGAGGCGCACTCGCTGCTCGAACTGGAGCCTCCGGCCCATACGCGGCTGCGCACGCTCGTCAACCGCGCCTT
CGTCTCCCGTCAGATCGAAGAGCTCAGGCCGGAAATCGAGGCGCTCTCGCATGCGGTGATCGACGGCTTCGAG
AAGGATGGCGAGACCGAGCTGTTGAAGACCTATGCCGAGACGATCCCGGTAACCATCATCGCGCGGATGCTCG
GCATTCCGGTCGAAGCGGCGCCGCGTCTGCTCGACTGGTCGCACCGCATGGTGAAGATGTACGTGTTCAATCC
CAGCCTGGAAACGGAGTTCGACGCCAACAATGCATCGGCGGAGTTCGCCGACTACCTGAAAGGGATCATAGCC
GAGAAGCGGACAAACCCCGCCGACGACCTGCTCACGCATATGATCACCTCGGAGAAAGACGGCGAGCGCCTGT
CGGACGCGGAGCTGATTTCGACCACGGTCCTCCTTCTGAATGCGGGCCACGAGGCGACGGTTCATCAGATCGG
CAATGCCGTGCGCACGATCCTGCAGTCCGGCCTCTCGCCCGCCGAACTTTTCTCGGACGAGAAGGCGACGGAG
CGCACCGTGGAGGAGTGTCTGCGCTTCGCCGCGCCCTTGCACATCTTCCAGCGCTACGCCCTCATGGATATCG
AGCTGGAGAACGGCATCGCCCTCAGGAAAGGGGACAAGATCGGCCTGATGCTGGGGGCGGCGAACGTCGATCC
GCGCAAATTCTCCTCTCCCGATACGTTCAGGCCGGACCGCAACGAAGGCGCCAATGTTTCGTTCGGCGCCGGC
CTGCACTTCTGCATCGGCGCACCGCTGGCACGGCTGGAGTTGCAGATTTCGCTGCCGATCCTGTTCCGACGGC
TTCCCGGGATGCGGCTCAAGAACGAGCCGCCGGTCAAGGACGCCTTCCACTTTCATGGTCTGGAGCGGCTCGA
TCTGGTCTGGTGAATGAACCATATCCTCCTCGTCGGTGCCGGCGGCGCCCTCGGCTCCGTCCTGCGCTACCTC
GTCGGCCTCTGGATGCTGCAGCGCGCCGGTCCCGCCTTTCCCTGGGGGACGCTGTTCGTGAACGTCACTGGCT
CCTTTCTGATCGGTTTTCTGGCGGAGTTCATCATGCACAAGATGGGTGCCTCGCCGGAAATGCGCGTGTTCCT
GATCACCGGCGTTCTGGGGGGCTACACGACGTTCTCGGCCTTTTCGCTCGACGCGATCGCCCTTCTCGAACAC
GGGCAGACGATGAGTGGCCTCGCCTATATCGTGGCGAGCGTCGGTCTTTCGATGCTTGCCGTGTTCGCCGGGC
TGGCCCTGATGCGCGCAATGGTCTAAATGAATGCCTTTCGTGCCAAGCCGCTGAAGTTTCCATGGCCGCCTCT
GCTCTATGGCGCGGCCGTCCTGATCGCGCTGGCCCTGGGCCACCTTTTCCCGATTCCGGTGGCGCACGAGCAC
AGTTGGATCCTCTCGCTGGTGGGCTACGGCCTCGTGGTGCTGGCGATCACCATCGATCTCTGGGCCGTCAAAA
CCCTCCTTGACCGCCATACAGCCGTACTTCCGAACCGGTGCGCCACCTGCCTCGTCACCTGCGGCCCATTTCG
TTTCACCCGCAACCCGATCTATTTCAGCTACACGCTGGCGATGACCGGCCTCGGATTGGCGACGATCAACCCC
TGGTTCTTCATCACGGCAATTGCCGCGGTCGGATTGACCACCCTTTTCGCGATCCGAAAGGAGGAGCGGCACC
TGCTGTCGCGCTTCGGTTTCGAGTTCGAGCGATATTGCCGGCACACTAGCCGTTGGATTTGAATGTCCGAACA
CAGCGCGACGACGACACCTGAAGCGCAAATGCGCACGGAGGCGACCGGAACCAACCGGATGGTTCTCCTGGAT
GCGCTGCGCGGCCTGGCACTCGTCGCCATGGCCCTCTATCACTTTGTCTGGGACCTCGAATTCTTCGGCTATG
TCGCGGCCGGCACCGCCGGGACCGGCGGCTGGCGGATGTTTGCACGCTTGATTGCCAGCAGCTTCCTGTTTCT
TGCCGGTTACAGCCTCGTGCTCGGCCAGCTCCCGAAACTCCGTCCGCGCGCCTTCGCCATACGTTTCGCGAAG

ATCGCCGGCGCAGCCGCGCTGATCAGCATCGGGACCTGGTTCGCCTTCCCTCAGTCGTTCATCTTCTTCGGCA
TTCTCCACGCCATTGCGGCGGCAAGCCTTGTCGGCCTCCTGTTCCTGAAATTGCCGGCCGCCGTTTCCTTTCT
TGCCGCTGCCGTGGCCTTCGCGGCTCCCCTCTATCTGCGCTCGTCGTTTTTCGACATGCCGGCGCTCTGGTGG
GTCGGCCTCTCGGAAACGATCCCGCGCTCGAATGACTACGTTCCTCTGCTTCCCTGGATTGCCCCTTTCCTGC
TCGGCCTCGGCACCGCGAAGCTGCTCCACCCGTTATTTTCCGCCCGCCGCAGCCGAAGCGCCGGGACCCGTAA
GCACCCGTGGATGACGCCTCTCGCCTTTGGCGGCCGTCACAGCCTTCTGATATATCTCATCCACCAGCCGCTG
CTTATCGCGGCAGTCTATCTCTTCTCTCTCGCCGTTCCCGCCCCGGCACCGGATCCGGCACGGGTCTATCTGC
TGAACTGCGAACGGGCCTGCAGCAACGAAAATTCCGGGATCTCCTGCAGCGCCTTTTGCGGTTGCACGCTCGC
CGGCCTGAAGGAACAGAACCTGTTCGACGATCTGAACCTGGGCAAGATCGACGTGAACACGGATGAACGCATT
GCGCGAATCGCCGGTCAGTGCACAATGGATGCACAATCAGGAGAGGCACAGTCAGGGGACTAGATGAAAGCCG
CCCTGATCGTCATGACCATTCTCGGTTGCGACGACAACGTTACCCAATGCCATTACATCTCGACCGTGAACGG
CGACTGGCCGTCGATCGCGCAGTGCGACACGGAATCGCAAAAGGAACTGCCGCGGTTCTCCGACAGGAACTAC
CCCGTCGTGGTGGCCGTGTGCGAGAGCTCAGGGCCGGGCATGACGGCGGAAAGCGAGCCGGTTCCGGAGCTGA
ATGCAACGCTTCCGGCTCAGCCGGGCGTGCAGCAAAACACAACATCAACGGCAGAAGGCGGAGAACCAACCTT
GCCGAGGCGCGCCCTGGCATTGATCAGCGGCGCCGTACCCGACCGGGAGAAACTGAAGGCGATCGTCAGTGCG
CCGGTCCACTACGTCGAAGACGGCTATTCCTGGGTGGCGCGCCGATTCGGCAATTGAATGGCCTCTCTCTTCG
ACCCGATCACGATCGGCGATATCAGCCTCAGCAACCGCGTGGTGATGGCACCTCTGACCCGGAACCGTTCACC
CCAGGCAGTGCCCAATGATCTTAACGTCGCCTATTACGAGCAGCGCGCATCCGCCGGCCTGCTGATCACCGAA
GGGACGGCAATAAGCCACCAGGGCCAGGGCTATGCCGACGTGCCCGGGCTTTACAAACCGGAGGCCCTCGCCG
GCTGGCGCAAGGTCACGGATTCGGTCCACAAAGCCGGTGGCAAGATCGTCGTGCAGATGTGGCATGTCGGGCG
GATATCCCACGACTCGCTCCAGCCGCAGGGCGGCAAACCGGTCGCCCCTTCGGCTATCCGCGCCAAATCGAAG
ACCTACCTCGTCAATCCCGATGGGACGGGCAGCTTTGCCGAAACCTCCGAGCCGAGGGCGCTCGAACCGGCGG
AGATCGCCGGCATCGTCGAGGATTACCGCAAGGCCTCTCGGGCCGCGATCGAAGCCGGATTCGACGGCGTCGA
GATCCATGCTGCCAACGGCTATCTGATCGACCAGTTCCTGCGCTCCGGCTCCAATGAACGCACGGACGCCTAT
GGCGGCTCGATCGAGAACCGCGCCCGCTTTCTCTTCGAGGTGGTCGATGCCGTTACCAAGGAGATCGGCGCTG
GCCGGGTCGGGATCCGCATCTCTCCCGTGACCCCCGCCAACGACGCCTTCGATCCGGACCCGCAGCCGCTCTT
CACCTATGTCGTCGAAGGCCTCGCCCGATATCCGCTCGCCTATGTGCACGTCATCGAAGGCGCCACCGGCGGA
GCGCGTGACCACCAGCAGGGCGATCGCCCCTTCGACTACACAAGCCTCCGTGCCGCCTACGCAGCGGCCGGCG
GCAGGGCGGCCTGGATGACGAACAACGGATACGATCGCGGTCTGGCGCTGAAGACGGTCGAAAGCGGCGAAGC
CGATTTGGTTGCTTTCGGCAAACCCTTCATCGCCAATCCGGATCTCGTTCGTCGTTTGAGAGACAACGTGGCC
CTGAATGCACCGGACCAGGCGACCTTCTATGGCGGTGGCGCCAAGGGTTATACGGATTATCCGTCGCTCGAAA
ACGTCGCCTGAATGCGAGGCCGAGCCCTCAAGCTTTCAGCGTCTCGGCGCCTGGTCGGGGATCTCATGCGATT
TTCGATGAAGGTGCCGCGGGTGAGCGTGCAGCGGCGGATGAATCTCGGGCCGTTGCTCGAGGCCAGAACATCG
CTTGAAAGCAGGCCATCCTGGACGGCGCTCTTCCTAAAAGGATACGGCCTTCTCGCTCAGGAGACGCCCGAGT
TGCGGCGCGCTTACGTCAAATTTCCCAGACCGCAGCTATACGAATATCCGGGAAGCATCGCTTCCATCGCGCA
TGAAAGGGAGTACGAGGGCGAACGAATCGTGCTGCTGACGACGATCAAGAATCCGGAGCGCCGTCCGATCGCG
GAGCTGGGGTCCTTGATGCAGACGGCGCGAACCAGTCCCGTGCTGGAGGTCAAGGAATTCCGGCGCGCCCTGA
AGATCGCCCGCCTCCCCGGTCCGATCAGATGGCTTCTCATGTGGCTCGGATTGAACATCGGCCGGCAAAGGGC
GCGCCGTTTCGGTACATTTCAGCTGTCGGTCTATTCGGGCCTCGGCGCGGAATCGCTCAACCCCCTCACGCCG
TTAACCTCGCTTCTCAATTACGGCCCGATCGACAACGATGGCTCGGTCACCGTGCGCATCCACTATGACCACC
GGGTCATGGATGGCGCCAACGTCGCGCGTGCCCTCGAGCGGTTCGAGAGGATATTGAACGGCGAAGTCACCGC
GGAGCTGGAAGGGCTTGCATCCGGGCGCGACGTAACCGCTGCGGCAGGTTCCGGAGCGGCGCGATGAATGACC
AGAAAACGTGCGAACTTGATCGTCGCTGCAATGCTTTGTTCGGCGACGGTCGTCACCTCCTCTGTCAATGCGC
AGGATCAGGCCGCACCACCGCTTGTGACGCAGATCGGCAACGGCAACTGGCTTCCGCAGGAGGAAGCGGAGTC
GCTGCGCGACGAACTCTACTATCAACGCGCGATCCATGCCTACATAACCATGCTTCCGGCGCTGAACGTGATC
GGTATGCGCGACGGTTCGGAGGCGACATTCGGTAAAGGCTACAACATCCTGCCAATCTGGAAGGACCGGATGG
ACTCGCGCACGTGGGTGCCGACACCGAACGCCGACGTAATCTACTCGATGAGCTATCTCGACCTGAAGGAAAC
CGGCCCGCTCGTCGTCGCCGCGCCACCCAACGTCATCGGCATGTTTACCGATTTCTTCCAGCGTACGATCACG
GATGTCGGACTGATTGGACCGGATCGCGCGCGCGGCGGCCTCTACCTCCTGCTGCCGCCTGATTATGACGGCG
AGGTTCCGCAGGGCTACTTCGCGCTCAAAGCGAAGACCTACAATGTCTTTCTGTTCTTCCGGACGATCATGGC
GAAGGGCGAAAACGGACCGGACCCCAAGCCCGCCGTGGCGCTCGCGGAGCAGACGCGCGTCTATCCGCTTTGG
ACGCCCGAGAAAGACGTCAAGCCGATGCAGTTTCCTAACGGCAGCGGCAAGCGCATCAACATGATGTATCCGG
TCGATAACGCCTACTGGACCAAGTTGAAGGAGTTCGTCGACCACGAGCCGGTCTCGGCGATCGATTCCGAACT
GCGCGGTGTGCTGGCATCGATCGGCATCATCAAGGGCCAGCCCTTCGAGCCGACCGGGAAGCAGCAGGAACTC
TTGAAAAAGGCGGTCGAGACAGCGCCCAAGATGATCTTGGCGCAGCGCCAGCTCGGCCGACCGGACGGACGCA
ATCGCTACTACACCGACAGGCAGTATGAAAATGTCTGGGCCAGCGGCACATCCGAATGGCTCCAGGACAGTTA
TCTCGATGTCGACCAGCGGGCTGGCTATTTCCAGGTTGCCTATTCATCGGCTCCGGCAATGGTCATGCGGACG
CTGGGGGCAGGTTCCAAATACCCGGTTGCATACCGAGATGCCGCGGGCGAGTTCCTCAGCGGCTCCAGCACCT

ACAGGCTGCATTTGCCACCCAATCCGCCGGCTGCCCTGTTCTGGGCGGTTACCGCCTACAACATCACCGACGG
CACGATGGTGGAAGCGCCGCAGCTGATGCCGTCCATCAACGGCTTCAACAAGGTCGCGACGAACCAGGACGGC
TCGGTGGACCTGTGGTTCGGACCGGAAAAACCTGCCGACGCGCAGGACTCGAACTGGATCCAGACCGTCGGCG
GCCGGAATTTCCTGATAGCGCTGCGTCTCTACGGAACCGGCGTCGAGTTCTTCGATCAGACCTGGAAGCCGGA
TGATGTGGTCATGGTGAAGTGAATGACTGCTTTGCATCCCATTTCGCGCCGGACCCTCATCGGCGGTCTTGCC
GCCACGTCGGCATTTGTCGTGCTGCATCCCTTCTCGACCCGCGCGGCTGCCAACCAGGCGCATCTCAGGATCA
TGGAAACCACGGACCTGCACGTCCACGTCTTTCCCTACGATTACTATGCCGACAAGCCGAACGACACGCTTGG
CCTAGCGCGCACCGCTTCGATCGTCGACGCCATCCGCGCCGAGGCGGCGAACGCGGTCCTCGTCGACAATGGC
GATTTTCTTCAGGGCAATCCGATGGGCGATTACATCGCCTACAAGCGCGGCATGAAGGAAGGCGACATGCATC
CGGTCATCGCCGCGATGAACGTGCTCGGCTACGACTGCGGCACGCTCGGCAATCACGAGTTCAACTACGGCCT
GGATTTCATGTTCAATGTGATCAACGGCGCAAACTTTCCGATCGTCTGCGCCAACCTGACCAAAGGCGCCATG
GCCGCCGCCCCGCGCCAGGACGAGCTTTTCCTCAAGCCCTACGTAATCCTCGATCGAAAAGTGAAGGACGGCG
CCGGTCAGGAACACCCCATCCGCATCGGCCTCATCGGGTTCGTACCGCCGCAGATCATGACCTGGGACGCCAA
GAACCTCGAAGGCAAGGCGAATGCGCGCGACATCGTCAAGGCCGCGGAAGCCTGGGTGCCGCAGATGCGCGAG
GAAGGCGCCGACATCGTCATCGCCCTCTCACATTCCGGCATAGGGCAGCAGGCCTATGCCGAAAATCTCGAAA
ACGCGTCCGTACCCCTGGCGGCAATCGAGGGTATCGACGCCATCGTCACCGGCCACAGCCACCTCGACTTTCC
GGGACCCAAGTTCGACGGAACTCCGGGCGTCGACAATGCCAAAGGGTTGATTTCAGGAAAGCCCGGCGTCATG
GGCGGCTTCTGGGGATCCCATCTCGGCCTCATCGATCTGCTGCTCGAACGCGACGGCGGCCAGTGGCGTGTGA
TCGGTTCGACAAGCGAGGCGCGGCCGATCTTTCGCCGGGAAGAAAAGAAGGTGATCGCCGAGGTCGGTGACAA
GCCGGAAGTGCTCGCAGCCGCGCAGAAGGACCACGAAGCGACGCTTGCCTATGTGCGCACCCCCGTCGGCAAA
ACTTCGGCACCGCTATACTCCTATTTCGCGCTGGTTGCGGACGATCCGTCGGTGCAGATCGTGAGCCAGGCCC
AGACGTGGTATATCCGCGAAATGCTCAAGGACACCGAGCACAGCGACCTGCCGGTCCTGTCCGCCGCAGCCCC
GTTCAAGGCCGGCGGGCGCGGTGGCGCCGAGTACTATACGGATGTTCCAGCGGGCGACATCGCCATCAAGAAT
GTCGCCGACCTCTACCTCTATCCGAATACGGTGCAGGCGGTCGTCATCACCGGCGAAGAGGTGCGCAACTGGC
TCGAAATGTCCGCCGGCATCTTCAACCGGATTGCACCGGGATCGGTCGATGCAAGCCCGATCAATGGCGACTT
CCCTTCCTATAATTTCGACGTGATCGACGGGGTCACCTACCAGATCGTTCTTTCGCAGCCTGCGAAATACGAC
AAGGACGGCAATGTGGCGAACCCGGAGGCCAGCCGCATCCGTGATCTGAAATTCGATGGCAGGCCGATTGATC
CCAAGCAGAGATTTGTCGTAGCGACCAACAACTACCGCGCCGGCGGCGGCGGCAATTTTCCGGGCATCGCCGG
CGACAAGGTCGTCTTCGTCGCGCCGGACACCAACCGCGACGTCATCGTGCGCTACATCGTCGAACAGGGAACG
ATCAACCCCTCGGCGGACGCGAACTGGACCTTCGCGCCGCAGAAGGACACGAGCGTGCTGTTCGACAGCGGCC
CGAGGGCGCGACAATTCCTGGCAGAGGTCAAGGCGGTGAAAATCGAGAATGCCGGCGACGGCAGCGACGGTTT
CGCCCGGTTCAGAATCAAGCTCTGAATGCTCCTGGCAGCCAGCAGTTTTGCCACTGGTGCTCTGGCCGCGCCC
GTGCATGCGATCGCCATGCATGGCGACCCCGCCTTGCCTGCCGATTTCAAGCACTTCCCTTACGTCAATCCGC
AGGTGAAGAAGGGCGGGAAGATCGCCTACGGGGTCGTCGGTACGTTCGACAGCCTCAATCCGTTCATCTTGAA
GAGCATGCGCACGACCGCGCGCGGCATGTGGGACCCTGGCTTTGGCAACCTGGTCTATGAATCCCTCATGCAG
CGCTCACAGGACGAGCCCTTCACGATGTACGGCCTGCTCGCCGAAACCGTCGAATGGGACGATGACCGCACCT
TCATCCAGTTCAACCTCAATCCCAAGGCGCGCTGGGCCGATGGCCGGCCGGTGACCGCCGAAGACGTGATCTT
CACCTTCGAATTGTTGCGCGACAAAGGGCGTGCGCCTTTCAGCAACCGCCTCTCGAAGGTTGCGAAGATGGAG
AAGGTCGGGGAACGTAGCGTGCGCTTCACCTTCACCGAGGAGGCCGATCGCGAGATTCCGCTTCTGCTGGGGC
TTTCGCCCGTGCTGCCGAAGCATGCGATCAACGTCGAGACGTTCGATCGGACGAGTCTGGAGCCGCCGCTCGG
CTCCGGCCCCTACCGGGTCGCGGAAGTCAGGCCTGGCGAGCGGATCGTCTATCGCCGAAACCCCGACTATTGG
GCCAAGGATCTGCCCTCGAAGGTGGGCCTCGACAATTACGATGAGATCTCCGTCGAATATTTCCTGCAGGAGA
ACACCCTTTTTGAAGCCTTCAAGAAGGGTGTGGTGGACATCTACGCCGAAGGCAGCGCGACGAAATGGGCCCG
GGCCTACGATTTTCCCGCCGTTCGCAGCGGCAATGTGATCAAGGAAACGTTCAAGCCGAAGACACCGTCCGGC
ATGCTCGGCTTCGTCTTCAATACGCGCAGACCGATGTTCGACAACATCAAGCTCAGGCAGGGGCTCGCTCTCG
TTTTCGACTTCGAATGGGTCAACAAGAATCTCTTCGACGGCGCCTATACGCGTACGCAGAGTTATTGGCAGAA
TTCGTCGCTTTCCTTCCTCGGCGTGGCAGCCGACAATCGTGAACTGGACCTCATGGGCGACGTGAGGGAGCGC
ATCAATCCGGCGATCCTCGACGGAAGCTATCGACTCCCCGTGACGGACGGCTCGGGCCGCGATCGCAATGTGC
TCCGGGAAGCGGTGACCCTTCTTCGCGAAGCGGGCTACTCCATCAAGGACGGCAAGATGGTCGACGCCAAGGG
GACGCCGCTGGCCTTCGAGATCATGAGCCAGAATGCCGGCCAGGAGAAGGTTGCGCTTGCCTATCAGCGCTTT
CTCGCGCCGCTCGGCATCGCAGCGCGGGTTCGGACCGTCGACGATTCACAATATCAGTTGCGCAGCCAGTCCT
TCGATTACGACGTCATCATCAAGTCCTTCCCCTCGTCGGTCTCTCCCGGTCTCGAGCAGATCAACCGCTGGAG
TTCGCAAACTCGGGACAGGCAAGGCAGCGACAACTTCGCAGGCGTCGCCGACAAGGACGTCGACAAGCTGATC
AACAACATCCTGCAGGCGCGCAACCCCGAGGACTTCACCGCCGCCGTTCGCGCGCACGACCGGCTCCTCGTCA
ACAATTCCTATCTGGTTCCGCTTTACCATCTCGACGCGCAATGGATTGCCCGCTGGAAGCATATCGGCCGGCC
GACGACGGTACCGCTTTACGGTTATCAGTTGCCGAGTTGGTGGGACGAGCGCGTTCAGTAAATGTCGCTTCTT
TCGGTCGAGGAAGCACTACAGAGAATTTTGGCCAGGGCCAATCCGCTGCGACGGACGGAGCGCCTTGCGCTGC
ATGAATGCCTCGGACGGGTGCTTGCCGAGAACGTAGCGGCCAAGCTGACCCACCCCCCTTTCGACAATTCCGC

GATGGATGGTTATGCCGTCCGCCACGAGGATATCGCCGAAGTCGGAGCGGAGCTCTCCGTTGTCGGCGAGTCC
GCCGCAGGACGCCGTTTTCGCGGTGGCGTGAAGGGCGGGGAGGCCGTACGCATCTTCACCGGCGCGCCGGTAC
CTGCAGGCGCCGATACCGTCATCATTCAGGAAGACACGGAAAAACTGCCGGACGGACGCATCCGCACCCTGTT
TGCGCCGGCAAAGGGCCGCCACATCCGTTTGGCGGGCCAGGATTTCCTGGCGGGTGACGTCGCGCTTTCCGCC
GGCAGCGCACTCGACGCCGGCCGCCTGACGCTCGCGGCCGGCATGAATCATCGGGAGCTGCCGGTTTTCGCGC
GACCGAAAATCGCAATCATCGCCACGGGCGACGAATTGTTGCCGCCGGGAAGTTCCCTTGGCCCCGACCAGAT
CATCGCTTCGAACAGTTTCGGGATCGCTGCGGTTGCCCGCGACAACGGGGCCGAAGTTCTTGATCTCGGCATC
GTCGGCGACGATCGGACAGCGATCGCCGGCGCCGTCGCCAAGGCACAGGCAGCCCAGGTGGACGTGCTCGTCA
CCCTCGGAGGAGCTTCGGTCGGCGATCACGATCTCGTCCAGTCGACGCTTCTCGGCTGCGGCATGACGCTTGA
TTTCTGGCGCATTGCCATGCGACCGGGAAAACCGCTGATGGTCGGGGATCTCGACGGCGTCACGGTGCTCGGC
CTTCCGGGAAACCCCGTTTCGAGCCTCGTCTGCGCCCTGCTCTTCCTCGAACCTCTGACGCGCAAGCTTGCGC
ATCTGCCGCCCCGTTCGAGACTGGTCGATGCACGGCTCGCCGCAACGCTGCCGGCCAATGACCGGCGCCAGGA
TTATATACGTGCCCGCCTCACGCTCGAGGCGGACGGTTCGCTCGTAGCGCATCCCTTCCCGCGCCAGGATTCG
TCCATGGTCAGCATTTTCGCGCAATCCGATGGGCTGATCGTGCGGCGACCGGGTGCTCCCGAAGCCGCGAGCG
GAGAGGCATGCAGCGTTTTCAGGCTCAGAGATCCCGCCTGAATGGCGAGACAGCTGACAGGAAAGCTGCCCCA
GTCCATCGACGAGACGAGGGCGATGCTGGAGGCGCAGGATTATCTCGCCGGAACCGCGCTGGCGACCGTTCTC
TTCCTGGCCCTCAGGATGAAGCGCCCGCTATTCCTCGAAGGCGAGGCCGGGGTCGGCAAGACCGAGATCGCCA
AGGTACTGGCGCGCGCCCTCGACCGCCCCCTCATCCGGCTTCAGTGCTACGAGGGTCTTGATGTCGCCTCGGC
CGTCTACGAATGGAACTATCCGGCTCAAATGCTGGAAATCCGCCTGTCGGAAGCGGCGGGCACCGTCGACCGC
CAGAGAGTGGAAAGCGACATCTTCTCCGAGCGGTTCCTCATAAGACGGCCGGTGCTGCAGGCGATATCGACGA
CTGCCGGGAGGGCGCCGGTGTTCCTGATCGACGAGCTCGACCGCACCGACGAAGCCTTCGAAGCCTTTCTCCT
GGAGGTGCTTTCCGATTTCCAGGTCACAATCCCCGAACTCGGGACGATCAAGGCCGACGAGCCGCCGATCGTC
ATCATCACCACCAACCGGACCCGCGAAATCCACGATGCCTTGAAGAGACGCTGTCTCTATCACTGGGTCGACT
ACCCCAAGGCGGCGCAGGAACTGGAGATCATCCGCCGCAAGGTTCCGGGCTGCAACGCGGCGCTTTCACGCGA
GATCGTTGCCTATGTTCAGAGGTTGAGGGCGCTCGACCTCTTCAAGAATCCCGGCGTCGCGGAAACGATCGAC
TGGGCCACGGCGCTCACCGAGCTCGACGCGCTGGCGCTCGATCCCGAAACGGTGGCCGACACGCTCGGTACGC
TTCTCAAATATCAGGACGACATCGCCCGGATCGAAGGTGCCGAAGGCCGCCGCCTGCTTGCCGAGGTGAAAGC
CGAATTGCTGGCGCAGGACTGAATGACGATTTCCAACACCCGCCCAATCACCCCGGACCTGATCGCCTCGCAC
GGGCTCAAGCCCGACGAATACGAACGCATCCTGAACCTGATCGGGCGCGAGCCGACCTTTACGGAGCTTGGCA
TCTTCTCGGCGATGTGGAACGAGCATTGCTCCTACAAATCCTCGAAGAAATGGCTGCGCACGCTTCCGACCAA
GGGGCCGCGCGTCATCCAGGGCCCTGGCGAGAACGCCGGCGTTGTCGACATCGACGACGGTGACTGCGTCGTC
TTCAAGATGGAGAGCCACAACCATCCCTCCTACATCGAACCCTACCAGGGAGCGGCAACGGGCGTCGGCGGCA
TCCTGCGTGATGTCTTCACGATGGGTGCGCGTCCGGTAGCGGCGATGAACGCGCTGCGGTTCGGTTCGCCGGA
TCACCCGAAGACCCGCCACCTCGTCTCGGGCGTCGTTGCAGGCGTCGGCGGCTACGGCAATTCCTTCGGCGTG
CCGACGGTCGGCGGCGAAGTCGAGTTCGACGCGCGCTACAATGGCAACATCCTCGTCAATGCCTTCGCGGCGG
GGCTCGCCAAGACCGACGCGATCTTCTACTCGAAGGCGGAAGGCGTCGGCCTCCCGGTCGTGTATCTCGGCGC
CAAGACCGGACGCGACGGTGTCGGCGGTGCGACGATGGCATCGGCCGAGTTCGACGAGTCGATCGAGGAGAAG
CGCCCGACCGTTCAGGTTGGCGACCCCTTCACCGAGAAATGCCTGCTCGAAGCCTGCCTGGAGCTGATGCAGA
CCGGCGCGGTCATTGCGATCCAGGACATGGGCGCTGCCGGCCTCACCTGCTCGGCCGTCGAAATGGGCGCCAA
GGGCGATCTCGGCATCGAACTCGATCTCGACAAGGTGCCGGTGCGCGAAGAGCGCATGACGGCTTACGAGATG
ATGCTTTCCGAAAGCCAGGAGCGCATGCTGATGGTGCTGCGCCCGGAAAAGGAAGAGGAAGCCAAGGCGATCT
TCGTCAAATGGGGCCTCGATTTCGCGATCGTGGGCAAGACCACCGACGATCTGCGTTTCCGCATCCTGCACCA
GGGCGAAGAAGTGGCGAACCTGCCGATCAAGGAGCTCGGCGACGAGGCGCCGGAATACGACCGCCCCTGGACG
CCGGCAAGGGCACCCTCGCCGCTCGCCACCAACGACGTCCCGCAGGCTGATGTTTCCGATGCGCTTCTGAAGC
TCGTCGGCTCCGCCAACAATTCCTCGCGCCGCTGGGTCTATGAGCAATATGACACGCTGATCCAGGGCAATTC
GCTGCAGCTGCCGGGCGGCGACGCCGGCGTGATCCGCGTCGAGGGCCATGAGACGAAGGCACTTGCCTTTTCT
TCGGACGTTACGCCGCGCTATGTCGAAGCCGATCCGTTCGAGGGCGGCAAGCAGGCGGTCGCCGAGTGCTGGC
GAAACCTGACTGCGACCGGCGCGCTGCCGCTCGCCGCGACCGATAATTTGAACTTCGGTAATCCCGAACGGCC
GGAGATCATGAGCCAGCTCGTCCATGCGATCAAAGGCATCGGCGAAGCCTGCCAGGCACTCGACTTCCCGATC
GTCTCCGGCAACGTCTCGCTCTACAACGAGACCAACGGCCAGGCGATCCTGCCGACCCCCACCATCGGAGGCG
TCGGCCTCGTCCGCGACTGGTCGAAGATGGCGCGGATTCGCTTTGCGGCGGCGAACGAAACCATCCTGCTCGC
CGGCGCTCCGGAGAGCTGGGGAACGCATATCGGCCAGTCGGTCTACATGCGCGATATACACGGCCGCACCGAC
GGTCCGGCGCCGCATGTCGACCTCGGCCATGAACGCAAGGTCGGCGATTTCGTTCGCGGCTTGATCGAGGACG
GCCTGGTTACGGCCGTGCACGATTGTTCCTCGGGCGGACTTGCGCTCGCCGTCGCCGAGATGGCCATCGCTTC
CGGCATCGGCGCGACGATCGATGCACCTGCGGAACACGACCCGATCCCGGTCTTCTACGGCGAAGACCAGGGC
CGCTATGTCGTAACCGTGGCCGAGGGTTCGGCCGAGACGGTGGCCGCACGTGCAAAAGCCGCCGGCGTTGCGC
TGCCGGTGATCGGCAGGACAGGCGGGAATGCGGTGCAGCTCGGAGACGCCAGGCCCGTATCCGTCGATGAATT
GCGTTCCGCACATGAAGCGTGGTTCCCCAATTACATGGGCGGCGATCTCGCACCGGACAATTGAATGAGAAGA

TTTGCCCGCACGACCCTGACTGCCGCCCTGCTTTATAGCGTGGCAGGCATTGGCGCTCATGCTCAGGAAACGC
CCGACGAACTCGATACCCGCATCGGCAAACTGACATTCGAGAGCGGCTACCCCTCGAAGGAAACCGTGGCCAA
GCTTTACGACGAAATGGATTTCCAGCGAGCGTCGCAAGCTTACCTGTGGGGCATTCCTGCCGTCGGCCTCAAC
GAATGGCGAAAGGCCCATTTCAACGTCTTCAAGGCCAAGAACGGTGAAATGCTTTCCTATCTCGATTTTGCCG
AGAAGCTTGGCATTTTGACGCCGAACTACACCACGCCTTACATCGCGACGTTCATCGATCTCAAAGAATCCGG
GCCGATGGTCGTGGTTGTACCGCCGGGGCTGATTGCAGGCATGGTGCTTGACCAGTGGCAGCGCGTCATGGCC
GATCTAGGCGTGGTCGGCCCGGATATGGGCAAGGGCGGAAAGTATCTGATCCTCCCGCCCGGATACGGCCCGG
TGGAGGCCGAGGGATACTTTGTCATCCAATCCACGAGCCGGGACGTGCTGGCGGGGTTCCGCCTGCTCGATGC
CGACAAGGAAAAGGCAATTGCAGAACTCATCCCGCAGATCAAGACCTACACCTGGACGCTTGACGGAACTGGC
GAGCCGATGCCCGTGCGCGCTGCGGGAGACGTCAAGTGGACCCAAATGCCGCCCCATGGAATGGCCTATTGGG
ACAGCCTGAACGAGGTCATTCAACGAGAACCCGTCGAAGAGCGTGATCGCTTCATGATGGCGCAGTTGAAGTT
CCTCGGCATTGAGAAAGGGAAACCCTATCAACCCGACGAACGCCAGAAGAAGATTCTTCAGGACGGCGCGATC
GTCGGCGAAGCCATGGCCAAGGTAAATACGACAGACAAGCGCGTCGAACCGCCCTTCTGGGAGGGCACGAACT
GGAAACATGCGCTTGTGGTTGCGACCGACCAGAAGGCCAACACCTATGACCAGCTCGATGAGCGTGCTGCCTG
GTTCTACGAAGCGGTCGTGATCTCCAAGGCAATGCTCACCCAAACTCCGGGCGTCGGCCAGCGGTACATAGCC
TCCTACAAGGATGCAGACGACGCATGGCTCTCGGGCGAGAACAACTATCGGCTTCATGTCCCTGCCGATCCGC
CGGCCAAGGGCTTCTGGTCTGTGACGGCCTACGACGAGTCGACCCGGCAGATGCCGGTCACCGAACAGGGACG
GCCGGACATCTCGTCGCGAAAAGAAGACATAGTAAAACACGAAGATGGATCGATTGACGTATATTTCGGTCCC
ACTGCACCGAAAGGTCACGAGGCGAACTGGGTGCAGACAAATGCCGACAAGGGATGGTTCGCGTATTTCCGGT
TCTACGGACCGACCGAGCCCTTCTTCGATAAATCCTGGTCGCTGCCTGATATCGAGAAGGTCAAATAGATGAC
TGAGAAGATGACCCTTGCCTTGATCTACGGCAGCGCACGGCAGGGCCGTTTCTGCGATACGGTCGCCAGCTGG
CTCATTCGTGAGCTCGAGACATCCGACGTGTTCAGCCTGACGATCATCGATCCCGTCAGAATGAAAAGCGCTC
GCGGCGACAAATCCGCCGGACCAGCCGAGTGGATGGAACGGAAGGTCGCCGAAGCGGACGCATTCATCGTCAT
CACTCCGGAATACAACCACGGCTACCCGGCGGCGCTGAAGGAGATGATCGATGCGGTCTATGAGCCCTGGCAC
GCAAAACCGGTCGCCTTCGTTTCCTATGGCGGCGCATCGGGCGGCATAAGGGCCGTCGAGCAATTGCGGCAGG
TCTTCGGCGAATTGCATGCCGTCACCCTGCGCGACGGCATCTCCTTCGCCAAGGCCTGGTCGAAGTTCGATGC
GGCGGGAAATCTCTACAAACCGGAAGAGATGCGAGCGCCGCTGTTCCTGATGATCGACCGGCTGACCTGGTGG
GCGCGCACGCTGAAATACGCGCGCAAGGCGACCCCTTATAACGAGATCGCCGCATGAATGAGCACGGACCACG
CCCGACTGGACGGGAAGATCGCCATTGTGACCGGGGGCACGCAGGGGCTCGGTGCCACGATTGCCGCACTCTT
CGCGGAGCGCGGCGCCGCCGGCGTCGTCATTTGCGGGCGCAACGCGGCGAAGGGCGAGGCGAAAGCTGCCGAG
ATCGCCGCGGCGACGGGTTCGAAGGTCGTCTATGTACCAGCGGATCTCGAGCGGGTGGAGGATGCCCGTGCCG
TCGCGGCGGCTTGCGACCGCGCCTTCGGCCGCGTCGATGCGCTGGTCAACGCCGCAGCGATCACCGACAGGGG
TACGATCCTCGACACCAGCCCGGAACTCTTCGACCGGATGTTCGCGGTCAATGTGCGGGCGCCATTCTTCCTG
ATGCAGGAGGTGATAAAGGTGATGCGGCGCGAGAAGACAGAGGGCACGATCGTCAATATCGGCTCCATGTCGG
CGAAGGCGGGTCAGCCCTTCATCGCAGCCTATTGTGCGTCGAAGGGGGCACTGGAGGCGCTGACCAAGAACAC
CGCCTATGCCGTCCTTCGCAACCGGATACGGGTCAACGGCCTCAACATCGGCTGGATGGCCTCGGAAGGCGAA
GACCGGATCCAGCGCGAATATCACGATGCGCCGGCGAACTGGCTCGAAAAGGCGGCAGCGAGCCAGCCCTTCG
GCAGGCTCGTCGACCCGGCCGAGGTCGCGCGTGCCTGCGCCTATCTCTCATCGGCAGAATCCGGCCTCATGAC
GGGTTCCGTCATCTGCTTCGATCAGTCGATCTGGGGTGCCTATGACGCCTCGCCTCATCCGGAGGCGCCGCTC
TGAATGAATGGACTGATGATTGCAGCCCCGAGCTCCGGCTCGGGCAAGACCACGGTCACGCTCGGCCTGATGC
GGGCATTGAGACGGCGCGGGCTCTCGATCGCGCCTGGAAAGGCGGGGCCGGACTATATCGACCCGGCTTTTCA
CACGGCTGCCAGCGGCAAACCCTGCTTCAATTATGACCCTTGGGCAATGCGCCCCGAGCTGCTCCTAGCCAAT
GCGGCGGCTGCGGCGGAAGACGGCTCGGTGCTGATCATGGAGGCGATGATGGGCCTCTTTGACGGTGCAGCGG
ACGGCAGGGGCGCGCCGGCGGATCTCGCCGCAGCACTTGGACTTGCAGTGATCCTGGTCGTCGATTGCGCGCG
CCTCTCCCATTCCGTGGCCGCCCTGGTCGGCGGCTATGCCCGTCACCGCGACGATGTCCGTGTCGCGGGCGTC
ATTCTGAACAGGGTCGGCAGCGACCGGCATGAGGGAATGCTGCGCGATGCGCTTGCCGGAATTGCGATGCCGG
TCTTCGGCGTACTGCGACAGGACGCTGCACTGAAGCTGCCCGAACGCCACCTGGGCCTCGTACAGGCAGGCGA
GCATGGATCGCTGGAAGCCTTCATCGATCACGCGGCAATGCGGGTCGCATCGGGTTGCGATCTCGAGGCGGTG
CTTGCTGCGGCGACGCCGCTGACGGTGGGAGAGAGGGCCGGAACGCTAAAGCCGCTCGGCCAGCGGACAGCCG
TCGCGCGCGACGTGGCCTTCGCCTTCTGCTACGAGCATCTTCTTTCGGGCTGGCGCGGGCAGGGGGCCGAAGT
CACATTCTTCTCGCCTCTTGCGGACGAGGCGCCCGATCCGCGTGCCGATGCGGTCTATCTCCCCGGCGGTTAT
CCCGAGCTTCATGCGGAACAGCTCTCCAATGCTTCGAATTTCCGCGCGGCCATGCACAAGGCAGCTGGGGGCG
GGGCGCGCGTTTTCGGGGAGTGCGGCGGCTACATGGTGCTCGGCGAGGGGCTTGTCGCGGCGGATGGCGGGCG
CTACGAGATGCTCGGGCTTTTGCCGCTCGTCACGAGCTTCGCCGAACGCAAGCGGCATCTCGGCTACCGGCGC
GTCACGCCGGTTGACGACGTGTTTTTCCGGGGACCGATGACGGCGCACGAATTTCACTATGCCACGATCGTTT
CGGAGGGCGCGGCTGAGCCGCTCTTCACGGTACGTGACGCGGCAGGGCTCGATCTCGGCCGCGCCGGCCTCCG
TCGCCGGAACGTTGCCGGCTCCTTCATGCATCTCATCGATTTCTCGGAATGAATGTTTCACTTGCGTCGTGCC
GCAATCGCTGTGTTGGGCGCAGCGTTTTACGGTTCCATACCGACAATTGCTGCCGAACCGGTTGGAGAAGCGG

TCCGGATCAGGACGGTGGTCACCGGCAGCGGCGGACCGCTCGTGGAGCGGGACGCCGTTCACCGGGACGAGCG
AATAAGCACGTCCAGATCCGGGCTCGGCCAGTTCGTGTTCCGCGACGGCAGTAAATTGGCGGTGGGGGCAGGC
TCTTCCGTCGTCATCGACAAGTTCGTCTTCAACGATTCGAAATCGGTGCAGCAACTCACCGTCGCCGCCGCCA
AAGGCACGTTTCGCTGGATCAGCGGAAATTCGAAGCACTCGGCCTATCAGATCCTGACACCTGCCGGAACGAT
CGGCGTACGCGGAACCGTGTTCGATTTCTACATCGGCAATGACGGAACGACGGCGATCGTTCTCCTGGAGGGC
GCGGCCCGGTTCTGCGGCCCGGCGGGATGCCAGCAGCTCACCCGACGCTGCGATTGCGTGATCGCAAAGCGCG
ACGGAACCATGACGGACACGCACAGGGCCGGTCGCCGCACACTGGCGACGCTCGGAAACCGGAAGGCCCTGCC
CTTTCTTTCGGGCGACCAAAGACTGTCTGCCTCCTTCGGCAGCATGAGCGGCAGTTGCGGTATTTCGGCCGCT
GTCGAGATCAAGCCGACCGCGCCGGAACGCGCGCGGCCGCCCGAAAGAGCAAGCCCGCAAAAGGCGCCCCGCC
CGGGCAAGACACCCAGCAACGAGCCGGGCAAGCGTGGCCTCGACAAGGGGAAGAAGGGGCAGGACACGGGCAA
GAAGGGGCAGGACACCGGCAAGAAGGGCCAGGATACCGGCAAGAACGGGCAACTGGACACAGGCACGAAGAAG
GGCCGCGGTTAGATGGCTACTCGAAACGTCGTTCTGACTGACGCACAATCAGCGCTGCTTGAACGGTTGGTCG
CCTCCGGGCGCTATCAAAATGCCTCGGAGGTCTTGCGTGCCGGGTTGCGGCTGCTCGAACGGGAAGAGGCCGA
GCTGGACGATTTGCGGGCAAGGCTGAGCACGGGACTCGATGAGGCCCGTCGCGGCGATCTAGCCGAAGGTACG
GGCGAAGAGGCGATCCGCCGTGCTTTCGCGGCAGCGCATGCAGCCTCCTGAATGTATGTCATGGCTCTTGCGA
CGGATTACGACGGCACACTTGCAGACTACGGTGCCGTCCGTCCTGAAACTCTGGAGACCTTGAAGAGGCTGAA
GGAGAGCGGGCGCAAGCTTCTGCTCGTGACCGGGCGCGAGCTGCCGGACCTCAAGAGCGTGTTTCCCGAAATC
GACGTGTTCGACAAGGTCGTCGTCGAGAACGGCGCGCTGCTCTATACGCCGGAAACCGGGGAGGAGCGGCTGC
TTGCACCCTCGCCGCCGGAGGCCTTCATCGAGCGCCTCAAGGAGAAGGGCGTCGACAGGATGTCGGTCGGCCG
TTCCATCGTCGCCACCTGGGAGCCGTTCCAGACAGCCGCGCTCGAGGCGATCAACGAACTCGGCCTCGAACTT
GAGATCATCTTCAACAAGGGCGCCGTCATGGTGCTTCCGACGGGGGTGAACAAGGCCACGGGGCTCAAGGCGG
CCCTCAAGGAGATGGGGCTTTCCTTTCTCAATGTGGTGGGGGTAGGCGATGCCGAGAACGATCACGCGCTTTT
AAGGATGTGCGGCTGCGGAGCCGCGGTCGCCAACGCCCTGCCGGCGCTCAAGGATACGGCCGACGTGGTCCTC
GAGGGTGTGCGCGGGGCCGGCGTCGAGCAACTGATGAACCGGATCATGGAGTCCGACTATGCGTTGTGCGCGA
GTGCCCGTCACCAGGTGCCGATCGGTGAGGATGACGAGGGGCAAGTGGAGATCGGACCGCAGGACGTGCTGCT
GATCGCGGGGAGTTCCGGCATCGGCAAGTCGAGGCTGGCGACCGCCCTTGCGGAGCGATTACGGGAGCGGCAC
TTCCAGCTCTGCATATTCGATCCCGAGGGCGACTATGAGGGGCTTGAAGGCGCCGTGACCGTGGGTAACGGGT
CTGTCGCGCCTACGGGCAGGGAGGTGCTGGAGCTGCTGGCCAATCCGGACGACAACGTCGTCGTCAGCGCAAC
AGGCGTCGAACCCAACGACCGGCCCGAGTTCTTCGCCAACCTGATGCCGGACCTGTCGGCGCTGCGTGCCAGG
ACGGGCAGGCCGCATTGGCTGATTATCGACGAGGCCCATCACCTCATGCCGGCAGCGCGCGACAGTGCCTCGC
TCGCGCTTTCCGACGACCGCTCCGGCATGATCATGATCACGGTCCATCCGGATTCCGTCTCACCGGATGCGCT
CAAGGAGATCACTGCGGTCGCAGCGCTCGGTCCAAAGGCGCGCGACGTAATCGCGACGATATGCTCCGTCTTC
GACGAGGCGCTGCCGGCCGGGCTGGACGCTTCTTTCGGGGAAGACGAGGTTCTTTTCTGGCGGCGCACGCCGC
CCACGCCGGTTCGGCGGATCAAGGCCGAACAACCCCGGGAAGCCCGCAAGCGGCATACGCGCAAATATGCGGA
GGGCCGGTTGGGCGAGGAGGCAAGCTTCTATTTCCGCGGTCCGAAGAACGAGATGAATTTGCGCGCCCACAAT
CTGACGATGTTTCTGCAGATCGCCGAAGGCATAGACGACAGGACCTGGGAGCACCATTTGCGTCGCGGCGACT
ACTCGAAGTGGTTCGAGGAACGCATCGGCGACGAGGAATTGGCCGGGGAGGCGGCAGGCATAGAAGAGGATCG
TTCGCTCTCGCCCGCCGAAAGCCGGCAGAGGCTGGCCGAGGCGGTGCGCCGGCGCTACACCGCGCCCGCATCC
GAAGCGGGTTGAATGAGCTTTCTCTCCGCCATACCGGTCATCGCACTTCTTGCCATGCTACCCGTTCTCCTTT
CTCTGCGGCCGGTTGCCGGTGTTCGGGAGTTCTCCGCCAGTTGCGCACTCTCGGCAGCGTCGGCAGGCGCCTT
GACGGTTGCGGCGGCGTTGCAGGCGCCGGTCGCCGCAATGTTCGGCTATGCGGGTCTCGCCGTTTCCCTGTTT
TTCGTCCTCCAGGGCCTCCGCCGGTTCCTCGCATTGTCGATTCCGGCCTCGCTTTGTCTGGTGCTCGCGTTCT
CGAGCGGCGTGCTCGGCTTCATCATCGCTCTCGGCGGCCACGACAGCCCGGCCGCCGGCATGATCGTCGTTGC
GGGCGTTGTCGCAGCTCTACTTCTCGTCCTTGGTCTGGCCGCAGCCGACCGCTGGCCGGGCGAAAAGCCGGCG
ATCCAGGTCGTTGTCGTCGGCTCCGCCGCCGTGGTTTTCACCGCCCTTGCCCATGCCCTCGGCCCATCGGCGC
ACGTCTTGCCGGGACCGGCGGGCTCGCCCGCTTCGAGCCTGTTCGATTTCCTGCTCGTCACCATGCGGGTGCT
GTGCCTTCCCCTCCTCTTCCTGGCCATGATCCTCACGATGCAGGCCCGGGTCATCGCCGGATTGAGGGCCATG
ATCGCACGCGACGGCTTGACCGGTGCGCTGTCACGCGGGACGCTCATGGAAGAGGGCGAGCGGATCTTCGCCG
AGTGCGTGGCACGCCAGGAGCCGGCTGCCTTCCTGCTGCTCGACCTCGATTTCTTCAAACAAATCAACGATCA
GTATGGGCACGCCTGCGGCGACATGGCGCTCGCGCATTTTGCCGATACCGTCTCGGCTTTCCTCGCCGGACGG
GGCGTCCTCGGACGGATCGGTGGCGAGGAATTCGGCATCGTCCTGCCGGACCACACGGAAGAGCAGGCAAGGG
CGCTTGCGGAGGATATCTGCCGCGTGGTGCGTGAGACGCCGGCCGGCCGCTCGCATCAGCGCATCCGTCTGAC
CGTGAGCATCGGGATCGCCGCCGCCATTGCGGGCGAGACGATTACCGACGTGATGATACGCGCCGACCTTGCT
CTCTACGACTCAAAGGCGGATGGTCGCGACCGTTGCTCGGTAGCAAGGGATCGCCGCATCGACGCCAGCGCCC
GCGCCCTTGCGGCAGCCGCCGCGCAATTGCGCGAAGCCCGTGCCGCCCGGTCGGAACTGCGCCACTCGGCGTG
AATGACAGCCACAGCGCGCAAAGCCCTTCTCTCCCTCGGATTCCTTGCGATCGCCGGCGCGCCGGCCCTGGCG
CAAGCTCCGGCTCAACCGGGGAACCCAGCCGCCGCGTGCGGCGGCGACCTCGGCTCCTTTCTGGAGGGCGTCA
AGGCCGAAGCGGTCGCCAAGGGCATCCCCGCAGACGTCGCCGATCGGGCGCTCGCAGGCGCCGCCATCGACCA

GAAGGTGCTGAGCCGCGACCGCGCTCAGGGCGTGTTCAAGCAGACCTTCACCGAATTTTCGAAGCGTACCGTC
AGCAAGTCGCGCCTCGACATCGGTGCGCAGAAGATGCGGGAATATGCCGACGTCTTTGCCCGGGCCGAGCAGG
AGTTCGGCGTACCGGCGCCCGTGATCACCGCATTCTGGGCCATGGAGACCGACTTCGGCGCCGTGCAGGGCGA
TTTCAATACGCGTGATGCGCTGGTGACGCTGGCGCATGACTGCCGCCGCCCGGAAATGTTCCGGCCGCAGCTT
CTCGCCGCAATCGAGATGGTGCAGCACGGCGATCTCGATCCCGCCGCGACCACCGGCGCCTGGGCGGGCGAGA
TCGGTCAGGTACAGATGCTGCCTGAGGACATCATCGCCTATGGCGTCGACGGCGACGGCGACGGCCATGTCAA
TCTCAAGAAGAGTTCTCCGGATGCCATTCTGACGGCGGCGAAATTCATCCAGAGCCTGGGCTTCAAGCCCGGG
GAACCATGGATTCAGGAGGTCCGCGTTCCGGAGCAGTTGCCATGGGAAAAGACCGGCCTGCAGCCCGGCATGA
CCGCCGGCGACTGGTTCGCACTCGGTGTCGCGCCGCGTGACGGCAACACCTCCAATAGTGGGCTCGAAGCCTC
GCTGGTGCTCCCTCAGGGCCGCAAAGGTGCTGCTTTCCTGACCTACCCGAACTTCAAGATCTATCTCGAGTGG
AACCAGTCCTTCATCTACACGACCTCCGCCGCCTATTTCGCGACCCGGCTTGCCGGTGCGCCTCCCTATCAAC
AGGGAAATCCGGAACCGGGGCTCGGCGACGCCGAGATGAAGGCCCTGCAGACCAGATTGCAGGCACTTGGGCA
TGACGTCGGCAAGATCGACGGCATTCTCGGCTCGGGCACGCGGGCCGCGCTGCAGAAGGAGCAGTTGAAGCTC
GGGATTCCCGCCGACGGGTGGGCAACACCGGGCCTGCTGGAGGCGCTTTAAATGACCAACATGATGAAGGCAC
TGGTCAAGACGAAGCCGGAGGTAGGGCTCTGGATGGAGCGCGTGCCGGTGCCGGAAATCGGGCCGAACGACGT
CCTGATCCGCGTCAGGAAATCGGCGATCTGCGGCACCGACGTCCACATCTGGAACTGGGATCAATGGGCGGAG
AAGACGATCCCAGTGCCGATGGTCGTCGGACACGAATTCATGGGCGAGGTCGTCGAGGTCGGGCCGGCCGTCA
GCAAGCATCATGTCGGCGAGCGGGTTTCCGGAGAAGGTCACATCGTCTGCGGCAAATGCCGCAATTGCCGCGC
GGGCAGGGGGCATCTCTGCCGCAACACGCTCGGCGTCGGCGTCAACCGCCCCGGATCCTTCGCCGAGTTCGTC
TGCCTTCCCGAATATAATGTCGTGTCGATCCCCGACGACGTGCCGGACGAGATCGCCGCAATCTTCGATCCCT
TCGGCAACGCCGTGCATACGGCGCTCTCCTTCGATCTCGTCGGCGAGGACGTTCTGGTCACCGGTGCCGGGCC
GATCGGCATCATGGGGGCGATGGTGGCCAAGCGTTGCGGTGCCCGCAAGGTGGTGATCACCGACATCAATCCC
GTTCGTCTCGATCTCGCGCGCAAGCTCGGCATCGACCACGTCGTCGACGCGTCGAAGGAAAATCTTGCCGACG
TCATGCGGGTGATCGGCATGACCGAGGGCTTCGATGTGGGGCTGGAAATGTCGGGCGCGGCGCCCGCTTTCCG
CGACATGATCGACAAGATGAACAATGGCGGCAAGATTGCGATCCTCGGCATTGCGCCGGCGGGCTTCGAGATC
GACTGGAACAAGGTCATCTTCAAGATGCTCAACCTCAAGGGCATCTACGGTCGCGAAATGTTCGAGACCTGGT
ACAAGATGATCGCCTTCGTCCAGGGCGGGCTCGACCTCTCGCCGATAATCACCCACCGTATCGGCATCGACGA
TTTTCGCGACGGCTTCGAGGCGATGCGCTCGGGCAATTCCGGCAAGGTGGTGATGGACTGGTAGATGCCTTCA
ATCAGGGACCACTCCGAGCGGGTCTACCGCATCGCACATCAGTCTTCCGCTGCGGTGACTTCGCCGGTCGCTG
CATCGTGGCGCCGTTGCATGACGCTGCACGGCCTGGCGCCGGAGGATGCGCGTTCGCCCTGGCGTCTTTCGGC
GATCGAGTTCCGGCAGGCGCGCGAAAGCTCCGGCGCCCTCATCGCAGAGGCGGCCGGCGAGCTCGACCGGCTG
TTCGCCACTGTCGGCAAGGCCGGGTGCTGCCTGCTGCTTACCGACGGAAAGGGCGTTGCACTGGAACGACGCG
GGGCCGGCGGCGACGATGCGGACTTTCGCGGGATCGGGTTGTGGTCGGGAACCGTGTGGAGCGAGGCGAGCGT
CGGGACCAATGGCATCGGTACGGCGATCGCGGACGAGCGTCCGGTCCTCATCCAGCGCGACCAGCACTTCCTC
AGCCGCAATATCGGCCTGAGCTGCGCCACGGCGCCGATCCGTGACGAGAGCGGCAAGCTCGCCGCGGCGATAG
ACATCTCCACCTGCCGTGACGACGCATCCGAGGCGACGCTTTCGATCCTCTCGCAGGCGGTGCGCGATGCCGC
AGCGCGAATCGAGGCCAATCTTTTCCGCCGCGCTTTCGTCGGCGCCCGCATCGTTCTCGTACCCGTCGACCGG
GCGGGACCTGCACTGCTTGCGGTCGACCGTGACGATCTCGTTCTCGGCGCGACGCGGGCTGCCCGGCTGTGGC
TCGGGCTCGACGACGAGCGTATCGCCGCCGGCGTCCCGGCTTCCGACCTGCTTCAGGAAGACCTCCCGGGCGA
GGGCGGCGAATTGCCGGACGCGGAACGTGCGGCGCTGCGCCGCGCGCTCTCCCGGGCGAAGGGCAACGTATCC
ATGGCGGCCGATCTCCTCGGCATCAGCCGCGCGACCCTCTACCGCAAGATGAAGCGATTGGCCCTGAACTGAA
TGATTCTATGGAGATCAAGCGCGAGTCGCCTGGCAATTGTAGTTGGTGCCGGCTTTTGGAGCATGCCTATACT
TGTTCAGGCGCAAGAAGCTCCCAAAGAAAACAGAACTTTGCTCGCTCCTATTGTCGTCACCGCGGACAAATCC
GACGCATTCGCGATCGGCGGTTCGGTCCAGCGGCTGGACCTGACGGATATGGAGCGCTTTTCCTATAGCGACC
CCAATCGGGTATTGCGGCAGATTCCGGGTGTTTATCTTCAAGAGGAAGAGGGGTTCGGCCTTAGGCCCAACAT
AGGCATCCGTGGTTCGGGTACAGACCGCAATGCCCGCATAACGGTCATGGAAGACGGCATCCTGATCGCGCCG
GCCCCCTATGCGGCGCCTGCGGCCTATTATTTTCCCAAGATGGCGCGCATGTCGGGGGTAGAGGTGGCCAAGG
GACCGGCAGCAATAAAATACGGTCCGATGACCGTTGGCGGCGCGATCAATCTTTTCTCGACCCCTATTCCCGA
TTCCGATATCGGAACGCTGACCGGAAAGGCGGACATTCTCGGTGGCAGCTATGGCGGCCGACGTGTCCACGCC
TGGACCGGAGGTTGGGCGCCGCTGGGCGACGCGTTCGAGATCGGCGGACTTATCGAAGGGCTTTACGATAAAT
CGGATGGCTTCAAGGATATCGATCGCGGCGGCGATACCGGATTCGATCTCACCGATTGGGGCGGCAAGCTGGG
CTTTCGCACGGCCGATGGAACGCAGAATTTCGAGTTCAAGTATCAAAACTACGATGAAACCAGCAATGAGACC
TATCTCGGCCTGACCCTGGACGATTTCAAGGACACGCCTTTCAGGCGCTATAACGCCAGTCAGAAGGACGTGA
TCGACGCCGCGCATAATATCTATCAATTCAGCCACAGTTACGAGATCACACCCGACATCAACCTGACGACGAC
CGTCTACCGTACCGATACCACGCGGGCCTGGTACAAGCTCAACGATGTCCGTAACGACTCCGATACTGGCTGG
GTATCCCTCAGCAACATCCTGGCGGACCCCGATACGTATTCGACGCAGATGGCCGATATCGTCGGTGCGGACG
GATATACCGGCCGAGCGGGGGCATTGCGTGTGCGCAACAACGCCCGCGCCTATCAGGCGACCGGCATTCAAAG
CGTGCTGACCACGAATTTCGATACCGGCGAACTCAGCCACGAACTCGAATTGTCCGCGCGCTATCATCAGGAC

GAGGAAGACCGCTTCCAGCAGGACGATCGCTACCAGATGGTCAATGGGCGCATGATCCTGACCTCCGCGGGCG
CGCCGGGCAGCCAGTCCAACCGCCTGAATGAAGCCAAGGCATGGGCTTTCTTCGCGCGCGACACGATCGAAGC
CGGACCGTTCACCTTCGTTCCCGGCCTGCGCTATGAATCGATCAATCTGGACCAGACGAACTGGGGGAGCGCC
GACCCCGGCCGCAACGGGCCGACCACTGCAAAACAGTCCTATGTCGATGTGTTCATCCCCGGCCTGTCGGCAA
CCTGGAGCGCCACCGACGAGGTTCTGCTGGTGGCCGGTGTCCATCGCGGCTTTGCCAGCCCCGCTCCGGGATC
GGACGTGGATCCCGAGACTTCATGGAACTACGAATCCGGCGTGAAGTATGATTCCGACGGCTGGCGCGCCGGT
GCCATAGGCTTCATCAACAGCTATTCGAACCTGCTTGGAACCTGCACCGCATCCACCGGAGGCAATTGCACCA
TTGGTGATCAGTTCGAGGGCGGCGAGGTCAGCGTCAAAGGCATCGAATTGACCGTTGGCCGCACATTTGGCAG
TATCGATAGTAACGGCTTCGAGGTTCCGGTAACGCTTGCCTACACCTACACGAACGCCAGGTTCCGCCGTTCC
GAGCCGGCCCAGGTCGATCACGCCCGTGCAGCGGTGTGTATCCGGATCGACCATGAAGTTCGGCAGGCATGCG
TCGCCATGACAGATTACGAGATCGGAAGGCTCGTCGATCAGGTGCCTCGGCAGTTCGGCCCGCAGAGCGTCGA
GCAGTTCGCCCGGCGGAGTGTTCTGATCCTCCGGGGCCAGGAAATCCGGATTGACGGCATCGCGCCGGACGAC
ATCCGCGGCCCGGTCGAACATCGACGCGAGCCGCCGCCCGAAAGGGCAGGCTTCCGTCGGCAATTCATGCAGC
AGCTTCAATTGCCGCAGGATCGACGGCCAGGCTTTCTTGAGGTCGACGGCGGAGAGCTCGCTTGCCGGCACAC
CCGGCAGCGCGCTGATGACGAGGCATGCTTCATCTGCCGCGATCCACTCGCAGACGGAGGGCGAGCCCAGGCG
GAACGGCGCCAGCCAAGCGACACGATCGCGCTCCCCCTCGAGCAGTGCTGCGGCCTTGCCGCTCGCAATCTTC
GCATAGGCGCCGCCGTCGCTGCGACGATAGACGCGGTCACCGGATTCGCCGTGACGAACCGGCGTCCATTCCC
GACCCGTGGGCAGTTTCGGAAGCAGAATTTCGTCACCGGCGTGGCGCCTGCTCATGAAGCCCGCCATTGCAAA
TGCGCAGACGCCATCATCCCCGTCCGCGATAGGTCGGCACGCCCTGATCCGGCAGCCAGACGCCCTCCGGCGG
GTTTCCCGTCTGCCAGAAAACGTCGATCGGGATGCCGCCGCGGGGATACCAGTAGGCCCCGATCCGGAGCCAC
TTCGGAGAAAGCAGCGAAACCAGGCGCTTGGCGATCTCGATCGTGCAATCCTCGTGGAAAGCGCCGTGGTTGC
GGAACGAATGCAGGAACAGCTTGAATGCTCAACCGGCATCATCGCCTGGCGCTTTTGGCTTGCACGGCAGCAA
TATTCGCGCTCCCGATCCCCCCGGTTCTGGCGCAGAGCGCGCCAACCGAAACGGCGGCGGAAGGAAATGCCAA
CACGACCGTGCTGAAGAAGATCGTCGCGAAAGGCGACCGCCTGGCAGGTGCGCAGCGTGGCGGCATCGCGGAT
ACGCCGCTTGCGACCGAGATCGACGCAAAGACCCTGGAGGAGAAGCAGGTCACGGATCTCGACGATCTCGGCC
GCAGCGTCGACGCAGGGATAAATGCATCGCGTGCCGACTTCGGTATCAATCTTCGCGGTCTTTCCGGTCCTCG
TATCGTGACCACGATCGACGGCGTTCCGATCCCCTATATCTCGAACAGCGCCCGTCAGGGTGCCTTTGCCTCG
ATCAATGCGAACGGCGGCGGGGACATGTTCGATTTCAATTCGCTCTCCGTGGTCGACATAGTGCGCGGGGCGG
ATTCGAGCCGTGGCGGTTCCGGCATGCTCGGGGGCGCCGTGGTGCTGCGCACGCTCGAGCCCGAGGATGTCAT
ATCCGACGGAAAGGATTGGGGCGCGATCTTCAGGTCCATCTATGACAGCGAAGACGACAGCATTGCGGGCTCG
GTTGCTGGAGCCCACAGATTCGGCCAGACCTCCGTTCTCTTTCAGGGCAGCTACCGCAAGGGGAATGAACGGG
ATAACGAGGGGACGGTCGGTGGCTACGGATCCGCCCGCACGGAGCCCAACCCTACCGATTTCGACCAGAACAA
CTTGCTCTTCAAGTTTCGGCATGAACTCGAAGGCGGTCACAGAAACGGCCTGACGGCGGAAAGCTTCCGCCGC
GACGCCGACAATGATCTCAGAGCGGAACAGGGGCGCCGGTACAAGATCGGCGACTATACCGGTTTTGAAGACC
GCGACCGCAAGCGCGTCTCGCTGGACTATGATTTCGAGGCCGCCTCGTCGGACGACTTCTTCAGCTTCGCCAG
GGCATCGCTCTACTGGCAGGATCTGGAACGCTCCTCCGGCTCGAATGGCCGGACGATCGCCGATGTGCCCTAC
GGGCGCGACAATTCGATCAGCAACGAGAGCGTGGGTTTCAACGGACGCGCAGGCAAGGATTTCGAAACCGGCG
GTTTCGATCATTCGCTAACCTTCGGCCTCGATGTCGCCCGTTCCGAGTGGAGCCAGTATACCTCGGCGGTTTG
CCCGACGCCGGCGACATGCCCGGCGCTCAACAACCAGTCCGAAGTCCCCGACGTGAGGAGCATGACGGTCGGC
GCCATATTGGAAGACCGCATTTCGGTCGGCGACAGCGCGTTTGCGCTCACGCCGGGGTTGCGCTTCGACTGGT
TCCAATATGACCCTCAGCTCAATGCAGGCTTCGAGTCGAATACAGGATCGGGAATCTTCGGGGATCTGAAGGC
CAGGGATGGGGTTCGCCTGTCGCCGAAGCTCCTCGCGACCTATGACGTTACGCCGGATGTCGAGCTGTTCGCA
CAATGGTCGATGGCCTTCCGGGCGCCGACCGTGGACGAGCTTTATAGCCGCTTCTACAACCCGTTCGGAAACT
ATGCCCAGCTCGGCAATCCGGATCTCAAGCCGGAAACCGGCAAAGGATTCGAGATCGGTGCCAATTTCGACAC
CGGTGAGCTGAGCGGCCGGGTCGCGGCGTTCCACAATATCTATGACAATTTCATTGAAACCGGCGATTCCATC
AACAGCGACACCGGGATCAGGGAATTCAAATATGCCAACGTCAACAAGGCCCGAATCTCGGGTATCGAGTTGA
GTGCGTTGAAAACCTTCGACAACGGCTTCAACCTCCATGCGTCGCTTGCCTACTCCTATGGCAAGAACGAGGA
CGAGGGCACGCGGCTGCGGACGGTCGCGCCATTCAAGGCGATCATCGGCGGCGGCTACAGCCAGGAAACCTTC
GGTGTCGATGTCTCGACGACGGTCTCGGCAGCCATGCCGGACGATAACGACAGCGAAACCTTCGATGCCCCCG
GCTACGGTCTCGTGGATATGACCGGGTGGTGGACGCCCGAAAGCTTCAAGGGATTGAGGGTAGAGGCCGGGGT
CTATAACATCTTCGACAAGAAGTATTTCAACGCGCTCGGCGTACGTGGCGTCGACCTCGCCTCATCGTCCGCT
CAGCCGCGCGACTTCTATTCCGAACCGGGACGCACGTTCAAAGTCTCGTTGACGCAACGCTTCTGAATGCAGC
CTCTTCCGCCTGAAAAACACGAGGACGCCGAGATAGCAGCCGGTTTCCTTTCGGCCATGGCAAATCCGAAACG
CCTGCTCATCCTCGACTCTCTCGTCAAGGAGGAAATGGCGGTTGGCGCCCTGGCCCATAAGGTCGGGCTGAGC
CAATCGGCTCTTTCTCAGCACCTTTCGAAGCTTCGTGCGCAGAACCTGGTCAGCACCCGTCGTGACGCACAGA
CGATCTATTATTCGAGCTCGTCCGACGCCGTATTGAAGATTCTGGGCGCACTATCCGACATTTACGGCGACGA
CACCGATGCCGTGGAAGAAAAGCCTCTCGTTCGCAAATCGGCGTAAATGGGTGGCAAGCGGCACCAGAATTCA
GGCAAGGGCGGCAAGGGCAGGGCGAAAGACGCGGCGAGCGCGCCGCAACCGAATGCCGGCAAGGTAACGCTCG

AGGGCCGCAGCTTCCTCTATGTCGGCGGCCGCGACTGCCAGGTCGCGCATCTTCGCCAGATCGCCAGTTCCTA
CGGTGCGGAACTCATCCACCATGACGGCGGTTTACGAGAGGCGGTATCGCGCATCGATCGTGTCCTGCCCTCG
GTCGACTGCGTCTTCTGCCCGATCGACTGCATCAGCCACGATGCGTGTCTTCGCGTGAAGACGGGCTGCAAGA
AGTGGGGCAAGGCCTTCGTGCCGCTGCGCAACGGCTCGAAGTCGAGCCTCGAGCGGGCGCTTCAGGATCTGAC
CGGAAACGGCAGCGAGAATTGAATGATCAGAGCATCCGATATTTCCGTCCGCCTCGCCGGAAGACAGGTGCTG
CACGGCATCTCCCTCGATGCCGCCCCCGGTGCAATGACTGCAATCGTCGGCCCGAACGGATCGGGAAAGACCA
CGACCTTGAAAGCGATCTCGGGCGAGCTCACGCCTTCGGCCGGCAAGGTCACCATCAACGGTCGCGACATCGC
AAGCCTGAAACCGTGGGAGCTTGCGCTCAAGCGCGGCGTGCTGCCGCAGTCGACCGTCATTTCCTTTCCGTTC
ACCGTTCGGGAGATCGTGCGCCTCGGCCTCATGGCCAACGGCGGCGAGGCTTCCGCCCACGGCCGGATTGCCG
ACCAGGCGCTCGAAGCCGTCGATCTTGCCGGATTTTCGGGGCGCTTCTACCAGGAGCTATCGGGCGGCGAACA
GCAGCGGGTCCAGCTCGCGCGCGTCCTCTGCCAGATCTCGGCCCCGGTCGCGGCCGGTGAACCACGCTATCTC
CTGCTCGATGAGCCGGTATCGAGCCTCGACATCCGCCACCAGCTCACGATCATGCGGCTTGCGCGCCAATTCT
GCGCCGACGGCGGCGGTGTCGTCGCGGTCATGCACGATCTCAACCTTACATCGATGTTTGCCGATCAGATCGT
GATGATGAAAGCCGGCCGCATCCGGGCGCGTGGAGCACCGAAAGACGTGCTGACCGACGAGACCATGGAAGCG
GTCTTCGGCTGCCGAATGCGGGTGAGCGTCGCTCCGGCACACGATATCCCGTTCGTATTGCCGCAGTCCGCCA
CGATGTAAATGTCCTACAGAATGTCGCGCGCGGCCTATGCCAACATGTTCGGTCCGACCGTGGGCGACAAGGT
GCGCCTCGCCGACACCGAGCTCTTCATCGAGGTCGAGAAGGATTTCACCACGCATGGCGAGGAGGTGAAGTTC
GGGGGCGGCAAGGTCATCCGTGACGGCATGGGCCAAAGCCAGGTGACGCGCGAAGGCGGCGCGGTCGACACGG
TGATCACCAATGCGCTTATCCTCGATCATTGGGGGATCGTGAAGGCGGATATCGGGCTGAAGGACGGGCGGAT
CGCGGCAATAGGCAAGGCCGGCAATCCGGACATGCAGCCGGGCGTCACCATTATCGTCGGCCCGGGCACGGAG
GTCATCGCCGGCGAGGGCAAGATCGTCACGGCCGGGGGCATGGACAGCCATATACATTTCATCTGCCCGCAGC
AGATCGAAGAGGCGTTGATGAGCGGCCTGACCTGCATGCTCGGCGGCGGGACGGGACCGGCGCACGGTACGCT
CGCCACGACCTGCACGCCCGGACCGTGGCACATCGCCCGCATGATCGAAGCGGCCGATGCGTTTCCGATGAAT
CTCGCCTTTGCGGGCAAGGGAAATGCGTCGCTGCCCGGCGCGCTCGTGGAAATGGTACTCGGCGGCGCGACCT
CGCTGAAGCTGCACGAGGATTGGGGCACGACGCCGGCCGCCATCGACTGCTGCCTCTCCGTTGCCGACGAATA
CGACGTGCAGGTTATGATCCATACCGACACACTCAACGAAAGCGGCTTCGTCGAGGATACGATCGCGGCGATC
AAGGGCAGGACGATCCACGCCTACCACACGGAAGGCGCCGGCGGCGGGCATGCGCCTGATATCATCAGGATCT
GCGGCCAGCCGAACGTCATCCCGTCCTCGACCAATCCGACGCGGCCCTATACGGTGAACACGCTTGCGGAACA
TCTCGACATGCTGATGGTATGCCATCACCTGTCGCCCACCATTCCGGAAGACATCGCCTTCGCCGAAAGCCGC
ATCCGCAAGGAGACGATCGCAGCGGAGGATATCCTCCACGATATCGGCGCCTTCTCGATCATTTCCTCCGACA
GCCAGGCGATGGGGCGGGTCGGCGAGGTGGCGATCCGCACCTGGCAGACCGCCGACAAGATGAAGCGCCAGCG
CGGACGGCTCAAGGAGGAGACCGGCGACAACGACAATTTCCGGGTCAAGCGCTACATCGCCAAATATACGATC
AACCCGGCGATAGCCCATGGCCTCAGCCATGAGATCGGCTCGCTCGAGGTCGGCAAGCGCGCCGATCTGGTCC
TCTGGAATCCCGCCTTCTTCGGCGTGAAGCCCGACATGGTGCTTCTCGGCGGCACGATCGCCGCAGCACCCAT
GGGCGACCCGAACGCCTCCATCCCCACGCCGCAGCCGGTCCACTACCGGCCGATGTTCGGCGCCTATGGAAGG
AGCCGGACCAACTCCTCGGTCACCTTCGTCTCGCAGGCGTCGCTCGATGCCGGGCTCGCCGGCAGGCTCGGCG
TCGCCAAGGAGCTCGTCGCTGTCCAGAATACCCGCGGCGGCATCGGCAAGGCGTCGATGATCCACAACAGCCT
GACGCCGCATATCGAGGTCGACCCGGAAACCTACGAGGTCCGCGCCGACGGCGAGTTCCTGACCTGCGAGCCG
GCGACGGTGCTGCCGATGGCGCAGCGGTATTTCCTGTTCTAGATGGCCTTTGGGACCGACACCATGACAAAAG
ACCCCATCCTCAAGGTTGAACGCCTGTCGATGCGCTTCGGCGGTCTCATGGCCATCAACGACTTCTCCTTCGA
GGCGGAGCGCGGCGAGATTACCGCGCTGATCGGTCCGAACGGAGCGGGCAAGACGACGGTGTTCAACTGCATC
ACCGGTTTCTACAAACCGACGATGGGCATGATCACCATGCGGCAGAAGTCGGGCGCCGAATTCCTTCTGGAAC
GGCTGCCGGATTTCGAGATCACCAAAAAGGCCAAGGTGGCTCGGACCTTCCAGAACATCCGCATGTTCTCGGG
CCTGACGGTGCTCGAAAATCTGCTCGTTGCCCAGCACAACAAGCTGATGCGGGCATCCGGCTATACCATTCTC
GGCCTGTTCGGCTTCCCGGCCTATCGCGAGGCATCCAGGGAATCGATCGAGCTCGCCAGGCACTGGCTCGAAA
AGGCATCGCTGACCGAGCGGGCCGACGATCCGGCCGGCGACCTGCCCTATGGCGCTCAGCGGCGGCTCGAGAT
CGCCCGCGCAATGTGTACCGGGCCGGAGCTTCTCTGCCTGGACGAGCCCGCGGCGGGTCTCAATCCGCGTGAA
TCGCTCGCCCTCAATGCGCTGCTGCAGGAGATCCGCCGCGATACCGGCACCTCCATCCTGTTGATCGAGCACG
ATATGTCCGTGGTGATGGAAATATCGGATCACGTCGTCGTGCTCGAATACGGTCAGAAGATTTCCGACGGCAA
CCCGGACTTCGTGAAGAACGATCCAAGGGTCATTGCGGCCTATCTGGGTGTCGAGGATGAAGAGGTTGAAGAG
GTGATCGGAGAAATCGAGGAAATCGAGGGCGAGCGTGGGGGGCCCGGGCAATGAATGAAAAAGTTGACCCTCA
ACGGACTGATCGGCGGCATCCTGATCGCCATCCTGCTCGTCACCGCTGCCGTCGGTCTCTTCTGGACACCGTT
CGACCCGATGAAACTCTCCTTCACGGCGCGGCTTGCAGCTCCCGGCCCCGCGCATCTGCTCGGCACCGACGAA
TTCGGCCGCGATGTCTTGAGCCGCCTGATGGTCGGCGCCCGCGCCAGCGTCTGGATCGGCGTGCTCACCGTCG
GCTTCGCCACTGTCTGCGGGACGCTCATCGGCCTCATCAGCGGTTATGCGCGCGGCTGGGTGGACGGCGTCAT
CATGGCTGTCAACAACGCGCTTCTCGCTTTTCCCGGCATCCTGCTGGCGCTAGGGCTGCTCGCCGTCTTCGGC
GCCAACCAGTACGGCATCATCTTCGCGCTCGGCATCGCCTATACGCCTTCCATGGCGCGCGTCGTGCGAGGCG
CCGTGCTTTCGCTGAGGGAGCGGGAGTTCATCGAGGCCTCGCGGGTAATGGGCAATGGCGAACTTTATACGAT

GTTGCGCCATATCCTGCCGAACTGCGTGGCGCCGATCACGGTTCTCGCCACCTCCATGTTCGGCTGGGCCATC
CTGTCGGAAAGCGCGCTCTCGTTTCTCGGCCTCGGCGTGCCGCCGCCGGCGCCGACCTGGGGCAACATGCTGG
CGGCCGGGCGGCCATTCCTCCAGCAGGCCGCATGGCTCGGATTGTTCCCGGGTCTGTGCATCGCGCTGACGCT
GCTTGGCATCAATCTTCTGGGCGACGCGCTCCGCGACAGGCTCGACCCGCGCATGAGAGGCCTGAAATGAATG
CGCCAGCCAGGGAGGGGAAAAGTGTACGCTGAGCTTTTCAGACTGAATGGGCGCGCCGCTTTCGTGACCGGCG
GCAGTCGCGGCATCGGCTTCGCCTGTGCCGAGGCGCTTGGCGAAGCCGGTGCGCGCGTGGCCATCTCCGCCCG
CTCGCGCGACGAAGGCGAAAAGGCTGTCCGGCAACTGAGGCAGAAGGGGATCGAGGCGATCTACCTGCCCGCC
GACATTTCCAACGAGAGCGCTGCGCAGCAGGTCGTCAGGCAGGCAGCAGCAGAGTTGGGCGGGCTCGACATCC
TCGTGAACAATGCCGGGATCGCGCGCCATTGCGACAGCCTGAAACTCGAGCCGGAAACCTGGGACGAGGTGAT
CAACACCAATCTGACCGGCCTTTTCTGGTGCTGTCGCGCGGCTATCGAGACGATGTCGGCGGCCGGGCGCGGC
TCGATCGTCAACATCGGATCGATCTCGGGTTACATCAGCAACCTGCCGCAGAACCAGGTCGCCTACAACGCCA
GCAAGGCGGGCGTGCACATGTTGACGAAGTCGCTCGCGGGCGAATTCGCCAAGAGCAACATCCGCATCAACGC
CGTCGCCCCCGGCTATATCGAGACGGCGATGACGCAGGGTGGTCTGGATGACCCCGAATGGTCGAAGATCTGG
CTCGGCATGACGCCTTTGGGCCGCGCGGGCAAGGCCAGCGAAGTGGCGGCGGCGGTGCTGTTCCTCGCTTCGG
ACGCCGCCAGCTATATCACCGGCTCGGTGCTCACCATCGACGGTGGCTACACCATCCACTGAATGGCACGGGC
ATTCGGCGGTCCGAACAAATACATCCAGCGTGCGGGCGAGATCGACAAGCTGGCGGCCTATCTGGCGCCGCTC
GGCAAACGCGCGCTCGTCCTGATCGACCGCGTGCTGTTCGACGCACTCAGCGAACGCATCGGCAAAAGCTGCG
GCGACTCGCTCGATATCCGCTTCGAGCGGTTCGGCGGAGAGTGCTGCACCTCGGAGATCGAGCGCGTACGCAA
GGTCGCAATCGAGCACGGCAGCGATATTCTCGTGGGCGTGGGTGGGGGCAAGACGGCCGACACCGCCAAGATC
GTCGCAATCGACACTGGCGCGCGCATCGTCATCGCACCGACGATCGCCTCGACCGACGCGCCCTGCAGCGCGA
TTGCCGTGCGCTACACCGAACATGGGGTTTACGAGGAGGCGCTCCGCCTGCCCCGCAACCCGGACGCCGTCGT
CGTCGATTCCGCGCTGGTGGCAGCCGCGCCGGCACGCTTCCTCGTCGCGGGGATCGGCGATGCGCTTTCGACC
TGGTTCGAGGCCCGCTCCAATATCGAGTCGCGCACCGACAATTATGTGGCCGGCGGCTTTCCCGCTACCGAAG
CCGGGATGGCGATCGCCCGGCATTGCCAGGATGTGCTGACACGCGATGCCGTCAAGGCGAAGATCGCCGTCGA
GGCCGGCCTGCTGACCCCAGCGGTCGAAAACATCATCGAGGCGAACACGCTGCTGTCGGGCCTCGGTTTCGAG
AATTGCGGCTGCTCGGCCGCCCATGGCATCCATGACGGCCTGACAGTGCTGGAGGAGGTGCATGGGTATTTCC
ACGGCGAGAAGGTCGCCTTCGGCACGCTTTGCCTGCTGATGCTGGAGAACCGCGACCGAGCCGAGATCGAGGC
GATGATACGCTTCTGCCGCAGCGTCGGACTGCCGACCAAACTGGCGGACCTCGGCATCGTCGATGACGTCCCC
GCCAAGATCGGCCGCGTGGCGGAAGCCGCCTGCCGGCCCGGCAACATCATCTATGCCACGCCGGTCACCATTA
CCGTACCGGCCGTGCGCGACGCGATCCTGGCGCTCGACGCCTTTTCACGCAGCATCCACTGAATGACCGGCAG
AAAGCCGCTCGTCATGATCACCGGCGCAAGCGCCGGCATCGGTCAGGAAACCGCACGCGTCTTCTCGGCTGCC
GGTTATCCCCTGCTTCTGATCGCCCGCCGATCCGAACTGATCGAGGCGATGGCGCTTCCGAACATGCTCGCGG
TCGCCGCCGACGTCCGTGACTATGATGCGCTCGCAAGCGCGATCCGGCAGGGTGAGGCGCGGTTCGGCCCGGT
CGACTGCCTCATCAACAATGCCGGCGTCTCCCGTCTCGCCCGGCTCGACGAGCAGGATCCTGCGCAATGGCGC
GATCTCGTCGACATCAACTGCCTCGGCGTTCTCAACGGCATGCACGCGGTCGCTCCCGGCATGAAGGAGCGGC
GCTGCGGCACCATTGTCAATGTCTCCTCCATCGCAGGCCGCAAGGTCTATCCGCACCATGACGTCTATGGCGG
CACCAAGCATTTCGTGCATGCCGTCAGCGAAGGCATGCGCCAGACCATGTCGTCATATCGCGTCCGCGTCATG
GTCATCTCGCCCGGTGTTGCGAAGTCGGAGATCGACAAGACCATCACCCACCAGGACGCCTACGATTTCTGGG
ACAGCGGCCGCAAACAGCTCGACGGCGGGCTGGATGCAAACGACATCGCGCGGACCATGCTCTTTGCCTACGA
GCTGCCGCAGGACGTCAATCTCCAGGAGATGACGATCACCCACACAGGCCAGGAATTCTGAATGATCAAGACT
TCCAGACTGTTTCTCGCAGCGGCAAGCTTCGCGCTTGCCTCTTCCACAGCCATTGCCGACGACGACAAGATCC
TGATCGGCGGCGCACTCTCGATGACGGGCATTCAGGCTCCCCTCGACACGCCCGGCTACAACGGCGCGAAGGT
CGCCGTGAAGTATCTCAACGACAATGGCGGTTTGCTGGGCAAGCAGATCGAGTTCATCAACATCGACGGCAAG
TCGGATCCGGTGACGGTCGGCAATGTCGCGGTCGAACTGATCGACAAGGGTGCTGAGATCATCGTCGCACCGT
GCGATTTCGACTTCGGTTCGCCCGCGAGCCGGGAGGCGCAATCGGCAGGCCTCGTCGGCATCTCGACTTGCGC
CTCCGACCCGCTCTATTCCTCCTGGTCGCTCGGCGACAAGCAGTTCACGCTCTCCATGTGGAATACCACGATG
GGCGCGACGGCCGCCGATTTCGCCGTGAAGGAAAAGGGCTGGAAGACGGCCTATGTCGTCACCGACCAGTTCA
TCGCCTATACCAAGTCGCTCTCGAAATATTTCGTGGAGCAATTCAAGGCCGATGGCGGCGAGATTCTGCTCGA
AGACACCTACACGAACGGCGACAACAATTTTTCCGCCCAGCTCGCCCGTCTCCAGGCGCTCGGCAAGAAGCCG
GACGTGATCTTCGTCTCCTCTTACGGCACCGACATCGGTGTGATCATCCGCGCGTTGCGCGAGGTCGGCTACG
ATGCGCCGGTTCTCGGCGGTGACGCCTATGACGACCCGGCCATGCATGAGGCGCTCGGCGAAAAATTCGGCAA
CGACGTCTATTTCGTCACGCATACCTGGATGGGCCCGGAGGCACATCCGGACATGCCGAAGTTCATCGAGCTC
TACAAGGAGATGTTCGGCAAGGCGCCGGACACGTCCTTCGTCTCGACGGGCTGGGATACGATCATGCTGCTCG
CCGAGGCGGTCAAGGCGGCCGGAACGACCGAAGGCGCAGCGCTCGCCAAGGCATTGGAAGATGGCCAGTTCAA
GCTGCTGACCGGCGATCTCGACTACGGCACGAACGAGGAAGGCCATGTGCCGAACAAGGCTGCGGCCGTGATC
GAGCTCAAGGGCGGCAAGCCGAGCTTCGTCGGATGGCGTAAGCCGGAATCCCTGCCTAAGCCCTGAATGAGCA
TCCAGACCTCTCAAACTCCGCCCGTCACGCGTCCTTCGCTTTTCCAGCGTCTGCGCCAAAGCGATGTGTGGTG
GTCGTTCACGCATAGCAAGACGGCGATGTTCAGCGCCGCGCTTCTGGCGATCCTGATCCTGACGGCACTGTTC

GCACCGCTGATCGCACCGCAGAACCCCTATGACGGCGCAGCGCTCGACATGTGGAAGGCCGAACTGCCGCCGG
TATGGGAGGAAGGTGGAGAATGGCCCTATCTTCTCGGCACCGATACGCAGGGCCGCGACATGCTGTCGGCCAT
TCTCTACGGTACGCGCATTTCCATCGTCATCGGCATCGCCTCGGTGGCGCTCTCCCTGCTGATCGGCATGAGC
GCGGGGCTGATCGCCGGCTACTTCGGCGGGTTCATCGACAATCTGCTGATGCGCTTCGGCGACGTCACGCTGT
CCATTCCGACCATCCTCGTCGCCATTCTGGTCTCGACGGTCGTGCGTCAGATGCTGCCCGTCAGCCTGCGAGA
GGTCGGCGCGTCTGCAGTGCTCATCCTGGCCATCTCGCTATCCGCCTGGGTGCAATATGCGCGCACGGTGCGC
GCGCAGGCCATCGTCGAAACCGGCAAGGACTACGTGTCGGCGGCGCGGCTGATCGGCGTGCCTGCCCGGCGCA
TCATGGCCAACCATATCCTGCCCAATACGCTGACGCCGATCATGGTGGCGGCGACGCTCAACTTCGGCATGGC
GATCCTCACCGAAGCCACGCTTTCGTTCCTGGGCATCGGCATGCCGCCGAGCCAGCCGTCTCTGGGAACGCTG
ATCCGCATCGGCAACCAGTTCCTGTTTTCCGGATCCTGGTGGATCGTGCTCTTCCCCGTCATTCAGCTCTGCC
TGCTGGTCGTGGCCGTCAACATGCTCGGCGACTGGCTGCGCGATGCTCTCAATCCGAAACTGAGGTAAATGAG
CAGCGATAAGAACATGCTTCGTTCGCTTGGCGACCACCCGCCGATCCTAGCGGACGGCCGTCGCGCACCGGAC
CGGCGCGAGATCTCGATGCGCTGGCTCTCGGGCACGTTCTTGACCGGCATCACCTCAAGTCTTCTGATGGGCG
TCGCGCTCTTTGCGGCGCTCGACGGCCGGCAGCAACTGGCGATTCCGGCGGAAGCCTTTGCCGCGCTCGATCC
GTCGGCGCCGGTCGGCGCCGCCAAACGGGGAGATCGCGTTCTTTCCCCCAACATCGTCGCCAAGCCAGCCGAC
AAGACCGTGATGGAAGTCTCGACCATGATCCATGACGGCGAGAAGGAGGTGGTGCGCCGCCAGCCCTTCGTGC
ATGTCAAGATGGCGCTTGCCGCCAACCATCAGACGTCCGAATCCTACCCCGACTTCGACCCGCTGGCGATCTT
CTCCACGGACGAGGCGGACGCCGAGGCGCCGCCGGTGAAGACCGGAACGATCTACGGCTCGGACGTCGAGTCC
GAGGTTGCCTTGAAGACCGTGGATTTTCCGCTGAACGGCGGCGGCTTCGCACTCGGGCCCTCGATGTCGCTCG
ACGAGGTCGAGGAGAACGTCCGCACCAACGGCTCGGTGCTGACCGAAGGCAATACCCAGGTCGCCTCGCTCTT
CTATGTCGACCCGCAACGCTTCGCTTCCGACGCCGACAACCTCGACCTGATGCAGGGCCTTGCCGCGCGCGTC
GTCGAGGAGAACATGAGCGTTTCCTCCTACGAGAACATCACGAAGCAGAGCATCGAATACGCCGACGACATCA
TACCGGTGCGCCGCGCCACGCCCATCGCGACGGCGATGATCAATGCGGGATATGCCAAGGCTCAGGCGGAGGA
CGCGGCCGGCTATCTCGAAGAAGCGCTCGGCGCCAAGGAACTCGGCCCCGGCGACGTGCTGCGCATCGGCATC
ATTCAGAAGGGCGAAGAGGCAAAGATCGTCCGCGCCACGATCTATTCCCATAGCCGGCACGTGCTGACCATGG
CAGTGGACGACCGCGGCCGTTTCATTCCGGGCGCCGAGCCGGCGAAGCTCGAAGCGGTGGCGGCTGCCTTCGA
CGATACCGGCAGGCCGGTGATCTCCAGCGGCCACGACCTGCCGAGGGTTTACGACGGCATCTACCGTGCGGCC
CTTTCCTACGGCATGAACGCCGACATGATCGCGCTCGTCGTCAAGCTTTTGGCAAGCAATGTCGACTTCCAGG
CTCAGCTGAAGCCGACGGACTCTCTCGAAGCGTTTTTCTCGGTCACCGATGAAAGCGGCCGGGCAACGGAAGA
TTCGGAACTGCTCTACGTCAACGCCAAATTCGGTGACGCCGAGACGCGGTTCTATCGCTTCCAGGATCCGGAC
GACAATTCGATCGATTATTTCGACAAGGACGGCAAGAGCATCCGCCAGTTCCTGTTGCGCAATCCCGTTCCGA
ACGGCCATTTCCGCTCCGGCTTCGGCATGCGCCGCCACCCTATCCTCGGCTTCTCGCGCATGCACACCGGCGT
CGACTGGTCCGCTCCGCGCGGCACGCCGATCATCGCGGCCGGAAACGGCGTCGTGCAGAAGGCCGGCTGGGAC
TCCGGCGGCTACGGCAACCAGACGCTGATCCGCCACGCCAACGGCTATGTGTCGTCCTATAACCACCAAAGCG
CGATCGCCAAAAGCGTCAAGCCGGGAACGAAGGTCGTCCAGGGTCAGGTGATCGGCTGGGTCGGAACGACCGG
CCTTTCGACCGGCCCGCACCTCCACTACGAGTTGATCGTCAACGGCAACAAGGTGGATCCCTTGCGTATCCGG
CTGCCGGGAGGAAAATCGCTCGGCGGCGAGGCGCTGGCGAAGTTCGAGAAGGAACGCGAACGCATCGACGAGC
TTCTCGGCGACGATGCGAACGAGGTCGCAAGCAAGTAGATGAATGAGAACCGTGTTCCCCGCCGCGAGTCAAA
CCCTCCGCCGCAGCAGCAGCAACGCCGCAGCGAGATGCGCGTGCGCCCGACGGGGCCGATCGTGCCTTCGGTC
AACGTTTCCGGCCATGCGCTCATGTGCGTCATCGCCATCATGTCCTTTCTCGCCTGCCTGACCCTCGGCGGCG
TCAGCATGGTGCGGGCGACCGCCCAGAGCTGGCAATCGCAGATCTCCCGTGAGATCACCATCCAGATCAAGCC
GGACGACAACCTCGACATGGAGAGGGCGCTCGCCGATGCCCGGGATCTGGCGCTGACCTTCAGCGGCACGACG
GGCGGCACGATCATCGACCGCGCGGCGACGGCACGCCTCCTGGAACCGTGGCTCGGTGGCGGGCTCGACCTGA
ACGAACTGCCCGTGCCGCGGCTCGTCGTCGTCACGATCGACGAGAGCAATCCCCCCGATTTCGCCGCGATGCG
AAAGGCGCTCACCGAAACCATCCCGCAGGCCTTCCTCGATGATCATCGCACCTGGGTCGATCGCCTGGTAGCA
ATGGCGAGTACAACCACGATGATCGGCACCGGCGTCTTGGTACTGGTCTTCTCCGCGATGGTGCTGACGGTGG
TTTTCGCCACCCGCGGCGCGCTGTCCGGCAATCGCCACATCGTCGAGGTCCTTCATTTCGTCGGAGCGGAAGC
GAGTTTCGTCGCCGCCGAATTCCAGAAGCACTTTCTCCGAATCAGTCTGAAGGGCGCCGGCGCCGGCGGCCTT
CTCGCTGCGCTGTCCTTTGCGCTGGCGAGCTTCTGGCAGTCGCAGACGCTTGCAACCCCTGAAACGGATCAGG
CGACCGCTCTGTTCGGTACCTTCGCCATCGGCTACACCGGCTATCTTGGTATCTTCGGCATTATCGTCGTCAT
TGCCCTGCTGACGACCTTGACGGCGCGCCTTACCGTCATGCGGACGATCTACGAGATCGACCTTATCCGATCG
GATCCGGGCCGGACCGACACCTTTCAGGGTTGAATGCGTTTCAGGAACCTTGTCCTCACCGCCGCTGTCGCAG
CCGCTCTTGCGGGCTGCACCAACGAGACGCTCGATTCGGTCAACCTGTCGTCCGTCAAGAACAAGACCGAGTA
CCAGCTTTCCAGCAAGATGGTCAGCAAGATGCGCGAGCTGGGGATGCAGAAGACGTCCCCGATCCTGTTGCGC
ATCTTCAAGGAAGAAGGGACGCTCGAAGTCTGGAAGGCGAACACGGCGAACCGGTTCCAGCTTCTGAAGAGCT
ACAAGATCTGCGCCTGGTCGGGAAAACTCGGGCCCAAGATGAAGGAGGGAGACCGGCAGGCGCCGGAGGGATT
CTATCCGCTCTATCCGAACCAGATGAACCCGAATTCGAGCTACTACCTGGCGATCAACACGGGCTATCCGAAC
ACCTACGACAAGGCCAACGGCCGCACCGGCACGCATCTGATGATCCACGGTGCCTGCTCTTCGGCCGGCTGCT

ATTCGATGACGAACGAGCAGATCATCGAGATTTTCGCGCTCGCCCGCGACGCGTTCAAGGGCGGCCAGGAAAG
CGTGCAGCTTCAGGCCTTTCCCTTCCGCATGACGGCGGAAAACATGGCGCGCCACCGCGACAATCCGAACATC
GAATTCTGGAAAATGCTGAAGGCCGGCTACGACCAGTTCGAAGTGACCAAGCGCCCGCCGGCAGTGAATGTCT
GCGAGAGGAAATACGTCTTCAATCAGCAGGCCGCGGGGCAGTTCAATGCGGCCGGCCAGTGCCCTGCCATGAC
GACGCCGCCGGCGCTGCAGATGGCGATGGCCAGCTACGACAAGGACTACCGGCGCGACTACGAGAAGGCATTG
AAGAAATTCGACGGCATGGTCTGGTATGAGCCGACCGAGGCCGAACGCAAGGCGGTCGTCGCCGACCAGCGCA
AGGGCCGCGAGCTTGCCTATGCGCCGACCGGCACCTCGCTCGATGCGGGCAAGCTGCTCAAGGTAAGCGATCT
CGAGAAGAAGCTTGCCGAACAGAAAGAGGCCGAGGAAGCCAAGCAGGCAACCTTGATCCAGAAGGAAGCGCTG
GAAAAGGCGGCCCGCGAAGGCAAGGGCCTGCCGGTGCCGCAGGCCAACCCGCTCGAGCGCCCCGTGGAGCAGG
CAGCGGTGCAAGCCGAGCCCGCGAGGAAGTCGCTCTGGGGTCTGTTCTCTCGCAGCGAGCCCGCGGCCGCGCC
GGCCGCACCCGCCGCCGAGGCTCCCGCGCAGCAGGGCGCAGGCCAGCCGGCCGCTCAACAGCCCGCACCGGGC
CAGCCGCAAGCCGCCGCCGGCGGGCAACAGGCAGCACCGCCGGCGCAGGACAATGCTCAGGTTGCCGCCGCTC
CCGCCGGGAATGCGCAAGCGCCGGCACCCGAACAGCAACCCGCCGAGCAGCCGAAGAAGCGCCCGTTCTGGAA
ACTCTGGGGCAATTGAATGACCTTTGCCTTCCATCCGAAGCGCCCCTTTACAGAGGACTTCCGCGCCGTCGGA
GGGGAACAGATCGAACGCGCGATCGCGATGCTGGAGGTCCAACCCGAAGGCGTGCATGAAGCGATCCACGATG
CACGCAAGGGCTTCAAGCGCCTGCGCTCGCTCTATCGCCTCGTGGCCGCGGATGCGCCACTCTTCCAGAGGCA
GGAAAACGCCCGCTTCCGCGCCATCGCACGCAGTCTCTCGACATTCCGCGACGCGGCAGCGCTTGTCGAGAAT
GCCGGGTATCTGCGCCGGCACGCGGCGAGCGAGGAGCAGCAGCTGGCCCTCGACAAGATCTGCTCGATCCTCG
CAAGCCGGCGCGACCGGATTGCCGAAGACGAAGGCGATCTCGACCGGAAGATCGAAGAAACCATCGTCAATTG
CCGAAAGGCGCACGCCGCACTCGCTCATGTTTCCTTCCATGACGGAAGGCGGAAATCCGCCCGCCGCCTGGCG
AAGGGCTGGCGGCAGACATTGCGGCGCGCCGCACGCGCCAGGGCGGCCTGTGCGGCAAGCTCCGATACGACGC
TGTTTCACGACCTGCGCAAGGGCGCACAGGATTACCGGATGCAGCTCTCCCTGCTTCGCGAGGCCTGGCCGTC
GGCAATGCGGGCGAAGAGAACCGAGGCGAGCGAACTGGTCGATGTGCTCGGCCATCTGAACGACATCGCCGCA
TTGATGTCGCTCGTCGACGAACGGCCGGACCTGGCGGGCAACAGCAGGGAACATCTTCTTTCGAATGTCGCGG
CCAGGCAGGACGAGCTGAGGCGTGAGGCCTTGAAGCGCGCGGATGCGGTCTTTCTCGACCGGCCGCGGAGGGA
GAGCCGAACCCTCGAACTGCTCTGGCTCGAAGCCGGAAGGTAGATGCACAAGGTTATTTCGATACGGATCCG
GGCGTCGACGATGCGATGGCCCTCCTCTTTCTGCACCGTCACCCGGCGATAGAGCTGGTCGGCATCACCACGG
TCTTCGGCAATGCCTCGATCGAGACGACGACGCGCAATGCGTTGTTCCTCAAAGGGGCATGGAATATTGCCGC
ACCGGTCGCCAAGGGCATCGGCGAGACGCTCGATGCCGGCCGGCCGCATGTCGGATGGCCGACAGGCATTCAC
GGCGATGACGGGCTCGGCAATATCGATGTGCCGGCGCAGATAGACCTGCCGCTCGACCCTCGTCCTGCCCACC
GCTTCATCATCGACACGGTGCGCGCCAATCCCGGGCAAGTGACGCTCATCGCCGTCGGCCGGATGACCAACCT
GGCGCGCGCATTGCGCGACGATCCGGAAATCGCCACGCTCGTCAAGGCGGTAGTGATCATGGGGGGCGCCTTC
GATGTTCCCGGCAACATCACGCCGGCGGCGGAAGCGAATATCCACGGCGACCCGGAAGCGGCCGATGTGGTGA
TGACGGCGCCATGGCCGGTGACCGTCATTGGTCTCGACGTGACGACGAAGACGGTGATGAGCCGTGCCATGCT
CTCCGATATCGCCGAACGCGGCGGCGCACCGGCGAGGCTTCTTTCCGACATCTCGCAATTCTACATCGACTTC
TACGAGCACCATGTGGATGACGGCATGATCGTGCACGACAGCTGCGCCTGCGCCTATGTCGTCGCGCCCGAAC
TCTTCCAGACCCGCAGCGGCGCCATCCGAGTGCTTTGCGGCGGCATCGCCGATGGCCAGACCATTCAAAAGCC
GGATGCACGCCTGTTTCCACCGAACGCCTGGGACGGCTTCCCAAGCCAGCAGGCGTGCATCGGAATCGATGCC
GAAGCGGTGCTGAAACTCATCGGCGATACGCTCGCGGACGGCAATTGAGTGATCGGTATCGGAGAAGACGGTG
TAGCCGGTCTCGGCGACGAGGCCAAGCGCCTCATTGAGGCCGCTCCCGTCGTCTTCGGCGGGGCCCGCCATTT
GGAACTCGCTGCGCCGCTGATCGGTGGCGAACGCCATATCTGGCAGAGCCCGTTTGAAAGATCGGTGGAGGCG
ATCGTCGCCCGTCGCGGCAGCCCCGTCGTTGTGCTCGCCTCCGGCGATCCGTTTCTTTACGGCGTCGGCGCCA
CGCTGGCGCGCCGCGTAGATGCCGCGGAAATGCGCACCATCCCCGCGCCGTCCGCCTTCAGCCTCGCGGCTTC
CCGGCTCGGCTGGCCGCTGCAGGAGGTCGCGACCGTTTCGCTGCACGGCCGTCCGCTCGATCTCATAAGGCCG
CATCTTCAGCCGGGCGCGCGCGTCCTGACGCTGACCTCGGACGAGAGCGGGCCGAAGGCGCTTGCCGGGCTGC
TTGCAGAAAGCGGATTCGGTCAGTCACGGCTTACGGTTCTCGAGGCGCTCGGCGGCGCAGGCGAGCGTGTCTC
CCGCCACCTCGCTTCCGGCTTCGCGATGGAGGAAATCAACGCGCTGAACGTCTGCGCGCTTGAGGTGGTGGCC
GATGCCGATGCACGCGTTCTGACGCTTGCGAACGGCCTGGACGAGCGGCTTTTCGAGCACGACGGCCAGATCA
CCAAGCGGGAGGTGCGGGCGCTGACCCTCTCGGCACTCGCGCCGCGCAAGGGCGAACTCCTCTGGGACATCGG
CGCCGGATCAGGCTCGATCGCGATCGAATGGATGCTTGCCGATCCGGCGATGCGCGCCGTCGCCGTAGAGGCG
TCGTCCGAACGGGCTGCGCGGATCGGCCGCAATGCGGTCCGCTTCGGCGTTCCGGGGCTTGTGGTCGTCGAAG
GGCAGGCGCCGGAGGCGCTGCGAGGGCTTCCGCGGCCGGACGTGATCTTCATCGGCGGCGGCGGCAGCGAGCC
CGGCGTGATGGAGGCGGCGATTGCAGCGCTCGCGCCCGGCGGACGTCTCGTGGCCAATGCCGTGACGACGGAG
ATGGAAGCGGTGCTGCTCGCCCGACATGCGCAGTTCGGCGGCTCGCTGATCCGCATCGACATCGCCCGGGCCT
CGCCCGTCGGCGCCATGACGGGCTGGCGGCCGGCCATGCCGGTCACGCAATGGTCGTGGATAAAGGCGTGAAT
GGAGCTGCTTGACGGATTCGATCTCAAGATCCTGAGCGAACTGCAGCGCGACGGTCATCTGACCAACAACGAA
TTGTCCGAGCGCGTCGCGCTGTCTCCTTCGCAATGTTCGCGCCGGCGTACGCGCCTGGAGGCGGAAGGCTTCA
TCACCGGCTACCAGGCGCAGATCGACCGTCAGAAGCTGGGGCTCGATCTGATGGTGGTGATTTCGGTCACGCT

GGCCACGCACAATCGCGACAATGCCAAGCGTTTCGCCAATCTCGTTTCGGCTCTGCCGGAGGTGCTGGAGGCC
TACGCGCTGACTGGTGAAATGGACTACCATCTGCGCGTCGTGACGCCGGATCTTGCCGGGCTCTCCCGCTTCG
TCAACGAGGTGCTCCTGCCGCATGACAGCGTGCAGCACGTCAAGACATCGATCGTACTGGAGACGCTCAAGAG
TTTCGAGGGGCTGCCGATACCGGCGCGGATGAAGGGGTAAATGCTTTCAATCAATCGGCGCGGCGCGCTGGGC
CTCATGGGCGCGACTGCGGGCAGCTTGGTCCTGCCGCGCTTCGCGTTCAGCCAAGGCAAGCGCCCATCCGTCA
CCATCGCCGTGCAGAAGATCACCATCAACAATACGCTCGACGTCTGGAACGAGCAGTCGAATGTCGGTGAGCG
CGTGTTCTTCCCAAACCTCTGGGAGGGTCTGATCCTGCGCGACTGGATGGGCAATCAGGGTCCGGTTCCCGGC
CTTGCGACGGAATGGAAGCGTATCGACGACAAGACCCTCGAGCTCAAGCTGCGCCAGGGTGTGAAGTTCCACA
ATGGCGACGAGCTCACGGCCGACGACGTCGTGTTCAGCTTCTCCGCGCAGCGCGTGTTCGGGGACACCCAGCC
CGCAGGCGGCAAGACGGTCTTCGAGGATGAGCACAAGCCGGCCACCGCCAAGGAACTGCCGGCAGTCGTGCCG
GGCACCGGGCGCCGCCTCTGGCCGGCGCTTGCCGGCGTCGAGGCCGTCGACAAACATACCGTTCGCTTCCACA
ACGCGACGCCGGACGTCACCATCGAGGGCCGCCTTTATGCCTTCGGCAGCCAGATTGCCAACCGTCGCGCCTG
GGACGAGGCGGCGACCTATATGGACTGGGCACGCAAGCCGATCACCACCGGCCCCTATATGGTCGGCGAACAT
AAACCGGACGTTTCCTTGACGCTCGTCGCCTTCGACGACTACTGGGGCGGCCGCCCGCCGCTGGAGCAGATTC
GCTTCGTCGAGGTTCCGGAGGTTTCGTCGCGCGTCAACGGCCTGCTCTCCGGCGAATACGACTTCGCCTGCGA
CCTGCCGCCGGACCAGATCGCCGCGGTTCAGGCCGCGCCCGGATATGAGGTCCAGGGCTCGACGATCCACAAC
CACCGCATTTCCGTCTTCAATGTACAGAACCCGACGCTTCAGGACCCGCTCGTCCGCCGGGCGATGACCCATT
CGATCGACCGCCAGGCGATCGTCGATGCGCTCTGGTCCGGTCAGACGACCGTGCCGGCCGGCCTGCAGTTCCC
CTTCTACGGTGACATGTTCGTCGAAGGCTGGACGGTTCCGGAATATAATCCGCAGCTCGCCAAGGATCTCCTG
AAGCAGGCCAATTACAAGGGCGATGCGATCCCGTTCCGACTTCTCAACAATTACTACACGAACCAGACTGCGA
ACGGCCAGATCATGGTCGAGATGTGGAAGCAGGTCGGCCTCAATGTCGAAATCCAGATGAAGGAAAACTGGGG
TCAGATCCACGACCCGTCCGGCGCGAAGGGCGTGCGCGACTGGTCGGCCGGCGCCGCCTTCAGCGATCCGGTC
TCCTCGATCGTCGCCCAGTTCGGGCCTAACGGCGAGGTTCAGCAGAAGAAGGACTGGGCAAATGCCGAGGCCA
ACCAGATGGCCCAGATCCTCGAAACGGAAACCGATCATGCGAAACGCAAGAAGGCATTCGCCCGCATGCTCGA
GATCTGTGAGCGCGAGGACCCGGTCTACCAGGTGCTTCACCAGAACGCGGTCTTCACCGGCATGAAGTCTTCG
TTGAACTGGAAGGCGGCGCCCGCCTTCGCCATGGACTTCCGCGCCGCCAACTGGGCCAGCTGAATGAATCAGG
TCACCCTTTTCCTTCGATCTGCGCTGCGGTACTTCTTCGCCGCTCTCGGCACTCTCGTGGTCGTGCTCCTCCT
GCTCGTCGGCTTTCTGGGCTTCACGGTGCCCGGCGCCCGCGTTGTCGCCTGGGCGATCGAGAAATATGCGGCG
ACCCCCGACCAGATCGTACGCATCGCCGACCCGGGCGCGCTTCTGACCGGCGACTTCACGGCCGGCACGATTA
CGCTCTTCGATGGCGAAGGCATTTATGCCGAGGTCCGCGACCTTTCGGTCGACTGGTCGCCGGCCGAACTATT
CTCGCTTCGCTTCGACTCGAGCAGGATTTCGGCCGGCTCGGTCCGCGTCGAGCGGCTGCCGGTCCCCTCGACC
GAAACCCGGGAAGTGCGCTCGACGTTTGCCCTTCCGGTTGACGTGAAGATCGATGCCTTCGATCTCAAGGAAA
TCGTGCTCGGCAAACAGATTGCCGGCGAGGATCATTTCCTGACTGCCAGAGGCAAGGTCGACGCGACGAATTC
CAGCATCGCTCTCGTGCTCGATGCCGCCGAACGGGACCGCCCGGATGCGCGCGCGACCGCCGATCTCGTCTTC
AACCCGGCCGGCAACCAGCTGAAGCTTGAAGCCGAGCTCGCGGAGCCGAAGGCCGGCCTTCTGGCGAGACTGC
TCCGCCTGCCGGGAGAACCTGCCGTCAACATGACCGTGACCGGACAGGGACCGCTTTCCGACTGGGCGGGCTC
GGGTACGGTTACGCTCGACGGAAGCGAGATCCTTCGGCTCGACGGGCAGCACGTGCTCGCGGCCGAGGGAATG
CACATGCTTACCGTATCCGGCGGCGGCGCTTTCGCCACGCTGATGCCACCGGCCTTCAGGCCTCTCTTCGAAG
GGGAGAGCAGGATCGACCTGACGGCCGCCTTCGACGGCACGAGCAAGGTCCGGATCGATGCAGGCAAGGTTTC
GACCGGCGCCATGACGCTCGATGTCTCCGGCACGGTCGACAGCCGCGGCGAGAACAACCTCCAGGCAAGGCTC
GCCGGTACGAGCGGGCCTTTCGATTTCCGCTGGCCGCTGAAAGACGGCGAGTTGCAGGCGCTTGTCGACACCG
CAAACCTGTCGCTGATCGGCGACGGGCAGTCGGCAATCCTCGATATCGCCGCCAATGTCCCGTCCGTGAACCT
GCCGCAGGGACGGCTCGGCGGCATCCGGCTGTCGGCACGGAGCGACGCGTTCAACCTGGAGAGCCGGTCGGGT
CCCCTGAAGACGACCGTCGAAGTCGGCGAGAGCCGCTTTGCAAATGCCGAGCTCGATCGGGCGGTGCAGGCGC
CCCTTAAGCTCGACGGCACCATCGCCGTCACCCCGGAAGACATCCGTTTCGACCCGCTGGAGATCGAAAGCGC
AAGCATCGGCGGCACGGTCACCGGCAGCTTCGATCGCACCGAAACGACGCTCGAGGCGGCATTCAAGGTCTTT
GCCGTGCGAGAGGTCCTCCCGCCGGGGCTCGCCGACAAGTTCGACAGAACCATCGCCCTTGCGGGCAACGCAG
TCGTGGAACAGGACGGCAGCGTCCGGCTGAGCGAACTCGACATCAAGTCCGGTGCGGTCGACGCCTCCGGCTC
GGTTGCGCTTGCCGGAAGCACTTTGAGTGCGGACATCCAGGGAACGCTGCCCGAACTCGGACGGCTGCTCGCC
GATGCGGAGGGCTCGGCCGCCTTCCACGCGGTCGCCTCCGGCCCACTGAACAAGCTCGGCATCGAAGCAGAGA
TAACCTCAAGCGGAGCGACGCTCGCCGGTCGCACATTGGACGATCTCGTCGTCAATGCCGATGCCACCGTGAC
GCCAGGGAGCCCCGAAGCGAAACTGACGGCAACCGGCACCCTCGACGGTCAGGCGATCGACGTCCGCGCCGAC
GTCGTCACCCGGGACGGCCGCACCTCGATCCCCACCCTCGAGGCAAAGATCGGCGACAACCGCCTCATCGGTG
CGATGGAGCTTACAGCCGACTTCAAGCCGAACGGGACGATCGACTTCAACCTGCCCGATCTCGGCCTCCTTGC
CGCCATGGCCGGGCAGCAGGCATCCGGCGATCTTGCCGGCTCCGCGACGATCCGGACCGCCGACGGCGTCACG
TCGCTCGCGCTGACGGCCCGCGGCGAAAGCATCAGGCGTGACGCATTGACCGTTGCCAAGCCCGTGGTCGACG
TTACCGTCGCCAATCTTGCCCGGCTGGCGATCAAGGGCGGTGTCCGGGCCGAAAGCGTCGTTCAAGGCGAAAA
CCGCCTGACGGACATCGATATCGCCTTCGAGCAAGAGGCGGAAAGAACGGGTTTCTCGGTCGGTGCCGAATTT

GAAGGCGCTCCGCTTGCGGCGACCGGCGATCTCGCGAGCCGTGACGGCCGGACCGAGATAAGCCTTGCATCGG
CCTCGGCGACAGCCAGGCGCATCCCCCTGCAGCTTGCCCGGCCGAGCGTCATCGCCATCGAAAACGGCGTTGT
GCATATCGATGGGCTGACGATCCAGGCATCGACGGGCACGGTCGCCGTGACCGGTACGGCAGGCGAGACCCTC
GATATTTCGGCGGATATCAGCGACCTGCCCGCCACCTTGATCCATACCGTCGCGCCGACGCTCGGGGCCGAGG
GCAAGATTACAGGCAAGGTGGACATTGCCGGGAATGGCGCGGCACCCGTCGTCCGCTACGATCTCACCTGGAG
CGGCGCCTCGATCGCCTCCGTGCGGTCGGCCGGCATAACGGCAGAGGACCTGTCGGCGAAGGGAACGGTGACC
GTCGCTGAGGACAGCATCCGCCTCGACCCCTTGGCCATCGAACGCGCGGGCTTTCGCGGGGACGTGACGGCCA
GCCTCGACCGCGCCGACAACACGGCCGAGGCGAATTTCCAGTTCGTTGCCGAACCCGCCGTGCTGCCGCCCGG
CATCGGCGCAAAATTCGATGCGCCGATCACCGTCTCCGGCAAGGTCGAGACAGGCGCCGGCGGCACCGTCGAA
CTGAGCGCGCTCGAAGTCAAATCCGATACCGTCAACGCCACCGGTTCGGCCGCACTTGCCGAGGGCGCGTTGA
CGGCCGAGATAGCGGGCGCATTGCCGGATCTCGGCAAGCTGCTCGCTGAAGCCGAAGGCAAGGCCGCCTTCAG
CGCCGACCTGTCCGGGCCGCTCGACGCGCTCGGCGTCAAGGCGGAAGTGACCTCCAGCGGCGCGGTCCTTGCG
GGACGCACGCTTTCGGATTTCGTGATCACCGCCGATGCAACGGCGAAACCCGGCAGTCCGGAGGCGAAGCTGA
CGGCGACGGGGGCTCTGGACGGCCAGGCGATCGACGTGAAGGCCGAGGTGGTCTCGAAGGACGGCCGCACCTC
GATCCCGACCCTGGAGGCGAAGGTCGGCGACAACAGATTGACCGGTGCGATCGACCTGACGGCAGACTTCAAG
CCCGAGGGCACGATCGACTTCAATCTCCCCGATCTCGGCCTTCTTGCCGCCATGGCCGGCGAGAAGGTCTCGG
GAGACCTCTCCGGTTCGGCAGCGATCAGAACGGCGAACGGCGTGACCTCCCTTTCGCTGAAAGCGAACGGCAG
CGGCATCAAGCGCGGCGATCTGACGATCGCAAAGCCGGTCGCCGATATCAGCATCGCCGATGTGGCGGCACTC
GCGGTCAGGGGAAGCGTCAGAGCGGAAAACGTCGCTCAGGGTCAAAACCGTGTCACCGGCCTCAATCTCACCT
TCGATCAGCAGGGCGGCAGGACCGGCTTTACCGTCGACGGCAGATATGACGGCGGTCCGCTTTCGGCAAAAGG
CGACATGGTCGCAAGCGGCGGCCGCACCGAAGTCCGCCTCGCCTCTTTCGCGGCAACGCCCAAGCGGATACCG
CTGAAGCTTGCACGCCCGACCATCGTCGCGATCGAGAACGGCACGGTGCTCCTCGATGCCCTGACCATCGAGG
CTTCCAAGGGCACCATCACGGTCGCCGGCTCTGCCGGACCGACGCTCGACATTTCGGCAAGACTCAATGCGCT
GCCTGCCGCACTGATCAACACCTTCGCCCCGACGCTCGGGGCGGAAGGCACGATCGCCGGGACGGTGGACGTG
GGCGGAACGGCAGCGGCTCCGGTGGTCGTCTACGACCTCAGATGGAGCGGCGCCTCGGTCGCAGCAGCGCGCG
GCGCCGGCGTATCGTCGCTGGAGATTGCTGCAAAGGGGCGCTTCGCCGACAACCGGGTGACCGTCGATGCCAC
GGTCTCCGGGCCGGGAGACCTTTCCTTCCGGGGCGGCGGCAATGTCGAGCTTGGCGGCAACATGCCGATCTCG
ATGAAATTCGCCGGCGACGTACCCTTCGCGCTGCTCGCCAATATCATGGCGGAACAGGGCTTCACCCTGACCG
GATCGGCCAAGGTCGACCTTTCGCTTTCCGGCCCGGCAAAGGCGCCGCAGATTGCCGGCACGGTCTCGACCGC
CGGAGCCCGGCTCGTGGACGTTCGCCGCAATCTGGCCCTCAACGACCTCACCGCCAATGTCGCACTCGACGGC
AGGCAGGCGACGATCTCTCGCCTCTCGGCCAATCTCGCCAGCGGCGGATCGATCGAGGCGAGCGGAACCGTGG
GCATCGTCCCCGGCTCCGGTTTCCCGGCGAATCTCAGAATCCGGCTCAACAATGCGACCTATGTCGACGGCAC
GCTGTTCACCGCCAACCTTGCAGGCGACCTGACGCTCAACGGCCCCCTCGTCTCGACTCCCGTGCTTGGCGGA
AGGCTGACGATCCGCAGGGCCGCGATCACCATACCGGAGAGGCTGCCGGCTTCGCTCTCGCAGATCGACATCA
AACACCGCAACGCCCCACCCCGGGTGCGGCAAATGACCAGAGACCTGCGCCGGGATACGTCCGCCGGCACCGA
GGCAAGCGGCGCGATCGCCTTCGATCTCGCCGTCAGTTCACCGGGCCAGTTCTTCGTGCGCGGACGCGGAATC
GATGCCGAACTCGGCGGCGATCTGACCATCCGCGGCACCGCCGTCCAGCCGATCGTTTCCGGTGGGTTCGACA
TGCGCCGCGGGCGCCTGGAGATCCTCGGCAAGCGGCTGACCTTCACCGACGGTCATATCGGTTTCGGCGGCGA
CCTCATTCCGACGCTCGATCTCGACGCGACCTCGAGCGCCGGTTCGACGACGATCACCGTCAACGTCGCCGGC
CCGGCCAACAACCCGACGGTGACCTTCTCCTCGTCGCCGGCCCTGCCGCAGGACGAGATCCTGGCGCAGCTCA
TCTTCAACCGGTCGCTGAACAATCTTTCCGCCTTCCAGATCGCCCAGCTCGCCTCGGCCGTGGGCCAGCTTGC
CGGCGGCGGGTCGACCTCGCTCCTCGACGGCCTGCGCAACAAGCTCGGCGTCGACGATCTCGACATCACGACC
GACGAGAGCGGCGGCGCCCAGGTCCGCGCCGGCAAATATCTGAACGACCGCACCTATCTCGAACTGCAACAGG
GCTCGGACTCCGCTTCCAGCAAGGCGGTCATCAACCTCGACATCGGCCGCGGCGTGAAGCTGAAGGGCGAAGC
GGCCGGCGACGGTTCGGCCGCCGGAGGCATCTTCTTCGAGAGGGAATATTGAATGGACGTGAGAGAATTGGAC
CTGCCACCCGCCTTCCGCGGCCTGCTTTCGGGCTACGGCTTCGAGAGGGACGCACTGGGCCAGTCGGCATCCA
CCATCTTTCTCCTCAAGGCCCAGAGCCGCCCTTTCCTTATCCTGAAGCATGAACCGTCAGGGCCGTTCACCGA
GTTGACCGGCGAGGCGGCGCGCCTGCACTGGCTCGCAGCCCAGGATATGCCGTGCCCGAGAGTTCTCGGCCAT
GACGATCATGCAGGAGAGACCTGGCTGTTGCTGGAGGCGGCCGAGGGCATGGACCTTGCCTCCTGCCGGCTGC
CGCACGCCGAACGCATCGCCATTCTCGCGGAAGCCCTTCGCCGGCTGCACCGGCTCGATCCGGCGGCTTGCCC
GTTCGACCGACGGCTGACCGCCAGCATTGCTGCGGCAAAAGCGCGGATGGAGGCCGGCCTTGTTGACGAGAGC
GATTTCGACGAGGAGCGGCAAGGCAGGCAAACGAGAGAACTCTTTGATGAGCTTCTCGCGCGCAGACCGAAAG
AGGAGCAATTGGTAGTCACCCATGGCGACGCCTGCCTCCCCAACTTCATCGCAGCCGGAAATCAATTCTCCGG
CTTCATCGACTGTGGCCGTCTGGGCGTCGCCGACATCTACCAGGATCTGGCACTCGCCTGCTGGAGCATCACC
CACAACCTGGGAGAGGAATGGGTCGCCCCGTTTCTGGATCGGTATGGGCTGGCCGCGCCAGACCCTGAAAAGC
TCGCCTTTTACCGGCTGCTGGACGAATTTTTCTGAATGATCTGCGGCCGCAACGTTCTTTTCGCTTCGCTTAT
GCTTTCGGCAGCACCGGCGCTTGCGGACGAGTGCACCCAGGCGCAGGCGGTCTATGCCGATCCGGCCGGAACC
TATGAGCTGCACTTCGAGCCCGTCGGATCGGAGGCGGCAGTCACCAGCAATCACTTCAAGGTCAGGATCGGGG

ACACCGGGGTTTTGCTCGATGGCGTCGTGATGCAGTCGGGCGAACCACTGCGGGCGAACGGGATCGTCATGAA
CGATTGCCCGACCGGCGACGTGACCGGCGCGGAACTCGAGGCCTGCACCGTCTGGCAGGGCCCGATCTATGCG
GTCGACAAGGCCGGCCAGATCGGACTGCTTCAGCCGGAGGACGCGCCCGCAGCCGATCGGATCCTGCTTCCGG
ATTTCGGACCGTCGCTGCGCGAATCGAGCGCCTGGGGCGAAGGCAAGGGTACCACCGACAGTTCCGATGTCTT
CCAGTTCAAGGGGTGTGGATCATGAATGAGCTTGCACACCGGTTTTACTTCCATCGTCGGCTTCGACTCGGCC
TGGACTGACAACCCGAAAGCTCCCGGAGCGATCTGCGTCATTCGTCTGGATGGAGACGGCGCTCATTTGTATC
TCGCACCCCGGCTTGCCTCGTTTGCGGAGGCTCTGGATGTCATCGAGCGCGAGCGCAAGGAGGCCCAGAAGTG
CCTTGTGGCACTGGATCAGCCAACGATCGTCCCGAACCTCACCGGCTCGCGTCCCGTCGACAGGGTAGCCGGA
TCGCTGATCTCCTGGATTGGAGGCGGCGTTCAGCCTGCAAGCCGGTCGAAGAAGGGTATGTTCGATGACGGCG
CACCGATCTGGCGGTTCAAGCAGACCTTGGCAGCGACCGAAGAGCCGGAACTCGCACGAGCAGCATCCAGCGG
CCTCTTCCTGATCGAGGTTTTTCCGGCTCTGGCACTGGCTGCTATCGAGGGAACCTTCTGTTCGAGGCTTGCA
GCCCCGAAGTACAATCCTGCCAACCGGAAGCGCTTCAAGATCAGCGACTGGCAGGCAGTCGCAGAAGCGGTCA
GGCGGTTCGGCATGCTGAACTCGCTGCGGCACCTCGACGAATGGTGCTTGATAGCGGCAAGCTCCGAGGCGCC
GAGGAAGGCGGACCAGGACAAGGTCGACGCATTGATCTGCGGCCTGATCGGGCTGCATTGGCTTGCCGCTCCG
CGTGACCAATCGGTGATGATCGGTGACCTGGAGAGTGGCTATATGATAGCGCCGGCGACGCGCGGCGTGCATG
AGAGGTTGCGCGCAGCGGCGCGACAGCGGGATGTGGCGATTGCGTGAATGGCCGTCGGCCTGTTCGGCGGGTC
CTTCAATCCGCCGCATGACGGGCATGCGCTCGTCGCCGAGACCGCATTGCGCCGGCTCGGGCTCGACCAGCTC
TGGTGGATGGTGACGCCCGGCAATCCGCTCAAGGACCGCAACCATCTCGCGCCGCTTGGCGAACGTATCGCCA
TGAGCGAGAAGATCGCCCGCAACCCGCGCATCAAGGTGACCGCTTTCGAGCAGGCGCTCGGTCAGAGCTACAC
GGCCCGCACGCTGGAAGTGATCCGCGCCCGCAACCGCGATGTCCGTTTCGTCTGGGTCATGGGCGCCGACAAT
CTCAAGAACTTTCACCGCTGGCAGGACTGGCGCAAGATCGTCGCCACTTTCCCGATCGCCGTCGTCGACCGGC
CGGGATCGACCCTTGCCTATCTGTCCTCGCCGATGGCGAGGGCTTTCAGCAGCGCCCGCGTCGACGAGGATGA
TGCGGGAACCCTCGCTTTCCGCCGCGCGCCCGCCTGGACCTTGATTCACGGCCCCCGTTCGGGCTTGAGCTCG
ACGGCGCTTCGTTCGGCCAAATCCGGGTGAATGAGCGCGGATGCTGTCTTCTGCGAGCTCGGTGCGCGCACGA
ATTTCTCCTTTCTCGAAGGGGCGGCGCCGGCCGAGGAGATGGTCGTATTCGCCAAGAAGGCGGGGCTTGCCGG
TCTCGGCATTGCCGACCGCAACAGCGTCGCCGGCGTCGTCAGGGCTCATGCCAAGGCGAAGGTGGAGGGCTAT
CCCTTCCAGCCGGGCGCCCGCCTGGTTTTTGCCGACGGCACGCCGGACATTCTCGCTTATCCGAAGAACAGGC
GGGGCTGGGGGCATCTCTGCCGCCTGCTCAGCGCCGGTAACCTGCGCTCGAAGAAGGGCGACTGCACGCTCCA
TCTCGCCGACCTGCTCGAATGGCAGGAGGAGCTTCTTCTGATCGTCATGCAAGGCGAAGGGCGGCCGGAACCG
GAGAGCCTGGAAGTGCTGCTCGGGACATTGAAGGAGCATGCGGGCAACCGGCTCTATCTCGGACTGGCGCCGC
ATTATGACGGTTTCGACCGGCATGATTTTGCCGTTCTGGCCGCAATTGCCCGAAAGGCCGGCATCGGGTTGCT
CGCCACCAATGACGCGCTCTATCACGATCCGCATTACAGGCCGCTCGCGGATGTCGTCACCTCCATCCGGGAG
CATGTGCCGATCGCCGGCGCCGGTTTCCTTCTGCAGAAGAATGCCGAAAGGCATCTGAAGGGCCCAAGGGAAA
TGGCCAGGCTTTTCAGCGACTATCCCGAGGCGATCGCCAATACCCGGAAATTTTTCCGCGAGCTCGCCTTCAG
CCTCGACGAGCTCAGCCACCAATATCCGGATGAAAACGCCGATGGCGAGACGCCCGCCGAAAGCTTGCGGCGG
CTCGTCGCGGAAGGTGCGGCGGAGCGCTATCCCGAAGGCGTGCCGGAAAAGGTGATGCGCCAGATCGACTATG
AGCTCGAGCTCATCCACGACAAGAAATACGAGCCTTATTTCCTGACCGTCCACAAGCTCGTGAAATTTGCCCG
CAGCGTGAATATTCTCTGCCAGGGGCGGGGCTCGGCGGCCAATTCCTCGGTCTGCTTCTGCCTCGGCATCACC
GATGTCGATCCGCAGAAATTCACCCTGCTGTTCGACCGATTCCTGTCGAAGGATCGCGACGAGCCGCCCGATA
TCGATGTCGACTTCGAGCACGAGCGGCGCGAAGAGGTGATCCAGTATATCTACAGGACTTACGGCAAGGAGCA
TGCGGGCCTGACCGCCGCGGTGATCAGCTATCGCTCGCGCTCCGCCGGGCGCGAGGTCGCCAAGGCCTTCGGC
CTTTCGGAAGACGTTCAATCGGCTCTCGTCAGTTCGATCTGGGGCTGGGGCACCTCGCCTTTCACCGAGGAGC
AGGCGAAAGGGGCGGGGCTCGACGCGGCGGACCCTCTCACCAGGCGCGTGCTTGCCTATGCCAGTCTGCTCAT
GAATTTTCCGCGCCACCTCTCGCAGCATGTGGGCGGCTTCGTGATTACCCGCGACAGGCTCGACGAGGTCGTG
CCGATCATGAACACGGCCATGCCCGACCGCTACATGATCGAATGGGACAAGGACGATCTCGATGAGCTGAAGA
TACTGAAGGTCGACGTGCTGGCGCTCGGCATGCTGACCTGCCTTGCCAAGGGTTTCAAGCTTCTGGAAGCGCA
TTACGGCGAGCCGATAACGCTGGCCGAAATCTACCAGGATCATCGGGACGCGGTTTACGACATGATCTGCCGC
GCCGATACGGTCGGCGTCTTCCAGATCGAAAGCCGGGCGCAGATGAGCATGCTGCCGCGCCTGCAGCCGCGCG
AAATGTACGATCTGGTGATCGAGGTAGCGATCGTTCGCCCCGGCCCCATCCAGGGCAATATGGTGCATCCCTA
TCTGAAACGGCGCGAAGCCCAGCGCAGGGGCGAGGCGGTCGTGTATCCCAGCCCGGAGTTGAAGGCGGTCCTG
GAAAGGACGCTGGGCGTGCCGCTGTTCCAGGAGCAGGCGATGCAGATCGCGATCACAGCGGCCGGTTTTTCGC
CCAGCGAGGCCGACCGCCTGCGCCGCGCCATGGCCACCTTCAAGCGGACCGGTACGATCCACACCTTCGAGCG
GAAGATGGTGGAGGGCATGGTCGCGAATGACTACGAGAGGGAATTCGCCGAGCGCTGCTTCAACCAGATCAAG
GGATTCGGCGAATACGGCTTCCCCGAAAGCCATGCTGCCTCCTTCGCCTCTCTCGTCTATGCCTCGGCCTGGC
TCAAGACCTATTATCCGGACATCTTCTGCGCCGCGCTCCTGAACGCACAGCCGATGGGTTTCTATGCGCCGGC
GCAACTGGTGCGCGATGCCCGTGAACACGGGGTCAGAATGCTGCCGGTCGATATCAATCATTCGGATTGGGAT
GCCCTGCTCGAAGGCGAGGGCGCCTTCGACAAGAATGCTGTCCACCCGCGCCACGCCAGCATGCGGGAGGTGA
TCAAAACCCGGAAGGCCGTGCGGCTGGGCTTCCGGCTGGTCAAGGGCCTGAAGCAGACGGATATGAAGGCGCT

CGTCGCGCGCCGGGGGGAGGGATACCGCTCCGTTCATGACCTGTGGCTTCGATCAGGGCTCTCCCGCTCCGTT
CTGGAGCGTCTTGCGGATGCCGATGCCTTCCGGTCGATAGGCCTCGACCGGCGGGCGGCCCTCTGGGCGGTAA
AGGCGCTCGACGAGCAGTCGGCGGTGGAGCGATTGCCGCTTTTCGAGGGGGCGGGTTCCGACGATCTGCAGAT
CGAGCCCAAGGTCGCTCTTCCCGATATGCCGGCAGGCGAACAGGTCATTCATGACTATCGCACCCTGACGCTC
TCCCTGAAGGCGCACCCCGTTTCCTTCATGCGGGAGGATTTTTCGCGAATGGGTATCCTGCGCAGCCGGGATC
TGGCGGCAACCGCAACTGGAAGATGGGTGACGGTGGCAGGCCTTGTACTGGTCAGGCAGCGGCCGGGCTCTGC
CAATGGCGTCATCTTCATGACGATCGAGGACGAAACAGGGATCGCCAATATCATCGTCTGGGAAAAGACGTTC
CAGAAATACCGGCGGCAGGTCATGGGATCGCGGCTTGTGAAAGTCCGCGGCCGCTTACAGAACCAGAGCGGCG
TCATTCACGTGGTCGCCGATCATCTGGAGGATATAACGCCGATGCTTGGCCTGCTGCGCCGGGAGGCGCGGCG
TTTCGGCGTCAACGACCGCGCGGACGGGGCCTTGCGGCCGAGCGCGGACGCCCGCGAAAAGAAAAAACTCCGG
CAATTGCGCCTGGGCCTGCCCGCTCGCGCAGCGCCGGAGGGCGAGGCGGCCGCCCAGGTCGCGGAGGTCATGC
CGAAGGGGCGCAATTTTCATTGAATGTTTGTAAGAAAGATCGCAACTGCACTCGCACTCGTAATGGCTGCATC
CGGCGTGGCTTCCGCTCAATCTCCGACGCGAATCCAGCAGTTCAACGCCTGGGGTGCATATTCCTACCAGGCC
GGTAACGGCAAGGTCTGCTATGTGCTTTCCGTGCCCAAGGAAAGGACCCCGGCAAGTGTCGATCACGGCGATA
TCTTCTTCCTCGTTTCGCAGCGTCCGGGACAGAACATCAGCTATGAGCCACAGGCGATGATGGGGTATCCGCT
GCAGGACAATTCCAAGGTCAACGTGGTGATCGACGGCCGCACCTTCGTCATGTTCACCAAGGGCAATTCCGCC
TGGGTGGAAAATGCCGCCGAAGAGCCGGCACTGGTCGCTGCGATGAAGTCGGGCAAGGCCATGTCGGTCAAGG
CGAAATCGCGGCGCGGCACGGAAACCTCCTACTCGTATTCGCTCTCCGGCATTTCGGCCGCCCTGAAGCAGAT
CGAGGCCTGCAAGTAAATGACGGTGGTGGTGAGGCCGAAGGCGCTCGGCGACTACGATACCCGCGAATTCCGG
ATGCTGATCGACGGTCAGTGGGTGGATGGCGCCGAGGGCCGCACGATCGAGCGGGTGGCGCCGGGTCACGGCG
TCGTCGTCAGCCGCTATCAGGCGGCGGCGAAAGCGGATGCCGAACGGGCGATCGCCGCCGCACGGCGCGCTTT
CGACGAAGGTCCATGGCCGCGCATGACCGCCTCGGAACGGTCGTTGATCCTGCTCAGGGCCGCCGACATGATT
GCCGCGCGAGCGGATGAGCTCGCTTTTCTCGACGCGGTCGAATCCGGCAAGCCGATCAGCCAGGCGAAGGGCG
AATTGGCGGGTGCCGCCGACATCTGGCGCTATGCGGCAGCACTTGCCCGCGAACTCTCGGGCGAAAGCTACAA
TACGCTCGGGGAGGGTACGCTCGGCGTCGTGCTGCGCGAGCCGATCGGCGTCGTATCGATCATCACGCCGTGG
AACTTCCCTTTCCTGATCGTCAGCCAGAAGCTTCCCTTCGCCCTTGCCGCCGGCTGCACGACGGTGGTGAAGC
CCTCTGAACTCACCTCGGCCTCGACGCTGGTTCTCGGCGAAATCCTGGAAGCGGCCGGCGTGCCCCAGGGGGT
CGTGAACATCATCGTCGGCACGGGGCCGGAGGCCGGCGCGCCGCTCACCACGCACCCGCATGTCGACATGGTC
TCCTTTACCGGCTCGACCGGAATCGGCCAGCTGACCATGGCGAATGCGGCGCAGACCCTGAAGAAGGTCTCGC
TGGAACTCGGCGGCAAGAACCCGCAGATCGTCTTTCCGGACGCAAACCTGGACGAATTCATCGATGCGGCCGT
CTTCGGCGCCTATTTCAATGCCGGCGAATGCTGCAATGCCGGCTCGCGGCTGATCCTGCACCGCGATATCGCC
GAGGAGGTGACGGCGCGCATCGCCAGCCTCTCGGCCAAGGTCAAGGTCGGCGATCCGCTCGACCCCGAAACGC
AGGTCGGCGCGATCATTACGCCGCAGCATCTGCAAAAGATCGCCGGCTATGTTTCTTCGGCGTCGAACGAAGG
CGCGAGAATAGCCCATGGCGGCACCACGCTCGATCTCGGCATGGGACAGTTCATGGCGCCGACCATCCTGTCG
GCCGTTCGGCCCGAAATGGCCGTGGCCCGGGAGGAAGTCTTCGGGCCGGTTCTCTCCGTTCTGACTTTCGAGA
AGACGGAAGAGGCGATCCGCATCGCCAATTCGATCGATTACGGGCTTTCCGCCGGAGTCTGGAGCCGCGACTT
CGATACCTGCCTGACGATCGGGCGGCGCGTACGCGCCGGCACGGTCTGGATGAACACCTTCATGGATGGCGCA
TCCGAGCTGCCTTTCGGCGGCTACCGGCAATCCGGGCTCGGCCGCGAGCTCGGTCGCCATGCTGTCGAGGACT
ATACGGAGACGAAGACGCTCAACATGCATATCGGCCGGCGCAGCAACTGGTGGATGCCGCGCTGAATGACCAT
CTTACCGAAACAGACCTTGCGAGTCGGCTTCGTCGGCACCGGCTTCATCGCGCATTTCCACCTGAAATCGCTC
ATCGGGGTACGCAATGTCGAGGTGACCGGCGTCTACAGCCGCAATCCCGAAAACCGCGCGCGCTTCGTGAGCG
AAGTCGAGGCGCTCGATCTCGGCGAGTGCCGCGCGCATGAGAGTCTCGAATCGCTCCTGTCCGCCGGCGACGT
CGATGCGGTCTGGATTCTGTCGCCGAACTACACCCGGCTCGACGTCATGCGGACGCTCCACCGGGAGATCAAG
GCGGGAAGAAGCAAGATCTTCGCGGTCGCCTGCGAGAAGCCGCTTGCGCGCACTGTGGCGGAGGCACGCGAGA
TGCTGCACCTGGCCGAGGATGCCGGTCTCAACCATGGCTATCTCGAAAACCAGGTCTTCTGCACGCCGGTGCT
GCGAGGCAAGGAGATCATCTGGCGGCGCGCCGCCTCGACCACCGGCCGGCCCTATCTCGCACGCGCCGCGGAA
GAACATGCCGGTCCGCACGAGCCCTGGTTCTGGCAGGGCGACAAGCAGGGCGGCGGGGTGCTTTCCGATATGA
TGTGCCACAGCGTCGAGGTGGCGCGCCACCTGCTCACGGCGCCCGGCGCGCCTCGCAATTCGCTGAAGGTCAA
GTCGGTGAACGGCACGGTGGCGAACCTCAAATGGACGCGGGCGGGCTATGCCGATGAGCTGCGCCGCCGCTTC
GGTGAGGATGTCGACTACCGCCGGCGCCCATCGGAGGATTTCGCCCGCGCCACCGTGGCGCTGGAGGACGAGG
AGGGCAACGAACTGATGATCGAGGCAACGACCTCCTGGGCCTATGTCGGCGCCGGCCTGCGCATCCAGCTCGA
GCTTCTCGGGCCCGAATATGCGCTGGAGTACAATTCGCTCGGCACCGGCTTGAAGATATTCCTGTCGCGGGCC
GTGCAGGGCACCGAGGGCGAGGACCTCGTCGAAAAACAGAATGCCGAGCAGGGCCTGATGCCGGTGCTTGAGG
ACGAGGCCGGCGTCTACGGCTATACGGACGAAAACCGCCACATGGTCGAATGCTTCCGCAAGGGGATAAAGCC
GCTCGAAACCTTCGAGGACGGCCTGGCGGTCGTCGAAATCCTGATGGGCCTCTACCGCTCCGCCGAGATCGGC
GCGACGCTGCATTTCCCCGCGCCGGAGCTGGAAGACTATGTGCCCGTCGTGGCGCGGACGGGGGCGTAGATGA
GGACGCGCCGCGAAATCCGCGCCCGCAAGGCGCTCCGGGACAGGATCGTCTATCACGGCATCGGCATCGGCAC
GGCCTTCTTCTTCCTGGCGCCCTTTGCCGTTTCCTTTCTTGCCTCCTTCCGGCCGAACAGCGAATCCGGCAGG

CCGCCGCTGCCGCCATGGCCGATCTCGGGGCTGAGTTTCGACGCCTACCGGGCGCTCGACAGCTTCGGTGCCG
GCATCTGGCAGCACATGTTCAATTCGCTCCTCGTGTCGATCGGAACGGTCGTCCTGACCGTCGCGGTCAGCGT
GCTCGCCGGCTACGGCTTCTCCCGCTACCGCTTCCCGTTCAAGAACGCGCTCTTCGTACTGATCATCGCGACG
CTGATGGTCCCGTTCCAATCGATCCTGACGCCGCTCTTCATCATTCTGGCGCGGCTCGGGCTCAACAATTCGC
TCGTCGGGCTGATGCTCGTCTATGTGACGCTGCAGCTTCCCTTTTCCGTCTTCATGATGCGCAACGCCTTCGA
TGCCGTGCCGAAGGAGATCGAGGAAGCGGCGCGCATCGACGGCGCCCGCGACCTGAAACTTCTCGTCCGGGTG
CTGTTGCCGCTGGTCATGCCCGGGATTGCGACCGTGTCGATCTTCGCCTTCCTCAATGCCTGGAACGAATTCT
TCGCCGCGCTCGTCCTCCTGTCCAGCAACGACAATTACACGCTTCCCGTTCTGATGACCGCCGTGCGCGCCGG
CCGTCTCGGCGCGATCAACTGGGGCGCGGTGCAGGCCGGCGTCGCGGTGATGGTCGTTCCCTGCCTCTTCGTG
TTCCTTCTTCTGCAACGCTATTACATGCGCGGGCTGATGGCCGGCGCGGTGAAATGAATGAGCAGTGAACAGA
GCAAACCCGCCGCGGCCCCCGTACTTCCGGGCACGTGGGGCATCAGGGAGACCGCGATCAAGTACGGCTTCCT
CATGCTGCTCGCCGGCATGGTTCTTTATTTCTCGCTCGTAACCGGAGGTTTTGCCTCCCCTCAAAGCGCAGTC
TTCATTCTGCAATCCGTGTCGATCACCGGCATTCTCGCCCTCGGCGTGACGGCGACGCTTGTCGTGGGGGGGT
TCGACCTCTCGATTGGCTCGGTCGCCACCACGGCGATGATGGCTTCCTCCTATGTTATGGTGGTGATGGGAGG
CGACGCGCTTACGGCAGCGCTCGTATGCCTCGCCGTCGGCGCGCTCGTCGGCCTGATCAACGGCTTCATCATC
GTCTACATGCGCGTTCCGGACCTTCTGGCTACCCTCGGCATGATGTTCCTGCTGCTCGGCCTTCAGCGCATCC
CGACCGAGGGCCGCTCGATTGCCGCGGGCATGACGCTGCCCGACGGTTCGGTCGCGACCGGCACTTTCAGTCC
GGCTTTCCTGGCGCTCGGGCGCCACCGCTTCGATCTCGTCCTGCCCAACCTCGTGCCGGTCTCGGTCGTGGTG
CTCGTGGTGCTCGCCATAGCGATCTGGTTCTTCCTCGAATACACCCGCTTCGGCCGCATAATGTATGCGGTCG
GGTCCAACGAGCGCGCGGCCGCGCTCGCAGGGGCGCCGGTCAATGCCTACAAGATTTCGGCCTACGTCATTTC
CGGCGTCTTCGCCTCTATCGGCGGCATTCTGCTTGCAGCACGCCTCGGCCGCGGCGATATCGCCTCCGGAAAC
AACCTGCTGCTCGACGCCGTCGCCGCCGCGCTGATCGGTTTTGCCGTGCTGGGTGCGGCCAAGCCGAACGCTT
TCGGAACGGCCGTCGGCGCGCTTTTCGTCGGGATCCTACTGCAGGGCCTGACGATGATGAATGCGCCTTACTA
CACGCAGGATTTCATCAAGGGCGCCGTGCTCGTCGTCGCCCTGATCTTTACCTTTGCGCTCTCGAAAAGAGGC
AAACGCTGAATGAAAGAGTTCAACGGCCGACGTTTGGAAATCGATGCGCTGCGCGCCTTGTGGGCGATCCGGC
GTCACGGCGGGATAACCCGCGCGGCCGAGGCCCTGGGCCTGTCGCAGTCTGCCGTCAGCCACAAGATCAAACG
GCTCGAGACGAGCCTCGATTGTGACCTGCTCGGCCGGAGCCCGGGCGGAGCGATGTTTACCGCAGCCGGAGAG
GATTTGCTGGATTATGCCGGGAAAATCCTCGGCTTGCATGACGAAGCGCTTCTGAGCTTATCGAAAGCGTCAC
TTGAAGGACGCATCGCGCTCGGTTTGACCGAAGACACGGCGTGCAGCGACCTCGCGCGGATATTGGGGAGATT
TCGCCGACTGCATCCGAATGTCGCCGTCCGCACCAAAGTCCGCATGAGTCTAGTCCTGCGAGCGATGCTGGAG
CGGGGGGAGCTAGACGCCGCGATCCTGCAGGTTTTCGCTCATGAAGTCCGGCCGACCGACGTCGTTCTGTTCC
GGGAGGAGCTCCATTGGGTCAAGCACCCGCAGCTGTCACTGCCGCAGCAGGGCCCGATTCCCTTCCTGTCCTT
CGACGACGAATGTTTCTATCGAAGATGGGCGCTCGACATCGGACAGGACGGCGGAGTGGTTCTTGAAACCGTA
TTCGAATGTGCAAGCGCTGCTGGTATCGTTGCCGCGGTCACTTCGGCCATGGGTGTAGCACTTCTGAGCGGGC
GTCATCTGCGCCCCGAAATGCAGATCGTCACCGAACGCTTGCCCACGCCGCCTGCTTTGGCATATGTCGCCCG
CCGCGCCCGAAAGGTGAGAAATTCCGCGCTAGATACGCTGGTCGCTGAAATCGAAAACGAAATCAGTCGCTAC
GGAGGGCTGGTTCTCGCGAGCTAAATGACGATGGAGGCGGATGAAATTCGCGTATCCGAGAGTGTGCCGGTCG
GTGCAGGGCACCGCCGCGGTACGCGCGCCATGGCGCCCGGCGACATTCCGGCGGTCGGACGGCTGTTCAACAG
GATTTTCCGAAGGCAGGACCACGCGCCCGGGAATGACCTTGGACGTTATCTCGAGACCGTCTTCTTCGGCAGC
CCTCACTATTCTCCCGAACTCGGCAGCGTCGTGCACGAAAACGGTGCGACCGGCATAGACAGCGCAATACTCG
CGGTACCGATGGCGTTCATGGTCAATGGCCGCCCGACCATGGCCCGTCTTCTCTGTGCCTTCATGGCGGATGG
AAAAGCAGGAGCCGTTGGAGCGGCGCGTCTTGCGCGCACCATGCGGGCGTCTCGCCAGGACATGTGCTTCTCC
GACAATGCGTCGCCGGTCAGCGCCGACCATTGGGTTGCGGGAGGTGGCATCGTCCTGCCGATCCAGAGCCTCG
AATGGCAGCGTTCGTTTCTGCCCTTGAGCGCCGCGGCTTTCCTTGCGGGCAGTCACTTCCCGATAGCGCGCTC
GCGCGTTGTCGTCGGCGTTCTCAGGCTGATTGACCGTGCACTGCGCCGGTTGCGGCCCTCCATGCGGCCGAAG
GCGGAGGGCGAATGCAGGATCGAGGCAGCGGGCGTCGAGGAGTTCCTCCAATGCGCGAGCCCGATGCTGGAAC
GCTTTGCGATCCGCCCGGCCTGGTCGCGAGCCGAATTCGATTGGCTCGTGCGCGTCGCTTCGATGAACCGGAG
CCTCGGGACGCTCCAATGCAGGAAAGTCGTTCTCGCGGACGGCCGCACGATCGGCGCCTTTCTTTTTTTCGGC
AAGCAAGGTGCCGTGGCCACGGTGCTCAATCTCGTCTGCGAAGAGGGGCGCGAACGCGACGTCGCCGCTGCGA
TGTTCGCGCGGCTCGATGCGGAAGGCTATGCCGTGGCGGTCGGCATGGCACAGCCGTTCCTGATGAACGCTAT
CTCGCGCCAGCGATGGCTGTCATTCCGGCACCGCGGCTACTTCTGCATGGTGACCCGCCATGCGGACCTCAAG
GAAGCGGCAGAGCGCAACGACATCTATATCGGCGGCCTCGCCTCCGAGACCTGGAGCCGGCTGCTGACGGATT
TTTAGATGTTCTCCGATCCAGATTTTCTGAAGCTCATCTTCGGCCGGCTTACCCTCGACGCGATCCCTTATCA
CGAGCCGATCCTCGTCGTGACCTTTCTCGCCGTCGCGCTCGGCGGCATCGCGCTGATCGGCCTCATCACTTAT
TACCGCTTCTGGGGGACGCTCTGGCGCGACTGGCTGACGAGCGTCGACCACAAGAAGATCGGCATCATGTACA
TCATTCTCGCGCTGGTGATGCTGCTGCGCGGATTTTCCGACGCGATCATGATGCGGCTGCAGCAGGCGATGGC
CTTCGGCGGCTCGGAAGGTTACCTGCCGCCGCATCACTACGACCAGGTCTTCACGGCACACGGCGTGATCATG
ATCTTCTTCATGGCGATGCCCTTCGTGACGGGGCTGATGAACTTCGTCGTGCCGCTGCAGATCGGTGCGCGCG

ACGTCTCCTTCCCCTTCCTCAACAATTTCAGCTTCTGGATGACGACGGCCGGCGCGATCATCATCATGCTGTC
GCTCTTCGTCGGCGAGTTCGCGCGGACCGGATGGCTTGCCTATCCGCCGCTGTCGGGCGCGGACTACAGCCCC
GGCGTCGGGGTCGACTATTACATCTGGGGCCTGCAGATCGCCGGCATCGGAACGACGCTGTCGGGGATAAACC
TCATCGCCACCATCGTGAAGATGCGCGCCCCCGGCATGACGATGATGAAGATGCCGGTCTTCACCTGGACGTC
GCTCTGCACCAATGTGCTGATCGTCGCCACCTTCCCGGTGCTGACCGCGACGCTGGCGCTGCTTACGCTCGAC
CGTTATGCCGGCACGAACTTCTTCACCAACGACCTCGGCGGCAATCCGATGATGTATGTCAACCTCATCTGGA
TCTGGGGCCATCCGGAGGTCTACATCCTCATCCTGCCGGCCTTCGGCATTTTCTCGGAAGTCGTCGCGACCTT
CTGCGGCAAGCGGCTCTTCGGCTATGCCTCGATGGTCTATGCCACGGTCGTCATCACCATCCTCTCCTACATC
GTCTGGCTGCACCACTTCTTCACCATGGGCTCGGGTGCGAGCGTCAACGCCTTCTTCGGCATCACGACGATGA
TCATCTCGATCCCGACCGGAGCGAAGATGTTCAACTGGCTCTTCACCATGTATCGCGGCCGCATCCGCTACGA
GGTGCCTATGCTGTGGACCATCGGTTTCATGGTGACCTTCGTCCTCGGCGGCATGACCGGCGTCCTGCTTGCC
GTTCCGCCGGCCGATTTCGTCCTGCACAATTCGCTGTTCCTCATCGCCCACTTCCACAATGTCATCATCGGCG
GCGTCGTCTTCGGGCTGATGGCAGGCGTCGTCTACTGGTTCCCGAAGGCCTTCGGATTCAGGCTCGATCCGTT
CTGGGGCAAGATGTCCTTCTGGTTCTGGCTGACCGGCTTCTACTTCGCCTTCATGCCGCTCTACGTGCTCGGG
CTCATGGGGGTCACGCGCCGCGTCAGCCAGTTCGAGGATCCCTCGCTGCAGATCTGGTTCGTCATCGCGGCCT
TCGGCGCCTTCCTGATCGCGCTCGGCATCCTGTCGATGCTGATCCAGCTCGTCGTCAGCTTCGTGAGGCGCGA
AGAGCTGCGCGACACGACCGGCGACCCCTGGGACGGGCGCACGCTCGAATGGTCGACCGCCTCGCCGCCACCG
GATTACAACTTCGCCTTCACCCCGCTCGTCCACGATCAGGACTCCTGGTGGGATATGAAAAAGCGCGGCCACA
GCCGGCCGCTTGTAGGCTTTAAGCCGATCCACATGCCGAAGAACACCGGCACAGGCGTCATCCTCGGAGCGAT
CAGCGTCGTCTTCGCCTTCGCCATGGTCTGGTACATGTGGTGGCTGGTGATCCTCTCCTTCGTCGCCATGGCG
GCGATCGCCATCGGCCACACCTTCAATTATGACCGCGACTTCCACATTCCCGCAAACGTCGTCGCCGACACAG
AGGGCAAGCGGACCGAAGCGCTTGCCGCCGAGGTGCAAGCATGAATGGCTCAAAGAGCAGGCAACGCGGATCT
CCCGCTGCATGGCGGCCGCGTGCCGCGATGGCTCGGCGACCGTATGACGCGCCTCGGCGCGTTGGTCACCGAA
GCGGTCGTTCATCACTACGGACGCGACGAGTTTTTAAGGCGGCTTGCGCATCCGTTCTGGTTCCAGTCCTTCG
GTGCCGTCATGGGCATGGACTGGCATTCCTCCGGTATTACCACGAGCGTCATCGGTGCATTGAAACGCGGTCT
GACCCCGCTTGCCGGCGAGCTGGGCATCCATGTCTGCGGCGGCCGCGGCCAGCACTCCCGCAAGACGCCGGGC
GAACTCATATCGATTGGCGATCGGGTCGGTTTCGATGGCGGCGCATTGGCAGAAGCGAGCCGCCTCGTCGCCA
AGGTCGACAGTGCCGCCGTTCAGGACGGCTTCGATCTCTACCTGCACGGTTTCATCGTCACGGACGATGCCAG
GTGGGTGGTCGTCCAGCAGGGCATGAACGGCGACCGCCGGCAGGCGAGGCGCTATCACTGGCTCTCGGAGGGC
CTGACAAGCTTCCTCGACGCGCCGCATAGCGCGATCGAAGGCAGAGAACAGGGCGAAATCGTCAATCTTGCCG
ACCGCCGCGCCGCCGCCTCGCGGACTGCGCAGCTCGATCTCCTCCAGTCCCTCGGGCCCGACGAGCTCCTGCG
CGAGGTCGCCCGGATCGAGTCTCGTGGGGCTCCTGTGGCCGCGCCGGCACAGCCGATGCTGCCGCATCTCTTC
ATGCCCGCCCATCACGACGTGCGCGAAAGCGACGTCAATCTCCGGCGCCTTCACGGCAACTTCGCCGCCGCCG
CCGACCGTGGTCCGGAGGATTTCAAGGACCTGCTGCTGGTGCCAGGCGTCGGCGCGCGGACGGTCAAGGCGCT
GGCGATGGTCGCGGAGGTGGTCCACGGGACGCCGTGCAGGTTCTCGGACCCCGCCCGCTTTTCGCTCGCCCAT
GGCGGCAAGGACCGCCATCCGTTCCCGGTGCCGTTGAAAGTCTATGACGAGACGATCAGGATCATGAAATCAG
CGGTCAGCAAGGGCCGCCTCGGGCGCGAGGAAGAGCTTGCGGCGCTGAAGCGGCTCGACGAGCAGTCGCGCCG
GCTGGAGCGCTACGTCACGGGCCCCGACCTCAAGGAGATCGTCGCGGGCGAATTCAGGGACTCCGCGCGTTTC
GGCGGGCGCAGCATATTCGGCTTGGAACCGCACGACGACGAGGCAACCGCGGCGGAGCCAAGCAACCGCACGC
GGCGCTGAATGACGACGAGCGAGATCAAGAACGTGCAGGGAATAGCAGCGATCGAGGCCCTGCTTGCCGCCGA
ATACGGCATCGCGGGAGAAATCCTGGCGCTGCCCGGCGAGCACGATCTCAATTTCCGCATTCAGGCGAGCGAC
GGTCGCGCGTTCCTGCTGAAGCTGCATGCCCTCGGCGCGCCCGAGGAGCTCGACATGCAGATCGCGGTTCTCG
ACCATCTCGCGCGCGAGGCGACGGAGCTGCCTGTCGCGAAGGTCCTGCCGAGCCGCTCCGGCGCCTCGTTCAC
CCGCGTCGAATTCGAAGGAGAGCGCGTCGCACGTCTTCTGACGTGGCTGCCCGGGGAAACCTGGGCACGAGCC
GCCGACCGCTCGAGCAACAGCGTCGAGACCCTTGGGGCGCTGCTCGGCAAGCTCGACCGCAGCCTCGCCGGTT
TCTCTCATCCCGGGACCAGGAGAGAGTACGCCTGGGATATAGCCCGGGCGGAGATGCACCTCGCCAATGTCGA
TCTGATCGAGGGTATGGAGAAGCGGCGAGCGGTTCGCGCGATCCTCGAGCACTTCGTCTCCACGGTGCTGCCG
CGCCTTAAGGCTTGCCCCCGGCAGGTCATCCATAATGACGCCAATGACTATAACGTGCTCGTCGGCGCCGACG
GCTGCGTCAGCGGACTGCTCGACTTCGGCGATATGGTCGAGAGCAATCGCGTGGTGGAAGTGGCGGTCGCTTC
CGCCTATGCGCTGATCGGTTCGCCGGATCCGATCGGCGCCATCGCGCGGCTGGCTGGCGGCTATCACGGCGTC
AATCCGCTCGGCGAAACGGAGGCCGAGCTGATCTTCGATCTCGTGCGCACCCGCTATGCCGTCAGCATGTGCA
TGGCGGCGAGGCAGATCCGCGACAACCCCGAAAACACCTATCTGCTCGTCAGCCAGGAGGACGTCTGGCGGGA
ACTGAGACGCCTCGAGCAAGAGAACCGGCCCTTTGCCATCGCCCGCCTTCGCGACGCCTGCGGTTTTGCACCG
ATTCCGAAGGCCGCGCGGGTGGTGCGGTGGCTGGAGAGCAATGCTCATGATTTTTCGGGCGTCATCAAGCCGG
GCATCGCAAGACCGAAACCAGCCGTCTTCGATTTTTCCGCGAACAGCTCCGAGACCTGGGCAGGTCTCGACAA
GGACGTGGCGCAAGCGCGCATCGAAGCACATATTCATGCGGAGGGGGCGGATTTCGGCCTTGGCCTCTACGGA
GAGGACAGAGCGGTCTACAAGGGTGACGCTTACCAGGCGACTGCAAGCCTGCGCCGCACGATTCACCTTGGCA
TCGATCTCTTCGCTCCCGCCAACGAGCCGGTGCATGCACCCTTCGCCGGCAAGGTCGCATTTTACCATGACGA

CGCGGTCCCTTACGGTTTCGGACCGACCATTCTGCTCGAACATAGGACGGGCGAGGGCGACGCGTTCTGGACG
CTCTACGGACATCTCTCCCGTGAAAGCGCCTCACGGCTGTCGATCGGCCAGCCGGTCGCCAGGGGCGAAGCTT
TCGCGGCCATGGGGAATCGCGCCGAAAACGGCGGGTGGGTTCCGCATCTGCATTTCCAGATCGTCACCGATCA
TCTCGGCCTCGAGGGTCGCATGCACGGCGTCGGCGTCAAAGAGCAGTGGCAGGTCTGGCGAGAGATCAGCCCC
GATCCGAGCGTCGTGCTCGGCCTTTCCGTTCCGGCGTCGGTGATCGTCGAGCGCGGCAAGGTGTTCCTGGTGC
GCGAACGCCAGCGCCGCATCGGTCGCTCCCTAAGCATCGCCTACGGCTCGGCGCCTCTCAAGATCGTTGCCGG
CGAGGGCGCCTACCTGATCGATGACGAGGGCACGCGCTGGCTCGACATGGTCAACAATGTCTGCCACGTCGGC
CATTGCCATCCGCGCGTCGTCAAGGCGGCGCAGATGCAGATGGCGCGGCTCAATACCAATTCGCGCTATCTGC
ATGACAGTCTGGTGGAATATTCGCGCCGGCTTGCGGCGCTCTTCCCTGATCCGCTGAACGTCTGCTTCTTCGT
CAATTCCGGCAGTGAGGCCAACGATCTCGCGATCCGGCTGGCGCGCGCCTATACCGGCAACCGCGACGTCATT
ACCGTCGATCACGCCTATCACGGCCATCTGACAAGCCTGATCGATGTCAGTCCCTATAAATTCGCGGGCAAGG
GCGGGGAAGGGCGGCCGGCGCATGTGCGGGTCGCCGAAATGCCGGACCTCTATCGTGGACGCTATCGCTATGG
CGATACAGACGCGGGGCGCAAATATGCCGAGGACGTGAAGCGTCAGATCGATGCTCTGGCGGCGGAAGGGCGC
AAACCCGCGCTCTTCTTCAGTGAGGGCATCCTCGGCACGGGCGGTCAACTGGTGCTCCCGGAAGGCTATCTCA
GAGGGGCCTATGCGCATGTGCGTGCGGCCGGTGGGTTGTGCCTCGCTGACGAGGTCCAGGTGGGGTTCGGCCG
TGTCGGCAGTCATATGTGGGCGCATGAAACGCAAGGAGTCGTGCCCGACATAGTCACCATGGGCAAGCCGATC
GGCAACGGCCACCCGATGGCGGCGGTGGTGACCACCGAGGCGATTGCCGCGGCCTTTGCAAACGGAATGGAAT
ATTTCAACACGTTCGGCGGAAACCCGGTATCGGCCGAGATCGGCCTCGCCGTGCTCGACATCATCCGCGATGA
GCGCCTCATGCACCATTGTGCGGTCGTCGGAAACCGTCTGATGGATGGAGCGCGCGAACTCGCCAGCCGCCAT
ACGATCATCGGCGACGTGCGCGGTTACGGGCTCTTCAACGGAATCGAGCTCGTGCGTGATCGCGACACCCTTG
AGCCGGCCGCAGCGGAGCTCGACTTCGTCATTGCCGAAATGAAGGACAGGCACCGCATCCTGCTGTCGAGCGA
TGGGCCGCAGCACAACGTGCTGAAAATCAAGCCGCCTGCGCCCTTCAGCGCAGACGATTGCGACCGTTTCCTC
GATGCCCTCGACGCGGTGCTGGCGCAGGTAAATGCCGCCTGAATGGAACACCAACTGGCCAGACAAATCGCAT
CGCGGAAGCCCTTCGATCCGACCTATGATCCGATCCGCGCGCCCAATCCGGGCGACGGCAAGGACTATGCGCC
GACCTACTGGATCGGCACGGCCGGCACTCCGCCTGCCGACGATGGGCCCGTTAGCGGCGATATGGACGTCGAC
GTCGCTATCGTCGGCTCCGGATATACGGGGCTCTCCTGCGCGATCCATCTGGCCCAGGAGCATGGCATCAAGG
CGACGGTGCTCGAAGCCAATGGCGTCGCCTGGGGCTGCAGCACCCGCAATGGCGGTCAGGCGCAGATTTCCGC
CGGCCGGCTCAAGCGTTCGCAATGGATCGAGCGCTGGGGCGTCGATGTGGCCAAAAGGCTGCATGGCGAAATC
AGCGAAGCGTTCGATCTGTTCCGGGACCTGATCCGCTCGCCCGAGATCGACTGCGACCCTCAGGACGGCGGCC
ATCTTTATATCGCCCATCGGGACAAGGTGATGCCGGCGCTCGAAGCCGAGTCTCGGCTTCTCAACGAGGTTTT
CGGCTACCGGTCGCGTATCGTCGGTCGCGATGAGGTCCACCGCGACTTCGTACGCGACGAGGAGGCGAGGGGG
GCGATGTACGAACCGGACGGCATGGGCATCCACGCGGCCAAGCTCGCCTTCGGCTACCTCAATCTGGCACGCA
AGCTCGGTGCGCGGGTCCACACGGCCAGCCCCGTTCTCGGCTGCGACTGGAAGAACGGCGCCTATCATCTGAC
TACGCCGGGCGGCACCGTTCGTGCGCGCGCCGTCTGCATTGCGACGGCGGGCTATACCTCGCCGGACCTGCAT
ACGCTGACGCGGCACCGGCTGATGCCGATCCTCTCGAACTCGATCGTCACGCGCCCGCTGACCGAGGAAGAAC
GCGCCGAACTGAACTTCAAGACGCGGATCCCGCTCACCGATACGCGCACGCTGCGCCACTACTACCGGATGAT
GCCGGACGGCCGCGTGCAGATCGGCAGCCGCAGCGCCATCACCGGCCGCGATGCGGTCAACCCCAAACATCTC
GATCGGCTGCTCGAAGGGCTCTACCGAAAGTTTCCGATCCTGAAGGGGATCACGATCGACTATTCCTGGTGGG
GCTGGGTCGATGTCAGCCACGACATGATGCCGCGGATCTTCCGGCCCGATCCGAAACAGCCGCTGTTCTATGC
GATGGGCTATGGCGGCAACGGCGTCATGTATTCCGCCCAGGCCGGCCGCCGCATGGCCCAGATGGTGGCGGGC
AAGGGCGGCGCACTCGACCTGCCGATCTTCACGTCGCCGCTCCCGAGCCATGGCCTGCTCACGCCTTTCAGAC
GCCTCGGCCAGTGGGGCATGTATCGCTGGTACTACCTCAAGGACGAAATTCTTTAGATGATCTCCTTCATCCT
TCGCCGGCTGGCGAGTGCGGTGCCGACGCTGTTCATCGTCGTCACCATATCCTTCTTTCTGATGCGTTTTGCC
CCCGGTGGGCCCTTCAACCTCGAGCGTCCTCTCCCGCCCTCCACGATGGAGAACCTGATGAGGACCTATCACC
TCGATCAGCCGCTCTGGCGCCAATACGCTACCTATCTAGGCAATGCGGTCACGGGCGACTTCGGTCCGAGCTA
CATCTACCATGACAACAATGTGGCGCAGCTGATCGGCAAGGGCCTGCCCTATTCGATGGAGCTCGGCTTTTAC
GCGCTGTTACTCGCGGTCGTCGGCGGCGTCATAACGGGAACGATCGCCGCATTGCGGCAGAACAGCTTCCTGG
ATTTCGCGGTCATGTCGGTTTCCACGATCGGGGTCACCGTTCCGAACTTCGTCGTCGGTCCCGTTCTGACGCT
GATCTTTGCGATCGTGCTCGCGTGGCTGCCCGCCGGCGGCTGGGGGGACGGTTCTCTCCGGTTCCTCATCCTG
CCGATGATCGCGCTCGCGCTTCCCCAGCTTGCAGTGTTCGCCAGGCTCACACGCGGCTCGATGATCGAGGCGC
TCAACACGGATCATATCCGCACCGCGAAAGCCTATGGCCTGCCGTCCCGCGCCGTGGTCGTGACGCACGCCAT
GCGCGGTGCCATGCTGCCGGTCGTCTCCTATCTTGCTCCTTGCGCCGCCGCCCTCCTTACGGGCTCGGCCGTG
GTGGAAACGATCTTCACTATACCGGGGGTCGGGCGCTACTTCGTGCTCGGAGCAATCAACCGCGACTATACGC
TGGTAATGGGCACCGTGATCCTCATCGCGATCTTCGTCATCCTCTTCAATCTTCTGGTCGATATTCTCTACGG
CCTGCTCGATCCGAGGGTTCGCCATGACTGAATGATCGAAATCGAGGAAATAACCAAGCGCTATGGCGACACG
ACGGTCGTGAGCGACGTGTCGCTGACGGTCGCGCCGCATACGGTCACCGTCGTCGTCGGAACCTCCGGCTCCG
GCAAGACGACGCTCCTCAGAATGATCAATCGCCTCGTCGAGCCGACGGCAGGGACGATCAAGATAGACGGCGA
GGACAATCGATCGATTCCGGGTTTCGAGCTGCGCCGGCGTATCGGCTACGCCATTCAAGGCCATGGCCTCTTC

CCGCACAGGACAGTCGCGCAGAACATCGCGACCGTGCCGACGCTGCTCGGCTGGGAGAAAGCCCGCATCGATG
CCAAGGTGGAGGAGCTGCTGAAGCTGTTCCAGCTCGATCCCTCGGAATTCGGACCGCGCTACCCTCACGAGCT
TTCCGGCGGGCAGCAGCAGCGCGTGGGCGTTGCAAGGGCACTTGCGGCGGAGCCCAACGTACTCCTGATGGAC
GAGCCCTTCGGCGCGCTCGATCCGATCATCCGCGCCAAGGCGCAGGACGACCTCGCCGCCATCCAGAAACATT
TCGGCACCACGATCATCCTCGTCACCCACGACATGGAAGAGGCCTTCCACCTTGCCGACAGGATCGCGGTGAT
GGACAAGGGCGAGGTGGTGCAATATGCGACGCCGGCCGAAATGCTCGTCAGGCCCGCCACGCCCTTCGTCGAG
ACGCTGGTCGGCGCCAGCGAGCGTCCATTCCGGCTGCTCGACATCGAAACGGTCGGCGAGGCGGTCGAGCCCG
GCTCGGCCGAGGGCCGGCCGGTTCCGGAAGTTCTCAGCCAGCGTGACGCGCTGTCGGAGCTTCTGTGGACGGG
CCGTTCGGCACTGCCGGTCGCGGCGGCGGACGGCGCGATCCTGGGCAAGATCACGCTCGAAGCGATCGTGAAG
CGTGCGGCGAGGCGGCAGTGAATGCGCGTGCCCCCCGGGATTGCCGACGATCTCACCTTTCTCACGGCCTTTC
GCCGCGACCTGCACGCTCATCCCGAACTCGGTTTCGAGGAAGAGCGGACGAGCGATCTTGTGGCGAGAATCCT
CGAAGAAGCAGGCCTCAGAGTCCACCGCGGCCTCGGCAAGACAGGCGTCGTTGGCACGCTGCAGGTCGGCAAC
GGCACGCGCGCGATCGGCCTGCGCGCCGACATGGATGCGCTCGCCATGCCGGAACTCGCCGACCGGCCCTACA
AATCCACGGTAGCGGGGAAGATGCATGCCTGCGGCCATGACGGCCATACGACGATGCTGCTCGGCGCGGCGCG
CCACCTTGCGGCGACGCGCAACTTCTCAGGAATGGTCCATTTCATCTTCCAGCCCGCCGAGGAAGGCCGCGGC
GGCGCCAGGCGCATGGTGGAGGACGGGCTTTTCGATCTTTTCCCTTGCGACGCGGTCTATGGACTGCACAACA
TGCCGGGGCTTGCAGTGGACGAGATGGCCGTCGTGGCGGGGCCGCAGCTTGCCTCCTCGGACAGCTGGCGCCT
CACCTTCCGCGGAGTCGGGACGCACGGCGCCAAGCCGCATCTCGGCCGGGATCCGATCACGGCGGCGGGGACA
TTCCTCGCTTCTCTGCAAACCGTCGTCGGCCGGGTCGTCGACCCGCTGCAGCCGGCGGTCGTCAGCGCCTGCG
CGCTGCAAGCAGGCGACCCCAAGGCGCTGAACGTCATTCCGGACACGGTCGAGATCGGCGGCACGGCGCGTGC
CTATACGCCGCATGTGCGCGACCAGTTGGAAGAAGAGATCGGCCGGTTGGCGCTGGGGACCGCGGCGATGTAC
GGCATCGAGGCCGATTATCGCTTCGAGCGGCGTATTCCCCCGGTCGTCAACGATCCCGACGCGACGGCACGCG
CCCTTTCGGCCGCGCGGTCCGTCTTCGGTGAGAAGGCGCTGACCAGCTTCCCACCCTCGACCGCCGGCGACGA
CTTTGCCTTCTTCGCGCTGGAGGCGCCCGGCTGCTATGTCTGGCTCGGGAATGGTCCCGCCGTCGACGGCGCG
CTGCACCATAACAGCGCCTACGACTTCAACGACGCGGCGATCGGCCCCGGGGCTGCCTTCTGGACCGCGCTGG
TGGAGCAGGAACTCAAGGCCTGAATGAACACTGGTCCTGATGCCGAAAAACTGCTGAACCGCATCCGGACGCG
CACCTTCACGATCGGTATTATGGGGCTTGGATACGTCGGATTGCCGCTCGCTATGAGCTCGCTTGAGGCGGGG
TTCAGGGTAATCGGTTTCGACATCGACCAGTCGCGGGTCGCGCTGCTCAACCGCGGCGAAAGCGGAATAAAGC
ATCTGCCTGGCCCAGCCATTCAGCAGGCTGTTGAGAGCAAGCGGTTTCGCGCCACCAGCGATTTCTCTGAGTT
GAGCATGCCTGATGCGATCCTGATAGCGGTTCCGACGCCGCTTACTAGAAATCGGGAGCCAAACCTGTCGTTT
GTCGAAAACAGCGCAAGGTCGATCGCTGAGCGGCTCCGTCCTGGGCAACTTGTCGTCTTAGAGTCGACCACTT
GGCCAGGCACGACACGAGAAATCGTGCGGCCGCTGCTGGAAGCGACGAACCTTGTCGTTGGAAAGGATTTTTT
TCTTGCATATTCGCCCGAGCGCGAAGACCCCGGAAACGAAGTGCACACTACCACCAGTGTTCCGAAGGTGATC
GGCGCCGACGATCCGTCTTCACTGGAAGTTGCGCAAGCACTGTACAGCCAGATTATCGTGCGGACTGTTCCCG
TGACTTCGACCGCAACCGCGGAGGCGGTCAAGCTAACGGAGAACATATTTCGCTCCGTGAACATTGCCTTGGT
CAACGAGCTGAAGGTCATTTTCGACAGGATGGGCATCGATGTTTGGGAAGTGATCGAAGCCGCCAAGACAAAG
CCGTTCGGATATATGGCGTTCTACCCCGGACCGGGCCTCGGCGGCCACTGCATCCCCATCGACCCGTTCTACC
TGACATGGAAGGCTCGCGAGTACGAAGTAGCAACGCGCTTCATAGAATTAGCTGGAGAGGTCAACACGAACAT
GCCGCACTACGTTGTTGACCGCCTTGTGGAGGCTTTGGACCGCAGAGCCCGCCGCGGCTTGAACGAATCTCGA
ATCCTATTGATCGGGATGGCTTACAAGAAGAATGTGGGGGATACACGAGAGAGCCCGTCGCTACGGCTAATCG
ACCTGATGGAGCGACGGGGCGCAGTCGTTGACTACCACGACCCTTACGTACCTGAGATTCCCGCCACAAGAGA
GTATGCCGAGTTAGCAGGTCGGCGCTCGGTGGATATTTCAGTTGACCGCCTGGCCTCGTTTGATGCCGTTGTC
ATTACGACAGATCATGATGCCATTGATTGGAAAACCGTCGTTGAAGGTGCAAAGCTGGTGGTCGACACCAGAA
ATGTCTGTGCACGCTTCCATGTGAGCGCCCCTAATGTGGTAAAGGCATAAATGCGTATCGTCATCGGTTCCGA
CCATGCTGGCTTCCACCTCAAGGCCACGATCATCGACCATGTCACCTCGCTCGGCCACGAGGTGTACGACGCA
GGCTCATACGATGCGAACCCCGTAGACTTCCCCGACATCGCAAAGATGGTCACTTCTTCGATCATCGAGGGCA
AGGCGGATCGCGGGCTGATGGTCTGCGGCACCGGCGTCGGCGCCTCGATCGCGGCAAACAAGGTGAAAGGTAT
CCGCGCAGCGGTCTGCCATGACGTGCATTCCGCCCATCAGTCGGTGGAGCATGACGACGTCAACGTCATGTGC
ATCGGCGCCCAGATCGTCGGCGCATGGTTGGCCAGGGATCTCGTTGCAGCCTACCTCGCTGCCGAATTCTCCA
CCGACGAGGACTTCCGGCGCCGCGTGCGCAAGCTTGCCGAAATGGACGAGCAGCGCTGAATGGAAGCTCTTGC
AGCCCTGGCGCGAAACTGCATCGTCACGCTGTTGTGCGGTTGCGCGCTTTTCGCACCTTCGCTTGCTGCCGAG
GAGGCCGATGATGTCGCGACCGGGACGCGGCTTGCCGAGCTGTTGCGTGCCGCTCGAAACGTTCTTTCGAATT
ACCAGTCGCTCATCAATGACCCGGCCCTCGGAGACAAGAAACTCGACGGTGAGCGTTTCACTGCCGAAGCGAT
CGCCCTTTATGGCGAGCGCACCGGCCATCCGTTGATGTCGGATGATCTCGGCGATCGCGACCGCAAGCTTCTG
CAGGCGCAGGTCGAGGCGATGCGGGAAGTCGTGAATGAGCAGCAGGACGACATAAACCGCCCCGGGATCGGGT
TCAAAGGTTTCGTGCCGGCCATTTTCGCGCGTCTGATGAACGAGAAATTTGCCGTCAAGGCGGGCAATGAGGC
GCTGGTCCGCGTTACGGCGCCGGAGGTTCTGGTGCGCAACCGCAAGTCGCTTCCCGATGCCTGGGAGGCGCGA
GTCATCAACGAGGTCTTCTCGGATCCGCAGAGGCCGAAGGGTGAACTCTATACGGAAGTGACGCAGGTGAACG

GCCGCCCGGCTTTCCGGATGCTGCTGCCGGAATATTACACGGAGTCCTGCCTTTCCTGTCACGGCTCGCCGAA
GGGTGAAATCGACGTCACCGGCTACCCGAAGGAAGGCGGCAAGGCGGGCGATCTGGGCGGTGCCATCAGCATC
GTCCTGTTCAAATGAATGATTTTCACCCCCAAAGGAAAACACCTCGTCGCGGGCGAATGGCTCGATGGGGCAG
GCACCTTCGCTTCGGCGCCGGCGCATGGCCCGGCGCATGATTTTGCCGTAGGCACCGTAGAGCTGGTGAACCG
GGCCTGCGAGGCGGCTGAGGAAGCCTTCTGGACCTATGGCTATTCCTCGCGCAAGGAGCGCGCCGCCTTCCTG
CGCGCCATCGCCGACGAGATCGAGGCCCGGGCGGAGGCGATTACCGAGATCGGCAGCCAGGAAACCGGCCTGC
CCGAGGCGCGGCTCAACGGCGAACGGGGCCGCACGACCGGCCAGCTCAGGCTCTTTGCCGATCATATCGAAAA
GGGTGATTATCTCGACCGGCGCGTCGATGCGGCGATGCCCGAGCGCCAGCCGGCGCCCCGTCAGGAGATACGG
CTGGTCCAGCGCCCCGTCGGTCCGGTCGCCGTCTTCGGCGCCTCGAATTTCCCGCTGGCCTTTTCGACCGCCG
GCGGCGATACGGCCGCGGCGCTCGCAGCCGGCTGCCCGGTCGTGGTAAAGGGTCATTCGGCGCATCCCGGCAC
CGGCGAGATCGTCGCCGAGGCCGTCGACGCAGCGATCCGCAAGACGGGTGTTCATCCCGGCGTATTCTCGCTG
ATCCAGGGCGGCAGCCGCGACGTCGGTCATGCGCTGGTGCAGCATCCCCATATCAAGGCCGTAGGCTTCACCG
GTTCGCTCGCCGGCGGACGTGCCCTGTTCGATCTTTGCGCGGCGCGCCCCGAACCGATCCCGTTCTTCGGCGA
ACTCGGTTCGGTCAACCCGATGTTCCTGCTGCCGGAAGCGTTGAAGGCACGGGCGGAAACGCTGGGGCAGGGA
TGGGCCGGTTCGCTCACCATGGGCGCCGGGCAGTTCTGCACCAATCCCGGAATCGCCGTCGTCATCGAAGGTG
CGGACGCCGACCGCTTCACCACCGCCGCCGTCGAGGCGCTTGCCAAGGTGGCACCGCAAACCATGCTGACGGA
CGGTATCGCCAAGGCCTACCGCGACGGTCAGGCGCGCTTTGCGACGCGCAACGCCGTCAAACCGCTGCTTGCG
ACCGAGTCGTCCGGCCGTGACGCATCGCCCAATCTCTTCGAGACGACGGGTGCCCAGTTCCTGGCCGACCATG
CGCTCGGCGAAGAAGTGTTCGGCCCGCTTGGACTGGTGGTTCGGGTGGGATCGCCGGCCGAAATGGAAGAGCT
CGCGCGCGGCTTCCAGGGCCAGCTGACCGCAACCATCCATATGGATGCGGGCGATCTGGAGACGGCGCGCCGT
CTGCGTCCGGTGCTCGAGCGCAAGGCGGGCCGGGTGCTGGTCAACGGCTTCCCGACCGGGGTTGAAGTCGTGG
ATTCGATGGTGCATGGAGGCCCCTATCCAGCCTCGACCAACTTCGGCGCAACCAGCGTCGGCACCATGTCGAT
TCGACGTTTCCTGCGTCCCGTCGCTTACCAGAACATGCCGGAAGACCTGCTGCCCGAGGACTTCCTCGGCTGA
ATGCGCACTCGCGCTGCTCTGGCCGTTCAGGCCGGAAAACCCCTTGTCGTGACCGAAGTCGAGCTCGAAGGCC
CGCGGGCCGGCGAGGTGCTGGTCGAGGTGAAGGCGACCGGCATCTGCCATACCGACGATTTCACGCTGTCGGG
CGCCGATCCGGAGGGGCTGTTCCCGGCGATCCTCGGTCATGAGGGCGCCGGCATCGTCGTCGATGTCGGCCCC
GGCGTCACCTCGGTGAAGAAGGGCGACCACGTCATCCCGCTCTATACGCCGGAATGCCGCTCCTGCCCCTCCT
GCCTCAGCCGCAAGACCAATCTGTGCACCGCCATCCGCGCCACCCAGGGGCAAGGGCTGATGCCGGACGGCAG
CTCGCGGTTCTCGATCAACGGCGACAAGATCCACCATTATATGGGCTGCTCGACCTTTTCGAACTTCACCGTG
CTGCCGGAGATCGCGCTCGCCAAGGTCAATCCGGACGCCCCCTTCGACAAGATCTGCTATATCGGCTGCGGCG
TCACCACCGGCATCGGCGCGGTGATCAACACCGCCAAGGTCGAGATCGGCGCCACCGCCATCGTCTTCGGTCT
CGGCGGCATCGGCCTCAACGTCATCCAGGGGCTTCGCCTTGCCGGCGCCGACATGATCATCGGCGTCGACCTC
AACAACGACAAGAAGCCCTGGGGCGAAAAGTTCGGCATGACCCACTTCGTCAATCCGAAGGAGGTCGGCGACG
ACATCGTCCCTTATCTCGTCAACCTGACGAAGCGCGGTGCCGACCAGATCGGCGGCGCCGACTACACCTTCGA
CTGCACCGGCAACACCAAGGTCATGCGCCAGGCGCTCGAAGCCTCGCATCGCGGCTGGGGCAAATCGGTGATC
ATCGGCGTCGCCGGCGCCGGCCAGGAGATTTCGACCAGGCCGTTCCAGCTCGTCACCGGCCGCACCTGGATGG
GCACCGCGTTTGGCGGCGCGCGCGGCCGCACCGATGTGCCGAAGATCGTCGACTGGTACATGGAGGGCAAGAT
CGAGATCGATCCGATGATCACCCACACCATGCCGCTCGAGGACATCAACAAGGGTTTCGAGCTCATGCATTCC
GGCGAGAGCATTCGAAGCGTGGTCCTCTATTGAATGAACACATATGAAAGGATCGATAGAGACCTGTCCGACC
GGGACTTCACCGTCGTGGGCAAGAGCGTCAAGCGTTCCGATACTCTGGAGAAGGTCACGGGGACGGCCAGATA
TGCAGGCGACGTCGCCTTGCCCGGCATGCTCCACGCCAAGATGAAGCGCAGCAACATCGCGCATGCGCGCATC
AGGAGCATCGACACGAGCAAGGCGCTGGCGCTCAAGGGCGTCAAGGCGGTGCTGACGCACGAGGACGTGCCGC
GCGTTTTGCATGCGGGCTCACCGCATCCGCGCTCGGCATCCGTTACCAAGGATCAGTACATCCTGGATGACAG
GGTGCGATATTGGGGCGAGGGCGTCGCCGCAGTCGCTGCCGTCAGCGAGGAAATCGCTGAGCGCGCGGTCGCG
CTCATCGAGGTCGAGTACGAGCCCTTGCCCGGCCTCTTCACCATCGAAGCCGCATCGGTGCCCGGCGCTCCGC
CGATCCATGAGAATGGTCTCGACCGGAACCAGGTGTTGCCGCCGGTCTTCGTCACCCGCGGCGACGTCGACAG
GGGTTTCGCCGAAGCGGATCTGATCCTCGAGCGCGAATACGATCTCGGCCGCCCGACACCGGCCTATATGGAG
CCCAATGTCTGTGTCAGCCAGTGGGACGGCAACGGCAAGCTCACCATGTGGACCTCGACCCAGAGCGCATTCA
TGGTCCGCGGCACGCTTGCCGAAGTGCTCGGCGTGCCGCTGAACAAGGTACGCGTTATCGTCGATCACATGGG
CGGCGGCTTCGGCGCGAAGCAGGATCTGTTTCAGAACGAGTTCCTCTGCGCCCTGCTCGCGCGACAGACGGGG
CGTCCGGTCAAGATGGAGTTCAGCCGCAAGGAAACGTTCCTCGGCGGCCGCTCGCGACACCCCGGCAAGATAT
GGCTGAAGCAGGGCTTCACCAAAGACGGCCGGATCGTCGCACGCGAAGCCAGGGTCACCTTCAACTCCGGCGC
CTACGGCTCGCACGGTCCAGGCGTCACCAATGTCGGCACCGCAGCGCTGACCTCGCTCTACCGTTGCGAGAAT
GTCCGGCTGGAAGGCCGCTGCATCTATACCAATTCGCCGATCGCCGGCGCCTTTCGCGGCTATGGTGTGGTTC
AGACCTACTATGCGCTCGACCTCATGATGGACGAGGCGGCCGAGAAGCTCGGCTTCGACCCGGCCGAGTTCAA
GTTGATGAACGCGGTGCGCGAGGGCGACATCGCACCCTCCGGGCATCCGATCGTCGGACACGGCCTCGGGGAT
TGCATCCGCCGCGTCATGGAGGAGACGAACTGGCACGAATTGCGCAGGCGGGAGAAGCCGGAAACCGTCAAGC
GCCGCGGGATCGGGATCGGCTGCGAAATGCACGGATCCAGTGCCTATCCGGGCATCAAGGAACAGGGCAACGC

AATCGTGAAGATGAACGAGGACGGAACGGTGACACTGATCACCGGCACCGCCGGCCTCGGCACCGGCGCGCAT
ACAGCGCTTTCGCAGATCGTCGCCGAGGAACTCGGCGTGCCCTTCGAAGCCGTCTCGGTCGTTCAAGGGGACA
CCGATATCGTCCCCTGGGATATCGGTGCCTTCGCCAGCCACACGACCTATCTCGGCGGACGGGCGGCTCAGCT
CGCGGCCGCCGACGTGCGCAGACAAGTGCTCGAACACGCCGCGCCCATGCTCAAGACCGAGCCCGAGAATCTC
GCGATCCGGGACGGTTTCGTGGTCGTCACCAACGGCTCCAACCGCAGCATCCGGGTTTCCGAGGCCGTCGGGC
CGCAACGGGGGATGCCGGCGGTGCAACTAGTCGGCGTAGGCACCTACATGCCGACGAAGTCCTACTCCTTCGC
CGCCCATTTCGCCGAAGTCGAGGTGGACACCGAGACCGGCGAGGTGGCCGTTCTCGAGGTCGTTCCGGTACAT
GAGATCGGCAGGGTCATCCATCCGATCGCCGCGGCCGGACAGATCGAGGGTGGCATTCAGCAGGGCATCGGCC
ACACCCTCAGCGAGGATTACGTCATCGACCTTACCGACGGACGTTCCCTCAATCCGAGCTTCGTCGATTACAA
GATGCCCCTGTCGATGGACATGCCGTCCATCCGCACCATCATCCTCGAGACGGCACCGGATCCCGGCGGTCCC
TACGGTGCCAAGGGCGTCGGCGAGGATCCGATCATCGCGATCGGGCCCGCCATCGCCAACGCCATCTACGACG
CCATCGGCGTCCGCTTCCACCACTACCCGATAACGCCCGAGCAGGTGTTGAACGCTCTCAAGTCCAAAGCCAA
CGAAACGAGGCAGTGAATGGCAAACAGTGAAAGTGGACAGCCGCTGGCGGTCGTCCCCCAGGCCTATGGCGCC
CTTTGGCGAGGCCTGCTCGGTCCTTTACGCACGATTATCCAGCCGGCCGCCGCTGTTGTTCTGGCTCTCGCAT
GCGGCGCGGCGCTCCTTGCCTTCAACGGTTACGAAGCACGCGCCGTGATCGATGTGATGGTGCTTGGCGTCTT
CCAGGACACCCGCTCGATCGCCGAAATTCTCCTGAAAGCAACGCCGCTGATCCTGATCGGCGTCGGCCTTTGC
GTCGCCTTCCGCTGCAGCATCTGGAACATCGGCGCCGAGGGGCAATTCTATGCCGGCGCCATCGCCGCAACCG
CCGTCGGCGTTTCCTTCGACGGGTTGACCGCGTGGATCTACGTCCCGCTGGTCATGATTGCAGGCGCTCTTGC
AGGTGCGGCATGGGCGGCGATCGCCGGACTTCTGAAGGTCTATTTCCGGGCCAGCGAGATCGTGACGACGATC
ATGCTGAATTACATCGCCATTATCATGACGAGCTATCTCGTGACCGGCCCGCTGCGGGATGCGGCTGCGGCCT
ATCCGCAATCGGCCCGGCTGGTCCAGGAGGCATGGATGCCGCGCATCCTGCCGCCGACCCGCCTCCATATCGG
CATCCTCGTCGCCCTGGCGCTGGCCGTCGTGATGTACATCTTTCTGTTCCGCAGCACCCGGGGCTATGCGCTG
AGGGTGGTCGGCATCAGCCCGGACGCCGCGCGCTATGCCGGGATCAGCGTTCCCAGGAACATCATGCTGTCGA
TCTGCCTGAGCGGCAGCATGGCCGGGCTCGCAGGGGCCTTCGAGATCGCCGGCGTCACCCGGCGGCTCTTTCA
GTCGATCTCTCCGGGCTATGGCTTCGAAGGCATCGCGGTCGCTCTTCTCGCAAACAACAATCCGATCGGAGCG
GTCTTCTCCGGCCTCTTGTTTGCAATTCTGCGAAGCGGCTCCGAGCTCATGCAGATCACGGCACAGGTGCCTC
AGGTTCTCGTGCTCGTGATTCAGGGCATCGTCATTCTCTCGGTCGTGGGATTTGCCGTGTTGCGGATCCCGTC
GATGCGCCCTTCCGAATAGATGATTGATTTTTCCCGTGCCCGCGAACGGATGATCACGTCGCATCTCTCGCGA
CGGGGGATAAGGGACAGACATGTCGTGGAGGCGATGGGGGTCGTCCCGCGGGAGGCATTCGTCGATCCCGGCT
TCGAGGAATTCGCCTATGAGGATTCGGCCCTTTCGATCAGCCATGGCCAGACCATCTCGCAGCCCTATATTGT
GGCGCTGATGATCGAACGCGCCGAAGTCCAGCCGGGCGATACGGTGCTCGAGATCGGGACCGGATCCGGTTAT
GCGGCGGCGGTGCTCAGTCGGATCGCGGCATATGTCTATACGATCGAGCGCCATGCCGGCCTCGCCGAGGTTG
CCGAGAGACGATTTGCCGAGCTCCGTTACGGGAATATCGATGTGCGTGTCGGCGACGGCACGGCCGGCTGGCC
CGAGGCAGGACCCTTCGACGCCATACTGGTTGCCGCCGGAGGTCCCGAGATACCGTATGCGCTGAAGGAGCAG
CTCGACCTCGGTGGCCATCTCGTCATACCCGTCGGCCCCCCTGAGGAGCAGCGCCTGATGAAGGTCACGCGGG
TCAACGCCACGACATTCGAGGAGCAGGACCTCGGCGGGGTCAGATTCGTGCCGCTCGTCGGCGAACACGGCTG
GCATGAGGAGCGGGTTCAATCCGGGGCAAGGCTCAGAGCGGCGCCCACCTTGCCCGAGCTGGTTGCAGAGGCG
GCCGAGCCGCTGCCCGATCTCGACGATCCGGCGTTCGGGCTGCTTTTCGATCGCTTTGCCGGGCGGCGGATCG
TGCTGCTCGGAGAGGCGAGCCACGGCACGTCGGAATTCTACCGCGCCCGCGCAGCCATCACGCGCCGCCTGAT
CGAGGAACACGGGTTCACGATCGTGGCGGTCGAGGCGGATTGGCCGGATGCGGCGGCAGTCGACCGCTATGTC
CGACATCGAAGTCAAGGCATGCGCCTCGACGCGCCGTTCCAGCGGTTCCCGACCTGGATGTGGCGCAACCGCG
AAGTGATGGATTTCGTCGAGTGGATGCGCGAACATAACGGCCGCAGGAGCTTGCCTGACCGGGCCGGCTTCTA
CGGTCTCGACATCTATAATATGCGCGGCTCCATTTCCTCGGTTCTCCGGTATCTCGATGAGACCGACCCCGAG
GCCGCCGCGGTCGCCCGCGAGCGCTACGGTTGCCTGACCCCCTGGCAGAACGAGCCGTCGACCTATGGCCGTG
CGGCCATGACCGCGGGCTTCCGCAAATGCGAAGAGGCCGTCGTCAGGCAATGCCGCGAACTCCTGGAGAGGCA
GCTCGAAGCCGGGCGCGACAGCGGCGACGAGCTTCTCGACGCCGTGCAGAACGCGCGCCTCGTCGCCTCGGCC
GAGCGGTACTACCGTATCATGTATTATGCTGGGGCCGACTCCTGGAACATGCGCGACACCCATATGTTCGAAA
CGCTCGAGCATCTGTTGAATGCACGCGGCGCTCGCTCGAAGGCGGTCGTGTGGGCGCACAATTCCCATATCGG
CGATGCCCGCCATACCGATATGGGCACGGCCCGCGAGGAGTTGAACATCGGTCAGCTCTGCCGCGAGCGGTTT
GGCGATCAGGCAGCGCTGATCGGCTTCGGCACGCATGGCGGCCATGTGGCTGCCGCAAGCGATTGGGACGGCG
ATATGGAGGTGAAGCCGATCCGGCCGTCGCTCGAAGGAAGCTACGAGCGCGTGATGCACGACTCAGGGGTAGG
GTGCTTCCTCATCGACTTCGCGCGCCACGCGCGTCTGCGCGACGGTCTCCTCAAGCCGATGCTCGAGCGCTTC
ATCGGGGTGATCTACCGGCCGGACACGGAACGCTTCAGCCATTATGCCCACGCTTCGCTGCCCCGGCAGTTCG
ACGCCTTCGTCTGGTTCGATCAGACGACGCCGGTCGAACCGCTCGGCGCGCAACACATAAAGGCGGGCGTGCC
GGACACGTTCCCGTTCGGCCTGTGAGTGAGCCGCGTGCTGACCCTGGCCGGGCGAACGGGGACGGGACGCGTC
GGCGCGGCTCTCGCCGCGCTCGTCATCCTCTTCGCAGCACTCTGGATGGGCGCTGCGATCGGCGAAACGGCGA
TCCCGTTCAAGACCGTTGCGCAGACGGTGGCGAACCGGCTGTGGAACGCCGGCTACCCGCTGGCACCGATCGA
CGAGGGCATCATCTGGAGCTACCGGCTGAGCCGGGCAGTCGTGGCCGCTTCCTGCGGGGCGTCGCTCGCGCTC

TCCGGCGCCGTGCTGCAATCGCTGCTGCGCAATCCGCTCGCCGACCCCTACATTCTCGGCATCTCCGCCGGCG
CCTCGACTGGCGCGGTCAGCGTCGCCATTCTCGGTGTGGGTGCCGGGATGCTGACGCTGCCGCTCGGCGCCTT
CGTCGGCGCGCTGGTCGCTTTCGTCCTGGTGAGCCTGCTGGCGGTGAAGGCCGGCCGCGGCACCGCGGCGATC
ATCCTGGCAGGTGTGGCGGGATCACAGCTCTTCAACGCGCTGACCTCCTTCATTGTCACCAAGGCGGCGACGG
CCGAGCAGGCGCGCGGCATCATGTTCTGGCTGCTCGGCAACCTTTCGGGCGTGCGCTGGCCGGATGCCTGGCT
CGCCGTGCCGGCGACGCTTCTCGGTCTCGTCGTATGTCTCTGGCATGCCCGTCCCCTCGACGCGTTTACCTTC
GGGTCGGAATCGGCTGCCTCGCTCGGCATCTCGGTCAGGCGAACCTATTTCGCCCTCGTCGGCGTCTCGGCGA
TGATGACGGCGGTGATGGTATCCATCGTGGGGTCGATAGGTTTCGTCGGCCTCGTCATCCCCCATGCGGCCCG
CATGCTCGTCGGCGTCCGCCACGGTGTCCTGCTGCCGGCAGCGGCGCTCATCGGCGCCGTATTCATGATCCTC
GCGGACATCCTGTCGCGCGTTCTCATTCCCGGCCAGGTCTTGCCGATCGGCGTGATCACGGCGCTCGTCGGCG
CCCCGGCCTTCGCGTTGATACTCGGACAAAGGAGGGACCGCGCATGAATGACCATCGAAGCCGAAAGCACTCT
TCCGGTGGCGATCGAGCCCGAGGAAAAGCACCACCACGAGAGCTACTCCGCTCTCGTCTGGCGCCGGTTGAAG
CGTTCCTGGACCGGCCTGCTCGGCCTTGTCCTCGTCTGCCTCCTGATCCTGATGGCGGTCTTCGCCGAGTTCC
TGTCGCCCGTCGATCCGAAGGCGACGGACGTAGCCTTCGCTCAGCCGCAGACGATCAGCTTCCGGGACAAGGA
GGGTAATTTCGTCTTTCCGCCGAGGAGCTATCCGGTGCGGGAGACGGAGGAGCTCGATCCCGTTACCTTCCAG
CCGATCATCGGACCGGATTACGACAACCCGCAGGTCCTCGGCTTCTTCGTCAAGGGCGCACCCTACAGGCTCT
TCGGGCTGATCCCGGCCGAGCGGCATCTGTTCGGTGCCGTCGACGGTACGCCGGTGCATCTGCTCGGCACCGA
CAAGTTCGGCCGCGACGTGCTGTCCCGCATCCTCTACGGCTCGCGCATCTCGCTGATGATCGCGCTCACCGTC
GTCTTCATCGTCACCGTGGTCGGGACCACCGTCGGCATGGTGTCCGGCTATTTCGGCGGGCGCTTCGATGCCT
GGGTGCAGCGTTTCGTCGAACTTGTGCTCGCCTTCCCGCAATTGCCGCTCTATCTGGCGCTCGCCTCGCTGAT
CCCGGTCACCGCGCCGACCAATGTCTTCCTCGCCTTCGTCATCATCGTGATGTCGGCGCTCGGCTGGGCGCAG
ATGTCGCGCGAGGTGCGCGGCAAGACCCTGGCGCTTGCGCGGATCGAATATGTGCGGGCGGCGATCGCCATCG
GCGCGACGGACCGGCGCATCATATTCCAGCACATCTTCCCGAATGTGATGAGCCACGTCATCGTCGCGGTGAC
GCTCGCCATCCCGCATGTGGTGCTGCTCGAGTCTTTCCTCGGTTTCCTCGGCTTTGCGGTCAAGCCGCCGCTG
ATCTCCTGGGGGCTGATGCTGCAGGATACGGCGAATTATTCCGCGATTGGTTCCTATCCCTGGATTCTCTCGC
CCGTCGCCTTCGTGCTCGTCACCGTTTTCGCCTTCAATGCGCTGGGCGACGGCCTGCGCGACGCAATCGACCC
CTATTGAGTGAAGCTCGATCCCTTCTATCTCATCGTCGACAGCGCCCAATGGATCGAACGGCTGGTGCCGCTC
GGCGTCAAACTCGTGCAGCTCCGCATCAAGGACCGGAACGAGGAAGACATCCGCCACCAGATACGGGTCGCCA
GGGCAGTCTGCTCCGCCCATGCATGCCAGCTGATCGTCAACGACTACTGGCAGCTGGCGATAGAGGAAGCCTG
CGATTTCATCCATCTCGGTCAGGAGGATCTGGCGGAGGCCGATCTTGCCGCCATCCGTGCCGCCGGGCTCAAG
CTCGGCGTCAGCACCCATGACGAGGCGGAACTGGCGAAGGCGCTCGCAGCCGAACCGGACTACGTCGCGCTCG
GGCCGATCTATCCGACGATCCTCAAGAAGATGAAGTGGGCGCCGCAGGGGCTGGAGCGGCTTTCATGGTGGCG
CGAGCGCGTTCACCCGCTCCCGCTCGTCGCCATCGGCGGCCTCAATACGGAGCGGATCGAAGGCGTCTTCGCG
CATGGCGCCGACAGCGCCGCCGTGGTGACTGACATCACGCTGAATGCCGATCCGGAAGGAAGGACAAGGGAGT
GGATACGGAAGACGGAGGCGTGGAGATAGGTGGGGCAAGCTGAGGCAGCAACGGGAAAAAAGAGACGCAAGGC
GCTTTGGGCCGCAGTTGCGGTGATAGCAATCACCGCCGCCGGCATGGCCTGCTACAAGACCATCGTAGAAAAA
CGCGTGAGCAGGATGATCGCCGAGCGCGGCGGCAAGGCGGGCAGCGTCGAAGCGGATTTCCTTGGGCGAATTC
ATCTGCGCGACGTGGCCCTGCCGCTGAAGGACGGGTCGGACGTTCGTATCGCCGCGGTTGACGGCCGGCCCAG
TATCTTTTTCCTCCAGGGCATGCTCGAAGTAAGCGGCCTTGAACTCAAGACTGCTTTCGGCACGGTCTCTGTC
CCGCGCGCGGTCGTCGAAGAAGCAGATTTTGATCGCGGCACCTTGGAGCAGATGTTCGGCGGGAAGAGCGAAC
TCTCGATCTCCAAGCGAGTAGAACTTTTCACGGCAAGGCGTATCTCCGCCCCGGAGATCGTCTTCGCACAGAT
CGTTGCCGGGACCGAGCAGAAGCTGATCTACAGGAACGTGGTCTCGGAAGGCATCGCCGATGGGCGTATCGCC
CGCTATTCGGCGAGCGGCGGCAGCTTCGAAGTCGGCGTGGACCGTTCGGGGGGAGAGGGCGGCAGGAAGAATA
GGGTGGCGGGTTCCATCGGAGCCGTGACGGCGCAGGATATCGATGCCGCCTATCTGGCGCGGCTCTATACCGA
AAAAGCCGGCTCGGACGACAACGAGGCAAAGCCTGTCTATGGGCCGATTTCGGTAAAGGACATCGCCTTCTCG
GATGGCGAGGCGAGCTTCGGCTATGACGAGGTTCGCAGCAGCGGCTTCAGCATGCGAATGCCGGCCGAGCCGC
TGGTCGAGACGCTCAAAAAGCTGAAATCGGTCTCGGCGCCGGATCAATTGCCGCCGGCGGAACGCCAAGCCTA
TTTCAATCGGGTCTTCTCGATTATCGACATGACGAAGAAGGCCGACATAGAAATGCTCGGCCTTACGATCGAC
GCGCCGATGAAGGACGGTGACGAAGGCGAGAGGATCAAGGTTTCGATAGATCGCCTCGCCCTCGAGGTCGATG
GACGGAAGCTTGACGGCACGCTGAATGGCCTCTCGATGGGAGAGGGCGCGGACTACGTCAAGGTCTCCGAAGC
GAGCATTTCGGGATTTTCCTGGAATTCGACCTTGGAGGGTCTGCAGAGGATGGTCGCGCTCGACGAGCAGCAG
CGCGAGAGATTTGCCTTCACGACACTGTTGCCGGAATTCGGTACGGTCCGGCTGGCGGGTCTGGATGCCGATC
TGCCCGATACAGAAACCGAAGACGCCGAGAGCGACACACAGGATTCGGTGCCGAGGCGCGTTCGCTTCTCGCT
CAAGGATTACGAGTTGGCGCTCACGAAGCCACGCAACGGCATTCCGACCGACATCCGCATCAGATATCAGGAT
CTGACCCTGCCGATCCCGGAGCATATGCACGACGAGCCGTTCGCGCAGCTTCGCAACCTCGGTTTCAATGAGG
TGGTCCTGTCGTCCAACATCGAAGCCGCCTGGGACGAGCCGAACCAGACTCTGGTCATCAAGGATATCTCCGC
ACGTGGAAAAGATATTGGCGGCTTTTCCCTCTCCGGCCTGCTGGGGGGCGTGAGCGGCGACTTCTTTTCCGGC
GACGAGGCGAAGATGCGGGCAGCGCTGTTCGGCCTCACCGCCCGTGAAGCAAAGCTGAAGATCGAGGACAAAG

GTCTCGCCGCCAAGGCCATAAAGGCCTATGCCGAGCAAAACGACATGACGGAGGATCAGGCGCGCGGCATGCT
CACGATGACGGCTTCGGTGGCGTTGCAGCAGTTTGCCGCGCAGGAGCCTCGGTTGCAGGACGTGCTGGACGCA
GCTTCGCAGTTCATTGCCAAACCGGGGATGTTCACGTTGACGGTCAAATCCAAATCCGCAAACGGAATCGGTG
CGCTCGAATTCGCGGTTGCTTCGCAAAATCCGCTCTTGCTTCTCGACAGGGTCGACTTCGAGGCGACGGCGGA
ATAGATGATCACCTCGTTTCTCGTCAAATCGCTGGATCGGACGATCGTCGTCGCTGTCGCGATCCTGCTGGCG
CTGGCCGTTATCGTTCCCGCGCTCAACCTCCTGACCGCGCCGGAACACCCGCTGCATGTGCCGACCTATCTGG
TCTCGCTCTTCGGCAAGTACCTGACCTATGCGCTCTTGGCTCTGGCGCTCGATCTTGTCTGGGGTTTCTGCGG
CATCCTCTCGCTGGGGCATGCGGCCTTCTTCGCGCTCGGCGGCTACGCCATGGGCATGTATCTGATGCGCCAG
ATCGGCGCACGCGGCTCCTACGGCAATCCGCTGCTGCCGGACTTCATGGTCTTTCTCAACTGGAAGGAGCTGC
CCTGGTTCTGGTACGGCTTCGACATGTTCTGGTTCGCAGCGCTGATGGTGGTGCTGGTGCCGGGCCTGCTTGC
CTTCGTCTTCGGCTGGTTCGCCTTCCGCTCGCGCGTCAACGGGGTCTATCTCTCGATCATCACCCAGGCGATG
ACCTATGCGCTGCTGCTCGCCTTTTTCCGCAACGACATGGGCTTCGGCGGCAATAACGGGCTCACCGATTTCA
AGGACATCCTCGGCTTCAACATCCAGGCCGACGGAACGCGTGCGGCACTCTTTGCAGCCTCCGCGCTGGCGCT
TGCCGTTTCGCTCGTCGTCACCTCGGGCATCGTCCGCTCGAAATTCGGCAAGGTGCTGGTGGCGCTGCGCGAC
GCGGAAAGCCGCACACGCTTTCTCGGCTATCGCGTCGAGCACATGAAGCTCTTCGCCTTCACGGTCTCGGCAA
CGATGGCGGGCGTCGCCGGCGCGCTCTACGTGCCGCAGGTCGGCATCATCAATCCGGGCGAGTTCGCCCCCGC
CAATTCGATCGAGGTGGTCATATGGACGGCCGTCGGCGGGCGCGGCACGCTGATCGGTCCGATCGTCGGCGCG
ATCCTCGTCAACGGCGGAAAAAGCATCTTCACCGCCGCCTTCCCCGAATTCTGGCTCTTTGCGCTCGGCGGGC
TCTTCGTCCTCGTCACGCTTTTCCTGCCGAAGGGCGTGGTCGGCACGGCGCAGAACTATCTCGCAAGCCGGCG
CGCATCACACGCGGCGGCCCGGAAGGAAAGCGAGAGCGAAAAAGCGGCGAGCCTTGCGGACGCCGAAACGGTG
GCCGCGGAGTGAATGACGACATCAGGAGCGGACGGAACGAAGGAGCGGCGCCCTGTGATTGCGCTGGCCATGG
GCGACCCCGCGGGAATCAGCCCGGAACTGACGGCGCGGCTGCTGGCCCTTTCCGACATCCGCGACGCGGCGCA
CATCATCGCCATCGGCGATCGCCGCATCCTCGACGAGGGCGCCCGGGTGGCCGGCGTCACGCTGGATCTGGAA
GCCGCTTCGCTCGATGCGCTCGACAACGCCGGCACCGCCCGCCACGTCTTCGTGGATCTCGCCCATCTCGATC
CGGCCGATGTGGTGCGCGGCGAGGCGACGCTGGCCGGCGGCACCTTCGCCACACGCAATTTCCGCACCGCGCT
CGAACTCGCCCATGCGGGCAAGGCCGATGCCGTCTGCTTCACGCCCTTCAACAAGAAGGCGATGCGCTTTGCC
TATCCCGGCTATGACGACGAGATCCGCTTCGTCGCCGACGTGCTGTCCTTCACCGGAAAGGTTCGTGAATTCA
ATGTTCTCGAAAAGGTGTGGAACGCGCGGGTGACGTCGCATATCCCGCTCAAGGACGTGGCATCCCACCTGTC
GGTGGAGGCGATCCTTGCCGAACTCAAACTGACGCAGGCCTGCCTCAAGGATGCCGGCTACGAGGAGGCGAAG
ATCGCGGTTGCGGGACTCAATCCGCATGCCGGCGACGGCGGCAGCTTCGGCATGGAAGAGATCGACATCATCG
AGCCTGCGGTCGAGAAGGCCAGGGCGCTCGGCTTTAATGTCGAGGGGCCGTTCCCTGCCGATACGGTCTTCCT
CCGGGCACTGAAGGAAGGCTTCAACGCGGTGATGACCATGTATCACGACCAGGGTCAGATCGCCATGAAGATC
ATCGGCTTCGACAAGGGTGTGACGATGATGGGCGGACTGCCGTTCCCGCTCTGCACGCCCGCCCACGGCACCG
CCTACGACATCGCCGGCAAGGGTATCGCCGATATAGGCGCAACACGCGAGGCGATCCTGCTTGCAGCCCGCAT
GGCGAAGAAAAAGAGGGCGCTTTCGGCGGCTGCCTGAATGTGGGGAGAAACCGGTTGGGGCATGCCGACGTCG
CTCGCCCTGCACCTGGCGCTCGCCTTCTTGCTGCTGGTTCGGCTGCCGGAATTGTCTGCGCCGGCAAAGGAAC
AAAGCGTCAGCGTCGAGCTCGTTCCGCAGCCGAAGAGTGCGGAAAAGCCCGAGCAAAAGCCGAACGCGGCCAA
GAGCGAGCAGGCGCGCCCTCAGCCTGAGGCGTTCGAGTCGGCGGCAGCGCCGGCCGAAAAGGAAAAGCCGCCT
CGGCCCGAGTTGCCTCCGGCCGCGCCGAAGAAGACCGAAACGCCGAGCGAGACGCCGGAGGCCTCGCGGCCCG
CCCCGGCAAAGACGGAGAACTCCGCCAGCGAAAAGCCGGCGGAAAGCAAGACAACGCTTGCGGAATTGCGCGT
CGATGCCCGGAGCGCAGCGAACGAGGCGGCCGATGCTGCCGCGGCGCCGCCGGCTGCAGCTGTTCCCCAGGAG
AAACCGGTTCTGGAGGAAGCAAGGGCGGAGCCGACGCAAAAGCCGGCCGAGGCGAAGGCCGAGCGCCAGTCGA
GCCAGCTCGTCCAGGCGAAGGAACTCTATTCTGAAAACGCCCTGTCGGACCCGCGCGTGAAACAGGCGATCGG
CAGGCTGCCGCCGAAAAAGCGGATCGTACAGCTCTGCAGCATCGAGGCGCTCGAGCAGGTGCGGCGGGAAATG
CCCGGCGCCTTTCCGGACATGCTCGTTCCCTTCGGCCCTTCCGGGGGCTTCATCTCGGAAACGGGACTAAACG
CCGATGGCGGCGCCTTCCGCAGCCGGTCGACATGGTACGCCATCGACTTCAAATGCGAAGTGAGCCCCGAAAC
GACATCGGTCACTTCCTTCAGCTTCGCGATAGGCAAGGCGATCCCGAAGAGCGAATGGGGTGCGCGACAACTG
CCGAGCAATTAAATGCAAGACGCATCGATACCGCGTCCGCGGATTTCCAATGCCATAGACAACGTAGCCCGCG
CGGTGGGCGGCCTGGCCATCGCCACCTTCACCGCCATCATCGTCTACGTCGTCATTTGCCGGTATGCCCTTTC
CTTCACACCACGCTGGTCGGAGGAAATCCCGCGTCTTCTATTGGTTTGGGTGACGTTCATCGGCGCGGTTTCC
GCCTTTGTCCGCAACACGCATCTTTGCGCCGGCCTGACGGACCTGCTTGTTGCGCCCGGCTGGTTCCGTTCCC
TCCTGGCCGTGCTCGCGCGTCTGGCATCCCTGCTGTTCCTGGTCGTCGTCATCTGGTCCGGATGGAAGATAAC
GGTGCTGACATGGTCGCATGAAACCACGGTCCTAAGCTGGCCCGCGGGGCTCGTCTATCTCGCGCTGCCGGTC
ACGGGTGCCATCGCCTTCGTTGCCCTCGTTGCCGCGGAACTGCGCAAATGAATGGACAACATCATTCTTGAAA
TGCGGGGCATTACGAAGACGTTTCCGGGCGTCAAGGCCCTGGACAATGTCTCGTTCAAGGTCCGCGAAGGCGA
GATCCACGCGCTTGTCGGGGAAAACGGCGCCGGCAAGTCGACCTTGATGAAGGTGCTGAGCGGAGTCTATCCG
GCCGGCACCTATGAGGGCGAGATCCATTACGACGGCGAGCTTCGCCAGTTCAGCACGATCTCGGACAGCGAAG
AGCTCGGCATCATCATCATCCATCAGGAACTGGCGCTCGTTCCGCTGCTTTCGATCGCCGAGAACATCTTTCT

CGGCAACGAGGTGGCCGAAAAGGGCGTGATCCACTGGCCGCAGACCTTTGCCCGCACCCAGGAGCTCCTGAAG
AAAGTGGGCCTGAACGAGTCCCCCGCGACGCTGATCACCGATATCGGCGTCGGCAAGCAGCAGCTGGTGGAGA
TTGCCAAGGCGCTTTCGAAGAAAGTCCGCCTGCTGATCCTCGACGAGCCGACCGCATCCCTCAACGAAAACGA
CTCCGACGCGCTTTTGAAGCTGCTCATGGAGTTCCGCAGCCAGGGCATGACGTCGATCATCATCTCTCACAAG
CTCAACGAGATTAAGAAGGTTGCCGACCAGATCACCATCCTGCGCGATGGCGGAACGGTAGAGACGCTCGACT
GTCACAAGGAAGACATCAGCGAGGACCGGATCATCAACGGCATGGTCGGCCGGGCCATGGAAGACCGCTATCC
GCCGCGTGAACCGAAGATCGGTGACACGCTGCTCGAGGTGAAGAACTGGAACGTCTACCATCAGCATCACCGC
GACCGGCAGTTCCTGCATGACGTCAGCTTCAAGGTTCGCGCCGGCGAGGTCGTCGGCATCGCCGGGCTGATGG
GCGCCGGCCGCACCGAGACGGCGATGAGCGTTTTCGGCAAGTCCTGGGGCCACAAGATCACCGGCGAGGTGAC
GATGCATGGCCGGCCGGTGGACGTGAGCACGATTCCCAAGGCCATCAAGGCAGGTCTTGCCTATGTAACGGAG
GACCGCAAGCAGCTCGGCCTCGTGCTGATCAACAACATCATGCACAACACCACGCTCGCCAATCTGCATGCGG
TCGCATCGAACGGCGTCATCGACGAGCGCAAGGAACGGAAGGTCGCCGCCGAATATCGCTCGAAGCTCCGCAT
CCGTTCCCATTCGATCTATCAGGAGACGGTCAACCTCTCTGGCGGGAACCAGCAGAAGGTCGTCCTGTCCAAG
TGGCTGTTCACCAATCCCGAAGTCCTCATACTCGATGAGCCGACGCGCGGTATCGACATCGGCGCGAAGTATG
AGATCTACACCATCATCAACCAGCTCGCGGCCGAGGGCAAAGGCATCCTGATGATCTCGTCGGAGATGCCGGA
GCTGCTCGGGACCTGCGATCGCATCTATGTCATGAACGAGGGGCGCATGGTGGCCGAGCTTTCCAAGGAGGAA
GCAAGCCAGGAATCGATCATGCGCGCCATCATGCGTTCAGGGGAGAAACACTAAATGATCAATGCGGACAGCA
GCCCGCGCCAGCCATATTCGACTTATCATCAATGGTATGCCAGTCAGGACAGAAACCGGCTCATCGCGAAATC
GAAGGAAGCCGAAAAGATTTTTCGTAAGACGGGCATCACCTTCGCAGTCTACGGACACGCGGATTCCTCCGAA
AAACTGATCCCCTTCGATCTCATACCCCGCATCATCTCCGGGCGCGAATGGCGCCGGCTCGCCCAAGGCATCG
AACAGCGCGTCATCGCGCTCAACGCCTTTCTCGACGACATCTACCACAAGCAGGAGATCATCCGCGCCGGGCG
CATCCCGCGCGAACTGATCGAGAAGAACGACGCCTTCCTGCCGCAGATGATCGGCTTCCGGCCGCCGGGCGGC
GTCTACACCCACATCGTCGGTACCGACATCGTACGCACCGGCGAAGACCAGTACTATGTGCTGGAGGACAATG
CCCGCACGCCGTCCGGCGTCAGCTACATGCTGGAAAACCGGGAAACCATGATGCAGATGTTTCCGGAACTCTT
CCACCAGAACCGCGTGCGGCCGGTGGAGAACTATCCCAATCTGCTTCGCCAGAGCCTTTCCTCGCTCGCTCCG
GCCGGCTGCGAGGGCAAGCCGCGCGTTGCCGTCCTCACGCCCGGAATCTACAACTCCGCCTTCTACGAGCACG
CATTCCTCGCGGATATGATGGGCGTCGAACTGGTGGAGGGCTCGGACCTGCGCGTCATCGACGGCAGGGTAAA
GATGCGCACGACACGCGGCTACGAGGCGATAGACGTGCTTTACCGGCGCGTCGACGACGATTTCCTGGACCCC
CTCACCTTCCGCCCGGATTCCGCCCTCGGGATACCGGGGATCATGGATGTCTATCGCGCCGGCAATATCACCA
TCGCCAATGCCCCCGGCACCGGCATTTCCGACGACAAGGCGATCTATTCCTACATGCCGGAAATCGTCGAATT
CTATACCGGCCGCAAACCCCTCCTCGAGAACGTGCCCACCTGGCGCTGCTCGGAACCGGAGAGCCTCAAATAC
GTGCTCGAGCATCTCGCCGAGCTCGTCGTCAAGGAGGTTCACGGCTCCGGCGGCTACGGCATGCTCGTCGGCC
CGACCGCGACAAAAAAGGAATGTGCAGCCTTCGCGGAGAAGCTCAAATCCCGCCCCGGCAACTACATTGCCCA
GCCGACATTGTCGCTCTCGACGGTGCCCATCATGGTCAACAAGGGCATTGCGCCGCGCCACGTCGATCTGCGT
CCCTATGTGCTCGTCTCCGACAAGGTTCAGATCATTCCGGGCGGCCTGACACGCGTGGCCCTGAAAGAAGGCT
CCCTGGTGGTGAATTCCAGCCAGGGCGGCGGGACCAAGGATACCTGGGTGCTGGAGGACTGAGTGAAGACGCT
CAAATTCCTGGTTCTGGCCGGCTCCCTCGTCGTGGCAGGCGGAGGAGGCTACGTAGCCGGCACAGGCGGCCTT
ACCCAACAGTTCTTCCTTGGGGCAACTGCACGAGAACCCACGCCGCCTGCGGCAAAGCCGGCGGGCAAGATCA
TCTACTACCGGCACCCGGACGGGGTGCCGGAATATTCGGCGGCGCCGAAGCAAACCGCGGACGGGCGCCCGTT
CGTCGCAGTCCGGCAGAGCGAGGATGTCTCTTTCGAGCCGCTGAAGGCGGTTGCCGAGGGCCCCGCCGCCGGC
GAGCGAAAAATCCTCTACTACCGCAACCCCATGGGGCTGCCGGATACCTCCAAGGTGCCGAAAAAGGACTCCA
TGGGAATGGATTACATTCCCGTTTACGAGGGTGAGGAGGCAGACGGCTCGACCGTGAAGGTGTCTCTCGGCAA
GCTCCAGCGGACGGGTGTGAAGACGGCATCGGCGGAGCGTGCCGTCATCGCCCGAAGGGTTCGCGCGCCCGGC
ATGATCGCCTTCGACGAGCGGCGCATCAGGATCGTCTCGACGCGGGTGGATGCGTTCATCGAAGACGTCGCGG
AAATCACGACCGGCGACCGGGTCCAGGAAGGCGAGAACCTCTTCCACTACTATTCGAAGGACGTCGCCAGGGC
GGGAGCGGAATACGCCACGGAACTGCGCGGCGGCGTCAAGCCGGGCCTCGAAGTCGGGGGAGCCCTGCAATTG
CGCAATCTCGGCGTGCAGGAAGAGACGATCCTTTCGATTGCCAGGGATCGTACGGTCCCGCGGAGCATTGCCT
ATCTTTCGCCGAGCGACGGCGTCGTCCTGGAACGCAATGCCATCCCGGGCATGATGGCCAAGGCCGGCGACGT
CCTGTTCCGCATCGCCGACACCTCCAGCATATGGGTCCTGGCCGACATTCCGGAGTATGATGCCGCAGCCGTG
AGAAAAGGTGCTCCGGCGAAGGTCACAGTACGAAACCTTCCGGGCAAGGTCTTCGAGGGCAGCGTCGATCTGA
TCTATCCCGAGCTTCGCTCGCAGACGCGGACGGCGAGGATCCGGATAGAGCTCCCCAATCCGGCGGGGCAGCT
CCTCGCCAACATGTATACCGAGGTCGAGATCGTTACCGGCAAACCGGAGCCCGTCATGACGGTCCCGAACAGC
GCCGTCATCGACACCGGCAACCGCCAGCTCGTCTTCGTGGACAAGGGCGAAGGCCGCTTCGAGCCGCGCGAGG
TCGCGCTCGGGGCACGCGACAGCGAAAGGACCGAGATCACGAAGGGGATCGAAGCCGGCGAGAAGGTCGTGAT
TTCAGCCAATTTCCTGCTCGACGCCGAAAGCAACCTCAACGCAGCGCTGAATGCGCTTGCCTCGGCGGAGGCG
CAGCCATGAATGGCACAGGCATCCGACAGCATCCGCTTCATCCTGAACGACTCCGAGATCGCCCTTTCCGACG
TCGCGCCGACGTCGACGCTGCTCTCTTTCCTGAGGCTGGAGCGCCGCCTCACCGGCACCAAGGAAGGCTGCGC
GGAAGGCGACTGCGGCGCCTGCACGGTGCTCGTGGGCCGGCTCGCCGAGGATGCCAGCGGAGCGAAAAAACTC

GTCTATCAAAGCGTCAACGCCTGCATCCGCTTCACAGGCTCGCTGAATGCCACCCATGTGGTGACGGTCGAGC
ACCTGGCTGCCGCAGACGGCACATTGCATCCGGTGCAGCAGGCACTGGTGGATTTTCACGGCTCCCAATGCGG
CTTCTGCACTCCGGGGATCGTGATGTCGCTTTACGGGCTCTGGCTTGCCGATGAAAACCCGAGCCGCGCGGCG
ATAGAAAAGGCGCTGCAGGGCAATCTCTGTCGCTGCACCGGCTACGAACCGATCGTGCGCGCGGCGGAGGCTG
CGGCCGCGGAGCGCCCGGCCGCTCTCATCGACCCGGTCACCCGCACGCGGGAAGCGGTTACGGCGCGCCTTCA
TGCGATGGGGCAGACGGAGACGATCGTTGTCCGCAGCGGCGCGGACTGTCTGATCGTACCCGTCGACGCTGCG
GACCTGGCAGGTGTTCTCGCTGATCATCCAGGCGCGACGATCGTCGCCGGTTGCACCGATGTCGGGCTCTGGG
TGACGAAGCAGATGCGCTCGCTCAATCCGGTGGTCTTCATCAATGGCATTGCCGAACTGCAGCGGATCGAAAG
CTCGACAGCAGGCCTGACGATCGGTGCAGGCGTCAGCTATACGGCGGCGCACGAGGCCCTGTCTTCAGCCTAT
CCGTCATTCGGCCGGCTGCTCGACCGCATCGGCGGCGACCAGGTGCGCAACATGGGCACGATCGGCGGCAATA
TCGCCAATGGCTCGCCGATCGGCGACAGCCCGCCGCCCCTGATCGTTCTCGGCGCGACGGTTACCCTGCGCTC
GAAGGACGGCCAGCGGACGCTGCCGCTCGAGGATTTCTTCATCGCCTATGGCAAGCAGGATCGCAGGCCCGGT
GAATTCGTCGAGAGCCTCTTCGTCCCCGCTCTGCCCGCGGACGAGCGCTTTGCCGCCTACAAGATCTCGAAGC
GCCGCGACGAGGACATTTCCGCCCTGCTCGGCGCCTTCCGCATCTCGCTCGACGGCGACCGGGTGAAAACCGC
ACGGATAGCCTTCGGCGGTATGGCGGCAACTCCGAAGCGGGCGAAGACCGTCGAAGCAGCGCTGATCGGTCAG
GCCTGGACGGAGGAGACGGTCCGTACTGCGCAGGCCGCCTTCGAGACCGACTATCAGCCGATCACCGACTGGC
GGGCCACCGGTGCCTACCGCATGCTTGCGGCAAAAAACCTGCTGATGCGCTTCTTCCTCGAGAGCATCGGAGA
GACGGTGCAGTTGCAGCGGTTCGAGGAAGTCGCATGAATGTCGTTCCTGAGAAGCGTGCGGCCCAGCAATCTG
ATCATTCTGACAGCGCTGCCGTTCATCATCTATCTTTTCGCCTCCTCCGGAGACTACCAACGTTCGCTGAGAG
CCATTCTCGGAGTCGAGAACGGCTCGTCCGCCTTCGTACCGGGTTTCCTTGCTCTGCTCGTCGCCTTTGGCTC
GGGTGTCGCCGCTCTCGTCTTTTCCCGAGCCAAGGCGCCGCGGCCGCGGCTGGCGCTCGCTGCCGCCGTACTC
AATCTCGCTGCGGCGGCCACCGTGCTTCTGAGCGACGCCGTTCACCCCTACCTTGCGTCGGTCGTCGCCAATG
CCGTCGATTCCTTCACGTCCGATCTCGTTGTGAAGGGGCTTAGCCCTCGGCAACTCACGGACGCCGCGGACAT
GACCCTGCGCGGCATCGAGGCCGTGCTTTTTCCGGCCTATTTCGCGCTGACACTGCTCGGCTTCATGCTTTTC
TTCGGTGCCGGCAAGGCATCCACGCCGGAAAGCATCATGCGCCGCTTGGGAGCGTTAAGCATCGGCCTCGTCA
ACGGTGCGGGGCTCCTGTTCATCCTCTTCTTTGCGCATATCGCCTTTGCCGCCGGCGTGGCGACGACGGTTCG
CGCAGCCATTGCCGCCTATCTTCTCGCCGCGATCATCGGCCTCATCTGGGTCGGATTGCTTCAGTTGCATTAT
ACGGCCCGCACCAGCATCATCTTCGCGCTCGCGACCCTCGGCCTGACGCTCGTCGCCGCCTGGTTCCTGTTGC
AGCCTCGCACGGCCTATGTGCTTGCGGGGCGGCTCGACGGCACGGTCGCGATCGTCAGCAACACGCCGTCCGG
CCTCATAGGCGCGGTGCGCTTCGGCCAATATCCGGGCGCGCCCGGTGAAGAGGCGGCGGTGCGAACCTTCCTT
GGCCCGGCGGAGGCAGTCGCGGCGATGGGAAAGAACGAGGTGACGGCGGCGCTTCTGCCGTCGGCAGCCGTAC
CCGGGGGCTTGCCGCTCCTTTGGGAAACGGCGGCTTTGAACGATCGCGACCGGGCCGCAGGCATCACTTTCGC
CGTCCTGGCAATTATCCTCGGTCTGCTCACCTTCGGCGGCTATCTGCACCGCCGTCATCCCCTGGCGATCGGC
TCGGAGTTCGTCGTCGACACGATTCGCGGCATTCCGATGCTGGTGATCGTGCTCTATGTCGGCCTGCCCTTAA
GCGGGGCGCTGAAGGATGCGACACAAGGCGTGCTCGATCCGCCGAACCTGATGCGCGGCATCGTCGCCATGGC
GCTCGCCTATTCCGCCTATCTGGCGGAAATCTTCCGCTCCGGCATCAATGCCATCCCGATCGGCCAGATCGAA
GCGGCGCGCAGCATCGGGCTCAACCGGTGGCAGACGGCGCGGCTGGTGGTCCTGCCGCAGGCGTTCCGGATCA
TCATTCCACCGCTCGGCAACGAGCTGATCGCGATCCTCAAGGACACGTCGCTCCTGTCGATTCTGTCGATCCG
CGACATCACCCAGCGCATGCGCGAGTTCCAGTCGGCGAGTTTCCTGCCCTTTGCGCCCTATAACTCGGCAGCC
ATCTTCTACATCTTCCTGACGCTCGCGGCGGCGAGCCTCATCAACATGATCGAGCGGAAATATGACATCAAGC
ATCGATAGATGAACCAGATGTCCAAGATTTCGGTGACGGTGAATGGCCGCCGCTATCCGTGGCCCCGCGTACC
GGCAATTGCGGTCTGCCTCGACGGATGCGAGCCGGCCTATCTCGATGCAGCGATCGACGCCGGGCTGATGCCC
GCGCTGAAACGCATCAAGGAACGGGGCGCCGTGCGTCTGGCGCACAGCGTCATCCCGAGCTTTACCAATCCCA
ACAATCTCTCGATTGCAACCGGCAGTCCGCCGGCGGTGCACGGAATTTGCGGGAACTACCTCTACGAGCCGTC
GACGGGCGAAGAGGTGATGATGAATGATCCGAAGTTCCTGCGCGCGCCCACCATTTTCCAGGCTTTCTATGAT
GCAGGCGCCCGGGTCGCGGTCGTCACGGCGAAGGACAAGCTGCGGGCGCTGCTCGGAAAAGGGCTCCGTTTCG
ACGAAGGTCGCGCCGTCTGCTTCTCGTCCGAAAAATCGGACAAGGCGACCCGCGCGGAGCACGGAATCGACAA
TGCTTCCGCCTGGCTCGGCCGGCCGGTTCCCGAGGTCTATTCGGCAGCGCTTTCGGAATTCGTCTTCGCTGCG
GGCGTCAAGCTGCTCAGGGAGTTTCGTCCCGACATCATGTATCTGACGACGACCGACTATGTTCAGCACAAAT
ATGCTCCCGGCGTCCCGGAAGCCAATTCCTTCTACGAAATGTTCGACCGGTATCTCGCCGAACTGGACGGGCT
CGGAGCAGCCATCGTGGTCACCGCCGATCACGGCATGAAGCCGAAGCACAAGGCGGACGGCTCACCCGACGTC
ATCTATGTTCAGGACCTGCTCGACGAATGGCTGGGCAAGGATGCGGCCCGCGTTATCCTGCCGATCACCGACC
CCTATGTCGTGCATCACGGCGCGCTCGGGTCCTTTGCCACCGCCTATCTTCCGGACGGATGCGACCGCAGCGA
GATCATGGCAAGGCTGAAAGCGATCCAAGGCGTCGACGTCGTGCTCGGCCGCGAGGAGGCGTGCCGGCGCTTC
GAGCTGCCGGAAGACCGCATCGGCGACATCGTCCTCGTCTCATCCGAAAACAAGACGCTCGGCACCAGCGAGC
ACCGGCACGACCTGGCCGCACTCGACGAACCGTTGCGCTCCCATGGCGGGCTGACCGAGCAGGAGGTTCCCTT
CATCGTCAACCGCGTGCTGCCCGAACTCCCGAACGCTCCCCGGCTGCGCAACTTCGATGCGTTTTTCTACGCG
GTCACGGCGGCGGCCGAGGCCGGTGCGGAGGGCGGGCTATGAATGACTCTCGTTGCCCTCTCGCTGCATCTCG

CCGGCGCGACCGTACTGCTGCTCTACGCGGTGCATCTCGTCCGCAGCGGCGTCGAGAACGCCTATGGCACGTC
GTTGAAGCGTGTGCTTGCAAGGGGAGGGGAGCGACGCATTTACGCCGCCGCGAGCGGCACGGTCCTTGCAGTC
CTGTTGCAAAGCTCGACCGCCGTGGCAATGCTCGCCTCCGGTCTTTCGGCGAGCGGCTATCTTACCCTGAACA
CCAGCCTTGCCGTCCTGCTGGGAGCGGATCTCGGCTCGGCGCTGGTGGTTCGTGTTCTTTCGTTCGACCTGGG
CTGGCTGTCGCCGCTCCTGCTTGCGGCCGGCGGGCTGCTTTACCTGAAGGGCAGAAGCGACAAGCCCCGCGAA
CTCGGCCGCATGCTGCTCGGCATCGGCTTCGTCCTCATGTCGCTGAAGCTGATCGGCGAGGCGACCTTGCCGT
TGAAGCAGAGCGACGTGTTGCCCGCCGTCGTCGGCTATCTCGCGGAGGACCAGGTGACGGCCTTCGCGGCGGC
GATGCTCTTCACCTGGATCGTGCATTCGAGCGTCGCGGTCCTGATCCTCATCCTGGGTTTTGCCGCACAGGGA
CTTTTGCCGGTCGAGGCAGGTCTGCCGCTGATCCTCGGCGCCAATCTCGGCAGCGGGTTGATCGCCATGTGGC
TGACGCGCGGGTTTTCAGCCGAGGCGCGGCGCGTCGCCGCCGGCAATCTCCTCTTCCGGGCTTTCGGCGCCGC
GATCGCCTTGCTGGTGATCGGCCCCTTGTCGGCAGAACTTCAACATCTTGGCGCCACCGCGGCGGAGCAGCTC
GTTCATTTTCATCTGGCCTTCAACGCCTGCCTCGTGATCTTTTGCCTGCCGCTCTCGCAGCTTGCCGCAAGCG
CCGTTACGTCGCTCATCAAATCGGATCTCAAGAGCTCCGAAGACAGCGACACGCTTGCGCGACGAATCAGCGC
GCTCGACCAGGCTCTGCTCGACAGCCCCGACGCGGCCCTCGCCGCCGCGACGCGCGAGGTGCTGTTCATGGGC
GAGGTGGTCGAGCGGATGCTCTGTCCGGTGATGGAGCTTCTGGAGCATCCGAGCCCGGCGAAGATCGAGGAAA
TCCGCCGGCTCGACGGATATGTCAACCGCGCCCACCGCGACATCAAGCTCTATCTCGCAGCAATCCATCGCGG
CGTGCTCGACGAGCGTCAGTCGCGGCGGGGTGTAGAACTCATCGACATCGCCATCAATCTCGAACATGCCGGC
GACATCGTATCCAAGACCCTGGTCGAAATCGCCCGCGACAAGCTGGAGAACGGCAACAACTTCTCCGACGAGG
GCTGGACCGAGCTCAAGGCGCTTCACGCCAGCGTGCGCGACAACATGCGTCTCGCATTCAACCTGCTGGTGTC
GGATGACCCGGTAATGGCGCGAAAGCTCGTCCGGGAGAAGGAGCGGGTACGCGCGCTCGTGCGGGAAAGTCAC
GAACGGCATCTGGCGAGATTGCGGGCCGGGCGCGCGGAAAGCGTCGAGTCCAGCAACATGCACCTCGAAGTCG
CGCGGGCACTCAAGGAGGTGAACTCCCTCCTGGCCACGCTCGCCTATCCGATCTTGAAGCAGCGAGGGGATCT
GCTCGAGAGCCGGCTTGCGCTTGTCAAAGGGTGAATGAACATGCCCCGCATTCTCAAAACTCTCGTCGCCGGC
ACCGCGCTCACATTGCTGGCGTCCGGCGCCTTCGCCGACGGCATCGGCGCATCGCTCCTGACCCAGCAGCACC
CGTTCTACATCGAGCTCGCCGAGGCGATGAAAGCCGAAGCCAAGGAGAAGAACGTCGCGCTCGAGGTGGCGAT
CGCCAATCAGGACCTCAACAAGCAGCTCGCCGACGTCGAGGACTTCATCGTCAAGGGCGTTGATGTGATCGTC
ATTTCGCCTGTCGACAGCCAGGGCGTACGCGCCGCCATCGCAAAGGCGGAAAAAGCCGGCATCAAGGTGATCA
CCGTCGACGTTCCCGCTAATGGCGCGACGGTGACGTCGCATATCGGCACCGATAATTTCACCGGCGGCGTGAA
GGCGGGCGAGCTCATGGCCGAGGTTCTCGGAAACAAGGGCAAGGTCGCCATTATCGACTATCCGACCGTTCAG
TCGGTGGTCAATCGCGTCAACGGCTTCAAGGAAGCGATCGCCAAGCATCCGGAAATGGAGATCGTCGCGACGC
AGACGGGCATCACCCGCGCCGAAGCCCTCGCTGCCGCACAGAACATGCTGCAGGCCAATCCCGACATTACCGG
CATTTTCGGTTTCGGCGACGATGCGGCCCTTGCCGCGGCCGTTGCCGTCAAGGCCGCCGGACTGGAATCGCAG
GTCAAGGTCATCGGTTTCGACGGCATGAAGGAAGCACGCGATGCCGTCGATGGCAATCCGGTGATGGTCGGCG
TGATCCAGCAATTCCCGGACCAGATGGGCAAGCAGGCGATCGACACCGCCGTAAAGGTCGTCGCCGGCGAGAC
GGTACCGGCCGAGCAGCCGATCGTACCGGGCGTCTATACCGGGAAATAGATGAGCCTGAATTCCGCATCGACT
GCCGCGACCGTCACCACCCCGGTCCATTCGTTGCTCGTGCCGTTTCCAGTCGTCTGTTTTACGCTCGCCCTTC
TGACTGATATCGCCTTCTGGCAGACGGGACATCTCATGTGGCAGAACTTCTCCGCCTGGCTGCTCTTTGCCGG
TATCATCGCCGGCGTGATCGCCGGTCTAGCCAGTGTGTTGGAGTTCCTGTTTCGAAGGGAGCGACGCAGGCAA
GGGGCGGTTTGGCTCCATGTCGTCGGCTACATCCTCGTCTTCGGCCTCGCCTTCGTCAACAACCTCGTCCATG
CCGCCGATGGCTGGACGGCTGTGGTGCCCTACGGGTTGATCCTCTCCGCGGCGACGGTGCTTCTGACGATCCT
CTTCGCTTTGCTTGGCCGCGCCACCCTCTATCGCACGCAATCCGGAGTGAGCGGTTATGAATAGATGTCCGGA
AATAAGGAAAACTCGGACCTGATCGTGGTGGCAATGCCGCTTTATGGCCACGCAGCCCTCGTCCTGGAGGCGA
TCGAGTCCGTGCTCGCTTCGAAGCTTGCCGGGTGCCGGGTGGCAGTGGTGGTGTCCGTCGATGGTGATCCGCG
ACAGGAGACATTCGATCAGTTGCTGCTCTATGCGGCGGCCCATCCTGCGGTTCATGTGCTTTTCGGGGCCAAT
GCCGGGCCGGGCGGGGCGCGCAATCGCGCGATCGACTACGTGCTCGCGAATCTGCCGGAAGCAGAGGCCGTCT
ATTTTCTCGATGCCGACAATCGCGTGCTGCCCGGGACCATCGAGACTCTCTACCGGCAACTGCGCGCCAGCGG
CTGCGGCTGGATCTATACCAATATCGACACGTTTTCGGTGAGCTGGCGCGCCCATTACGGCAATCGCTACTCG
CGGCTTCTGCACTGCATCACGGACAATATCTGCGACACCGGTTCGATGATCTCGATCGACGTTTTCCGGGCGG
GGGTCCGCTTCGACGACGACAGGCAGAACGGTTTCGAGGATTGGGAGTTCTGGCTGTCCTGCATCGAGCACGG
CTTCGTGGGGGCGCCGTGCCACGACACGACCTTCGAATACCGGCTGAGGGCGGAAAGCCGCTTCAAGGAGGCG
AACCGCGATCGCGCCGCCTCGGTGAGCTTCCTCAGAAAGCGCCACCGGGCGCTGTTCCAGCGGCCAATGCTGG
TCGATTTCGAGCACGAGGAATGCCCGCGCTATCTTTTCGCACGGACGGAAGATGCCGCCATTTCGTTCTTTAC
GGATCCGACCAAGACGCCCAGGCGTCTTCGCCTCGACGATATCATTCCAGCCTTCTGGGCGAGCATCGGCGAG
CCTGACAACGTTCACTTCCCGCCCTTCCTGATCGCCGGAAGCGGTGCCACGCTCGATCTGCTGCTGCGCTCCC
GGATGCTGCCGAACGTGCTCTCCCATCTGGAGCGCCTGAGCGAGAAGGCCAACGTGGTCTTCGTCCAGCTCGG
CAACGATGCGGCTCAGCGCAAGATCGAGCCGGTCTTCCTGGAGGCCGGCGCCCAGCACAACGCACCGGCCGAC
CTCATCTTCCTGTCCACTTCGCTCGTGCGCGACGTCATTCATAACAAGGCGCTGGACTGGTTTGCCTCCATCG
GCAACCAGCAGGTCTGGCCGACATCGGCAATTCTGAAAGTCCGCTTCCCCTTCCCGAGAAGCCTGCCGCGCCG

CTCGCTCATCACGCCCCAGCAGGTGATGATCAATTGCGTCAATGCCATCGCGACCAGTCCGCTGCGGCGGACC
GCCGGCAAACGCTGGACCTGGCGTCCGGCACGGCTCGTTCCCTATTCCGACCTCCACAAGGCGCTTCGCCACG
AGATCGGTGGCTCGCCCGTGTTGCCGCTCGGTCATAGCGAGGGAAGCAGGAAGACCGCAGCCCTGCTTGTTCC
GAATGCATCCTTCGGCGGTGCCGAGAAGGTCGTATATGCGGCCTCGCGCGAGCTGAAGGCCGCCGGCTACGAG
ACCCATCTCTTCGTGCTCGGCACATCCAGGATGGATGTGATCGACGAGTTCGATCAGAGCTTCGACTATATTC
ACTTCTGGGACCAAGGCATTCCCGCCTGGGGCGGCTCCGGCTCGTTCCTGGGGCAGGACTTCATCGCCGAGGG
CCACGACGTCGATTGGGCTGCGCTCAAGGGTCAGCTTTCCGGCTTCGACCTCGTGATCAACAATCACGTCATG
GCGGTGCATCCGCTTATCGCGCGGTTACGCTCGGAGGGAACGCGCACCGCCTGCTACCTTCACGTGGTCGACA
ATACCTCCTTCAAAAGGCCCGCCGGTCAGCCCTTCGCGGCGATTGCCCACGAGCATTGCTACGATGCCTTCCT
GACCTGCTCGGAGCAGCTCAAGATCTATCTGCACAGCTTCGGCGTTCCCTATGAAAAGATCTTCGCCGTCCCC
AACGGCGCGAGCTTCTCCGTGCCGCCCAAGGTTCTCGCGGAGGTGCTGTCGGTCAGGCGCATCGAACGCAAGG
ACGATCGGCTTCGGGTCCTCTATATGGGTCGGCTCGATCAGCAGAAGGGGATCGACCGTCTCGCTGCGGCGTT
CGCCGAATTGCGGGCTTCGCGCGTGCCATTCGATGCCCGGGCGATCGGCGGGGAAATCCTCGCGGATGCCACC
TTGTCGTGGACGGATCGCCTGAAGGACCTCGGCGTCGAGGTGCGCCCGCCGGTGTTCGCAAGCAAGGACCTGA
TCAAGGCCCTCGGTTGGGCGGATGTCCTTCTGATGCCGTCGCGCTGGGAGGGCGCTCCCTTGATGATCGCCGA
GGCGCAGCAGCTCGGCTGCGTCCCGATCGCGACGGCAGTCGGTGCCGTCGACGAACTCATAACCGACGGCGAG
GACGGCATCCTCATCGAGGCCGCCGCCGATCCCCAGGTGGTCCGGGACATGGCAAAGGCCATCGAGGAGGTGG
CCAACAACCGCCAGATGCTCGCGCCCCTCATGGAGGGGTGCCTGAGAACGGCGGCACGCCGCTCATGGACGTC
CTCCTTTTCCGAGTTCCTCGGCTGGTGCGACCGTTCCGTGAACAATTCATCGCTTTCGCGCGCAACGGTCATC
CGTGGGCGCGAAGCATCCAATCCCGGAGTTGCGGCCGTGGGCTGAATGGCGGGAGAACTGGAATTCATGAGCC
GGGAGGTCGTTTCGGGCCGCCTGTCGAGGCGCGATTTTCTCGGGCGCGCGGCAGCGCTCGGCGTAACGCTCGC
CTCGGCCCAGGTGCTGCTCGGCAATGCCGCGCGGGCGAGCGGGCCCGTCAAGGGCGGCATATTGCGGGCCGGG
CTCGTCGGCGGCGAATCCACGAACAGCCTCGATCCGGCGACCTGGGCGAGCCAGGTGCCCTATACATTCGGCC
GCTGCTGGGGAGAGCAGTTGCTCGAGGTATCTCCGGCGGGCGAGATAGAATATCGGCTGGCTGAATCCTACGA
AGCGTCGAAGGACGCGAAGACCTGGGCGTTCACGATCCGCAAGGGGGTGACCTTCCATAACGGCAAGGAACTG
ACGCCCGCCGATGTCGTCGCCACATTCGAGCGTCATGCCGACCAGAATTCGAAATCGGCTGCCCTGCCCTTCG
TCCAGGAATTCGAGACCATCAAGGCAGACGGCGACAAGGTCGTCATCACGCTGCGCCAGCCGAACGCCGACAT
GCCGTTCATCGTCAGCGATTATCATCTTATGATCCAGCCGAACGGCGGCAAGGATGACCCGACGGCCGGCATC
GGAACCGGCCCCTACAAAGTCGTCGTCAACGAACCGGGCGTAAAGCATGTCGGTGAGCGTCAGGACGGCTATT
GGGGAGCGGCCGAGCGCGGCCATGCCGATCAGATCGAAATCACTGTCATCAATGACGCAACGGCGCGCACCGC
CTCGCTCCAGGCGGGCGGCGTGCACATCATTAATCGCATCGAGCCGAAGATCGTCGCGCTCGTCAAACGCATT
CCCGGAGTCACGATCCGCAACGTCCCCGGCAAGGGCCACTATGTCCTGATCGCCCATTGCAACACGGCGCCCT
TCGACAACAACGACCTGCGGCTTGCGCTGAAATACGCGGTGGACCGCGAGGAAATGGTGGCGAAGATTCTCGC
CGGATACGGCACGGTCGGCAACGATACGCCGATGATCAAGGGTTACCCGCTCTTCGACGACGATATCGAGCAG
CGCGTCTTTGATCCGGACAGGGCGAAGTTTCACTTCAAGAAATCGGGCCATGACGGTTCGATCCTGCTGCGGA
CCTCCGACGTCGCCTTCCCAGGGGCAGTCGACGCCGCGCAGCTTTTCCAGGCAAGCGCCGCAAAGTGCGGCAT
CGACGTCGAGCTGAAGCGCGAGCCCGGTGACGGCTACTGGTCCGACGTCTGGAACAAGCAGCCCTTCAGCATG
TCTTACTGGACCGGCCGGCCGACGCAGGACCAGGTCTATTCCATCGCCTATCTTTCAAAGGCGGAATGGAACG
ACACCCGCTTCTTCCGCGAGGATTTCGACAAGCTGATCTTTGCGGCGCGCGGCGAGCTGGACGAGGCGAAGCG
CAAGGTTCTCTATCGCGAGGCGGCAATGATCCTGCGCGATGAGGGTGGTGTGATCGCGCCGATGTTCAACAAT
TACATCGATGCCACCAGCGACAAGGTCGGCGGCTGGCTCGACGATCCGAACGGCGAACTCATGGGTGGACATG
CGCTGACCAAGTGCTGGCTCGCCGCCTGAATGCTCCAGCAGATCAAAGGCCTGCATCACGTAACGTCGATGGC
GGACGACGCCCGCACGAACAACCGGTTCTTCACCGACACGCTCGCCCTGCGCCGCGTCAAGAAGACGGTGAAC
TTCGACTCGCCTGACGTCTACCATCTCTACTATGGCGACGCGACCGGCACCCCCGGCACCGTGATGACCTATT
TTCCCTTTCCGCAGATGCCGCGCGGTCGCGCGGGCACGGGAGAGGTGGGGACGACCGTCTTCTCCGTCCCGCG
GGGCTCGCTCGGCTTCTGGCAGGAGCGCTTGGCGGCACTCGGTGCGAGCGGTCTCAAGCAGGCGGAAAGCTTT
GGCGAGAAGCGCCTGAACTTTGCCGGTCCGGACGGCGACGGCTTCGCTCTCGTCGAGGTCGAGGACGACCCGC
GTACGCCCTGGACCGAGGGCGGCATCACCGAGGATCACGCCATCCGCGGCTTCCATTCGGTCGCCATGCGCCT
ACGCGACGAAGGCGCGACGGCGGAACTGCTGAAGTTCATGGGTTACGAGGAACTCGACCGCAAGGACGGCGTC
ACCAGGCTGATCGTGCCCGGCGGCAACGGCGCGAGCCTCGTCGACCTGGAGACGCTGCCAAACATCAGCCGCG
CTGCGCAGGGCGCCGGTTCGGTGCACCACGTCGCATTCGCCGTCGACAACCGCGAGAAGCAGCTGGAGGTGCG
CAAGGCGCTGATGGACACCGGCTACCATGTGACGCCGGTGATCGACCGGGATTATTTCTGGGCGATCTATTTC
CGAACGCCGGGCGGCGTTCTCTTCGAGATCGCCACGAACGAGCCCGGCTTCGACCGGGACGAGGACATCGCGC
ATCTGGGCGAGGCGCTGAAGCTGCCGCAACAGCACACGCATCTGCGCCCGGTTCTCGAAAGCCATTTGCAGAA
GCTGGAAGCCTAGATGGCCACTTCCGTGTCTACACGGCACCTCAGCGAGAACGGCGCTCTTATCGAGCGTCAC
TGGCAGGAGCTCAACAATCGCCGGCGTCTCTATACCTGGCTCGGTCTCGCATTTCTCGCGTTGACGCTTTTCA
GTTCGCTCTGGTTCGCGAACGAGTCGAACGCGGGCAAGTTCTTCGACCGGCTGCCGCACTTCTTCGATTTCGT
CGGACATCTCATGCCGCGCGATCCGATAGAGATCGTTCGCGCCCTCTTCGACCTGCCTTCGCCCTATGACGAC

GGCAGTTTCAAATACAATTACCCGGATGGGCGCCTCTATGTGACCGAGAGCTTCTACATCCCGGAATATGTTC
ACAAGATGCTGGAGACCGTGAACATCGCCATCTTTTCGACTATCATCGGAGTCTTCTTCGGCTTCATCCTGTG
CTTCCTCGCCGCGAAAAATCTGATGCCCAATCCCTGGATCAGGGGCATCGTACGCCGGCTGATGGAAATCCTG
CGCGCCTTTCCGGAAGTCGTCATCGCCGGCTTTTTCCTGGCAATCCTTTCGCTTGGGCCTATTCCCGCCATCG
CTGCGGTGTCGATCCACACGATCGGCGCGCTCGGCAAGCTGTTCTTCGAAGTGGTCGAGAATGCCGACATGAA
ACCGGAGGAAGGTCTGCGTGCCGTCGGCGCCAACTGGATCGAGCGGGTCTGGTTCGGCATGGTCCCCCAGGTG
CTGCCCAATTTCACCAGCTATTTTCTGCTCCGGCTCGAAATCAACGTGCGCGCCTCGACGATCATCGGCGCCG
TCGGCGGGGGCGGCATCGGGGAACTCCTGCGCCTGTCGATCGGCCAGGGCCATGAAGCAAAGACGCTCGCGAT
CGTCCTCCTGCTCTTCGCCACGATCTTCGCCGTCGATCAGTTCTCCGCCTGGCTTCGCCGCCGCCTCGTCGGC
GACCAGGCCTTTCAGCTTGCGCAATAGATGACCACCGCCGATCGAGTATCCGGAACGCTCATCGTCGTCGTCG
GTCCGAGCGGTGCCGGGAAAGACAGCGTCATGGGCTTTGCCGCCCGCCATTTCGCGCAGCGGCCCGATATTCT
CTTCGTCCGGCGGGCGATCACAAGACCGTCGGATGCAGGCGGCGAAGTGCACGAAAGCGTGTCCGACGCCGAA
TTCGAGGAGATGAGCCGGAGCGGCGCTTTCGCCGTTTCGTGGCAGGCGCATGGGTTGAGCTACGCCATCCCGG
CAGAGATCGCCGAAAAGATCGCCAAAGGCATAACCGCGATCGTAAACGGCAGCCGCGCCGCCCTCCCCGCCAT
TCGTCAGGCGTTCGGTACGATCGCCGTTGCGGTGATCACCGCCGAGCCGCCGGTGCTGGCGAAGCGGCTGGCG
GAGCGCGGCCGGGAGAGCGAGGAAGACGTGCTCCGCCGCCTGACGCGGCAGGCACCCGACGTCATCGCCGATG
CCGACGTAACGGTCATCGACAACAGCGGCAGGCTGGATGTGGCGGGCCGGCAATTCGTCGCGCTGGTCGAGCG
GCACTCAGCTAAGCGCCATCACCCTGCCTGAATGTCGATCAAGCGCTATGGTGCCGAACAGGTGGGCGCAGGC
GGAAAAACATTGCCTTTCGCACGTGCCGTGGAGGCGGGCGGCTGGCTTCACGTCTCCGGTCAGGTCGCGATGG
AAAACGGCGAGATCATCGGCGGCAACATCGTCGCCCAGACGCACAAGGCGATCGGCAATCTTCTGGCCATCTT
GAAAGAGGCCGGCTACGGTGTCGAGCACGTGGTGAGAGTTGGCGTCTGGCTGGACGATCCGCGCGATTTCTGG
AGCTTCAACCGGATCTATCAGGATTATTTCGGAGCTCATCCGCCGGCGCGCGCCTGCGTACAGTCCTCCATGA
TGGTCGATTGCAAGGTGGAGATCGATTGCGTCGCCTATAAGCCGAAGGACGCCTGAATGCGACTGAACTTCAC
CAGGACACTGATCGGTGCCGCCATTGCGCTTGCACTCACCACCGCGGCTGCCGAAGCCGAGGTCGTCGTTTCC
TCGAAGATCGACACGGAGGGCGGCGTTCTCGGCAACATCATCCTCTCGGTCCTGAACGCCAACGACATCAAGA
CGACGGATCGCGTGCAACTCGGCGCCACCCCTGTCGTGCGCAAGGCGATCATCGCCGGCGAGATCGACATCTA
TCCCGAATATACCGGCAACGCCGCCTTCTTCTTCGAGAAGGCCAACGATCCGGCCTGGAAGGATGCGGCGAAA
GCCTATGAAGAGGCGAAGAAGCTCGACTATGAAGCCAACAAGATCGTCTGGCTGACGCCGTCTCCGGCCAATA
ACACCTGGGCGATCGCGCTGCGCAAGGACATTGCCGAGAAGAACAACCTCAAGACGCTATCGGATTTCGGCAA
ATTCGTTTCTGGCGGCGGCGAGGTCGTGCTCGCGGCTTCGTCGGAATTCGTCAACTCCGCCGCTGCCTTGCCT
GCGTTCCAGACGACCTATTCCTTCAAGCTGAAACCGGAACAGCTCATCACGCTCTCCGGAGGCGATACCGCCG
CGACGATCGCGGCTGCGGCAAACCAGACGAATGGCGCCAATGCGGCAATGGTCTACGGCACCGATGGAGGCAT
TGCGCCCTCCGGTCTCGTGGTTCTCGAGGACGACAAGGCCGTCCAGCCGGTCTATCAGCCGGCGCCGATCATC
CGCGAAGCGGTTCTCAAGGAAAATCCGGCGATCGAAGAGATCCTGGAGCCCGTCTTCGAAAAGCTCGACCTCA
CCACCCTGCAGGAGCTCAATGGCCGCGTGCAGGTCGGCGGCGAGTCGGCCAAGGCCGTTGCCGAGGACTTTCT
GAAGGCAAACGGCTTCCTGAAATAGATGAGCGTTGCCAATTTCTTCGACGAAATGTCCTATGGCCCGGCCCCC
GAAGCCGATACCGAGGCGCGCGCCTGGCTCACTCGGCATGAAGGCAAGCTCGGCCACTTCATAAACGGCGCAT
TCGTCGGTTCAGCATCAGGCAAATCCTTCGACACGTTCGAGCCCGCAACGGGCAAATTGCTCGCGAAAATCGC
ACATGGCGGGCGCGACGACGTGAACGCGGCGGTGGCGGCAGCGCGCAAGGCGCAGGGCCCGTGGGCAAAGCTC
TCCGGACATGCCCGCGCCCGGCACCTCTATGCGCTCGCGCGCCTGATTCAGCGCCATGCGCGCCTGATCGCCG
TCGTCGAAGCGCTCGACAACGGCAAGCCGATCCGGGAAACGCGCGACATCGACATACCGCTTGCCGCTCGCCA
TTTCTATCATCATGCCGGCTGGGCACAGCTGCAGGAGACGGAATTCGCCGACCAGGTGCCCGTGGGCGTTGTC
GGACAGGTCATCCCCTGGAACTTCCCCTTCCTGATGCTCGCCTGGAAGGTTGCGCCGGCGCTGGCGCTCGGCA
ATTCCGTCATCCTGAAGCCCGCGGAGTTTACGCCCTTGACGGCGCTGCTCTTCGCGGAGCTTGCCGCTGCAGC
GGGGCTCCCCCCGGGCGTGCTGAACGTGGTGACGGGCGAGGGCGAGACGGGGGCGCTGATCGTCGAGCACGAG
GATATCGACAAGATCGCCTTTACCGGCTCGACCGAAGTTGGACGCCTCATCCGCGAGAAGACAGCGGGGAGCG
GCAAGTCGCTGACGCTGGAGCTTGGCGGGAAGTCGCCCTTCATCGTCTTTGACGATGCCGATATCGACGCGGC
GGTCGAAGGCGTCGTGGATGCGATCTGGTTCAATCAGGGCCAGGTCTGCTGCGCCGGTTCACGCCTTCTGGTG
CAGGAGGGCGTGGCGCCGGTCTTTCATGACCGGTTGAAGCGGCGCATGGAGACGCTCCGCGTCGGTCATCCGC
TCGACAAGGCGATCGATATGGCGGCGATCGTCGCGCCGGTGCAGTTGCAGCGAATCGAAGAGCTGGTGGCGAA
GGGCGTCGCCGAAGGCGCATCGATGCATCAGCCGAAGATCGAACTCCCGAAGGGCGGCAGCTTCTTTCGGCCG
ACGCTGTTGACCGGCGTCCAGCCGACTTCCGTCGTCGCGACCGAGGAAATCTTCGGGCCGGTGGCCGTATCGA
TGACCTTCCGTACGCCGGAGGAGGCGATCCAGCTTGCAAATCATAGCCGCTACGGCCTTGCCGCGAGCGTCTG
GAGCGAAACGATCGGTCTTGCCCTGCATGTTGCCGCAAAGCTTGCGGCCGGTGTCGTCTGGGTCAATGCGACC
AACCTCTTCGATGCGTCGAGCGGCTTCGGCGGAAAGCGCGAATCCGGCTTCGGCCGTGAGGGCGGGCGAGAGG
GCTGCTACGAATATCTGAAGCCGAAGGCCTGGATGGGGCGCAAGCTGCGCCCCGAACACGGCTTGCCGGCCCC
GAAGCAGGCCGCCGGTGATTTCGCCGTTCCGGCGCTCGATCGCACTGCCAAGCTCTTCGTCGGCGGCAAGCAG
GCGCGGCCGGACGGGAACTATTCGCGGCCCGTGCTTTCCCCGAAAGGCAAGGTGATCGGCGAAGTCGGCGAGG

GCAACCGCAAGGATATCCGCAATGCCGTCGTTGCGGCGCGGGGCGCCTCGGGCTGGTCTTCGGCAACAGCGCA
TAACCGCGCCCAGATCCTCTATTACATCGCCGAGAATCTGAGCAGCCGCGGGGCGGAATTCGCCGACCGCATC
GCCGCCATGACGGGGGCCTCGGCAGCCAATGCCCGAGCGGAGGTCGAAGCTTCGATTGCCCGGCTGTTCAGCT
ACGGAGCCTGGGCGGACAAATACGAGGGCACCGTGCACCAGCCGCCCCTGCGCGGCGTGGCACTGGCCATGCC
GGAACCCCAGGGCGTCGTCGGCGTCATCTGCCCCCCGGAAGCGCCGCTGCTCGGGCTTGTCAGTCTCGTGGCA
CCGCTGATTGCAGTCGGAAACCGGGTGGTCGCCGTGCCGAGCGAAGCGCATCCTCTGGCTGCGACCGACTTCT
ATTCGGTCCTCGAGACCTCGGATGTGCCGCCCGGCGTCATCAATATCGTCACCGGATCTGCGATCGAACTCGC
CAAGGCACTCGCCGCGCACAACGACGTGGACGCGCTCTGGGCGTTCGGCTCGCCGGAACTTTCGACACTCGTC
GAGAAGCTGTCCGTCGGCAACCTCAAGCGTACCTTCGTGGATTACGGCAAGGCGATCGATTGGACGGATCGCT
CGGCCGCCGAAGGACAGGCGTTCCTGCGGCGAGCGGTCGATGTCAAGAACATCTGGATTCCCTATGGCGAATA
GATGACGATGGCATCGATCTGGCAAACGCTTGCCAACAGGCTCGATCGCCTGCAGGAGACAGGCGGATCGATC
GACTTCTGGCTGCGGGACGACGATGCCGTCGAGCCGACGGCGGCCCTTCACCGACTGCTCGACCTTACCGACC
GCTTTTCGGTGCCGGTGAGCTTGGCCGTCATACCGGCACATACCGACGAGCGCCTCTCGCGCTGCCTCGCCGG
CCGTCCGCATGTCGATGTTGCTGTCCACGGGTGGTCCCATCAAAACCATGCCCCTGTGACTGAAAAACGCCAG
GAACTGGGAGGACACAGGTCGCGCGAGATGGTTGCGGCCGACTTGCGCGCCGGTTGGGCACGCCTGCGCGCGC
TTTATCCCACCGCTATGGTGCCGCTCCTCGTTCCGCCATGGAACCGGATCGATCCGGGGCTGGTCGCCGGATT
GCACGAGATCGGGTTTAGGGCCCTTTCGGTTTTCGGACCGGAGGAGGGGGCAAAATCCGCCGCATTACGCGTT
CCCGGGCTGCAGCTCATAAATGCCCATGTGGATGTGATGGACTGGCACGGCACCCGCGGCTGCCGCCCTTATG
GCGAGATCCTTCGCGACATCGATAGCCGACTGGATGAAATCGAGAAGGGCGGCGGGACGGTGGGAATTCTCAC
CCACCACCTCGTTCACGACGAGAGCGTATGGGAATTCCTCTCCGGACTTTTCGACGTCACCGCCTCGCACGCG
GCCTGCCGCTGGCGCAGGGTCGCCGATCTTATAGATCGATGAATGGGACGCTGGCCGGTTTACGACGAGGAGC
AGATCGAGGATGTCGTGTCGGTCCTCAGATCTGGCGAGGTGAATGCCTGGACGGGACCGCACGTACGCAACTT
CGAGGCTGCCTATGAGCGCTTTCTCGGCGTAAAGCATGCGGTGGCTCTCGCCAACGGAACGGTGGCGTTGGAC
CTCGCGCTCTTCGCGCTCGGACTTGAGCCGGGCGATGAGGTCATCGTGACGCCGCGCAGTTTCATTGCCTCGG
CCTCCACGGTGCCGATGGCGGGTGGAGTTGCCGTCTTCGCCGACGTCGATCGCGACAGCCAGAATATCACCGC
GGAAACCATAAGGGCGAAGCTGACGCCGAGGACCAAGGGCGTCATCGTCGTGCATCTGGCCGGCTGGCCCTGC
GATATGCCCGCAATCATGGCCCTTGCCCGCGAAAAGGGGCTGTGGGTGATAGAGGATTGCGCGCAGGCGCACG
GCGCCGAAATCGACGGACGGCCGGTCGGCAGTTTCGGTGACATCGCGGCATTCTCCTTCTGCCAGGACAAGAT
CATCACCACCGGAGGCGAAGGCGGGCTCGTGGCAATGAACGACGACGAGCTCTGGAAGAAGGCGTGGAGCCGC
AAGGACCATGGCAAGTCGTATGACGCCGTCTACAACAAAGAGCACCCTCCGGGCTTCCGCTGGCTGCATGAGT
CCATCGGTACCAACTGGCGCATGATGTCGATCCAGGCGGTCATGGGATCGCGTCAGTTACAGCGCCTGACGGA
ATGGCGCAGCATAAGAGCGCACAATGCGGCTATTATCGCCAGAGCGGCCGAGGAGATCGAGGCTTTGCGCACG
CCCTTGCCGCCCCGTGGGATTCAGCACGCCTGGTACAGGTTCTATACGTTTCTTCGGCCGGAGCGCCTGCGGC
CCGACTGGAGCCGGGACCGGATCATTGCCGAAATCAACGGCGCCGGCGTCGCATGCTTCAGCGGCAGTTGCTC
GGAAATCTACCTGGAAAAGGCCTTTGCGGACATCGGCATGGCTCCGGCGCAGAGATTGCCCGACGCGCGCGAG
CTCGGCGAGACGAGTCTTGCCTTCCTGGTCGATCCCGCGCTCGATACGGCGGCAATGGAGAAGGCGGCACATG
TCCTGCGGGAGGTGTTTGCAAGGGCCAGCATCGGCAGGCCAGCCCTCGATGCCGAGCGGCGTTAGATGATTCT
GAAGGGAACCAATCTTTCTGACACGATCCTCGGCACTTTGAGTGACGATGAGCTCTGGGGCTATGCGGGGGAT
GATTATCTCGAGGGCCGCGAAGGCAATGACAAGCTTTTCGGCGGCGATGGTAACGACTACATCGTCGGCGGGC
TCGGAGACGACTATGCCGAAGGCGGCGATGGTAACGATCGGTTCGACGGCGGCCTTGGGCACGACACCTTCAA
GGGCGGATTAGGAAACGACATCTTCGACGGCGGAGACGGCAGCGACGTGCTGTACGGCGGCGATGGCCATGAC
CATTTCGTGGGTGGTCGCGGACACGACAAGATCTATGGTGGCGCGGGCGAAGAATACATCGATGCAGGCGACG
GCGACGACATCATCTATGCCGGCGCGGGCGACGACGGCTTCAACAATCGCATAGATCCGGCAACCGGACAGCT
GACGCAGCAGGCCGTCGGCGGGGGCGCCGGCGACGATAGGATCTACGGCGAAGAGGGCAATGACGCGCTGAAG
GGCCAATCCGGCAATGATTGGGTTTATGGCGGCATCGGCGACGACATCGTCGATGGCGGCGACGGGAACAACT
TTCTCGATGGGGGTGACGGCAATGATGTGCTGGACTCGGAGGGCGGCGCGGACGAGGCACATGGCGGCAGCGG
CAATGATCGGATCGCCGTGGGCGCCGGAAACGATCTGGTCTTCGGGGATGACGGTGACGACGTCCTGACCGGT
GAAGGCGGCGACGATGAGCTGAGCGGCGGCCACGACGACGACCGAATCTTTGGCGGCGACGGCAACGACACCC
TGCGTGGCGACGCGGGCCAGGACGTTCTGGTGGGCGAGTCGGGGGCCGACATTCTCTGGGGCGGAACAGAATC
CGATCGTTTCGTCTTCAAAGGCGCCGCAGTGCTCGGCAGTCAGGATACCGTCATGGATTTCCAGGATGGCGTC
GACTTTCTCCTCATCGAAAAGCTCGGGGTCAGCCAATATTCCAGTTCCGGCGCCTCGGGCACGGTCTACGCCT
ATGACACTACAGACGGCGATGTGCAGGTAATGGGCTACGACTCGGCCGGAAACGCCTTCTCCATCCTGGTGGA
CGATCCCAACGGCGCGCTCGCGGCTGCGAACTTTTCGAGGAGTGATTTCCTTTTCGCCTGAATGAGGAAACAC
ATTCCGCCCGCATCGCGACCCGATCCGGAACCGACGTCGCCGGTCGAGCCGATAACGCTCTGGCGCGCCTTCG
TTCCGCGCTGGGCACATATGCCGCTATCCGGCGCGGGTGCCGCTCGCTTCGGTGGCCGCTGGAACCCGATCGG
GATGCCTGCGATTTATGCTGCGCGCGAGCTTTCCACTGCCTGGGCTGAATATAATCAGGGTTTCGTGCAGCAT
CCGGCGCTCATCGTGCAGCTCGAGTTGCGCGGGGCGAGGCTCGCGGACCTCACGGATGCCAGCGTTCTTCTGG
AGCTTGGTGTGGATGAGGCGATCCATCGCTGCGAATGGCGTGATGCGCTCGACAAAGGCGCGGTGCCGGAGAC

CCACAGGCTGCAGTCGGAGCTTCTGGCGCGCGACTATCACGGGGTGATCTATCCATCCTTCATGTCGCCCGGC
GGCACCTGCGTCGCGCTCTGGCGCTGGAACGGTGCGGAAGAACCGCGTCTCGACGTCATCGATCCCGATGGCC
GGCTGCCGAAATCGCCCGCCTCATGGGTTTGAATGTTTTCGAAGCTCCTGATTGCCAATCGCGGTGAAATCGC
CTGCCGCATCATCCGCACCGCGCGACGGCTCGGCATCCGCACCGTCGCCGTCTATTCCGACGCTGACGGAGAC
GCGCTGCACGTGGCGCTCGCCGACGAGGCGATCCGGATCGGGGGGCGCCCGCCGCCGAAAGCTATCTGGCGA
GCGCTCCTATCGTTCAAGCGGCAAGAAGCGTCGGCGCACAAGCCATTCATCCGGGCTATGGTTTCCTGTCGGA
GAACGCCGATTTCGCGGAGGCTGTCGCAGAAGCAGGCATGATATTCGTCGGGCCGCCGCCGGCCGCAATCCGC
GCCATGGGTCTCAAGGACGCGGCCAAGGCTCTCATGGAACGATCCGGCGTACCGGTCGTGCCCGGCTACCACG
GCGAAGAGCAGGATGCATCCTTTCTTGCCGACAGGGCACGGGAAATCGGCTATCCGGTGCTGATCAAGGCGCG
GGCGGGTGGCGGCGGCAAAGGCATGCGCCGGGTCGAGCGGCAGGAGGATTTCGGCCCCGCGCTGGAGGCCGCA
CGGCGGGAGGCCGAATCCGCCTTCGGCGACGGTTCGGTGCTTCTCGAACGCTATCTGACGAAGCCGCGTCACA
TCGAGATGCAGGTCTTCGGCGACCGGCACGGCAATATCGTCCACCTCTTCGAGCGCGACTGCTCGTTGCAACG
CCGGCATCAGAAGGTGATAGAGGAGGCGCCGGCGCCCGGCATGACGGCCGAGGTCCGCCGTGCGATGGGGGAT
GCGGCGGTCAGGGCCGCCCAGGCGATCGGCTATGTCGGTGCAGGCACCGTGGAGTTCATCGCCGACGTGACCA
ACGGCCTCTGGCCGGATCACTTCTATTTCATGGAGATGAACACGCGGCTGCAGGTGGAGCATCCCGTCACGGA
AGCGATCACCGGCATCGATCTCGTGGAATGGCAGTTGCGGGTCGCTAGCGGCGAGCCGCTGCCGAAGAAACAG
GCCGATATTTCAATGAACGGCTGGGCCTTCGAGGCGCGCCTCTATGCGGAGGATCCGGCCCGGGGCTTCCTGC
CGGCGACCGGCCGGCTGACGGAATTGAGCTTTCCCGAGGGGACTTCGCGGGTCGATTCCGGCGTCCGGCAGGG
GGATACGATCACGCCCTATTACGATCCGCTGATTGCCAAGTTGATCGTACATGGCCAGAACCGTTCGGCTGCG
CTCGGCCGGTTGCAGGATGCTCTCAAGGAATGCCGCATAGGCGGCACGGTCACGAACCGCGACTTTCTCATCC
GCCTGACGGAAGAGCATGATTTCCGCTCTGGCCACCCGGATACGGGGCTGATCGACCGGGAGATCGAGCGACT
GACCGCTCCGGTCGCACCTGGTGACGAGGCGCTGGCGCTGGCGGCGATAGTTTCGACCGGCGCGCTCGACCCG
AACAGGTCGACCGATCCATGGTCGTCGCTCGGCTCCTGGCAGATCTGGGGCGACGCGCACCGCATGGTGGTGA
TCGAACATGCGGACGTGCGCGCAACCGTCACCTTGGCTTCGCGCGGCCGTGACCAGTTCGCCGTGCGTGCCGG
TGCGAGCACGCTGCCCGTCCTCGTGCTCGACCGCTTCGAGGGCGGTGCCCGGCTCGAGGTGGCCGGGCAGAAG
CGCCTGATCCGTTTCTCACGGGATCGCGAGGCGTTGACGCTCTTCCATGGCGGGCGCAACCTGGTGTTCCATG
TGCCGGACGGATTGACCGGCGGGCAGAGCAGCGAGATCGCCGACGACGAGTTGGTTGCGCCGATGCCGGGTCT
CGTGAAGCTGGTGCGGGTCGGCGCCGGCGACGCAGTGACAAAGGGACAGGCGCTTGTTGTGATGGAGGCGATG
AAGATGGAACTGACGCTCTCGGCCTCACGCGAGGGCACGATCGCCAATGTGCATGTGGCGGAAGGCGCTCAGG
TCAGCGAGGGCACGGTGCTCGTGACGCTGATGGAGGAGGCTGCGCAATGATTGATCATCGACACGCATCTGCA
TCTGATCGACAAATCCGCGCTCAACTATCCGTGGCTCGCCGGCGTTCCCGCGCTCGATCGCGATTTTCTCTAT
GCCACCTATGCGGCGGAGGCAAAGCGCGTCGGCGTTGCCGCCTCGCTGCATATGGAAGTGGACGTCGACCCGG
CTGAGATCGAACTCGAAACGCGCGAGGTCGCGCGTCTTGCGGGTGAGCCCGGAAGCCTCCTCAAAGGCGCGAT
CGCGGCTTGCCGCCCGGAAGATGAAGGCTTCGCCGCCTATCTCGAGCGGCAGGAGGAGAACGCTTTCGTCAAA
GGTTTCAGGCGGGTGCTGCACGTCGTTGCCGACGACCTCTCGGAGCAGCCTCTTTTCCGCGAAAACGTCAAGC
GGCTCTCGGGCACCCGCTTCACCTTCGATCTTTGCGTCCTGCCGCATCAGATTCCGAAAGCTATTGCCCTCGC
CGACCTTGCGCCCGACGTTCAGTTCATTCTCGATCATTGCGGCGTTCCGGACATAAAGGGTCACGCGGAGCAC
CCCTGGCGCGACCATATGACCGAGATCGCCCGCCGCCCGAATGTCGTCGCCAAGATCTCCGGCGTGGTCGCCT
ATGCGGAGGAAGACTGGGCGCTCGATTCCATCCGCCCCTATGTCGAGCACACGATCTCGGTTTTCGGATGGGA
CCGGGTGGTCTGGGGCAGCGACTGGCCGGTCTGCACCCTCGGCGGCAACCTCTCGACCTGGGTCGCGGCCACC
CAGGCCCTCATCGAAGGCTGCAGTCCCCAGGAACGGAGGAAGCTTCTCTCCGGCAATGCGCAACGGATCTGGA
ATCTGATCTGAATGAACAAGCACATCGCAGCGCTCACGGTCGCATGCCTCGCCGCAACCTCCGCCAATGCCGC
CGGCCTGCCCGAGCGGATCAAGTCGGCCGGTAAGGTCGTCGTCGCCAACCAGCCGAACTATCCGCCGATGGAA
TATGTCGATCCGGCGACCAACGAACTCAGGGGTGTCGACATCGATCTCGGCGTTGCGCTTGCCAAGGAGCTCG
GCACCACGGTCGAATGGTCGAATATCGGCTTCGAACAGATGATCTCGTCGCTTCAGACAGGCCGCGTCGACCT
TATCCATAGCGGCATGAGCGATCTTCCCAAGCGCCGCGATACGCTCGATTTCATCGATTACATGAAATCCGGC
GCGCAGTTCTACACGTCCTTTGCCCGCAAGGACGAGTTCAAGACGCTCACCGATTTCTGCGGCAAGATAGTCG
GCATGAGCCGCCGCACCTCCTTCCCGGACGAGACGGCGAAATGGAGTACTGCCAATTGCGAAGCCAAGGGGCT
GCCGGCCGTCAAGGTCGTCGGCACGGAAGGCTCCGCCGATGCCCGCGCCCAGCTGAAGCAGGGCCGGCTCGAC
GCCGCCGTCCAGGGTTCCGAAACGCTGCCCTATCTGATGGATCTCGAGAAGGACACCTACGCCGTCGTGGGCG
AGCCCTTCACCGTGGTCTATCAGGGCATCGGTTTCGCCAAGAAGGACACGGAACTGCGCGACGCCTATGCCGC
AGCGCTGAAGGCCCTGATGGAATCAGGCGAATACCAGGCGATTTTCGCCAAGTACGGCCTCGAGGGCACCTTG
CTCGATGGCATTTACGTCAATGGCGAAGCGGTGAAGTAAATGAGCTTAAGTTATTCAGAAAGATTAATTCTCG
CCAATCAATACGCGATTCTTGAGAATCTGGACGTAGGCAACGCACTCCTCCATCGGAGCGCGCGTGAGATCGT
CGAGCGCGGTTATGAATACCTTTATGATACGCTCAATCCGTCGGTGGAAGCAGAAGTCGTTCCCACCGCAATC
GGTCAGGAGGTGTATGATATTCTCGACATGTTCCGCGCGATCGAACGGTCGATGATAGACATCGGCAGGGCCC
CTTCCGAGCTCGAGGCGAATTTTGAAGGCTTTGACCCGAACAACGCCCGCGCTCATTTCCACTTCTGTGAGTT
CGTGCGGCGCTCCCTCGATTATTGGCAGGATCTCGACAAATACCCCGACACCTCGCACGACGCCTCGTCGCTC

ATGCGCTATCGCGCTATGCTGCTGAGATGGAAGTCTCTCGGTGCTCCATCGGCCCTTTCAGAGGCCCAGATCG
CCGAACTCGCGGGTACAGCCAACGCCTTGTCGGGGACCGCACATAAGGTGCACACACTGGTGCCGAAGACAGC
TATGCGGCTGTAGGTGGGCAGATCCGCGAACGAAAAACGAAATGGCGTCCCGCTCATTCATGGGGCCAGCGAT
CTGCCGTCGGTGACGGTTGACGATTATAATCTGGAGCTGCGTGACGGCGACGGCTTCCTTGGCGACAGGGCCA
ACAAATTTGCCTTCCAGGAGAAGCTCGACGCGTGGCGCAAACGCGTGCGCAAAGGCGGCGACGATCCGCTGGG
GCAGGCTTTGACCCAGGATCTGTCCAAAAAGCAAGTCGATGCCCTTCTGCGCGGCGACGACAAGGAGGCGGCC
GCCCTGATCATCGGTGCGGTCGACGATTTCGCCGGCGAACTCGCCAGCGTTCTCGAACGGTTTCTGCAACAAA
AGAACTGGAAGAATACGGAACGCGTGGTAATCGGCGGAGGCTTCCGGGGAAGCGCAGTAGGTGAATTGGCCAT
TGCCCGGGCAATGGTGCTACTGAAGGCTGAAGGCATCAAGATCGAACTCTCCCCTATCGTTCACCATCCGGAT
GATGCGGGACTTATCGGTGCCGCGCATCTCATGCCCGCCTGGATGCTGAAGGGACACAAGGCAATCCTCGCCA
TCGACATCGGCGGCACCAATATCCGCGTGGGCATAGTCGAGCTGCACCTGAAGGACGAAACCGATCTCTCCAA
GGCCAAGGTCTGGAAATCGGATATCTGGCGACATGCGGATGACAAGCCCAACAGAAGCACGACCATCGAGGGG
CTCATCGGCATGATCGAGAAGCTTATAGCCAAGGCGGACAAGGCGGACCTCGCGCCGGCGCCGGTCATCGGCG
TGGCCTGCCCCGGTGTGATCAACGAGGATGGCTCGATCCTTCGCGGCGGCCAGAACCTGCCGGGCGGCAACTG
GGAAAGCGAGCATTTCAACCTGCCCGCCGCCCTTAAGGACGCCATTCCGCAAATCGGCGATCACGAGACCTTC
GTGATCATGCACAACGACGCCGTCGTGCAAGGTCTGTCGCAGATACCCTTCGTGCAGAATGCATCGAGTTGGG
GGATCCTGACGATCGGGACTGGCCTCGGCAACGCGCATTTCAGCAACAAGGCCGAAAACTGAATGGGACAAGC
CGTACGAATCCCCACGAACGAGCGTCAAAGGTTGCTCGCAGTCCGTTCGCTCAATTCAATCGGCAGCGCTCCC
ACGCCCGAACTCGCGACCTTGGCCGAGCTTGCCAAAGGCATCTTCGACGTCTCCTACGCCGCCATCAACATCA
TCGACGAAGACTGGCAGCGGATCGGCGGTCAGGCGGGCTTAAGGATCGGCGAATGCTCGCGCGAGATGTCCAT
ATGCACGCGGGTCGTCTTCGCAGACGAACTCCTCATTGTCCCGGACCTGGCGGAAGACCGTGAGCTGAAGACG
AGACCATATGTGGTCGGAAGCCCGCACTTCCGCTTTTACGCCGGCGCGCCCGTCTATCTCGAGAACCTGCCCG
TCGGCAGCTTCTGCATCCTCGATGTCCGGCCCAGACATCTTTCCGACGGGCAGGCGCAGAACCTGCGCCGATT
TGCGGCTGTCGCCAGTGCGCTTTTGAGCTTGCAGAAGGCAAATCTCGTGATGGGGCTCGCCCATAACCAACTA
CAGAATGCGGCCATTACCGATCCGTTGACCGGCCTGTTCAACCGCTCGGCCCTGTCGTCGATCGTCGACGGGG
CTCTCCACAACGCCATCGCCGCGGGTCAGACCTTCGGCGCTCTTTATCTCGACATGGATGGTTTCAAGGCGAT
CAACGACGAGCTTGGCCATCACGCCGGCGACGAGGTTTTGTACGAAGCCGCCAACCGAATTCGCTCGGTCATC
AGAGCAGGCGATATCGCGATCCGCATGGGCGGCGACGAATTCGCCGTTTTCATTCCAAATCCGCCCAACGCAC
CGGCCCTGGCAGCGGTCTCGCAGCGGCTGGTAGCGGCCTTTCGCGAACCCTTCCAGGTCGACGGCAAGGCGGT
CCTTGCACGCATCAGCATCGGCGGCGCCCTTGCCCCGCTCAACGGGCAGCGACGAGCGGAGCTGCTGAACAGC
TGCGACAAGGCTCTCTACTCGGCAAAGCGTGACGGCCGCGACCGGTTCGTCATCCTCGACGCCTGAATGAGTT
CCTGCCAGTCCAATCTTCCTTCGAACCTCACTGTCAGCCTTCGCGAACTGCGGATGGTCTTCGAGCGGTTGAT
ACAGGTCACGCGGCTCGACGCGGGCCTCGTGCCCTCGCTCAGGGATTGCGCCCTTTATTCCGCAGCGCTCGGC
CTCGGCGGCTTCGCCCGCCTGGCTCACAACCTCGAGGCGGTGCGATCGGCAAATCCGCATGCGCTGAGGCTGA
CAGCGGACGCCGAGCGCCTAACTGTCGACTGCGGCGGGCAGCACGCGTGGATCGCCGCCGATGCGGTGCTCGA
TCTCGCAGCGGAGCGTCTGCGCACGCAGGGTTCGGGGAGGGTCGAGGTCCGTAGCGCCACCGAAATCGGCGAA
CTGAGGGTTGTCGAGGCTCTTGCGCAGCGTTTTCGACTGCAAGCTGAGGTCGCCATCGACGAGACAGCCGGCT
CTGCCGAGATCGTCGTCTCGCCCGCCCGGAAGGGAATGGATGTGACGGTTCTCGAAAGAATCCGGCGCAACGG
GCTGACCGTCGATGCGGCTCTCTGGTGGCAGCTGTTCCACCGGTCCGCCGAAGCGCTCGCGCCGGATTCCTAC
GTGTCGCGCCGCCACGCCGGTCCGATCATCGTCGAGGCCGACGGCAAGGTCATCGGCCGGCAGGACGAGGACG
AGACGGACCTCTCACTGCTGATCGCCGGCGCCGCACAGCACGGCACGACCGCAATCAATAACTGAATGAATCC
GGAAACCACTGCAGTCCCCTATGAGCGCCTCGGCATACTGATCGATGGCCGCTGGATATACGAGGCAGAGCGG
AGCACCGAGGTGGTGGATCCCGCGACGGGCCAGACGATTGCCCGGCTGCCTTTTGCGGCCGATGGCGAGATTG
CCGAAGCCGTGGCCTCGTCGCAGCGCGCTTTCGAGAGCTGGAAGGATCGCTCGCCGCTCGAGCGGGGCCGGAT
CCTGCGCCGCTTCGCAGACCTCGCGCGCAAGCATGCCGACGAGATCGCCCGTAACATGACGCGCGACCAGGGC
AAGCCGCTCAGCGAGGCGATCGGCGAGATCCGTTTCGCCGCCGACCATGCCGATTGGCATGCGGAGGAAGCGC
GCCGCATCTACGGGCGGGTCATTCCCGCCCGCGATCCGCGCGTGCAGCAGATGGTCCTTCGCGAGCCGGTCGG
GGTCTGCATCGCCTTCACGCCTTGGAATTTCCCCTTCAGCCAGGCGCTGCGCAAGGTCGTGGCGGCGCTTGCG
AGCGGCTGCACCATCATCCTCAAGGGACCGGGGGAATCCCCCTCCTCCACGGTCGCGATCGGCCGGCTGATGC
AAGAGGCCGGGCTGCCGGACGGCTGTCTCAACATCCTCTGGGGCGACCCGGCCCACCTGTCGGAGACGCTGCT
GGCGGCGCCGGAAGTCAGAAAAATCTCCTTCACCGGTTCGGTCGAGGTCGGGAAGCATCTCGCGAGCCTTGCA
GGCAGGCACATGAAACGGTCCACGATGGAGCTCGGGGGGCATGCACCGGTGATCCTCTTCGACGACGCCGACA
TCGAGGCGGCAGCCGATGCGCTTGCCGGCCAGAAGGTGCGTAATGCCGGCCAGGTCTGCATCTCTCCGACGCG
GTTTTACGTGCAGGCGAAAGGGCACGACCGTTTCCTGGCACGCTTTGCCGAGAAGATCGCATCGACAAGGGTC
GGCAACGGTTTCCAGGAGAGCGTACAGATGGGCCCGCTCTGCCACGGCCGACGCGTCGCTGCCATGGAAGGTT
TCGTCCAGGACGCGCGCGAGCAGGGTGCCGAGATCGTCACCGGCGGCGAACGCGTCGGGAACGCGGGCTTTTT
CTATGCGCCAACCGTGGTCGCCGCCGGCACCGACGAACTGCGCCTGATGAAGGAGGAGCCCTTCGGACCGATC
GCCGTCGTCACGCCCTTCGGCGATTTCGACGAGGTGATCCGGCGCGCCAACAGTCTGCCCTTCGGTCTGGCGT

CCTATGTGTTCACAGGCTCGAGCAGCCGCGCACAGAATGCCGCACGCGCTCTTGCAGCCGGAATGGTGAGCGT
CAACCATTTCGGCCTGGCGTTGCCGGAGACTCCCTTCGGCGGAATCAACGACAGCGGCTACGGGAGCGAGGGA
GGCTCCGAGACATTCGACGGGTATCTGAACACGAAGTTCGTCACGCGTTTCGACCAAATCCTGCAGTGATTGC
TCTGCACCGCTGTGGCATGGTCGCTGTTCCAGCTCTGGTACGCGTCTCCCCTACCCTTCGTCTTCGGCTTCGG
CATCCTCAACGATACCGAAGCTCGCGCCATCCATCTCGGTTTCGCGCTGTTCCTGATTTTTCTTGCCTATCCC
GCGCTGAAAAGTTCGCCCCGCGACCGGGTGCCGCTCGCCGATTGGGTGCTCGCGGCGCTCGGCGCCTTCGCGG
GCGCCTATCTGTTCCTCTTTTATGGCGAGCTTGCCGGACGGCCCGGCCAGCCCTCGATGCTCGATCTCGTCAC
CGGCACCGCTGGCATCCTGCTCCTGCTCGAGGCCACGCGCCGCGCGCTCGGTCTGCCGATGGTGTTCGTCGCC
GGCGTCTTCATCTTCTATACCTTCGCGGGGCAGTACATGCCCGACGTCATCCAGCATCGCGGCGCTTCGCTGG
TGAAGTTCATCAACCATCAATGGCTGACGACGGAGGGTGTTTTCGGCATTGCGCTCGGCGTGTCGACGAGTTT
CGTTTTCCTCTTCGTCCTGTTCGGAACGCTGCTCGAGAAGGCCGGTGCCGGCAACTGGATGATGCAGATCTCC
ATCGCGCTGCTCGGACATCTGCGCGGCGGCCCGGCCAAGGTGGCGGTCGTCTCGTCGGCCTTGAACGGCGTCG
TTTCCGGCTCGTCGGTCTCCAACGTCGTGTCGGGCGGCATCTTCACCATTCCGCTGATGAAACGCACCGGGCT
TTCCGGCGTGAAGGCCGGCGCGATCGAAGCCACGGCCTCGATCAACGGCCAGATCATGCCGCCCGTCATGGGC
GCCGCGGCATTCCTGATGGTCGAATATGTCGGCATTCCCTACTCGGAAATCGTCAAGCATGCGCTGCTGCCGG
CGGTCTTCTCCTACATAGCGCTTCTCTACATGGTGCATCTGGAGGCGGTGAAGCTGAACATGCAGCCGATCCC
CCAGCGTCCGACTCCGATGCGCGAGCGGTGGCTTCGCATGGGCCTCGGACTTGCCGGCAGCGTGCTGGCAATC
TGCCTTCTCTATTACGGAATCATCGCCATTCGGGCCGCATTCGGAGAAAGCGCGCCTCTGCTGCTCGCGATTG
CCGGCGTGGCGCTCTACATCGCGACGATCTGGTACTCGTCGCGCTATCCGGACCTGGAGCTCGACGATCCAGA
CGCGCCGATCCTGGAACTGCCGCGGGCATGGGATGTGACGCGGACCGGGCTGGATTTCCTCATCCCGATCGTC
GTCCTGCTCTGGTGCCTGATGGTCGAGCAGCTTTCGCCCGGGCTTTCGGCCTTCTGGGCAACGCTGGCGATCA
TCGCCATCGTCGCCACCCGCAAGCCGCTGATGGCGATCTTCCGGAAGGAGGATGTTGCTGGTTCGGTCCGCGC
CGCCGCCTGGGACCTGACCGACGGCCTGGCGCTCGGTGCCCGCAACATGATCGGCATCGCGGTCGCCACTGCC
ACCGCCGGTATCGTCGTCGGAACGATCACGCTCACGGGCCTCGGCCTGATGATGACGGAGCTCGTCGAGTTCA
TTTCGGGCGGCAACGTCATCATGATGCTGATCCTCATCGCCGCGATCAGCCTAGTGCTCGGCATGGGAATTCC
GACCACCGCCAATTACATCCTGGTGGCCACCCTCATGGCGCCGGTCGTCGTCGAGCTCGGCTCGCAGGCGGGC
CTCGCCATACCGCTGATCGCAGTCCACCTCTTCGTTTTCTATTTCGGAATCATGGCCGACATCACGCCGCCGG
TTGGGCTGGCTGCCTTCGCCGCGGCGGCGATTTCCAAGGAAGACCCGATCGCCACGGGCTTTCAGGGTGCGCT
CTATTCGCTGCGCACCGCGATCCTCCCGTTCGTTTTCATATTCAACCCGGCGATCCTGCTGATCGGCGTCGAC
ACCTGGGCGCATACGATCTGGGTGGCGGCCATATCGCTCGCTGCGATCCTCATTTTCTCGGCCGCGACGATGA
ACTGGTTCGTGACAAAGAGCCGGTTGTGGGAGAGCGCGGTGCTGCTGCTCGTCTGCTTCACGCTGTTCCGTCC
CGATTGGTGGCTCAACCAGATCTCGCCGCCCTATGAAGAACTGCCCGCCTCAGAATTCCTGCGAGCTGTCGAG
GAGGCTCCGGCGAAAAGCCGGATCAACTTCGTGGTTCAGGGCTTCGACCTGATGGGCGACGAGGTCCGCAAGA
CCGTCAACGTGCCGCTCGGCGAGCCCGGCGAACCGCTTCAACGGCTGCAATCGATCGGGCTCACGGTCACACC
GGCGGGCGAGAGTCTGATGATCAGCAATGTCGACTTCGGCTCCTATGCAAAGCGCATCGGCCTCGATGTCGGC
TTTGACATCGTCGCAGTGCTGAGAAAAGCCGAGCAACCGTCAAGCGCCATCCCGGTGGGTATCGCGCTGGCTG
TGACCGCGGGAATTGCCGGCTTACAGTTCGCGCGCAAAAGGGCCGATCGCAGCGAAGCCGGCCTGACGGGGGC
CGCGCGCAAATGAATGAACATCAGCATATCTCAGCGGACGGACACGATGTCCGCCGTTATCGTCACTGGGCCG
GGTACCGTGCGCATAGAGACGTTGCCGTTGCCGCAACCGGGGCGCGGCCAGGTGCGGATAAGGCTCGAAGGCT
GCGGCGTATGCGCCTCCAATCTCGTTCCCTGGGCGGGTCCGGAATGGATGCAGTTTCCGACGGAACCCGGCGC
GCTCGGACACGAGGGCTGGGGAATTATCGACGCGGTCGGCGAGGACGTGCACGGTTTCGCGCCGGGCGATCGC
GTGGCGGCGCTTTCCTACCATGCCTATGCCACGCACGATATCGCCGATCAGAGCGCGATCGCGCCCTTGCCGG
CGTCTCTTGCAGGTCAGCCCTTTCCCGGCGAACCGCTCGGCTGTGCCTTCAACATCTTCCGGCGCAGCGCGAT
CAGTCCGGGAGAGACCGTGGCGATCGTCGGCATCGGTTTTCTCGGGATTCTTCTGACCGAGCTCGCAAGCGCT
GCCGGCGCGCGGGTGATAGCGATATCGCGCAGGCCGTCTTCGCTCGCCGCGGCCAAGCAGGCCGGCGCGAGCC
TAGTGATTCCCATGGACGATCACTGGCGGATCATCGAAAAGGTTAAGCAACTGACCGGCGGGCGCTTCTGCGA
TTGCGTCATCGAGGCGGTCGGCAAGCAATGGCCGCTCGATCTTGCCGGCGAACTGACGAAGGAGCGCGGCAGG
CTCGTCGTCGCAGGATATCACCAGGATGGCCCGCGGCAGGTGAACATGCAGCTCTGGAACTGGCGCGGCCTCG
ACGTGATCAACGCGCATGAACGCGATCCTGCCGTCTACATGCGCGGCATGAACGAGGCGATCGAAGCGACCGC
AGCGGGTCGGTTCTCTCCCTCGCGGCTCTACACCCATCGGTTCCCGCTCGAAGAACTCGGCGCCGCGCTCGAC
ATGACGCGCGACCGGCCGGAAGGTTTCATCAAAGCGCTGGTGATCTGCCATGAGTGAATGACCCGCCTGCGGC
TCCGTTCGCTGGTCCTGCTCCTGCTTGCCGCCTTCCCGGCGGCGGCGGCCGAGGAGCCTGCCGGCTACCGCGA
GAGCGAGTACCGCGCCGAGGTGCCGGCGACGCTTTCCGGTGCGACGGTCGTGTCGACCGAAGCTGCCCATGCA
TTGTGGAAGACCGGTCAGGTCGCTTTCATCGACGTCCTACCGCGGCCGCCGAAGCCGACCAATCTCCCCGAGG
GGACGGTCTGGAACGAAAAGCCGCGCCTGTCCATTCCATCTGCCCTATGGCTGCCGAATGTCGGCTATGGCCA
GCTGGCCGACGCCATGCACGCCTATTTTCGCGCCGGCCTGGAAAAGGCAACCGGTGGCGACAAGGACCGGCCG
GTGCTGTTCTTTTGCCTCGACGGCTGCTGGATGTCCTGGAATGCCGGAAAGCGCGCCCTGGAATATGGCTACA
CACGTGTCTTCTGGTATCCGCAGGGCACCGATGGCTGGACGGCCGCCGGCTATCCCACGGAGCGCATCGAGCC

GGAGCCCGGCGGCCGGTGAATGGCGGGCAAACAACTGCTTTTGATGGATGGAATCCATAAACGATTCGGACCG
CTCATCGTCAGCGCAGGCGTGACCCTCGACGTCGAGGAGAACGAGATTCACGCGCTGCTTGGCGAAAACGGCG
CCGGCAAGACCGTTCTGATGAGCATTCTTTGCGGTGTTCTCTCGCCCAATGAAGGGCAGATCGTCTTCAAGGG
ACGGCCACTCAAGCTAGGCTCCCCCCGCGAAGCCATCAAACATCGCATCGGCATGGTCCACCAGCACTTCATG
CTGGTCCCCAACCTCACGGTCGCGGAAAACTACGTGCTAGGACAGGGCTCGCCCTTCAGCATGATCCGCGACA
TGAGGGCGGTACATGCGCGCATTCTGGAGCTCAGCGACCGCTACGGTCTCGACGTCGCGCCCGACGCGCTCGT
CTCCAGCCTGTCGGTTGGCGAACGGCAGCGGGTCGAGATCTTGAAGGTGCTCTACCACGGCATCGAACTGCTG
ATCCTGGACGAGCCGACCGCGGTCCTGACGCCTCAGGAAACCGACCGTCTCCTGCATCTGCTTCGCCAACTCG
TCGCAGACGGAAAGACGGTCATCTTCATTTCGCACAAACTGGACGAGGTGATGCGCGTCAGTGATCGCGTCAG
CGTCATGCGCGATGCCAAGGTCGTCCGTACGGTGAAAACGTCCGAGACGACCGCGCGCGAGCTGGCGCGCATG
ATGGTCGGCAGAGACGTTCTGATGGATTTGCCGCGGGCACCTCTCGAGCCCGGCCGTGTCGTGCTGTCGGTCG
AGCATCTCAGCTGCGACGGCGAAGCCGGGCTTCCGGCGCTGCGAGACGTATCTTTCGAGGTCAGGGCCAATGA
AATAGTCGGGATCGCAGGCGTCTCCGGCAATGGCCAGAGCGAGCTGGCGCTGGCCCTGACGGGGCTTCTTCCG
GTTGCCTCCGGCAGCGTCACGCTCGACGGGACCCAGCTGGCGGGCCTGTCGTCCTACGAGATCAATCAATTGC
CTATGGCGCATATCCCCGAGGACCGCCACAGGATGGGCATCGTGCTGCCGCTGCCACTGACGGAGAATGTGAT
CCTTCAGCGGTTCGACAAGCCTCCCTTCTCGTCTCGTGGATTGCTCGACCTGAACGAGATCACGAGGCAGACC
CGTGATCTCATGCGCCGCTTCAAGGTCAAGGCGTCGGGACCGGGCGACCGCATCCGGAATTTGTCGGGCGGCA
ATCAGCAGAAGCTCGTCGTCGGGCGCGAGTTGGACCGCCGGTTCGATTTCCTGCTGATCAATCAGCTTACGCG
CGGTATCGACATCGGAGCGACCGAGCTGGTGATGCACAAGATTCTCGAGCAGCGCAGCGCCGGCAAGGCCATT
CTGCTGATATCCACGGAGCTCGAGGAATTGTTCACGCTCTCCGACCGCATCCTCGTCATGTATGAAGGCCGCC
TCGTCGGCGAGATTCCGCCCGACCGCAGCCGTCTCGAAGAGATCGGGCTGCTGATGGCCGGCAAATCTCCGCT
ATCGGCAGCTTGAATGCCGAATGCTTCCGACATCCTCATCGACACGCTTATCGAATGGAAGGTCGACGTCGTC
TTCGGCCTGCCCGGAGACGGCATCAACGGCATCATGGAGGCGCTCAGGAGGCGGCAGGATCGCATCCGGTTCG
TTTCCGTGCGCCACGAGCAATCGGCCGCCTTCATGGCATGCGCCTATGCCAAATTCACCGGCAGACTCGGCGT
CTGCCTCGCCACATCGGGCCCGGGCGGCACAAATCTGCTGACCGGCCTCTACGATGCGAAGCTGGACCAGATG
CCGGTGCTCGCGATCACCGGCATGCAGTATCACGACCTGATCGAGACGTTCTCCCAGCAGGACGTCGATCTCA
CCCGCGTCTTCGAAAACGTCGCCGTCTACAACGCGCAGGTGAACGACGCAGCCCACATGGAAAACCTCGCGAA
CCTTGCCTGCCGGTCGGCGCTCTCCAAGCGCGGCGTGGCGCATCTTTCGATCGCCAACGACGTGCAGGAGCGG
ATGGCTGATGGCGGGCGGTCCCGCCGCAACCGGCCGGGGCACATGCCAAGCCGGTATTTCGCCGGCCAGCTCG
TGCCGCTAGAAAGCGATCTTTCGAGAGCGGCGGATCTCCTGAATGCGGGCAGGAAGGTGGCAATTCTTGCGGG
GCGCGGCGCACTCGAGGCGAAGGAGGCTCTGCGCGAGACGGCGGAACTGCTGGGCGCGCCGGTGGCAAAGGCT
CTTCTCGGCAAGGCGGTGCTGCCCGACGATGATCCGCTGACCACCGGCGGCATCGGCATACTCGGCACCGCGC
CGTCGCAGGAGATCATGCAGCAATGCGACACGCTGCTCATCGTCGGCTCGACCTTCCCCTATATCGAATATTA
TCCCAGGCCGAACACGGCAGCCGGAATCCAGATCGACCGTGACCCGCAGCGCATCGGTCTGCGCTACCCCGTC
GAGGTCGGGCTGGTCGGCGAGGCAGGCGAGACCCTCCGCATGCTCAACAAGCGGCTCAAACGCAAGCAGGACA
GGTCGTTCCTCGAGCGGGCGCAAGAGCAGACGCGAAACTGGCGCCGGGAACTGCGGGCGATCGAAGGCGACAG
GAGTTCTCCCTTGAAACCGCAGGCGGCGGTCGCGGCTTTCGGCCGGCGCATCGCGAAGAAGGGTATCGTCGTC
ACCGATTCCGGACAGAACACCGAGCTTGCGGCCCGTCATGTCGATCTCGGTGCGGACCACATGTTCGCGGTCT
CCGGCGCCCTCGCCTCGATGGCTTCGGGCCTGCCCTATGCTATCGCGGCGGGTATCGCGATGCCGGACCGCCC
GGTCTATGCGGTCGTCGGCGACGGCGGCTTCGCGATGCAGCTCGGCGAATTTGCGACAGCCGTCGGATACCGG
ATTCCTTTGAAGCTCCTGGTGATCCGAAACGACATGCTGAACCAGATCGCCTGGGAGCAGATGATGTTTCTCG
GCAATCCGCAGTTTGCCTGCGAATTGCCGCCGATCGATTTCGCCGCGGCGGCGGAGGCCATGGGCGGCCGGGG
CTTCACCGTCCGGTCCTTCGGCGAACTGGACGGGATCATGGATCAGGCCTTCGCCGTGGATGGGCCGGTGGTC
ATCCAGGCGCTGGTCGACCGCTACGAGCCGCTGATGCCGCCGAAAATGCCGGCGGACTACGCCCGGAACTTCC
GCGCCGCGCTTCCGGAAACGCCGGGGCACGAGAAGATCGAGGAAAACCTCAGGAATTCCTCCGTCGGCAGGAA
GGTAACCGACGAGGAGCCGCAGCCGCCGCATGAGGCGGCGCCGGACGAGAACACCGGCGAATTGCCGGGAATT
CCGTAGATGGACAAGGTCATTCTGATCACCGGCGCCTCCGGCGGCATCGGCGAGGGCATCGCTCGAGAACTTG
GCGTTGCCGGTGCGAAAATATTGTTGGGTGCACGCCGCCAGGCCCGTATCGAGGCCATTGCCACCGAGATTCG
AGACGCAGGAGGTACCGCCCTGGCGCAGGTGCTGGACGTGACCGACCGTCACTCCATGGCAGCCTTTGCGCAG
GCCGCGGTCGACACTTGGGGACGCATCGACGTCCTGGTGAACAATGCCGGCGTCATGCCGCTCTCGCCGCTTG
CCGCAGTTAAGGTGGGCGAGTGGGAGCGCATGATCGACGTGAACATCAAGGGCGTGCTTTGGGGCATCGGGGC
CGTATTGCCAATCATGGAGGCACAGCGTAGCGGGCAGATCATCAATATCGGCTCGATCGGAGCGCTGTCGGTC
GTACCCACAGCCGCGGTATATTGCGCAACGAAGTTCGCAGTCCGCGCGATCTCTGACGGACTGCGCCAGGAGA
GTACCAATATCCGCGTCACCTGCGTCAATCCCGGCGTCGTCGAAAGCGAACTTGCTGCAACCATCACTCACGA
GGAGACCATGGCGGCGATGGACACCTATCGCGCCATCGCGCTTCAGCCTGCCGACATCGCCCGTGCGGTCCGG
CAGGTGATCGAGGCCCCGCATTGCGTGGACACCACCGAGATCACGATCAGACCGACCGCATCGGGCAATTGAA
TGAACGCCGTAGAACTGCGCGCGATTCAAGCGCCGATAAAGGAACGCTATCGTGCAGAGCCCGGGGCAGCCCG
TTTCACGCTGAAGGCGCGCGGGACACTTGACGAAAAGAACATCGCCTGCAAGGTCGAGACTGGGCACGCACTG

GCAGTGGCCGGCCTCCACAGGGCTACTGGCGGGACCGGCCTTGAACTTTGCTCGGGCGACATGCTGCTGGAGG
CCCTCGTCGCTTGCGCGGGCGTGACGCTCAAGGCCGTCGCAATGGCCCTCGAGATTCCGCTGTCAGCCGGCAC
AGTATCCGCTGAAGGCGATCTCGACTTTCGCGGCACTCTGGGTGTCGTCAAAGATGTACCGGTGGGTTTCGAC
CAGATCCGCATTATCTTCGAGCTTGAGACGGACGCCCCACAGGAGAAGTTGGATCAGTTGCTAAAGCTGACTG
AACGCTACTGCGTCGTGTATCAGACCATCCGCAACGGGCCCTCGATGGCTGTCGAGATGAGACGCTCGGTGTA
AGTGTCGAGATTTAACCCTACCAGAGAAACAGGTTCGATTTACGATGCCGCTGCGGCGTGGCGAGACCGGTGC
CTCTTGGCGGATGGTTCGGTGCTATCAGATGAGGCCCTCTGGGCGGTCGCCCTGCTCGACGAACTCGACGAGC
GCTTCGTCAAGAATATCGACGCAGGCGAAGGCGATTTCCTTTCCAAGCTGGAGGTGCAGCTGTCGTCCGGCTC
GCCCCAGTGCCGACAGTTGATGGCTGAGAGCCTGTGGTTGCTGCTGCTTTTCCCGTCGAATGTCGGCGCCGCC
AGAAAGCGCGAAAACGTGCGCGAGGTCTGGTCTTGGTCGGGCGACGAACTGGACGAACACCATCCGTTTGTCG
ACGACAAGGTTCTCGCGGGCATCGGCTCGGCCGGTACAGCCTATAACACCCACCGTTGGCGCGAGCTTGTTTT
TCTGATCGGCGCGGTGCGCGACTTCAAGCGACGTGAGAGCGCCCAGCGCCACGACATCCTCCGTGAGCCATGG
GCGTTTTCCGGCTGGCTGAGCAGCCTGTCCGAGGCGCGCCACCGGCAGTTGCTACAGATCCTGCCGCACCTGT
TATTTCCAGAAAATTTCGAGCGGATCAGCTCCGAGGGCGACAAGCGCCTGATCCTAGCCGGGTTCGGCGACGC
GCCGGAGAAGGAAATCCGGAAGTGGAGCACCACCGAGATCGATCGCGCCTTGCTCAACCTGCGCGCTCGGCTT
GAGCAGGAGCAAGGTGTCGACATCGACTTTTACCAGGAAGAGTTCTCCGCCCATTGGAAGACCCAGACGCAAA
ATTGGCTCCTGAGCTGGAACCCTTCCAGGTGGACATGGGACACCCTCGCCGAAGACAGGGCCGCCACGAACAT
TGGCGAAAAGGCTGAGAACCGCTGGCGCTGCGCATCGACGAAGGCGCGCGAGGGCGACCGCGTCTTCCTGGTG
CGGACTGGAGAGCCACCAAAGGGCATCGTCGCCGTTGGCAAGGTCACCCGTGCACCATATGAAGCCCCACATT
GGGAAGCGGCCCGCGCAGAAGCGGGCGAAACGACGCGCTTCATCGACGTCGCTTTCGACGCCGTTCGGGACGC
GTCGATCGACCAGATCGTGAAGCTCGAAGAGTTGGAGCGTCGGGAAGCCGGTCAGGAGTGGAACCCTCAATCA
TCGGGGATAGAGATCAAAAGCAAGCCGGCGCGGACACTCGAGAGGCTGTGGAAGGAGTTACCCCCCGTTGCCG
GACCATCCGCGCCCGATGATGAAACAGCTGCTACCAAGGCGACACGCTCGACCGGATCGCCCCTGAACCTGAT
CCTCTACGGGCCGCCAGGGACCGGAAAAACCTACCGGCTGATGAACGACTATGTGCCGAACTACCGCGATGAG
GCCGAGGACCGCTCCGAATTCATTACCTTCCACCAGAGCTACGCCTACGAGGACTTCGTCGAGGGCATCCGGC
CGTTGGCCAAGAACGGCAGCATCACCTACGAGGTCCGACCCGGGGTACTGAAACGACTCTGCGCACGCGCACG
CCAGTCGCCCCAGAAGCGCTTTGCCCTATTCATCGACGAAATCAACCGGGGAAACATAGCTAAGATCTTCGGC
GAGTTGATATCATTGGTGGAGGTCGACAAGCGGATCCACACCGACACGTCGGGGAAGCGGTTACCCGGCTGCA
AGGGCCTGGAAGTCACGTTGCCCTACTCGGGCGAACGTTTTGGCGTGCCAGCGAATGTCGACGTGATCGGCAC
GATGAACACCGCGGATCGCTCGATCGCGCTCCTCGACAGCGCGCTGCGCCGCCGCTTTCGGTTCGAGGAGCTC
ACACCTAAACCCGAGCTTCTGCAAACGGTGGATGATGACGCCGGCGACACGATCGACCTGCGACAGTTGCTCG
CGGCCATGAATACGCGCCTATCGCGGCTGCTGCATCGGGATCAAACGTTGGGACATAGCTACTTCTGCAACGT
CAAGTCGTTTGGCGATCTTCGCAAAGTCTTCGCACGCGAAATCCTGCCCTTCCTGCAGGAGGCCTTCTACGAC
GACTGGCGACAGATCCGGCTTGTCCTGGCCGACCAGACTGTGGGCGAGGAATTCCAGCTGGTACGGGCGCATA
CGCAAAGCGCCCAGCGGCTCTTCCCCCTTGCCGACGGCAACGAGATAGGCGACGGCGAGACATTCGAAGTCGT
TCGCGAGGACGACATCACGGCCGACGCGATCCGCAAAATCTACGAGCCACCGGAATGAATGAACGACAAGATC
ACCAACTGGACCAGATCCGACGACGCTATGGTCGAAACGGCCATTCGTCGCGGAGCGACACGCCGCGAGCTCC
TGCAGATGATGCTGGCCGGCGGTGCCGCCCTCTCCGCCGGCAGCCTGGTGCTCGGCCGAGCCGGGAATGCGGT
TGCTGCAACGCCGGTCTCCGGCGGTACGCTCAGAGCGGCCGGGTGGTCGGCTTCCACCGCCGACACGCTCGAT
CCGGCCAAGGCATCGCTCTCCACCGACTATGTCCGCTGCTGCTCCTTCTATAACCGCCTCGCCTTCCTCGATA
AAGGCGGCACGCCGCAGATGGAGCTGGCCGAAGCCATCGAAACCAAGGATGCGAAGACCTGGACCGTCAAGCT
GAGGAAGGGCGTCACCTTCCATGATGGCAAGCCGCTAACAGCCGACGACGTGATCTTCTCGCTGAAGCGCCAC
CTCGACCCGTCTGTCGGTTCAAAGGTCGCCAAGATCGCCGCGCAGATGACGGGCTTCAAGGCGGTGGACAAGC
AGACGGTCGAAATCACGCTTGCGAGCCCTAATGCCGACCTGCCGACGATCCTCTCGATGCACCACTTCATGAT
CGTCGCCGACGGCACCACCGACTTCTCAAAAGGGAACGGCACCGGCGCTTTCGTGAGAGAAGTCTTCGAGCCC
GGCGTGCGCTCCGTCGGCATCAAGAACAAGAACTACTGGAAGTCCGGACCGAACGTGGACTCTTTCGAGTACT
TCGCGATCAGCGACGACAGTTCCCGGGTGAATGCGCTCTTGTCCGGCGACATCCACCTCGCCGCCACGATCAA
TCCGCGCTCCATGCGCCTGGTCGAGAGTCAGGGCGCCGGCTTCGTCCTCTCGAAGACGACCTCCGGCAACTAC
ACCAACCTCAACATGCGGCTGGACATGGAACCCGGCAGCAAGCGCGACTTCGTCGAAGGCATGAAGTATCTCG
TCAACCGCGAGCAGATCGTTAAGTCGGCGCTTCGGGGTCTGGGTGAGGTCGGCAACGACCAGCCGGTATCCCC
GGCCAACTTCTATCACAATCCCGACCTGAAGCCGCGCGCCTTCGACCCCGAGAAGGCGAAGTTCCACTTCGAG
AAGGCCGGCATGCTAGGCCAGTCGATCCCGGTGGTTGCCTCCGATGCAGCGAACTCCGCGGTCGATATGGCGA
TGATCATCCAGGCCTCCGCTGCCGAAATCGGATTGAAGCTCGATGTCCAGCGCGTTCCCGCAGACGGTTACTG
GGATAATTACTGGCTGAAGGCGCCGGTCCACTTCGGCAACATCAACCCGCGCCCCACGCCGGATATCCTGTTC
TCTCTGCTCTACTCCTCGGAGGCTCCGTGGAACGAGAGCCAATACAAGTCGGAGAAGTTCGATAAGATGCTGA
TCGAGGCGCGTGGCTCGCTCGACCAGGAGAGGCGCAAGGCGATCTATAATGAGATGCAGGTGATGGTCGCTAG
TGAAGCCGGCACCATCATTCCGGCCTATATCTCCAATGTCGACGCGATCACCGCCAAGCTTAAGGGCCTGGAA
GCCAATCCGCTTGGCGGGCAGATGGGTTATGCTTTTGCGGAATATGTCTGGCTCGAGGCCTGAATGTTCGGCA

AGAAATCCATTTCGGAAGAACGGAGAGCCGAAGCAGCGGTCCAGCCCGTCGTCGAAAACGAACTGCAGGCCGC
GCACGCCCCTGTCCAAGTTCGGAAGCCAGCCCAGGTTCCGGCAACTGCGAAACCCGAGAATGCCGAACAATAT
TACAGCTTGAAGAAGGAAATCTTCAGCGCCCTCATCGCGACGATCGACGTTGCAGCACTCTCCAATATGGATG
GCGAGCAGGCGCGCAACGAAATCGGCGCGATTATAAACGACATCGTCGCGGCCAAGAAGGCCGGCATTTCGAT
GGCCGAGCAGAACGACCTTCTCAGCGACATTTGCAACGACATCTTGGGTTACGGCCCGCTCGAGCCCCTGCTC
GCACGCGACGACATCGCCGACATCATGGTCAATGGCGCAAACCAGGTCTTCATCGAAGTCAACGGCAGGGTGC
AGGAGACGGGAATCCGCTTTCGCGACAACGAGCAGCTACTCAATATCTGCCAGCGCATCGTCAGCCAGGTTGG
CCGGCGCGTCGACGAATCGAGCCCTATCTGCGACGCGCGTCTGGGCGACGGTTCCCGTGTCAACGTGATTGCG
CCTCCGCTGGCAATCGACGGCCCCACGCTGACGATCCGCAAGTTCAAGAAGGAAAAGCTGACCCTCGACCAGT
TGGTCCGGTTCGGCTCGATCTCGCCGGAGGGCGCCGAGGTTCTCAAGATCATCGGACGTGTGCGCTGCAACGT
CCTCATTTCCGGCGGTACCGGCTCCGGCAAGACAACGCTTCTGAACTGCCTGACCGGCTATATCGACCACGGC
GAACGCGTCATCACCTGCGAGGATGCGGCAGAGCTTCAACTGCAGCAACCGCATGTGGTGCGCCTCGAAACGC
GGCCGCCGAACATCGAGGGGCAGGGCGAGATCACCATGCGCAACCTCGTGAAGAACTGCCTGCGCATGAGGCC
CGAGCGGATCATCGTCGGCGAGGTGCGCGGGCCCGAGGCCTTCGACCTGCTGCAGGCGATGAACACGGGCCAC
GACGGCTCGATGGGAACGCTCCACGCCAACTCCCCGCGCGAAGCGATGGCACGCATCGAGGCGATGATCACCA
TGGGCGGGAGCTCGCTGCCGGCCAAGACGATCCGGGAGATGCTCGTCTCGTCAGTCGACGTGATCGTGCAGGC
AGCGCGACTTCGCGATGGTTCGCGCCGGATCACCCACATCACCGAAGTGCTCGGCATGGAAGGCGAGGTGGTC
ACGACGCAGGATCTCTTCGTCTACGACATTCTCGGCGAAGACGAGAAGGGAAACATCATCGGAAGACACCGGT
CGACCGGGATAGGTCGCCCGGCATTCTGGGATCGCGCACGCTATTACGGCGAAGAGGCCCGGCTTGCCGCCGC
CCTCGACGCGGCGGAATTGAAGGCCGCCGCCTGAATGTCTATCAGTCTTCGCGGCCGCAGCGTCCTGACGCTC
GACGAATTCACTTCAGGGGAGATCGGGTTTCTGCTGCATATCGCCGCCAGGGTGAAGGCCGAAAAGCGAAGAG
GCAATGAAATCCCCCTTCTCAGCGGCAAGAACATCGCGCTGCTTTGCGAGGAGGGTTCGATGAACGCCCTCTT
CGGATCTGAAGTTGCGGCCTTTGATCAAGGCGCGCGAGTCGTTGGTCTGACGTTGCCCGCCGGCCATGGCGGG
CAATGCGCATCACTGAAGGATATGGCCCGTGTGCTTGGCAGGATCTACAACGCGGTCGAATATTGCGGTCGCA
GCCAGGGCGCCGTCGAAGAACTGGCGGAATACGCCCGCGTTCCCGTCTACAATGGCTCTTCCGAAGAATGCCA
TCCCATCCAGGCTCTGGCAGACTTCTTCACGATGCAGGAAGCTGTCGACAAGCCGTTGCAGGAGGTCACGCTA
GCGTTTCTGGGCGACGGTCGCAGTCCTATCGCCCGTTCGCTCGCGACCGGAGCGGCCAAGCTCGGCATGGACT
TCCGCATCGTCTCGCCGCCAATTTTCTGGCCAGCTCCTTCCTTCATCGACGACGTGACCTCGGAAGCGCATGA
GAATGACGGGAGTTTCACGGTCCTGGAGCATGTCGGCGCCGGGGTCTTAGGCGCCGACTTCGTCTACACGGCT
TCGTGGCTGGGAGAGCAAGAAGCGGGCTGGGCCGAGCGCATCAGGCTGCTGGCGCCTTTTGGCGTCAGAGCCA
ATGTCATGGAAGCCACTGGCAATCCGGGTAGCAAGTTCATGCATTGCCTGCCTGCCTTCCACAACAGTGGCTC
AAAGGCCGGAGCCCGCGTCGCGAAGCAGTTCGGTCTCGACTGCATGGAAGTCTCGGACACGGTTTTTGAATCG
AAAGCTTCTGTGGTGTTCGATCAGGCGGAAAACCGGATGCACACGATCAAGGCCATTTTCATCGCGACCATGG
GGGACCGGGAGACCGATGCAAGAGATAGAGACCCTTAGATGGCACTGAAGGCAAAACCGCATCCGAGCCTGTT
GGCCATTCTCTTCCCCGTCGCCGTCACCGCCGCTGCTGTCTATGTCGTCGGCTGGCGCTGGCCGGGGCTCGCC
GCCGGCATGTCGGGGAAGACGGCATATTGGTTCCTGCGGGCGGCGCCCGTGCCAGCGCTACTGTTCGGGCCGC
TCGCCGGCCTGTTGGCCGTTTGGGCCTTGCCATTGCACCGGCGAAGGCCAGTCGCAATGGCGAGCCTCGCCTG
TTTTCTGACGGTCGCGGGCTTCTACGCGCTCCGTGAATTCGGCCGGCTGTCCCCCTCTGTGGAAAGCGGTGCG
CTATCATGGGACCGGGCGCTGTCCTACCTCGACATGGTCGCTGTCGTCGGCGCGGTCGTTGGCTTTATGGCCG
TCGCTGTCTCCGCGCGCATCTCCACCGTCGTGCCCGAACCGGTAAAGCGCGCTAAGCGCGGAACCTTCGGTGA
CGCGGACTGGCTGCCGATGGCGGCGGCTGGAAAGCTGTTTCCGCCGGACGGCGAGATCGTCATCGGCGAGCGC
TACCGCGTCGACAAGGACATTGTCCACGAACTGCCCTTCGATCCAAACGATCCGGCGACTTGGGGCCAAGGCG
GCAAGGCCCCTTTGCTGACCTACCGCCAGGACTTCGATTCCACCCATATGCTGTTCTTTGCGGGCTCGGGTGG
ATACAAGACGACCAGCAACGTCGTGCCGACGGCGCTGCGTTATACCGGGCCGCTCATCTGCCTCGATCCGTCG
ACCGAGGTCGCGCCCATGGTGGTCGAGCACCGAACCCGCGTGCTCAGCCGCGAGGTGATGGTTCTTGATCCGA
CGAACCCGATCATGGGCTTCAACGTGCTCGACGGGATCGAGCACTCACGGCAAAAGGAAGAGGACATCGTCGG
CATTGCCCACATGCTGCTGTCAGAAAGCGTGCGCTTCGAAAGCTCGACCGGCTCCTACTTCCAGAACCAGGCG
CACAATCTCCTGACCGGTCTGCTCACCCATGTGATGCTGTCGCCCGAATATGCCGGACGGCGGACGTTACGCA
GTCTCCGGCAGATCGTATCCGAGCCAGAGCCGTCGGTGCTGGCGATGCTGCGTGACATTCAGGAGCATTCCGC
GTCCGCTTTCATCCGCGAAACGCTCGGCGTCTTCACCAATATGACCGAGCAGACTTTTTCGGGCGTCTACTCT
ACGGCGTCGAAGGATACGCAGTGGCTGTCGCTCGACAGCTACGCCGCACTCGTTTGCGGCAACGCGTTCAAGT
CGAGCGATATCGTCTCGGGCAAGAAGGACGTGTTCCTGAACATCCCCGCCTCGATCCTGCGCTCCTATCCGGG
CATCGGCCGCGTCATCATCGGCTCGCTGATCAATGCCATGATCCAGGCCGATGGCTCATTCAAGCGCCGGGCG
TTGTTCATGTTGGATGAGGTCGATCTCCTCGGCTACATGCGCCTCCTCGAGGAGGCACGCGACCGCGGCCGCA
AGTACGGCATCAGCATGATGCTGCTGTACCAATCCCTGGGGCAGCTGGAGCGGCATTTCGGGAGGGACGGTGC
GGTGTCGTGGATCGACGGATGCGCCTTCGCCTCCTATGCCGCAGTTAAGGCGCTGGACACGGCGCGCAACATT
TCGGCGCAATGCGGAGAAATGACGGTTGAGGTAAAAGGAAGCTCCCGCAATATCGGCTGGGACACGAAGAACA
GCGCATCGCGCAAATCGGAAAACGTCAATTACCAGCGCCGGCCGCTGATCATACCGCACGAAATCACCCAGTC

GATGCGAAAGGACGAACAGATCATCATCGTCCAGGGCCACAGCCCGATCCGCTGCGGGCGCGCGATCTACTTC
CGACGGAAGGACATGAACGAGGCGGCGAAGGCCAATCGCTTCGTCAAGGCGATCCCTTGAATGGCTGAAAAAC
AACATATCGACTTGGAGGCCGCACCTGCGAGCGCCAAAGACCTGCGTATGCAACTCCTTCAACGACAGATGGA
GGAGATGGAGAAGGAGCGACAGTTCAAAGCGGTACAGGAGCAAAAGCTGACTGACTTTGCCGGCGTATTCCTT
GAGGAGCACGTCACGCCAGAGGAGGTCGCCGTCGTGCGCCGCCTCGTGGCGAACGCCGTACGCGACGGAAAAT
TCGAGGCCATGGTTTACAGCTTCCCCTCGAATCTTTGCACCGATAGTGGCCGCGCAATAAACAGCGCCGACCG
CGATTGGCCGCAGACTCTCCAGGGCAAGGCGAAGGAATTCTACGAACGATATCAAAAATTCGGCAAGCCTCAG
GGCTTCAAGCTGAAAGCTATGATTGTCAACTTTCCGGGTGGAATGCCAGGTGACGTGGGCTTCTTCCTGAACT
GGGCTCCTGATGACGCTTAGGTGCTGGAATTCCTGATCTGCTCGCTGCTCACCATTTTTCCGGACTACCTCGT
CCGCCGCTATGTCCAGGGCAAGCGTGTCGGCCGTGAGATAAATCTCTATTCCATGTGGTTTGAGCTCCGATAC
GGCATAACGGCCTGCCTTATCCTCACCATTTCACTGATCACAATGATCTTCTACTTCCATCCATCTACTAGCA
ACGTCACCGCGGTGTTTCGCACGGTAACGATCATGCCCGAGTCAACCGGGCGAGTAGCAGAGGTTTATGTTGA
CCTCAACGAGAAGGTTTCTGCCGGTGCTCCCCTCTTCCGCCTCGACGATTCCGAGCAACGGGCAGCACTTGAG
ACCGCCCGCCGACGCGTCGCCGAGATCGAGGCAGAGGCAATTGTTGCGCAGAGCGAACTGGCCTCCGCCGACG
GCCTTATCGCCCAAGCCGAGGGCGACTATCTCCAGGCGCTGAATGAACTCGAGACGCAGGTCGAATTGAACCA
GCGCAACCCTAACATCGTCGCAAGGCGCGAAATCGAGCGCCTTCAGGTCGCTGTCGACGGTCGCGATGGTGCG
CGTGCCGCGTCGATCTCCAACAAGCGGACCTTGGAAACGAAAATCGCCTCACTCCTGCCGGCGCAGAAGGCGA
GCGCTGAAGCGGCGCTGACCCAAGCCCAGGTGGAGCTCGACAAGACGATCGTACGGGCTGGAACCGCCGGCGT
AGTCCAGCAGTTCGCGCTCCGCGCTGGAGATATTGTCAATCCGATGATTCGCTCGGCCGGCATCCTGGTTCCC
GAGCAACGCCGCATTGGCTTGATCGCTGGCTTCGGCCAGATCGAGGCACAGGTGATGAAGGCCGGGATGATTG
CCGAGGCGACCTGCATCGGCAAGCCGTTCACCATCATCCCGATGGTGGTGACGGAGGTGCAAGACGTTATCGC
CGCCGGCCAAATTCGTCCGACCGACCAGCTCGTCGACGCGCAGCAACTTGCCAGTCCTGGCACGCTGACTGCC
TATCTGGAGCCGCTCTACGAGGGACAGTTCACCGGCGTACCACCTGGAAGCAGTTGCATTGCCAACGCGTACA
CGAGCAATCACGATGCACTACATGCGCCCGGCATCAGCACACCGCATTGGCTATTTCTGCACGTCGTCGATGC
GGTGGGATTGGTGCACGCCATGATCCTCAGGCTTCAGGCCCTGCTCTTGCCGGTCCAGACGCTAGTCTTCAAG
GGCCATTGAATGGCGAACGACGAACTGAGCGCCGGCTTCACCGGGAAGCCGACCGAACCGCCCACGGGTGTCC
GAAAGATAGCTAAGGTAGCCAGCGGCGCAGTTGGCCGCGAAGCGAACGGTGTCGTGGTAGGGGCCGCAGATCA
CCCATACACTGCAACAGGATTGGCGCTCACAGTCGGCGCCATTGCCTTCGCGGTCGGTTATACTATGGGTCGA
TCGTCAAGCGAAAAGGGCCGAAGTTACTGGCGATAGATGTCGACGCACAAAATTGCGATCGTAGCGATGCAGG
GCGTGCAATTGCTCGATGTGATCGGCCCGTCCGACGTGTTCGCGGAAGCAAACCGCCAGAGTGGCCTCGGCTA
CTACTCGGTAGAGGTCGTCAGCACTTCTGGCAGCCCCGTCAGAGGATCATCTGGATTGGCACTGATTCCCGAT
CGCCTAATCGACGAAGAACCGTCCGAAGTCGATACGCTGCTTGTAGCAGGCGATCCGCTCATCCAGGAGCGGC
CGCTCACCGAAAACCTCCTGCGCTGGGTGAAGCAGACCTCGGCCAGAGCCAATCGCGTTGGGTCCGTCTGCTC
CGGGGCTTTTGTGCTCGGCGAGGCAGGTTTACTTACCGGCAAGCGAGCGACCACGCATTGGTCGCAGGCATCG
GAGTTTGCAAGGCGTTTTCCGGATGTGAAGCTTGAACCAAATCGCATTTTTACAAAGGACGGCAACGTTTGGA
CATCGGCCGGGGTTACCGCTGGGATCGATCTGGCGCTTGCGATTGTCGAGCAAGACCTCGGTTTAGAGATTGC
CCTCAGAGTCGCTCGAGAGCTCGTGGTGTTTCTCAAACGGCCCGGCGGACAGACACAGTTCAGCACGCTCCTG
GCCGGGCAGATGGCCCAGAAGACGCCGATCCGTGCGGCCCAGGACTTCGTCGCCGACAACCTCGCCGCCGACC
TCACCGTGGCGGCCCTGGCGAAGCGCGTGGGAATGAGCGAACGAAACTTTTCAAGGCAGTTCAAACAGGAGGT
CGGCATGACACCGGCCGACTATGTCGAAAGCATGAGGCTAGAGGCGGGTCGCAGATTGTCCGAGGACACCAGC
ATTCCACTCAAGAGGATCGCCATTGATATAGGCTTCAGCGACGATGTCGCCTTCCGGCGATCCTTTGCCCGCA
GATTTGGCACATCCCCGGCTGCCTATCGGAGGAGCTTCGGGTCCTAGATGGGCATGCCCGCGGGAGCGATCTT
TCTCGTCTTCATGACCCTGCAGATTGTCTGCATCGCCGGCGCGCTCCTCTATCCGGACGAGTTCCGCTACCTC
TCGCCGCAGAACCTGACGATCCTGATGAAGGCGATCCCCGTGCTCGGGTGCCTCGCGCTCGGAGCCGGCGTAT
TGATGATCGCCGGTGAATTCGACCTTTCCATCGGCTCCGTGTACACGTTCACGGCCGTGCTGATGGCCTCGCT
CGTCAATGCAGGGCTGAGCGCCTTCATCGCAGCACCTATCGGTATTCTGACCGGCCTGCTGATCGGCTCGCTC
AACGGCCATATCACCCTGCGCTTCGGCCTGCCGTCCTTCATCGTCACGCTCGGTGGGCTCCTCTTCTGGCGCG
GCGCGGTGCTGCTCTACAACGGTGCCGTCCAGGTGCGTTTCGACCCGGAGCCGGTCTTCACCAGCCTCTTTTC
CGGCACTCTGTTCGGCGTGAACGCCGCCTTCATCTGGATCGTCCTCTTCGTCATAGGCTTTCACCTGCTGCTA
CACCGCCACCGCTTCGGCAACCACGTCTTCGCGACGGGTGGAAATCGCGGTGCGGCCGAGGCGATCGGCATCA
ATACCAGCCGCGTCAAACTGATTGCCTTTGCGATTGCGGGCGGCATGGCTGCGGTCGCGGGCATTCTTGCCAC
GGCCCGCGTGGGCAGCGTGCAGCCCGGGCAGGGTGCGGGTCTGGAACTCCAGGCGATCGCCGCCTGCGTCATC
GGCGGCCTTTCGCTACGCGGAGGACGCGGATCGATCATCGGCATCTTTCTCGGCGTGCTGCTCATCCACACGA
TAACCGACGTCCTGCTTCTCCTGCGGGCACCCGGCTTCTATCTCGACATGTTCATCGCCACGCTGATCGTGCT
GGCCGCCATTTTCAACCATCTAATCGAGCGGCGAGGTCTTGCGTGAATGATCAGAGCCTTCGGCTGGAACGAG
TTTCTCGTCATCGTCGCCGCGGCACAGTGGACGATCGCCCTTTCCGCGATCGCCTTTGCCGGCGGCAGCGTCG
GCGGACTGCTGGTCGCATTGATGCGCGTCTCCGAGACGCGAGCGCTGCGCCTTTTCGCCACGGGCTTCATCCG
CGTCTTCCAGGGGACGCCGCTTCTGATGCAGCTCTTTCTGGTATTCTTCGGCATGAAGATCTTCGGCTTTGCG

ATCAATCCCTGGATTGCCGCGACCATTGCGCTCGCGCTCCACGCCAGCGCCTTCCTGGGCGAGATCTGGCGCG
GCTGCATCGAGGCCGTGCCGAAGGGACAGCGCGAGGCGGCGACTGCGCTCGGCCTGCGCTATTTCCGGTCGAT
GCGTCATGTGATCCTGCCTCAGGCGGCCCGTATCGCCGTCGCACCCACCGTCGGTTTCCTGGTGCAGCTCATA
AAGGGCACCTCGCTCGCATCGATCATCGGCTTTACCGAGCTGACCCGGCAAGGGCAGATCATCAACAATGCGA
CGTTCAGTCCGTTCCTGGTGTTCGGTACCGTCGCCGCCGTCTATTTTCTGCTTTGCTGGCCGCTGTCGCTGAT
CGCACGCCGAATGGAAACACGTTTTTCCCGGGCAACGGCACGTTGAATGGGCAACAATGAGAATGGCGGCATA
GGCTTTTGCGTCTTCGTCGTCGTAATTGGATTTAGCACGGGTGCTGTTGCGCAGGAGCCAGCGAATCAATCCG
AAGCTGCGACGAGCCTGGAAGAAATCGTAGTCACCGGCGGACGATCCGCTCAGCAGATTTCTGAAATCGCGCG
AACCATTTACGTCGTCGATTCGGATCAGATCCAGGCCGAGGCCCGGTCGGGCAAGACGCTGCAGCAGATTCTG
GGCGAAACCATTCCGAGCTTCGACCCCGCCAGCGACGGAGCGCGTACGTCATTCGGCCAGAACCTGCGTGGCC
GGCCACCACTGATCCTTATCGACGGTGTCTCGATGAACTCGGCGCGCTCGCTGAGCCGACAATTCGATGCCAT
CGATCCGTTCAACATCGAACGGGTGGAGGTCCTGTCTGGGGCAACTGCAATCTACGGCGGGAACGCAACCGGC
GGCATCATCAACATCATCACGAAGAAGGGCAAAGACGCGGAACCGGGCCTGCATGCGGAAGTGACCGGCGGCA
TGGGCAGCGGCTTTGCCGGCAGCCAGGACTTCGACCGCAATGCGGCGGGTGCGGTCACCTATAACAGCGAAAA
CTGGGATGCCCGCCTTTCGATCGCCGGCAACAGGACCAGCGCCTTTTATGACGGCAGCGGCACGCTGCTGATC
CCCGACATCACGCAGACGTCCACCGCATTCAACGAGCGCATCGACCTGATGGGGTCTATCGGCTACCAGATCG
ACGACGACCGTCGCCTCAAATTTTCCGGGCAATATTTCGACAGCAAGCAGGACTCGGATTACGGGCTTTATTA
CGGGCCCTTCTTTGCAGCGCTCGCCGACCCGAGCCTGTTTGAAACCCGTTCCGGATACGAGTCCGATTTCAAT
CCGCAGACACGCCGCTCGATGTTGAACGTCACCTATACCGATAATGACGTTTTCGGTCAGCAGCTATTGCTTC
AGGGATCTTACCGGACTGAGCGGATAAAGTTCCATCCCTTCCCTGCTTCGGGCAATAGCGAGACGGGTCCTTA
CTTTTACGGCAGTTCGCAGGACACAGATTATTACGGCATCAGGGCTGCCTTGGTTGCGGAACCCACCGACGCG
CTGAAAATCACCTATGGCGTCGATGCGGATATGGATTCCTTCACGGCGCGTCAGAACATCTTCGACATGGTGG
CAGCCGGGCAATCCGGGGGGCTCGATTTCAATACGATCGGCAAGACCGGTCTTTATCCATCCATCGATGTCTC
CACCGTTGCCGGCTTTGCTGAGGCCTCGTACGAGGCTACCGACAGGCTCACTCTGAACGGCGGGGTGCGCTAT
CAGTTCGTCAACACTGAGGTATCCGACTTCATCGGAGCGGCTCAGCAGGTCGCCATCCTGCAGGGCAGAGCAA
CGTCTGCCGACACCATACCTGGCGGCGAAGTCAATTACGACGCCGCTTTGTTCAGTGCAGGCGCAACCTACCA
GTTGACGAATACGCAGCAGGTCTACGCCAATTTCATCCAGGGCTTCGAACTACCAGATCCGGCCAAGTACTAT
GGAATTGGCAACTACTCCTTCTCCGGTGGACACTACACGCTTGTCAATAGCGTGAACGTGGGCGATTCCGCGC
TCGAGGCGATCAAGACCAACTCATTCGAAATCGGCTATCGTCTCGATGACGGCACCTTCAATCTTGAAACGGC
GGCCTACTACTCGCTCTCCGATCGTTCCATCAATCTCAATCGCTCATCGCTTGCCGTTGAGGTCATCGATCGG
GAGAGACGCGTCTATGGCATCGAAGGAAAGGCGGGCGTAAAGCTCGATCATGGCTTCGATGTTGGAGTTCTGG
GGCACTGGGTCAGAACCGAGGTCAAGGGAGCCGACGGTTGGGAAAAGGACTCCGTCGGCAGCGCGAGCGTGTC
CAAACTCGGCGGCTATGTCGGCTGGACCAATGACGCTCTCAGCCTGAGGTTTTCCGGTCAGCACATCTTCGAA
CTCACCGACGCCCAGAATTTCACGATCGACGATTATACGTTGTTTGATCTAACCGGCGGCTACAGGTTCGAGA
ATACCGACACGACGCTGAATTTCGGTATCCATAATGTCTTCGATACTGATTACACCACCGTCTGGGGCTCTCG
CGCCAAAGCCCTTTACGGCGGCCTAGCCGACGAGTCGGTGTTCGACTACAAGGGCCGCGGCCGCACGTTCGCG
GTCTCGCTGACAAAGGTTTTTTAAGTGCACATCCGGTTGCTTGGCGGACTTGAAGTAAGTTCCGGAGATGGCC
AGCCCGTTCAGTTCACAACGCGCAAGGCGGCACTTCTCTTTGCCGCTCTGGTCCTGGCAGGTCGCCGCGGCCA
GCACCGCGAGCTACTTTCCGAAGGGTTTTGGCCGGGACGAGGCGATGCACAGGCACGTAACAGCTTGCGCCAA
GCCCTTGCCGAGATTAGGCGGTGGTTTCCTCCCGGCCGCAATGCCGACATTTCCGTCGAAAGCAATCAGGAAA
CCGTTGCACTGGTGGTTCCTCCAGAGAAGGTGGACATTTGGCTCTTTGAGAGAAAGCTCGCTGAAGGTGAAAC
CGCGGACCTGGCCTTCGCTGCTGACCTCTATCGCGGTGATCTTCTAGCGGGCGTGGCCGTCCCGGACGAGCTG
GAGGAAAGGTTCCGACCATATCGGAGAAGATATCGGGAGAGAGCACTGCAGTTAGTGGAACGAATGAGCCTTG
CCCTGTCCGATGCTGGATCCGCGGCCGAAGCGGCTTGCGAGCGGCTTGCGGAAACGCTGCTCGCCTCGGACCC
GACTGCCGAGCCGGCTCACCGGGCGTTAATGCGCATCCATGCTGTGAGGGGTCATGAAAACGCGGCCTTGCGG
CAGTTTGAGTCCTGCCGGGCGCTGCTGATGAAGCACCTGGAGGTGGAACCGGAGGCGCAGACGAACTCTCTGG
CGGCGTCGCTGCAATTGCGGCAGGGACAGGGACACCGGCAGACCGGGTCGACCTACAATTTCGCACCGCATAC
GCAGCCCATCCGGGCTCCCACGAGACCGCATGACCAGCCGTCGGTGGCGGTGATGCCCTTCCAGAATCTTGGT
GCGGCGGACGACGAGTATTTTGCGGACGGCGTGGTGGAGGAGATCACTGCCGCGCTATCGCGCGTTCGCGACT
TCTTTGTGATCGCCCGCCAGTCTGCCTTCACCTACAAGGGACGCTTCGTCGACGTGCGTGAGGTCGGCAGGGA
GCTTGGCGTCAGTTATGTGGTCGAGGGCACCGTTCGGAGGGGCGGCGACCGACTGCGCATTTCCGTACAACTG
ATCGATGCCGAGACGCGGACACAATTGTGGTCCGAGCGCTACGAGGGCGCGGTCGAAGACATTTTTGCATTCC
AGGACAGGATCGCGGCGCAGGTGGCCGGGGCTATCCATCCAGCCATCCGGGGCGCTGAGATCGAGTTGGCCAA
GCGCAAGCCGCCAGCTAGCCTGCGCGCCTACGACCTCGTCATGCGAGCCTACCCGCACCTTTGGGGCCGCCGC
AAGGAGGCTAACGACGAGGCTATCGAGCTACTGCGGAAAACGATCGCCATCGATCCGAGCTATGGCCGCGCGC
ATGCACTTTTGGCTTGGTGTCACGCCGCAAACGCCGCATATCTATGGACGAAGCAACCAGAACTCGAGTTGGA
AAAGGCGCTAAAAGCCGTTGAGGCTGCGGCTTCGATCAGCAACGATCCAACCGCGTTGACTGCCGCCGGTGCG
GCAATGAGCATTTGCGGCGACCAGGAGCGCGCCAGCATTTTCATCGAAAAGGCGCTCGCGCTTGATCCAAACA

ACGCATGGGCATGGACGCGCCTGGGCTGGATCGCGATTTACAGGGATGACGCCGTGCGCGCAACGGAATGGTT
CGAGCGAGCGATGATGCTTAGTCCGATGGATCCCCTCACATTCAACATGAGAATAGGATTGGCCGCATCTATG
GCCGTGGCCGGTTCTCTTTCCCAGGCGGTCGCTATCGCCCGTGAACTCATCGCCAGTTATCCGAACGTCATAA
TGTTGTATCGTTTCCTTGCCACTTGGTCGGCGATGAATGGGGATCTTGAGACTGCTCGCTGGGCCGCTCGGAA
GCTGCTGGCAGCCCAGCCAGACTTCACGATCGAGAAGTTCCGCTCACTCCCTATGTTTCGAGACATCCCCAAA
TGGGCAGACAAGGTGTCCGAAGCCTTGAGGTTGGCGGGAGTGCCCGATCACTGAATGTCGCATGAAAAGTTCC
TGTCCGCGCAGATCGGCCGCCGGACGCTGCTTGCGTCCATGGCCGCGGCCGGCGCTTCGGCGGGTCTTTCGAC
CCTCGGCGTCAGCCGCGCCTTTGCCCAGGAGCCGGAAAAGCCGGCCGAGATCATCGTTCGCGCCTGGGGCGGC
AGCTGGGTCGATGCGCTCAAGGCTGGCGTGTCCGACAGCTTTACCAAGATGACCGGGATTGCCGTCCGTCACG
ACCTGACCGAGGACAACGAGATCCAGCCGAAGGTCTGGGCCGCCGTTGCCCAGAAGCGCGTCCCGCCGATCCA
CATCAACTGGGATACGACGACGAACGCCACCAAGTCCGCTTTGCGCGGCGTGACCGAAGACCTGTCCGACCTG
CCGAACCTCAAGAACGCCACGGATCTCGCAAAGCCGGTCGGCCTCGACGGCTATCCGATCGTCAACACCTACG
GTTATGTCTACGTGCTCGCCTATCGCCCTTCGGCCTTCCCGAACGGCGCGCCAAAATCCTGGAAGGATCTGCT
CGACCCCAAGCTCAAGGGCCGGATCGCATTGTATAACGACGGGATCGGCTTCCACTTCCCCGCACAAGTCGCC
GGCGGCGGTAAACTCGAGGACATTCCCGCCAACATGCAGCCGGCCTGGGATTTCATCTCCAAGATCAAGGAGC
AACAGCCGCTGCTCGGCGAGGACCCGGATTTTACCACCTGGTTCCAGAAGGGCGAGATCGACGCCGCCTGCAC
CATCTCGACCAATGCGCGCGAAGCCAAGAAGAACGGCATCGAGATCGCCTGGGTCGTGCCGGAGGAAGGCGCC
AAGTTCGACACCGACGGCCTTTGGATACCGAAGGGTCTGCCCGAGAACGAGCTTTACTGGGCCAAGCAATATA
TCAACCACGCCCTGACCAAGGAAGCGCAGCAGATCTGGCTGGACGGTCTCGGCTTGCCCGGCGTCATCCCCGG
CATCACGCCGCCCGCCGATCTCGTGGGCGATCCCTCCTATCCAACAACGGAGGAGGACTTCAAGCAGCTGATC
CGTATCTCGGCCAAGGTACAGGTCGAGAACGAGAGCCAGTGGTTCGCCAAGTTCAAGGAAATCATGCAGGGCT
GAATGCCGCTACATAGTAGGAAAAAGATGATCGGCCTGGCGCTGTGCCTGGGGGCGGTCGTCGTCGTCGTTGC
CGGGTGGACCTGGGCCCGCGAGAGCGGTGCGGCGTCGACCGACAACGCCTATGTCCGCGGGGATGTTACCTCG
CTTGCCCCAAAGGTCGCAGGCTACGTCACGGCTATCGAAGTCGAGGACAACCAAGCGGTACGGGCGGGCGACG
TGCTCTTCCGGATCGACGATCGGGACTACCGCGCAAAGCTTGCGCAAGCGGTGGCCAATGTCGAAGCGGCAGA
GGCACGGCTGACCAACGTCGATGCGGAGATGGCGCTTCAGCATGCCCTGATCCGCCAGGCCGAAGCGCAGCGA
CGTTCGGTCGTGGCCGAGTTGAACCTGGCGGCCAAGGCCTCTGACCGCCGCCGCGAGCTTATCCGCAGCAACA
CGATCAGCCAGGCCCATGTCGACGAAAGCGACGCGGCGAAATCGAGGGCCGAGGCGAACGTGTTGGCGGCTTC
CGCAACGGTGGAGGCGCAGCAGCAGCGCATCGCCGTGCTTGCCGCGCAGCGCGAAGCCGCCGTCGCTGCGGTG
GCACAGGCGGAGGCCGCGCGCGACCTCGCCGGGATTGATCTGGAGAGCACCGTGGTGCGCGCCCCCGTCGGCG
GCGTCATCGGCAACCGGCAGGTGCGCGTCGGCCGGCTCGTTGCGCCGGGCGCTTCCCTGCTCGATATCGTTCC
GCTCGACAATGTATGGATCGTCGCGAATGTCAAGGAAACCCAGCTCGAACATATCCGGCCCGGTCAGCGCGCC
AGCATCACCATCGATGGTTACCCGAGGGGAGCGCTTGAAGGCGTGGTGGACAGTTTTTCACCCGGCAGCGGCT
CTGCCTTCAGCCTGCTGCCCGCAGACAACGCCACGGGCAACTTCGTCCGCGTCGTCCAGCGCGTTCCGGTGAA
GATCCGGTTCGCCGGCAATCCGCTGTCGGGCCGCCTCCTGCCGGGCCTGTCAGCGCGTGTCGAGATCGATCTG
GAGAGCGGTTCATGAATGCTGCGGGAGGATGTGAAGGATCTGCTGTGGTTCATCGAGGTGGCGAGGGAGCAGA
GCTTCACCAAGGCCGCGGCCACGCTCGGCACCTCTCAGTCGACCCTCAGCCATACGATCAAGCAGTTGGAGGC
GCGGCTGGGAGTACGCCTGCTGACGCGGACGACCCGCAGCGTGGCGACAACGGAGGCTGGCGAGCGCCTGCTA
CAGTCGCTCGCTCCAAGATTTGAGGGGATCGAGGCAGATCTTGCAGATCTGGTGGCTTTCCGGGAGAAACCGT
CGGGCACAGTGCGCCTGACGCTCTCTGATCATGCTCTCCAGACGACGGTCTGGCCTAAGCTGAGCCGTGTCCT
GTCCGACAATCCCGACGTCAAGGTCGAGCTCTACAGCGACAACGGGATGCGCAACATCGTCGAGGAGCGTTTC
GACGCCGGCGTCCGGCTCGGCGAGAGCGTCGACAAGGACATGATCGCCGTGCGCATCGGCCCCGACTGGAGGC
TGCGGGCCGTCGCCTCCCCGGAATACCTCGCCAGGCACGGGATCCCCAAGACACCGCAGGACCTCGTCAGCCA
TGTCTGCATCAACACGCGACAGGCAACATGGGGCGGCTTCTATGCCTGGGAATTCGAGAAGGACGGGCGGGAG
CTTCGGGTCCGGGTCGACGGGCAGCTCAGCTTCAACTCCTCGATCTCCCAGATCGACGCCGCCGTTAAAGGAT
ACGGGATCGCCTATATACCGGAGGATCTCCTGGAAGGACATATCCAGGCGGGAGCGCTGCAGGCTGTCCTCGA
CGATTGGAGCGAGCCGTTCACGGGTTACCACCTTTACTATCCCAGTCGACGTCAGATGTCCCCCGCGATGGCA
CTGATCGTAGATGCTCTGAGACATCGGAGCTGAATGCCCAAGGAAGACTTCAACGATCTGCTGTGGTTTCTCG
CCGTGGCCGAGGAGCGCAGCTTCACCAGGGCCGCTGCCAAGCTCGGCATAACCCAATCGACGCTCAGTCACAC
GATCAAGCGCCTCGAAACGAGGATGGGCCTGAGGCTTCTGACCAGGACCACCCGGAGCGTTGCCGTGACGGAA
GCTGGCGAGCGCCTTCTTCGTTCGCTGGCGCCGCGGATATCGGCCATAAGGGACGAAATCGCGGCCCTTATGG
CCTTTCGCGACAAACCGTCCGGCTCGGTCAAAATCACTTTGTCCGACCACGCCCTGGAGAGCCTCGTCTGGCC
GAAACTACAGCCGGTTCTTCTGGAATACCCCGACATTCGGCTCGAGTTGAGCAGGGACAACGGCTTCAGGAAC
ATCGTGGAAGATGATTTCGACGCAGGCATTCGCCTCGGCGAGAGCGTCGAAAAGGACATGATCGCGGTCCGTA
TCGGACCGGACTGGCGGCTTCTGGCAGTGGCTTCGCCCGACTACCTGGCAGATCGTCCGCTGCCAGAGCATCC
ACAGGAACTGATCCAGCACAATTGCATCAACTCGCGCCAGGCAACAGGCGGCGGACATTATGTATGGGAGTTC
GAGAAGAACGGGCAAGAGCTCCGCGTCAGGGTCGACGGACAACTCACCTTCAATAGCTCATACCCCATGGTCG
ATGCCGCCCTGAAAGGTCACGGCATAGCCTATCTGCCGGAAGATCTCGTCGCGGGGCACATTGCGGCAGGACG

CCTGGTAGCGCTGCTCGAGCCCTGGTCGCCTCCATTTGCCGGGTACTACATTTACTACCCTAGCCGACACCAC
AACTCACCCGCATTCAAGGTGATCGTGGACGCCCTGCGACACCACGGCCCACCAGATTCCGAGGGAACGCGAG
ATGCTTAAATGGGAAACAAATCTGTTCGCGTCATCGCGGGAGCAATGGTGGTCGCCGGCTGGGCCGGCTACGC
CGCTGCGCAAAGTGCCTCGGAAATCAAGATCGTCCTGCCGGAGCAGCCGGCCAATCTCGAGCCCTGCGGTACC
ATCATCACCAATGTCGGCCAGATATTGAGCCGCAACGTGGTCGAGCCGCTGACCATCATCGATCCGAAAAGCG
GCCAGCCGACACCCGGTCTTGCGACCGAGTGGAAGCAGACCGATCCGAACACCTGGCAGCTCAAGCTGCGCGA
AGGCGTCAAGTTCCAGGACGGAGCCGCCTTCGACGCAGAGGCGGTCAAATTCTCCATCGAGCGGATGACCGGC
GGCAAGCTGACCTGCAGCAACATTGCCAAGTTCGGCGATGCCAAGCTGACGGTCACGCCGCTCGATGAGCTGA
CGGTCGAGATCAAGTCGGACAAGCCGCAGCCGATCCTGCCGACGCTGCTCAGCGTGGTCATGATCGTCTCGCC
GAACACGCCGGCGGACAAGGCCGTCAACGACCCGGTCGGTACCGGGCCGTTCAAGCTTTCGAGCTTCACGCCG
CAGACAGTCGTGCTGGAAGCCTTCGACGGCTACTGGGGCGAGAAGCCGGCCATCGCCAAGGCCAGCTATGTCT
GGCGCCCGGAATCCTCGATCCGCGCCGCCATGGTGGAAACCGGCGAGGCCGACCTGACGCCGTCGATCGCGAT
CCAGGATGCGACCAATCCGGAAACGGACTTCGCCTATCTGAACTCCGAAACTACCGCGATCCGCATCGATGCC
GGTTTTGCTCCGCTCGACGACGTCCGAATCCGCAAGGCGCTGAATCTGGCGATCGACTGGGATGGCCTCGCGC
AGCTCTTCGGCGAGGACGTGCAGCGGGCTTCGCAGATGGTCGTCACCGGCATCAACGGCCACGACGACAAGCT
GGCGCCCTGGACGTTCGATGCCGAAAAGGCGCGTGCGCTCATCGCCGAGGCCAAGGCCGCGGGCGTTCCGGTC
GATACCGAAATCGAGCTGATCGGCCGCAACGGCATCTACCCCAACGGTACGGAAGCCATGGAAGCCATGATGG
CCATGTGGCAGGATGTCGGCCTGAACGTGAAGCTGACGATGCTCGACGTGAATGATTGGCTGCGCTACCTGCA
GAAGCCCTTCCCGGAGAGCCGCGGGCCGAACCTGCTGCAGATGATGCATGACAACAACAAGGGTGATGCCGCC
TTCACCATTCCGATCTTCTACACCTCGGCCGGAAGCTATTCGACCTTCAGCGACGCGGCACTCGACAAGGAGG
TCGCCGATGCCATGGCTGCCACCGGTGAGGACCGTACGGCAAAGTTCAAGGCGATCTTCGCGAAGGTGCATGA
CGATCTCGCGGTCGACATCCCGATGTTCCACATGATCGGCTACACCCGGGTCGGCAGCCGCCTGGAGTGGAAG
CCGGACATCACGACCAACAGCGAGATCCCGCTCGCCAATATCGCTCTCAAGGACTGAATGTCGACATTCATCA
AGAACTTCATCGAGCGTGAGACGATCAGCCGTCGCCACTTTCTGCTTGCCTCGGCCGCAGGTCTGGGGGCAGC
GGTCCTGCCAAACCCGCTTGGCGGCAGCGCGCGCGCTGCATTGACGGATCCCGCGATCGCCTGGAGCTATCGC
GACCGCGCTTCGGCCTATTGGAACTCGGTCGTCTCCGGCGGCGAAGCATTCGTCGAATCGCTCGGCAAGCCGA
AGAGCGCGCTCGTAAGTCTGATCAACGAAGGATCATCCGAGAAATCTCTGGCCGACATCAAGGCCTTCCTTGC
CAAGAACAATGGAAATTGCGCCCTGGCCTGCGATGCCAATGACAGCCCGAATGCGCGGCCGATCGTGGAGGCG
GTGGCGGCTGCGGGCGGCTACATCTCCACGATCTGGAACAAGACCGATGATCTCCATCCATGGGATTTCGGCG
ACAACTACGTGTCGCACATGACCTGGTCGGACGAGAAGCCCGCCGAAGAAACCGCGCGCATCCTGTTCGAAGC
CATGGGCGGCGAGGGCGGTGTCGTCCACCTGGGCGGCATTGCCGCCAACAACCCTGCGGTCGAGCGGCTCAAC
GGTCTGAAGAATGCGCTGAAGGACTTTCCCAACATCGAACTGCTCGACGCGCAGCCGGCGGACTGGGACACGC
AGAAGGGCGCGGCTTTGATGTCCTCCTTCCTGACCCGCTACGGCGACAGGATCAAGGGCGTGCATTGCGCCAA
CGACAACATCGCCTACGGCGTCATCGAGGCGCTGCGCGCCGAAGGCATCGAGGGCATGCCGATCGTGTCCTAT
GACGGCAATCCCGAAGCGGTGCAGATGGTCATGGACGGCCAGCTGCTCGCGACGGTCTTCACCAATCCGCACT
GGGGCGGTGGCATCACGTCGGCGCTCGCCTACCATGCGGCAACCGGCAGCTTCAAGCCGTCGGAAGAGCCGAA
AGAACATCGCGAGTTCTACGGACCGACCGTCCTGGTCAGCAAGAAGGATGCCGAGGAGTTCAAGGCCAAATAT
CTGGATTCGGTACCGAAATACGATTGGGCCGACTTCTGGGGTCCCTCGAACGGACAAATTCAGTATCGGTCCT
GAATGAAGCGCATCTACGTCGTTGGCACGGCCGACACGAAGGGGGAGGAACTCGTTTATCTTGCGTCCTGCGT
TGAGGCAGCGGGCGGCCGGCCCGTTTTGGTCGATGTCGGCACGCGTCGCCCCACGGTTCTGGTCGACATCTCC
GCCGAAACCGTGGCCGCGGTGCATCCGGGCGGCGCCGCCGCGGTGCTTTCCGGCAACGATCGGGGAACAGCGA
TCGCTGCCATGGGCGAGGCCTTCGCAAGGTTCCTGCCCGCCCGGGACGACGTCGCCGGCGTGGTGGGCATGGG
TGGCGGCGGCGGGACGTCGATTATCACCGCCGGCATGCGCAGGCTGCCGCTCGGGCTACCGAAGGTCATGGTC
TCGACGCTCGCGTCGGGCGACGTGGGACCTTATGTTGACGTCTCAGACATCATCATGATGCCGTCGGTTACCG
ACATGGCCGGGCTCAACCGCGTGAGCCGCGTGATACTAAAAAACGCGGCCGAGGCGATTACCGCCATGGCGAA
CAGGCCGGCGGAGGAAACAGCCTCGAAGCCCGCTATCGGCCTGACCATGTTTGGCGTGACGACGCCCTGTGTG
ACTGCCATCGTTGAACGACTGAAGGCCGACCATGACTGTTTGGTTTTCCACGCCACAGGCACCGGCGGACGAG
CAATGGAGAAGCTTGCAGACAGCGGGCTTCTATCGGGTGTGCTCGATATCACGACAACGGAGGTGTGCGATCT
TGTCTTCGGCGGCGTGCTGCCGGCCACCGAGGATCGTTTCGGCGCAATTGCGCGCACCGATTTGCCTTATGTC
GGCTCCGTCGGGGCTTTGGATATGGTCAATTTCTGGGCACCAGAGACAGTACCGGAGCGATATTCCGGCCGGC
TCTTGTATCGCCACAACCCCAACGTCACGCTCATGCGCACGACTCCGGAGGAATGCGCCGCGATCGGAAGGTG
GATTGGCGCAAAGCTGAACCTCTGCAGCGGACCTCTCCGGTTCCTGATCCCGGAGCGAGGCGTGTCGGCGCTG
GATATCGAGGGCGGCGCCTTCTTCGATCCTGCGGCGGATGCGGCGCTCTTCGAAGCATTGGAGACCACCGTCA
ATAGAAGTGACCGGCGCCGCATCGAACGCCTTCCGCTCCACATCAACGATCCGCAGTTCGCCGAGGCCGCAGT
CGCCGCCTATCGAGACATTGCCAACCCCTGAATGAAATATCAAACCCAGAAGGTCGCGATGCTGTATTTCTAC
GGTGCGCTCGGCCTGTTTCTCGCCCAGATCCTCTTTGGCGTGCTTGCCGGCACCATCTACGTGCTGCCCAACA
CGCTCTCCGAACTGCTGCCGTTCAACATCGTCCGCATGGTGCACACCAACGCGCTGATCGTCTGGCTGCTGAT
GGGTTTCATGGGTGCCACCTATTACCTCTTGCCGGAAGAGGCGGAGACGGAGCTCTACAGCCCGAAGATCGCC

ATCGCCCAGTTTTGGATCTTCCTGATCGCGGCGGCCGTTGCCGTCGTCGGCTACCTCCTCCACATCCACGAAG
GCCGGGAATTCCTCGAGCAGCCTTTCGCGATCAAGGTCGGCATCGTCGTCGTCGCGCTGATGTTTCTCTTCAA
CATCACCCTGACCGTACTGAAGGGCCGCAAGACAACGGTGACCAACATCCTGATCTTCGGCCTTTGGGGCGTG
GCGATCTTCTTCCTGTTCGCCTTCTACAACCCGGTCAATCTCGCGCTCGACAAGATGTACTGGTGGTATGTCG
TGCATCTCTGGGTCGAAGGGGTCTGGGAGCTGATCATGGCTTCCGTCCTGGCCTTCTTGATGATCAAGCTCAA
TGGCATCGACCGCGAGGTCGTCGAAAAGTGGCTCTATGTCATCGTCGGCCTGGCGCTCTTCTCAGGCATCCTC
GGCACCGGCCACCACTACTACTGGATCGGCGCGCCCGGCTACTGGCAATGGATCGGCTCGCTCTTCTCCACGC
TCGAGGTCGCACCCTTCTTCACCATGGTGGTCTTCACCTTCGTCATGACCTGGAAGGCCGGCCGCAAGCATCC
GAACAAGGCCGCGCTCCTCTGGTCGATCGGCTGCTCGGTGATGGCCTTCTTCGGCGCCGGCGTCTGGGGCTTC
CTGCATACGCTTTCGTCGGTCAACTACTACACCCATGGCACCCAGGTCACCGCCGCGCACGGGCATCTCGCCT
TCTTCGGCGCATACGTCATGCTGAATCTGGCTGTCATGGCCTATGCGATACCGGAGATTCGGGGGCGAGCGCC
CTATAACCAGTGGCTGTCGATCGCCAGCTTCTGGGCCATGTGCAGCGCCATGTCCGTCATGACCTTCGCGCTC
ACCTTCGCCGGTGTCGTTCAGGTCCACCTGCAGCGCGTCCTCGGCGAGAGCTTCATGGCCGTGCAGGAGCAAC
TGGCTCTCTTCTACTGGATCCGCCTCGGGTCCGGTGTCGTCGTACTCATCTCGGCGCTGATGTTCTTCTGGGC
AGTCCTCGTACCCGGCAAGAGCGCTAGCCCGGCCCTCAACCAAGCCGTCCAACCCGCCGAATGAATGACCGGC
GAACTGACGCGGCGTGAAATGTTGAAGGCGCATGCGGCCGGCATCGCCGCAGCTACGGCCGGGATCGCGCTTC
CGGCGGCGGCGCAGCCCGTGCCGGGCGGCGTCGAAGCACTTCAGATAACGTGGTCGAAGGCGCCCTGCCGTTT
CTGCGGTACCGGCTGCGGCGTCATGGTCGGGGTCAAGGAGGGACAGGTCGTCGCCACACATGGCGACATGCAG
GCTGAGGTGAACCGCGGCCTCAACTGCATCAAGGGCTACTTCCTGTCCAAAATCATGTACGGCACCGACCGTC
TGAAGACGCCACTTCTGCGCAAGCGCAACGGCGCCTTTGCCAAGGACGGCGAGTTCGAGCCGGTGAGCTGGGA
CGAGGCCTTCGACGTCATGGCGGAGCAGGCGAAGAAGGTGCTGAAGGACAAGGGCCCGACCGCCGTCGGCATG
TTCGGCTCTGGGCAGTGGACCATCTTCGAGGGTTACGCGGCCACGAAGCTTATGCGCGCCGGGTTCCGCTCCA
ACAATCTCGATCCCAATGCCCGCCACTGCATGGCCTCGGCAGCCTATGCTTTCATGCGCACCTTTGGCATGGA
CGAGCCGATGGGCTGCTACGACGACTTCGAGCACGCGGACGCGTTCGTGCTTTGGGGCTCGAACATGGCGGAG
ATGCACCCGATCCTGTGGACGCGCCTCGCCGACCGTCGGCTCGGGCACGAGCATGTGAAGGTCGCGGTGCTGT
CGACCTTCACCCACCGCAGCATGGATCTTGCGGATATTCCGATCGTCTTCAAACCGGGGACCGATCTGGCGAT
CCTCAACTACATCGCCAATCACATTATTCAGACCGGCCGAGTCAATGAGGATTTCGTCAGGAAGCATACGACC
TTCATGGTCGGCGCGACCGACATTGGCTATGGCCTGAGGCCCGACAATCCGCTCGAGGTCAAGGCCGTGAACG
CCAAGGATGCGGCGAAAATGACGCCGAGTGACTTCGAGAGCTTCAAATCCTTCGTCTCGGAGTACACGCTCGA
CAAGGTGGTCGAACTCACGGGGGTCGAAGCCGGGTTCCTAGAACAATTGGCCGACCTTTATGCCGATCCGAAG
CGCAAGGTCATGTCGCTCTGGACTATGGGGTTCAACCAGCATGTGCGCGGTGTTTGGGTCAACCAGATGGTGT
ACAATCTCCATCTCCTGACCGGGAAGATCTCCGAGCCCGGCAACAGCCCGTTCTCGCTAACCGGTCAACCTTC
CGCCTGCGGCACGGCACGCGAGGTAGGCACCTTCGCGCACCGCCTGCCAGCCGACATGACCGTGACCAATCCG
GAGCACCGCAAGCATGCCGAGGAGATCTGGCGCATTCCCCACGGCATCATTCCTGAGAAGCCCGGATACCATG
CCGTCGAGCAGGACCGAATGCTGAAGGACGGCAAGCTCAATTTCTATTGGGTGCAGGTCAACAACAACGTCCA
AGCGGCTCCCAATACGCAGAACGAGACTTATCAGGGCTACCGCAATCCGGACAATTTTATCGTCGTCTCCGAC
GTCTATCCGACGATCACGGCGATGAGCGCCGACCTCATCCTGCCGGCTGCCATGTGGGTGGAGAAGGAGGGCG
CCTACGGCAATGCCGAGCGTCGTACCCATGTCTGGCACCAGCTCGTCGATGCGCCCGGCGAGGCGCGTTCCGA
TCTCTGGCAGATGGTGGAGTTTTCCAAGCGCTTCACCACCGACGAGGTCTGGTCGACCGACATCCTCGATGCC
AATCCCGGCTATCGTGGCAAGACCCTTTACGACGTGCTCTTCAAGAACGGCAATGTCGACAGCTTCCCGGCAA
GCGAGATCAACAAGGAATACGCCAACCGGGAGGCCGAGGCCTTCGGCTTTTACATCCAGAAAGGCCTGTTCGA
GGAATATGCCTCCTTTGGGCGTGGACACGGGCATGACCTGGCGCCCTATGACCGCTATCACGACGAACGGGGA
CTCCGCTGGCCTGTCGTCGACGGCAAGGAAACGCTCTGGCGCTACCGGGAAGGCTACGATCCCTACGTGAAGC
CGGGGGAGGGCGTCAAGTTCTATGGTCGGCCGGACGGCAAGGCGGTGATCCTCGCCGTTCCTTACGAGCCGCC
GGCGGAATCTCCCGACGACGAATACAATGTGTGGCTGGTGACGGGCCGCGTCCTTGAGCACTGGCACTCCGGC
TCGATGACGATGCGGGTGCCGGAACTCTACAAGGCGTTCCCCGGTGCCGTCTGCTTTATGAATGCCGGTGACG
CCCGCGATCGCGGCATCAACCAGGGGGCCGAGGTCAGGATCGTTTCCCGCCGCGGCGAGATTCGCGCCCGCGT
CGAGACGCGCGGCCGCAACCGCATGCCGCCGGGCGTCATCTTCGTACCCTGGTTCGATGCCAGCAGGCTGATC
AACAAGGTGACACTCGACGCAACCGATCCCATCTCCAAACAGACGGATTTCAAAAAATGCGCGGTCAAAATCG
TCTCTGTCGCATGAATGCTCCACCAACCCAAAACCAAAGCTACAGTCGGACGGCTCGATGCCGAAACCGTCAA
TGCCGCCCGCGTGCGAGGTCCGCTTTACGAGAAGCGTCGGAAGATCTTCCCGAAGCGGGCCGAGGGTCGTTTC
CGCCGGTTCAAATGGCTGGTGATGCTGGTGACGCTCGGCATCTACTATCTGACGCCGTGGATCCGCTGGGACC
GCGGGGCGCATGCGCCGGATCAGGCAGTCCTTATCGACCTTGCTTCCCGGCGCTTCTATTTCTTCTTCATAGA
GATTTGGCCGCAGGAGTTCTTCTTCGTCGCGGGGCTCCTGGTGATGGCGGGCTTCGGGCTTTTCCTCGTGACG
TCGGCAGTTGGGCGGGCCTGGTGCGGCTACGCCTGCCCGCAGACCGTCTGGGTCGATCTCTTCCTCGTCGTTG
AGCGCTTCATCGAGGGCGACCGCAACGCCCGCATGCGCCTCGACGCCGGACCGTGGAGCCTCGACAAGATACG
CAAGCGCGTGGCCAAGCATGCCATTTGGGTGGCGATCGGCGTTGCAACCGGCGGCGCGTGGATATTCTATTTC
GCCGATGCGCCCTCGCTCATGTCGAGCCTAGTCGCGCTCGATGCGCCGCCGGTCGCCTATACCACGATCGGCA

TTCTGACCGCGACCACCTACGTCTTCGGCGGACTGATGCGAGAGCAGGTCTGCACCTATATGTGCCCATGGCC
GCGCATCCAGGCGGCCATGCTAGACGAGAACTCGCTCGTCGTAACCTACAACGACTGGCGGGGCGAGCCGCGC
TCGCGGCATGCGAAGAAGGCGGCTGCGGCCGGCGAGGTGGTCGGGGATTGCGTCGACTGCAACGCCTGCGTTG
CCGTCTGTCCCATGGGCATCGACATCCGCGACGGCCAGCAGCTCGAATGCATCACCTGCGCGCTCTGCATCGA
CGCCTGCGACGGTGTGATGGACAAGCTCGGCCGCGAGCGCGGGCTGATCTCATACGCGACGCTCAGCGACTAT
GCAGCCAATATGGCCCTCGCGACGAGTGGCGGGACTGCGGCGATCGATCCGAGCCGCGTGCGCGACGCCCATG
GCGCTTTCCGTGACAAGGTGAGGCATCTCAACTGGCGCATCGTCTTCCGGCCCCGCGTTCTCGTTTATTTCGG
CGTCTGGGCGACGGTCGGATTCGGTCTTCTTTTCGGCCTGCTGGCGCGCGACCGGCTAGAACTGAACGTCCTG
CACGACCGCAACCCCCAGTTCGTCGTCGAATCCGACGGCTCGGTGCGCAACGGCTACATGGTCAAGCTTCTCA
ACATGATCCCAGAACAGCGCACCATTAGCCTGACGATCGAGGGTATGCCCGCCGCAACCATGCGCGTGGCCGG
ACAGGCGACGGGCGACGGACGCCGGGTCACGATCGGTGTCGAGCCGGACAAGGTCACACCGCTCAAAGTCTTC
GTCACGCTGCCGAAGGGCAGATTTGCCGAGGCGGAAGAGGGCTTCTCCCTCATTGCGGAGGATCCGTCCAGCC
ACGAACGCGATGTGTATCAGGCCAATTTCAATCTACCGGGAGCTGCAGGACGATGAATGGCTCGATTGACCAT
AAACGGCCGAACAAGGGATATCGAAGTGGATCCGGAGACGCCACTTCTCTGGGTGCTACGTGAACAGGCGGGC
CTCACCGGCACGAAATTCGGCTGCGGGGTTGCGCAATGTGGCGCCTGCACCGTGCTTATCGACGGCGTGGCGA
CGCGTTCCTGCACCATGCCGGCAAGCGCGATCGCGCAGGATCAACAGATCGTCACGATCGAGGGCCTGTCCGA
GGACGGCTCTCATCCGGTCCAGCAAGCCTGGCTTGCGCTCGACGTTCCGCAATGCGGCTACTGTCAGGCCGGC
ATGATCATGGCGGCTGCCGCCCTGCTCAACAGCACACCAACCCCAAGCGACGACGATATCAGCGCGGAAATCA
CCAATATCTGCCGCTGTGGCACCTATAACCGCGTCCGTGCGGCGATCAAGCTCGCGGCGGAAACCATGAGCAC
GTCCCAGAAAGGATGAATGCAATCGACGCCTCACATTGCCGTTGTGGATGATCATCGCGATATTCGCGACCTG
GTAGGAAAATATCTGACACAACAGGGTTACCGCGTTTCCCTCGCGGAAAGCGGCGAAGCGCTTCGACGTCTTC
TGGAAAAAGGCGCCCCAGATCTCGTCGTGCTGGACATCATGATGCCCGGCGAGGATGGATTGACCGTTTGCCG
TCAGTTGCGCACGACAACGGAGCTTCCGGTCATCTTTCTGACAGCGATGGCGGACGGGACGGACCGGATCGTC
GGCCTGGAACTCGGCGCCGACGATTACATCACCAAGCCGTTCAACCCGCGCGAACTCCTGGCAAGAATCCGGG
CGGTGCTGCGGCGTGTCAACAGCCTTCCTCCCCGGCGCGGGAGGATCAAGGCCAAGACCATCCGCTTCGATAT
CTGGGAACTCGACACTGGACGCCGGGAGCTCGTCGGACGCGATGGCGTCAGCGTGGCGTTGAGCACGGCGGAG
TTCCGGCTGCTCAATGCCTTCCTGGAACACCCCGGTATGGTCTTGAGCCGTGACCAGCTACTTGATCTGACCG
CAGGGCGAGCCGCCGAGAGCTTCGATCGCAGCATTGACAACCAGGTCAGTCGGTTGCGCAAGAAGATCGAGAG
CGACCCGAAAAATCCATCTTTGATCAAGACGCACTGGGGTGGAGGATACAGCTTCGTGGCGGAGGTAGAAGCC
TACTGAATGGCCACCATCACCCATGTCTTCACCATCAACCATGTCGCCGAAATGCTCGGCGAAGACCGGGAAC
TCCTGGAGGCGATCGTCAGCAATTCCGACAATTTGACCTATGGAAGTATCATCAGCGTCGTACACGGTGACGA
TGAGACAATCACCGCACTGACCGACGATGGCATGGATGAGCTGAGGCAAATGCTTGCCGATGCGCGCCGTTCG
CCTGAAGCCTGGAACGACTTCCTCGACTGCTTCGTCGACGACGAAGAAGTCATCGCTCGCGTCAAGGCGAAAT
CTCCGCGGTAAATGACAGAAACGGCCGCTTCGTCAATAGATAAGCAAGCTCATCTGTTCACCATATCTAATGC
GAGCGGCGGTGAGTACGTTGAGGGTTGGCGCGTCACAGTTCTGCTTGTTACGCTATTTCTTGTCAGCGCCACT
TCCCAGATCGACCGGACGCTTCCCTTCGTCCTCGCCGAGTCGATAAAGGAAGAGCTCGATCTGAGCGATACCG
AAATGGGTTTGCTCACCGGCATCGCCTTTGCGGTATGTTACACGCTGCTGTCGATGCCGCTCGCCCGCTCCGC
TGATCGAGGATCGCCCCGGTTCGTCCTCGTTTTGTGTATCCTATTCTGGAGCGCGATGACATTGCTCGGCGGT
CTCGCCATGAGTTTCCTGTTCCTCGCCTTCACCCGCTTCGGCGTCGCGTTCGGCGAGGCCGGTGGGACGCCCT
CCAGCCACGCGATCATCGCACGCAAGATCCCGCCCGAGCGCCGCGGGCTGGCGCTCGGCATTTTTTCCATGGG
AATTCCACTCGGCACCATGGTGGGTTTCGCTGTTGGCGGCGCTATCGGCGATACGCTCGGCTGGCGCACAGCG
CTGATAGGCGCAGGCGCCATTGGCGTGCTGATCGGTCTGCTGGCGTTCGTCGTCGTCGGTCCGACCCCACCAC
GCAAGAGCTCGGCCGCCAGTAGCGAACCTTTCCTGCGGTCGAGCTTGCAACTGCTTTCGTCCCCGTCATTTCG
CTGGCTGTTCATCGGCTCGGTCGCACTCGGCTTCGCCGCAGCGCCGTTCTACGCTTTTGCAGTTCCCTTCCTA
ATTCGCACTCATGACTACAGCGCCAGCGAGGCAGGACTTGCGTTCGGGCTGCTGCAAGGCCTGATGGGGATTT
GCGGTACGCTGATCGGCGGACGCCTGTTCGACCGCGCCGTGCGCTCCGGCACCGGCCGGGTCCTCGGACCGCC
CGCTATCCTCTTCATGATCGCAAGCCTGACGACGACAGCTGCGCTGTTCGCGCCGGTCGGGTGGCTTTCCATC
CTACTCTTCGTGCCCGGCATGCTGTCCTTCGCATTTCTGCTGCCCTGGTGTTTCGGCGCCGCGCATCTCGTGG
CGGGCAAGGGCAAGGAAGCGATGGCGACGAGCCTTGCGATGATCGGCTCCGGGCTCCTGGGGCCCGCTCTCGG
TCCTCTCATCGTCGGCATGATCAGCGACGCGGCGACCGCGGCACAGGTCTCCAATGGCTTGGGGCTCGGTTTG
GTGACGGTCCCGATCGCAAGCATCCTGACCGGGATCGCCATGCTGATTGCAAACCAGCGCATTGCTGCGGTCC
TTCGAAAACACGACGTCTCTTAAATGACCAAGTGGACGAGCAAGAACATTCCATCGCAGCATGGCCGTACAGC
GGTTGTCACCGGCACCGGAGGATTGGGCTACGAGGGCGCGCTGGCCTTGGCCCGAGCCGGTGCAAGCGTCGTG
ATCGCCGGGCGCAATCCCCAGAAGGGGGCTGAAGCGGTGGCGGCGATCAGACAAGCCGTGCCGGGCGGACAAG
TGCGGTTTGGCAAGTTGGATCTCGCCAGCCTCGCGTCGATCGCTGAGTTCGCCGCGCAGCTTGCACGGGACCA
GGAGGGTCTCGATTTCCTGATCAACAATGCGGGCGTCATGACGCCGCCGGAGCGGCGCCAGACGACCGACGGC
TTCGAATTGCAGTTCGGCACCAACTATCTCGGCCATTTCGCCCTGACCGGACATTTGCTGCCATTGCTGAAGA
AGGGGCGAAATGCGCGCGTCGTCACCCTCGGCAGCGTGGCGGCGAGGAGTGGAGCGATCAATTTCGATGACCT

TCAAGCTCAGCGCGGCTACAAGCCGATGCCGGTTTACAGCCAATCGAAGCTCGCCTGCGTCATGTTCGCGTTC
GAATTGAGCCGGCGCAGCAAGGCGGCCGGGTGGGGCGTCGAGAGCATAGCCGCGCATCCCGGTATCTCGCGCA
CCGATCTGCTTCCGAACGGCGCGGGGCAGACGAGTGCTGCCGGGATGGCACGCCGCTTCTTACCGTTTCTGTT
CCAGCCCGCGTGGCAGGGTGCGCTGCCGACGCTGTATGCGGCGACCGATCCGGCGGCGCGCGACGGCGCCTAC
TATGGACCTGACAGGCTGGGCGGACGCGTGGCTATCCGACCGAGGAGCAGCCTCCCAAACAGGCGCTCGATA
CTTCAGTGGCCGCGCGCCTCTGGGACGCGTCTCAGGAACTGACGAGCGTGACGTTCGTGTGAATGCCTAAGAT
GAAAGCCGCGATCTTCGTCGCGCCTGGTCGTATTGTACTTGACGAGAAGCAGATCCCCGACGTCGGTCCGCTC
GACGCGCTCGTGCGGATCACGACCACCACAATATGCGGCACCGACATCCACATCTTAAGGGGTGAGTATCCGG
TCGCACGCGGCCTGACGATAGGGCACGAGCCGGTGGGCGTGATTGAGAAGCTCGGGTCCGCCGTGCGGGGATA
CAGCGAGGGTCAGCGTGTGATCGCCGGGGCGATCTGCACCTCGGGCCACAGTAATGCGGCCCTGTGCGGCTGC
CATGCCCAGGACGGCCCCGGGACGAACCACGGTTGGAAAGGCATGGGCGGCTGGAAGTTTGGCAACACCATTG
ACGGTTCGCAGGCCGAGTACGTCCTCGTGCCTGACGCCATGGCCAACCTGGCTCCAGTGCCGGACGGACTAGC
CGACGAGCAGGTGCTGATGTGCCCCGATATCATGTCCACGGGCTTCTCCGGGGCGGAGAGCGGTGCCGTCCGC
TTTGGCGACGCCGTGGCGGTGTTCGCGCAGGGGCCCATTGGACTGTGTGCGACCGCGGGTGCGCGTCTCATCG
GCGCAACGACGATTATCGCGGTCGAGAGCGTCCCCGCCCGCATGGAGATGGCCCGCCGCATGGGCGCCGACCA
TGTCGTGGATTTCACGGTATCGGACCCCACTGCAGAAATCATGCGTCTGACCGACGGGCGCGGGGTAGATGTG
GCCATTGAGGCGCTCGGAAGACAGGAGACCTTCGAAGGTGCTCTTCGCGTCCTTAGGCCCGGAGGTACGCTGT
CGTCCCTTGGCGTCTATTCAGGTGATCTAAGAATTCCGCTCGACGGCTTCCTCGCAGGTTTGGGAGACCATAC
TATTCGGACCACCCTCTGCCCGGGCGGCAAGGAACGCATGCGTCGTTTGATGGAGGTGATCGCATCCGGCCGC
GTCGACACGCGTCCTCTGGTGACTCACCGCTTCAAGCTGGACCAAATCGAGGAGGCATACGACCTTTTTGCAA
ACCAGCGTGACGGGGTGCTCAAGGTCGCGATCAATCCATGAATGGATGACGACCTCTCTACTGGGCTTGGCCA
GAAGGAGGCGGAGGTCAGGCTCACGCAATTTGGGCCCAATATGCTTCCCGAACCACAGGCGTCCTCCCTATTC
GCAACATTCTTGCGCCAGTTTCGCAGTCCGCTGATCTATATTCTACTGGCCGCCACGCTTGTGTCGCTCGCGC
TCGGCGATGTGCGCGACGCTCTGTTCATCGCAATCGTCCTCGTCGCGAACGGCACTATAGGCTGCATGCAGGA
ACACTCGGCCGGCAAGGCGGCCCTCGCGCTGCGCAAGCTGGAGCAGCCTAAGGCCACCGTGGTCCGCGACGGA
CACGTGCAGGAGATCGACGCCCGCCTCCTGGTACCGGGTGATCTGGTCCTGATTGAAGCCGGCGGACGCGTGC
CGGCGGATCTCCGGCTTCTCTCAGCCACCGATCTTGTCTGCGACGAATCCCTGCTGACCGGAGAATCGGCACC
GGTCCATAAAACCCTAACTAGTGTTGACGCCACTCCCGAGGTGAATGCCCGCCTCATGGCCTTTGCGGGAACG
CTCGTAACGCGCGGCCGCGGACGGGGGTCAATTGCAGCGACGGGTGCGGCTACCGAGATTGGAAAAATCGCGG
CCGAGATCGGCAAGGCATCCGTCTCCAAGCCCCCCTTGATGATCAGAATGGAACGCTTTTCGCAGTCCATCGC
CTGGGTCGTCGCTGCTGCCGTAGTGCTTCTCATTCTCGTCGGGATCGCTCGGTCGATGTCGCCGAGCGATCTC
TTCATGATGTCGGTTGGCCTGGCGGTGAGCGCGATCCCGGAAGGGCTGCCGATAGCGATCTCGGTCGCGCTGG
CAATCAGCATGCGGCGCATGGCGAAAGCACATGTCATCGTGCGGCGGATGCCGGCCGTGGAAGCGCTTGGCTC
CTGCACCATGATCGCCACCGACAAGACGGGCACCTTGACGCTCAACGAACTGACCGTAACGGATATTCGCTTG
CCGGACGGCAATGAAATCGTCTGCGATACCGGTTCTGATCTGGACTCCTGCACGATCCGTGGCGATGGAACTC
CTCCCGCTGAGGCGCGGGAACGGGCCCTTGCCCTGTTAAAGGCCGCTTCGCTGCCAAATGAAGGATTGCTTAC
CCGAAAAGACAACGGCTGGACCGCCGTAGGGGATACGGTCGACATTGCGCTGCTGGCAGCGGCCTACAAGGGA
GGTCTTCGGCGTGAGGCAATCGAGGATGACTATCCTCTCGTCGCCCGCATTCCTTACGAGCCGGACCTGAAAT
ACGCCGCCTCATTTCACCGCCACGGCGACAGCATCCGCATCTTTGTAAAAGGTGCCGCAGAAACCCTGATCGA
CATGGCCGACAGGATGGACATGGATGGTCGCGCCGAGCCGATCGATCGGGAAGCCCTTCTTCGCCAAAAGGAA
GAGATGGCCGCGCGTGGCTTGCGCGTACTTGCCTTTGCGGAGGGCGAAACGGCGGTCGAGTCGGATGGAGGCT
TCGGTCGGCACCTGCTCGTCGATCTCGTCTTCCTGGGGCTCGCCGGAATGCAGGATCCCGTGCGGCCGGAGGT
CCCGCAGGCAATTCGCGATTGCCACTCAGCAGGGTTGGACGTGGCAATGGTCACTGGCGACGATCCCAAGACA
GCAGCGGCGATCGCCTCCCAGGCCGGGCTAATCTTCACCGAAGATCAGGTAGTCACCGGCGAAGCTGTGCGCA
GAGCAGAGGAGAACGGACAGGAAAGCCTCGACACACTGACCCGCCATGGCCGCATCTATGCACGTGTGGCTCC
TTCGCAAAAACTGGCACTCGTGCTCTCGCTCGCACGCAACGGCCACTTCGTCGCTGTCACGGGCGACGGCGTC
AACGATGCGCCGGCGCTGAAGCACGCGCATATCGGCGTTGCGATGGGACGAAAGGGGACGGAGGTGGCGAAGG
AAAGCGCCGATATCATCATCACCGATGATAACTTCGCTTCGATCGTCAGCGGAATCCGCGAGGGCCGGGTCGC
CTATGCGAATATCCGCAAGGTCATTTTCATGCTGATGTCGACAGGCGCCGCAGAACTGCTGCTCTTTCTGCTG
GCAATTCCACTCGGCTTGCCGATGCCCCTGCTACCCGTGCAATTGCTTTGGCTCAACCTTGTAACCAATGGCA
TTCAGGACATTGCCCTTGCCGGTGAAAGTCCAGAAGGCGACGAACTTAGCCGCGCGCCACGCAGGCCGTCCGA
ACCGATCTTCGATCGGCTGATGATCCGGCGCATATGGCAGTCCACGCTCGTCATGGGAGCCGGCGGTTTCGCG
ATGTTCTATGTTCTGCTCGAGCAGGGTTACGGCGAGAGCGAAGCCCGCAATCTGCTGCTGTTGTTGTTCGTGC
TTTTCGAGAACTTCCAGACGCTCGCCAGCCGCTCCGAGCGCAAGTCCGTCTTTCAGCTTGGCTTTCTTGCCAA
CCCGCTTCTCCTTCTCTCAATCGTAGCGGCCCAGGGCCTTCATATCGCTGCCATGTACACTCCGATCCTCAGT
GAGACGCTGCAAGTTTCACCGATCTCGTTCTCAGAGTGGGCGCTCCTGCTTGTAGCGGCATCGTCTGCCCTCC
TGGTCGTGGAAATTGACAAGTGGCGGGCTCGCCACACGGCGCGCGGACGATCTCGCGGGCAATGAATGAGCAT
TCGCAACCTTCACCATGCGATCGATCCAAGCTCTCTTGCGATAATTGGTGCGTCTGATAGCGACGGATCGCTT

GGCCGGGTGGTTATCGAGAATGTAATCCGGGCTGGATTTGAGGGAGAAATCTGGCCGATCAACCCTAAACATG
ACCAGGTCGCCGGCCATCGCTGCTATCGCCGAGTGGCGGATGTTCCCGGTGTGCCCGACCTTGCCGTCATTGT
CACTCCACCGCAGACCGTGCCGGCTCTGATCCATGATCTGGGTATAAAAGGAACGCGTGCTGCGGTGGTCATC
ACGGCCGGTATCAGCGCCGATCAAGACTTGAGACAAGCGATGCTCGACGCCGCAAAACCATTTCTCCTTCGCA
TAATCGGCCCCAACACAGTGGGCCTCATCGTCCCGTCCGCGAAGCTGAATGCGAGCTTCGCGCATTTGCAGGC
ACAGCCAGGCGGTATCGCGTTGCTTTCGCAATCTGGAGCGATCGCGACTTCGCTGATCGACTGGGCAGCCGAC
AACGATGTCGGGTTCTCCAAGGTCGTTTCCCTCGGCGACATGGCCGATGCAGACGCCGGCGATTTCCTCGATC
TGCTAGCTGGAGATCCCGAGACGCATGCGATCGTCATGTATCTCGAGGCCATCTCCAATCCACGGAAATTCCT
ATCGGCAGCACGGGCCGCGGCTCGGGTCAAACCGATCGTCGCCATCAAGGCGGGCCGCCACGCCGAGGCGGCC
AAAGCGGCCGCCACACATACAGGCGCGCTTTCAGGAGCCGATCGCGTCGTCGACGCGGCTTTGCGTCGTGCCG
GTATCTTGAGAATTGAAGGTCTCGGCGAACTGTTCGATGCGACGGAAACCATCGCGCGCTTTCCCCCTCTTGA
GCACAGCCGTGTCGCAATCGTCACCAATGGCGGCGGTGCCGGCGTTCTCGCCGTGGATCGGCTGATCGACTTC
GGGTGCGCGCTCGCGGATCTTTCTCCAGAGACCGTCGGCACCCTAGATCGAAATCTACCCGCCAATTGGTCGC
GCGCCAACCCCGTCGATATTGTCGGTGATGCACCACCCTACCGCTACAAGACGGCCGTTGAGACGATTGCTCG
GGATGTGGGAGTGGACATCCTGATCGTCATGAATTGCCCGACGGGTCTTGCGTCCCCCGTTGATGCGGCTCAT
GCGATCGCTTCCCTTGCGCAGGCCGGGACAATTTCCGGCAAACCGGTTCTCACATGCTGGCTGGGCGGTCGGA
CCGCTCGTGAGGGTCGCACTGTGCTGCAGCAGGCGGGATTGGCGAGCTACGATACGCCGTCTGACGTTGCCTT
GGCGGCTTCTTACCTCACCAAATGGTCGAAAGCGCAGCGGGTGTTGGTGAGGGTGCCAGAGGGCCGAGACGAC
GAAGTGCATTGCAACCGCGATCTGGCGCGATCCGTTCTCCAGAGGGTGGCGGCCGAAGGTCGCCGAATGCTGA
AGGAACCCGAAGCCAAGGCCGTTCTCGCAGCCTATGGCATCCCCGTCCCTCAAACGATGATCGCAACGTCGCC
GAAGGAGACTGAAGCGATCGCCGGCTTGCTTCTTGCGGGCGCGCCGAAGCTAGTGGTCAAATTGATTTCCAAG
TCGATCACGCACAAATCCGATGTGGGCGGTGTCGTCCTTGACATCCTGTCCCCCGTCGCAGCTCGCGAAGCTG
CCGAGGCAATCGCTGCGCGGCTCAAGGCGCATGACCCGACTGCCGTTGTAGACGGTTTTGCGGTGCAGCCGAT
GATTGAACGCAGACATGCGTGGGAACTGCTCCTCGGCGTCACCCGAGATCCGATCTTCGGTCCGGTCATTCTA
TTCGGATCGGGCGGCGTTTCCGTGGAAGTGGTCGCAGACACCGCGGTGGCGCTTCCCCCACTCGACGCCGTCC
TCGCCGGCGACCTGATCGATGAGACACGAGTTGGCAAGCTCCTCGCCGGTTTCCGCAGCGAGCCGCCGGCAGA
CAGAGCAGCGATCTGCAAAGCGCTCTCGGCTCTATCGCAGCTAATCGTCGATTTCCCATGCGTGCTTTCGATG
GATATCAACCCGCTGGTCGCCAGCGCCGAGGGCGTCATCGCGCTCGACGTGCGTATCGAAGTCGACCCACAGG
CTGTCGCGTGTCCGGGCCCCAATCGCGATCTTGCGATCCGCCCATATCCGGGCGATTGGCAAAAGCAGGTCAC
GCTTGGTGAGCGGATTTATCATCTGCGCCCGATCAGACCCGCAGACGCCGCACTGTATCCGGATTTTATGGCA
GAAACGTCTTCTGGTGACATCCATTTCCGTTTCCTCTCCTCGCGCAAGCACTTTCAGGACCAGATGTTGGTGA
GACTCACGCAGATCGACTACGAGCGCGAGATGGCCTTCGTCGCTCTCGATAATGAAAGTGACGAGCTTGCCGG
AATTGCCAGGCTTTACGCGGATCCCGATCACGAGTCCGCAGAATATGGCTTGCTGGTGAGGACGGATCTGCAG
GGGCGTGGCCTTGGCTGGGCTCTCCTGTCTCATCTGCGGGACTATGCTGCGGCCGATGGTCTGCAACGCATCG
AAGGCCTCGTTCTGAGTGAAAATTCGAAAATGCTGAAAATGTGCAGGGAATTCGGATTCTCAATCTCGACGCA
CCCGGCCGATGCCACCCTGAGGATAGCAACACTGGGACTGCAATGTCAGGCAGGTGTTTCTTGAATGAGCGTT
CAGGTCCGAAGCATCAAGGAAGGTCAACCGGCAGGCATTAGTTCTTCGAGTTTGCCTTCAGCGGATCTTGCTT
GCCTGTTCAGCCGCCAAGCGCTCGAAAAGTTCGAGCCGGGTGAAGCGATCTTCTGGGAGGGCGACGAGGCGAC
AAATCTTTTCGAAGTAGCAGATGGAATGCTGCGAGCACTGCGTTTGCTCGGCGACGGTCGTCGAATTATCGTG
GGCTTCATGCGCCCCGGAGATCTCCTCGGTGTGTCACTCAAGGAGCGTTATCTTTACACGGTCGAGGCAATCT
CCCCGGCGCAAATTCGCCGCTTTCCTCGTCGCGTCTTTGAAGACGAGGTGGCCCGCCATCCACGCTTACAGGA
GCAGCTATTCTCCAAGCTTCGTGATGAGATGACTGCTGCGCAGGATCAAGCCGTCCTTCTTTCCCGCAGGAGT
GCCGAGGAGAAGTTCGCCAACTTCCTGCTGCTGATGAAGGAGAGAGGGAACTCGGAGCATCGTGCCATTGTTG
ACCTCCCCATGACCCGACTGGATATCGCCAATTATCTTGGCATGACGATCGAGACCGTATCTCGCACTATAAC
AAGGCTAGCAAACGGCGGTATTATCGCGGCAGCAGGACGCCGCTCGATCAAGGTCTTGAAGATGGAAACGCTT
CGCCTGCTGGCCGAGGGTGACGAAGGCGAGCAATGGATGCCCTCTCTCGCTCGTCCTGGTCGGTACGCACCCG
CAAACGCTTGAATGCTGTCGCGGTATTTCGAGGAGGAAGGATACCGCGTTACCCTGGCCGGCGACGGACGGGA
GATGCGCGACTGCCTCGATAAACAATCAATCGACATCATTCTCCTCGATCTGGTACTGCCCGGCGAGGACGGG
CTGACGCTTGCGCGCGACCTCCGCGCGCGCTCGGATGTGCCTCTCATCATGCTGACGGGCCGCGACGACGTCG
TCGATCGGGTCGTTGGACTGGAGGTGGGTGCCGACGACTACATTGCCAAACCCTTCCATCTGCGCGAGGTGTT
GGCGCGCGTCAGGGGTGTTCTTCGTGGCCGTCAACCACAAGCGTCGGCGACTGTCGATCTGGCGTCAGAAGTC
TACTCATTTGAGGGGTTGCGCCTCGACATAGCGCGCCGCCAACTCACGTCGGATGATGGCAACGAGATCCTGC
TGACGACCGGCGAATTCGATATGCTCTGTGTCCTAGTGAAGCACGCCGGCCGTGTCTTGCAGCGCGAATTGCT
TATGGATCTGACACGAGGTCGTAATCTCGAAGCTTTCGACCGCACGATCGACGCGCAAATTTCCCGTTTGCGG
CGGAAAATCGAGCGTGACGCCAGCCAGCCAGCTCTGATCAAGTCGGTCCGAGGCGTCGGATATGTCTTCAGTG
CAAGGGCGACACGGCAGGAAGCGTAGATGAAGCGTCGCATTCTTTTGCAAGGCACCACTGCATTGATGGTGCT
GTCGGCACTTCCGGCTTTCGCGCAGGATGACATCAGGGCGGTCGCCCGTGAGGCGTATATCTACACCTATCCA
ATGGTGAAGAACTACCTCACGATGTACCAATACGCGCTGGACCCGGGCGGCAGCCAATACAAGGGGCCACTCA

ACACTCTGGTCAGCATCGCCCGGGTCTACACGCCTGAAGATACGGCAATCATCACGCCGAATTCCGATACGCC
CTATTCCTTCATCGTCTTCGATCTGAGGGCGGAGCCTGTCGTAGTGACGATGCCGCCAATCGAGAAGGATCGC
TACTATTCGCTGCAGCTCATCGATCTATACACCAATAATGTTGACTATCCGGGAACGCGGGTCGACGGCAATG
GCGGCGGCGATTTTCTAATCACCGGCCCCGGCTGGAAGGGCGATGTGCCGAAGGGCATTAAGCGCGTGATCGA
AATGCCGACCACGCTCGCGTTGGGCATCATCAGGACGCAGCTCTTCTCGCCCGACGATCTCGAGAAGGTGAAG
CAGATCCAGGCGCGCTACAAGGCCGCGGTCCTGTCGGCCTATGCGGGGGCTCCGGCTCCGGCTGCTCCTCCGT
CGATCGACTGGTTGCTTATCTCGGACGAGCTGATGGTCACGGACTACTGGTCGATTGCCGCCTTCCTGCTGCA
GTTCGCTCCACCATATTCGGGTGATGAGGCGCAGCGGGAAAACCTCGCCAAGCTTGGTATCCGGGATAGAGGA
GTGTGGCCCGGGACGGATCTTTCACCCGAGACTGTGGCACTGATGAAAGAAGTGGCGGTCGCGACGGAAAAGG
AGATTCGTGACGAGGCTGCCCGGTTGACGGATTCGTCCAAGATATTCGGTACGCCCGAATTCATGAAGGGCCG
GTTCATGGTCCGGGCCGCGGCGGCGCAGGGCGGGATCTATGGTAACTCCGTGCAGGAGGCACTTTATGTCATT
TATGCCTTCGACGCACAGAAGGCGCCACTTGACGGTAAGACAGGAAGATACAAGCTGACTTTCACCCCCAGGA
CGCTTCCACCGGTTGATGCTTTTTGGTCGCTGACCATGTATGATAGGCAAAATCAATTCCTTGTCGACAACCC
CATCGACCGTTATCTGATCAATTCGCCGATGCTTGCCGGACTCAAGAAGAGCAACAAGGGCGAGATCGTCCTG
TACCTGCAGCGCGAAAGCCCAGGAGCGGAGCTGGAATCAAACTGGCTCCCCGCCCCGAGCGAAATCTTCTATG
TGGTCATGAGGCTCTATCTGCCCAGGGCGGAGGCGTTGGACGGGCGCTGGGCGCCGCCGCCGATCGAAGCTCT
TTCGTGAATGCCGGCCGCCAAGGCGCAGGCACTGGTCCAGGGTCTGGTGATGATCGATGCCGATACGGCAGCC
GATCTCGCTGCCCTCGAACGGCTGGCGCTCTGGGCCTTGTCACAATATTCTCCGGTTGTCGCGATGGACCCTC
CCGACGGTATCGTCATGGATACCGAAGGCGCCGATCATCTTCAGGGCGGCGAGGACTTGATGCTGTCCGGCCT
CGTCAATCGCTTCCGCGGCCGGGGTCTCGCTGCCCGCGCGGCAATCGCCGATACCTGGGGTGCCGCGCATGCG
CTTGCCCGGACCACAGATCGCGAGACGGTCATCGTCGGGCGCGGCGATGCCGCCAGGGCCGCGAACCGACTGC
CACTGTCGTCGCTGCGGCTGCCGGCCGAAACTGTTCAAAGCCTTCGAACCCTCGGCTTTACCACCGTCGGCGA
CTTGGCTGCCACACCGCGGGCACCGCTGACGCTGCGCTTCGGCCTGGAGGTCGGCCGCCGTCTCGATCAGATG
TTCGGCCATATCGCTGAGCCGATAGATCCGATCCGGCCGGCGGAACTTGTCGAGGTCCAAAAATCTTTCGCTG
AGCCCATCGGCGCGCCGGAAACGATCGAGAAATATGTGAGCCGCCTGGTGCGGCAGCTCTGTCTTGAGCTCGA
GAAGCGCGGGCTTGGGGTCAGACGCGCCGACCTCATCGTCCATCGCGTCGATAACACCATTCAGGCACTGAGG
GCGGGGACGGCGAAGCCGGTCCGTGACATCGCCTGGCTGACGAAACTCTTCCGCGACCGCATCGAGAGGATCG
AACCAGGCTTCGGCATCGAGAAGCTCGGTCTTGCGGCCATCCTCGCCGAGCCGCTGATTGAGGCACAGTCAGC
CTCGTCACTCATTGAAGAACAGGTAACCGATGTGACGCCGCTCATCGACGTGCTTGGCAATCGCGGTGGACAG
CGGATATTCCGTGTGGCCCCGGTCGCAAGCGACGTCCCGGAACGTAGCGTCCGGCGGATCGCTCCGATCGCCG
ACGAGGACGGCGCCACCTGGCCGCTCAACTGGCCAAGACCGCCGCGGCTTCTGGCGCGTCCCGAACTGATCGA
AGTGATCGCGCTTTTGCCCGATCATCCGCCCGTCTCGTTCACGTGGCGCAGCAAGCGGCGCAGAGTGAAGCGC
GCCGACGGACCGGAGCGGATATTTGGGGAGTGGTGGAAACGCAGCTCAGAATGGGTTGCGGTCCGCGACTATT
TCGTCGTCGAGGATGATAGGGGCGAGCGCTTCTGGATTTTCCGATCCGGCGACGGCGTCGATGCGGAAACAGG
ATCGCATAAGTGGTTCATGCACGGGATGTTTGCGTGAATGAAGCGAATCGTCTTGCTGCTATCACCTCTGGTA
ATCTTGCTTAGCCCGATCATTGACGCGTTTCAGGGAGTGTATATTGACCCCAGAAGCGATGCAGGGTACGCCG
TCATCGCTTGTGTTGCCATAATTGGGCTCGCCCTGGGCATGATTGCAACCGTTTGCTATAAAAGGGGAGCTGT
TGGCAACGTCGTAACAGCAGGCACACTCGCCGTTACCCTTTTCCTTTTTGGCGATCTTTCATACGGTGTTTTC
TGGAGACTAGCAGACCACATCGGAATGGGGGGCGCCGCCGCATTGGCGTTTGCTGGACTGGTTGTACTTATTC
TTATCCTATTCAAGCTCATGGCCGCTGTGCCCCGTATGATGGCCGCATTTGCCGTAGCCCTACTGGGCTCTAC
AGTTCTTGATCGCGGCTTGATTACTGAGGCGAGCGCCGAAGAACCCGCGCCGGCCGTGATTTATATCGTGACT
GATGAAATGATCGGCATTTCCGGGATCGACACAAGATTACCCCATGGAGCAGAAGCCAAAGCCGCATTGTCGC
GGGTTTTCCAGAAACATGGGTTTCGGCTCCATTCCAAGGCTTTCAGCCGCCACATTCTGACTCAGGTTTCAGT
ACCAGCCGCCTTGAACATGGACTATTCTTATAATTTTCCCGGCGATAGATCGCATTATGCCTACCCGGGGGAG
GTAACAAAGTTCAAGGAACTGTCGTTATTCAATCTGTGGCATCGACAAGGATTATCCGTCAATGTCTTCCAGT
CTGCGCATCTTGATTACTGCAAAACTGAAGCGCTAGTCGACTGTCACACCTTCGCATCATTTGACGGAAGTAG
ATTTATTCAACGGCAGCGGATCGAGGGCGGCCCATACCGGGACGTTCCACCCGCTTCCGCTTCGCTGGCGGAT
GTTAAGGCGCTGGTCGAGGGAAACAGGCAAAGCCTTGCAATGGCAGCCGTTGGCTTCGTGGCCTCCAACTTGC
TGGAGACGAAGGAGACGGCACTTCAAGAATGGCACCCGCGATCTTACGACCAACTAGCCTTCCCTTACTGGCT
CGGACAGTTTCAGAACATGATTCTAGATAAAGGTCGTGGAAACGCCTTTTTTGCACATTTTCTATTTCCGCAT
AGTCCCTCGGTCTACAACAAAGATTGCAAACCAACGAACCGTTGGGTTGAGAGGTCCTATCTTACGGAAGTCC
GCGGGTTGGCAGGTCAAGAGCTAGATGAAGCGCGGGTGAAAGAATATGGATTCTATTTCGCTCAAACGCTGTG
TCTCGCCAAGCAACTTGATAAGTTCTTTAATGCGGTTCTCAGCGATAAGAGATTTCAGGACGCAACGATAGTC
GTTCATGGCGACCACGGCTCACGTATTTCTGCGGGTAGAAATGTCGAGACTATGTCTCCGAGGGATTACGTCG
ACAATTATTCCGCTCTTTACGCGGTCAGAAAGCCGGGCGTGGCGACGGGTACGGACTACAAGCTACGCTCGGT
CCAGTGGTTAAACGCTTCTCTGTTCCGTGGCAATGTATCAGAGGTTGCCCCGACCGTAGTAGGATCTATGCAG
GGTAAATCTGACGATCCAGCAGTGTACGCACCCATTACCGACCCAGACCACGTTGCTATAATGCCGATGCATG
ACTTTGACGCATCCCATTGAATGGCAAGCACCAATTTCCGTCCGCTGCACGACCGCGTTGTCGTCCGCCGCGT

CGAGTCTGAAGAAAAGACCAAGGGCGGCGTCATCATTCCGGACACCGCTAAGGAAAAGCCGCAGGAAGGCGAA
ATCGTGGCTGTCGGTTCGGGTGCCCGTGACGAAAGCGGCAAGGTTGTTCCGCTCGATGTGAAGGCTGGCGACC
GCATCCTGTTCGGCAAGTGGTCCGGCACCGAAGTCAAGATCAACGGCGAAGACCTTCTGATCATGAAGGAAGC
CGACATCATGGGTGTCATCGGCTGAATGCTTGTCAAGGATGTGATGACGACAAAGGTTGTCAAACTGTCGCCT
GACAACAGCGTTAGACAGGCAGCCAAGCTGATGTTCGACCACCACGTAAGCGGCGTCCCGGTCGTTGACGACG
ATGGGCGCTTGCTCGGCGTCATCAGTGAGGGCGACCTGATCCGGCGGACGGAACTCTGCAGTGGAGCATCCGT
GTTGATGGCGGAGATGGCGATCGATCCCGATGACAGGGCGAACGCCTTCATCAGGCGATGCTCGTGGCGCGTC
GGCGACGTCATGACGGCCAACCCCGTAACCATTGAGGAGGAGGCGCCCCTTGCGCGCGTTGCAGGGCTCATGC
AGGAACGCGGTATCAAGCGCATCCCCGTAATGCGCGATGGGGAACTGGTCGGCATCGTCAGCCGCGCCGACCT
GCTGCAGGCAATTTTTTCAACGAAGCCAGACGAAACAGCAGCGGGCGACGAAGCCATTCGACGCAGCATCCTT
GTTCGCCTCGGCGAAAACACGGGTCTGGAAGAACTGGACGTGACTGTCACGGTCACCGAGGGGATCGTGCACT
TTTGGGGCCAAGTCGAGACGGCCGCTTGTCGCAGGGCCGCGCGGATCGTGGCTGAGAGCGTCCACGGGGTCAG
GGGCATCGTGGAGCATTTTCCAGATCCCTACACTCAATAGGTGACGCAGTCTGCAGATAATAAAAGACCTGAC
TCCGGCGTGTCCGACGACTCGATCAACATGCTGCTGCGGTTTGCCGTTATGATGCTTCGTTCCGGGGGCACCG
CGATCCGTACCCGCGAACTGGTGGGGGCGATGGCGCACCGCCTCAACCTGGAAACGCCATCGGTGAGTCTCAC
GCTCGAGTCCGTCACCCTCAGTGTTTACCACGGCAGCGAACAGTTCGTAGCGATGCGCGAGGTCGGTCCGCCC
GGAATCGACTTCAGGCGCATCGGTGAGTTGGAGCGGCTTGCGAACGCGTTAGAGCCAACGGCACCGCCACACC
GGATAGCGAAAGAACTTGCTGAAATCGAAGCGCGGGCGCCTCAATATACCGGCTGGCAGATGGCGATCGCGAT
CGGCCTGGCCAGCGGAGGCTTTGCGTTCCTCAACGGTGCGGCCCTGCCGGAAATGGCCAGCGCCGCGATCGGT
GGCGGCATAGGTCAAGGATTGCGCTGGTGGTTGACCCGCCGCCAATTGACACACTTCGGCACGGCGGCACTGG
CTGCCATAACCGCTGCAGGAACTTACGTTCTGGTGGCAGCGCTGGCGCGCCGTGCAGGGTTTGCGTTCTCGCA
CTATGCTGCGGGCTTCATCTCCACCATACTATTTCTCATCCCCGGCGTTTCGCTGATTGCAGGACTGTTCGAT
CTGTTGCAGCATCAGACGGTGGCCGCCTTGAGCCGACTGGCATATGGCGCGCTGATCCTGTTCATCGTCGCTT
CGGGGCTGAGCATCGTGGTGACTGTTGCGGCCATCGAGCTGTTGCCACGCTCGGCGCCGGCCGAGCTTGCCTA
TCCGTTGCGCCTTTCGTTGCGCGCCGTCGCGAGCTTCGTTGCCGGCTGCGGCTTCGCCATGCTGTTCAACAGC
ACGCCACTTTCGTTGGTTGTCGCGGGCATCGTGGCGCTGGCGGCGAATAGCGTGCGCCTCGTCCTGATCGACA
TGGGAATGCTGTTGGCGCCGGCGGCGTTCATCGCCGCGTTGTTGATAGGAATCATCGCCATTCTTGTGAGCCG
GCGGTTGGACGCAGAGCTCATGGCGATTGTTACCGCGCCGGTCGTCATCATGATTCCGGGTCTCTACGTATTC
GAGATGCTTGTTCTGCTCAACCGGGGGCAGACGCTCGAGGCCATGCAGGCCTCGGGGGCAGGGATCTTCGTGA
TCAGCGCGCTCGCGATGGGGTTGAGCGTGGCGCGCCTCGCAGTCCCGTGGGAAAGTTAGATGATGCCGTTCAA
CCGGGCGAACAACGGCGTCGGTGCGCGCTCGTTTCGCGCTAAGTTCATCCTTGTCGTCGGCGGCGCCGTGCTT
TTCGACCTGCTCGTCACCGGCGGCCTTGCTCTCTGGAACGTGCAGCGACTCTCGCGAGACGCGACGGCACAAG
TCGGTGAGGGCCTTGAGGCGGCAAACGAGGACTATATCCGCTCCTACGCGGAAACGACGGCCGAGCGCGTCAA
TCTCCTTTTAGACCAAGTTCACTCTGACGTCGGCGCTCTGGCGGGTGTGCTGCAGGCGCAGATCGACCATCCC
GAAAGGGAAACTCAGATCGGCGAAGCCTTGGCCCGCGGTGCGCCCGAAGCCGTCACGGTCAGCTACAACGAGA
AGGGCGGGTGGGCACAGAACTTGCCTGGATCTTCGTCAGTCGTCAGCGTTTGGGGCTACCTGCTCGGCTCCAA
CCGCGTCCCGTTGCCGGAGGTGCAGCGAGAGATCGAGAACAGCGCGGTAGTTGACCTCGTTGCTCCTAGCCTT
CTGGCAAACGGCCTATCGAAACTGCAAATGTACTATATCGGCCCGAAGGAGCGGCCGATTTTTCGCACATCTC
CGTACACCGACCAGGCCCAGACCTTCGACAGGCTGTACCCGGGCCACAACCAGGCGGAATTCTGGCCGTTCTT
CTTCCCGGGACTGTACGAATCATGGGAGCAATGGGCGGCGGATCCAGCGTCCCGCCCGGTAGAAAACGACATC
ACACAGACTGCACCCTACACGGACGCGATCACCGGCAAGCTGATCGTCAGCTTCTTCCAGCCGCTCTGGTCGC
GCGGCCGTGACCGCGTCGCGGGTGCCGCCGGCGCCGATATCACCCTCGACCAACTGGCAGAGATTGTTGAGAA
TGTCAGGGTCGCTGACAAAGGCTTCGGCTTCCTCACCATGTCCGACGGCAATGTCGTCGCCATTAATCCGGTC
GGCGAAAAGGTGATCGGATTGCAGACCTCGAGCGACGTCAACGGCCAAGGTGTGACGGGTCTAAATCGCTCAT
TGCGCCACAGCGCACAATCGGCGATCGCCAAATTACCCTTGGAGCAGGACGGGCTGCTGCAGCATGTCCTGCT
TGAGGAAAACGGCGAGAAGGTGCCCTATCTCGTCATGCTGAAACAGTTGCAGCCGAGCAACCTTTGGGCCTCC
GGTCCGGTTCGTCGTGAATCCATGCTGCTTGGCATCGTGGTGCCGGAGCGTGAAATCTATGCCTCCCTCTTCG
CCGCACAGGCCGGCATCAACAAGGCGGCGAACAGAATCCTAGTCTATCAAGTGCTAGCTCTGCTCATCTCGCT
CCTTTTTGTCACAGCGGCCGTGTTCGCCATCTCCGGGCGGATCACGAGCGGCCTCAGCGCGCTTGCCGATGCG
GCAAAACGAATCCAGCTCAAGGACTATTCCGTCCGCGTCAACATTCCGACACGCGACGAGGTCGGCGAGGCCG
GAAACGCCTTTAACCGCATGGCCGAACAGATCAGCTATCACACCGAAAACCTGGAGCAACTCGTCCAGGATCG
AACGAAGGAGATTGAAGAGGCAAACCTGCAAATATCCGGCCTGAATGAGCAGCTGCGCAGCGAGAACGTGCGC
CTCGGGGCCGAACTGGACATCGCGCGCCAGATCCAGATGATGGTCCTGCCAAGGGCCAGCGAGCTTGAGGAGA
TCGCGGACCTGGAGATCGCTGCCTACATGCGACCCGCAGACGAGGTAGGCGGTGACTATTATGATGTGCTGAG
GGACGGAGAGCGGCTGAAGATCGGTATTGGCGATGTCACCGGACACGGCCTCGAGAGCGGCGTGCTCATGCTG
ATGGTGCAGTCGGTCGCACGGGCACTGCAGGAATCCGGTGAAATGGACCCCTCGCAGTTCCTCAACAGGCTCA
ATCGAGCGATTTACAAGAACATCGTCAGAACCAGCACGGACAAGCATCTGTCGCTTGCATTTTTGGACTACCA
GGCTGGACAGCTGACATTGTCGGGTCAGCATGAGGAAGTGATCATGGTTCGGGCTGCCGGAGCCGTCGAGCGA

ATTGATACGATCGATCTCGGTTTGCCGATTGGCTTGGAGTCCGACATCTCGGCCTTTGTGGCGACGCACGAGA
TCCCCTTTGCACGCGGCGACATGGCTATTCTTTACACAGACGGCGTGACCGAGGCTGAGAGTTGCAGCAGAGA
ACTGTTTGGAATCGAGCGGCTTTGCGCAAGCGCCCGTCGCCTTTCCGGTCGGAGCGCCGAGGAAGTCAAGACC
GGCATTCTCGACGACCTGGTGGCGCATATCGGCACGCAGAAGATCCATGACGACATCACGTTAGTAGTGATGA
GGCACAGGTAGATGAACCTATTCGGCACACTGAGGGCGCCGCGCGAACTGCTTTTCGGCGCCGGGCAGCGCCA
TGCCCTTGGGGGCATCGCCGCCAAGCTCGGCCATCGCGCGCTGATCGTCACGGATACGCGCCTTGCCGTGGAT
GCCGACTTGCTTGCACTGGTGCGGCGACTCGAAGAGGCAGGGCTTGAGGTGATGGTGGACAGCTCCACCCTGC
CGGACGTCCCGGTGGAATCGGCTATCGTGAGCGCTGCAGCGGCCAGAGGCTTTGCGCCGGACCTCGTCATCGG
CATCGGTGGCGGGTCCTGTCTCGACATGGCGAAATGCGTCACCCTCCTCCTTACCCACGGCGGGCGGCCACAG
GACTATTACGGCGAATATGCCGTGCCGGGACCCGTGATGCCATTGATCGCCATTCCCACCACCGCCGGCACCG
GCTCGGAGGTGACGCCCGTCGCCGTGCTTTCGGATGCCGAGCGTAGCCTGAAAGTTGGCATTTCAAGTCCGCA
CCTGATTCCGGCGGTATCGATCTGCGACCCCGAACTCACGCTCTCCTGCCCATCCGGCCTGACTGCGATCGCC
GGCGCGGACGCCCTGACGCATGCGATCGAAGCCTTCACGGCGATCCGGCGCGAGCCCGTCCCCGGTATCGCGC
AGCAGCGCGTCTTCGTCGGTAAGAACGAGCTTTCCGATCACTTTGCCCTTAGCGCCATTACCCTCCTGTGGCA
GGGGCTGGAGAGGGCCTGCAAGGACGGCGCCGACGCCGGCGCGCGCGAAAAGGTGATGCTCGGCGCAACGCTC
GCCGGTCTTGCTTTCGGGGTGGCGGGTACTGCCGCGGCCCACGCCATCCAGTACCCTGTCGGCGCGCTCACCC
ATACGGCGCATGGCCTCGGGGTCGCCTGTCTAATGCCCTATGTCATGACTTGGAATGCGCCGCTCATCCGCGA
CGAACTCGCGCAGATCGCGCATGCGGCCGGGCTCGGCGGACCGGATGAAGTGATCCCTGCCCTCGTTTTGCTC
TTCGAACGCATCGGCATTCCCGCCACCCTGCGGGACCTCGGCCTCGAGGAAGACCGGATCGATTGGGTGGCGG
AACAAAGCTCGGGTATCGCACGCCTAATCCAGAACAATCCCCGCCCCTTGAACCCGCACGAGATGCGCAACCT
CGTCGCCGCCGCCCATTGCGGCGATCGCTCCCGCCTGAACTGAATGACCATCCGTAAGATGCTTCTGGCATCG
GCCGCGATCACTTGCGCCGCGATGCCCGCCTCCGCATTTGCCGACACCTCGGCCAAGAAGATCGCCCTTTCGA
ACAACTATGCCGGCAATTCGTGGCGCCAGGCCATGCTGACGAGTTGGGAAAAGGTGACGGGCGAAGCCGTGAA
GGCCGGCATCGTCGCCTCGGCCGACGCCTTCACCACCGCCGAGAACCAGGCAACGGAACAGGCCGCGCAGATC
CAGAATATGATCCTGCAGGGCTATGACGCGATCGTGCTGAACGCCGCCTCCCCGACAGCGCTGAACGGCGCGG
TCAAGGAGGCCTGCGATGCCGGCATTACCGTCGTCTCCTTCGACGGCATCGTGACCGAACCCTGCGCCTGGCG
CATTGCCGTCGATTTCAAGGAAATGGGCCGCAGTCAGGTCGAGTATCTTTCGAACAAGCTTCCGGACGGCGGC
AATCTGCTCGAGATCCGCGGCCTTGCCGGCGTATTCGTCGACGACGAGATCTCGGCCGGCATCCATGAGGGCG
TCAAGCAGTTCCCCCAGTTCAAGATCGCCGGCTCCGTGCACGGAGACTGGGCGCAAGACGTGGCGCAGAAGGC
CGTTGCCGGCATCCTGCCGAGCTTGCCTGACATTGTCGGCGTGGTGACGCAGGGCGGAGACGGTTATGGTGCC
GCGCAGGCCATCGCCGCGACCGACCGGAAGATGCCGATCATCGTGATGGGCAATCGCGAGGATGAACTGAAAT
GGTGGAAGGAGCAGAAGGACGCGAACGGCTATGAAACCATGTCGGTCTCGATAGCGCCTGGTGTCTCCACTCT
CGCCTTCTGGGTCGCCCAGCAGATCCTCGACGGCAAGGAGGTAAAGAAGGACCTCGTCGTTCCCTTTCTGCGC
ATAGATCAGGACAATCTCGAAGCGAACCTCGCAAATACGCAGGCCGGTGGCGTCGCCAATGTGGAGTACACGC
AGGAAGACGCGATCAAGGTCATCGAGTCGGCAAAGTGAATGAGCAGTTTTCGACGGAAGTTGACGACTTCCGC
TGTCGCGGCCATCTGCAGCTTGGTTGCTTCCACAGCGGGCGCCCAGACGGTGTTGAAGGCGTCGCACCAATTC
CCGGGGGGCAAGGGCGACATTCGCGACGAGATGGTGCAACTGATCGCCCGCGAGGTCGCCGCCGCCAATGTCG
GCCTCGAAATCCAGGTGTTCCCGGGGTCGTCTCTTTACAAGCCGAACGATCAGTGGAATGCGGTTACACGTGG
CCTGCTCGATATGACTTCGTTCCCCTTGGACTATGCTTCCGGCCGCCACCCGGAATTCTCCGCGACGCTGATG
CCCGGCCTCGTGGGAAACTTCGACCGCGCCATGCGGCTTAACGACTCCGAGTTCATGGGGGACATAAAGAAGG
TCATCGAGAATGCCGGCGCCTTGGTGATCGCCGATGCGTGGCTCTCCGGTGCCTTCGCCTCCAAGAAGAATTG
CATCACCTCCCCCGACACGATCAAGGGACAGGTGATCCGCGCCGCCGGGCCGGCCTTCGAGGAGATGCTCGTG
GAAGCCGGCGCTTCGATCTCATCCATGCCTTCGTCGGAAATCTACACCGGCATGCAGACGGGCGTCCTGGATG
CGGCGAACACCTCGTCGGCGAGTTTCGTCTCCTATCGCCTGTTCGAACAGGCCAAGTGCCTCACGGCGCCGGG
AGAAAATGCCCTCTGGTTCATGTACGAACCGGTGCTGGTATCGAAGCGCGTCTTCGATGGCCTGACGGAAGAG
CAGCAAAAGGCAATCCTCGCTGCCGGCGAGAAAGCGGAAGTCTATTTCAACGAAGAGGTCCGCAAGGGCGATC
AGGTCATGATCGACACCTACAAGAAGGCAGGCGTCGAAGTCGTCGAGATGTCGAAGGAGGATTACGATGCCTG
GCTCGAGCTCGCCAAGGCGTCGTCCTACAAGAACTTCGCCGCCAATGTTCCCGGCGGCGACAAGCTGATCGAA
AAGGCGCTCGCCGTGAAGTAAATGCTCAAGGCCTATTCACAGGCTGTCGGCAATGCTTCGCGTGGCCTCGCGG
CCATTGCGACGGCCCTGCTCATTGCAGCGATGCTCGTCGTCTGCCAGATGATCATGCAGCGCTATGTATTTCG
TCAGGCGACGATCTGGCAGACGGACTTCGTCGTGTTTTCCGCAACCGCAGCCATGTTTCTCGGTGCGCCATAC
GTCTTGCTCAAGGGCGGCCATGTGGGCATCGACGTCGTCGAAATGGTGGTGGGCGAACGTACGCGATACGTTC
TGCGCATCATTGCGGGCCTGCTCGGGCTCCTGTTCTGCGCCGTCATGCTGATTGCCACATGGATTCAGTTCCA
TGACGCGTGGGCGGGAAACTGGAAACACTCCAGCGTCTGGGCGCCGCCGCTTTGGGTGCCGCTCGCTGCCCTG
CCCGTCAGCTTTGCCATGCTGTGCCTGCAATATGTCGCGCAGATTCTGACGCTCCTCACCGCCCCGGCAGTCC
CGGCAGCCATGGGCCACGGCGCGGCCGAACCGGGTTCGGCCCCTGGCGCCGACCGTCATCCACAGGAGATCAT
TCAGTGAATGATGCCGTTCGTCATGGACGAACTGCAGCGGAATTATACGGTTCACAGGCTTTACCAGGCCGCC
GATCGGCCGGCGCTCGAAGCGGCACTCCCATCGATCCGCGCGGTTGCTACGGGCGGCGGCGCGGGCCTTTCCA

ATGAATGGATGGAGAAGCTTCCATCACTGGGGATCATCGCAATCAACGGGGTCGGGACGGACAAGGTGGATCT
CGCCCGGGCGCGCCGTCGCAACATCGATGTGACGACGACGCCGGGTGTCCTCGCGGACGACGTCGCTGACCTC
GGTATGGCGCTCATGCTCGCCGTCCTGCGGAGGGTCGGCGATGGCGACAGGCTGGTGCGCGAAGGCCGCTGGG
CCGCTGGCGAACAACTACCGCTCGGCCATAGCCCGAAGGGAAAGCGGATCGGCGTACTCGGCCTCGGTCAGAT
CGGGCGGGCACTGGCGTCGCGCGCGGAGGCCTTCGGCATGTCGGTGCGATACTGGAACCGGTCCACCCTTTCC
GGCGTCGACTGGATCGCGCATCAGAGCCCGGTCGACCTCGCGCGCGACAGCGACGTTCTGGCCGTCTGCGTGG
CGGCGAGTGCGGCGACGCAGAACATCGTCGACGCGTCCCTTCTTCAGGCGCTCGGTCCCGAGGGTATCGTGGT
AAACGTCGCGCGTGGCAACGTCGTCGACGAGGACGCCCTGATCGAAGCGTTGAGGTCCGGAACGATCGCGGGT
GCCGGGCTCGATGTCTTCGTCAACGAACCCGCAATCCGCAGCGAATTCCATACCACGCCGAATACGGTGCTCA
TGCCGCATCAGGGCAGCGCGACGGTTGAGACGCGCATGGCCATGGGCAAGCTCGTGCTTGCAAATCTCGCCGC
CCACTTTGCCGGCGAAAAAGCGCCGAACACCGTCAATTGA
>2B40 Concatenation of 3 sequences
ATGGACATCAGCATTGCGCGTACGTTCCTCGAGGTGGTCAAGACGGGAAGCTTCGTGAATGCCGCTGCCAATC
TGAACATCTCCCAGACGGCAGTGAGTGCGCGAATTCGCGTACTCGAGGAACAACTCGATCGGCCGATGTTCGT
TCGGAACAAGGCCGGTGCCAGGCTGACCCCCGCCGGAGAGCAGTTTTTCCGTTTTGCAACGAGCCTAGTGCAA
GTATGGGAGGCGGCGCGAACCACGGTCGCTTTGCCCCCGGGACGCGAGGCCATGGTGACGATCGGTGCTGAGC
TCAGCCTGTGGAATCCGCTGTTGAAGCATTGGCTTTTGTGGATGCGCCGCGAATGCGCCAGCGTCGCCATTCG
AACGACGATAGACAGATCGGAGCGGCTGATGGAACTCGTGCAGGACGGTTCACTTGACGCGGCGGTCCTTTAT
GGAGCGCCAAATCGTCCAGGCACGCTTACGGAGCTGCTTTTCGAAGAAAAGCTCGTTCTCGTTCGCACTACAG
CTTTGTCGCAGCCGCCCGGACCGGAAGACCTCGTTGGCATCGATTGGGGAGAGGATTTTGCCGGCAGCTATAG
GGCGGCCTTTCCGGGCGGCCGGAATCCGGTGGTAGCGATCAGTTATGGGCCGCTTGCTCTCGATTACATTCTG
GAAGTCGGCGGATCCGGATATTTTCGGCAGGGCTTCGTTCGGCCCTATCTTGTGGACGGCCGCCTCGAGCTCG
TTCCCAACAGTCCGGAATTTGCCTACCCCGCTTATCTCGTTTCTTCCGCAAAGGCGGATCGGTCTATGCTGGA
TCGCATTCGGGCAGGATTGAGGGCCGCCGCATCCAGTGCCGGCGTCTGAATGTTCGGCATAGACATCACCGTC
CTGGGATTGGCCGGCCTCGTCGCCCTGGCGGCGGCAGCACTCGCCTATGGTGTGCTCTACCCCCAGATCGAGA
CCGAGAAGAAGGCGGAGGGCCGCCTGCGCCGGGTCAGCGCCTCCGAGACGGACCGCACCAAGATCAAGGCGGC
GCGCGACCGCGTCAACGAGATGTCGAAGCGACGCAAATCCGTTCAGGATTCGCTGAAGGAACTCGAGAAAAAG
CAGCAGGAAAAATCCGCCAATACGGCGCCTTCGATGAAGAAGCGCCTGCTGCAGGCCGGTCTTTCGATATCGC
TCGCGCAGTTCTACCTGTTCAGCGCGCTTTTCGGACTGTTCGCCTTCCTTGTCGTCCTCCTGGCAGGGGCGGG
GCTGCTCATTGCCGCGGGCGTTGCCCTGATAGGCGCGGCCGGCCTGCCGCGATGGATCGTCGGCTCCATGGTC
AAACGCCGTTGCAGAAAGTTTCTCGACGAGTTTCCGAATTCGCTCGATGTCATGGTCCGTTCGATCAAGTCGG
GGCTGCCGCTGAACGACGCCTTGCGGCTGATCGCCAGCGACGGGCAGGAGCCGGTCAGGACGGAATTCCGCCG
TGTCGTCGAGTCCCAGCAGGTGGGCCTCAACGTTCCCGAAGCCTGCGCCCGCATGATTCACAGCATCCCGCTG
CCGGAAGTGAACTTCTTCGCGATCGTCATCGCCATTCAGGCGCAGGCGGGCGGCAATCTGTCCGAAGCGCTCG
GCAATCTCTCCAAGGTGCTGCGCGAACGCAGGAAGATGAAGGCGAAGGTCAGCGCGCTGTCGATGGAGGCCAA
GGCGTCCGCCTGTATCATCGGCGCGCTGCCGTTCATCGTCGCAACCCTCGTCTATCTGACTTCGCCCGATTAC
ATGATGGTTCTCTTCACCGACCCGCGCGGCCACATCATCATGGGCGCTTCCGCCGTCTGGATGAGCATCGGCA
TCTGGGTGATGCGCAACATGATCAACTTCGACATCTGAGTGAATTCATCCGCACCCCCGATCCGCGTGCTCAG
CGCCGGGCTCGGCAATATGGGCCGCAGCCACGCGCTCGCCTATCATCAGAACCCCGGTTTCGAAATCGCCGGG
CTGGTCAACCGCTCGAAGGTGGCGCTGCCCGAGGCTCTTGCAGGATACGAGATTCTGCCGTCCTTTCCGGAGG
CGCTCGCCGAACTGGAGCCGGAGCTCTGCTCGATCAACACCTATTCGGACACGCATGCGGACTACGCCGTGAT
GGCGATGGAGGCGGGAGCCCATGTCTTCGTGGAGAAGCCGCTGGCAACGACGGTCGCCGATGCCGAGCGCGTC
GTCGCCTGTGCGCGGGCCAATGGCCGCAAGCTGGTCGTCGGCTATATCCTGCGCCACCACCCGTCCTGGATGC
GGCTGATCGCGGAAGCCCGCAAGCTCGGAGGGCCTTACGTCTTCCGCATGAACCTGAACCAGCAATCGAGCGG
CCCGACCTGGGCGACGCACAAGTCGCTGATGCAGACGACACCGCCGATCGTCGATTGCGGCGTCCACTATGTC
GACGTCATGTGCCAGATCACCGATGCCAACCCCGTCGAGGTGCGCGGCATGGGCTTGAGGCTCTCGAACGAGA
TAGCCGCGGATATGTACAATTACGGGCATCTGCAGGTGATCTTCGAGGACGGCTCGGTGGGCTGGTACGAGGC
GGGCTGGGGGCCGATGATCTCCGAGACGGCCTTCTTCGTGAAGGACGTGATGTCTCCGAACGGCTCCGTGTCG
ATCGTCATGGACCAGAACGCCAAGTCCGACGACATCGACGTGCACACGAAAACCTCCGTCATCCGCGTGCACA
GCGCCGCTACGGGACCGGACGGCCGGTTCCTGAAATCCGACGAGGACCTGGTGATGGAGGGCGAGCCCGGCCA
CCAGGAGCTTTGCGACCGCGAGCAGGCCTTCGTGCTCAAGGCCATCCGAGAAGACATCGATCTTGGCCGGCAC
ATGGACGATGCGGTGCAGTCGCTGAAGATCTGTCTCGCCGCCGACGACAGTGTGCGCACCGGCAAGCCGGTCA
AGCTTTAAATGACGAAATTTCGGAATTTCGTTGTCAGCTGCGCACTCGCCGCCGCCTCCGCGGTTCCCCCTGT
AACGTCCGTTCAGGCCATGCCGCGCCCGGCGCTCGACATTGCGATCAAAGCGCCGGCCGGCGTCGAGAATGCC
ACGCATCGCGGCCGTTACGGGTGGCGCGGCGGCTATTCTCGCCGCGGCTATTATGGAGGATCCGGCTTCTATC
CGGGCTGGAGCCCCTATTACAGGCGGTCCGGGATCAGCTTCTATTTCGGACCCAGCTACCGTTACCGCCCGTA
TTACGGGAACCGCTATTATTACGGACCGCGCTTTTATGGCCCCGGCTATTACAGCTATCCCGGGCCCGCATAT
TACGGTTATCGCTCCTATAGGCCACGCTACTATGGCGGCGGCTACGTCCGGAGCGGCGGCAACAGCCATGTCA

GCTGGTGCCTGTCGCGCTATCGGTCCTATAACCCTGCCTCCAACCGCTACCTGACCTATGGCGGGGTCTACCG
GGTCTGCTACTCGCCCTATCGTTAGATGACTTCGTTCTTTGATTCCGAATCTCCCTCCAACGTCGCCGAATAT
TCGGTGTCGGAATTGTCCGGCTCGATCAAGCGCACGGTCGAGCAGGCCTTCGAGCATGTCCGGGTGCGGGGCG
AGATTTCCGGCTACCGCGGCCCGCATTCCTCGGGACACGCTTATTTCGCCCTGAAGGACGATCGCGCCCGCAT
CGATGCGGTCGTCTGGAAGACCACCTTCGCGCGGCTGAAGTTCCGGCCGGAGGAGGGGATGGAGGTCATCGCG
ACCGGCAGGGTCACGACCTTCCCCGGCTCGTCCAAATATCAGATCGTCATCGACTCGCTTGAGCCGGCCGGTG
CCGGGGCGCTGATGGCGCTTATCGAGGAACGCAAGCGCAAGCTTGCGGCGGAGGGCCTTTTCGATGCGGGCCG
CAAGCGGCCGCTGCCTTTCATGCCGAGGGTCGTCGGCGTCGTGACCTCGCCGACCGGAGCTGTGATCCGCGAC
ATCCTGCACCGCATTGCCGACCGTTTCCCGGTCCATGTCATCGTCTGGCCGGTGCGCGTGCAGGGCGACGGCG
CCTGCGAGGAGATCGTGGCGGCGATCGAGGGTTTCAACGCGCTGCAGCCGGGCGGCTCCATTCCCCGGCCCGA
CGTGCTGATCGTCGCGCGCGGCGGCGGCAGCCTCGAGGATCTCTGGTGCTTCAACGACGAGGCGATGGTGCGC
GCCGCGGCGGCTTCCGCCATCCCTCTGATCTCGGCCGTCGGTCACGAGACGGACTGGACGCTGATCGACCATG
CGGCCGACCAGCGGGCGCCGACGCCGACCGGTGCGGCCGAAATGGCGGTGCCGGTGAAGGCCGATCTCGAGGC
CCAGCTCGCCAGTCTCGCAGCCCGGCTCAAGGGCGCCGCGGCGCGGCAGATGGACAATCGCCGCCAGACGCTG
CGCTCGCTTGCGCGGGCGCTACCCTCGCTCGACCAATTGCTCGCCTTGCCTCGGCGTCGCTTCGACGAGGCGG
CCGCCGGACTCGGCCGCGGCCTGCAGATGAACACCGCCAACAAGCGGCGAAGCTTCGAACGCAATGCGGCCCA
TCTCCGGCCGGAGCTCCTGACGGCAAGGATCGTCGATCGCCGTCAGCGCGTCTTGGATGCCGTCAACCGGGCC
GAGCGCATCGTCGAGCGTAAGGTTCAACGTGGCGCTCAGCGCATTTCGTCTGCCGATGCCTCGCTCCGCGTTC
TGCCGTCGCGGCTGATCGGCCAGATTCATCGCGCATCGGATCGCGTTTCCAGCCTGGGCAGGCGTGGGGATGC
GGCGATCGCCGCCGATCTGCGCAGGATGAAAAGCGCGCTTGCAGCACAGGACCGCGTGCTGCAGTCGCTCTCC
TATCACAGCGTCCTGCAGCGCGGCTTCGCGCTGGTGCGCGATGCCGCGGGCGAGCCGGTTAAACAGGCGGCTG
CCGTGCATCCCGGGATGGCGCTTTCGCTGGAATTCGCCGATGGCCGCATCGCGGCGGTCGCCGGCGAGGACGG
CACTGCGCCGCAGTCGCCGAAGAAGCGGCCTGCACGCTCCGGCGAGCCGACGAAGCAGGGCAGCCTCTTCTGA
ATGAATTTCTCACGCTTCTTCGTCGACCGCCCGGTCTTCGCGGGCGTTCTTTCCGTGATCATCTTCGTCGCGG
GCCTGATCGGCATGACCGGCCTGCCGATTTCCGAATATCCGGAAGTCGTGCCGCCGCAGATCGTCGTCAGGGC
GCAATATCCGGGCGCCAACCCGGCCGTCATCGCCGAAACCGTCGCCACACCGCTCGAAGAGCAGATCAACGGC
GTCGAGGGCATGCTCTACATGCAGAGCCAGGCGACCGCCGATGGCCTGATGACGCTGACCGTCACCTTCGAGC
TCGGAACCGATCCGGATCAGGCCCAGCAGCTGGTGCAGAACCGCGTCTCGCAGGGCGAGCCGCGGCTGCCGGA
AGAGGTGCGCCGGCTCGGCGTCACCACCGTCAAGAGCTCGCCCGACCTGACGCTCGTCGTGCACCTGATTTCA
CCCAACGGGCAATACGACATCAACTATCTGCGCAACTACGGCGTCCTGAACGTCAAGGACCGGCTGGCGCGCG
TAGATGGTGTCGGCCAGGTGCAGATCTTCGGCGGCGGCGACTATTCCATGCGCGTCTGGATCGACCCCGAAAA
GGCCGCAGAGCGCGGGCTTGCCGCGAGCGACATCGCCAATGCCGTCCGCGGGCAGAACGTCCAGGCAGCCGCC
GGCGTCATCGGCGCTTCGCCGTCGGTTCCCGGCCTGGACCTGCAACTCTCCGTCAACGCTCAGGGCCGCCTCA
AGACACCCGAGGATTTTGCCGACATCGTCGTCAAATCGGGCGAGAGCGGGGAGATCACGCGCCTGGGCGACGT
CGCCCGCGTCGAAATGGGAGCCGCTGACTACTCGCTGCGCTCGCTTCTCGACAACAAGGCCGCCGTCGGCATG
GGCGTGTTCCAGGCGCCCGGCTCGAACGCCATCCAGATCTCCGAAAACGTCCACAAGGTCATGGCCGAGCTGA
AGCAGACCATGCCTGATGGCGTCGATTACGAGATCGTCTACGACACGACGCAGTTCGTCCGGGCGTCGATCGA
ATCGGTCATCCACACGCTGCTCGAAGCCATCGCGCTCGTGGTCGTCGTCGTCATCGTCTTCCTGCAGACCTGG
CGCGCCTCGATCATCCCGCTCGTCGCCGTGCCCGTCTCGATCGTCGGTACCTTCGCCGTGATGTATGTCTTCG
GCTTCTCGATCAACGCGCTCAGCCTCTTCGGTCTGGTGCTGGCGATCGGCATCGTCGTCGACGACGCCATCGT
CGTCGTGGAGAACGTCGAACGCAATATCTCCCAGGGCCTCTCACCGGTGCAGGCGACCTATCGCGCCATGCAG
GAGGTCTCTGGGCCGATCATCGCGATCGCGCTGGTCCTCGTCGCGGTCTTCGTGCCGCTCGCCTTCATCACCG
GGCTCACCGGCCAGTTCTACCGCCAGTTCGCGCTGACCATCGCGATTTCGACGGTGATTTCGGCCCTCAACTC
GCTGACGCTCTCGCCGGCGCTCGCCGCCCTTCTCCTGAAGGACCACCACGCCCCGAAGGACCGGCTGACGCGG
GTCATGGATGGGTTGTTCGGCTGGTTCTTCCGCGGCTTCAACCGCTTCTTCGGAGCGAGTTCGGAGGCCTATG
GCCGCGGCGTCGGCGGCATCCTCACCCGCAAGTCGCTGGTCATGGGCGTCTATGTCGTCCTCCTCGGCGTCAC
CTTCGTGCTCTTCCGCGCGGTGCCCGGCGGCTTCGTGCCGGCGCAGGACAAGCAATACCTCATCGGCTTTGCG
CAGCTGCCGGACGCGGCGACGCTCGATCGCTCGGAAGATGTCATCCGGCGCATGAGCGAGATCGCCCTCAAGC
ACCCGGGCGTCGAACACGCCATCGCCTTCCCGGGCCTGTCGATCAACGGCTTCACCAATTCGTCCAACTCCGG
CATCGTCTTCGTGTCGCTGAAGCCGTTCGAGGAGCGTACGACGCCCGAACTTTCGGGCGGCGCCATCGCCATG
CAGTTGAACCAGGAGTTCGGGGCGATACAGGATGCCTTCATCGCCATGTTCCCGCCACCGCCCGTCCAGGGTC
TCGGCACGACCGGCGGCTTCAAGCTGCAGATCGAGGACAGGAACGGCCTCGGCTACCGGACGCTGGACGATGC
GACCAAGGCCGTCCTCGCCAAGGCGATGGCGGCACCGGAACTCGCCGGGCTCTATTCGAGCTTCCAGATCAAC
GTGCCGCAGCTCTATGCCGACCTCGACCGCACCAAGGCCCGTCAGCTCGGCGTCGCGGTCACGGACGTGTTCG
AGACGCTGCAGATCTATCTTGGCTCGCTCTACGTCAACGACTTCAACGCTTTCGGCCGCACCTACAGCGTGCG
CATCCAGGCCGATGCGAGCTATCGCAGCCACGCCGACGACATCGGCAAGCTGAAGGTGCGTTCGCAGACGGGC
GAGATGATCCCGCTGTCGGCCCTGCTCAACGTCGAGCAGACGGTCGGCGCCGAGCGGGCCATCCGCTACAACG
GCTTCCTCGCCGCCGATATCAATGGCGGACCGGCACCCGGCTTCTCTTCCGGTCAGGCGCAGGCGGCCATCGA

GCGGATTGCGGCCGAGACTCTGCCGCCCGGCATTTCATACGAGTGGACGGACCTGACCTACCAGCAAATCCTC
GCCGGCAATTCTGGCATCCTCGTCTTCCCGCTGGCGCTGCTGCTCGTCTATCTCGTGCTTGCGGCCCAGTATG
AGAGCCTGCTGCTGCCGATCGCGATCATCCTGATCGTGCCGATGGGCATCATGGCGGCGCTGACGGGCGTCTG
GCTGACCGGCGGCGACAACAACGTCTTCACCCAGATCGGCCTAATCGTTCTGGTGGGTCTGTCGGCAAAGAAC
GCTATCCTGATCGTCGAATTCGCCCGCGAACTGGAACTGTCCGGCAGCAATGCCGTCAGTGCGGCGATCGAGG
CGAGCCGGCTCCGGCTCAGGCCGATCCTGATGACCTCGATGGCCTTCATCATGGGTGTCGTGCCGCTCGTCAC
CTCCACCGGCGCCGGCGCGGAAATGCGCTCCGCCATGGGCGTGGCGGTCTTTGCCGGCATGATCGGCGTCACG
GCCTTCGGCATCTTCATGACGCCCGTCTTCTATGTGCTCATCCGCAAGCTTTCCGGCGAAAAGCCGCTGAAGC
ACACCGGAGCGCATATAGAAGCCCCGCATCTCGCCCCCGGCGAGTAGATGTCACTGACAGATGGCCCCTATGC
GGGGCCGCCCCAGCCGGATGTGGTCGACCCGAGCGGCGGGGCCAATCAAACGCGCGTGCGCGCCTATAACGAA
CGGCTGGTCATGTCGCTGGTGCGCCGTCACGGCAGCTTGTCCAAGGCCGAGATCGCGCGCCGATCCGGTCTAT
CGGCGCAGACCGCCTCCGTCATCATGCGGTCGCTCGAAGCCGACGGGCTGCTCGTCCGCGGCGCGCCGGTGCG
CGGCCGGGTTGGACAGCCCTCCATCCCCATGCGGCTCAATCCGGATGCGGTCTATTCCTTCGGCGTGAAGATC
GGCCGGCGCAGCGCCGATCTGGTGCTGATGGATTTTCTCGGCACCATCCGGCTGCACCTGCATCAGATCCATA
CCTATCCGCTGCCCGAGGAGATCGTCAGCTTCATCGTCGGGGGCATCGGCAAGCTCGAGGGACAGCTTTGCCC
CGGCGAGCGCGGCCGCATTGCCGGCGTCGGCATCGCCACGCCGTTCGAACTGTGGAACTGGTCGGAGGAAGTC
GGCGCGCCGCGCGAAGAGATGGACAGGTGGCGCGATTTCGACCTGCAGGCGGCGGTCGCCTCGCGCATCCGGC
ATCCGGTTTTCCTTCAGAACGACGGAACCAGCGCCTGCGGTGCCGAACTCGCCTTTGGTGTGGGCGCCAGTTA
TCCCGATTTCGTCTATTTCTATATCGGCTCCTTTATCGGCGGCGGCGTCGTGATCAACTCCGCCCTCTTTTCC
GGACGAACCGGCACCGCCGGCGCCGTCGGCCCGCTGCCTGTCGCCGGCAAGGACGGCAAGTCGACGCAGTTGC
TGAAAATCGCCTCGGTCTTCGTCCTGGAAAAGCTCCTGCGCGAACGCGGCATCGACCCGGAGCCGCTCTGGTA
CTCCGCCGACGACTGGATCGATTTCGGCGAACCGCTCGAAATATGGATTCAGGATGCGGCGGCAGCGCTTGCG
CAAGCCGTCGTCTCCGCGGTTTCCGTCGTCGATTTTTCGGCGGCCGTGATCGACGGCGGCTTTCCGCCCTGGG
TGCGTGCGCGCCTTCTTGCCGCGACGCGAAAGGCCCTCCAGACGCTCGACCTGCAGGGCGTCACGGTTCCGGA
CCTCGTCGAAGGCACCGTCGGCAGCCACGCCCGCGCGATCGGCGGCGCAAGCCTGCCGCTCTTCTCCCGCTAT
CTGCTGGACACCAATGTCCTCTTCAAGGAGCTTTAATTGAGGAACGAGGATATCGTGTTGGATGGCGCGAAGG
TGTTCTGGCGCACCCTGCTGCCGAGGTTGAAGGCGGAGCGCGGGCTTCTCGGCCTGTCCGTCGTGCTCGCCTT
CCTCGCCGCACTCGCGGGCATCCATCCGCTGGCCGTCCTGCCTTTCTGGATCGTGCTCGTCGCGGCCATCCTC
AACCGGGGAGCGCCGCCCGTCCAGACAGCGCCGGCGAGCGTCGGCGCTGCTGTGGAAACCAAGGCCGACCCGC
TTGCTCCCCTGCTCGACGCGCTCGACATGCCGGTGCTCGTCGTGGCGGCCGACGAGACCGTCGTCTTCCAGAA
CGCCGCCGCAGAGAAGGCCTTCGGAACTATCCCCCAGGACAGCTACCTTTCCGCCCGCGTCCGTTCGCCCGGC
ATTCTCGACATGGTGCGCGAGACGATCGCGACAGGCAAGGTCAACCAGATCGAGCATGCGGAAAGGCTGCCTT
CGGAGGCGGTCTACGTGGTTCGCGTCGCGCCCGTGGACATCGGCTCCGACGTTTCCGGCCGGCGCATCTTTCT
GCTGACCTATCGCGATATCTCGCAGGCTCGCCGCATCGACCGGATGCGCTCCGATTTCGTCGCCAATGCCAGC
CATGAGCTGCGCACGCCGCTCGCATCGCTGCGCGGGTTCATCGAGACGCTGCAAGGCCCTGCCCGCGACGACC
GGAAGGCCCAGGAGAAATTCTTCGGCATCATGCACGAGCAGGTCACGCGCATGAGCCGGCTCGTGGACGACCT
CCTGTCGCTGTCGCGCCTCGAGTTGAAGTCGCACATCGCGCCGGACGATGCCGTCGATCTCGGGCCGCTGCTC
GGACACGTGCGCGACAGCCTGGCGCCGCTTGCGGGCGAACTCGACGTGACGATCGCGCTGGAGGTTCCGGAGA
CTCCGGTTTTCGTCCAGGGGGATCGGGACGAATTGGTGGAGGTCTTCGAAAACCTGATCGAAAACGCCTGCAA
ATACGGGCAGGAAGGCAAGCAGGTGGATGTGCGGCTGACCGGCGGGAATGGTCACCAGGCGGAGGTGACGGTT
ACCGACCACGGTCCGGGAATACCTGCCGAGCATGTCCCGCGCATCACCGAGCGTTTCTACCGGGTCAATGTCG
AGGCGAGCCGGTCGAAGAAAGGCACCGGCCTGGGCCTTGCGATCGTCAAGCATATCCTCACGCGTCACCGCGC
CCGCCTGCTCATCCATTCGGAGGTGGGGAAGGGCACGGTCTTCAAGGTCCGCTTCTGAATGGAGCAGAACCTA
TCCACGATACGCAAAGCCGCGGGTAGCGAGGGAGAGCCGACCATCGCGCTTGCGCTCGGCGGAGGCGGTGCGC
GGGGACTTGCCCACATCCATGTCATCGAGGCGCTCGATGAGATGGGGCTCCGCCCCGCAGTCGTGGCCGGTTC
GTCGATCGGCGCCCTCATGGGCGCGGGCGTGGCCGCTGGCATGAGCGGGAAGGAAATCCGCGAACACGTGCTT
TCGACCGTCGGCCGGCGTGGGGAGGTGCTCAACCGCCTATGGCGACTGAGGCCGACCAGTCTTGCCGAGGCTG
TGGCGAGCGGTTTCCGCGTCGGCCAGTTCAATATAGAGCGCGTGCTGAAGGCCTTCCTGCCCGAGGCGGTGCC
TCTCCGCTTCTCCGATCTGGCACTCCCCCTTAAAATCATCGTCACGGACTATTACGGGCAGACGGAGCGGGTA
TGCGAAAGCGGCGACCTCTACAAGGCGCTCGCTGCCTCCTGCGCCCTGCCGGCGGTCTTCATGCCGGTGAAGA
TCGACGGCCGCGTGATGATCGACGGCGGCATCTATAATCCGATCCCCTTCGATCACCTGCGCGGACTGGCAGA
TATCGTCGTCGCCGTCGATGTCGTCGGCGGGCCGGACGGTGACGGCGAGACGATCCCGAGCCGTATCGACAGC
CTGTTCGGCGCGACCCAGTTGATGATGCAGTCGATCATCACCATGAAGATGCAGGCCGGCGCACCGGATATTC
TCCTGCGTCCCGATGTCGGACGGTTCCGCGTGCTCGATTTCCTGCGCGCCCAGGAACTGCTTGCCGCCACCAG
CGGGACCAAGGACGAGGTGAAGCGCGCGCTGGCAGAGCGCATCGATCGGTGGAGGAAAGGGATATAAATGGTT
TCAGAAGCGCCCCCGTTCTGGTGGACCAAGGCCGACTGGCGCGCCTACGCTCTTTGGCCCTTTTCCTGGGTGT
ACGGCCGGATCGCCGGGATGCGCATGGACCGGGCGCGGCGGGCGACCTCCGCCGTACCGCTGATCTGCATCGG
CAACTTCACCGTCGGCGGAGCCGGAAAGACGCCGACGGCGATCGCGATTGCGCGGGCCGCCAGGGCACGCGGC

CTGAAGCCCGCTTTCCTGAGCCGCGGTTATGGCGGGTCGCTGGACGTCACGACCGTGGTCGACCCCGAGCACC
ATCGCGCGCGCGATGTCGGCGACGAGCCGCTGCTGCTTGCCCGCGAGGCGCTGACCGTGATCTGCCGCCGCAG
AGTGGATGGCGCACGCAAGCTTGCCGCGGAAGGCGCTGACATCATCATCATGGATGACGGATTCCAGAGTGCC
CGGCTCGTCTTCGATTTCGCCCTTCTCGTGGTCGATTCCGGCCGCGGTATCGGCAACGGCCATCTCGTGCCTT
CCGGCCCCGTTCGGGCGCCGATCGGCAATCAGCTGCGCCATGCCAACGCTCTGCTGAAACTCGGCCACGGTTC
CGCCGCCGATCCTCTCGTCCGCCGGGCGGCTCGGGCCGGCAAGCCCGTCTATGTGGCCGAAACGGTGCGCACG
GATGCAGGGTCGCTCGACGGCGTCAAGGTCCTTGCCTGGGCCGGAATTGCCGATCCGGAGAAGTTCTTCAAGA
CCGTTCGCGAGACCGGCGCGGTCATCGAAGAGACCCGGAGCTTCCCGGATCACCATCATTTCTCCGAGGACGA
GATTGCCGACCTAATCGACCGCGCCGCAAGCCGGGGCTACACGCTGGTGACCACGGCCAAGGACATGGTCCGT
CTGGAGCCCGGCCACGGGCGGGCAGGGGAACTGGCGGCAAAGAGCCGCGTGATCGAGATCGAAGTGCGCTTCG
ACGATCCGGCAGCGCCGGGAAAGATCATCGACGCGGCACTTGCCTCCGCCCGTGCCCGCCGATTGCGCGAGCG
TAAGGCGGGCTAGTTGGCAAGAAAAACGTCCCGCAGCATCCGATATGCCGACCTGAACGCGGACGACAATGCG
GAGACGCGTCGGCTGATCGATGCCGGCAGCCGCCTTGCCGAGGAGGTCGCGCGTCTCTTTCCGCCCGAGCCCG
ACATGGCCGACCGCCACTACTGGCTCGAAGCGATGATCAACCACGTTCCCGATTATATCTATGCGAAGGATAC
GGCGGGGCGCTTCCTGATCGCCAACGAGGTGACGGTTGCCGACAACGGCCTCGAAAGCCTGCGCGACCTCGTC
GGCAAGACCGATTTCGATCTGCACCCGCACGAGTTCGCCCAGGCCGTCGCCGATACCGAGCGACGCGTTTTGG
AAACCGGCGAACCGATCTTCGGCATCGAGGAACGCGCCATCGTGACCAAGGACCGCGATCGCTGGCTGATGAC
CTCGAAAGTGCCCTTGCGCAGCAAGAGCGGGAGGATCATCGGTATCGTCGGCATCTCGCGCGATATCAGCGAT
CGCAAGGCCGCGGAGCGCCTGCTCGAAGGTCAGGCCCGGCTGCTGGAAATGATCGCCAACGGCAAGCCCCTTG
GCGAGTTCTTCCACGAGCTGGTGCTGCTGATCGAAGCGCTGCTGCCCGGGATCAAGGGCTCCATCCTGCTCCT
TTCGGAGGATGGCCGGCATCTCCTGCACGGCGCTGCCCCCAGCCTCGACGCGGGCTTTTGCGCTGCCATCCAT
GGCCTCGAGATCGGCCCTGCGGCCGGCTCCTGCGGAACGGCCGCCTGGTGCGGCGAACAGGTGATCGTTTCCG
ACGTCTTCGCCGACCCGCTCTGGGAGGAATACGCGCCTCTCCTGAGGCCTTCCGGCCTCCGTTCCTGCTGGTC
CACGCCCATCCTGTCGCGCGAGCGAAAGGTGCTCGGGACATTCGCGCTCTATGCGCAGGAGGTCGGAACGCCG
AGCGAGCAGCAGCAGGAACTGATTGCCATGGCCGCTCATCTCGCCGGAATAGCAATCGAGCGCAAGCGGGCGG
AGGACCGCATCGGTTTCATGGCCCATCACGATGCCTTGACGGGGCTGCCGAACCGCGTGCTTTTCGAGGAGCA
GGTCGCCGGCATGCTCGAGGAGATCCGCGAGCGGGATCAATGGGCCGTGCTCGCCTTTCTCGACCTCGACGAT
TTCAAACTGATCAATGACAGCCTTGGGCACGCCACCGGTGACGAGCTCCTGAAGATCGTAGCCGGGCGCATGC
GGGCCGCCGTCCGGAGATCCGATTCCGTCGTGCGCGTCGGCGGCGACGAGTTCATCATCCTCCTGAACGGCCT
GCCGAAGGAGCGGGACGTCGTGCTATCGCGGCTGGAGGATATCCGCACTGCAATCGCCATGCCGGTGCAGTTG
CAGGGCCGCTCTCTGCAGGTGAGCTGCAGCATGGGCGTGGCCTGCTTCCCCAACCAGGGCAAGACGGCGGGCG
AACTGCTCGCCAATGCCGACATGGCCATGTACCGCGCCAAGGAGCTCGGCCGCAACAACATCCAGGTCTTCAC
CGAGGAGATGGCCGCCAGAGCCCATGAAAAACTCCAGTCGCAAGAGGAGCTTCGCGAGGCGCTTGCGCGGGAG
GAGTTCCTGCTGCATTTCCAGCCGCAGATGAGCCTCGAGACCGGCCGCATCTTCGCTGCGGAGGCGCTGTTGC
GCTGGCAGCACCCGGTGCGCGGCATCATCTCGCCTGCGGCATTCATCCCGCTCGCCGAGGAAACCGGTCTGAT
CGTGCCGATCGGAGATTGGGTCCTGCGCACCGCCTGCCGCCAGTTGAAGGCCTGGCAGGACGCCGGCCTGCCG
CCGCTGATCGTCAGCGTCAATGTTTCCGCGCGGCAATTCCGCGAGCGCAACTGGGCAGCGCGTGTGGCGGCGG
TTTTGGAGGAAACCTGCCTCGAGGCCCGTTACCTCGAACTCGAGCTCACCGAAAGTCTGATCATGCAGGACGT
GCCGGGAGCGATCGCCACAATGCATGCGCTGGAGGCGATCGGCGTGCACCTTGCCATCGACGATTTCGGGACA
GGCTATTCGAGCCTCAGCGCGCTCAAGCGCTTCCCGGTGCGCCGGTTGAAGATCGATCGGTCCTTCGTGACGG
ACATTCCGCACGATGCCGACGACATGGCGATTACTTCCGCGATCATCTCGCTTGCGCAGAAGCTCGGTCTGCG
CGTCATTGCCGAGGGTGTCGAGACGGAGGCCCAGGTCGAGTTTCTGCAAAAGAGCGGCTGCGGCGAGATCCAG
GGCTACTTCTTCAGCCAGCCCTTGCCGGGCGAGGATTTCGAAGTGCTGCTTTCCTCTGAGCGGCGGTGAATGG
ATGCCGCGGCCCTGACATTCCTGGCCCTCGGTCTTCCCTTTGCCGCGGCCGCCGTCGCACCGGCCCTGACCCG
GCTTCTCGGCGGGAATGCCGCCTGGCTGCTGGCGCTTGCGCCACTCGCCATCTTCATGCATCTCGCAGGTTTC
ATTCCGGAAGTCGCCGCTGGCGAGATCGTTACCGGCGGCTATATCTGGATACCGTCCTTCAACGTCAGCTTCT
CCTGGCTGATCGACGGCCTGTCACTGACCTTCGCGCTTCTGATTTCGGGCATCGGGACACTGATCGTCCTCTA
TTCGGGCGGATATCTGAAGGGGCATCCCGACCAGGGCCGCTTCTTTTCGTTCATCCTGATGTTCATGGGCTCG
ATGCAGGGGCTCGTCGTCTCGGACAGCTTCCTGATGCTCTTCGTCTTCTGGGAGCTGACGTCGATCACTTCGT
TCCTGCTGATCGGCTTCGATCACACCCGCGAGGCGGCGCGCCGCGCGGCTTTGCAGGCCCTGGTCGTGACGGG
GGGAGGCGGGCTTGCCTTGCTCGCCGGGCTCCTCATCCTGTGGAACGTCAGCGGCGTCACGCAATTTTCGCTG
CTCCTGTCCTTCGGCGACGAACTGAAGCAAAGCCCTTTCTATCTCGCGGCGCTGCTCCTCGTCCTCGGCGGCG
CATTCACCAAGTCGGCACAGTTTCCGTTTCACTTCTGGCTGCCGAACGCCATGGAAGCGCCGACGCCCGTTTC
GGCCTATCTGCACTCGGCGACGATGGTGAAGGCGGGGGTCTATCTTCTGATGCGGCTCAACCCGGTGCTCGGC
GGTACGACGGAGTGGCAGGTTCTGCTGCCGCTCTTCGGCGTGACGACGCTCGTCGCCGGAGCCGCCCTGGCAG
TGCGCCAGACGGACCTGAAGATGATGCTGGCCTATACGACCATGTCGTCGCTCGGTCTTCTGGTCATGCTGAC
GGGCCTCGGCCTGCCCTACGCCATCGAAGCCGCAGTGCTTTACTTGGTGGCGCATGCCCTGTTCAAGGGGGCG
CTGTTCATGGTGGCCGGCATCATCGATCATGAAACCGGTACGCGCGATCTGACGAAGCTCGGCGGCCTGCGCA

GGGCGATGCCTCTGACCTTCGCCATAGCTCTTGCGGCGGCCCTGTCGATGGGCGGCCTGCCGCCCTTTTTCGG
GTTCCTTGCAAAAGAGGAAATCTACGCCGCACTCTCCGGTTTCGACCGCCGGTCCATGGTCTTCGCGGCATTG
ACCATCCTCGGCAACGGCCTGATGTTCGCCGTCGGCTTCGCGATCGCTCTAAAACCGTTTCTGGGCCCGAAGG
TCGATACGCCGAGGCATGCCCACGAGGCACCGGTGCTCCTCTGGATCGGCCCGGCAATCCTGGCTGCGAAGGG
CCTTTCCATCGCGCTGATGTCGGGCCTTGTTCATCGGTTCGTGACCTCGCCCATGGCCTCGGCCGTTGCCGGC
GAACCGAGAACCGTGACGATTTCTCTCGCACCGCATATCGGCGTGCCGCTGTTGCTCTCCGTCGTGACGCTGG
CGGTCGGCGTCGTTCTGTTCCTCAATCTGACGCGGCTGCGCGCAGCCATGGCGGTGATCCTTGCCGATATCGG
CTGGGGACCGGATCGAGGCTTCGACCAGTTCATACGCGGCCTCGTGCGGTTTGCGGTCGCCGCGACCCGCCGA
TTGCAGGGCGGCCGTCTCGATAACTACATGACCGTGACCTTCCTGCTCGTCGCCGCCGTCATGCTCGCATTGC
CGCTGCGCTACGGCGAATTCCCGCGCGCACCTTTCTTTCCGGGCGACGTTCCGCTGCACGAATTCGCGATCAT
GGCGATCGCCGTGCTTGGCCTCTTCGCCGTGGTGCTGGCCGCGGACCGGCTGACGGCGATCGTCTCGCTCGGA
ATTCAGGGCTTCGCCGTCGCGATGCTCTTCCTGCTTTACGGCGCTCCGGATCTCGCCTTCACCCAGTTCATGA
TAGAGACGCTGTCCGTCGTCGTGCTGGCACTGGTGATGACCCGGCTGCGGCTGTCTCCTTCGGATCACCGACC
ACTCAGGCAGAAGCTTCCCGACTTCACGATCGCGCTCGCCTGCGGAATCGGATTCGGCCTTTTCCTTCTCCGG
GTTACCGGCGTGCCGTTCGATGCGACGCTGACGGACTTCTTCAACCTCTATTCGAAATCGATCGCGCACGGCG
CGAACGTGGTCAACGTCATCATCGTCGACTTCCGAGGCACGGATACGCTTGGCGAAATTGCCGTCGTGCTGGT
TGCGGGCCTGTCCATCCTTTCCCTCGTGCGGTTGCGGGCGGGATCGTTGAGACGCGGCGAAACGCGCGAGATC
GCGGCCGCTGATGCGAACACGAAGGAGCCGGTATGAGTGGCCCGAGGTCAAGGCAAGGTTATTCCAGTCAGCA
GGGTTCAGCTCGTTCAGCCACAGACCAACGGGAGGATCATACGCATCCTGGTCACAGAGGGGCAGCCGGTGAA
GGCTGGTGACTTGTTAGTCGGCATGGATACCGTCTCGGTTGAGAGCCAGATCAAGCTCAGGCTGACCGAGGTT
CGTCGCCAACGTCGCGGGGCGACGGTTGCCCGAGTAATCATTGCGGCGCTGACAAATGTTGCAGTCGCAAACG
ATGATGTGCTTGCGGCAGGCAGAGCTGCCTTCGAAAGGGAGCGGCATGACCTGGAGGTTATCGACGGAGCGGA
GGCACTTATAGAGGCTGTGCTAACGTCTCTTCGAGATCAGGTTGCGCGGATCAATGCGCAACTTGATCGTGTC
GAACAATCGCGAATAACCCAGCAGGCGCGACTGCAGAAAGCTCGGTCTGATCTGGATATTTCCTCCAGGAAAT
TTTTGTCAGCCGGCAAGTTGAAGGACAAGGGAGCCATCAGCGAAGTAGACTACTTCGAGCGATTGCGTGAGCA
AAGAGCACGCGAACTGGAGGTGCTCATCTCCGAGAGCGAATTGAATATCCTTGCAGCGGAAGCGAACGCGTTG
GCTAGACAGCGGCAGAGCATCATTTCGACCGCAGTCGAGAACTATCAGAAACAACTGAACGAGGCGAGCACGT
CCCTTGCAGGGCTGGAAGCGGAACTGAGCGCGGCTAGGAACCAACTGGCCTTGCTCGCGATTCGCGCACCAGC
AGATGGCAAGGTCGAGAACCTCACGGTATTTACCATCGGCGGTTTCGTGGAAGCAGGTTCAACACTCATGTCC
ATCGTACCTTCCGGCGATGGCATGGAGATTGAAGCTTTCTTCGACAACCGTGACATCGGCTTTCTTGAAAAAG
GGCAAGAGGCATTTGTGAAGTTTGACGCCTTTCCGGCCGAACGTTTCGGTATTGTTCGCGGTCGGGTGACTAG
AGTTGGTGCGGATGCGCGCGGCGACATCGTCCCAGGGAAATGGGTCTACGCAATCCACCTTGTACTCGACCAA
GAGACAATTGGGATAGGCGGGCGCGACATAGGGTTCTCGCCCGGCATGACTGCGACGATCGATGTGATAACGG
GTGAACGGCGGCTCATTAGTTACTTCTTCGAGCCAATAATCAAGGCAATTCAAGACGGATTAGGGGAACGTTG
AATGGCCTATTTTCTGCAGCAGATCGCCAATGCCGTACCCGTGGCCGCGCTTTATGCGGCGCTCGCCTTCGGA
TACGCGATCGCCTTTGCTGTCACACGGCGGGCGGACCTGACCTATGGCGCGCTCTTCGCCTTTGCCGGGCAAA
TCTTCGTGCTCTTCGCGGATGTCGGCTGGAACCGGCTGTGGCTGGTACTGCCGGCGGCGCTCGGGCTCGGGGC
GACAGCGGCGCTCGCATTCGGGCTAGGTGCCGGTCTTATTGCCGGCCGCTACGTCATGCGGCCCCTGGCTTTT
TCCTCCGCCAATGCCGTGGTCGTCGCCTCGCTCGGAAGCCTGCTGGTGCTGATGGAAACGGCCCGGCTCGCCT
CCGACACCAGAAGCCTCTGGCTGCCGCCCTTCCTGAACGACGTGGTGGTGTTCTGGCCGAACCCTGCCTTTCC
GGTCGCGCTGACGGTCGTGCAGCTCGTCAACACCGCGCTCATGATGGCCCTTGTCGCCGGCGGCCATTGGCTG
TTGACCCGTTCCTATGCAGGCCGCTACTGGCGCGCCGTTTCCGAGGATCGCAAGGCCGCCGCGCTTTGCGGCA
TCGATCCGGGAGCGGTCTATATCGCCGCCTATGGCGTGGCGTCGCTGATCGCTGCGTTCTGCGGGATTCTCGC
TGCTTCCTATTACGGCAACATGGATTTCGGCACCGGGCTGACCTTCGGGGTCAAGGTCCTGTTCATCGCCGCC
ATCGGCGGGCAGACTTCGCCGTTATTCGCGGCACTGGGCGCAGCCGGCATCGGCCTCATGGAGACGGTGTGGA
GCGCCTATGGACCCATCCTCTGGCGGGACTTCGCCATATTCGGCTTTCTGGTGATCGTGCTCGTGGTCACGCG
CCGGGAAAAGATCATTCCCTGAATGAACGACAGGACAAATCTTGTGGACAGGATCACCGGACACCGCCGCATG
CGCCGCAATCGCAAGGCGGATTGGACGCGCCGGCTGGTCCGGGAGAACCGCCTGACGGTGGACGACCTGATCT
GGCCGATCTTCGTCGTGCCCGGCAGCGGCATCGTCGAGCCGATCGATGCGATGCCGGGCGTCAACCGCATGAG
CGTCGACAAGGCGGTCGAGGCCGCGAAGGAGGCGGCCGGCCTCGGCATCCCGGCCATCGCCACCTTCCCCAAT
ATCGACATGGCGCTTCGCGACGAGACCGGATCCGACAGCCTCAACGCCGACAATCTGATCAATCAGGCGACGC
GGGCGATCAAGAAGGCCGTACCCGACATCGGGATCATCACCGATGTCGCGCTCGACCCGTTCACCAGCCACGG
GCACGACGGCATCCTGCGGGGCGGCGAGATCGTCAACGACGAGACGGTGGAGGCGATCGCCAGGGCGGCGGTC
ATTCAGGCCGACGCCGGCTCCGACATCATCGCGCCCTCCGACATGATGGACGGGCGGATCGGCGCGATCCGCG
AGGCGCTCGACGCGGCCGGCCACCAGAATGTCGGCATCATGTCCTATGCGACGAAATTCGCCTCCGCCTTCTA
TGGCCCTTATCGAGAGGCGATCGGCACCGGCGGCCTGCTCAAGGGCGACAAGAAGACCTATTACATCGATCCG
TCGAACGGTACGGAGGCGATCCGCGATGCGGCACTCGATGTGGAGGAAGGCGCGGACATGCTGATGGTGAAGC
CCGGCCTGCCCTATCTCGATATCTGCTGGCGGATGAAGGAAGCCTTCGGCCTGCCGGTCTTCGCGTATCAGGT

TTCCGGCGAATACGCGCAGATCAAGGCAGCCGCCGCCAATGGCTGGATCGATGGCGAGCGGGTGATGCTGGAG
ACGCTGCTCGCCTTCAAGCGGGCGGGTTGCGACGGGATCCTCAGCTACTTTGCCGTCGACGTGGCTCGAATTC
TCGCGAAACGCTGAATGTTTACAGGCATCATCACCGACATCGGTACGGTCGAGAAGGTCACACCTCTCGAGGA
AGGCATCAAGCTTCGCGTCGCCACGAATTACGACCCGCAGACGATCGATCTGGGTGCCTCGGTCGCGCATGCC
GGCATCTGCCTGACCGTCACGTCACTTCCGGAAGCCGGCAGCAACGAACGCTGGTTCGAGGTCGAGGCATGGG
AGGAAGCGCTGAGGCTGACGACGATCGCCGACTGGCGTGAGGGCACGCGCATCAATCTTGAGCGCTCGCTGAA
GATCGGCGACGAACTGGGCGGTCACATCGTCTCCGGCCATGTCGACGGCAAAGCGGAGATCCTTGCCGTCGAA
GCGGAGGGGGAAGCCGTGCGCTTCCGCCTCAGGGCACCCGAACATCTCGCGAAATTCATCGCGCCCAAGGGCT
CGGTGGCGCTCGACGGGACGTCGCTCACCGTGAACAAGGTGGATGGCGTCGAATTCGACGTATTGCTGATCCG
ACATTCGCTCGAAGTGACGACCTGGGGCGAACGCAAGTCCGGCGATCTCGTCAATTTCGAGGTGGACACGATG
GCGCGCTACGCCGCGCGGTTGGCGGAATTCCCGGCTCCCAAGCAGGAATAAATGACGCTCAAGATGAAGATGA
TTGCCGCAGCATCCCTGATCGCGCTTGCTGCCGGCGTCGCCCTGCCCGGGCCGGCGGCGGCGCTCGATGCCAA
GCAGAAGGAGGAATTCGGCGCCTTCATCAAGGAGTACCTGCTCGCCAATCCGGAGATCATGCTGGAAGTGCAG
GAAGCGCTCACCTCCAAGCAGCGGGCCAAGCAGCAGGAAACCGCCCAGGCGGCAATCGCCGACAACAAGAAGG
CGATCTTCAACTCTGACTACGACATAGCCCTCGGCAATCCGGATGGCGATGTGACGATCGTCGAGTTCTTCGA
TTACAACTGCGGCTATTGCAAACGGGCCATGTCCGACATGGACGACATCCTCGCCAAGGACAAGAATGTCCGA
TTCGTTCTCAAGGAGCTCCCAATTCTCGGACCGGATTCGCTCGCGGCCCACAAGGTCAGCGCCGCCTTCCGCG
TGATCGCACCGGAGAAATACGGCGATTTCCATCGCGCCCTGCTCGGGGCAGAGGAACGGGCGACGGAGGAAAC
CGCGATCGCCGTTGCGGCCAAGCTCGGGGTGACGGAAGCGCAGTTGCGCGAAAAGATGGATGACGGGCCGTAT
GATGCCTCGGTGCGCGAAGCCTATACGCTCGCCAACGAGCTCGGCATCACGGGAACGCCTTCCTATGTCGTCG
GCAACGAGGCGGTCTTCGGCGCTGTCGGGGCGCCTGAGATCGAACAGAAAGTCGCCAACATGCGTGCGTGCGG
CAAGACGGTCTGCTGAATGTCCGAACACAGCGCGACGACGACACCTGAAGCGCAAATGCGCACGGAGGCGACC
GGAACCAACCGGATGGTTCTCCTGGATGCGCTGCGCGGCCTGGCACTCGTCGCCATGGCCCTCTATCACTTTG
TCTGGGACCTCGAATTCTTCGGCTATGTCGCGGCCGGCACCGCCGGGACCGGCGGCTGGCGGATGTTTGCACG
CTTGATTGCCAGCAGCTTCCTGTTTCTTGCCGGTTACAGCCTCGTGCTCGGCCAGCTCCCGAAACTCCGTCCG
CGCGCCTTCGCCATACGTTTCGCGAAGATCGCCGGCGCAGCCGCGCTGATCAGCATCGGGACCTGGTTCGCCT
TCCCTCAGTCGTTCATCTTCTTCGGCATTCTCCACGCCATTGCGGCGGCAAGCCTTGTCGGCCTCCTGTTCCT
GAAATTGCCGGCCGCCGTTTCCTTTCTTGCCGCTGCCGTGGCCTTCGCGGCTCCCCTCTATCTGCGCTCGTCG
TTTTTCGACATGCCGGCGCTCTGGTGGGTCGGCCTCTCGGAAACGATCCCGCGCTCGAATGACTACGTTCCTC
TGCTTCCCTGGATTGCCCCTTTCCTGCTCGGCCTCGGCACCGCGAAGCTGCTCCACCCGTTATTTTCCGCCCG
CCGCAGCCGAAGCGCCGGGACCCGTAAGCACCCGTGGATGACGCCTCTCGCCTTTGGCGGCCGTCACAGCCTT
CTGATATATCTCATCCACCAGCCGCTGCTTATCGCGGCAGTCTATCTCTTCTCTCTCGCCGTTCCCGCCCCGG
CACCGGATCCGGCACGGGTCTATCTGCTGAACTGCGAACGGGCCTGCAGCAACGAAAATTCCGGGATCTCCTG
CAGCGCCTTTTGCGGTTGCACGCTCGCCGGCCTGAAGGAACAGAACCTGTTCGACGATCTGAACCTGGGCAAG
ATCGACGTGAACACGGATGAACGCATTGCGCGAATCGCCGGTCAGTGCACAATGGATGCACAATCAGGAGAGG
CACAGTCAGGGGACTAGATGAACTTCGAAGCAGCCCGCATCAAGATGGTGGACAATCAGATCCGGACCACGGA
TGTGACCTCCCATTCAGTCCTGTCTGCTTTCCTGTCGGTCCCGCGTGAGGAGTTCGTGCCTGCAAAGATGCGG
GAGCTGGCCTATATCGACACCGATATCGAGCTCGTCGGCGGTTCCCAGCCGCGTTACCTGATGGAGCCGTCGC
CGTTGGCAAAGCTCCTCCAGCTCGCGGAGATCTCCAAATCGGATCTGGTCCTCGAGATCGGCTGCGGGACGGG
TTACGCCTCGGCTCTGCTTTCGCTTCTCGCCGGTTCCGTCGTGGCGCTGGAGAGCGACGAAGCCCTGGCCGCC
ACGGCCACGGCAACGCTGGCACGCCTCGGTTACGACAACGTCGCCGTGGTGAGCGGCGATCTGACCAAGGGAT
ATGCGGCGGAAGCGCCCTATGACGTCATCTTCATCGACGGTGCCGTCGAGATGCTCCCTGCCGAGCTGTTCGA
GCAGCTTCGCGACGGCGGCCGGCTCGTGGCCGTGGAAGGTTTTGGTAACGCATCTCGTGCGAAACTTTACGTG
CGCGAATCGGGCATGACGTCCGAGCGAGCTGACTTCAACACCGCCGTGAAGCCGCTTCCCGGCTTCCGCCGCG
ACCGTTCTTTTGTTTTTTGAATGGTTTCAGATGCGCGTAACGGCGCCGTCGCGATGACGAGGCGCTCGGGTGT
GTCGTTCGCAGCGAACCTGGCATTCGAGCATATCAGCCATCGTTACCATTCCAAGGATACGATCAAGGGCGTA
ACGCTCAAGGCCGAAGCCGGCGAAGTGCTCTGCCTGCTCGGACCGTCCGGCTCCGGCAAGACAACGCTGTTGC
GTATCGCCGCGGGCATCGAGAAGCAGACGGGCGGGCGCCTTCTGCTGAACGGACAGGAAATATCCGGCCCTTC
CATCTTCGTTCCGCCCGAGCAACGCGGCGTCGGCCTGATGTTCCAGGACTTCGCCCTTTTTCCTCATATGACC
ATCCGTGAGAACGTCTGTTTCGGCTTGACCGAATTGCCGAAGAAGAAGGCATTGCTGCAGGCCGATGCCGCGC
TGGAGCGGGTCGGCCTTCTGCATTATGCCGAGCGGTATCCGCATGTGCTCTCCGGCGGCGAGCAGCAGCGCGT
GGCGCTGGCCCGTGCACTTGCGCCGCGGCCGGCGGTGCTGCTGATGGACGAGCCTTTTTCCGGCCTCGATTCC
CGCCTGAAGGATTCTATCCGCGCCGACACGCTGGCCATACTGCGCGAGACGCGCGCGACCGCCATCGTCGTAA
CGCACGACGCCGAGGAGGCGATGCGTATGGCTGACCGGATCGCCCTCCTGAAGGACGGCCGCCTGGTGCAGGT
CGGCACGGCGGACGAGCTCTATCGCCGGCCCTGCGATCTCTTCACTGCCGGCTTCTTCTCCGAAATAAACGTC
TTCGATGCGCGCGTGCGGGACGGTCATGTCGAAACGCCGCTGGGGGTTGTGGCAACCGGGCAATATACGGAGG
GGCAGCCACTGAGCGTAGCCGTGCGTCTTTCCGGCATCCGGCTCGACGAGACCGGTGGAGAAATTCCGGCGCG
CATCGTGTCCCGGCGCTTCCTGGGCGTCGTGGAGCTTTTGGAACTCGCCGTGCCGCAGTCCGAACGCACGGTT

CGCGCCCGCATCCGCGCCGATTTGCTGCCGCAAGGATTGCGTGACGTCACGCTTTCCGTTAACGAGCGCGACA
TATTGGTGTTTGAAAAAGACGCATCAACACCTTAGATGCCGAAGCGCCAAGACATCAAATCGATCCTCATCAT
CGGCGCGGGGCCGATCGTCATCGGTCAGGCATGCGAATTCGACTACTCCGGCACCCAGGCGTGCAAGGCGTTG
AAGGAGGAAGGCTACCGCGTCATCCTGGTCAACTCCAACCCGGCGACGATCATGACCGATCCGGGTCTGGCGG
ACGCGACCTATGTCGAACCGATCACGCCAGAGGTCGTCGCCAAGATCATCGCCAAGGAACGCCCCGACGCGCT
GCTGCCGACCATGGGCGGACAGACGGCGCTCAACACGGCCCTGTCGCTGCGCCGCATGGGCGTGCTCGACCGC
TACAATGTCGAGATGATCGGTGCGAAGCCCGAGGCGATCGACAAGGCGGAAGACCGCGCCCTGTTCCGCGAGG
CCATGGCGCATATCGGCCTCGAGACGCCCAAGTCGCGTCTGGCGAACGCCACGGACATCAAGGACCACGACCG
CAAGTCGCACGAGGCGGAGCGCTCAGCGCTCAAGGCGAAGCTTTCCGGTGACGAACTCGACAAGGCGCTGGAC
GAACTCGAAAACCAGTGGAACCTCGGCGAAGGCGATCGCAAGCAGCGCTATGTGAACCACGCCATGGCGATCG
CCGCACAGGCGCTCGACGATGTGGGCCTCCCCGCGATCATCCGGCCGTCCTTCACGCTCGGCGGCACCGGGGG
CGGCATCGCCTATAACCGTTCGGAGTTCTTCGAGATCGTCGGCAGCGGTCTCGACGCGTCGCCGACGACGGAA
GTCCTGATCGAGGAATCGGTCCTCGGCTGGAAGGAATATGAAATGGAGGTCGTCCGCGACAAGGCGGACAACT
GCATCATCATCTGCTCGATCGAGAACATCGATCCGATGGGCGTCCACACGGGCGATTCGATCACCGTTGCGCC
GGCACTGACCTTGACCGACAAGGAATATCAGATCATGCGCAACGCCTCGATCGCGGTGCTGCGCGAGATCGGC
GTCGAAACGGGCGGCTCGAACGTCCAGTTCGCCGTCAATCCCGAGAATGGCCGCCTCGTCGTCATCGAGATGA
ACCCGCGCGTCTCGCGCTCCTCCGCACTCGCATCGAAGGCCACCGGCTTCCCGATTGCCAAGATCGCCGCCAA
GCTCGCAGTCGGCTATACGCTCGACGAACTCGAAAACGATATCACCGGCGGCGCGACGCCTGCATCCTTCGAG
CCTTCGATCGACTACGTCGTCACCAAGATCCCGCGCTTCGCCTTCGAGAAATTCCCGGGAGCCGAGCCGACGC
TCACGACCGCAATGAAGTCGGTGGGCGAAGTCATGGCGATCGGCCGGACCTTCGCCGAATCGCTGCAGAAGGC
GCTGCGCGGTCTCGAGACCGGACTGACCGGACTTGACGAGATCGAGGTGCCTGATTTCGACGACAATGGCGAC
GGCCGCAATGCCATCCGCGCCGCGCTCGGCACGCCGACGCCGGACCGCCTGCGCATGGTCGCACAGGCGCTGC
GTCTCGGCATGAGCGAAGCCGAAGTGCATGAAGCCTGCAAGATCGACCCCTGGTTCATTGCCCAGTTCAAGGC
GATCGTCGACATGGAGGCGCGCATCCGCGAGCACGGCCTGCCCGCCGACGCCGAGAACCTGCGCATGCTGAAG
GCAATGGGCTTCTCCGACGCCCGGCTTGCGACGCTTACCGGCAAGCGTCCGAAAGAGGTGGCCGAGCTCCGCA
ACGCACTGAACGTGCGCCCCGTCTACAAGCGCATCGACACCTGCGCGGCCGAGTTCGCATCGCCGACCGCTTA
CATGTATTCGACCTATGAGACGCCTTTCGTCGGTGCCGCCCGGTCGGAGGCGCAGGTTTCCGACCGGAAAAAG
GTCGTCATCCTCGGCGGCGGCCCGAACCGGATCGGCCAGGGCATCGAATTCGACTACTGCTGCTGCCATGCCG
CCTTCGCACTGAAGGACGCCGGCTACGAAGCGATCATGATCAACTGCAACCCGGAGACGGTTTCGACCGACTA
CGACACCTCCGACCGGCTCTATTTCGAGCCCCTGACGGCAGAGGATGTGATCGAGATCATGCGTGCCGAACAG
GAAAACGGCACGTTGCATGGCGTCATTGTCCAGTTCGGCGGCCAGACTCCGCTGAAGCTTGCCGAAGCGCTGG
AAAAGAACGGCATCCCGATCCTCGGCACCGCGCCGGACGCGATCGATCTCGCCGAGGACCGTGATCGCTTCCA
GAAGCTCCTGATGAAGCTCGATCTCAACCAGCCGAACAACGGCATTGCCTACTCCGTCGAGCAGGCCCGCCTC
GTTGCCGGCGAAATCGGCTTCCCGCTGGTCGTGCGCCCGTCCTATGTTCTCGGCGGCCGCGCCATGCAGATCA
TCCATTCGGAAAGCATGCTGCAGAGCTATTTGCTCGACACCGTTCCGGGGCTGGTTCCTGAAGATATCAAGCA
GCGCTATCCGAACGACAAGACCGGCCAGATCAACACCTTGCTCGGCAAGAACCCGCTCCTCTTCGACAGCTAC
CTGACGAATGCGATCGAAGTGGACGTCGATTGCCTTTGTGACGGCAAGGATGTCTTCGTTTCGGGCATCATGG
AGCATATCGAGGAGGCCGGCATCCACTCGGGCGACTCCGCCTGCTCGCTGCCGGTGCATTCGCTCGGCACGGA
CATGGTCGACGAACTGGAGCGTCAGACGGGGGCGCTCGCCAGAGCGCTGAACGTCGGCGGCCTGATGAACGTG
CAGTTCGCCATCAAGGACGGCACCATCTATGTGCTCGAAGTAAACCCGCGCGCCTCACGCACCGTTCCCTTCG
TCGCCAAGACGATCGGCGCGCCGATCGCCAAGATCGCCGCCCGCGTCATGGCCGGCGAAATGCTCGACGAGGC
GATCGCCGCCTATGGCAAGAAGCCGAATCCGCGCAACCTGAAACATATCGCCGTCAAGGAGGCCGTCTTCCCC
TTCGCCCGCTTCCCGGGCGTCGATACGCTGCTCGGCCCGGAAATGCGTTCGACCGGCGAGGTCATCGGGCTCG
ACACCGATTATGCGCTGGCCTTTGCCAAGTCGCAGCTCGGCGCCGGCGTCGACCTGCCGCGCGACGGAACGGT
CTTCGTCTCGGTTCGCGACGAGGACAAGGAACGGGTGCTGCCCGCGATCCGCATTCTCTCCGACATCGGCTTC
AAGGTCATGGCGACCGGCGGCACGGCGCGCTTCCTCGGCGAGCAGGGCATCGTTGCAACCAAGATCAACAAGG
TCCTGGAAGGACGGCCACATGTCGAGGACGCCATCCGCAACCGGCAGGTCCAGCTCGTGATCAACACGACCGA
CGGCAACAAGGCGATCTCCGACTCGAAATCGCTTCGCCGCGCGACGCTGATGCAGAAGGTGCCCTATTACACC
ACGATGGCCGGCGCCGAGGCCGCGGCACTCGCCATCAAGGCACTGAAGGCGGGCAACCTCGAAGTGCGTCCGC
TGCAGAGCTACTTCGAAACCTGATTGAACGCGAATCTGATCCGGCATGCTGTCCATCTCGCGGCGATCCTGGG
GCTCTATCTCTCCGGCGCGATGCTGATTCCGGCCCTGGTCGATCTCTATTATGGTCACCCGGACTGGCAGGTC
TTCGCCGTGACCGCCTTCATGTCGGGCGCAATCTCGTCGACGACGTTCATGGCGACCCGCGCAGGCCCCCCGC
CCTTCTCGAAGAAATTCGGCTTCCTGGTTGTGAATGTCCTGTGGATCGTGTTTTCGATCGTCGGCGCCATTCC
ACTCTGGCTGTCCTCGGTAAAGCTGGATTTTGCCCAGGCCCTGTTCGAGTCCGTCTCGGCGGTAACGACGACC
GGCTCGACCGTAATCGTCGGGCTCGATGACGCGCCGCCCGGCCTCTTGCTCTGGCGGTCGCTCCTGAGCTGGT
TCGGCGGCATCGGCATCGTCGTTCTCGGCCTGTTCATCATCCCCTATCTGCGTGTCGGCGGAATGTCCTTCTT
CAAGATGGAATCCTCCGACACCAGCGACAAACCCTTTGCCCGGCTCGCGAGCTTCGGCCGCGCCTTCCTCGTC
GTGTACGTGTCGATCACTCTGCTGTGCGCGATCAGCTATGCCATGCTCGGGATGAACAGGTTCGACGCGATCA

ACCACGCGATGACGACCGTCGCGACGGGCGGCTTTTCTACGCACGACGCGTCATTCGGCTACTTTTCCAGCAT
TCCCCTGCTCTGGGCCAGCACGTTCTTCATGACGGTCTGCAGCCTGCCCTTCTCCATCCTGATCGTGCTCGTC
GTGCGCGGGCGCCTGGACGCCCTTCGCGATCCTCAGATCATCGTATTTCTGGGCTATCTCACCGCATTCTCGG
TTGCGGTCGCGGCCTATCACCGGCTCGCCAACGGCGTCGAGTTCCACCTCGCGCTTACCTATGCCTTCTTCAA
CATGACGTCGATATTGTCGACCACGGGCTATGCGAGCGAAGATTACAGCCTTTGGGGACCGTTCGTGGTCATG
GTCGCGTTCATATGCACGTTCATCGGCGGTTGTTCAGGCTCGACAGCGGGCGGCATGAAGGCCTATCGGTTCG
TCGTGCTCTTCAACGCCATCCGGTCTGCCCTGAACAAGCTCGTCTACCCGAATGCGATCTACGCGGTCCGGTA
CGGGAAGAATACGGTCGACGCGGACGTGCAGCGCGCCATTCTGCTGTTCTTCATCACCTATATCCTGCTCTGG
GTCTTCGGCAGTCTGGTCATGGGTGCCCTCGGCTATGATCTCGTCACGGCCGTGTCGGCCGTCATCACGTGTC
TCTCCAATGTCGGCCCCGGCCTCGGCAGCCTTATCGGCCCTGCGGGCAATTTCTCGACACTCCAGGATCCCGA
GCTCTATCTGCTGTCGCTGATGATGCTGCTCGGGCGTCTGGAGGTCCTGACCGTCCTCGTCGTGCTCACTCCG
ATCTTCTGGAAGCAGTAAATGCCGAGATACCTGCTCCTGCTGGCGGCACTCACGAACGCCACCGTCATCCTCG
CCGACACGCAGGCAGAGGCGGGTCCGCTTGCCGACGCTGCGGCGAAGGCCGAACAGCAGGCGAGCTCCGGCGA
TGCCGCCGGCGCCCAGGAAACCTTGCGCAAGGCCATGAGCGACTTTTCGCAGACCCTTCCCTTCGCCATCGGC
AAGGCGGTCTTCGTCACAGCAGCACCGGCCGGCTTTGCAATGTACGAGCCCCGGCCCGATCCGGTATTCGAAG
CCGGCGAAACGCTCATATCCTATGTCGAGCCCGTGGGACTTACCTGGAAGGAGGCAAGCGACGAGGGTAAGCT
CGAGACACGCTTCACGGTCGACTTCGACATCCTGAATCCGAAGGGAGAGGTCCTCGCCAGCCAGAAGGCGTTC
GGCGATTTCACCTTCAAGGGCTATCAGCGCAACCAGGAAATCTATGCGACGCTGAGGATCGACGTCGCCGGGG
CACCATCAGGGAATTATGTGCTGCGCTACCGCTTCAACGACACCAACAGCGGCAGGAGCGCAAGCGTCGATCA
ACCATTCGAGATCTCCGGCCCTTGAATGCGCATTGAACTCGAAAAGCGCGCGAAGGCCTCGACGCTGTTTTCC
ATCCTTTCGCCGTTCATCGCATTTGCGCTGACCATCGTCTTCGGCGGCGTGATGTTCGCACTTCTCGGCAAGA
ATCCGGTAACCGCGCTCTACAGCTTTTTCGTGGAGCCGCTGAGCGAGGTCTGGTCGCTGCACGAACTCGCGAT
CAAGGCGGCGCCGCTGATCCTGATCGGCGTCGGGCTCTCCGTCTGCTTCCGCTCCAACAACTGGAACATCGGC
GCAGAGGGTCAGTTCATCATGGGCGCAATCGCCGGCTCGGCCCTGCCGGTACTCTTCTACGATTGGCAGTCAC
CGTTGATATTGCCGCTGATGATGCTTCTCGGCATGCTTGGCGGAGCGCTCTTCGCGGCGATACCGGCCTTCCT
GAAGGCGCATATGAATACGAACGAGATATTGACGAGCCTGATGCTGGTCTATGTGGCCCAGCTCTTTCTCGAC
TGGCTCGTACGTGGCCCTTGGCGCAACCCGCAGGGCATGAACTTCCCGGAAACGCGAACGTTCGGCGCGGATG
CGATCCTTCCCGAAATGCTCGCCTCGGGGCGTACGCATTGGGGTTTTGCCTTCGCGATCATCGCGGCGGTGCT
GGTGTGGTTCATGATGCGCTATACGTTGAAGGGCTTCGAAATCACCGTTCTCGGCCAGTCCGAGCGGGCAGGG
CGCTTTGCCGGCTTTTCCTCGCGCAGGATGATCTGGTTCTCGATGCTGTTTTCCGGCGCGCTGGCGGGGCTCG
CCGGTATTTCGGAAGTGAGCGGCGCCATGCAGCAGCTGCGTCCGGTGATCTCGCCCGGTTACGGATTCACGGC
TATCATCGTCGCGTTCCTCGGCCGGCTCAATCCGCTCGGAATCGTGGCCGCCGGTCTCGTCCTGGCGTTGACT
TATCTCGGCGGCGAGGCCGCGCAGCTTTCGATCGGCGTATCCGAAAAGGTGACGCGCGTCTTCCAGGGGCTGC
TGCTGTTCTTCGTGCTGTCTTGCGACGCGCTCATTCACTACCGCATCCGGCTGGTGTGGGACCGCTTCGCAGC
GGTCAGGACGGAGGAGGCGCACTGAATGCGGAGTCTCCGCCAACCTGGTGCACCGTTCACGCCGAGGGTAATC
TCTCTCGGGTGCCACGCCGAGAAGGCGCGATTGACCTTCAAACCGGGCCGGAGCGTGCTCGAAGCCGTCCATG
AGGCGCTCGCGGAAACAGGCTGCGACAGCGCGATGATAGAGGTCAGGGGCGGTGCGTTTGCGCCGCTTGCCTA
TGTCACACCGACTCTCTCGGATGACGGCATTCATGCCGCCTGGTACAGCGACATACATGCGCCTGATGGCCGC
GCACCCATCGAAGATCTGGTCATGACGTTCGGCCGGCGCGACGGGGAGCCGTTCCTGCACGGCCATGGTGTCT
GGCGGCACGAGGACGGGTTGCGCGCAGCCGGGCACGTAATGCCGAAGGACTCGCAATTCGCCGAACTGGTGGA
GGCGGAAGCCTGGCTCATGTCGGGCGCCATCATGGACCAACTGGAGGACAGCGAGACCCGGTTCCGTCTGTTC
ACGCCTGTGCCTCATACCGCCGCACCGCCCATCACGACGCGGCGCGCCGTTCTCTGCCGGTTGAAACCGAACG
AACCGATCCATCCGGCGATCGAGCGCACCGCAGCCGAGCACGGCATCGAGCGGGCGACTTTGCACGGCATCGG
CAGTCTCGTCGGCTGCACTTTCGCCGACGGCCAGGTCATGGAATCGGCCGCTTCGGAATTGCTTATTCGCAAG
GGGACGCTTTCACCCGATCGAAGCGGACAGGCTAAAGCGAAACTCGATATCGCGATCGTCGATACGGAATGCA
CCATATTCGAGGGTGAGATAGCCTACGGCGTTGATGCCGTTTGCATAACGTTCGAGGTGCTGATCGTCGAAGA
GTGAATGGCCTGGCAACCGGCGGAAAACAGATACGAGAAGATGAAGTACAATCGCTGCGGCAGGAGCGGCTTG
AAGCTGCCGGCGATTTCGCTGGGTCTTTGGCACAATTTCGGTGGCGACACGCCGCATGAGCGCAAGGTGGACA
TGTGCCGCACGGCCTTCGATCTCGGCATCACCCATTTCGATCTGGCCAATAATTACGGTCCGCCGCCCGGCTC
GGCCGAAATCGCCTTCGGCGAGATCATGCGCAGCGATTTCGCCGCCCTGCGCGACGAACTCATCATCTCGTCC
AAGGCGGGGTATGACATGTGGCCCGGCCCCTATGGCGAGTGGGGCAGCCGGAAATATCTTGTCGCCTCTTGCG
ATCAGAGCCTCAAACGCATGGGGCTCGACTATGTCGACATCTTCTATTCCCATCGCTTCGACCCGGAAACGCC
CCTCGAAGAGACTTGCGGAGCGCTCGATCACATCGTCCGCTCGGGGCGTGCGCTCTATGTCGGCATCTCCTCG
TATAACTCCCAGCGTACCCGCGAGGCGGCGGTAATCCTGAAGGAGATGGGCACGCCCTGTCTCATCCACCAGC
CGAGCTATTCGATGCTCAACCGTTGGGTCGAGGATGACGGTCTCGTCGACACGCTGGAGGAACTCGGCGTCGG
CTCGATCGTCTTTTCGCCGCTCGCCCAGGGCATGCTGACGACGAAGTACCTGAACGGCATTCCCGAGGACAGC
CGCGCCGCGCAGAACCACTTCCTCAAGAAGGACTTCATCCGTCCGGGGATCATCGACAATATCCGCAAGCTGA
ACGCCATCGCCGAACGGCGCGGCCAGACGCTCGCCCAGATGGCACTCGCCTGGGTGCTGCGCGGCGGCCGCAT

CACCTCGGCGCTGATCGGCGCCAGCCGTTCCGCACAGATCGTCGACTGCGTCAAGGCGCTGGAAAACGACACT
TTCACACCAGAGGAACTGAGCGAGATCGATCTTTACGCCCGCGAGGCCGACGTCAATCTCTGGGCCAAATCCG
CCGAGCTCTGAATGAGCAAGGCTTATAAGGACCACCTCGGCCGTCCGATCGTTGCCGTCACCGGCATGGGCGT
CATCACCTCGCTCGGGCAGGGGCTCGACGACAACTGGGCGGCGCTCACCGGGGGCGTATCGGGCATTCACAAG
ATCACCCGGTTCCCGACCGATGCTCTTTCGACCCGCATCAGCGGCACGGTCGACTTCATCGAGATTCCTGCGG
AGAATGCCGTCGAGCGTTCCTATGCGATGGCGCGCGAAACGACGCTGGAGGCCCTGGCTCAGGCCGGCCTTTC
CGGCGATTTCAACGGCCCGCTCTTCCTGGCGGCCCCGCCGATCGAGCCCGAATGGAATGCCCGTTTCGAGCTT
GCCGACCGCTCGCCGCCGTCGAGCCAGCCGGGCGACGCCTATAATCGGTTCCTCGCGGCAATGCGGCAGAAGG
CGGATCCGACCTTCCACGAGGCCGTGCTATTCGGCTCGATCTCGGAGCGTCTTGCGGACCGCTTCGGTACGCG
CGGCCTTCCGGTGACGCTTTCGACGGCTTGCGCATCCGGGGCGACCGCCATACAGCTCGGCGTCGAAGCCATC
CGCCAGGGCAGGACCGACAGGGCGCTCACGGTTGCGACGGACGGCTCGGTCAGTGCCGAGGCCCTCATCCGCT
TCTCGCTGCTCTCGGCGCTCTCCACACAGAACGAGCCGCCGGAAAAGGCTTCGAAGCCCTTCACCAAGGATCG
CGACGGTTTCGTGATTGCGGAAGGCGCCGCGACGCTGGTGCTCGAATCGCTGGAAGCGGCGGTCGCTCGCGGC
GCCCGCATCTACGGCATTCTCAAGGGCTGCGGCGAAAAGGCCGACCTCTTCCACCGCACCCGTTCCTCCCCCG
ATGGCGGACCGGCGATCGCGACGATCCGCGCGGCACTTGCGGATGCGGGCATCGACGAAGCCGGGATCGGCTA
CATCAATGCCCATGGCACCTCCACGCCCGAAAACGACAAGATGGAGTATCTGTCGATGTCCGCGGTCTTCGGC
GAGCGGTTGCCGTCGATCCCGGTCTCATCCAACAAGTCGATGATCGGTCACACGCTGACGGCAGCCGGTGCGG
TCGAAGCGGTCTTCTCGCTCCAGACGATGCTCAGCGGCACGCTGCCGCCGACGATCAACTACCAGAACCCCGA
CCCGGCGATCGTTCTCGATGTCGTGCCGAACGTGAAGCGCAGCCAGCAGGTGACTGCAGTGCTGTCGAACTCC
TTCGGCTTCGGCGGCCAGAACGCCAGCCTTGTCATGACCGCCGAGCCGGCCTGAATGAGGCGGCTGGCAGCGA
TCATGGACGCGGACGTCGTCGGCTACAGCCGGCTGATGGGACTGGATGAAGCGGGCACGTACCGGGCGGTCAA
ACGGTGCCGTGATGCCTTCATTCTGCCTTTGGTCGCGGCCCATGGCGGAAGGATAGTCAAGCAGGCGGGAGAC
GGCACGCTTGCGGAATTCGCAAGCGTTCTCGACGCGGTCGCCTGCGCGGTCGCGATCCAGCGAACGATGCATG
ACCACGCCGGAGGCGCCGAAGCGGAGCGTCTGGAATTGCGCATCGGCGTCCATCTCGGTGATATCGTCGCTGA
CGATGGCGATATCCATGGCGAAGGCATTGCCGTTGCCGAGCACCTGCAGGAGATGGCGCCGCCCGGGGGCGTC
TGCGTGTCGCAGCAGGTCTATGACCAGATCGGCTTGAAGGTGGATTTCCCCTTGACCGAAATCGGCTGCAGGA
CATTTGCCGATCTCCCCGGTCCGCTGCGCGCCTGGCGCTGGCAGCCGGGCGGCGCGGCAGGCGAAGAGCCGCC
GGCGCCGGCGCAAACCGATCCCCTGCCCGGTAAGAAGCGCCCCTCGATCGCCGTCCTGCCCTTCGTCAATCTT
TCGAGCGTCGAAGAACAGGAACATTTCTCCGACGGCTTTACCGAGGAACTGATCGCCACGCTCGCCCGATGCC
GGTGGTTGCGCGTCGTCGCCCGCAACTCCTCCTTCACCTTCAAAGGGAAGGCAGCCGACGTAAGGAGGGTGGC
GGAAGACCTGGGTGTCAAATACGTGATCGAGGGCAGCATACGTCGCGCGGCGAACCGCATCCGCATCACGGCG
CAATTGCTGAGCGGCGAGACCGGCATGCTGCTCTGGGCAGAACGCTACGACCGCATGCTGGACGATGTTTTCG
TGCTCCAGGACGAGATCGCGGGGCAGATCACCGGCACCGTGGAGCCGGAACTCGGCTTCATCGAATTCGCTGC
GCTCCGCGGCCACACCGCGACGGACATGGATGCCTGGAACATTTATCTGAAGGGGCTATGGCACCTTTACAAG
TTCGATCTGGAGAATCTGAGGATATCCAAAGCGCTCTTCGAGCGGGCGATCGATCTCGAGCCCGCCTTTGCCC
AGGCCCATGCGCGGCTTGCCTATGTCCATATACAGCTCGGCTGGTATGGCCCCCTGGACGAGCGGGCCGAGCG
GATCGCCGACGCGACGGCGCTCGCGGAACGCGCAATCGCGCTCGACGACCGTGAGCCGGCTGCCCATCTTGCG
CTCGGCCGGGCACGGGCGCTCGGCGGCCAGCCGCAGCGCGGCATAGACCACCTGCGCAATGCGCTGAGGCTCG
ATGCAAGCTTCGCCCAGGGCCACTTCGCCCTCGGGCAAGCGCTTTGCTATGTCTGCCGCCCGGAGGAAGGCAT
TGCCGCGATCAACGAAGCCTTCCGGCTGAGCCCCCGCGATCCGCATTTGTGGACGTTCTACAACATGGTGGCC
ATCGCCCACTATCAGTCGGGTCGCTTCGCGCAAGCCGCCGAAGCGGCCCGCGCCTCCCTGCGCCAGGAGAACG
CCACCTTCTGGCCGGCAATGGTCCTGGCAGCCTCCCTCGGTGCCACGGAGCGGTCCGACGAAGCCGGTTCAGC
CGTGGCGAACCTTCTTCGCCGGCGGCCGGACATGACCGTGAAAACGGCCCGCGCCGAATTCTACTTCGGCAGC
GTGCCGGTCATGCCGGAAGATTTCATCGATCGTTTCGTCCGCGATCTGCACCGCGCCGGCCTGCCTGACTGAA
TGGCGCAATTGATCCTCAACCACCTGACCAAGGACTTCGGCGGCAAAGGTGCAGACCGGGTAAGGCCGGCTGT
TTCGGACGTGTCGCTGGCGCTGCGCAAGCAGGGATTTCTCGCACTGCTCGGGCCTTCCGGCTGCGGCAAGACG
ACGGTGCTCCGGATGATCGCCGGCTTCGAGCAGCCGACGGACGGCTCGATCGAGCTCGGCGAGCGCAGGCTTT
CGGATGCAACCCGCGTGCTGCCGCCGGAAAAGCGCAACATGGCGATGGTGTTTCAGTCCTATGCGCTCTGGCC
GCATATGACGGTGGCGGAGAATGTCGGCTACCCGTTGAAGGTCCGCGGCATTTCCGGGGAAGCCTGGCGCAAG
CGGGTAGGCGACGCGCTTTCGCTGGTGAAGCTTACGGATTTCGCCGATCGGCGGCCGGCTGTACTTTCCGGCG
GTCAGCGGCAGCGTGTGGCGCTGGCGCGCTGCCTGGTCACGGAACCGGACGTGGTGCTGCTCGATGAGCCCCT
CGCCAATCTCGATCAGCATTTGAGGAAGTCGATGGAGGAGACCTTCCGCGCCTTTCACGAGCGCTCCGGCGCG
ACGATGATCTATGTGACTCACGACCAGGCCGAGGCGATGGCGCTTGCGACCGATGTCGCGGTGATGTCGGAAG
GCAAGCTTCTTCAGGTGGCGCCGCCGGCGGAAATCTATGCGCGGCCGGAGGGGCGGGTCGTCGGGGGCTTGAT
CGGTCGCGGCGCGATCGTGCGGTTGAATTTGCCGGAGGGAGGGGCGCGTGCGCTCGATTGGCCGCTCCTCGGC
GCCGCGCTTGGTGAGATGCCCGCGGGTGAGGAGCATGCAGGCGGGGGGCCGCTTTCCGACGTGCTCGTCCGTC
CGGAGCAGGTTCATCGGGATAGCGAAGGCATTCCGATGCGGGTTCTCTCCTCGGTCTTCGCGGGCGAACGCTT
CGCTCTCACACTGGCGCTGCCGGACGGGCAGAGCGTCAAAGCCTATAGCAGCGTGCCGCTCTGCGAGGGCGCT

CGCGTTCCTTCGTCGTGAGTGATCATCCTCGAGACCGGGCGGCTCAGGCTCCGCGCCTGGATCGAGACCGATC
GCGAACTCTTTGCCGAGATCAACAGCGATCCCGCGGTCATGGAGTTCTTCCCGTTCCGCCGCACCCGCGCGGA
GGCCGACGCGCTTTTCGACCACAACAACCGCAACATCCGCGAAACCGGCTTCGGCTTCTTCGCGCTCGCGTTC
CGTGAGAACGACGCGCCGATCGGCTTCTGCGGACTTGCACGCACCGACCTCGAACCGCACCTGCCCGACGGCA
CCGTCGAGATCGGCTGGCGCCTGGCTCTCCGCCATTGGGGAAAGGGCTACGTGACGGAGGCAGCGCGGGCGCT
TCTCGATTATGGATTCAGCGAGAGGAAGCTCGGGGAGATCGTTGCCATCGCCGTTCCGGCCAACACTCGCTCC
ACCGCCGTGATGACGCGGATCGGCATGCGTCCTGATTCCAAGCGCGACTTCGATCATTCCGGCGTCCCGGACA
CGCATCCCCATCTCAGACGCCACGTGCTTTACGCAACAACCGCGGAAGAATGGCGGCGGGATGGCTCGGCCGC
GAAGCGGTAGATGAATGAGATGAAGTGCAGGATCCGCGGGGCGCATCTGAAGACTCTTTTGGCGAGCGGAGTT
GCGCTCGCGCCGCTGATGATGAGCGGAATCGCGCTGGCGCAGGAGGGAAACGCCACGCAGCTCGAGCGAATCG
TCGTCGAGGGCGGCAATGCGGCCGGCGCCAGCGCGACCGGACCGGTGGACGGCTACGTCGCAAAGGCGACCGC
GACCGGGTCGAAGACCGCCATGCCGCTCAACGAAATCCCCCAGTCGGTCTCCGTCGTCGGGCGTGAGGAACTG
GACGACCGCGCCGTGGTCAACAAGGTCGATGAAGCATTGCGCTATACGCCGGGCGTCCTGTCGGCACCATTCG
GCACCGACCCGGATACGGACTGGTTCTACATCCGCGGCTTCGATGCGGCACAGACGGGCCTCTTCCTCGACGG
ACTGCCGCTCTTCAGCTTCGGCTTCGGCAATTTCCAGGTCGACCCCTTCATGCTGGAGCGGGTCGAGGTGCTG
AAAGGGCCGGCCTCCGTGCTTTATGGCGGGTCCAATCCGGGCGGCATCATCAACCTGATCAGCAAGCGGCCGC
TTGACGAGCCGCTCTACTACACCGAGGTCGGCATCAACAGCAACGGCAATGCCTTCACCGGCTTCGACGTGAA
CGACAAGCTGAACGATGACGGAACGGTTCGCTACCGCCTCACCGGCAAGGTCGCCGGCGGCGACAACTATTCG
GACTATTCCGAAGACCTGCGCGGCTTCATCCTTCCTCAGGTAACCTATGCGCCTGATGACGCAACCAGCCTGA
CCGTCTTCGGCCTGCTCCAGGGCCTCGATCAGGTCCATGTCGGCAACGGCTTCCTGCCCTATGTCGGAACCGT
CGAAGACGCGCCCTTCGGCAAGATCGACCGGGATGCCTATTACAGCGAGCCTGATATCGACGAGGGCAGCTAT
ACGCAGCAGATGCTCGGCTACGAGTTCAAGCACGACTTCGACAATGGCTGGACCTTCACCCAGAACGCGCGCT
ACGCCAATCTTCACAAACACGAGAAGTACCCGTACACCTATGGCTATGTCGGCGGCGCACCTACCGGCCCGGA
CTACCTCCTGAACCGCATCGGCTTCGAGGCGACGTCGAAAGTCGACAGCTTCTCGATCGACAACCGGACGGAA
ACGGATTTCGATCTCGGCGCCACGACCCACACCTTCCTCGCCGGCCTCGATTACAAATACTACCGGCTCGACC
ATATTCAGGCATGCTGCGGCGCGACCCCGATCAGCGCAACCAATCCGGTTTACGGCACACCTCAGGGTGCAAA
TTTCGTATATCTCGATCAGATCGTCACGCAGCAGCAGATCGGCCTCTACGCACAGGACCAGATTCGTTTTGGC
GACGGCTGGCTGGTTACGCTCAACGGCCGCTACGACTATGTGGACACCAAATCCGACGCCGCGATCGGCACGA
GCTACGAATCGAATGACGGTGCCTTCAGTGGCCGTGCCGGCCTCGCTTACGAGTTCGACAACGGCCTCACGCC
CTATGTAAGCGCTGCGACTTTCTTCAATCCGCTGGTCGGCACGGGCACGAGCGATCCATCGGATCCGACGAAT
CCGGCAAAGATGGTCGCGCTCGAGCCGGAGGAAGGCTATCAATACGAGGCGGGCGTCAAATACGAGCCCTCCT
TTATCGACGGGCTGTTGACTGCATCAGTCTTCCAGATAACCAAGCAGAACGTCTCAATTGCCGTTCCCGGCTT
CTTCGTCAACTCGCAGCTTGGCGAGGTCCGTTCGAGGGGCGTCGAACTCGAGGGCAAGATCAATCTCAATACC
AACTGGAAGATCATAAGCGCCTTCTCCTATACCGATCTCGAGGTCACGGAAGATCTCAACGCCAGTCTCATCG
GCAATACACCGGTTCTCATTCCCGAAACACAGGCGTCGCTGTGGCTCGACTACACGGTTGCGAACGGCACGTT
CGAGGGCGTGAGCCTCGGCGCCGGCGTGCGCTACCAGGGGGAATCCTGGGCCGACGCGGAGAACACGAAGAAA
GTCCCCGCTGCGACACTCGTGGACGCCGCTATCCGCTACGAGAAGAACGACTGGACGGCGTCGCTCAACGTCG
CCAACCTGTTCGACAAGGAATATGTTGCGGGTTGCCAGGGTCTCCAGACCTGCGGCTATGGCGAAAGCCGGAC
CTTCACGCTGAAGCTCAGCAAGAAGTGGTAAATGGCTTCAGTAACGCACTCCAGCCCAAGGGGCAGCAGGTTC
AGGATCAGCAAGAGGGCCTTGCCCTATGTGCTCTCCCTGCCGGCGCTTCTCGTGTGCATCGGTATCCTGATCC
CGTTCTTCACGGCGGTGATCTATTCCTTCCAGCGCTACCGGCTCAGCCAGCCCTGGGCACGCCAGTTCAACTG
GGGTGAGAACTATCTGAACTTCTTCACCGATCCGGGTTTCTGGAACACGCTGAAGATCTCGCTGCTCTATGCC
GGCGTCACCGTGACCCTGGAGCTGCTGCTCGGTCTCGGCATTGCGCTGCTCCTGCAGCGCCGCTCGACGGTCA
ACAACTTCATCTCGATCATGCTGCTCCTGCCGCTGATGACGGCGCCGGCGCTTGCGGCGCTGATGTGGAAGCT
GATGACCAATCCCGGCTTCGGGATCCTGAGCTATCTCGCAAGTCTCGTGGGCCTTGAGAACTTCCGCTGGGCA
TCGTCGCCGGACACGGCGCTGCTGACCGTCGTGCTCGTGGACATCTGGGTCTATACGCCCTTCATCATGATCC
TGCTGCTCGCGGGCCTGCGCTCGCTGCCGACGCAGCCATTCGAAGCGGCCGCGCTCGACGGCGTGCCGCGCAG
CTTCGTCTTTTTCCGCATCACCCTGCCGATGCTCACGCCCTATATCCTGACGGCGACGCTCTTTCGCCTGCTC
GATTCCATCCAGCAGTTCGACATCATCTACGCGATGACGCAGGGCGGGCCCGGAGACACGCTGACGGTCTTCC
AGGTCGAGGCTTACCTCAACTTCTTCCAGTCGACCAATGTGGGGCGTTCCGCGGCGCTGCTGATCATCCTGTG
GGCGATCACCTACGCGCTGTCGAACATCTTTATCAAGAACTGGCTCCGGCTGCGCGAACGCGCCCGCGGCGAA
GCGTAAATGACGCTGGAGGAGCTCTACCAGGCCGGGCGCAAGGTCGTCACCGGCAGCCTGACATTCACCGCGG
AGGACATCGTCCGCTTCGCCCGCGATTTCGACCCGCAACCCTTCCACGTCGATGAGGAGCAGGCGAGACAGTC
GCTTTTCGGCGGCCTTTGCGCCTCCGGCTGGCATACCAGCGCCGGCTGGATGCAGTGCTTCCTGCGGTTCTGG
AAGGAGGAGATCCGCCGCCTTGCAGCCGAGGGATTGCACGCGCCGAAGCTCGGCCCCTCGCCCGGGTTCAAGG
AACTCAGGTGGTTGAAGCCGGTCTATGCCGGCGACACGATCACCTATGCGGTCACTCTACTGGAGGCCCGGAT
CGTCGCCTCGCGACCCGGCTGGCGGATCAACACGATCCTCTGCGAGGGCGAGAACCAGCACGGCGAGCCGGTC
ATCCGCTTCGAGAGCAAGGTCATCGAATTCGCTTGAATGTTCGATCCGATCGTTGCGTTCTTCCAGCGTGTAT

TCACCGCGATCGGCCGTGGCATCGGATTGGCGGTGGCGTGGCTTCTCTGGCCGTTCGTCGCGCTCGGCAACTG
GTATCGGGGACGAAGCTGGATCATCAAGGGGCCGATCGGCCTGATCCTCCTCTGCCTCGTGCTCTTCTACGCC
TACTTTGTCTGGCAGACGCAAGCCTGGACGAATTTCGACCCGGACTATGTCGACCGATACAAGCTCGCCGACC
GCAGGACGCCGGCCGGTTCACCCGTCAGCGGTCCGGGGGCAACAACGGGACAGTCAGGCTCGGCGGCTGACGC
AAACCAGGCCGCCGCGGTCGCTCCTTCCGGTGCGGACAGTACGACCGAAGCCCTTGCCGTGGCCCAGCTTCCG
GCCGGGACGGTCTGCCAGACTTCGGCCATCGTCGACGTGGCGGCCGACCTCATCGACCTCAATGTCAATCAGA
ATGCCTGGATATCGTCGATGCTCGGCTACAAGCTCGGCTTTTTCGGGATTGACTGGGACGACACGCCCTGGCT
CGACAACAAGGCGTCCTTCCAGCGCGGCGTCAACCAGGCGGTGCGCCGCACGTCGGTGGAACTCGTCGATTCG
CTTGGGCGCGTCCGTGGCACGTCGGGGGTCAACAACGAGCTCCAGAACGCGCGCGGAAGCATCCAGTTCGACG
AGGAAACCTGGTATTTCGGCCTGAACCCCTTCGGCCCCAAAACCCCGACCCCGAGCTTCTACCGCTCGGCTAT
GCGCGATCTCAGGAAGTTCAACGTCTCGCTCGGCAAGTGCGAAGCGATCTTCGACGGCCGATCCGACAACATG
GTCGAGTTCCTCGACCGGGTCGCGAACGATCTCGGCAATACCTCGGCGATCATCCGCGAACGTTCGGAGTTCC
ACAATGGCGGCTGGTTCGACACGCGGGCGGACGACCGCTTCTGGTTCGCCTTCGGCCAGCTCTACGGCTATTA
CGGCATTCTCTCGGCCGCCGGCGCCGATTTCGAGAATGTCGTCACCCAACGTGGCCTGGCGCCGATCTATACC
GAAACGATGAAGCAGCTTCGCGCGGCACTCAGGATCCAGCCGCTCATCATCTCGAACGGGCGCGAGGACGGCT
GGATCATGCCGACGCACCTCGCGACGATGGGCTTCTACATCCTCCGGGTTCGATCGAACCTCGTGGAGATGCG
CGATATTCTTGCCCGTTGAATGAACTTCTCCGCCTGGTCCATCCGCAATCCGGTCGCGCCCATCCTGGCGTTT
TTCGTGCTCGTGGTGCTCGGATGGCAGTCGTTCAATTCGCTGCCCATCACGCGCTTCCCGAACATCGACGTGC
CGATCGTTTCGATCGCCGTCACACAGAGCGGTGCGGCGCCCGCCGAACTGGAAACCCAGGTCACCAAGGAGAT
CGAGGACGCGGTCGCCGGCGTCTCCGGCGTCGACCATATCGAGTCGACGATCACCGACGGCATTTCGACGACC
GCCGTCATCTTCCGCATGGAAGTCCCGACGACGCAGGCCGTGCAGGATGTCAAGGATGCCATCGACCGCATCC
GAAGCGACCTGCCGACCTCGATCGAAGAGCCGATCGTTTCCAAGGTCGATGTGGAAGGCCAGGCGATCCAGAC
ATTTTCCGTCTCGTCGCCCGGCATGACGCTGGAAGAGCTGTCCTGGTTCGTCGACGACACGATCAAGCGCGCC
ATCCAGGGCCAAACCGGCATCGGCCGTGTCGACCGCTACGGCGGCTCCGACCGCGAGGTCCGGGTCGAGCTCG
ATGAGGACCGTCTGAATTCCTTTGGCATCAGCGCCGCCGACGTCAACGCCCAGCTTCGCCGAATGAACATGGA
CCTCGGTTCCGGCCGCGGCCAGGTCGGCGGCAGCGAACAGGCGATCCGCACATTGGGCGACACGCGCGACGTA
ACGGCGCTGGCGGGTACGATGATCTCGCTGCCGAACGGCCGCTTCGTCCGTCTGTCGGAACTCGGCAAGGTGA
TCGACACCTATGAGGAGCCGAAGTCGTTCTCCCGCTTCAACGGGCAGCCGGGCGTCACCTTCGCCGTGTTCCG
CGCCAAGGGGGCGAGCGAAGTGTCGGTCGCGGAAACCGTCGGCAAGACGCTCGATGAAATCCGCGCGAAGCAT
CCGGATGTCTCCATCGAGATGGTGGACGATTCGGTCTACTTCACCTACGGCAACTACGAAGCGGCGATTCATA
CGCTGATCGAGGGCGCCGTGCTCGCCGTCATCGTCGTTATGCTGTTCCTGCGCAACTGGCGCGCGACCTTGAT
CTCGGCGGTCGCACTGCCGCTCTCAGCCATCCCCACCTTCTGGGTGATGGAACTGCTCGGCTTCTCGCTGAAC
CTCGTCAGCTTCCTGGCGATGACGCTCGCGACGGGTATTCTCGTCGACGATGCGATCGTGGAGATCGAGAACA
TCGAGCGTCATATCCGCATGGGCAAGTCGCCTTACCGCGCAGCGATCGAGGCTGCGGACGAGATCGGCCTTGC
GGTGATCGCCACCACCTTCACGATCATCGCCGTCTTCGTGCCCGTATCCTTCATGCCTGGCATTCCGGGACAG
TACTTCATCCAGTTCGGCCTGACGGTCGCCGTCTCGGTGTTCTTCTCGCTCCTGGTCGCCCGCCTGATCACGC
CGGTCATGGCCGCCTATCTGATGAAGCCTTCGGATGTCAGCGGGCATCATGACGACGAAGGCTTCATGATGCG
CCAGTACACGCGCCTCGTACGCTTCACCACGAAGCGCTGGTACACGCGCTACTCCACCCTGCTTGCGGCGATC
CTGGTCTCGGTCGGTTCCGTGGTCGCGCTGCTCGTCTTCGTCCCGGGCAGCTTCCTGCCGCCGGAAGACAGCT
CGCGCGTCAGCCTGTCCATCGAACTGCCGCCGGACGCAATGCTCGCGGATACGGACCGGACCACCACCGAGAT
CTACAATCGCGTCAAGGATATCGACGGCGTGGAAAACGTCTTCGTACTCGGCGGCGCCTCGCCCAAGGGCGAC
CTCGAACTGCGACGCGCTGCCGTCACGGTGCTGCTCGAGAAGCTCGATCATTCGCTGATCAACAAGGTCGTCA
ACGACGTGATCGGCCGCACACCGCTGATCGGCCAATACCTGCCGAAGCTGCCCCCGGCCGGTCGGATCAAGCC
GCAATCGGAGATCGAGAAGGAAATCTTCGCCCGATTGCGATCGATTCCCGATGTCCGCGTCACCAAGCTGAAC
GATCGCGGCGAGCGTGATCTGTCGTTCAACCTGCTTTCCAGCAACGAGGCGGATCTCGACAAGGCGGTCGCCA
CCCTGGAAGCGGATCTGCGCCGAGATCCGCTGCTTGCCAACGTTAGCCCCGAGGGAGCGCTGCCCCGACCGGA
ACTGCAGATCCGGCCGCGCGACGAGCAGATGTCGCGCCTTGGCATCACGACGGCGCAGATCTCCGAGGTGATC
CGCATCGCCACGATCGGCGATATCGACGCGCAGTTGACGAAGATTGCGCTCGATGGGCGCCTGATCCCGATCC
GGGTGCAGCTCGACCGTGATTTTCGCACCGACCTGGCGGCGATCCGCAACCTGAAGGTCCAGACGGCATCCGG
AGCGACCGTTCCGCTGTCGAGCGTCGCGGACATCAACTATGCGGAAGGGCCGAGTTCCATCAAGCGCTACGAC
CGCTACCGCGTCGTCAAGCTCGGCGCCGACCTGCCCGCCGGCGTGGCGCTCGACACGGCATCGGCCCGCTTCA
GGGAAATCGCCGCCGACGCCGATCTGCCTGAAACGGTCCAGTTCCTTGAAAGCGGCGACGCCGAGGTGCAGGC
CGAGATGCAGGAGAGCTTCGGCAACGCCATGCTGCTGGGTCTGATGATGGTGCTGGTCGTGCTCATCCTCCTG
TTCAAGGATGTGATACAGCCTTTCACCATCCTGTTCTCGCTGCCGCTCGCGATCGGCGGCGTGGCAGCCGCCT
TGATCCTGACTCAGAACGCGCTGTCGATGCCGGTGCTGATCGGTATCCTGATGCTGATGGGCATCGTCACCAA
GAACGCCATCCTGCTCGTCGACTTCGGTATCGAGATGATGCATCACGGCATGGACCGGACGCTCGCCATGATC
GAGGCCGGACGGAAGCGGGCTCGTCCGATCGTCATGACCTCGATCGCCATGTCCGCGGGCATGCTGCCCTCGG
CGCTGGGCGTCGGCGAAGGCGGTTCCTTCCGTGCACCGATGGCGATCGCCGTCATCGGAGGCATCATCGTTTC

GACGGTCCTGAGCCTCGTCGTGGTTCCGTCCTTCTTCCTGATCATGGACGACCTGTCGCGTCTCCTCGCCTGG
GCTTTCGGCCGCTTCATCGGCAAGAAGGACGAGGAGGCACTGCCGCTCGACCAGGAGGCGCTGAGCAAGCTTG
CCGCCGAACAGGGCAGCACGATCGAGAGCCTCGAAGAGCGCATCAAGGCGCTCGAAGACGAGAAGCGCCGCAA
GTCCGACGGCAAGGTCATCAGCCATCCGGCGCTGGCCGCGGAATAGATGTCGCGTTTAAACAGGTTTCTGATA
TCGGCACTGGCCGCAGCGGCTATTGCCGCGCCGGCGCTGGCCACTTCGGCAAGTGCCGCGACGCTGCGTTGGG
GCAGCCGGGCAGATATCTATTCGCTCGATCCGGATTCCGTTCCCTCGACATCCAATCTGGCGTTCCTGAACCA
CATCTATGAAGGTCTGATCCGTTATGGCCCGAACTTCGAGATCGAGCCGGCGCTCGCCACCGAGTGGAAGCTG
ATCGACAGCAAACACTGGCGTTTCACCCTGCGCAAAGGCGTGAAATTCCACGACGGCGCGGATTTCACCGCCG
ACGACGTGGTCGCCTCGATGAACCGCGTTTCGGACCCGACTTCGCCCCTGCGCGGCAATATTCCGCTCTATGT
CGGCGTCAAGAAGGTCGATGATTTCACGGTCGATATCGAGGTGTCGGCGCCGAGCGCGCTGTTCCTGAACGAC
ATGAGCAATATCTTCATGTTCAGCGCCAAATGGCTGAAGGACAACAACGCGGAAAAGCCGACCGATATTGCGT
CCAACACCGAGAATTACGCCACGCACAACACGAACGGCACAGGCCCGTTCAAGCTGGAAAGCCGCGTTCCGGA
CAGCAAGACCGTTCTCCTCGTCAACGATCAGTGGTGGGATCAGAAGAAGCACAATCTGGACCGGATCGAATAT
ATTCCGATCTCGTCGGCGGCAACGCGTGTAGCGGCGCTTCTTTCCAGCGAAATCGATCTGCTCGATTCCGCCC
CCATTCAGGATCTTCCCCGTCTGGAATCCTCGCCGGGCATCACCGTGAGCAAGCGCACGGAGCTGCGTACGGT
TTTCATCGGCTTCAACGGTAAGGAGAAGCTTGAAGACGGGCGTCCGAACCCGTTCCTGGACCTTCGCGTCCGT
CAGGCCGTGGAAGCAAGCATCGATCGCGATCTCATCAACAAGAAGATCATGCGCGGTCTGGCGCGGCCTTCCG
GCTCGCTCATCGCACCGGAAATCGCCGGCTATGCGAAGTCGCTCGACACTTATCAGCCTGCCGATTCCAAGCT
TGCCCAGAAGCTGCTCGAAGATGCCGGCCAGAAGGATCTCGCCTTCACCTATCTGTGCATGAACGACGAGAGC
ATCAACGAGGAAGACATCTGCTCGGGCATTGCAAACATGCTCAAGCGTGCCGGCTTCCAGCCCACCATCGACA
TGGGCCCCCGCGCCGTGCAGCAGCCGAAGCGCACCAATGGCAAGGCCGACATGTTCAATCTGAGCTGGGCAAA
CGAACCGACACTCGATGCCTATTCGCTGCTGTCTCAGGTTCTCTCCACACGCAGCGGCTCGACGGGCGTGTCC
AACTATGGCGGCTGGTCCTATCCGGAACTCGACGATCTGGTGAAGAAGGCGGCACAGGAACCTGACACCGCCA
AGCGTCTCGCGCTGGAAGAACAGGCGCTGAAAATCGCCAAGGACAAGGCCATCCTGATCCCGCTTCACCAGCA
GCCGATCGCCTGGGGCATGCTGGACAAGGTCAAGAGCGTGGATTTCCGCGCCGACAACAAGCCGCGCCACTGG
CACACGCAAATGGCCGAATAGATGCAGCCGCAGGAGGCGCGCGTGTGGGCAACCCTGGCCGGTGCACTTCTTT
TCATCAGCGGGCTTCTCTACATGGCATGGGAGGCGCTCGGACGGCGACGGCTGAGCGAGCCGCCGCAGCCCGC
GGAACGCAATCCCAAGCAGACGCTTGAGCCGCGCGGGCAAGGCCTGCGCTTTCTGAGCCCGTTGCGCAACTGG
CTGGGCGTCGCGCTCATGCTGGTCGGCGGAATTCTCCTGCTCTTCGGCATGCGCTGAATGAGACACGCGGAAA
TCGGAAAGCTCGACAAATCCCAGGCCAGGCATGTCTGGGACGTGGCGGAGTATTGCAGGCGGACCGGCATCAA
CAAGGCCGAGGAACGCCGCCTGATCAGGGTTCTCGGGCGATTTGCATCCAGCCACGAGCTTCAGATGAACATC
GTGCGTCCTCAGACGCGCATCCGCTGAGTGAACGCCAGGGCCTATTTCTATCTCGGTATTACCGCGCTCTTTT
GGGGCGGCAATTCGGTAGCGGGCAAGATGGCCGTCGGGCACGTCAGCCCGATGATGCTGACGACCTTGCGCTG
GTTGCTCGCGCTTGCGGTCATCCTGGTCCTGATGACGCCGCAGATCCGCCGCGACTGGCAAAAGATCCGCCAG
CACTGGCTGCAGCTGCTTGCCTATGGCGCCGTCGGCTTCACCATGTTCAATGCGTTCCTGTATTCCGCGGTGC
AATACACCAGCGCCATCAATGCCGTCATCCTTCAGGCCGGCATTCCGATGCTGATCTTCATCTTCAATTTCGC
GCTTTTCAAGATGAAGGCGTCGCTCGCCCAGGTCATCGGCTTTACCGTCACATTGGTCGGCGTGCTCATTACC
GCCGCCCATGGCGATCTCGCGAGCCTCATGCGCCTGAGCTTCAATTTCGGCGACGCGCTGATGATCCTCGCCT
GCATCGTCTATGCCGCATATACGGTTGCGCTGCGGTGGAAACCGGCGATGCATTGGCAGAGCTTCATAGCCGC
CCCGGCCTTCGGGGCGCTGCTGAGCGCCATACCGCTCCTCTTGTGGGAGATGGGGAAAGGCACGGCGATCATG
CCGGATGCGACCGGCTGGGCGATCGTGATCTATGCGGCGATCTTCCCCTCGCTGATGTCGCAGGTTCTCTATG
TTCGCGGCGTCGAGATGATCGGCGCCAACCGTGCCGGTCTGTTCATCAACGCCATTCCGGTGTTCGGGACCCT
TCTCTCCGTCCTTCTGGTCGGCGAGATATTTCACCCGTTTCATCTCGCGGCGTTGGCACTCGTCCTCGGCGGC
ATCGCCATCGCCGAACGCGGCCGGCCGAAGCTGCCGCGGTGAATGAGAAATAACCTGCTTCCCGTTGCCGCGC
TGCTGCTCGGTACCCTGTTTCTCTTCCTCGGAAACGGCCTGCAGGGCCTGCTCCTGCCGGTGCGCGGCACCGC
GGAGGGCTATCCGACAACCATCCTCGGCCTGTTCGGTACGCTCTGGGCCACCGGTTTCGTGCTTGGCTGCTTC
TTCGCGCCGAACGTCGTCAAGCGCATAGGGCATGTGCGCGCCTTCAGCGTGTTCACGGCCTTGATCGCCATCG
TTTCCCTGCTGACGGGCATTCTGATCGACCCGATCTGGTGGCTGGCGCTCCGCGCAGTCACGGGTTTCTCCAC
TGCCGGAACGTCGATGATCATCGAAAGCTGGCTGAACGAGCGCGCCACCAATGAGAGCCGCGGCGTGATCTTC
TCGCTCTATATCGCCATCACCCTTTTCGGCGTCGTCGGCGGCCAGATGATGATTCCCTTCGGCGAAACGTCGA
CGACCTTCTTCTTCATGATCTGCGGCATTCTCTACTGCGTGGCCATGCTGCCGACGCTGTTGTCCAGGGCCGC
CTCGCCGCAGCCGCTGAAACAGGTCCGCCTCGATCTGCGCGGCCTCTACCGCAATTCGCCGGTTTCCTTCCTC
GGCATCCTGCTCATCGGCATTGCCAACGGCGCATTCGGCACGCTCGGCGCGGTCTTCGGCCGCCAGGCGGGGC
TGTCGGACAGCACAGTTGCGGCGATGATGAGCGTCGCGATCTTCTCGGGCGCCGTCATGCAACTGCCGGCCGG
CCGCATCTCCGACCGTATCGACCGCCGCTATGTGCTTGCCGCGCTCGCCGGGGTCGGCGCTCTCGCCGGCCTC
CTGATCTTCCTCGTCGAGCCCGGCCAGGTCTGGATCGTTCTGACGTTGATCGCGATCTACGGCGCGGCCGCCA
ATGCGCTCTACCCGATCGCGGTTTCCCACGCCAACGACTTCGCCACGCCCGAGGATTTCGTGAAGGTTTCCGG
CGGGCTCCTGCTGCTCTACGGCATCGGCACGATCATCGGCCCGACGATCGGCGGGCCGATAATGACCGCCAGC

GGGCCTTATGGCCTCTTCATGATCACGGCCTGTGCGCATATGCTGATCACTGCCTATGCAATCGTCCGCAGCC
GCCGGCGCGCACCGGTGCCCGCGGCCGAGCGCGAAAACTTCTCGCCGGTCAATGCCGGCACGGCGACGACGCC
GGAGAGCCTGCAGCTCTCGCCGCGTGCGGCTCCGCTGGAGGAACTGCCGGACGAAGGCGCCGACGATCCCCAG
GAAGAGGAGAGATCGAATGAGCCTGTATGAATGAGACGGAACAGGAAAGTCAAAATCCTCGCGACGCTCGGGC
CGGCATCGGCCGACGAGCAAATGATTCAGAAGCTGCACGAGGCGGGGGCCGATCTGTTCCGTATCAACATGAG
CCATGCAAGCCATGAGGTCATGCGCACGCTGATCGAGCGGATCCGTGTGGTCGAGGCGCGCTGCGGCCGTCCG
ATCGGCATCCTCGCCGATCTGCAGGGGCCGAAGCTGCGCGTCGGAAAGTTCGCCGAAGGCAAGGTCGAGCTCA
AGATCGGCCAGACATTCACGCTCGACAACCGCGACGTGCCCGGCGACAGCCGCCGCGTCTATCTTCCGCATCC
GGAGATCCTCGAAGCGGTGAAGCCCGGCCATCGCCTGCTCATCGACGATGGCAAGCTGCATCTGCGCGCCGAA
AAGACCGATGGCAAGAGCATCGTCACCACGGTCGTTTCCGGCACGCGGATTTCGGATCGCAAGGGCGTCAGCC
TGCCCGATACCCTGCTTGGCGTCGGCGCGCTGACGGACAAGGACCGCGTCGACCTCGACGCCGTGCTGGCGAC
AGGAGAGGTCGATTGGGTGGCGCTTTCCTTCATCCAGCGCCCTGAGGACCTTGCCGAGGTTCGCAAGATCGCC
CGCGGCCGCGTTGGACTGATGTCGAAGATCGAGAAGCCGCAGGCGATCGAGCGGATAGACGAGATCATCGAGC
TTTCGGATGCGCTGATGGTGGCACGCGGCGACCTGGGCGTCGAAATGCCGCTCGAATCCGTACCGGGACTTCA
GAAGCAACTGACGCGTGCCTGCCGGCGAGCCGGCAAGCCGGTCGTCGTCGCCACGCAGATGCTCGAATCGATG
ATCTCCTCGCCTGTGCCGACGCGCGCAGAAGTGTCGGACGTGGCTACCGCCGTGTTCGAGGGTGCCGATGCGG
TGATGCTTTCGGCCGAATCCGCCTCCGGCGAGTACCCCGTCGAGGCGGTTTCTACCATGGCGTCGATCGCCAG
CAACGTCGAGCGCGATCCGCATTATCCGGGGATCATCTACGCTCAGCGCACGCCGCCGGAGGCAACGGGCGCG
GACGCCATTTCGCTTGCCGCTCACCAGATCGCCGAGACGCTCAAGCTTGCCGCAATCGTGACCTACACCTCCT
CTGGCACCACCGGCCTGCGCGCGGCGCGCGAGCGCCCCCAGGTGCCTGTCATCGCGCTTTCGCCGATCGTCCA
GACGGCCCGTCGCCTTTCCGTGGTCTGGGGGCTGCACTGTGTCGTCACAGAGGACGCGACCGACCTCGACGAT
ATGGTCAACCGCGCCTGTCGAATCGTCTCGACGGAAGGCTTCGGCAAGCCGGGAGACCGCATCATCGTTACCG
CCGGCGTGCCGCTCGGTACCCCCGGCGCCACCAACATGCTGCGCATCGCCTATATCGGCTCGGACGGCCAATC
GGGCATCTGAATGACTGCGAAAATCCTCCTTGCCGAAGACGACAACGACATGCGCCGGTTTCTTGTGAAGGCG
CTGGAAAAAGCCGGCTACAAGGTTCTTTCCTATGACAACGGCGCCAGCGCCTATGACCGGCTTCGCGAAGAAC
CCTTTTCGCTTCTCCTGACCGACATCGTCATGCCGGAGATGGACGGCATCGAACTCGCGCGCCGCGCGACCGA
ACTCGACCCCGACCTGAAAGTGATGTTCATCACCGGCTTCGCCGCCGTTGCGCTGAACCCGGATTCCAAGGCG
CCGAAAGACGCCAAGGTCCTTTCCAAGCCCTTCCACCTGCGCGACCTCGTCAACGAGGTCAACAAGATGCTGG
CCGCCTGAATGAACATCCATGAATACCAGGCCAAGGCTCTGTTGAAGAGCTATGGCGCGCCGGTCGCGGAAGG
CGTCGCGATCTTCTCCGCCGACGAGGCCGAGGCGGCCGCGAAGAAGCTGCCGGGGCCGCTTTACGTGGTGAAG
AGCCAGATCCACGCCGGCGGCCGCGGCAAGGGCAAGTTCAAGGAACTCGGCCCCGATGCGAAGGGCGGCGTGC
GCCTGGCCAAGTCGGTGGACGAGGTCGTCGCCAATGCCAAGGACATGCTCGGCAACACGCTGGTGACCAAGCA
GACCGGTCCGGCCGGCAAGCAGGTCAACCGCCTCTATATCGAGGACGGCGCCGATATCGACCGCGAGCTCTAT
CTCTCGATCCTCGTCGATCGCTCCGTCGGCCAGGTGGCCTTCGTCGTTTCGACCGAGGGCGGCATGGATATCG
AAGCGGTCGCCGAACATACGCCGGAGAAGATCGTCACCGTTGCGATCGATCCGGAAAAGGGTGTGACCGCAGA
GAATCTGAAGACGCTGGCCGACGCGCTGAAGCTCGAAGGCGAAGCGCGTGCCGACGCCGAGAAGCTCTTCCCG
ATCCTCTACAAGGCATTCGTCGAAAAGGACATGAGCCTGCTCGAGGTCAATCCGCTGATCGTCATGACGAACG
GCCGGATGCGCGTCCTCGACGCCAAGGTCTCCTTCGACGGCAATGCGCTCTTCCGCCATGAGGACGTGGTCGC
GCTGCGCGACACGACGGAAGAGGACGACAAGGAAATCGAGGCGTCGAAATACGACCTCGCCTATGTCGCCCTG
GACGGCAATATCGGCTGTATGGTCAACGGCGCCGGTCTTGCCATGGCGACGATGGACATCATCAAGCTCTACG
GCGCCGAGCCCGCGAACTTCCTCGATGTCGGCGGCGGCGCCTCGAAGGAAAAGGTCACCCAGGCCTTCAAGAT
CATCACGGCCGATCCGGCGGTGAAGGGCATCCTCGTCAACATCTTCGGCGGCATCATGAAATGCGATGTCATC
GCCGAGGGCGTGCTCGCAGCGGTCAAGGAAGTCGGCCTCAAGGTGCCGCTCGTCGTGCGCCTGGAAGGCACCA
ATGTCGAGCTCGGCAAGAAGATCATCAATGAATCGGGCCTCAACGTGATTTCCGCCGATGATCTGGACGACGC
CGCCCAGAAGATCGTTGCAGCCGTGAAGGGAGCCTGAATGATACGTAGAGCTTCACTTGTCCTGGTCGGGGCG
CTGATGGGCGCGACGGCGATGGGCGTCGTCTACTCGGCAACCATGCCGGCCGTGGCAGCAAATTCGTCGACAT
ACCGCGAACTGGCGATCTTCGGCGACGTCTTCGAGCGCGTGCGCGCGCAATACGTGACGCCGCCGCAGGACGA
CAAGCTGATCGAGAACGCGATCAACGGCATGCTCACGTCTCTCGACCCGCATTCGAGCTACATGAATTCGACC
GACGCCGAAGATATGCGCACCCAGACGCGCGGCGAGTTCGGCGGCCTCGGCATCGAGGTCACCATGGAGGAAG
ATCTCGTCAAGGTGACCAGTCCGATCGACGACACGCCCGCCGCGCGCGCCGGTGTGCTCGCAGGCGATTTCAT
CTCGAAGATCGACGGTCAGGACGTCCGCGGCCTCAAGCTCGAGGAAGCGGTCGACAAGATGCGCGGTGCCGTC
GGCACGCCGATCAAGCTGACGATCCTGCGCAAGGGAGCCGAAAAGCCGATCGAGCTGACGATCGTACGCGACG
TGATCGCAGTACGCGCGGTGAAGGTCCGTGTCGAAGGAGATGTCGGCTATCTCAGGGTCATCTCGTTTACGGA
GAAGACCTTCGAAGACCTGAAGAAGGGCATCGAGAAGATCCAGGCGGAAGTTCCCGCGGACAAGCTCAAGGGC
TATGTTCTCGACCTGCGCCTCAATCCGGGCGGCCTGCTCGACCAGGCGATCAATGTCTCGGACGCATTCCTGG
AGCGTGGCGAGGTCGTTTCGACCCGCGGCCGCAATCCGGATGAAACCCGCCGCTTCAATGCGACCCCGGGCGA
TCTGGCCGGCGGCAAGCCCGTCGTCGTGCTCGTCAACGGCGGTTCGGCATCCGCGTCGGAAATCGTGGCCGGC
GCTCTCCAGGACCTGAAGCGCGCCACCGTTCTCGGCACGCGCTCCTTCGGCAAGGGTTCGGTGCAGACGATCA

TCCCGCTTGGCGACGCCGGCGCGCTGCGTCTGACCACGGCGCTCTATTACACGCCGTCCGGCAAGTCGATCCA
GGGCACCGGCATCACGCCTGACATCAAGGTGGAGCAGCCGCTGCCGCCCGAGCTTCTGGGCAAGGTGGAAGCG
CAGGGCGAATCCGATCTGCGCGGCCACATTCCGGGCCAGGAAGAGACCGAAGAGGGCTCCGGCTCGGTAGCTT
ACGTGCCGCCGGAAACCAAGGACGACATTCAGCTGAACTATGCGTTCGACCTGTTGCGCGGCAAGAAGACGGA
TCCGTCCTTCCCGGCCAATCCGGAACAGGCGCAGCTGAAGGAATAGATGACCATCGCCGCCGGCGCCTTCGCC
CTCGGCGGCATCCTGCTTTTCGTCGGCCTCGCCGGCGCGGCACTGCTTGGACAGTCGGCCGGCAGCGCCGGCG
GTGGTCCGCTTTTCGATGCCTATATCTGGCGCATCACACGCTTCACGCTGCTGCAGGCCAGCCTCTCGACACT
TCTTGCCATCCTATTCGCCATACCGGTTGCCCGGGCACTTGCCCGCCAGGCTTCGTTCCCCGGCCGCATCTGG
GTGCTGCGGCTTCTGGCGCTGCCACTCGGCCTGCCGGCGCTCGTCGCCGCACTCGGGCTCATCGAAATCTGGG
GCCGGCAGGGGCTCTTGAACAGGGTGCTTTCGGCAGCCGGGCTGGAGCAGCCGGTCAGCATCTATGGCCTCTC
CGGCATTCTCATTGCCCATGTCTTCTTCAACATGCCGCTCGCCGCACGCTTGATGCTTGCCGGCATCGAGCGC
ATTCCGGCCGAATACTGGCGCACCAGCGCCAATCTCGGCATGGGATCCTTCGCCACCTTTCGCTTCATCGAGT
GGCCGGTTATCCGCGGATTGCTTCCCGGCATCGCCGGGCTCATCTTCATGCTCTGCGCCACCAGCTTCACGCT
CGTGCTGACGCTTGGCGGCGGGCCGGCCGCCAGCACGATCGAAGTGGCGATCTATCAGGCGCTGCGCTTCGAC
TTCGACCCGCCGCGGGCGATCGCCCTTTCGGCACTTCAGGTCGCCCTTACCGGAGCGCTGCTTCTCGTCCTGA
AATTGATCGCGCCGGCACCGCTGGAGGGAGAGACCAGCGGCAGGGCCATCCGGCGTTTCGACGGCGCAAGCGG
TCTGTCGCGGCTTGCCGACCGGATCTGGCTGATACTCGCGGTCATCCTCGTCGGCCTGCCATTTGCGGCCATC
GCCTATAGCGGATCGAAGGCCGACCTCGTCCGTCTCGTGAGCGAGCCGGCATTCCATCGGGCGCTCGCCATGA
GCGCGACGATCGCCCTTGTCTCCGCCACGATCTCCGTCGCCATCGCGGCGCTGATGATCCGTACCGAACGGCT
CGCGCTGGCGCAGCGGCGCCCCGGAGCGGCGCCTCGCCTCTTTGCAGGCACGATCGGCGCGAGCACCTCCTTC
ATACTGCTCGTGCCTCCCGTCGTGCTCGGCGCCGGATGGTTCCTGCTCCTGAGACCTTTCGGCGACGTCGCCC
GTTTCGCGCCGGCCGTCGTCGTCGCCATCAATGCGCTGATGGCGCTTCCCTTCGTTCACCGGGTGCTCGCGCC
GGCCATGGCGACGCATGCCGCGCGCACCGCGCGCCTTGCCGCGAGCCTTGGCATCGGCGGCTTTCACCGCCTG
CTGTGGATCGACTGGCCGGGCCTGCGCAAGCCTCTCTTTGTCGCCTTTTCCTTTGCCATGGCCCTCTCGCTCG
GCGATCTGGGAGCAGTCGCTCTTTTCGGCTCCGAGGACATGGTAACCCTCCCCTATCTGCTCTATAGCCGGAT
GGGCAGCTATCGCACCGCGGATGCGGCCGGACTGGCGCTGGTTCTCGGCCTTCTCTGCCTTGTCCTGACGGTG
CTCGGCACGGCGGGAGAGGAGAGGCCGCCCGAAGGTAAAAGCACATGAGTGAAAGAGAATAGTTTCGTATCCG
CCGACGATATTCGCTCGGCCTTTTCCGCTGCGATGTCGTTGATGTACCGCGAGGAAGTGCCGGCCTACGGCAC
GCTGATGGAGCTCGTCGCCAGGGTCAACGGCGAAACGCTCTCGGCCGACGCCACCCTGAAGGGGCGCCTGGAA
GCGACCGATTCGCTCGAACGCATTTCCGAGGAGCGTCACGGCGCGATCCGTCTCGGCACGCCGGCGGAGCTTT
CGATGATGCGCCGGGTATTTGCCGTGATGGGCATGTATCCGGTCGGCTACTACGATCTGTCGACCGCCGGGGT
GCCGGTCCACTCCACGGCCTTCCGGCCGGTGGGCGACGCGGCTCTGAAGCACAACCCCTTCCGCGTCTTCACC
TCGCTTCTGAGGCTCGACCTCATTTCCGACGAGGCCCTTCGCGCCGAGGCGGAGGCGATCCTGAAAGAGCGCC
GGATCTTTACATCCGGTGCGGTCGAGCTTACCGAAAAGGCCGAGCGGGACGGCGGCCTCGACAAGGCCGATGC
CGAGCGTTTCGTCGCCGAAGTGCTCGAGACCTTCCGCTGGCACGACGAGGCGAATGTCAGCGCCGGCATGTAC
GAGCGCCTGCACGATGCGCACCGGCTGATCGCCGACGTGGTCTCCTTCAAGGGGCCGCATATCAACCATTTGA
CGCCACGGACGCTCGACATCGACCGCGTCCAGGCGCTGATGCCGGAATATGGCATTGCCCCGAAGGCCGTCGT
CGAAGGACCGCCGACACGCAAATGCCCGGTCCTGCTTCGCCAGACGTCCTTCAAGGCGCTCGAAGAGCCGGTT
TCCTTCCGCGACTCCGACGGCAGCTGGAAGACCGGCTCGCATACCGCCCGCTTCGGCGAGATCGAGCAGCGCG
GCATCGCTTTGACGCCTAAGGGCCGAGGTCTCTACGACCGGCTGCTCGACGAATCGCGCAAGATCGTACGCCC
GGCCGCCGATGGTTCAAACGCCAGGGAATACGAAGCGGCCTTGGCCCAGGTCTTCGAGGCGTTCCCCGACAGC
TGGGCGGAGCTCCGCGAGGCGGGCCTCGGCTATTTCAGCTACTCCCTGACGGATAAGGGCCGGCGGACGAAGC
TGCCCGGCCGGCGTGATCTCAATTCCCTGATCGCCGACGGCCTCGTTCAATTCGATCCGATCGTCTATGAGGA
CTTCCTTCCCGTCAGCGCCGCCGGTATCTTCCAGTCGAACCTCGGCGACGGTGCGCAGCAGGAATTCGAAGCG
AGCCCGAACCAGAAGCGCTTCGAGACCGATCTCGGCGTCGCCGTGCTCAACGAATTCGATCACTATGCCGGCA
TCGAGCAGGCATCGATCGAAGGCTGCCTCAAGGCGCTTACCGCCGCCATGGCAGCGGAGTGAATGATTGACGA
GCACCATATCGACGCATTGACGAAGCTCCTTGGCGACAAGGGCGTCGTCACGCGTCGGGAGGACATGGAGGCT
TACGAGGCCGGCGCACGCTACGACCGCGGCCGCGCGGCGGCCGTCCTGCGTCCGGTAACGACGGAAGAGGTCT
CGGCCGCCGTCGGTTACTGCGTCCGCAGCGGCATCGCCCTCATCCCCCAGTCGGGCAATACGGGCCTCGTCTC
GGGATCGACGCCGGACGAGTCCGGCAGCGAGGTCGTTGTCAGCCTCGACCGGCTGACCCGCCAATTCGCGCTC
GACCTCGACAATCGCTCCGTGCGCGTCGATGCCGGCTTCCGCCTGTCCGAGCTCAATCGGCGCCTGGAGGAAC
ACGGCCTGTTCTTCCCGATCGATCTCGGCGCCGACCCGCGGATCGGCGGCATGATCGCCACCAATACCGGCGG
CTCGCGCTTCCTCAAATATGGCGACGTGCGCCGCAACACGCTGGGTCTGAAAGTGGTCCTCGCCGACGAGGCG
GGCACGGTGCTCGACCTCGGCTCGGACCTGCGCAAGAACAATACCGGCGTCGACTGGAAGCAGATCTTCATCG
GCACCTCGGGCGCCTTCGGCATCGTCACCGAATGCGTGCTCAATCTCGAGCGCCTGCCGAAGCAGACGGCGAC
CGCCTTCCTCGTGCCGGCGAGCGGCGCGCATGTCCTGCCGCTCCTGAAGGCCATGGAAGAGCGGCTCGGCGCC
TATCTCTCGGCCTTCGAGGGAATGTCGAGGAACGCGATCGCCGCGGCCTTCGCCCATGTGCCGGCGCTGAAAA
ACCCCTTCCAGGGCGGCAAGGTCCCCGACTACGTGATCCTCGCCGAAATCTCCCGCACCTCGAGCCCGCGCGA

AGGCGAGCAGCCGCTCGACGCGGTTCTCGAAAGCGTGCTCGCGGAGATATGGGAGATGGAAGAGGTACTGCTC
GCCGATGCACTCGTCGGCCCGCCGCACGAGATCTGGGCGCTGCGCCATGCGCTTTCGGAGGGCGTAAAACACC
TCGGCAAGCTGATCGCCTTCGATCTCTCCTTCCGCAGGGGTGACATCATGGCCTTCTGCGACCATATGAAGAC
GGAAATGCCGGAAAAATTTCCGGGCGTCACGGTCTGCGATTTCGGCCATATCGGCGACGGCGGCGTTCACTTC
AACCTGGTCGTCGCCAAGGATAGTCCGCTACTGGCCGATGCGACCTTCGAGCAGCGACTGCGCGAATGGGTCT
TTGCGGTGGCGGTCGAGCAATATCACGGCAGCTTCAGCGCGGAACATGCGCTCGGCCGCCGCAACCAGGCTTT
TTACGATCTCTACACGCCGGAAAAACTGAAGAACATGGCGGCCGGCCTCAAGACGCTCACCTCGCCGGGCAAG
CTCGGCAGCGTGCGCTTCGGTTAGATGGAGAGCCAGCCGGATATCGTCATCATCGGATCGGGAGTCGGCGGCG
CAACGGTCGCTGCGGGCCTTGCTGCCTCGGGCGCCGAGATACTGATCCTCGAAGCGGGCTCGCATATCGAGGA
CCTGCCGGTCAATCGCGACCAGCGGGCGATCTTCCAGCGCGGCCATTTCCGCCCGAAGGAGACCTGGTACGAG
GCCGGCGGCCCCGGCTTCAATCCCGGCAATTACTACAATGTCGGCGGCAATTCGAAGTTCTACGGCGCGGTGC
TGACCCGCTATCGCCGGGAGGATTTCGAGGAGATGCAGCATCGGGACGGCGTCTCCCCGGCCTGGCCCTTTTC
CTATGAGGAGCTCGAGCCGTGGTATTCCAGGGCAGAGCAGCTTTACCAAGTGCGAGGCAAATTGGGCGAGGAT
CCGACGGAGCCCGCTCATTCGAGCGGCTATCCCCATGGCCCGGTGCCGGACGAGCCTGCGATCGCCATGGTCC
GCGAGCGCCTGGCCGAGATCGGCCTCCATCCCTATTCGCTGCCGCTCGGCGTCGATATCGAGCGCTGGCTCGC
CAAGGGCAAGACGCCGTGGGACGCGCATCCGAACTCGTCCGACGGCAAGATGGATGCCGAAACGGCGGCGCTG
GCGGCGGCGCTGAAATTTCCGAATGTGCGGCTGCAGACCGGCTCGCGAGTGACCCGGCTCGTAACCGGGCCGG
ACGGCAAGGCGATCGAAACCGTGCTCTACGCCAGGGACGGGGCAGAGCACAGTCTTTCGCCGAAGCTCGTCAT
CCTCTGCGCCGGCGCGGTGCAGTCGGCCGTACTGCTCCTGCGCTCGGCCGACGACAGCAATCCGACCGGGCTT
GCCAACCGGTCCGATCAGGTCGGCCGCAATTTCATGAACCACAATTCGAGCGCCGTCATCGCGCTCAGCCCCT
GGTACCGGAACGACTCGGTCTATCAGAAGACCTTCGGCCTCAACGACTTCTATCTCTCCGACGGTGCGGGCGG
GCCGCCGCTCGGCAATGTCCAGCTTCTCGGCCGCATTTCCGGCGCGATACTGAAGGCGAACATGCCGGCAATG
CCGGAATGGCTGCTAAACCGGGTGTCCGCCCGCGGCATCGACTTCTACGCCATGAGCGAGGACCTGCCCTCAC
CGGAAAGCCGCGTCATGGTCGACGGCGAGCGCGTTGTGCTGAAATGGGTGCGCACCAATTGGCAGGCCCATCT
CGACCTCGTCGCCAGGCTCAAGGCGGTGCTGAAGAAAGCCGGCTTCCCGATCGTGCTCGCACGGGCCTTCGAC
AAGCGGACGCCCTCGCACCAGTGCGGCACGGTCCGGATCGGCAATGATCCCGGCCAGGCGCCGCTCGACGTCT
ATTGCCGCGCCTTCGATCATCCGAATCTCTTCGTCGTCGATGCCGGCTTCCTGCCGACCTCCGCCGCCGTCAA
TCCGGCGCTGACCGTCGCGGCACAGGCGCTGCGTGTCGCCGACCATATCGTGAAACAGGATCTGCGGCCATGA
ATGAGCGAGGTCAAGTCGCACGAATTCGATAGTTTCCTGCAGCGATCTGCGGCGATCTACCGGCTATTCCTGC
TCTACGGTCCCGATCGCGGCCTCGTCTCGGAACGCGCGTCCGAGCTCGCCGGCAGCTTCGGCATCCCCCTCGA
TGATCCCTTCGCCGTCGTCAAGCTGGACGCAGCTACCTTGCAGGGCGCCGGTAGCGTGCTTGACGAGGTCAAT
GCCATCGGCCTGTTCGGCGGCGACAAGCTGGTCTGGGTGCGCGGCGCGTCCGCTGAGAAGGCGCTCACCGAAG
CGCTGCAGATCCTCGCGGCCGATCCCCCGGCCGGCGCCCGTCTCATCGTCGAAGCCGGCGACCTCAAGAAGGG
GGCGGCGCTCAGGAAGGTTGGCGAGACTTCCCGCTCCGTCGCTTCGATCGCCTGCTACAGCGACGACATCAGG
GGCCTGCAGGCACTTGTCGACACGGAACTTTCGGAAGCGGGCCTGCGCATCGGTCCCGCCGCGCGCGAACGCC
TCGTCGAGGCGCTCGGGGGCGACCGCATGGCTTCGCGCAACGAGCTTCGCAAACTCGCCCTCTATTGCCGCGG
CAAGGATATGGTCGATGAGGATGACGTCCTCGGTATCGTCGGCGACGCCAGCGCCATTTCGGTCGACGATGCG
GTCGATGCGGTGTTGAAGGGCGACGCCGACGCGCTGCTGCATGCGATGAAGAAGATCACGACATCCAAGACGC
CGCCTTTCCTGGTGCTTCAGGCCTGCCTCAGGCAATTCCAGCAGCTCGACGTGATGCGAGCCGAAATGGATGC
GAGCCGCCAGTCTGCGGGCCAGGTCGTCGCAAGCCTCGGCCGCGGCCTGCACTTCCGCCGCAAGCCGGTTGTC
GAGGCGGCGCTGAAACACTGGACCGGGCCGGCGATTCGACGCGAATTGGGCCGCCTGCAGGCGACGATCTATC
AAAGCCGCAGCCGCCAGAGCCTCGAGGAAAGCCTGGTGATCCAGAATCTCCTGGCGATCACCATCCAGTCGGC
GAGGCGTTGAATGCCGCCCGACACCGCGCTTGAAAGCTTGCTGCGACGGGATCGGGCAATCGTGGCAGCGTCG
CTCGTCGCATTGACGGTGGTCGCCTGGACATACACGCTCTGGCTGGCGGCAGCCATGAACATCGACGGCATGA
GCATGTCTGCCCCCGGCATGGAAGCGGACATGAAGATGGACCCTGCCATGGATATGGATGGCATGGAGATGGG
ATCCCGTGGCGCCCTGTCGCTCGGCACCGTCCTCGGCATCTCGCCGCGTCCCTGGAGTTCCGTGGAAGCCGGC
GTCACCCTGACCATGTGGATCGTGATGATGGTCGGCATGATGCTGCCTTCGGCAACGCCGATGATCCTGCTCT
ACGCCCGGGTCGGCCGGCAAAGCCGGATAGCGGGCAAGCCCTTCGCCGCGACCGGTTTCTTCGCCGGCGGCTA
TCTCCTTGCCTGGGCGGGATTCGCGCTCGTCGCGACGCTTGGACAATGGCTCATGGAGGGAACCCTGCTGACG
CCGGCGCTGGCAAGCGCCAGTCGCATCTTCAGCGGTGTCGTGCTGGTGATCGCGGGTCTCTACCAATGGACGT
CGCTCAAGGATGCCTGCCTCAGCCAATGTCAGACGCCGATCGTCTTCCTGCAGCGGCACGGCGGTTTTCGCCG
CGATCCCGCGGGAGCCGTCGGACTGGGCCTGCGCCACGGGGTCTATTGCATCGGCTGCTGCTGGGCGCTGATG
ACGCTTCTTTTCGTCGGCGGCATCATGAATGTGCTCTGGATTGCGGCAATCGCCATCTTCGTGCTCGCGGAGA
AGGTGATACCGACCGGCCGCGTTCTCTCACGCGTCGCCGGATCTCTGCTCGCGGCGTTCGGCCTTTGGCAGCT
CATTTCGGCTTCGATTTAAATGACTGCGAAGAAGACGATCAGCGGAGCGCGGATTTTCGACGGCATCGACTGG
CACGACGGGGCAGCCCTCGTGATCGAGGCAGGACACGTCAAGGCGATTGCGCCGGCGGGAAGCGTCCCCGCCG
GTGGCGAGACCATCGACGCGCGTGGCCTGCTTCTCGTGCCCGGCTTCATCGATCTGCAGGTGAACGGCGGCGG
CGGCGCGCTTCTGAACGAAAAGCCGTCCCTCGAAAGTATCCGGCAGATCTGCGCGGCGCATGCGCAATTCGGC

ACGACGGCGCTGCTGCCGACGCTGATCACCGACACGCGCGCGGTCAGGACCGCGGCCATCGCCGCAGGCATCG
AGGCGAAAGCCGCGGCGGTTCCCGGCTTCCTAGGCCTGCATCTCGAAGGCCCGCATCTTTCCGTCGCGCGCAA
GGGGGCTCACGATCCGGCGCTGATCCGTCCGATGGGCGATGACGATCTTGCCGAGATACTCGCCTGCGCAAAG
GCGCTCAGCCGTCTGATGCTCACCGTGGCCCCGGAAAACGCCACCAAGGAGCAGGTGCGGGCGCTGGCCGATG
CCGGCGTCGTGGTGAGCCTCGGCCATACCGACGTGGATTACGACACCGCCTGCGCCTATGCCAAAGCGGGTGC
CCGGACCGTCACGCATCTCTTCAACGCCATGAGCGGACTCGGCCATCGCGAGCCTGGCGTGGTCGGCGCGGCG
CTTTCGACCGGCACGCTCCACGCCGGCATGATCGCCGACGGTTTTCACGTTCACCCGGCCTCGATGGGCATCG
CGCTGCGCGGCAAGAAAGGCCCGGGACAGATCTTTCTGGTCACCGACGCCATGTCGCCGATCGGCACCGATCA
GACGAGCTTCTTCCTAAACGGACGGGAAATCCTGCGCCAGGGCGGGCGCCTGACGCTTGCCGACGGCACGCTT
GCCGGCGCCGATATCGACATGCTTTCGTCTGTCCGCTTCGTCCACCAAAAGCTTGGCCTGCCGATCGAGGAGG
CGGTCCGCATGGCCTCCGCCTATCCCGCCGACGCCATGGGGATCGCCTCGCACAAGGGCCGGCTCCTGCCGGG
TACGGATGCGGATTTCGTGCTGCTCACGCCGGAACTCGCAATGAAATCGACCTGGATCGGCGGGGAGACGGTT
TTTGCGGCTTGAATGAGCGACGAAGCGCCCACTTCATCCCCGATTTTACCCGTCGAGAAACCGGCCGCCTACC
GTGTTCTGGCGCGCAAATACCGCCCCAAGGACTTCTCCGACCTGATGGTCGGCCAGGAGCCGATGGTGCGCAC
GCTCACCAACGCCTTCGAAACCGGCCGCATCGCCCAGGCCTACATGCTGACCGGCGTTCGCGGCGTCGGCAAG
ACGACGACGGCGCGTATCCTGGCACGGGCGCTGAACTACAAGACCGCCGAGATCGACAAGCCCACCATCGACC
TGCGCGTCCCCGGCGAACATTGCCAGGCGATCATGGACGGCCGCCATGTCGACGTGATCGAGATGGACGCCGC
CTCGCATACCGGGATCGACGATATCCGCGAGATCATCGAGCAGGTACGCTATCGGCCGGTCTCCGCGCGCTAC
AAGGTCTATATCATCGACGAAGTGCACATGCTCTCGACGCAGGCCTTCAATGGCCTGCTGAAGACGCTCGAAG
AGCCGCCGGAGCATGTGAAGTTCATTTTCGCCACGACCGAAATCCGCAAGGTTCCGATTACCGTCCTGTCGCG
CTGCCAGCGCTTCGATCTCCGGCGCATCAGCGCTTCCGATCTGGTCGGCCTCTTCTCCACCATTCTCGGCAAG
GAGGGCGTGCCCTTCGATCCGGAGGCGCTGGCGATGGTGGCGCGGGCAGCGGAAGGCTCGGCGCGCGACGGCC
TGTCGCTGCTCGATCAGGCGATCGCCCATGGCGGCGGTTCGGTCGAGATCGAAACCGTGCGCTCGATGCTCGG
CCTTGCCGATCGGGCGCGGATCGTCGACCTGTTCGAGCATGTCATCAAGGGCGATGTCGCCGGCGCGCTCGAC
GAGTTCGCCGCGCAATACGAAGCCGGCGCCAATCCGACCGTGGTCCTCACCGACCTTGCCGACTTCACCCACC
TCGTGACAAGGCTGAAATATGTGCCGGACGCGGTCAACGACCAGTCGCTGAGCGAGATCGAGCGCACGCGCGG
GGCCGAATTCGCCGGCAGCGTTGCCGTGACGACGCTTTCGCGCGTCTGGCAGATGTTGCTCAAGGGCATTCCC
GAGGCGGAGAGCTCTGCGCGCCCCGCAGGTGCTGCCGAGATGGTGCTGATCAGGCTCGCCCATGCCGCCCATC
TGCCTTCGCCCGAAGAGGCCGCGCGGCGGCTTCTCGATCTTTCGGGCGGCGAAGGCGGCGGTCGCCCGGCCCC
GAACGGCGGTGGTGGCGGCGGCGGTGCGCAGGCGCCTGCAGGCACCCCCGTCGAGGCTCGCGCCGTGGAGACG
GCAGTCTCGCGTCCAAGCGGTAATGGCGCGACCATGCTGCGCGCCGTGCCGAGCTCCGCGCCGCCCCAGCCGA
TCAGTGTCGGCCGCATCGAAGAGCGCACGGTCGCGAGCGCCGCTGCGAAACCCGAACCGAAGGTTCCCGTCAA
TTCCATCGGCGACATCGCTGATCTCTGCGCCAAGAATCGCGACATCAAGCTGAAGACGATGGTGCGCGGTTTC
CTGAGGCTCGTGCATATCGAACCCGGCCGGCTCGACGTCAATCTGCCCGACGACGCACCGAAGACGCTGCTGA
ACGAACTCGCCGTCAAGCTCAAGGAATGGACGGGTATCCATTGGGTCGTGAGCTACAGTCGGGAGCAGGGCGA
GCCGACCCTTGTCGAGGCAGAGCAGCGCGCGCAGGAGCAGCGCGTCAACGATGCGCGCCAGGACCCGGACGTC
GCTGCCATTCTTGCGCGCTTTCCCGGGGCCAGGATCACCGACGTGCGCATCCGCGCGGCGGAAGAGGAGCTCG
AGAGCCTCGCTCCGGCCGCGGCCGAATCCGAGGACGGCGACATCGTCCCGGACGACGACATCGAGTAGATGAC
CATTACCGAGAGCAGGAATGCGGCCGTTGCGGAGATCGAGGAGGTAATCGGGGACTGGGCGGAGGCGCTGCGC
CGGAAGGATGCCGCCCGCGTCCTCGCCCACGGCACGGAAGATTGCCTCGTCTTTTCGCTGGCGCCCCCCCTCA
AGGCTTCCGACGCAGATGCCGGCGGCCTTGAGCAGTGGTTTTCCACGTGGAAAGGGCCGCTCGGCTTCACCCT
CGAGGAGCTCGACGTTTCGGTGAGCGGCGACGTCGCCTTCGCCACTGCGCTGACGCATCTTTCCGGCGAGAAG
CACGATGGCCAGAAGGCGGCGCTCTGGTATCGCCTGACCTTGGGCCTGACACGCACCGACAGGGGATGGAAGA
TCGCCCATCAGCACGAATCCGTCCCCTTCTACATGGACGGAAGCTTCAAGGCGGCGATCGATCTCGATCCCCG
GTCCGAGGTCGACTGGCAAGCGCCGTCGCGCCCGCCCATGCCCGGCGTCATCGCCTATCTCAACGTGGAGGAT
GCAGATGCGGCGGCGGCCTTCTATGTCCGCGCTTTCGGCGCCAAGGAGGAGGACCGCAAACATGCCCAGGATG
GAAAGCGCCTCATCCACTGCCACCTGACGATCAATGGCGGCGCATTGATGCTGAACGATCCTTTCCCGGAATT
CGGCCATCCCTGGAAGCCGCCGGAGGGCTTCGTGCTGCACCTCGTCGTCGACGACGCTGATTTCTGGTGGAAT
CGGGCGGTGGAGGCCGGCGCAGAGGTGACGATGCCGCTGGAGATCGCATTCTGGGGCGACCATTACGGCCAGC
TCCGGGATCCCTTTGGAGTTACCTGGGGGATCGTCGCACCGGTGAAGGAAGGCGGCTGAATGTTCAAGTTCCA
AGCCAGATCGCTGGCGACAAAGTTGATCGCGGTGACGGGAGGCACGATCGCCCTCGTGCTGCTCGCGTCGAAT
TACGTGCTCATCTCCCAGACGCAGGACCGTGTGGAAACGCTGGTCTTCGACGGCGCGAAGACGGAAGCGCGGG
CGATCGCGTCCGACATCGCGGGAAGCGTCGGCGAACTTGCCGCCGCCGCGCGGACCATGTCGGGCGTTCTGGG
GCGCGGCCATGCCGGGCAGTCCACCGACCGCGCAGGCGCCATCAATCTCCTGAAGGCCAATCTGGAGCAGCAT
GGCTTTGCCTTCGGCAGCTGGTTCGCCGAAGAGCCCAAGGCCTATGACGGAAAGGACGTCATCGACAACACGG
AACGTGGCGGCAATGCCGACGGTGCGTTCACGCCCTATTGGTCCAAGGACCGCAACGGAAATATCCAGCTCTC
GACCTTCAAGGCCGATTATGCGGCCGAGTGGTACGGCCTCGCCGCGAAGAGCGGCAAGGGCGCCATCACCCAG
CCATATCTCGCCGAGGGCACCGACGTCCCGACCACGATGACGTCGATCGCCTACCCCGTCATGTCGAACGGCA

GGATGATCGGCGTCTCGGGTGTCGATATCTCGCTCGCCGCACTCGCCGACCGTCTCTCGGCGGTCAAGCCTTT
CGGCTCCGGCCGGGTCTACCTGCTGTCGCAGAGCGGCAAGTGGCTCGCCGCACCTATCCCCGAACTGCTGATG
AAGGAATACGACGGCGAAGGCGTTGAGTCCGTGAAGGATGCCCTTTCCACCGGCACGCCCCGCATGATCGAGA
ACCTGACTTACGACGGCAACGAGCCTTTCGACCGAGTCGTCTATCCTTTCAGCCTGCCCGACGTGAACGCGCA
GTGGCTGGTTCTGGTCGATGTTCCGCGCTCAGCCATCAATGCGCCGGTTCGCGATCAGACCTATATGATGATC
GTCGGCGGCCTGGTGGTCCTCGGCGCCGTGCTCGCCGGCCTCTATTTCGCCGTTCGCCGCTTCGTCCAGAAGC
CGCTTGCCGGACTCGTGGGCGACGTCGAGACGCTCAGCAACGGAGAATATACCCGGCCGATTTCCGGTCAGGA
ACGCTCCGACGAGACAGGCGCCGTCGCCAGGTCGCTCGAGGGCTTCCGTCACCAGCTTGCCGACAACAGGCGG
CTCGAAAGCGAGGCCCGCCACGAGAGAGAACAGGCCGAACTGGAACGCGGACGTTCGGAAAACGAACGCACCG
AGGCCAGCGCGCTGCAGCGCGACATCGTCGCACGCCTTGGCAAGGCCCTGTCGCATCTCTCCGCCGGTGACCT
CGCCTTCCGCCTTACCGGCGACTTCCCCGGCGAATACGCGCAGCTGAAGCGCGATTTCAACGCGACGATGGAG
AGCCTCGAAGAGACGATCCGCACCGTCAACCACTCCGTCGTCAATATCGGCAGTGGCACCAGCGAGATCTCGG
GCGCCGCCAACGACCTGTCGCATCGGACTGAACAGCAGGCAGCAAGCCTCGAGGAGACCGCGGCCGCCCTTGA
CGAACTGACCTCGCAAGTGAACGCCAGCGCCGAGAACGCCAAGGTCGCCGCGAAGTCCGTCGAGGTCGCAAGC
AGCGACGCGGAACAGTCCGGCGAGGTGGTGCAGAAGGCGATCGCCGCCATGCAGGGTATCGAGCAGTCGTCGC
ATGAAGTCAGCCGGATCATCGGCGTCATCGATGAAATCGCCTTTCAGACCAACCTTCTGGCGCTCAATGCCGG
GGTCGAAGCCGCCCGCGCCGGCGACGCCGGCAAGGGCTTCGCGGTCGTCGCACAGGAAGTCCGCGAACTCGCG
CAGCGCTCCGCCAATGCCGCAAAGGAAATCAAGACGCTGATCAATACATCGGCGGGCCAGGTTCGCGAGGGCG
TCGACCTCGTCGGCAAGGCCGGCGGCGCGCTCGAGAAGATCGCCGAGCAGGTGGTGCAGATCAACGGGCTCAT
CCGCCAGATCTCCAGCTCCGCCTCGGAACAGGCGGTCGGCCTCAAGGAGATCAATTCCGCCGTCAATCAGATG
GACCAGGTGACACAGCAGAACGCCGCCATGGTGGAAGAGACGACTGCCGCCAGCATGGCGCTGAACGACGAGG
CCCGCGCCCTCAGCGCGCTGGTCGCCCGTTTCCAGATCGCCCCGCAGGCTGCCCAGGCCCAAGCCTCGGCCGA
AATGCTGCGCGGTACGGCCGAGCGGATGCGTGCGGCTGCTCCCGCGGAAAATCGTCCGGCACAGGCGCCGCGC
AGTGCCGCCTACTCCAATTCGACTCAAAGAGTGCTTGCCAAGACATCCGGCGCCAATGCGCTCGCGCAGGACA
ATTGGGAAGAATTTTGAATGACAGCAGATACGACCATAGATCCCGCGGCGCCAAGAACCTTCCTGTTCTTGCA
GGGACCGGCGTCGCCGGTCTTCGCCAAGGTCGCCGGGCATCTCGAAGTGCTGGGTCATTGCTGTCTGCGCATC
AACCTCAATCCCGGCGACCAGATATTCTGGCGGCGCCGGGGCGGCCATAACTACCGTGGCTCGTTGGCGCGGT
GGCCGGCCTATGTGGAGGCCTTCATCCGCCGCCACGCCGTCACCGACCTCGTCCTGCTCGGCGAAGAGCGCTC
CTATCACCGGATCGCCACAGCTGCCGCGCGAAAAGCCGGCGTCCGAGTCTTTGTGGTCGAAATGGGTTATCTC
AGGCCGGACTGGTTGACGCTCGAGCGCGGCGGCATGTCGTCCAATTCGCACTTTCCGGTCGATCCCGCGCAAA
TCCTGCGAGCGGCAACGGGATTGCCCGAGCCCGACTGGCAACGGCGCTACCGGCAGAGCTTTCTTGCGGAGGC
GGCATACGACCTCCTCTACAACCTGCCGAACGTGTTTTTCTGCTTCCTCTTTCCCGGCTACCGCCGGCACGGC
ATCTTTCATCCGCTCGCCGAATATGCCGGGTGGATCCGGCGGCTGGCGGCCGGCAAAGGCGAGCAGCGGCGCG
CGGATGCGTTGATCCGCTCGCTTTCGGCGAAAAATGCGCCGTACTTCGTCTATCCGCTTCAACTTGAAACGGA
CTATCAATTGCGTGCGCATTCACCCTTCGGCAGCCAGCGGGAAGCGATCGACGGCATCCTTGCCTCTTTCGCG
CGCCGTGCGCCGGCAGGAACCAGACTCGCCGTCAAGGTGCACCCGCTCGACAACAGCCTGATCTCCTGGCGCA
GGATCGTCGCGGACAAGGCCGCCTCACTCGGTGTCGGCGACAGGGTCCTCTATCTCGACGGCGGCAATCTCGA
TTCGCTCACGGAGCACAGTTGCGGAATGGTAACGGTCAACTCGACCGCCGGACTGCATGCTCTGAAGCAGGGC
AAGCCCGTGAAGGTCCTGGGAAAGGCCGTCTTCGACATTGCCGGTCTCACCGACCAGCAGCCGCTCGACGCGT
TCTGGACGGCGCCCGAGGCCCCGGATCCGGTGCTGAGCGGGGCAATGTTCAGGCTGATGGCCGCTTCGATCCA
GGTGCGCGGGAACTTCTATTCCCGTGCGGGTACGGATGCCGGCGCCAAGGCGATCGCCGAGCGGCTGCACCGC
AACGACGTCAATGAACCGGGCGCCTTCGTCGACCCGCCACCCCGGCAAAAGCCGGTGAGACTCAGCGAAGATC
AGGCGACGCCGGCGCGCAGCAGATCGTGATTGCAGACCGGTCTTTATGTCGCGCTTTCGTCGCAGATGGCGCT
TGAAAAGCGCCTGAACACGCTTGCCGACAATATTGCCAACTCCAACACGGTCGGCTTCCGCGCGACGGAAGTG
AAGTTCAACCAGGTGCTGGGCGACACCAAGCCGACAAAGGTCTCCTATGTGTCGGAGGGCGAGGAATTCCTGA
GCACCAAAACCGGCGCGCTCGCGCGCACCGGCTCCGCACTCGATTTCGCCATCAAGGGTGACGCCTGGTTCTC
GATCGACACGCCCGGCGGCCCGGCGCTGACGCGCGACGGCCGGTTCACCCTGACGGAGACCGGCGAGCTCGTA
ACCATCAAGGGATATCCGGTGCTCGACGCCGGCGGCGCGCCGATCCAGCTGAACGGCGGTGCCGGTGAAATTG
CCGTCGGTGCCGATGGTGCCATTCACCAGAACGGCGTCCAGATCGCCCTGCTCGGGCTTTACGAGGCGGATTT
CAGCAAAGGCTTCATGCGTTACGACAACAGTTCCGTTATGCCGGCCGCCCAGCCGGAACCGGTCGTTGACCGG
TTCGATGTCGGCGTGATACAGGGCTTCCTGGAGGAATCGAACGTGAACGCCATACAGGAAATGTCGCAGCTGA
TCATGATCACCCGCGCCTTCGACAACGTCACCGCACTTATGCGCGACAGTGAGGGATCGCTCGATAAGGCCAT
CGAGACGCTCGGCGGCAGCCGTTGAATGCCGGAGACGATCGGCTTTCTGAAGCCAAGGCAGGCCGTCCTCGAT
CGTCGGCGGGAAATCGTCGCCGATCTTGCCGATCTGTTGCCGGAAGGCGGCCTCATCAGCGACGAGCGCGGCC
TCAAGCCCTTCGAGACGGATGCGTTTATCGCCTATCGGCGGATGCCGCTCGCGGTCGTTCTTCCCGAGACGAC
GGAGCACGTGGCCGCGGTGCTCAAATATTGCAGCCGCTACGGCATTCCGATCGTGCCGCGGGGCGCCGGCACC
TCGCTCTCCGGCGGCGCCATTCCTCAGGAGGACGCGATCGTCGTCGGTCTGTCGAAGATGTCGCGCACCCTGG
ATATCGACCTGTTCAACCGCACGGCGACGGTTCAGGCGGGCGTGACCAATCTCAATATTTCCGATGCGGTTTC

GGCCGACGGCTTCTTCTATGCCCCGGATCCGAGTTCGCAGCTCGCCTGCACCATAGGCGGGAACATCGGGATG
AATTCCGGCGGCGCGCACTGTCTGAAGTACGGGGTTACGACCAACAACCTTCTGGGCGTCAAGATGGTGCTTT
TCGACGGCACCGTGATCGAACTCGGCGGCAAGGCGCTCGACGCGCCGGGATACGACCTGCTTGGGCTCGTCTG
CGGTTCGGAGGGGCAGCTCGGCATCGTCACGGAAGCGACGGTGCGGCTGATTGCGAAGCCCGAAGGTGCGCGG
CCCGTGCTCTTCGGATTTGCCTCTTCCGAGTCCGCCGGCAGCTGCGTCGCCGATATCATTGGATCGGGCATCA
TCCCCGTCGCAATCGAATTCATGGACAGGCCGGCGATCGAGATCTGCGAGGCCTTTGCCCAGGCCGGCTATCC
GCTCGACGTCGAAGCATTGCTGATCGTCGAAGTGGAAGGCTCCGAGGCTGAGATGGATGCGACGCTCGCCGGC
ATCATCGAGATCGCCCGCCGCCACGGCGTGATGACGATCAGGGAATCGCAATCGGCTCTGGAAGCGGCACTGA
TCTGGAAGGGGCGTAAGTCGGCCTTCGGCGCAACCGGCCGGATTGCCGACTATATCTGCATGGACGGAACGGT
GCCGCTGAGCCAGCTCTCCCATGTCTTGCGCCGGACCGGGGAGATCGTCGCCGGCTACGGCCTGCGGGTAGCG
AATGTCTTCCATGCCGGCGATGGCAACATGCATCCGCTGATCCTCTACAACATCAACGATCCGGAGGAAGCTG
CCCGCGCGGAAGCTGCCGGCAACGACATACTGAAGCTTTGCGTCGAGGCGGGCGGCTGCCTGACCGGGGAACA
CGGCGTCGGCATCGAGAAACGCGATCTGATGCTGCACCAGTACAGCAGGGCCGATCTCGGCCAGCAGATGGCG
GCGCGCGCCGCCTTCGATCCGCAATGGCTTATGAACCCGTCGAAGGTTTTTCCGCTCGAAGGACGGCCCGCGG
CATGAATGAAAGAGAGACTTATATCTTCCGTTGCCGCTGGCGCGCTTCTCGTGGGCGTTGCCGTTGCCCCGAT
GAGCTTCGCACAGCAAACCACGACGCCGCCGGCTACGGCACCTGAAACCCAGACCATGGAACCGGCTCCGGCT
CCGGCCCCGGCTACACCGCCGGCCGCCGACGAGCAGGCGCAGACCCCGGATACGGCAGACAAGACGACCGATA
TGGCGGCTGGTGACGCCACCTACCTGACCGAGCAGGCGGAGAATCAAGTTTCCGCCAACACCTATATCGGCCA
GTCCGTCTACAATGCCAGCGATGAAAGCATCGGCGAGATCAACGACCTGATCATCGAGAAGGAAGGCGGTATC
GCTGCAGCTGTCGTCGGAGTTGGCGGCTTCCTCGGTATCGGGGAAAAGAACGTCGCAGTTCCGTTCGACAAGA
TCGCAGTCGCCGACCAGCCCGACTCGGATGACATCAAGCTGACGACCACCGAAGCGGCCGACTCTCTGAAGGC
CGCACCGGAGTTCAAGACGAAATCGCAGCTGATGGCCGAGCGTAACGCCGACCAGCCGGTCGATACGTCGACG
ACTTCCGCAACGGGTACAACCCCGGCACCGGCGCCTGCGGAACCTGCCCCTCAGCAGTAAATGACCCTTCCGA
CAGAAATGACCTACGTCGATCTGCCTGGCCCCGGAGGGCCGGAGAATATGGTGCTGGCGCGAGGGCCGCTGCC
TGAGGTCCGGCCCGGCGACATTCTGATCCGCGTCGAAGCTTTCGGCATCAATCGGCCGGACGTCCTGCAGCGC
AAGGGAGACTACCCGCTGCCCGCTGGGGCGAGCCCCATCCTCGGGCTCGAAGCTGCGGGCGAAGTGGTTGCTC
TCGGCGAGGGAGCGACCGGCTTCTCGACCGGCGACAAGGTCTGCGCTCTTGCCAATGGCGGCGCTTACGCGGA
ATATTGCGCGGTTCCCGCCACGCAGGCCCTTCGCTGGCCCAAGGGCTACGATGCCGCGAAGGCGGCAGCCCTG
CCGGAGACCTTCTTCACCGTCTGGGCCAACGTCTTCGACATGGCCCGCCTGAAGTCCGGCGAAACATTCCTCG
TCCATGGAGGATCGAGCGGTATCGGTACGACCGCGATCCAGCTTGCGAGGGCCTTCGGTGCGGAGGTCTTCGC
GACCGCCGGCAACGCTGCGAAGTGCGAGGCCTGCGAAGCACTCGGGGCGAAACGTGCGATCAATTATCGCGAG
GAGGATTTCAAGGCTGTCGTGCTGCAAGAGACCGGCGGCCGCGGGGTCGACGTCATTCTCGACATGGTGGGCG
GGCGCTACTTCGATCGCAACATTGGCAGCCTCGCCAAGGACGGGCGCCTGTCGATCATCGCTTTCCTTGGCGG
CGCCCGGGTGGACGGCGCCAACATTGCGCCAATTCTCATGAAGCGCCTGCAGGTAATGGGCTCGCTGCTCCGC
CCGCGCACCGCCGCCGAGAAGCAGGCGATCCGCGACGGGCTTGCGGAAAAGGTCTGGCCGCTGCTGGAGGGGG
GCGACGTTGCACCGGTCATCCAGGCGGTCCTGCCCTTCGCCGAGATTGCCGGCGCCCATCGGCTGATGGAGGA
GGGCGATCATATCGGCAAGATCGTGATGCGGATCGGGTAGATGACCGAGCAACAAGAACTCCCCGAACGCGAG
AGCATGGAATTCGACGTCGTGATCGTCGGGGCGGGACCGGCGGGGCTTGCCGCGGCGATCCGGCTGAAGCAGG
TCAGTCCGGAACTCTCGGTCGTGGTGCTGGAGAAGGGCGCCGAAGTGGGCGCACATATCCTCTCCGGCGCCGT
GGTCGACCCGATCGGCATCGACCGGCTCTTGCCGGGCTGGCGCGACGAACCGGACCACCCGTTCAAGACGGAA
GTCACCGACGATCAGTTCCTCTTTCTCGGCCCCGCAGGCTCTGTCCGCCTTCCCAATTTCCTGATGCCGCCGC
TGATGAACAATCACGGCAACTACGTGGTATCGCTGGGCAATGTCTGTCGCTGGCTCGCCACCCAGGCGGAAGC
GCTCGGCGTCGAGATCTATCCGGGTTTTGCCGCAACGGAAGTGCTCTACAATGACGAGGGCGCGGTGATCGGC
GTTGCCACCGGCGACATGGGCATCGAGCGGAACGGCGAGCCGGGGCCGAACTTCGCCCGCGGCATGGAGCTGC
TCGGCAAGTACACCCTCATCGGCGAGGGCGTGCGTGGCTCGCTCGCCAAGCAGCTGATTGCCAAATACAAGCT
CGACGCGGGCTGCGACGTGCCGAAATTCGGCATCGGCCTGAAGGAGCTCTGGGAGGTGAAACCGGAAAATCAT
AAGCCGGGCCTCGTGCAGCATTCCTTCGGCTGGCCGCTCGGCATGAAGACCGGCGGCGGCTCCTTCCTCTATC
ACCTTGAGGACAATCTGGTGGCCGTCGGTTTCGTCGTCCACCTCAACTACAAGAACCCGTATCTCTATCCGTT
CGAGGAATTCCAGCGCTTCAAGACGCATCCGGCGATCCGCGGCACTTTCGAGGGTGGAAAAAGGCTCTCCTAC
GGCGCCCGCGCCATCACCGAAGGCGGCTACCAGTCGGTGCCGAAGCTGTCTTTCCCCGGCGGGGCGCTGATCG
GCTGCTCGGCCGGCTTCGTCAACGTGCCGCGCATCAAGGGCAGCCACAATGCGGTGCTGTCCGGCATTCTCGC
CGCGGAGAAGCTTGCGGCGGCGATCGCCGACGGCCGCGCCAATGACGAGCCGATCGAGATCGAGCGCGGCTGG
CGCGAGAGCGCTATCGGCCAGGACCTGAAGCGGGTCCGGAACGTCAAGCCGCTGTGGTCGAAATTCGGCACCG
CCGTTGGGGTGGCGCTCGGCGGTCTCGACATGTGGACCAACCAGCTCTTCGGCTTTTCCTTCTTCGGGACGCT
GAAGCACGGCAAGACCGATGCGCAATCGCTCGAGCCGGCCGCCAAGCACGAGAGGATCGACTATCCGAAACCG
GACGGGGTGCTGACCTTCGACCGGCTCTCCTCGGTGTTCCTGTCGAACACCAATCACGAGGAGGACCAGCCGG
TGCATCTGCAGGTGAAGGACTGGGACCTGCAGAAGCGCTCGGAATACGAGGTCTATGCCGGCCCATCTTCGCG
CTACTGTCCGGCCGGCGTCTATGAATGGGTGGAGAAGGACGGCCAGCAGACCTTCGTCATCAACGCCCAGAAC

TGCGTCCACTGCAAGACCTGCGACATCAAGGACCCGAACCAGAACATCAACTGGGTGCCGCCACAAGGGGGCG
AAGGGCCGGTCTATCCGAACATGTGAATGAGAACGATGATTGCGATCGCCTGTGCGGCAGGAATGGCGGGTGC
CGCTTCGGCACAACAGTCCTCGCAGACGGCGACGGCCGAGTTCGTGGGCAAGGACGGCACGGACATCGGCCGG
GCCACTTTGACGGCAGGCGGCAAGGGCGTGCTGATCGAGATGGAGATAAGCGGCCTGCCGAAGGATACCTGGG
TGGCTTTTCACGCTCACGAAATGGGGCGCTGCGATCCCAAGGACGGGTTCGAGTCGGCGGGAAAGCATTTTGC
CGGCGAAGATGAGAATGCCGAGCACGGCTTCCTCGCTTCCAATGGCCCGCATGCCGGCGACATGCCGAACCAG
TATGTCGGTGCCGATGGAACCTTGCGCGCCCAGGTCTTCAACAGCTTCGTGTCCCTCGACGACAGGGCTACCG
CCCTTCGCGGCCGGGCCCTGGTGATCCATGTGCATTCGGACGACAATCGCAGCCAGCCCTCCGGCGATGCCGG
CGACCGGCTGGCCTGCGCGGTGGTGAAGTAGATGGCCGCCCCCGTCACCGTGCGCGTCCAGCGCGACGACTTC
GACATCGCCGCCGAAGTCTCGGCGCTTTGCCGTGCGCGGACTGACATCGGTGCGGTGGTCACGTTTTCGGGGC
TGTGCCGGGACGAGGCCGGAGCCCTCAGTGCGCTCGAGCTCGAGCATTATCCCGGCATGGCAGAGGCGGAGAT
CGAACGGATATGCCACGAAGCGGTCGAGCGCTTCGGCCTGCAGGCTGCAACCGCCATCCATCGCTTCGGCCGC
ATGGAGCCGGGAGCGAATATCGTCCTGGTGGTAACCGCCTCGCCGCACCGGCAGGCGGCATTCGATGGCGCGA
ATTTCATCATGGATTTCCTGAAGACCTCGGCCCCCTTCTGGAAGAAGGAACATCACGCCGACGGCAGCGCAGG
CGATTGGGTCAGCGCGAAGGATGCGGACGACGCGGCACGCGATCGCTGGACGCGCCGCTGAATGGTCGAATCT
GAAGCGCAGGGACGTCTGCCCGGTATCCGCCCGGTCTCCGTTGTGGATATCGGCTCCAACTCCATTCGTCTCG
TCGTCTATGAAGGGCTGAGCCGTTCGCCGGCGATGCTTTTCAACGAGAAGGTGATGTGCGGCCTCGGCAAGGG
CATCGATGCGACCGGCCGAATGGAGGAGGAAAGCGTGGCGAGGGCGCTGAGGGCGCTGCACCGCTTTCGTGCG
CTCTCCGACCAGGCGCGCGCGACCGAGATGTATGTGCTCGCGACCGCTGCGGCGCGGGAGGCCACCAACGGAG
CGGCCTTCATCGAAAGGGCGGAAGCCATCCTCGGGCAAAGGGTGAAGATCCTGAGCGGTGAGGAGGAAGCCTA
TTATTCGGCGATGGGCATCGTCAGCAGCTACCACGAGCCGGATGGCGTCGTCGGCGATCTCGGCGGCGGCTCG
CTCGAGCTTGTCGATGTAAACGGCAAGCGGATCGGCAAAGGCATAACGCTGCCGCTGGGCGGCATTCGCCTCT
TCGAACATTCGAGCGGCTCGCTCAGCAAGGCGCGGACCTGGGTACGCAAGTTCATGAAAAATGCCGAGGTCCT
GAAGAAGGGGACCGGACGCACGTTCTACGCCGTGGGCGGAACCTGGCGAGCGATCGCCAAGCTGCACATGGAG
ATGCACAATTACCCGCTGCACATGATGCAGGGTTACGAAATCTCCTATGACGAAGCGCTGGCGATCCTCGCCG
AAGTGATCGAGCCGAAGAATATCAAGCCCTCCGCCTATGCGACGATTTCCAAGAGCCGCCGGAGCCTGCTGCC
CTACGGGGCGGTGACGATGCAGGAGGCAATCTCCATCATGCAACCCGAGCGCATCTCCTTTTCCGCGCTGGGC
GTCCGCGAGGGCTATCTCTTTTCGCTGCTTTCCGAGGAGGAGCGCCGGCAGGACCCGCTGCTGACCGCTGCCA
ACGAACTCGCCATCCTTCGCGCCCGCTCGCCCGAGCATGCGCGCGAGCTCGCCGAATGGACCGGCCGCCTGGT
GCCGTTCTTCGGCGTCGAGGAGACGGAAGAGGAGAGGCGCTACCGTCAGGCCGCCTGTTTGTTGGCGGATATC
AGCTGGCGGGCGCATCCCGACTATCGCGGTCTGCAGGCGCTGAACATCATTGCGCACTCGGCCTTCACGGGTA
TCACCCATGCGGGGCGGGCCTATATCGCGCTCGCGAATTACTATCGCTTCGAAGGGCTCAACGACGACGGCGC
CACGAGCCCGCTCGCCGGCATCACGACGCCGCGCCTGCACGAACTGGCCAAGCTCCTGGGCGGGCTGTTGCGC
GTCGCCTATCTCTTTTCGGCCTCCATGCCGGGTGTGGCGCGCCACCTCACGCTCCGGCGATCTTCGAAGCCCG
ACATCGATCTCGAATTCGTCGTCCCGGCAGCCTATGCCGACTTCGAGGGCGAACGCCTCGACGGCCGGCTCCA
GCAGCTCGGCAAGCTTACCGGCAAGAAGCTCGCCTTCTCCTTCGAGCGCTGAATGATACATACGCGCAAAGTC
CGCCTGATGCTGGCCAGCGCCGCCCTCCTTACCCTCGCCGGACCGGCCATGGCTCTGGACGGCGCGGACATGA
TGCAAAAGCTCAATGCAGCGACGTCGCCCAACGGCACTTCGGTGACCTACGACAAGGCGGAGGCCGACGGCGA
CGTGGTGACGGTCACCGGCATGAAGCTGCAGGTGGTGGCGAAGCCCGGCACGTCCATCGACATAGGCGAAGTG
ACGTTCGAAGGCGTCGAGGAAAAAGGCGGCGGCTACTATGCCGAGACCGTCTCCTTCCCGGATATCGCTGTCA
GCGACAAGGATGCAAGCTTCTCGGTCAAGGACATCTCGATCGGCGGCCTGTCCATTCCGGGCAATGCGAGCGG
TACCACGATCGATGACATCCTGCTTTATGAAACGGCCGGCACCGGCCCCATGACCGTAAACGTCAAAGGCAAG
GACGTCTTCACCGCCGAGAGTACGGAAACCAATCTTACGCGGCAGGAAAACGATGCGGGCTTCGACTTCGACG
CGACGCTTGCCGGAATCAAGGCGGATCTGTCGCAGATCGAGGATGCCAAGGCAAAGGAGGCGGTCGAGAAGCT
TGGGCTGACGACGCTCGACGGCGGAGTCACGATGAAGGGCAGCTGGGAATTGCAAACCGGCAACCTTGCTCTC
GACGAATACGCGCTGGACTTCAAGAATGTCGGCCGACTGAATATCGCGATGGACTTATCCGGCTATACGCTGC
AATTCATGAAGTCGTTGCAGGAGGCAGCCAAAGCGGCTGAGGCAAACCCGAACAGGAAGGAAGCCGACCAGGC
GATGGCGCTGGCGATGCTCGGCCTCATGCAGCAGCTTACCTTCAACAGCGCCTCGATCCGCTTCGACGATGCA
TCGATCACGAAAAAGGCGCTCGACTACGCCGGCAGCCAGCAGGGCGTCACCGGCGAACAGCTGGCGCAATCGC
TGAAGGGGCTCGTGCCGATCATGATGGCGCAGCTCAATCTCCCGGAACTGCAGAACCAGGTCTCCAGCGCCGT
CAACGCCTTTCTCGACAATCCGAAGAGCCTGACGATCAGCGCTGAGCCGGAAAAACCCGTGCCGTTCCCGATG
ATCATGGGTGCTGCGATGGGGGCTCCCAACACCGTCCCGTCCGTGCTGGGCGTCAAGGTGACGGCGAACGACT
GAATGAACAGCATTGCGAAAATCTCCGTCATCGCCACCCTCTCCGCCCTTACCTTCGCCGGAATCTCCCAAGC
GGCGAGCGTCGAAATGAACTGGCCGGACCGGATCGACCAGACCGTGCGCACCTTCAAGGGCGACAATTTCCGC
GTTGTCGATGTCGATACGTTGAGCCGCCAGAACCAGACCCGTATGCGAGTAGACAAGGCAACACCGGGGCAGC
GTGCCGCATTGCAAGCCGCTGTCGAAGCCAACAGACCGCTGCTCGCTTCGCTTAGGGCACGCAATGTCGAACT
GGACAACGTCGCCGCGGCGAAGCAGGCGGCCGATGGCGGACTGACGATCTACCTCCGCTGAATGATCCGTTCT
GTCGTGCGCGGCTTCGGGGCAGCACTGCCGAAGCGAGTGATGACCAATAAGGAAATCGAATCCAGGGTCGACA

CGTCCGACGAATGGATCGTGCAGCGGACCGGCATCCGCCAGCGCTATATCGCAGGCGAGGGCGAGACGAGCGC
CTCTCTCGGCGAGGCGGCGGCGCGGGCGGCGCTCGAGAGAGCCGGGCTGACGCCGGACGACGTCGACCTGATC
ATCGTCGCGACCTCCACTCCGGACAACACCTTCCCTGCAACCGCAGTCAACATCCAGAACCGCCTCGGCATGC
GGCACGGTGCGGCATTCGACATGCAGGCGGTCTGTTCCGGCTTCGTCTACGCGGTGGCGACGGCCGACGCCTA
TATTCGCGGCGGCCTCTCCAAACGCGCTCTTGTCATCGGCGCAGAGACCTTCTCGCGGCTTCTCGACTGGACC
GACCGGACGACGTGTGTGCTGTTCGGCGACGGCGCCGGCGCAATCGTTCTGGAGGCGCAGGAGGCGGCCGGGA
CCAAGGCTGATCGGGGCGTTCTGACGGCGCAATTGCGCTCCGACGGCGCCCACCGCGACAAGCTCTATGTCGA
CGGCGGGCCGTCGACGACCGGCACCGTCGGCCATCTCCGGATGGAAGGGCGCGAAGTCTTCAAGCACGCGGTC
GGCATGATCACCGACGTGATCGAGGCGGCTTTCGAGGCAACCGGAACGACCGCGGACGACATCGATTGGCTGG
TTCCGCACCAGGCCAATCGCCGCATTATTGACGGCTCGGCCAAGAAGCTGGGCATCCCTCTCGAAAAGGTTGT
GGTTACCGTCGACCTGCATGGCAACACCTCCGCGGCATCGATTCCGCTTGCCCTCGATGCCGCCGCCTCTGAC
GGCCGCATCAAGAAGGGTGATCTCGTAATGCTCGAAGCGATGGGCGGGGGCTTCACCTGGGGCTCCGTTCTTC
TTCGCTGGTAAATGACCACGAGTCGCGAGCCGCCCGACGCCCCTCTGCGCCTGCTCGAGGTACTGGAACCCGG
CGGCGGAGGGTCCGGACGCCACTTTCTCGATCTTTGCCGCGGCATGCATGCGCGCGGCCACCATGTCGAAGCG
ATCTATTCTCCCGTTCGGGCCGAAGACGGCTTCGTGCGCGAACTCAAGGCGCTCGGCCTTCCCGCCATTCATG
CGGTCGGCATGCGCCGGGCGCCCGGCCCCTCCGACTGGTCCTGCTTACGCGCCATAAATCGCATCATCCGCAC
TGCCGGGCCGTTCGACGTCATTCACGGCCACAGTTCCAAGGCCGGAGCCCTGACGCGCCTGCGCCTCCCGGGA
AGGCACGTGCCGCGCGTCTACACGCCGCATGCCTTCAGGACGATGGACCCGACGCTCGGGCGCGGCGGCCGAC
TGATTTACGGGGCGATCGAAACACTTCTCGCCCGCTTCTTCACCGACCATCTCGTCACGGTCTCCGGTGACGA
ATTCGCCCATGCGCTGTCGATCGGCATTTCGGGAAAGGGCATGTCCGTTATCGTCAACGGCGTCGACACCCCT
TCGCCCGATATGGCGCAGACGGTGCGCGCCAGCTTCGGCATTCCGGCGGACGCCTTCGTTTTCGGCTTCATCG
GCCGGCTCTCGGCACAAAAGGCGCCCGAGCGTCTCCTCAATGCCTTCGGCAAGGCCGCGTCGGCGGTCAGGAA
CAGTCACGTCGTCATGATCGGATCGGGAGAATTGGAGGAAGAGGTCCGCGCCGCGATCGCCGCAAGCGGCTTG
CAGAACCGCATCCATCTGACCTCGGCCTTTACGGGACCTCAGGCGGTACCCGCATTCGATCTCCTCGTCATGC
CGAGCCGCTACGAGGCAATGTCCTATGTGATGCTCGAAGCGGCAGCCGCCGGCCGGCCGATCATCATTTCCGA
CGTCGGCGGAGCCGGAACGGCCGTCGATCACGGAGAGAACGGCTACATCGTTCCGAACAGCGAGGACGTCTCA
CCGCTTGCAAGAGCGATGATAGCGGCGGCCGACCCGGATATGTTTCGGCACCTTGCCGGCGCCGCCGCGGCGC
GCCGGGACCGTTTTACACTGAAACGTATGCTCGACGAGACGGAAGAGGTTTACCGACGAATGGCAGCGCAGCG
CCGCCGACAGCAGTCAGTAAGATCGAATCAAGAATACTGGAAACCGACGGAAATGATGAAAGCCGCTCAATCA
CTGCATGGGGGCTAAATGAACCACGACGCCTACCCGGACTATTATCTTGCCGACATCCTGCGCGAGACGAAGC
CCATCGCGCTCGTCGGCGCCTCGCCGAAGCCGGAACGGCCGAGCTATCGCGTCATGGCCTTCCTTCTCCGCAA
GGGATACCGCGTCATTCCGGTCAATCCCGGCCATGCGGGCGGCACGATCCTCGACCAGCCGGTGGTAGCGCGC
CTCGCGGATATAGGGGAGCCGATCGACATGGTGGACGTCTTCCGTGCGGCTTCGGCCTTGCCCGCGCTCGTCG
ACGAGATCCTGGCGCTGCCGGCCCTGCCCAAGGTCATCTGGGGACAACTGTCCGTCCGCGACGACGCAGCTGC
CGCCAAGGCTGAGGCCGCAGGCATCAAGGTCGTCATGGACCGTTGCCCGGCAATCGAATATCCGCGACTGGTC
GGATGAATGTCCATCGCCCCCGGTATCACCATCGACGGCCCCGCCCGGCGCGTCTCCCTTGACGTCCGCAACC
CCCGTTTCTTCCGCAATCCGCTGCCGGCCTACGCGGCGCTCCATGCGCAATGTCCGGCCTTTTTCTGGGAAGA
GCCGCAGCAGTGGTTCTTTGCGGGCTACGAGCAGGTGAACAGCCTGTTGCGCGACCGTCGCTTCGGCCGACAG
ATCCTGCATGTCGCGACCCGGGAAGAACTCGGCATGCCGGAGCCGAAACCGCATCTGAAAGATTTCGATGCAC
TCGAGGCGCACTCGCTGCTCGAACTGGAGCCTCCGGCCCATACGCGGCTGCGCACGCTCGTCAACCGCGCCTT
CGTCTCCCGTCAGATCGAAGAGCTCAGGCCGGAAATCGAGGCGCTCTCGCATGCGGTGATCGACGGCTTCGAG
AAGGATGGCGAGACCGAGCTGTTGAAGACCTATGCCGAGACGATCCCGGTAACCATCATCGCGCGGATGCTCG
GCATTCCGGTCGAAGCGGCGCCGCGTCTGCTCGACTGGTCGCACCGCATGGTGAAGATGTACGTGTTCAATCC
CAGCCTGGAAACGGAGTTCGACGCCAACAATGCATCGGCGGAGTTCGCCGACTACCTGAAAGGGATCATAGCC
GAGAAGCGGACAAACCCCGCCGACGACCTGCTCACGCATATGATCACCTCGGAGAAAGACGGCGAGCGCCTGT
CGGACGCGGAGCTGATTTCGACCACGGTCCTCCTTCTGAATGCGGGCCACGAGGCGACGGTTCATCAGATCGG
CAATGCCGTGCGCACGATCCTGCAGTCCGGCCTCTCGCCCGCCGAACTTTTCTCGGACGAGAAGGCGACGGAG
CGCACCGTGGAGGAGTGTCTGCGCTTCGCCGCGCCCTTGCACATCTTCCAGCGCTACGCCCTCATGGATATCG
AGCTGGAGAACGGCATCGCCCTCAGGAAAGGGGACAAGATCGGCCTGATGCTGGGGGCGGCGAACGTCGATCC
GCGCAAATTCTCCTCTCCCGATACGTTCAGGCCGGACCGCAACGAAGGCGCCAATGTTTCGTTCGGCGCCGGC
CTGCACTTCTGCATCGGCGCACCGCTGGCACGGCTGGAGTTGCAGATTTCGCTGCCGATCCTGTTCCGACGGC
TTCCCGGGATGCGGCTCAAGAACGAGCCGCCGGTCAAGGACGCCTTCCACTTTCATGGTCTGGAGCGGCTCGA
TCTGGTCTGGTGAATGAACCATATCCTCCTCGTCGGTGCCGGCGGCGCCCTCGGCTCCGTCCTGCGCTACCTC
GTCGGCCTCTGGATGCTGCAGCGCGCCGGTCCCGCCTTTCCCTGGGGGACGCTGTTCGTGAACGTCACTGGCT
CCTTTCTGATCGGTTTTCTGGCGGAGTTCATCATGCACAAGATGGGTGCCTCGCCGGAAATGCGCGTGTTCCT
GATCACCGGCGTTCTGGGGGGCTACACGACGTTCTCGGCCTTTTCGCTCGACGCGATCGCCCTTCTCGAACAC
GGGCAGACGATGAGTGGCCTCGCCTATATCGTGGCGAGCGTCGGTCTTTCGATGCTTGCCGTGTTCGCCGGGC
TGGCCCTGATGCGCGCAATGGTCTAAATGAATGCCTTTCGTGCCAAGCCGCTGAAGTTTCCATGGCCGCCTCT

GCTCTATGGCGCGGCCGTCCTGATCGCGCTGGCCCTGGGCCACCTTTTCCCGATTCCGGTGGCGCACGAGCAC
AGTTGGATCCTCTCGCTGGTGGGCTACGGCCTCGTGGTGCTGGCGATCACCATCGATCTCTGGGCCGTCAAAA
CCCTCCTTGACCGCCATACAGCCGTACTTCCGAACCGGTGCGCCACCTGCCTCGTCACCTGCGGCCCATTTCG
TTTCACCCGCAACCCGATCTATTTCAGCTACACGCTGGCGATGACCGGCCTCGGATTGGCGACGATCAACCCC
TGGTTCTTCATCACGGCAATTGCCGCGGTCGGATTGACCACCCTTTTCGCGATCCGAAAGGAGGAGCGGCACC
TGCTGTCGCGCTTCGGTTTCGAGTTCGAGCGATATTGCCGGCACACTAGCCGTTGGATTTGAATGTCCGAACA
CAGCGCGACGACGACACCTGAAGCGCAAATGCGCACGGAGGCGACCGGAACCAACCGGATGGTTCTCCTGGAT
GCGCTGCGCGGCCTGGCACTCGTCGCCATGGCCCTCTATCACTTTGTCTGGGACCTCGAATTCTTCGGCTATG
TCGCGGCCGGCACCGCCGGGACCGGCGGCTGGCGGATGTTTGCACGCTTGATTGCCAGCAGCTTCCTGTTTCT
TGCCGGTTACAGCCTCGTGCTCGGCCAGCTCCCGAAACTCCGTCCGCGCGCCTTCGCCATACGTTTCGCGAAG
ATCGCCGGCGCAGCCGCGCTGATCAGCATCGGGACCTGGTTCGCCTTCCCTCAGTCGTTCATCTTCTTCGGCA
TTCTCCACGCCATTGCGGCGGCAAGCCTTGTCGGCCTCCTGTTCCTGAAATTGCCGGCCGCCGTTTCCTTTCT
TGCCGCTGCCGTGGCCTTCGCGGCTCCCCTCTATCTGCGCTCGTCGTTTTTCGACATGCCGGCGCTCTGGTGG
GTCGGCCTCTCGGAAACGATCCCGCGCTCGAATGACTACGTTCCTCTGCTTCCCTGGATTGCCCCTTTCCTGC
TCGGCCTCGGCACCGCGAAGCTGCTCCACCCGTTATTTCCGCCCGCCGCAGCCGAAGCGCCGGGACCCGTAA
GCACCCGTGGATGACGCCTCTCGCCTTTGGCGGCCGTCACAGCCTTCTGATATATCTCATCCACCAGCCGCTG
CTTATCGCGGCAGTCTATCTCTTCTCTCTCGCCGTTCCCGCCCCGGCACCGGATCCGGCACGGGTCTATCTGC
TGAACTGCGAACGGGCCTGCAGCAACGAAAATTCCGGGATCTCCTGCAGCGCCTTTTGCGGTTGCACGCTCGC
CGGCCTGAAGGAACAGAACCTGTTCGACGATCTGAACCTGGGCAAGATCGACGTGAACACGGATGAACGCATT
GCGCGAATCGCCGGTCAGTGCACAATGGATGCACAATCAGGAGAGGCACAGTCAGGGGACTAGATGAAAGCCG
CCCTGATCGTCATGACCATTCTCGGTTGCGACGACAACGTTACCCAATGCCATTACATCTCGACCGTGAACGG
CGACTGGCCGTCGATCGCGCAGTGCGACACGGAATCGCAAAAGGAACTGCCGCGGTTCTCCGACAGGAACTAC
CCCGTCGTGGTGGCCGTGTGCGAGAGCTCAGGGCCGGGCATGACGGCGGAAAGCGAGCCGGTTCCGGAGCTGA
ATGCAACGCTTCCGGCTCAGCCGGGCGTGCAGCAAAACACAACATCAACGGCAGAAGGCGGAGAACCAACCTT
GCCGAGGCGCGCCCTGGCATTGATCAGCGGCGCCGTACCCGACCGGGAGAAACTGAAGGCGATCGTCAGTGCG
CCGGTCCACTACGTCGAAGACGGCTATTCCTGGGTGGCGCGCCGATTCGGCAATTGAATGGCCTCTCTCTTCG
ACCCGATCACGATCGGCGATATCAGCCTCAGCAACCGCGTGGTGATGGCACCTCTGACCCGGAACCGTTCACC
CCAGGCAGTGCCCAATGATCTTAACGTCGCCTATTACGAGCAGCGCGCATCCGCCGGCCTGCTGATCACCGAA
GGGACGGCAATAAGCCACCAGGGCCAGGGCTATGCCGACGTGCCCGGGCTTTACAAACCGGAGGCCCTCGCCG
GCTGGCGCAAGGTCACGGATTCGGTCCACAAAGCCGGTGGCAAGATCGTCGTGCAGATGTGGCATGTCGGGCG
GATATCCCACGACTCGCTCCAGCCGCAGGGCGGCAAACCGGTCGCCCCTTCGGCTATCCGCGCCAAATCGAAG
ACCTACCTCGTCAATCCCGATGGGACGGGCAGCTTTGCCGAAACCTCCGAGCCGAGGGCGCTCGAACCGGCGG
AGATCGCCGGCATCGTCGAGGATTACCGCAAGGCCTCTCGGGCCGCGATCGAAGCCGGATTCGACGGCGTCGA
GATCCATGCTGCCAACGGCTATCTGATCGACCAGTTCCTGCGCTCCGGCTCCAATGAACGCACGGACGCCTAT
GGCGGCTCGATCGAGAACCGCGCCCGCTTTCTCTTCGAGGTGGTCGATGCCGTTACCAAGGAGATCGGCGCTG
GCCGGGTCGGGATCCGCATCTCTCCCGTGACCCCCGCCAACGACGCCTTCGATCCGGACCCGCAGCCGCTCTT
CACCTATGTCGTCGAAGGCCTCGCCCGATATCCGCTCGCCTATGTGCACGTCATCGAAGGCGCCACCGGCGGA
GCGCGTGACCACCAGCAGGGCGATCGCCCCTTCGACTACACAAGCCTCCGTGCCGCCTACGCAGCGGCCGGCG
GCAGGGCGGCCTGGATGACGAACAACGGATACGATCGCGGTCTGGCGCTGAAGACGGTCGAAAGCGGCGAAGC
CGATTTGGTTGCTTTCGGCAAACCCTTCATCGCCAATCCGGATCTCGTTCGTCGTTTGAGAGACAACGTGGCC
CTGAATGCACCGGACCAGGCGACCTTCTATGGCGGTGGCGCCAAGGGTTATACGGATTATCCGTCGCTCGAAA
ACGTCGCCTGAATGCGAGGCCGAGCCCTCAAGCTTTCAGCGTCTCGGCGCCTGGTCGGGGATCTCATGCGATT
TTCGATGAAGGTGCCGCGGGTGAGCGTGCAGCGGCGGATGAATCTCGGGCCGTTGCTCGAGGCCAGAACATCG
CTTGAAAGCAGGCCATCCTGGACGGCGCTCTTCCTAAAAGGATACGGCCTTCTCGCTCAGGAGACGCCCGAGT
TGCGGCGCGCTTACGTCAAATTTCCCAGACCGCAGCTATACGAATATCCGGGAAGCATCGCTTCCATCGCGCA
TGAAAGGGAGTACGAGGGCGAACGAATCGTGCTGCTGACGACGATCAAGAATCCGGAGCGCCGTCCGATCGCG
GAGCTGGGGTCCTTGATGCAGACGGCGCGAACCAGTCCCGTGCTGGAGGTCAAGGAATTCCGGCGCGCCCTGA
AGATCGCCCGCCTCCCCGGTCCGATCAGATGGCTTCTCATGTGGCTCGGATTGAACATCGGCCGGCAAAGGGC
GCGCCGTTTCGGTACATTTCAGCTGTCGGTCTATTCGGGCCTCGGCGCGGAATCGCTCAACCCCCTCACGCCG
TTAACCTCGCTTCTCAATTACGGCCCGATCGACAACGATGGCTCGGTCACCGTGCGCATCCACTATGACCACC
GGGTCATGGATGGCGCCAACGTCGCGCGTGCCCTCGAGCGGTTCGAGAGGATATTGAACGGCGAAGTCACCGC
GGAGCTGGAAGGGCTTGCATCCGGGCGCGACGTAACCGCTGCGGCAGGTTCCGGAGCGGCGCGATGAATGACC
AGAAAACGTGCGAACTTGATCGTCGCTGCAATGCTTTGTTCGGCGACGGTCGTCACCTCCTCTGTCAATGCGC
AGGATCAGGCCGCACCACCGCTTGTGACGCAGATCGGCAACGGCAACTGGCTTCCGCAGGAGGAAGCGGAGTC
GCTGCGCGACGAACTCTACTATCAACGCGCGATCCATGCCTACATAACCATGCTTCCGGCGCTGAACGTGATC
GGTATGCGCGACGGTTCGGAGGCGACATTCGGTAAAGGCTACAACATCCTGCCAATCTGGAAGGACCGGATGG
ACTCGCGCACGTGGGTGCCGACACCGAACGCCGACGTAATCTACTCGATGAGCTATCTCGACCTGAAGGAAAC
CGGCCCGCTCGTCGTCGCCGCGCCACCCAACGTCATCGGCATGTTTACCGATTTCTTCCAGCGTACGATCACG

GATGTCGGACTGATTGGACCGGATCGCGCGCGGCGGCCTCTACCTCCTGCTGCCGCCTGATTATGACGGCG
AGGTTCCGCAGGGCTACTTCGCGCTCAAAGCGAAGACCTACAATGTCTTTCTGTTCTTCCGGACGATCATGGC
GAAGGGCGAAAACGGACCGGACCCCAAGCCCGCCGTGGCGCTCGCGGAGCAGACGCGCGTCTATCCGCTTTGG
ACGCCCGAGAAAGACGTCAAGCCGATGCAGTTTCCTAACGGCAGCGGCAAGCGCATCAACATGATGTATCCGG
TCGATAACGCCTACTGGACCAAGTTGAAGGAGTTCGTCGACCACGAGCCGGTCTCGGCGATCGATTCCGAACT
GCGCGGTGTGCTGGCATCGATCGGCATCATCAAGGGCCAGCCCTTCGAGCCGACCGGGAAGCAGCAGGAACTC
TTGAAAAAGGCGGTCGAGACAGCGCCCAAGATGATCTTGGCGCAGCGCCAGCTCGGCCGACCGGACGGACGCA
ATCGCTACTACACCGACAGGCAGTATGAAAATGTCTGGGCCAGCGGCACATCCGAATGGCTCCAGGACAGTTA
TCTCGATGTCGACCAGCGGGCTGGCTATTTCCAGGTTGCCTATTCATCGGCTCCGGCAATGGTCATGCGGACG
CTGGGGGCAGGTTCCAAATACCCGGTTGCATACCGAGATGCCGCGGGCGAGTTCCTCAGCGGCTCCAGCACCT
ACAGGCTGCATTTGCCACCCAATCCGCCGGCTGCCCTGTTCTGGGCGGTTACCGCCTACAACATCACCGACGG
CACGATGGTGGAAGCGCCGCAGCTGATGCCGTCCATCAACGGCTTCAACAAGGTCGCGACGAACCAGGACGGC
TCGGTGGACCTGTGGTTCGGACCGGAAAAACCTGCCGACGCGCAGGACTCGAACTGGATCCAGACCGTCGGCG
GCCGGAATTTCCTGATAGCGCTGCGTCTCTACGGAACCGGCGTCGAGTTCTTCGATCAGACCTGGAAGCCGGA
TGATGTGGTCATGGTGAAGTGAATGACTGCTTTGCATCCCATTTCGCGCCGGACCCTCATCGGCGGTCTTGCC
GCCACGTCGGCATTTGTCGTGCTGCATCCCTTCTCGACCCGCGCGGCTGCCAACCAGGCGCATCTCAGGATCA
TGGAAACCACGGACCTGCACGTCCACGTCTTTCCCTACGATTACTATGCCGACAAGCCGAACGACACGCTTGG
CCTAGCGCGCACCGCTTCGATCGTCGACGCCATCCGCGCCGAGGCGGCGAACGCGGTCCTCGTCGACAATGGC
GATTTTCTTCAGGGCAATCCGATGGGCGATTACATCGCCTACAAGCGCGGCATGAAGGAAGGCGACATGCATC
CGGTCATCGCCGCGATGAACGTGCTCGGCTACGACTGCGGCACGCTCGGCAATCACGAGTTCAACTACGGCCT
GGATTTCATGTTCAATGTGATCAACGGCGCAAACTTTCCGATCGTCTGCGCCAACCTGACCAAAGGCGCCATG
GCCGCCGCCCCGCGCCAGGACGAGCTTTTCCTCAAGCCCTACGTAATCCTCGATCGAAAAGTGAAGGACGGCG
CCGGTCAGGAACACCCCATCCGCATCGGCCTCATCGGGTTCGTACCGCCGCAGATCATGACCTGGGACGCCAA
GAACCTCGAAGGCAAGGCGAATGCGCGCGACATCGTCAAGGCCGCGGAAGCCTGGGTGCCGCAGATGCGCGAG
GAAGGCGCCGACATCGTCATCGCCCTCTCACATTCCGGCATAGGGCAGCAGGCCTATGCCGAAAATCTCGAAA
ACGCGTCCGTACCCCTGGCGGCAATCGAGGGTATCGACGCCATCGTCACCGGCCACAGCCACCTCGACTTTCC
GGGACCCAAGTTCGACGGAACTCCGGGCGTCGACAATGCCAAAGGGTTGATTTCAGGAAAGCCCGGCGTCATG
GGCGGCTTCTGGGGATCCCATCTCGGCCTCATCGATCTGCTGCTCGAACGCGACGGCGGCCAGTGGCGTGTGA
TCGGTTCGACAAGCGAGGCGCGGCCGATCTTTCGCCGGGAAGAAAAGAAGGTGATCGCCGAGGTCGGTGACAA
GCCGGAAGTGCTCGCAGCCGCGCAGAAGGACCACGAAGCGACGCTTGCCTATGTGCGCACCCCCGTCGGCAAA
ACTTCGGCACCGCTATACTCCTATTTCGCGCTGGTTGCGGACGATCCGTCGGTGCAGATCGTGAGCCAGGCCC
AGACGTGGTATATCCGCGAAATGCTCAAGGACACCGAGCACAGCGACCTGCCGGTCCTGTCCGCCGCAGCCCC
GTTCAAGGCCGGCGGGCGCGGTGGCGCCGAGTACTATACGGATGTTCCAGCGGGCGACATCGCCATCAAGAAT
GTCGCCGACCTCTACCTCTATCCGAATACGGTGCAGGCGGTCGTCATCACCGGCGAAGAGGTGCGCAACTGGC
TCGAAATGTCCGCCGGCATCTTCAACCGGATTGCACCGGGATCGGTCGATGCAAGCCCGATCAATGGCGACTT
CCCTTCCTATAATTTCGACGTGATCGACGGGGTCACCTACCAGATCGTTCTTTCGCAGCCTGCGAAATACGAC
AAGGACGGCAATGTGGCGAACCCGGAGGCCAGCCGCATCCGTGATCTGAAATTCGATGGCAGGCCGATTGATC
CCAAGCAGAGATTTGTCGTAGCGACCAACAACTACCGCGCCGGCGGCGGCGGCAATTTTCCGGGCATCGCCGG
CGACAAGGTCGTCTTCGTCGCGCCGGACACCAACCGCGACGTCATCGTGCGCTACATCGTCGAACAGGGAACG
ATCAACCCCTCGGCGGACGCGAACTGGACCTTCGCGCCGCAGAAGGACACGAGCGTGCTGTTCGACAGCGGCC
CGAGGGCGCGACAATTCCTGGCAGAGGTCAAGGCGGTGAAAATCGAGAATGCCGGCGACGGCAGCGACGGTTT
CGCCCGGTTCAGAATCAAGCTCTGAATGCTCCTGGCAGCCAGCAGTTTTGCCACTGGTGCTCTGGCCGCGCCC
GTGCATGCGATCGCCATGCATGGCGACCCCGCCTTGCCTGCCGATTTCAAGCACTTCCCTTACGTCAATCCGC
AGGTGAAGAAGGGCGGGAAGATCGCCTACGGGGTCGTCGGTACGTTCGACAGCCTCAATCCGTTCATCTTGAA
GAGCATGCGCACGACCGCGCGCGGCATGTGGGACCCTGGCTTTGGCAACCTGGTCTATGAATCCCTCATGCAG
CGCTCACAGGACGAGCCCTTCACGATGTACGGCCTGCTCGCCGAAACCGTCGAATGGGACGATGACCGCACCT
TCATCCAGTTCAACCTCAATCCCAAGGCGCGCTGGGCCGATGGCCGGCCGGTGACCGCCGAAGACGTGATCTT
CACCTTCGAATTGTTGCGCGACAAAGGGCGTGCGCCTTTCAGCAACCGCCTCTCGAAGGTTGCGAAGATGGAG
AAGGTCGGGGAACGTAGCGTGCGCTTCACCTTCACCGAGGAGGCCGATCGCGAGATTCCGCTTCTGCTGGGGC
TTTCGCCCGTGCTGCCGAAGCATGCGATCAACGTCGAGACGTTCGATCGGACGAGTCTGGAGCCGCCGCTCGG
CTCCGGCCCCTACCGGGTCGCGGAAGTCAGGCCTGGCGAGCGGATCGTCTATCGCCGAAACCCCGACTATTGG
GCCAAGGATCTGCCCTCGAAGGTGGGCCTCGACAATTACGATGAGATCTCCGTCGAATATTTCCTGCAGGAGA
ACACCCTTTTTGAAGCCTTCAAGAAGGGTGTGGTGGACATCTACGCCGAAGGCAGCGCGACGAAATGGGCCCG
GGCCTACGATTTTCCCGCCGTTCGCAGCGGCAATGTGATCAAGGAAACGTTCAAGCCGAAGACACCGTCCGGC
ATGCTCGGCTTCGTCTTCAATACGCGCAGACCGATGTTCGACAACATCAAGCTCAGGCAGGGGCTCGCTCTCG
TTTTCGACTTCGAATGGGTCAACAAGAATCTCTTCGACGGCGCCTATACGCGTACGCAGAGTTATTGGCAGAA
TTCGTCGCTTTCCTTCCTCGGCGTGGCAGCCGACAATCGTGAACTGGACCTCATGGGCGACGTGAGGGAGCGC
ATCAATCCGGCGATCCTCGACGGAAGCTATCGACTCCCCGTGACGGACGGCTCGGGCCGCGATCGCAATGTGC

TCCGGGAAGCGGTGACCCTTCTTCGCGAAGCGGGCTACTCCATCAAGGACGGCAAGATGGTCGACGCCAAGGG
GACGCCGCTGGCCTTCGAGATCATGAGCCAGAATGCCGGCCAGGAGAAGGTTGCGCTTGCCTATCAGCGCTTT
CTCGCGCCGCTCGGCATCGCAGCGCGGGTTCGGACCGTCGACGATTCACAATATCAGTTGCGCAGCCAGTCCT
TCGATTACGACGTCATCATCAAGTCCTTCCCCTCGTCGGTCTCTCCCGGTCTCGAGCAGATCAACCGCTGGAG
TTCGCAAACTCGGGACAGGCAAGGCAGCGACAACTTCGCAGGCGTCGCCGACAAGGACGTCGACAAGCTGATC
AACAACATCCTGCAGGCGCGCAACCCCGAGGACTTCACCGCCGCCGTTCGCGCGCACGACCGGCTCCTCGTCA
ACAATTCCTATCTGGTTCCGCTTTACCATCTCGACGCGCAATGGATTGCCCGCTGGAAGCATATCGGCCGGCC
GACGACGGTACCGCTTTACGGTTATCAGTTGCCGAGTTGGTGGGACGAGCGCGTTCAGTAAATGTCGCTTCTT
TCGGTCGAGGAAGCACTACAGAGAATTTTGGCCAGGGCCAATCCGCTGCGACGGACGGAGCGCCTTGCGCTGC
ATGAATGCCTCGGACGGGTGCTTGCCGAGAACGTAGCGGCCAAGCTGACCCACCCCCCTTTCGACAATTCCGC
GATGGATGGTTATGCCGTCCGCCACGAGGATATCGCCGAAGTCGGAGCGGAGCTCTCCGTTGTCGGCGAGTCC
GCCGCAGGACGCCGTTTTCGCGGTGGCGTGAAGGGCGGGGAGGCCGTACGCATCTTCACCGGCGCGCCGGTAC
CTGCAGGCGCCGATACCGTCATCATTCAGGAAGACACGGAAAAACTGCCGGACGGACGCATCCGCACCCTGTT
TGCGCCGGCAAAGGGCCGCCACATCCGTTTGGCGGGCCAGGATTTCCTGGCGGGTGACGTCGCGCTTTCCGCC
GGCAGCGCACTCGACGCCGGCCGCCTGACGCTCGCGGCCGGCATGAATCATCGGGAGCTGCCGGTTTTCGCGC
GACCGAAAATCGCAATCATCGCCACGGGCGACGAATTGTTGCCGCCGGGAAGTTCCCTTGGCCCCGACCAGAT
CATCGCTTCGAACAGTTTCGGGATCGCTGCGGTTGCCCGCGACAACGGGGCCGAAGTTCTTGATCTCGGCATC
GTCGGCGACGATCGGACAGCGATCGCCGGCGCCGTCGCCAAGGCACAGGCAGCCCAGGTGGACGTGCTCGTCA
CCCTCGGAGGAGCTTCGGTCGGCGATCACGATCTCGTCCAGTCGACGCTTCTCGGCTGCGGCATGACGCTTGA
TTTCTGGCGCATTGCCATGCGACCGGGAAAACCGCTGATGGTCGGGGATCTCGACGGCGTCACGGTGCTCGGC
CTTCCGGGAAACCCCGTTTCGAGCCTCGTCTGCGCCCTGCTCTTCCTCGAACCTCTGACGCGCAAGCTTGCGC
ATCTGCCGCCCCGTTCGAGACTGGTCGATGCACGGCTCGCCGCAACGCTGCCGGCCAATGACCGGCGCCAGGA
TTATATACGTGCCCGCCTCACGCTCGAGGCGGACGGTTCGCTCGTAGCGCATCCCTTCCCGCGCCAGGATTCG
TCCATGGTCAGCATTTTCGCGCAATCCGATGGGCTGATCGTGCGGCGACCGGGTGCTCCCGAAGCCGCGAGCG
GAGAGGCATGCAGCGTTTTCAGGCTCAGAGATCCCGCCTGAATGGCGAGACAGCTGACAGGAAAGCTGCCCCA
GTCCATCGACGAGACGAGGGCGATGCTGGAGGCGCAGGATTATCTCGCCGGAACCGCGCTGGCGACCGTTCTC
TTCCTGGCCCTCAGGATGAAGCGCCCGCTATTCCTCGAAGGCGAGGCCGGGGTCGGCAAGACCGAGATCGCCA
AGGTACTGGCGCGCGCCCTCGACCGCCCCCTCATCCGGCTTCAGTGCTACGAGGGTCTTGATGTCGCCTCGGC
CGTCTACGAATGGAACTATCCGGCTCAAATGCTGGAAATCCGCCTGTCGGAAGCGGCGGGCACCGTCGACCGC
CAGAGAGTGGAAAGCGACATCTTCTCCGAGCGGTTCCTCATAAGACGGCCGGTGCTGCAGGCGATATCGACGA
CTGCCGGGAGGGCGCCGGTGTTCCTGATCGACGAGCTCGACCGCACCGACGAAGCCTTCGAAGCCTTTCTCCT
GGAGGTGCTTTCCGATTTCCAGGTCACAATCCCCGAACTCGGGACGATCAAGGCCGACGAGCCGCCGATCGTC
ATCATCACCACCAACCGGACCCGCGAAATCCACGATGCCTTGAAGAGACGCTGTCTCTATCACTGGGTCGACT
ACCCCAAGGCGGCGCAGGAACTGGAGATCATCCGCCGCAAGGTTCCGGGCTGCAACGCGGCGCTTTCACGCGA
GATCGTTGCCTATGTTCAGAGGTTGAGGGCGCTCGACCTCTTCAAGAATCCCGGCGTCGCGGAAACGATCGAC
TGGGCCACGGCGCTCACCGAGCTCGACGCGCTGGCGCTCGATCCCGAAACGGTGGCCGACACGCTCGGTACGC
TTCTCAAATATCAGGACGACATCGCCCGGATCGAAGGTGCCGAAGGCCGCCGCCTGCTTGCCGAGGTGAAAGC
CGAATTGCTGGCGCAGGACTGAATGACGATTTCCAACACCCGCCCAATCACCCCGGACCTGATCGCCTCGCAC
GGGCTCAAGCCCGACGAATACGAACGCATCCTGAACCTGATCGGGCGCGAGCCGACCTTTACGGAGCTTGGCA
TCTTCTCGGCGATGTGGAACGAGCATTGCTCCTACAAATCCTCGAAGAAATGGCTGCGCACGCTTCCGACCAA
GGGGCCGCGCGTCATCCAGGGCCCTGGCGAGAACGCCGGCGTTGTCGACATCGACGACGGTGACTGCGTCGTC
TTCAAGATGGAGAGCCACAACCATCCCTCCTACATCGAACCCTACCAGGGAGCGGCAACGGGCGTCGGCGGCA
TCCTGCGTGATGTCTTCACGATGGGTGCGCGTCCGGTAGCGGCGATGAACGCGCTGCGGTTCGGTTCGCCGGA
TCACCCGAAGACCCGCCACCTCGTCTCGGGCGTCGTTGCAGGCGTCGGCGGCTACGGCAATTCCTTCGGCGTG
CCGACGGTCGGCGGCGAAGTCGAGTTCGACGCGCGCTACAATGGCAACATCCTCGTCAATGCCTTCGCGGCGG
GGCTCGCCAAGACCGACGCGATCTTCTACTCGAAGGCGGAAGGCGTCGGCCTCCCGGTCGTGTATCTCGGCGC
CAAGACCGGACGCGACGGTGTCGGCGGTGCGACGATGGCATCGGCCGAGTTCGACGAGTCGATCGAGGAGAAG
CGCCCGACCGTTCAGGTTGGCGACCCCTTCACCGAGAAATGCCTGCTCGAAGCCTGCCTGGAGCTGATGCAGA
CCGGCGCGGTCATTGCGATCCAGGACATGGGCGCTGCCGGCCTCACCTGCTCGGCCGTCGAAATGGGCGCCAA
GGGCGATCTCGGCATCGAACTCGATCTCGACAAGGTGCCGGTGCGCGAAGAGCGCATGACGGCTTACGAGATG
ATGCTTTCCGAAAGCCAGGAGCGCATGCTGATGGTGCTGCGCCCGGAAAAGGAAGAGGAAGCCAAGGCGATCT
TCGTCAAATGGGGCCTCGATTTCGCGATCGTGGGCAAGACCACCGACGATCTGCGTTTCCGCATCCTGCACCA
GGGCGAAGAAGTGGCGAACCTGCCGATCAAGGAGCTCGGCGACGAGGCGCCGGAATACGACCGCCCCTGGACG
CCGGCAAGGGCACCCTCGCCGCTCGCCACCAACGACGTCCCGCAGGCTGATGTTTCCGATGCGCTTCTGAAGC
TCGTCGGCTCCGCCAACAATTCCTCGCGCCGCTGGGTCTATGAGCAATATGACACGCTGATCCAGGGCAATTC
GCTGCAGCTGCCGGGCGGCGACGCCGGCGTGATCCGCGTCGAGGGCCATGAGACGAAGGCACTTGCCTTTTCT
TCGGACGTTACGCCGCGCTATGTCGAAGCCGATCCGTTCGAGGGCGGCAAGCAGGCGGTCGCCGAGTGCTGGC
GAAACCTGACTGCGACCGGCGCGCTGCCGCTCGCCGCGACCGATAATTTGAACTTCGGTAATCCCGAACGGCC

GGAGATCATGAGCCAGCTCGTCCATGCGATCAAAGGCATCGGCGAAGCCTGCCAGGCACTCGACTTCCCGATC
GTCTCCGGCAACGTCTCGCTCTACAACGAGACCAACGGCCAGGCGATCCTGCCGACCCCCACCATCGGAGGCG
TCGGCCTCGTCCGCGACTGGTCGAAGATGGCGCGGATTCGCTTTGCGGCGGCGAACGAAACCATCCTGCTCGC
CGGCGCTCCGGAGAGCTGGGGAACGCATATCGGCCAGTCGGTCTACATGCGCGATATACACGGCCGCACCGAC
GGTCCGGCGCCGCATGTCGACCTCGGCCATGAACGCAAGGTCGGCGATTTCGTTCGCGGCTTGATCGAGGACG
GCCTGGTTACGGCCGTGCACGATTGTTCCTCGGGCGGACTTGCGCTCGCCGTCGCCGAGATGGCCATCGCTTC
CGGCATCGGCGCGACGATCGATGCACCTGCGGAACACGACCCGATCCCGGTCTTCTACGGCGAAGACCAGGGC
CGCTATGTCGTAACCGTGGCCGAGGGTTCGGCCGAGACGGTGGCCGCACGTGCAAAAGCCGCCGGCGTTGCGC
TGCCGGTGATCGGCAGGACAGGCGGGAATGCGGTGCAGCTCGGAGACGCCAGGCCCGTATCCGTCGATGAATT
GCGTTCCGCACATGAAGCGTGGTTCCCCAATTACATGGGCGGCGATCTCGCACCGGACAATTGAATGAGAAGA
TTTGCCCGCACGACCCTGACTGCCGCCCTGCTTTATAGCGTGGCAGGCATTGGCGCTCATGCTCAGGAAACGC
CCGACGAACTCGATACCCGCATCGGCAAACTGACATTCGAGAGCGGCTACCCCTCGAAGGAAACCGTGGCCAA
GCTTTACGACGAAATGGATTTCCAGCGAGCGTCGCAAGCTTACCTGTGGGGCATTCCTGCCGTCGGCCTCAAC
GAATGGCGAAAGGCCCATTTCAACGTCTTCAAGGCCAAGAACGGTGAAATGCTTTCCTATCTCGATTTTGCCG
AGAAGCTTGGCATTTTGACGCCGAACTACACCACGCCTTACATCGCGACGTTCATCGATCTCAAAGAATCCGG
GCCGATGGTCGTGGTTGTACCGCCGGGGCTGATTGCAGGCATGGTGCTTGACCAGTGGCAGCGCGTCATGGCC
GATCTAGGCGTGGTCGGCCCGGATATGGGCAAGGGCGGAAAGTATCTGATCCTCCCGCCCGGATACGGCCCGG
TGGAGGCCGAGGGATACTTTGTCATCCAATCCACGAGCCGGGACGTGCTGGCGGGGTTCCGCCTGCTCGATGC
CGACAAGGAAAAGGCAATTGCAGAACTCATCCCGCAGATCAAGACCTACACCTGGACGCTTGACGGAACTGGC
GAGCCGATGCCCGTGCGCGCTGCGGGAGACGTCAAGTGGACCCAAATGCCGCCCCATGGAATGGCCTATTGGG
ACAGCCTGAACGAGGTCATTCAACGAGAACCCGTCGAAGAGCGTGATCGCTTCATGATGGCGCAGTTGAAGTT
CCTCGGCATTGAGAAAGGGAAACCCTATCAACCCGACGAACGCCAGAAGAAGATTCTTCAGGACGGCGCGATC
GTCGGCGAAGCCATGGCCAAGGTAAATACGACAGACAAGCGCGTCGAACCGCCCTTCTGGGAGGGCACGAACT
GGAAACATGCGCTTGTGGTTGCGACCGACCAGAAGGCCAACACCTATGACCAGCTCGATGAGCGTGCTGCCTG
GTTCTACGAAGCGGTCGTGATCTCCAAGGCAATGCTCACCCAAACTCCGGGCGTCGGCCAGCGGTACATAGCC
TCCTACAAGGATGCAGACGACGCATGGCTCTCGGGCGAGAACAACTATCGGCTTCATGTCCCTGCCGATCCGC
CGGCCAAGGGCTTCTGGTCTGTGACGGCCTACGACGAGTCGACCCGGCAGATGCCGGTCACCGAACAGGGACG
GCCGGACATCTCGTCGCGAAAAGAAGACATAGTAAAACACGAAGATGGATCGATTGACGTATATTTCGGTCCC
ACTGCACCGAAAGGTCACGAGGCGAACTGGGTGCAGACAAATGCCGACAAGGGATGGTTCGCGTATTTCCGGT
TCTACGGACCGACCGAGCCCTTCTTCGATAAATCCTGGTCGCTGCCTGATATCGAGAAGGTCAAATAGATGAC
TGAGAAGATGACCCTTGCCTTGATCTACGGCAGCGCACGGCAGGGCCGTTTCTGCGATACGGTCGCCAGCTGG
CTCATTCGTGAGCTCGAGACATCCGACGTGTTCAGCCTGACGATCATCGATCCCGTCAGAATGAAAAGCGCTC
GCGGCGACAAATCCGCCGGACCAGCCGAGTGGATGGAACGGAAGGTCGCCGAAGCGGACGCATTCATCGTCAT
CACTCCGGAATACAACCACGGCTACCCGGCGGCGCTGAAGGAGATGATCGATGCGGTCTATGAGCCCTGGCAC
GCAAAACCGGTCGCCTTCGTTTCCTATGGCGGCGCATCGGGCGGCATAAGGGCCGTCGAGCAATTGCGGCAGG
TCTTCGGCGAATTGCATGCCGTCACCCTGCGCGACGGCATCTCCTTCGCCAAGGCCTGGTCGAAGTTCGATGC
GGCGGGAAATCTCTACAAACCGGAAGAGATGCGAGCGCCGCTGTTCCTGATGATCGACCGGCTGACCTGGTGG
GCGCGCACGCTGAAATACGCGCGCAAGGCGACCCCTTATAACGAGATCGCCGCATGAATGAGCACGGACCACG
CCCGACTGGACGGGAAGATCGCCATTGTGACCGGGGGCACGCAGGGGCTCGGTGCCACGATTGCCGCACTCTT
CGCGGAGCGCGGCGCCGCCGGCGTCGTCATTTGCGGGCGCAACGCGGCGAAGGGCGAGGCGAAAGCTGCCGAG
ATCGCCGCGGCGACGGGTTCGAAGGTCGTCTATGTACCAGCGGATCTCGAGCGGGTGGAGGATGCCCGTGCCG
TCGCGGCGGCTTGCGACCGCGCCTTCGGCCGCGTCGATGCGCTGGTCAACGCCGCAGCGATCACCGACAGGGG
TACGATCCTCGACACCAGCCCGGAACTCTTCGACCGGATGTTCGCGGTCAATGTGCGGGCGCCATTCTTCCTG
ATGCAGGAGGTGATAAAGGTGATGCGGCGCGAGAAGACAGAGGGCACGATCGTCAATATCGGCTCCATGTCGG
CGAAGGCGGGTCAGCCCTTCATCGCAGCCTATTGTGCGTCGAAGGGGGCACTGGAGGCGCTGACCAAGAACAC
CGCCTATGCCGTCCTTCGCAACCGGATACGGGTCAACGGCCTCAACATCGGCTGGATGGCCTCGGAAGGCGAA
GACCGGATCCAGCGCGAATATCACGATGCGCCGGCGAACTGGCTCGAAAAGGCGGCAGCGAGCCAGCCCTTCG
GCAGGCTCGTCGACCCGGCCGAGGTCGCGCGTGCCTGCGCCTATCTCTCATCGGCAGAATCCGGCCTCATGAC
GGGTTCCGTCATCTGCTTCGATCAGTCGATCTGGGGTGCCTATGACGCCTCGCCTCATCCGGAGGCGCCGCTC
TGAATGAATGGACTGATGATTGCAGCCCCGAGCTCCGGCTCGGGCAAGACCACGGTCACGCTCGGCCTGATGC
GGGCATTGAGACGGCGCGGGCTCTCGATCGCGCCTGGAAAGGCGGGGCCGGACTATATCGACCCGGCTTTTCA
CACGGCTGCCAGCGGCAAACCCTGCTTCAATTATGACCCTTGGGCAATGCGCCCCGAGCTGCTCCTAGCCAAT
GCGGCGGCTGCGGCGGAAGACGGCTCGGTGCTGATCATGGAGGCGATGATGGGCCTCTTTGACGGTGCAGCGG
ACGGCAGGGGCGCGCCGGCGGATCTCGCCGCAGCACTTGGACTTGCAGTGATCCTGGTCGTCGATTGCGCGCG
CCTCTCCCATTCCGTGGCCGCCCTGGTCGGCGGCTATGCCCGTCACCGCGACGATGTCCGTGTCGCGGGCGTC
ATTCTGAACAGGGTCGGCAGCGACCGGCATGAGGGAATGCTGCGCGATGCGCTTGCCGGAATTGCGATGCCGG
TCTTCGGCGTACTGCGACAGGACGCTGCACTGAAGCTGCCCGAACGCCACCTGGGCCTCGTACAGGCAGGCGA
GCATGGATCGCTGGAAGCCTTCATCGATCACGCGGCAATGCGGGTCGCATCGGGTTGCGATCTCGAGGCGGTG

CTTGCTGCGGCGACGCCGCTGACGGTGGGAGAGAGGGCCGGAACGCTAAAGCCGCTCGGCCAGCGGACAGCCG
TCGCGCGCGACGTGGCCTTCGCCTTCTGCTACGAGCATCTTCTTTCGGGCTGGCGCGGGCAGGGGGCCGAAGT
CACATTCTTCTCGCCTCTTGCGGACGAGGCGCCCGATCCGCGTGCCGATGCGGTCTATCTCCCCGGCGGTTAT
CCCGAGCTTCATGCGGAACAGCTCTCCAATGCTTCGAATTTCCGCGCGGCCATGCACAAGGCAGCTGGGGGCG
GGGCGCGCGTTTTCGGGGAGTGCGGCGGCTACATGGTGCTCGGCGAGGGGCTTGTCGCGGCGGATGGCGGGCG
CTACGAGATGCTCGGGCTTTTGCCGCTCGTCACGAGCTTCGCCGAACGCAAGCGGCATCTCGGCTACCGGCGC
GTCACGCCGGTTGACGACGTGTTTTTCCGGGGACCGATGACGGCGCACGAATTTCACTATGCCACGATCGTTT
CGGAGGGCGCGGCTGAGCCGCTCTTCACGGTACGTGACGCGGCAGGGCTCGATCTCGGCCGCGCCGGCCTCCG
TCGCCGGAACGTTGCCGGCTCCTTCATGCATCTCATCGATTTCTCGGAATGAATGTTTCACTTGCGTCGTGCC
GCAATCGCTGTGTTGGGCGCAGCGTTTTACGGTTCCATACCGACAATTGCTGCCGAACCGGTTGGAGAAGCGG
TCCGGATCAGGACGGTGGTCACCGGCAGCGGCGGACCGCTCGTGGAGCGGGACGCCGTTCACCGGGACGAGCG
AATAAGCACGTCCAGATCCGGGCTCGGCCAGTTCGTGTTCCGCGACGGCAGTAAATTGGCGGTGGGGGCAGGC
TCTTCCGTCGTCATCGACAAGTTCGTCTTCAACGATTCGAAATCGGTGCAGCAACTCACCGTCGCCGCCGCCA
AAGGCACGTTTCGCTGGATCAGCGGAAATTCGAAGCACTCGGCCTATCAGATCCTGACACCTGCCGGAACGAT
CGGCGTACGCGGAACCGTGTTCGATTTCTACATCGGCAATGACGGAACGACGGCGATCGTTCTCCTGGAGGGC
GCGGCCCGGTTCTGCGGCCCGGCGGGATGCCAGCAGCTCACCCGACGCTGCGATTGCGTGATCGCAAAGCGCG
ACGGAACCATGACGGACACGCACAGGGCCGGTCGCCGCACACTGGCGACGCTCGGAAACCGGAAGGCCCTGCC
CTTTCTTTCGGGCGACCAAAGACTGTCTGCCTCCTTCGGCAGCATGAGCGGCAGTTGCGGTATTTCGGCCGCT
GTCGAGATCAAGCCGACCGCGCCGGAACGCGCGCGGCCGCCCGAAAGAGCAAGCCCGCAAAAGGCGCCCCGCC
CGGGCAAGACACCCAGCAACGAGCCGGGCAAGCGTGGCCTCGACAAGGGGAAGAAGGGGCAGGACACGGGCAA
GAAGGGGCAGGACACCGGCAAGAAGGGCCAGGATACCGGCAAGAACGGGCAACTGGACACAGGCACGAAGAAG
GGCCGCGGTTAGATGGCTACTCGAAACGTCGTTCTGACTGACGCACAATCAGCGCTGCTTGAACGGTTGGTCG
CCTCCGGGCGCTATCAAAATGCCTCGGAGGTCTTGCGTGCCGGGTTGCGGCTGCTCGAACGGGAAGAGGCCGA
GCTGGACGATTTGCGGGCAAGGCTGAGCACGGGACTCGATGAGGCCCGTCGCGGCGATCTAGCCGAAGGTACG
GGCGAAGAGGCGATCCGCCGTGCTTTCGCGGCAGCGCATGCAGCCTCCTGAATGTATGTCATGGCTCTTGCGA
CGGATTACGACGGCACACTTGCAGACTACGGTGCCGTCCGTCCTGAAACTCTGGAGACCTTGAAGAGGCTGAA
GGAGAGCGGGCGCAAGCTTCTGCTCGTGACCGGGCGCGAGCTGCCGGACCTCAAGAGCGTGTTTCCCGAAATC
GACGTGTTCGACAAGGTCGTCGTCGAGAACGGCGCGCTGCTCTATACGCCGGAAACCGGGGAGGAGCGGCTGC
TTGCACCCTCGCCGCCGGAGGCCTTCATCGAGCGCCTCAAGGAGAAGGGCGTCGACAGGATGTCGGTCGGCCG
TTCCATCGTCGCCACCTGGGAGCCGTTCCAGACAGCCGCGCTCGAGGCGATCAACGAACTCGGCCTCGAACTT
GAGATCATCTTCAACAAGGGCGCCGTCATGGTGCTTCCGACGGGGGTGAACAAGGCCACGGGGCTCAAGGCGG
CCCTCAAGGAGATGGGGCTTTCCTTTCTCAATGTGGTGGGGGTAGGCGATGCCGAGAACGATCACGCGCTTTT
AAGGATGTGCGGCTGCGGAGCCGCGGTCGCCAACGCCCTGCCGGCGCTCAAGGATACGGCCGACGTGGTCCTC
GAGGGTGTGCGCGGGGCCGGCGTCGAGCAACTGATGAACCGGATCATGGAGTCCGACTATGCGTTGTGCGCGA
GTGCCCGTCACCAGGTGCCGATCGGTGAGGATGACGAGGGGCAAGTGGAGATCGGACCGCAGGACGTGCTGCT
GATCGCGGGGAGTTCCGGCATCGGCAAGTCGAGGCTGGCGACCGCCCTTGCGGAGCGATTACGGGAGCGGCAC
TTCCAGCTCTGCATATTCGATCCCGAGGGCGACTATGAGGGGCTTGAAGGCGCCGTGACCGTGGGTAACGGGT
CTGTCGCGCCTACGGGCAGGGAGGTGCTGGAGCTGCTGGCCAATCCGGACGACAACGTCGTCGTCAGCGCAAC
AGGCGTCGAACCCAACGACCGGCCCGAGTTCTTCGCCAACCTGATGCCGGACCTGTCGGCGCTGCGTGCCAGG
ACGGGCAGGCCGCATTGGCTGATTATCGACGAGGCCCATCACCTCATGCCGGCAGCGCGCGACAGTGCCTCGC
TCGCGCTTTCCGACGACCGCTCCGGCATGATCATGATCACGGTCCATCCGGATTCCGTCTCACCGGATGCGCT
CAAGGAGATCACTGCGGTCGCAGCGCTCGGTCCAAAGGCGCGCGACGTAATCGCGACGATATGCTCCGTCTTC
GACGAGGCGCTGCCGGCCGGGCTGGACGCTTCTTTCGGGGAAGACGAGGTTCTTTTCTGGCGGCGCACGCCGC
CCACGCCGGTTCGGCGGATCAAGGCCGAACAACCCCGGGAAGCCCGCAAGCGGCATACGCGCAAATATGCGGA
GGGCCGGTTGGGCGAGGAGGCAAGCTTCTATTTCCGCGGTCCGAAGAACGAGATGAATTTGCGCGCCCACAAT
CTGACGATGTTTCTGCAGATCGCCGAAGGCATAGACGACAGGACCTGGGAGCACCATTTGCGTCGCGGCGACT
ACTCGAAGTGGTTCGAGGAACGCATCGGCGACGAGGAATTGGCCGGGGAGGCGGCAGGCATAGAAGAGGATCG
TTCGCTCTCGCCCGCCGAAAGCCGGCAGAGGCTGGCCGAGGCGGTGCGCCGGCGCTACACCGCGCCCGCATCC
GAAGCGGGTTGAATGAGCTTTCTCTCCGCCATACCGGTCATCGCACTTCTTGCCATGCTACCCGTTCTCCTTT
CTCTGCGGCCGGTTGCCGGTGTTCGGGAGTTCTCCGCCAGTTGCGCACTCTCGGCAGCGTCGGCAGGCGCCTT
GACGGTTGCGGCGGCGTTGCAGGCGCCGGTCGCCGCAATGTTCGGCTATGCGGGTCTCGCCGTTTCCCTGTTT
TTCGTCCTCCAGGGCCTCCGCCGGTTCCTCGCATTGTCGATTCCGGCCTCGCTTTGTCTGGTGCTCGCGTTCT
CGAGCGGCGTGCTCGGCTTCATCATCGCTCTCGGCGGCCACGACAGCCCGGCCGCCGGCATGATCGTCGTTGC
GGGCGTTGTCGCAGCTCTACTTCTCGTCCTTGGTCTGGCCGCAGCCGACCGCTGGCCGGGCGAAAAGCCGGCG
ATCCAGGTCGTTGTCGTCGGCTCCGCCGCCGTGGTTTTCACCGCCCTTGCCCATGCCCTCGGCCCATCGGCGC
ACGTCTTGCCGGGACCGGCGGGCTCGCCCGCTTCGAGCCTGTTCGATTTCCTGCTCGTCACCATGCGGGTGCT
GTGCCTTCCCCTCCTCTTCCTGGCCATGATCCTCACGATGCAGGCCCGGGTCATCGCCGGATTGAGGGCCATG
ATCGCACGCGACGGCTTGACCGGTGCGCTGTCACGCGGGACGCTCATGGAAGAGGGCGAGCGGATCTTCGCCG

AGTGCGTGGCACGCCAGGAGCCGGCTGCCTTCCTGCTGCTCGACCTCGATTTCTTCAAACAAATCAACGATCA
GTATGGGCACGCCTGCGGCGACATGGCGCTCGCGCATTTTGCCGATACCGTCTCGGCTTTCCTCGCCGGACGG
GGCGTCCTCGGACGGATCGGTGGCGAGGAATTCGGCATCGTCCTGCCGGACCACACGGAAGAGCAGGCAAGGG
CGCTTGCGGAGGATATCTGCCGCGTGGTGCGTGAGACGCCGGCCGGCCGCTCGCATCAGCGCATCCGTCTGAC
CGTGAGCATCGGGATCGCCGCCGCCATTGCGGGCGAGACGATTACCGACGTGATGATACGCGCCGACCTTGCT
CTCTACGACTCAAAGGCGGATGGTCGCGACCGTTGCTCGGTAGCAAGGGATCGCCGCATCGACGCCAGCGCCC
GCGCCCTTGCGGCAGCCGCCGCGCAATTGCGCGAAGCCCGTGCCGCCCGGTCGGAACTGCGCCACTCGGCGTG
AATGACAGCCACAGCGCGCAAAGCCCTTCTCTCCCTCGGATTCCTTGCGATCGCCGGCGCGCCGGCCCTGGCG
CAAGCTCCGGCTCAACCGGGGAACCCAGCCGCCGCGTGCGGCGGCGACCTCGGCTCCTTTCTGGAGGGCGTCA
AGGCCGAAGCGGTCGCCAAGGGCATCCCCGCAGACGTCGCCGATCGGGCGCTCGCAGGCGCCGCCATCGACCA
GAAGGTGCTGAGCCGCGACCGCGCTCAGGGCGTGTTCAAGCAGACCTTCACCGAATTTTCGAAGCGTACCGTC
AGCAAGTCGCGCCTCGACATCGGTGCGCAGAAGATGCGGGAATATGCCGACGTCTTTGCCCGGGCCGAGCAGG
AGTTCGGCGTACCGGCGCCCGTGATCACCGCATTCTGGGCCATGGAGACCGACTTCGGCGCCGTGCAGGGCGA
TTTCAATACGCGTGATGCGCTGGTGACGCTGGCGCATGACTGCCGCCGCCCGGAAATGTTCCGGCCGCAGCTT
CTCGCCGCAATCGAGATGGTGCAGCACGGCGATCTCGATCCCGCCGCGACCACCGGCGCCTGGGCGGGCGAGA
TCGGTCAGGTACAGATGCTGCCTGAGGACATCATCGCCTATGGCGTCGACGGCGACGGCGACGGCCATGTCAA
TCTCAAGAAGAGTTCTCCGGATGCCATTCTGACGGCGGCGAAATTCATCCAGAGCCTGGGCTTCAAGCCCGGG
GAACCATGGATTCAGGAGGTCCGCGTTCCGGAGCAGTTGCCATGGGAAAAGACCGGCCTGCAGCCCGGCATGA
CCGCCGGCGACTGGTTCGCACTCGGTGTCGCGCCGCGTGACGGCAACACCTCCAATAGTGGGCTCGAAGCCTC
GCTGGTGCTCCCTCAGGGCCGCAAAGGTGCTGCTTTCCTGACCTACCCGAACTTCAAGATCTATCTCGAGTGG
AACCAGTCCTTCATCTACACGACCTCCGCCGCCTATTTCGCGACCCGGCTTGCCGGTGCGCCTCCCTATCAAC
AGGGAAATCCGGAACCGGGGCTCGGCGACGCCGAGATGAAGGCCCTGCAGACCAGATTGCAGGCACTTGGGCA
TGACGTCGGCAAGATCGACGGCATTCTCGGCTCGGGCACGCGGGCCGCGCTGCAGAAGGAGCAGTTGAAGCTC
GGGATTCCCGCCGACGGGTGGGCAACACCGGGCCTGCTGGAGGCGCTTTAAATGACCAACATGATGAAGGCAC
TGGTCAAGACGAAGCCGGAGGTAGGGCTCTGGATGGAGCGCGTGCCGGTGCCGGAAATCGGGCCGAACGACGT
CCTGATCCGCGTCAGGAAATCGGCGATCTGCGGCACCGACGTCCACATCTGGAACTGGGATCAATGGGCGGAG
AAGACGATCCCAGTGCCGATGGTCGTCGGACACGAATTCATGGGCGAGGTCGTCGAGGTCGGGCCGGCCGTCA
GCAAGCATCATGTCGGCGAGCGGGTTTCCGGAGAAGGTCACATCGTCTGCGGCAAATGCCGCAATTGCCGCGC
GGGCAGGGGGCATCTCTGCCGCAACACGCTCGGCGTCGGCGTCAACCGCCCCGGATCCTTCGCCGAGTTCGTC
TGCCTTCCCGAATATAATGTCGTGTCGATCCCCGACGACGTGCCGGACGAGATCGCCGCAATCTTCGATCCCT
TCGGCAACGCCGTGCATACGGCGCTCTCCTTCGATCTCGTCGGCGAGGACGTTCTGGTCACCGGTGCCGGGCC
GATCGGCATCATGGGGGCGATGGTGGCCAAGCGTTGCGGTGCCCGCAAGGTGGTGATCACCGACATCAATCCC
GTTCGTCTCGATCTCGCGCGCAAGCTCGGCATCGACCACGTCGTCGACGCGTCGAAGGAAAATCTTGCCGACG
TCATGCGGGTGATCGGCATGACCGAGGGCTTCGATGTGGGGCTGGAAATGTCGGGCGCGGCGCCCGCTTTCCG
CGACATGATCGACAAGATGAACAATGGCGGCAAGATTGCGATCCTCGGCATTGCGCCGGCGGGCTTCGAGATC
GACTGGAACAAGGTCATCTTCAAGATGCTCAACCTCAAGGGCATCTACGGTCGCGAAATGTTCGAGACCTGGT
ACAAGATGATCGCCTTCGTCCAGGGCGGGCTCGACCTCTCGCCGATAATCACCCACCGTATCGGCATCGACGA
TTTTCGCGACGGCTTCGAGGCGATGCGCTCGGGCAATTCCGGCAAGGTGGTGATGGACTGGTAGATGCCTTCA
ATCAGGGACCACTCCGAGCGGGTCTACCGCATCGCACATCAGTCTTCCGCTGCGGTGACTTCGCCGGTCGCTG
CATCGTGGCGCCGTTGCATGACGCTGCACGGCCTGGCGCCGGAGGATGCGCGTTCGCCCTGGCGTCTTTCGGC
GATCGAGTTCCGGCAGGCGCGCGAAAGCTCCGGCGCCCTCATCGCAGAGGCGGCCGGCGAGCTCGACCGGCTG
TTCGCCACTGTCGGCAAGGCCGGGTGCTGCCTGCTGCTTACCGACGGAAAGGGCGTTGCACTGGAACGACGCG
GGGCCGGCGGCGACGATGCGGACTTTCGCGGGATCGGGTTGTGGTCGGGAACCGTGTGGAGCGAGGCGAGCGT
CGGGACCAATGGCATCGGTACGGCGATCGCGGACGAGCGTCCGGTCCTCATCCAGCGCGACCAGCACTTCCTC
AGCCGCAATATCGGCCTGAGCTGCGCCACGGCGCCGATCCGTGACGAGAGCGGCAAGCTCGCCGCGGCGATAG
ACATCTCCACCTGCCGTGACGACGCATCCGAGGCGACGCTTTCGATCCTCTCGCAGGCGGTGCGCGATGCCGC
AGCGCGAATCGAGGCCAATCTTTTCCGCCGCGCTTTCGTCGGCGCCCGCATCGTTCTCGTACCCGTCGACCGG
GCGGGACCTGCACTGCTTGCGGTCGACCGTGACGATCTCGTTCTCGGCGCGACGCGGGCTGCCCGGCTGTGGC
TCGGGCTCGACGACGAGCGTATCGCCGCCGGCGTCCCGGCTTCCGACCTGCTTCAGGAAGACCTCCCGGGCGA
GGGCGGCGAATTGCCGGACGCGGAACGTGCGGCGCTGCGCCGCGCGCTCTCCCGGGCGAAGGGCAACGTATCC
ATGGCGGCCGATCTCCTCGGCATCAGCCGCGCGACCCTCTACCGCAAGATGAAGCGATTGGCCCTGAACTGAA
TGATTCTATGGAGATCAAGCGCGAGTCGCCTGGCAATTGTAGTTGGTGCCGGCTTTTGGAGCATGCCTATACT
TGTTCAGGCGCAAGAAGCTCCCAAAGAAAACAGAACTTTGCTCGCTCCTATTGTCGTCACCGCGGACAAATCC
GACGCATTCGCGATCGGCGGTTCGGTCCAGCGGCTGGACCTGACGGATATGGAGCGCTTTTCCTATAGCGACC
CCAATCGGGTATTGCGGCAGATTCCGGGTGTTTATCTTCAAGAGGAAGAGGGGTTCGGCCTTAGGCCCAACAT
AGGCATCCGTGGTTCGGGTACAGACCGCAATGCCCGCATAACGGTCATGGAAGACGGCATCCTGATCGCGCCG
GCCCCCTATGCGGCGCCTGCGGCCTATTATTTTCCCAAGATGGCGCGCATGTCGGGGGTAGAGGTGGCCAAGG
GACCGGCAGCAATAAAATACGGTCCGATGACCGTTGGCGGCGCGATCAATCTTTTCTCGACCCCTATTCCCGA

TTCCGATATCGGAACGCTGACCGGAAAGGCGGACATTCTCGGTGGCAGCTATGGCGGCCGACGTGTCCACGCC
TGGACCGGAGGTTGGGCGCCGCTGGGCGACGCGTTCGAGATCGGCGGACTTATCGAAGGGCTTTACGATAAAT
CGGATGGCTTCAAGGATATCGATCGCGGCGGCGATACCGGATTCGATCTCACCGATTGGGGCGGCAAGCTGGG
CTTTCGCACGGCCGATGGAACGCAGAATTTCGAGTTCAAGTATCAAAACTACGATGAAACCAGCAATGAGACC
TATCTCGGCCTGACCCTGGACGATTTCAAGGACACGCCTTTCAGGCGCTATAACGCCAGTCAGAAGGACGTGA
TCGACGCCGCGCATAATATCTATCAATTCAGCCACAGTTACGAGATCACACCCGACATCAACCTGACGACGAC
CGTCTACCGTACCGATACCACGCGGGCCTGGTACAAGCTCAACGATGTCCGTAACGACTCCGATACTGGCTGG
GTATCCCTCAGCAACATCCTGGCGGACCCCGATACGTATTCGACGCAGATGGCCGATATCGTCGGTGCGGACG
GATATACCGGCCGAGCGGGGGCATTGCGTGTGCGCAACAACGCCCGCGCCTATCAGGCGACCGGCATTCAAAG
CGTGCTGACCACGAATTTCGATACCGGCGAACTCAGCCACGAACTCGAATTGTCCGCGCGCTATCATCAGGAC
GAGGAAGACCGCTTCCAGCAGGACGATCGCTACCAGATGGTCAATGGGCGCATGATCCTGACCTCCGCGGGCG
CGCCGGGCAGCCAGTCCAACCGCCTGAATGAAGCCAAGGCATGGGCTTTCTTCGCGCGCGACACGATCGAAGC
CGGACCGTTCACCTTCGTTCCCGGCCTGCGCTATGAATCGATCAATCTGGACCAGACGAACTGGGGGAGCGCC
GACCCCGGCCGCAACGGGCCGACCACTGCAAAACAGTCCTATGTCGATGTGTTCATCCCCGGCCTGTCGGCAA
CCTGGAGCGCCACCGACGAGGTTCTGCTGGTGGCCGGTGTCCATCGCGGCTTTGCCAGCCCCGCTCCGGGATC
GGACGTGGATCCCGAGACTTCATGGAACTACGAATCCGGCGTGAAGTATGATTCCGACGGCTGGCGCGCCGGT
GCCATAGGCTTCATCAACAGCTATTCGAACCTGCTTGGAACCTGCACCGCATCCACCGGAGGCAATTGCACCA
TTGGTGATCAGTTCGAGGGCGGCGAGGTCAGCGTCAAAGGCATCGAATTGACCGTTGGCCGCACATTTGGCAG
TATCGATAGTAACGGCTTCGAGGTTCCGGTAACGCTTGCCTACACCTACACGAACGCCAGGTTCCGCCGTTCC
GAGCCGGCCCAGGTCGATCACGCCCGTGCAGCGGTGTGTATCCGGATCGACCATGAAGTTCGGCAGGCATGCG
TCGCCATGACAGATTACGAGATCGGAAGGCTCGTCGATCAGGTGCCTCGGCAGTTCGGCCCGCAGAGCGTCGA
GCAGTTCGCCCGGCGGAGTGTTCTGATCCTCCGGGGCCAGGAAATCCGGATTGACGGCATCGCGCCGGACGAC
ATCCGCGGCCCGGTCGAACATCGACGCGAGCCGCCGCCCGAAAGGGCAGGCTTCCGTCGGCAATTCATGCAGC
AGCTTCAATTGCCGCAGGATCGACGGCCAGGCTTTCTTGAGGTCGACGGCGGAGAGCTCGCTTGCCGGCACAC
CCGGCAGCGCGCTGATGACGAGGCATGCTTCATCTGCCGCGATCCACTCGCAGACGGAGGGCGAGCCCAGGCG
GAACGGCGCCAGCCAAGCGACACGATCGCGCTCCCCCTCGAGCAGTGCTGCGGCCTTGCCGCTCGCAATCTTC
GCATAGGCGCCGCCGTCGCTGCGACGATAGACGCGGTCACCGGATTCGCCGTGACGAACCGGCGTCCATTCCC
GACCCGTGGGCAGTTTCGGAAGCAGAATTTCGTCACCGGCGTGGCGCCTGCTCATGAAGCCCGCCATTGCAAA
TGCGCAGACGCCATCATCCCCGTCCGCGATAGGTCGGCACGCCCTGATCCGGCAGCCAGACGCCCTCCGGCGG
GTTTCCCGTCTGCCAGAAAACGTCGATCGGGATGCCGCCGCGGGGATACCAGTAGGCCCCGATCCGGAGCCAC
TTCGGAGAAAGCAGCGAAACCAGGCGCTTGGCGATCTCGATCGTGCAATCCTCGTGGAAAGCGCCGTGGTTGC
GGAACGAATGCAGGAACAGCTTGAATGCTCAACCGGCATCATCGCCTGGCGCTTTTGGCTTGCACGGCAGCAA
TATTCGCGCTCCCGATCCCCCCGGTTCTGGCGCAGAGCGCGCCAACCGAAACGGCGGCGGAAGGAAATGCCAA
CACGACCGTGCTGAAGAAGATCGTCGCGAAAGGCGACCGCCTGGCAGGTGCGCAGCGTGGCGGCATCGCGGAT
ACGCCGCTTGCGACCGAGATCGACGCAAAGACCCTGGAGGAGAAGCAGGTCACGGATCTCGACGATCTCGGCC
GCAGCGTCGACGCAGGGATAAATGCATCGCGTGCCGACTTCGGTATCAATCTTCGCGGTCTTTCCGGTCCTCG
TATCGTGACCACGATCGACGGCGTTCCGATCCCCTATATCTCGAACAGCGCCCGTCAGGGTGCCTTTGCCTCG
ATCAATGCGAACGGCGGCGGGGACATGTTCGATTTCAATTCGCTCTCCGTGGTCGACATAGTGCGCGGGGCGG
ATTCGAGCCGTGGCGGTTCCGGCATGCTCGGGGGCGCCGTGGTGCTGCGCACGCTCGAGCCCGAGGATGTCAT
ATCCGACGGAAAGGATTGGGGCGCGATCTTCAGGTCCATCTATGACAGCGAAGACGACAGCATTGCGGGCTCG
GTTGCTGGAGCCCACAGATTCGGCCAGACCTCCGTTCTCTTTCAGGGCAGCTACCGCAAGGGGAATGAACGGG
ATAACGAGGGGACGGTCGGTGGCTACGGATCCGCCCGCACGGAGCCCAACCCTACCGATTTCGACCAGAACAA
CTTGCTCTTCAAGTTTCGGCATGAACTCGAAGGCGGTCACAGAAACGGCCTGACGGCGGAAAGCTTCCGCCGC
GACGCCGACAATGATCTCAGAGCGGAACAGGGGCGCCGGTACAAGATCGGCGACTATACCGGTTTTGAAGACC
GCGACCGCAAGCGCGTCTCGCTGGACTATGATTTCGAGGCCGCCTCGTCGGACGACTTCTTCAGCTTCGCCAG
GGCATCGCTCTACTGGCAGGATCTGGAACGCTCCTCCGGCTCGAATGGCCGGACGATCGCCGATGTGCCCTAC
GGGCGCGACAATTCGATCAGCAACGAGAGCGTGGGTTTCAACGGACGCGCAGGCAAGGATTTCGAAACCGGCG
GTTTCGATCATTCGCTAACCTTCGGCCTCGATGTCGCCCGTTCCGAGTGGAGCCAGTATACCTCGGCGGTTTG
CCCGACGCCGGCGACATGCCCGGCGCTCAACAACCAGTCCGAAGTCCCCGACGTGAGGAGCATGACGGTCGGC
GCCATATTGGAAGACCGCATTTCGGTCGGCGACAGCGCGTTTGCGCTCACGCCGGGGTTGCGCTTCGACTGGT
TCCAATATGACCCTCAGCTCAATGCAGGCTTCGAGTCGAATACAGGATCGGGAATCTTCGGGGATCTGAAGGC
CAGGGATGGGGTTCGCCTGTCGCCGAAGCTCCTCGCGACCTATGACGTTACGCCGGATGTCGAGCTGTTCGCA
CAATGGTCGATGGCCTTCCGGGCGCCGACCGTGGACGAGCTTTATAGCCGCTTCTACAACCCGTTCGGAAACT
ATGCCCAGCTCGGCAATCCGGATCTCAAGCCGGAAACCGGCAAAGGATTCGAGATCGGTGCCAATTTCGACAC
CGGTGAGCTGAGCGGCCGGGTCGCGGCGTTCCACAATATCTATGACAATTTCATTGAAACCGGCGATTCCATC
AACAGCGACACCGGGATCAGGGAATTCAAATATGCCAACGTCAACAAGGCCCGAATCTCGGGTATCGAGTTGA
GTGCGTTGAAAACCTTCGACAACGGCTTCAACCTCCATGCGTCGCTTGCCTACTCCTATGGCAAGAACGAGGA
CGAGGGCACGCGGCTGCGGACGGTCGCGCCATTCAAGGCGATCATCGGCGGCGGCTACAGCCAGGAAACCTTC

GGTGTCGATGTCTCGACGACGGTCTCGGCAGCCATGCCGGACGATAACGACAGCGAAACCTTCGATGCCCCCG
GCTACGGTCTCGTGGATATGACCGGGTGGTGGACGCCCGAAAGCTTCAAGGGATTGAGGGTAGAGGCCGGGGT
CTATAACATCTTCGACAAGAAGTATTTCAACGCGCTCGGCGTACGTGGCGTCGACCTCGCCTCATCGTCCGCT
CAGCCGCGCGACTTCTATTCCGAACCGGGACGCACGTTCAAAGTCTCGTTGACGCAACGCTTCTGAATGCAGC
CTCTTCCGCCTGAAAAACACGAGGACGCCGAGATAGCAGCCGGTTTCCTTTCGGCCATGGCAAATCCGAAACG
CCTGCTCATCCTCGACTCTCTCGTCAAGGAGGAAATGGCGGTTGGCGCCCTGGCCCATAAGGTCGGGCTGAGC
CAATCGGCTCTTTCTCAGCACCTTTCGAAGCTTCGTGCGCAGAACCTGGTCAGCACCCGTCGTGACGCACAGA
CGATCTATTATTCGAGCTCGTCCGACGCCGTATTGAAGATTCTGGGCGCACTATCCGACATTTACGGCGACGA
CACCGATGCCGTGGAAGAAAAGCCTCTCGTTCGCAAATCGGCGTAAATGGGTGGCAAGCGGCACCAGAATTCA
GGCAAGGGCGGCAAGGGCAGGGCGAAAGACGCGGCGAGCGCGCCGCAACCGAATGCCGGCAAGGTAACGCTCG
AGGGCCGCAGCTTCCTCTATGTCGGCGGCCGCGACTGCCAGGTCGCGCATCTTCGCCAGATCGCCAGTTCCTA
CGGTGCGGAACTCATCCACCATGACGGCGGTTTACGAGAGGCGGTATCGCGCATCGATCGTGTCCTGCCCTCG
GTCGACTGCGTCTTCTGCCCGATCGACTGCATCAGCCACGATGCGTGTCTTCGCGTGAAGACGGGCTGCAAGA
AGTGGGGCAAGGCCTTCGTGCCGCTGCGCAACGGCTCGAAGTCGAGCCTCGAGCGGGCGCTTCAGGATCTGAC
CGGAAACGGCAGCGAGAATTGAATGATCAGAGCATCCGATATTTCCGTCCGCCTCGCCGGAAGACAGGTGCTG
CACGGCATCTCCCTCGATGCCGCCCCCGGTGCAATGACTGCAATCGTCGGCCCGAACGGATCGGGAAAGACCA
CGACCTTGAAAGCGATCTCGGGCGAGCTCACGCCTTCGGCCGGCAAGGTCACCATCAACGGTCGCGACATCGC
AAGCCTGAAACCGTGGGAGCTTGCGCTCAAGCGCGGCGTGCTGCCGCAGTCGACCGTCATTTCCTTTCCGTTC
ACCGTTCGGGAGATCGTGCGCCTCGGCCTCATGGCCAACGGCGGCGAGGCTTCCGCCCACGGCCGGATTGCCG
ACCAGGCGCTCGAAGCCGTCGATCTTGCCGGATTTTCGGGGCGCTTCTACCAGGAGCTATCGGGCGGCGAACA
GCAGCGGGTCCAGCTCGCGCGCGTCCTCTGCCAGATCTCGGCCCCGGTCGCGGCCGGTGAACCACGCTATCTC
CTGCTCGATGAGCCGGTATCGAGCCTCGACATCCGCCACCAGCTCACGATCATGCGGCTTGCGCGCCAATTCT
GCGCCGACGGCGGCGGTGTCGTCGCGGTCATGCACGATCTCAACCTTACATCGATGTTTGCCGATCAGATCGT
GATGATGAAAGCCGGCCGCATCCGGGCGCGTGGAGCACCGAAAGACGTGCTGACCGACGAGACCATGGAAGCG
GTCTTCGGCTGCCGAATGCGGGTGAGCGTCGCTCCGGCACACGATATCCCGTTCGTATTGCCGCAGTCCGCCA
CGATGTAAATGTCCTACAGAATGTCGCGCGCGGCCTATGCCAACATGTTCGGTCCGACCGTGGGCGACAAGGT
GCGCCTCGCCGACACCGAGCTCTTCATCGAGGTCGAGAAGGATTTCACCACGCATGGCGAGGAGGTGAAGTTC
GGGGGCGGCAAGGTCATCCGTGACGGCATGGGCCAAAGCCAGGTGACGCGCGAAGGCGGCGCGGTCGACACGG
TGATCACCAATGCGCTTATCCTCGATCATTGGGGGATCGTGAAGGCGGATATCGGGCTGAAGGACGGGCGGAT
CGCGGCAATAGGCAAGGCCGGCAATCCGGACATGCAGCCGGGCGTCACCATTATCGTCGGCCCGGGCACGGAG
GTCATCGCCGGCGAGGGCAAGATCGTCACGGCCGGGGGCATGGACAGCCATATACATTTCATCTGCCCGCAGC
AGATCGAAGAGGCGTTGATGAGCGGCCTGACCTGCATGCTCGGCGGCGGGACGGGACCGGCGCACGGTACGCT
CGCCACGACCTGCACGCCCGGACCGTGGCACATCGCCCGCATGATCGAAGCGGCCGATGCGTTTCCGATGAAT
CTCGCCTTTGCGGGCAAGGGAAATGCGTCGCTGCCCGGCGCGCTCGTGGAAATGGTACTCGGCGGCGCGACCT
CGCTGAAGCTGCACGAGGATTGGGGCACGACGCCGGCCGCCATCGACTGCTGCCTCTCCGTTGCCGACGAATA
CGACGTGCAGGTTATGATCCATACCGACACACTCAACGAAAGCGGCTTCGTCGAGGATACGATCGCGGCGATC
AAGGGCAGGACGATCCACGCCTACCACACGGAAGGCGCCGGCGGCGGGCATGCGCCTGATATCATCAGGATCT
GCGGCCAGCCGAACGTCATCCCGTCCTCGACCAATCCGACGCGGCCCTATACGGTGAACACGCTTGCGGAACA
TCTCGACATGCTGATGGTATGCCATCACCTGTCGCCCACCATTCCGGAAGACATCGCCTTCGCCGAAAGCCGC
ATCCGCAAGGAGACGATCGCAGCGGAGGATATCCTCCACGATATCGGCGCCTTCTCGATCATTTCCTCCGACA
GCCAGGCGATGGGGCGGGTCGGCGAGGTGGCGATCCGCACCTGGCAGACCGCCGACAAGATGAAGCGCCAGCG
CGGACGGCTCAAGGAGGAGACCGGCGACAACGACAATTTCCGGGTCAAGCGCTACATCGCCAAATATACGATC
AACCCGGCGATAGCCCATGGCCTCAGCCATGAGATCGGCTCGCTCGAGGTCGGCAAGCGCGCCGATCTGGTCC
TCTGGAATCCCGCCTTCTTCGGCGTGAAGCCCGACATGGTGCTTCTCGGCGGCACGATCGCCGCAGCACCCAT
GGGCGACCCGAACGCCTCCATCCCCACGCCGCAGCCGGTCCACTACCGGCCGATGTTCGGCGCCTATGGAAGG
AGCCGGACCAACTCCTCGGTCACCTTCGTCTCGCAGGCGTCGCTCGATGCCGGGCTCGCCGGCAGGCTCGGCG
TCGCCAAGGAGCTCGTCGCTGTCCAGAATACCCGCGGCGGCATCGGCAAGGCGTCGATGATCCACAACAGCCT
GACGCCGCATATCGAGGTCGACCCGGAAACCTACGAGGTCCGCGCCGACGGCGAGTTCCTGACCTGCGAGCCG
GCGACGGTGCTGCCGATGGCGCAGCGGTATTTCCTGTTCTAGATGGCCTTTGGGACCGACACCATGACAAAAG
ACCCCATCCTCAAGGTTGAACGCCTGTCGATGCGCTTCGGCGGTCTCATGGCCATCAACGACTTCTCCTTCGA
GGCGGAGCGCGGCGAGATTACCGCGCTGATCGGTCCGAACGGAGCGGGCAAGACGACGGTGTTCAACTGCATC
ACCGGTTTCTACAAACCGACGATGGGCATGATCACCATGCGGCAGAAGTCGGGCGCCGAATTCCTTCTGGAAC
GGCTGCCGGATTTCGAGATCACCAAAAAGGCCAAGGTGGCTCGGACCTTCCAGAACATCCGCATGTTCTCGGG
CCTGACGGTGCTCGAAAATCTGCTCGTTGCCCAGCACAACAAGCTGATGCGGGCATCCGGCTATACCATTCTC
GGCCTGTTCGGCTTCCCGGCCTATCGCGAGGCATCCAGGGAATCGATCGAGCTCGCCAGGCACTGGCTCGAAA
AGGCATCGCTGACCGAGCGGGCCGACGATCCGGCCGGCGACCTGCCCTATGGCGCTCAGCGGCGGCTCGAGAT
CGCCCGCGCAATGTGTACCGGGCCGGAGCTTCTCTGCCTGGACGAGCCCGCGGCGGGTCTCAATCCGCGTGAA
TCGCTCGCCCTCAATGCGCTGCTGCAGGAGATCCGCCGCGATACCGGCACCTCCATCCTGTTGATCGAGCACG

ATATGTCCGTGGTGATGGAAATATCGGATCACGTCGTCGTGCTCGAATACGGTCAGAAGATTTCCGACGGCAA
CCCGGACTTCGTGAAGAACGATCCAAGGGTCATTGCGGCCTATCTGGGTGTCGAGGATGAAGAGGTTGAAGAG
GTGATCGGAGAAATCGAGGAAATCGAGGGCGAGCGTGGGGGGCCCGGGCAATGAATGAAAAAGTTGACCCTCA
ACGGACTGATCGGCGGCATCCTGATCGCCATCCTGCTCGTCACCGCTGCCGTCGGTCTCTTCTGGACACCGTT
CGACCCGATGAAACTCTCCTTCACGGCGCGGCTTGCAGCTCCCGGCCCCGCGCATCTGCTCGGCACCGACGAA
TTCGGCCGCGATGTCTTGAGCCGCCTGATGGTCGGCGCCCGCGCCAGCGTCTGGATCGGCGTGCTCACCGTCG
GCTTCGCCACTGTCTGCGGGACGCTCATCGGCCTCATCAGCGGTTATGCGCGCGGCTGGGTGGACGGCGTCAT
CATGGCTGTCAACAACGCGCTTCTCGCTTTTCCCGGCATCCTGCTGGCGCTAGGGCTGCTCGCCGTCTTCGGC
GCCAACCAGTACGGCATCATCTTCGCGCTCGGCATCGCCTATACGCCTTCCATGGCGCGCGTCGTGCGAGGCG
CCGTGCTTTCGCTGAGGGAGCGGGAGTTCATCGAGGCCTCGCGGGTAATGGGCAATGGCGAACTTTATACGAT
GTTGCGCCATATCCTGCCGAACTGCGTGGCGCCGATCACGGTTCTCGCCACCTCCATGTTCGGCTGGGCCATC
CTGTCGGAAAGCGCGCTCTCGTTTCTCGGCCTCGGCGTGCCGCCGCCGGCGCCGACCTGGGGCAACATGCTGG
CGGCCGGGCGGCCATTCCTCCAGCAGGCCGCATGGCTCGGATTGTTCCCGGGTCTGTGCATCGCGCTGACGCT
GCTTGGCATCAATCTTCTGGGCGACGCGCTCCGCGACAGGCTCGACCCGCGCATGAGAGGCCTGAAATGAATG
CGCCAGCCAGGGAGGGGAAAAGTGTACGCTGAGCTTTTCAGACTGAATGGGCGCGCCGCTTTCGTGACCGGCG
GCAGTCGCGGCATCGGCTTCGCCTGTGCCGAGGCGCTTGGCGAAGCCGGTGCGCGCGTGGCCATCTCCGCCCG
CTCGCGCGACGAAGGCGAAAAGGCTGTCCGGCAACTGAGGCAGAAGGGGATCGAGGCGATCTACCTGCCCGCC
GACATTTCCAACGAGAGCGCTGCGCAGCAGGTCGTCAGGCAGGCAGCAGCAGAGTTGGGCGGGCTCGACATCC
TCGTGAACAATGCCGGGATCGCGCGCCATTGCGACAGCCTGAAACTCGAGCCGGAAACCTGGGACGAGGTGAT
CAACACCAATCTGACCGGCCTTTTCTGGTGCTGTCGCGCGGCTATCGAGACGATGTCGGCGGCCGGGCGCGGC
TCGATCGTCAACATCGGATCGATCTCGGGTTACATCAGCAACCTGCCGCAGAACCAGGTCGCCTACAACGCCA
GCAAGGCGGGCGTGCACATGTTGACGAAGTCGCTCGCGGGCGAATTCGCCAAGAGCAACATCCGCATCAACGC
CGTCGCCCCCGGCTATATCGAGACGGCGATGACGCAGGGTGGTCTGGATGACCCCGAATGGTCGAAGATCTGG
CTCGGCATGACGCCTTTGGGCCGCGCGGGCAAGGCCAGCGAAGTGGCGGCGGCGGTGCTGTTCCTCGCTTCGG
ACGCCGCCAGCTATATCACCGGCTCGGTGCTCACCATCGACGGTGGCTACACCATCCACTGAATGGCACGGGC
ATTCGGCGGTCCGAACAAATACATCCAGCGTGCGGGCGAGATCGACAAGCTGGCGGCCTATCTGGCGCCGCTC
GGCAAACGCGCGCTCGTCCTGATCGACCGCGTGCTGTTCGACGCACTCAGCGAACGCATCGGCAAAAGCTGCG
GCGACTCGCTCGATATCCGCTTCGAGCGGTTCGGCGGAGAGTGCTGCACCTCGGAGATCGAGCGCGTACGCAA
GGTCGCAATCGAGCACGGCAGCGATATTCTCGTGGGCGTGGGTGGGGGCAAGACGGCCGACACCGCCAAGATC
GTCGCAATCGACACTGGCGCGCGCATCGTCATCGCACCGACGATCGCCTCGACCGACGCGCCCTGCAGCGCGA
TTGCCGTGCGCTACACCGAACATGGGGTTTACGAGGAGGCGCTCCGCCTGCCCCGCAACCCGGACGCCGTCGT
CGTCGATTCCGCGCTGGTGGCAGCCGCGCCGGCACGCTTCCTCGTCGCGGGGATCGGCGATGCGCTTTCGACC
TGGTTCGAGGCCCGCTCCAATATCGAGTCGCGCACCGACAATTATGTGGCCGGCGGCTTTCCCGCTACCGAAG
CCGGGATGGCGATCGCCCGGCATTGCCAGGATGTGCTGACACGCGATGCCGTCAAGGCGAAGATCGCCGTCGA
GGCCGGCCTGCTGACCCCAGCGGTCGAAAACATCATCGAGGCGAACACGCTGCTGTCGGGCCTCGGTTTCGAG
AATTGCGGCTGCTCGGCCGCCCATGGCATCCATGACGGCCTGACAGTGCTGGAGGAGGTGCATGGGTATTTCC
ACGGCGAGAAGGTCGCCTTCGGCACGCTTTGCCTGCTGATGCTGGAGAACCGCGACCGAGCCGAGATCGAGGC
GATGATACGCTTCTGCCGCAGCGTCGGACTGCCGACCAAACTGGCGGACCTCGGCATCGTCGATGACGTCCCC
GCCAAGATCGGCCGCGTGGCGGAAGCCGCCTGCCGGCCCGGCAACATCATCTATGCCACGCCGGTCACCATTA
CCGTACCGGCCGTGCGCGACGCGATCCTGGCGCTCGACGCCTTTTCACGCAGCATCCACTGAATGACCGGCAG
AAAGCCGCTCGTCATGATCACCGGCGCAAGCGCCGGCATCGGTCAGGAAACCGCACGCGTCTTCTCGGCTGCC
GGTTATCCCCTGCTTCTGATCGCCCGCCGATCCGAACTGATCGAGGCGATGGCGCTTCCGAACATGCTCGCGG
TCGCCGCCGACGTCCGTGACTATGATGCGCTCGCAAGCGCGATCCGGCAGGGTGAGGCGCGGTTCGGCCCGGT
CGACTGCCTCATCAACAATGCCGGCGTCTCCCGTCTCGCCCGGCTCGACGAGCAGGATCCTGCGCAATGGCGC
GATCTCGTCGACATCAACTGCCTCGGCGTTCTCAACGGCATGCACGCGGTCGCTCCCGGCATGAAGGAGCGGC
GCTGCGGCACCATTGTCAATGTCTCCTCCATCGCAGGCCGCAAGGTCTATCCGCACCATGACGTCTATGGCGG
CACCAAGCATTTCGTGCATGCCGTCAGCGAAGGCATGCGCCAGACCATGTCGTCATATCGCGTCCGCGTCATG
GTCATCTCGCCCGGTGTTGCGAAGTCGGAGATCGACAAGACCATCACCCACCAGGACGCCTACGATTTCTGGG
ACAGCGGCCGCAAACAGCTCGACGGCGGGCTGGATGCAAACGACATCGCGCGGACCATGCTCTTTGCCTACGA
GCTGCCGCAGGACGTCAATCTCCAGGAGATGACGATCACCCACACAGGCCAGGAATTCTGAATGATCAAGACT
TCCAGACTGTTTCTCGCAGCGGCAAGCTTCGCGCTTGCCTCTTCCACAGCCATTGCCGACGACGACAAGATCC
TGATCGGCGGCGCACTCTCGATGACGGGCATTCAGGCTCCCCTCGACACGCCCGGCTACAACGGCGCGAAGGT
CGCCGTGAAGTATCTCAACGACAATGGCGGTTTGCTGGGCAAGCAGATCGAGTTCATCAACATCGACGGCAAG
TCGGATCCGGTGACGGTCGGCAATGTCGCGGTCGAACTGATCGACAAGGGTGCTGAGATCATCGTCGCACCGT
GCGATTTCGACTTCGGTTCGCCCGCGAGCCGGGAGGCGCAATCGGCAGGCCTCGTCGGCATCTCGACTTGCGC
CTCCGACCCGCTCTATTCCTCCTGGTCGCTCGGCGACAAGCAGTTCACGCTCTCCATGTGGAATACCACGATG
GGCGCGACGGCCGCCGATTTCGCCGTGAAGGAAAAGGGCTGGAAGACGGCCTATGTCGTCACCGACCAGTTCA
TCGCCTATACCAAGTCGCTCTCGAAATATTTCGTGGAGCAATTCAAGGCCGATGGCGGCGAGATTCTGCTCGA

AGACACCTACACGAACGGCGACAACAATTTTTCCGCCCAGCTCGCCCGTCTCCAGGCGCTCGGCAAGAAGCCG
GACGTGATCTTCGTCTCCTCTTACGGCACCGACATCGGTGTGATCATCCGCGCGTTGCGCGAGGTCGGCTACG
ATGCGCCGGTTCTCGGCGGTGACGCCTATGACGACCCGGCCATGCATGAGGCGCTCGGCGAAAAATTCGGCAA
CGACGTCTATTTCGTCACGCATACCTGGATGGGCCCGGAGGCACATCCGGACATGCCGAAGTTCATCGAGCTC
TACAAGGAGATGTTCGGCAAGGCGCCGGACACGTCCTTCGTCTCGACGGGCTGGGATACGATCATGCTGCTCG
CCGAGGCGGTCAAGGCGGCCGGAACGACCGAAGGCGCAGCGCTCGCCAAGGCATTGGAAGATGGCCAGTTCAA
GCTGCTGACCGGCGATCTCGACTACGGCACGAACGAGGAAGGCCATGTGCCGAACAAGGCTGCGGCCGTGATC
GAGCTCAAGGGCGGCAAGCCGAGCTTCGTCGGATGGCGTAAGCCGGAATCCCTGCCTAAGCCCTGAATGAGCA
TCCAGACCTCTCAAACTCCGCCCGTCACGCGTCCTTCGCTTTTCCAGCGTCTGCGCCAAAGCGATGTGTGGTG
GTCGTTCACGCATAGCAAGACGGCGATGTTCAGCGCCGCGCTTCTGGCGATCCTGATCCTGACGGCACTGTTC
GCACCGCTGATCGCACCGCAGAACCCCTATGACGGCGCAGCGCTCGACATGTGGAAGGCCGAACTGCCGCCGG
TATGGGAGGAAGGTGGAGAATGGCCCTATCTTCTCGGCACCGATACGCAGGGCCGCGACATGCTGTCGGCCAT
TCTCTACGGTACGCGCATTTCCATCGTCATCGGCATCGCCTCGGTGGCGCTCTCCCTGCTGATCGGCATGAGC
GCGGGGCTGATCGCCGGCTACTTCGGCGGGTTCATCGACAATCTGCTGATGCGCTTCGGCGACGTCACGCTGT
CCATTCCGACCATCCTCGTCGCCATTCTGGTCTCGACGGTCGTGCGTCAGATGCTGCCCGTCAGCCTGCGAGA
GGTCGGCGCGTCTGCAGTGCTCATCCTGGCCATCTCGCTATCCGCCTGGGTGCAATATGCGCGCACGGTGCGC
GCGCAGGCCATCGTCGAAACCGGCAAGGACTACGTGTCGGCGGCGCGGCTGATCGGCGTGCCTGCCCGGCGCA
TCATGGCCAACCATATCCTGCCCAATACGCTGACGCCGATCATGGTGGCGGCGACGCTCAACTTCGGCATGGC
GATCCTCACCGAAGCCACGCTTTCGTTCCTGGGCATCGGCATGCCGCCGAGCCAGCCGTCTCTGGGAACGCTG
ATCCGCATCGGCAACCAGTTCCTGTTTTCCGGATCCTGGTGGATCGTGCTCTTCCCCGTCATTCAGCTCTGCC
TGCTGGTCGTGGCCGTCAACATGCTCGGCGACTGGCTGCGCGATGCTCTCAATCCGAAACTGAGGTAAATGAG
CAGCGATAAGAACATGCTTCGTTCGCTTGGCGACCACCCGCCGATCCTAGCGGACGGCCGTCGCGCACCGGAC
CGGCGCGAGATCTCGATGCGCTGGCTCTCGGGCACGTTCTTGACCGGCATCACCTCAAGTCTTCTGATGGGCG
TCGCGCTCTTTGCGGCGCTCGACGGCCGGCAGCAACTGGCGATTCCGGCGGAAGCCTTTGCCGCGCTCGATCC
GTCGGCGCCGGTCGGCGCCGCCAAACGGGGAGATCGCGTTCTTTCCCCCAACATCGTCGCCAAGCCAGCCGAC
AAGACCGTGATGGAAGTCTCGACCATGATCCATGACGGCGAGAAGGAGGTGGTGCGCCGCCAGCCCTTCGTGC
ATGTCAAGATGGCGCTTGCCGCCAACCATCAGACGTCCGAATCCTACCCCGACTTCGACCCGCTGGCGATCTT
CTCCACGGACGAGGCGGACGCCGAGGCGCCGCCGGTGAAGACCGGAACGATCTACGGCTCGGACGTCGAGTCC
GAGGTTGCCTTGAAGACCGTGGATTTTCCGCTGAACGGCGGCGGCTTCGCACTCGGGCCCTCGATGTCGCTCG
ACGAGGTCGAGGAGAACGTCCGCACCAACGGCTCGGTGCTGACCGAAGGCAATACCCAGGTCGCCTCGCTCTT
CTATGTCGACCCGCAACGCTTCGCTTCCGACGCCGACAACCTCGACCTGATGCAGGGCCTTGCCGCGCGCGTC
GTCGAGGAGAACATGAGCGTTTCCTCCTACGAGAACATCACGAAGCAGAGCATCGAATACGCCGACGACATCA
TACCGGTGCGCCGCGCCACGCCCATCGCGACGGCGATGATCAATGCGGGATATGCCAAGGCTCAGGCGGAGGA
CGCGGCCGGCTATCTCGAAGAAGCGCTCGGCGCCAAGGAACTCGGCCCCGGCGACGTGCTGCGCATCGGCATC
ATTCAGAAGGGCGAAGAGGCAAAGATCGTCCGCGCCACGATCTATTCCCATAGCCGGCACGTGCTGACCATGG
CAGTGGACGACCGCGGCCGTTTCATTCCGGGCGCCGAGCCGGCGAAGCTCGAAGCGGTGGCGGCTGCCTTCGA
CGATACCGGCAGGCCGGTGATCTCCAGCGGCCACGACCTGCCGAGGGTTTACGACGGCATCTACCGTGCGGCC
CTTTCCTACGGCATGAACGCCGACATGATCGCGCTCGTCGTCAAGCTTTTGGCAAGCAATGTCGACTTCCAGG
CTCAGCTGAAGCCGACGGACTCTCTCGAAGCGTTTTTCTCGGTCACCGATGAAAGCGGCCGGGCAACGGAAGA
TTCGGAACTGCTCTACGTCAACGCCAAATTCGGTGACGCCGAGACGCGGTTCTATCGCTTCCAGGATCCGGAC
GACAATTCGATCGATTATTTCGACAAGGACGGCAAGAGCATCCGCCAGTTCCTGTTGCGCAATCCCGTTCCGA
ACGGCCATTTCCGCTCCGGCTTCGGCATGCGCCGCCACCCTATCCTCGGCTTCTCGCGCATGCACACCGGCGT
CGACTGGTCCGCTCCGCGCGGCACGCCGATCATCGCGGCCGGAAACGGCGTCGTGCAGAAGGCCGGCTGGGAC
TCCGGCGGCTACGGCAACCAGACGCTGATCCGCCACGCCAACGGCTATGTGTCGTCCTATAACCACCAAAGCG
CGATCGCCAAAAGCGTCAAGCCGGGAACGAAGGTCGTCCAGGGTCAGGTGATCGGCTGGGTCGGAACGACCGG
CCTTTCGACCGGCCCGCACCTCCACTACGAGTTGATCGTCAACGGCAACAAGGTGGATCCCTTGCGTATCCGG
CTGCCGGGAGGAAAATCGCTCGGCGGCGAGGCGCTGGCGAAGTTCGAGAAGGAACGCGAACGCATCGACGAGC
TTCTCGGCGACGATGCGAACGAGGTCGCAAGCAAGTAGATGAATGAGAACCGTGTTCCCCGCCGCGAGTCAAA
CCCTCCGCCGCAGCAGCAGCAACGCCGCAGCGAGATGCGCGTGCGCCCGACGGGGCCGATCGTGCCTTCGGTC
AACGTTTCCGGCCATGCGCTCATGTGCGTCATCGCCATCATGTCCTTTCTCGCCTGCCTGACCCTCGGCGGCG
TCAGCATGGTGCGGGCGACCGCCCAGAGCTGGCAATCGCAGATCTCCCGTGAGATCACCATCCAGATCAAGCC
GGACGACAACCTCGACATGGAGAGGGCGCTCGCCGATGCCCGGGATCTGGCGCTGACCTTCAGCGGCACGACG
GGCGGCACGATCATCGACCGCGCGGCGACGGCACGCCTCCTGGAACCGTGGCTCGGTGGCGGGCTCGACCTGA
ACGAACTGCCCGTGCCGCGGCTCGTCGTCGTCACGATCGACGAGAGCAATCCCCCCGATTTCGCCGCGATGCG
AAAGGCGCTCACCGAAACCATCCCGCAGGCCTTCCTCGATGATCATCGCACCTGGGTCGATCGCCTGGTAGCA
ATGGCGAGTACAACCACGATGATCGGCACCGGCGTCTTGGTACTGGTCTTCTCCGCGATGGTGCTGACGGTGG
TTTTCGCCACCCGCGGCGCGCTGTCCGGCAATCGCCACATCGTCGAGGTCCTTCATTTCGTCGGAGCGGAAGC
GAGTTTCGTCGCCGCCGAATTCCAGAAGCACTTTCTCCGAATCAGTCTGAAGGGCGCCGGCGCCGGCGGCCTT

CTCGCTGCGCTGTCCTTTGCGCTGGCGAGCTTCTGGCAGTCGCAGACGCTTGCAACCCCTGAAACGGATCAGG
CGACCGCTCTGTTCGGTACCTTCGCCATCGGCTACACCGGCTATCTTGGTATCTTCGGCATTATCGTCGTCAT
TGCCCTGCTGACGACCTTGACGGCGCGCCTTACCGTCATGCGGACGATCTACGAGATCGACCTTATCCGATCG
GATCCGGGCCGGACCGACACCTTTCAGGGTTGAATGCGTTTCAGGAACCTTGTCCTCACCGCCGCTGTCGCAG
CCGCTCTTGCGGGCTGCACCAACGAGACGCTCGATTCGGTCAACCTGTCGTCCGTCAAGAACAAGACCGAGTA
CCAGCTTTCCAGCAAGATGGTCAGCAAGATGCGCGAGCTGGGGATGCAGAAGACGTCCCCGATCCTGTTGCGC
ATCTTCAAGGAAGAAGGGACGCTCGAAGTCTGGAAGGCGAACACGGCGAACCGGTTCCAGCTTCTGAAGAGCT
ACAAGATCTGCGCCTGGTCGGGAAAACTCGGGCCCAAGATGAAGGAGGGAGACCGGCAGGCGCCGGAGGGATT
CTATCCGCTCTATCCGAACCAGATGAACCCGAATTCGAGCTACTACCTGGCGATCAACACGGGCTATCCGAAC
ACCTACGACAAGGCCAACGGCCGCACCGGCACGCATCTGATGATCCACGGTGCCTGCTCTTCGGCCGGCTGCT
ATTCGATGACGAACGAGCAGATCATCGAGATTTTCGCGCTCGCCCGCGACGCGTTCAAGGGCGGCCAGGAAAG
CGTGCAGCTTCAGGCCTTTCCCTTCCGCATGACGGCGGAAAACATGGCGCGCCACCGCGACAATCCGAACATC
GAATTCTGGAAAATGCTGAAGGCCGGCTACGACCAGTTCGAAGTGACCAAGCGCCCGCCGGCAGTGAATGTCT
GCGAGAGGAAATACGTCTTCAATCAGCAGGCCGCGGGGCAGTTCAATGCGGCCGGCCAGTGCCCTGCCATGAC
GACGCCGCCGGCGCTGCAGATGGCGATGGCCAGCTACGACAAGGACTACCGGCGCGACTACGAGAAGGCATTG
AAGAAATTCGACGGCATGGTCTGGTATGAGCCGACCGAGGCCGAACGCAAGGCGGTCGTCGCCGACCAGCGCA
AGGGCCGCGAGCTTGCCTATGCGCCGACCGGCACCTCGCTCGATGCGGGCAAGCTGCTCAAGGTAAGCGATCT
CGAGAAGAAGCTTGCCGAACAGAAAGAGGCCGAGGAAGCCAAGCAGGCAACCTTGATCCAGAAGGAAGCGCTG
GAAAAGGCGGCCCGCGAAGGCAAGGGCCTGCCGGTGCCGCAGGCCAACCCGCTCGAGCGCCCCGTGGAGCAGG
CAGCGGTGCAAGCCGAGCCCGCGAGGAAGTCGCTCTGGGGTCTGTTCTCTCGCAGCGAGCCCGCGGCCGCGCC
GGCCGCACCCGCCGCCG??????CCGCGCAGCAGGGCGCAGGCCAGCCGGCCGCTCAACAGCCCGCACCGGGC
CAGCCGCAAGCCGCCGCCGGCGGGCAACAGGCAGCACCGCCGGCGCAGGACAATGCTCAGGTTGCCGCCGCTC
CCGCCGGGAATGCGCAAGCGCCGGCACCCGAACAGCAACCCGCCGAGCAGCCGAAGAAGCGCCCGTTCTGGAA
ACTCTGGGGCAATTGAATGACCTTTGCCTTCCATCCGAAGCGCCCCTTTACAGAGGACTTCCGCGCCGTCGGA
GGGGAACAGATCGAACGCGCGATCGCGATGCTGGAGGTCCAACCCGAAGGCGTGCATGAAGCGATCCACGATG
CACGCAAGGGCTTCAAGCGCCTGCGCTCGCTCTATCGCCTCGTGGCCGCGGATGCGCCACTCTTCCAGAGGCA
GGAAAACGCCCGCTTCCGCGCCATCGCACGCAGTCTCTCGACATTCCGCGACGCGGCAGCGCTTGTCGAGAAT
GCCGGGTATCTGCGCCGGCACGCGGCGAGCGAGGAGCAGCAGCTGGCCCTCGACAAGATCTGCTCGATCCTCG
CAAGCCGGCGCGACCGGATTGCCGAAGACGAAGGCGATCTCGACCGGAAGATCGAAGAAACCATCGTCAATTG
CCGAAAGGCGCACGCCGCACTCGCTCATGTTTCCTTCCATGACGGAAGGCGGAAATCCGCCCGCCGCCTGGCG
AAGGGCTGGCGGCAGACATTGCGGCGCGCCGCACGCGCCAGGGCGGCCTGTGCGGCAAGCTCCGATACGACGC
TGTTTCACGACCTGCGCAAGGGCGCACAGGATTACCGGATGCAGCTCTCCCTGCTTCGCGAGGCCTGGCCGTC
GGCAATGCGGGCGAAGAGAACCGAGGCGAGCGAACTGGTCGATGTGCTCGGCCATCTGAACGACATCGCCGCA
TTGATGTCGCTCGTCGACGAACGGCCGGACCTGGCGGGCAACAGCAGGGAACATCTTCTTTCGAATGTCGCGG
CCAGGCAGGACGAGCTGAGGCGTGAGGCCTTGAAGCGCGCGGATGCGGTCTTTCTCGACCGGCCGCGGAGGGA
GAGCCGAACCCTCGAACTGCTCTGGCTCGAAGCCGGAAGGTAGATGCACAAGGTTATTTTCGATACGGATCCG
GGCGTCGACGATGCGATGGCCCTCCTCTTTCTGCACCGTCACCCGGCGATAGAGCTGGTCGGCATCACCACGG
TCTTCGGCAATGCCTCGATCGAGACGACGACGCGCAATGCGTTGTTCCTCAAAGGGGCATGGAATATTGCCGC
ACCGGTCGCCAAGGGCATCGGCGAGACGCTCGATGCCGGCCGGCCGCATGTCGGATGGCCGACAGGCATTCAC
GGCGATGACGGGCTCGGCAATATCGATGTGCCGGCGCAGATAGACCTGCCGCTCGACCCTCGTCCTGCCCACC
GCTTCATCATCGACACGGTGCGCGCCAATCCCGGGCAAGTGACGCTCATCGCCGTCGGCCGGATGACCAACCT
GGCGCGCGCATTGCGCGACGATCCGGAAATCGCCACGCTCGTCAAGGCGGTAGTGATCATGGGGGGCGCCTTC
GATGTTCCCGGCAACATCACGCCGGCGGCGGAAGCGAATATCCACGGCGACCCGGAAGCGGCCGATGTGGTGA
TGACGGCGCCATGGCCGGTGACCGTCATTGGTCTCGACGTGACGACGAAGACGGTGATGAGCCGTGCCATGCT
CTCCGATATCGCCGAACGCGGCGGCGCACCGGCGAGGCTTCTTTCCGACATCTCGCAATTCTACATCGACTTC
TACGAGCACCATGTGGATGACGGCATGATCGTGCACGACAGCTGCGCCTGCGCCTATGTCGTCGCGCCCGAAC
TCTTCCAGACCCGCAGCGGCGCCATCCGAGTGCTTTGCGGCGGCATCGCCGATGGCCAGACCATTCAAAAGCC
GGATGCACGCCTGTTTCCACCGAACGCCTGGGACGGCTTCCCAAGCCAGCAGGCGTGCATCGGAATCGATGCC
GAAGCGGTGCTGAAACTCATCGGCGATACGCTCGCGGACGGCAATTGAGTGATCGGTATCGGAGAAGACGGTG
TAGCCGGTCTCGGCGACGAGGCCAAGCGCCTCATTGAGGCCGCTCCCGTCGTCTTCGGCGGGGCCCGCCATTT
GGAACTCGCTGCGCCGCTGATCGGTGGCGAACGCCATATCTGGCAGAGCCCGTTTGAAAGATCGGTGGAGGCG
ATCGTCGCCCGTCGCGGCAGCCCCGTCGTTGTGCTCGCCTCCGGCGATCCGTTTCTTTACGGCGTCGGCGCCA
CGCTGGCGCGCCGCGTAGATGCCGCGGAAATGCGCACCATCCCCGCGCCGTCCGCCTTCAGCCTCGCGGCTTC
CCGGCTCGGCTGGCCGCTGCAGGAGGTCGCGACCGTTTCGCTGCACGGCCGTCCGCTCGATCTCATAAGGCCG
CATCTTCAGCCGGGCGCGCGCGTCCTGACGCTGACCTCGGACGAGAGCGGGCCGAAGGCGCTTGCCGGGCTGC
TTGCAGAAAGCGGATTCGGTCAGTCACGGCTTACGGTTCTCGAGGCGCTCGGCGGCGCAGGCGAGCGTGTCTC
CCGCCACCTCGCTTCCGGCTTCGCGATGGAGGAAATCAACGCGCTGAACGTCTGCGCGCTTGAGGTGGTGGCC
GATGCCGATGCACGCGTTCTGACGCTTGCGAACGGCCTGGACGAGCGGCTTTTCGAGCACGACGGCCAGATCA

CCAAGCGGGAGGTGCGGGCGCTGACCCTCTCGGCACTCGCGCCGCGCAAGGGCGAACTCCTCTGGGACATCGG
CGCCGGATCAGGCTCGATCGCGATCGAATGGATGCTTGCCGATCCGGCGATGCGCGCCGTCGCCGTAGAGGCG
TCGTCCGAACGGGCTGCGCGGATCGGCCGCAATGCGGTCCGCTTCGGCGTTCCGGGGCTTGTGGTCGTCGAAG
GGCAGGCGCCGGAGGCGCTGCGAGGGCTTCCGCGGCCGGACGTGATCTTCATCGGCGGCGGCGGCAGCGAGCC
CGGCGTGATGGAGGCGGCGATTGCAGCGCTCGCGCCCGGCGGACGTCTCGTGGCCAATGCCGTGACGACGGAG
ATGGAAGCGGTGCTGCTCGCCCGACATGCGCAGTTCGGCGGCTCGCTGATCCGCATCGACATCGCCCGGGCCT
CGCCCGTCGGCGCCATGACGGGCTGGCGGCCGGCCATGCCGGTCACGCAATGGTCGTGGATAAAGGCGTGAAT
GGAGCTGCTTGACGGATTCGATCTCAAGATCCTGAGCGAACTGCAGCGCGACGGTCATCTGACCAACAACGAA
TTGTCCGAGCGCGTCGCGCTGTCTCCTTCGCAATGTTCGCGCCGGCGTACGCGCCTGGAGGCGGAAGGCTTCA
TCACCGGCTACCAGGCGCAGATCGACCGTCAGAAGCTGGGGCTCGATCTGATGGTGGTGATTTCGGTCACGCT
GGCCACGCACAATCGCGACAATGCCAAGCGTTTCGCCAATCTCGTTTCGGCTCTGCCGGAGGTGCTGGAGGCC
TACGCGCTGACTGGTGAAATGGACTACCATCTGCGCGTCGTGACGCCGGATCTTGCCGGGCTCTCCCGCTTCG
TCAACGAGGTGCTCCTGCCGCATGACAGCGTGCAGCACGTCAAGACATCGATCGTACTGGAGACGCTCAAGAG
TTTCGAGGGGCTGCCGATACCGGCGCGGATGAAGGGGTAAATGCTTTCAATCAATCGGCGCGGCGCGCTGGGC
CTCATGGGCGCGACTGCGGGCAGCTTGGTCCTGCCGCGCTTCGCGTTCAGCCAAGGCAAGCGCCCATCCGTCA
CCATCGCCGTGCAGAAGATCACCATCAACAATACGCTCGACGTCTGGAACGAGCAGTCGAATGTCGGTGAGCG
CGTGTTCTTCCCAAACCTCTGGGAGGGTCTGATCCTGCGCGACTGGATGGGCAATCAGGGTCCGGTTCCCGGC
CTTGCGACGGAATGGAAGCGTATCGACGACAAGACCCTCGAGCTCAAGCTGCGCCAGGGTGTGAAGTTCCACA
ATGGCGACGAGCTCACGGCCGACGACGTCGTGTTCAGCTTCTCCGCGCAGCGCGTGTTCGGGGACACCCAGCC
CGCAGGCGGCAAGACGGTCTTCGAGGATGAGCACAAGCCGGCCACCGCCAAGGAACTGCCGGCAGTCGTGCCG
GGCACCGGGCGCCGCCTCTGGCCGGCGCTTGCCGGCGTCGAGGCCGTCGACAAACATACCGTTCGCTTCCACA
ACGCGACGCCGGACGTCACCATCGAGGGCCGCCTTTATGCCTTCGGCAGCCAGATTGCCAACCGTCGCGCCTG
GGACGAGGCGGCGACCTATATGGACTGGGCACGCAAGCCGATCACCACCGGCCCCTATATGGTCGGCGAACAT
AAACCGGACGTTTCCTTGACGCTCGTCGCCTTCGACGACTACTGGGGCGGCCGCCCGCCGCTGGAGCAGATTC
GCTTCGTCGAGGTTCCGGAGGTTTCGTCGCGCGTCAACGGCCTGCTCTCCGGCGAATACGACTTCGCCTGCGA
CCTGCCGCCGGACCAGATCGCCGCGGTTCAGGCCGCGCCCGGATATGAGGTCCAGGGCTCGACGATCCACAAC
CACCGCATTTCCGTCTTCAATGTACAGAACCCGACGCTTCAGGACCCGCTCGTCCGCCGGGCGATGACCCATT
CGATCGACCGCCAGGCGATCGTCGATGCGCTCTGGTCCGGTCAGACGACCGTGCCGGCCGGCCTGCAGTTCCC
CTTCTACGGTGACATGTTCGTCGAAGGCTGGACGGTTCCGGAATATAATCCGCAGCTCGCCAAGGATCTCCTG
AAGCAGGCCAATTACAAGGGCGATGCGATCCCGTTCCGACTTCTCAACAATTACTACACGAACCAGACTGCGA
ACGGCCAGATCATGGTCGAGATGTGGAAGCAGGTCGGCCTCAATGTCGAAATCCAGATGAAGGAAAACTGGGG
TCAGATCCACGACCCGTCCGGCGCGAAGGGCGTGCGCGACTGGTCGGCCGGCGCCGCCTTCAGCGATCCGGTC
TCCTCGATCGTCGCCCAGTTCGGGCCTAACGGCGAGGTTCAGCAGAAGAAGGACTGGGCAAATGCCGAGGCCA
ACCAGATGGCCCAGATCCTCGAAACGGAAACCGATCATGCGAAACGCAAGAAGGCATTCGCCCGCATGCTCGA
GATCTGTGAGCGCGAGGACCCGGTCTACCAGGTGCTTCACCAGAACGCGGTCTTCACCGGCATGAAGTCTTCG
TTGAACTGGAAGGCGGCGCCCGCCTTCGCCATGGACTTCCGCGCCGCCAACTGGGCCAGCTGAATGAATCAGG
TCACCCTTTTCCTTCGATCTGCGCTGCGGTACTTCTTCGCCGCTCTCGGCACTCTCGTGGTCGTGCTCCTCCT
GCTCGTCGGCTTTCTGGGCTTCACGGTGCCCGGCGCCCGCGTTGTCGCCTGGGCGATCGAGAAATATGCGGCG
ACCCCCGACCAGATCGTACGCATCGCCGACCCGGGCGCGCTTCTGACCGGCGACTTCACGGCCGGCACGATTA
CGCTCTTCGATGGCGAAGGCATTTATGCCGAGGTCCGCGACCTTTCGGTCGACTGGTCGCCGGCCGAACTATT
CTCGCTTCGCTTCGACTCGAGCAGGATTTCGGCCGGCTCGGTCCGCGTCGAGCGGCTGCCGGTCCCCTCGACC
GAAACCCGGGAAGTGCGCTCGACGTTTGCCCTTCCGGTTGACGTGAAGATCGATGCCTTCGATCTCAAGGAAA
TCGTGCTCGGCAAACAGATTGCCGGCGAGGATCATTTCCTGACTGCCAGAGGCAAGGTCGACGCGACGAATTC
CAGCATCGCTCTCGTGCTCGATGCCGCCGAACGGGACCGCCCGGATGCGCGCGCGACCGCCGATCTCGTCTTC
AACCCGGCCGGCAACCAGCTGAAGCTTGAAGCCGAGCTCGCGGAGCCGAAGGCCGGCCTTCTGGCGAGACTGC
TCCGCCTGCCGGGAGAACCTGCCGTCAACATGACCGTGACCGGACAGGGACCGCTTTCCGACTGGGCGGGCTC
GGGTACGGTTACGCTCGACGGAAGCGAGATCCTTCGGCTCGACGGGCAGCACGTGCTCGCGGCCGAGGGAATG
CACATGCTTACCGTATCCGGCGGCGGCGCTTTCGCCACGCTGATGCCACCGGCCTTCAGGCCTCTCTTCGAAG
GGGAGAGCAGGATCGACCTGACGGCCGCCTTCGACGGCACGAGCAAGGTCCGGATCGATGCAGGCAAGGTTTC
GACCGGCGCCATGACGCTCGATGTCTCCGGCACGGTCGACAGCCGCGGCGAGAACAACCTCCAGGCAAGGCTC
GCCGGTACGAGCGGGCCTTTCGATTTCCGCTGGCCGCTGAAAGACGGCGAGTTGCAGGCGCTTGTCGACACCG
CAAACCTGTCGCTGATCGGCGACGGGCAGTCGGCAATCCTCGATATCGCCGCCAATGTCCCGTCCGTGAACCT
GCCGCAGGGACGGCTCGGCGGCATCCGGCTGTCGGCACGGAGCGACGCGTTCAACCTGGAGAGCCGGTCGGGT
CCCCTGAAGACGACCGTCGAAGTCGGCGAGAGCCGCTTTGCAAATGCCGAGCTCGATCGGGCGGTGCAGGCGC
CCCTTAAGCTCGACGGCACCATCGCCGTCACCCCGGAAGACATCCGTTTCGACCCGCTGGAGATCGAAAGCGC
AAGCATCGGCGGCACGGTCACCGGCAGCTTCGATCGCACCGAAACGACGCTCGAGGCGGCATTCAAGGTCTTT
GCCGTGCGAGAGGTCCTCCCGCCGGGGCTCGCCGACAAGTTCGACAGAACCATCGCCCTTGCGGGCAACGCAG
TCGTGGAACAGGACGGCAGCGTCCGGCTGAGCGAACTCGACATCAAGTCCGGTGCGGTCGACGCCTCCGGCTC

GGTTGCGCTTGCCGGAAGCACTTTGAGTGCGGACATCCAGGGAACGCTGCCCGAACTCGGACGGCTGCTCGCC
GATGCGGAGGGCTCGGCCGCCTTCCACGCGGTCGCCTCCGGCCCACTGAACAAGCTCGGCATCGAAGCAGAGA
TAACCTCAAGCGGAGCGACGCTCGCCGGTCGCACATTGGACGATCTCGTCGTCAATGCCGATGCCACCGTGAC
GCCAGGGAGCCCCGAAGCGAAACTGACGGCAACCGGCACCCTCGACGGTCAGGCGATCGACGTCCGCGCCGAC
GTCGTCACCCGGGACGGCCGCACCTCGATCCCCACCCTCGAGGCAAAGATCGGCGACAACCGCCTCATCGGTG
CGATGGAGCTTACAGCCGACTTCAAGCCGAACGGGACGATCGACTTCAACCTGCCCGATCTCGGCCTCCTTGC
CGCCATGGCCGGGCAGCAGGCATCCGGCGATCTTGCCGGCTCCGCGACGATCCGGACCGCCGACGGCGTCACG
TCGCTCGCGCTGACGGCCCGCGGCGAAAGCATCAGGCGTGACGCATTGACCGTTGCCAAGCCCGTGGTCGACG
TTACCGTCGCCAATCTTGCCCGGCTGGCGATCAAGGGCGGTGTCCGGGCCGAAAGCGTCGTTCAAGGCGAAAA
CCGCCTGACGGACATCGATATCGCCTTCGAGCAAGAGGCGGAAAGAACGGGTTTCTCGGTCGGTGCCGAATTT
GAAGGCGCTCCGCTTGCGGCGACCGGCGATCTCGCGAGCCGTGACGGCCGGACCGAGATAAGCCTTGCATCGG
CCTCGGCGACAGCCAGGCGCATCCCCCTGCAGCTTGCCCGGCCGAGCGTCATCGCCATCGAAAACGGCGTTGT
GCATATCGATGGGCTGACGATCCAGGCATCGACGGGCACGGTCGCCGTGACCGGTACGGCAGGCGAGACCCTC
GATATTTCGGCGGATATCAGCGACCTGCCCGCCACCTTGATCCATACCGTCGCGCCGACGCTCGGGGCCGAGG
GCAAGATTACAGGCAAGGTGGACATTGCCGGGAATGGCGCGGCACCCGTCGTCCGCTACGATCTCACCTGGAG
CGGCGCCTCGATCGCCTCCGTGCGGTCGGCCGGCATAACGGCAGAGGACCTGTCGGCGAAGGGAACGGTGACC
GTCGCTGAGGACAGCATCCGCCTCGACCCCTTGGCCATCGAACGCGCGGGCTTTCGCGGGGACGTGACGGCCA
GCCTCGACCGCGCCGACAACACGGCCGAGGCGAATTTCCAGTTCGTTGCCGAACCCGCCGTGCTGCCGCCCGG
CATCGGCGCAAAATTCGATGCGCCGATCACCGTCTCCGGCAAGGTCGAGACAGGCGCCGGCGGCACCGTCGAA
CTGAGCGCGCTCGAAGTCAAATCCGATACCGTCAACGCCACCGGTTCGGCCGCACTTGCCGAGGGCGCGTTGA
CGGCCGAGATAGCGGGCGCATTGCCGGATCTCGGCAAGCTGCTCGCTGAAGCCGAAGGCAAGGCCGCCTTCAG
CGCCGACCTGTCCGGGCCGCTCGACGCGCTCGGCGTCAAGGCGGAAGTGACCTCCAGCGGCGCGGTCCTTGCG
GGACGCACGCTTTCGGATTTCGTGATCACCGCCGATGCAACGGCGAAACCCGGCAGTCCGGAGGCGAAGCTGA
CGGCGACGGGGGCTCTGGACGGCCAGGCGATCGACGTGAAGGCCGAGGTGGTCTCGAAGGACGGCCGCACCTC
GATCCCGACCCTGGAGGCGAAGGTCGGCGACAACAGATTGACCGGTGCGATCGACCTGACGGCAGACTTCAAG
CCCGAGGGCACGATCGACTTCAATCTCCCCGATCTCGGCCTTCTTGCCGCCATGGCCGGCGAGAAGGTCTCGG
GAGACCTCTCCGGTTCGGCAGCGATCAGAACGGCGAACGGCGTGACCTCCCTTTCGCTGAAAGCGAACGGCAG
CGGCATCAAGCGCGGCGATCTGACGATCGCAAAGCCGGTCGCCGATATCAGCATCGCCGATGTGGCGGCACTC
GCGGTCAGGGGAAGCGTCAGAGCGGAAAACGTCGCTCAGGGTCAAAACCGTGTCACCGGCCTCAATCTCACCT
TCGATCAGCAGGGCGGCAGGACCGGCTTTACCGTCGACGGCAGATATGACGGCGGTCCGCTTTCGGCAAAAGG
CGACATGGTCGCAAGCGGCGGCCGCACCGAAGTCCGCCTCGCCTCTTTCGCGGCAACGCCCAAGCGGATACCG
CTGAAGCTTGCACGCCCGACCATCGTCGCGATCGAGAACGGCACGGTGCTCCTCGATGCCCTGACCATCGAGG
CTTCCAAGGGCACCATCACGGTCGCCGGCTCTGCCGGACCGACGCTCGACATTTCGGCAAGACTCAATGCGCT
GCCTGCCGCACTGATCAACACCTTCGCCCCGACGCTCGGGGCGGAAGGCACGATCGCCGGGACGGTGGACGTG
GGCGGAACGGCAGCGGCTCCGGTGGTCGTCTACGACCTCAGATGGAGCGGCGCCTCGGTCGCAGCAGCGCGCG
GCGCCGGCGTATCGTCGCTGGAGATTGCTGCAAAGGGGCGCTTCGCCGACAACCGGGTGACCGTCGATGCCAC
GGTCTCCGGGCCGGGAGACCTTTCCTTCCGGGGCGGCGGCAATGTCGAGCTTGGCGGCAACATGCCGATCTCG
ATGAAATTCGCCGGCGACGTACCCTTCGCGCTGCTCGCCAATATCATGGCGGAACAGGGCTTCACCCTGACCG
GATCGGCCAAGGTCGACCTTTCGCTTTCCGGCCCGGCAAAGGCGCCGCAGATTGCCGGCACGGTCTCGACCGC
CGGAGCCCGGCTCGTGGACGTTCGCCGCAATCTGGCCCTCAACGACCTCACCGCCAATGTCGCACTCGACGGC
AGGCAGGCGACGATCTCTCGCCTCTCGGCCAATCTCGCCAGCGGCGGATCGATCGAGGCGAGCGGAACCGTGG
GCATCGTCCCCGGCTCCGGTTTCCCGGCGAATCTCAGAATCCGGCTCAACAATGCGACCTATGTCGACGGCAC
GCTGTTCACCGCCAACCTTGCAGGCGACCTGACGCTCAACGGCCCCCTCGTCTCGACTCCCGTGCTTGGCGGA
AGGCTGACGATCCGCAGGGCCGCGATCACCATACCGGAGAGGCTGCCGGCTTCGCTCTCGCAGATCGACATCA
AACACCGCAACGCCCCACCCCGGGTGCGGCAAATGACCAGAGACCTGCGCCGGGATACGTCCGCCGGCACCGA
GGCAAGCGGCGCGATCGCCTTCGATCTCGCCGTCAGTTCACCGGGCCAGTTCTTCGTGCGCGGACGCGGAATC
GATGCCGAACTCGGCGGCGATCTGACCATCCGCGGCACCGCCGTCCAGCCGATCGTTTCCGGTGGGTTCGACA
TGCGCCGCGGGCGCCTGGAGATCCTCGGCAAGCGGCTGACCTTCACCGACGGTCATATCGGTTTCGGCGGCGA
CCTCATTCCGACGCTCGATCTCGACGCGACCTCGAGCGCCGGTTCGACGACGATCACCGTCAACGTCGCCGGC
CCGGCCAACAACCCGACGGTGACCTTCTCCTCGTCGCCGGCCCTGCCGCAGGACGAGATCCTGGCGCAGCTCA
TCTTCAACCGGTCGCTGAACAATCTTTCCGCCTTCCAGATCGCCCAGCTCGCCTCGGCCGTGGGCCAGCTTGC
CGGCGGCGGGTCGACCTCGCTCCTCGACGGCCTGCGCAACAAGCTCGGCGTCGACGATCTCGACATCACGACC
GACGAGAGCGGCGGCGCCCAGGTCCGCGCCGGCAAATATCTGAACGACCGCACCTATCTCGAACTGCAACAGG
GCTCGGACTCCGCTTCCAGCAAGGCGGTCATCAACCTCGACATCGGCCGCGGCGTGAAGCTGAAGGGCGAAGC
GGCCGGCGACGGTTCGGCCGCCGGAGGCATCTTCTTCGAGAGGGAATATTGAATGGACGTGAGAGAATTGGAC
CTGCCACCCGCCTTCCGCGGCCTGCTTTCGGGCTACGGCTTCGAGAGGGACGCACTGGGCCAGTCGGCATCCA
CCATCTTTCTCCTCAAGGCCCAGAGCCGCCCTTTCCTTATCCTGAAGCATGAACCGTCAGGGCCGTTCACCGA
GTTGACCGGCGAGGCGGCGCGCCTGCACTGGCTCGCAGCCCAGGATATGCCGTGCCCGAGAGTTCTCGGCCAT

GACGATCATGCAGGAGAGACCTGGCTGTTGCTGGAGGCGGCCGAGGGCATGGACCTTGCCTCCTGCCGGCTGC
CGCACGCCGAACGCATCGCCATTCTCGCGGAAGCCCTTCGCCGGCTGCACCGGCTCGATCCGGCGGCTTGCCC
GTTCGACCGACGGCTGACCGCCAGCATTGCTGCGGCAAAAGCGCGGATGGAGGCCGGCCTTGTTGACGAGAGC
GATTTCGACGAGGAGCGGCAAGGCAGGCAAACGAGAGAACTCTTTGATGAGCTTCTCGCGCGCAGACCGAAAG
AGGAGCAATTGGTAGTCACCCATGGCGACGCCTGCCTCCCCAACTTCATCGCAGCCGGAAATCAATTCTCCGG
CTTCATCGACTGTGGCCGTCTGGGCGTCGCCGACATCTACCAGGATCTGGCACTCGCCTGCTGGAGCATCACC
CACAACCTGGGAGAGGAATGGGTCGCCCCGTTTCTGGATCGGTATGGGCTGGCCGCGCCAGACCCTGAAAAGC
TCGCCTTTTACCGGCTGCTGGACGAATTTTTCTGAATGATCTGCGGCCGCAACGTTCTTTTCGCTTCGCTTAT
GCTTTCGGCAGCACCGGCGCTTGCGGACGAGTGCACCCAGGCGCAGGCGGTCTATGCCGATCCGGCCGGAACC
TATGAGCTGCACTTCGAGCCCGTCGGATCGGAGGCGGCAGTCACCAGCAATCACTTCAAGGTCAGGATCGGGG
ACACCGGGGTTTTGCTCGATGGCGTCGTGATGCAGTCGGGCGAACCACTGCGGGCGAACGGGATCGTCATGAA
CGATTGCCCGACCGGCGACGTGACCGGCGCGGAACTCGAGGCCTGCACCGTCTGGCAGGGCCCGATCTATGCG
GTCGACAAGGCCGGCCAGATCGGACTGCTTCAGCCGGAGGACGCGCCCGCAGCCGATCGGATCCTGCTTCCGG
ATTTCGGACCGTCGCTGCGCGAATCGAGCGCCTGGGGCGAAGGCAAGGGTACCACCGACAGTTCCGATGTCTT
CCAGTTCAAGGGGTGTGGATCATGAATGAGCTTGCACACCGGTTTTACTTCCATCGTCGGCTTCGACTCGGCC
TGGACTGACAACCCGAAAGCTCCCGGAGCGATCTGCGTCATTCGTCTGGATGGAGACGGCGCTCATTTGTATC
TCGCACCCCGGCTTGCCTCGTTTGCGGAGGCTCTGGATGTCATCGAGCGCGAGCGCAAGGAGGCCCAGAAGTG
CCTTGTGGCACTGGATCAGCCAACGATCGTCCCGAACCTCACCGGCTCGCGTCCCGTCGACAGGGTAGCCGGA
TCGCTGATCTCCTGGATTGGAGGCGGCGTTCAGCCTGCAAGCCGGTCGAAGAAGGGTATGTTCGATGACGGCG
CACCGATCTGGCGGTTCAAGCAGACCTTGGCAGCGACCGAAGAGCCGGAACTCGCACGAGCAGCATCCAGCGG
CCTCTTCCTGATCGAGGTTTTTCCGGCTCTGGCACTGGCTGCTATCGAGGGAACCTTCTGTTCGAGGCTTGCA
GCCCCGAAGTACAATCCTGCCAACCGGAAGCGCTTCAAGATCAGCGACTGGCAGGCAGTCGCAGAAGCGGTCA
GGCGGTTCGGCATGCTGAACTCGCTGCGGCACCTCGACGAATGGTGCTTGATAGCGGCAAGCTCCGAGGCGCC
GAGGAAGGCGGACCAGGACAAGGTCGACGCATTGATCTGCGGCCTGATCGGGCTGCATTGGCTTGCCGCTCCG
CGTGACCAATCGGTGATGATCGGTGACCTGGAGAGTGGCTATATGATAGCGCCGGCGACGCGCGGCGTGCATG
AGAGGTTGCGCGCAGCGGCGCGACAGCGGGATGTGGCGATTGCGTGAATGGCCGTCGGCCTGTTCGGCGGGTC
CTTCAATCCGCCGCATGACGGGCATGCGCTCGTCGCCGAGACCGCATTGCGCCGGCTCGGGCTCGACCAGCTC
TGGTGGATGGTGACGCCCGGCAATCCGCTCAAGGACCGCAACCATCTCGCGCCGCTTGGCGAACGTATCGCCA
TGAGCGAGAAGATCGCCCGCAACCCGCGCATCAAGGTGACCGCTTTCGAGCAGGCGCTCGGTCAGAGCTACAC
GGCCCGCACGCTGGAAGTGATCCGCGCCCGCAACCGCGATGTCCGTTTCGTCTGGGTCATGGGCGCCGACAAT
CTCAAGAACTTTCACCGCTGGCAGGACTGGCGCAAGATCGTCGCCACTTTCCCGATCGCCGTCGTCGACCGGC
CGGGATCGACCCTTGCCTATCTGTCCTCGCCGATGGCGAGGGCTTTCAGCAGCGCCCGCGTCGACGAGGATGA
TGCGGGAACCCTCGCTTTCCGCCGCGCGCCCGCCTGGACCTTGATTCACGGCCCCCGTTCGGGCTTGAGCTCG
ACGGCGCTTCGTTCGGCCAAATCCGGGTGAATGAGCGCGGATGCTGTCTTCTGCGAGCTCGGTGCGCGCACGA
ATTTCTCCTTTCTCGAAGGGGCGGCGCCGGCCGAGGAGATGGTCGTATTCGCCAAGAAGGCGGGGCTTGCCGG
TCTCGGCATTGCCGACCGCAACAGCGTCGCCGGCGTCGTCAGGGCTCATGCCAAGGCGAAGGTGGAGGGCTAT
CCCTTCCAGCCGGGCGCCCGCCTGGTTTTTGCCGACGGCACGCCGGACATTCTCGCTTATCCGAAGAACAGGC
GGGGCTGGGGGCATCTCTGCCGCCTGCTCAGCGCCGGTAACCTGCGCTCGAAGAAGGGCGACTGCACGCTCCA
TCTCGCCGACCTGCTCGAATGGCAGGAGGAGCTTCTTCTGATCGTCATGCAAGGCGAAGGGCGGCCGGAACCG
GAGAGCCTGGAAGTGCTGCTCGGGACATTGAAGGAGCATGCGGGCAACCGGCTCTATCTCGGACTGGCGCCGC
ATTATGACGGTTTCGACCGGCATGATTTTGCCGTTCTGGCCGCAATTGCCCGAAAGGCCGGCATCGGGTTGCT
CGCCACCAATGACGCGCTCTATCACGATCCGCATTACAGGCCGCTCGCGGATGTCGTCACCTCCATCCGGGAG
CATGTGCCGATCGCCGGCGCCGGTTTCCTTCTGCAGAAGAATGCCGAAAGGCATCTGAAGGGCCCAAGGGAAA
TGGCCAGGCTTTTCAGCGACTATCCCGAGGCGATCGCCAATACCCGGAAATTTTTCCGCGAGCTCGCCTTCAG
CCTCGACGAGCTCAGCCACCAATATCCGGATGAAAACGCCGATGGCGAGACGCCCGCCGAAAGCTTGCGGCGG
CTCGTCGCGGAAGGTGCGGCGGAGCGCTATCCCGAAGGCGTGCCGGAAAAGGTGATGCGCCAGATCGACTATG
AGCTCGAGCTCATCCACGACAAGAAATACGAGCCTTATTTCCTGACCGTCCACAAGCTCGTGAAATTTGCCCG
CAGCGTGAATATTCTCTGCCAGGGGCGGGGCTCGGCGGCCAATTCCTCGGTCTGCTTCTGCCTCGGCATCACC
GATGTCGATCCGCAGAAATTCACCCTGCTGTTCGACCGATTCCTGTCGAAGGATCGCGACGAGCCGCCCGATA
TCGATGTCGACTTCGAGCACGAGCGGCGCGAAGAGGTGATCCAGTATATCTACAGGACTTACGGCAAGGAGCA
TGCGGGCCTGACCGCCGCGGTGATCAGCTATCGCTCGCGCTCCGCCGGGCGCGAGGTCGCCAAGGCCTTCGGC
CTTTCGGAAGACGTTCAATCGGCTCTCGTCAGTTCGATCTGGGGCTGGGGCACCTCGCCTTTCACCGAGGAGC
AGGCGAAAGGGGCGGGGCTCGACGCGGCGGACCCTCTCACCAGGCGCGTGCTTGCCTATGCCAGTCTGCTCAT
GAATTTTCCGCGCCACCTCTCGCAGCATGTGGGCGGCTTCGTGATTACCCGCGACAGGCTCGACGAGGTCGTG
CCGATCATGAACACGGCCATGCCCGACCGCTACATGATCGAATGGGACAAGGACGATCTCGATGAGCTGAAGA
TACTGAAGGTCGACGTGCTGGCGCTCGGCATGCTGACCTGCCTTGCCAAGGGTTTCAAGCTTCTGGAAGCGCA
TTACGGCGAGCCGATAACGCTGGCCGAAATCTACCAGGATCATCGGGACGCGGTTTACGACATGATCTGCCGC
GCCGATACGGTCGGCGTCTTCCAGATCGAAAGCCGGGCGCAGATGAGCATGCTGCCGCGCCTGCAGCCGCGCG

AAATGTACGATCTGGTGATCGAGGTAGCGATCGTTCGCCCCGGCCCCATCCAGGGCAATATGGTGCATCCCTA
TCTGAAACGGCGCGAAGCCCAGCGCAGGGGCGAGGCGGTCGTGTATCCCAGCCCGGAGTTGAAGGCGGTCCTG
GAAAGGACGCTGGGCGTGCCGCTGTTCCAGGAGCAGGCGATGCAGATCGCGATCACAGCGGCCGGTTTTTCGC
CCAGCGAGGCCGACCGCCTGCGCCGCGCCATGGCCACCTTCAAGCGGACCGGTACGATCCACACCTTCGAGCG
GAAGATGGTGGAGGGCATGGTCGCGAATGACTACGAGAGGGAATTCGCCGAGCGCTGCTTCAACCAGATCAAG
GGATTCGGCGAATACGGCTTCCCCGAAAGCCATGCTGCCTCCTTCGCCTCTCTCGTCTATGCCTCGGCCTGGC
TCAAGACCTATTATCCGGACATCTTCTGCGCCGCGCTCCTGAACGCACAGCCGATGGGTTTCTATGCGCCGGC
GCAACTGGTGCGCGATGCCCGTGAACACGGGGTCAGAATGCTGCCGGTCGATATCAATCATTCGGATTGGGAT
GCCCTGCTCGAAGGCGAGGGCGCCTTCGACAAGAATGCTGTCCACCCGCGCCACGCCAGCATGCGGGAGGTGA
TCAAAACCCGGAAGGCCGTGCGGCTGGGCTTCCGGCTGGTCAAGGGCCTGAAGCAGACGGATATGAAGGCGCT
CGTCGCGCGCCGGGGGGAGGGATACCGCTCCGTTCATGACCTGTGGCTTCGATCAGGGCTCTCCCGCTCCGTT
CTGGAGCGTCTTGCGGATGCCGATGCCTTCCGGTCGATAGGCCTCGACCGGCGGGCGGCCCTCTGGGCGGTAA
AGGCGCTCGACGAGCAGTCGGCGGTGGAGCGATTGCCGCTTTTCGAGGGGGCGGGTTCCGACGATCTGCAGAT
CGAGCCCAAGGTCGCTCTTCCCGATATGCCGGCAGGCGAACAGGTCATTCATGACTATCGCACCCTGACGCTC
TCCCTGAAGGCGCACCCCGTTTCCTTCATGCGGGAGGATTTTTCGCGAATGGGTATCCTGCGCAGCCGGGATC
TGGCGGCAACCGCAACTGGAAGATGGGTGACGGTGGCAGGCCTTGTACTGGTCAGGCAGCGGCCGGGCTCTGC
CAATGGCGTCATCTTCATGACGATCGAGGACGAAACAGGGATCGCCAATATCATCGTCTGGGAAAAGACGTTC
CAGAAATACCGGCGGCAGGTCATGGGATCGCGGCTTGTGAAAGTCCGCGGCCGCTTACAGAACCAGAGCGGCG
TCATTCACGTGGTCGCCGATCATCTGGAGGATATAACGCCGATGCTTGGCCTGCTGCGCCGGGAGGCGCGGCG
TTTCGGCGTCAACGACCGCGCGGACGGGGCCTTGCGGCCGAGCGCGGACGCCCGCGAAAAGAAAAAACTCCGG
CAATTGCGCCTGGGCCTGCCCGCTCGCGCAGCGCCGGAGGGCGAGGCGGCCGCCCAGGTCGCGGAGGTCATGC
CGAAGGGGCGCAATTTTCATTGAATGTTTGTAAGAAAGATCGCAACTGCACTCGCACTCGTAATGGCTGCATC
CGGCGTGGCTTCCGCTCAATCTCCGACGCGAATCCAGCAGTTCAACGCCTGGGGTGCATATTCCTACCAGGCC
GGTAACGGCAAGGTCTGCTATGTGCTTTCCGTGCCCAAGGAAAGGACCCCGGCAAGTGTCGATCACGGCGATA
TCTTCTTCCTCGTTTCGCAGCGTCCGGGACAGAACATCAGCTATGAGCCACAGGCGATGATGGGGTATCCGCT
GCAGGACAATTCCAAGGTCAACGTGGTGATCGACGGCCGCACCTTCGTCATGTTCACCAAGGGCAATTCCGCC
TGGGTGGAAAATGCCGCCGAAGAGCCGGCACTGGTCGCTGCGATGAAGTCGGGCAAGGCCATGTCGGTCAAGG
CGAAATCGCGGCGCGGCACGGAAACCTCCTACTCGTATTCGCTCTCCGGCATTTCGGCCGCCCTGAAGCAGAT
CGAGGCCTGCAAGTAAATGACGGTGGTGGTGAGGCCGAAGGCGCTCGGCGACTACGATACCCGCGAATTCCGG
ATGCTGATCGACGGTCAGTGGGTGGATGGCGCCGAGGGCCGCACGATCGAGCGGGTGGCGCCGGGTCACGGCG
TCGTCGTCAGCCGCTATCAGGCGGCGGCGAAAGCGGATGCCGAACGGGCGATCGCCGCCGCACGGCGCGCTTT
CGACGAAGGTCCATGGCCGCGCATGACCGCCTCGGAACGGTCGTTGATCCTGCTCAGGGCCGCCGACATGATT
GCCGCGCGAGCGGATGAGCTCGCTTTTCTCGACGCGGTCGAATCCGGCAAGCCGATCAGCCAGGCGAAGGGCG
AATTGGCGGGTGCCGCCGACATCTGGCGCTATGCGGCAGCACTTGCCCGCGAACTCTCGGGCGAAAGCTACAA
TACGCTCGGGGAGGGTACGCTCGGCGTCGTGCTGCGCGAGCCGATCGGCGTCGTATCGATCATCACGCCGTGG
AACTTCCCTTTCCTGATCGTCAGCCAGAAGCTTCCCTTCGCCCTTGCCGCCGGCTGCACGACGGTGGTGAAGC
CCTCTGAACTCACCTCGGCCTCGACGCTGGTTCTCGGCGAAATCCTGGAAGCGGCCGGCGTGCCCCAGGGGGT
CGTGAACATCATCGTCGGCACGGGGCCGGAGGCCGGCGCGCCGCTCACCACGCACCCGCATGTCGACATGGTC
TCCTTTACCGGCTCGACCGGAATCGGCCAGCTGACCATGGCGAATGCGGCGCAGACCCTGAAGAAGGTCTCGC
TGGAACTCGGCGGCAAGAACCCGCAGATCGTCTTTCCGGACGCAAACCTGGACGAATTCATCGATGCGGCCGT
CTTCGGCGCCTATTTCAATGCCGGCGAATGCTGCAATGCCGGCTCGCGGCTGATCCTGCACCGCGATATCGCC
GAGGAGGTGACGGCGCGCATCGCCAGCCTCTCGGCCAAGGTCAAGGTCGGCGATCCGCTCGACCCCGAAACGC
AGGTCGGCGCGATCATTACGCCGCAGCATCTGCAAAAGATCGCCGGCTATGTTTCTTCGGCGTCGAACGAAGG
CGCGAGAATAGCCCATGGCGGCACCACGCTCGATCTCGGCATGGGACAGTTCATGGCGCCGACCATCCTGTCG
GCCGTTCGGCCCGAAATGGCCGTGGCCCGGGAGGAAGTCTTCGGGCCGGTTCTCTCCGTTCTGACTTTCGAGA
AGACGGAAGAGGCGATCCGCATCGCCAATTCGATCGATTACGGGCTTTCCGCCGGAGTCTGGAGCCGCGACTT
CGATACCTGCCTGACGATCGGGCGGCGCGTACGCGCCGGCACGGTCTGGATGAACACCTTCATGGATGGCGCA
TCCGAGCTGCCTTTCGGCGGCTACCGGCAATCCGGGCTCGGCCGCGAGCTCGGTCGCCATGCTGTCGAGGACT
ATACGGAGACGAAGACGCTCAACATGCATATCGGCCGGCGCAGCAACTGGTGGATGCCGCGCTGAATGACCAT
CTTACCGAAACAGACCTTGCGAGTCGGCTTCGTCGGCACCGGCTTCATCGCGCATTTCCACCTGAAATCGCTC
ATCGGGGTACGCAATGTCGAGGTGACCGGCGTCTACAGCCGCAATCCCGAAAACCGCGCGCGCTTCGTGAGCG
AAGTCGAGGCGCTCGATCTCGGCGAGTGCCGCGCGCATGAGAGTCTCGAATCGCTCCTGTCCGCCGGCGACGT
CGATGCGGTCTGGATTCTGTCGCCGAACTACACCCGGCTCGACGTCATGCGGACGCTCCACCGGGAGATCAAG
GCGGGAAGAAGCAAGATCTTCGCGGTCGCCTGCGAGAAGCCGCTTGCGCGCACTGTGGCGGAGGCACGCGAGA
TGCTGCACCTGGCCGAGGATGCCGGTCTCAACCATGGCTATCTCGAAAACCAGGTCTTCTGCACGCCGGTGCT
GCGAGGCAAGGAGATCATCTGGCGGCGCGCCGCCTCGACCACCGGCCGGCCCTATCTCGCACGCGCCGCGGAA
GAACATGCCGGTCCGCACGAGCCCTGGTTCTGGCAGGGCGACAAGCAGGGCGGCGGGGTGCTTTCCGATATGA
TGTGCCACAGCGTCGAGGTGGCGCGCCACCTGCTCACGGCGCCCGGCGCGCCTCGCAATTCGCTGAAGGTCAA

GTCGGTGAACGGCACGGTGGCGAACCTCAAATGGACGCGGGCGGGCTATGCCGATGAGCTGCGCCGCCGCTTC
GGTGAGGATGTCGACTACCGCCGGCGCCCATCGGAGGATTTCGCCCGCGCCACCGTGGCGCTGGAGGACGAGG
AGGGCAACGAACTGATGATCGAGGCAACGACCTCCTGGGCCTATGTCGGCGCCGGCCTGCGCATCCAGCTCGA
GCTTCTCGGGCCCGAATATGCGCTGGAGTACAATTCGCTCGGCACCGGCTTGAAGATATTCCTGTCGCGGGCC
GTGCAGGGCACCGAGGGCGAGGACCTCGTCGAAAAACAGAATGCCGAGCAGGGCCTGATGCCGGTGCTTGAGG
ACGAGGCCGGCGTCTACGGCTATACGGACGAAAACCGCCACATGGTCGAATGCTTCCGCAAGGGGATAAAGCC
GCTCGAAACCTTCGAGGACGGCCTGGCGGTCGTCGAAATCCTGATGGGCCTCTACCGCTCCGCCGAGATCGGC
GCGACGCTGCATTTCCCCGCGCCGGAGCTGGAAGACTATGTGCCCGTCGTGGCGCGGACGGGGGCGTAGATGA
GGACGCGCCGCGAAATCCGCGCCCGCAAGGCGCTCCGGGACAGGATCGTCTATCACGGCATCGGCATCGGCAC
GGCCTTCTTCTTCCTGGCGCCCTTTGCCGTTTCCTTTCTTGCCTCCTTCCGGCCGAACAGCGAATCCGGCAGG
CCGCCGCTGCCGCCATGGCCGATCTCGGGGCTGAGTTTCGACGCCTACCGGGCGCTCGACAGCTTCGGTGCCG
GCATCTGGCAGCACATGTTCAATTCGCTCCTCGTGTCGATCGGAACGGTCGTCCTGACCGTCGCGGTCAGCGT
GCTCGCCGGCTACGGCTTCTCCCGCTACCGCTTCCCGTTCAAGAACGCGCTCTTCGTACTGATCATCGCGACG
CTGATGGTCCCGTTCCAATCGATCCTGACGCCGCTCTTCATCATTCTGGCGCGGCTCGGGCTCAACAATTCGC
TCGTCGGGCTGATGCTCGTCTATGTGACGCTGCAGCTTCCCTTTTCCGTCTTCATGATGCGCAACGCCTTCGA
TGCCGTGCCGAAGGAGATCGAGGAAGCGGCGCGCATCGACGGCGCCCGCGACCTGAAACTTCTCGTCCGGGTG
CTGTTGCCGCTGGTCATGCCCGGGATTGCGACCGTGTCGATCTTCGCCTTCCTCAATGCCTGGAACGAATTCT
TCGCCGCGCTCGTCCTCCTGTCCAGCAACGACAATTACACGCTTCCCGTTCTGATGACCGCCGTGCGCGCCGG
CCGTCTCGGCGCGATCAACTGGGGCGCGGTGCAGGCCGGCGTCGCGGTGATGGTCGTTCCCTGCCTCTTCGTG
TTCCTTCTTCTGCAACGCTATTACATGCGCGGGCTGATGGCCGGCGCGGTGAAATGAATGAGCAGTGAACAGA
GCAAACCCGCCGCGGCCCCCGTACTTCCGGGCACGTGGGGCATCAGGGAGACCGCGATCAAGTACGGCTTCCT
CATGCTGCTCGCCGGCATGGTTCTTTATTTCTCGCTCGTAACCGGAGGTTTTGCCTCCCCTCAAAGCGCAGTC
TTCATTCTGCAATCCGTGTCGATCACCGGCATTCTCGCCCTCGGCGTGACGGCGACGCTTGTCGTGGGGGGGT
TCGACCTCTCGATTGGCTCGGTCGCCACCACGGCGATGATGGCTTCCTCCTATGTTATGGTGGTGATGGGAGG
CGACGCGCTTACGGCAGCGCTCGTATGCCTCGCCGTCGGCGCGCTCGTCGGCCTGATCAACGGCTTCATCATC
GTCTACATGCGCGTTCCGGACCTTCTGGCTACCCTCGGCATGATGTTCCTGCTGCTCGGCCTTCAGCGCATCC
CGACCGAGGGCCGCTCGATTGCCGCGGGCATGACGCTGCCCGACGGTTCGGTCGCGACCGGCACTTTCAGTCC
GGCTTTCCTGGCGCTCGGGCGCCACCGCTTCGATCTCGTCCTGCCCAACCTCGTGCCGGTCTCGGTCGTGGTG
CTCGTGGTGCTCGCCATAGCGATCTGGTTCTTCCTCGAATACACCCGCTTCGGCCGCATAATGTATGCGGTCG
GGTCCAACGAGCGCGCGGCCGCGCTCGCAGGGGCGCCGGTCAATGCCTACAAGATTTCGGCCTACGTCATTTC
CGGCGTCTTCGCCTCTATCGGCGGCATTCTGCTTGCAGCACGCCTCGGCCGCGGCGATATCGCCTCCGGAAAC
AACCTGCTGCTCGACGCCGTCGCCGCCGCGCTGATCGGTTTTGCCGTGCTGGGTGCGGCCAAGCCGAACGCTT
TCGGAACGGCCGTCGGCGCGCTTTTCGTCGGGATCCTACTGCAGGGCCTGACGATGATGAATGCGCCTTACTA
CACGCAGGATTTCATCAAGGGCGCCGTGCTCGTCGTCGCCCTGATCTTTACCTTTGCGCTCTCGAAAAGAGGC
AAACGCTGAATGAAAGAGTTCAACGGCCGACGTTTGGAAATCGATGCGCTGCGCGCCTTGTGGGCGATCCGGC
GTCACGGCGGGATAACCCGCGCGGCCGAGGCCCTGGGCCTGTCGCAGTCTGCCGTCAGCCACAAGATCAAACG
GCTCGAGACGAGCCTCGATTGTGACCTGCTCGGCCGGAGCCCGGGCGGAGCGATGTTTACCGCAGCCGGAGAG
GATTTGCTGGATTATGCCGGGAAAATCCTCGGCTTGCATGACGAAGCGCTTCTGAGCTTATCGAAAGCGTCAC
TTGAAGGACGCATCGCGCTCGGTTTGACCGAAGACACGGCGTGCAGCGACCTCGCGCGGATATTGGGGAGATT
TCGCCGACTGCATCCGAATGTCGCCGTCCGCACCAAAGTCCGCATGAGTCTAGTCCTGCGAGCGATGCTGGAG
CGGGGGGAGCTAGACGCCGCGATCCTGCAGGTTTTCGCTCATGAAGTCCGGCCGACCGACGTCGTTCTGTTCC
GGGAGGAGCTCCATTGGGTCAAGCACCCGCAGCTGTCACTGCCGCAGCAGGGCCCGATTCCCTTCCTGTCCTT
CGACGACGAATGTTTCTATCGAAGATGGGCGCTCGACATCGGACAGGACGGCGGAGTGGTTCTTGAAACCGTA
TTCGAATGTGCAAGCGCTGCTGGTATCGTTGCCGCGGTCACTTCGGCCATGGGTGTAGCACTTCTGAGCGGGC
GTCATCTGCGCCCCGAAATGCAGATCGTCACCGAACGCTTGCCCACGCCGCCTGCTTTGGCATATGTCGCCCG
CCGCGCCCGAAAGGTGAGAAATTCCGCGCTAGATACGCTGGTCGCTGAAATCGAAAACGAAATCAGTCGCTAC
GGAGGGCTGGTTCTCGCGAGCTAAATGACGATGGAGGCGGATGAAATTCGCGTATCCGAGAGTGTGCCGGTCG
GTGCAGGGCACCGCCGCGGTACGCGCGCCATGGCGCCCGGCGACATTCCGGCGGTCGGACGGCTGTTCAACAG
GATTTTCCGAAGGCAGGACCACGCGCCCGGGAATGACCTTGGACGTTATCTCGAGACCGTCTTCTTCGGCAGC
CCTCACTATTCTCCCGAACTCGGCAGCGTCGTGCACGAAAACGGTGCGACCGGCATAGACAGCGCAATACTCG
CGGTACCGATGGCGTTCATGGTCAATGGCCGCCCGACCATGGCCCGTCTTCTCTGTGCCTTCATGGCGGATGG
AAAAGCAGGAGCCGTTGGAGCGGCGCGTCTTGCGCGCACCATGCGGGCGTCTCGCCAGGACATGTGCTTCTCC
GACAATGCGTCGCCGGTCAGCGCCGACCATTGGGTTGCGGGAGGTGGCATCGTCCTGCCGATCCAGAGCCTCG
AATGGCAGCGTTCGTTTCTGCCCTTGAGCGCCGCGGCTTTCCTTGCGGGCAGTCACTTCCCGATAGCGCGCTC
GCGCGTTGTCGTCGGCGTTCTCAGGCTGATTGACCGTGCACTGCGCCGGTTGCGGCCCTCCATGCGGCCGAAG
GCGGAGGGCGAATGCAGGATCGAGGCAGCGGGCGTCGAGGAGTTCCTCCAATGCGCGAGCCCGATGCTGGAAC
GCTTTGCGATCCGCCCGGCCTGGTCGCGAGCCGAATTCGATTGGCTCGTGCGCGTCGCTTCGATGAACCGGAG
CCTCGGGACGCTCCAATGCAGGAAAGTCGTTCTCGCGGACGGCCGCACGATCGGCGCCTTTCTTTTTTTCGGC

AAGCAAGGTGCCGTGGCCACGGTGCTCAATCTCGTCTGCGAAGAGGGGCGCGAACGCGACGTCGCCGCTGCGA
TGTTCGCGCGGCTCGATGCGGAAGGCTATGCCGTGGCGGTCGGCATGGCACAGCCGTTCCTGATGAACGCTAT
CTCGCGCCAGCGATGGCTGTCATTCCGGCACCGCGGCTACTTCTGCATGGTGACCCGCCATGCGGACCTCAAG
GAAGCGGCAGAGCGCAACGACATCTATATCGGCGGCCTCGCCTCCGAGACCTGGAGCCGGCTGCTGACGGATT
TTTAGATGTTCTCCGATCCAGATTTTCTGAAGCTCATCTTCGGCCGGCTTACCCTCGACGCGATCCCTTATCA
CGAGCCGATCCTCGTCGTGACCTTTCTCGCCGTCGCGCTCGGCGGCATCGCGCTGATCGGCCTCATCACTTAT
TACCGCTTCTGGGGGACGCTCTGGCGCGACTGGCTGACGAGCGTCGACCACAAGAAGATCGGCATCATGTACA
TCATTCTCGCGCTGGTGATGCTGCTGCGCGGATTTTCCGACGCGATCATGATGCGGCTGCAGCAGGCGATGGC
CTTCGGCGGCTCGGAAGGTTACCTGCCGCCGCATCACTACGACCAGGTCTTCACGGCACACGGCGTGATCATG
ATCTTCTTCATGGCGATGCCCTTCGTGACGGGGCTGATGAACTTCGTCGTGCCGCTGCAGATCGGTGCGCGCG
ACGTCTCCTTCCCCTTCCTCAACAATTTCAGCTTCTGGATGACGACGGCCGGCGCGATCATCATCATGCTGTC
GCTCTTCGTCGGCGAGTTCGCGCGGACCGGATGGCTTGCCTATCCGCCGCTGTCGGGCGCGGACTACAGCCCC
GGCGTCGGGGTCGACTATTACATCTGGGGCCTGCAGATCGCCGGCATCGGAACGACGCTGTCGGGGATAAACC
TCATCGCCACCATCGTGAAGATGCGCGCCCCCGGCATGACGATGATGAAGATGCCGGTCTTCACCTGGACGTC
GCTCTGCACCAATGTGCTGATCGTCGCCACCTTCCCGGTGCTGACCGCGACGCTGGCGCTGCTTACGCTCGAC
CGTTATGCCGGCACGAACTTCTTCACCAACGACCTCGGCGGCAATCCGATGATGTATGTCAACCTCATCTGGA
TCTGGGGCCATCCGGAGGTCTACATCCTCATCCTGCCGGCCTTCGGCATTTTCTCGGAAGTCGTCGCGACCTT
CTGCGGCAAGCGGCTCTTCGGCTATGCCTCGATGGTCTATGCCACGGTCGTCATCACCATCCTCTCCTACATC
GTCTGGCTGCACCACTTCTTCACCATGGGCTCGGGTGCGAGCGTCAACGCCTTCTTCGGCATCACGACGATGA
TCATCTCGATCCCGACCGGAGCGAAGATGTTCAACTGGCTCTTCACCATGTATCGCGGCCGCATCCGCTACGA
GGTGCCTATGCTGTGGACCATCGGTTTCATGGTGACCTTCGTCCTCGGCGGCATGACCGGCGTCCTGCTTGCC
GTTCCGCCGGCCGATTTCGTCCTGCACAATTCGCTGTTCCTCATCGCCCACTTCCACAATGTCATCATCGGCG
GCGTCGTCTTCGGGCTGATGGCAGGCGTCGTCTACTGGTTCCCGAAGGCCTTCGGATTCAGGCTCGATCCGTT
CTGGGGCAAGATGTCCTTCTGGTTCTGGCTGACCGGCTTCTACTTCGCCTTCATGCCGCTCTACGTGCTCGGG
CTCATGGGGGTCACGCGCCGCGTCAGCCAGTTCGAGGATCCCTCGCTGCAGATCTGGTTCGTCATCGCGGCCT
TCGGCGCCTTCCTGATCGCGCTCGGCATCCTGTCGATGCTGATCCAGCTCGTCGTCAGCTTCGTGAGGCGCGA
AGAGCTGCGCGACACGACCGGCGACCCCTGGGACGGGCGCACGCTCGAATGGTCGACCGCCTCGCCGCCACCG
GATTACAACTTCGCCTTCACCCCGCTCGTCCACGATCAGGACTCCTGGTGGGATATGAAAAAGCGCGGCCACA
GCCGGCCGCTTGTAGGCTTTAAGCCGATCCACATGCCGAAGAACACCGGCACAGGCGTCATCCTCGGAGCGAT
CAGCGTCGTCTTCGCCTTCGCCATGGTCTGGTACATGTGGTGGCTGGTGATCCTCTCCTTCGTCGCCATGGCG
GCGATCGCCATCGGCCACACCTTCAATTATGACCGCGACTTCCACATTCCCGCAAACGTCGTCGCCGACACAG
AGGGCAAGCGGACCGAAGCGCTTGCCGCCGAGGTGCAAGCATGAATGGCTCAAAGAGCAGGCAACGCGGATCT
CCCGCTGCATGGCGGCCGCGTGCCGCGATGGCTCGGCGACCGTATGACGCGCCTCGGCGCGTTGGTCACCGAA
GCGGTCGTTCATCACTACGGACGCGACGAGTTTTTAAGGCGGCTTGCGCATCCGTTCTGGTTCCAGTCCTTCG
GTGCCGTCATGGGCATGGACTGGCATTCCTCCGGTATTACCACGAGCGTCATCGGTGCATTGAAACGCGGTCT
GACCCCGCTTGCCGGCGAGCTGGGCATCCATGTCTGCGGCGGCCGCGGCCAGCACTCCCGCAAGACGCCGGGC
GAACTCATATCGATTGGCGATCGGGTCGGTTTCGATGGCGGCGCATTGGCAGAAGCGAGCCGCCTCGTCGCCA
AGGTCGACAGTGCCGCCGTTCAGGACGGCTTCGATCTCTACCTGCACGGTTTCATCGTCACGGACGATGCCAG
GTGGGTGGTCGTCCAGCAGGGCATGAACGGCGACCGCCGGCAGGCGAGGCGCTATCACTGGCTCTCGGAGGGC
CTGACAAGCTTCCTCGACGCGCCGCATAGCGCGATCGAAGGCAGAGAACAGGGCGAAATCGTCAATCTTGCCG
ACCGCCGCGCCGCCGCCTCGCGGACTGCGCAGCTCGATCTCCTCCAGTCCCTCGGGCCCGACGAGCTCCTGCG
CGAGGTCGCCCGGATCGAGTCTCGTGGGGCTCCTGTGGCCGCGCCGGCACAGCCGATGCTGCCGCATCTCTTC
ATGCCCGCCCATCACGACGTGCGCGAAAGCGACGTCAATCTCCGGCGCCTTCACGGCAACTTCGCCGCCGCCG
CCGACCGTGGTCCGGAGGATTTCAAGGACCTGCTGCTGGTGCCAGGCGTCGGCGCGCGGACGGTCAAGGCGCT
GGCGATGGTCGCGGAGGTGGTCCACGGGACGCCGTGCAGGTTCTCGGACCCCGCCCGCTTTTCGCTCGCCCAT
GGCGGCAAGGACCGCCATCCGTTCCCGGTGCCGTTGAAAGTCTATGACGAGACGATCAGGATCATGAAATCAG
CGGTCAGCAAGGGCCGCCTCGGGCGCGAGGAAGAGCTTGCGGCGCTGAAGCGGCTCGACGAGCAGTCGCGCCG
GCTGGAGCGCTACGTCACGGGCCCCGACCTCAAGGAGATCGTCGCGGGCGAATTCAGGGACTCCGCGCGTTTC
GGCGGGCGCAGCATATTCGGCTTGGAACCGCACGACGACGAGGCAACCGCGGCGGAGCCAAGCAACCGCACGC
GGCGCTGAATGACGACGAGCGAGATCAAGAACGTGCAGGGAATAGCAGCGATCGAGGCCCTGCTTGCCGCCGA
ATACGGCATCGCGGGAGAAATCCTGGCGCTGCCCGGCGAGCACGATCTCAATTTCCGCATTCAGGCGAGCGAC
GGTCGCGCGTTCCTGCTGAAGCTGCATGCCCTCGGCGCGCCCGAGGAGCTCGACATGCAGATCGCGGTTCTCG
ACCATCTCGCGCGCGAGGCGACGGAGCTGCCTGTCGCGAAGGTCCTGCCGAGCCGCTCCGGCGCCTCGTTCAC
CCGCGTCGAATTCGAAGGAGAGCGCGTCGCACGTCTTCTGACGTGGCTGCCCGGGGAAACCTGGGCACGAGCC
GCCGACCGCTCGAGCAACAGCGTCGAGACCCTTGGGGCGCTGCTCGGCAAGCTCGACCGCAGCCTCGCCGGTT
TCTCTCATCCCGGGACCAGGAGAGAGTACGCCTGGGATATAGCCCGGGCGGAGATGCACCTCGCCAATGTCGA
TCTGATCGAGGGTATGGAGAAGCGGCGAGCGGTTCGCGCGATCCTCGAGCACTTCGTCTCCACGGTGCTGCCG
CGCCTTAAGGCTTGCCCCCGGCAGGTCATCCATAATGACGCCAATGACTATAACGTGCTCGTCGGCGCCGACG

GCTGCGTCAGCGGACTGCTCGACTTCGGCGATATGGTCGAGAGCAATCGCGTGGTGGAAGTGGCGGTCGCTTC
CGCCTATGCGCTGATCGGTTCGCCGGATCCGATCGGCGCCATCGCGCGGCTGGCTGGCGGCTATCACGGCGTC
AATCCGCTCGGCGAAACGGAGGCCGAGCTGATCTTCGATCTCGTGCGCACCCGCTATGCCGTCAGCATGTGCA
TGGCGGCGAGGCAGATCCGCGACAACCCCGAAAACACCTATCTGCTCGTCAGCCAGGAGGACGTCTGGCGGGA
ACTGAGACGCCTCGAGCAAGAGAACCGGCCCTTTGCCATCGCCCGCCTTCGCGACGCCTGCGGTTTTGCACCG
ATTCCGAAGGCCGCGCGGGTGGTGCGGTGGCTGGAGAGCAATGCTCATGATTTTTCGGGCGTCATCAAGCCGG
GCATCGCAAGACCGAAACCAGCCGTCTTCGATTTTTCCGCGAACAGCTCCGAGACCTGGGCAGGTCTCGACAA
GGACGTGGCGCAAGCGCGCATCGAAGCACATATTCATGCGGAGGGGGCGGATTTCGGCCTTGGCCTCTACGGA
GAGGACAGAGCGGTCTACAAGGGTGACGCTTACCAGGCGACTGCAAGCCTGCGCCGCACGATTCACCTTGGCA
TCGATCTCTTCGCTCCCGCCAACGAGCCGGTGCATGCACCCTTCGCCGGCAAGGTCGCATTTTACCATGACGA
CGCGGTCCCTTACGGTTTCGGACCGACCATTCTGCTCGAACATAGGACGGGCGAGGGCGACGCGTTCTGGACG
CTCTACGGACATCTCTCCCGTGAAAGCGCCTCACGGCTGTCGATCGGCCAGCCGGTCGCCAGGGGCGAAGCTT
TCGCGGCCATGGGGAATCGCGCCGAAAACGGCGGGTGGGTTCCGCATCTGCATTTCCAGATCGTCACCGATCA
TCTCGGCCTCGAGGGTCGCATGCACGGCGTCGGCGTCAAAGAGCAGTGGCAGGTCTGGCGAGAGATCAGCCCC
GATCCGAGCGTCGTGCTCGGCCTTTCCGTTCCGGCGTCGGTGATCGTCGAGCGCGGCAAGGTGTTCCTGGTGC
GCGAACGCCAGCGCCGCATCGGTCGCTCCCTAAGCATCGCCTACGGCTCGGCGCCTCTCAAGATCGTTGCCGG
CGAGGGCGCCTACCTGATCGATGACGAGGGCACGCGCTGGCTCGACATGGTCAACAATGTCTGCCACGTCGGC
CATTGCCATCCGCGCGTCGTCAAGGCGGCGCAGATGCAGATGGCGCGGCTCAATACCAATTCGCGCTATCTGC
ATGACAGTCTGGTGGAATATTCGCGCCGGCTTGCGGCGCTCTTCCCTGATCCGCTGAACGTCTGCTTCTTCGT
CAATTCCGGCAGTGAGGCCAACGATCTCGCGATCCGGCTGGCGCGCGCCTATACCGGCAACCGCGACGTCATT
ACCGTCGATCACGCCTATCACGGCCATCTGACAAGCCTGATCGATGTCAGTCCCTATAAATTCGCGGGCAAGG
GCGGGGAAGGGCGGCCGGCGCATGTGCGGGTCGCCGAAATGCCGGACCTCTATCGTGGACGCTATCGCTATGG
CGATACAGACGCGGGGCGCAAATATGCCGAGGACGTGAAGCGTCAGATCGATGCTCTGGCGGCGGAAGGGCGC
AAACCCGCGCTCTTCTTCAGTGAGGGCATCCTCGGCACGGGCGGTCAACTGGTGCTCCCGGAAGGCTATCTCA
GAGGGGCCTATGCGCATGTGCGTGCGGCCGGTGGGTTGTGCCTCGCTGACGAGGTCCAGGTGGGGTTCGGCCG
TGTCGGCAGTCATATGTGGGCGCATGAAACGCAAGGAGTCGTGCCCGACATAGTCACCATGGGCAAGCCGATC
GGCAACGGCCACCCGATGGCGGCGGTGGTGACCACCGAGGCGATTGCCGCGGCCTTTGCAAACGGAATGGAAT
ATTTCAACACGTTCGGCGGAAACCCGGTATCGGCCGAGATCGGCCTCGCCGTGCTCGACATCATCCGCGATGA
GCGCCTCATGCACCATTGTGCGGTCGTCGGAAACCGTCTGATGGATGGAGCGCGCGAACTCGCCAGCCGCCAT
ACGATCATCGGCGACGTGCGCGGTTACGGGCTCTTCAACGGAATCGAGCTCGTGCGTGATCGCGACACCCTTG
AGCCGGCCGCAGCGGAGCTCGACTTCGTCATTGCCGAAATGAAGGACAGGCACCGCATCCTGCTGTCGAGCGA
TGGGCCGCAGCACAACGTGCTGAAAATCAAGCCGCCTGCGCCCTTCAGCGCAGACGATTGCGACCGTTTCCTC
GATGCCCTCGACGCGGTGCTGGCGCAGGTAAATGCCGCCTGAATGGAACACCAACTGGCCAGACAAATCGCAT
CGCGGAAGCCCTTCGATCCGACCTATGATCCGATCCGCGCGCCCAATCCGGGCGACGGCAAGGACTATGCGCC
GACCTACTGGATCGGCACGGCCGGCACTCCGCCTGCCGACGATGGGCCCGTTAGCGGCGATATGGACGTCGAC
GTCGCTATCGTCGGCTCCGGATATACGGGGCTCTCCTGCGCGATCCATCTGGCCCAGGAGCATGGCATCAAGG
CGACGGTGCTCGAAGCCAATGGCGTCGCCTGGGGCTGCAGCACCCGCAATGGCGGTCAGGCGCAGATTTCCGC
CGGCCGGCTCAAGCGTTCGCAATGGATCGAGCGCTGGGGCGTCGATGTGGCCAAAAGGCTGCATGGCGAAATC
AGCGAAGCGTTCGATCTGTTCCGGGACCTGATCCGCTCGCCCGAGATCGACTGCGACCCTCAGGACGGCGGCC
ATCTTTATATCGCCCATCGGGACAAGGTGATGCCGGCGCTCGAAGCCGAGTCTCGGCTTCTCAACGAGGTTTT
CGGCTACCGGTCGCGTATCGTCGGTCGCGATGAGGTCCACCGCGACTTCGTACGCGACGAGGAGGCGAGGGGG
GCGATGTACGAACCGGACGGCATGGGCATCCACGCGGCCAAGCTCGCCTTCGGCTACCTCAATCTGGCACGCA
AGCTCGGTGCGCGGGTCCACACGGCCAGCCCCGTTCTCGGCTGCGACTGGAAGAACGGCGCCTATCATCTGAC
TACGCCGGGCGGCACCGTTCGTGCGCGCGCCGTCTGCATTGCGACGGCGGGCTATACCTCGCCGGACCTGCAT
ACGCTGACGCGGCACCGGCTGATGCCGATCCTCTCGAACTCGATCGTCACGCGCCCGCTGACCGAGGAAGAAC
GCGCCGAACTGAACTTCAAGACGCGGATCCCGCTCACCGATACGCGCACGCTGCGCCACTACTACCGGATGAT
GCCGGACGGCCGCGTGCAGATCGGCAGCCGCAGCGCCATCACCGGCCGCGATGCGGTCAACCCCAAACATCTC
GATCGGCTGCTCGAAGGGCTCTACCGAAAGTTTCCGATCCTGAAGGGGATCACGATCGACTATTCCTGGTGGG
GCTGGGTCGATGTCAGCCACGACATGATGCCGCGGATCTTCCGGCCCGATCCGAAACAGCCGCTGTTCTATGC
GATGGGCTATGGCGGCAACGGCGTCATGTATTCCGCCCAGGCCGGCCGCCGCATGGCCCAGATGGTGGCGGGC
AAGGGCGGCGCACTCGACCTGCCGATCTTCACGTCGCCGCTCCCGAGCCATGGCCTGCTCACGCCTTTCAGAC
GCCTCGGCCAGTGGGGCATGTATCGCTGGTACTACCTCAAGGACGAAATTCTTTAGATGATCTCCTTCATCCT
TCGCCGGCTGGCGAGTGCGGTGCCGACGCTGTTCATCGTCGTCACCATATCCTTCTTTCTGATGCGTTTTGCC
CCCGGTGGGCCCTTCAACCTCGAGCGTCCTCTCCCGCCCTCCACGATGGAGAACCTGATGAGGACCTATCACC
TCGATCAGCCGCTCTGGCGCCAATACGCTACCTATCTAGGCAATGCGGTCACGGGCGACTTCGGTCCGAGCTA
CATCTACCATGACAACAATGTGGCGCAGCTGATCGGCAAGGGCCTGCCCTATTCGATGGAGCTCGGCTTTTAC
GCGCTGTTACTCGCGGTCGTCGGCGGCGTCATAACGGGAACGATCGCCGCATTGCGGCAGAACAGCTTCCTGG
ATTTCGCGGTCATGTCGGTTTCCACGATCGGGGTCACCGTTCCGAACTTCGTCGTCGGTCCCGTTCTGACGCT

GATCTTTGCGATCGTGCTCGCGTGGCTGCCCGCCGGCGGCTGGGGGGACGGTTCTCTCCGGTTCCTCATCCTG
CCGATGATCGCGCTCGCGCTTCCCCAGCTTGCAGTGTTCGCCAGGCTCACACGCGGCTCGATGATCGAGGCGC
TCAACACGGATCATATCCGCACCGCGAAAGCCTATGGCCTGCCGTCCCGCGCCGTGGTCGTGACGCACGCCAT
GCGCGGTGCCATGCTGCCGGTCGTCTCCTATCTTGCTCCTTGCGCCGCCGCCCTCCTTACGGGCTCGGCCGTG
GTGGAAACGATCTTCACTATACCGGGGGTCGGGCGCTACTTCGTGCTCGGAGCAATCAACCGCGACTATACGC
TGGTAATGGGCACCGTGATCCTCATCGCGATCTTCGTCATCCTCTTCAATCTTCTGGTCGATATTCTCTACGG
CCTGCTCGATCCGAGGGTTCGCCATGACTGAATGATCGAAATCGAGGAAATAACCAAGCGCTATGGCGACACG
ACGGTCGTGAGCGACGTGTCGCTGACGGTCGCGCCGCATACGGTCACCGTCGTCGTCGGAACCTCCGGCTCCG
GCAAGACGACGCTCCTCAGAATGATCAATCGCCTCGTCGAGCCGACGGCAGGGACGATCAAGATAGACGGCGA
GGACAATCGATCGATTCCGGGTTTCGAGCTGCGCCGGCGTATCGGCTACGCCATTCAAGGCCATGGCCTCTTC
CCGCACAGGACAGTCGCGCAGAACATCGCGACCGTGCCGACGCTGCTCGGCTGGGAGAAAGCCCGCATCGATG
CCAAGGTGGAGGAGCTGCTGAAGCTGTTCCAGCTCGATCCCTCGGAATTCGGACCGCGCTACCCTCACGAGCT
TTCCGGCGGGCAGCAGCAGCGCGTGGGCGTTGCAAGGGCACTTGCGGCGGAGCCCAACGTACTCCTGATGGAC
GAGCCCTTCGGCGCGCTCGATCCGATCATCCGCGCCAAGGCGCAGGACGACCTCGCCGCCATCCAGAAACATT
TCGGCACCACGATCATCCTCGTCACCCACGACATGGAAGAGGCCTTCCACCTTGCCGACAGGATCGCGGTGAT
GGACAAGGGCGAGGTGGTGCAATATGCGACGCCGGCCGAAATGCTCGTCAGGCCCGCCACGCCCTTCGTCGAG
ACGCTGGTCGGCGCCAGCGAGCGTCCATTCCGGCTGCTCGACATCGAAACGGTCGGCGAGGCGGTCGAGCCCG
GCTCGGCCGAGGGCCGGCCGGTTCCGGAAGTTCTCAGCCAGCGTGACGCGCTGTCGGAGCTTCTGTGGACGGG
CCGTTCGGCACTGCCGGTCGCGGCGGCGGACGGCGCGATCCTGGGCAAGATCACGCTCGAAGCGATCGTGAAG
CGTGCGGCGAGGCGGCAGTGAATGCGCGTGCCCCCCGGGATTGCCGACGATCTCACCTTTCTCACGGCCTTTC
GCCGCGACCTGCACGCTCATCCCGAACTCGGTTTCGAGGAAGAGCGGACGAGCGATCTTGTGGCGAGAATCCT
CGAAGAAGCAGGCCTCAGAGTCCACCGCGGCCTCGGCAAGACAGGCGTCGTTGGCACGCTGCAGGTCGGCAAC
GGCACGCGCGCGATCGGCCTGCGCGCCGACATGGATGCGCTCGCCATGCCGGAACTCGCCGACCGGCCCTACA
AATCCACGGTAGCGGGGAAGATGCATGCCTGCGGCCATGACGGCCATACGACGATGCTGCTCGGCGCGGCGCG
CCACCTTGCGGCGACGCGCAACTTCTCAGGAATGGTCCATTTCATCTTCCAGCCCGCCGAGGAAGGCCGCGGC
GGCGCCAGGCGCATGGTGGAGGACGGGCTTTTCGATCTTTTCCCTTGCGACGCGGTCTATGGACTGCACAACA
TGCCGGGGCTTGCAGTGGACGAGATGGCCGTCGTGGCGGGGCCGCAGCTTGCCTCCTCGGACAGCTGGCGCCT
CACCTTCCGCGGAGTCGGGACGCACGGCGCCAAGCCGCATCTCGGCCGGGATCCGATCACGGCGGCGGGGACA
TTCCTCGCTTCTCTGCAAACCGTCGTCGGCCGGGTCGTCGACCCGCTGCAGCCGGCGGTCGTCAGCGCCTGCG
CGCTGCAAGCAGGCGACCCCAAGGCGCTGAACGTCATTCCGGACACGGTCGAGATCGGCGGCACGGCGCGTGC
CTATACGCCGCATGTGCGCGACCAGTTGGAAGAAGAGATCGGCCGGTTGGCGCTGGGGACCGCGGCGATGTAC
GGCATCGAGGCCGATTATCGCTTCGAGCGGCGTATTCCCCCGGTCGTCAACGATCCCGACGCGACGGCACGCG
CCCTTTCGGCCGCGCGGTCCGTCTTCGGTGAGAAGGCGCTGACCAGCTTCCCACCCTCGACCGCCGGCGACGA
CTTTGCCTTCTTCGCGCTGGAGGCGCCCGGCTGCTATGTCTGGCTCGGGAATGGTCCCGCCGTCGACGGCGCG
CTGCACCATAACAGCGCCTACGACTTCAACGACGCGGCGATCGGCCCCGGGGCTGCCTTCTGGACCGCGCTGG
TGGAGCAGGAACTCAAGGCCTGAATGAACACTGGTCCTGATGCCGAAAAACTGCTGAACCGCATCCGGACGCG
CACCTTCACGATCGGTATTATGGGGCTTGGATACGTCGGATTGCCGCTCGCTATGAGCTCGCTTGAGGCGGGG
TTCAGGGTAATCGGTTTCGACATCGACCAGTCGCGGGTCGCGCTGCTCAACCGCGGCGAAAGCGGAATAAAGC
ATCTGCCTGGCCCAGCCATTCAGCAGGCTGTTGAGAGCAAGCGGTTTCGCGCCACCAGCGATTTCTCTGAGTT
GAGCATGCCTGATGCGATCCTGATAGCGGTTCCGACGCCGCTTACTAGAAATCGGGAGCCAAACCTGTCGTTT
GTCGAAAACAGCGCAAGGTCGATCGCTGAGCGGCTCCGTCCTGGGCAACTTGTCGTCTTAGAGTCGACCACTT
GGCCAGGCACGACACGAGAAATCGTGCGGCCGCTGCTGGAAGCGACGAACCTTGTCGTTGGAAAGGATTTTTT
TCTTGCATATTCGCCCGAGCGCGAAGACCCCGGAAACGAAGTGCACACTACCACCAGTGTTCCGAAGGTGATC
GGCGCCGACGATCCGTCTTCACTGGAAGTTGCGCAAGCACTGTACAGCCAGATTATCGTGCGGACTGTTCCCG
TGACTTCGACCGCAACCGCGGAGGCGGTCAAGCTAACGGAGAACATATTTCGCTCCGTGAACATTGCCTTGGT
CAACGAGCTGAAGGTCATTTTCGACAGGATGGGCATCGATGTTTGGGAAGTGATCGAAGCCGCCAAGACAAAG
CCGTTCGGATATATGGCGTTCTACCCCGGACCGGGCCTCGGCGGCCACTGCATCCCCATCGACCCGTTCTACC
TGACATGGAAGGCTCGCGAGTACGAAGTAGCAACGCGCTTCATAGAATTAGCTGGAGAGGTCAACACGAACAT
GCCGCACTACGTTGTTGACCGCCTTGTGGAGGCTTTGGACCGCAGAGCCCGCCGCGGCTTGAACGAATCTCGA
ATCCTATTGATCGGGATGGCTTACAAGAAGAATGTGGGGGATACACGAGAGAGCCCGTCGCTACGGCTAATCG
ACCTGATGGAGCGACGGGGCGCAGTCGTTGACTACCACGACCCTTACGTACCTGAGATTCCCGCCACAAGAGA
GTATGCCGAGTTAGCAGGTCGGCGCTCGGTGGATATTTCAGTTGACCGCCTGGCCTCGTTTGATGCCGTTGTC
ATTACGACAGATCATGATGCCATTGATTGGAAAACCGTCGTTGAAGGTGCAAAGCTGGTGGTCGACACCAGAA
ATGTCTGTGCACGCTTCCATGTGAGCGCCCCTAATGTGGTAAAGGCATAAATGCGTATCGTCATCGGTTCCGA
CCATGCTGGCTTCCACCTCAAGGCCACGATCATCGACCATGTCACCTCGCTCGGCCACGAGGTGTACGACGCA
GGCTCATACGATGCGAACCCCGTAGACTTCCCCGACATCGCAAAGATGGTCACTTCTTCGATCATCGAGGGCA
AGGCGGATCGCGGGCTGATGGTCTGCGGCACCGGCGTCGGCGCCTCGATCGCGGCAAACAAGGTGAAAGGTAT
CCGCGCAGCGGTCTGCCATGACGTGCATTCCGCCCATCAGTCGGTGGAGCATGACGACGTCAACGTCATGTGC

ATCGGCGCCCAGATCGTCGGCGCATGGTTGGCCAGGGATCTCGTTGCAGCCTACCTCGCTGCCGAATTCTCCA
CCGACGAGGACTTCCGGCGCCGCGTGCGCAAGCTTGCCGAAATGGACGAGCAGCGCTGAATGGAAGCTCTTGC
AGCCCTGGCGCGAAACTGCATCGTCACGCTGTTGTGCGGTTGCGCGCTTTTCGCACCTTCGCTTGCTGCCGAG
GAGGCCGATGATGTCGCGACCGGGACGCGGCTTGCCGAGCTGTTGCGTGCCGCTCGAAACGTTCTTTCGAATT
ACCAGTCGCTCATCAATGACCCGGCCCTCGGAGACAAGAAACTCGACGGTGAGCGTTTCACTGCCGAAGCGAT
CGCCCTTTATGGCGAGCGCACCGGCCATCCGTTGATGTCGGATGATCTCGGCGATCGCGACCGCAAGCTTCTG
CAGGCGCAGGTCGAGGCGATGCGGGAAGTCGTGAATGAGCAGCAGGACGACATAAACCGCCCCGGGATCGGGT
TCAAAGGTTTCGTGCCGGCCATTTTCGCGCGTCTGATGAACGAGAAATTTGCCGTCAAGGCGGGCAATGAGGC
GCTGGTCCGCGTTACGGCGCCGGAGGTTCTGGTGCGCAACCGCAAGTCGCTTCCCGATGCCTGGGAGGCGCGA
GTCATCAACGAGGTCTTCTCGGATCCGCAGAGGCCGAAGGGTGAACTCTATACGGAAGTGACGCAGGTGAACG
GCCGCCCGGCTTTCCGGATGCTGCTGCCGGAATATTACACGGAGTCCTGCCTTTCCTGTCACGGCTCGCCGAA
GGGTGAAATCGACGTCACCGGCTACCCGAAGGAAGGCGGCAAGGCGGGCGATCTGGGCGGTGCCATCAGCATC
GTCCTGTTCAAATGAATGATTTTCACCCCCAAAGGAAAACACCTCGTCGCGGGCGAATGGCTCGATGGGGCAG
GCACCTTCGCTTCGGCGCCGGCGCATGGCCCGGCGCATGATTTTGCCGTAGGCACCGTAGAGCTGGTGAACCG
GGCCTGCGAGGCGGCTGAGGAAGCCTTCTGGACCTATGGCTATTCCTCGCGCAAGGAGCGCGCCGCCTTCCTG
CGCGCCATCGCCGACGAGATCGAGGCCCGGGCGGAGGCGATTACCGAGATCGGCAGCCAGGAAACCGGCCTGC
CCGAGGCGCGGCTCAACGGCGAACGGGGCCGCACGACCGGCCAGCTCAGGCTCTTTGCCGATCATATCGAAAA
GGGTGATTATCTCGACCGGCGCGTCGATGCGGCGATGCCCGAGCGCCAGCCGGCGCCCCGTCAGGAGATACGG
CTGGTCCAGCGCCCCGTCGGTCCGGTCGCCGTCTTCGGCGCCTCGAATTTCCCGCTGGCCTTTTCGACCGCCG
GCGGCGATACGGCCGCGGCGCTCGCAGCCGGCTGCCCGGTCGTGGTAAAGGGTCATTCGGCGCATCCCGGCAC
CGGCGAGATCGTCGCCGAGGCCGTCGACGCAGCGATCCGCAAGACGGGTGTTCATCCCGGCGTATTCTCGCTG
ATCCAGGGCGGCAGCCGCGACGTCGGTCATGCGCTGGTGCAGCATCCCCATATCAAGGCCGTAGGCTTCACCG
GTTCGCTCGCCGGCGGACGTGCCCTGTTCGATCTTTGCGCGGCGCGCCCCGAACCGATCCCGTTCTTCGGCGA
ACTCGGTTCGGTCAACCCGATGTTCCTGCTGCCGGAAGCGTTGAAGGCACGGGCGGAAACGCTGGGGCAGGGA
TGGGCCGGTTCGCTCACCATGGGCGCCGGGCAGTTCTGCACCAATCCCGGAATCGCCGTCGTCATCGAAGGTG
CGGACGCCGACCGCTTCACCACCGCCGCCGTCGAGGCGCTTGCCAAGGTGGCACCGCAAACCATGCTGACGGA
CGGTATCGCCAAGGCCTACCGCGACGGTCAGGCGCGCTTTGCGACGCGCAACGCCGTCAAACCGCTGCTTGCG
ACCGAGTCGTCCGGCCGTGACGCATCGCCCAATCTCTTCGAGACGACGGGTGCCCAGTTCCTGGCCGACCATG
CGCTCGGCGAAGAAGTGTTCGGCCCGCTTGGACTGGTGGTTCGGGTGGGATCGCCGGCCGAAATGGAAGAGCT
CGCGCGCGGCTTCCAGGGCCAGCTGACCGCAACCATCCATATGGATGCGGGCGATCTGGAGACGGCGCGCCGT
CTGCGTCCGGTGCTCGAGCGCAAGGCGGGCCGGGTGCTGGTCAACGGCTTCCCGACCGGGGTTGAAGTCGTGG
ATTCGATGGTGCATGGAGGCCCCTATCCAGCCTCGACCAACTTCGGCGCAACCAGCGTCGGCACCATGTCGAT
TCGACGTTTCCTGCGTCCCGTCGCTTACCAGAACATGCCGGAAGACCTGCTGCCCGAGGACTTCCTCGGCTGA
ATGCGCACTCGCGCTGCTCTGGCCGTTCAGGCCGGAAAACCCCTTGTCGTGACCGAAGTCGAGCTCGAAGGCC
CGCGGGCCGGCGAGGTGCTGGTCGAGGTGAAGGCGACCGGCATCTGCCATACCGACGATTTCACGCTGTCGGG
CGCCGATCCGGAGGGGCTGTTCCCGGCGATCCTCGGTCATGAGGGCGCCGGCATCGTCGTCGATGTCGGCCCC
GGCGTCACCTCGGTGAAGAAGGGCGACCACGTCATCCCGCTCTATACGCCGGAATGCCGCTCCTGCCCCTCCT
GCCTCAGCCGCAAGACCAATCTGTGCACCGCCATCCGCGCCACCCAGGGGCAAGGGCTGATGCCGGACGGCAG
CTCGCGGTTCTCGATCAACGGCGACAAGATCCACCATTATATGGGCTGCTCGACCTTTTCGAACTTCACCGTG
CTGCCGGAGATCGCGCTCGCCAAGGTCAATCCGGACGCCCCCTTCGACAAGATCTGCTATATCGGCTGCGGCG
TCACCACCGGCATCGGCGCGGTGATCAACACCGCCAAGGTCGAGATCGGCGCCACCGCCATCGTCTTCGGTCT
CGGCGGCATCGGCCTCAACGTCATCCAGGGGCTTCGCCTTGCCGGCGCCGACATGATCATCGGCGTCGACCTC
AACAACGACAAGAAGCCCTGGGGCGAAAAGTTCGGCATGACCCACTTCGTCAATCCGAAGGAGGTCGGCGACG
ACATCGTCCCTTATCTCGTCAACCTGACGAAGCGCGGTGCCGACCAGATCGGCGGCGCCGACTACACCTTCGA
CTGCACCGGCAACACCAAGGTCATGCGCCAGGCGCTCGAAGCCTCGCATCGCGGCTGGGGCAAGTCGGTGATC
ATCGGCGTCGCCGGCGCCGGCCAGGAGATTTCGACCAGGCCGTTCCAGCTCGTCACCGGCCGCACCTGGATGG
GCACCGCGTTTGGCGGCGCGCGCGGCCGCACCGATGTGCCGAAGATCGTCGACTGGTACATGGAGGGCAAGAT
CGAGATCGATCCGATGATCACCCACACCATGCCGCTCGAGGACATCAACAAGGGTTTCGAGCTCATGCATTCC
GGCGAGAGCATTCGAAGCGTGGTCCTCTATTGAATGAACACATATGAAAGGATCGATAGAGACCTGTCCGACC
GGGACTTCACCGTCGTGGGCAAGAGCGTCAAGCGTTCCGATACTCTGGAGAAGGTCACGGGGACGGCCAGATA
TGCAGGCGACGTCGCCTTGCCCGGCATGCTCCACGCCAAGATGAAGCGCAGCAACATCGCGCATGCGCGCATC
AGGAGCATCGACACGAGCAAGGCGCTGGCGCTCAAGGGCGTCAAGGCGGTGCTGACGCACGAGGACGTGCCGC
GCGTTTTGCATGCGGGCTCACCGCATCCGCGCTCGGCATCCGTTACCAAGGATCAGTACATCCTGGATGACAG
GGTGCGATATTGGGGCGAGGGCGTCGCCGCAGTCGCTGCCGTCAGCGAGGAAATCGCTGAGCGCGCGGTCGCG
CTCATCGAGGTCGAGTACGAGCCCTTGCCCGGCCTCTTCACCATCGAAGCCGCATCGGTGCCCGGCGCTCCGC
CGATCCATGAGAATGGTCTCGACCGGAACCAGGTGTTGCCGCCGGTCTTCGTCACCCGCGGCGACGTCGACAG
GGGTTTCGCCGAAGCGGATCTGATCCTCGAGCGCGAATACGATCTCGGCCGCCCGACACCGGCCTATATGGAG
CCCAATGTCTGTGTCAGCCAGTGGGACGGCAACGGCAAGCTCACCATGTGGACCTCGACCCAGAGCGCATTCA

TGGTCCGCGGCACGCTTGCCGAAGTGCTCGGCGTGCCGCTGAACAAGGTACGCGTTATCGTCGATCACATGGG
CGGCGGCTTCGGCGCGAAGCAGGATCTGTTTCAGAACGAGTTCCTCTGCGCCCTGCTCGCGCGACAGACGGGG
CGTCCGGTCAAGATGGAGTTCAGCCGCAAGGAAACGTTCCTCGGCGGCCGCTCGCGACACCCCGGCAAGATAT
GGCTGAAGCAGGGCTTCACCAAAGACGGCCGGATCGTCGCACGCGAAGCCAGGGTCACCTTCAACTCCGGCGC
CTACGGCTCGCACGGTCCAGGCGTCACCAATGTCGGCACCGCAGCGCTGACCTCGCTCTACCGTTGCGAGAAT
GTCCGGCTGGAAGGCCGCTGCATCTATACCAATTCGCCGATCGCCGGCGCCTTTCGCGGCTATGGTGTGGTTC
AGACCTACTATGCGCTCGACCTCATGATGGACGAGGCGGCCGAGAAGCTCGGCTTCGACCCGGCCGAGTTCAA
GTTGATGAACGCGGTGCGCGAGGGCGACATCGCACCCTCCGGGCATCCGATCGTCGGACACGGCCTCGGGGAT
TGCATCCGCCGCGTCATGGAGGAGACGAACTGGCACGAATTGCGCAGGCGGGAGAAGCCGGAAACCGTCAAGC
GCCGCGGGATCGGGATCGGCTGCGAAATGCACGGATCCAGTGCCTATCCGGGCATCAAGGAACAGGGCAACGC
AATCGTGAAGATGAACGAGGACGGAACGGTGACACTGATCACCGGCACCGCCGGCCTCGGCACCGGCGCGCAT
ACAGCGCTTTCGCAGATCGTCGCCGAGGAACTCGGCGTGCCCTTCGAAGCCGTCTCGGTCGTTCAAGGGGACA
CCGATATCGTCCCCTGGGATATCGGTGCCTTCGCCAGCCACACGACCTATCTCGGCGGACGGGCGGCTCAGCT
CGCGGCCGCCGACGTGCGCAGACAAGTGCTCGAACACGCCGCGCCCATGCTCAAGACCGAGCCCGAGAATCTC
GCGATCCGGGACGGTTTCGTGGTCGTCACCAACGGCTCCAACCGCAGCATCCGGGTTTCCGAGGCCGTCGGGC
CGCAACGGGGGATGCCGGCGGTGCAACTAGTCGGCGTAGGCACCTACATGCCGACGAAGTCCTACTCCTTCGC
CGCCCATTTCGCCGAAGTCGAGGTGGACACCGAGACCGGCGAGGTGGCCGTTCTCGAGGTCGTTCCGGTACAT
GAGATCGGCAGGGTCATCCATCCGATCGCCGCGGCCGGACAGATCGAGGGTGGCATTCAGCAGGGCATCGGCC
ACACCCTCAGCGAGGATTACGTCATCGACCTTACCGACGGACGTTCCCTCAATCCGAGCTTCGTCGATTACAA
GATGCCCCTGTCGATGGACATGCCGTCCATCCGCACCATCATCCTCGAGACGGCACCGGATCCCGGCGGTCCC
TACGGTGCCAAGGGCGTCGGCGAGGATCCGATCATCGCGATCGGGCCCGCCATCGCCAACGCCATCTACGACG
CCATCGGCGTCCGCTTCCACCACTACCCGATAACGCCCGAGCAGGTGTTGAACGCTCTCAAGTCCAAAGCCAA
CGAAACGAGGCAGTGAATGGCAAACAGTGAAAGTGGACAGCCGCTGGCGGTCGTCCCCCAGGCCTATGGCGCC
CTTTGGCGAGGCCTGCTCGGTCCTTTACGCACGATTATCCAGCCGGCCGCCGCTGTTGTTCTGGCTCTCGCAT
GCGGCGCGGCGCTCCTTGCCTTCAACGGTTACGAAGCACGCGCCGTGATCGATGTGATGGTGCTTGGCGTCTT
CCAGGACACCCGCTCGATCGCCGAAATTCTCCTGAAAGCAACGCCGCTGATCCTGATCGGCGTCGGCCTTTGC
GTCGCCTTCCGCTGCAGCATCTGGAACATCGGCGCCGAGGGGCAATTCTATGCCGGCGCCATCGCCGCAACCG
CCGTCGGCGTTTCCTTCGACGGGTTGACCGCGTGGATCTACGTCCCGCTGGTCATGATTGCAGGCGCTCTTGC
AGGTGCGGCATGGGCGGCGATCGCCGGACTTCTGAAGGTCTATTTCCGGGCCAGCGAGATCGTGACGACGATC
ATGCTGAATTACATCGCCATTATCATGACGAGCTATCTCGTGACCGGCCCGCTGCGGGATGCGGCTGCGGCCT
ATCCGCAATCGGCCCGGCTGGTCCAGGAGGCATGGATGCCGCGCATCCTGCCGCCGACCCGCCTCCATATCGG
CATCCTCGTCGCCCTGGCGCTGGCCGTCGTGATGTACATCTTTCTGTTCCGCAGCACCCGGGGCTATGCGCTG
AGGGTGGTCGGCATCAGCCCGGACGCCGCGCGCTATGCCGGGATCAGCGTTCCCAGGAACATCATGCTGTCGA
TCTGCCTGAGCGGCAGCATGGCCGGGCTCGCAGGGGCCTTCGAGATCGCCGGCGTCACCCGGCGGCTCTTTCA
GTCGATCTCTCCGGGCTATGGCTTCGAAGGCATCGCGGTCGCTCTTCTCGCAAACAACAATCCGATCGGAGCG
GTCTTCTCCGGCCTCTTGTTTGCAATTCTGCGAAGCGGCTCCGAGCTCATGCAGATCACGGCACAGGTGCCTC
AGGTTCTCGTGCTCGTGATTCAGGGCATCGTCATTCTCTCGGTCGTGGGATTTGCCGTGTTGCGGATCCCGTC
GATGCGCCCTTCCGAATAGATGATTGATTTTTCCCGTGCCCGCGAACGGATGATCACGTCGCATCTCTCGCGA
CGGGGGATAAGGGACAGACATGTCGTGGAGGCGATGGGGGTCGTCCCGCGGGAGGCATTCGTCGATCCCGGCT
TCGAGGAATTCGCCTATGAGGATTCGGCCCTTTCGATCAGCCATGGCCAGACCATCTCGCAGCCCTATATTGT
GGCGCTGATGATCGAACGCGCCGAAGTCCAGCCGGGCGATACGGTGCTCGAGATCGGGACCGGATCCGGTTAT
GCGGCGGCGGTGCTCAGTCGGATCGCGGCATATGTCTATACGATCGAGCGCCATGCCGGCCTCGCCGAGGTTG
CCGAGAGACGATTTGCCGAGCTCCGTTACGGGAATATCGATGTGCGTGTCGGCGACGGCACGGCCGGCTGGCC
CGAGGCAGGACCCTTCGACGCCATACTGGTTGCCGCCGGAGGTCCCGAGATACCGTATGCGCTGAAGGAGCAG
CTCGACCTCGGTGGCCATCTCGTCATACCCGTCGGCCCCCCTGAGGAGCAGCGCCTGATGAAGGTCACGCGGG
TCAACGCCACGACATTCGAGGAGCAGGACCTCGGCGGGGTCAGATTCGTGCCGCTCGTCGGCGAACACGGCTG
GCATGAGGAGCGGGTTCAATCCGGGGCAAGGCTCAGAGCGGCGCCCACCTTGCCCGAGCTGGTTGCAGAGGCG
GCCGAGCCGCTGCCCGATCTCGACGATCCGGCGTTCGGGCTGCTTTTCGATCGCTTTGCCGGGCGGCGGATCG
TGCTGCTCGGAGAGGCGAGCCACGGCACGTCGGAATTCTACCGCGCCCGCGCAGCCATCACGCGCCGCCTGAT
CGAGGAACACGGGTTCACGATCGTGGCGGTCGAGGCGGATTGGCCGGATGCGGCGGCAGTCGACCGCTATGTC
CGACATCGAAGTCAAGGCATGCGCCTCGACGCGCCGTTCCAGCGGTTCCCGACCTGGATGTGGCGCAACCGCG
AAGTGATGGATTTCGTCGAGTGGATGCGCGAACATAACGGCCGCAGGAGCTTGCCTGACCGGGCCGGCTTCTA
CGGTCTCGACATCTATAATATGCGCGGCTCCATTTCCTCGGTTCTCCGGTATCTCGATGAGACCGACCCCGAG
GCCGCCGCGGTCGCCCGCGAGCGCTACGGTTGCCTGACCCCCTGGCAGAACGAGCCGTCGACCTATGGCCGTG
CGGCCATGACCGCGGGCTTCCGCAAATGCGAAGAGGCCGTCGTCAGGCAATGCCGCGAACTCCTGGAGAGGCA
GCTCGAAGCCGGGCGCGACAGCGGCGACGAGCTTCTCGACGCCGTGCAGAACGCGCGCCTCGTCGCCTCGGCC
GAGCGGTACTACCGTATCATGTATTATGCTGGGGCCGACTCCTGGAACATGCGCGACACCCATATGTTCGAAA
CGCTCGAGCATCTGTTGAATGCACGCGGCGCTCGCTCGAAGGCGGTCGTGTGGGCGCACAATTCCCATATCGG

CGATGCCCGCCATACCGATATGGGCACGGCCCGCGAGGAGTTGAACATCGGTCAGCTCTGCCGCGAGCGGTTT
GGCGATCAGGCAGCGCTGATCGGCTTCGGCACGCATGGCGGCCATGTGGCTGCCGCAAGCGATTGGGACGGCG
ATATGGAGGTGAAGCCGATCCGGCCGTCGCTCGAAGGAAGCTACGAGCGCGTGATGCACGACTCAGGGGTAGG
GTGCTTCCTCATCGACTTCGCGCGCCACGCGCGTCTGCGCGACGGTCTCCTCAAGCCGATGCTCGAGCGCTTC
ATCGGGGTGATCTACCGGCCGGACACGGAACGCTTCAGCCATTATGCCCACGCTTCGCTGCCCCGGCAGTTCG
ACGCCTTCGTCTGGTTCGATCAGACGACGCCGGTCGAACCGCTCGGCGCGCAACACATAAAGGCGGGCGTGCC
GGACACGTTCCCGTTCGGCCTGTGAGTGAGCCGCGTGCTGACCCTGGCCGGGCGAACGGGGACGGGACGCGTC
GGCGCGGCTCTCGCCGCGCTCGTCATCCTCTTCGCAGCACTCTGGATGGGCGCTGCGATCGGCGAAACGGCGA
TCCCGTTCAAGACCGTTGCGCAGACGGTGGCGAACCGGCTGTGGAACGCCGGCTACCCGCTGGCACCGATCGA
CGAGGGCATCATCTGGAGCTACCGGCTGAGCCGGGCAGTCGTGGCCGCTTCCTGCGGGGCGTCGCTCGCGCTC
TCCGGCGCCGTGCTGCAATCGCTGCTGCGCAATCCGCTCGCCGACCCCTACATTCTCGGCATCTCCGCCGGCG
CCTCGACTGGCGCGGTCAGCGTCGCCATTCTCGGTGTGGGTGCCGGGATGCTGACGCTGCCGCTCGGCGCCTT
CGTCGGCGCGCTGGTCGCTTTCGTCCTGGTGAGCCTGCTGGCGGTGAAGGCCGGCCGCGGCACCGCGGCGATC
ATCCTGGCAGGTGTGGCGGGATCACAGCTCTTCAACGCGCTGACCTCCTTCATTGTCACCAAGGCGGCGACGG
CCGAGCAGGCGCGCGGCATCATGTTCTGGCTGCTCGGCAACCTTTCGGGCGTGCGCTGGCCGGATGCCTGGCT
CGCCGTGCCGGCGACGCTTCTCGGTCTCGTCGTATGTCTCTGGCATGCCCGTCCCCTCGACGCGTTTACCTTC
GGGTCGGAATCGGCTGCCTCGCTCGGCATCTCGGTCAGGCGAACCTATTTCGCCCTCGTCGGCGTCTCGGCGA
TGATGACGGCGGTGATGGTATCCATCGTGGGGTCGATAGGTTTCGTCGGCCTCGTCATCCCCCATGCGGCCCG
CATGCTCGTCGGCGTCCGCCACGGTGTCCTGCTGCCGGCAGCGGCGCTCATCGGCGCCGTATTCATGATCCTC
GCGGACATCCTGTCGCGCGTTCTCATTCCCGGCCAGGTCTTGCCGATCGGCGTGATCACGGCGCTCGTCGGCG
CCCCGGCCTTCGCGTTGATACTCGGACAAAGGAGGGACCGCGCATGAATGACCATCGAAGCCGAAAGCACTCT
TCCGGTGGCGATCGAGCCCGAGGAAAAGCACCACCACGAGAGCTACTCCGCTCTCGTCTGGCGCCGGTTGAAG
CGTTCCTGGACCGGCCTGCTCGGCCTTGTCCTCGTCTGCCTCCTGATCCTGATGGCGGTCTTCGCCGAGTTCC
TGTCGCCCGTCGATCCGAAGGCGACGGACGTAGCCTTCGCTCAGCCGCAGACGATCAGCTTCCGGGACAAGGA
GGGTAATTTCGTCTTTCCGCCGAGGAGCTATCCGGTGCGGGAGACGGAGGAGCTCGATCCCGTTACCTTCCAG
CCGATCATCGGACCGGATTACGACAACCCGCAGGTCCTCGGCTTCTTCGTCAAGGGCGCACCCTACAGGCTCT
TCGGGCTGATCCCGGCCGAGCGGCATCTGTTCGGTGCCGTCGACGGTACGCCGGTGCATCTGCTCGGCACCGA
CAAGTTCGGCCGCGACGTGCTGTCCCGCATCCTCTACGGCTCGCGCATCTCGCTGATGATCGCGCTCACCGTC
GTCTTCATCGTCACCGTGGTCGGGACCACCGTCGGCATGGTGTCCGGCTATTTCGGCGGGCGCTTCGATGCCT
GGGTGCAGCGTTTCGTCGAACTTGTGCTCGCCTTCCCGCAATTGCCGCTCTATCTGGCGCTCGCCTCGCTGAT
CCCGGTCACCGCGCCGACCAATGTCTTCCTCGCCTTCGTCATCATCGTGATGTCGGCGCTCGGCTGGGCGCAG
ATGTCGCGCGAGGTGCGCGGCAAGACCCTGGCGCTTGCGCGGATCGAATATGTGCGGGCGGCGATCGCCATCG
GCGCGACGGACCGGCGCATCATATTCCAGCACATCTTCCCGAATGTGATGAGCCACGTCATCGTCGCGGTGAC
GCTCGCCATCCCGCATGTGGTGCTGCTCGAGTCTTTCCTCGGTTTCCTCGGCTTTGCGGTCAAGCCGCCGCTG
ATCTCCTGGGGGCTGATGCTGCAGGATACGGCGAATTATTCCGCGATTGGTTCCTATCCCTGGATTCTCTCGC
CCGTCGCCTTCGTGCTCGTCACCGTTTTCGCCTTCAATGCGCTGGGCGACGGCCTGCGCGACGCAATCGACCC
CTATTGAGTGAAGCTCGATCCCTTCTATCTCATCGTCGACAGCGCCCAATGGATCGAACGGCTGGTGCCGCTC
GGCGTCAAACTCGTGCAGCTCCGCATCAAGGACCGGAACGAGGAAGACATCCGCCACCAGATACGGGTCGCCA
GGGCAGTCTGCTCCGCCCATGCATGCCAGCTGATCGTCAACGACTACTGGCAGCTGGCGATAGAGGAAGCCTG
CGATTTCATCCATCTCGGTCAGGAGGATCTGGCGGAGGCCGATCTTGCCGCCATCCGTGCCGCCGGGCTCAAG
CTCGGCGTCAGCACCCATGACGAGGCGGAACTGGCGAAGGCGCTCGCAGCCGAACCGGACTACGTCGCGCTCG
GGCCGATCTATCCGACGATCCTCAAGAAGATGAAGTGGGCGCCGCAGGGGCTGGAGCGGCTTTCATGGTGGCG
CGAGCGCGTTCACCCGCTCCCGCTCGTCGCCATCGGCGGCCTCAATACGGAGCGGATCGAAGGCGTCTTCGCG
CATGGCGCCGACAGCGCCGCCGTGGTGACTGACATCACGCTGAATGCCGATCCGGAAGGAAGGACAAGGGAGT
GGATACGGAAGACGGAGGCGTGGAGATAGGTGGGGCAAGCTGAGGCAGCAACGGGAAAAAAGAGACGCAAGGC
GCTTTGGGCCGCAGTTGCGGTGATAGCAATCACCGCCGCCGGCATGGCCTGCTACAAGACCATCGTAGAAAAA
CGCGTGAGCAGGATGATCGCCGAGCGCGGCGGCAAGGCGGGCAGCGTCGAAGCGGATTTCCTTGGGCGAATTC
ATCTGCGCGACGTGGCCCTGCCGCTGAAGGACGGGTCGGACGTTCGTATCGCCGCGGTTGACGGCCGGCCCAG
TATCTTTTTCCTCCAGGGCATGCTCGAAGTAAGCGGCCTTGAACTCAAGACTGCTTTCGGCACGGTCTCTGTC
CCGCGCGCGGTCGTCGAAGAAGCAGATTTTGATCGCGGCACCTTGGAGCAGATGTTCGGCGGGAAGAGCGAAC
TCTCGATCTCCAAGCGAGTAGAACTTTTCACGGCAAGGCGTATCTCCGCCCCGGAGATCGTCTTCGCACAGAT
CGTTGCCGGGACCGAGCAGAAGCTGATCTACAGGAACGTGGTCTCGGAAGGCATCGCCGATGGGCGTATCGCC
CGCTATTCGGCGAGCGGCGGCAGCTTCGAAGTCGGCGTGGACCGTTCGGGGGGAGAGGGCGGCAGGAAGAATA
GGGTGGCGGGTTCCATCGGAGCCGTGACGGCGCAGGATATCGATGCCGCCTATCTGGCGCGGCTCTATACCGA
AAAAGCCGGCTCGGACGACAACGAGGCAAAGCCTGTCTATGGGCCGATTTCGGTAAAGGACATCGCCTTCTCG
GATGGCGAGGCGAGCTTCGGCTATGACGAGGTTCGCAGCAGCGGCTTCAGCATGCGAATGCCGGCCGAGCCGC
TGGTCGAGACGCTCAAAAAGCTGAAATCGGTCTCGGCGCCGGATCAATTGCCGCCGGCGGAACGCCAAGCCTA
TTTCAATCGGGTCTTCTCGATTATCGACATGACGAAGAAGGCCGACATAGAAATGCTCGGCCTTACGATCGAC

GCGCCGATGAAGGACGGTGACGAAGGCGAGAGGATCAAGGTTTCGATAGATCGCCTCGCCCTCGAGGTCGATG
GACGGAAGCTTGACGGCACGCTGAATGGCCTCTCGATGGGAGAGGGCGCGGACTACGTCAAGGTCTCCGAAGC
GAGCATTTCGGGATTTTCCTGGAATTCGACCTTGGAGGGTCTGCAGAGGATGGTCGCGCTCGACGAGCAGCAG
CGCGAGAGATTTGCCTTCACGACACTGTTGCCGGAATTCGGTACGGTCCGGCTGGCGGGTCTGGATGCCGATC
TGCCCGATACAGAAACCGAAGACGCCGAGAGCGACACACAGGATTCGGTGCCGAGGCGCGTTCGCTTCTCGCT
CAAGGATTACGAGTTGGCGCTCACGAAGCCACGCAACGGCATTCCGACCGACATCCGCATCAGATATCAGGAT
CTGACCCTGCCGATCCCGGAGCATATGCACGACGAGCCGTTCGCGCAGCTTCGCAACCTCGGTTTCAATGAGG
TGGTCCTGTCGTCCAACATCGAAGCCGCCTGGGACGAGCCGAACCAGACTCTGGTCATCAAGGATATCTCCGC
ACGTGGAAAAGATATTGGCGGCTTTTCCCTCTCCGGCCTGCTGGGGGGCGTGAGCGGCGACTTCTTTTCCGGC
GACGAGGCGAAGATGCGGGCAGCGCTGTTCGGCCTCACCGCCCGTGAAGCAAAGCTGAAGATCGAGGACAAAG
GTCTCGCCGCCAAGGCCATAAAGGCCTATGCCGAGCAAAACGACATGACGGAGGATCAGGCGCGCGGCATGCT
CACGATGACGGCTTCGGTGGCGTTGCAGCAGTTTGCCGCGCAGGAGCCTCGGTTGCAGGACGTGCTGGACGCA
GCTTCGCAGTTCATTGCCAAACCGGGGATGTTCACGTTGACGGTCAAATCCAAATCCGCAAACGGAATCGGTG
CGCTCGAATTCGCGGTTGCTTCGCAAAATCCGCTCTTGCTTCTCGACAGGGTCGACTTCGAGGCGACGGCGGA
ATAGATGATCACCTCGTTTCTCGTCAAATCGCTGGATCGGACGATCGTCGTCGCTGTCGCGATCCTGCTGGCG
CTGGCCGTTATCGTTCCCGCGCTCAACCTCCTGACCGCGCCGGAACACCCGCTGCATGTGCCGACCTATCTGG
TCTCGCTCTTCGGCAAGTACCTGACCTATGCGCTCTTGGCTCTGGCGCTCGATCTTGTCTGGGGTTTCTGCGG
CATCCTCTCGCTGGGGCATGCGGCCTTCTTCGCGCTCGGCGGCTACGCCATGGGCATGTATCTGATGCGCCAG
ATCGGCGCACGCGGCTCCTACGGCAATCCGCTGCTGCCGGACTTCATGGTCTTTCTCAACTGGAAGGAGCTGC
CCTGGTTCTGGTACGGCTTCGACATGTTCTGGTTCGCAGCGCTGATGGTGGTGCTGGTGCCGGGCCTGCTTGC
CTTCGTCTTCGGCTGGTTCGCCTTCCGCTCGCGCGTCAACGGGGTCTATCTCTCGATCATCACCCAGGCGATG
ACCTATGCGCTGCTGCTCGCCTTTTTCCGCAACGACATGGGCTTCGGCGGCAATAACGGGCTCACCGATTTCA
AGGACATCCTCGGCTTCAACATCCAGGCCGACGGAACGCGTGCGGCACTCTTTGCAGCCTCCGCGCTGGCGCT
TGCCGTTTCGCTCGTCGTCACCTCGGGCATCGTCCGCTCGAAATTCGGCAAGGTGCTGGTGGCGCTGCGCGAC
GCGGAAAGCCGCACACGCTTTCTCGGCTATCGCGTCGAGCACATGAAGCTCTTCGCCTTCACGGTCTCGGCAA
CGATGGCGGGCGTCGCCGGCGCGCTCTACGTGCCGCAGGTCGGCATCATCAATCCGGGCGAGTTCGCCCCCGC
CAATTCGATCGAGGTGGTCATATGGACGGCCGTCGGCGGGCGCGGCACGCTGATCGGTCCGATCGTCGGCGCG
ATCCTCGTCAACGGCGGAAAAAGCATCTTCACCGCCGCCTTCCCCGAATTCTGGCTCTTTGCGCTCGGCGGGC
TCTTCGTCCTCGTCACGCTTTTCCTGCCGAAGGGCGTGGTCGGCACGGCGCAGAACTATCTCGCAAGCCGGCG
CGCATCACACGCGGCGGCCCGGAAGGAAAGCGAGAGCGAAAAAGCGGCGAGCCTTGCGGACGCCGAAACGGTG
GCCGCGGAGTGAATGACGACATCAGGAGCGGACGGAACGAAGGAGCGGCGCCCTGTGATTGCGCTGGCCATGG
GCGACCCCGCGGGAATCAGCCCGGAACTGACGGCGCGGCTGCTGGCCCTTTCCGACATCCGCGACGCGGCGCA
CATCATCGCCATCGGCGATCGCCGCATCCTCGACGAGGGCGCCCGGGTGGCCGGCGTCACGCTGGATCTGGAA
GCCGCTTCGCTCGATGCGCTCGACAACGCCGGCACCGCCCGCCACGTCTTCGTGGATCTCGCCCATCTCGATC
CGGCCGATGTGGTGCGCGGCGAGGCGACGCTGGCCGGCGGCACCTTCGCCACACGCAATTTCCGCACCGCGCT
CGAACTCGCCCATGCGGGCAAGGCCGATGCCGTCTGCTTCACGCCCTTCAACAAGAAGGCGATGCGCTTTGCC
TATCCCGGCTATGACGACGAGATCCGCTTCGTCGCCGACGTGCTGTCCTTCACCGGAAAGGTTCGTGAATTCA
ATGTTCTCGAAAAGGTGTGGAACGCGCGGGTGACGTCGCATATCCCGCTCAAGGACGTGGCATCCCACCTGTC
GGTGGAGGCGATCCTTGCCGAACTCAAACTGACGCAGGCCTGCCTCAAGGATGCCGGCTACGAGGAGGCGAAG
ATCGCGGTTGCGGGACTCAATCCGCATGCCGGCGACGGCGGCAGCTTCGGCATGGAAGAGATCGACATCATCG
AGCCTGCGGTCGAGAAGGCCAGGGCGCTCGGCTTTAATGTCGAGGGGCCGTTCCCTGCCGATACGGTCTTCCT
CCGGGCACTGAAGGAAGGCTTCAACGCGGTGATGACCATGTATCACGACCAGGGTCAGATCGCCATGAAGATC
ATCGGCTTCGACAAGGGTGTGACGATGATGGGCGGACTGCCGTTCCCGCTCTGCACGCCCGCCCACGGCACCG
CCTACGACATCGCCGGCAAGGGTATCGCCGATATAGGCGCAACACGCGAGGCGATCCTGCTTGCAGCCCGCAT
GGCGAAGAAAAAGAGGGCGCTTTCGGCGGCTGCCTGAATGTGGGGAGAAACCGGTTGGGGCATGCCGACGTCG
CTCGCCCTGCACCTGGCGCTCGCCTTCTTGCTGCTGGTTCGGCTGCCGGAATTGTCTGCGCCGGCAAAGGAAC
AAAGCGTCAGCGTCGAGCTCGTTCCGCAGCCGAAGAGTGCGGAAAAGCCCGAGCAAAAGCCGAACGCGGCCAA
GAGCGAGCAGGCGCGCCCTCAGCCTGAGGCGTTCGAGTCGGCGGCAGCGCCGGCCGAAAAGGAAAAGCCGCCT
CGGCCCGAGTTGCCTCCGGCCGCGCCGAAGAAGACCGAAACGCCGAGCGAGACGCCGGAGGCCTCGCGGCCCG
CCCCGGCAAAGACGGAGAACTCCGCCAGCGAAAAGCCGGCGGAAAGCAAGACAACGCTTGCGGAATTGCGCGT
CGATGCCCGGAGCGCAGCGAACGAGGCGGCCGATGCTGCCGCGGCGCCGCCGGCTGCAGCTGTTCCCCAGGAG
AAACCGGTTCTGGAGGAAGCAAGGGCGGAGCCGACGCAAAAGCCGGCCGAGGCGAAGGCCGAGCGCCAGTCGA
GCCAGCTCGTCCAGGCGAAGGAACTCTATTCTGAAAACGCCCTGTCGGACCCGCGCGTGAAACAGGCGATCGG
CAGGCTGCCGCCGAAAAAGCGGATCGTACAGCTCTGCAGCATCGAGGCGCTCGAGCAGGTGCGGCGGGAAATG
CCCGGCGCCTTTCCGGACATGCTCGTTCCCTTCGGCCCTTCCGGGGGCTTCATCTCGGAAACGGGACTAAACG
CCGATGGCGGCGCCTTCCGCAGCCGGTCGACATGGTACGCCATCGACTTCAAATGCGAAGTGAGCCCCGAAAC
GACATCGGTCACTTCCTTCAGCTTCGCGATAGGCAAGGCGATCCCGAAGAGCGAATGGGGTGCGCGACAACTG
CCGAGCAATTAAATGCAAGACGCATCGATACCGCGTCCGCGGATTTCCAATGCCATAGACAACGTAGCCCGCG

CGGTGGGCGGCCTGGCCATCGCCACCTTCACCGCCATCATCGTCTACGTCGTCATTTGCCGGTATGCCCTTTC
CTTCACACCACGCTGGTCGGAGGAAATCCCGCGTCTTCTATTGGTTTGGGTGACGTTCATCGGCGCGGTTTCC
GCCTTTGTCCGCAACACGCATCTTTGCGCCGGCCTGACGGACCTGCTTGTTGCGCCCGGCTGGTTCCGTTCCC
TCCTGGCCGTGCTCGCGCGTCTGGCATCCCTGCTGTTCCTGGTCGTCGTCATCTGGTCCGGATGGAAGATAAC
GGTGCTGACATGGTCGCATGAAACCACGGTCCTAAGCTGGCCCGCGGGGCTCGTCTATCTCGCGCTGCCGGTC
ACGGGTGCCATCGCCTTCGTTGCCCTCGTTGCCGCGGAACTGCGCAAATGAATGGACAACATCATTCTTGAAA
TGCGGGGCATTACGAAGACGTTTCCGGGCGTCAAGGCCCTGGACAATGTCTCGTTCAAGGTCCGCGAAGGCGA
GATCCACGCGCTTGTCGGGGAAAACGGCGCCGGCAAGTCGACCTTGATGAAGGTGCTGAGCGGAGTCTATCCG
GCCGGCACCTATGAGGGCGAGATCCATTACGACGGCGAGCTTCGCCAGTTCAGCACGATCTCGGACAGCGAAG
AGCTCGGCATCATCATCATCCATCAGGAACTGGCGCTCGTTCCGCTGCTTTCGATCGCCGAGAACATCTTTCT
CGGCAACGAGGTGGCCGAAAAGGGCGTGATCCACTGGCCGCAGACCTTTGCCCGCACCCAGGAGCTCCTGAAG
AAAGTGGGCCTGAACGAGTCCCCCGCGACGCTGATCACCGATATCGGCGTCGGCAAGCAGCAGCTGGTGGAGA
TTGCCAAGGCGCTTTCGAAGAAAGTCCGCCTGCTGATCCTCGACGAGCCGACCGCATCCCTCAACGAAAACGA
CTCCGACGCGCTTTTGAAGCTGCTCATGGAGTTCCGCAGCCAGGGCATGACGTCGATCATCATCTCTCACAAG
CTCAACGAGATTAAGAAGGTTGCCGACCAGATCACCATCCTGCGCGATGGCGGAACGGTAGAGACGCTCGACT
GTCACAAGGAAGACATCAGCGAGGACCGGATCATCAACGGCATGGTCGGCCGGGCCATGGAAGACCGCTATCC
GCCGCGTGAACCGAAGATCGGTGACACGCTGCTCGAGGTGAAGAACTGGAACGTCTACCATCAGCATCACCGC
GACCGGCAGTTCCTGCATGACGTCAGCTTCAAGGTTCGCGCCGGCGAGGTCGTCGGCATCGCCGGGCTGATGG
GCGCCGGCCGCACCGAGACGGCGATGAGCGTTTTCGGCAAGTCCTGGGGCCACAAGATCACCGGCGAGGTGAC
GATGCATGGCCGGCCGGTGGACGTGAGCACGATTCCCAAGGCCATCAAGGCAGGTCTTGCCTATGTAACGGAG
GACCGCAAGCAGCTCGGCCTCGTGCTGATCAACAACATCATGCACAACACCACGCTCGCCAATCTGCATGCGG
TCGCATCGAACGGCGTCATCGACGAGCGCAAGGAACGGAAGGTCGCCGCCGAATATCGCTCGAAGCTCCGCAT
CCGTTCCCATTCGATCTATCAGGAGACGGTCAACCTCTCTGGCGGGAACCAGCAGAAGGTCGTCCTGTCCAAG
TGGCTGTTCACCAATCCCGAAGTCCTCATACTCGATGAGCCGACGCGCGGTATCGACATCGGCGCGAAGTATG
AGATCTACACCATCATCAACCAGCTCGCGGCCGAGGGCAAAGGCATCCTGATGATCTCGTCGGAGATGCCGGA
GCTGCTCGGGACCTGCGATCGCATCTATGTCATGAACGAGGGGCGCATGGTGGCCGAGCTTTCCAAGGAGGAA
GCAAGCCAGGAATCGATCATGCGCGCCATCATGCGTTCAGGGGAGAAACACTAAATGATCAATGCGGACAGCA
GCCCGCGCCAGCCATATTCGACTTATCATCAATGGTATGCCAGTCAGGACAGAAACCGGCTCATCGCGAAATC
GAAGGAAGCCGAAAAGATTTTTCGTAAGACGGGCATCACCTTCGCAGTCTACGGACACGCGGATTCCTCCGAA
AAACTGATCCCCTTCGATCTCATACCCCGCATCATCTCCGGGCGCGAATGGCGCCGGCTCGCCCAAGGCATCG
AACAGCGCGTCATCGCGCTCAACGCCTTTCTCGACGACATCTACCACAAGCAGGAGATCATCCGCGCCGGGCG
CATCCCGCGCGAACTGATCGAGAAGAACGACGCCTTCCTGCCGCAGATGATCGGCTTCCGGCCGCCGGGCGGC
GTCTACACCCACATCGTCGGTACCGACATCGTACGCACCGGCGAAGACCAGTACTATGTGCTGGAGGACAATG
CCCGCACGCCGTCCGGCGTCAGCTACATGCTGGAAAACCGGGAAACCATGATGCAGATGTTTCCGGAACTCTT
CCACCAGAACCGCGTGCGGCCGGTGGAGAACTATCCCAATCTGCTTCGCCAGAGCCTTTCCTCGCTCGCTCCG
GCCGGCTGCGAGGGCAAGCCGCGCGTTGCCGTCCTCACGCCCGGAATCTACAACTCCGCCTTCTACGAGCACG
CATTCCTCGCGGATATGATGGGCGTCGAACTGGTGGAGGGCTCGGACCTGCGCGTCATCGACGGCAGGGTAAA
GATGCGCACGACACGCGGCTACGAGGCGATAGACGTGCTTTACCGGCGCGTCGACGACGATTTCCTGGACCCC
CTCACCTTCCGCCCGGATTCCGCCCTCGGGATACCGGGGATCATGGATGTCTATCGCGCCGGCAATATCACCA
TCGCCAATGCCCCCGGCACCGGCATTTCCGACGACAAGGCGATCTATTCCTACATGCCGGAAATCGTCGAATT
CTATACCGGCCGCAAACCCCTCCTCGAGAACGTGCCCACCTGGCGCTGCTCGGAACCGGAGAGCCTCAAATAC
GTGCTCGAGCATCTCGCCGAGCTCGTCGTCAAGGAGGTTCACGGCTCCGGCGGCTACGGCATGCTCGTCGGCC
CGACCGCGACAAAAAAGGAATGTGCAGCCTTCGCGGAGAAGCTCAAATCCCGCCCCGGCAACTACATTGCCCA
GCCGACATTGTCGCTCTCGACGGTGCCCATCATGGTCAACAAGGGCATTGCGCCGCGCCACGTCGATCTGCGT
CCCTATGTGCTCGTCTCCGACAAGGTTCAGATCATTCCGGGCGGCCTGACACGCGTGGCCCTGAAAGAAGGCT
CCCTGGTGGTGAATTCCAGCCAGGGCGGCGGGACCAAGGATACCTGGGTGCTGGAGGACTGAGTGAAGACGCT
CAAATTCCTGGTTCTGGCCGGCTCCCTCGTCGTGGCAGGCGGAGGAGGCTACGTAGCCGGCACAGGCGGCCTT
ACCCAACAGTTCTTCCTTGGGGCAACTGCACGAGAACCCACGCCGCCTGCGGCAAAGCCGGCGGGCAAGATCA
TCTACTACCGGCACCCGGACGGGGTGCCGGAATATTCGGCGGCGCCGAAGCAAACCGCGGACGGGCGCCCGTT
CGTCGCAGTCCGGCAGAGCGAGGATGTCTCTTTCGAGCCGCTGAAGGCGGTTGCCGAGGGCCCCGCCGCCGGC
GAGCGAAAAATCCTCTACTACCGCAACCCCATGGGGCTGCCGGATACCTCCAAGGTGCCGAAAAAGGACTCCA
TGGGAATGGATTACATTCCCGTTTACGAGGGTGAGGAGGCAGACGGCTCGACCGTGAAGGTGTCTCTCGGCAA
GCTCCAGCGGACGGGTGTGAAGACGGCATCGGCGGAGCGTGCCGTCATCGCCCGAAGGGTTCGCGCGCCCGGC
ATGATCGCCTTCGACGAGCGGCGCATCAGGATCGTCTCGACGCGGGTGGATGCGTTCATCGAAGACGTCGCGG
AAATCACGACCGGCGACCGGGTCCAGGAAGGCGAGAACCTCTTCCACTACTATTCGAAGGACGTCGCCAGGGC
GGGAGCGGAATACGCCACGGAACTGCGCGGCGGCGTCAAGCCGGGCCTCGAAGTCGGGGGAGCCCTGCAATTG
CGCAATCTCGGCGTGCAGGAAGAGACGATCCTTTCGATTGCCAGGGATCGTACGGTCCCGCGGAGCATTGCCT
ATCTTTCGCCGAGCGACGGCGTCGTCCTGGAACGCAATGCCATCCCGGGCATGATGGCCAAGGCCGGCGACGT

CCTGTTCCGCATCGCCGACACCTCCAGCATATGGGTCCTGGCCGACATTCCGGAGTATGATGCCGCAGCCGTG
AGAAAAGGTGCTCCGGCGAAGGTCACAGTACGAAACCTTCCGGGCAAGGTCTTCGAGGGCAGCGTCGATCTGA
TCTATCCCGAGCTTCGCTCGCAGACGCGGACGGCGAGGATCCGGATAGAGCTCCCCAATCCGGCGGGGCAGCT
CCTCGCCAACATGTATACCGAGGTCGAGATCGTTACCGGCAAACCGGAGCCCGTCATGACGGTCCCGAACAGC
GCCGTCATCGACACCGGCAACCGCCAGCTCGTCTTCGTGGACAAGGGCGAAGGCCGCTTCGAGCCGCGCGAGG
TCGCGCTCGGGGCACGCGACAGCGAAAGGACCGAGATCACGAAGGGGATCGAAGCCGGCGAGAAGGTCGTGAT
TTCAGCCAATTTCCTGCTCGACGCCGAAAGCAACCTCAACGCAGCGCTGAATGCGCTTGCCTCGGCGGAGGCG
CAGCCATGAATGGCACAGGCATCCGACAGCATCCGCTTCATCCTGAACGACTCCGAGATCGCCCTTTCCGACG
TCGCGCCGACGTCGACGCTGCTCTCTTTCCTGAGGCTGGAGCGCCGCCTCACCGGCACCAAGGAAGGCTGCGC
GGAAGGCGACTGCGGCGCCTGCACGGTGCTCGTGGGCCGGCTCGCCGAGGATGCCAGCGGAGCGAAAAAACTC
GTCTATCAAAGCGTCAACGCCTGCATCCGCTTCACAGGCTCGCTGAATGCCACCCATGTGGTGACGGTCGAGC
ACCTGGCTGCCGCAGACGGCACATTGCATCCGGTGCAGCAGGCACTGGTGGATTTTCACGGCTCCCAATGCGG
CTTCTGCACTCCGGGGATCGTGATGTCGCTTTACGGGCTCTGGCTTGCCGATGAAAACCCGAGCCGCGCGGCG
ATAGAAAAGGCGCTGCAGGGCAATCTCTGTCGCTGCACCGGCTACGAACCGATCGTGCGCGCGGCGGAGGCTG
CGGCCGCGGAGCGCCCGGCCGCTCTCATCGACCCGGTCACCCGCACGCGGGAAGCGGTTACGGCGCGCCTTCA
TGCGATGGGGCAGACGGAGACGATCGTTGTCCGCAGCGGCGCGGACTGTCTGATCGTACCCGTCGACGCTGCG
GACCTGGCAGGTGTTCTCGCTGATCATCCAGGCGCGACGATCGTCGCCGGTTGCACCGATGTCGGGCTCTGGG
TGACGAAGCAGATGCGCTCGCTCAATCCGGTGGTCTTCATCAATGGCATTGCCGAACTGCAGCGGATCGAAAG
CTCGACAGCAGGCCTGACGATCGGTGCAGGCGTCAGCTATACGGCGGCGCACGAGGCCCTGTCTTCAGCCTAT
CCGTCATTCGGCCGGCTGCTCGACCGCATCGGCGGCGACCAGGTGCGCAACATGGGCACGATCGGCGGCAATA
TCGCCAATGGCTCGCCGATCGGCGACAGCCCGCCGCCCCTGATCGTTCTCGGCGCGACGGTTACCCTGCGCTC
GAAGGACGGCCAGCGGACGCTGCCGCTCGAGGATTTCTTCATCGCCTATGGCAAGCAGGATCGCAGGCCCGGT
GAATTCGTCGAGAGCCTCTTCGTCCCCGCTCTGCCCGCGGACGAGCGCTTTGCCGCCTACAAGATCTCGAAGC
GCCGCGACGAGGACATTTCCGCCCTGCTCGGCGCCTTCCGCATCTCGCTCGACGGCGACCGGGTGAAAACCGC
ACGGATAGCCTTCGGCGGTATGGCGGCAACTCCGAAGCGGGCGAAGACCGTCGAAGCAGCGCTGATCGGTCAG
GCCTGGACGGAGGAGACGGTCCGTACTGCGCAGGCCGCCTTCGAGACCGACTATCAGCCGATCACCGACTGGC
GGGCCACCGGTGCCTACCGCATGCTTGCGGCAAAAAACCTGCTGATGCGCTTCTTCCTCGAGAGCATCGGAGA
GACGGTGCAGTTGCAGCGGTTCGAGGAAGTCGCATGAATGTCGTTCCTGAGAAGCGTGCGGCCCAGCAATCTG
ATCATTCTGACAGCGCTGCCGTTCATCATCTATCTTTTCGCCTCCTCCGGAGACTACCAACGTTCGCTGAGAG
CCATTCTCGGAGTCGAGAACGGCTCGTCCGCCTTCGTACCGGGTTTCCTTGCTCTGCTCGTCGCCTTTGGCTC
GGGTGTCGCCGCTCTCGTCTTTTCCCGAGCCAAGGCGCCGCGGCCGCGGCTGGCGCTCGCTGCCGCCGTACTC
AATCTCGCTGCGGCGGCCACCGTGCTTCTGAGCGACGCCGTTCACCCCTACCTTGCGTCGGTCGTCGCCAATG
CCGTCGATTCCTTCACGTCCGATCTCGTTGTGAAGGGGCTTAGCCCTCGGCAACTCACGGACGCCGCGGACAT
GACCCTGCGCGGCATCGAGGCCGTGCTTTTTCCGGCCTATTTCGCGCTGACACTGCTCGGCTTCATGCTTTTC
TTCGGTGCCGGCAAGGCATCCACGCCGGAAAGCATCATGCGCCGCTTGGGAGCGTTAAGCATCGGCCTCGTCA
ACGGTGCGGGGCTCCTGTTCATCCTCTTCTTTGCGCATATCGCCTTTGCCGCCGGCGTGGCGACGACGGTTCG
CGCAGCCATTGCCGCCTATCTTCTCGCCGCGATCATCGGCCTCATCTGGGTCGGATTGCTTCAGTTGCATTAT
ACGGCCCGCACCAGCATCATCTTCGCGCTCGCGACCCTCGGCCTGACGCTCGTCGCCGCCTGGTTCCTGTTGC
AGCCTCGCACGGCCTATGTGCTTGCGGGGCGGCTCGACGGCACGGTCGCGATCGTCAGCAACACGCCGTCCGG
CCTCATAGGCGCGGTGCGCTTCGGCCAATATCCGGGCGCGCCCGGTGAAGAGGCGGCGGTGCGAACCTTCCTT
GGCCCGGCGGAGGCAGTCGCGGCGATGGGAAAGAACGAGGTGACGGCGGCGCTTCTGCCGTCGGCAGCCGTAC
CCGGGGGCTTGCCGCTCCTTTGGGAAACGGCGGCTTTGAACGATCGCGACCGGGCCGCAGGCATCACTTTCGC
CGTCCTGGCAATTATCCTCGGTCTGCTCACCTTCGGCGGCTATCTGCACCGCCGTCATCCCCTGGCGATCGGC
TCGGAGTTCGTCGTCGACACGATTCGCGGCATTCCGATGCTGGTGATCGTGCTCTATGTCGGCCTGCCCTTAA
GCGGGGCGCTGAAGGATGCGACACAAGGCGTGCTCGATCCGCCGAACCTGATGCGCGGCATCGTCGCCATGGC
GCTCGCCTATTCCGCCTATCTGGCGGAAATCTTCCGCTCCGGCATCAATGCCATCCCGATCGGCCAGATCGAA
GCGGCGCGCAGCATCGGGCTCAACCGGTGGCAGACGGCGCGGCTGGTGGTCCTGCCGCAGGCGTTCCGGATCA
TCATTCCACCGCTCGGCAACGAGCTGATCGCGATCCTCAAGGACACGTCGCTCCTGTCGATTCTGTCGATCCG
CGACATCACCCAGCGCATGCGCGAGTTCCAGTCGGCGAGTTTCCTGCCCTTTGCGCCCTATAACTCGGCAGCC
ATCTTCTACATCTTCCTGACGCTCGCGGCGGCGAGCCTCATCAACATGATCGAGCGGAAATATGACATCAAGC
ATCGATAGATGAACCAGATGTCCAAGATTTCGGTGACGGTGAATGGCCGCCGCTATCCGTGGCCCCGCGTACC
GGCAATTGCGGTCTGCCTCGACGGATGCGAGCCGGCCTATCTCGATGCAGCGATCGACGCCGGGCTGATGCCC
GCGCTGAAACGCATCAAGGAACGGGGCGCCGTGCGTCTGGCGCACAGCGTCATCCCGAGCTTTACCAATCCCA
ACAATCTCTCGATTGCAACCGGCAGTCCGCCGGCGGTGCACGGAATTTGCGGGAACTACCTCTACGAGCCGTC
GACGGGCGAAGAGGTGATGATGAATGATCCGAAGTTCCTGCGCGCGCCCACCATTTTCCAGGCTTTCTATGAT
GCAGGCGCCCGGGTCGCGGTCGTCACGGCGAAGGACAAGCTGCGGGCGCTGCTCGGAAAAGGGCTCCGTTTCG
ACGAAGGTCGCGCCGTCTGCTTCTCGTCCGAAAAATCGGACAAGGCGACCCGCGCGGAGCACGGAATCGACAA
TGCTTCCGCCTGGCTCGGCCGGCCGGTTCCCGAGGTCTATTCGGCAGCGCTTTCGGAATTCGTCTTCGCTGCG

GGCGTCAAGCTGCTCAGGGAGTTTCGTCCCGACATCATGTATCTGACGACGACCGACTATGTTCAGCACAAAT
ATGCTCCCGGCGTCCCGGAAGCCAATTCCTTCTACGAAATGTTCGACCGGTATCTCGCCGAACTGGACGGGCT
CGGAGCAGCCATCGTGGTCACCGCCGATCACGGCATGAAGCCGAAGCACAAGGCGGACGGCTCACCCGACGTC
ATCTATGTTCAGGACCTGCTCGACGAATGGCTGGGCAAGGATGCGGCCCGCGTTATCCTGCCGATCACCGACC
CCTATGTCGTGCATCACGGCGCGCTCGGGTCCTTTGCCACCGCCTATCTTCCGGACGGATGCGACCGCAGCGA
GATCATGGCAAGGCTGAAAGCGATCCAAGGCGTCGACGTCGTGCTCGGCCGCGAGGAGGCGTGCCGGCGCTTC
GAGCTGCCGGAAGACCGCATCGGCGACATCGTCCTCGTCTCATCCGAAAACAAGACGCTCGGCACCAGCGAGC
ACCGGCACGACCTGGCCGCACTCGACGAACCGTTGCGCTCCCATGGCGGGCTGACCGAGCAGGAGGTTCCCTT
CATCGTCAACCGCGTGCTGCCCGAACTCCCGAACGCTCCCCGGCTGCGCAACTTCGATGCGTTTTTCTACGCG
GTCACGGCGGCGGCCGAGGCCGGTGCGGAGGGCGGGCTATGAATGACTCTCGTTGCCCTCTCGCTGCATCTCG
CCGGCGCGACCGTACTGCTGCTCTACGCGGTGCATCTCGTCCGCAGCGGCGTCGAGAACGCCTATGGCACGTC
GTTGAAGCGTGTGCTTGCAAGGGGAGGGGAGCGACGCATTTACGCCGCCGCGAGCGGCACGGTCCTTGCAGTC
CTGTTGCAAAGCTCGACCGCCGTGGCAATGCTCGCCTCCGGTCTTTCGGCGAGCGGCTATCTTACCCTGAACA
CCAGCCTTGCCGTCCTGCTGGGAGCGGATCTCGGCTCGGCGCTGGTGGTTCGTGTTCTTTCGTTCGACCTGGG
CTGGCTGTCGCCGCTCCTGCTTGCGGCCGGCGGGCTGCTTTACCTGAAGGGCAGAAGCGACAAGCCCCGCGAA
CTCGGCCGCATGCTGCTCGGCATCGGCTTCGTCCTCATGTCGCTGAAGCTGATCGGCGAGGCGACCTTGCCGT
TGAAGCAGAGCGACGTGTTGCCCGCCGTCGTCGGCTATCTCGCGGAGGACCAGGTGACGGCCTTCGCGGCGGC
GATGCTCTTCACCTGGATCGTGCATTCGAGCGTCGCGGTCCTGATCCTCATCCTGGGTTTTGCCGCACAGGGA
CTTTTGCCGGTCGAGGCAGGTCTGCCGCTGATCCTCGGCGCCAATCTCGGCAGCGGGTTGATCGCCATGTGGC
TGACGCGCGGGTTTTCAGCCGAGGCGCGGCGCGTCGCCGCCGGCAATCTCCTCTTCCGGGCTTTCGGCGCCGC
GATCGCCTTGCTGGTGATCGGCCCCTTGTCGGCAGAACTTCAACATCTTGGCGCCACCGCGGCGGAGCAGCTC
GTTCATTTTCATCTGGCCTTCAACGCCTGCCTCGTGATCTTTTGCCTGCCGCTCTCGCAGCTTGCCGCAAGCG
CCGTTACGTCGCTCATCAAATCGGATCTCAAGAGCTCCGAAGACAGCGACACGCTTGCGCGACGAATCAGCGC
GCTCGACCAGGCTCTGCTCGACAGCCCCGACGCGGCCCTCGCCGCCGCGACGCGCGAGGTGCTGTTCATGGGC
GAGGTGGTCGAGCGGATGCTCTGTCCGGTGATGGAGCTTCTGGAGCATCCGAGCCCGGCGAAGATCGAGGAAA
TCCGCCGGCTCGACGGATATGTCAACCGCGCCCACCGCGACATCAAGCTCTATCTCGCAGCAATCCATCGCGG
CGTGCTCGACGAGCGTCAGTCGCGGCGGGGTGTAGAACTCATCGACATCGCCATCAATCTCGAACATGCCGGC
GACATCGTATCCAAGACCCTGGTCGAAATCGCCCGCGACAAGCTGGAGAACGGCAACAACTTCTCCGACGAGG
GCTGGACCGAGCTCAAGGCGCTTCACGCCAGCGTGCGCGACAACATGCGTCTCGCATTCAACCTGCTGGTGTC
GGATGACCCGGTAATGGCGCGAAAGCTCGTCCGGGAGAAGGAGCGGGTACGCGCGCTCGTGCGGGAAAGTCAC
GAACGGCATCTGGCGAGATTGCGGGCCGGGCGCGCGGAAAGCGTCGAGTCCAGCAACATGCACCTCGAAGTCG
CGCGGGCACTCAAGGAGGTGAACTCCCTCCTGGCCACGCTCGCCTATCCGATCTTGAAGCAGCGAGGGGATCT
GCTCGAGAGCCGGCTTGCGCTTGTCAAAGGGTGAATGAACATGCCCCGCATTCTCAAAACTCTCGTCGCCGGC
ACCGCGCTCACATTGCTGGCGTCCGGCGCCTTCGCCGACGGCATCGGCGCATCGCTCCTGACCCAGCAGCACC
CGTTCTACATCGAGCTCGCCGAGGCGATGAAAGCCGAAGCCAAGGAGAAGAACGTCGCGCTCGAGGTGGCGAT
CGCCAATCAGGACCTCAACAAGCAGCTCGCCGACGTCGAGGACTTCATCGTCAAGGGCGTTGATGTGATCGTC
ATTTCGCCTGTCGACAGCCAGGGCGTACGCGCCGCCATCGCAAAGGCGGAAAAAGCCGGCATCAAGGTGATCA
CCGTCGACGTTCCCGCTAATGGCGCGACGGTGACGTCGCATATCGGCACCGATAATTTCACCGGCGGCGTGAA
GGCGGGCGAGCTCATGGCCGAGGTTCTCGGAAACAAGGGCAAGGTCGCCATTATCGACTATCCGACCGTTCAG
TCGGTGGTCAATCGCGTCAACGGCTTCAAGGAAGCGATCGCCAAGCATCCGGAAATGGAGATCGTCGCGACGC
AGACGGGCATCACCCGCGCCGAAGCCCTCGCTGCCGCACAGAACATGCTGCAGGCCAATCCCGACATTACCGG
CATTTTCGGTTTCGGCGACGATGCGGCCCTTGCCGCGGCCGTTGCCGTCAAGGCCGCCGGACTGGAATCGCAG
GTCAAGGTCATCGGTTTCGACGGCATGAAGGAAGCACGCGATGCCGTCGATGGCAATCCGGTGATGGTCGGCG
TGATCCAGCAATTCCCGGACCAGATGGGCAAGCAGGCGATCGACACCGCCGTAAAGGTCGTCGCCGGCGAGAC
GGTACCGGCCGAGCAGCCGATCGTACCGGGCGTCTATACCGGGAAATAGATGAGCCTGAATTCCGCATCGACT
GCCGCGACCGTCACCACCCCGGTCCATTCGTTGCTCGTGCCGTTTCCAGTCGTCTGTTTTACGCTCGCCCTTC
TGACTGATATCGCCTTCTGGCAGACGGGACATCTCATGTGGCAGAACTTCTCCGCCTGGCTGCTCTTTGCCGG
TATCATCGCCGGCGTGATCGCCGGTCTAGCCAGTGTGTTGGAGTTCCTGTTTCGAAGGGAGCGACGCAGGCAA
GGGGCGGTTTGGCTCCATGTCGTCGGCTACATCCTCGTCTTCGGCCTCGCCTTCGTCAACAACCTCGTCCATG
CCGCCGATGGCTGGACGGCTGTGGTGCCCTACGGGTTGATCCTCTCCGCGGCGACGGTGCTTCTGACGATCCT
CTTCGCTTTGCTTGGCCGCGCCACCCTCTATCGCACGCAATCCGGAGTGAGCGGTTATGAATAGATGTCCGGA
AATAAGGAAAACTCGGACCTGATCGTGGTGGCAATGCCGCTTTATGGCCACGCAGCCCTCGTCCTGGAGGCGA
TCGAGTCCGTGCTCGCTTCGAAGCTTGCCGGGTGCCGGGTGGCAGTGGTGGTGTCCGTCGATGGTGATCCGCG
ACAGGAGACATTCGATCAGTTGCTGCTCTATGCGGCGGCCCATCCTGCGGTTCATGTGCTTTTCGGGGCCAAT
GCCGGGCCGGGCGGGGCGCGCAATCGCGCGATCGACTACGTGCTCGCGAATCTGCCGGAAGCAGAGGCCGTCT
ATTTTCTCGATGCCGACAATCGCGTGCTGCCCGGGACCATCGAGACTCTCTACCGGCAACTGCGCGCCAGCGG
CTGCGGCTGGATCTATACCAATATCGACACGTTTTCGGTGAGCTGGCGCGCCCATTACGGCAATCGCTACTCG
CGGCTTCTGCACTGCATCACGGACAATATCTGCGACACCGGTTCGATGATCTCGATCGACGTTTTCCGGGCGG

GGGTCCGCTTCGACGACGACAGGCAGAACGGTTTCGAGGATTGGGAGTTCTGGCTGTCCTGCATCGAGCACGG
CTTCGTGGGGGCGCCGTGCCACGACACGACCTTCGAATACCGGCTGAGGGCGGAAAGCCGCTTCAAGGAGGCG
AACCGCGATCGCGCCGCCTCGGTGAGCTTCCTCAGAAAGCGCCACCGGGCGCTGTTCCAGCGGCCAATGCTGG
TCGATTTCGAGCACGAGGAATGCCCGCGCTATCTTTTCGCACGGACGGAAGATGCCGCCATTTCGTTCTTTAC
GGATCCGACCAAGACGCCCAGGCGTCTTCGCCTCGACGATATCATTCCAGCCTTCTGGGCGAGCATCGGCGAG
CCTGACAACGTTCACTTCCCGCCCTTCCTGATCGCCGGAAGCGGTGCCACGCTCGATCTGCTGCTGCGCTCCC
GGATGCTGCCGAACGTGCTCTCCCATCTGGAGCGCCTGAGCGAGAAGGCCAACGTGGTCTTCGTCCAGCTCGG
CAACGATGCGGCTCAGCGCAAGATCGAGCCGGTCTTCCTGGAGGCCGGCGCCCAGCACAACGCACCGGCCGAC
CTCATCTTCCTGTCCACTTCGCTCGTGCGCGACGTCATTCATAACAAGGCGCTGGACTGGTTTGCCTCCATCG
GCAACCAGCAGGTCTGGCCGACATCGGCAATTCTGAAAGTCCGCTTCCCCTTCCCGAGAAGCCTGCCGCGCCG
CTCGCTCATCACGCCCCAGCAGGTGATGATCAATTGCGTCAATGCCATCGCGACCAGTCCGCTGCGGCGGACC
GCCGGCAAACGCTGGACCTGGCGTCCGGCACGGCTCGTTCCCTATTCCGACCTCCACAAGGCGCTTCGCCACG
AGATCGGTGGCTCGCCCGTGTTGCCGCTCGGTCATAGCGAGGGAAGCAGGAAGACCGCAGCCCTGCTTGTTCC
GAATGCATCCTTCGGCGGTGCCGAGAAGGTCGTATATGCGGCCTCGCGCGAGCTGAAGGCCGCCGGCTACGAG
ACCCATCTCTTCGTGCTCGGCACATCCAGGATGGATGTGATCGACGAGTTCGATCAGAGCTTCGACTATATTC
ACTTCTGGGACCAAGGCATTCCCGCCTGGGGCGGCTCCGGCTCGTTCCTGGGGCAGGACTTCATCGCCGAGGG
CCACGACGTCGATTGGGCTGCGCTCAAGGGTCAGCTTTCCGGCTTCGACCTCGTGATCAACAATCACGTCATG
GCGGTGCATCCGCTTATCGCGCGGTTACGCTCGGAGGGAACGCGCACCGCCTGCTACCTTCACGTGGTCGACA
ATACCTCCTTCAAAAGGCCCGCCGGTCAGCCCTTCGCGGCGATTGCCCACGAGCATTGCTACGATGCCTTCCT
GACCTGCTCGGAGCAGCTCAAGATCTATCTGCACAGCTTCGGCGTTCCCTATGAAAAGATCTTCGCCGTCCCC
AACGGCGCGAGCTTCTCCGTGCCGCCCAAGGTTCTCGCGGAGGTGCTGTCGGTCAGGCGCATCGAACGCAAGG
ACGATCGGCTTCGGGTCCTCTATATGGGTCGGCTCGATCAGCAGAAGGGGATCGACCGTCTCGCTGCGGCGTT
CGCCGAATTGCGGGCTTCGCGCGTGCCATTCGATGCCCGGGCGATCGGCGGGGAAATCCTCGCGGATGCCACC
TTGTCGTGGACGGATCGCCTGAAGGACCTCGGCGTCGAGGTGCGCCCGCCGGTGTTCGCAAGCAAGGACCTGA
TCAAGGCCCTCGGTTGGGCGGATGTCCTTCTGATGCCGTCGCGCTGGGAGGGCGCTCCCTTGATGATCGCCGA
GGCGCAGCAGCTCGGCTGCGTCCCGATCGCGACGGCAGTCGGTGCCGTCGACGAACTCATAACCGACGGCGAG
GACGGCATCCTCATCGAGGCCGCCGCCGATCCCCAGGTGGTCCGGGACATGGCAAAGGCCATCGAGGAGGTGG
CCAACAACCGCCAGATGCTCGCGCCCCTCATGGAGGGGTGCCTGAGAACGGCGGCACGCCGCTCATGGACGTC
CTCCTTTTCCGAGTTCCTCGGCTGGTGCGACCGTTCCGTGAACAATTCATCGCTTTCGCGCGCAACGGTCATC
CGTGGGCGCGAAGCATCCAATCCCGGAGTTGCGGCCGTGGGCTGAATGGCGGGAGAACTGGAATTCATGAGCC
GGGAGGTCGTTTCGGGCCGCCTGTCGAGGCGCGATTTTCTCGGGCGCGCGGCAGCGCTCGGCGTAACGCTCGC
CTCGGCCCAGGTGCTGCTCGGCAATGCCGCGCGGGCGAGCGGGCCCGTCAAGGGCGGCATATTGCGGGCCGGG
CTCGTCGGCGGCGAATCCACGAACAGCCTCGATCCGGCGACCTGGGCGAGCCAGGTGCCCTATACATTCGGCC
GCTGCTGGGGAGAGCAGTTGCTCGAGGTATCTCCGGCGGGCGAGATAGAATATCGGCTGGCTGAATCCTACGA
AGCGTCGAAGGACGCGAAGACCTGGGCGTTCACGATCCGCAAGGGGGTGACCTTCCATAACGGCAAGGAACTG
ACGCCCGCCGATGTCGTCGCCACATTCGAGCGTCATGCCGACCAGAATTCGAAATCGGCTGCCCTGCCCTTCG
TCCAGGAATTCGAGACCATCAAGGCAGACGGCGACAAGGTCGTCATCACGCTGCGCCAGCCGAACGCCGACAT
GCCGTTCATCGTCAGCGATTATCATCTTATGATCCAGCCGAACGGCGGCAAGGATGACCCGACGGCCGGCATC
GGAACCGGCCCCTACAAAGTCGTCGTCAACGAACCGGGCGTAAAGCATGTCGGTGAGCGTCAGGACGGCTATT
GGGGAGCGGCCGAGCGCGGCCATGCCGATCAGATCGAAATCACTGTCATCAATGACGCAACGGCGCGCACCGC
CTCGCTCCAGGCGGGCGGCGTGCACATCATTAATCGCATCGAGCCGAAGATCGTCGCGCTCGTCAAACGCATT
CCCGGAGTCACGATCCGCAACGTCCCCGGCAAGGGCCACTATGTCCTGATCGCCCATTGCAACACGGCGCCCT
TCGACAACAACGACCTGCGGCTTGCGCTGAAATACGCGGTGGACCGCGAGGAAATGGTGGCGAAGATTCTCGC
CGGATACGGCACGGTCGGCAACGATACGCCGATGATCAAGGGTTACCCGCTCTTCGACGACGATATCGAGCAG
CGCGTCTTTGATCCGGACAGGGCGAAGTTTCACTTCAAGAAATCGGGCCATGACGGTTCGATCCTGCTGCGGA
CCTCCGACGTCGCCTTCCCAGGGGCAGTCGACGCCGCGCAGCTTTTCCAGGCAAGCGCCGCAAAGTGCGGCAT
CGACGTCGAGCTGAAGCGCGAGCCCGGTGACGGCTACTGGTCCGACGTCTGGAACAAGCAGCCCTTCAGCATG
TCTTACTGGACCGGCCGGCCGACGCAGGACCAGGTCTATTCCATCGCCTATCTTTCAAAGGCGGAATGGAACG
ACACCCGCTTCTTCCGCGAGGATTTCGACAAGCTGATCTTTGCGGCGCGCGGCGAGCTGGACGAGGCGAAGCG
CAAGGTTCTCTATCGCGAGGCGGCAATGATCCTGCGCGATGAGGGTGGTGTGATCGCGCCGATGTTCAACAAT
TACATCGATGCCACCAGCGACAAGGTCGGCGGCTGGCTCGACGATCCGAACGGCGAACTCATGGGTGGACATG
CGCTGACCAAGTGCTGGCTCGCCGCCTGAATGCTCCAGCAGATCAAAGGCCTGCATCACGTAACGTCGATGGC
GGACGACGCCCGCACGAACAACCGGTTCTTCACCGACACGCTCGCCCTGCGCCGCGTCAAGAAGACGGTGAAC
TTCGACTCGCCTGACGTCTACCATCTCTACTATGGCGACGCGACCGGCACCCCCGGCACCGTGATGACCTATT
TTCCCTTTCCGCAGATGCCGCGCGGTCGCGCGGGCACGGGAGAGGTGGGGACGACCGTCTTCTCCGTCCCGCG
GGGCTCGCTCGGCTTCTGGCAGGAGCGCTTGGCGGCACTCGGTGCGAGCGGTCTCAAGCAGGCGGAAAGCTTT
GGCGAGAAGCGCCTGAACTTTGCCGGTCCGGACGGCGACGGCTTCGCTCTCGTCGAGGTCGAGGACGACCCGC
GTACGCCCTGGACCGAGGGCGGCATCACCGAGGATCACGCCATCCGCGGCTTCCATTCGGTCGCCATGCGCCT

ACGCGACGAAGGCGCGACGGCGGAACTGCTGAAGTTCATGGGTTACGAGGAACTCGACCGCAAGGACGGCGTC
ACCAGGCTGATCGTGCCCGGCGGCAACGGCGCGAGCCTCGTCGACCTGGAGACGCTGCCAAACATCAGCCGCG
CTGCGCAGGGCGCCGGTTCGGTGCACCACGTCGCATTCGCCGTCGACAACCGCGAGAAGCAGCTGGAGGTGCG
CAAGGCGCTGATGGACACCGGCTACCATGTGACGCCGGTGATCGACCGGGATTATTTCTGGGCGATCTATTTC
CGAACGCCGGGCGGCGTTCTCTTCGAGATCGCCACGAACGAGCCCGGCTTCGACCGGGACGAGGACATCGCGC
ATCTGGGCGAGGCGCTGAAGCTGCCGCAACAGCACACGCATCTGCGCCCGGTTCTCGAAAGCCATTTGCAGAA
GCTGGAAGCCTAGATGGCCACTTCCGTGTCTACACGGCACCTCAGCGAGAACGGCGCTCTTATCGAGCGTCAC
TGGCAGGAGCTCAACAATCGCCGGCGTCTCTATACCTGGCTCGGTCTCGCATTTCTCGCGTTGACGCTTTTCA
GTTCGCTCTGGTTCGCGAACGAGTCGAACGCGGGCAAGTTCTTCGACCGGCTGCCGCACTTCTTCGATTTCGT
CGGACATCTCATGCCGCGCGATCCGATAGAGATCGTTCGCGCCCTCTTCGACCTGCCTTCGCCCTATGACGAC
GGCAGTTTCAAATACAATTACCCGGATGGGCGCCTCTATGTGACCGAGAGCTTCTACATCCCGGAATATGTTC
ACAAGATGCTGGAGACCGTGAACATCGCCATCTTTTCGACTATCATCGGAGTCTTCTTCGGCTTCATCCTGTG
CTTCCTCGCCGCGAAAAATCTGATGCCCAATCCCTGGATCAGGGGCATCGTACGCCGGCTGATGGAAATCCTG
CGCGCCTTTCCGGAAGTCGTCATCGCCGGCTTTTTCCTGGCAATCCTTTCGCTTGGGCCTATTCCCGCCATCG
CTGCGGTGTCGATCCACACGATCGGCGCGCTCGGCAAGCTGTTCTTCGAAGTGGTCGAGAATGCCGACATGAA
ACCGGAGGAAGGTCTGCGTGCCGTCGGCGCCAACTGGATCGAGCGGGTCTGGTTCGGCATGGTCCCCCAGGTG
CTGCCCAATTTCACCAGCTATTTTCTGCTCCGGCTCGAAATCAACGTGCGCGCCTCGACGATCATCGGCGCCG
TCGGCGGGGGCGGCATCGGGGAACTCCTGCGCCTGTCGATCGGCCAGGGCCATGAAGCAAAGACGCTCGCGAT
CGTCCTCCTGCTCTTCGCCACGATCTTCGCCGTCGATCAGTTCTCCGCCTGGCTTCGCCGCCGCCTCGTCGGC
GACCAGGCCTTTCAGCTTGCGCAATAGATGACCACCGCCGATCGAGTATCCGGAACGCTCATCGTCGTCGTCG
GTCCGAGCGGTGCCGGGAAAGACAGCGTCATGGGCTTTGCCGCCCGCCATTTCGCGCAGCGGCCCGATATTCT
CTTCGTCCGGCGGGCGATCACAAGACCGTCGGATGCAGGCGGCGAAGTGCACGAAAGCGTGTCCGACGCCGAA
TTCGAGGAGATGAGCCGGAGCGGCGCTTTCGCCGTTTCGTGGCAGGCGCATGGGTTGAGCTACGCCATCCCGG
CAGAGATCGCCGAAAAGATCGCCAAAGGCATAACCGCGATCGTAAACGGCAGCCGCGCCGCCCTCCCCGCCAT
TCGTCAGGCGTTCGGTACGATCGCCGTTGCGGTGATCACCGCCGAGCCGCCGGTGCTGGCGAAGCGGCTGGCG
GAGCGCGGCCGGGAGAGCGAGGAAGACGTGCTCCGCCGCCTGACGCGGCAGGCACCCGACGTCATCGCCGATG
CCGACGTAACGGTCATCGACAACAGCGGCAGGCTGGATGTGGCGGGCCGGCAATTCGTCGCGCTGGTCGAGCG
GCACTCAGCTAAGCGCCATCACCCTGCCTGAATGTCGATCAAGCGCTATGGTGCCGAACAGGTGGGCGCAGGC
GGAAAAACATTGCCTTTCGCACGTGCCGTGGAGGCGGGCGGCTGGCTTCACGTCTCCGGTCAGGTCGCGATGG
AAAACGGCGAGATCATCGGCGGCAACATCGTCGCCCAGACGCACAAGGCGATCGGCAATCTTCTGGCCATCTT
GAAAGAGGCCGGCTACGGTGTCGAGCACGTGGTGAGAGTTGGCGTCTGGCTGGACGATCCGCGCGATTTCTGG
AGCTTCAACCGGATCTATCAGGATTATTTCGGAGCTCATCCGCCGGCGCGCGCCTGCGTACAGTCCTCCATGA
TGGTCGATTGCAAGGTGGAGATCGATTGCGTCGCCTATAAGCCGAAGGACGCCTGAATGCGACTGAACTTCAC
CAGGACACTGATCGGTGCCGCCATTGCGCTTGCACTCACCACCGCGGCTGCCGAAGCCGAGGTCGTCGTTTCC
TCGAAGATCGACACGGAGGGCGGCGTTCTCGGCAACATCATCCTCTCGGTCCTGAACGCCAACGACATCAAGA
CGACGGATCGCGTGCAACTCGGCGCCACCCCTGTCGTGCGCAAGGCGATCATCGCCGGCGAGATCGACATCTA
TCCCGAATATACCGGCAACGCCGCCTTCTTCTTCGAGAAGGCCAACGATCCGGCCTGGAAGGATGCGGCGAAA
GCCTATGAAGAGGCGAAGAAGCTCGACTATGAAGCCAACAAGATCGTCTGGCTGACGCCGTCTCCGGCCAATA
ACACCTGGGCGATCGCGCTGCGCAAGGACATTGCCGAGAAGAACAACCTCAAGACGCTATCGGATTTCGGCAA
ATTCGTTTCTGGCGGCGGCGAGGTCGTGCTCGCGGCTTCGTCGGAATTCGTCAACTCCGCCGCTGCCTTGCCT
GCGTTCCAGACGACCTATTCCTTCAAGCTGAAACCGGAACAGCTCATCACGCTCTCCGGAGGCGATACCGCCG
CGACGATCGCGGCTGCGGCAAACCAGACGAATGGCGCCAATGCGGCAATGGTCTACGGCACCGATGGAGGCAT
TGCGCCCTCCGGTCTCGTGGTTCTCGAGGACGACAAGGCCGTCCAGCCGGTCTATCAGCCGGCGCCGATCATC
CGCGAAGCGGTTCTCAAGGAAAATCCGGCGATCGAAGAGATCCTGGAGCCCGTCTTCGAAAAGCTCGACCTCA
CCACCCTGCAGGAGCTCAATGGCCGCGTGCAGGTCGGCGGCGAGTCGGCCAAGGCCGTTGCCGAGGACTTTCT
GAAGGCAAACGGCTTCCTGAAATAGATGAGCGTTGCCAATTTCTTCGACGAAATGTCCTATGGCCCGGCCCCC
GAAGCCGATACCGAGGCGCGCGCCTGGCTCACTCGGCATGAAGGCAAGCTCGGCCACTTCATAAACGGCGCAT
TCGTCGGTTCAGCATCAGGCAAATCCTTCGACACGTTCGAGCCCGCAACGGGCAAATTGCTCGCGAAAATCGC
ACATGGCGGGCGCGACGACGTGAACGCGGCGGTGGCGGCAGCGCGCAAGGCGCAGGGCCCGTGGGCAAAGCTC
TCCGGACATGCCCGCGCCCGGCACCTCTATGCGCTCGCGCGCCTGATTCAGCGCCATGCGCGCCTGATCGCCG
TCGTCGAAGCGCTCGACAACGGCAAGCCGATCCGGGAAACGCGCGACATCGACATACCGCTTGCCGCTCGCCA
TTTCTATCATCATGCCGGCTGGGCACAGCTGCAGGAGACGGAATTCGCCGACCAGGTGCCCGTGGGCGTTGTC
GGACAGGTCATCCCCTGGAACTTCCCCTTCCTGATGCTCGCCTGGAAGGTTGCGCCGGCGCTGGCGCTCGGCA
ATTCCGTCATCCTGAAGCCCGCGGAGTTTACGCCCTTGACGGCGCTGCTCTTCGCGGAGCTTGCCGCTGCAGC
GGGGCTCCCCCCGGGCGTGCTGAACGTGGTGACGGGCGAGGGCGAGACGGGGGCGCTGATCGTCGAGCACGAG
GATATCGACAAGATCGCCTTTACCGGCTCGACCGAAGTTGGACGCCTCATCCGCGAGAAGACAGCGGGGAGCG
GCAAGTCGCTGACGCTGGAGCTTGGCGGGAAGTCGCCCTTCATCGTCTTTGACGATGCCGATATCGACGCGGC
GGTCGAAGGCGTCGTGGATGCGATCTGGTTCAATCAGGGCCAGGTCTGCTGCGCCGGTTCACGCCTTCTGGTG

CAGGAGGGCGTGGCGCCGGTCTTTCATGACCGGTTGAAGCGGCGCATGGAGACGCTCCGCGTCGGTCATCCGC
TCGACAAGGCGATCGATATGGCGGCGATCGTCGCGCCGGTGCAGTTGCAGCGAATCGAAGAGCTGGTGGCGAA
GGGCGTCGCCGAAGGCGCATCGATGCATCAGCCGAAGATCGAACTCCCGAAGGGCGGCAGCTTCTTTCGGCCG
ACGCTGTTGACCGGCGTCCAGCCGACTTCCGTCGTCGCGACCGAGGAAATCTTCGGGCCGGTGGCCGTATCGA
TGACCTTCCGTACGCCGGAGGAGGCGATCCAGCTTGCAAATCATAGCCGCTACGGCCTTGCCGCGAGCGTCTG
GAGCGAAACGATCGGTCTTGCCCTGCATGTTGCCGCAAAGCTTGCGGCCGGTGTCGTCTGGGTCAATGCGACC
AACCTCTTCGATGCGTCGAGCGGCTTCGGCGGAAAGCGCGAATCCGGCTTCGGCCGTGAGGGCGGGCGAGAGG
GCTGCTACGAATATCTGAAGCCGAAGGCCTGGATGGGCGCAAGCTGCGCCCCGAACACGGCTTGCCGGCCCC
GAAGCAGGCCGCCGGTGATTTCGCCGTTCCGGCGCTCGATCGCACTGCCAAGCTCTTCGTCGGCGGCAAGCAG
GCGCGGCCGGACGGGAACTATTCGCGGCCCGTGCTTTCCCCGAAAGGCAAGGTGATCGGCGAAGTCGGCGAGG
GCAACCGCAAGGATATCCGCAATGCCGTCGTTGCGGCGCGGGGCGCCTCGGGCTGGTCTTCGGCAACAGCGCA
TAACCGCGCCCAGATCCTCTATTACATCGCCGAGAATCTGAGCAGCCGCGGGGCGGAATTCGCCGACCGCATC
GCCGCCATGACGGGGGCCTCGGCAGCCAATGCCCGAGCGGAGGTCGAAGCTTCGATTGCCCGGCTGTTCAGCT
ACGGAGCCTGGGCGGACAAATACGAGGGCACCGTGCACCAGCCGCCCCTGCGCGGCGTGGCACTGGCCATGCC
GGAACCCCAGGGCGTCGTCGGCGTCATCTGCCCCCCGGAAGCGCCGCTGCTCGGGCTTGTCAGTCTCGTGGCA
CCGCTGATTGCAGTCGGAAACCGGGTGGTCGCCGTGCCGAGCGAAGCGCATCCTCTGGCTGCGACCGACTTCT
ATTCGGTCCTCGAGACCTCGGATGTGCCGCCCGGCGTCATCAATATCGTCACCGGATCTGCGATCGAACTCGC
CAAGGCACTCGCCGCGCACAACGACGTGGACGCGCTCTGGGCGTTCGGCTCGCCGGAACTTTCGACACTCGTC
GAGAAGCTGTCCGTCGGCAACCTCAAGCGTACCTTCGTGGATTACGGCAAGGCGATCGATTGGACGGATCGCT
CGGCCGCCGAAGGACAGGCGTTCCTGCGGCGAGCGGTCGATGTCAAGAACATCTGGATTCCCTATGGCGAATA
GATGACGATGGCATCGATCTGGCAAACGCTTGCCAACAGGCTCGATCGCCTGCAGGAGACAGGCGGATCGATC
GACTTCTGGCTGCGGGACGACGATGCCGTCGAGCCGACGGCGGCCCTTCACCGACTGCTCGACCTTACCGACC
GCTTTTCGGTGCCGGTGAGCTTGGCCGTCATACCGGCACATACCGACGAGCGCCTCTCGCGCTGCCTCGCCGG
CCGTCCGCATGTCGATGTTGCTGTCCACGGGTGGTCCCATCAAAACCATGCCCCTGTGACTGAAAAACGCCAG
GAACTGGGAGGACACAGGTCGCGCGAGATGGTTGCGGCCGACTTGCGCGCCGGTTGGGCACGCCTGCGCGCGC
TTTATCCCACCGCTATGGTGCCGCTCCTCGTTCCGCCATGGAACCGGATCGATCCGGGGCTGGTCGCCGGATT
GCACGAGATCGGGTTTAGGGCCCTTTCGGTTTTCGGACCGGAGGAGGGGGCAAAATCCGCCGCATTACGCGTT
CCCGGGCTGCAGCTCATAAATGCCCATGTGGATGTGATGGACTGGCACGGCACCCGCGGCTGCCGCCCTTATG
GCGAGATCCTTCGCGACATCGATAGCCGACTGGATGAAATCGAGAAGGGCGGCGGGACGGTGGGAATTCTCAC
CCACCACCTCGTTCACGACGAGAGCGTATGGGAATTCCTCTCCGGACTTTTCGACGTCACCGCCTCGCACGCG
GCCTGCCGCTGGCGCAGGGTCGCCGATCTTATAGATCGATGAATGGGACGCTGGCCGGTTTACGACGAGGAGC
AGATCGAGGATGTCGTGTCGGTCCTCAGATCTGGCGAGGTGAATGCCTGGACGGGACCGCACGTACGCAACTT
CGAGGCTGCCTATGAGCGCTTTCTCGGCGTAAAGCATGCGGTGGCTCTCGCCAACGGAACGGTGGCGTTGGAC
CTCGCGCTCTTCGCGCTCGGACTTGAGCCGGGCGATGAGGTCATCGTGACGCCGCGCAGTTTCATTGCCTCGG
CCTCCACGGTGCCGATGGCGGGTGGAGTTGCCGTCTTCGCCGACGTCGATCGCGACAGCCAGAATATCACCGC
GGAAACCATAAGGGCGAAGCTGACGCCGAGGACCAAGGGCGTCATCGTCGTGCATCTGGCCGGCTGGCCCTGC
GATATGCCCGCAATCATGGCCCTTGCCCGCGAAAAGGGGCTGTGGGTGATAGAGGATTGCGCGCAGGCGCACG
GCGCCGAAATCGACGGACGGCCGGTCGGCAGTTTCGGTGACATCGCGGCATTCTCCTTCTGCCAGGACAAGAT
CATCACCACCGGAGGCGAAGGCGGGCTCGTGGCAATGAACGACGACGAGCTCTGGAAGAAGGCGTGGAGCCGC
AAGGACCATGGCAAGTCGTATGACGCCGTCTACAACAAAGAGCACCCTCCGGGCTTCCGCTGGCTGCATGAGT
CCATCGGTACCAACTGGCGCATGATGTCGATCCAGGCGGTCATGGGATCGCGTCAGTTACAGCGCCTGACGGA
ATGGCGCAGCATAAGAGCGCACAATGCGGCTATTATCGCCAGAGCGGCCGAGGAGATCGAGGCTTTGCGCACG
CCCTTGCCGCCCCGTGGGATTCAGCACGCCTGGTACAGGTTCTATACGTTTCTTCGGCCGGAGCGCCTGCGGC
CCGACTGGAGCCGGGACCGGATCATTGCCGAAATCAACGGCGCCGGCGTCGCATGCTTCAGCGGCAGTTGCTC
GGAAATCTACCTGGAAAAGGCCTTTGCGGACATCGGCATGGCTCCGGCGCAGAGATTGCCCGACGCGCGCGAG
CTCGGCGAGACGAGTCTTGCCTTCCTGGTCGATCCCGCGCTCGATACGGCGGCAATGGAGAAGGCGGCACATG
TCCTGCGGGAGGTGTTTGCAAGGGCCAGCATCGGCAGGCCAGCCCTCGATGCCGAGCGGCGTTAGATGATTCT
GAAGGGAACCAATCTTTCTGACACGATCCTCGGCACTTTGAGTGACGATGAGCTCTGGGGCTATGCGGGGGAT
GATTATCTCGAGGGCCGCGAAGGCAATGACAAGCTTTTCGGCGGCGATGGTAACGACTACATCGTCGGCGGGC
TCGGAGACGACTATGCCGAAGGCGGCGATGGTAACGATCGGTTCGACGGCGGCCTTGGGCACGACACCTTCAA
GGGCGGATTAGGAAACGACATCTTCGACGGCGGAGACGGCAGCGACGTGCTGTACGGCGGCGATGGCCATGAC
CATTTCGTGGGTGGTCGCGGACACGACAAGATCTATGGTGGCGCGGGCGAAGAATACATCGATGCAGGCGACG
GCGACGACATCATCTATGCCGGCGCGGGCGACGACGGCTTCAACAATCGCATAGATCCGGCAACCGGACAGCT
GACGCAGCAGGCCGTCGGCGGGGGCGCCGGCGACGATAGGATCTACGGCGAAGAGGGCAATGACGCGCTGAAG
GGCCAATCCGGCAATGATTGGGTTTATGGCGGCATCGGCGACGACATCGTCGATGGCGGCGACGGGAACAACT
TTCTCGATGGGGGTGACGGCAATGATGTGCTGGACTCGGAGGGCGGCGCGGACGAGGCACATGGCGGCAGCGG
CAATGATCGGATCGCCGTGGGCGCCGGAAACGATCTGGTCTTCGGGGATGACGGTGACGACGTCCTGACCGGT
GAAGGCGGCGACGATGAGCTGAGCGGCGGCCACGACGACGACCGAATCTTTGGCGGCGACGGCAACGACACCC

TGCGTGGCGACGCGGGCCAGGACGTTCTGGTGGGCGAGTCGGGGGCCGACATTCTCTGGGGCGGAACAGAATC
CGATCGTTTCGTCTTCAAAGGCGCCGCAGTGCTCGGCAGTCAGGATACCGTCATGGATTTCCAGGATGGCGTC
GACTTTCTCCTCATCGAAAAGCTCGGGGTCAGCCAATATTCCAGTTCCGGCGCCTCGGGCACGGTCTACGCCT
ATGACACTACAGACGGCGATGTGCAGGTAATGGGCTACGACTCGGCCGGAAACGCCTTCTCCATCCTGGTGGA
CGATCCCAACGGCGCGCTCGCGGCTGCGAACTTTTCGAGGAGTGATTTCCTTTTCGCCTGAATGAGGAAACAC
ATTCCGCCCGCATCGCGACCCGATCCGGAACCGACGTCGCCGGTCGAGCCGATAACGCTCTGGCGCGCCTTCG
TTCCGCGCTGGGCACATATGCCGCTATCCGGCGCGGGTGCCGCTCGCTTCGGTGGCCGCTGGAACCCGATCGG
GATGCCTGCGATTTATGCTGCGCGCGAGCTTTCCACTGCCTGGGCTGAATATAATCAGGGTTTCGTGCAGCAT
CCGGCGCTCATCGTGCAGCTCGAGTTGCGCGGGGCGAGGCTCGCGGACCTCACGGATGCCAGCGTTCTTCTGG
AGCTTGGTGTGGATGAGGCGATCCATCGCTGCGAATGGCGTGATGCGCTCGACAAAGGCGCGGTGCCGGAGAC
CCACAGGCTGCAGTCGGAGCTTCTGGCGCGCGACTATCACGGGGTGATCTATCCATCCTTCATGTCGCCCGGC
GGCACCTGCGTCGCGCTCTGGCGCTGGAACGGTGCGGAAGAACCGCGTCTCGACGTCATCGATCCCGATGGCC
GGCTGCCGAAATCGCCCGCCTCATGGGTTTGAATGTTTTCGAAGCTCCTGATTGCCAATCGCGGTGAAATCGC
CTGCCGCATCATCCGCACCGCGCGACGGCTCGGCATCCGCACCGTCGCCGTCTATTCCGACGCTGACGGAGAC
GCGCTGCACGTGGCGCTCGCCGACGAGGCGATCCGGATCGGGGGGGCGCCCGCCGCCGAAAGCTATCTGGCGA
GCGCTCCTATCGTTCAAGCGGCAAGAAGCGTCGGCGCACAAGCCATTCATCCGGGCTATGGTTTCCTGTCGGA
GAACGCCGATTTCGCGGAGGCTGTCGCAGAAGCAGGCATGATATTCGTCGGGCCGCCGCCGGCCGCAATCCGC
GCCATGGGTCTCAAGGACGCGGCCAAGGCTCTCATGGAACGATCCGGCGTACCGGTCGTGCCCGGCTACCACG
GCGAAGAGCAGGATGCATCCTTTCTTGCCGACAGGGCACGGGAAATCGGCTATCCGGTGCTGATCAAGGCGCG
GGCGGGTGGCGGCGGCAAAGGCATGCGCCGGGTCGAGCGGCAGGAGGATTTCGGCCCCGCGCTGGAGGCCGCA
CGGCGGGAGGCCGAATCCGCCTTCGGCGACGGTTCGGTGCTTCTCGAACGCTATCTGACGAAGCCGCGTCACA
TCGAGATGCAGGTCTTCGGCGACCGGCACGGCAATATCGTCCACCTCTTCGAGCGCGACTGCTCGTTGCAACG
CCGGCATCAGAAGGTGATAGAGGAGGCGCCGGCGCCCGGCATGACGGCCGAGGTCCGCCGTGCGATGGGGGAT
GCGGCGGTCAGGGCCGCCCAGGCGATCGGCTATGTCGGTGCAGGCACCGTGGAGTTCATCGCCGACGTGACCA
ACGGCCTCTGGCCGGATCACTTCTATTTCATGGAGATGAACACGCGGCTGCAGGTGGAGCATCCCGTCACGGA
AGCGATCACCGGCATCGATCTCGTGGAATGGCAGTTGCGGGTCGCTAGCGGCGAGCCGCTGCCGAAGAAACAG
GCCGATATTTCAATGAACGGCTGGGCCTTCGAGGCGCGCCTCTATGCGGAGGATCCGGCCCGGGGCTTCCTGC
CGGCGACCGGCCGGCTGACGGAATTGAGCTTTCCCGAGGGGACTTCGCGGGTCGATTCCGGCGTCCGGCAGGG
GGATACGATCACGCCCTATTACGATCCGCTGATTGCCAAGTTGATCGTACATGGCCAGAACCGTTCGGCTGCG
CTCGGCCGGTTGCAGGATGCTCTCAAGGAATGCCGCATAGGCGGCACGGTCACGAACCGCGACTTTCTCATCC
GCCTGACGGAAGAGCATGATTTCCGCTCTGGCCACCCGGATACGGGGCTGATCGACCGGGAGATCGAGCGACT
GACCGCTCCGGTCGCACCTGGTGACGAGGCGCTGGCGCTGGCGGCGATAGTTTCGACCGGCGCGCTCGACCCG
AACAGGTCGACCGATCCATGGTCGTCGCTCGGCTCCTGGCAGATCTGGGGCGACGCGCACCGCATGGTGGTGA
TCGAACATGCGGACGTGCGCGCAACCGTCACCTTGGCTTCGCGCGGCCGTGACCAGTTCGCCGTGCGTGCCGG
TGCGAGCACGCTGCCCGTCCTCGTGCTCGACCGCTTCGAGGGCGGTGCCCGGCTCGAGGTGGCCGGGCAGAAG
CGCCTGATCCGTTTCTCACGGGATCGCGAGGCGTTGACGCTCTTCCATGGCGGGCGCAACCTGGTGTTCCATG
TGCCGGACGGATTGACCGGCGGGCAGAGCAGCGAGATCGCCGACGACGAGTTGGTTGCGCCGATGCCGGGTCT
CGTGAAGCTGGTGCGGGTCGGCGCCGGCGACGCAGTGACAAAGGGACAGGCGCTTGTTGTGATGGAGGCGATG
AAGATGGAACTGACGCTCTCGGCCTCACGCGAGGGCACGATCGCCAATGTGCATGTGGCGGAAGGCGCTCAGG
TCAGCGAGGGCACGGTGCTCGTGACGCTGATGGAGGAGGCTGCGCAATGATTGATCATCGACACGCATCTGCA
TCTGATCGACAAATCCGCGCTCAACTATCCGTGGCTCGCCGGCGTTCCCGCGCTCGATCGCGATTTTCTCTAT
GCCACCTATGCGGCGGAGGCAAAGCGCGTCGGCGTTGCCGCCTCGCTGCATATGGAAGTGGACGTCGACCCGG
CTGAGATCGAACTCGAAACGCGCGAGGTCGCGCGTCTTGCGGGTGAGCCCGGAAGCCTCCTCAAAGGCGCGAT
CGCGGCTTGCCGCCCGGAAGATGAAGGCTTCGCCGCCTATCTCGAGCGGCAGGAGGAGAACGCTTTCGTCAAA
GGTTTCAGGCGGGTGCTGCACGTCGTTGCCGACGACCTCTCGGAGCAGCCTCTTTTCCGCGAAAACGTCAAGC
GGCTCTCGGGCACCCGCTTCACCTTCGATCTTTGCGTCCTGCCGCATCAGATTCCGAAAGCTATTGCCCTCGC
CGACCTTGCGCCCGACGTTCAGTTCATTCTCGATCATTGCGGCGTTCCGGACATAAAGGGTCACGCGGAGCAC
CCCTGGCGCGACCATATGACCGAGATCGCCCGCCGCCCGAATGTCGTCGCCAAGATCTCCGGCGTGGTCGCCT
ATGCGGAGGAAGACTGGGCGCTCGATTCCATCCGCCCCTATGTCGAGCACACGATCTCGGTTTTCGGATGGGA
CCGGGTGGTCTGGGGCAGCGACTGGCCGGTCTGCACCCTCGGCGGCAACCTCTCGACCTGGGTCGCGGCCACC
CAGGCCCTCATCGAAGGCTGCAGTCCCCAGGAACGGAGGAAGCTTCTCTCCGGCAATGCGCAACGGATCTGGA
ATCTGATCTGAATGAACAAGCACATCGCAGCGCTCACGGTCGCATGCCTCGCCGCAACCTCCGCCAATGCCGC
CGGCCTGCCCGAGCGGATCAAGTCGGCCGGTAAGGTCGTCGTCGCCAACCAGCCGAACTATCCGCCGATGGAA
TATGTCGATCCGGCGACCAACGAACTCAGGGGTGTCGACATCGATCTCGGCGTTGCGCTTGCCAAGGAGCTCG
GCACCACGGTCGAATGGTCGAATATCGGCTTCGAACAGATGATCTCGTCGCTTCAGACAGGCCGCGTCGACCT
TATCCATAGCGGCATGAGCGATCTTCCCAAGCGCCGCGATACGCTCGATTTCATCGATTACATGAAATCCGGC
GCGCAGTTCTACACGTCCTTTGCCCGCAAGGACGAGTTCAAGACGCTCACCGATTTCTGCGGCAAGATAGTCG
GCATGAGCCGCCGCACCTCCTTCCCGGACGAGACGGCGAAATGGAGTACTGCCAATTGCGAAGCCAAGGGGCT

GCCGGCCGTCAAGGTCGTCGGCACGGAAGGCTCCGCCGATGCCCGCGCCCAGCTGAAGCAGGGCCGGCTCGAC
GCCGCCGTCCAGGGTTCCGAAACGCTGCCCTATCTGATGGATCTCGAGAAGGACACCTACGCCGTCGTGGGCG
AGCCCTTCACCGTGGTCTATCAGGGCATCGGTTTCGCCAAGAAGGACACGGAACTGCGCGACGCCTATGCCGC
AGCGCTGAAGGCCCTGATGGAATCAGGCGAATACCAGGCGATTTTCGCCAAGTACGGCCTCGAGGGCACCTTG
CTCGATGGCATTTACGTCAATGGCGAAGCGGTGAAGTAAATGAGCTTAAGTTATTCAGAAAGATTAATTCTCG
CCAATCAATACGCGATTCTTGAGAATCTGGACGTAGGCAACGCACTCCTCCATCGGAGCGCGCGTGAGATCGT
CGAGCGCGGTTATGAATACCTTTATGATACGCTCAATCCGTCGGTGGAAGCAGAAGTCGTTCCCACCGCAATC
GGTCAGGAGGTGTATGATATTCTCGACATGTTCCGCGCGATCGAACGGTCGATGATAGACATCGGCAGGGCCC
CTTCCGAGCTCGAGGCGAATTTTGAAGGCTTTGACCCGAACAACGCCCGCGCTCATTTCCACTTCTGTGAGTT
CGTGCGGCGCTCCCTCGATTATTGGCAGGATCTCGACAAATACCCCGACACCTCGCACGACGCCTCGTCGCTC
ATGCGCTATCGCGCTATGCTGCTGAGATGGAAGTCTCTCGGTGCTCCATCGGCCCTTTCAGAGGCCCAGATCG
CCGAACTCGCGGGTACAGCCAACGCCTTGTCGGGGACCGCACATAAGGTGCACACACTGGTGCCGAAGACAGC
TATGCGGCTGTAGGTGGGCAGATCCGCGAACGAAAAACGAAATGGCGTCCCGCTCATTCATGGGGCCAGCGAT
CTGCCGTCGGTGACGGTTGACGATTATAATCTGGAGCTGCGTGACGGCGACGGCTTCCTTGGCGACAGGGCCA
ACAAATTTGCCTTCCAGGAGAAGCTCGACGCGTGGCGCAAACGCGTGCGCAAAGGCGGCGACGATCCGCTGGG
GCAGGCTTTGACCCAGGATCTGTCCAAAAAGCAAGTCGATGCCCTTCTGCGCGGCGACGACAAGGAGGCGGCC
GCCCTGATCATCGGTGCGGTCGACGATTTCGCCGGCGAACTCGCCAGCGTTCTCGAACGGTTTCTGCAACAAA
AGAACTGGAAGAATACGGAACGCGTGGTAATCGGCGGAGGCTTCCGGGGAAGCGCAGTAGGTGAATTGGCCAT
TGCCCGGGCAATGGTGCTACTGAAGGCTGAAGGCATCAAGATCGAACTCTCCCCTATCGTTCACCATCCGGAT
GATGCGGGACTTATCGGTGCCGCGCATCTCATGCCCGCCTGGATGCTGAAGGGACACAAGGCAATCCTCGCCA
TCGACATCGGCGGCACCAATATCCGCGTGGGCATAGTCGAGCTGCACCTGAAGGACGAAACCGATCTCTCCAA
GGCCAAGGTCTGGAAATCGGATATCTGGCGACATGCGGATGACAAGCCCAACAGAAGCACGACCATCGAGGGG
CTCATCGGCATGATCGAGAAGCTTATAGCCAAGGCGGACAAGGCGGACCTCGCGCCGGCGCCGGTCATCGGCG
TGGCCTGCCCCGGTGTGATCAACGAGGATGGCTCGATCCTTCGCGGCGGCCAGAACCTGCCGGGCGGCAACTG
GGAAAGCGAGCATTTCAACCTGCCCGCCGCCCTTAAGGACGCCATTCCGCAAATCGGCGATCACGAGACCTTC
GTGATCATGCACAACGACGCCGTCGTGCAAGGTCTGTCGCAGATACCCTTCGTGCAGAATGCATCGAGTTGGG
GGATCCTGACGATCGGGACTGGCCTCGGCAACGCGCATTTCAGCAACAAGGCCGAAAACTGAATGGGACAAGC
CGTACGAATCCCCACGAACGAGCGTCAAAGGTTGCTCGCAGTCCGTTCGCTCAATTCAATCGGCAGCGCTCCC
ACGCCCGAACTCGCGACCTTGGCCGAGCTTGCCAAAGGCATCTTCGACGTCTCCTACGCCGCCATCAACATCA
TCGACGAAGACTGGCAGCGGATCGGCGGTCAGGCGGGCTTAAGGATCGGCGAATGCTCGCGCGAGATGTCCAT
ATGCACGCGGGTCGTCTTCGCAGACGAACTCCTCATTGTCCCGGACCTGGCGGAAGACCGTGAGCTGAAGACG
AGACCATATGTGGTCGGAAGCCCGCACTTCCGCTTTTACGCCGGCGCGCCCGTCTATCTCGAGAACCTGCCCG
TCGGCAGCTTCTGCATCCTCGATGTCCGGCCCAGACATCTTTCCGACGGGCAGGCGCAGAACCTGCGCCGATT
TGCGGCTGTCGCCAGTGCGCTTTTGAGCTTGCAGAAGGCAAATCTCGTGATGGGGCTCGCCCATAACCAACTA
CAGAATGCGGCCATTACCGATCCGTTGACCGGCCTGTTCAACCGCTCGGCCCTGTCGTCGATCGTCGACGGGG
CTCTCCACAACGCCATCGCCGCGGGTCAGACCTTCGGCGCTCTTTATCTCGACATGGATGGTTTCAAGGCGAT
CAACGACGAGCTTGGCCATCACGCCGGCGACGAGGTTTTGTACGAAGCCGCCAACCGAATTCGCTCGGTCATC
AGAGCAGGCGATATCGCGATCCGCATGGGCGGCGACGAATTCGCCGTTTTCATTCCAAATCCGCCCAACGCAC
CGGCCCTGGCAGCGGTCTCGCAGCGGCTGGTAGCGGCCTTTCGCGAACCCTTCCAGGTCGACGGCAAGGCGGT
CCTTGCACGCATCAGCATCGGCGGCGCCCTTGCCCCGCTCAACGGGCAGCGACGAGCGGAGCTGCTGAACAGC
TGCGACAAGGCTCTCTACTCGGCAAAGCGTGACGGCCGCGACCGGTTCGTCATCCTCGACGCCTGAATGAGTT
CCTGCCAGTCCAATCTTCCTTCGAACCTCACTGTCAGCCTTCGCGAACTGCGGATGGTCTTCGAGCGGTTGAT
ACAGGTCACGCGGCTCGACGCGGGCCTCGTGCCCTCGCTCAGGGATTGCGCCCTTTATTCCGCAGCGCTCGGC
CTCGGCGGCTTCGCCCGCCTGGCTCACAACCTCGAGGCGGTGCGATCGGCAAATCCGCATGCGCTGAGGCTGA
CAGCGGACGCCGAGCGCCTAACTGTCGACTGCGGCGGGCAGCACGCGTGGATCGCCGCCGATGCGGTGCTCGA
TCTCGCAGCGGAGCGTCTGCGCACGCAGGGTTCGGGGAGGGTCGAGGTCCGTAGCGCCACCGAAATCGGCGAA
CTGAGGGTTGTCGAGGCTCTTGCGCAGCGTTTCGACTGCAAGCTGAGGTCGCCATCGACGAGACAGCCGGCT
CTGCCGAGATCGTCGTCTCGCCCGCCCGGAAGGGAATGGATGTGACGGTTCTCGAAAGAATCCGGCGCAACGG
GCTGACCGTCGATGCGGCTCTCTGGTGGCAGCTGTTCCACCGGTCCGCCGAAGCGCTCGCGCCGGATTCCTAC
GTGTCGCGCCGCCACGCCGGTCCGATCATCGTCGAGGCCGACGGCAAGGTCATCGGCCGGCAGGACGAGGACG
AGACGGACCTCTCACTGCTGATCGCCGGCGCCGCACAGCACGGCACGACCGCAATCAATAACTGAATGAATCC
GGAAACCACTGCAGTCCCCTATGAGCGCCTCGGCATACTGATCGATGGCCGCTGGATATACGAGGCAGAGCGG
AGCACCGAGGTGGTGGATCCCGCGACGGGCCAGACGATTGCCCGGCTGCCTTTTGCGGCCGATGGCGAGATTG
CCGAAGCCGTGGCCTCGTCGCAGCGCGCTTTCGAGAGCTGGAAGGATCGCTCGCCGCTCGAGCGGGGCCGGAT
CCTGCGCCGCTTCGCAGACCTCGCGCGCAAGCATGCCGACGAGATCGCCCGTAACATGACGCGCGACCAGGGC
AAGCCGCTCAGCGAGGCGATCGGCGAGATCCGTTTCGCCGCCGACCATGCCGATTGGCATGCGGAGGAAGCGC
GCCGCATCTACGGGCGGGTCATTCCCGCCCGCGATCCGCGCGTGCAGCAGATGGTCCTTCGCGAGCCGGTCGG
GGTCTGCATCGCCTTCACGCCTTGGAATTTCCCCTTCAGCCAGGCGCTGCGCAAGGTCGTGGCGGCGCTTGCG

AGCGGCTGCACCATCATCCTCAAGGGACCGGGGGAATCCCCCTCCTCCACGGTCGCGATCGGCCGGCTGATGC
AAGAGGCCGGGCTGCCGGACGGCTGTCTCAACATCCTCTGGGGCGACCCGGCCCACCTGTCGGAGACGCTGCT
GGCGGCGCCGGAAGTCAGAAAAATCTCCTTCACCGGTTCGGTCGAGGTCGGGAAGCATCTCGCGAGCCTTGCA
GGCAGGCACATGAAACGGTCCACGATGGAGCTCGGGGGGCATGCACCGGTGATCCTCTTCGACGACGCCGACA
TCGAGGCGGCAGCCGATGCGCTTGCCGGCCAGAAGGTGCGTAATGCCGGCCAGGTCTGCATCTCTCCGACGCG
GTTTTACGTGCAGGCGAAAGGGCACGACCGTTTCCTGGCACGCTTTGCCGAGAAGATCGCATCGACAAGGGTC
GGCAACGGTTTCCAGGAGAGCGTACAGATGGGCCCGCTCTGCCACGGCCGACGCGTCGCTGCCATGGAAGGTT
TCGTCCAGGACGCGCGCGAGCAGGGTGCCGAGATCGTCACCGGCGGCGAACGCGTCGGGAACGCGGGCTTTTT
CTATGCGCCAACCGTGGTCGCCGCCGGCACCGACGAACTGCGCCTGATGAAGGAGGAGCCCTTCGGACCGATC
GCCGTCGTCACGCCCTTCGGCGATTTCGACGAGGTGATCCGGCGCGCCAACAGTCTGCCCTTCGGTCTGGCGT
CCTATGTGTTCACAGGCTCGAGCAGCCGCGCACAGAATGCCGCACGCGCTCTTGCAGCCGGAATGGTGAGCGT
CAACCATTTCGGCCTGGCGTTGCCGGAGACTCCCTTCGGCGGAATCAACGACAGCGGCTACGGGAGCGAGGGA
GGCTCCGAGACATTCGACGGGTATCTGAACACGAAGTTCGTCACGCGTTTCGACCAAATCCTGCAGTGATTGC
TCTGCACCGCTGTGGCATGGTCGCTGTTCCAGCTCTGGTACGCGTCTCCCCTACCCTTCGTCTTCGGCTTCGG
CATCCTCAACGATACCGAAGCTCGCGCCATCCATCTCGGTTTCGCGCTGTTCCTGATTTTTCTTGCCTATCCC
GCGCTGAAAAGTTCGCCCCGCGACCGGGTGCCGCTCGCCGATTGGGTGCTCGCGGCGCTCGGCGCCTTCGCGG
GCGCCTATCTGTTCCTCTTTTATGGCGAGCTTGCCGGACGGCCCGGCCAGCCCTCGATGCTCGATCTCGTCAC
CGGCACCGCTGGCATCCTGCTCCTGCTCGAGGCCACGCGCCGCGCGCTCGGTCTGCCGATGGTGTTCGTCGCC
GGCGTCTTCATCTTCTATACCTTCGCGGGGCAGTACATGCCCGACGTCATCCAGCATCGCGGCGCTTCGCTGG
TGAAGTTCATCAACCATCAATGGCTGACGACGGAGGGTGTTTTCGGCATTGCGCTCGGCGTGTCGACGAGTTT
CGTTTTCCTCTTCGTCCTGTTCGGAACGCTGCTCGAGAAGGCCGGTGCCGGCAACTGGATGATGCAGATCTCC
ATCGCGCTGCTCGGACATCTGCGCGGCGGCCCGGCCAAGGTGGCGGTCGTCTCGTCGGCCTTGAACGGCGTCG
TTTCCGGCTCGTCGGTCTCCAACGTCGTGTCGGGCGGCATCTTCACCATTCCGCTGATGAAACGCACCGGGCT
TTCCGGCGTGAAGGCCGGCGCGATCGAAGCCACGGCCTCGATCAACGGCCAGATCATGCCGCCCGTCATGGGC
GCCGCGGCATTCCTGATGGTCGAATATGTCGGCATTCCCTACTCGGAAATCGTCAAGCATGCGCTGCTGCCGG
CGGTCTTCTCCTACATAGCGCTTCTCTACATGGTGCATCTGGAGGCGGTGAAGCTGAACATGCAGCCGATCCC
CCAGCGTCCGACTCCGATGCGCGAGCGGTGGCTTCGCATGGGCCTCGGACTTGCCGGCAGCGTGCTGGCAATC
TGCCTTCTCTATTACGGAATCATCGCCATTCGGGCCGCATTCGGAGAAAGCGCGCCTCTGCTGCTCGCGATTG
CCGGCGTGGCGCTCTACATCGCGACGATCTGGTACTCGTCGCGCTATCCGGACCTGGAGCTCGACGATCCAGA
CGCGCCGATCCTGGAACTGCCGCGGGCATGGGATGTGACGCGGACCGGGCTGGATTTCCTCATCCCGATCGTC
GTCCTGCTCTGGTGCCTGATGGTCGAGCAGCTTTCGCCCGGGCTTTCGGCCTTCTGGGCAACGCTGGCGATCA
TCGCCATCGTCGCCACCCGCAAGCCGCTGATGGCGATCTTCCGGAAGGAGGATGTTGCTGGTTCGGTCCGCGC
CGCCGCCTGGGACCTGACCGACGGCCTGGCGCTCGGTGCCCGCAACATGATCGGCATCGCGGTCGCCACTGCC
ACCGCCGGTATCGTCGTCGGAACGATCACGCTCACGGGCCTCGGCCTGATGATGACGGAGCTCGTCGAGTTCA
TTTCGGGCGGCAACGTCATCATGATGCTGATCCTCATCGCCGCGATCAGCCTAGTGCTCGGCATGGGAATTCC
GACCACCGCCAATTACATCCTGGTGGCCACCCTCATGGCGCCGGTCGTCGTCGAGCTCGGCTCGCAGGCGGGC
CTCGCCATACCGCTGATCGCAGTCCACCTCTTCGTTTTCTATTTCGGAATCATGGCCGACATCACGCCGCCGG
TTGGGCTGGCTGCCTTCGCCGCGGCGGCGATTTCCAAGGAAGACCCGATCGCCACGGGCTTTCAGGGTGCGCT
CTATTCGCTGCGCACCGCGATCCTCCCGTTCGTTTTCATATTCAACCCGGCGATCCTGCTGATCGGCGTCGAC
ACCTGGGCGCATACGATCTGGGTGGCGGCCATATCGCTCGCTGCGATCCTCATTTTCTCGGCCGCGACGATGA
ACTGGTTCGTGACAAAGAGCCGGTTGTGGGAGAGCGCGGTGCTGCTGCTCGTCTGCTTCACGCTGTTCCGTCC
CGATTGGTGGCTCAACCAGATCTCGCCGCCCTATGAAGAACTGCCCGCCTCAGAATTCCTGCGAGCTGTCGAG
GAGGCTCCGGCGAAAAGCCGGATCAACTTCGTGGTTCAGGGCTTCGACCTGATGGGCGACGAGGTCCGCAAGA
CCGTCAACGTGCCGCTCGGCGAGCCCGGCGAACCGCTTCAACGGCTGCAATCGATCGGGCTCACGGTCACACC
GGCGGGCGAGAGTCTGATGATCAGCAATGTCGACTTCGGCTCCTATGCAAAGCGCATCGGCCTCGATGTCGGC
TTTGACATCGTCGCAGTGCTGAGAAAAGCCGAGCAACCGTCAAGCGCCATCCCGGTGGGTATCGCGCTGGCTG
TGACCGCGGGAATTGCCGGCTTACAGTTCGCGCGCAAAAGGGCCGATCGCAGCGAAGCCGGCCTGACGGGGGC
CGCGCGCAAATGAATGAACATCAGCATATCTCAGCGGACGGACACGATGTCCGCCGTTATCGTCACTGGGCCG
GGTACCGTGCGCATAGAGACGTTGCCGTTGCCGCAACCGGGGCGCGGCCAGGTGCGGATAAGGCTCGAAGGCT
GCGGCGTATGCGCCTCCAATCTCGTTCCCTGGGCGGGTCCGGAATGGATGCAGTTTCCGACGGAACCCGGCGC
GCTCGGACACGAGGGCTGGGGAATTATCGACGCGGTCGGCGAGGACGTGCACGGTTTCGCGCCGGGCGATCGC
GTGGCGGCGCTTTCCTACCATGCCTATGCCACGCACGATATCGCCGATCAGAGCGCGATCGCGCCCTTGCCGG
CGTCTCTTGCAGGTCAGCCCTTTCCCGGCGAACCGCTCGGCTGTGCCTTCAACATCTTCCGGCGCAGCGCGAT
CAGTCCGGGAGAGACCGTGGCGATCGTCGGCATCGGTTTTCTCGGGATTCTTCTGACCGAGCTCGCAAGCGCT
GCCGGCGCGCGGGTGATAGCGATATCGCGCAGGCCGTCTTCGCTCGCCGCGGCCAAGCAGGCCGGCGCGAGCC
TAGTGATTCCCATGGACGATCACTGGCGGATCATCGAAAAGGTTAAGCAACTGACCGGCGGGCGCTTCTGCGA
TTGCGTCATCGAGGCGGTCGGCAAGCAATGGCCGCTCGATCTTGCCGGCGAACTGACGAAGGAGCGCGGCAGG
CTCGTCGTCGCAGGATATCACCAGGATGGCCCGCGGCAGGTGAACATGCAGCTCTGGAACTGGCGCGGCCTCG

ACGTGATCAACGCGCATGAACGCGATCCTGCCGTCTACATGCGCGGCATGAACGAGGCGATCGAAGCGACCGC
AGCGGGTCGGTTCTCTCCCTCGCGGCTCTACACCCATCGGTTCCCGCTCGAAGAACTCGGCGCCGCGCTCGAC
ATGACGCGCGACCGGCCGGAAGGTTTCATCAAAGCGCTGGTGATCTGCCATGAGTGAATGACCCGCCTGCGGC
TCCGTTCGCTGGTCCTGCTCCTGCTTGCCGCCTTCCCGGCGGCGGCGGCCGAGGAGCCTGCCGGCTACCGCGA
GAGCGAGTACCGCGCCGAGGTGCCGGCGACGCTTTCCGGTGCGACGGTCGTGTCGACCGAAGCTGCCCATGCA
TTGTGGAAGACCGGTCAGGTCGCTTTCATCGACGTCCTACCGCGGCCGCCGAAGCCGACCAATCTCCCCGAGG
GGACGGTCTGGAACGAAAAGCCGCGCCTGTCCATTCCATCTGCCCTATGGCTGCCGAATGTCGGCTATGGCCA
GCTGGCCGACGCCATGCACGCCTATTTTCGCGCCGGCCTGGAAAAGGCAACCGGTGGCGACAAGGACCGGCCG
GTGCTGTTCTTTTGCCTCGACGGCTGCTGGATGTCCTGGAATGCCGGAAAGCGCGCCCTGGAATATGGCTACA
CACGTGTCTTCTGGTATCCGCAGGGCACCGATGGCTGGACGGCCGCCGGCTATCCCACGGAGCGCATCGAGCC
GGAGCCCGGCGGCCGGTGAATGGCGGGCAAACAACTGCTTTTGATGGATGGAATCCATAAACGATTCGGACCG
CTCATCGTCAGCGCAGGCGTGACCCTCGACGTCGAGGAGAACGAGATTCACGCGCTGCTTGGCGAAAACGGCG
CCGGCAAGACCGTTCTGATGAGCATTCTTTGCGGTGTTCTCTCGCCCAATGAAGGGCAGATCGTCTTCAAGGG
ACGGCCACTCAAGCTAGGCTCCCCCCGCGAAGCCATCAAACATCGCATCGGCATGGTCCACCAGCACTTCATG
CTGGTCCCCAACCTCACGGTCGCGGAAAACTACGTGCTAGGACAGGGCTCGCCCTTCAGCATGATCCGCGACA
TGAGGGCGGTACATGCGCGCATTCTGGAGCTCAGCGACCGCTACGGTCTCGACGTCGCGCCCGACGCGCTCGT
CTCCAGCCTGTCGGTTGGCGAACGGCAGCGGGTCGAGATCTTGAAGGTGCTCTACCACGGCATCGAACTGCTG
ATCCTGGACGAGCCGACCGCGGTCCTGACGCCTCAGGAAACCGACCGTCTCCTGCATCTGCTTCGCCAACTCG
TCGCAGACGGAAAGACGGTCATCTTCATTTCGCACAAACTGGACGAGGTGATGCGCGTCAGTGATCGCGTCAG
CGTCATGCGCGATGCCAAGGTCGTCCGTACGGTGAAAACGTCCGAGACGACCGCGCGCGAGCTGGCGCGCATG
ATGGTCGGCAGAGACGTTCTGATGGATTTGCCGCGGGCACCTCTCGAGCCCGGCCGTGTCGTGCTGTCGGTCG
AGCATCTCAGCTGCGACGGCGAAGCCGGGCTTCCGGCGCTGCGAGACGTATCTTTCGAGGTCAGGGCCAATGA
AATAGTCGGGATCGCAGGCGTCTCCGGCAATGGCCAGAGCGAGCTGGCGCTGGCCCTGACGGGGCTTCTTCCG
GTTGCCTCCGGCAGCGTCACGCTCGACGGGACCCAGCTGGCGGGCCTGTCGTCCTACGAGATCAATCAATTGC
CTATGGCGCATATCCCCGAGGACCGCCACAGGATGGGCATCGTGCTGCCGCTGCCACTGACGGAGAATGTGAT
CCTTCAGCGGTTCGACAAGCCTCCCTTCTCGTCTCGTGGATTGCTCGACCTGAACGAGATCACGAGGCAGACC
CGTGATCTCATGCGCCGCTTCAAGGTCAAGGCGTCGGGACCGGGCGACCGCATCCGGAATTTGTCGGGCGGCA
ATCAGCAGAAGCTCGTCGTCGGGCGCGAGTTGGACCGCCGGTTCGATTTCCTGCTGATCAATCAGCTTACGCG
CGGTATCGACATCGGAGCGACCGAGCTGGTGATGCACAAGATTCTCGAGCAGCGCAGCGCCGGCAAGGCCATT
CTGCTGATATCCACGGAGCTCGAGGAATTGTTCACGCTCTCCGACCGCATCCTCGTCATGTATGAAGGCCGCC
TCGTCGGCGAGATTCCGCCCGACCGCAGCCGTCTCGAAGAGATCGGGCTGCTGATGGCCGGCAAATCTCCGCT
ATCGGCAGCTTGAATGCCGAATGCTTCCGACATCCTCATCGACACGCTTATCGAATGGAAGGTCGACGTCGTC
TTCGGCCTGCCCGGAGACGGCATCAACGGCATCATGGAGGCGCTCAGGAGGCGGCAGGATCGCATCCGGTTCG
TTTCCGTGCGCCACGAGCAATCGGCCGCCTTCATGGCATGCGCCTATGCCAAATTCACCGGCAGACTCGGCGT
CTGCCTCGCCACATCGGGCCCGGGCGGCACAAATCTGCTGACCGGCCTCTACGATGCGAAGCTGGACCAGATG
CCGGTGCTCGCGATCACCGGCATGCAGTATCACGACCTGATCGAGACGTTCTCCCAGCAGGACGTCGATCTCA
CCCGCGTCTTCGAAAACGTCGCCGTCTACAACGCGCAGGTGAACGACGCAGCCCACATGGAAAACCTCGCGAA
CCTTGCCTGCCGGTCGGCGCTCTCCAAGCGCGGCGTGGCGCATCTTTCGATCGCCAACGACGTGCAGGAGCGG
ATGGCTGATGGCGGGCGGTCCCGCCGCAACCGGCCGGGGCACATGCCAAGCCGGTATTTCGCCGGCCAGCTCG
TGCCGCTAGAAAGCGATCTTTCGAGAGCGGCGGATCTCCTGAATGCGGGCAGGAAGGTGGCAATTCTTGCGGG
GCGCGGCGCACTCGAGGCGAAGGAGGCTCTGCGCGAGACGGCGGAACTGCTGGGCGCGCCGGTGGCAAAGGCT
CTTCTCGGCAAGGCGGTGCTGCCCGACGATGATCCGCTGACCACCGGCGGCATCGGCATACTCGGCACCGCGC
CGTCGCAGGAGATCATGCAGCAATGCGACACGCTGCTCATCGTCGGCTCGACCTTCCCCTATATCGAATATTA
TCCCAGGCCGAACACGGCAGCCGGAATCCAGATCGACCGTGACCCGCAGCGCATCGGTCTGCGCTACCCCGTC
GAGGTCGGGCTGGTCGGCGAGGCAGGCGAGACCCTCCGCATGCTCAACAAGCGGCTCAAACGCAAGCAGGACA
GGTCGTTCCTCGAGCGGGCGCAAGAGCAGACGCGAAACTGGCGCCGGGAACTGCGGGCGATCGAAGGCGACAG
GAGTTCTCCCTTGAAACCGCAGGCGGCGGTCGCGGCTTTCGGCCGGCGCATCGCGAAGAAGGGTATCGTCGTC
ACCGATTCCGGACAGAACACCGAGCTTGCGGCCCGTCATGTCGATCTCGGTGCGGACCACATGTTCGCGGTCT
CCGGCGCCCTCGCCTCGATGGCTTCGGGCCTGCCCTATGCTATCGCGGCGGGTATCGCGATGCCGGACCGCCC
GGTCTATGCGGTCGTCGGCGACGGCGGCTTCGCGATGCAGCTCGGCGAATTTGCGACAGCCGTCGGATACCGG
ATTCCTTTGAAGCTCCTGGTGATCCGAAACGACATGCTGAACCAGATCGCCTGGGAGCAGATGATGTTTCTCG
GCAATCCGCAGTTTGCCTGCGAATTGCCGCCGATCGATTTCGCCGCGGCGGCGGAGGCCATGGGCGGCCGGGG
CTTCACCGTCCGGTCCTTCGGCGAACTGGACGGGATCATGGATCAGGCCTTCGCCGTGGATGGGCCGGTGGTC
ATCCAGGCGCTGGTCGACCGCTACGAGCCGCTGATGCCGCCGAAAATGCCGGCGGACTACGCCCGGAACTTCC
GCGCCGCGCTTCCGGAAACGCCGGGGCACGAGAAGATCGAGGAAAACCTCAGGAATTCCTCCGTCGGCAGGAA
GGTAACCGACGAGGAGCCGCAGCCGCCGCATGAGGCGGCGCCGGACGAGAACACCGGCGAATTGCCGGGAATT
CCGTAGATGGACAAGGTCATTCTGATCACCGGCGCCTCCGGCGGCATCGGCGAGGGCATCGCTCGAGAACTTG
GCGTTGCCGGTGCGAAAATATTGTTGGGTGCACGCCGCCAGGCCCGTATCGAGGCCATTGCCACCGAGATTCG

AGACGCAGGAGGTACCGCCCTGGCGCAGGTGCTGGACGTGACCGACCGTCACTCCATGGCAGCCTTTGCGCAG
GCCGCGGTCGACACTTGGGGACGCATCGACGTCCTGGTGAACAATGCCGGCGTCATGCCGCTCTCGCCGCTTG
CCGCAGTTAAGGTGGGCGAGTGGGAGCGCATGATCGACGTGAACATCAAGGGCGTGCTTTGGGGCATCGGGGC
CGTATTGCCAATCATGGAGGCACAGCGTAGCGGGCAGATCATCAATATCGGCTCGATCGGAGCGCTGTCGGTC
GTACCCACAGCCGCGGTATATTGCGCAACGAAGTTCGCAGTCCGCGCGATCTCTGACGGACTGCGCCAGGAGA
GTACCAATATCCGCGTCACCTGCGTCAATCCCGGCGTCGTCGAAAGCGAACTTGCTGCAACCATCACTCACGA
GGAGACCATGGCGGCGATGGACACCTATCGCGCCATCGCGCTTCAGCCTGCCGACATCGCCCGTGCGGTCCGG
CAGGTGATCGAGGCCCCGCATTGCGTGGACACCACCGAGATCACGATCAGACCGACCGCATCGGGCAATTGAA
TGAACGCCGTAGAACTGCGCGCGATTCAAGCGCCGATAAAGGAACGCTATCGTGCAGAGCCCGGGGCAGCCCG
TTTCACGCTGAAGGCGCGCGGGACACTTGACGAAAAGAACATCGCCTGCAAGGTCGAGACTGGGCACGCACTG
GCAGTGGCCGGCCTCCACAGGGCTACTGGCGGGACCGGCCTTGAACTTTGCTCGGGCGACATGCTGCTGGAGG
CCCTCGTCGCTTGCGCGGGCGTGACGCTCAAGGCCGTCGCAATGGCCCTCGAGATTCCGCTGTCAGCCGGCAC
AGTATCCGCTGAAGGCGATCTCGACTTTCGCGGCACTCTGGGTGTCGTCAAAGATGTACCGGTGGGTTTCGAC
CAGATCCGCATTATCTTCGAGCTTGAGACGGACGCCCCACAGGAGAAGTTGGATCAGTTGCTAAAGCTGACTG
AACGCTACTGCGTCGTGTATCAGACCATCCGCAACGGGCCCTCGATGGCTGTCGAGATGAGACGCTCGGTGTA
AGTGTCGAGATTTAACCCTACCAGAGAAACAGGTTCGATTTACGATGCCGCTGCGGCGTGGCGAGACCGGTGC
CTCTTGGCGGATGGTTCGGTGCTATCAGATGAGGCCCTCTGGGCGGTCGCCCTGCTCGACGAACTCGACGAGC
GCTTCGTCAAGAATATCGACGCAGGCGAAGGCGATTTCCTTTCCAAGCTGGAGGTGCAGCTGTCGTCCGGCTC
GCCCCAGTGCCGACAGTTGATGGCTGAGAGCCTGTGGTTGCTGCTGCTTTTCCCGTCGAATGTCGGCGCCGCC
AGAAAGCGCGAAAACGTGCGCGAGGTCTGGTCTTGGTCGGGCGACGAACTGGACGAACACCATCCGTTTGTCG
ACGACAAGGTTCTCGCGGGCATCGGCTCGGCCGGTACAGCCTATAACACCCACCGTTGGCGCGAGCTTGTTTT
TCTGATCGGCGCGGTGCGCGACTTCAAGCGACGTGAGAGCGCCCAGCGCCACGACATCCTCCGTGAGCCATGG
GCGTTTTCCGGCTGGCTGAGCAGCCTGTCCGAGGCGCGCCACCGGCAGTTGCTACAGATCCTGCCGCACCTGT
TATTTCCAGAAAATTTCGAGCGGATCAGCTCCGAGGGCGACAAGCGCCTGATCCTAGCCGGGTTCGGCGACGC
GCCGGAGAAGGAAATCCGGAAGTGGAGCACCACCGAGATCGATCGCGCCTTGCTCAACCTGCGCGCTCGGCTT
GAGCAGGAGCAAGGTGTCGACATCGACTTTTACCAGGAAGAGTTCTCCGCCCATTGGAAGACCCAGACGCAAA
ATTGGCTCCTGAGCTGGAACCCTTCCAGGTGGACATGGGACACCCTCGCCGAAGACAGGGCCGCCACGAACAT
TGGCGAAAAGGCTGAGAACCGCTGGCGCTGCGCATCGACGAAGGCGCGCGAGGGCGACCGCGTCTTCCTGGTG
CGGACTGGAGAGCCACCAAAGGGCATCGTCGCCGTTGGCAAGGTCACCCGTGCACCATATGAAGCCCCACATT
GGGAAGCGGCCCGCGCAGAAGCGGGCGAAACGACGCGCTTCATCGACGTCGCTTTCGACGCCGTTCGGGACGC
GTCGATCGACCAGATCGTGAAGCTCGAAGAGTTGGAGCGTCGGGAAGCCGGTCAGGAGTGGAACCCTCAATCA
TCGGGGATAGAGATCAAAAGCAAGCCGGCGCGGACACTCGAGAGGCTGTGGAAGGAGTTACCCCCCGTTGCCG
GACCATCCGCGCCCGATGATGAAACAGCTGCTACCAAGGCGACACGCTCGACCGGATCGCCCCTGAACCTGAT
CCTCTACGGGCCGCCAGGGACCGGAAAAACCTACCGGCTGATGAACGACTATGTGCCGAACTACCGCGATGAG
GCCGAGGACCGCTCCGAATTCATTACCTTCCACCAGAGCTACGCCTACGAGGACTTCGTCGAGGGCATCCGGC
CGTTGGCCAAGAACGGCAGCATCACCTACGAGGTCCGACCCGGGGTACTGAAACGACTCTGCGCACGCGCACG
CCAGTCGCCCCAGAAGCGCTTTGCCCTATTCATCGACGAAATCAACCGGGGAAACATAGCTAAGATCTTCGGC
GAGTTGATATCATTGGTGGAGGTCGACAAGCGGATCCACACCGACACGTCGGGGAAGCGGTTACCCGGCTGCA
AGGGCCTGGAAGTCACGTTGCCCTACTCGGGCGAACGTTTTGGCGTGCCAGCGAATGTCGACGTGATCGGCAC
GATGAACACCGCGGATCGCTCGATCGCGCTCCTCGACAGCGCGCTGCGCCGCCGCTTTCGGTTCGAGGAGCTC
ACACCTAAACCCGAGCTTCTGCAAACGGTGGATGATGACGCCGGCGACACGATCGACCTGCGACAGTTGCTCG
CGGCCATGAATACGCGCCTATCGCGGCTGCTGCATCGGGATCAAACGTTGGGACATAGCTACTTCTGCAACGT
CAAGTCGTTTGGCGATCTTCGCAAAGTCTTCGCACGCGAAATCCTGCCCTTCCTGCAGGAGGCCTTCTACGAC
GACTGGCGACAGATCCGGCTTGTCCTGGCCGACCAGACTGTGGGCGAGGAATTCCAGCTGGTACGGGCGCATA
CGCAAAGCGCCCAGCGGCTCTTCCCCCTTGCCGACGGCAACGAGATAGGCGACGGCGAGACATTCGAAGTCGT
TCGCGAGGACGACATCACGGCCGACGCGATCCGCAAAATCTACGAGCCACCGGAATGAATGAACGACAAGATC
ACCAACTGGACCAGATCCGACGACGCTATGGTCGAAACGGCCATTCGTCGCGGAGCGACACGCCGCGAGCTCC
TGCAGATGATGCTGGCCGGCGGTGCCGCCCTCTCCGCCGGCAGCCTGGTGCTCGGCCGAGCCGGGAATGCGGT
TGCTGCAACGCCGGTCTCCGGCGGTACGCTCAGAGCGGCCGGGTGGTCGGCTTCCACCGCCGACACGCTCGAT
CCGGCCAAGGCATCGCTCTCCACCGACTATGTCCGCTGCTGCTCCTTCTATAACCGCCTCGCCTTCCTCGATA
AAGGCGGCACGCCGCAGATGGAGCTGGCCGAAGCCATCGAAACCAAGGATGCGAAGACCTGGACCGTCAAGCT
GAGGAAGGGCGTCACCTTCCATGATGGCAAGCCGCTAACAGCCGACGACGTGATCTTCTCGCTGAAGCGCCAC
CTCGACCCGTCTGTCGGTTCAAAGGTCGCCAAGATCGCCGCGCAGATGACGGGCTTCAAGGCGGTGGACAAGC
AGACGGTCGAAATCACGCTTGCGAGCCCTAATGCCGACCTGCCGACGATCCTCTCGATGCACCACTTCATGAT
CGTCGCCGACGGCACCACCGACTTCTCAAAAGGGAACGGCACCGGCGCTTTCGTGAGAGAAGTCTTCGAGCCC
GGCGTGCGCTCCGTCGGCATCAAGAACAAGAACTACTGGAAGTCCGGACCGAACGTGGACTCTTTCGAGTACT
TCGCGATCAGCGACGACAGTTCCCGGGTGAATGCGCTCTTGTCCGGCGACATCCACCTCGCCGCCACGATCAA
TCCGCGCTCCATGCGCCTGGTCGAGAGTCAGGGCGCCGGCTTCGTCCTCTCGAAGACGACCTCCGGCAACTAC

ACCAACCTCAACATGCGGCTGGACATGGAACCCGGCAGCAAGCGCGACTTCGTCGAAGGCATGAAGTATCTCG
TCAACCGCGAGCAGATCGTTAAGTCGGCGCTTCGGGGTCTGGGTGAGGTCGGCAACGACCAGCCGGTATCCCC
GGCCAACTTCTATCACAATCCCGACCTGAAGCCGCGCGCCTTCGACCCCGAGAAGGCGAAGTTCCACTTCGAG
AAGGCCGGCATGCTAGGCCAGTCGATCCCGGTGGTTGCCTCCGATGCAGCGAACTCCGCGGTCGATATGGCGA
TGATCATCCAGGCCTCCGCTGCCGAAATCGGATTGAAGCTCGATGTCCAGCGCGTTCCCGCAGACGGTTACTG
GGATAATTACTGGCTGAAGGCGCCGGTCCACTTCGGCAACATCAACCCGCGCCCCACGCCGGATATCCTGTTC
TCTCTGCTCTACTCCTCGGAGGCTCCGTGGAACGAGAGCCAATACAAGTCGGAGAAGTTCGATAAGATGCTGA
TCGAGGCGCGTGGCTCGCTCGACCAGGAGAGGCGCAAGGCGATCTATAATGAGATGCAGGTGATGGTCGCTAG
TGAAGCCGGCACCATCATTCCGGCCTATATCTCCAATGTCGACGCGATCACCGCCAAGCTTAAGGGCCTGGAA
GCCAATCCGCTTGGCGGGCAGATGGGTTATGCTTTTGCGGAATATGTCTGGCTCGAGGCCTGAATGTTCGGCA
AGAAATCCATTTCGGAAGAACGGAGAGCCGAAGCAGCGGTCCAGCCCGTCGTCGAAAACGAACTGCAGGCCGC
GCACGCCCCTGTCCAAGTTCGGAAGCCAGCCCAGGTTCCGGCAACTGCGAAACCCGAGAATGCCGAACAATAT
TACAGCTTGAAGAAGGAAATCTTCAGCGCCCTCATCGCGACGATCGACGTTGCAGCACTCTCCAATATGGATG
GCGAGCAGGCGCGCAACGAAATCGGCGCGATTATAAACGACATCGTCGCGGCCAAGAAGGCCGGCATTTCGAT
GGCCGAGCAGAACGACCTTCTCAGCGACATTTGCAACGACATCTTGGGTTACGGCCCGCTCGAGCCCCTGCTC
GCACGCGACGACATCGCCGACATCATGGTCAATGGCGCAAACCAGGTCTTCATCGAAGTCAACGGCAGGGTGC
AGGAGACGGGAATCCGCTTTCGCGACAACGAGCAGCTACTCAATATCTGCCAGCGCATCGTCAGCCAGGTTGG
CCGGCGCGTCGACGAATCGAGCCCTATCTGCGACGCGCGTCTGGGCGACGGTTCCCGTGTCAACGTGATTGCG
CCTCCGCTGGCAATCGACGGCCCCACGCTGACGATCCGCAAGTTCAAGAAGGAAAAGCTGACCCTCGACCAGT
TGGTCCGGTTCGGCTCGATCTCGCCGGAGGGCGCCGAGGTTCTCAAGATCATCGGACGTGTGCGCTGCAACGT
CCTCATTTCCGGCGGTACCGGCTCCGGCAAGACAACGCTTCTGAACTGCCTGACCGGCTATATCGACCACGGC
GAACGCGTCATCACCTGCGAGGATGCGGCAGAGCTTCAACTGCAGCAACCGCATGTGGTGCGCCTCGAAACGC
GGCCGCCGAACATCGAGGGGCAGGGCGAGATCACCATGCGCAACCTCGTGAAGAACTGCCTGCGCATGAGGCC
CGAGCGGATCATCGTCGGCGAGGTGCGCGGGCCCGAGGCCTTCGACCTGCTGCAGGCGATGAACACGGGCCAC
GACGGCTCGATGGGAACGCTCCACGCCAACTCCCCGCGCGAAGCGATGGCACGCATCGAGGCGATGATCACCA
TGGGCGGGAGCTCGCTGCCGGCCAAGACGATCCGGGAGATGCTCGTCTCGTCAGTCGACGTGATCGTGCAGGC
AGCGCGACTTCGCGATGGTTCGCGCCGGATCACCCACATCACCGAAGTGCTCGGCATGGAAGGCGAGGTGGTC
ACGACGCAGGATCTCTTCGTCTACGACATTCTCGGCGAAGACGAGAAGGGAAACATCATCGGAAGACACCGGT
CGACCGGGATAGGTCGCCCGGCATTCTGGGATCGCGCACGCTATTACGGCGAAGAGGCCCGGCTTGCCGCCGC
CCTCGACGCGGCGGAATTGAAGGCCGCCGCCTGAATGTCTATCAGTCTTCGCGGCCGCAGCGTCCTGACGCTC
GACGAATTCACTTCAGGGGAGATCGGGTTTCTGCTGCATATCGCCGCCAGGGTGAAGGCCGAAAAGCGAAGAG
GCAATGAAATCCCCCTTCTCAGCGGCAAGAACATCGCGCTGCTTTGCGAGGAGGGTTCGATGAACGCCCTCTT
CGGATCTGAAGTTGCGGCCTTTGATCAAGGCGCGCGAGTCGTTGGTCTGACGTTGCCCGCCGGCCATGGCGGG
CAATGCGCATCACTGAAGGATATGGCCCGTGTGCTTGGCAGGATCTACAACGCGGTCGAATATTGCGGTCGCA
GCCAGGGCGCCGTCGAAGAACTGGCGGAATACGCCCGCGTTCCCGTCTACAATGGCTCTTCCGAAGAATGCCA
TCCCATCCAGGCTCTGGCAGACTTCTTCACGATGCAGGAAGCTGTCGACAAGCCGTTGCAGGAGGTCACGCTA
GCGTTTCTGGGCGACGGTCGCAGTCCTATCGCCCGTTCGCTCGCGACCGGAGCGGCCAAGCTCGGCATGGACT
TCCGCATCGTCTCGCCGCCAATTTTCTGGCCAGCTCCTTCCTTCATCGACGACGTGACCTCGGAAGCGCATGA
GAATGACGGGAGTTTCACGGTCCTGGAGCATGTCGGCGCCGGGGTCTTAGGCGCCGACTTCGTCTACACGGCT
TCGTGGCTGGGAGAGCAAGAAGCGGGCTGGGCCGAGCGCATCAGGCTGCTGGCGCCTTTTGGCGTCAGAGCCA
ATGTCATGGAAGCCACTGGCAATCCGGGTAGCAAGTTCATGCATTGCCTGCCTGCCTTCCACAACAGTGGCTC
AAAGGCCGGAGCCCGCGTCGCGAAGCAGTTCGGTCTCGACTGCATGGAAGTCTCGGACACGGTTTTTGAATCG
AAAGCTTCTGTGGTGTTCGATCAGGCGGAAAACCGGATGCACACGATCAAGGCCATTTTCATCGCGACCATGG
GGGACCGGGAGACCGATGCAAGAGATAGAGACCCTTAGATGGCACTGAAGGCAAAACCGCATCCGAGCCTGTT
GGCCATTCTCTTCCCCGTCGCCGTCACCGCCGCTGCTGTCTATGTCGTCGGCTGGCGCTGGCCGGGGCTCGCC
GCCGGCATGTCGGGGAAGACGGCATATTGGTTCCTGCGGGCGGCGCCCGTGCCAGCGCTACTGTTCGGGCCGC
TCGCCGGCCTGTTGGCCGTTTGGGCCTTGCCATTGCACCGGCGAAGGCCAGTCGCAATGGCGAGCCTCGCCTG
TTTTCTGACGGTCGCGGGCTTCTACGCGCTCCGTGAATTCGGCCGGCTGTCCCCCTCTGTGGAAAGCGGTGCG
CTATCATGGGACCGGGCGCTGTCCTACCTCGACATGGTCGCTGTCGTCGGCGCGGTCGTTGGCTTTATGGCCG
TCGCTGTCTCCGCGCGCATCTCCACCGTCGTGCCCGAACCGGTAAAGCGCGCTAAGCGCGGAACCTTCGGTGA
CGCGGACTGGCTGCCGATGGCGGCGGCTGGAAAGCTGTTTCCGCCGGACGGCGAGATCGTCATCGGCGAGCGC
TACCGCGTCGACAAGGACATTGTCCACGAACTGCCCTTCGATCCAAACGATCCGGCGACTTGGGGCCAAGGCG
GCAAGGCCCCTTTGCTGACCTACCGCCAGGACTTCGATTCCACCCATATGCTGTTCTTTGCGGGCTCGGGTGG
ATACAAGACGACCAGCAACGTCGTGCCGACGGCGCTGCGTTATACCGGGCCGCTCATCTGCCTCGATCCGTCG
ACCGAGGTCGCGCCCATGGTGGTCGAGCACCGAACCCGCGTGCTCAGCCGCGAGGTGATGGTTCTTGATCCGA
CGAACCCGATCATGGGCTTCAACGTGCTCGACGGGATCGAGCACTCACGGCAAAAGGAAGAGGACATCGTCGG
CATTGCCCACATGCTGCTGTCAGAAAGCGTGCGCTTCGAAAGCTCGACCGGCTCCTACTTCCAGAACCAGGCG
CACAATCTCCTGACCGGTCTGCTCACCCATGTGATGCTGTCGCCCGAATATGCCGGACGGCGGACGTTACGCA

GTCTCCGGCAGATCGTATCCGAGCCAGAGCCGTCGGTGCTGGCGATGCTGCGTGACATTCAGGAGCATTCCGC
GTCCGCTTTCATCCGCGAAACGCTCGGCGTCTTCACCAATATGACCGAGCAGACTTTTTCGGGCGTCTACTCT
ACGGCGTCGAAGGATACGCAGTGGCTGTCGCTCGACAGCTACGCCGCACTCGTTTGCGGCAACGCGTTCAAGT
CGAGCGATATCGTCTCGGGCAAGAAGGACGTGTTCCTGAACATCCCCGCCTCGATCCTGCGCTCCTATCCGGG
CATCGGCCGCGTCATCATCGGCTCGCTGATCAATGCCATGATCCAGGCCGATGGCTCATTCAAGCGCCGGGCG
TTGTTCATGTTGGATGAGGTCGATCTCCTCGGCTACATGCGCCTCCTCGAGGAGGCACGCGACCGCGGCCGCA
AGTACGGCATCAGCATGATGCTGCTGTACCAATCCCTGGGGCAGCTGGAGCGGCATTTCGGGAGGGACGGTGC
GGTGTCGTGGATCGACGGATGCGCCTTCGCCTCCTATGCCGCAGTTAAGGCGCTGGACACGGCGCGCAACATT
TCGGCGCAATGCGGAGAAATGACGGTTGAGGTAAAAGGAAGCTCCCGCAATATCGGCTGGGACACGAAGAACA
GCGCATCGCGCAAATCGGAAAACGTCAATTACCAGCGCCGGCCGCTGATCATACCGCACGAAATCACCCAGTC
GATGCGAAAGGACGAACAGATCATCATCGTCCAGGGCCACAGCCCGATCCGCTGCGGGCGCGCGATCTACTTC
CGACGGAAGGACATGAACGAGGCGGCGAAGGCCAATCGCTTCGTCAAGGCGATCCCTTGAATGGCTGAAAAAC
AACATATCGACTTGGAGGCCGCACCTGCGAGCGCCAAAGACCTGCGTATGCAACTCCTTCAACGACAGATGGA
GGAGATGGAGAAGGAGCGACAGTTCAAAGCGGTACAGGAGCAAAAGCTGACTGACTTTGCCGGCGTATTCCTT
GAGGAGCACGTCACGCCAGAGGAGGTCGCCGTCGTGCGCCGCCTCGTGGCGAACGCCGTACGCGACGGAAAAT
TCGAGGCCATGGTTTACAGCTTCCCCTCGAATCTTTGCACCGATAGTGGCCGCGCAATAAACAGCGCCGACCG
CGATTGGCCGCAGACTCTCCAGGGCAAGGCGAAGGAATTCTACGAACGATATCAAAAATTCGGCAAGCCTCAG
GGCTTCAAGCTGAAAGCTATGATTGTCAACTTTCCGGGTGGAATGCCAGGTGACGTGGGCTTCTTCCTGAACT
GGGCTCCTGATGACGCTTAGGTGCTGGAATTCCTGATCTGCTCGCTGCTCACCATTTTTCCGGACTACCTCGT
CCGCCGCTATGTCCAGGGCAAGCGTGTCGGCCGTGAGATAAATCTCTATTCCATGTGGTTTGAGCTCCGATAC
GGCATAACGGCCTGCCTTATCCTCACCATTTCACTGATCACAATGATCTTCTACTTCCATCCATCTACTAGCA
ACGTCACCGCGGTGTTTCGCACGGTAACGATCATGCCCGAGTCAACCGGGCGAGTAGCAGAGGTTTATGTTGA
CCTCAACGAGAAGGTTTCTGCCGGTGCTCCCCTCTTCCGCCTCGACGATTCCGAGCAACGGGCAGCACTTGAG
ACCGCCCGCCGACGCGTCGCCGAGATCGAGGCAGAGGCAATTGTTGCGCAGAGCGAACTGGCCTCCGCCGACG
GCCTTATCGCCCAAGCCGAGGGCGACTATCTCCAGGCGCTGAATGAACTCGAGACGCAGGTCGAATTGAACCA
GCGCAACCCTAACATCGTCGCAAGGCGCGAAATCGAGCGCCTTCAGGTCGCTGTCGACGGTCGCGATGGTGCG
CGTGCCGCGTCGATCTCCAACAAGCGGACCTTGGAAACGAAAATCGCCTCACTCCTGCCGGCGCAGAAGGCGA
GCGCTGAAGCGGCGCTGACCCAAGCCCAGGTGGAGCTCGACAAGACGATCGTACGGGCTGGAACCGCCGGCGT
AGTCCAGCAGTTCGCGCTCCGCGCTGGAGATATTGTCAATCCGATGATTCGCTCGGCCGGCATCCTGGTTCCC
GAGCAACGCCGCATTGGCTTGATCGCTGGCTTCGGCCAGATCGAGGCACAGGTGATGAAGGCCGGGATGATTG
CCGAGGCGACCTGCATCGGCAAGCCGTTCACCATCATCCCGATGGTGGTGACGGAGGTGCAAGACGTTATCGC
CGCCGGCCAAATTCGTCCGACCGACCAGCTCGTCGACGCGCAGCAACTTGCCAGTCCTGGCACGCTGACTGCC
TATCTGGAGCCGCTCTACGAGGGACAGTTCACCGGCGTACCACCTGGAAGCAGTTGCATTGCCAACGCGTACA
CGAGCAATCACGATGCACTACATGCGCCCGGCATCAGCACACCGCATTGGCTATTTCTGCACGTCGTCGATGC
GGTGGGATTGGTGCACGCCATGATCCTCAGGCTTCAGGCCCTGCTCTTGCCGGTCCAGACGCTAGTCTTCAAG
GGCCATTGAATGGCGAACGACGAACTGAGCGCCGGCTTCACCGGGAAGCCGACCGAACCGCCCACGGGTGTCC
GAAAGATAGCTAAGGTAGCCAGCGGCGCAGTTGGCCGCGAAGCGAACGGTGTCGTGGTAGGGGCCGCAGATCA
CCCATACACTGCAACAGGATTGGCGCTCACAGTCGGCGCCATTGCCTTCGCGGTCGGTTATACTATGGGTCGA
TCGTCAAGCGAAAAGGGCCGAAGTTACTGGCGATAGATGTCGACGCACAAAATTGCGATCGTAGCGATGCAGG
GCGTGCAATTGCTCGATGTGATCGGCCCGTCCGACGTGTTCGCGGAAGCAAACCGCCAGAGTGGCCTCGGCTA
CTACTCGGTAGAGGTCGTCAGCACTTCTGGCAGCCCCGTCAGAGGATCATCTGGATTGGCACTGATTCCCGAT
CGCCTAATCGACGAAGAACCGTCCGAAGTCGATACGCTGCTTGTAGCAGGCGATCCGCTCATCCAGGAGCGGC
CGCTCACCGAAAACCTCCTGCGCTGGGTGAAGCAGACCTCGGCCAGAGCCAATCGCGTTGGGTCCGTCTGCTC
CGGGGCTTTTGTGCTCGGCGAGGCAGGTTTACTTACCGGCAAGCGAGCGACCACGCATTGGTCGCAGGCATCG
GAGTTTGCAAGGCGTTTTCCGGATGTGAAGCTTGAACCAAATCGCATTTTTACAAAGGACGGCAACGTTTGGA
CATCGGCCGGGGTTACCGCTGGGATCGATCTGGCGCTTGCGATTGTCGAGCAAGACCTCGGTTTAGAGATTGC
CCTCAGAGTCGCTCGAGAGCTCGTGGTGTTTCTCAAACGGCCCGGCGGACAGACACAGTTCAGCACGCTCCTG
GCCGGGCAGATGGCCCAGAAGACGCCGATCCGTGCGGCCCAGGACTTCGTCGCCGACAACCTCGCCGCCGACC
TCACCGTGGCGGCCCTGGCGAAGCGCGTGGGAATGAGCGAACGAAACTTTTCAAGGCAGTTCAAACAGGAGGT
CGGCATGACACCGGCCGACTATGTCGAAAGCATGAGGCTAGAGGCGGGTCGCAGATTGTCCGAGGACACCAGC
ATTCCACTCAAGAGGATCGCCATTGATATAGGCTTCAGCGACGATGTCGCCTTCCGGCGATCCTTTGCCCGCA
GATTTGGCACATCCCCGGCTGCCTATCGGAGGAGCTTCGGGTCCTAGATGGGCATGCCCGCGGGAGCGATCTT
TCTCGTCTTCATGACCCTGCAGATTGTCTGCATCGCCGGCGCGCTCCTCTATCCGGACGAGTTCCGCTACCTC
TCGCCGCAGAACCTGACGATCCTGATGAAGGCGATCCCCGTGCTCGGGTGCCTCGCGCTCGGAGCCGGCGTAT
TGATGATCGCCGGTGAATTCGACCTTTCCATCGGCTCCGTGTACACGTTCACGGCCGTGCTGATGGCCTCGCT
CGTCAATGCAGGGCTGAGCGCCTTCATCGCAGCACCTATCGGTATTCTGACCGGCCTGCTGATCGGCTCGCTC
AACGGCCATATCACCCTGCGCTTCGGCCTGCCGTCCTTCATCGTCACGCTCGGTGGGCTCCTCTTCTGGCGCG
GCGCGGTGCTGCTCTACAACGGTGCCGTCCAGGTGCGTTTCGACCCGGAGCCGGTCTTCACCAGCCTCTTTTC

CGGCACTCTGTTCGGCGTGAACGCCGCCTTCATCTGGATCGTCCTCTTCGTCATAGGCTTTCACCTGCTGCTA
CACCGCCACCGCTTCGGCAACCACGTCTTCGCGACGGGTGGAAATCGCGGTGCGGCCGAGGCGATCGGCATCA
ATACCAGCCGCGTCAAACTGATTGCCTTTGCGATTGCGGGCGGCATGGCTGCGGTCGCGGGCATTCTTGCCAC
GGCCCGCGTGGGCAGCGTGCAGCCCGGGCAGGGTGCGGGTCTGGAACTCCAGGCGATCGCCGCCTGCGTCATC
GGCGGCCTTTCGCTACGCGGAGGACGCGGATCGATCATCGGCATCTTTCTCGGCGTGCTGCTCATCCACACGA
TAACCGACGTCCTGCTTCTCCTGCGGGCACCCGGCTTCTATCTCGACATGTTCATCGCCACGCTGATCGTGCT
GGCCGCCATTTTCAACCATCTAATCGAGCGGCGAGGTCTTGCGTGAATGATCAGAGCCTTCGGCTGGAACGAG
TTTCTCGTCATCGTCGCCGCGGCACAGTGGACGATCGCCCTTTCCGCGATCGCCTTTGCCGGCGGCAGCGTCG
GCGGACTGCTGGTCGCATTGATGCGCGTCTCCGAGACGCGAGCGCTGCGCCTTTTCGCCACGGGCTTCATCCG
CGTCTTCCAGGGGACGCCGCTTCTGATGCAGCTCTTTCTGGTATTCTTCGGCATGAAGATCTTCGGCTTTGCG
ATCAATCCCTGGATTGCCGCGACCATTGCGCTCGCGCTCCACGCCAGCGCCTTCCTGGGCGAGATCTGGCGCG
GCTGCATCGAGGCCGTGCCGAAGGGACAGCGCGAGGCGGCGACTGCGCTCGGCCTGCGCTATTTCCGGTCGAT
GCGTCATGTGATCCTGCCTCAGGCGGCCCGTATCGCCGTCGCACCCACCGTCGGTTTCCTGGTGCAGCTCATA
AAGGGCACCTCGCTCGCATCGATCATCGGCTTTACCGAGCTGACCCGGCAAGGGCAGATCATCAACAATGCGA
CGTTCAGTCCGTTCCTGGTGTTCGGTACCGTCGCCGCCGTCTATTTTCTGCTTTGCTGGCCGCTGTCGCTGAT
CGCACGCCGAATGGAAACACGTTTTTCCCGGGCAACGGCACGTTGAATGGGCAACAATGAGAATGGCGGCATA
GGCTTTTGCGTCTTCGTCGTCGTAATTGGATTTAGCACGGGTGCTGTTGCGCAGGAGCCAGCGAATCAATCCG
AAGCTGCGACGAGCCTGGAAGAAATCGTAGTCACCGGCGGACGATCCGCTCAGCAGATTTCTGAAATCGCGCG
AACCATTTACGTCGTCGATTCGGATCAGATCCAGGCCGAGGCCCGGTCGGGCAAGACGCTGCAGCAGATTCTG
GGCGAAACCATTCCGAGCTTCGACCCCGCCAGCGACGGAGCGCGTACGTCATTCGGCCAGAACCTGCGTGGCC
GGCCACCACTGATCCTTATCGACGGTGTCTCGATGAACTCGGCGCGCTCGCTGAGCCGACAATTCGATGCCAT
CGATCCGTTCAACATCGAACGGGTGGAGGTCCTGTCTGGGGCAACTGCAATCTACGGCGGGAACGCAACCGGC
GGCATCATCAACATCATCACGAAGAAGGGCAAAGACGCGGAACCGGGCCTGCATGCGGAAGTGACCGGCGGCA
TGGGCAGCGGCTTTGCCGGCAGCCAGGACTTCGACCGCAATGCGGCGGGTGCGGTCACCTATAACAGCGAAAA
CTGGGATGCCCGCCTTTCGATCGCCGGCAACAGGACCAGCGCCTTTTATGACGGCAGCGGCACGCTGCTGATC
CCCGACATCACGCAGACGTCCACCGCATTCAACGAGCGCATCGACCTGATGGGGTCTATCGGCTACCAGATCG
ACGACGACCGTCGCCTCAAATTTTCCGGGCAATATTTCGACAGCAAGCAGGACTCGGATTACGGGCTTTATTA
CGGGCCCTTCTTTGCAGCGCTCGCCGACCCGAGCCTGTTTGAAACCCGTTCCGGATACGAGTCCGATTTCAAT
CCGCAGACACGCCGCTCGATGTTGAACGTCACCTATACCGATAATGACGTTTTCGGTCAGCAGCTATTGCTTC
AGGGATCTTACCGGACTGAGCGGATAAAGTTCCATCCCTTCCCTGCTTCGGGCAATAGCGAGACGGGTCCTTA
CTTTTACGGCAGTTCGCAGGACACAGATTATTACGGCATCAGGGCTGCCTTGGTTGCGGAACCCACCGACGCG
CTGAAAATCACCTATGGCGTCGATGCGGATATGGATTCCTTCACGGCGCGTCAGAACATCTTCGACATGGTGG
CAGCCGGGCAATCCGGGGGGCTCGATTTCAATACGATCGGCAAGACCGGTCTTTATCCATCCATCGATGTCTC
CACCGTTGCCGGCTTTGCTGAGGCCTCGTACGAGGCTACCGACAGGCTCACTCTGAACGGCGGGGTGCGCTAT
CAGTTCGTCAACACTGAGGTATCCGACTTCATCGGAGCGGCTCAGCAGGTCGCCATCCTGCAGGGCAGAGCAA
CGTCTGCCGACACCATACCTGGCGGCGAAGTCAATTACGACGCCGCTTTGTTCAGTGCAGGCGCAACCTACCA
GTTGACGAATACGCAGCAGGTCTACGCCAATTTCATCCAGGGCTTCGAACTACCAGATCCGGCCAAGTACTAT
GGAATTGGCAACTACTCCTTCTCCGGTGGACACTACACGCTTGTCAATAGCGTGAACGTGGGCGATTCCGCGC
TCGAGGCGATCAAGACCAACTCATTCGAAATCGGCTATCGTCTCGATGACGGCACCTTCAATCTTGAAACGGC
GGCCTACTACTCGCTCTCCGATCGTTCCATCAATCTCAATCGCTCATCGCTTGCCGTTGAGGTCATCGATCGG
GAGAGACGCGTCTATGGCATCGAAGGAAAGGCGGGCGTAAAGCTCGATCATGGCTTCGATGTTGGAGTTCTGG
GGCACTGGGTCAGAACCGAGGTCAAGGGAGCCGACGGTTGGGAAAAGGACTCCGTCGGCAGCGCGAGCGTGTC
CAAACTCGGCGGCTATGTCGGCTGGACCAATGACGCTCTCAGCCTGAGGTTTTCCGGTCAGCACATCTTCGAA
CTCACCGACGCCCAGAATTTCACGATCGACGATTATACGTTGTTTGATCTAACCGGCGGCTACAGGTTCGAGA
ATACCGACACGACGCTGAATTTCGGTATCCATAATGTCTTCGATACTGATTACACCACCGTCTGGGGCTCTCG
CGCCAAAGCCCTTTACGGCGGCCTAGCCGACGAGTCGGTGTTCGACTACAAGGGCCGCGGCCGCACGTTCGCG
GTCTCGCTGACAAAGGTTTTTTAAGTGCACATCCGGTTGCTTGGCGGACTTGAAGTAAGTTCCGGAGATGGCC
AGCCCGTTCAGTTCACAACGCGCAAGGCGGCACTTCTCTTTGCCGCTCTGGTCCTGGCAGGTCGCCGCGGCCA
GCACCGCGAGCTACTTTCCGAAGGGTTTTGGCCGGGACGAGGCGATGCACAGGCACGTAACAGCTTGCGCCAA
GCCCTTGCCGAGATTAGGCGGTGGTTTCCTCCCGGCCGCAATGCCGACATTTCCGTCGAAAGCAATCAGGAAA
CCGTTGCACTGGTGGTTCCTCCAGAGAAGGTGGACATTTGGCTCTTTGAGAGAAAGCTCGCTGAAGGTGAAAC
CGCGGACCTGGCCTTCGCTGCTGACCTCTATCGCGGTGATCTTCTAGCGGGCGTGGCCGTCCCGGACGAGCTG
GAGGAAAGGTTCCGACCATATCGGAGAAGATATCGGGAGAGAGCACTGCAGTTAGTGGAACGAATGAGCCTTG
CCCTGTCCGATGCTGGATCCGCGGCCGAAGCGGCTTGCGAGCGGCTTGCGGAAACGCTGCTCGCCTCGGACCC
GACTGCCGAGCCGGCTCACCGGGCGTTAATGCGCATCCATGCTGTGAGGGGTCATGAAAACGCGGCCTTGCGG
CAGTTTGAGTCCTGCCGGGCGCTGCTGATGAAGCACCTGGAGGTGGAACCGGAGGCGCAGACGAACTCTCTGG
CGGCGTCGCTGCAATTGCGGCAGGGACAGGGACACCGGCAGACCGGGTCGACCTACAATTTCGCACCGCATAC
GCAGCCCATCCGGGCTCCCACGAGACCGCATGACCAGCCGTCGGTGGCGGTGATGCCCTTCCAGAATCTTGGT

GCGGCGGACGACGAGTATTTTGCGGACGGCGTGGTGGAGGAGATCACTGCCGCGCTATCGCGCGTTCGCGACT
TCTTTGTGATCGCCCGCCAGTCTGCCTTCACCTACAAGGGACGCTTCGTCGACGTGCGTGAGGTCGGCAGGGA
GCTTGGCGTCAGTTATGTGGTCGAGGGCACCGTTCGGAGGGGCGGCGACCGACTGCGCATTTCCGTACAACTG
ATCGATGCCGAGACGCGGACACAATTGTGGTCCGAGCGCTACGAGGGCGCGGTCGAAGACATTTTTGCATTCC
AGGACAGGATCGCGGCGCAGGTGGCCGGGGCTATCCATCCAGCCATCCGGGGCGCTGAGATCGAGTTGGCCAA
GCGCAAGCCGCCAGCTAGCCTGCGCGCCTACGACCTCGTCATGCGAGCCTACCCGCACCTTTGGGGCCGCCGC
AAGGAGGCTAACGACGAGGCTATCGAGCTACTGCGGAAAACGATCGCCATCGATCCGAGCTATGGCCGCGCGC
ATGCACTTTTGGCTTGGTGTCACGCCGCAAACGCCGCATATCTATGGACGAAGCAACCAGAACTCGAGTTGGA
AAAGGCGCTAAAAGCCGTTGAGGCTGCGGCTTCGATCAGCAACGATCCAACCGCGTTGACTGCCGCCGGTGCG
GCAATGAGCATTTGCGGCGACCAGGAGCGCGCCAGCATTTTCATCGAAAAGGCGCTCGCGCTTGATCCAAACA
ACGCATGGGCATGGACGCGCCTGGGCTGGATCGCGATTTACAGGGATGACGCCGTGCGCGCAACGGAATGGTT
CGAGCGAGCGATGATGCTTAGTCCGATGGATCCCCTCACATTCAACATGAGAATAGGATTGGCCGCATCTATG
GCCGTGGCCGGTTCTCTTTCCCAGGCGGTCGCTATCGCCCGTGAACTCATCGCCAGTTATCCGAACGTCATAA
TGTTGTATCGTTTCCTTGCCACTTGGTCGGCGATGAATGGGGATCTTGAGACTGCTCGCTGGGCCGCTCGGAA
GCTGCTGGCAGCCCAGCCAGACTTCACGATCGAGAAGTTCCGCTCACTCCCTATGTTTCGAGACATCCCCAAA
TGGGCAGACAAGGTGTCCGAAGCCTTGAGGTTGGCGGGAGTGCCCGATCACTGAATGTCGCATGAAAAGTTCC
TGTCCGCGCAGATCGGCCGCCGGACGCTGCTTGCGTCCATGGCCGCGGCCGGCGCTTCGGCGGGTCTTTCGAC
CCTCGGCGTCAGCCGCGCCTTTGCCCAGGAGCCGGAAAAGCCGGCCGAGATCATCGTTCGCGCCTGGGGCGGC
AGCTGGGTCGATGCGCTCAAGGCTGGCGTGTCCGACAGCTTTACCAAGATGACCGGGATTGCCGTCCGTCACG
ACCTGACCGAGGACAACGAGATCCAGCCGAAGGTCTGGGCCGCCGTTGCCCAGAAGCGCGTCCCGCCGATCCA
CATCAACTGGGATACGACGACGAACGCCACCAAGTCCGCTTTGCGCGGCGTGACCGAAGACCTGTCCGACCTG
CCGAACCTCAAGAACGCCACGGATCTCGCAAAGCCGGTCGGCCTCGACGGCTATCCGATCGTCAACACCTACG
GTTATGTCTACGTGCTCGCCTATCGCCCTTCGGCCTTCCCGAACGGCGCGCCAAAATCCTGGAAGGATCTGCT
CGACCCCAAGCTCAAGGGCCGGATCGCATTGTATAACGACGGGATCGGCTTCCACTTCCCCGCACAAGTCGCC
GGCGGCGGTAAACTCGAGGACATTCCCGCCAACATGCAGCCGGCCTGGGATTTCATCTCCAAGATCAAGGAGC
AACAGCCGCTGCTCGGCGAGGACCCGGATTTTACCACCTGGTTCCAGAAGGGCGAGATCGACGCCGCCTGCAC
CATCTCGACCAATGCGCGCGAAGCCAAGAAGAACGGCATCGAGATCGCCTGGGTCGTGCCGGAGGAAGGCGCC
AAGTTCGACACCGACGGCCTTTGGATACCGAAGGGTCTGCCCGAGAACGAGCTTTACTGGGCCAAGCAATATA
TCAACCACGCCCTGACCAAGGAAGCGCAGCAGATCTGGCTGGACGGTCTCGGCTTGCCCGGCGTCATCCCCGG
CATCACGCCGCCCGCCGATCTCGTGGGCGATCCCTCCTATCCAACAACGGAGGAGGACTTCAAGCAGCTGATC
CGTATCTCGGCCAAGGTACAGGTCGAGAACGAGAGCCAGTGGTTCGCCAAGTTCAAGGAAATCATGCAGGGCT
GAATGCCGCTACATAGTAGGAAAAAGATGATCGGCCTGGCGCTGTGCCTGGGGGCGGTCGTCGTCGTCGTTGC
CGGGTGGACCTGGGCCCGCGAGAGCGGTGCGGCGTCGACCGACAACGCCTATGTCCGCGGGGATGTTACCTCG
CTTGCCCCAAAGGTCGCAGGCTACGTCACGGCTATCGAAGTCGAGGACAACCAAGCGGTACGGGCGGGCGACG
TGCTCTTCCGGATCGACGATCGGGACTACCGCGCAAAGCTTGCGCAAGCGGTGGCCAATGTCGAAGCGGCAGA
GGCACGGCTGACCAACGTCGATGCGGAGATGGCGCTTCAGCATGCCCTGATCCGCCAGGCCGAAGCGCAGCGA
CGTTCGGTCGTGGCCGAGTTGAACCTGGCGGCCAAGGCCTCTGACCGCCGCCGCGAGCTTATCCGCAGCAACA
CGATCAGCCAGGCCCATGTCGACGAAAGCGACGCGGCGAAATCGAGGGCCGAGGCGAACGTGTTGGCGGCTTC
CGCAACGGTGGAGGCGCAGCAGCAGCGCATCGCCGTGCTTGCCGCGCAGCGCGAAGCCGCCGTCGCTGCGGTG
GCACAGGCGGAGGCCGCGCGCGACCTCGCCGGGATTGATCTGGAGAGCACCGTGGTGCGCGCCCCCGTCGGCG
GCGTCATCGGCAACCGGCAGGTGCGCGTCGGCCGGCTCGTTGCGCCGGGCGCTTCCCTGCTCGATATCGTTCC
GCTCGACAATGTATGGATCGTCGCGAATGTCAAGGAAACCCAGCTCGAACATATCCGGCCCGGTCAGCGCGCC
AGCATCACCATCGATGGTTACCCGAGGGGAGCGCTTGAAGGCGTGGTGGACAGTTTTTCACCCGGCAGCGGCT
CTGCCTTCAGCCTGCTGCCCGCAGACAACGCCACGGGCAACTTCGTCCGCGTCGTCCAGCGCGTTCCGGTGAA
GATCCGGTTCGCCGGCAATCCGCTGTCGGGCCGCCTCCTGCCGGGCCTGTCAGCGCGTGTCGAGATCGATCTG
GAGAGCGGTTCATGAATGCTGCGGGAGGATGTGAAGGATCTGCTGTGGTTCATCGAGGTGGCGAGGGAGCAGA
GCTTCACCAAGGCCGCGGCCACGCTCGGCACCTCTCAGTCGACCCTCAGCCATACGATCAAGCAGTTGGAGGC
GCGGCTGGGAGTACGCCTGCTGACGCGGACGACCCGCAGCGTGGCGACAACGGAGGCTGGCGAGCGCCTGCTA
CAGTCGCTCGCTCCAAGATTTGAGGGGATCGAGGCAGATCTTGCAGATCTGGTGGCTTTCCGGGAGAAACCGT
CGGGCACAGTGCGCCTGACGCTCTCTGATCATGCTCTCCAGACGACGGTCTGGCCTAAGCTGAGCCGTGTCCT
GTCCGACAATCCCGACGTCAAGGTCGAGCTCTACAGCGACAACGGGATGCGCAACATCGTCGAGGAGCGTTTC
GACGCCGGCGTCCGGCTCGGCGAGAGCGTCGACAAGGACATGATCGCCGTGCGCATCGGCCCCGACTGGAGGC
TGCGGGCCGTCGCCTCCCCGGAATACCTCGCCAGGCACGGGATCCCCAAGACACCGCAGGACCTCGTCAGCCA
TGTCTGCATCAACACGCGACAGGCAACATGGGGCGGCTTCTATGCCTGGGAATTCGAGAAGGACGGGCGGGAG
CTTCGGGTCCGGGTCGACGGGCAGCTCAGCTTCAACTCCTCGATCTCCCAGATCGACGCCGCCGTTAAAGGAT
ACGGGATCGCCTATATACCGGAGGATCTCCTGGAAGGACATATCCAGGCGGGAGCGCTGCAGGCTGTCCTCGA
CGATTGGAGCGAGCCGTTCACGGGTTACCACCTTTACTATCCCAGTCGACGTCAGATGTCCCCCGCGATGGCA
CTGATCGTAGATGCTCTGAGACATCGGAGCTGAATGCCCAAGGAAGACTTCAACGATCTGCTGTGGTTTCTCG

CCGTGGCCGAGGAGCGCAGCTTCACCAGGGCCGCTGCCAAGCTCGGCATAACCCAATCGACGCTCAGTCACAC
GATCAAGCGCCTCGAAACGAGGATGGGCCTGAGGCTTCTGACCAGGACCACCCGGAGCGTTGCCGTGACGGAA
GCTGGCGAGCGCCTTCTTCGTTCGCTGGCGCCGCGGATATCGGCCATAAGGGACGAAATCGCGGCCCTTATGG
CCTTTCGCGACAAACCGTCCGGCTCGGTCAAAATCACTTTGTCCGACCACGCCCTGGAGAGCCTCGTCTGGCC
GAAACTACAGCCGGTTCTTCTGGAATACCCCGACATTCGGCTCGAGTTGAGCAGGGACAACGGCTTCAGGAAC
ATCGTGGAAGATGATTTCGACGCAGGCATTCGCCTCGGCGAGAGCGTCGAAAAGGACATGATCGCGGTCCGTA
TCGGACCGGACTGGCGGCTTCTGGCAGTGGCTTCGCCCGACTACCTGGCAGATCGTCCGCTGCCAGAGCATCC
ACAGGAACTGATCCAGCACAATTGCATCAACTCGCGCCAGGCAACAGGCGGCGGACATTATGTATGGGAGTTC
GAGAAGAACGGGCAAGAGCTCCGCGTCAGGGTCGACGGACAACTCACCTTCAATAGCTCATACCCCATGGTCG
ATGCCGCCCTGAAAGGTCACGGCATAGCCTATCTGCCGGAAGATCTCGTCGCGGGGCACATTGCGGCAGGACG
CCTGGTAGCGCTGCTCGAGCCCTGGTCGCCTCCATTTGCCGGGTACTACATTTACTACCCTAGCCGACACCAC
AACTCACCCGCATTCAAGGTGATCGTGGACGCCCTGCGACACCACGGCCCACCAGATTCCGAGGGAACGCGAG
ATGCTTAAATGGGAAACAAATCTGTTCGCGTCATCGCGGGAGCAATGGTGGTCGCCGGCTGGGCCGGCTACGC
CGCTGCGCAAAGTGCCTCGGAAATCAAGATCGTCCTGCCGGAGCAGCCGGCCAATCTCGAGCCCTGCGGTACC
ATCATCACCAATGTCGGCCAGATATTGAGCCGCAACGTGGTCGAGCCGCTGACCATCATCGATCCGAAAAGCG
GCCAGCCGACACCCGGTCTTGCGACCGAGTGGAAGCAGACCGATCCGAACACCTGGCAGCTCAAGCTGCGCGA
AGGCGTCAAGTTCCAGGACGGAGCCGCCTTCGACGCAGAGGCGGTCAAATTCTCCATCGAGCGGATGACCGGC
GGCAAGCTGACCTGCAGCAACATTGCCAAGTTCGGCGATGCCAAGCTGACGGTCACGCCGCTCGATGAGCTGA
CGGTCGAGATCAAGTCGGACAAGCCGCAGCCGATCCTGCCGACGCTGCTCAGCGTGGTCATGATCGTCTCGCC
GAACACGCCGGCGGACAAGGCCGTCAACGACCCGGTCGGTACCGGGCCGTTCAAGCTTTCGAGCTTCACGCCG
CAGACAGTCGTGCTGGAAGCCTTCGACGGCTACTGGGGCGAGAAGCCGGCCATCGCCAAGGCCAGCTATGTCT
GGCGCCCGGAATCCTCGATCCGCGCCGCCATGGTGGAAACCGGCGAGGCCGACCTGACGCCGTCGATCGCGAT
CCAGGATGCGACCAATCCGGAAACGGACTTCGCCTATCTGAACTCCGAAACTACCGCGATCCGCATCGATGCC
GGTTTTGCTCCGCTCGACGACGTCCGAATCCGCAAGGCGCTGAATCTGGCGATCGACTGGGATGGCCTCGCGC
AGCTCTTCGGCGAGGACGTGCAGCGGGCTTCGCAGATGGTCGTCACCGGCATCAACGGCCACGACGACAAGCT
GGCGCCCTGGACGTTCGATGCCGAAAAGGCGCGTGCGCTCATCGCCGAGGCCAAGGCCGCGGGCGTTCCGGTC
GATACCGAAATCGAGCTGATCGGCCGCAACGGCATCTACCCCAACGGTACGGAAGCCATGGAAGCCATGATGG
CCATGTGGCAGGATGTCGGCCTGAACGTGAAGCTGACGATGCTCGACGTGAATGATTGGCTGCGCTACCTGCA
GAAGCCCTTCCCGGAGAGCCGCGGGCCGAACCTGCTGCAGATGATGCATGACAACAACAAGGGTGATGCCGCC
TTCACCATTCCGATCTTCTACACCTCGGCCGGAAGCTATTCGACCTTCAGCGACGCGGCACTCGACAAGGAGG
TCGCCGATGCCATGGCTGCCACCGGTGAGGACCGTACGGCAAAGTTCAAGGCGATCTTCGCGAAGGTGCATGA
CGATCTCGCGGTCGACATCCCGATGTTCCACATGATCGGCTACACCCGGGTCGGCAGCCGCCTGGAGTGGAAG
CCGGACATCACGACCAACAGCGAGATCCCGCTCGCCAATATCGCTCTCAAGGACTGAATGTCGACATTCATCA
AGAACTTCATCGAGCGTGAGACGATCAGCCGTCGCCACTTTCTGCTTGCCTCGGCCGCAGGTCTGGGGGCAGC
GGTCCTGCCAAACCCGCTTGGCGGCAGCGCGCGCGCTGCATTGACGGATCCCGCGATCGCCTGGAGCTATCGC
GACCGCGCTTCGGCCTATTGGAACTCGGTCGTCTCCGGCGGCGAAGCATTCGTCGAATCGCTCGGCAAGCCGA
AGAGCGCGCTCGTAAGTCTGATCAACGAAGGATCATCCGAGAAATCTCTGGCCGACATCAAGGCCTTCCTTGC
CAAGAACAATGGAAATTGCGCCCTGGCCTGCGATGCCAATGACAGCCCGAATGCGCGGCCGATCGTGGAGGCG
GTGGCGGCTGCGGGCGGCTACATCTCCACGATCTGGAACAAGACCGATGATCTCCATCCATGGGATTTCGGCG
ACAACTACGTGTCGCACATGACCTGGTCGGACGAGAAGCCCGCCGAAGAAACCGCGCGCATCCTGTTCGAAGC
CATGGGCGGCGAGGGCGGTGTCGTCCACCTGGGCGGCATTGCCGCCAACAACCCTGCGGTCGAGCGGCTCAAC
GGTCTGAAGAATGCGCTGAAGGACTTTCCCAACATCGAACTGCTCGACGCGCAGCCGGCGGACTGGGACACGC
AGAAGGGCGCGGCTTTGATGTCCTCCTTCCTGACCCGCTACGGCGACAGGATCAAGGGCGTGCATTGCGCCAA
CGACAACATCGCCTACGGCGTCATCGAGGCGCTGCGCGCCGAAGGCATCGAGGGCATGCCGATCGTGTCCTAT
GACGGCAATCCCGAAGCGGTGCAGATGGTCATGGACGGCCAGCTGCTCGCGACGGTCTTCACCAATCCGCACT
GGGGCGGTGGCATCACGTCGGCGCTCGCCTACCATGCGGCAACCGGCAGCTTCAAGCCGTCGGAAGAGCCGAA
AGAACATCGCGAGTTCTACGGACCGACCGTCCTGGTCAGCAAGAAGGATGCCGAGGAGTTCAAGGCCAAATAT
CTGGATTCGGTACCGAAATACGATTGGGCCGACTTCTGGGGTCCCTCGAACGGACAAATTCAGTATCGGTCCT
GAATGAAGCGCATCTACGTCGTTGGCACGGCCGACACGAAGGGGGGAGGAACTCGTTTATCTTGCGTCCTGCGT
TGAGGCAGCGGGCGGCCGGCCCGTTTTGGTCGATGTCGGCACGCGTCGCCCCACGGTTCTGGTCGACATCTCC
GCCGAAACCGTGGCCGCGGTGCATCCGGGCGGCGCCGCCGCGGTGCTTTCCGGCAACGATCGGGGAACAGCGA
TCGCTGCCATGGGCGAGGCCTTCGCAAGGTTCCTGCCCGCCCGGGACGACGTCGCCGGCGTGGTGGGCATGGG
TGGCGGCGGCGGGACGTCGATTATCACCGCCGGCATGCGCAGGCTGCCGCTCGGGCTACCGAAGGTCATGGTC
TCGACGCTCGCGTCGGGCGACGTGGGACCTTATGTTGACGTCTCAGACATCATCATGATGCCGTCGGTTACCG
ACATGGCCGGGCTCAACCGCGTGAGCCGCGTGATACTAAAAAACGCGGCCGAGGCGATTACCGCCATGGCGAA
CAGGCCGGCGGAGGAAACAGCCTCGAAGCCCGCTATCGGCCTGACCATGTTTGGCGTGACGACGCCCTGTGTG
ACTGCCATCGTTGAACGACTGAAGGCCGACCATGACTGTTTGGTTTTCCACGCCACAGGCACCGGCGGACGAG
CAATGGAGAAGCTTGCAGACAGCGGGCTTCTATCGGGTGTGCTCGATATCACGACAACGGAGGTGTGCGATCT

TGTCTTCGGCGGCGTGCTGCCGGCCACCGAGGATCGTTTCGGCGCAATTGCGCGCACCGATTTGCCTTATGTC
GGCTCCGTCGGGGCTTTGGATATGGTCAATTTCTGGGCACCAGAGACAGTACCGGAGCGATATTCCGGCCGGC
TCTTGTATCGCCACAACCCCAACGTCACGCTCATGCGCACGACTCCGGAGGAATGCGCCGCGATCGGAAGGTG
GATTGGCGCAAAGCTGAACCTCTGCAGCGGACCTCTCCGGTTCCTGATCCCGGAGCGAGGCGTGTCGGCGCTG
GATATCGAGGGCGGCGCCTTCTTCGATCCTGCGGCGGATGCGGCGCTCTTCGAAGCATTGGAGACCACCGTCA
ATAGAAGTGACCGGCGCCGCATCGAACGCCTTCCGCTCCACATCAACGATCCGCAGTTCGCCGAGGCCGCAGT
CGCCGCCTATCGAGACATTGCCAACCCCTGAATGAAATATCAAACCCAGAAGGTCGCGATGCTGTATTTCTAC
GGTGCGCTCGGCCTGTTTCTCGCCCAGATCCTCTTTGGCGTGCTTGCCGGCACCATCTACGTGCTGCCCAACA
CGCTCTCCGAACTGCTGCCGTTCAACATCGTCCGCATGGTGCACACCAACGCGCTGATCGTCTGGCTGCTGAT
GGGTTTCATGGGTGCCACCTATTACCTCTTGCCGGAAGAGGCGGAGACGGAGCTCTACAGCCCGAAGATCGCC
ATCGCCCAGTTTTGGATCTTCCTGATCGCGGCGGCCGTTGCCGTCGTCGGCTACCTCCTCCACATCCACGAAG
GCCGGGAATTCCTCGAGCAGCCTTTCGCGATCAAGGTCGGCATCGTCGTCGTCGCGCTGATGTTTCTCTTCAA
CATCACCCTGACCGTACTGAAGGGCCGCAAGACAACGGTGACCAACATCCTGATCTTCGGCCTTTGGGGCGTG
GCGATCTTCTTCCTGTTCGCCTTCTACAACCCGGTCAATCTCGCGCTCGACAAGATGTACTGGTGGTATGTCG
TGCATCTCTGGGTCGAAGGGGTCTGGGAGCTGATCATGGCTTCCGTCCTGGCCTTCTTGATGATCAAGCTCAA
TGGCATCGACCGCGAGGTCGTCGAAAAGTGGCTCTATGTCATCGTCGGCCTGGCGCTCTTCTCAGGCATCCTC
GGCACCGGCCACCACTACTACTGGATCGGCGCGCCCGGCTACTGGCAATGGATCGGCTCGCTCTTCTCCACGC
TCGAGGTCGCACCCTTCTTCACCATGGTGGTCTTCACCTTCGTCATGACCTGGAAGGCCGGCCGCAAGCATCC
GAACAAGGCCGCGCTCCTCTGGTCGATCGGCTGCTCGGTGATGGCCTTCTTCGGCGCCGGCGTCTGGGGCTTC
CTGCATACGCTTTCGTCGGTCAACTACTACACCCATGGCACCCAGGTCACCGCCGCGCACGGGCATCTCGCCT
TCTTCGGCGCATACGTCATGCTGAATCTGGCTGTCATGGCCTATGCGATACCGGAGATTCGGGGGCGAGCGCC
CTATAACCAGTGGCTGTCGATCGCCAGCTTCTGGGCCATGTGCAGCGCCATGTCCGTCATGACCTTCGCGCTC
ACCTTCGCCGGTGTCGTTCAGGTCCACCTGCAGCGCGTCCTCGGCGAGAGCTTCATGGCCGTGCAGGAGCAAC
TGGCTCTCTTCTACTGGATCCGCCTCGGGTCCGGTGTCGTCGTACTCATCTCGGCGCTGATGTTCTTCTGGGC
AGTCCTCGTACCCGGCAAGAGCGCTAGCCCGGCCCTCAACCAAGCCGTCCAACCCGCCGAATGAATGACCGGC
GAACTGACGCGGCGTGAAATGTTGAAGGCGCATGCGGCCGGCATCGCCGCAGCTACGGCCGGGATCGCGCTTC
CGGCGGCGGCGCAGCCCGTGCCGGGCGGCGTCGAAGCACTTCAGATAACGTGGTCGAAGGCGCCCTGCCGTTT
CTGCGGTACCGGCTGCGGCGTCATGGTCGGGGTCAAGGAGGGACAGGTCGTCGCCACACATGGCGACATGCAG
GCTGAGGTGAACCGCGGCCTCAACTGCATCAAGGGCTACTTCCTGTCCAAAATCATGTACGGCACCGACCGTC
TGAAGACGCCACTTCTGCGCAAGCGCAACGGCGCCTTTGCCAAGGACGGCGAGTTCGAGCCGGTGAGCTGGGA
CGAGGCCTTCGACGTCATGGCGGAGCAGGCGAAGAAGGTGCTGAAGGACAAGGGCCCGACCGCCGTCGGCATG
TTCGGCTCTGGGCAGTGGACCATCTTCGAGGGTTACGCGGCCACGAAGCTTATGCGCGCCGGGTTCCGCTCCA
ACAATCTCGATCCCAATGCCCGCCACTGCATGGCCTCGGCAGCCTATGCTTTCATGCGCACCTTTGGCATGGA
CGAGCCGATGGGCTGCTACGACGACTTCGAGCACGCGGACGCGTTCGTGCTTTGGGGCTCGAACATGGCGGAG
ATGCACCCGATCCTGTGGACGCGCCTCGCCGACCGTCGGCTCGGGCACGAGCATGTGAAGGTCGCGGTGCTGT
CGACCTTCACCCACCGCAGCATGGATCTTGCGGATATTCCGATCGTCTTCAAACCGGGGACCGATCTGGCGAT
CCTCAACTACATCGCCAATCACATTATTCAGACCGGCCGAGTCAATGAGGATTTCGTCAGGAAGCATACGACC
TTCATGGTCGGCGCGACCGACATTGGCTATGGCCTGAGGCCCGACAATCCGCTCGAGGTCAAGGCCGTGAACG
CCAAGGATGCGGCGAAAATGACGCCGAGTGACTTCGAGAGCTTCAAATCCTTCGTCTCGGAGTACACGCTCGA
CAAGGTGGTCGAACTCACGGGGGTCGAAGCCGGGTTCCTAGAACAATTGGCCGACCTTTATGCCGATCCGAAG
CGCAAGGTCATGTCGCTCTGGACTATGGGGTTCAACCAGCATGTGCGCGGTGTTTGGGTCAACCAGATGGTGT
ACAATCTCCATCTCCTGACCGGGAAGATCTCCGAGCCCGGCAACAGCCCGTTCTCGCTAACCGGTCAACCTTC
CGCCTGCGGCACGGCACGCGAGGTAGGCACCTTCGCGCACCGCCTGCCAGCCGACATGACCGTGACCAATCCG
GAGCACCGCAAGCATGCCGAGGAGATCTGGCGCATTCCCCACGGCATCATTCCTGAGAAGCCCGGATACCATG
CCGTCGAGCAGGACCGAATGCTGAAGGACGGCAAGCTCAATTTCTATTGGGTGCAGGTCAACAACAACGTCCA
AGCGGCTCCCAATACGCAGAACGAGACTTATCAGGGCTACCGCAATCCGGACAATTTTATCGTCGTCTCCGAC
GTCTATCCGACGATCACGGCGATGAGCGCCGACCTCATCCTGCCGGCTGCCATGTGGGTGGAGAAGGAGGGCG
CCTACGGCAATGCCGAGCGTCGTACCCATGTCTGGCACCAGCTCGTCGATGCGCCCGGCGAGGCGCGTTCCGA
TCTCTGGCAGATGGTGGAGTTTTCCAAGCGCTTCACCACCGACGAGGTCTGGTCGACCGACATCCTCGATGCC
AATCCCGGCTATCGTGGCAAGACCCTTTACGACGTGCTCTTCAAGAACGGCAATGTCGACAGCTTCCCGGCAA
GCGAGATCAACAAGGAATACGCCAACCGGGAGGCCGAGGCCTTCGGCTTTTACATCCAGAAAGGCCTGTTCGA
GGAATATGCCTCCTTTGGGCGTGGACACGGGCATGACCTGGCGCCCTATGACCGCTATCACGACGAACGGGGA
CTCCGCTGGCCTGTCGTCGACGGCAAGGAAACGCTCTGGCGCTACCGGGAAGGCTACGATCCCTACGTGAAGC
CGGGGGAGGGCGTCAAGTTCTATGGTCGGCCGGACGGCAAGGCGGTGATCCTCGCCGTTCCTTACGAGCCGCC
GGCGGAATCTCCCGACGACGAATACAATGTGTGGCTGGTGACGGGCCGCGTCCTTGAGCACTGGCACTCCGGC
TCGATGACGATGCGGGTGCCGGAACTCTACAAGGCGTTCCCCGGTGCCGTCTGCTTTATGAATGCCGGTGACG
CCCGCGATCGCGGCATCAACCAGGGGGCCGAGGTCAGGATCGTTTCCCGCCGCGGCGAGATTCGCGCCCGCGT
CGAGACGCGCGGCCGCAACCGCATGCCGCCGGGCGTCATCTTCGTACCCTGGTTCGATGCCAGCAGGCTGATC

AACAAGGTGACACTCGACGCAACCGATCCCATCTCCAAACAGACGGATTTCAAAAAATGCGCGGTCAAAATCG
TCTCTGTCGCATGAATGCTCCACCAACCCAAAACCAAAGCTACAGTCGGACGGCTCGATGCCGAAACCGTCAA
TGCCGCCCGCGTGCGAGGTCCGCTTTACGAGAAGCGTCGGAAGATCTTCCCGAAGCGGGCCGAGGGTCGTTTC
CGCCGGTTCAAATGGCTGGTGATGCTGGTGACGCTCGGCATCTACTATCTGACGCCGTGGATCCGCTGGGACC
GCGGGGCGCATGCGCCGGATCAGGCAGTCCTTATCGACCTTGCTTCCCGGCGCTTCTATTTCTTCTTCATAGA
GATTTGGCCGCAGGAGTTCTTCTTCGTCGCGGGGCTCCTGGTGATGGCGGGCTTCGGGCTTTTCCTCGTGACG
TCGGCAGTTGGGCGGGCCTGGTGCGGCTACGCCTGCCCGCAGACCGTCTGGGTCGATCTCTTCCTCGTCGTTG
AGCGCTTCATCGAGGGCGACCGCAACGCCCGCATGCGCCTCGACGCCGGACCGTGGAGCCTCGACAAGATACG
CAAGCGCGTGGCCAAGCATGCCATTTGGGTGGCGATCGGCGTTGCAACCGGCGGCGCGTGGATATTCTATTTC
GCCGATGCGCCCTCGCTCATGTCGAGCCTAGTCGCGCTCGATGCGCCGCCGGTCGCCTATACCACGATCGGCA
TTCTGACCGCGACCACCTACGTCTTCGGCGGACTGATGCGAGAGCAGGTCTGCACCTATATGTGCCCATGGCC
GCGCATCCAGGCGGCCATGCTAGACGAGAACTCGCTCGTCGTAACCTACAACGACTGGCGGGGCGAGCCGCGC
TCGCGGCATGCGAAGAAGGCGGCTGCGGCCGGCGAGGTGGTCGGGGATTGCGTCGACTGCAACGCCTGCGTTG
CCGTCTGTCCCATGGGCATCGACATCCGCGACGGCCAGCAGCTCGAATGCATCACCTGCGCGCTCTGCATCGA
CGCCTGCGACGGTGTGATGGACAAGCTCGGCCGCGAGCGCGGGCTGATCTCATACGCGACGCTCAGCGACTAT
GCAGCCAATATGGCCCTCGCGACGAGTGGCGGGACTGCGGCGATCGATCCGAGCCGCGTGCGCGACGCCCATG
GCGCTTTCCGTGACAAGGTGAGGCATCTCAACTGGCGCATCGTCTTCCGGCCCCGCGTTCTCGTTTATTTCGG
CGTCTGGGCGACGGTCGGATTCGGTCTTCTTTTCGGCCTGCTGGCGCGCGACCGGCTAGAACTGAACGTCCTG
CACGACCGCAACCCCCAGTTCGTCGTCGAATCCGACGGCTCGGTGCGCAACGGCTACATGGTCAAGCTTCTCA
ACATGATCCCAGAACAGCGCACCATTAGCCTGACGATCGAGGGTATGCCCGCCGCAACCATGCGCGTGGCCGG
ACAGGCGACGGGCGACGGACGCCGGGTCACGATCGGTGTCGAGCCGGACAAGGTCACACCGCTCAAAGTCTTC
GTCACGCTGCCGAAGGGCAGATTTGCCGAGGCGGAAGAGGGCTTCTCCCTCATTGCGGAGGATCCGTCCAGCC
ACGAACGCGATGTGTATCAGGCCAATTTCAATCTACCGGGAGCTGCAGGACGATGAATGGCTCGATTGACCAT
AAACGGCCGAACAAGGGATATCGAAGTGGATCCGGAGACGCCACTTCTCTGGGTGCTACGTGAACAGGCGGGC
CTCACCGGCACGAAATTCGGCTGCGGGGTTGCGCAATGTGGCGCCTGCACCGTGCTTATCGACGGCGTGGCGA
CGCGTTCCTGCACCATGCCGGCAAGCGCGATCGCGCAGGATCAACAGATCGTCACGATCGAGGGCCTGTCCGA
GGACGGCTCTCATCCGGTCCAGCAAGCCTGGCTTGCGCTCGACGTTCCGCAATGCGGCTACTGTCAGGCCGGC
ATGATCATGGCGGCTGCCGCCCTGCTCAACAGCACACCAACCCCAAGCGACGACGATATCAGCGCGGAAATCA
CCAATATCTGCCGCTGTGGCACCTATAACCGCGTCCGTGCGGCGATCAAGCTCGCGGCGGAAACCATGAGCAC
GTCCCAGAAAGGATGAATGCAATCGACGCCTCACATTGCCGTTGTGGATGATCATCGCGATATTCGCGACCTG
GTAGGAAAATATCTGACACAACAGGGTTACCGCGTTTCCCTCGCGGAAAGCGGCGAAGCGCTTCGACGTCTTC
TGGAAAAAGGCGCCCCAGATCTCGTCGTGCTGGACATCATGATGCCCGGCGAGGATGGATTGACCGTTTGCCG
TCAGTTGCGCACGACAACGGAGCTTCCGGTCATCTTTCTGACAGCGATGGCGGACGGGACGGACCGGATCGTC
GGCCTGGAACTCGGCGCCGACGATTACATCACCAAGCCGTTCAACCCGCGCGAACTCCTGGCAAGAATCCGGG
CGGTGCTGCGGCGTGTCAACAGCCTTCCTCCCCGGCGCGGGAGGATCAAGGCCAAGACCATCCGCTTCGATAT
CTGGGAACTCGACACTGGACGCCGGGAGCTCGTCGGACGCGATGGCGTCAGCGTGGCGTTGAGCACGGCGGAG
TTCCGGCTGCTCAATGCCTTCCTGGAACACCCCGGTATGGTCTTGAGCCGTGACCAGCTACTTGATCTGACCG
CAGGGCGAGCCGCCGAGAGCTTCGATCGCAGCATTGACAACCAGGTCAGTCGGTTGCGCAAGAAGATCGAGAG
CGACCCGAAAAATCCATCTTTGATCAAGACGCACTGGGGTGGAGGATACAGCTTCGTGGCGGAGGTAGAAGCC
TACTGAATGGCCACCATCACCCATGTCTTCACCATCAACCATGTCGCCGAAATGCTCGGCGAAGACCGGGAAC
TCCTGGAGGCGATCGTCAGCAATTCCGACAATTTGACCTATGGAAGTATCATCAGCGTCGTACACGGTGACGA
TGAGACAATCACCGCACTGACCGACGATGGCATGGATGAGCTGAGGCAAATGCTTGCCGATGCGCGCCGTTCG
CCTGAAGCCTGGAACGACTTCCTCGACTGCTTCGTCGACGACGAAGAAGTCATCGCTCGCGTCAAGGCGAAAT
CTCCGCGGTAAATGACAGAAACGGCCGCTTCGTCAATAGATAAGCAAGCTCATCTGTTCACCATATCTAATGC
GAGCGGCGGTGAGTACGTTGAGGGTTGGCGCGTCACAGTTCTGCTTGTTACGCTATTTCTTGTCAGCGCCACT
TCCCAGATCGACCGGACGCTTCCCTTCGTCCTCGCCGAGTCGATAAAGGAAGAGCTCGATCTGAGCGATACCG
AAATGGGTTTGCTCACCGGCATCGCCTTTGCGGTATGTTACACGCTGCTGTCGATGCCGCTCGCCCGCTCCGC
TGATCGAGGATCGCCCCGGTTCGTCCTCGTTTTGTGTATCCTATTCTGGAGCGCGATGACATTGCTCGGCGGT
CTCGCCATGAGTTTCCTGTTCCTCGCCTTCACCCGCTTCGGCGTCGCGTTCGGCGAGGCCGGTGGGACGCCCT
CCAGCCACGCGATCATCGCACGCAAGATCCCGCCCGAGCGCCGCGGGCTGGCGCTCGGCATTTTTTCCATGGG
AATTCCACTCGGCACCATGGTGGGTTTCGCTGTTGGCGGCGCTATCGGCGATACGCTCGGCTGGCGCACAGCG
CTGATAGGCGCAGGCGCCATTGGCGTGCTGATCGGTCTGCTGGCGTTCGTCGTCGTCGGTCCGACCCCACCAC
GCAAGAGCTCGGCCGCCAGTAGCGAACCTTTCCTGCGGTCGAGCTTGCAACTGCTTTCGTCCCCGTCATTTCG
CTGGCTGTTCATCGGCTCGGTCGCACTCGGCTTCGCCGCAGCGCCGTTCTACGCTTTTGCAGTTCCCTTCCTA
ATTCGCACTCATGACTACAGCGCCAGCGAGGCAGGACTTGCGTTCGGGCTGCTGCAAGGCCTGATGGGGATTT
GCGGTACGCTGATCGGCGGACGCCTGTTCGACCGCGCCGTGCGCTCCGGCACCGGCCGGGTCCTCGGACCGCC
CGCTATCCTCTTCATGATCGCAAGCCTGACGACGACAGCTGCGCTGTTCGCGCCGGTCGGGTGGCTTTCCATC
CTACTCTTCGTGCCCGGCATGCTGTCCTTCGCATTTCTGCTGCCCTGGTGTTTCGGCGCCGCGCATCTCGTGG

CGGGCAAGGGCAAGGAAGCGATGGCGACGAGCCTTGCGATGATCGGCTCCGGGCTCCTGGGGCCCGCTCTCGG
TCCTCTCATCGTCGGCATGATCAGCGACGCGGCGACCGCGGCACAGGTCTCCAATGGCTTGGGGCTCGGTTTG
GTGACGGTCCCGATCGCAAGCATCCTGACCGGGATCGCCATGCTGATTGCAAACCAGCGCATTGCTGCGGTCC
TTCGAAAACACGACGTCTCTTAAATGACCAAGTGGACGAGCAAGAACATTCCATCGCAGCATGGCCGTACAGC
GGTTGTCACCGGCACCGGAGGATTGGGCTACGAGGGCGCGCTGGCCTTGGCCCGAGCCGGTGCAAGCGTCGTG
ATCGCCGGGCGCAATCCCCAGAAGGGGGCTGAAGCGGTGGCGGCGATCAGACAAGCCGTGCCGGGCGGACAAG
TGCGGTTTGGCAAGTTGGATCTCGCCAGCCTCGCGTCGATCGCTGAGTTCGCCGCGCAGCTTGCACGGGACCA
GGAGGGTCTCGATTTCCTGATCAACAATGCGGGCGTCATGACGCCGCCGGAGCGGCGCCAGACGACCGACGGC
TTCGAATTGCAGTTCGGCACCAACTATCTCGGCCATTTCGCCCTGACCGGACATTTGCTGCCATTGCTGAAGA
AGGGGCGAAATGCGCGCGTCGTCACCCTCGGCAGCGTGGCGGCGAGGAGTGGAGCGATCAATTTCGATGACCT
TCAAGCTCAGCGCGGCTACAAGCCGATGCCGGTTTACAGCCAATCGAAGCTCGCCTGCGTCATGTTCGCGTTC
GAATTGAGCCGGCGCAGCAAGGCGGCCGGGTGGGGCGTCGAGAGCATAGCCGCGCATCCCGGTATCTCGCGCA
CCGATCTGCTTCCGAACGGCGCGGGGCAGACGAGTGCTGCCGGGATGGCACGCCGCTTCTTACCGTTTCTGTT
CCAGCCCGCGTGGCAGGGTGCGCTGCCGACGCTGTATGCGGCGACCGATCCGGCGGCGCGCGACGGCGCCTAC
TATGGACCTGACAGGCTGGGCGGGACGCGTGGCTATCCGACCGAGGAGCAGCCTCCCAAACAGGCGCTCGATA
CTTCAGTGGCCGCGCGCCTCTGGGACGCGTCTCAGGAACTGACGAGCGTGACGTTCGTGTGAATGCCTAAGAT
GAAAGCCGCGATCTTCGTCGCGCCTGGTCGTATTGTACTTGACGAGAAGCAGATCCCCGACGTCGGTCCGCTC
GACGCGCTCGTGCGGATCACGACCACCACAATATGCGGCACCGACATCCACATCTTAAGGGGTGAGTATCCGG
TCGCACGCGGCCTGACGATAGGGCACGAGCCGGTGGGCGTGATTGAGAAGCTCGGGTCCGCCGTGCGGGGATA
CAGCGAGGGTCAGCGTGTGATCGCCGGGGCGATCTGCACCTCGGGCCACAGTAATGCGGCCCTGTGCGGCTGC
CATGCCCAGGACGGCCCCGGGACGAACCACGGTTGGAAAGGCATGGGCGGCTGGAAGTTTGGCAACACCATTG
ACGGTTCGCAGGCCGAGTACGTCCTCGTGCCTGACGCCATGGCCAACCTGGCTCCAGTGCCGGACGGACTAGC
CGACGAGCAGGTGCTGATGTGCCCCGATATCATGTCCACGGGCTTCTCCGGGGCGGAGAGCGGTGCCGTCCGC
TTTGGCGACGCCGTGGCGGTGTTCGCGCAGGGGCCCATTGGACTGTGTGCGACCGCGGGTGCGCGTCTCATCG
GCGCAACGACGATTATCGCGGTCGAGAGCGTCCCCGCCCGCATGGAGATGGCCCGCCGCATGGGCGCCGACCA
TGTCGTGGATTTCACGGTATCGGACCCCACTGCAGAAATCATGCGTCTGACCGACGGGCGCGGGGTAGATGTG
GCCATTGAGGCGCTCGGAAGACAGGAGACCTTCGAAGGTGCTCTTCGCGTCCTTAGGCCCGGAGGTACGCTGT
CGTCCCTTGGCGTCTATTCAGGTGATCTAAGAATTCCGCTCGACGGCTTCCTCGCAGGTTTGGGAGACCATAC
TATTCGGACCACCCTCTGCCCGGGCGGCAAGGAACGCATGCGTCGTTTGATGGAGGTGATCGCATCCGGCCGC
GTCGACACGCGTCCTCTGGTGACTCACCGCTTCAAGCTGGACCAAATCGAGGAGGCATACGACCTTTTTGCAA
ACCAGCGTGACGGGGTGCTCAAGGTCGCGATCAATCCATGAATGGATGACGACCTCTCTACTGGGCTTGGCCA
GAAGGAGGCGGAGGTCAGGCTCACGCAATTTGGGCCCAATATGCTTCCCGAACCACAGGCGTCCTCCCTATTC
GCAACATTCTTGCGCCAGTTTCGCAGTCCGCTGATCTATATTCTACTGGCCGCCACGCTTGTGTCGCTCGCGC
TCGGCGATGTGCGCGACGCTCTGTTCATCGCAATCGTCCTCGTCGCGAACGGCACTATAGGCTGCATGCAGGA
ACACTCGGCCGGCAAGGCGGCCCTCGCGCTGCGCAAGCTGGAGCAGCCTAAGGCCACCGTGGTCCGCGACGGA
CACGTGCAGGAGATCGACGCCCGCCTCCTGGTACCGGGTGATCTGGTCCTGATTGAAGCCGGCGGACGCGTGC
CGGCGGATCTCCGGCTTCTCTCAGCCACCGATCTTGTCTGCGACGAATCCCTGCTGACCGGAGAATCGGCACC
GGTCCATAAAACCCTAACTAGTGTTGACGCCACTCCCGAGGTGAATGCCCGCCTCATGGCCTTTGCGGGAACG
CTCGTAACGCGCGGCCGCGGACGGGGGTCAATTGCAGCGACGGGTGCGGCTACCGAGATTGGAAAAATCGCGG
CCGAGATCGGCAAGGCATCCGTCTCCAAGCCCCCTTGATGATCAGAATGGAACGCTTTTCGCAGTCCATCGC
CTGGGTCGTCGCTGCTGCCGTAGTGCTTCTCATTCTCGTCGGGATCGCTCGGTCGATGTCGCCGAGCGATCTC
TTCATGATGTCGGTTGGCCTGGCGGTGAGCGCGATCCCGGAAGGGCTGCCGATAGCGATCTCGGTCGCGCTGG
CAATCAGCATGCGGCGCATGGCGAAAGCACATGTCATCGTGCGGCGGATGCCGGCCGTGGAAGCGCTTGGCTC
CTGCACCATGATCGCCACCGACAAGACGGGCACCTTGACGCTCAACGAACTGACCGTAACGGATATTCGCTTG
CCGGACGGCAATGAAATCGTCTGCGATACCGGTTCTGATCTGGACTCCTGCACGATCCGTGGCGATGGAACTC
CTCCCGCTGAGGCGCGGGAACGGGCCCTTGCCCTGTTGAAGGCCGCTTCGCTGCCAAATGAAGGATTGCTTAC
CCGAAAAGACAACGGCTGGACCGCCGTAGGGGATACGGTCGACATTGCGCTGCTGGCAGCGGCCTACAAGGGA
GGTCTTCGGCGTGAGGCAATCGAGGATGACTATCCTCTCGTCGCCCGCATTCCTTACGAGCCGGACCTGAAAT
ACGCCGCCTCATTTCACCGCCACGGCGACAGCATCCGCATCTTTGTAAAAGGTGCCGCAGAAACCCTGATCGA
CATGGCCGACAGGATGGACATGGATGGTCGCGCCGAGCCGATCGATCGGGAAGCCCTTCTTCGCCAAAAGGAA
GAGATGGCCGCGCGTGGCTTGCGCGTACTTGCCTTTGCGGAGGGCGAAACGGCGGTCGAGTCGGATGGAGGCT
TCGGTCGGCACCTGCTCGTCGATCTCGTCTTCCTGGGGCTCGCCGGAATGCAGGATCCCGTGCGGCCGGAGGT
CCCGCAGGCAATTCGCGATTGCCACTCAGCAGGGTTGGACGTGGCAATGGTCACTGGCGACGATCCCAAGACA
GCAGCGGCGATCGCCTCCCAGGCCGGGCTAATCTTCACCGAAGATCAGGTAGTCACCGGCGAAGCTGTGCGCA
GAGCAGAGGAGAACGGACAGGAAAGCCTCGACACACTGACCCGCCATGGCCGCATCTATGCACGTGTGGCTCC
TTCGCAAAAACTGGCACTCGTGCTCTCGCTCGCACGCAACGGCCACTTCGTCGCTGTCACGGGCGACGGCGTC
AACGATGCGCCGGCGCTGAAGCACGCGCATATCGGCGTTGCGATGGGACGAAAGGGGACGGAGGTGGCGAAGG
AAAGCGCCGATATCATCATCACCGATGATAACTTCGCTTCGATCGTCAGCGGAATCCGCGAGGGCCGGGTCGC

CTATGCGAATATCCGCAAGGTCATTTTCATGCTGATGTCGACAGGCGCCGCAGAACTGCTGCTCTTTCTGCTG
GCAATTCCACTCGGCTTGCCGATGCCCCTGCTACCCGTGCAATTGCTTTGGCTCAACCTTGTAACCAATGGCA
TTCAGGACATTGCCCTTGCCGGTGAAAGTCCAGAAGGCGACGAACTTAGCCGCGCGCCACGCAGGCCGTCCGA
ACCGATCTTCGATCGGCTGATGATCCGGCGCATATGGCAGTCCACGCTCGTCATGGGAGCCGGCGGTTTCGCG
ATGTTCTATGTTCTGCTCGAGCAGGGTTACGGCGAGAGCGAAGCCCGCAATCTGCTGCTGTTGTTGTTCGTGC
TTTTCGAGAACTTCCAGACGCTCGCCAGCCGCTCCGAGCGCAAGTCCGTCTTTCAGCTTGGCTTTCTTGCCAA
CCCGCTTCTCCTTCTCTCAATCGTAGCGGCCCAGGGCCTTCATATCGCTGCCATGTACACTCCGATCCTCAGT
GAGACGCTGCAAGTTTCACCGATCTCGTTCTCAGAGTGGGCGCTCCTGCTTGTAGCGGCATCGTCTGCCCTCC
TGGTCGTGGAAATTGACAAGTGGCGGGCTCGCCACACGGCGCGCGGACGATCTCGCGGGCAATGAATGAGCAT
TCGCAACCTTCACCATGCGATCGATCCAAGCTCTCTTGCGATAATTGGTGCGTCTGATAGCGACGGATCGCTT
GGCCGGGTGGTTATCGAGAATGTAATCCGGGCTGGATTTGAGGGAGAAATCTGGCCGATCAACCCTAAACATG
ACCAGGTCGCCGGCCATCGCTGCTATCGCCGAGTGGCGGATGTTCCCGGTGTGCCCGACCTTGCCGTCATTGT
CACTCCACCGCAGACCGTGCCGGCTCTGATCCATGATCTGGGTATAAAAGGAACGCGTGCTGCGGTGGTCATC
ACGGCCGGTATCAGCGCCGATCAAGACTTGAGACAAGCGATGCTCGACGCCGCAAAACCATTTCTCCTTCGCA
TAATCGGCCCCAACACAGTGGGCCTCATCGTCCCGTCCGCGAAGCTGAATGCGAGCTTCGCGCATTTGCAGGC
ACAGCCAGGCGGTATCGCGTTGCTTTCGCAATCTGGAGCGATCGCGACTTCGCTGATCGACTGGGCAGCCGAC
AACGATGTCGGGTTCTCCAAGGTCGTTTCCCTCGGCGACATGGCCGATGCAGACGCCGGCGATTTCCTCGATC
TGCTAGCTGGAGATCCCGAGACGCATGCGATCGTCATGTATCTCGAGGCCATCTCCAATCCACGGAAATTCCT
ATCGGCAGCACGGGCCGCGGCTCGGGTCAAACCGATCGTCGCCATCAAGGCGGGCCGCCACGCCGAGGCGGCC
AAAGCGGCCGCCACACATACAGGCGCGCTTTCAGGAGCCGATCGCGTCGTCGACGCGGCTTTGCGTCGTGCCG
GTATCTTGAGAATTGAAGGTCTCGGCGAACTGTTCGATGCGACGGAAACCATCGCGCGCTTTCCCCCTCTTGA
GCACAGCCGTGTCGCAATCGTCACCAATGGCGGCGGTGCCGGCGTTCTCGCCGTGGATCGGCTGATCGACTTC
GGGTGCGCGCTCGCGGATCTTTCTCCAGAGACCGTCGGCACCCTAGATCGAAATCTACCCGCCAATTGGTCGC
GCGCCAACCCCGTCGATATTGTCGGTGATGCACCACCCTACCGCTACAAGACGGCCGTTGAGACGATTGCTCG
GGATGTGGGAGTGGACATCCTGATCGTCATGAATTGCCCGACGGGTCTTGCGTCCCCCGTTGATGCGGCTCAT
GCGATCGCTTCCCTTGCGCAGGCCGGGACAATTTCCGGCAAACCGGTTCTCACATGCTGGCTGGGCGGTCGGA
CCGCTCGTGAGGGTCGCACTGTGCTGCAGCAGGCGGGATTGGCGAGCTACGATACGCCGTCTGACGTTGCCTT
GGCGGCTTCTTACCTCACCAAATGGTCGAAAGCGCAGCGGGTGTTGGTGAGGGTGCCAGAGGGCCGAGACGAC
GAAGTGCATTGCAACCGCGATCTGGCGCGATCCGTTCTCCAGAGGGTGGCGGCCGAAGGTCGCCGAATGCTGA
AGGAACCCGAAGCCAAGGCCGTTCTCGCAGCCTATGGCATCCCCGTCCCTCAAACGATGATCGCAACGTCGCC
GAAGGAGACTGAAGCGATCGCCGGCTTGCTTCTTGCGGGCGCGCCGAAGCTAGTGGTCAAATTGATTTCCAAG
TCGATCACGCACAAATCCGATGTGGGCGGTGTCGTCCTTGACATCCTGTCCCCCGTCGCAGCTCGCGAAGCTG
CCGAGGCAATCGCTGCGCGGCTCAAGGCGCATGACCCGACTGCCGTTGTAGACGGTTTTGCGGTGCAGCCGAT
GATTGAACGCAGACATGCGTGGGAACTGCTCCTCGGCGTCACCCGAGATCCGATCTTCGGTCCGGTCATTCTA
TTCGGATCGGGCGGCGTTTCCGTGGAAGTGGTCGCAGACACCGCGGTGGCGCTTCCCCCACTCGACGCCGTCC
TCGCCGGCGACCTGATCGATGAGACACGAGTTGGCAAGCTCCTCGCCGGTTTCCGCAGCGAGCCGCCGGCAGA
CAGAGCAGCGATCTGCAAAGCGCTCTCGGCTCTATCGCAGCTAATCGTCGATTTCCCATGCGTGCTTTCGATG
GATATCAACCCGCTGGTCGCCAGCGCCGAGGGCGTCATCGCGCTCGACGTGCGTATCGAAGTCGACCCACAGG
CTGTCGCGTGTCCGGGCCCCAATCGCGATCTTGCGATCCGCCCATATCCGGGCGATTGGCAAAAGCAGGTCAC
GCTTGGTGAGCGGATTTATCATCTGCGCCCGATCAGACCCGCAGACGCCGCACTGTATCCGGATTTTATGGCA
GAAACGTCTTCTGGTGACATCCATTTCCGTTTCCTCTCCTCGCGCAAGCACTTTCAGGACCAGATGTTGGTGA
GACTCACGCAGATCGACTACGAGCGCGAGATGGCCTTCGTCGCTCTCGATAATGAAAGTGACGAGCTTGCCGG
AATTGCCAGGCTTTACGCGGATCCCGATCACGAGTCCGCAGAATATGGCTTGCTGGTGAGGACGGATCTGCAG
GGGCGTGGCCTTGGCTGGGCTCTCCTGTCTCATCTGCGGGACTATGCTGCGGCCGATGGTCTGCAACGCATCG
AAGGCCTCGTTCTGAGTGAAAATTCGAAAATGCTGAAAATGTGCAGGGAATTCGGATTCTCAATCTCGACGCA
CCCGGCCGATGCCACCCTGAGGATAGCAACACTGGGACTGCAATGTCAGGCAGGTGTTTCTTGAATGAGCGTT
CAGGTCCGAAGCATCAAGGAAGGTCAACCGGCAGGCATTAGTTCTTCGAGTTTGCCTTCAGCGGATCTTGCTT
GCCTGTTCAGCCGCCAAGCGCTCGAAAAGTTCGAGCCGGGTGAAGCGATCTTCTGGGAGGGCGACGAGGCGAC
AAATCTTTTCGAAGTAGCAGATGGAATGCTGCGAGCACTGCGTTTGCTCGGCGACGGTCGTCGAATTATCGTG
GGCTTCATGCGCCCCGGAGATCTCCTCGGTGTGTCACTCAAGGAGCGTTATCTTTACACGGTCGAGGCAATCT
CCCCGGCGCAAATTCGCCGCTTTCCTCGTCGCGTCTTTGAAGACGAGGTGGCCCGCCATCCACGCTTACAGGA
GCAGCTATTCTCCAAGCTTCGTGATGAGATGACTGCTGCGCAGGATCAAGCCGTCCTTCTTTCCCGCAGGAGT
GCCGAGGAGAAGTTCGCCAACTTCCTGCTGCTGATGAAGGAGAGAGGGAACTCGGAGCATCGTGCCATTGTTG
ACCTCCCCATGACCCGACTGGATATCGCCAATTATCTTGGCATGACGATCGAGACCGTATCTCGCACTATAAC
AAGGCTAGCAAACGGCGGTATTATCGCGGCAGCAGGACGCCGCTCGATCAAGGTCTTGAAGATGGAAACGCTT
CGCCTGCTGGCCGAGGGTGACGAAGGCGAGCAATGGATGCCCTCTCTCGCTCGTCCTGGTCGGTACGCACCCG
CAAACGCTTGAATGCTGTCGCGGTATTTCGAGGAGGAAGGATACCGCGTTACCCTGGCCGGCGACGGACGGGA
GATGCGCGACTGCCTCGATAAACAATCAATCGACATCATTCTCCTCGATCTGGTACTGCCCGGCGAGGACGGG

CTGACGCTTGCGCGCGACCTCCGCGCGCGCTCGGATGTGCCTCTCATCATGCTGACGGGCCGCGACGACGTCG
TCGATCGGGTCGTTGGACTGGAGGTGGGTGCCGACGACTACATTGCCAAACCCTTCCATCTGCGCGAGGTGTT
GGCGCGCGTCAGGGGTGTTCTTCGTGGCCGTCAACCACAAGCGTCGGCGACTGTCGATCTGGCGTCAGAAGTC
TACTCATTTGAGGGGTTGCGCCTCGACATAGCGCGCCGCCAACTCACGTCGGATGATGGCAACGAGATCCTGC
TGACGACCGGCGAATTCGATATGCTCTGTGTCCTAGTGAAGCACGCCGGCCGTGTCTTGCAGCGCGAATTGCT
TATGGATCTGACACGAGGTCGTAATCTCGAAGCTTTCGACCGCACGATCGACGCGCAAATTTCCCGTTTGCGG
CGGAAAATCGAGCGTGACGCCAGCCAGCCAGCTCTGATCAAGTCGGTCCGAGGCGTCGGATATGTCTTCAGTG
CAAGGGCGACACGGCAGGAAGCGTAGATGAAGCGTCGCATTCTTTTGCAAGGCACCACTGCATTGATGGTGCT
GTCGGCACTTCCGGCTTTCGCGCAGGATGACATCAGGGCGGTCGCCCGTGAGGCGTATATCTACACCTATCCA
ATGGTGAAGAACTACCTCACGATGTACCAATACGCGCTGGACCCGGGCGGCAGCCAATACAAGGGGCCACTCA
ACACTCTGGTCAGCATCGCCCGGGTCTACACGCCTGAAGATACGGCAATCATCACGCCGAATTCCGATACGCC
CTATTCCTTCATCGTCTTCGATCTGAGGGCGGAGCCTGTCGTAGTGACGATGCCGCCAATCGAGAAGGATCGC
TACTATTCGCTGCAGCTCATCGATCTATACACCAATAATGTTGACTATCCGGGAACGCGGGTCGACGGCAATG
GCGGCGGCGATTTTCTAATCACCGGCCCCGGCTGGAAGGGCGATGTGCCGAAGGGCATTAAGCGCGTGATCGA
AATGCCGACCACGCTCGCGTTGGGCATCATCAGGACGCAGCTCTTCTCGCCCGACGATCTCGAGAAGGTGAAG
CAGATCCAGGCGCGCTACAAGGCCGCGGTCCTGTCGGCCTATGCGGGGGCTCCGGCTCCGGCTGCTCCTCCGT
CGATCGACTGGTTGCTTATCTCGGACGAGCTGATGGTCACGGACTACTGGTCGATTGCCGCCTTCCTGCTGCA
GTTCGCTCCACCATATTCGGGTGATGAGGCGCAGCGGGAAAACCTCGCCAAGCTTGGTATCCGGGATAGAGGA
GTGTGGCCCGGGACGGATCTTTCACCCGAGACTGTGGCACTGATGAAAGAAGTGGCGGTCGCGACGGAAAAGG
AGATTCGTGACGAGGCTGCCCGGTTGACGGATTCGTCCAAGATATTCGGTACGCCCGAATTCATGAAGGGCCG
GTTCATGGTCCGGGCCGCGGCGGCGCAGGGCGGGATCTATGGTAACTCCGTGCAGGAGGCACTTTATGTCATT
TATGCCTTCGACGCACAGAAGGCGCCACTTGACGGTAAGACAGGAAGATACAAGCTGACTTTCACCCCCAGGA
CGCTTCCACCGGTTGATGCTTTTTGGTCGCTGACCATGTATGATAGGCAAAATCAATTCCTTGTCGACAACCC
CATCGACCGTTATCTGATCAATTCGCCGATGCTTGCCGGACTCAAGAAGAGCAACAAGGGCGAGATCGTCCTG
TACCTGCAGCGCGAAAGCCCAGGAGCGGAGCTGGAATCAAACTGGCTCCCCGCCCCGAGCGAAATCTTCTATG
TGGTCATGAGGCTCTATCTGCCCAGGGCGGAGGCGTTGGACGGGCGCTGGGCGCCGCCGCCGATCGAAGCTCT
TTCGTGAATGCCGGCCGCCAAGGCGCAGGCACTGGTCCAGGGTCTGGTGATGATCGATGCCGATACGGCAGCC
GATCTCGCTGCCCTCGAACGGCTGGCGCTCTGGGCCTTGTCACAATATTCTCCGGTTGTCGCGATGGACCCTC
CCGACGGTATCGTCATGGATACCGAAGGCGCCGATCATCTTCAGGGCGGCGAGGACTTGATGCTGTCCGGCCT
CGTCAATCGCTTCCGCGGCCGGGGTCTCGCTGCCCGCGCGGCAATCGCCGATACCTGGGGTGCCGCGCATGCG
CTTGCCCGGACCACAGATCGCGAGACGGTCATCGTCGGGCGCGGCGATGCCGCCAGGGCCGCGAACCGACTGC
CACTGTCGTCGCTGCGGCTGCCGGCCGAAACTGTTCAAAGCCTTCGAACCCTCGGCTTTACCACCGTCGGCGA
CTTGGCTGCCACACCGCGGGCACCGCTGACGCTGCGCTTCGGCCTGGAGGTCGGCCGCCGTCTCGATCAGATG
TTCGGCCATATCGCTGAGCCGATAGATCCGATCCGGCCGGCGGAACTTGTCGAGGTCCAAAAATCTTTCGCTG
AGCCCATCGGCGCGCCGGAAACGATCGAGAAATATGTGAGCCGCCTGGTGCGGCAGCTCTGTCTTGAGCTCGA
GAAGCGCGGGCTTGGGGTCAGACGCGCCGACCTCATCGTCCATCGCGTCGATAACACCATTCAGGCACTGAGG
GCGGGGACGGCGAAGCCGGTCCGTGACATCGCCTGGCTGACGAAACTCTTCCGCGACCGCATCGAGAGGATCG
AACCAGGCTTCGGCATCGAGAAGCTCGGTCTTGCGGCCATCCTCGCCGAGCCGCTGATTGAGGCACAGTCAGC
CTCGTCACTCATTGAAGAACAGGTAACCGATGTGACGCCGCTCATCGACGTGCTTGGCAATCGCGGTGGACAG
CGGATATTCCGTGTGGCCCCGGTCGCAAGCGACGTCCCGGAACGTAGCGTCCGGCGGATCGCTCCGATCGCCG
ACGAGGACGGCGCCACCTGGCCGCTCAACTGGCCAAGACCGCCGCGGCTTCTGGCGCGTCCCGAACTGATCGA
AGTGATCGCGCTTTTGCCCGATCATCCGCCCGTCTCGTTCACGTGGCGCAGCAAGCGGCGCAGAGTGAAGCGC
GCCGACGGACCGGAGCGGATATTTGGGGAGTGGTGGAAACGCAGCTCAGAATGGGTTGCGGTCCGCGACTATT
TCGTCGTCGAGGATGATAGGGGCGAGCGCTTCTGGATTTTCCGATCCGGCGACGGCGTCGATGCGGAAACAGG
ATCGCATAAGTGGTTCATGCACGGGATGTTTGCGTGAATGAAGCGAATCGTCTTGCTGCTATCACCTCTGGTA
ATCTTGCTTAGCCCGATCATTGACGCGTTTCAGGGAGTGTATATTGACCCCAGAAGCGATGCAGGGTACGCCG
TCATCGCTTGTGTTGCCATAATTGGGCTCGCCCTGGGCATGATTGCAACCGTTTGCTATAAAAGGGGAGCTGT
TGGCAACGTCGTAACAGCAGGCACACTCGCCGTTACCCTTTTCCTTTTTGGCGATCTTTCATACGGTGTTTTC
TGGAGACTAGCAGACCACATCGGAATGGGGGGCGCCGCCGCATTGGCGTTTGCTGGACTGGTTGTACTTATTC
TTATCCTATTCAAGCTCATGGCCGCTGTGCCCCGTATGATGGCCGCATTTGCCGTAGCCCTACTGGGCTCTAC
AGTTCTTGATCGCGGCTTGATTACTGAGGCGAGCGCCGAAGAACCCGCGCCGGCCGTGATTTATATCGTGACT
GATGAAATGATCGGCATTTCCGGGATCGACACAAGATTACCCCATGGAGCAGAAGCCAAAGCCGCATTGTCGC
GGGTTTTCCAGAAACATGGGTTTCGGCTCCATTCCAAGGCTTTCAGCCGCCACATTCTGACTCAGGTTTCAGT
ACCAGCCGCCTTGAACATGGACTATTCTTATAATTTTCCCGGCGATAGATCGCATTATGCCTACCCGGGGGAG
GTAACAAAGTTCAAGGAACTGTCGTTATTCAATCTGTGGCATCGACAAGGATTATCCGTCAATGTCTTCCAGT
CTGCGCATCTTGATTACTGCAAAACTGAAGCGCTAGTCGACTGTCACACCTTCGCATCATTTGACGGAAGTAG
ATTTATTCAACGGCAGCGGATCGAGGGCGGCCCATACCGGGACGTTCCACCCGCTTCCGCTTCGCTGGCGGAT
GTTAAGGCGCTGGTCGAGGGAAACAGGCAAAGCCTTGCAATGGCAGCCGTTGGCTTCGTGGCCTCCAACTTGC

TGGAGACGAAGGAGACGGCACTTCAAGAATGGCACCCGCGATCTTACGACCAACTAGCCTTCCCTTACTGGCT
CGGACAGTTTCAGAACATGATTCTAGATAAAGGTCGTGGAAACGCCTTTTTTGCACATTTTCTATTTCCGCAT
AGTCCCTCGGTCTACAACAAAGATTGCAAACCAACGAACCGTTGGGTTGAGAGGTCCTATCTTACGGAAGTCC
GCGGGTTGGCAGGTCAAGAGCTAGATGAAGCGCGGGTGAAAGAATATGGATTCTATTTCGCTCAAACGCTGTG
TCTCGCCAAGCAACTTGATAAGTTCTTTAATGCGGTTCTCAGCGATAAGAGATTTCAGGACGCAACGATAGTC
GTTCATGGCGACCACGGCTCACGTATTTCTGCGGGTAGAAATGTCGAGACTATGTCTCCGAGGGATTACGTCG
ACAATTATTCCGCTCTTTACGCGGTCAGAAAGCCGGGCGTGGCGACGGGTACGGACTACAAGCTACGCTCGGT
CCAGTGGTTAAACGCTTCTCTGTTCCGTGGCAATGTATCAGAGGTTGCCCCGACCGTAGTAGGATCTATGCAG
GGTAAATCTGACGATCCAGCAGTGTACGCACCCATTACCGACCCAGACCACGTTGCTATAATGCCGATGCATG
ACTTTGACGCATCCCATTGAATGGCAAGCACCAATTTCCGTCCGCTGCACGACCGCGTTGTCGTCCGCCGCGT
CGAGTCTGAAGAAAAGACCAAGGGCGGCGTCATCATTCCGGACACCGCTAAGGAAAAGCCGCAGGAAGGCGAA
ATCGTGGCTGTCGGTTCGGGTGCCCGTGACGAAAGCGGCAAGGTTGTTCCGCTCGATGTGAAGGCTGGCGACC
GCATCCTGTTCGGCAAGTGGTCCGGCACCGAAGTCAAGATCAACGGCGAAGACCTTCTGATCATGAAGGAAGC
CGACATCATGGGTGTCATCGGCTGAATGCTTGTCAAGGATGTGATGACGACAAAGGTTGTCAAACTGTCGCCT
GACAACAGCGTTAGACAGGCAGCCAAGCTGATGTTCGACCACCACGTAAGCGGCGTCCCGGTCGTTGACGACG
ATGGGCGCTTGCTCGGCGTCATCAGTGAGGGCGACCTGATCCGGCGGACGGAACTCTGCAGTGGAGCATCCGT
GTTGATGGCGGAGATGGCGATCGATCCCGATGACAGGGCGAACGCCTTCATCAGGCGATGCTCGTGGCGCGTC
GGCGACGTCATGACGGCCAACCCCGTAACCATTGAGGAGGAGGCGCCCCTTGCGCGCGTTGCAGGGCTCATGC
AGGAACGCGGTATCAAGCGCATCCCCGTAATGCGCGATGGGGAACTGGTCGGCATCGTCAGCCGCGCCGACCT
GCTGCAGGCAATTTTTTCAACGAAGCCAGACGAAACAGCAGCGGGCGACGAAGCCATTCGACGCAGCATCCTT
GTTCGCCTCGGCGAAAACACGGGTCTGGAAGAACTGGACGTGACTGTCACGGTCACCGAGGGGATCGTGCACT
TTTGGGGCCAAGTCGAGACGGCCGCTTGTCGCAGGGCCGCGCGGATCGTGGCTGAGAGCGTCCACGGGGTCAG
GGGCATCGTGGAGCATTTTCCAGATCCCTACACTCAATAGGTGACGCAGTCTGCAGATAATAAAAGACCTGAC
TCCGGCGTGTCCGACGACTCGATCAACATGCTGCTGCGGTTTGCCGTTATGATGCTTCGTTCCGGGGGCACCG
CGATCCGTACCCGCGAACTGGTGGGGGCGATGGCGCACCGCCTCAACCTGGAAACGCCATCGGTGAGTCTCAC
GCTCGAGTCCGTCACCCTCAGTGTTTACCACGGCAGCGAACAGTTCGTAGCGATGCGCGAGGTCGGTCCGCCC
GGAATCGACTTCAGGCGCATCGGTGAGTTGGAGCGGCTTGCGAACGCGTTAGAGCCAACGGCACCGCCACACC
GGATAGCGAAAGAACTTGCTGAAATCGAAGCGCGGGCGCCTCAATATACCGGCTGGCAGATGGCGATCGCGAT
CGGCCTGGCCAGCGGAGGCTTTGCGTTCCTCAACGGTGCGGCCCTGCCGGAAATGGCCAGCGCCGCGATCGGT
GGCGGCATAGGTCAAGGATTGCGCTGGTGGTTGACCCGCCGCCAATTGACACACTTCGGCACGGCGGCACTGG
CTGCCATAACCGCTGCAGGAACTTACGTTCTGGTGGCAGCGCTGGCGCGCCGTGCAGGGTTTGCGTTCTCGCA
CTATGCTGCGGGCTTCATCTCCACCATACTATTTCTCATCCCCGGCGTTTCGCTGATTGCAGGACTGTTCGAT
CTGTTGCAGCATCAGACGGTGGCCGCCTTGAGCCGACTGGCATATGGCGCGCTGATCCTGTTCATCGTCGCTT
CGGGGCTGAGCATCGTGGTGACTGTTGCGGCCATCGAGCTGTTGCCACGCTCGGCGCCGGCCGAGCTTGCCTA
TCCGTTGCGCCTTTCGTTGCGCGCCGTCGCGAGCTTCGTTGCCGGCTGCGGCTTCGCCATGCTGTTCAACAGC
ACGCCACTTTCGTTGGTTGTCGCGGGCATCGTGGCGCTGGCGGCGAATAGCGTGCGCCTCGTCCTGATCGACA
TGGGAATGCTGTTGGCGCCGGCGGCGTTCATCGCCGCGTTGTTGATAGGAATCATCGCCATTCTTGTGAGCCG
GCGGTTGGACGCAGAGCTCATGGCGATTGTTACCGCGCCGGTCGTCATCATGATTCCGGGTCTCTACGTATTC
GAGATGCTTGTTCTGCTCAACCGGGGGCAGACGCTCGAGGCCATGCAGGCCTCGGGGGCAGGGATCTTCGTGA
TCAGCGCGCTCGCGATGGGGTTGAGCGTGGCGCGCCTCGCAGTCCCGTGGGAAAGTTAGATGATGCCGTTCAA
CCGGGCGAACAACGGCGTCGGTGCGCGCTCGTTTCGCGCTAAGTTCATCCTTGTCGTCGGCGGCGCCGTGCTT
TTCGACCTGCTCGTCACCGGCGGCCTTGCTCTCTGGAACGTGCAGCGACTCTCGCGAGACGCGACGGCACAAG
TCGGTGAGGGCCTTGAGGCGGCAAACGAGGACTATATCCGCTCCTACGCGGAAACGACGGCCGAGCGCGTCAA
TCTCCTTTTAGACCAAGTTCACTCTGACGTCGGCGCTCTGGCGGGTGTGCTGCAGGCGCAGATCGACCATCCC
GAAAGGGAAACTCAGATCGGCGAAGCCTTGGCCCGCGGTGCGCCCGAAGCCGTCACGGTCAGCTACAACGAGA
AGGGCGGGTGGGCACAGAACTTGCCTGGATCTTCGTCAGTCGTCAGCGTTTGGGGCTACCTGCTCGGCTCCAA
CCGCGTCCCGTTGCCGGAGGTGCAGCGAGAGATCGAGAACAGCGCGGTAGTTGACCTCGTTGCTCCTAGCCTT
CTGGCAAACGGCCTATCGAAACTGCAAATGTACTATATCGGCCCGAAGGAGCGGCCGATTTTTCGCACATCTC
CGTACACCGACCAGGCCCAGACCTTCGACAGGCTGTACCCGGGCCACAACCAGGCGGAATTCTGGCCGTTCTT
CTTCCCGGGACTGTACGAATCATGGGAGCAATGGGCGGCGGATCCAGCGTCCCGCCCGGTAGAAAACGACATC
ACACAGACTGCACCCTACACGGACGCGATCACCGGCAAGCTGATCGTCAGCTTCTTCCAGCCGCTCTGGTCGC
GCGGCCGTGACCGCGTCGCGGGTGCCGCCGGCGCCGATATCACCCTCGACCAACTGGCAGAGATTGTTGAGAA
TGTCAGGGTCGCTGACAAAGGCTTCGGCTTCCTCACCATGTCCGACGGCAATGTCGTCGCCATTAATCCGGTC
GGCGAAAAGGTGATCGGATTGCAGACCTCGAGCGACGTCAACGGCCAAGGTGTGACGGGTCTAAATCGCTCAT
TGCGCCACAGCGCACAATCGGCGATCGCCAAATTACCCTTGGAGCAGGACGGGCTGCTGCAGCATGTCCTGCT
TGAGGAAAACGGCGAGAAGGTGCCCTATCTCGTCATGCTGAAACAGTTGCAGCCGAGCAACCTTTGGGCCTCC
GGTCCGGTTCGTCGTGAATCCATGCTGCTTGGCATCGTGGTGCCGGAGCGTGAAATCTATGCCTCCCTCTTCG
CCGCACAGGCCGGCATCAACAAGGCGGCGAACAGAATCCTAGTCTATCAAGTGCTAGCTCTGCTCATCTCGCT

CCTTTTTGTCACAGCGGCCGTGTTCGCCATCTCCGGGCGGATCACGAGCGGCCTCAGCGCGCTTGCCGATGCG
GCAAAACGAATCCAGCTCAAGGACTATTCCGTCCGCGTCAACATTCCGACACGCGACGAGGTCGGCGAGGCCG
GAAACGCCTTTAACCGCATGGCCGAACAGATCAGCTATCACACCGAAAACCTGGAGCAACTCGTCCAGGATCG
AACGAAGGAGATTGAAGAGGCAAACCTGCAAATATCCGGCCTGAATGAGCAGCTGCGCAGCGAGAACGTGCGC
CTCGGGGCCGAACTGGACATCGCGCGCCAGATCCAGATGATGGTCCTGCCAAGGGCCAGCGAGCTTGAGGAGA
TCGCGGACCTGGAGATCGCTGCCTACATGCGACCCGCAGACGAGGTAGGCGGTGACTATTATGATGTGCTGAG
GGACGGAGAGCGGCTGAAGATCGGTATTGGCGATGTCACCGGACACGGCCTCGAGAGCGGCGTGCTCATGCTG
ATGGTGCAGTCGGTCGCACGGGCACTGCAGGAATCCGGTGAAATGGACCCCTCGCAGTTCCTCAACAGGCTCA
ATCGAGCGATTTACAAGAACATCGTCAGAACCAGCACGGACAAGCATCTGTCGCTTGCATTTTTGGACTACCA
GGCTGGACAGCTGACATTGTCGGGTCAGCATGAGGAAGTGATCATGGTTCGGGCTGCCGGAGCCGTCGAGCGA
ATTGATACGATCGATCTCGGTTTGCCGATTGGCTTGGAGTCCGACATCTCGGCCTTTGTGGCGACGCACGAGA
TCCCCTTTGCACGCGGCGACATGGCTATTCTTTACACAGACGGCGTGACCGAGGCTGAGAGTTGCAGCAGAGA
ACTGTTTGGAATCGAGCGGCTTTGCGCAAGCGCCCGTCGCCTTTCCGGTCGGAGCGCCGAGGAAGTCAAGACC
GGCATTCTCGACGACCTGGTGGCGCATATCGGCACGCAGAAGATCCATGACGACATCACGTTAGTAGTGATGA
GGCACAGGTAGATGAACCTATTCGGCACACTGAGGGCGCCGCGCGAACTGCTTTTCGGCGCCGGGCAGCGCCA
TGCCCTTGGGGGCATCGCCGCCAAGCTCGGCCATCGCGCGCTGATCGTCACGGATACGCGCCTTGCCGTGGAT
GCCGACTTGCTTGCACTGGTGCGGCGACTCGAAGAGGCAGGGCTTGAGGTGATGGTGGACAGCTCCACCCTGC
CGGACGTCCCGGTGGAATCGGCTATCGTGAGCGCTGCAGCGGCCAGAGGCTTTGCGCCGGACCTCGTCATCGG
CATCGGTGGCGGGTCCTGTCTCGACATGGCGAAATGCGTCACCCTCCTCCTTACCCACGGCGGGCGGCCACAG
GACTATTACGGCGAATATGCCGTGCCGGGACCCGTGATGCCATTGATCGCCATTCCCACCACCGCCGGCACCG
GCTCGGAGGTGACGCCCGTCGCCGTGCTTTCGGATGCCGAGCGTAGCCTGAAAGTTGGCATTTCAAGTCCGCA
CCTGATTCCGGCGGTATCGATCTGCGACCCCGAACTCACGCTCTCCTGCCCATCCGGCCTGACTGCGATCGCC
GGCGCGGACGCCCTGACGCATGCGATCGAAGCCTTCACGGCGATCCGGCGCGAGCCCGTCCCCGGTATCGCGC
AGCAGCGCGTCTTCGTCGGTAAGAACGAGCTTTCCGATCACTTTGCCCTTAGCGCCATTACCCTCCTGTGGCA
GGGGCTGGAGAGGGCCTGCAAGGACGGCGCCGACGCCGGCGCGCGCGAAAAGGTGATGCTCGGCGCAACGCTC
GCCGGTCTTGCTTTCGGGGTGGCGGGTACTGCCGCGGCCCACGCCATCCAGTACCCTGTCGGCGCGCTCACCC
ATACGGCGCATGGCCTCGGGGTCGCCTGTCTAATGCCCTATGTCATGACTTGGAATGCGCCGCTCATCCGCGA
CGAACTCGCGCAGATCGCGCATGCGGCCGGGCTCGGCGGACCGGATGAAGTGATCCCTGCCCTCGTTTTGCTC
TTCGAACGCATCGGCATTCCCGCCACCCTGCGGGACCTCGGCCTCGAGGAAGACCGGATCGATTGGGTGGCGG
AACAAAGCTCGGGTATCGCACGCCTAATCCAGAACAATCCCCGCCCCTTGAACCCGCACGAGATGCGCAACCT
CGTCGCCGCCGCCCATTGCGGCGATCGCTCCCGCCTGAACTGAATGACCATCCGTAAGATGCTTCTGGCATCG
GCCGCGATCACTTGCGCCGCGATGCCCGCCTCCGCATTTGCCGACACCTCGGCCAAGAAGATCGCCCTTTCGA
ACAACTATGCCGGCAATTCGTGGCGCCAGGCCATGCTGACGAGTTGGGAAAAGGTGACGGGCGAAGCCGTGAA
GGCCGGCATCGTCGCCTCGGCCGACGCCTTCACCACCGCCGAGAACCAGGCAACGGAACAGGCCGCGCAGATC
CAGAATATGATCCTGCAGGGCTATGACGCGATCGTGCTGAACGCCGCCTCCCCGACAGCGCTGAACGGCGCGG
TCAAGGAGGCCTGCGATGCCGGCATTACCGTCGTCTCCTTCGACGGCATCGTGACCGAACCCTGCGCCTGGCG
CATTGCCGTCGATTTCAAGGAAATGGGCCGCAGTCAGGTCGAGTATCTTTCGAACAAGCTTCCGGACGGCGGC
AATCTGCTCGAGATCCGCGGCCTTGCCGGCGTATTCGTCGACGACGAGATCTCGGCCGGCATCCATGAGGGCG
TCAAGCAGTTCCCCCAGTTCAAGATCGCCGGCTCCGTGCACGGAGACTGGGCGCAAGACGTGGCGCAGAAGGC
CGTTGCCGGCATCCTGCCGAGCTTGCCTGACATTGTCGGCGTGGTGACGCAGGGCGGAGACGGTTATGGTGCC
GCGCAGGCCATCGCCGCGACCGACCGGAAGATGCCGATCATCGTGATGGGCAATCGCGAGGATGAACTGAAAT
GGTGGAAGGAGCAGAAGGACGCGAACGGCTATGAAACCATGTCGGTCTCGATAGCGCCTGGTGTCTCCACTCT
CGCCTTCTGGGTCGCCCAGCAGATCCTCGACGGCAAGGAGGTAAAGAAGGACCTCGTCGTTCCCTTTCTGCGC
ATAGATCAGGACAATCTCGAAGCGAACCTCGCAAATACGCAGGCCGGTGGCGTCGCCAATGTGGAGTACACGC
AGGAAGACGCGATCAAGGTCATCGAGTCGGCAAAGTGAATGAGCAGTTTTCGACGGAAGTTGACGACTTCCGC
TGTCGCGGCCATCTGCAGCTTGGTTGCTTCCACAGCGGGCGCCCAGACGGTGTTGAAGGCGTCGCACCAATTC
CCGGGGGGCAAGGGCGACATTCGCGACGAGATGGTGCAACTGATCGCCCGCGAGGTCGCCGCCGCCAATGTCG
GCCTCGAAATCCAGGTGTTCCCGGGGTCGTCTCTTTACAAGCCGAACGATCAGTGGAATGCGGTTACACGTGG
CCTGCTCGATATGACTTCGTTCCCCTTGGACTATGCTTCCGGCCGCCACCCGGAATTCTCCGCGACGCTGATG
CCCGGCCTCGTGGGAAACTTCGACCGCGCCATGCGGCTTAACGACTCCGAGTTCATGGGGGACATAAAGAAGG
TCATCGAGAATGCCGGCGCCTTGGTGATCGCCGATGCGTGGCTCTCCGGTGCCTTCGCCTCCAAGAAGAATTG
CATCACCTCCCCCGACACGATCAAGGGACAGGTGATCCGCGCCGCCGGGCCGGCCTTCGAGGAGATGCTCGTG
GAAGCCGGCGCTTCGATCTCATCCATGCCTTCGTCGGAAATCTACACCGGCATGCAGACGGGCGTCCTGGATG
CGGCGAACACCTCGTCGGCGAGTTTCGTCTCCTATCGCCTGTTCGAACAGGCCAAGTGCCTCACGGCGCCGGG
AGAAAATGCCCTCTGGTTCATGTACGAACCGGTGCTGGTATCGAAGCGCGTCTTCGATGGCCTGACGGAAGAG
CAGCAAAAGGCAATCCTCGCTGCCGGCGAGAAAGCGGAAGTCTATTTCAACGAAGAGGTCCGCAAGGGCGATC
AGGTCATGATCGACACCTACAAGAAGGCAGGCGTCGAAGTCGTCGAGATGTCGAAGGAGGATTACGATGCCTG
GCTCGAGCTCGCCAAGGCGTCGTCCTACAAGAACTTCGCCGCCAATGTTCCCGGCGGCGACAAGCTGATCGAA

AAGGCGCTCGCCGTGAAGTAAATGCTCAAGGCCTATTCACAGGCTGTCGGCAATGCTTCGCGTGGCCTCGCGG
CCATTGCGACGGCCCTGCTCATTGCAGCGATGCTCGTCGTCTGCCAGATGATCATGCAGCGCTATGTATTTCG
TCAGGCGACGATCTGGCAGACGGACTTCGTCGTGTTTTCCGCAACCGCAGCCATGTTTCTCGGTGCGCCATAC
GTCTTGCTCAAGGGCGGCCATGTGGGCATCGACGTCGTCGAAATGGTGGTGGGCGAACGTACGCGATACGTTC
TGCGCATCATTGCGGGCCTGCTCGGGCTCCTGTTCTGCGCCGTCATGCTGATTGCCACATGGATTCAGTTCCA
TGACGCGTGGGCGGGAAACTGGAAACACTCCAGCGTCTGGGCGCCGCCGCTTTGGGTGCCGCTCGCTGCCCTG
CCCGTCAGCTTTGCCATGCTGTGCCTGCAATATGTCGCGCAGATTCTGACGCTCCTCACCGCCCCGGCAGTCC
CGGCAGCCATGGGCCACGGCGCGGCCGAACCGGGTTCGGCCCCTGGCGCCGACCGTCATCCACAGGAGATCAT
TCAGTGAATGATGCCGTTCGTCATGGACGAACTGCAGCGGAATTATACGGTTCACAGGCTTTACCAGGCCGCC
GATCGGCCGGCGCTCGAAGCGGCACTCCCATCGATCCGCGCGGTTGCTACGGGCGGCGGCGCGGGCCTTTCCA
ATGAATGGATGGAGAAGCTTCCATCACTGGGGATCATCGCAATCAACGGGGTCGGGACGGACAAGGTGGATCT
CGCCCGGGCGCGCCGTCGCAACATCGATGTGACGACGACGCCGGGTGTCCTCGCGGACGACGTCGCTGACCTC
GGTATGGCGCTCATGCTCGCCGTCCTGCGGAGGGTCGGCGATGGCGACAGGCTGGTGCGCGAAGGCCGCTGGG
CCGCTGGCGAACAACTACCGCTCGGCCATAGCCCGAAGGGAAAGCGGATCGGCGTACTCGGCCTCGGTCAGAT
CGGGCGGGCACTGGCGTCGCGCGCGGAGGCCTTCGGCATGTCGGTGCGATACTGGAACCGGTCCACCCTTTCC
GGCGTCGACTGGATCGCGCATCAGAGCCCGGTCGACCTCGCGCGCGACAGCGACGTTCTGGCCGTCTGCGTGG
CGGCGAGTGCGGCGACGCAGAACATCGTCGACGCGTCCCTTCTTCAGGCGCTCGGTCCCGAGGGTATCGTGGT
AAACGTCGCGCGTGGCAACGTCGTCGACGAGGACGCCCTGATCGAAGCGTTGAGGTCCGGAACGATCGCGGGT
GCCGGGCTCGATGTCTTCGTCAACGAACCCGCAATCCGCAGCGAATTCCATACCACGCCGAATACGGTGCTCA
TGCCGCATCAGGGCAGCGCGACGGTTGAGACGCGCATGGCCATGGGCAAGCTCGTGCTTGCAAATCTCGCCGC
CCACTTTGCCGGCGAAAAAGCGCCGAACACCGTCAATTGA
>6E40 Concatenation of 3 sequences
ATGGACATCAGCATTGCGCGTACGTTCCTCGAGGTGGTCAAGACGGGAAGCTTCGTGAATGCCGCTGCCAATC
TGAACATCTCCCAGACGGCAGTGAGTGCGCGAATTCGCGTACTCGAGGAACAACTCGATCGGCCGATGTTCGT
TCGGAACAAGGCCGGTGCCAGGCTGACCCCCGCCGGAGAGCAGTTTTTCCGTTTTGCAACGAGCCTAGTGCAA
GTATGGGAGGCGGCGCGAACCACGGTCGCTTTGCCCCCGGGACGCGAGGCCATGGTGACGATCGGTGCTGAGC
TCAGCCTGTGGAATCCGCTGTTGAAGCATTGGCTTTTGTGGATGCGCCGCGAATGCGCCAGCGTCGCCATTCG
AACGACGATAGACAGATCGGAGCGGCTGATGGAACTCGTGCAGGACGGTTCACTTGACGCGGCGGTCCTTTAT
GGAGCGCCAAATCGTCCAGGCACGCTTACGGAGCTGCTTTTCGAAGAAAAGCTCGTTCTCGTTCGCACTACAG
CTTTGTCGCAGCCGCCCGGACCGGAAGACCTCGTTGGCATCGATTGGGGAGAGGATTTTGCCGGCAGCTATAG
GGCGGCCTTTCCGGGCGGCCGGAATCCGGTGGTAGCGATCAGTTATGGGCCGCTTGCTCTCGATTACATTCTG
GAAGTCGGCGGATCCGGATATTTTCGGCAGGGCTTCGTTCGGCCCTATCTTGTGGACGGCCGCCTCGAGCTCG
TTCCCAACAGTCCGGAATTTGCCTACCCCGCTTATCTCGTTTCTTCCGCAAAGGCGGATCGGTCTATGCTGGA
TCGCATTCGGGCAGGATTGAGGGCCGCCGCATCCAGTGCCGGCGTCTGAATGTTCGGCATAGACATCACCGTC
CTGGGATTGGCCGGCCTCGTCGCCCTGGCGGCGGCAGCACTCGCCTATGGTGTGCTCTACCCCCAGATCGAGA
CCGAGAAGAAGGCGGAGGGCCGCCTGCGCCGGGTCAGCGCCTCCGAGACGGACCGCACCAAGATCAAGGCGGC
GCGCGACCGCGTCAACGAGATGTCGAAGCGACGCAAATCCGTTCAGGATTCGCTGAAGGAACTCGAGAAAAAG
CAGCAGGAAAAATCCGCCAATACGGCGCCTTCGATGAAGAAGCGCCTGCTGCAGGCCGGTCTTTCGATATCGC
TCGCGCAGTTCTACCTGTTCAGCGCGCTTTTCGGACTGTTCGCCTTCCTTGTCGTCCTCCTGGCAGGGGCGGG
GCTGCTCATTGCCGCGGGCGTTGCCCTGATAGGCGCGGCCGGCCTGCCGCGATGGATCGTCGGCTCCATGGTC
AAACGCCGTTGCAGAAAGTTTCTCGACGAGTTTCCGAATTCGCTCGATGTCATGGTCCGTTCGATCAAGTCGG
GGCTGCCGCTGAACGACGCCTTGCGGCTGATCGCCAGCGACGGGCAGGAGCCGGTCAGGACGGAATTCCGCCG
TGTCGTCGAGTCCCAGCAGGTGGGCCTCAACGTTCCCGAAGCCTGCGCCCGCATGATTCACAGCATCCCGCTG
CCGGAAGTGAACTTCTTCGCGATCGTCATCGCCATTCAGGCGCAGGCGGGCGGCAATCTGTCCGAAGCGCTCG
GCAATCTCTCCAAGGTGCTGCGCGAACGCAGGAAGATGAAGGCGAAGGTCAGCGCGCTGTCGATGGAGGCCAA
GGCGTCCGCCTGTATCATCGGCGCGCTGCCGTTCATCGTCGCAACCCTCGTCTATCTGACTTCGCCCGATTAC
ATGATGGTTCTCTTCACCGACCCGCGCGGCCACATCATCATGGGCGCTTCCGCCGTCTGGATGAGCATCGGCA
TCTGGGTGATGCGCAACATGATCAACTTCGACATCTGAGTGAATTCATCCGCACCCCCGATCCGCGTGCTCAG
CGCCGGGCTCGGCAATATGGGCCGCAGCCACGCGCTCGCCTATCATCAGAACCCCGGTTTCGAAATCGCCGGG
CTGGTCAACCGCTCGAAGGTGGCGCTGCCCGAGGCTCTTGCAGGATACGAGATTCTGCCGTCCTTTCCGGAGG
CGCTCGCCGAACTGGAGCCGGAGCTCTGCTCGATCAACACCTATTCGGACACGCATGCGGACTACGCCGTGAT
GGCGATGGAGGCGGGAGCCCATGTCTTCGTGGAGAAGCCGCTGGCAACGACGGTCGCCGATGCCGAGCGCGTC
GTCGCCTGTGCGCGGGCCAATGGCCGCAAGCTGGTCGTCGGCTATATCCTGCGCCACCACCCGTCCTGGATGC
GGCTGATCGCGGAAGCCCGCAAGCTCGGAGGGCCTTACGTCTTCCGCATGAACCTGAACCAGCAATCGAGCGG
CCCGACCTGGGCGACGCACAAGTCGCTGATGCAGACGACACCGCCGATCGTCGATTGCGGCGTCCACTATGTC
GACGTCATGTGCCAGATCACCGATGCCAACCCCGTCGAGGTGCGCGGCATGGGCTTGAGGCTCTCGAACGAGA
TAGCCGCGGATATGTACAATTACGGGCATCTGCAGGTGATCTTCGAGGACGGCTCGGTGGGCTGGTACGAGGC
GGGCTGGGGGCCGATGATCTCCGAGACGGCCTTCTTCGTGAAGGACGTGATGTCTCCGAACGGCTCCGTGTCG

ATCGTCATGGACCAGAACGCCAAGTCCGACGACATCGACGTGCACACGAAAACCTCCGTCATCCGCGTGCACA
GCGCCGCTACGGGACCGGACGGCCGGTTCCTGAAATCCGACGAGGACCTGGTGATGGAGGGCGAGCCCGGCCA
CCAGGAGCTTTGCGACCGCGAGCAGGCCTTCGTGCTCAAGGCCATCCGAGAAGACATCGATCTTGGCCGGCAC
ATGGACGATGCGGTGCAGTCGCTGAAGATCTGTCTCGCCGCCGACGACAGTGTGCGCACCGGCAAGCCGGTCA
AGCTTTAAATGACGAAATTTCGGAATTTCGTTGTCAGCTGCGCACTCGCCGCCGCCTCCGCGGTTCCCCCTGT
AACGTCCGTTCAGGCCATGCCGCGCCCGGCGCTCGACATTGCGATCAAAGCGCCGGCCGGCGTCGAGAATGCC
ACGCATCGCGGCCGTTACGGGTGGCGCGGCGGCTATTCTCGCCGCGGCTATTATGGAGGATCCGGCTTCTATC
CGGGCTGGAGCCCCTATTACAGGCGGTCCGGGATCAGCTTCTATTTCGGACCCAGCTACCGTTACCGCCCGTA
TTACGGGAACCGCTATTATTACGGACCGCGCTTTTATGGCCCCGGCTATTACAGCTATCCCGGGCCCGCATAT
TACGGTTATCGCTCCTATAGGCCACGCTACTATGGCGGCGGCTACGTCCGGAGCGGCGGCAACAGCCATGTCA
GCTGGTGCCTGTCGCGCTATCGGTCCTATAACCCTGCCTCCAACCGCTACCTGACCTATGGCGGGGTCTACCG
GGTCTGCTACTCGCCCTATCGTTAGATGACTTCGTTCTTTGATTCCGAATCTCCCTCCAACGTCGCCGAATAT
TCGGTGTCGGAATTGTCCGGCTCGATCAAGCGCACGGTCGAGCAGGCCTTCGAGCATGTCCGGGTGCGGGGCG
AGATTTCCGGCTACCGCGGCCCGCATTCCTCGGGACACGCTTATTTCGCCCTGAAGGACGATCGCGCCCGCAT
CGATGCGGTCGTCTGGAAGACCACCTTCGCGCGGCTGAAGTTCCGGCCGGAGGAGGGGATGGAGGTCATCGCG
ACCGGCAGGGTCACGACCTTCCCCGGCTCGTCCAAATATCAGATCGTCATCGACTCGCTTGAGCCGGCCGGTG
CCGGGGCGCTGATGGCGCTTATCGAGGAACGCAAGCGCAAGCTTGCGGCGGAGGGCCTTTTCGATGCGGGCCG
CAAGCGGCCGCTGCCTTTCATGCCGAGGGTCGTCGGCGTCGTGACCTCGCCGACCGGAGCTGTGATCCGCGAC
ATCCTGCACCGCATTGCCGACCGTTTCCCGGTCCATGTCATCGTCTGGCCGGTGCGCGTGCAGGGCGACGGCG
CCTGCGAGGAGATCGTGGCGGCGATCGAGGGTTTCAACGCGCTGCAGCCGGGCGGCTCCATTCCCCGGCCCGA
CGTGCTGATCGTCGCGCGCGGCGGCGGCAGCCTCGAGGATCTCTGGTGCTTCAACGACGAGGCGATGGTGCGC
GCCGCGGCGGCTTCCGCCATCCCTCTGATCTCGGCCGTCGGTCACGAGACGGACTGGACGCTGATCGACCATG
CGGCCGACCAGCGGGCGCCGACGCCGACCGGTGCGGCCGAAATGGCGGTGCCGGTGAAGGCCGATCTCGAGGC
CCAGCTCGCCAGTCTCGCAGCCCGGCTCAAGGGCGCCGCGGCGCGGCAGATGGACAATCGCCGCCAGACGCTG
CGCTCGCTTGCGCGGGCGCTACCCTCGCTCGACCAATTGCTCGCCTTGCCTCGGCGTCGCTTCGACGAGGCGG
CCGCCGGACTCGGCCGCGGCCTGCAGATGAACACCGCCAACAAGCGGCGAAGCTTCGAACGCAATGCGGCCCA
TCTCCGGCCGGAGCTCCTGACGGCAAGGATCGTCGATCGCCGTCAGCGCGTCTTGGATGCCGTCAACCGGGCC
GAGCGCATCGTCGAGCGTAAGGTTCAACGTGGCGCTCAGCGCATTTCGTCTGCCGATGCCTCGCTCCGCGTTC
TGCCGTCGCGGCTGATCGGCCAGATTCATCGCGCATCGGATCGCGTTTCCAGCCTGGGCAGGCGTGGGGATGC
GGCGATCGCCGCCGATCTGCGCAGGATGAAAAGCGCGCTTGCAGCACAGGACCGCGTGCTGCAGTCGCTCTCC
TATCACAGCGTCCTGCAGCGCGGCTTCGCGCTGGTGCGCGATGCCGCGGGCGAGCCGGTTAAACAGGCGGCTG
CCGTGCATCCCGGGATGGCGCTTTCGCTGGAATTCGCCGATGGCCGCATCGCGGCGGTCGCCGGCGAGGACGG
CACTGCGCCGCAGTCGCCGAAGAAGCGGCCTGCACGCTCCGGCGAGCCGACGAAGCAGGGCAGCCTCTTCTGA
ATGAATTTCTCACGCTTCTTCGTCGACCGCCCGGTCTTCGCGGGCGTTCTTTCCGTGATCATCTTCGTCGCGG
GCCTGATCGGCATGACCGGCCTGCCGATTTCCGAATATCCGGAAGTCGTGCCGCCGCAGATCGTCGTCAGGGC
GCAATATCCGGGCGCCAACCCGGCCGTCATCGCCGAAACCGTCGCCACACCGCTCGAAGAGCAGATCAACGGC
GTCGAGGGCATGCTCTACATGCAGAGCCAGGCGACCGCCGATGGCCTGATGACGCTGACCGTCACCTTCGAGC
TCGGAACCGATCCGGATCAGGCCCAGCAGCTGGTGCAGAACCGCGTCTCGCAGGGCGAGCCGCGGCTGCCGGA
AGAGGTGCGCCGGCTCGGCGTCACCACCGTCAAGAGCTCGCCCGACCTGACGCTCGTCGTGCACCTGATTTCA
CCCAACGGGCAATACGACATCAACTATCTGCGCAACTACGGCGTCCTGAACGTCAAGGACCGGCTGGCGCGCG
TAGATGGTGTCGGCCAGGTGCAGATCTTCGGCGGCGGCGACTATTCCATGCGCGTCTGGATCGACCCCGAAAA
GGCCGCAGAGCGCGGGCTTGCCGCGAGCGACATCGCCAATGCCGTCCGCGGGCAGAACGTCCAGGCAGCCGCC
GGCGTCATCGGCGCTTCGCCGTCGGTTCCCGGCCTGGACCTGCAACTCTCCGTCAACGCTCAGGGCCGCCTCA
AGACACCCGAGGATTTTGCCGACATCGTCGTCAAATCGGGCGAGAGCGGGGAGATCACGCGCCTGGGCGACGT
CGCCCGCGTCGAAATGGGAGCCGCTGACTACTCGCTGCGCTCGCTTCTCGACAACAAGGCCGCCGTCGGCATG
GGCGTGTTCCAGGCGCCCGGCTCGAACGCCATCCAGATCTCCGAAAACGTCCACAAGGTCATGGCCGAGCTGA
AGCAGACCATGCCTGATGGCGTCGATTACGAGATCGTCTACGACACGACGCAGTTCGTCCGGGCGTCGATCGA
ATCGGTCATCCACACGCTGCTCGAAGCCATCGCGCTCGTGGTCGTCGTCGTCATCGTCTTCCTGCAGACCTGG
CGCGCCTCGATCATCCCGCTCGTCGCCGTGCCCGTCTCGATCGTCGGTACCTTCGCCGTGATGTATGTCTTCG
GCTTCTCGATCAACGCGCTCAGCCTCTTCGGTCTGGTGCTGGCGATCGGCATCGTCGTCGACGACGCCATCGT
CGTCGTGGAGAACGTCGAACGCAATATCTCCCAGGGCCTCTCACCGGTGCAGGCGACCTATCGCGCCATGCAG
GAGGTCTCTGGGCCGATCATCGCGATCGCGCTGGTCCTCGTCGCGGTCTTCGTGCCGCTCGCCTTCATCACCG
GGCTCACCGGCCAGTTCTACCGCCAGTTCGCGCTGACCATCGCGATTTCGACGGTGATTTCGGCCCTCAACTC
GCTGACGCTCTCGCCGGCGCTCGCCGCCCTTCTCCTGAAGGACCACCACGCCCCGAAGGACCGGCTGACGCGG
GTCATGGATGGGTTGTTCGGCTGGTTCTTCCGCGGCTTCAACCGCTTCTTCGGAGCGAGTTCGGAGGCCTATG
GCCGCGGCGTCGGCGGCATCCTCACCCGCAAGTCGCTGGTCATGGGCGTCTATGTCGTCCTCCTCGGCGTCAC
CTTCGTGCTCTTCCGCGCGGTGCCCGGCGGCTTCGTGCCGGCGCAGGACAAGCAATACCTCATCGGCTTTGCG
CAGCTGCCGGACGCGGCGACGCTCGATCGCTCGGAAGATGTCATCCGGCGCATGAGCGAGATCGCCCTCAAGC

ACCCGGGCGTCGAACACGCCATCGCCTTCCCGGGCCTGTCGATCAACGGCTTCACCAATTCGTCCAACTCCGG
CATCGTCTTCGTGTCGCTGAAGCCGTTCGAGGAGCGTACGACGCCCGAACTTTCGGGCGGCGCCATCGCCATG
CAGTTGAACCAGGAGTTCGGGGCGATACAGGATGCCTTCATCGCCATGTTCCCGCCACCGCCCGTCCAGGGTC
TCGGCACGACCGGCGGCTTCAAGCTGCAGATCGAGGACAGGAACGGCCTCGGCTACCGGACGCTGGACGATGC
GACCAAGGCCGTCCTCGCCAAGGCGATGGCGGCACCGGAACTCGCCGGGCTCTATTCGAGCTTCCAGATCAAC
GTGCCGCAGCTCTATGCCGACCTCGACCGCACCAAGGCCCGTCAGCTCGGCGTCGCGGTCACGGACGTGTTCG
AGACGCTGCAGATCTATCTTGGCTCGCTCTACGTCAACGACTTCAACGCTTTCGGCCGCACCTACAGCGTGCG
CATCCAGGCCGATGCGAGCTATCGCAGCCACGCCGACGACATCGGCAAGCTGAAGGTGCGTTCGCAGACGGGC
GAGATGATCCCGCTGTCGGCCCTGCTCAACGTCGAGCAGACGGTCGGCGCCGAGCGGGCCATCCGCTACAACG
GCTTCCTCGCCGCCGATATCAATGGCGGACCGGCACCCGGCTTCTCTTCCGGTCAGGCGCAGGCGGCCATCGA
GCGGATTGCGGCCGAGACTCTGCCGCCCGGCATTTCATACGAGTGGACGGACCTGACCTACCAGCAAATCCTC
GCCGGCAATTCTGGCATCCTCGTCTTCCCGCTGGCGCTGCTGCTCGTCTATCTCGTGCTTGCGGCCCAGTATG
AGAGCCTGCTGCTGCCGATCGCGATCATCCTGATCGTGCCGATGGGCATCATGGCGGCGCTGACGGGCGTCTG
GCTGACCGGCGGCGACAACAACGTCTTCACCCAGATCGGCCTAATCGTTCTGGTGGGTCTGTCGGCAAAGAAC
GCTATCCTGATCGTCGAATTCGCCCGCGAACTGGAACTGTCCGGCAGCAATGCCGTCAGTGCGGCGATCGAGG
CGAGCCGGCTCCGGCTCAGGCCGATCCTGATGACCTCGATGGCCTTCATCATGGGTGTCGTGCCGCTCGTCAC
CTCCACCGGCGCCGGCGCGGAAATGCGCTCCGCCATGGGCGTGGCGGTCTTTGCCGGCATGATCGGCGTCACG
GCCTTCGGCATCTTCATGACGCCCGTCTTCTATGTGCTCATCCGCAAGCTTTCCGGCGAAAAGCCGCTGAAGC
ACACCGGAGCGCATATAGAAGCCCCGCATCTCGCCCCCGGCGAGTAGATGTCACTGACAGATGGCCCCTATGC
GGGGCCGCCCCAGCCGGATGTGGTCGACCCGAGCGGCGGGGCCAATCAAACGCGCGTGCGCGCCTATAACGAA
CGGCTGGTCATGTCGCTGGTGCGCCGTCACGGCAGCTTGTCCAAGGCCGAGATCGCGCGCCGATCCGGTCTAT
CGGCGCAGACCGCCTCCGTCATCATGCGGTCGCTCGAAGCCGACGGGCTGCTCGTCCGCGGCGCGCCGGTGCG
CGGCCGGGTTGGACAGCCCTCCATCCCCATGCGGCTCAATCCGGATGCGGTCTATTCCTTCGGCGTGAAGATC
GGCCGGCGCAGCGCCGATCTGGTGCTGATGGATTTTCTCGGCACCATCCGGCTGCACCTGCATCAGATCCATA
CCTATCCGCTGCCCGAGGAGATCGTCAGCTTCATCGTCGGGGGCATCGGCAAGCTCGAGGGACAGCTTTGCCC
CGGCGAGCGCGGCCGCATTGCCGGCGTCGGCATCGCCACGCCGTTCGAACTGTGGAACTGGTCGGAGGAAGTC
GGCGCGCCGCGCGAAGAGATGGACAGGTGGCGCGATTTCGACCTGCAGGCGGCGGTCGCCTCGCGCATCCGGC
ATCCGGTTTTCCTTCAGAACGACGGAACCAGCGCCTGCGGTGCCGAACTCGCCTTTGGTGTGGGCGCCAGTTA
TCCCGATTTCGTCTATTTCTATATCGGCTCCTTTATCGGCGGCGGCGTCGTGATCAACTCCGCCCTCTTTTCC
GGACGAACCGGCACCGCCGGCGCCGTCGGCCCGCTGCCTGTCGCCGGCAAGGACGGCAAGTCGACGCAGTTGC
TGAAAATCGCCTCGGTCTTCGTCCTGGAAAAGCTCCTGCGCGAACGCGGCATCGACCCGGAGCCGCTCTGGTA
CTCCGCCGACGACTGGATCGATTTCGGCGAACCGCTCGAAATATGGATTCAGGATGCGGCGGCAGCGCTTGCG
CAAGCCGTCGTCTCCGCGGTTTCCGTCGTCGATTTTTCGGCGGCCGTGATCGACGGCGGCTTTCCGCCCTGGG
TGCGTGCGCGCCTTCTTGCCGCGACGCGAAAGGCCCTCCAGACGCTCGACCTGCAGGGCGTCACGGTTCCGGA
CCTCGTCGAAGGCACCGTCGGCAGCCACGCCCGCGCGATCGGCGGCGCAAGCCTGCCGCTCTTCTCCCGCTAT
CTGCTGGACACCAATGTCCTCTTCAAGGAGCTTTAATTGAGGAACGAGGATATCGTGTTGGATGGCGCGAAGG
TGTTCTGGCGCACCCTGCTGCCGAGGTTGAAGGCGGAGCGCGGGCTTCTCGGCCTGTCCGTCGTGCTCGCCTT
CCTCGCCGCACTCGCGGGCATCCATCCGCTGGCCGTCCTGCCTTTCTGGATCGTGCTCGTCGCGGCCATCCTC
AACCGGGGAGCGCCGCCCGTCCAGACAGCGCCGGCGAGCGTCGGCGCTGCTGTGGAAACCAAGGCCGACCCGC
TTGCTCCCCTGCTCGACGCGCTCGACATGCCGGTGCTCGTCGTGGCGGCCGACGAGACCGTCGTCTTCCAGAA
CGCCGCCGCAGAGAAGGCCTTCGGAACTATCCCCCAGGACAGCTACCTTTCCGCCCGCGTCCGTTCGCCCGGC
ATTCTCGACATGGTGCGCGAGACGATCGCGACAGGCAAGGTCAACCAGATCGAGCATGCGGAAAGGCTGCCTT
CGGAGGCGGTCTACGTGGTTCGCGTCGCGCCCGTGGACATCGGCTCCGACGTTTCCGGCCGGCGCATCTTTCT
GCTGACCTATCGCGATATCTCGCAGGCTCGCCGCATCGACCGGATGCGCTCCGATTTCGTCGCCAATGCCAGC
CATGAGCTGCGCACGCCGCTCGCATCGCTGCGCGGGTTCATCGAGACGCTGCAAGGCCCTGCCCGCGACGACC
GGAAGGCCCAGGAGAAATTCTTCGGCATCATGCACGAGCAGGTCACGCGCATGAGCCGGCTCGTGGACGACCT
CCTGTCGCTGTCGCGCCTCGAGTTGAAGTCGCACATCGCGCCGGACGATGCCGTCGATCTCGGGCCGCTGCTC
GGACACGTGCGCGACAGCCTGGCGCCGCTTGCGGGCGAACTCGACGTGACGATCGCGCTGGAGGTTCCGGAGA
CTCCGGTTTTCGTCCAGGGGGATCGGGACGAATTGGTGGAGGTCTTCGAAAACCTGATCGAAAACGCCTGCAA
ATACGGGCAGGAAGGCAAGCAGGTGGATGTGCGGCTGACCGGCGGGAATGGTCACCAGGCGGAGGTGACGGTT
ACCGACCACGGTCCGGGAATACCTGCCGAGCATGTCCCGCGCATCACCGAGCGTTTCTACCGGGTCAATGTCG
AGGCGAGCCGGTCGAAGAAAGGCACCGGCCTGGGCCTTGCGATCGTCAAGCATATCCTCACGCGTCACCGCGC
CCGCCTGCTCATCCATTCGGAGGTGGGGAAGGGCACGGTCTTCAAGGTCCGCTTCTGAATGGAGCAGAACCTA
TCCACGATACGCAAAGCCGCGGGTAGCGAGGGAGAGCCGACCATCGCGCTTGCGCTCGGCGGAGGCGGTGCGC
GGGGACTTGCCCACATCCATGTCATCGAGGCGCTCGATGAGATGGGGCTCCGCCCCGCAGTCGTGGCCGGTTC
GTCGATCGGCGCCCTCATGGGCGCGGGCGTGGCCGCTGGCATGAGCGGGAAGGAAATCCGCGAACACGTGCTT
TCGACCGTCGGCCGGCGTGGGGAGGTGCTCAACCGCCTATGGCGACTGAGGCCGACCAGTCTTGCCGAGGCTG
TGGCGAGCGGTTTCCGCGTCGGCCAGTTCAATATAGAGCGCGTGCTGAAGGCCTTCCTGCCCGAGGCGGTGCC

TCTCCGCTTCTCCGATCTGGCACTCCCCCTTAAAATCATCGTCACGGACTATTACGGGCAGACGGAGCGGGTA
TGCGAAAGCGGCGACCTCTACAAGGCGCTCGCTGCCTCCTGCGCCCTGCCGGCGGTCTTCATGCCGGTGAAGA
TCGACGGCCGCGTGATGATCGACGGCGGCATCTATAATCCGATCCCCTTCGATCACCTGCGCGGACTGGCAGA
TATCGTCGTCGCCGTCGATGTCGTCGGCGGGCCGGACGGTGACGGCGAGACGATCCCGAGCCGTATCGACAGC
CTGTTCGGCGCGACCCAGTTGATGATGCAGTCGATCATCACCATGAAGATGCAGGCCGGCGCACCGGATATTC
TCCTGCGTCCCGATGTCGGACGGTTCCGCGTGCTCGATTTCCTGCGCGCCCAGGAACTGCTTGCCGCCACCAG
CGGGACCAAGGACGAGGTGAAGCGCGCGCTGGCAGAGCGCATCGATCGGTGGAGGAAAGGGATATAAATGGTT
TCAGAAGCGCCCCCGTTCTGGTGGACCAAGGCCGACTGGCGCGCCTACGCTCTTTGGCCCTTTTCCTGGGTGT
ACGGCCGGATCGCCGGGATGCGCATGGACCGGGCGCGGCGGGCGACCTCCGCCGTACCGCTGATCTGCATCGG
CAACTTCACCGTCGGCGGAGCCGGAAAGACGCCGACGGCGATCGCGATTGCGCGGGCCGCCAGGGCACGCGGC
CTGAAGCCCGCTTTCCTGAGCCGCGGTTATGGCGGGTCGCTGGACGTCACGACCGTGGTCGACCCCGAGCACC
ATCGCGCGCGCGATGTCGGCGACGAGCCGCTGCTGCTTGCCCGCGAGGCGCTGACCGTGATCTGCCGCCGCAG
AGTGGATGGCGCACGCAAGCTTGCCGCGGAAGGCGCTGACATCATCATCATGGATGACGGATTCCAGAGTGCC
CGGCTCGTCTTCGATTTCGCCCTTCTCGTGGTCGATTCCGGCCGCGGTATCGGCAACGGCCATCTCGTGCCTT
CCGGCCCCGTTCGGGCGCCGATCGGCAATCAGCTGCGCCATGCCAACGCTCTGCTGAAACTCGGCCACGGTTC
CGCCGCCGATCCTCTCGTCCGCCGGGCGGCTCGGGCCGGCAAGCCCGTCTATGTGGCCGAAACGGTGCGCACG
GATGCAGGGTCGCTCGACGGCGTCAAGGTCCTTGCCTGGGCCGGAATTGCCGATCCGGAGAAGTTCTTCAAGA
CCGTTCGCGAGACCGGCGCGGTCATCGAAGAGACCCGGAGCTTCCCGGATCACCATCATTTCTCCGAGGACGA
GATTGCCGACCTAATCGACCGCGCCGCAAGCCGGGGCTACACGCTGGTGACCACGGCCAAGGACATGGTCCGT
CTGGAGCCCGGCCACGGGCGGGCAGGGGAACTGGCGGCAAAGAGCCGCGTGATCGAGATCGAAGTGCGCTTCG
ACGATCCGGCAGCGCCGGGAAAGATCATCGACGCGGCACTTGCCTCCGCCCGTGCCCGCCGATTGCGCGAGCG
TAAGGCGGGCTAGTTGGCAAGAAAAACGTCCCGCAGCATCCGATATGCCGACCTGAACGCGGACGACAATGCG
GAGACGCGTCGGCTGATCGATGCCGGCAGCCGCCTTGCCGAGGAGGTCGCGCGTCTCTTTCCGCCCGAGCCCG
ACATGGCCGACCGCCACTACTGGCTCGAAGCGATGATCAACCACGTTCCCGATTATATCTATGCGAAGGATAC
GGCGGGGCGCTTCCTGATCGCCAACGAGGTGACGGTTGCCGACAACGGCCTCGAAAGCCTGCGCGACCTCGTC
GGCAAGACCGATTTCGATCTGCACCCGCACGAGTTCGCCCAGGCCGTCGCCGATACCGAGCGACGCGTTTTGG
AAACCGGCGAACCGATCTTCGGCATCGAGGAACGCGCCATCGTGACCAAGGACCGCGATCGCTGGCTGATGAC
CTCGAAAGTGCCCTTGCGCAGCAAGAGCGGGAGGATCATCGGTATCGTCGGCATCTCGCGCGATATCAGCGAT
CGCAAGGCCGCGGAGCGCCTGCTCGAAGGTCAGGCCCGGCTGCTGGAAATGATCGCCAACGGCAAGCCCCTTG
GCGAGTTCTTCCACGAGCTGGTGCTGCTGATCGAAGCGCTGCTGCCCGGGATCAAGGGCTCCATCCTGCTCCT
TTCGGAGGATGGCCGGCATCTCCTGCACGGCGCTGCCCCCAGCCTCGACGCGGGCTTTTGCGCTGCCATCCAT
GGCCTCGAGATCGGCCCTGCGGCCGGCTCCTGCGGAACGGCCGCCTGGTGCGGCGAACAGGTGATCGTTTCCG
ACGTCTTCGCCGACCCGCTCTGGGAGGAATACGCGCCTCTCCTGAGGCCTTCCGGCCTCCGTTCCTGCTGGTC
CACGCCCATCCTGTCGCGCGAGCGAAAGGTGCTCGGGACATTCGCGCTCTATGCGCAGGAGGTCGGAACGCCG
AGCGAGCAGCAGCAGGAACTGATTGCCATGGCCGCTCATCTCGCCGGAATAGCAATCGAGCGCAAGCGGGCGG
AGGACCGCATCGGTTTCATGGCCCATCACGATGCCTTGACGGGGCTGCCGAACCGCGTGCTTTTCGAGGAGCA
GGTCGCCGGCATGCTCGAGGAGATCCGCGAGCGGGATCAATGGGCCGTGCTCGCCTTTCTCGACCTCGACGAT
TTCAAACTGATCAATGACAGCCTTGGGCACGCCACCGGTGACGAGCTCCTGAAGATCGTAGCCGGGCGCATGC
GGGCCGCCGTCCGGAGATCCGATTCCGTCGTGCGCGTCGGCGGCGACGAGTTCATCATCCTCCTGAACGGCCT
GCCGAAGGAGCGGGACGTCGTGCTATCGCGGCTGGAGGATATCCGCACTGCAATCGCCATGCCGGTGCAGTTG
CAGGGCCGCTCTCTGCAGGTGAGCTGCAGCATGGGCGTGGCCTGCTTCCCCAACCAGGGCAAGACGGCGGGCG
AACTGCTCGCCAATGCCGACATGGCCATGTACCGCGCCAAGGAGCTCGGCCGCAACAACATCCAGGTCTTCAC
CGAGGAGATGGCCGCCAGAGCCCATGAAAAACTCCAGTCGCAAGAGGAGCTTCGCGAGGCGCTTGCGCGGGAG
GAGTTCCTGCTGCATTTCCAGCCGCAGATGAGCCTCGAGACCGGCCGCATCTTCGCTGCGGAGGCGCTGTTGC
GCTGGCAGCACCCGGTGCGCGGCATCATCTCGCCTGCGGCATTCATCCCGCTCGCCGAGGAAACCGGTCTGAT
CGTGCCGATCGGAGATTGGGTCCTGCGCACCGCCTGCCGCCAGTTGAAGGCCTGGCAGGACGCCGGCCTGCCG
CCGCTGATCGTCAGCGTCAATGTTTCCGCGCGGCAATTCCGCGAGCGCAACTGGGCAGCGCGTGTGGCGGCGG
TTTTGGAGGAAACCTGCCTCGAGGCCCGTTACCTCGAACTCGAGCTCACCGAAAGTCTGATCATGCAGGACGT
GCCGGGAGCGATCGCCACAATGCATGCGCTGGAGGCGATCGGCGTGCACCTTGCCATCGACGATTTCGGGACA
GGCTATTCGAGCCTCAGCGCGCTCAAGCGCTTCCCGGTGCGCCGGTTGAAGATCGATCGGTCCTTCGTGACGG
ACATTCCGCACGATGCCGACGACATGGCGATTACTTCCGCGATCATTTCGCTTGCGCAGAAGCTCGGTCTGCG
CGTCATTGCCGAGGGTGTCGAGACGGAGGCCCAGGTCGAGTTTCTGCAAAAGAGCGGCTGCGGCGAGATCCAG
GGCTACTTCTTCAGCCAGCCCTTGCCGGGCGAGGATTTCGAAGTGCTGCTTTCCTCTGAGCGGCGGTGAATGG
ATGCCGCGGCCCTGACATTCCTGGCCCTCGGTCTTCCCTTTGCCGCGGCCGCCGTCGCACCGGCCCTGACCCG
GCTTCTCGGCGGGAATGCCGCCTGGCTGCTGGCGCTTGCGCCACTCGCCATCTTCATGCATCTCGCAGGTTTC
ATTCCGGAAGTCGCCGCTGGCGAGATCGTTACCGGCGGCTATATCTGGATACCGTCCTTCAACGTCAGCTTCT
CCTGGCTGATCGACGGCCTGTCACTGACCTTCGCGCTTCTGATTTCGGGCATCGGGACACTGATCGTCCTCTA
TTCGGGCGGATATCTGAAGGGGCATCCCGACCAGGGCCGCTTCTTTTCGTTCATCCTGATGTTCATGGGCTCG

ATGCAGGGGCTCGTCGTCTCGGACAGCTTCCTGATGCTCTTCGTCTTCTGGGAGCTGACGTCGATCACTTCGT
TCCTGCTGATCGGCTTCGATCACACCCGCGAGGCGGCGCGCCGCGCGGCTTTGCAGGCCCTGGTCGTGACGGG
GGGAGGCGGGCTTGCCTTGCTCGCCGGGCTCCTCATCCTGTGGAACGTCAGCGGCGTCACGCAATTTTCGCTG
CTCCTGTCCTTCGGCGACGAACTGAAGCAAAGCCCTTTCTATCTCGCGGCGCTGCTCCTCGTCCTCGGCGGCG
CATTCACCAAGTCGGCACAGTTTCCGTTTCACTTCTGGCTGCCGAACGCCATGGAAGCGCCGACGCCCGTTTC
GGCCTATCTGCACTCGGCGACGATGGTGAAGGCGGGGGTCTATCTTCTGATGCGGCTCAACCCGGTGCTCGGC
GGTACGACGGAGTGGCAGGTTCTGCTGCCGCTCTTCGGCGTGACGACGCTCGTCGCCGGAGCCGCCCTGGCAG
TGCGCCAGACGGACCTGAAGATGATGCTGGCCTATACGACCATGTCGTCGCTCGGTCTTCTGGTCATGCTGAC
GGGCCTCGGCCTGCCCTACGCCATCGAAGCCGCAGTGCTTTACTTGGTGGCGCATGCCCTGTTCAAGGGGGCG
CTGTTCATGGTGGCCGGCATCATCGATCATGAAACCGGTACGCGCGATCTGACGAAGCTCGGCGGCCTGCGCA
GGGCGATGCCTCTGACCTTCGCCATAGCTCTTGCGGCGGCCCTGTCGATGGGCGGCCTGCCGCCCTTTTTCGG
GTTCCTTGCAAAAGAGGAAATCTACGCCGCACTCTCCGGTTTCGACCGCCGGTCCATGGTCTTCGCGGCATTG
ACCATCCTCGGCAACGGCCTGATGTTCGCCGTCGGCTTCGCGATCGCTCTAAAACCGTTTCTGGGCCCGAAGG
TCGATACGCCGAGGCATGCCCACGAGGCACCGGTGCTCCTCTGGATCGGCCCGGCAATCCTGGCTGCGAAGGG
CCTTTCCATCGCGCTGATGTCGGGCCTTGTTCATCGGTTCGTGACCTCGCCCATGGCCTCGGCCGTTGCCGGC
GAACCGAGAACCGTGACGATTTCTCTCGCACCGCATATCGGCGTGCCGCTGTTGCTCTCCGTCGTGACGCTGG
CGGTCGGCGTCGTTCTGTTCCTCAATCTGACGCGGCTGCGCGCAGCCATGGCGGTGATCCTTGCCGATATCGG
CTGGGGACCGGATCGAGGCTTCGACCAGTTCATACGCGGCCTCGTGCGGTTTGCGGTCGCCGCGACCCGCCGA
TTGCAGGGCGGCCGTCTCGATAACTACATGACCGTGACCTTCCTGCTCGTCGCCGCCGTCATGCTCGCATTGC
CGCTGCGCTACGGCGAATTCCCGCGCGCACCTTTCTTTCCGGGCGACGTTCCGCTGCACGAATTCGCGATCAT
GGCGATCGCCGTGCTTGGCCTCTTCGCCGTGGTGCTGGCCGCGGACCGGCTGACGGCGATCGTCTCGCTCGGA
ATTCAGGGCTTCGCCGTCGCGATGCTCTTCCTGCTTTACGGCGCTCCGGATCTCGCCTTCACCCAGTTCATGA
TAGAGACGCTGTCCGTCGTCGTGCTGGCACTGGTGATGACCCGGCTGCGGCTGTCTCCTTCGGATCACCGACC
ACTCAGGCAGAAGCTTCCCGACTTCACGATCGCGCTCGCCTGCGGAATCGGATTCGGCCTTTTCCTTCTCCGG
GTTACCGGCGTGCCGTTCGATGCGACGCTGACGGACTTCTTCAACCTCTATTCGAAATCGATCGCGCACGGCG
CGAACGTGGTCAACGTCATCATCGTCGACTTCCGAGGCACGGATACGCTTGGCGAAATTGCCGTCGTGCTGGT
TGCGGGCCTGTCCATCCTTTCCCTCGTGCGGTTGCGGGCGGGATCGTTGAGACGCGGCGAAACGCGCGAGATC
GCGGCCGCTGATGCGAACACGAAGGAGCCGGTATGAGTGGCCCGAGGTCAAGGCAAGGTTATTCCAGTCAGCA
GGGTTCAGCTCGTTCAGCCACAGACCAACGGGAGGATCATACGCATCCTGGTCACAGAGGGGCAGCCGGTGAA
GGCTGGTGACTTGTTAGTCGGCATGGATACCGTCTCGGTTGAGAGCCAGATCAAGCTCAGGCTGACCGAGGTT
CGTCGCCAACGTCGCGGGGCGACGGTTGCCCGAGTAATCATTGCGGCGCTGACAAATGTTGCAGTCGCAAACG
ATGATGTGCTTGCGGCAGGCAGAGCTGCCTTCGAAAGGGAGCGGCATGACCTGGAGGTTATCGACGGAGCGGA
GGCACTTATAGAGGCTGTGCTAACGTCTCTTCGAGATCAGGTTGCGCGGATCAATGCGCAACTTGATCGTGTC
GAACAATCGCGAATAACCCAGCAGGCGCGACTGCAGAAAGCTCGGTCTGATCTGGATATTTCCTCCAGGAAAT
TTTTGTCAGCCGGCAAGTTGAAGGACAAGGGAGCCATCAGCGAAGTAGACTACTTCGAGCGATTGCGTGAGCA
AAGAGCACGCGAACTGGAGGTGCTCATCTCCGAGAGCGAATTGAATATCCTTGCAGCGGAAGCGAACGCGTTG
GCTAGACAGCGGCAGAGCATCATTTCGACCGCAGTCGAGAACTATCAGAAACAACTGAACGAGGCGAGCACGT
CCCTTGCAGGGCTGGAAGCGGAACTGAGCGCGGCTAGGAACCAACTGGCCTTGCTCGCGATTCGCGCACCAGC
AGATGGCAAGGTCGAGAACCTCACGGTATTTACCATCGGCGGTTTCGTGGAAGCAGGTTCAACACTCATGTCC
ATCGTACCTTCCGGCGATGGCATGGAGATTGAAGCTTTCTTCGACAACCGTGACATCGGCTTTCTTGAAAAAG
GGCAAGAGGCATTTGTGAAGTTTGACGCCTTTCCGGCCGAACGTTTCGGTATTGTTCGCGGTCGGGTGACTAG
AGTTGGTGCGGATGCGCGCGGCGACATCGTCCCAGGGAAATGGGTCTACGCAATCCACCTTGTACTCGACCAA
GAGACAATTGGGATAGGCGGGCGCGACATAGGGTTCTCGCCCGGCATGACTGCGACGATCGATGTGATAACGG
GTGAACGGCGGCTCATTAGTTACTTCTTCGAGCCAATAATCAAGGCAATTCAAGACGGATTAGGGGAACGTTG
AATGGCCTATTTTCTGCAGCAGATCGCCAATGCCGTACCCGTGGCCGCGCTTTATGCGGCGCTCGCCTTCGGA
TACGCGATCGCCTTTGCTGTCACACGGCGGGCGGACCTGACCTATGGCGCGCTCTTCGCCTTTGCCGGGCAAA
TCTTCGTGCTCTTCGCGGATGTCGGCTGGAACCGGCTGTGGCTGGTACTGCCGGCGGCGCTCGGGCTCGGGGC
GACAGCGGCGCTCGCATTCGGGCTAGGTGCCGGTCTTATTGCCGGCCGCTACGTCATGCGGCCCCTGGCTTTT
TCCTCCGCCAATGCCGTGGTCGTCGCCTCGCTCGGAAGCCTGCTGGTGCTGATGGAAACGGCCCGGCTCGCCT
CCGACACCAGAAGCCTCTGGCTGCCGCCCTTCCTGAACGACGTGGTGGTGTTCTGGCCGAACCCTGCCTTTCC
GGTCGCGCTGACGGTCGTGCAGCTCGTCAACACCGCGCTCATGATGGCCCTTGTCGCCGGCGGCCATTGGCTG
TTGACCCGTTCCTATGCAGGCCGCTACTGGCGCGCCGTTTCCGAGGATCGCAAGGCCGCCGCGCTTTGCGGCA
TCGATCCGGGAGCGGTCTATATCGCCGCCTATGGCGTGGCGTCGCTGATCGCTGCGTTCTGCGGGATTCTCGC
TGCTTCCTATTACGGCAACATGGATTTCGGCACCGGGCTGACCTTCGGGGTCAAGGTCCTGTTCATCGCCGCC
ATCGGCGGGCAGACTTCGCCGTTATTCGCGGCACTGGGCGCAGCCGGCATCGGCCTCATGGAGACGGTGTGGA
GCGCCTATGGACCCATCCTCTGGCGGGACTTCGCCATATTCGGCTTTCTGGTGATCGTGCTCGTGGTCACGCG
CCGGGAAAAGATCATTCCCTGAATGAACGACAGGACAAATCTTGTGGACAGGATCACCGGACACCGCCGCATG
CGCCGCAATCGCAAGGCGGATTGGACGCGCCGGCTGGTCCGGGAGAACCGCCTGACGGTGGACGACCTGATCT

GGCCGATCTTCGTCGTGCCCGGCAGCGGCATCGTCGAGCCGATCGATGCGATGCCGGGCGTCAACCGCATGAG
CGTCGACAAGGCGGTCGAGGCCGCGAAGGAGGCGGCCGGCCTCGGCATCCCGGCCATCGCCACCTTCCCCAAT
ATCGACATGGCGCTTCGCGACGAGACCGGATCCGACAGCCTCAACGCCGACAATCTGATCAATCAGGCGACGC
GGGCGATCAAGAAGGCCGTACCCGACATCGGGATCATCACCGATGTCGCGCTCGACCCGTTCACCAGCCACGG
GCACGACGGCATCCTGCGGGGCGGCGAGATCGTCAACGACGAGACGGTGGAGGCGATCGCCAGGGCGGCGGTC
ATTCAGGCCGACGCCGGCTCCGACATCATCGCGCCCTCCGACATGATGGACGGGCGGATCGGCGCGATCCGCG
AGGCGCTCGACGCGGCCGGCCACCAGAATGTCGGCATCATGTCCTATGCGACGAAATTCGCCTCCGCCTTCTA
TGGCCCTTATCGAGAGGCGATCGGCACCGGCGGCCTGCTCAAGGGCGACAAGAAGACCTATTACATCGATCCG
TCGAACGGTACGGAGGCGATCCGCGATGCGGCACTCGATGTGGAGGAAGGCGCGGACATGCTGATGGTGAAGC
CCGGCCTGCCCTATCTCGATATCTGCTGGCGGATGAAGGAAGCCTTCGGCCTGCCGGTCTTCGCGTATCAGGT
TTCCGGCGAATACGCGCAGATCAAGGCAGCCGCCGCCAATGGCTGGATCGATGGCGAGCGGGTGATGCTGGAG
ACGCTGCTCGCCTTCAAGCGGGCGGGTTGCGACGGGATCCTCAGCTACTTTGCCGTCGACGTGGCTCGAATTC
TCGCGAAACGCTGAATGTTTACAGGCATCATCACCGACATCGGTACGGTCGAGAAGGTCACACCTCTCGAGGA
AGGCATCAAGCTTCGCGTCGCCACGAATTACGACCCGCAGACGATCGATCTGGGTGCCTCGGTCGCGCATGCC
GGCATCTGCCTGACCGTCACGTCACTTCCGGAAGCCGGCAGCAACGAACGCTGGTTCGAGGTCGAGGCATGGG
AGGAAGCGCTGAGGCTGACGACGATCGCCGACTGGCGTGAGGGCACGCGCATCAATCTTGAGCGCTCGCTGAA
GATCGGCGACGAACTGGGCGGTCACATCGTCTCCGGCCATGTCGACGGCAAAGCGGAGATCCTTGCCGTCGAA
GCGGAGGGGGAAGCCGTGCGCTTCCGCCTCAGGGCACCCGAACATCTCGCGAAATTCATCGCGCCCAAGGGCT
CGGTGGCGCTCGACGGGACGTCGCTCACCGTGAACAAGGTGGATGGCGTCGAATTCGACGTATTGCTGATCCG
ACATTCGCTCGAAGTGACGACCTGGGGCGAACGCAAGTCCGGCGATCTCGTCAATTTCGAGGTGGACACGATG
GCGCGCTACGCCGCGCGGTTGGCGGAATTCCCGGCTCCCAAGCAGGAATAAATGACGCTCAAGATGAAGATGA
TTGCCGCAGCATCCCTGATCGCGCTTGCTGCCGGCGTCGCCCTGCCCGGGCCGGCGGCGGCGCTCGATGCCAA
GCAGAAGGAGGAATTCGGCGCCTTCATCAAGGAGTACCTGCTCGCCAATCCGGAGATCATGCTGGAAGTGCAG
GAAGCGCTCACCTCCAAGCAGCGGGCCAAGCAGCAGGAAACCGCCCAGGCGGCAATCGCCGACAACAAGAAGG
CGATCTTCAACTCTGACTACGACATAGCCCTCGGCAATCCGGATGGCGATGTGACGATCGTCGAGTTCTTCGA
TTACAACTGCGGCTATTGCAAACGGGCCATGTCCGACATGGACGACATCCTCGCCAAGGACAAGAATGTCCGA
TTCGTTCTCAAGGAGCTCCCAATTCTCGGACCGGATTCGCTCGCGGCCCACAAGGTCAGCGCCGCCTTCCGCG
TGATCGCACCGGAGAAATACGGCGATTTCCATCGCGCCCTGCTCGGGGCAGAGGAACGGGCGACGGAGGAAAC
CGCGATCGCCGTTGCGGCCAAGCTCGGGGTGACGGAAGCGCAGTTGCGCGAAAAGATGGATGACGGGCCGTAT
GATGCCTCGGTGCGCGAAGCCTATACGCTCGCCAACGAGCTCGGCATCACGGGAACGCCTTCCTATGTCGTCG
GCAACGAGGCGGTCTTCGGCGCTGTCGGGGCGCCTGAGATCGAACAGAAAGTCGCCAACATGCGTGCGTGCGG
CAAGACGGTCTGCTGAATGTCCGAACACAGCGCGACGACGACACCTGAAGCGCAAATGCGCACGGAGGCGACC
GGAACCAACCGGATGGTTCTCCTGGATGCGCTGCGCGGCCTGGCACTCGTCGCCATGGCCCTCTATCACTTTG
TCTGGGACCTCGAATTCTTCGGCTATGTCGCGGCCGGCACCGCCGGGACCGGCGGCTGGCGGATGTTTGCACG
CTTGATTGCCAGCAGCTTCCTGTTTCTTGCCGGTTACAGCCTCGTGCTCGGCCAGCTCCCGAAACTCCGTCCG
CGCGCCTTCGCCATACGTTTCGCGAAGATCGCCGGCGCAGCCGCGCTGATCAGCATCGGGACCTGGTTCGCCT
TCCCTCAGTCGTTCATCTTCTTCGGCATTCTCCACGCCATTGCGGCGGCAAGCCTTGTCGGCCTCCTGTTCCT
GAAATTGCCGGCCGCCGTTTCCTTTCTTGCCGCTGCCGTGGCCTTCGCGGCTCCCCTCTATCTGCGCTCGTCG
TTTTTCGACATGCCGGCGCTCTGGTGGGTCGGCCTCTCGGAAACGATCCCGCGCTCGAATGACTACGTTCCTC
TGCTTCCCTGGATTGCCCCTTTCCTGCTCGGCCTCGGCACCGCGAAGCTGCTCCACCCGTTATTTTCCGCCCG
CCGCAGCCGAAGCGCCGGGACCCGTAAGCACCCGTGGATGACGCCTCTCGCCTTTGGCGGCCGTCACAGCCTT
CTGATATATCTCATCCACCAGCCGCTGCTTATCGCGGCAGTCTATCTCTTCTCTCTCGCCGTTCCCGCCCCGG
CACCGGATCCGGCACGGGTCTATCTGCTGAACTGCGAACGGGCCTGCAGCAACGAAAATTCCGGGATCTCCTG
CAGCGCCTTTTGCGGTTGCACGCTCGCCGGCCTGAAGGAACAGAACCTGTTCGACGATCTGAACCTGGGCAAG
ATCGACGTGAACACGGATGAACGCATTGCGCGAATCGCCGGTCAGTGCACAATGGATGCACAATCAGGAGAGG
CACAGTCAGGGGACTAGATGAACTTCGAAGCAGCCCGCATCAAGATGGTGGACAATCAGATCCGGACCACGGA
TGTGACCTCCCATTCAGTCCTGTCTGCTTTCCTGTCGGTCCCGCGTGAGGAGTTCGTGCCTGCCAAGATGCGG
GAGCTGGCCTATATCGACACCGATATCGAGCTCGTCGGCGGTTCCCAGCCGCGTTACCTGATGGAGCCGTCGC
CGTTGGCAAAGCTCCTCCAGCTCGCGGAGATCTCCAAATCGGATCTGGTCCTCGAGATCGGCTGCGGGACGGG
TTACGCCTCGGCTCTGCTTTCGCTTCTCGCCGGTTCCGTCGTGGCGCTGGAGAGCGACGAAGCCCTGGCCGCC
ACGGCCACGGCAACGCTGGCACGCCTCGGTTACGACAACGTCGCCGTGGTGAGCGGCGATCTGACCAAGGGAT
ATGCGGCGGAAGCGCCCTATGACGTCATCTTCATCGACGGTGCCGTCGAGATGCTCCCTGCCGAGCTGTTCGA
GCAGCTTCGCGACGGCGGCCGGCTCGTGGCCGTGGAAGGTTTTGGTAACGCATCTCGTGCGAAACTTTACGTG
CGCGAATCGGGCATGACGTCCGAGCGAGCTGACTTCAACACCGCCGTGAAGCCGCTTCCCGGCTTCCGCCGCG
ACCGTTCTTTTGTTTTTTGAATGGTTTCAGATGCGCGTAACGGCGCCGTCGCGATGACGAGGCGCTCGGGTGT
GTCGTTCGCAGCGAACCTGGCATTCGAGCATATCAGCCATCGTTACCATTCCAAGGATACGATCAAGGGCGTA
ACGCTCAAGGCCGAAGCCGGCGAAGTGCTCTGCCTGCTCGGACCGTCCGGCTCCGGCAAGACAACGCTGTTGC
GTATCGCCGCGGGCATCGAGAAGCAGACGGGCGGGCGCCTTCTGCTGAACGGACAGGAAATATCCGGCCCTTC

CATCTTCGTTCCGCCCGAGCAACGCGGCGTCGGCCTGATGTTCCAGGACTTCGCCCTTTTTCCTCATATGACC
ATCCGTGAGAACGTCTGTTTCGGCTTGACCGAATTGCCGAAGAAGAAGGCATTGCTGCAGGCCGATGCCGCGC
TGGAGCGGGTCGGCCTTCTGCATTATGCCGAGCGGTATCCGCATGTGCTCTCCGGCGGCGAGCAGCAGCGCGT
GGCGCTGGCCCGTGCACTTGCGCCGCGGCCGGCGGTGCTGCTGATGGACGAGCCTTTTTCCGGCCTCGATTCC
CGCCTGAAGGATTCTATCCGCGCCGACACGCTGGCCATACTGCGCGAGACGCGCGCGACCGCCATCGTCGTAA
CGCACGACGCCGAGGAGGCGATGCGTATGGCTGACCGGATCGCCCTCCTGAAGGACGGCCGCCTGGTGCAGGT
CGGCACGGCGGACGAGCTCTATCGCCGGCCCTGCGATCTCTTCACTGCCGGCTTCTTCTCCGAAATAAACGTC
TTCGATGCGCGCGTGCGGGACGGTCATGTCGAAACGCCGCTGGGGGTTGTGGCAACCGGGCAATATACGGAGG
GGCAGCCACTGAGCGTAGCCGTGCGTCTTTCCGGCATCCGGCTCGACGAGACCGGTGGAGAAATTCCGGCGCG
CATCGTGTCCCGGCGCTTCCTGGGCGTCGTGGAGCTTTTGGAACTCGCCGTGCCGCAGTCCGAACGCACGGTT
CGCGCCCGCATCCGCGCCGATTTGCTGCCGCAAGGATTGCGTGACGTCACGCTTTCCGTTAACGAGCGCGACA
TATTGGTGTTTGAAAAAGACGCATCAACACCTTAGATGCCGAAGCGCCAAGACATCAAATCGATCCTCATCAT
CGGCGCGGGGCCGATCGTCATCGGTCAGGCATGCGAATTCGACTACTCCGGCACCCAGGCGTGCAAGGCGTTG
AAGGAGGAAGGCTACCGCGTCATCCTGGTCAACTCCAACCCGGCGACGATCATGACCGATCCGGGTCTGGCGG
ACGCGACCTATGTCGAACCGATCACGCCAGAGGTCGTCGCCAAGATCATCGCCAAGGAACGCCCCGACGCGCT
GCTGCCGACCATGGGCGGACAGACGGCGCTCAACACGGCCCTGTCGCTGCGCCGCATGGGCGTGCTCGACCGC
TACAATGTCGAGATGATCGGTGCGAAGCCCGAGGCGATCGACAAGGCGGAAGACCGCGCCCTGTTCCGCGAGG
CCATGGCGCATATCGGCCTCGAGACGCCCAAGTCGCGTCTGGCGAACGCCACGGACATCAAGGACCACGACCG
CAAGTCGCACGAGGCGGAGCGCTCAGCGCTCAAGGCGAAGCTTTCCGGTGACGAACTCGACAAGGCGCTGGAC
GAACTCGAAAACCAGTGGAACCTCGGCGAAGGCGATCGCAAGCAGCGCTATGTGAACCACGCCATGGCGATCG
CCGCACAGGCGCTCGACGATGTGGGCCTCCCCGCGATCATCCGGCCGTCCTTCACGCTCGGCGGCACCGGGGG
CGGCATCGCCTATAACCGTTCGGAGTTCTTCGAGATCGTCGGCAGCGGTCTCGACGCGTCGCCGACGACGGAA
GTCCTGATCGAGGAATCGGTCCTCGGCTGGAAGGAATATGAAATGGAGGTCGTCCGCGACAAGGCGGACAACT
GCATCATCATCTGCTCGATCGAGAACATCGATCCGATGGGCGTCCACACGGGCGATTCGATCACCGTTGCGCC
GGCACTGACCTTGACCGACAAGGAATATCAGATCATGCGCAACGCCTCGATCGCGGTGCTGCGCGAGATCGGC
GTCGAAACGGGCGGCTCGAACGTCCAGTTCGCCGTCAATCCCGAGAATGGCCGCCTCGTCGTCATCGAGATGA
ACCCGCGCGTCTCGCGCTCCTCCGCACTCGCATCGAAGGCCACCGGCTTCCCGATTGCCAAGATCGCCGCCAA
GCTCGCAGTCGGCTATACGCTCGACGAACTCGAAAACGATATCACCGGCGGCGCGACGCCTGCATCCTTCGAG
CCTTCGATCGACTACGTCGTCACCAAGATCCCGCGCTTCGCCTTCGAGAAATTCCCGGGAGCCGAGCCGACGC
TCACGACCGCAATGAAGTCGGTGGGCGAAGTCATGGCGATCGGCCGGACCTTCGCCGAATCGCTGCAGAAGGC
GCTGCGCGGTCTCGAGACCGGACTGACCGGACTTGACGAGATCGAGGTGCCTGATTTCGACGACAATGGCGAC
GGCCGCAATGCCATCCGCGCCGCGCTCGGCACGCCGACGCCGGACCGCCTGCGCATGGTCGCACAGGCGCTGC
GTCTCGGCATGAGCGAAGCCGAAGTGCATGAAGCCTGCAAGATCGACCCCTGGTTCATTGCCCAGTTCAAGGC
GATCGTCGACATGGAGGCGCGCATCCGCGAGCACGGCCTGCCCGCCGACGCCGAGAACCTGCGCATGCTGAAG
GCAATGGGCTTCTCCGACGCCCGGCTTGCGACGCTTACCGGCAAGCGTCCGAAAGAGGTGGCCGAGCTCCGCA
ACGCACTGAACGTGCGCCCCGTCTACAAGCGCATCGACACCTGCGCGGCCGAGTTCGCATCGCCGACCGCTTA
CATGTATTCGACCTATGAGACGCCTTTCGTCGGTGCCGCCCGGTCGGAGGCGCAGGTTTCCGACCGGAAAAAG
GTCGTCATCCTCGGCGGCGGCCCGAACCGGATCGGCCAGGGCATCGAATTCGACTACTGCTGCTGCCATGCCG
CCTTCGCACTGAAGGACGCCGGCTACGAAGCGATCATGATCAACTGCAACCCGGAGACGGTTTCGACCGACTA
CGACACCTCCGACCGGCTCTATTTCGAGCCCCTGACGGCAGAGGATGTGATCGAGATCATGCGTGCCGAACAG
GAAAACGGCACGTTGCATGGCGTCATTGTCCAGTTCGGCGGCCAGACTCCGCTGAAGCTTGCCGAAGCGCTGG
AAAAGAACGGCATCCCGATCCTCGGCACCGCGCCGGACGCGATCGATCTCGCCGAGGACCGTGATCGCTTCCA
GAAGCTCCTGATGAAGCTCGATCTCAACCAGCCGAACAACGGCATTGCCTACTCCGTCGAGCAGGCCCGCCTC
GTTGCCGGCGAAATCGGCTTCCCGCTGGTCGTGCGCCCGTCCTATGTTCTCGGCGGCCGCGCCATGCAGATCA
TCCATTCGGAAAGCATGCTGCAGAGCTATTTGCTCGACACCGTTCCGGGGCTGGTTCCTGAAGATATCAAGCA
GCGCTATCCGAACGACAAGACCGGCCAGATCAACACCTTGCTCGGCAAGAACCCGCTCCTCTTCGACAGCTAC
CTGACGAATGCGATCGAAGTGGACGTCGATTGCCTTTGTGACGGCAAGGATGTCTTCGTTTCGGGCATCATGG
AGCATATCGAGGAGGCCGGCATCCACTCGGGCGACTCCGCCTGCTCGCTGCCGGTGCATTCGCTCGGCACGGA
CATGGTCGACGAACTGGAGCGTCAGACGGGGGCGCTCGCCAGAGCGCTGAACGTCGGCGGCCTGATGAACGTG
CAGTTCGCCATCAAGGACGGCACCATCTATGTGCTCGAAGTAAACCCGCGCGCCTCACGCACCGTTCCCTTCG
TCGCCAAGACGATCGGCGCGCCGATCGCCAAGATCGCCGCCCGCGTCATGGCCGGCGAAATGCTCGACGAGGC
GATCGCCGCCTATGGCAAGAAGCCGAATCCGCGCAACCTGAAACATATCGCCGTCAAGGAGGCCGTCTTCCCC
TTCGCCCGCTTCCCGGGCGTCGATACGCTGCTCGGCCCGGAAATGCGTTCGACCGGCGAGGTCATCGGGCTCG
ACACCGATTATGCGCTGGCCTTTGCCAAGTCGCAGCTCGGCGCCGGCGTCGACCTGCCGCGCGACGGAACGGT
CTTCGTCTCGGTTCGCGACGAGGACAAGGAACGGGTGCTGCCCGCGATCCGCATTCTCTCCGACATCGGCTTC
AAGGTCATGGCGACCGGCGGCACGGCGCGCTTCCTCGGCGAGCAGGGCATCGTTGCAACCAAGATCAACAAGG
TCCTGGAAGGACGGCCACATGTCGAGGACGCCATCCGCAACCGGCAGGTCCAGCTCGTGATCAACACGACCGA
CGGCAACAAGGCGATCTCCGACTCGAAATCGCTTCGCCGCGCGACGCTGATGCAGAAGGTGCCCTATTACACC

ACGATGGCCGGCGCCGAGGCCGCGGCACTCGCCATCAAGGCACTGAAGGCGGGCAACCTCGAAGTGCGTCCGC
TGCAGAGCTACTTCGAAACCTGATTGAACGCGAATCTGATCCGGCATGCTGTCCATCTCGCGGCGATCCTGGG
GCTCTATCTCTCCGGCGCGATGCTGATTCCGGCCCTGGTCGATCTCTATTATGGTCACCCGGACTGGCAGGTC
TTCGCCGTGACCGCCTTCATGTCGGGCGCAATCTCGTCGACGACGTTCATGGCGACCCGCGCAGGCCCCCCGC
CCTTCTCGAAGAAATTCGGCTTCCTGGTTGTGAATGTCCTGTGGATCGTGTTTTCGATCGTCGGCGCCATTCC
ACTCTGGCTGTCCTCGGTAAAGCTGGATTTTGCCCAGGCCCTGTTCGAGTCCGTCTCGGCGGTAACGACGACC
GGCTCGACCGTAATCGTCGGGCTCGATGACGCGCCGCCCGGCCTCTTGCTCTGGCGGTCGCTCCTGAGCTGGT
TCGGCGGCATCGGCATCGTCGTTCTCGGCCTGTTCATCATCCCCTATCTGCGTGTCGGCGGAATGTCCTTCTT
CAAGATGGAATCCTCCGACACCAGCGACAAACCCTTTGCCCGGCTCGCGAGCTTCGGCCGCGCCTTCCTCGTC
GTGTACGTGTCGATCACTCTGCTGTGCGCGATCAGCTATGCCATGCTCGGGATGAACAGGTTCGACGCGATCA
ACCACGCGATGACGACCGTCGCGACGGGCGGCTTTTCTACGCACGACGCGTCATTCGGCTACTTTTCCAGCAT
TCCCCTGCTCTGGGCCAGCACGTTCTTCATGACGGTCTGCAGCCTGCCCTTCTCCATCCTGATCGTGCTCGTC
GTGCGCGGGCGCCTGGACGCCCTTCGCGATCCTCAGATCATCGTATTTCTGGGCTATCTCACCGCATTCTCGG
TTGCGGTCGCGGCCTATCACCGGCTCGCCAACGGCGTCGAGTTCCACCTCGCGCTTACCTATGCCTTCTTCAA
CATGACGTCGATATTGTCGACCACGGGCTATGCGAGCGAAGATTACAGCCTTTGGGGACCGTTCGTGGTCATG
GTCGCGTTCATATGCACGTTCATCGGCGGTTGTTCAGGCTCGACAGCGGGCGGCATGAAGGCCTATCGGTTCG
TCGTGCTCTTCAACGCCATCCGGTCTGCCCTGAACAAGCTCGTCTACCCGAATGCGATCTACGCGGTCCGGTA
CGGGAAGAATACGGTCGACGCGGACGTGCAGCGCGCCATTCTGCTGTTCTTCATCACCTATATCCTGCTCTGG
GTCTTCGGCAGTCTGGTCATGGGTGCCCTCGGCTATGATCTCGTCACGGCCGTGTCGGCCGTCATCACGTGTC
TCTCCAATGTCGGCCCCGGCCTCGGCAGCCTTATCGGCCCTGCGGGCAATTTCTCGACACTCCAGGATCCCGA
GCTCTATCTGCTGTCGCTGATGATGCTGCTCGGGCGTCTGGAGGTCCTGACCGTCCTCGTCGTGCTCACTCCG
ATCTTCTGGAAGCAGTAAATGCCGAGATACCTGCTCCTGCTGGCGGCACTCACGAACGCCACCGTCATCCTCG
CCGACACGCAGGCAGAGGCGGGTCCGCTTGCCGACGCTGCGGCGAAGGCCGAACAGCAGGCGAGCTCCGGCGA
TGCCGCCGGCGCCCAGGAAACCTTGCGCAAGGCCATGAGCGACTTTTCGCAGACCCTTCCCTTCGCCATCGGC
AAGGCGGTCTTCGTCACAGCAGCACCGGCCGGCTTTGCAATGTACGAGCCCCGGCCCGATCCGGTATTCGAAG
CCGGCGAAACGCTCATATCCTATGTCGAGCCCGTGGGACTTACCTGGAAGGAGGCAAGCGACGAGGGTAAGCT
CGAGACACGCTTCACGGTCGACTTCGACATCCTGAATCCGAAGGGAGAGGTCCTCGCCAGCCAGAAGGCGTTC
GGCGATTTCACCTTCAAGGGCTATCAGCGCAACCAGGAAATCTATGCGACGCTGAGGATCGACGTCGCCGGGG
CACCATCAGGGAATTATGTGCTGCGCTACCGCTTCAACGACACCAACAGCGGCAGGAGCGCAAGCGTCGATCA
ACCATTCGAGATCTCCGGCCCTTGAATGCGCATTGAACTCGAAAAGCGCGCGAAGGCCTCGACGCTGTTTTCC
ATCCTTTCGCCGTTCATCGCATTTGCGCTGACCATCGTCTTCGGCGGCGTGATGTTCGCACTTCTCGGCAAGA
ATCCGGTAACCGCGCTCTACAGCTTTTTCGTGGAGCCGCTGAGCGAGGTCTGGTCGCTGCACGAACTCGCGAT
CAAGGCGGCGCCGCTGATCCTGATCGGCGTCGGGCTCTCCGTCTGCTTCCGCTCCAACAACTGGAACATCGGC
GCAGAGGGTCAGTTCATCATGGGCGCAATCGCCGGCTCGGCCCTGCCGGTACTCTTCTACGATTGGCAGTCAC
CGTTGATATTGCCGCTGATGATGCTTCTCGGCATGCTTGGCGGAGCGCTCTTCGCGGCGATACCGGCCTTCCT
GAAGGCGCATATGAATACGAACGAGATATTGACGAGCCTGATGCTGGTCTATGTGGCCCAGCTCTTTCTCGAC
TGGCTCGTACGTGGCCCTTGGCGCAACCCGCAGGGCATGAACTTCCCGGAAACGCGAACGTTCGGCGCGGATG
CGATCCTTCCCGAAATGCTCGCCTCGGGGCGTACGCATTGGGGTTTTGCCTTCGCGATCATCGCGGCGGTGCT
GGTGTGGTTCATGATGCGCTATACGTTGAAGGGCTTCGAAATCACCGTTCTCGGCCAGTCCGAGCGGGCAGGG
CGCTTTGCCGGCTTTTCCTCGCGCAGGATGATCTGGTTCTCGATGCTGTTTTCCGGCGCGCTGGCGGGGCTCG
CCGGTATTTCGGAAGTGAGCGGCGCCATGCAGCAGCTGCGTCCGGTGATCTCGCCCGGTTACGGATTCACGGC
TATCATCGTCGCGTTCCTCGGCCGGCTCAATCCGCTCGGAATCGTGGCCGCCGGTCTCGTCCTGGCGTTGACT
TATCTCGGCGGCGAGGCCGCGCAGCTTTCGATCGGCGTATCCGAAAAGGTGACGCGCGTCTTCCAGGGGCTGC
TGCTGTTCTTCGTGCTGTCTTGCGACGCGCTCATTCACTACCGCATCCGGCTGGTGTGGGACCGCTTCGCAGC
GGTCAGGACGGAGGAGGCGCACTGAATGCGGAGTCTCCGCCAACCTGGTGCACCGTTCACGCCGAGGGTAATC
TCTCTCGGGTGCCACGCCGAGAAGGCGCGATTGACCTTCAAACCGGGCCGGAGCGTGCTCGAAGCCGTCCATG
AGGCGCTCGCGGAAACAGGCTGCGACAGCGCGATGATAGAGGTCAGGGGCGGTGCGTTTGCGCCGCTTGCCTA
TGTCACACCGACTCTCTCGGATGACGGCATTCATGCCGCCTGGTACAGCGACATACATGCGCCTGATGGCCGC
GCACCCATCGAAGATCTGGTCATGACGTTCGGCCGGCGCGACGGGGAGCCGTTCCTGCACGGCCATGGTGTCT
GGCGGCACGAGGACGGGTTGCGCGCAGCCGGGCACGTAATGCCGAAGGACTCGCAATTCGCCGAACTGGTGGA
GGCGGAAGCCTGGCTCATGTCGGGCGCCATCATGGACCAACTGGAGGACAGCGAGACCCGGTTCCGTCTGTTC
ACGCCTGTGCCTCATACCGCCGCACCGCCCATCACGACGCGGCGCGCCGTTCTCTGCCGGTTGAAACCGAACG
AACCGATCCATCCGGCGATCGAGCGCACCGCAGCCGAGCACGGCATCGAGCGGGCGACTTTGCACGGCATCGG
CAGTCTCGTCGGCTGCACTTTCGCCGACGGCCAGGTCATGGAATCGGCCGCTTCGGAATTGCTTATTCGCAAG
GGGACGCTTTCACCCGATCGAAGCGGACAGGCTAAAGCGAAACTCGATATCGCGATCGTCGATACGGAATGCA
CCATATTCGAGGGTGAGATAGCCTACGGCGTTGATGCCGTTTGCATAACGTTCGAGGTGCTGATCGTCGAAGA
GTGAATGGCCTGGCAACCGGCGGAAAACAGATACGAGAAGATGAAGTACAATCGCTGCGGCAGGAGCGGCTTG
AAGCTGCCGGCGATTTCGCTGGGTCTTTGGCACAATTTCGGTGGCGACACGCCGCATGAGCGCAAGGTGGACA

TGTGCCGCACGGCCTTCGATCTCGGCATCACCCATTTCGATCTGGCCAATAATTACGGTCCGCCGCCCGGCTC
GGCCGAAATCGCCTTCGGCGAGATCATGCGCAGCGATTTCGCCGCCCTGCGCGACGAACTCATCATCTCGTCC
AAGGCGGGGTATGACATGTGGCCCGGCCCCTATGGCGAGTGGGGCAGCCGGAAATATCTTGTCGCCTCTTGCG
ATCAGAGCCTCAAACGCATGGGGCTCGACTATGTCGACATCTTCTATTCCCATCGCTTCGACCCGGAAACGCC
CCTCGAAGAGACTTGCGGAGCGCTCGATCACATCGTCCGCTCGGGGCGTGCGCTCTATGTCGGCATCTCCTCG
TATAACTCCCAGCGTACCCGCGAGGCGGCGGTAATCCTGAAGGAGATGGGCACGCCCTGTCTCATCCACCAGC
CGAGCTATTCGATGCTCAACCGTTGGGTCGAGGATGACGGTCTCGTCGACACGCTGGAGGAACTCGGCGTCGG
CTCGATCGTCTTTTCGCCGCTCGCCCAGGGCATGCTGACGACGAAGTACCTGAACGGCATTCCCGAGGACAGC
CGCGCCGCGCAGAACCACTTCCTCAAGAAGGACTTCATCCGTCCGGGGATCATCGACAATATCCGCAAGCTGA
ACGCCATCGCCGAACGGCGCGGCCAGACGCTCGCCCAGATGGCACTCGCCTGGGTGCTGCGCGGCGGCCGCAT
CACCTCGGCGCTGATCGGCGCCAGCCGTTCCGCACAGATCGTCGACTGCGTCAAGGCGCTGGAAAACGACACT
TTCACACCAGAGGAACTGAGCGAGATCGATCTTTACGCCCGCGAGGCCGACGTCAATCTCTGGGCCAAATCCG
CCGAGCTCTGAATGAGCAAGGCTTATAAGGACCACCTCGGCCGTCCGATCGTTGCCGTCACCGGCATGGGCGT
CATCACCTCGCTCGGGCAGGGGCTCGACGACAACTGGGCGGCGCTCACCGGGGGCGTATCGGGCATTCACAAG
ATCACCCGGTTCCCGACCGATGCTCTTTCGACCCGCATCAGCGGCACGGTCGACTTCATCGAGATTCCTGCGG
AGAATGCCGTCGAGCGTTCCTATGCGATGGCGCGCGAAACGACGCTGGAGGCCCTGGCTCAGGCCGGCCTTTC
CGGCGATTTCAACGGCCCGCTCTTCCTGGCGGCCCCGCCGATCGAGCCCGAATGGAATGCCCGTTTCGAGCTT
GCCGACCGCTCGCCGCCGTCGAGCCAGCCGGGCGACGCCTATAATCGGTTCCTCGCGGCAATGCGGCAGAAGG
CGGATCCGACCTTCCACGAGGCCGTGCTATTCGGCTCGATCTCGGAGCGTCTTGCGGACCGCTTCGGTACGCG
CGGCCTTCCGGTGACGCTTTCGACGGCTTGCGCATCCGGGGCGACCGCCATACAGCTCGGCGTCGAAGCCATC
CGCCAGGGCAGGACCGACAGGGCGCTCACGGTTGCGACGGACGGCTCGGTCAGTGCCGAGGCCCTCATCCGCT
TCTCGCTGCTCTCGGCGCTCTCCACACAGAACGAGCCGCCGGAAAAGGCTTCGAAGCCCTTCACCAAGGATCG
CGACGGTTTCGTGATTGCGGAAGGCGCCGCGACGCTGGTGCTCGAATCGCTGGAAGCGGCGGTCGCTCGCGGC
GCCCGCATCTACGGCATTCTCAAGGGCTGCGGCGAAAAGGCCGACCTCTTCCACCGCACCCGTTCCTCCCCCG
ATGGCGGACCGGCGATCGCGACGATCCGCGCGGCACTTGCGGATGCGGGCATCGACGAAGCCGGGATCGGCTA
CATCAATGCCCATGGCACCTCCACGCCCGAAAACGACAAGATGGAGTATCTGTCGATGTCCGCGGTCTTCGGC
GAGCGGTTGCCGTCGATCCCGGTCTCATCCAACAAGTCGATGATCGGTCACACGCTGACGGCAGCCGGTGCGG
TCGAAGCGGTCTTCTCGCTCCAGACGATGCTCAGCGGCACGCTGCCGCCGACGATCAACTACCAGAACCCCGA
CCCGGCGATCGTTCTCGATGTCGTGCCGAACGTGAAGCGCAGCCAGCAGGTGACTGCAGTGCTGTCGAACTCC
TTCGGCTTCGGCGGCCAGAACGCCAGCCTTGTCATGACCGCCGAGCCGGCCTGAATGAGGCGGCTGGCAGCGA
TCATGGACGCGGACGTCGTCGGCTACAGCCGGCTGATGGGACTGGATGAAGCGGGCACGTACCGGGCGGTCAA
ACGGTGCCGTGATGCCTTCATTCTGCCTTTGGTCGCGGCCCATGGCGGAAGGATAGTCAAGCAGGCGGGAGAC
GGCACGCTTGCGGAATTCGCAAGCGTTCTCGACGCGGTCGCCTGCGCGGTCGCGATCCAGCGAACGATGCATG
ACCACGCCGGAGGCGCCGAAGCGGAGCGTCTGGAATTGCGCATCGGCGTCCATCTCGGTGATATCGTCGCTGA
CGATGGCGATATCCATGGCGAAGGCATTGCCGTTGCCGAGCACCTGCAGGAGATGGCGCCGCCCGGGGGCGTC
TGCGTGTCGCAGCAGGTCTATGACCAGATCGGCTTGAAGGTGGATTTCCCCTTGACCGAAATCGGCTGCAGGA
CATTTGCCGATCTCCCCGGTCCGCTGCGCGCCTGGCGCTGGCAGCCGGGCGGCGCGGCAGGCGAAGAGCCGCC
GGCGCCGGCGCAAACCGATCCCCTGCCCGGTAAGAAGCGCCCCTCGATCGCCGTCCTGCCCTTCGTCAATCTT
TCGAGCGTCGAAGAACAGGAACATTTCTCCGACGGCTTTACCGAGGAACTGATCGCCACGCTCGCCCGATGCC
GGTGGTTGCGCGTCGTCGCCCGCAACTCCTCCTTCACCTTCAAAGGGAAGGCAGCCGACGTAAGGAGGGTGGC
GGAAGACCTGGGTGTCAAATACGTGATCGAGGGCAGCATACGTCGCGCGGCGAACCGCATCCGCATCACGGCG
CAATTGCTGAGCGGCGAGACCGGCATGCTGCTCTGGGCAGAACGCTACGACCGCATGCTGGACGATGTTTTCG
TGCTCCAGGACGAGATCGCGGGGCAGATCACCGGCACCGTGGAGCCGGAACTCGGCTTCATCGAATTCGCTGC
GCTCCGCGGCCACACCGCGACGGACATGGATGCCTGGAACATTTATCTGAAGGGGCTATGGCACCTTTACAAG
TTCGATCTGGAGAATCTGAGGATATCCAAAGCGCTCTTCGAGCGGGCGATCGATCTCGAGCCCGCCTTTGCCC
AGGCCCATGCGCGGCTTGCCTATGTCCATATACAGCTCGGCTGGTATGGCCCCCTGGACGAGCGGGCCGAGCG
GATCGCCGACGCGACGGCGCTCGCGGAACGCGCAATCGCGCTCGACGACCGTGAGCCGGCTGCCCATCTTGCG
CTCGGCCGGGCACGGGCGCTCGGCGGCCAGCCGCAGCGCGGCATAGACCACCTGCGCAATGCGCTGAGGCTCG
ATGCAAGCTTCGCCCAGGGCCACTTCGCCCTCGGGCAAGCGCTTTGCTATGTCTGCCGCCCGGAGGAAGGCAT
TGCCGCGATCAACGAAGCCTTCCGGCTGAGCCCCCGCGATCCGCATTTGTGGACGTTCTACAACATGGTGGCC
ATCGCCCACTATCAGTCGGGTCGCTTCGCGCAAGCCGCCGAAGCGGCCCGCGCCTCCCTGCGCCAGGAGAACG
CCACCTTCTGGCCGGCAATGGTCCTGGCAGCCTCCCTCGGTGCCACGGAGCGGTCCGACGAAGCCGGTTCAGC
CGTGGCGAACCTTCTTCGCCGGCGGCCGGACATGACCGTGAAAACGGCCCGCGCCGAATTCTACTTCGGCAGC
GTGCCGGTCATGCCGGAAGATTTCATCGATCGTTTCGTCCGCGATCTGCACCGCGCCGGCCTGCCTGACTGAA
TGGCGCAATTGATCCTCAACCACCTGACCAAGGACTTCGGCGGCAAAGGTGCAGACCGGGTAAGGCCGGCTGT
TTCGGACGTGTCGCTGGCGCTGCGCAAGCAGGGATTTCTCGCACTGCTCGGGCCTTCCGGCTGCGGCAAGACG
ACGGTGCTCCGGATGATCGCCGGCTTCGAGCAGCCGACGGACGGCTCGATCGAGCTCGGCGAGCGCAGGCTTT
CGGATGCAACCCGCGTGCTGCCGCCGGAAAAGCGCAACATGGCGATGGTGTTTCAGTCCTATGCGCTCTGGCC

GCATATGACGGTGGCGGAGAATGTCGGCTACCCGTTGAAGGTCCGCGGCATTTCCGGGGAAGCCTGGCGCAAG
CGGGTAGGCGACGCGCTTTCGCTGGTGAAGCTTACGGATTTCGCCGATCGGCGGCCGGCTGTACTTTCCGGCG
GTCAGCGGCAGCGTGTGGCGCTGGCGCGCTGCCTGGTCACGGAACCGGACGTGGTGCTGCTCGATGAGCCCCT
CGCCAATCTCGATCAGCATTTGAGGAAGTCGATGGAGGAGACCTTCCGCGCCTTTCACGAGCGCTCCGGCGCG
ACGATGATCTATGTGACTCACGACCAGGCCGAGGCGATGGCGCTTGCGACCGATGTCGCGGTGATGTCGGAAG
GCAAGCTTCTTCAGGTGGCGCCGCCGGCGGAAATCTATGCGCGGCCGGAGGGGCGGGTCGTCGGGGGCTTGAT
CGGTCGCGGCGCGATCGTGCGGTTGAATTTGCCGGAGGGAGGGGCGCGTGCGCTCGATTGGCCGCTCCTCGGC
GCCGCGCTTGGTGAGATGCCCGCGGGTGAGGAGCATGCAGGCGGGGGGCCGCTTTCCGACGTGCTCGTCCGTC
CGGAGCAGGTTCATCGGGATAGCGAAGGCATTCCGATGCGGGTTCTCTCCTCGGTCTTCGCGGGCGAACGCTT
CGCTCTCACACTGGCGCTGCCGGACGGGCAGAGCGTCAAAGCCTATAGCAGCGTGCCGCTCTGCGAGGGCGCT
CGCGTTCCTTCGTCGTGAGTGATCATCCTCGAGACCGGGCGGCTCAGGCTCCGCGCCTGGATCGAGACCGATC
GCGAACTCTTTGCCGAGATCAACAGCGATCCCGCGGTCATGGAGTTCTTCCCGTTCCGCCGCACCCGCGCGGA
GGCCGACGCGCTTTTCGACCACAACAACCGCAACATCCGCGAAACCGGCTTCGGCTTCTTCGCGCTCGCGTTC
CGTGAGAACGACGCGCCGATCGGCTTCTGCGGACTTGCACGCACCGACCTCGAACCGCACCTGCCCGACGGCA
CCGTCGAGATCGGCTGGCGCCTGGCTCTCCGCCATTGGGGAAAGGGCTACGTGACGGAGGCAGCGCGGGCGCT
TCTCGATTATGGATTCAGCGAGAGGAAGCTCGGGGAGATCGTTGCCATCGCCGTTCCGGCCAACACTCGCTCC
ACCGCCGTGATGACGCGGATCGGCATGCGTCCTGATTCCAAGCGCGACTTCGATCATTCCGGCGTCCCGGACA
CGCATCCCCATCTCAGACGCCACGTGCTTTACGCAACAACCGCGGAAGAATGGCGGCGGGATGGCTCGGCCGC
GAAGCGGTAGATGAATGAGATGAAGTGCAGGATCCGCGGGGCGCATCTGAAGACTCTTTTGGCGAGCGGAGTT
GCGCTCGCGCCGCTGATGATGAGCGGAATCGCGCTGGCGCAGGAGGGAAACGCCACGCAGCTCGAGCGAATCG
TCGTCGAGGGCGGCAATGCGGCCGGCGCCAGCGCGACCGGACCGGTGGACGGCTACGTCGCAAAGGCGACCGC
GACCGGGTCGAAGACCGCCATGCCGCTCAACGAAATCCCCCAGTCGGTCTCCGTCGTCGGGCGTGAGGAACTG
GACGACCGCGCCGTGGTCAACAAGGTCGATGAAGCATTGCGCTATACGCCGGGCGTCCTGTCGGCACCATTCG
GCACCGACCCGGATACGGACTGGTTCTACATCCGCGGCTTCGATGCGGCACAGACGGGCCTCTTCCTCGACGG
ACTGCCGCTCTTCAGCTTCGGCTTCGGCAATTTCCAGGTCGACCCCTTCATGCTGGAGCGGGTCGAGGTGCTG
AAAGGGCCGGCCTCCGTGCTTTATGGCGGGTCCAATCCGGGCGGCATCATCAACCTGATCAGCAAGCGGCCGC
TTGACGAGCCGCTCTACTACACCGAGGTCGGCATCAACAGCAACGGCAATGCCTTCACCGGCTTCGACGTGAA
CGACAAGCTGAACGATGACGGAACGGTTCGCTACCGCCTCACCGGCAAGGTCGCCGGCGGCGACAACTATTCG
GACTATTCCGAAGACCTGCGCGGCTTCATCCTTCCTCAGGTAACCTATGCGCCTGATGACGCAACCAGCCTGA
CCGTCTTCGGCCTGCTCCAGGGCCTCGATCAGGTCCATGTCGGCAACGGCTTCCTGCCCTATGTCGGAACCGT
CGAAGACGCGCCCTTCGGCAAGATCGACCGGGATGCCTATTACAGCGAGCCTGATATCGACGAGGGCAGCTAT
ACGCAGCAGATGCTCGGCTACGAGTTCAAGCACGACTTCGACAATGGCTGGACCTTCACCCAGAACGCGCGCT
ACGCCAATCTTCACAAACACGAGAAGTACCCGTACACCTATGGCTATGTCGGCGGCGCACCTACCGGCCCGGA
CTACCTCCTGAACCGCATCGGCTTCGAGGCGACGTCGAAAGTCGACAGCTTCTCGATCGACAACCGGACGGAA
ACGGATTTCGATCTCGGCGCCACGACCCACACCTTCCTCGCCGGCCTCGATTACAAATACTACCGGCTCGACC
ATATTCAGGCATGCTGCGGCGCGACCCCGATCAGCGCAACCAATCCGGTTTACGGCACACCTCAGGGTGCAAA
TTTCGTATATCTCGATCAGATCGTCACGCAGCAGCAGATCGGCCTCTACGCACAGGACCAGATTCGTTTTGGC
GACGGCTGGCTGGTTACGCTCAACGGCCGCTACGACTATGTGGACACCAAATCCGACGCCGCGATCGGCACGA
GCTACGAATCGAATGACGGTGCCTTCAGTGGCCGTGCCGGCCTCGCTTACGAGTTCGACAACGGCCTCACGCC
CTATGTAAGCGCTGCGACTTTCTTCAATCCGCTGGTCGGCACGGGCACGAGCGATCCATCGGATCCGACGAAT
CCGGCAAAGATGGTCGCGCTCGAGCCGGAGGAAGGCTATCAATACGAGGCGGGCGTCAAATACGAGCCCTCCT
TTATCGACGGGCTGTTGACTGCATCAGTCTTCCAGATAACCAAGCAGAACGTCTCAATTGCCGTTCCCGGCTT
CTTCGTCAACTCGCAGCTTGGCGAGGTCCGTTCGAGGGGCGTCGAACTCGAGGGCAAGATCAATCTCAATACC
AACTGGAAGATCATAAGCGCCTTCTCCTATACCGATCTCGAGGTCACGGAAGATCTCAACGCCAGTCTCATCG
GCAATACACCGGTTCTCATTCCCGAAACACAGGCGTCGCTGTGGCTCGACTACACGGTTGCGAACGGCACGTT
CGAGGGCGTGAGCCTCGGCGCCGGCGTGCGCTACCAGGGGGAATCCTGGGCCGACGCGGAGAACACGAAGAAA
GTCCCCGCTGCGACACTCGTGGACGCCGCTATCCGCTACGAGAAGAACGACTGGACGGCGTCGCTCAACGTCG
CCAACCTGTTCGACAAGGAATATGTTGCGGGTTGCCAGGGTCTCCAGACCTGCGGCTATGGCGAAAGCCGGAC
CTTCACGCTGAAGCTCAGCAAGAAGTGGTAAATGGCTTCAGTAACGCACTCCAGCCCAAGGGGCAGCAGGTTC
AGGATCAGCAAGAGGGCCTTGCCCTATGTGCTCTCCCTGCCGGCGCTTCTCGTGTGCATCGGTATCCTGATCC
CGTTCTTCACGGCGGTGATCTATTCCTTCCAGCGCTACCGGCTCAGCCAGCCCTGGGCACGCCAGTTCAACTG
GGGTGAGAACTATCTGAACTTCTTCACCGATCCGGGTTTCTGGAACACGCTGAAGATCTCGCTGCTCTATGCC
GGCGTCACCGTGACCCTGGAGCTGCTGCTCGGTCTCGGCATTGCGCTGCTCCTGCAGCGCCGCTCGACGGTCA
ACAACTTCATCTCGATCATGCTGCTCCTGCCGCTGATGACGGCGCCGGCGCTTGCGGCGCTGATGTGGAAGCT
GATGACCAATCCCGGCTTCGGGATCCTGAGCTATCTCGCAAGTCTCGTGGGCCTTGAGAACTTCCGCTGGGCA
TCGTCGCCGGACACGGCGCTGCTGACCGTCGTGCTCGTGGACATCTGGGTCTATACGCCCTTCATCATGATCC
TGCTGCTCGCGGGCCTGCGCTCGCTGCCGACGCAGCCATTCGAAGCGGCCGCGCTCGACGGCGTGCCGCGCAG
CTTCGTCTTTTTCCGCATCACCCTGCCGATGCTCACGCCCTATATCCTGACGGCGACGCTCTTTCGCCTGCTC

GATTCCATCCAGCAGTTCGACATCATCTACGCGATGACGCAGGGCGGGCCCGGAGACACGCTGACGGTCTTCC
AGGTCGAGGCTTACCTCAACTTCTTCCAGTCGACCAATGTGGGGCGTTCCGCGGCGCTGCTGATCATCCTGTG
GGCGATCACCTACGCGCTGTCGAACATCTTTATCAAGAACTGGCTCCGGCTGCGCGAACGCGCCCGCGGCGAA
GCGTAAATGACGCTGGAGGAGCTCTACCAGGCCGGGCGCAAGGTCGTCACCGGCAGCCTGACATTCACCGCGG
AGGACATCGTCCGCTTCGCCCGCGATTTCGACCCGCAACCCTTCCACGTCGATGAGGAGCAGGCGAGACAGTC
GCTTTTCGGCGGCCTTTGCGCCTCCGGCTGGCATACCAGCGCCGGCTGGATGCAGTGCTTCCTGCGGTTCTGG
AAGGAGGAGATCCGCCGCCTTGCAGCCGAGGGATTGCACGCGCCGAAGCTCGGCCCCTCGCCCGGGTTCAAGG
AACTCAGGTGGTTGAAGCCGGTCTATGCCGGCGACACGATCACCTATGCGGTCACTCTACTGGAGGCCCGGAT
CGTCGCCTCGCGACCCGGCTGGCGGATCAACACGATCCTCTGCGAGGGCGAGAACCAGCACGGCGAGCCGGTC
ATCCGCTTCGAGAGCAAGGTCATCGAATTCGCTTGAATGTTCGATCCGATCGTTGCGTTCTTCCAGCGTGTAT
TCACCGCGATCGGCCGTGGCATCGGATTGGCGGTGGCGTGGCTTCTCTGGCCGTTCGTCGCGCTCGGCAACTG
GTATCGGGGACGAAGCTGGATCATCAAGGGGCCGATCGGCCTGATCCTCCTCTGCCTCGTGCTCTTCTACGCC
TACTTTGTCTGGCAGACGCAAGCCTGGACGAATTTCGACCCGGACTATGTCGACCGATACAAGCTCGCCGACC
GCAGGACGCCGGCCGGTTCACCCGTCAGCGGTCCGGGGGCAACAACGGGACAGTCAGGCTCGGCGGCTGACGC
AAACCAGGCCGCCGCGGTCGCTCCTTCCGGTGCGGACAGTACGACCGAAGCCCTTGCCGTGGCCCAGCTTCCG
GCCGGGACGGTCTGCCAGACTTCGGCCATCGTCGACGTGGCGGCCGACCTCATCGACCTCAATGTCAATCAGA
ATGCCTGGATATCGTCGATGCTCGGCTACAAGCTCGGCTTTTTCGGGATTGACTGGGACGACACGCCCTGGCT
CGACAACAAGGCGTCCTTCCAGCGCGGCGTCAACCAGGCGGTGCGCCGCACGTCGGTGGAACTCGTCGATTCG
CTTGGGCGCGTCCGTGGCACGTCGGGGGTCAACAACGAGCTCCAGAACGCGCGCGGAAGCATCCAGTTCGACG
AGGAAACCTGGTATTTCGGCCTGAACCCCTTCGGCCCCAAAACCCCGACCCCGAGCTTCTACCGCTCGGCTAT
GCGCGATCTCAGGAAGTTCAACGTCTCGCTCGGCAAGTGCGAAGCGATCTTCGACGGCCGATCCGACAACATG
GTCGAGTTCCTCGACCGGGTCGCGAACGATCTCGGCAATACCTCGGCGATCATCCGCGAACGTTCGGAGTTCC
ACAATGGCGGCTGGTTCGACACGCGGGCGGACGACCGCTTCTGGTTCGCCTTCGGCCAGCTCTACGGCTATTA
CGGCATTCTCTCGGCCGCCGGCGCCGATTTCGAGAATGTCGTCACCCAACGTGGCCTGGCGCCGATCTATACC
GAAACGATGAAGCAGCTTCGCGCGGCACTCAGGATCCAGCCGCTCATCATCTCGAACGGGCGCGAGGACGGCT
GGATCATGCCGACGCACCTCGCGACGATGGGCTTCTACATCCTCCGGGTTCGATCGAACCTCGTGGAGATGCG
CGATATTCTTGCCCGTTGAATGAACTTCTCCGCCTGGTCCATCCGCAATCCGGTCGCGCCCATCCTGGCGTTT
TTCGTGCTCGTGGTGCTCGGATGGCAGTCGTTCAATTCGCTGCCCATCACGCGCTTCCCGAACATCGACGTGC
CGATCGTTTCGATCGCCGTCACACAGAGCGGTGCGGCGCCCGCCGAACTGGAAACCCAGGTCACCAAGGAGAT
CGAGGACGCGGTCGCCGGCGTCTCCGGCGTCGACCATATCGAGTCGACGATCACCGACGGCATTTCGACGACC
GCCGTCATCTTCCGCATGGAAGTCCCGACGACGCAGGCCGTGCAGGATGTCAAGGATGCCATCGACCGCATCC
GAAGCGACCTGCCGACCTCGATCGAAGAGCCGATCGTTTCCAAGGTCGATGTGGAAGGCCAGGCGATCCAGAC
ATTTTCCGTCTCGTCGCCCGGCATGACGCTGGAAGAGCTGTCCTGGTTCGTCGACGACACGATCAAGCGCGCC
ATCCAGGGCCAAACCGGCATCGGCCGTGTCGACCGCTACGGCGGCTCCGACCGCGAGGTCCGGGTCGAGCTCG
ATGAGGACCGTCTGAATTCCTTTGGCATCAGCGCCGCCGACGTCAACGCCCAGCTTCGCCGAATGAACATGGA
CCTCGGTTCCGGCCGCGGCCAGGTCGGCGGCAGCGAACAGGCGATCCGCACATTGGGCGACACGCGCGACGTA
ACGGCGCTGGCGGGTACGATGATCTCGCTGCCGAACGGCCGCTTCGTCCGTCTGTCGGAACTCGGCAAGGTGA
TCGACACCTATGAGGAGCCGAAGTCGTTCTCCCGCTTCAACGGGCAGCCGGGCGTCACCTTCGCCGTGTTCCG
CGCCAAGGGGGCGAGCGAAGTGTCGGTCGCGGAAACCGTCGGCAAGACGCTCGATGAAATCCGCGCGAAGCAT
CCGGATGTCTCCATCGAGATGGTGGACGATTCGGTCTACTTCACCTACGGCAACTACGAAGCGGCGATTCATA
CGCTGATCGAGGGCGCCGTGCTCGCCGTCATCGTCGTTATGCTGTTCCTGCGCAACTGGCGCGCGACCTTGAT
CTCGGCGGTCGCACTGCCGCTCTCAGCCATCCCCACCTTCTGGGTGATGGAACTGCTCGGCTTCTCGCTGAAC
CTCGTCAGCTTCCTGGCGATGACGCTCGCGACGGGTATTCTCGTCGACGATGCGATCGTGGAGATCGAGAACA
TCGAGCGTCATATCCGCATGGGCAAGTCGCCTTACCGCGCAGCGATCGAGGCTGCGGACGAGATCGGCCTTGC
GGTGATCGCCACCACCTTCACGATCATCGCCGTCTTCGTGCCCGTATCCTTCATGCCTGGCATTCCGGGACAG
TACTTCATCCAGTTCGGCCTGACGGTCGCCGTCTCGGTGTTCTTCTCGCTCCTGGTCGCCCGCCTGATCACGC
CGGTCATGGCCGCCTATCTGATGAAGCCTTCGGATGTCAGCGGGCATCATGACGACGAAGGCTTCATGATGCG
CCAGTACACGCGCCTCGTACGCTTCACCACGAAGCGCTGGTACACGCGCTACTCCACCCTGCTTGCGGCGATC
CTGGTCTCGGTCGGTTCCGTGGTCGCGCTGCTCGTCTTCGTCCCGGGCAGCTTCCTGCCGCCGGAAGACAGCT
CGCGCGTCAGCCTGTCCATCGAACTGCCGCCGGACGCAATGCTCGCGGATACGGACCGGACCACCACCGAGAT
CTACAATCGCGTCAAGGATATCGACGGCGTGGAAAACGTCTTCGTACTCGGCGGCGCCTCGCCCAAGGGCGAC
CTCGAACTGCGACGCGCTGCCGTCACGGTGCTGCTCGAGAAGCTCGATCATTCGCTGATCAACAAGGTCGTCA
ACGACGTGATCGGCCGCACACCGCTGATCGGCCAATACCTGCCGAAGCTGCCCCCGGCCGGTCGGATCAAGCC
GCAATCGGAGATCGAGAAGGAAATCTTCGCCCGATTGCGATCGATTCCCGATGTCCGCGTCACCAAGCTGAAC
GATCGCGGCGAGCGTGATCTGTCGTTCAACCTGCTTTCCAGCAACGAGGCGGATCTCGACAAGGCGGTCGCCA
CCCTGGAAGCGGATCTGCGCCGAGATCCGCTGCTTGCCAACGTTAGCCCCGAGGGAGCGCTGCCCCGACCGGA
ACTGCAGATCCGGCCGCGCGACGAGCAGATGTCGCGCCTTGGCATCACGACGGCGCAGATCTCCGAGGTGATC
CGCATCGCCACGATCGGCGATATCGACGCGCAGTTGACGAAGATTGCGCTCGATGGGCGCCTGATCCCGATCC

GGGTGCAGCTCGACCGTGATTTTCGCACCGACCTGGCGGCGATCCGCAACCTGAAGGTCCAGACGGCATCCGG
AGCGACCGTTCCGCTGTCGAGCGTCGCGGACATCAACTATGCGGAAGGGCCGAGTTCCATCAAGCGCTACGAC
CGCTACCGCGTCGTCAAGCTCGGCGCCGACCTGCCCGCCGGCGTGGCGCTCGACACGGCATCGGCCCGCTTCA
GGGAAATCGCCGCCGACGCCGATCTGCCTGAAACGGTCCAGTTCCTTGAAAGCGGCGACGCCGAGGTGCAGGC
CGAGATGCAGGAGAGCTTCGGCAACGCCATGCTGCTGGGTCTGATGATGGTGCTGGTCGTGCTCATCCTCCTG
TTCAAGGATGTGATACAGCCTTTCACCATCCTGTTCTCGCTGCCGCTCGCGATCGGCGGCGTGGCAGCCGCCT
TGATCCTGACTCAGAACGCGCTGTCGATGCCGGTGCTGATCGGTATCCTGATGCTGATGGGCATCGTCACCAA
GAACGCCATCCTGCTCGTCGACTTCGGTATCGAGATGATGCATCACGGCATGGACCGGACGCTCGCCATGATC
GAGGCCGGACGGAAGCGGGCTCGTCCGATCGTCATGACCTCGATCGCCATGTCCGCGGGCATGCTGCCCTCGG
CGCTGGGCGTCGGCGAAGGCGGTTCCTTCCGTGCACCGATGGCGATCGCCGTCATCGGAGGCATCATCGTTTC
GACGGTCCTGAGCCTCGTCGTGGTTCCGTCCTTCTTCCTGATCATGGACGACCTGTCGCGTCTCCTCGCCTGG
GCTTTCGGCCGCTTCATCGGCAAGAAGGACGAGGAGGCACTGCCGCTCGACCAGGAGGCGCTGAGCAAGCTTG
CCGCCGAACAGGGCAGCACGATCGAGAGCCTCGAAGAGCGCATCAAGGCGCTCGAAGACGAGAAGCGCCGCAA
GTCCGACGGCAAGGTCATCAGCCATCCGGCGCTGGCCGCGGAATAGATGTCGCGTTTAAACAGGTTTCTGATA
TCGGCACTGGCCGCAGCGGCTATTGCCGCGCCGGCGCTGGCCACTTCGGCAAGTGCCGCGACGCTGCGTTGGG
GCAGCCGGGCAGATATCTATTCGCTCGATCCGGATTCCGTTCCCTCGACATCCAATCTGGCGTTCCTGAACCA
CATCTATGAAGGTCTGATCCGTTATGGCCCGAACTTCGAGATCGAGCCGGCGCTCGCCACCGAGTGGAAGCTG
ATCGACAGCAAACACTGGCGTTTCACCCTGCGCAAAGGCGTGAAATTCCACGACGGCGCGGATTTCACCGCCG
ACGACGTGGTCGCCTCGATGAACCGCGTTTCGGACCCGACTTCGCCCCTGCGCGGCAATATTCCGCTCTATGT
CGGCGTCAAGAAGGTCGATGATTTCACGGTCGATATCGAGGTGTCGGCGCCGAGCGCGCTGTTCCTGAACGAC
ATGAGCAATATCTTCATGTTCAGCGCCAAATGGCTGAAGGACAACAACGCGGAAAAGCCGACCGATATTGCGT
CCAACACCGAGAATTACGCCACGCACAACACGAACGGCACAGGCCCGTTCAAGCTGGAAAGCCGCGTTCCGGA
CAGCAAGACCGTTCTCCTCGTCAACGATCAGTGGTGGGATCAGAAGAAGCACAATCTGGACCGGATCGAATAT
ATTCCGATCTCGTCGGCGGCAACGCGTGTAGCGGCGCTTCTTTCCAGCGAAATCGATCTGCTCGATTCCGCCC
CCATTCAGGATCTTCCCCGTCTGGAATCCTCGCCGGGCATCACCGTGAGCAAGCGCACGGAGCTGCGTACGGT
TTTCATCGGCTTCAACGGTAAGGAGAAGCTTGAAGACGGGCGTCCGAACCCGTTCCTGGACCTTCGCGTCCGT
CAGGCCGTGGAAGCAAGCATCGATCGCGATCTCATCAACAAGAAGATCATGCGCGGTCTGGCGCGGCCTTCCG
GCTCGCTCATCGCACCGGAAATCGCCGGCTATGCGAAGTCGCTCGACACTTATCAGCCTGCCGATTCCAAGCT
TGCCCAGAAGCTGCTCGAAGATGCCGGCCAGAAGGATCTCGCCTTCACCTATCTGTGCATGAACGACGAGAGC
ATCAACGAGGAAGACATCTGCTCGGGCATTGCAAACATGCTCAAGCGTGCCGGCTTCCAGCCCACCATCGACA
TGGGCCCCCGCGCCGTGCAGCAGCCGAAGCGCACCAATGGCAAGGCCGACATGTTCAATCTGAGCTGGGCAAA
CGAACCGACACTCGATGCCTATTCGCTGCTGTCTCAGGTTCTCTCCACACGCAGCGGCTCGACGGGCGTGTCC
AACTATGGCGGCTGGTCCTATCCGGAACTCGACGATCTGGTGAAGAAGGCGGCACAGGAACCTGACACCGCCA
AGCGTCTCGCGCTGGAAGAACAGGCGCTGAAAATCGCCAAGGACAAGGCCATCCTGATCCCGCTTCACCAGCA
GCCGATCGCCTGGGGCATGCTGGACAAGGTCAAGAGCGTGGATTTCCGCGCCGACAACAAGCCGCGCCACTGG
CACACGCAAATGGCCGAATAGATGCAGCCGCAGGAGGCGCGCGTGTGGGCAACCCTGGCCGGTGCACTTCTTT
TCATCAGCGGGCTTCTCTACATGGCATGGGAGGCGCTCGGACGGCGACGGCTGAGCGAGCCGCCGCAGCCCGC
GGAACGCAATCCCAAGCAGACGCTTGAGCCGCGCGGGCAAGGCCTGCGCTTTCTGAGCCCGTTGCGCAACTGG
CTGGGCGTCGCGCTCATGCTGGTCGGCGGAATTCTCCTGCTCTTCGGCATGCGCTGAATGAGACACGCGGAAA
TCGGAAAGCTCGACAAATCCCAGGCCAGGCATGTCTGGGACGTGGCGGAGTATTGCAGGCGGACCGGCATCAA
CAAGGCCGAGGAACGCCGCCTGATCAGGGTTCTCGGGCGATTTGCATCCAGCCACGAGCTTCAGATGAACATC
GTGCGTCCTCAGACGCGCATCCGCTGAGTGAACGCCAGGGCCTATTTCTATCTCGGTATTACCGCGCTCTTTT
GGGGCGGCAATTCGGTAGCGGGCAAGATGGCCGTCGGGCACGTCAGCCCGATGATGCTGACGACCTTGCGCTG
GTTGCTCGCGCTTGCGGTCATCCTGGTCCTGATGACGCCGCAGATCCGCCGCGACTGGCAAAAGATCCGCCAG
CACTGGCTGCAGCTGCTTGCCTATGGCGCCGTCGGCTTCACCATGTTCAATGCGTTCCTGTATTCCGCGGTGC
AATACACCAGCGCCATCAATGCCGTCATCCTTCAGGCCGGCATTCCGATGCTGATCTTCATCTTCAATTTCGC
GCTTTTCAAGATGAAGGCGTCGCTCGCCCAGGTCATCGGCTTTACCGTCACATTGGTCGGCGTGCTCATTACC
GCCGCCCATGGCGATCTCGCGAGCCTCATGCGCCTGAGCTTCAATTTCGGCGACGCGCTGATGATCCTCGCCT
GCATCGTCTATGCCGCATATACGGTTGCGCTGCGGTGGAAACCGGCGATGCATTGGCAGAGCTTCATAGCCGC
CCCGGCCTTCGGGGCGCTGCTGAGCGCCATACCGCTCCTCTTGTGGGAGATGGGGAAAGGCACGGCGATCATG
CCGGATGCGACCGGCTGGGCGATCGTGATCTATGCGGCGATCTTCCCCTCGCTGATGTCGCAGGTTCTCTATG
TTCGCGGCGTCGAGATGATCGGCGCCAACCGTGCCGGTCTGTTCATCAACGCCATTCCGGTGTTCGGGACCCT
TCTCTCCGTCCTTCTGGTCGGCGAGATATTTCACCCGTTTCATCTCGCGGCGTTGGCACTCGTCCTCGGCGGC
ATCGCCATCGCCGAACGCGGCCGGCCGAAGCTGCCGCGGTGAATGAGAAATAACCTGCTTCCCGTTGCCGCGC
TGCTGCTCGGTACCCTGTTTCTCTTCCTCGGAAACGGCCTGCAGGGCCTGCTCCTGCCGGTGCGCGGCACCGC
GGAGGGCTATCCGACAACCATCCTCGGCCTGTTCGGTACGCTCTGGGCCACCGGTTTCGTGCTTGGCTGCTTC
TTCGCGCCGAACGTCGTCAAGCGCATAGGGCATGTGCGCGCCTTCAGCGTGTTCACGGCCTTGATCGCCATCG
TTTCCCTGCTGACGGGCATTCTGATCGACCCGATCTGGTGGCTGGCGCTCCGCGCAGTCACGGGTTTCTCCAC

TGCCGGAACGTCGATGATCATCGAAAGCTGGCTGAACGAGCGCGCCACCAATGAGAGCCGCGGCGTGATCTTC
TCGCTCTATATCGCCATCACCCTTTTCGGCGTCGTCGGCGGCCAGATGATGATTCCCTTCGGCGAAACGTCGA
CGACCTTCTTCTTCATGATCTGCGGCATTCTCTACTGCGTGGCCATGCTGCCGACGCTGTTGTCCAGGGCCGC
CTCGCCGCAGCCGCTGAAACAGGTCCGCCTCGATCTGCGCGGCCTCTACCGCAATTCGCCGGTTTCCTTCCTC
GGCATCCTGCTCATCGGCATTGCCAACGGCGCATTCGGCACGCTCGGCGCGGTCTTCGGCCGCCAGGCGGGGC
TGTCGGACAGCACAGTTGCGGCGATGATGAGCGTCGCGATCTTCTCGGGCGCCGTCATGCAACTGCCGGCCGG
CCGCATCTCCGACCGTATCGACCGCCGCTATGTGCTTGCCGCGCTCGCCGGGGTCGGCGCTCTCGCCGGCCTC
CTGATCTTCCTCGTCGAGCCCGGCCAGGTCTGGATCGTTCTGACGTTGATCGCGATCTACGGCGCGGCCGCCA
ATGCGCTCTACCCGATCGCGGTTTCCCACGCCAACGACTTCGCCACGCCCGAGGATTTCGTGAAGGTTTCCGG
CGGGCTCCTGCTGCTCTACGGCATCGGCACGATCATCGGCCCGACGATCGGCGGGCCGATAATGACCGCCAGC
GGGCCTTATGGCCTCTTCATGATCACGGCCTGTGCGCATATGCTGATCACTGCCTATGCAATCGTCCGCAGCC
GCCGGCGCGCACCGGTGCCCGCGGCCGAGCGCGAAAACTTCTCGCCGGTCAATGCCGGCACGGCGACGACGCC
GGAGAGCCTGCAGCTCTCGCCGCGTGCGGCTCCGCTGGAGGAACTGCCGGACGAAGGCGCCGACGATCCCCAG
GAAGAGGAGAGATCGAATGAGCCTGTATGAATGAGACGGAACAGGAAAGTCAAAATCCTCGCGACGCTCGGGC
CGGCATCGGCCGACGAGCAAATGATTCAGAAGCTGCACGAGGCGGGGGCCGATCTGTTCCGTATCAACATGAG
CCATGCAAGCCATGAGGTCATGCGCACGCTGATCGAGCGGATCCGTGTGGTCGAGGCGCGCTGCGGCCGTCCG
ATCGGCATCCTCGCCGATCTGCAGGGGCCGAAGCTGCGCGTCGGAAAGTTCGCCGAAGGCAAGGTCGAGCTCA
AGATCGGCCAGACATTCACGCTCGACAACCGCGACGTGCCCGGCGACAGCCGCCGCGTCTATCTTCCGCATCC
GGAGATCCTCGAAGCGGTGAAGCCCGGCCATCGCCTGCTCATCGACGATGGCAAGCTGCATCTGCGCGCCGAA
AAGACCGATGGCAAGAGCATCGTCACCACGGTCGTTTCCGGCACGCGGATTTCGGATCGCAAGGGCGTCAGCC
TGCCCGATACCCTGCTTGGCGTCGGCGCGCTGACGGACAAGGACCGCGTCGACCTCGACGCCGTGCTGGCGAC
AGGAGAGGTCGATTGGGTGGCGCTTTCCTTCATCCAGCGCCCTGAGGACCTTGCCGAGGTTCGCAAGATCGCC
CGCGGCCGCGTTGGACTGATGTCGAAGATCGAGAAGCCGCAGGCGATCGAGCGGATAGACGAGATCATCGAGC
TTTCGGATGCGCTGATGGTGGCACGCGGCGACCTGGGCGTCGAAATGCCGCTCGAATCCGTACCGGGACTTCA
GAAGCAACTGACGCGTGCCTGCCGGCGAGCCGGCAAGCCGGTCGTCGTCGCCACGCAGATGCTCGAATCGATG
ATCTCCTCGCCTGTGCCGACGCGCGCAGAAGTGTCGGACGTGGCTACCGCCGTGTTCGAGGGTGCCGATGCGG
TGATGCTTTCGGCCGAATCCGCCTCCGGCGAGTACCCCGTCGAGGCGGTTTCTACCATGGCGTCGATCGCCAG
CAACGTCGAGCGCGATCCGCATTATCCGGGGATCATCTACGCTCAGCGCACGCCGCCGGAGGCAACGGGCGCG
GACGCCATTTCGCTTGCCGCTCACCAGATCGCCGAGACGCTCAAGCTTGCCGCAATCGTGACCTACACCTCCT
CTGGCACCACCGGCCTGCGCGCGGCGCGCGAGCGCCCCCAGGTGCCTGTCATCGCGCTTTCGCCGATCGTCCA
GACGGCCCGTCGCCTTTCCGTGGTCTGGGGGCTGCACTGTGTCGTCACAGAGGACGCGACCGACCTCGACGAT
ATGGTCAACCGCGCCTGTCGAATCGTCTCGACGGAAGGCTTCGGCAAGCCGGGAGACCGCATCATCGTTACCG
CCGGCGTGCCGCTCGGTACCCCCGGCGCCACCAACATGCTGCGCATCGCCTATATCGGCTCGGACGGCCAATC
GGGCATCTGAATGACTGCGAAAATCCTCCTTGCCGAAGACGACAACGACATGCGCCGGTTTCTTGTGAAGGCG
CTGGAAAAAGCCGGCTACAAGGTTCTTTCCTATGACAACGGCGCCAGCGCCTATGACCGGCTTCGCGAAGAAC
CCTTTTCGCTTCTCCTGACCGACATCGTCATGCCGGAGATGGACGGCATCGAACTCGCGCGCCGCGCGACCGA
ACTCGACCCCGACCTGAAAGTGATGTTCATCACCGGCTTCGCCGCCGTTGCGCTGAACCCGGATTCCAAGGCG
CCGAAAGACGCCAAGGTCCTTTCCAAGCCCTTCCACCTGCGCGACCTCGTCAACGAGGTCAACAAGATGCTGG
CCGCCTGAATGAACATCCATGAATACCAGGCCAAGGCTCTGTTGAAGAGCTATGGCGCGCCGGTCGCGGAAGG
CGTCGCGATCTTCTCCGCCGACGAGGCCGAGGCGGCCGCGAAGAAGCTGCCGGGGCCGCTTTACGTGGTGAAG
AGCCAGATCCACGCCGGCGGCCGCGGCAAGGGCAAGTTCAAGGAACTCGGCCCCGATGCGAAGGGCGGCGTGC
GCCTGGCCAAGTCGGTGGACGAGGTCGTCGCCAATGCCAAGGACATGCTCGGCAACACGCTGGTGACCAAGCA
GACCGGTCCGGCCGGCAAGCAGGTCAACCGCCTCTATATCGAGGACGGCGCCGATATCGACCGCGAGCTCTAT
CTCTCGATCCTCGTCGATCGCTCCGTCGGCCAGGTGGCCTTCGTCGTTTCGACCGAGGGCGGCATGGATATCG
AAGCGGTCGCCGAACATACGCCGGAGAAGATCGTCACCGTTGCGATCGATCCGGAAAAGGGTGTGACCGCAGA
GAATCTGAAGACGCTGGCCGACGCGCTGAAGCTCGAAGGCGAAGCGCGTGCCGACGCCGAGAAGCTCTTCCCG
ATCCTCTACAAGGCATTCGTCGAAAAGGACATGAGCCTGCTCGAGGTCAATCCGCTGATCGTCATGACGAACG
GCCGGATGCGCGTCCTCGACGCCAAGGTCTCCTTCGACGGCAATGCGCTCTTCCGCCATGAGGACGTGGTCGC
GCTGCGCGACACGACGGAAGAGGACGACAAGGAAATCGAGGCGTCGAAATACGACCTCGCCTATGTCGCCCTG
GACGGCAATATCGGCTGTATGGTCAACGGCGCCGGTCTTGCCATGGCGACGATGGACATCATCAAGCTCTACG
GCGCCGAGCCCGCGAACTTCCTCGATGTCGGCGGCGGCGCCTCGAAGGAAAAGGTCACCCAGGCCTTCAAGAT
CATCACGGCCGATCCGGCGGTGAAGGGCATCCTCGTCAACATCTTCGGCGGCATCATGAAATGCGATGTCATC
GCCGAGGGCGTGCTCGCAGCGGTCAAGGAAGTCGGCCTCAAGGTGCCGCTCGTCGTGCGCCTGGAAGGCACCA
ATGTCGAGCTCGGCAAGAAGATCATCAATGAATCGGGCCTCAACGTGATTTCCGCCGATGATCTGGACGACGC
CGCCCAGAAGATCGTTGCAGCCGTGAAGGGAGCCTGAATGATACGTAGAGCTTCACTTGTCCTGGTCGGGGCG
CTGATGGGCGCGACGGCGATGGGCGTCGTCTACTCGGCAACCATGCCGGCCGTGGCAGCAAATTCGTCGACAT
ACCGCGAACTGGCGATCTTCGGCGACGTCTTCGAGCGCGTGCGCGCGCAATACGTGACGCCGCCGCAGGACGA
CAAGCTGATCGAGAACGCGATCAACGGCATGCTCACGTCTCTCGACCCGCATTCGAGCTACATGAATTCGACC

GACGCCGAAGATATGCGCACCCAGACGCGCGGCGAGTTCGGCGGCCTCGGCATCGAGGTCACCATGGAGGAAG
ATCTCGTCAAGGTGACCAGTCCGATCGACGACACGCCCGCCGCGCGCGCCGGTGTGCTCGCAGGCGATTTCAT
CTCGAAGATCGACGGTCAGGACGTCCGCGGCCTCAAGCTCGAGGAAGCGGTCGACAAGATGCGCGGTGCCGTC
GGCACGCCGATCAAGCTGACGATCCTGCGCAAGGGAGCCGAAAAGCCGATCGAGCTGACGATCGTACGCGACG
TGATCGCAGTACGCGCGGTGAAGGTCCGTGTCGAAGGAGATGTCGGCTATCTCAGGGTCATCTCGTTTACGGA
GAAGACCTTCGAAGACCTGAAGAAGGGCATCGAGAAGATCCAGGCGGAAGTTCCCGCGGACAAGCTCAAGGGC
TATGTTCTCGACCTGCGCCTCAATCCGGGCGGCCTGCTCGACCAGGCGATCAATGTCTCGGACGCATTCCTGG
AGCGTGGCGAGGTCGTTTCGACCCGCGGCCGCAATCCGGATGAAACCCGCCGCTTCAATGCGACCCCGGGCGA
TCTGGCCGGCGGCAAGCCCGTCGTCGTGCTCGTCAACGGCGGTTCGGCATCCGCGTCGGAAATCGTGGCCGGC
GCTCTCCAGGACCTGAAGCGCGCCACCGTTCTCGGCACGCGCTCCTTCGGCAAGGGTTCGGTGCAGACGATCA
TCCCGCTTGGCGACGCCGGCGCGCTGCGTCTGACGACGGCGCTCTATTACACGCCGTCCGGCAAGTCGATCCA
GGGCACCGGCATCACGCCTGACATCAAGGTGGAGCAGCCGCTGCCGCCCGAGCTTCTGGGCAAGGTGGAAGCG
CAGGGCGAATCCGATCTGCGCGGCCACATTCCGGGCCAGGAAGAGACCGAAGAGGGCTCCGGCTCGGTAGCTT
ACGTGCCGCCGGAAACCAAGGACGACATTCAGCTGAACTATGCGTTCGACCTGTTGCGCGGCAAGAAGACGGA
TCCGTCCTTCCCGGCCAATCCGGAACAGGCGCAGCTGAAGGAATAGATGACCATCGCCGCCGGCGCCTTCGCC
CTCGGCGGCATCCTGCTTTTCGTCGGCCTCGCCGGCGCGGCACTGCTTGGACAGTCGGCCGGCAGCGCCGGCG
GTGGTCCGCTTTTCGATGCCTATATCTGGCGCATCACACGCTTCACGCTGCTGCAGGCCAGCCTCTCGACACT
TCTTGCCATCCTATTCGCCATACCGGTTGCCCGGGCACTTGCCCGCCAGGCTTCGTTCCCCGGCCGCATCTGG
GTGCTGCGGCTTCTGGCGCTGCCACTCGGCCTGCCGGCGCTCGTCGCCGCACTCGGGCTCATCGAAATCTGGG
GCCGGCAGGGGCTCTTGAACAGGGTGCTTTCGGCAGCCGGGCTGGAGCAGCCGGTCAGCATCTATGGCCTCTC
CGGCATTCTCATTGCCCATGTCTTCTTCAACATGCCGCTCGCCGCACGCTTGATGCTTGCCGGCATCGAGCGC
ATTCCGGCCGAATACTGGCGCACCAGCGCCAATCTCGGCATGGGATCCTTCGCCACCTTTCGCTTCATCGAGT
GGCCGGTTATCCGCGGATTGCTTCCCGGCATCGCCGGGCTCATCTTCATGCTCTGCGCCACCAGCTTCACGCT
CGTGCTGACGCTTGGCGGCGGGCCGGCCGCCAGCACGATCGAAGTGGCGATCTATCAGGCGCTGCGCTTCGAC
TTCGACCCGCCGCGGGCGATCGCCCTTTCGGCACTTCAGGTCGCCCTTACCGGAGCGCTGCTTCTCGTCCTGA
AATTGATCGCGCCGGCACCGCTGGAGGGAGAGACCAGCGGCAGGGCCATCCGGCGTTTCGACGGCGCAAGCGG
TCTGTCGCGGCTTGCCGACCGGATCTGGCTGATACTCGCGGTCATCCTCGTCGGCCTGCCATTTGCGGCCATC
GCCTATAGCGGATCGAAGGCCGACCTCGTCCGTCTCGTGAGCGAGCCGGCATTCCATCGGGCGCTCGCCATGA
GCGCGACGATCGCCCTTGTCTCCGCCACGATCTCCGTCGCCATCGCGGCGCTGATGATCCGTACCGAACGGCT
CGCGCTGGCGCAGCGGCGCCCCGGAGCGGCGCCTCGCCTCTTTGCAGGCACGATCGGCGCGAGCACCTCCTTC
ATACTGCTCGTGCCTCCCGTCGTGCTCGGCGCCGGATGGTTCCTGCTCCTGAGACCTTTCGGCGACGTCGCCC
GTTTCGCGCCGGCCGTCGTCGTCGCCATCAATGCGCTGATGGCGCTTCCCTTCGTTCACCGGGTGCTCGCGCC
GGCCATGGCGACGCATGCCGCGCGCACCGCGCGCCTTGCCGCGAGCCTTGGCATCGGCGGCTTTCACCGCCTG
CTGTGGATCGACTGGCCGGGCCTGCGCAAGCCTCTCTTTGTCGCCTTTTCCTTTGCCATGGCCCTCTCGCTCG
GCGATCTGGGAGCAGTCGCTCTTTTCGGCTCCGAGGACATGGTAACCCTCCCCTATCTGCTCTATAGCCGGAT
GGGCAGCTATCGCACCGCGGATGCGGCCGGACTGGCGCTGGTTCTCGGCCTTCTCTGCCTTGTCCTGACGGTG
CTCGGCACGGCGGGAGAGGAGAGGCCGCCCGAAGGTAAAAGCACATGAGTGAAAGAGAATAGTTTCGTATCCG
CCGACGATATTCGCTCGGCCTTTTCCGCTGCGATGTCGTTGATGTACCGCGAGGAAGTGCCGGCCTACGGCAC
GCTGATGGAGCTCGTCGCCAGGGTCAACGGCGAAACGCTCTCGGCCGACGCCACCCTGAAGGGGCGCCTGGAA
GCGACCGATTCGCTCGAACGCATTTCCGAGGAGCGTCACGGCGCGATCCGTCTCGGCACGCCGGCGGAGCTTT
CGATGATGCGCCGGGTATTTGCCGTGATGGGCATGTATCCGGTCGGCTACTACGATCTGTCGACCGCCGGGGT
GCCGGTCCACTCCACGGCCTTCCGGCCGGTGGGCGACGCGGCTCTGAAGCACAACCCCTTCCGCGTCTTCACC
TCGCTTCTGAGGCTCGACCTCATTTCCGACGAGGCCCTTCGCGCCGAGGCGGAGGCGATCCTGAAAGAGCGCC
GGATCTTTACATCCGGTGCGGTCGAGCTTACCGAAAAGGCCGAGCGGGACGGCGGCCTCGACAAGGCCGATGC
CGAGCGTTTCGTCGCCGAAGTGCTCGAGACCTTCCGCTGGCACGACGAGGCGAATGTCAGCGCCGGCATGTAC
GAGCGCCTGCACGATGCGCACCGGCTGATCGCCGACGTGGTCTCCTTCAAGGGGCCGCATATCAACCATTTGA
CGCCACGGACGCTCGACATCGACCGCGTCCAGGCGCTGATGCCGGAATATGGCATTGCCCCGAAGGCCGTCGT
CGAAGGACCGCCGACACGCAAATGCCCGGTCCTGCTTCGCCAGACGTCCTTCAAGGCGCTCGAAGAGCCGGTT
TCCTTCCGCGACTCCGACGGCAGCTGGAAGACCGGCTCGCATACCGCCCGCTTCGGCGAGATCGAGCAGCGCG
GCATCGCTTTGACGCCTAAGGGCCGAGGTCTCTACGACCGGCTGCTCGACGAATCGCGCAAGATCGTACGCCC
GGCCGCCGATGGTTCAAACGCCAGGGAATACGAAGCGGCCTTGGCCCAGGTCTTCGAGGCGTTCCCCGACAGC
TGGGCGGAGCTCCGCGAGGCGGGCCTCGGCTATTTCAGCTACTCCCTGACGGATAAGGGCCGGCGGACGAAGC
TGCCCGGCCGGCGTGATCTCAATTCCCTGATCGCCGACGGCCTCGTTCAATTCGATCCGATCGTCTATGAGGA
CTTCCTTCCCGTCAGCGCCGCCGGTATCTTCCAGTCGAACCTCGGCGACGGTGCGCAGCAGGAATTCGAAGCG
AGCCCGAACCAGAAGCGCTTCGAGACCGATCTCGGCGTCGCCGTGCTCAACGAATTCGATCACTATGCCGGCA
TCGAGCAGGCATCGATCGAAGGCTGCCTCAAGGCGCTTACCGCCGCCATGGCAGCGGAGTGAATGATTGACGA
GCACCATATCGACGCATTGACGAAGCTCCTTGGCGACAAGGGCGTCGTCACGCGTCGGGAGGACATGGAGGCT
TACGAGGCCGGCGCACGCTACGACCGCGGCCGCGCGGCGGCCGTCCTGCGTCCGGTAACGACGGAAGAGGTCT

CGGCCGCCGTCGGTTACTGCGTCCGCAGCGGCATCGCCCTCATCCCCCAGTCGGGCAATACGGGCCTCGTCTC
GGGATCGACGCCGGACGAGTCCGGCAGCGAGGTCGTTGTCAGCCTCGACCGGCTGACCCGCCAATTCGCGCTC
GACCTCGACAATCGCTCCGTGCGCGTCGATGCCGGCTTCCGCCTGTCCGAGCTCAATCGGCGCCTGGAGGAAC
ACGGCCTGTTCTTCCCGATCGATCTCGGCGCCGACCCGCGGATCGGCGGCATGATCGCCACCAATACCGGCGG
CTCGCGCTTCCTCAAATATGGCGACGTGCGCCGCAACACGCTGGGTCTGAAAGTGGTCCTCGCCGACGAGGCG
GGCACGGTGCTCGACCTCGGCTCGGACCTGCGCAAGAACAATACCGGCGTCGACTGGAAGCAGATCTTCATCG
GCACCTCGGGCGCCTTCGGCATCGTCACCGAATGCGTGCTCAATCTCGAGCGCCTGCCGAAGCAGACGGCGAC
CGCCTTCCTCGTGCCGGCGAGCGGCGCGCATGTCCTGCCGCTCCTGAAGGCCATGGAAGAGCGGCTCGGCGCC
TATCTCTCGGCCTTCGAGGGAATGTCGAGGAACGCGATCGCCGCGGCCTTCGCCCATGTGCCGGCGCTGAAAA
ACCCCTTCCAGGGCGGCAAGGTCCCCGACTACGTGATCCTCGCCGAAATCTCCCGCACCTCGAGCCCGCGCGA
AGGCGAGCAGCCGCTCGACGCGGTTCTCGAAAGCGTGCTCGCGGAGATATGGGAGATGGAAGAGGTACTGCTC
GCCGATGCACTCGTCGGCCCGCCGCACGAGATCTGGGCGCTGCGCCATGCGCTTTCGGAGGGCGTAAAACACC
TCGGCAAGCTGATCGCCTTCGATCTCTCCTTCCGCAGGGGTGACATCATGGCCTTCTGCGACCATATGAAGAC
GGAAATGCCGGAAAAATTTCCGGGCGTCACGGTCTGCGATTTCGGCCATATCGGCGACGGCGGCGTTCACTTC
AACCTGGTCGTCGCCAAGGATAGTCCGCTACTGGCCGATGCGACCTTCGAGCAGCGACTGCGCGAATGGGTCT
TTGCGGTGGCGGTCGAGCAATATCACGGCAGCTTCAGCGCGGAACATGCGCTCGGCCGCCGCAACCAGGCTTT
TTACGATCTCTACACGCCGGAAAAACTGAAGAACATGGCGGCCGGCCTCAAGACGCTCACCTCGCCGGGCAAG
CTCGGCAGCGTGCGCTTCGGTTAGATGGAGAGCCAGCCGGATATCGTCATCATCGGATCGGGAGTCGGCGGCG
CAACGGTCGCTGCGGGCCTTGCTGCCTCGGGCGCCGAGATACTGATCCTCGAAGCGGGCTCGCATATCGAGGA
CCTGCCGGTCAATCGCGACCAGCGGGCGATCTTCCAGCGCGGCCATTTCCGCCCGAAGGAGACCTGGTACGAG
GCCGGCGGCCCCGGCTTCAATCCCGGCAATTACTACAATGTCGGCGGCAATTCGAAGTTCTACGGCGCGGTGC
TGACCCGCTATCGCCGGGAGGATTTCGAGGAGATGCAGCATCGGGACGGCGTCTCCCCGGCCTGGCCCTTTTC
CTATGAGGAGCTCGAGCCGTGGTATTCCAGGGCAGAGCAGCTTTACCAAGTGCGAGGCAAATTGGGCGAGGAT
CCGACGGAGCCCGCTCATTCGAGCGGCTATCCCCATGGCCCGGTGCCGGACGAGCCTGCGATCGCCATGGTCC
GCGAGCGCCTGGCCGAGATCGGCCTCCATCCCTATTCGCTGCCGCTCGGCGTCGATATCGAGCGCTGGCTCGC
CAAGGGCAAGACGCCGTGGGACGCGCATCCGAACTCGTCCGACGGCAAGATGGATGCCGAAACGGCGGCGCTG
GCGGCGGCGCTGAAATTTCCGAATGTGCGGCTGCAGACCGGCTCGCGAGTGACCCGGCTCGTAACCGGGCCGG
ACGGCAAGGCGATCGAAACCGTGCTCTACGCCAGGGACGGGGCAGAGCACAGTCTTTCGCCGAAGCTCGTCAT
CCTCTGCGCCGGCGCGGTGCAGTCGGCCGTACTGCTCCTGCGCTCGGCCGACGACAGCAATCCGACCGGGCTT
GCCAACCGGTCCGATCAGGTCGGCCGCAATTTCATGAACCACAATTCGAGCGCCGTCATCGCGCTCAGCCCCT
GGTACCGGAACGACTCGGTCTATCAGAAGACCTTCGGCCTCAACGACTTCTATCTCTCCGACGGTGCGGGCGG
GCCGCCGCTCGGCAATGTCCAGCTTCTCGGCCGCATTTCCGGCGCGATACTGAAGGCGAACATGCCGGCAATG
CCGGAATGGCTGCTAAACCGGGTGTCCGCCCGCGGCATCGACTTCTACGCCATGAGCGAGGACCTGCCCTCAC
CGGAAAGCCGCGTCATGGTCGACGGCGAGCGCGTTGTGCTGAAATGGGTGCGCACCAATTGGCAGGCCCATCT
CGACCTCGTCGCCAGGCTCAAGGCGGTGCTGAAGAAAGCCGGCTTCCCGATCGTGCTCGCACGGGCCTTCGAC
AAGCGGACGCCCTCGCACCAGTGCGGCACGGTCCGGATCGGCAATGATCCCGGCCAGGCGCCGCTCGACGTCT
ATTGCCGCGCCTTCGATCATCCGAATCTCTTCGTCGTCGATGCCGGCTTCCTGCCGACCTCCGCCGCCGTCAA
TCCGGCGCTGACCGTCGCGGCACAGGCGCTGCGTGTCGCCGACCATATCGTGAAACAGGATCTGCGGCCATGA
ATGAGCGAGGTCAAGTCGCACGAATTCGATAGTTTCCTGCAGCGATCTGCGGCGATCTACCGGCTATTCCTGC
TCTACGGTCCCGATCGCGGCCTCGTCTCGGAACGCGCGTCCGAGCTCGCCGGCAGCTTCGGCATCCCCCTCGA
TGATCCCTTCGCCGTCGTCAAGCTGGACGCAGCTACCTTGCAGGGCGCCGGTAGCGTGCTTGACGAGGTCAAT
GCCATCGGCCTGTTCGGCGGCGACAAGCTGGTCTGGGTGCGCGGCGCGTCCGCTGAGAAGGCGCTCACCGAAG
CGCTGCAGATCCTCGCGGCCGATCCCCCGGCCGGCGCCCGTCTCATCGTCGAAGCCGGCGACCTCAAGAAGGG
GGCGGCGCTCAGGAAGGTTGGCGAGACTTCCCGCTCCGTCGCTTCGATCGCCTGCTACAGCGACGACATCAGG
GGCCTGCAGGCACTTGTCGACACGGAACTTTCGGAAGCGGGCCTGCGCATCGGTCCCGCCGCGCGCGAACGCC
TCGTCGAGGCGCTCGGGGGCGACCGCATGGCTTCGCGCAACGAGCTTCGCAAACTCGCCCTCTATTGCCGCGG
CAAGGATATGGTCGATGAGGATGACGTCCTCGGTATCGTCGGCGACGCCAGCGCCATTTCGGTCGACGATGCG
GTCGATGCGGTGTTGAAGGGCGACGCCGACGCGCTGCTGCATGCGATGAAGAAGATCACGACATCCAAGACGC
CGCCTTTCCTGGTGCTTCAGGCCTGCCTCAGGCAATTCCAGCAGCTCGACGTGATGCGAGCCGAAATGGATGC
GAGCCGCCAGTCTGCGGGCCAGGTCGTCGCAAGCCTCGGCCGCGGCCTGCACTTCCGCCGCAAGCCGGTTGTC
GAGGCGGCGCTGAAACACTGGACCGGGCCGGCGATTCGACGCGAATTGGGCCGCCTGCAGGCGACGATCTATC
AAAGCCGCAGCCGCCAGAGCCTCGAGGAAAGCCTGGTGATCCAGAATCTCCTGGCGATCACCATCCAGTCGGC
GAGGCGTTGAATGCCGCCCGACACCGCGCTTGAAAGCTTGCTGCGACGGGATCGGGCAATCGTGGCAGCGTCG
CTCGTCGCATTGACGGTGGTCGCCTGGACATACACGCTCTGGCTGGCGGCAGCCATGAACATCGACGGCATGA
GCATGTCTGCCCCCGGCATGGAAGCGGACATGAAGATGGACCCTGCCATGGATATGGATGGCATGGAGATGGG
ATCCCGTGGCGCCCTGTCGCTCGGCACCGTCCTCGGCATCTCGCCGCGTCCCTGGAGTTCCGTGGAAGCCGGC
GTCACCCTGACCATGTGGATCGTGATGATGGTCGGCATGATGCTGCCTTCGGCAACGCCGATGATCCTGCTCT
ACGCCCGGGTCGGCCGGCAAAGCCGGATAGCGGGCAAGCCCTTCGCCGCGACCGGTTTCTTCGCCGGCGGCTA

TCTCCTTGCCTGGGCGGGATTCGCGCTCGTCGCGACGCTTGGACAATGGCTCATGGAGGGAACCCTGCTGACG
CCGGCGCTGGCAAGCGCCAGTCGCATCTTCAGCGGTGTCGTGCTGGTGATCGCGGGTCTCTACCAATGGACGT
CGCTCAAGGATGCCTGCCTCAGCCAATGTCAGACGCCGATCGTCTTCCTGCAGCGGCACGGCGGTTTTCGCCG
CGATCCCGCGGGAGCCGTCGGACTGGGCCTGCGCCACGGGGTCTATTGCATCGGCTGCTGCTGGGCGCTGATG
ACGCTTCTTTTCGTCGGCGGCATCATGAATGTGCTCTGGATTGCGGCAATCGCCATCTTCGTGCTCGCGGAGA
AGGTGATACCGACCGGCCGCGTTCTCTCACGCGTCGCCGGATCTCTGCTCGCGGCGTTCGGCCTTTGGCAGCT
CATTTCGGCTTCGATTTAAATGACTGCGAAGAAGACGATCAGCGGAGCGCGGATTTTCGACGGCATCGACTGG
CACGACGGGGCAGCCCTCGTGATCGAGGCAGGACACGTCAAGGCGATTGCGCCGGCGGGAAGCGTCCCCGCCG
GTGGCGAGACCATCGACGCGCGTGGCCTGCTTCTCGTGCCCGGCTTCATCGATCTGCAGGTGAACGGCGGCGG
CGGCGCGCTTCTGAACGAAAAGCCGTCCCTCGAAAGTATCCGGCAGATCTGCGCGGCGCATGCGCAATTCGGC
ACGACGGCGCTGCTGCCGACGCTGATCACCGACACGCGCGCGGTCAGGACCGCGGCCATCGCCGCAGGCATCG
AGGCGAAAGCCGCGGCGGTTCCCGGCTTCCTAGGCCTGCATCTCGAAGGCCCGCATCTTTCCGTCGCGCGCAA
GGGGGCTCACGATCCGGCGCTGATCCGTCCGATGGGCGATGACGATCTTGCCGAGATACTCGCCTGCGCAAAG
GCGCTCAGCCGTCTGATGCTCACCGTGGCCCCGGAAAACGCCACCAAGGAGCAGGTGCGGGCGCTGGCCGATG
CCGGCGTCGTGGTGAGCCTCGGCCATACCGACGTGGATTACGACACCGCCTGCGCCTATGCCAAAGCGGGTGC
CCGGACCGTCACGCATCTCTTCAACGCCATGAGCGGACTCGGCCATCGCGAGCCTGGCGTGGTCGGCGCGGCG
CTTTCGACCGGCACGCTCCACGCCGGCATGATCGCCGACGGTTTTCACGTTCACCCGGCCTCGATGGGCATCG
CGCTGCGCGGCAAGAAAGGCCCGGGACAGATCTTTCTGGTCACCGACGCCATGTCGCCGATCGGCACCGATCA
GACGAGCTTCTTCCTAAACGGACGGGAAATCCTGCGCCAGGGCGGGCGCCTGACGCTTGCCGACGGCACGCTT
GCCGGCGCCGATATCGACATGCTTTCGTCTGTCCGCTTCGTCCACCAAAAGCTTGGCCTGCCGATCGAGGAGG
CGGTCCGCATGGCCTCCGCCTATCCCGCCGACGCCATGGGGATCGCCTCGCACAAGGGCCGGCTCCTGCCGGG
TACGGATGCGGATTTCGTGCTGCTCACGCCGGAACTCGCAATGAAATCGACCTGGATCGGCGGGGAGACGGTT
TTTGCGGCTTGAATGAGCGACGAAGCGCCCACTTCATCCCCGATTTTACCCGTCGAGAAACCGGCCGCCTACC
GTGTTCTGGCGCGCAAATACCGCCCCAAGGACTTCTCCGACCTGATGGTCGGCCAGGAGCCGATGGTGCGCAC
GCTCACCAACGCCTTCGAAACCGGCCGCATCGCCCAGGCCTACATGCTGACCGGCGTTCGCGGCGTCGGCAAG
ACGACGACGGCGCGTATCCTGGCACGGGCGCTGAACTACAAGACCGCCGAGATCGACAAGCCCACCATCGACC
TGCGCGTCCCCGGCGAACATTGCCAGGCGATCATGGACGGCCGCCATGTCGACGTGATCGAGATGGACGCCGC
CTCGCATACCGGGATCGACGATATCCGCGAGATCATCGAGCAGGTACGCTATCGGCCGGTCTCCGCGCGCTAC
AAGGTCTATATCATCGACGAAGTGCACATGCTCTCGACGCAGGCCTTCAATGGCCTGCTGAAGACGCTCGAAG
AGCCGCCGGAGCATGTGAAGTTCATTTTCGCCACGACCGAAATCCGCAAGGTTCCGATTACCGTCCTGTCGCG
CTGCCAGCGCTTCGATCTCCGGCGCATCAGCGCTTCCGATCTGGTCGGCCTCTTCTCCACCATTCTCGGCAAG
GAGGGCGTGCCCTTCGATCCGGAGGCGCTGGCGATGGTGGCGCGGGCAGCGGAAGGCTCGGCGCGCGACGGCC
TGTCGCTGCTCGATCAGGCGATCGCCCATGGCGGCGGTTCGGTCGAGATCGAAACCGTGCGCTCGATGCTCGG
CCTTGCCGATCGGGCGCGGATCGTCGACCTGTTCGAGCATGTCATCAAGGGCGATGTCGCCGGCGCGCTCGAC
GAGTTCGCCGCGCAATACGAAGCCGGCGCCAATCCGACCGTGGTCCTCACCGACCTTGCCGACTTCACCCACC
TCGTGACAAGGCTGAAATATGTGCCGGACGCGGTCAACGACCAGTCGCTGAGCGAGATCGAGCGCACGCGCGG
GGCCGAATTCGCCGGCAGCGTTGCCGTGACGACGCTTTCGCGCGTCTGGCAGATGTTGCTCAAGGGCATTCCC
GAGGCGGAGAGCTCTGCGCGCCCCGCAGGTGCTGCCGAGATGGTGCTGATCAGGCTCGCCCATGCCGCCCATC
TGCCTTCGCCCGAAGAGGCCGCGCGGCGGCTTCTCGATCTTTCGGGCGGCGAAGGCGGCGGTCGCCCGGCCCC
GAACGGCGGTGGTGGCGGCGGCGGTGCGCAGGCGCCTGCAGGCACCCCCGTCGAGGCTCGCGCCGTGGAGACG
GCAGTCTCGCGTCCAAGCGGTAATGGCGCGACCATGCTGCGCGCCGTGCCGAGCTCCGCGCCGCCCCAGCCGA
TCAGTGTCGGCCGCATCGAAGAGCGCACGGTCGCGAGCGCCGCTGCGAAACCCGAACCGAAGGTTCCCGTCAA
TTCCATCGGCGACATCGCTGATCTCTGCGCCAAGAATCGCGACATCAAGCTGAAGACGATGGTGCGCGGTTTC
CTGAGGCTCGTGCATATCGAACCCGGCCGGCTCGACGTCAATCTGCCCGACGACGCACCGAAGACGCTGCTGA
ACGAACTCGCCGTCAAGCTCAAGGAATGGACGGGTATCCATTGGGTCGTGAGCTACAGTCGGGAGCAGGGCGA
GCCGACCCTTGTCGAGGCAGAGCAGCGCGCGCAGGAGCAGCGCGTCAACGATGCGCGCCAGGACCCGGACGTC
GCTGCCATTCTTGCGCGCTTTCCCGGGGCCAGGATCACCGACGTGCGCATCCGCGCGGCGGAAGAGGAGCTCG
AGAGCCTCGCTCCGGCCGCGGCCGAATCCGAGGACGGCGACATCGTCCCGGACGACGACATCGAGTAGATGAC
CATTACCGAGAGCAGGAATGCGGCCGTTGCGGAGATCGAGGAGGTAATCGGGGACTGGGCGGAGGCGCTGCGC
CGGAAGGATGCCGCCCGCGTCCTCGCCCACGGCACGGAAGATTGCCTCGTCTTTTCGCTGGCGCCCCCCCTCA
AGGCTTCCGACGCAGATGCCGGCGGCCTTGAGCAGTGGTTTTCCACGTGGAAAGGGCCGCTCGGCTTCACCCT
CGAGGAGCTCGACGTTTCGGTGAGCGGCGACGTCGCCTTCGCCACTGCGCTGACGCATCTTTCCGGCGAGAAG
CACGATGGCCAGAAGGCGGCGCTCTGGTATCGCCTGACCTTGGGCCTGACACGCACCGACAGGGGATGGAAGA
TCGCCCATCAGCACGAATCCGTCCCCTTCTACATGGACGGAAGCTTCAAGGCGGCGATCGATCTCGATCCCCG
GTCCGAGGTCGACTGGCAAGCGCCGTCGCGCCCGCCCATGCCCGGCGTCATCGCCTATCTCAACGTGGAGGAT
GCAGATGCGGCGGCGGCCTTCTATGTCCGCGCTTTCGGCGCCAAGGAGGAGGACCGCAAACATGCCCAGGATG
GAAAGCGCCTCATCCACTGCCACCTGACGATCAATGGCGGCGCATTGATGCTGAACGATCCTTTCCCGGAATT
CGGCCATCCCTGGAAGCCGCCGGAGGGCTTCGTGCTGCACCTCGTCGTCGACGACGCTGATTTCTGGTGGAAT

CGGGCGGTGGAGGCCGGCGCAGAGGTGACGATGCCGCTGGAGATCGCATTCTGGGGCGACCATTACGGCCAGC
TCCGGGATCCCTTTGGAGTTACCTGGGGGATCGTCGCACCGGTGAAGGAAGGCGGCTGAATGTTCAAGTTCCA
AGCCAGATCGCTGGCGACAAAGTTGATCGCGGTGACGGGAGGCACGATCGCCCTCGTGCTGCTCGCGTCGAAT
TACGTGCTCATCTCCCAGACGCAGGACCGTGTGGAAACGCTGGTCTTCGACGGCGCGAAGACGGAAGCGCGGG
CGATCGCGTCCGACATCGCGGGAAGCGTCGGCGAACTTGCCGCCGCCGCGCGGACCATGTCGGGCGTTCTGGG
GCGCGGCCATGCCGGGCAGTCCACCGACCGCGCAGGCGCCATCAATCTCCTGAAGGCCAATCTGGAGCAGCAT
GGCTTTGCCTTCGGCAGCTGGTTCGCCGAAGAGCCCAAGGCCTATGACGGAAAGGACGTCATCGACAACACGG
AACGTGGCGGCAATGCCGACGGTGCGTTCACGCCCTATTGGTCCAAGGACCGCAACGGAAATATCCAGCTCTC
GACCTTCAAGGCCGATTATGCGGCCGAGTGGTACGGCCTCGCCGCGAAGAGCGGCAAGGGCGCCATCACCCAG
CCATATCTCGCCGAGGGCACCGACGTCCCGACCACGATGACGTCGATCGCCTACCCCGTCATGTCGAACGGCA
GGATGATCGGCGTCTCGGGTGTCGATATCTCGCTCGCCGCACTCGCCGACCGTCTCTCGGCGGTCAAGCCTTT
CGGCTCCGGCCGGGTCTACCTGCTGTCGCAGAGCGGCAAGTGGCTCGCCGCACCTATCCCCGAACTGCTGATG
AAGGAATACGACGGCGAAGGCGTTGAGTCCGTGAAGGATGCCCTTTCCACCGGCACGCCCCGCATGATCGAGA
ACCTGACTTACGACGGCAACGAGCCTTTCGACCGAGTCGTCTATCCTTTCAGCCTGCCCGACGTGAACGCGCA
GTGGCTGGTTCTGGTCGATGTTCCGCGCTCAGCCATCAATGCGCCGGTTCGCGATCAGACCTATATGATGATC
GTCGGCGGCCTGGTGGTCCTCGGCGCCGTGCTCGCCGGCCTCTATTTCGCCGTTCGCCGCTTCGTCCAGAAGC
CGCTTGCCGGACTCGTGGGCGACGTCGAGACGCTCAGCAACGGAGAATATACCCGGCCGATTTCCGGTCAGGA
ACGCTCCGACGAGACAGGCGCCGTCGCCAGGTCGCTCGAGGGCTTCCGTCACCAGCTTGCCGACAACAGGCGG
CTCGAAAGCGAGGCCCGCCACGAGAGAGAACAGGCCGAACTGGAACGCGGACGTTCGGAAAACGAACGCACCG
AGGCCAGCGCGCTGCAGCGCGACATCGTCGCACGCCTTGGCAAGGCCCTGTCGCATCTCTCCGCCGGTGACCT
CGCCTTCCGCCTTACCGGCGACTTCCCCGGCGAATACGCGCAGCTGAAGCGCGATTTCAACGCGACGATGGAG
AGCCTCGAAGAGACGATCCGCACCGTCAACCACTCCGTCGTCAATATCGGCAGTGGCACCAGCGAGATCTCGG
GCGCCGCCAACGACCTGTCGCATCGGACTGAACAGCAGGCAGCAAGCCTCGAGGAGACCGCGGCCGCCCTTGA
CGAACTGACCTCGCAAGTGAACGCCAGCGCCGAGAACGCCAAGGTCGCCGCGAAGTCCGTCGAGGTCGCAAGC
AGCGACGCGGAACAGTCCGGCGAGGTGGTGCAGAAGGCGATCGCCGCCATGCAGGGTATCGAGCAGTCGTCGC
ATGAAGTCAGCCGGATCATCGGCGTCATCGATGAAATCGCCTTTCAGACCAACCTTCTGGCGCTCAATGCCGG
GGTCGAAGCCGCCCGCGCCGGCGACGCCGGCAAGGGCTTCGCGGTCGTCGCACAGGAAGTCCGCGAACTCGCG
CAGCGCTCCGCCAATGCCGCAAAGGAAATCAAGACGCTGATCAATACATCGGCGGGCCAGGTTCGCGAGGGCG
TCGACCTCGTCGGCAAGGCCGGCGGCGCGCTCGAGAAGATCGCCGAGCAGGTGGTGCAGATCAACGGGCTCAT
CCGCCAGATCTCCAGCTCCGCCTCGGAACAGGCGGTCGGCCTCAAGGAGATCAATTCCGCCGTCAATCAGATG
GACCAGGTGACACAGCAGAACGCCGCCATGGTGGAAGAGACGACTGCCGCCAGCATGGCGCTGAACGACGAGG
CCCGCGCCCTCAGCGCGCTGGTCGCCCGTTTCCAGATCGCCCCGCAGGCTGCCCAGGCCCAAGCCTCGGCCGA
AATGCTGCGCGGTACGGCCGAGCGGATGCGTGCGGCTGCTCCCGCGGAAAATCGTCCGGCACAGGCGCCGCGC
AGTGCCGCCTACTCCAATTCGACTCAAAGAGTGCTTGCCAAGACATCCGGCGCCAATGCGCTCGCGCAGGACA
ATTGGGAAGAATTTTGAATGACAGCAGATACGACCATAGATCCCGCGGCGCCAAGAACCTTCCTGTTCTTGCA
GGGACCGGCGTCGCCGGTCTTCGCCAAGGTCGCCGGGCATCTCGAAGTGCTGGGTCATTGCTGTCTGCGCATC
AACCTCAATCCCGGCGACCAGATATTCTGGCGGCGCCGGGGCGGCCATAACTACCGTGGCTCGTTGGCGCGGT
GGCCGGCCTATGTGGAGGCCTTCATCCGCCGCCACGCCGTCACCGACCTCGTCCTGCTCGGCGAAGAGCGCTC
CTATCACCGGATCGCCACAGCTGCCGCGCGAAAAGCCGGCGTCCGAGTCTTTGTGGTCGAAATGGGTTATCTC
AGGCCGGACTGGTTGACGCTCGAGCGCGGCGGCATGTCGTCCAATTCGCACTTTCCGGTCGATCCCGCGCAAA
TCCTGCGAGCGGCAACGGGATTGCCCGAGCCCGACTGGCAACGGCGCTACCGGCAGAGCTTTCTTGCGGAGGC
GGCATACGACCTCCTCTACAACCTGCCGAACGTGTTTTTCTGCTTCCTCTTTCCCGGCTACCGCCGGCACGGC
ATCTTTCATCCGCTCGCCGAATATGCCGGGTGGATCCGGCGGCTGGCGGCCGGCAAAGGCGAGCAGCGGCGCG
CGGATGCGTTGATCCGCTCGCTTTCGGCGAAAAATGCGCCGTACTTCGTCTATCCGCTTCAACTTGAAACGGA
CTATCAATTGCGTGCGCATTCACCCTTCGGCAGCCAGCGGGAAGCGATCGACGGCATCCTTGCCTCTTTCGCG
CGCCGTGCGCCGGCAGGAACCAGACTCGCCGTCAAGGTGCACCCGCTCGACAACAGCCTGATCTCCTGGCGCA
GGATCGTCGCGGACAAGGCCGCCTCACTCGGTGTCGGCGACAGGGTCCTCTATCTCGACGGCGGCAATCTCGA
TTCGCTCACGGAGCACAGTTGCGGAATGGTAACGGTCAACTCGACCGCCGGACTGCATGCTCTGAAGCAGGGC
AAGCCCGTGAAGGTCCTGGGAAAGGCCGTCTTCGACATTGCCGGTCTCACCGACCAGCAGCCGCTCGACGCGT
TCTGGACGGCGCCCGAGGCCCCGGATCCGGTGCTGAGCGGGGCAATGTTCAGGCTGATGGCCGCTTCGATCCA
GGTGCGCGGGAACTTCTATTCCCGTGCGGGTACGGATGCCGGCGCCAAGGCGATCGCCGAGCGGCTGCACCGC
AACGACGTCAATGAACCGGGCGCCTTCGTCGACCCGCCACCCCGGCAAAAGCCGGTGAGACTCAGCGAAGATC
AGGCGACGCCGGCGCGCAGCAGATCGTGATTGCAGACCGGTCTTTATGTCGCGCTTTCGTCGCAGATGGCGCT
TGAAAAGCGCCTGAACACGCTTGCCGACAATATTGCCAACTCCAACACGGTCGGCTTCCGCGCGACGGAAGTG
AAGTTCAACCAGGTGCTGGGCGACACCAAGCCGACAAAGGTCTCCTATGTGTCGGAGGGCGAGGAATTCCTGA
GCACCAAAACCGGCGCGCTCGCGCGCACCGGCTCCGCACTCGATTTCGCCATCAAGGGTGACGCCTGGTTCTC
GATCGACACGCCCGGCGGCCCGGCGCTGACGCGCGACGGCCGGTTCACCCTGACGGAGACCGGCGAGCTCGTA
ACCATCAAGGGATATCCGGTGCTCGACGCCGGCGGCGCGCCGATCCAGCTGAACGGCGGTGCCGGTGAAATTG

CCGTCGGTGCCGATGGTGCCATTCACCAGAACGGCGTCCAGATCGCCCTGCTCGGGCTTTACGAGGCGGATTT
CAGCAAAGGCTTCATGCGTTACGACAACAGTTCCGTTATGCCGGCCGCCCAGCCGGAACCGGTCGTTGACCGG
TTCGATGTCGGCGTGATGCAGGGCTTCCTGGAGGAATCGAACGTGAACGCCATACAGGAAATGTCGCAGCTGA
TCATGATCACCCGCGCCTTCGACAACGTCACCGCACTTATGCGCGACAGTGAGGGATCGCTCGATAAGGCCAT
CGAGACGCTCGGCGGCAGCCGTTGAATGCCGGAGACGATCGGCTTTCTGAAGCCAAGGCAGGCCGTCCTCGAT
CGTCGGCGGGAAATCGTCGCCGATCTTGCCGATCTGTTGCCGGAAGGCGGCCTCATCAGCGACGAGCGCGGCC
TCAAGCCCTTCGAGACGGATGCGTTTATCGCCTATCGGCGGATGCCGCTCGCGGTCGTTCTTCCCGAGACGAC
GGAGCACGTGGCCGCGGTGCTCAAATATTGCAGCCGCTACGGCATTCCGATCGTGCCGCGGGGCGCCGGCACC
TCGCTCTCCGGCGGCGCCATTCCTCAGGAGGACGCGATCGTCGTCGGTCTGTCGAAGATGTCGCGCACCCTGG
ATATCGACCTGTTCAACCGCACGGCGACGGTTCAGGCGGGCGTGACCAATCTCAATATTTCCGATGCGGTTTC
GGCCGACGGCTTCTTCTATGCCCCGGATCCGAGTTCGCAGCTCGCCTGCACCATAGGCGGGAACATCGGGATG
AATTCCGGCGGCGCGCACTGTCTGAAGTACGGGGTTACGACCAACAACCTTCTGGGCGTCAAGATGGTGCTTT
TCGACGGCACCGTGATCGAACTCGGCGGCAAGGCGCTCGACGCGCCGGGATACGACCTGCTTGGGCTCGTCTG
CGGTTCGGAGGGGCAGCTCGGCATCGTCACGGAAGCGACGGTGCGGCTGATTGCGAAGCCCGAAGGTGCGCGG
CCCGTGCTCTTCGGATTTGCCTCTTCCGAGTCCGCCGGCAGCTGCGTCGCCGATATCATTGGATCGGGCATCA
TCCCCGTCGCAATCGAATTCATGGACAGGCCGGCGATCGAGATCTGCGAGGCCTTTGCCCAGGCCGGCTATCC
GCTCGACGTCGAAGCATTGCTGATCGTCGAAGTGGAAGGCTCCGAGGCTGAGATGGATGCGACGCTCGCCGGC
ATCATCGAGATCGCCCGCCGCCACGGCGTGATGACGATCAGGGAATCGCAATCGGCTCTGGAAGCGGCACTGA
TCTGGAAGGGGCGTAAGTCGGCCTTCGGCGCAACCGGCCGGATTGCCGACTATATCTGCATGGACGGAACGGT
GCCGCTGAGCCAGCTCTCCCATGTCTTGCGCCGGACCGGGGAGATCGTCGCCGGCTACGGCCTGCGGGTAGCG
AATGTCTTCCATGCCGGCGATGGCAACATGCATCCGCTGATCCTCTACAACATCAACGATCCGGAGGAAGCTG
CCCGCGCGGAAGCTGCCGGCAACGACATACTGAAGCTTTGCGTCGAGGCGGGCGGCTGCCTGACCGGGGAACA
CGGCGTCGGCATCGAGAAACGCGATCTGATGCTGCACCAGTACAGCAGGGCCGATCTCGGCCAGCAGATGGCG
GCGCGCGCCGCCTTCGATCCGCAATGGCTTATGAACCCGTCGAAGGTTTTTCCGCTCGAAGGACGGCCCGCGG
CATGAATGAAAGAGAGACTTATATCTTCCGTTGCCGCTGGCGCGCTTCTCGTGGGCGTTGCCGTTGCCCCGAT
GAGCTTCGCACAGCAAACCACGACGCCGCCGGCTACGGCACCTGAAACCCAGACCATGGAACCGGCTCCGGCT
CCGGCCCCGGCTACACCGCCGGCCGCCGACGAGCAGGCGCAGACCCCGGATACGGCAGACAAGACGACCGATA
TGGCGGCTGGTGACGCCACCTACCTGACCGAGCAGGCGGAGAATCAAGTTTCCGCCAACACCTATATCGGCCA
GTCCGTCTACAATGCCAGCGATGAAAGCATCGGCGAGATCAACGACCTGATCATCGAGAAGGAAGGCGGTATC
GCTGCAGCTGTCGTCGGAGTTGGCGGCTTCCTCGGTATCGGGGAAAAGAACGTCGCAGTTCCGTTCGACAAGA
TCGCAGTCGCCGACCAGCCCGACTCGGATGACATCAAGCTGACGACCACCGAAACGGCCGACTCTCTGAAGGC
CGCACCGGAGTTCAAGACGAAATCGCAGCTGATGGCCGAGCGTAACGCCGACCAGCCGGTCGATACGTCGACG
ACTTCCGCAACGGGTACAACCCCGGCACCGGCGCCTGCGGAACCTGCCCCTCAGCAGTAAATGACCCTTCCGA
CAGAAATGACCTACGTCGATCTGCCTGGCCCCGGAGGGCCGGAGAATATGGTGCTGGCGCGAGGGCCGCTGCC
TGAGGTCCGGCCCGGCGACATTCTGATCCGCGTCGAAGCTTTCGGCATCAATCGGCCGGACGTCCTGCAGCGC
AAGGGAGACTACCCGCTGCCCGCTGGGGCGAGCCCCATCCTCGGGCTCGAAGCTGCGGGCGAAGTGGTTGCTC
TCGGCGAGGGAGCGACCGGCTTCTCGACCGGCGACAAGGTCTGCGCTCTTGCCAATGGCGGCGCTTACGCGGA
ATATTGCGCGGTTCCCGCCACGCAGGCCCTTCGCTGGCCCAAGGGCTACGATGCCGCGAAGGCGGCAGCCCTG
CCGGAGACCTTCTTCACCGTCTGGGCCAACGTCTTCGACATGGCCCGCCTGAAGTCCGGCGAAACATTCCTCG
TCCATGGAGGATCGAGCGGTATCGGTACGACCGCGATCCAGCTTGCGAGGGCCTTCGGTGCGGAGGTCTTCGC
GACCGCCGGCAACGCTGCGAAGTGCGAGGCCTGCGAAGCACTCGGGGCGAAACGTGCGATCAATTATCGCGAG
GAGGATTTCAAGGCTGTCGTGCTGCAAGAGACCGGCGGCCGCGGGGTCGACGTCATTCTCGACATGGTGGGCG
GGCGCTACTTCGATCGCAACATTGGCAGCCTCGCCAAGGACGGGCGCCTGTCGATCATCGCTTTCCTTGGCGG
CGCCCGGGTGGACGGCGCCAACATTGCGCCAATTCTCATGAAGCGCCTGCAGGTAATGGGCTCGCTGCTCCGC
CCGCGCACCGCCGCCGAGAAGCAGGCGATCCGCGACGGGCTTGCGGAAAAGGTCTGGCCGCTGCTGGAGGGGG
GCGACGTTGCACCGGTCATCCAGGCGGTCCTGCCCTTCGCCGAGATTGCCGGCGCCCATCGGCTGATGGAGGA
GGGCGATCATATCGGCAAGATCGTGATGCGGATCGGGTAGATGACCGAGCAACAAGAACTCCCCGAACGCGAG
AGCATGGAATTCGACGTCGTGATCGTCGGGGCGGGACCGGCGGGGCTTGCCGCGGCGATCCGGCTGAAGCAGG
TCAGTCCGGAACTCTCGGTCGTGGTGCTGGAGAAGGGCGCCGAAGTGGGCGCACATATCCTCTCCGGCGCCGT
GGTCGACCCGATCGGCATCGACCGGCTCTTGCCGGGCTGGCGCGACGAACCGGACCACCCGTTCAAGACGGAA
GTCACCGACGATCAGTTCCTCTTTCTCGGCCCCGCAGGCTCTGTCCGCCTTCCCAATTTCCTGATGCCGCCGC
TGATGAACAATCACGGCAACTACGTGGTATCGCTGGGCAATGTCTGTCGCTGGCTCGCCACCCAGGCGGAAGC
GCTCGGCGTCGAGATCTATCCGGGTTTTGCCGCAACGGAAGTGCTCTACAATGACGAGGGCGCGGTGATCGGC
GTTGCCACCGGCGACATGGGCATCGAGCGGAACGGCGAGCCGGGGCCGAACTTCGCCCGCGGCATGGAGCTGC
TCGGCAAGTACACCCTCATCGGCGAGGGCGTGCGTGGCTCGCTCGCCAAGCAGCTGATTGCCAAATACAAGCT
CGACGCGGGCTGCGACGTGCCGAAATTCGGCATCGGCCTGAAGGAGCTCTGGGAGGTGAAACCGGAAAATCAT
AAGCCGGGCCTCGTGCAGCATTCCTTCGGCTGGCCGCTCGGCATGAAGACCGGCGGCGGCTCCTTCCTCTATC
ACCTTGAGGACAATCTGGTGGCCGTCGGTTTCGTCGTCCACCTCAACTACAAGAACCCGTATCTCTATCCGTT

CGAGGAATTCCAGCGCTTCAAGACGCATCCGGCGATCCGCGGCACTTTCGAGGGTGGAAAAAGGCTCTCCTAC
GGCGCCCGCGCCATCACCGAAGGCGGCTACCAGTCGGTGCCGAAGCTGTCTTTCCCCGGCGGGGCGCTGATCG
GCTGCTCGGCCGGCTTCGTCAACGTGCCGCGCATCAAGGGCAGCCACAATGCGGTGCTGTCCGGCATTCTCGC
CGCGGAGAAGCTTGCGGCGGCGATCGCCGACGGCCGCGCCAATGACGAGCCGATCGAGATCGAGCGCGGCTGG
CGCGAGAGCGCTATCGGCCAGGACCTGAAGCGGGTCCGGAACGTCAAGCCGCTGTGGTCGAAATTCGGCACCG
CCGTTGGGGTGGCGCTCGGCGGTCTCGACATGTGGACCAACCAGCTCTTCGGCTTTTCCTTCTTCGGGACGCT
GAAGCACGGCAAGACCGATGCGCAATCGCTCGAGCCGGCCGCCAAGCACGAGAGGATCGACTATCCGAAACCG
GACGGGGTGCTGACCTTCGACCGGCTCTCCTCGGTGTTCCTGTCGAACACCAATCACGAGGAGGACCAGCCGG
TGCATCTGCAGGTGAAGGACTGGGACCTGCAGAAGCGCTCGGAATACGAGGTCTATGCCGGCCCATCTTCGCG
CTACTGTCCGGCCGGCGTCTATGAATGGGTGGAGAAGGACGGCCAGCAGACCTTCGTCATCAACGCCCAGAAC
TGCGTCCACTGCAAGACCTGCGACATCAAGGACCCGAACCAGAACATCAACTGGGTGCCGCCACAAGGGGGCG
AAGGGCCGGTCTATCCGAACATGTGAATGAGAACGATGATTGCGATCGCCTGTGCGGCAGGAATGGCGGGTGC
CGCTTCGGCACAACAGTCCTCGCAGACGGCGACGGCCGAGTTCGTGGGCAAGGACGGCACGGACATCGGCCGG
GCCACTTTGACGGCAGGCGGCAAGGGCGTGCTGATCGAGATGGAGATAAGCGGCCTGCCGAAGGATACCTGGG
TGGCTTTTCACGCTCACGAAATGGGGCGCTGCGATCCCAAGGACGGGTTCGAGTCGGCGGGAAAGCATTTTGC
CGGCGAAGATGAGAATGCCGAGCACGGCTTCCTCGCTTCCAATGGCCCGCATGCCGGCGACATGCCGAACCAG
TATGTCGGTGCCGATGGAACCTTGCGCGCCCAGGTCTTCAACAGCTTCGTGTCCCTCGACGACAGGGCTACCG
CCCTTCGCGGCCGGGCCCTGGTGATCCATGTGCATTCGGACGACAATCGCAGCCAGCCCTCCGGCGATGCCGG
CGACCGGCTGGCCTGCGCGGTGGTGAAGTAGATGGCCGCCCCCGTCACCGTGCGCGTCCAGCGCGACGACTTC
GACATCGCCGCCGAAGTCTCGGCGCTTTGCCGTGCGCGGACTGACATCGGTGCGGTGGTCACGTTTTCGGGGC
TGTGCCGGGACGAGGCCGGAGCCCTCAGTGCGCTCGAGCTCGAGCATTATCCCGGCATGGCAGAGGCGGAGAT
CGAACGGATATGCCACGAAGCGGTCGAGCGCTTCGGCCTGCAGGCTGCAACCGCCATCCATCGCTTCGGCCGC
ATGGAGCCGGGAGCGAATATCGTCCTGGTGGTAACCGCCTCGCCGCACCGGCAGGCGGCATTCGATGGCGCGA
ATTTCATCATGGATTTCCTGAAGACCTCGGCCCCCTTCTGGAAGAAGGAACATCACGCCGACGGCAGCGCAGG
CGATTGGGTCAGCGCGAAGGATGCGGACGACGCGGCACGCGATCGCTGGACGCGCCGCTGAATGGTCGAATCT
GAAGCGCAGGGACGTCTGCCCGGTATCCGCCCGGTCTCCGTTGTGGATATCGGCTCCAACTCCATTCGTCTCG
TCGTCTATGAAGGGCTGAGCCGTTCGCCGGCGATGCTTTTCAACGAGAAGGTGATGTGCGGCCTCGGCAAGGG
CATCGATGCGACCGGCCGAATGGAGGAGGAAAGCGTGGCGAGGGCGCTGAGGGCGCTGCACCGCTTTCGTGCG
CTCTCCGACCAGGCGCGCGCGACCGAGATGTATGTGCTCGCGACCGCTGCGGCGCGGGAGGCCACCAACGGAG
CGGCCTTCATCGAAAGGGCGGAAGCCATCCTCGGGCAAAGGGTGAAGATCCTGAGCGGTGAGGAGGAAGCCTA
TTATTCGGCGATGGGCATCGTCAGCAGCTACCACGAGCCGGATGGCGTCGTCGGCGATCTCGGCGGCGGCTCG
CTCGAGCTTGTCGATGTAAACGGCAAGCGGATCGGCAAAGGCATAACGCTGCCGCTGGGCGGCATTCGCCTCT
TCGAACATTCGAGCGGCTCGCTCAGCAAGGCGCGGACCTGGGTACGCAAGTTCATGAAAAATGCCGAGGTCCT
GAAGAAGGGGACCGGACGCACGTTCTACGCCGTGGGCGGAACCTGGCGAGCGATCGCCAAGCTGCACATGGAG
ATGCACAATTACCCGCTGCACATGATGCAGGGTTACGAAATCTCCTATGACGAAGCGCTGGCGATCCTCGCCG
AAGTGATCGAGCCGAAGAATATCAAGCCCTCCGCCTATGCGACGATTTCCAAGAGCCGCCGGAGCCTGCTGCC
CTACGGGGCGGTGACGATGCAGGAGGCAATCTCCATCATGCAACCCGAGCGCATCTCCTTTTCCGCGCTGGGC
GTCCGCGAGGGCTATCTCTTTTCGCTGCTTTCCGAGGAGGAGCGCCGGCAGGACCCGCTGCTGACCGCTGCCA
ACGAACTCGCCATCCTTCGCGCCCGCTCGCCCGAGCATGCGCGCGAGCTCGCCGAATGGACCGGCCGCCTGGT
GCCGTTCTTCGGCGTCGAGGAGACGGAAGAGGAGAGGCGCTACCGTCAGGCCGCCTGTTTGTTGGCGGATATC
AGCTGGCGGGCGCATCCCGACTATCGCGGTCTGCAGGCGCTGAACATCATTGCGCACTCGGCCTTCACGGGTA
TCACCCATGCGGGGCGGGCCTATATCGCGCTCGCGAATTACTATCGCTTCGAAGGGCTCAACGACGACGGCGC
CACGAGCCCGCTCGCCGGCATCACGACGCCGCGCCTGCACGAACTGGCCAAGCTCCTGGGCGGGCTGTTGCGC
GTCGCCTATCTCTTTTCGGCCTCCATGCCGGGTGTGGCGCGCCACCTCACGCTCCGGCGATCTTCGAAGCCCG
ACATCGATCTCGAATTCGTCGTCCCGGCAGCCTATGCCGACTTCGAGGGCGAACGCCTCGACGGCCGGCTCCA
GCAGCTCGGCAAGCTTACCGGCAAGAAGCTCGCCTTCTCCTTCGAGCGCTGAATGATACATACGCGCAAAGTC
CGCCTGATGCTGGCCAGCGCCGCCCTCCTTACCCTCGCCGGACCGGCCATGGCTCTGGACGGCGCGGACATGA
TGCAAAAGCTCAATGCAGCGACGTCGCCCAACGGCACTTCGGTGACCTACGACAAGGCGGAGGCCGACGGCGA
CGTGGTGACGGTCACCGGCATGAAGCTGCAGGTGGTGGCGAAGCCCGGCACGTCCATCGACATAGGCGAAGTG
ACGTTCGAAGGCGTCGAGGAAAAAGGCGGCGGCTACTATGCCGAGACCGTCTCCTTCCCGGATATCGCTGTCA
GCGACAAGGATGCAAGCTTCTCGGTCAAGGACATCTCGATCGGCGGCCTGTCCATTCCGGGCAATGCGAGCGG
TACCACGATCGATGACATCCTGCTTTATGAAACGGCCGGCACCGGCCCCATGACCGTAAACGTCAAAGGCAAG
GACGTCTTCACCGCCGAGAGTACGGAAACCAATCTTACGCGGCAGGAAAACGATGCGGGCTTCGACTTCGACG
CGACGCTTGCCGGAATCAAGGCGGATCTGTCGCAGATCGAGGATGCCAAGGCAAAGGAGGCGGTCGAGAAGCT
TGGGCTGACGACGCTCGACGGCGGAGTCACGATGAAGGGCAGCTGGGAATTGCAAACCGGCAACCTTGCTCTC
GACGAATACGCGCTGGACTTCAAGAATGTCGGCCGACTGAATATCGCGATGGACTTATCCGGCTATACGCTGC
AATTCATGAAGTCGTTGCAGGAGGCAGCCAAAGCGGCTGAGGCAAACCCGAACAGGAAGGAAGCCGACCAGGC
GATGGCGCTGGCGATGCTCGGCCTCATGCAGCAGCTTACCTTCAACAGCGCCTCGATCCGCTTCGACGATGCA

TCGATCACGAAAAAGGCGCTCGACTACGCCGGCAGCCAGCAGGGCGTCACCGGCGAACAGCTGGCGCAATCGC
TGAAGGGGCTCGTGCCGATCATGATGGCGCAGCTCAATCTCCCGGAACTGCAGAACCAGGTCTCCAGCGCCGT
CAACGCCTTTCTCGACAATCCGAAGAGCCTGACGATCAGCGCTGAGCCGGAAAAACCCGTGCCGTTCCCGATG
ATCATGGGTGCTGCGATGGGGGCTCCCAACACCGTCCCGTCCGTGCTGGGCGTCAAGGTGACGGCGAACGACT
GAATGAACAGCATTGCGAAAATCTCCGTCATCGCCACCCTCTCCGCCCTTACCTTCGCCGGAATCTCCCAAGC
GGCGAGCGTCGAAATGAACTGGCCGGACCGGATCGACCAGACCGTGCGCACCTTCAAGGGCGACAATTTCCGC
GTTGTCGATGTCGATACGTTGAGCCGCCAGAACCAGACCCGTATGCGAGTAGACAAGGCAACACCGGGGCAGC
GTGCCGCATTGCAAGCCGCTGTCGAAGCCAACAGACCGCTGCTCGCTTCGCTTAGGGCACGCAATGTCGAACT
GGACAACGTCGCCGCGGCGAAGCAGGCGGCCGATGGCGGACTGACGATCTACCTCCGCTGAATGATCCGTTCT
GTCGTGCGCGGCTTCGGGGCAGCACTGCCGAAGCGAGTGATGACCAATAAGGAAATCGAATCCAGGGTCGACA
CGTCCGACGAATGGATCGTGCAGCGGACCGGCATCCGCCAGCGCTTTATCGCAGGCGAGGGCGAGACGAGCGC
CTCTCTCGGCGAGGCGGCGGCGCGGGCGGCGCTCGAGAGAGCCGGGCTGACGCCGGACGACGTCGACCTGATC
ATCGTCGCGACCTCCACTCCGGACAACACCTTCCCTGCAACCGCAGTCAACATCCAGAACCGCCTCGGCATGC
GGCACGGTGCGGCATTCGACATGCAGGCGGTCTGTTCCGGCTTCGTCTACGCGGTGGCGACGGCCGACGCCTA
TATTCGCGGCGGCCTCTCCAAACGCGCTCTTGTCATCGGCGCAGAGACCTTCTCGCGGCTTCTCGACTGGACC
GACCGGACGACGTGTGTGCTGTTCGGCGACGGCGCCGGCGCAATCGTTCTGGAGGCGCAGGAGGCGGCCGGGA
CCAAGGCTGATCGGGGCGTTCTGACGGCGCAATTGCGCTCCGACGGCGCCCACCGCGACAAGCTCTATGTCGA
CGGCGGGCCGTCGACGACCGGCACCGTCGGCCATCTCCGGATGGAAGGGCGCGAAGTCTTCAAGCACGCGGTC
GGCATGATCACCGACGTGATCGAGGCGGCTTTCGAGGCAACCGGAACGACCGCGGACGACATCGATTGGCTGG
TTCCGCACCAGGCCAATCGCCGCATTATTGACGGCTCGGCCAAGAAGCTGGGCATCCCTCTCGAAAAGGTTGT
GGTTACCGTCGACCTGCATGGCAACACCTCCGCGGCATCGATTCCGCTTGCCCTCGATGCCGCCGCCTCTGAC
GGCCGCATCAAGAAGGGTGATCTCGTAATGCTCGAAGCGATGGGCGGGGGCTTCACCTGGGGCTCCGTTCTTC
TTCGCTGGTAAATGACCACGAGTCGCGAGCCGCCCGACGCCCCTCTGCGCCTGCTCGAGGTACTGGAACCCGG
CGGCGGAGGGTCCGGACGCCACTTTCTCGATCTTTGCCGCGGCATGCATGCGCGCGGCCACCATGTCGAAGCG
ATCTATTCTCCCGTTCGGGCCGAAGACGGCTTCGTGCGCGAACTCAAGGCGCTCGGCCTTCCCGCCATTCATG
CGGTCGGCATGCGCCGGGCGCCCGGCCCCTCCGACTGGTCCTGCTTACGCGCCATAAATCGCATCATCCGCAC
TGCCGGGCCGTTCGACGTCATTCACGGCCACAGTTCCAAGGCCGGAGCCCTGACGCGCCTGCGCCTCCCGGGA
AGGCACGTGCCGCGCGTCTACACGCCGCATGCCTTCAGGACGATGGACCCGACGCTCGGGCGCGGCGGCCGAC
TGATTTACGGGGCGATCGAAACACTTCTCGCCCGCTTCTTCACCGACCATCTCGTCACGGTCTCCGGTGACGA
ATTCGCCCATGCGCTGTCGATCGGCATTTCGGGAAAGGGCATGTCCGTTATCGTCAACGGCGTCGACACCCCT
TCGCCCGATATGGCGCAGACGGTGCGCGCCAGCTTCGGCATTCCGGCGGACGCCTTCGTTTTCGGCTTCATCG
GCCGGCTCTCGGCACAAAAGGCGCCCGAGCGTCTCCTCAATGCCTTCGGCAAGGCCGCGTCGGCGGTCAGGAA
CAGTCACGTCGTCATGATCGGATCGGGAGAATTGGAGGAAGAGGTCCGCGCCGCGATCGCCGCAAGCGGCTTG
CAGAACCGCATCCATCTGACCTCGGCCTTTACGGGACCTCAGGCGGTACCCGCATTCGATCTCCTCGTCATGC
CGAGCCGCTACGAGGCAATGTCCTATGTGATGCTCGAAGCGGCAGCCGCCGGCCGGCCGATCATCATTTCCGA
CGTCGGCGGAGCCGGAACGGCCGTCGATCACGGAGAGAACGGCTACATCGTTCCGAACAGCGAGGACGTCTCA
CCGCTTGCAAGAGCGATGATAGCGGCGGCCGACCCGGATATGTTTCGGCACCTTGCCGGCGCCGCCGCGGCGC
GCCGGGACCGTTTTACACTGAAACGTATGCTCGACGAGACGGAAGAGGTTTACCGACGAATGGCAGCGCAGCG
CCGCCGACAGCAGTCAGTAAGATCGAATCAAGAATACTGGAAACCGACGGAAATGATGAAAGCCGCTCAATCA
CTGCATGGGGGCTAAATGAACCACGACGCCTACCCGGACTATTATCTTGCCGACATCCTGCGCGAGACGAAGA
CCATCGCGCTCGTCGGCGCCTCGCCGAAGCCGGAACGGCCGAGCTATCGCGTCATGGCCTTCCTTCTCCGCAA
GGGATACCGCGTCATTCCGGTCAATCCCGGCCATGCGGGCGGCACGATCCTCGACCAGCCGGTGGTAGCGCGC
CTCGCGGATATAGGGGAGCCGATCGACATGGTGGACGTCTTCCGTGCGGCTTCGGCCTTGCCCGCGCTCGTCG
ACGAGATCCTGGCGCTGCCGGCCCTGCCCAAGGTCATCTGGGGACAACTGTCCGTCCGCGACGACGCAGCTGC
CGCCAAGGCTGAGGCCGCAGGCATCAAGGTCGTCATGGACCGTTGCCCGGCAATCGAATATCCGCGACTGGTC
GGATGAATGTCCATCGCCCCCGGTATCACCATCGACGGCCCCGCCCGGCGCGTCTCCCTTGACGTCCGCAACC
CCCGTTTCTTCCGCAATCCGCTGCCGGCCTACGCGGCGCTCCATGCGCAATGTCCGGCCTTTTTCTGGGAAGA
GCCGCAGCAGTGGTTCTTTGCGGGCTACGAGCAGGTGAACAGCCTGTTGCGCGACCGTCGCTTCGGCCGACAG
ATCCTGCATGTCGCGACCCGGGAAGAACTCGGCATGCCGGAGCCGAAACCGCATCTGAAAGATTTCGATGCAC
TCGAGGCGCACTCGCTGCTCGAACTGGAGCCTCCGGCCCATACGCGGCTGCGCACGCTCGTCAACCGCGCCTT
CGTCTCCCGTCAGATCGAAGAGCTCAGGCCGGAAATCGAGGCGCTCTCGCATGCGGTGATCGACGGCTTCGAG
AAGGATGGCGAGACCGAGCTGTTGAAGACCTATGCCGAGACGATCCCGGTAACCATCATCGCGCGGATGCTCG
GCATTCCGGTCGAAGCGGCGCCGCGTCTGCTCGACTGGTCGCACCGCATGGTGAAGATGTACGTGTTCAATCC
CAGCCTGGAAACGGAGTTCGACGCCAACAATGCATCGGCGGAGTTCGCCGACTACCTGAAAGGGATCATAGCC
GAGAAGCGGACAAACCCCGCCGACGACCTGCTCACGCATATGATCACCTCGGAGAAAGACGGCGAGCGCCTGT
CGGACGCGGAGCTGATTTCGACCACGGTCCTCCTTCTGAATGCGGGCCACGAGGCGACGGTTCATCAGATCGG
CAATGCCGTGCGCACGATCCTGCAGTCCGGCCTCTCGCCCGCCGAACTTTTCTCGGACGAGAAGGCGACGGAG
CGCACCGTGGAGGAGTGTCTGCGCTTCGCCGCGCCCTTGCACATCTTCCAGCGCTACGCCCTCATGGATATCG

AGCTGGAGAACGGCATCGCCCTCAGGAAAGGGGACAAGATCGGCCTGATGCTGGGGGCGGCGAACGTCGATCC
GCGCAAATTCTCCTCTCCCGATACGTTCAGGCCGGACCGCAACGAAGGCGCCAATGTTTCGTTCGGCGCCGGC
CTGCACTTCTGCATCGGCGCACCGCTGGCACGGCTGGAGTTGCAGATTTCGCTGCCGATCCTGTTCCGACGGC
TTCCCGGGATGCGGCTCAAGAACGAGCCGCCGGTCAAGGACGCCTTCCACTTTCATGGTCTGGAGCGGCTCGA
TCTGGTCTGGTGAATGAACCATATCCTCCTCGTCGGTGCCGGCGGCGCCCTCGGCTCCGTCCTGCGCTACCTC
GTCGGCCTCTGGATGCTGCAGCGCGCCGGTCCCGCCTTTCCCTGGGGGACGCTGTTCGTGAACGTCACTGGCT
CCTTTCTGATCGGTTTTCTGGCGGAGTTCATCATGCACAAGATGGGTGCCTCGCCGGAAATGCGCGTGTTCCT
GATCACCGGCGTTCTGGGGGGCTACACGACGTTCTCGGCCTTTTCGCTCGACGCGATCGCCCTTCTCGAACAC
GGGCAGACGATGAGTGGCCTCGCCTATATCGTGGCGAGCGTCGGTCTTTCGATGCTTGCCGTGTTCGCCGGGC
TGGCCCTGATGCGCGCAATGGTCTAAATGAATGCCTTTCGTGCCAAGCCGCTGAAGTTTCCATGGCCGCCTCT
GCTCTATGGCGCGGCCGTCCTGATCGCGCTGGCCCTGGGCCACCTTTTCCCGATTCCGGTGGCGCACGAGCAC
AGTTGGATCCTCTCGCTGGTGGGCTACGGCCTCGTGGTGCTGGCGATCACCATCGATCTCTGGGCCGTCAAAA
CCCTCCTTGACCGCCATACAGCCGTACTTCCGAACCGGTGCGCCACCTGCCTCGTCACCTGCGGCCCATTTCG
TTTCACCCGCAACCCGATCTATTTCAGCTACACGCTGGCGATGACCGGCCTCGGATTGGCGACGATCAACCCC
TGGTTCTTCATCACGGCAATTGCCGCGGTCGGATTGACCACCCTTTTCGCGATCCGAAAGGAGGAGCGGCACC
TGCTGTCGCGCTTCGGTTTCGAGTTCGAGCGATATTGCCGGCACACTAGCCGTTGGATTTGAATGTCCGAACA
CAGCGCGACGACGACACCTGAAGCGCAAATGCGCACGGAGGCGACCGGAACCAACCGGATGGTTCTCCTGGAT
GCGCTGCGCGGCCTGGCACTCGTCGCCATGGCCCTCTATCACTTTGTCTGGGACCTCGAATTCTTCGGCTATG
TCGCGGCCGGCACCGCCGGGACCGGCGGCTGGCGGATGTTTGCACGCTTGATTGCCAGCAGCTTCCTGTTTCT
TGCCGGTTACAGCCTCGTGCTCGGCCAGCTCCCGAAACTCCGTCCGCGCGCCTTCGCCATACGTTTCGCGAAG
ATCGCCGGCGCAGCCGCGCTGATCAGCATCGGGACCTGGTTCGCCTTCCCTCAGTCGTTCATCTTCTTCGGCA
TTCTCCACGCCATTGCGGCGGCAAGCCTTGTCGGCCTCCTGTTCCTGAAATTGCCGGCCGCCGTTTCCTTTCT
TGCCGCTGCCGTGGCCTTCGCGGCTCCCCTCTATCTGCGCTCGTCGTTTTTCGACATGCCGGCGCTCTGGTGG
GTCGGCCTCTCGGAAACGATCCCGCGCTCGAATGACTACGTTCCTCTGCTTCCCTGGATTGCCCCTTTCCTGC
TCGGCCTCGGCACCGCGAAGCTGCTCCACCCGTTATTTTCCGCCCGCCGCAGCCGAAGCGCCGGGACCCGTAA
GCACCCGTGGATGACGCCTCTCGCCTTTGGCGGCCGTCACAGCCTTCTGATATATCTCATCCACCAGCCGCTG
CTTATCGCGGCAGTCTATCTCTTCTCTCTCGCCGTTCCCGCCCCGGCACCGGATCCGGCACGGGTCTATCTGC
TGAACTGCGAACGGGCCTGCAGCAACGAAAATTCCGGGATCTCCTGCAGCGCCTTTTGCGGTTGCACGCTCGC
CGGCCTGAAGGAACAGAACCTGTTCGACGATCTGAACCTGGGCAAGATCGACGTGAACACGGATGAACGCATT
GCGCGAATCGCCGGTCAGTGCACAATGGATGCACAATCAGGAGAGGCACAGTCAGGGGACTAGATGAAAGCCG
CCCTGATCGTCATGACCATTCTCGGTTGCGACGACAACGTTACCCAATGCCATTACATCTCGACCGTGAACGG
CGACTGGCCGTCGATCGCGCAGTGCGACACGGAATCGCAAAAGGAACTGCCGCGGTTCTCCGACAGGAACTAC
CCCGTCGTGGTGGCCGTGTGCGAGAGCTCAGGGCCGGGCATGACGGCGGAAAGCGAGCCGGTTCCGGAGCTGA
ATGCAACGCTTCCGGCTCAGCCGGGCGTGCAGCAAAACACAACATCAACGGCAGAAGGCGGAGAACCAACCTT
GCCGAGGCGCGCCCTGGCATTGATCAGCGGCGCCGTACCCGACCGGGAGAAACTGAAGGCGATCGTCAGTGCG
CCGGTCCACTACGTCGAAGACGGCTATTCCTGGGTGGCGCGCCGATTCGGCAATTGAATGGCCTCTCTCTTCG
ACCCGATCACGATCGGCGATATCAGCCTCAGCAACCGCGTGGTGATGGCACCTCTGACCCGGAACCGTTCACC
CCAGGCAGTGCCCAATGATCTTAACGTCGCCTATTACGAGCAGCGCGCATCCGCCGGCCTGCTGATCACCGAA
GGGACGGCAATAAGCCACCAGGGCCAGGGCTATGCCGACGTGCCCGGGCTTTACAAACCGGAGGCCCTCGCCG
GCTGGCGCAAGGTCACGGATTCGGTCCACAAAGCCGGTGGCAAGATCGTCGTGCAGATGTGGCATGTCGGGCG
GATATCCCACGACTCGCTCCAGCCGCAGGGCGGCAAACCGGTCGCCCCTTCGGCTATCCGCGCCAAATCGAAG
ACCTACCTCGTCAATCCCGATGGGACGGGCAGCTTTGCCGAAACCTCCGAGCCGAGGGCGCTCGAACCGGCGG
AGATCGCCGGCATCGTCGAGGATTACCGCAAGGCCTCTCGGGCCGCGATCGAAGCCGGATTCGACGGCGTCGA
GATCCATGCTGCCAACGGCTATCTGATCGACCAGTTCCTGCGCTCCGGCTCCAATGAACGCACGGACGCCTAT
GGCGGCTCGATCGAGAACCGCGCCCGCTTTCTCTTCGAGGTGGTCGATGCCGTTACCAAGGAGATCGGCGCTG
GCCGGGTCGGGATCCGCATCTCTCCCGTGACCCCCGCCAACGACGCCTTCGATCCGGACCCGCAGCCGCTCTT
CACCTATGTCGTCGAAGGCCTCGCCCGATATCCGCTCGCCTATGTGCACGTCATCGAAGGCGCCACCGGCGGA
GCGCGTGACCACCAGCAGGGCGATCGCCCCTTCGACTACACAAGCCTCCGTGCCGCCTACGCAGCGGCCGGCG
GCAGGGCGGCCTGGATGACGAACAACGGATACGATCGCGGTCTGGCGCTGAAGACGGTCGAAAGCGGCGAAGC
CGATTTGGTTGCTTTCGGCAAACCCTTCATCGCCAATCCGGATCTCGTTCGTCGTTTGAGAGACAACGTGGCC
CTGAATGCACCGGACCAGGCGACCTTCTATGGCGGTGGCGCCAAGGGTTATACGGATTATCCGTCGCTCGAAA
ACGTCGCCTGAATGCGAGGCCGAGCCCTCAAGCTTTCAGCGTCTCGGCGCCTGGTCGGGGATCTCATGCGATT
TTCGATGAAGGTGCCGCGGGTGAGCGTGCAGCGGCGGATGAATCTCGGGCCGTTGCTCGAGGCCAGAACATCG
CTTGAAAGCAGGCCATCCTGGACGGCGCTCTTCCTAAAAGGATACGGCCTTCTCGCTCAGGAGACGCCCGAGT
TGCGGCGCGCTTACGTCAAATTTCCCAGACCGCAGCTATACGAATATCCGGGAAGCATCGCTTCCATCGCGCA
TGAAAGGGAGTACGAGGGCGAACGAATCGTGCTGCTGACGACGATCAAGAATCCGGAGCGCCGTCCGATCGCG
GAGCTGGGGTCCTTGATGCAGACGGCGCGAACCAGTCCCGTGCTGGAGGTCAAGGAATTCCGGCGCGCCCTGA
AGATCGCCCGCCTCCCCGGTCCGATCAGATGGCTTCTCATGTGGCTCGGATTGAACATCGGCCGGCAAAGGGC

GCGCCGTTTCGGTACATTTCAGCTGTCGGTCTATTCGGGCCTCGGCGCGGAATCGCTCAACCCCCTCACGCCG
TTAACCTCGCTTCTCAATTACGGCCCGATCGACAACGATGGCTCGGTCACCGTGCGCATCCACTATGACCACC
GGGTCATGGATGGCGCCAACGTCGCGCGTGCCCTCGAGCGGTTCGAGAGGATATTGAACGGCGAAGTCACCGC
GGAGCTGGAAGGGCTTGCATCCGGGCGCGACGTAACCGCTGCGGCAGGTTCCGGAGCGGCGCGATGAATGACC
AGAAAACGTGCGAACTTGATCGTCGCTGCAATGCTTTGTTCGGCGACGGTCGTCACCTCCTCTGTCAATGCGC
AGGATCAGGCCGCACCACCGCTTGTGACGCAGATCGGCAACGGCAACTGGCTTCCGCAGGAGGAAGCGGAGTC
GCTGCGCGACGAACTCTACTATCAACGCGCGATCCATGCCTACATAACCATGCTTCCGGCGCTGAACGTGATC
GGTATGCGCGACGGTTCGGAGGCGACATTCGGTAAAGGCTACAACATCCTGCCAATCTGGAAGGACCGGATGG
ACTCGCGCACGTGGGTGCCGACACCGAACGCCGACGTAATCTACTCGATGAGCTATCTCGACCTGAAGGAAAC
CGGCCCGCTCGTCGTCGCCGCGCCACCCAACGTCATCGGCATGTTTACCGATTTCTTCCAGCGTACGATCACG
GATGTCGGACTGATTGGACCGGATCGCGCGCGCGGCGGCCTCTACCTCCTGCTGCCGCCTGATTATGACGGCG
AGGTTCCGCAGGGCTACTTCGCGCTCAAAGCGAAGACCTACAATGTCTTTCTGTTCTTCCGGACGATCATGGC
GAAGGGCGAAAACGGACCGGACCCCAAGCCCGCCGTGGCGCTCGCGGAGCAGACGCGCGTCTATCCGCTTTGG
ACGCCCGAGAAAGACGTCAAGCCGATGCAGTTTCCTAACGGCAGCGGCAAGCGCATCAACATGATGTATCCGG
TCGATAACGCCTACTGGACCAAGTTGAAGGAGTTCGTCGACCACGAGCCGGTCTCGGCGATCGATTCCGAACT
GCGCGGTGTGCTGGCATCGATCGGCATCATCAAGGGCCAGCCCTTCGAGCCGACCGGGAAGCAGCAGGAACTC
TTGAAAAAGGCGGTCGAGACAGCGCCCAAGATGATCTTGGCGCAGCGCCAGCTCGGCCGACCGGACGGACGCA
ATCGCTACTACACCGACAGGCAGTATGAAAATGTCTGGGCCAGCGGCACATCCGAATGGCTCCAGGACAGTTA
TCTCGATGTCGACCAGCGGGCTGGCTATTTCCAGGTTGCCTATTCATCGGCTCCGGCAATGGTCATGCGGACG
CTGGGGGCAGGTTCCAAATACCCGGTTGCATACCGAGATGCCGCGGGCGAGTTCCTCAGCGGCTCCAGCACCT
ACAGGCTGCATTTGCCACCCAATCCGCCGGCTGCCCTGTTCTGGGCGGTTACCGCCTACAACATCACCGACGG
CACGATGGTGGAAGCGCCGCAGCTGATGCCGTCCATCAACGGCTTCAACAAGGTCGCGACGAACCAGGACGGC
TCGGTGGACCTGTGGTTCGGACCGGAAAAACCTGCCGACGCGCAGGACTCGAACTGGATCCAGACCGTCGGCG
GCCGGAATTTCCTGATAGCGCTGCGTCTCTACGGAACCGGCGTCGAGTTCTTCGATCAGACCTGGAAGCCGGA
TGATGTGGTCATGGTGAAGTGAATGACTGCTTTGCATCCCATTTCGCGCCGGACCCTCATCGGCGGTCTTGCC
GCCACGTCGGCATTTGTCGTGCTGCATCCCTTCTCGACCCGCGCGGCTGCCAACCAGGCGCATCTCAGGATCA
TGGAAACCACGGACCTGCACGTCCACGTCTTTCCCTACGATTACTATGCCGACAAGCCGAACGACACGCTTGG
CCTAGCGCGCACCGCTTCGATCGTCGACGCCATCCGCGCCGAGGCGGCGAACGCGGTCCTCGTCGACAATGGC
GATTTTCTTCAGGGCAATCCGATGGGCGATTACATCGCCTACAAGCGCGGCATGAAGGAAGGCGACATGCATC
CGGTCATCGCCGCGATGAACGTGCTCGGCTACGACTGCGGCACGCTCGGCAATCACGAGTTCAACTACGGCCT
GGATTTCATGTTCAATGTGATCAACGGCGCAAACTTTCCGATCGTCTGCGCCAACCTGACCAAAGGCGCCATG
GCCGCCGCCCCGCGCCAGGACGAGCTTTTCCTCAAGCCCTACGTAATCCTCGATCGAAAAGTGAAGGACGGCG
CCGGTCAGGAACACCCCATCCGCATCGGCCTCATCGGGTTCGTACCGCCGCAGATCATGACCTGGGACGCCAA
GAACCTCGAAGGCAAGGCGAATGCGCGCGACATCGTCAAGGCCGCGGAAGCCTGGGTGCCGCAGATGCGCGAG
GAAGGCGCCGACATCGTCATCGCCCTCTCACATTCCGGCATAGGGCAGCAGGCCTATGCCGAAAATCTCGAAA
ACGCGTCCGTACCCCTGGCGGCAATCGAGGGTATCGACGCCATCGTCACCGGCCACAGCCACCTCGACTTTCC
GGGACCCAAGTTCGACGGAACTCCGGGCGTCGACAATGCCAAAGGGTTGATTTCAGGAAAGCCCGGCGTCATG
GGCGGCTTCTGGGGATCCCATCTCGGCCTCATCGATCTGCTGCTCGAACGCGACGGCGGCCAGTGGCGTGTGA
TCGGTTCGACAAGCGAGGCGCGGCCGATCTTTCGCCGGGAAGAAAAGAAGGTGATCGCCGAGGTCGGTGACAA
GCCGGAAGTGCTCGCAGCCGCGCAGAAGGACCACGAAGCGACGCTTGCCTATGTGCGCACCCCCGTCGGCAAA
ACTTCGGCACCGCTATACTCCTATTTCGCGCTGGTTGCGGACGATCCGTCGGTGCAGATCGTGAGCCAGGCCC
AGACGTGGTATATCCGCGAAATGCTCAAGGACACCGAGCACAGCGACCTGCCGGTCCTGTCCGCCGCAGCCCC
GTTCAAGGCCGGCGGGCGCGGTGGCGCCGAGTACTATACGGATGTTCCAGCGGGCGACATCGCCATCAAGAAT
GTCGCCGACCTCTACCTCTATCCGAATACGGTGCAGGCGGTCGTCATCACCGGCGAAGAGGTGCGCAACTGGC
TCGAAATGTCCGCCGGCATCTTCAACCGGATTGCACCGGGATCGGTCGATGCAAGCCTGATCAATGGCGACTT
CCCTTCCTATAATTTCGACGTGATCGACGGGGTCACCTACCAGATCGTTCTTTCGCAGCCTGCGAAATACGAC
AAGGACGGCAATGTGGCGAACCCGGAGGCCAGCCGCATCCGTGATCTGAAATTCGATGGCAGGCCGATTGATC
CCAAGCAGAGATTTGTCGTAGCGACCAACAACTACCGCGCCGGCGGCGGCGGCAATTTTCCGGGCATCGCCGG
CGACAAGGTCGTCTTCGTCGCGCCGGACACCAACCGCGACGTCATCGTGCGCTACATCGTCGAACAGGGAACG
ATCAACCCCTCGGCGGACGCGAACTGGACCTTCGCGCCGCAGAAGGACACGAGCGTGCTGTTCGACAGCGGCC
CGAGGGCGCGACAATTCCTGGCAGAGGTCAAGGCGGTGAAAATCGAGAATGCCGGCGACGGCAGCGACGGTTT
CGCCCGGTTCAGAATCAAGCTCTGAATGCTCCTGGCAGCCAGCAGTTTTGCCACTGGTGCTCTGGCCGCGCCC
GTGCATGCGATCGCCATGCATGGCGACCCCGCCTTGCCTGCCGATTTCAAGCACTTCCCTTACGTCAATCCGC
AGGTGAAGAAGGGCGGGAAGATCGCCTACGGGGTCGTCGGTACGTTCGACAGCCTCAATCCGTTCATCTTGAA
GAGCATGCGCACGACCGCGCGCGGCATGTGGGACCCTGGCTTTGGCAACCTGGTCTATGAATCCCTCATGCAG
CGCTCACAGGACGAGCCCTTCACGATGTACGGCCTGCTCGCCGAAACCGTCGAATGGGACGATGACCGCACCT
TCATCCAGTTCAACCTCAATCCCAAGGCGCGCTGGGCCGATGGCCGGCCGGTGACCGCCGAAGACGTGATCTT
CACCTTCGAATTGTTGCGCGACAAAGGGCGTGCGCCTTTCAGCAACCGCCTCTCGAAGGTTGCGAAGATGGAG

AAGGTCGGGGAACGTAGCGTGCGCTTCACCTTCACCGAGGAGGCCGATCGCGAGATTCCGCTTCTGCTGGGGC
TTTCGCCCGTGCTGCCGAAGCATGCGATCAACGTCGAGACGTTCGATCGGACGAGTCTGGAGCCGCCGCTCGG
CTCCGGCCCCTACCGGGTCGCGGAAGTCAGGCCTGGCGAGCGGATCGTCTATCGCCGAAACCCCGACTATTGG
GCCAAGGATCTGCCCTCGAAGGTGGGCCTCGACAATTACGATGAGATCTCCGTCGAATATTTCCTGCAGGAGA
ACACCCTTTTTGAAGCCTTCAAGAAGGGTGTGGTGGACATCTACGCCGAAGGCAGCGCGACGAAATGGGCCCG
GGCCTACGATTTTCCCGCCGTTCGCAGCGGCAATGTGATCAAGGAAACGTTCAAGCCGAAGACACCGTCCGGC
ATGCTCGGCTTCGTCTTCAATACGCGCAGACCGATGTTCGACAACATCAAGCTCAGGCAGGGGCTCGCTCTCG
TTTTCGACTTCGAATGGGTCAACAAGAATCTCTTCGACGGCGCCTATACGCGTACGCAGAGTTATTGGCAGAA
TTCGTCGCTTTCCTTCCTCGGCGTGGCAGCCGACAATCGTGAACTGGACCTCATGGGCGACGTGAGGGAGCGC
ATCAATCCGGCGATCCTCGACGGAAGCTATCGACTCCCCGTGACGGACGGCTCGGGCCGCGATCGCAATGTGC
TCCGGGAAGCGGTGACCCTTCTTCGCGAAGCGGGCTACTCCATCAAGGACGGCAAGATGGTCGACGCCAAGGG
GACGCCGCTGGCCTTCGAGATCATGAGCCAGAATGCCGGCCAGGAGAAGGTTGCGCTTGCCTATCAGCGCTTT
CTCGCGCCGCTCGGCATCGCAGCGCGGGTTCGGACCGTCGACGATTCACAATATCAGTTGCGCAGCCAGTCCT
TCGATTACGACGTCATCATCAAGTCCTTCCCCTCGTCGGTCTCTCCCGGTCTCGAGCAGATCAACCGCTGGAG
TTCGCAAACTCGGGACAGGCAAGGCAGCGACAACTTCGCAGGCGTCGCCGACAAGGACGTCGACAAGCTGATC
AACAACATCCTGCAGGCGCGCAACCCCGAGGACTTCACCGCCGCCGTTCGCGCGCACGACCGGCTCCTCGTCA
ACAATTCCTATCTGGTTCCGCTTTACCATCTCGACGCGCAATGGATTGCCCGCTGGAAGCATATCGGCCGGCC
GACGACGGTACCGCTTTACGGTTATCAGTTGCCGAGTTGGTGGGACGAGCGCGTTCAGTAAATGTCGCTTCTT
TCGGTCGAGGAAGCACTACAGAGAATTTTGGCCAGGGCCAATCCGCTGCGACGGACGGAGCGCCTTGCGCTGC
ATGAATGCCTCGGACGGGTGCTTGCCGAGAACGTAGCGGCCAAGCTGACCCACCCCCCTTTCGACAATTCCGC
GATGGATGGTTATGCCGTCCGCCACGAGGATATCGCCGAAGTCGGAGCGGAGCTCTCCGTTGTCGGCGAGTCC
GCCGCAGGACGCCGTTTTCGCGGTGGCGTGAAGGGCGGGGAGGCCGTACGCATCTTCACCGGCGCGCCGGTAC
CTGCAGGCGCCGATACCGTCATCATTCAGGAAGACACGGAAAAACTGCCGGACGGACGCATCCGCACCCTGTT
TGCGCCGGCAAAGGGCCGCCACATCCGTTTGGCGGGCCAGGATTTCCTGGCGGGTGACGTCGCGCTTTCCGCC
GGCAGCGCACTCGACGCCGGCCGCCTGACGCTCGCGGCCGGCATGAATCATCGGGAGCTGCCGGTTTTCGCGC
GACCGAAAATCGCAATCATCGCCACGGGCGACGAATTGTTGCCGCCGGGAAGTTCCCTTGGCCCCGACCAGAT
CATCGCTTCGAACAGTTTCGGGATCGCTGCGGTTGCCCGCGACAACGGGGCCGAAGTTCTTGATCTCGGCATC
GTCGGCGACGATCGGACAGCGATCGCCGGCGCCGTCGCCAAGGCACAGGCAGCCCAGGTGGACGTGCTCGTCA
CCCTCGGAGGAGCTTCGGTCGGCGATCACGATCTCGTCCAGTCGACGCTTCTCGGCTGCGGCATGACGCTTGA
TTTCTGGCGCATTGCCATGCGACCGGGAAAACCGCTGATGGTCGGGGATCTCGACGGCGTCACGGTGCTCGGC
CTTCCGGGAAACCCCGTTTCGAGCCTCGTCTGCGCCCTGCTCTTCCTCGAACCTCTGACGCGCAAGCTTGCGC
ATCTGCCGCCCCGTTCGAGACTGGTCGATGCACGGCTCGCCGCAACGCTGCCGGCCAATGACCGGCGCCAGGA
TTATATACGTGCCCGCCTCACGCTCGAGGCGGACGGTTCGCTCGTAGCGCATCCCTTCCCGCGCCAGGATTCG
TCCATGGTCAGCATTTTCGCGCAATCCGATGGGCTGATCGTGCGGCGACCGGGTGCTCCCGAAGCCGCGAGCG
GAGAGGCATGCAGCGTTTTCAGGCTCAGAGATCCCGCCTGAATGGCGAGACAGCTGACAGGAAAGCTGCCCCA
GTCCATCGACGAGACGAGGGCGATGCTGGAGGCGCAGGATTATCTCGCCGGAACCGCGCTGGCGACCGTTCTC
TTCCTGGCCCTCAGGATGAAGCGCCCGCTATTCCTCGAAGGCGAGGCCGGGGTCGGCAAGACCGAGATCGCCA
AGGTACTGGCGCGCGCCCTCGACCGCCCCCTCATCCGGCTTCAGTGCTACGAGGGTCTTGATGTCGCCTCGGC
CGTCTACGAATGGAACTATCCGGCTCAAATGCTGGAAATCCGCCTGTCGGAAGCGGCGGGCACCGTCGACCGC
CAGAGAGTGGAAAGCGACATCTTCTCCGAGCGGTTCCTCATAAGACGGCCGGTGCTGCAGGCGATATCGACGA
CTGCCGGGAGGGCGCCGGTGTTCCTGATCGACGAGCTCGACCGCACCGACGAAGCCTTCGAAGCCTTTCTCCT
GGAGGTGCTTTCCGATTTCCAGGTCACAATCCCCGAACTCGGGACGATCAAGGCCGACGAGCCGCCGATCGTC
ATCATCACCACCAACCGGACCCGCGAAATCCACGATGCCTTGAAGAGACGCTGTCTCTATCACTGGGTCGACT
ACCCCAAGGCGGCGCAGGAACTGGAGATCATCCGCCGCAAGGTTCCGGGCTGCAACGCGGCGCTTTCACGCGA
GATCGTTGCCTATGTTCAGAGGTTGAGGGCGCTCGACCTCTTCAAGAATCCCGGCGTCGCGGAAACGATCGAC
TGGGCCACGGCGCTCACCGAGCTCGACGCGCTGGCGCTCGATCCCGAAACGGTGGCCGACACGCTCGGTACGC
TTCTCAAATATCAGGACGACATCGCCCGGATCGAAGGTGCCGAAGGCCGCCGCCTGCTTGCCGAGGTGAAAGC
CGAATTGCTGGCGCAGGACTGAATGACGATTTCCAACACCCGCCCAATCACCCCGGACCTGATCGCCTCGCAC
GGGCTCAAGCCCGACGAATACGAACGCATCCTGAACCTGATCGGGCGCGAGCCGACCTTTACGGAGCTTGGCA
TCTTCTCGGCGATGTGGAACGAGCATTGCTCCTACAAATCCTCGAAGAAATGGCTGCGCACGCTTCCGACCAA
GGGGCCGCGCGTCATCCAGGGCCCTGGCGAGAACGCCGGCGTTGTCGACATCGACGACGGTGACTGCGTCGTC
TTCAAGATGGAGAGCCACAACCATCCCTCCTACATCGAACCCTACCAGGGAGCGGCAACGGGCGTCGGCGGCA
TCCTGCGTGATGTCTTCACGATGGGTGCGCGTCCGGTAGCGGCGATGAACGCGCTGCGGTTCGGTTCGCCGGA
TCACCCGAAGACCCGCCACCTCGTCTCGGGCGTCGTTGCAGGCGTCGGCGGCTACGGCAATTCCTTCGGCGTG
CCGACGGTCGGCGGCGAAGTCGAGTTCGACGCGCGCTACAATGGCAACATCCTCGTCAATGCCTTCGCGGCGG
GGCTCGCCAAGACCGACGCGATCTTCTACTCGAAGGCGGAAGGCGTCGGCCTCCCGGTCGTGTATCTCGGCGC
CAAGACCGGACGCGACGGTGTCGGCGGTGCGACGATGGCATCGGCCGAGTTCGACGAGTCGATCGAGGAGAAG
CGCCCGACCGTTCAGGTTGGCGACCCCTTCACCGAGAAATGCCTGCTCGAAGCCTGCCTGGAGCTGATGCAGA

CCGGCGCGGTCATTGCGATCCAGGACATGGGCGCTGCCGGCCTCACCTGCTCGGCCGTCGAAATGGGCGCCAA
GGGCGATCTCGGCATCGAACTCGATCTCGACAAGGTGCCGGTGCGCGAAGAGCGCATGACGGCTTACGAGATG
ATGCTTTCCGAAAGCCAGGAGCGCATGCTGATGGTGCTGCGCCCGGAAAAGGAAGAGGAAGCCAAGGCGATCT
TCGTCAAATGGGGCCTCGATTTCGCGATCGTGGGCAAGACCACCGACGATCTGCGTTTCCGCATCCTGCACCA
GGGCGAAGAAGTGGCGAACCTGCCGATCAAGGAGCTCGGCGACGAGGCGCCGGAATACGACCGCCCCTGGACG
CCGGCAAGGGCACCCTCGCCGCTCGCCACCAACGACGTCCCGCAGGCTGATGTTTCCGATGCGCTTCTGAAGC
TCGTCGGCTCCGCCAACAATTCCTCGCGCCGCTGGGTCTATGAGCAATATGACACGCTGATCCAGGGCAATTC
GCTGCAGCTGCCGGGCGGCGACGCCGGCGTGATCCGCGTCGAGGGCCATGAGACGAAGGCACTTGCCTTTTCT
TCGGACGTTACGCCGCGCTATGTCGAAGCCGATCCGTTCGAGGGCGGCAAGCAGGCGGTCGCCGAGTGCTGGC
GAAACCTGACTGCGACCGGCGCGCTGCCGCTCGCCGCGACCGATAATTTGAACTTCGGTAATCCCGAACGGCC
GGAGATCATGAGCCAGCTCGTCCATGCGATCAAAGGCATCGGCGAAGCCTGCCAGGCACTCGACTTCCCGATC
GTCTCCGGCAACGTCTCGCTCTACAACGAGACCAACGGCCAGGCGATCCTGCCGACCCCCACCATCGGAGGCG
TCGGCCTCGTCCGCGACTGGTCGAAGATGGCGCGGATTCGCTTTGCGGCGGCGAACGAAACCATCCTGCTCGC
CGGCGCTCCGGAGAGCTGGGGAACGCATATCGGCCAGTCGGTCTACATGCGCGATATACACGGCCGCACCGAC
GGTCCGGCGCCGCATGTCGACCTCGGCCATGAACGCAAGGTCGGCGATTTCGTTCGCGGCTTGATCGAGGACG
GCCTGGTTACGGCCGTGCACGATTGTTCCTCGGGCGGACTTGCGCTCGCCGTCGCCGAGATGGCCATCGCTTC
CGGCATCGGCGCGACGATCGATGCACCTGCGGAACACGACCCGATCCCGGTCTTCTACGGCGAAGACCAGGGC
CGCTATGTCGTAACCGTGGCCGAGGGTTCGGCCGAGACGGTGGCCGCACGTGCAAAAGCCGCCGGCGTTGCGC
TGCCGGTGATCGGCAGGACAGGCGGGAATGCGGTGCAGCTCGGAGACGCCAGGCCCGTATCCGTCGATGAATT
GCGTTCCGCACATGAAGCGTGGTTCCCCAATTACATGGGCGGCGATCTCGCACCGGACAATTGAATGAGAAGA
TTTGCCCGCACGACCCTGACTGCCGCCCTGCTTTATAGCGTGGCAGGCATTGGCGCTCATGCTCAGGAAACGC
CCGACGAACTCGATACCCGCATCGGCAAACTGACATTCGAGAGCGGCTACCCCTCGAAGGAAACCGTGGCCAA
GCTTTACGACGAAATGGATTTCCAGCGAGCGTCGCAAGCTTACCTGTGGGGCATTCCTGCCGTCGGCCTCAAC
GAATGGCGAAAGGCCCATTTCAACGTCTTCAAGGCCAAGAACGGTGAAATGCTTTCCTATCTCGATTTTGCCG
AGAAGCTTGGCATTTTGACGCCGAACTACACCACGCCTTACATCGCGACGTTCATCGATCTCAAAGAATCCGG
GCCGATGGTCGTGGTTGTACCGCCGGGGCTGATTGCAGGCATGGTGCTTGACCAGTGGCAGCGCGTCATGGCC
GATCTAGGCGTGGTCGGCCCGGATATGGGCAAGGGCGGAAAGTATCTGATCCTCCCGCCCGGATACGGCCCGG
TGGAGGCCGAGGGATACTTTGTCATCCAATCCACGAGCCGGGACGTGCTGGCGGGGTTCCGCCTGCTCGATGC
CGACAAGGAAAAGGCAATTGCAGAACTCATCCCGCAGATCAAGACCTACACCTGGACGCTTGACGGAACTGGC
GAGCCGATGCCCGTGCGCGCTGCGGGAGACGTCAAGTGGACCCAAATGCCGCCCCATGGAATGGCCTATTGGG
ACAGCCTGAACGAGGTCATTCAACGAGAACCCGTCGAAGAGCGTGATCGCTTCATGATGGCGCAGTTGAAGTT
CCTCGGCATTGAGAAAGGGAAACCCTATCAACCCGACGAACGCCAGAAGAAGATTCTTCAGGACGGCGCGATC
GTCGGCGAAGCCATGGCCAAGGTAAATACGACAGACAAGCGCGTCGAACCGCCCTTCTGGGAGGGCACGAACT
GGAAACATGCGCTTGTGGTTGCGACCGACCAGAAGGCCAACACCTATGACCAGCTCGATGAGCGTGCTGCCTG
GTTCTACGAAGCGGTCGTGATCTCCAAGGCAATGCTCACCCAAACTCCGGGCGTCGGCCAGCGGTACATAGCC
TCCTACAAGGATGCAGACGACGCATGGCTCTCGGGCGAGAACAACTATCGGCTTCATGTCCCTGCCGATCCGC
CGGCCAAGGGCTTCTGGTCTGTGACGGCCTACGACGAGTCGACCCGGCAGATGCCGGTCACCGAACAGGGACG
GCCGGACATCTCGTCGCGAAAAGAAGACATAGTAAAACACGAAGATGGATCGATTGACGTATATTTCGGTCCC
ACTGCACCGAAAGGTCACGAGGCGAACTGGGTGCAGACAAATGCCGACAAGGGATGGTTCGCGTATTTCCGGT
TCTACGGACCGACCGAGCCCTTCTTCGATAAATCCTGGTCGCTGCCTGATATCGAGAAGGTCAAATAGATGAC
TGAGAAGATGACCCTTGCCTTGATCTACGGCAGCGCACGGCAGGGCCGTTTCTGCGATACGGTCGCCAGCTGG
CTCATTCGTGAGCTCGAGACATCCGACGTGTTCAGCCTGACGATCATCGATCCCGTCAGAATGAAAAGCGCTC
GCGGCGACAAATCCGCCGGACCAGCCGAGTGGATGGAACGGAAGGTCGCCGAAGCGGACGCATTCATCGTCAT
CACTCCGGAATACAACCACGGCTACCCGGCGGCGCTGAAGGAGATGATCGATGCGGTCTATGAGCCCTGGCAC
GCAAAACCGGTCGCCTTCGTTTCCTATGGCGGCGCATCGGGCGGCATAAGGGCCGTCGAGCAATTGCGGCAGG
TCTTCGGCGAATTGCATGCCGTCACCCTGCGCGACGGCATCTCCTTCGCCAAGGCCTGGTCGAAGTTCGATGC
GGCGGGAAATCTCTACAAACCGGAAGAGATGCGAGCGCCGCTGTTCCTGATGATCGACCGGCTGACCTGGTGG
GCGCGCACGCTGAAATACGCGCGCAAGGCGACCCCTTATAACGAGATCGCCGCATGAATGAGCACGGACCACG
CCCGACTGGACGGGAAGATCGCCATTGTGACCGGGGGCACGCAGGGGCTCGGTGCCACGATTGCCGCACTCTT
CGCGGAGCGCGGCGCCGCCGGCGTCGTCATTTGCGGGCGCAACGCGGCGAAGGGCGAGGCGAAAGCTGCCGAG
ATCGCCGCGGCGACGGGTTCGAAGGTCGTCTATGTACCAGCGGATCTCGAGCGGGTGGAGGATGCCCGTGCCG
TCGCGGCGGCTTGCGACCGCGCCTTCGGCCGCGTCGATGCGCTGGTCAACGCCGCAGCGATCACCGACAGGGG
TACGATCCTCGACACCAGCCCGGAACTCTTCGACCGGATGTTCGCGGTCAATGTGCGGGCGCCATTCTTCCTG
ATGCAGGAGGTGATAAAGGTGATGCGGCGCGAGAAGACAGAGGGCACGATCGTCAATATCGGCTCCATGTCGG
CGAAGGCGGGTCAGCCCTTCATCGCAGCCTATTGTGCGTCGAAGGGGGCACTGGAGGCGCTGACCAAGAACAC
CGCCTATGCCGTCCTTCGCAACCGGATACGGGTCAACGGCCTCAACATCGGCTGGATGGCCTCGGAAGGCGAA
GACCGGATCCAGCGCGAATATCACGATGCGCCGGCGAACTGGCTCGAAAAGGCGGCAGCGAGCCAGCCCTTCG
GCAGGCTCGTCGACCCGGCCGAGGTCGCGCGTGCCTGCGCCTATCTCTCATCGGCAGAATCCGGCCTCATGAC

GGGTTCCGTCATCTGCTTCGATCAGTCGATCTGGGGTGCCTATGACGCCTCGCCTCATCCGGAGGCGCCGCTC
TGAATGAATGGACTGATGATTGCAGCCCCGAGCTCCGGCTCGGGCAAGACCACGGTCACGCTCGGCCTGATGC
GGGCATTGAGACGGCGCGGGCTCTCGATCGCGCCTGGAAAGGCGGGGCCGGACTATATCGACCCGGCTTTTCA
CACGGCTGCCAGCGGCAAACCCTGCTTCAATTATGACCCTTGGGCAATGCGCCCCGAGCTGCTCCTAGCCAAT
GCGGCGGCTGCGGCGGAAGACGGCTCGGTGCTGATCATGGAGGCGATGATGGGCCTCTTTGACGGTGCAGCGG
ACGGCAGGGGCGCGCCGGCGGATCTCGCCGCAGCACTTGGACTTGCAGTGATCCTGGTCGTCGATTGCGCGCG
CCTCTCCCATTCCGTGGCCGCCCTGGTCGGCGGCTATGCCCGTCACCGCGACGATGTCCGTGTCGCGGGCGTC
ATTCTGAACAGGGTCGGCAGCGACCGGCATGAGGGAATGCTGCGCGATGCGCTTGCCGGAATTGCGATGCCGG
TCTTCGGCGTACTGCGACAGGACGCTGCACTGAAGCTGCCCGAACGCCACCTGGGCCTCGTACAGGCAGGCGA
GCATGGATCGCTGGAAGCCTTCATCGATCACGCGGCAATGCGGGTCGCATCGGGTTGCGATCTCGAGGCGGTG
CTTGCTGCGGCGACGCCGCTGACGGTGGGAGAGAGGGCCGGAACGCTAAAGCCGCTCGGCCAGCGGACAGCCG
TCGCGCGCGACGTGGCCTTCGCCTTCTGCTACGAGCATCTTCTTTCGGGCTGGCGCGGGCAGGGGGCCGAAGT
CACATTCTTCTCGCCTCTTGCGGACGAGGCGCCCGATCCGCGTGCCGATGCGGTCTATCTCCCCGGCGGTTAT
CCCGAGCTTCATGCGGAACAGCTCTCCAATGCTTCGAATTTCCGCGCGGCCATGCACAAGGCAGCTGGGGGCG
GGGCGCGCGTTTTCGGGGAGTGCGGCGGCTACATGGTGCTCGGCGAGGGGCTTGTCGCGGCGGATGGCGGGCG
CTACGAGATGCTCGGGCTTTTGCCGCTCGTCACGAGCTTCGCCGAACGCAAGCGGCATCTCGGCTACCGGCGC
GTCACGCCGGTTGACGACGTGTTTTTCCGGGGACCGATGACGGCGCACGAATTTCACTATGCCACGATCGTTT
CGGAGGGCGCGGCTGAGCCGCTCTTCACGGTACGTGACGCGGCAGGGCTCGATCTCGGCCGCGCCGGCCTCCG
TCGCCGGAACGTTGCCGGCTCCTTCATGCATCTCATCGATTTCTCGGAATGAATGTTTCACTTGCGTCGTGCC
GCAATCGCTGTGTTGGGCGCAGCGTTTTACGGTTCCATACCGACAATTGCTGCCGAACCGGTTGGAGAAGCGG
TCCGGATCAGGACGGTGGTCACCGGCAGCGGCGGACCGCTCGTGGAGCGGGACGCCGTTCACCGGGACGAGCG
AATAAGCACGTCCAGATCCGGGCTCGGCCAGTTCGTGTTCCGCGACGGCAGTAAATTGGCGGTGGGGGCAGGC
TCTTCCGTCGTCATCGACAAGTTCGTCTTCAACGATTCGAAATCGGTGCAGCAACTCACCGTCGCCGCCGCCA
AAGGCACGTTTCGCTGGATCAGCGGAAATTCGAAGCACTCGGCCTATCAGATCCTGACACCTGCCGGAACGAT
CGGCGTACGCGGAACCGTGTTCGATTTCTACATCGGCAATGACGGAACGACGGCGATCGTTCTCCTGGAGGGC
GCGGCCCGGTTCTGCGGCCCGGCGGGATGCCAGCAGCTCACCCGACGCTGCGATTGCGTGATCGCAAAGCGCG
ACGGAACCATGACGGACACGCACAGGGCCGGTCGCCGCACACTGGCGACGCTCGGAAACCGGAAGGCCCTGCC
CTTTCTTTCGGGCGACCAAAGACTGTCTGCCTCCTTCGGCAGCATGAGCGGCAGTTGCGGTATTTCGGCCGCT
GTCGAGATCAAGCCGACCGCGCCGGAACGCGCGCGGCCGCCCGAAAGAGCAAGCCCGCAAAAGGCGCCCCGCC
CGGGCAAGACACCCAGCAACGAGCCGGGCAAGCGTGGCCTCGACAAGGGGAAGAAGGGGCAGGACACGGGCAA
GAAGGGGCAGGACACCGGCAAGAAGGGCCAGGATACCGGCAAGAACGGGCAACTGGACACAGGCACGAAGAAG
GGCCGCGGTTAGATGGCTACTCGAAACGTCGTTCTGACTGACGCACAATCAGCGCTGCTTGAACGGTTGGTCG
CCTCCGGGCGCTATCAAAATGCCTCGGAGGTCTTGCGTGCCGGGTTGCGGCTGCTCGAACGGGAAGAGGCCGA
GCTGGACGATTTGCGGGCAAGGCTGAGCACGGGACTCGATGAGGCCCGTCGCGGCGATCTAGCCGAAGGTACG
GGCGAAGAGGCGATCCGCGGTGCTTTCGCGGCAGCGCATGCAGCCTCCTGAATGTATGTCATGGCTCTTGCGA
CGGATTACGACGGCACACTTGCAGACTACGGTGCCGTCCGTCCTGAAACTCTGGAGACCTTGAAGAGGCTGAA
GGAGAGCGGGCGCAAGCTTCTGCTCGTGACCGGGCGCGAGCTGCCGGACCTCAAGAGCGTGTTTCCCGAAATC
GACGTGTTCGACAAGGTCGTCGTCGAGAACGGCGCGCTGCTCTATACGCCGGAAACCGGGGAGGAGCGGCTGC
TTGCACCCTCGCCGCCGGAGGCCTTCATCGAGCGCCTCAAGGAGAAGGGCGTCGACAGGATGTCGGTCGGCCG
TTCCATCGTCGCCACCTGGGAGCCGTTCCAGACAGCCGCGCTCGAGGCGATCAACGAACTCGGCCTCGAACTT
GAGATCATCTTCAACAAGGGCGCCGTCATGGTGCTTCCGACGGGGGTGAACAAGGCCACGGGGCTCAAGGCGG
CCCTCAAGGAGATGGGGCTTTCCTTTCTCAATGTGGTGGGGGTAGGCGATGCCGAGAACGATCACGCGCTTTT
AAGGATGTGCGGCTGCGGAGCCGCGGTCGCCAACGCCCTGCCGGCGCTCAAGGATACGGCCGACGTGGTCCTC
GAGGGTGTGCGCGGGGCCGGCGTCGAGCAACTGATGAACCGGATCATGGAGTCCGACTATGCGTTGTGCGCGA
GTGCCCGTCACCAGGTGCCGATCGGTGAGGATGACGAGGGGCAAGTGGAGATCGGACCGCAGGACGTGCTGCT
GATCGCGGGGAGTTCCGGCATCGGCAAGTCGAGGCTGGCGACCGCCCTTGCGGAGCGATTACGGGAGCGGCAC
TTCCAGCTCTGCATATTCGATCCCGAGGGCGACTATGAGGGGCTTGAAGGCGCCGTGACCGTGGGTAACGGGT
CTGTCGCGCCTACGGGCAGGGAGGTGCTGGAGCTGCTGGCCAATCCGGACGACAACGTCGTCGTCAGCGCAAC
AGGCGTCGAACCCAACGACCGGCCCGAGTTCTTCGCCAACCTGATGCCGGACCTGTCGGCGCTGCGTGCCAGG
ACGGGCAGGCCGCATTGGCTGATTATCGACGAGGCCCATCACCTCATGCCGGCAGCGCGCGACAGTGCCTCGC
TCGCGCTTTCCGACGACCGCTCCGGCATGATCATGATCACGGTCCATCCGGATTCCGTCTCACCGGATGCGCT
CAAGGAGATCACTGCGGTCGCAGCGCTCGGTCCAAAGGCGCGCGACGTAATCGCGACGATATGCTCCGTCTTC
GACGAGGCGCTGCCGGCCGGGCTGGACGCTTCTTTCGGGGAAGACGAGGTTCTTTTCTGGCGGCGCACGCCGC
CCACGCCGGTTCGGCGGATCAAGGCCGAACAACCCCGGGAAGCCCGCAAGCGGCATACGCGCAAATATGCGGA
GGGCCGGTTGGGCGAGGAGGCAAGCTTCTATTTCCGCGGTCCGAAGAACGAGATGAATTTGCGCGCCCACAAT
CTGACGATGTTTCTGCAGATCGCCGAAGGCATAGACGACAGGACCTGGGAGCACCATTTGCGTCGCGGCGACT
ACTCGAAGTGGTTCGAGGAACGCATCGGCGACGAGGAATTGGCCGGGGAGGCGGCAGGCATAGAAGAGGATCG
TTCGCTCTCGCCCGCCGAAAGCCGGCAGAGGCTGGCCGAGGCGGTGCGCCGGCGCTACACCGCGCCCGCATCC

GAAGCGGGTTGAATGAGCTTTCTCTCCGCCATACCGGTCATCGCACTTCTTGCCATGCTACCCGTTCTCCTTT
CTCTGCGGCCGGTTGCCGGTGTTCGGGAGTTCTCCGCCAGTTGCGCACTCTCGGCAGCGTCGGCAGGCGCCTT
GACGGTTGCGGCGGCGTTGCAGGCGCCGGTCGCCGCAATGTTCGGCTATGCGGGTCTCGCCGTTTCCCTGTTT
TTCGTCCTCCAGGGCCTCCGCCGGTTCCTCGCATTGTCGATTCCGGCCTCGCTTTGTCTGGTGCTCGCGTTCT
CGAGCGGCGTGCTCGGCTTCATCATCGCTCTCGGCGGCCACGACAGCCCGGCCGCCGGCATGATCGTCGTTGC
GGGCGTTGTCGCAGCTCTACTTCTCGTCCTTGGTCTGGCCGCAGCCGACCGCTGGCCGGGCGAAAAGCCGGCG
ATCCAGGTCGTTGTCGTCGGCTCCGCCGCCGTGGTTTTCACCGCCCTTGCCCATGCCCTCGGCCCATCGGCGC
ACGTCTTGCCGGGACCGGCGGGCTCGCCCGCTTCGAGCCTGTTCGATTTCCTGCTCGTCACCATGCGGGTGCT
GTGCCTTCCCCTCCTCTTCCTGGCCATGATCCTCACGATGCAGGCCCGGGTCATCGCCGGATTGAGGGCCATG
ATCGCACGCGACGGCTTGACCGGTGCGCTGTCACGCGGGACGCTCATGGAAGAGGGCGAGCGGATCTTCGCCG
AGTGCGTGGCACGCCAGGAGCCGGCTGCCTTCCTGCTGCTCGACCTCGATTTCTTCAAACAAATCAACGATCA
GTATGGGCACGCCTGCGGCGACATGGCGCTCGCGCATTTTGCCGATACCGTCTCGGCTTTCCTCGCCGGACGG
GGCGTCCTCGGACGGATCGGTGGCGAGGAATTCGGCATCGTCCTGCCGGACCACACGGAAGAGCAGGCAAGGG
CGCTTGCGGAGGATATCTGCCGCGTGGTGCGTGAGACGCCGGCCGGCCGCTCGCATCAGCGCATCCGTCTGAC
CGTGAGCATCGGGATCGCCGCCGCCATTGCGGGCGAGACGATTACCGACGTGATGATACGCGCCGACCTTGCT
CTCTACGACTCAAAGGCGGATGGTCGCGACCGTTGCTCGGTAGCAAGGGATCGCCGCATCGACGCCAGCGCCC
GCGCCCTTGCGGCAGCCGCCGCGCAATTGCGCGAAGCCCGTGCCGCCCGGTCGGAACTGCGCCACTCGGCGTG
AATGACAGCCACAGCGCGCAAAGCCCTTCTCTCCCTCGGATTCCTTGCGATCGCCGGCGCGCCGGCCCTGGCG
CAAGCTCCGGCTCAACCGGGGAACCCAGCCGCCGCGTGCGGCGGCGACCTCGGCTCCTTTCTGGAGGGCGTCA
AGGCCGAAGCGGTCGCCAAGGGCATCCCCGCAGACGTCGCCGATCGGGCGCTCGCAGGCGCCGCCATCGACCA
GAAGGTGCTGAGCCGCGACCGCGCTCAGGGCGTGTTCAAGCAGACCTTCACCGAATTTTCGAAGCGTACCGTC
AGCAAGTCGCGCCTCGACATCGGTGCGCAGAAGATGCGGGAATATGCCGACGTCTTTGCCCGGGCCGAGCAGG
AGTTCGGCGTACCGGCGCCCGTGATCACCGCATTCTGGGCCATGGAGACCGACTTCGGCGCCGTGCAGGGCGA
TTTCAATACGCGTGATGCGCTGGTGACGCTGGCGCATGACTGCCGCCGCCCGGAAATGTTCCGGCCGCAGCTT
CTCGCCGCAATCGAGATGGTGCAGCACGGCGATCTCGATCCCGCCGCGACCACCGGCGCCTGGGCGGGCGAGA
TCGGTCAGGTACAGATGCTGCCTGAGGACATCATCGCCTATGGCGTCGACGGCGACGGCGACGGCCATGTCAA
TCTCAAGAAGAGTTCTCCGGATGCCATTCTGACGGCGGCGAAATTCATCCAGAGCCTGGGCTTCAAGCCCGGG
GAACCATGGATTCAGGAGGTCCGCGTTCCGGAGCAGTTGCCATGGGAAAAGACCGGCCTGCAGCCCGGCATGA
CCGCCGGCGACTGGTTCGCACTCGGTGTCGCGCCGCGTGACGGCAACACCTCCAATAGTGGGCTCGAAGCCTC
GCTGGTGCTCCCTCAGGGCCGCAAAGGTGCTGCTTTCCTGACCTACCCGAACTTCAAGATCTATCTCGAGTGG
AACCAGTCCTTCATCTACACGACCTCCGCCGCCTATTTCGCGACCCGGCTTGCCGGTGCGCCTCCCTATCAAC
AGGGAAATCCGGAACCGGGGCTCGGCGACGCCGAGATGAAGGCCCTGCAGACCAGATTGCAGGCACTTGGGCA
TGACGTCGGCAAGATCGACGGCATTCTCGGCTCGGGCACGCGGGCCGCGCTGCAGAAGGAGCAGTTGAAGCTC
GGGATTCCCGCCGACGGGTGGGCAACACCGGGCCTGCTGGAGGCGCTTTAAATGACCAACATGATGAAGGCAC
TGGTCAAGACGAAGCCGGAGGTAGGGCTCTGGATGGAGCGCGTGCCGGTGCCGGAAATCGGGCCGAACGACGT
CCTGATCCGCGTCAGGAAATCGGCGATCTGCGGCACCGACGTCCACATCTGGAACTGGGATCAATGGGCGGAG
AAGACGATCCCAGTGCCGATGGTCGTCGGACACGAATTCATGGGCGAGGTCGTCGAGGTCGGGCCGGCCGTCA
GCAAGCATCATGTCGGCGAGCGGGTTTCCGGAGAAGGTCACATCGTCTGCGGCAAATGCCGCAATTGCCGCGC
GGGCAGGGGGCATCTCTGCCGCAACACGCTCGGCGTCGGCGTCAACCGCCCCGGATCCTTCGCCGAGTTCGTC
TGCCTTCCCGAATATAATGTCGTGTCGATCCCCGACGACGTGCCGGACGAGATCGCCGCAATCTTCGATCCCT
TCGGCAACGCCGTGCATACGGCGCTCTCCTTCGATCTCGTCGGCGAGGACGTTCTGGTCACCGGTGCCGGGCC
GATCGGCATCATGGGGGCGATGGTGGCCAAGCGTTGCGGTGCCCGCAAGGTGGTGATCACCGACATCAATCCC
GTTCGTCTCGATCTCGCGCGCAAGCTCGGCATCGACCACGTCGTCGACGCGTCGAAGGAAAATCTTGCCGACG
TCATGCGGGTGATCGGCATGACCGAGGGCTTCGATGTGGGGCTGGAAATGTCGGGCGCGGCGCCCGCTTTCCG
CGACATGATCGACAAGATGAACAATGGCGGCAAGATTGCGATCCTCGGCATTGCGCCGGCGGGCTTCGAGATC
GACTGGAACAAGGTCATCTTCAAGATGCTCAACCTCAAGGGCATCTACGGTCGCGAAATGTTCGAGACCTGGT
ACAAGATGATCGCCTTCGTCCAGGGCGGGCTCGACCTCTCGCCGATAATCACCCACCGTATCGGCATCGACGA
TTTTCGCGACGGCTTCGAGGCGATGCGCTCGGGCAATTCCGGCAAGGTGGTGATGGACTGGTAGATGCCTTCA
ATCAGGGACCACTCCGAGCGGGTCTACCGCATCGCACATCAGTCTTCCGCTGCGGTGACTTCGCCGGTCGCTG
CATCGTGGCGCCGTTGCATGACGCTGCACGGCCTGGCGCCGGAGGATGCGCGTTCGCCCTGGCGTCTTTCGGC
GATCGAGTTCCGGCAGGCGCGCGAAAGCTCCGGCGCCCTCATCGCAGAGGCGGCCGGCGAGCTCGACCGGCTG
TTCGCCACTGTCGGCAAGGCCGGGTGCTGCCTGCTGCTTACCGACGGAAAGGGCGTTGCACTGGAACGACGCG
GGGCCGGCGGCGACGATGCGGACTTTCGCGGGATCGGGTTGTGGTCGGGAACCGTGTGGAGCGAGGCGAGCGT
CGGGACCAATGGCATCGGTACGGCGATCGCGGACGAGCGTCCGGTCCTCATCCAGCGCGACCAGCACTTCCTC
AGCCGCAATATCGGCCTGAGCTGCGCCACGGCGCCGATCCGTGACGAGAGCGGCAAGCTCGCCGCGGCGATAG
ACATCTCCACCTGCCGTGACGACGCATCCGAGGCGACGCTTTCGATCCTCTCGCAGGCGGTGCGCGATGCCGC
AGCGCGAATCGAGGCCAATCTTTTCCGCCGCGCTTTCGTCGGCGCCCGCATCGTTCTCGTACCCGTCGACCGG
GCGGGACCTGCACTGCTTGCGGTCGACCGTGACGATCTCGTTCTCGGCGCGACGCGGGCTGCCCGGCTGTGGC

TCGGGCTCGACGACGAGCGTATCGCCGCCGGCGTCCCGGCTTCCGACCTGCTTCAGGAAGACCTCCCGGGCGA
GGGCGGCGAATTGCCGGACGCGGAACGTGCGGCGCTGCGCCGCGCGCTCTCCCGGGCGAAGGGCAACGTATCC
ATGGCGGCCGATCTCCTCGGCATCAGCCGCGCGACCCTCTACCGCAAGATGAAGCGATTGGCCCTGAACTGAA
TGATTCTATGGAGATCAAGCGCGAGTCGCCTGGCAATTGTAGTTGGTGCCGGCTTTTGGAGCATGCCTATACT
TGTTCAGGCGCAAGAAGCTCCCAAAGAAAACAGAACTTTGCTCGCTCCTATTGTCGTCACCGCGGACAAATCC
GACGCATTCGCGATCGGCGGTTCGGTCCAGCGGCTGGACCTGACGGATATGGAGCGCTTTTCCTATAGCGACC
CCAATCGGGTATTGCGGCAGATTCCGGGTGTTTATCTTCAAGAGGAAGAGGGGTTCGGCCTTAGGCCCAACAT
AGGCATCCGTGGTTCGGGTACAGACCGCAATGCCCGCATAACGGTCATGGAAGACGGCATCCTGATCGCGCCG
GCCCCCTATGCGGCGCCTGCGGCCTATTATTTTCCCAAGATGGCGCGCATGTCGGGGGTAGAGGTGGCCAAGG
GACCGGCAGCAATAAAATACGGTCCGATGACCGTTGGCGGCGCGATCAATCTTTTCTCGACCCCTATTCCCGA
TTCCGATATCGGAACGCTGACCGGAAAGGCGGACATTCTCGGTGGCAGCTATGGCGGCCGACGTGTCCACGCC
TGGACCGGAGGTTGGGCGCCGCTGGGCGACGCGTTCGAGATCGGCGGACTTATCGAAGGGCTTTACGATAAAT
CGGATGGCTTCAAGGATATCGATCGCGGCGGCGATACCGGATTCGATCTCACCGATTGGGGCGGCAAGCTGGG
CTTTCGCACGGCCGATGGAACGCAGAATTTCGAGTTCAAGTATCAAAACTACGATGAAACCAGCAATGAGACC
TATCTCGGCCTGACCCTGGACGATTTCAAGGACACGCCTTTCAGGCGCTATAACGCCAGTCAGAAGGACGTGA
TCGACGCCGCGCATAATATCTATCAATTCAGCCACAGTTACGAGATCACACCCGACATCAACCTGACGACGAC
CGTCTACCGTACCGATACCACGCGGGCCTGGTACAAGCTCAACGATGTCCGTAACGACTCCGATACTGGCTGG
GTATCCCTCAGCAACATCCTGGCGGACCCCGATACGTATTCGACGCAGATGGCCGATATCGTCGGTGCGGACG
GATATACCGGCCGAGCGGGGGCATTGCGTGTGCGCAACAACGCCCGCGCCTATCAGGCGACCGGCATTCAAAG
CGTGCTGACCACGAATTTCGATACCGGCGAACTCAGCCACGAACTCGAATTGTCCGCGCGCTATCATCAGGAC
GAGGAAGACCGCTTCCAGCAGGACGATCGCTACCAGATGGTCAATGGGCGCATGATCCTGACCTCCGCGGGCG
CGCCGGGCAGCCAGTCCAACCGCCTGAATGAAGCCAAGGCATGGGCTTTCTTCGCGCGCGACACGATCGAAGC
CGGACCGTTCACCTTCGTTCCCGGCCTGCGCTATGAATCGATCAATCTGGACCAGACGAACTGGGGGAGCGCC
GACCCCGGCCGCAACGGGCCGACCACTGCAAAACAGTCCTATGTCGATGTGTTCATCCCCGGCCTGTCGGCAA
CCTGGAGCGCCACCGACGAGGTTCTGCTGGTGGCCGGTGTCCATCGCGGCTTTGCCAGCCCCGCTCCGGGATC
GGACGTGGATCCCGAGACTTCATGGAACTACGAATCCGGCGTGAAGTATGATTCCGACGGCTGGCGCGCCGGT
GCCATAGGCTTCATCAACAGCTATTCGAACCTGCTTGGAACCTGCACCGCATCCACCGGAGGCAATTGCACCA
TTGGTGATCAGTTCGAGGGCGGCGAGGTCAGCGTCAAAGGCATCGAATTGACCGTTGGCCGCACATTTGGCAG
TATCGATAGTAACGGCTTCGAGGTTCCGGTAACGCTTGCCTACACCTACACGAACGCCAGGTTCCGCCGTTCC
GAGCCGGCCCAGGTCGATCACGCCCGTGCAGCGGTGTGTATCCGGATCGACCATGAAGTTCGGCAGGCATGCG
TCGCCATGACAGATTACGAGATCGGAAGGCTCGTCGATCAGGTGCCTCGGCAGTTCGGCCCGCAGAGCGTCGA
GCAGTTCGCCCGGCGGAGTGTTCTGATCCTCCGGGGCCAGGAAATCCGGATTGACGGCATCGCGCCGGACGAC
ATCCGCGGCCCGGTCGAACATCGACGCGAGCCGCCGCCCGAAAGGGCAGGCTTCCGTCGGCAATTCATGCAGC
AGCTTCAATTGCCGCAGGATCGACGGCCAGGCTTTCTTGAGGTCGACGGCGGAGAGCTCGCTTGCCGGCACAC
CCGGCAGCGCGCTGATGACGAGGCATGCTTCATCTGCCGCGATCCACTCGCAGACGGAGGGCGAGCCCAGGCG
GAACGGCGCCAGCCAAGCGACACGATCGCGCTCCCCCTCGAGCAGTGCTGCGGCCTTGCCGCTCGCAATCTTC
GCATAGGCGCCGCCGTCGCTGCGACGATAGACGCGGTCACCGGATTCGCCGTGACGAACCGGCGTCCATTCCC
GACCCGTGGGCAGTTTCGGAAGCAGAATTTCGTCACCGGCGTGGCGCCTGCTCATGAAGCCCGCCATTGCAAA
TGCGCAGACGCCATCATCCCCGTCCGCGATAGGTCGGCACGCCCTGATCCGGCAGCCAGACGCCCTCCGGCGG
GTTTCCCGTCTGCCAGAAAACGTCGATCGGGATGCCGCCGCGGGGATACCAGTAGGCCCCGATCCGGAGCCAC
TTCGGAGAAAGCAGCGAAACCAGGCGCTTGGCGATCTCGATCGTGCAATCCTCGTGGAAAGCGCCGTGGTTGC
GGAACGAATGCAGGAACAGCTTGAATGCTCAACCGGCATCATCGCCTGGCGCTTTTGGCTTGCACGGCAGCAA
TATTCGCGCTCCCGATCCCCCCGGTTCTGGCGCAGAGCGCGCCAACCGAAACGGCGGCGGAAGGAAATGCCAA
CACGACCGTGCTGAAGAAGATCGTCGCGAAAGGCGACCGCCTGGCAGGTGCGCAGCGTGGCGGCATCGCGGAT
ACGCCGCTTGCGACCGAGATCGACGCAAAGACCCTGGAGGAGAAGCAGGTCACGGATCTCGACGATCTCGGCC
GCAGCGTCGACGCAGGGATAAATGCATCGCGTGCCGACTTCGGTATCAATCTTCGCGGTCTTTCCGGTCCTCG
TATCGTGACCACGATCGACGGCGTTCCGATCCCCTATATCTCGAACAGCGCCCGTCAGGGTGCCTTTGCCTCG
ATCAATGCGAACGGCGGCGGGGACATGTTCGATTTCAATTCGCTCTCCGTGGTCGACATAGTGCGCGGGGCGG
ATTCGAGCCGTGGCGGTTCCGGCATGCTCGGGGGCGCCGTGGTGCTGCGCACGCTCGAGCCCGAGGATGTCAT
ATCCGACGGAAAGGATTGGGGCGCGATCTTCAGGTCCATCTATGACAGCGAAGACGACAGCATTGCGGGCTCG
GTTGCTGGAGCCCACAGATTCGGCCAGACCTCCGTTCTCTTTCAGGGCAGCTACCGCAAGGGGAATGAACGGG
ATAACGAGGGGACGGTCGGTGGCTACGGATCCGCCCGCACGGAGCCCAACCCTACCGATTTCGACCAGAACAA
CTTGCTCTTCAAGTTTCGGCATGAACTCGAAGGCGGTCACAGAAACGGCCTGACGGCGGAAAGCTTCCGCCGC
GACGCCGACAATGATCTCAGAGCGGAACAGGGGCGCCGGTACAAGATCGGCGACTATACCGGTTTTGAAGACC
GCGACCGCAAGCGCGTCTCGCTGGACTATGATTTCGAGGCCGCCTCGTCGGACGACTTCTTCAGCTTCGCCAG
GGCATCGCTCTACTGGCAGGATCTGGAACGCTCCTCCGGCTCGAATGGCCGGACGATCGCCGATGTGCCCTAC
GGGCGCGACAATTCGATCAGCAACGAGAGCGTGGGTTTCAACGGACGCGCAGGCAAGGATTTCGAAACCGGCG
GTTTCGATCATTCGCTAACCTTCGGCCTCGATGTCGCCCGTTCCGAGTGGAGCCAGTATACCTCGGCGGTTTG

CCCGACGCCGGCGACATGCCCGGCGCTCAACAACCAGTCCGAAGTCCCCGACGTGAGGAGCATGACGGTCGGC
GCCATATTGGAAGACCGCATTTCGGTCGGCGACAGCGCGTTTGCGCTCACGCCGGGGTTGCGCTTCGACTGGT
TCCAATATGACCCTCAGCTCAATGCAGGCTTCGAGTCGAATACAGGATCGGGAATCTTCGGGGATCTGAAGGC
CAGGGATGGGGTTCGCCTGTCGCCGAAGCTCCTCGCGACCTATGACGTTACGCCGGATGTCGAGCTGTTCGCA
CAATGGTCGATGGCCTTCCGGGCGCCGACCGTGGACGAGCTTTATAGCCGCTTCTACAACCCGTTCGGAAACT
ATGCCCAGCTCGGCAATCCGGATCTCAAGCCGGAAACCGGCAAAGGATTCGAGATCGGTGCCAATTTCGACAC
CGGTGAGCTGAGCGGCCGGGTCGCGGCGTTCCACAATATCTATGACAATTTCATTGAAACCGGCGATTCCATC
AACAGCGACACCGGGATCAGGGAATTCAAATATGCCAACGTCAACAAGGCCCGAATCTCGGGTATCGAGTTGA
GTGCGTTGAAAACCTTCGACAACGGCTTCAACCTCCATGCGTCGCTTGCCTACTCCTATGGCAAGAACGAGGA
CGAGGGCACGCGGCTGCGGACGGTCGCGCCATTCAAGGCGATCATCGGCGGCGGCTACAGCCAGGAAACCTTC
GGTGTCGATGTCTCGACGACGGTCTCGGCAGCCATGCCGGACGATAACGACAGCGAAACCTTCGATGCCCCCG
GCTACGGTCTCGTGGATATGACCGGGTGGTGGACGCCCGAAAGCTTCAAGGGATTGAGGGTAGAGGCCGGGGT
CTATAACATCTTCGACAAGAAGTATTTCAACGCGCTCGGCGTACGTGGCGTCGACCTCGCCTCATCGTCCGCT
CAGCCGCGCGACTTCTATTCCGAACCGGGACGCACGTTCAAAGTCTCGTTGACGCAACGCTTCTGAATGCAGC
CTCTTCCGCCTGAAAAACACGAGGACGCCGAGATAGCAGCCGGTTTCCTTTCGGCCATGGCAAATCCGAAACG
CCTGCTCATCCTCGACTCTCTCGTCAAGGAGGAAATGGCGGTTGGCGCCCTGGCCCATAAGGTCGGGCTGAGC
CAATCGGCTCTTTCTCAGCACCTTTCGAAGCTTCGTGCGCAGAACCTGGTCAGCACCCGTCGTGACGCACAGA
CGATCTATTATTCGAGCTCGTCCGACGCCGTATTGAAGATTCTGGGCGCACTATCCGACATTTACGGCGACGA
CACCGATGCCGTGGAAGAAAAGCCTCTCGTTCGCAAATCGGCGTAAATGGGTGGCAAGCGGCACCAGAATTCA
GGCAAGGGCGGCAAGGGCAGGGCGAAAGACGCGGCGAGCGCGCCGCAACCGAATGCCGGCAAGGTAACGCTCG
AGGGCCGCAGCTTCCTCTATGTCGGCGGCCGCGACTGCCAGGTCGCGCATCTTCGCCAGATCGCCAGTTCCTA
CGGTGCGGAACTCATCCACCATGACGGCGGTTTACGAGAGGCGGTATCGCGCATCGATCGTGTCCTGCCCTCG
GTCGACTGCGTCTTCTGCCCGATCGACTGCATCAGCCACGATGCGTGTCTTCGCGTGAAGACGGGCTGCAAGA
AGTGGGGCAAGGCCTTCGTGCCGCTGCGCAACGGCTCGAAGTCGAGCCTCGAGCGGGCGCTTCAGGATCTGAC
CGGAAACGGCAGCGAGAATTGAATGATCAGAGCATCCGATATTTCCGTCCGCCTCGCCGGAAGACAGGTGCTG
CACGGCATCTCCCTCGATGCCGCCCCCGGTGCAATGACTGCAATCGTCGGCCCGAACGGATCGGGAAAGACCA
CGACCTTGAAAGCGATCTCGGGCGAGCTCACGCCTTCGGCCGGCAAGGTCACCATCAACGGTCGCGACATCGC
AAGCCTGAAACCGTGGGAGCTTGCGCTCAAGCGCGGCGTGCTGCCGCAGTCGACCGTCATTTCCTTTCCGTTC
ACCGTTCGGGAGATCGTGCGCCTCGGCCTCATGGCCAACGGCGGCGAGGCTTCCGCCCACGGCCGGATTGCCG
ACCAGGCGCTCGAAGCCGTCGATCTTGCCGGATTTTCGGGGCGCTTCTACCAGGAGCTATCGGGCGGCGAACA
GCAGCGGGTCCAGCTCGCGCGCGTCCTCTGCCAGATCTCGGCCCCGGTCGCGGCCGGTGAACCACGCTATCTC
CTGCTCGATGAGCCGGTATCGAGCCTCGACATCCGCCACCAGCTCACGATCATGCGGCTTGCGCGCCAATTCT
GCGCCGACGGCGGCGGTGTCGTCGCGGTCATGCACGATCTCAACCTTACATCGATGTTTGCCGATCAGATCGT
GATGATGAAAGCCGGCCGCATCCGGGCGCGTGGAGCACCGAAAGACGTGCTGACCGACGAGACCATGGAAGCG
GTCTTCGGCTGCCGAATGCGGGTGAGCGTCGCTCCGGCACACGATATCCCGTTCGTATTGCCGCAGTCCGCCA
CGATGTAAATGTCCTACAGAATGTCGCGCGCGGCCTATGCCAACATGTTCGGTCCGACCGTGGGCGACAAGGT
GCGCCTCGCCGACACCGAGCTCTTCATCGAGGTCGAGAAGGATTTCACCACGCATGGCGAGGAGGTGAAGTTC
GGGGGCGGCAAGGTCATCCGTGACGGCATGGGCCAAAGCCAGGTGACGCGCGAAGGCGGCGCGGTCGACACGG
TGATCACCAATGCGCTTATCCTCGATCATTGGGGGATCGTGAAGGCGGATATCGGGCTGAAGGACGGGCGGAT
CGCGGCAATAGGCAAGGCCGGCAATCCGGACATGCAGCCGGGCGTCACCATTATCGTCGGCCCGGGCACGGAG
GTCATCGCCGGCGAGGGCAAGATCGTCACGGCCGGGGGCATGGACAGCCATATACATTTCATCTGCCCGCAGC
AGATCGAAGAGGCGTTGATGAGCGGCCTGACCTGCATGCTCGGCGGCGGGACGGGACCGGCGCACGGTACGCT
CGCCACGACCTGCACGCCCGGACCGTGGCACATCGCCCGCATGATCGAAGCGGCCGATGCGTTTCCGATGAAT
CTCGCCTTTGCGGGCAAGGGAAATGCGTCGCTGCCCGGCGCGCTCGTGGAAATGGTACTCGGCGGCGCGACCT
CGCTGAAGCTGCACGAGGATTGGGGCACGACGCCGGCCGCCATCGACTGCTGCCTCTCCGTTGCCGACGAATA
CGACGTGCAGGTTATGATCCATACCGACACACTCAACGAAAGCGGCTTCGTCGAGGATACGATCGCGGCGATC
AAGGGCAGGACGATCCACGCCTACCACACGGAAGGCGCCGGCGGCGGGCATGCGCCTGATATCATCAGGATCT
GCGGCCAGCCGAACGTCATCCCGTCCTCGACCAATCCGACGCGGCCCTATACGGTGAACACGCTTGCGGAACA
TCTCGACATGCTGATGGTATGCCATCACCTGTCGCCCACCATTCCGGAAGACATCGCCTTCGCCGAAAGCCGC
ATCCGCAAGGAGACGATCGCAGCGGAGGATATCCTCCACGATATCGGCGCCTTCTCGATCATTTCCTCCGACA
GCCAGGCGATGGGGCGGGTCGGCGAGGTGGCGATCCGCACCTGGCAGACCGCCGACAAGATGAAGCGCCAGCG
CGGACGGCTCAAGGAGGAGACCGGCGACAACGACAATTTCCGGGTCAAGCGCTACATCGCCAAATATACGATC
AACCCGGCGATAGCCCATGGCCTCAGCCATGAGATCGGCTCGCTCGAGGTCGGCAAGCGCGCCGATCTGGTCC
TCTGGAATCCCGCCTTCTTCGGCGTGAAGCCCGACATGGTGCTTCTCGGCGGCACGATCGCCGCAGCACCCAT
GGGCGACCCGAACGCCTCCATCCCCACGCCGCAGCCGGTCCACTACCGGCCGATGTTCGGCGCCTATGGAAGG
AGCCGGACCAACTCCTCGGTCACCTTCGTCTCGCAGGCGTCGCTCGATGCCGGGCTCGCCGGCAGGCTCGGCG
TCGCCAAGGAGCTCGTCGCTGTCCAGAATACCCGCGGCGGCATCGGCAAGGCGTCGATGATCCACAACAGCCT
GACGCCGCATATCGAGGTCGACCCGGAAACCTACGAGGTCCGCGCCGACGGCGAGTTCCTGACCTGCGAGCCG

GCGACGGTGCTGCCGATGGCGCAGCGGTATTTCCTGTTCTAGATGGCCTTTGGGACCGACACCATGACAAAAG
ACCCCATCCTCAAGGTTGAACGCCTGTCGATGCGCTTCGGCGGTCTCATGGCCATCAACGACTTCTCCTTCGA
GGCGGAGCGCGGCGAGATTACCGCGCTGATCGGTCCGAACGGAGCGGGCAAGACGACGGTGTTCAACTGCATC
ACCGGTTTCTACAAACCGACGATGGGCATGATCACCATGCGGCAGAAGTCGGGCGCCGAATTCCTTCTGGAAC
GGCTGCCGGATTTCGAGATCACCAAAAAGGCCAAGGTGGCTCGGACCTTCCAGAACATCCGCATGTTCTCGGG
CCTGACGGTGCTCGAAAATCTGCTCGTTGCCCAGCACAACAAGCTGATGCGGGCATCCGGCTATACCATTCTC
GGCCTGTTCGGCTTCCCGGCCTATCGCGAGGCATCCAGGGAATCGATCGAGCTCGCCAGGCACTGGCTCGAAA
AGGCATCGCTGACCGAGCGGGCCGACGATCCGGCCGGCGACCTGCCCTATGGCGCTCAGCGGCGGCTCGAGAT
CGCCCGCGCAATGTGTACCGGGCCGGAGCTTCTCTGCCTGGACGAGCCCGCGGCGGGTCTCAATCCGCGTGAA
TCGCTCGCCCTCAATGCGCTGCTGCAGGAGATCCGCCGCGATACCGGCACCTCCATCCTGTTGATCGAGCACG
ATATGTCCGTGGTGATGGAAATATCGGATCACGTCGTCGTGCTCGAATACGGTCAGAAGATTTCCGACGGCAA
CCCGGACTTCGTGAAGAACGATCCAAGGGTCATTGCGGCCTATCTGGGTGTCGAGGATGAAGAGGTTGAAGAG
GTGATCGGAGAAATCGAGGAAATCGAGGGCGAGCGTGGGGGGCCCGGGCAATGAATGAAAAAGTTGACCCTCA
ACGGACTGATCGGCGGCATCCTGATCGCCATCCTGCTCGTCACCGCTGCCGTCGGTCTCTTCTGGACACCGTT
CGACCCGATGAAACTCTCCTTCACGGCGCGGCTTGCAGCTCCCGGCCCCGCGCATCTGCTCGGCACCGACGAA
TTCGGCCGCGATGTCTTGAGCCGCCTGATGGTCGGCGCCCGCGCCAGCGTCTGGATCGGCGTGCTCACCGTCG
GCTTCGCCACTGTCTGCGGGACGCTCATCGGCCTCATCAGCGGTTATGCGCGCGGCTGGGTGGACGGCGTCAT
CATGGCTGTCAACAACGCGCTTCTCGCTTTTCCCGGCATCCTGCTGGCGCTAGGGCTGCTCGCCGTCTTCGGC
GCCAACCAGTACGGCATCATCTTCGCGCTCGGCATCGCCTATACGCCTTCCATGGCGCGCGTCGTGCGAGGCG
CCGTGCTTTCGCTGAGGGAGCGGGAGTTCATCGAGGCCTCGCGGGTAATGGGCAATGGCGAACTTTATACGAT
GTTGCGCCATATCCTGCCGAACTGCGTGGCGCCGATCACGGTTCTCGCCACCTCCATGTTCGGCTGGGCCATC
CTGTCGGAAAGCGCGCTCTCGTTTCTCGGCCTCGGCGTGCCGCCGCCGGCGCCGACCTGGGGCAACATGCTGG
CGGCCGGGCGGCCATTCCTCCAGCAGGCCGCATGGCTCGGATTGTTCCCGGGTCTGTGCATCGCGCTGACGCT
GCTTGGCATCAATCTTCTGGGCGACGCGCTCCGCGACAGGCTCGACCCGCGCATGAGAGGCCTGAAATGAATG
CGCCAGCCAGGGAGGGGAAAAGTGTACGCTGAGCTTTTCAGACTGAATGGGCGCGCCGCTTTCGTGACCGGCG
GCAGTCGCGGCATCGGCTTCGCCTGTGCCGAGGCGCTTGGCGAAGCCGGTGCGCGCGTGGCCATCTCCGCCCG
CTCGCGCGACGAAGGCGAAAAGGCTGTCCGGCAACTGAGGCAGAAGGGGATCGAGGCGATCTACCTGCCCGCC
GACATTTCCAACGAGAGCGCTGCGCAGCAGGTCGTCAGGCAGGCAGCAGCAGAGTTGGGCGGGCTCGACATCC
TCGTGAACAATGCCGGGATCGCGCGCCATTGCGACAGCCTGAAACTCGAGCCGGAAACCTGGGACGAGGTGAT
CAACACCAATCTGACCGGCCTTTTCTGGTGCTGTCGCGCGGCTATCGAGACGATGTCGGCGGCCGGGCGCGGC
TCGATCGTCAACATCGGATCGATCTCGGGTTACATCAGCAACCTGCCGCAGAACCAGGTCGCCTACAACGCCA
GCAAGGCGGGCGTGCACATGTTGACGAAGTCGCTCGCGGGCGAATTCGCCAAGAGCAACATCCGCATCAACGC
CGTCGCCCCCGGCTATATCGAGACGGCGATGACGCAGGGTGGTCTGGATGACCCCGAATGGTCGAAGATCTGG
CTCGGCATGACGCCTTTGGGCCGCGCGGGCAAGGCCAGCGAAGTGGCGGCGGCGGTGCTGTTCCTCGCTTCGG
ACGCCGCCAGCTATATCACCGGCTCGGTGCTCACCATCGACGGTGGCTACACCATCCACTGAATGGCACGGGC
ATTCGGCGGTCCGAACAAATACATCCAGCGTGCGGGCGAGATCGACAAGCTGGCGGCCTATCTGGCGCCGCTC
GGCAAACGCGCGCTCGTCCTGATCGACCGCGTGCTGTTCGACGCACTCAGCGAACGCATCGGCAAAAGCTGCG
GCGACTCGCTCGATATCCGCTTCGAGCGGTTCGGCGGAGAGTGCTGCACCTCGGAGATCGAGCGCGTACGCAA
GGTCGCAATCGAGCACGGCAGCGATATTCTCGTGGGCGTGGGTGGGGGCAAGACGGCCGACACCGCCAAGATC
GTCGCAATCGACACTGGCGCGCGCATCGTCATCGCACCGACGATCGCCTCGACCGACGCGCCCTGCAGCGCGA
TTGCCGTGCGCTACACCGAACATGGGGTTTACGAGGAGGCGCTCCGCCTGCCCCGCAACCCGGACGCCGTCGT
CGTCGATTCCGCGCTGGTGGCAGCCGCGCCGGCACGCTTCCTCGTCGCGGGGATCGGCGATGCGCTTTCGACC
TGGTTCGAGGCCCGCTCCAATATCGAGTCGCGCACCGACAATTATGTGGCCGGCGGCTTTCCCGCTACCGAAG
CCGGGATGGCGATCGCCCGGCATTGCCAGGATGTGCTGACACGCGATGCCGTCAAGGCGAAGATCGCCGTCGA
GGCCGGCCTGCTGACCCCAGCGGTCGAAAACATCATCGAGGCGAACACGCTGCTGTCGGGCCTCGGTTTCGAG
AATTGCGGCTGCTCGGCCGCCCATGGCATCCATGACGGCCTGACAGTGCTGGAGGAGGTGCATGGGTATTTCC
ACGGCGAGAAGGTCGCCTTCGGCACGCTTTGCCTGCTGATGCTGGAGAACCGCGACCGAGCCGAGATCGAGGC
GATGATACGCTTCTGCCGCAGCGTCGGACTGCCGACCAAACTGGCGGACCTCGGCATCGTCGATGACGTCCCC
GCCAAGATCGGCCGCGTGGCGGAAGCCGCCTGCCGGCCCGGCAACATCATCTATGCCACGCCGGTCACCATTA
CCGTACCGGCCGTGCGCGACGCGATCCTGGCGCTCGACGCCTTTTCACGCAGCATCCACTGAATGACCGGCAG
AAAGCCGCTCGTCATGATCACCGGCGCAAGCGCCGGCATCGGTCAGGAAACCGCACGCGTCTTCTCGGCTGCC
GGTTATCCCCTGCTTCTGATCGCCCGCCGATCCGAACTGATCGAGGCGATGGCGCTTCCGAACATGCTCGCGG
TCGCCGCCGACGTCCGTGACTATGATGCGCTCGCAAGCGCGATCCGGCAGGGTGAGGCGCGGTTCGGCCCGGT
CGACTGCCTCATCAACAATGCCGGCGTCTCCCGTCTCGCCCGGCTCGACGAGCAGGATCCTGCGCAATGGCGC
GATCTCGTCGACATCAACTGCCTCGGCGTTCTCAACGGCATGCACGCGGTCGCTCCCGGCATGAAGGAGCGGC
GCTGCGGCACCATTGTCAATGTCTCCTCCATCGCAGGCCGCAAGGTCTATCCGCACCATGACGTCTATGGCGG
CACCAAGCATTTCGTGCATGCCGTCAGCGAAGGCATGCGCCAGACCATGTCGTCATATCGCGTCCGCGTCATG
GTCATCTCGCCCGGTGTTGCGAAGTCGGAGATCGACAAGACCATCACCCACCAGGACGCCTACGATTTCTGGG

ACAGCGGCCGCAAACAGCTCGACGGCGGGCTGGATGCAAACGACATCGCGCGGACCATGCTCTTTGCCTACGA
GCTGCCGCAGGACGTCAATCTCCAGGAGATGACGATCACCCACACAGGCCAGGAATTCTGAATGATCAAGACT
TCCAGACTGTTTCTCGCAGCGGCAAGCTTCGCGCTTGCCTCTTCCACAGCCATTGCCGACGACGACAAGATCC
TGATCGGCGGCGCACTCTCGATGACGGGCATTCAGGCTCCCCTCGACACGCCCGGCTACAACGGCGCGAAGGT
CGCCGTGAAGTATCTCAACGACAATGGCGGTTTGCTGGGCAAGCAGATCGAGTTCATCAACATCGACGGCAAG
TCGGATCCGGTGACGGTCGGCAATGTCGCGGTCGAACTGATCGACAAGGGTGCTGAGATCATCGTCGCACCGT
GCGATTTCGACTTCGGTTCGCCCGCGAGCCGGGAGGCGCAATCGGCAGGCCTCGTCGGCATCTCGACTTGCGC
CTCCGACCCGCTCTATTCCTCCTGGTCGCTCGGCGACAAGCAGTTCACGCTCTCCATGTGGAATACCACGATG
GGCGCGACGGCCGCCGATTTCGCCGTGAAGGAAAAGGGCTGGAAGACGGCCTATGTCGTCACCGACCAGTTCA
TCGCCTATACCAAGTCGCTCTCGAAATATTTCGTGGAGCAATTCAAGGCCGATGGCGGCGAGATTCTGCTCGA
AGACACCTACACGAACGGCGACAACAATTTTTCCGCCCAGCTCGCCCGTCTCCAGGCGCTCGGCAAGAAGCCG
GACGTGATCTTCGTCTCCTCTTACGGCACCGACATCGGTGTGATCATCCGCGCGTTGCGCGAGGTCGGCTACG
ATGCGCCGGTTCTCGGCGGTGACGCCTATGACGACCCGGCCATGCATGAGGCGCTCGGCGAAAAATTCGGCAA
CGACGTCTATTTCGTCACGCATACCTGGATGGGCCCGGAGGCACATCCGGACATGCCGAAGTTCATCGAGCTC
TACAAGGAGATGTTCGGCAAGGCGCCGGACACGTCCTTCGTCTCGACGGGCTGGGATACGATCATGCTGCTCG
CCGAGGCGGTCAAGGCGGCCGGAACGACCGAAGGCGCAGCGCTCGCCAAGGCATTGGAAGATGGCCAGTTCAA
GCTGCTGACCGGCGATCTCGACTACGGCACGAACGAGGAAGGCCATGTGCCGAACAAGGCTGCGGCCGTGATC
GAGCTCAAGGGCGGCAAGCCGAGCTTCGTCGGATGGCGTAAGCCGGAATCCCTGCCTAAGCCCTGAATGAGCA
TCCAGACCTCTCAAACTCCGCCCGTCACGCGTCCTTCGCTTTTCCAGCGTCTGCGCCAAAGCGATGTGTGGTG
GTCGTTCACGCATAGCAAGACGGCGATGTTCAGCGCCGCGCTTCTGGCGATCCTGATCCTGACGGCACTGTTC
GCACCGCTGATCGCACCGCAGAACCCCTATGACGGCGCAGCGCTCGACATGTGGAAGGCCGAACTGCCGCCGG
TATGGGAGGAAGGTGGAGAATGGCCCTATCTTCTCGGCACCGATACGCAGGGCCGCGACATGCTGTCGGCCAT
TCTCTACGGTACGCGCATTTCCATCGTCATCGGCATCGCCTCGGTGGCGCTCTCCCTGCTGATCGGCATGAGC
GCGGGGCTGATCGCCGGCTACTTCGGCGGGTTCATCGACAATCTGCTGATGCGCTTCGGCGACGTCACGCTGT
CCATTCCGACCATCCTCGTCGCCATTCTGGTCTCGACGGTCGTGCGTCAGATGCTGCCCGTCAGCCTGCGAGA
GGTCGGCGCGTCTGCAGTGCTCATCCTGGCCATCTCGCTATCCGCCTGGGTGCAATATGCGCGCACGGTGCGC
GCGCAGGCCATCGTCGAAACCGGCAAGGACTACGTGTCGGCGGCGCGGCTGATCGGCGTGCCTGCCCGGCGCA
TCATGGCCAACCATATCCTGCCCAATACGCTGACGCCGATCATGGTGGCGGCGACGCTCAACTTCGGCATGGC
GATCCTCACCGAAGCCACGCTTTCGTTCCTGGGCATCGGCATGCCGCCGAGCCAGCCGTCTCTGGGAACGCTG
ATCCGCATCGGCAACCAGTTCCTGTTTTCCGGATCCTGGTGGATCGTGCTCTTCCCCGTCATTCAGCTCTGCC
TGCTGGTCGTGGCCGTCAACATGCTCGGCGACTGGCTGCGCGATGCTCTCAATCCGAAACTGAGGTAAATGAG
CAGCGATAAGAACATGCTTCGTTCGCTTGGCGACCACCCGCCGATCCTAGCGGACGGCCGTCGCGCACCGGAC
CGGCGCGAGATCTCGATGCGCTGGCTCTCGGGCACGTTCTTGACCGGCATCACCTCAAGTCTTCTGATGGGCG
TCGCGCTCTTTGCGGCGCTCGACGGCCGGCAGCAACTGGCGATTCCGGCGGAAGCCTTTGCCGCGCTCGATCC
GTCGGCGCCGGTCGGCGCCGCCAAACGGGGAGATCGCGTTCTTTCCCCCAACATCGTCGCCAAGCCAGCCGAC
AAGACCGTGATGGAAGTCTCGACCATGATCCATGACGGCGAGAAGGAGGTGGTGCGCCGCCAGCCCTTCGTGC
ATGTCAAGATGGCGCTTGCCGCCAACCATCAGACGTCCGAATCCTACCCCGACTTCGACCCGCTGGCGATCTT
CTCCACGGACGAGGCGGACGCCGAGGCGCCGCCGGTGAAGACCGGAACGATCTACGGCTCGGACGTCGAGTCC
GAGGTTGCCTTGAAGACCGTGGATTTTCCGCTGAACGGCGGCGGCTTCGCACTCGGGCCCTCGATGTCGCTCG
ACGAGGTCGAGGAGAACGTCCGCACCAACGGCTCGGTGCTGACCGAAGGCAATACCCAGGTCGCCTCGCTCTT
CTATGTCGACCCGCAACGCTTCGCTTCCGACGCCGACAACCTCGACCTGATGCAGGGCCTTGCCGCGCGCGTC
GTCGAGGAGAACATGAGCGTTTCCTCCTACGAGAACATCACGAAGCAGAGCATCGAATACGCCGACGACATCA
TACCGGTGCGCCGCGCCACGCCCATCGCGACGGCGATGATCAATGCGGGATATGCCAAGGCTCAGGCGGAGGA
CGCGGCCGGCTATCTCGAAGAAGCGCTCGGCGCCAAGGAACTCGGCCCCGGCGACGTGCTGCGCATCGGCATC
ATTCAGAAGGGCGAAGAGGCAAAGATCGTCCGCGCCACGATCTATTCCCATAGCCGGCACGTGCTGACCATGG
CAGTGGACGACCGCGGCCGTTTCATTCCGGGCGCCGAGCCGGCGAAGCTCGAAGCGGTGGCGGCTGCCTTCGA
CGATACCGGCAGGCCGGTGATCTCCAGCGGCCACGACCTGCCGAGGGTTTACGACGGCATCTACCGTGCGGCC
CTTTCCTACGGCATGAACGCCGACATGATCGCGCTCGTCGTCAAGCTTTTGGCAAGCAATGTCGACTTCCAGG
CTCAGCTGAAGCCGACGGACTCTCTCGAAGCGTTTTTCTCGGTCACCGATGAAAGCGGCCGGGCAACGGAAGA
TTCGGAACTGCTCTACGTCAACGCCAAATTCGGTGACGCCGAGACGCGGTTCTATCGCTTCCAGGATCCGGAC
GACAATTCGATCGATTATTTCGACAAGGACGGCAAGAGCATCCGCCAGTTCCTGTTGCGCAATCCCGTTCCGA
ACGGCCATTTCCGCTCCGGCTTCGGCATGCGCCGCCACCCTATCCTCGGCTTCTCGCGCATGCACACCGGCGT
CGACTGGTCCGCTCCGCGCGGCACGCCGATCATCGCGGCCGGAAACGGCGTCGTGCAGAAGGCCGGCTGGGAC
TCCGGCGGCTACGGCAACCAGACGCTGATCCGCCACGCCAACGGCTATGTGTCGTCCTATAACCACCAAAGCG
CGATCGCCAAAAGCGTCAAGCCGGGAACGAAGGTCGTCCAGGGTCAGGTGATCGGCTGGGTCGGAACGACCGG
CCTTTCGACCGGCCCGCACCTCCACTACGAGTTGATCGTCAACGGCAACAAGGTGGATCCCTTGCGTATCCGG
CTGCCGGGAGGAAAATCGCTCGGCGGCGAGGCGCTGGCGAAGTTCGAGAAGGAACGCGAACGCATCGACGAGC
TTCTCGGCGACGATGCGAACGAGGTCGCAAGCAAGTAGATGAATGAGAACCGTGTTCCCCGCCGCGAGTCAAA

CCCTCCGCCGCAGCAGCAGCAACGCCGCAGCGAGATGCGCGTGCGCCCGACGGGGCCGATCGTGCCTTCGGTC
AACGTTTCCGGCCATGCGCTCATGTGCGTCATCGCCATCATGTCCTTTCTCGCCTGCCTGACCCTCGGCGGCG
TCAGCATGGTGCGGGCGACCGCCCAGAGCTGGCAATCGCAGATCTCCCGTGAGATCACCATCCAGATCAAGCC
GGACGACAACCTCGACATGGAGAGGGCGCTCGCCGATGCCCGGGATCTGGCGCTGACCTTCAGCGGCACGACG
GGCGGCACGATCATCGACCGCGCGGCGACGGCACGCCTCCTGGAACCGTGGCTCGGTGGCGGGCTCGACCTGA
ACGAACTGCCCGTGCCGCGGCTCGTCGTCGTCACGATCGACGAGAGCAATCCCCCCGATTTCGCCGCGATGCG
AAAGGCGCTCACCGAAACCATCCCGCAGGCCTTCCTCGATGATCATCGCACCTGGGTCGATCGCCTGGTAGCA
ATGGCGAGTACAACCACGATGATCGGCACCGGCGTCTTGGTACTGGTCTTCTCCGCGATGGTGCTGACGGTGG
TTTTCGCCACCCGCGGCGCGCTGTCCGGCAATCGCCACATCGTCGAGGTCCTTCATTTCGTCGGAGCGGAAGC
GAGTTTCGTCGCCGCCGAATTCCAGAAGCACTTTCTCCGAATCAGTCTGAAGGGCGCCGGCGCCGGCGGCCTT
CTCGCTGCGCTGTCCTTTGCGCTGGCGAGCTTCTGGCAGTCGCAGACGCTTGCAACCCCTGAAACGGATCAGG
CGACCGCTCTGTTCGGTACCTTCGCCATCGGCTACACCGGCTATCTTGGTATCTTCGGCATTATCGTCGTCAT
TGCCCTGCTGACGACCTTGACGGCGCGCCTTACCGTCATGCGGACGATCTACGAGATCGACCTTATCCGATCG
GATCCGGGCCGGACCGACACCTTTCAGGGTTGAATGCGTTTCAGGAACCTTGTCCTCACCGCCGCTGTCGCAG
CCGCTCTTGCGGGCTGCACCAACGAGACGCTCGATTCGGTCAACCTGTCGTCCGTCAAGAACAAGACCGAGTA
CCAGCTTTCCAGCAAGATGGTCAGCAAGATGCGCGAGCTGGGGATGCAGAAGACGTCCCCGATCCTGTTGCGC
ATCTTCAAGGAAGAAGGGACGCTCGAAGTCTGGAAGGCGAACACGGCGAACCGGTTCCAGCTTCTGAAGAGCT
ACAAGATCTGCGCCTGGTCGGGAAAACTCGGGCCCAAGATGAAGGAGGGAGACCGGCAGGCGCCGGAGGGATT
CTATCCGCTCTATCCGAACCAGATGAACCCGAATTCGAGCTACTACCTGGCGATCAACACGGGCTATCCGAAC
ACCTACGACAAGGCCAACGGCCGCACCGGCACGCATCTGATGATCCACGGTGCCTGCTCTTCGGCCGGCTGCT
ATTCGATGACGAACGAGCAGATCATCGAGATTTTCGCGCTCGCCCGCGACGCGTTCAAGGGCGGCCAGGAAAG
CGTGCAGCTTCAGGCCTTTCCCTTCCGCATGACGGCGGAAAACATGGCGCGCCACCGCGACAATCCGAACATC
GAATTCTGGAAAATGCTGAAGGCCGGCTACGACCAGTTCGAAGTGACCAAGCGCCCGCCGGCAGTGAATGTCT
GCGAGAGGAAATACGTCTTCAATCAGCAGGCCGCGGGGCAGTTCAATGCGGCCGGCCAGTGCCCTGCCATGAC
GACGCCGCCGGCGCTGCAGATGGCGATGGCCAGCTACGACAAGGACTACCGGCGCGACTACGAGAAGGCATTG
AAGAAATTCGACGGCATGGTCTGGTATGAGCCGACCGAGGCCGAACGCAAGGCGGTCGTCGCCGACCAGCGCA
AGGGCCGCGAGCTTGCCTATGCGCCGACCGGCACCTCGCTCGATGCGGGCAAGCTGCTCAAGGTAAGCGATCT
CGAGAAGAAGCTTGCCGAACAGAAAGAGGCCGAGGAAGCCAAGCAGGCAACCTTGATCCAGAAGGAAGCGCTG
GAAAAGGCGGCCCGCGAAGGCAAGGGCCTGCCGGTGCCGCAGGCCAACCCGCTCGAGCGCCCCGTGGAGCAGG
CAGCGGTGCAAGCCGAGCCCGCGAGGAAGTCGCTCTGGGGTCTGTTCTCTCGCAGCGAGCCCGCGGCCGCGCC
GGCCGCACCCGCCGCCGAGGCTCCCGCGCAGCAGGGCGCAGGCCAGCCGGCCGCTCAACAGCCCGCACCGGGC
CAGCCGCAAGCCGCCGCCGGCGGGCAACAGGCAGCACCGCCGGCGCAGGACAATGCTCAGGTTGCCGCCGCTC
CCGCCGGGAATGCGCAAGCGCCGGCACCCGAACAGCAACCCGCCGAGCAGCCGAAGAAGCGCCCGTTCTGGAA
ACTCTGGGGCAATTGAATGACCTTTGCCTTCCATCCGAAGCGCCCCTTTACAGAGGACTTCCGCGCCGTCGGA
GGGGAACAGATCGAACGCGCGATCGCGATGCTGGAGGTCCAACCCGAAGGCGTGCATGAAGCGATCCACGATG
CACGCAAGGGCTTCAAGCGCCTGCGCTCGCTCTATCGCCTCGTGGCCGCGGATGCGCCACTCTTCCAGAGGCA
GGAAAACGCCCGCTTCCGCGCCATCGCACGCAGTCTCTCGACATTCCGCGACGCGGCAGCGCTTGTCGAGAAT
GCCGGGTATCTGCGCCGGCACGCGGCGAGCGAGGAGCAGCAGCTGGCCCTCGACAAGATCTGCTCGATCCTCG
CAAGCCGGCGCGACCGGATTGCCGAAGACGAAGGCGATCTCGACCGGAAGATCGAAGAAACCATCGTCAATTG
CCGAAAGGCGCACGCCGCACTCGCTCATGTTTCCTTCCATGACGGAAGGCGGAAATCCGCCCGCCGCCTGGCG
AAGGGCTGGCGGCAGACATTGCGGCGCGCCGCACGCGCCAGGGCGGCCTGTGCGGCAAGCTCCGATACGACGC
TGTTTCACGACCTGCGCAAGGGCGCACAGGATTACCGGATGCAGCTCTCCCTGCTTCGCGAGGCCTGGCCGTC
GGCAATGCGGGCGAAGAGAACCGAGGCGAGCGAACTGGTCGATGTGCTCGGCCATCTGAACGACATCGCCGCA
TTGATGTCGCTCGTCGACGAACGGCCGGACCTGGCGGGCAACAGCAGGGAACATCTTCTTTCGAATGTCGCGG
CCAGGCAGGACGAGCTGAGGCGTGAGGCCTTGAAGCGCGCGGATGCGGTCTTTCTCGACCGGCCGCGGAGGGA
GAGCCGAACCCTCGAACTGCTCTGGCTCGAAGCCGGAAGGTAGATGCACAAGGTTATTTTCGATACGGATCCG
GGCGTCGACGATGCGATGGCCCTCCTCTTTCTGCACCGTCACCCGGCGATAGAGCTGGTCGGCATCACCACGG
TCTTCGGCAATGCCTCGATCGAGACGACGACGCGCAATGCGTTGTTCCTCAAAGGGGCATGGAATATTGCCGC
ACCGGTCGCCAAGGGCATCGGCGAGACGCTCGATGCCGGCCGGCCGCATGTCGGATGGCCGACAGGCATTCAC
GGCGATGACGGGCTCGGCAATATCGATGTGCCGGCGCAGATAGACCTGCCGCTCGACCCTCGTCCTGCCCACC
GCTTCATCATCGACACGGTGCGCGCCAATCCCGGGCAAGTGACGCTCATCGCCGTCGGCCGGATGACCAACCT
GGCGCGCGCATTGCGCGACGATCCGGAAATCGCCACGCTCGTCAAGGCGGTAGTGATCATGGGGGGCGCCTTC
GATGTTCCCGGCAACATCACGCCGGCGGCGGAAGCGAATATCCACGGCGACCCGGAAGCGGCCGATGTGGTGA
TGACGGCGCCATGGCCGGTGACCGTCATTGGTCTCGACGTGACGACGAAGACGGTGATGAGCCGTGCCATGCT
CTCCGATATCGCCGAACGCGGCGGCGCACCGGCGAGGCTTCTTTCCGACATCTCGCAATTCTACATCGACTTC
TACGAGCACCATGTGGATGACGGCATGATCGTGCACGACAGCTGCGCCTGCGCCTATGTCGTCGCGCCCGAAC
TCTTCCAGACCCGCAGCGGCGCCATCCGAGTGCTTTGCGGCGGCATCGCCGATGGCCAGACCATTCAAAAGCC
GGATGCACGCCTGTTTCCACCGAACGCCTGGGACGGCTTCCCAAGCCAGCAGGCGTGCATCGGAATCGATGCC

GAAGCGGTGCTGAAACTCATCGGCGATACGCTCGCGGACGGCAATTGAGTGATCGGTATCGGAGAAGACGGTG
TAGCCGGTCTCGGCGACGAGGCCAAGCGCCTCATTGAGGCCGCTCCCGTCGTCTTCGGCGGGGCCCGCCATTT
GGAACTCGCTGCGCCGCTGATCGGTGGCGAACGCCATATCTGGCAGAGCCCGTTTGAAAGATCGGTGGAGGCG
ATCGTCGCCCGTCGCGGCAGCCCCGTCGTTGTGCTCGCCTCCGGCGATCCGTTTCTTTACGGCGTCGGCGCCA
CGCTGGCGCGCCGCGTAGATGCCGCGGAAATGCGCACCATCCCCGCGCCGTCCGCCTTCAGCCTCGCGGCTTC
CCGGCTCGGCTGGCCGCTGCAGGAGGTCGCGACCGTTTCGCTGCACGGCCGTCCGCTCGATCTCATAAGGCCG
CATCTTCAGCCGGGCGCGCGCGTCCTGACGCTGACCTCGGACGAGAGCGGGCCGAAGGCGCTTGCCGGGCTGC
TTGCAGAAAGCGGATTCGGTCAGTCACGGCTTACGGTTCTCGAGGCGCTCGGCGGCGCAGGCGAGCGTGTCTC
CCGCCACCTCGCTTCCGGCTTCGCGATGGAGGAAATCAACGCGCTGAACGTCTGCGCGCTTGAGGTGGTGGCC
GATGCCGATGCACGCGTTCTGACGCTTGCGAACGGCCTGGACGAGCGGCTTTTCGAGCACGACGGCCAGATCA
CCAAGCGGGAGGTGCGGGCGCTGACCCTCTCGGCACTCGCGCCGCGCAAGGGCGAACTCCTCTGGGACATCGG
CGCCGGATCAGGCTCGATCGCGATCGAATGGATGCTTGCCGATCCGGCGATGCGCGCCGTCGCCGTAGAGGCG
TCGTCCGAACGGGCTGCGCGGATCGGCCGCAATGCGGTCCGCTTCGGCGTTCCGGGGCTTGTGGTCGTCGAAG
GGCAGGCGCCGGAGGCGCTGCGAGGGCTTCCGCGGCCGGACGTGATCTTCATCGGCGGCGGCGGCAGCGAGCC
CGGCGTGATGGAGGCGGCGATTGCAGCGCTCGCGCCCGGCGGACGTCTCGTGGCCAATGCCGTGACGACGGAG
ATGGAAGCGGTGCTGCTCGCCCGACATGCGCAGTTCGGCGGCTCGCTGATCCGCATCGACATCGCCCGGGCCT
CGCCCGTCGGCGCCATGACGGGCTGGCGGCCGGCCATGCCGGTCACGCAATGGTCGTGGATAAAGGCGTGAAT
GGAGCTGCTTGACGGATTCGATCTCAAGATCCTGAGCGAACTGCAGCGCGACGGTCATCTGACCAACAACGAA
TTGTCCGAGCGCGTCGCGCTGTCTCCTTCGCAATGTTCGCGCCGGCGTACGCGCCTGGAGGCGGAAGGCTTCA
TCACCGGCTACCAGGCGCAGATCGACCGTCAGAAGCTGGGGCTCGATCTGATGGTGGTGATTTCGGTCACGCT
GGCCACGCACAATCGCGACAATGCCAAGCGTTTCGCCAATCTCGTTTCGGCTCTGCCGGAGGTGCTGGAGGCC
TACGCGCTGACTGGTGAAATGGACTACCATCTGCGCGTCGTGACGCCGGATCTTGCCGGGCTCTCCCGCTTCG
TCAACGAGGTGCTCCTGCCGCATGACAGCGTGCAGCACGTCAAGACATCGATCGTACTGGAGACGCTCAAGAG
TTTCGAGGGGCTGCCGATACCGGCGCGGATGAAGGGGTAAATGCTTTCAATCAATCGGCGCGGCGCGCTGGGC
CTCATGGGCGCGACTGCGGGCAGCTTGGTCCTGCCGCGCTTCGCGTTCAGCCAAGGCAAGCGCCCATCCGTCA
CCATCGCCGTGCAGAAGATCACCATCAACAATACGCTCGACGTCTGGAACGAGCAGTCGAATGTCGGTGAGCG
CGTGTTCTTCCCAAACCTCTGGGAGGGTCTGATCCTGCGCGACTGGATGGGCAATCAGGGTCCGGTTCCCGGC
CTTGCGACGGAATGGAAGCGTATCGACGACAAGACCCTCGAGCTCAAGCTGCGCCAGGGTGTGAAGTTCCACA
ATGGCGACGAGCTCACGGCCGACGACGTCGTGTTCAGCTTCTCCGCGCAGCGCGTGTTCGGGGACACCCAGCC
CGCAGGCGGCAAGACGGTCTTCGAGGATGAGCACAAGCCGGCCACCGCCAAGGAACTGCCGGCAGTCGTGCCG
GGCACCGGGCGCCGCCTCTGGCCGGCGCTTGCCGGCGTCGAGGCCGTCGACAAACATACCGTTCGCTTCCACA
ACGCGACGCCGGACGTCACCATCGAGGGCCGCCTTTATGCCTTCGGCAGCCAGATTGCCAACCGTCGCGCCTG
GGACGAGGCGGCGACCTATATGGACTGGGCACGCAAGCCGATCACCACCGGCCCCTATATGGTCGGCGAACAT
AAACCGGACGTTTCCTTGACGCTCGTCGCCTTCGACGACTACTGGGGCGGCCGCCCGCCGCTGGAGCAGATTC
GCTTCGTCGAGGTTCCGGAGGTTTCGTCGCGCGTCAACGGCCTGCTCTCCGGCGAATACGACTTCGCCTGCGA
CCTGCCGCCGGACCAGATCGCCGCGGTTCAGGCCGCGCCCGGATATGAGGTCCAGGGCTCGACGATCCACAAC
CACCGCATTTCCGTCTTCAATGTACAGAACCCGACGCTTCAGGACCCGCTCGTCCGCCGGGCGATGACCCATT
CGATCGACCGCCAGGCGATCGTCGATGCGCTCTGGTCCGGTCAGACGACCGTGCCGGCCGGCCTGCAGTTCCC
CTTCTACGGTGACATGTTCGTCGAAGGCTGGACGGTTCCGGAATATAATCCGCAGCTCGCCAAGGATCTCCTG
AAGCAGGCCAATTACAAGGGCGATGCGATCCCGTTCCGACTTCTCAACAATTACTACACGAACCAGACTGCGA
ACGGCCAGATCATGGTCGAGATGTGGAAGCAGGTCGGCCTCAATGTCGAAATCCAGATGAAGGAAAACTGGGG
TCAGATCCACGACCCGTCCGGCGCGAAGGGCGTGCGCGACTGGTCGGCCGGCGCCGCCTTCAGCGATCCGGTC
TCCTCGATCGTCGCCCAGTTCGGGCCTAACGGCGAGGTTCAGCAGAAGAAGGACTGGGCAAATGCCGAGGCCA
ACCAGATGGCCCAGATCCTCGAAACGGAAACCGATCATGCGAAACGCAAGAAGGCATTCGCCCGCATGCTCGA
GATCTGTGAGCGCGAGGACCCGGTCTACCAGGTGCTTCACCAGAACGCGGTCTTCACCGGCATGAAGTCTTCG
TTGAACTGGAAGGCGGCGCCCGCCTTCGCCATGGACTTCCGCGCCGCCAACTGGGCCAGCTGAATGAATCAGG
TCACCCTTTTCCTTCGATCTGCGCTGCGGTACTTCTTCGCCGCTCTCGGCACTCTCGTGGTCGTGCTCCTCCT
GCTCGTCGGCTTTCTGGGCTTCACGGTGCCCGGCGCCCGCGTTGTCGCCTGGGCGATCGAGAAATATGCGGCG
ACCCCCGACCAGATCGTACGCATCGCCGACCCGGGCGCGCTTCTGACCGGCGACTTCACGGCCGGCACGATTA
CGCTCTTCGATGGCGAAGGCATTTATGCCGAGGTCCGCGACCTTTCGGTCGACTGGTCGCCGGCCGAACTATT
CTCGCTTCGCTTCGACTCGAGCAGGATTTCGGCCGGCTCGGTCCGCGTCGAGCGGCTGCCGGTCCCCTCGACC
GAAACCCGGGAAGTGCGCTCGACGTTTGCCCTTCCGGTTGACGTGAAGATCGATGCCTTCGATCTCAAGGAAA
TCGTGCTCGGCAAACAGATTGCCGGCGAGGATCATTTCCTGACTGCCAGAGGCAAGGTCGACGCGACGAATTC
CAGCATCGCTCTCGTGCTCGATGCCGCCGAACGGGACCGCCCGGATGCGCGCGCGACCGCCGATCTCGTCTTC
AACCCGGCCGGCAACCAGCTGAAGCTTGAAGCCGAGCTCGCGGAGCCGAAGGCCGGCCTTCTGGCGAGACTGC
TCCGCCTGCCGGGAGAACCTGCCGTCAACATGACCGTGACCGGACAGGGACCGCTTTCCGACTGGGCGGGCTC
GGGTACGGTTACGCTCGACGGAAGCGAGATCCTTCGGCTCGACGGGCAGCACGTGCTCGCGGCCGAGGGAATG
CACATGCTTACCGTATCCGGCGGCGGCGCTTTCGCCACGCTGATGCCACCGGCCTTCAGGCCTCTCTTCGAAG

GGGAGAGCAGGATCGACCTGACGGCCGCCTTCGACGGCACGAGCAAGGTCCGGATCGATGCAGGCAAGGTTTC
GACCGGCGCCATGACGCTCGATGTCTCCGGCACGGTCGACAGCCGCGGCGAGAACAACCTCCAGGCAAGGCTC
GCCGGTACGAGCGGGCCTTTCGATTTCCGCTGGCCGCTGAAAGACGGCGAGTTGCAGGCGCTTGTCGACACCG
CAAACCTGTCGCTGATCGGCGACGGGCAGTCGGCAATCCTCGATATCGCCGCCAATGTCCCGTCCGTGAACCT
GCCGCAGGGACGGCTCGGCGGCATCCGGCTGTCGGCACGGAGCGACGCGTTCAACCTGGAGAGCCGGTCGGGT
CCCCTGAAGACGACCGTCGAAGTCGGCGAGAGCCGCTTTGCAAATGCCGAGCTCGATCGGGCGGTGCAGGCGC
CCCTTAAGCTCGACGGCACCATCGCCGTCACCCCGGAAGACATCCGTTTCGACCCGCTGGAGATCGAAAGCGC
AAGCATCGGCGGCACGGTCACCGGCAGCTTCGATCGCACCGAAACGACGCTCGAGGCGGCATTCAAGGTCTTT
GCCGTGCGAGAGGTCCTCCCGCCGGGGCTCGCCGACAAGTTCGACAGAACCATCGCCCTTGCGGGCAACGCAG
TCGTGGAACAGGACGGCAGCGTCCGGCTGAGCGAACTCGACATCAAGTCCGGTGCGGTCGACGCCTCCGGCTC
GGTTGCGCTTGCCGGAAGCACTTTGAGTGCGGACATCCAGGGAACGCTGCCCGAACTCGGACGGCTGCTCGCC
GATGCGGAGGGCTCGGCCGCCTTCCACGCGGTCGCCTCCGGCCCACTGAACAAGCTCGGCATCGAAGCAGAGA
TAACCTCAAGCGGAGCGACGCTCGCCGGTCGCACATTGGACGATCTCGTCGTCAATGCCGATGCCACCGTGAC
GCCAGGGAGCCCCGAAGCGAAACTGACGGCAACCGGCACCCTCGACGGTCAGGCGATCGACGTCCGCGCCGAC
GTCGTCACCCGGGACGGCCGCACCTCGATCCCCACCCTCGAGGCAAAGATCGGCGACAACCGCCTCATCGGTG
CGATGGAGCTTACAGCCGACTTCAAGCCGAACGGGACGATCGACTTCAACCTGCCCGATCTCGGCCTCCTTGC
CGCCATGGCCGGGCAGCAGGCATCCGGCGATCTTGCCGGCTCCGCGACGATCCGGACCGCCGACGGCGTCACG
TCGCTCGCGCTGACGGCCCGCGGCGAAAGCATCAGGCGTGACGCATTGACCGTTGCCAAGCCCGTGGTCGACG
TTACCGTCGCCAATCTTGCCCGGCTGGCGATCAAGGGCGGTGTCCGGGCCGAAAGCGTCGTTCAAGGCGAAAA
CCGCCTGACGGACATCGATATCGCCTTCGAGCAAGAGGCGGAAAGAACGGGTTTCTCGGTCGGTGCCGAATTT
GAAGGCGCTCCGCTTGCGGCGACCGGCGATCTCGCGAGCCGTGACGGCCGGACCGAGATAAGCCTTGCATCGG
CCTCGGCGACAGCCAGGCGCATCCCCCTGCAGCTTGCCCGGCCGAGCGTCATCGCCATCGAAAACGGCGTTGT
GCATATCGATGGGCTGACGATCCAGGCATCGACGGGCACGGTCGCCGTGACCGGTACGGCAGGCGAGACCCTC
GATATTTCGGCGGATATCAGCGACCTGCCCGCCACCTTGATCCATACCGTCGCGCCGACGCTCGGGGCCGAGG
GCAAGATTACAGGCAAGGTGGACATTGCCGGGAATGGCGCGGCACCCGTCGTCCGCTACGATCTCACCTGGAG
CGGCGCCTCGATCGCCTCCGTGCGGTCGGCCGGCATAACGGCAGAGGACCTGTCGGCGAAGGGAACGGTGACC
GTCGCTGAGGACAGCATCCGCCTCGACCCCTTGGCCATCGAACGCGCGGGCTTTCGCGGGGACGTGACGGCCA
GCCTCGACCGCGCCGACAACACGGCCGAGGCGAATTTCCAGTTCGTTGCCGAACCCGCCGTGCTGCCGCCCGG
CATCGGCGCAAAATTCGATGCGCCGATCACCGTCTCCGGCAAGGTCGAGACAGGCGCCGGCGGCACCGTCGAA
CTGAGCGCGCTCGAAGTCAAATCCGATACCGTCAACGCCACCGGTTCGGCCGCACTTGCCGAGGGCGCGTTGA
CGGCCGAGATAGCGGGCGCATTGCCGGATCTCGGCAAGCTGCTCGCTGAAGCCGAAGGCAAGGCCGCCTTCAG
CGCCGACCTGTCCGGGCCGCTCGACGCGCTCGGCGTCAAGGCGGAAGTGACCTCCAGCGGCGCGGTCCTTGCG
GGACGCACGCTTTCGGATTTCGTGATCACCGCCGATGCAACGGCGAAACCCGGCAGTCCGGAGGCGAAGCTGA
CGGCGACGGGGGCTCTGGACGGCCAGGCGATCGACGTGAAGGCCGAGGTGGTCTCGAAGGACGGCCGCACCTC
GATCCCGACCCTGGAGGCGAAGGTCGGCGACAACAGATTGACCGGTGCGATCGACCTGACGGCAGACTTCAAG
CCCGAGGGCACGATCGACTTCAATCTCCCCGATCTCGGCCTTCTTGCCGCCATGGCCGGCGAGAAGGTCTCGG
GAGACCTCTCCGGTTCGGCAGCGATCAGAACGGCGAACGGCGTGACCTCCCTTTCGCTGAAAGCGAACGGCAG
CGGCATCAAGCGCGGCGATCTGACGATCGCAAAGCCGGTCGCCGATATCAGCATCGCCGATGTGGCGGCACTC
GCGGTCAGGGGAAGCGTCAGAGCGGAAAACGTCGCTCAGGGTCAAAACCGTGTCACCGGCCTCAATCTCACCT
TCGATCAGCAGGGCGGCAGGACCGGCTTTACCGTCGACGGCAGATATGACGGCGGTCCGCTTTCGGCAAAAGG
CGACATGGTCGCAAGCGGCGGCCGCACCGAAGTCCGCCTCGCCTCTTTCGCGGCAACGCCCAAGCGGATACCG
CTGAAGCTTGCACGCCCGACCATCGTCGCGATCGAGAACGGCACGGTGCTCCTCGATGCCCTGACCATCGAGG
CTTCCAAGGGCACCATCACGGTCGCCGGCTCTGCCGGACCGACGCTCGACATTTCGGCAAGACTCAATGCGCT
GCCTGCCGCACTGATCAACACCTTCGCCCCGACGCTCGGGGCGGAAGGCACGATCGCCGGGACGGTGGACGTG
GGCGGAACGGCAGCGGCTCCGGTGGTCGTCTACGACCTCAGATGGAGCGGCGCCTCGGTCGCAGCAGCGCGCG
GCGCCGGCGTATCGTCGCTGGAGATTGCTGCAAAGGGGCGCTTCGCCGACAACCGGGTGACCGTCGATGCCAC
GGTCTCCGGGCCGGGAGACCTTTCCTTCCGGGGCGGCGGCAATGTCGAGCTTGGCGGCAACATGCCGATCTCG
ATGAAATTCGCCGGCGACGTACCCTTCGCGCTGCTCGCCAATATCATGGCGGAACAGGGCTTCACCCTGACCG
GATCGGCCAAGGTCGACCTTTCGCTTTCCGGCCCGGCAAAGGCGCCGCAGATTGCCGGCACGGTCTCGACCGC
CGGAGCCCGGCTCGTGGACGTTCGCCGCAATCTGGCCCTCAACGACCTCACCGCCAATGTCGCACTCGACGGC
AGGCAGGCGACGATCTCTCGCCTCTCGGCCAATCTCGCCAGCGGCGGATCGATCGAGGCGAGCGGAACCGTGG
GCATCGTCCCCGGCTCCGGTTTCCCGGCGAATCTCAGAATCCGGCTCAACAATGCGACCTATGTCGACGGCAC
GCTGTTCACCGCCAACCTTGCAGGCGACCTGACGCTCAACGGCCCCCTCGTCTCGACTCCCGTGCTTGGCGGA
AGGCTGACGATCCGCAGGGCCGCGATCACCATACCGGAGAGGCTGCCGGCTTCGCTCTCGCAGATCGACATCA
AACACCGCAACGCCCCACCCCGGGTGCGGCAAATGACCAGAGACCTGCGCCGGGATACGTCCGCCGGCACCGA
GGCAAGCGGCGCGATCGCCTTCGATCTCGCCGTCAGTTCACCGGGCCAGTTCTTCGTGCGCGGACGCGGAATC
GATGCCGAACTCGGCGGCGATCTGACCATCCGCGGCACCGCCGTCCAGCCGATCGTTTCCGGTGGGTTCGACA
TGCGCCGCGGGCGCCTGGAGATCCTCGGCAAGCGGCTGACCTTCACCGACGGTCATATCGGTTTCGGCGGCGA

CCTCATTCCGACGCTCGATCTCGACGCGACCTCGAGCGCCGGTTCGACGACGATCACCGTCAACGTCGCCGGC
CCGGCCAACAACCCGACGGTGACCTTCTCCTCGTCGCCGGCCCTGCCGCAGGACGAGATCCTGGCGCAGCTCA
TCTTCAACCGGTCGCTGAACAATCTTTCCGCCTTCCAGATCGCCCAGCTCGCCTCGGCCGTGGGCCAGCTTGC
CGGCGGCGGGTCGACCTCGCTCCTCGACGGCCTGCGCAACAAGCTCGGCGTCGACGATCTCGACATCACGACC
GACGAGAGCGGCGGCGCCCAGGTCCGCGCCGGCAAATATCTGAACGACCGCACCTATCTCGAACTGCAACAGG
GCTCGGACTCCGCTTCCAGCAAGGCGGTCATCAACCTCGACATCGGCCGCGGCGTGAAGCTGAAGGGCGAAGC
GGCCGGCGACGGTTCGGCCGCCGGAGGCATCTTCTTCGAGAGGGAATATTGAATGGACGTGAGAGAATTGGAC
CTGCCACCCGCCTTCCGCGGCCTGCTTTCGGGCTACGGCTTCGAGAGGGACGCACTGGGCCAGTCGGCATCCA
CCATCTTTCTCCTCAAGGCCCAGAGCCGCCCTTTCCTTATCCTGAAGCATGAACCGTCAGGGCCGTTCACCGA
GTTGACCGGCGAGGCGGCGCGCCTGCACTGGCTCGCAGCCCAGGATATGCCGTGCCCGAGAGTTCTCGGCCAT
GACGATCATGCAGGAGAGACCTGGCTGTTGCTGGAGGCGGCCGAGGGCATGGACCTTGCCTCCTGCCGGCTGC
CGCACGCCGAACGCATCGCCATTCTCGCGGAAGCCCTTCGCCGGCTGCACCGGCTCGATCCGGCGGCTTGCCC
GTTCGACCGACGGCTGACCGCCAGCATTGCTGCGGCAAAAGCGCGGATGGAGGCCGGCCTTGTTGACGAGAGC
GATTTCGACGAGGAGCGGCAAGGCAGGCAAACGAGAGAACTCTTTGATGAGCTTCTCGCGCGCAGACCGAAAG
AGGAGCAATTGGTAGTCACCCATGGCGACGCCTGCCTCCCCAACTTCATCGCAGCCGGAAATCAATTCTCCGG
CTTCATCGACTGTGGCCGTCTGGGCGTCGCCGACATCTACCAGGATCTGGCACTCGCCTGCTGGAGCATCACC
CACAACCTGGGAGAGGAATGGGTCGCCCCGTTTCTGGATCGGTATGGGCTGGCCGCGCCAGACCCTGAAAAGC
TCGCCTTTTACCGGCTGCTGGACGAATTTTTCTGAATGATCTGCGGCCGCAACGTTCTTTTCGCTTCGCTTAT
GCTTTCGGCAGCACCGGCGCTTGCGGACGAGTGCACCCAGGCGCAGGCGGTCTATGCCGATCCGGCCGGAACC
TATGAGCTGCACTTCGAGCCCGTCGGATCGGAGGCGGCAGTCACCAGCAATCACTTCAAGGTCAGGATCGGGG
ACACCGGGGTTTTGCTCGATGGCGTCGTGATGCAGTCGGGCGAACCACTGCGGGCGAACGGGATCGTCATGAA
CGATTGCCCGACCGGCGACGTGACCGGCGCGGAACTCGAGGCCTGCACCGTCTGGCAGGGCCCGATCTATGCG
GTCGACAAGGCCGGCCAGATCGGACTGCTTCAGCCGGAGGACGCGCCCGCAGCCGATCGGATCCTGCTTCCGG
ATTTCGGACCGTCGCTGCGCGAATCGAGCGCCTGGGGCGAAGGCAAGGGTACCACCGACAGTTCCGATGTCTT
CCAGTTCAAGGGGTGTGGATCATGAATGAGCTTGCACACCGGTTTTACTTCCATCGTCGGCTTCGACTCGGCC
TGGACTGACAACCCGAAAGCTCCCGGAGCGATCTGCGTCATTCGTCTGGATGGAGACGGCGCTCATTTGTATC
TCGCACCCCGGCTTGCCTCGTTTGCGGAGGCTCTGGATGTCATCGAGCGCGAGCGCAAGGAGGCCCAGAAGTG
CCTTGTGGCACTGGATCAGCCAACGATCGTCCCGAACCTCACCGGCTCGCGTCCCGTCGACAGGGTAGCCGGA
TCGCTGATCTCCTGGATTGGAGGCGGCGTTCAGCCTGCAAGCCGGTCGAAGAAGGGTATGTTCGATGACGGCG
CACCGATCTGGCGGTTCAAGCAGACCTTGGCAGCGACCGAAGAGCCGGAACTCGCACGAGCAGCATCCAGCGG
CCTCTTCCTGATCGAGGTTTTTCCGGCTCTGGCACTGGCTGCTATCGAGGGAACCTTCTGTTCGAGGCTTGCA
GCCCCGAAGTACAATCCTGCCAACCGGAAGCGCTTCAAGATCAGCGACTGGCAGGCAGTCGCAGAAGCGGTCA
GGCGGTTCGGCATGCTGAACTCGCTGCGGCACCTCGACGAATGGTGCTTGATAGCGGCAAGCTCCGAGGCGCC
GAGGAAGGCGGACCAGGACAAGGTCGACGCATTGATCTGCGGCCTGATCGGGCTGCATTGGCTTGCCGCTCCG
CGTGACCAATCGGTGATGATCGGTGACCTGGAGAGTGGCTATATGATAGCGCCGGCGACGCGCGGCGTGCATG
AGAGGTTGCGCGCAGCGGCGCGACAGCGGGATGTGGCGATTGCGTGAATGGCCGTCGGCCTGTTCGGCGGGTC
CTTCAATCCGCCGCATGACGGGCATGCGCTCGTCGCCGAGACCGCATTGCGCCGGCTCGGGCTCGACCAGCTC
TGGTGGATGGTGACGCCCGGCAATCCGCTCAAGGACCGCAACCATCTCGCGCCGCTTGGCGAACGTATCGCCA
TGAGCGAGAAGATCGCCCGCAACCCGCGCATCAAGGTGACCGCTTTCGAGCAGGCGCTCGGTCAGAGCTACAC
GGCCCGCACGCTGGAAGTGATCCGCGCCCGCAACCGCGATGTCCGTTTCGTCTGGGTCATGGGCGCCGACAAT
CTCAAGAACTTTCACCGCTGGCAGGACTGGCGCAAGATCGTCGCCACTTTCCCGATCGCCGTCGTCGACCGGC
CGGGATCGACCCTTGCCTATCTGTCCTCGCCGATGGCGAGGGCTTTCAGCAGCGCCCGCGTCGACGAGGATGA
TGCGGGAACCCTCGCTTTCCGCCGCGCGCCCGCCTGGACCTTGATTCACGGCCCCCGTTCGGGCTTGAGCTCG
ACGGCGCTTCGTTCGGCCAAATCCGGGTGAATGAGCGCGGATGCTGTCTTCTGCGAGCTCGGTGCGCGCACGA
ATTTCTCCTTTCTCGAAGGGGCGGCGCCGGCCGAGGAGATGGTCGTATTCGCCAAGAAGGCGGGGCTTGCCGG
TCTCGGCATTGCCGACCGCAACAGCGTCGCCGGCGTCGTCAGGGCTCATGCCAAGGCGAAGGTGGAGGGCTAT
CCCTTCCAGCCGGGCGCCCGCCTGGTTTTTGCCGACGGCACGCCGGACATTCTCGCTTATCCGAAGAACAGGC
GGGGCTGGGGGCATCTCTGCCGCCTGCTCAGCGCCGGTAACCTGCGCTCGAAGAAGGGCGACTGCACGCTCCA
TCTCGCCGACCTGCTCGAATGGCAGGAGGAGCTTCTTCTGATCGTCATGCAAGGCGAAGGGCGGCCGGAACCG
GAGAGCCTGGAAGTGCTGCTCGGGACATTGAAGGAGCATGCGGGCAACCGGCTCTATCTCGGACTGGCGCCGC
ATTATGACGGTTTCGACCGGCATGATTTTGCCGTTCTGGCCGCAATTGCCCGAAAGGCCGGCATCGGGTTGCT
CGCCACCAATGACGCGCTCTATCACGATCCGCATTACAGGCCGCTCGCGGATGTCGTCACCTCCATCCGGGAG
CATGTGCCGATCGCCGGCGCCGGTTTCCTTCTGCAGAAGAATGCCGAAAGGCATCTGAAGGGCCCAAGGGAAA
TGGCCAGGCTTTTCAGCGACTATCCCGAGGCGATCGCCAATACCCGGAAATTTTTCCGCGAGCTCGCCTTCAG
CCTCGACGAGCTCAGCCACCAATATCCGGATGAAAACGCCGATGGCGAGACGCCCGCCGAAAGCTTGCGGCGG
CTCGTCGCGGAAGGTGCGGCGGAGCGCTATCCCGAAGGCGTGCCGGAAAAGGTGATGCGCCAGATCGACTATG
AGCTCGAGCTCATCCACGACAAGAAATACGAGCCTTATTTCCTGACCGTCCACAAGCTCGTGAAATTTGCCCG
CAGCGTGAATATTCTCTGCCAGGGGCGGGGCTCGGCGGCCAATTCCTCGGTCTGCTTCTGCCTCGGCATCACC

GATGTCGATCCGCAGAAATTCACCCTGCTGTTCGACCGATTCCTGTCGAAGGATCGCGACGAGCCGCCCGATA
TCGATGTCGACTTCGAGCACGAGCGGCGCGAAGAGGTGATCCAGTATATCTACAGGACTTACGGCAAGGAGCA
TGCGGGCCTGACCGCCGCGGTGATCAGCTATCGCTCGCGCTCCGCCGGGCGCGAGGTCGCCAAGGCCTTCGGC
CTTTCGGAAGACGTTCAATCGGCTCTCGTCAGTTCGATCTGGGGCTGGGGCACCTCGCCTTTCACCGAGGAGC
AGGCGAAAGGGGCGGGGCTCGACGCGGCGGACCCTCTCACCAGGCGCGTGCTTGCCTATGCCAGTCTGCTCAT
GAATTTTCCGCGCCACCTCTCGCAGCATGTGGGCGGCTTCGTGATTACCCGCGACAGGCTCGACGAGGTCGTG
CCGATCATGAACACGGCCATGCCCGACCGCTACATGATCGAATGGGACAAGGACGATCTCGATGAGCTGAAGA
TACTGAAGGTCGACGTGCTGGCGCTCGGCATGCTGACCTGCCTTGCCAAGGGTTTCAAGCTTCTGGAAGCGCA
TTACGGCGAGCCGATAACGCTGGCCGAAATCTACCAGGATCATCGGGACGCGGTTTACGACATGATCTGCCGC
GCCGATACGGTCGGCGTCTTCCAGATCGAAAGCCGGGCGCAGATGAGCATGCTGCCGCGCCTGCAGCCGCGCG
AAATGTACGATCTGGTGATCGAGGTAGCGATCGTTCGCCCCGGCCCCATCCAGGGCAATATGGTGCATCCCTA
TCTGAAACGGCGCGAAGCCCAGCGCAGGGGCGAGGCGGTCGTGTATCCCAGCCCGGAGTTGAAGGCGGTCCTG
GAAAGGACGCTGGGCGTGCCGCTGTTCCAGGAGCAGGCGATGCAGATCGCGATCACAGCGGCCGGTTTTTCGC
CCAGCGAGGCCGACCGCCTGCGCCGCGCCATGGCCACCTTCAAGCGGACCGGTACGATCCACACCTTCGAGCG
GAAGATGGTGGAGGGCATGGTCGCGAATGACTACGAGAGGGAATTCGCCGAGCGCTGCTTCAACCAGATCAAG
GGATTCGGCGAATACGGCTTCCCCGAAAGCCATGCTGCCTCCTTCGCCTCTCTCGTCTATGCCTCGGCCTGGC
TCAAGACCTATTATCCGGACATCTTCTGCGCCGCGCTCCTGAACGCACAGCCGATGGGTTTCTATGCGCCGGC
GCAACTGGTGCGCGATGCCCGTGAACACGGGGTCAGAATGCTGCCGGTCGATATCAATCATTCGGATTGGGAT
GCCCTGCTCGAAGGCGAGGGCGCCTTCGACAAGAATGCTGTCCACCCGCGCCACGCCAGCATGCGGGAGGTGA
TCAAAACCCGGAAGGCCGTGCGGCTGGGCTTCCGGCTGGTCAAGGGCCTGAAGCAGACGGATATGAAGGCGCT
CGTCGCGCGCCGGGGGGAGGGATACCGCTCCGTTCATGACCTGTGGCTTCGATCAGGGCTCTCCCGCTCCGTT
CTGGAGCGTCTTGCGGATGCCGATGCCTTCCGGTCGATAGGCCTCGACCGGCGGGCGGCCCTCTGGGCGGTAA
AGGCGCTCGACGAGCAGTCGGCGGTGGAGCGATTGCCGCTTTTCGAGGGGGCGGGTTCCGACGATCTGCAGAT
CGAGCCCAAGGTCGCTCTTCCCGATATGCCGGCAGGCGAACAGGTCATTCATGACTATCGCACCCTGACGCTC
TCCCTGAAGGCGCACCCCGTTTCCTTCATGCGGGAGGATTTTTCGCGAATGGGTATCCTGCGCAGCCGGGATC
TGGCGGCAACCGCAACTGGAAGATGGGTGACGGTGGCAGGCCTTGTACTGGTCAGGCAGCGGCCGGGCTCTGC
CAATGGCGTCATCTTCATGACGATCGAGGACGAAACAGGGATCGCCAATATCATCGTCTGGGAAAAGACGTTC
CAGAAATACCGGCGGCAGGTCATGGGATCGCGGCTTGTGAAAGTCCGCGGCCGCTTACAGAACCAGAGCGGCG
TCATTCACGTGGTCGCCGATCATCTGGAGGATATAACGCCGATGCTTGGCCTGCTGCGCCGGGAGGCGCGGCG
TTTCGGCGTCAACGACCGCGCGGACGGGGCCTTGCGGCCGAGCGCGGACGCCCGCGAAAAGAAAAAACTCCGG
CAATTGCGCCTGGGCCTGCCCGCTCGCGCAGCGCCGGAGGGCGAGGCGGCCGCCCAGGTCGCGGAGGTCATGC
CGAAGGGGCGCAATTTTCATTGAATGTTTGTAAGAAAGATCGCAACTGCACTCGCACTCGTAATGGCTGCATC
CGGCGTGGCTTCCGCTCAATCTCCGACGCGAATCCAGCAGTTCAACGCCTGGGGTGCATATTCCTACCAGGCC
GGTAACGGCAAGGTCTGCTATGTGCTTTCCGTGCCCAAGGAAAGGACCCCGGCAAGTGTCGATCACGGCGATA
TCTTCTTCCTCGTTTCGCAGCGTCCGGGACAGAACATCAGCTATGAGCCACAGGCGATGATGGGGTATCCGCT
GCAGGACAATTCCAAGGTCAACGTGGTGATCGACGGCCGCACCTTCGTCATGTTCACCAAGGGCAATTCCGCC
TGGGTGGAAAATGCCGCCGAAGAGCCGGCACTGGTCGCTGCGATGAAGTCGGGCAAGGCCATGTCGGTCAAGG
CGAAATCGCGGCGCGGCACGGAAACCTCCTACTCGTATTCGCTCTCCGGCATTTCGGCCGCCCTGAAGCAGAT
CGAGGCCTGCAAGTAAATGACGGTGGTGGTGAGGCCGAAGGCGCTCGGCGACTACGATACCCGCGAATTCCGG
ATGCTGATCGACGGTCAGTGGGTGGATGGCGCCGAGGGCCGCACGATCGAGCGGGTGGCGCCGGGTCACGGCG
TCGTCGTCAGCCGCTATCAGGCGGCGGCGAAAGCGGATGCCGAACGGGCGATCGCCGCCGCACGGCGCGCTTT
CGACGAAGGTCCATGGCCGCGCATGACCGCCTCGGAACGGTCGTTGATCCTGCTCAGGGCCGCCGACATGATT
GCCGCGCGAGCGGATGAGCTCGCTTTTCTCGACGCGGTCGAATCCGGCAAGCCGATCAGCCAGGCGAAGGGCG
AATTGGCGGGTGCCGCCGACATCTGGCGCTATGCGGCAGCACTTGCCCGCGAACTCTCGGGCGAAAGCTACAA
TACGCTCGGGGAGGGTACGCTCGGCGTCGTGCTGCGCGAGCCGATCGGCGTCGTATCGATCATCACGCCGTGG
AACTTCCCTTTCCTGATCGTCAGCCAGAAGCTTCCCTTCGCCCTTGCCGCCGGCTGCACGACGGTGGTGAAGC
CCTCTGAACTCACCTCGGCCTCGACGCTGGTTCTCGGCGAAATCCTGGAAGCGGCCGGCGTGCCCCAGGGGGT
CGTGAACATCATCGTCGGCACGGGGCCGGAGGCCGGCGCGCCGCTCACCACGCACCCGCATGTCGACATGGTC
TCCTTTACCGGCTCGACCGGAATCGGCCAGCTGACCATGGCGAATGCGGCGCAGACCCTGAAGAAGGTCTCGC
TGGAACTCGGCGGCAAGAACCCGCAGATCGTCTTTCCGGACGCAAACCTGGACGAATTCATCGATGCGGCCGT
CTTCGGCGCCTATTTCAATGCCGGCGAATGCTGCAATGCCGGCTCGCGGCTGATCCTGCACCGCGATATCGCC
GAGGAGGTGACGGCGCGCATCGCCAGCCTCTCGGCCAAGGTCAAGGTCGGCGATCCGCTCGACCCCGAAACGC
AGGTCGGCGCGATCATTACGCCGCAGCATCTGCAAAAGATCGCCGGCTATGTTTCTTCGGCGTCGAACGAAGG
CGCGAGAATAGCCCATGGCGGCACCACGCTCGATCTCGGCATGGGACAGTTCATGGCGCCGACCATCCTGTCG
GCCGTTCGGCCCGAAATGGCCGTGGCCCGGGAGGAAGTCTTCGGGCCGGTTCTCTCCGTTCTGACTTTCGAGA
AGACGGAAGAGGCGATCCGCATCGCCAATTCGATCGATTACGGGCTTTCCGCCGGAGTCTGGAGCCGCGACTT
CGATACCTGCCTGACGATCGGGCGGCGCGTACGCGCCGGCACGGTCTGGATGAACACCTTCATGGATGGCGCA
TCCGAGCTGCCTTTCGGCGGCTACCGGCAATCCGGGCTCGGCCGCGAGCTCGGTCGCCATGCTGTCGAGGACT

ATACGGAGACGAAGACGCTCAACATGCATATCGGCCGGCGCAGCAACTGGTGGATGCCGCGCTGAATGACCAT
CTTACCGAAACAGACCTTGCGAGTCGGCTTCGTCGGCACCGGCTTCATCGCGCATTTCCACCTGAAATCGCTC
ATCGGGGTACGCAATGTCGAGGTGACCGGCGTCTACAGCCGCAATCCCGAAAACCGCGCGCGCTTCGTGAGCG
AAGTCGAGGCGCTCGATCTCGGCGAGTGCCGCGCGCATGAGAGTCTCGAATCGCTCCTGTCCGCCGGCGACGT
CGATGCGGTCTGGATTCTGTCGCCGAACTACACCCGGCTCGACGTCATGCGGACGCTCCACCGGGAGATCAAG
GCGGGAAGAAGCAAGATCTTCGCGGTCGCCTGCGAGAAGCCGCTTGCGCGCACTGTGGCGGAGGCACGCGAGA
TGCTGCACCTGGCCGAGGATGCCGGTCTCAACCATGGCTATCTCGAAAACCAGGTCTTCTGCACGCCGGTGCT
GCGAGGCAAGGAGATCATCTGGCGGCGCGCCGCCTCGACCACCGGCCGGCCCTATCTCGCACGCGCCGCGGAA
GAACATGCCGGTCCGCACGAGCCCTGGTTCTGGCAGGGCGACAAGCAGGGCGGCGGGGTGCTTTCCGATATGA
TGTGCCACAGCGTCGAGGTGGCGCGCCACCTGCTCACGGCGCCCGGCGCGCCTCGCAATTCGCTGAAGGTCAA
GTCGGTGAACGGCACGGTGGCGAACCTCAAATGGACGCGGGCGGGCTATGCCGATGAGCTGCGCCGCCGCTTC
GGTGAGGATGTCGACTACCGCCGGCGCCCATCGGAGGATTTCGCCCGCGCCACCGTGGCGCTGGAGGACGAGG
AGGGCAACGAACTGATGATCGAGGCAACGACCTCCTGGGCCTATGTCGGCGCCGGCCTGCGCATCCAGCTCGA
GCTTCTCGGGCCCGAATATGCGCTGGAGTACAATTCGCTCGGCACCGGCTTGAAGATATTCCTGTCGCGGGCC
GTGCAGGGCACCGAGGGCGAGGACCTCGTCGAAAAACAGAATGCCGAGCAGGGCCTGATGCCGGTGCTTGAGG
ACGAGGCCGGCGTCTACGGCTATACGGACGAAAACCGCCACATGGTCGAATGCTTCCGCAAGGGGATAAAGCC
TCTCGAAACCTTCGAGGACGGCCTGGCGGTCGTCGAAATCCTGATGGGCCTCTACCGCTCCGCCGAGATCGGC
GCGACGCTGCATTTCCCCGCGCCGGAGCTGGAAGACTATGTGCCCGTCGTGGCGCGGACGGGGGCGTAGATGA
GGACGCGCCGCGAAATCCGCGCCCGCAAGGCGCTCCGGGACAGGATCGTCTATCACGGCATCGGCATCGGCAC
GGCCTTCTTCTTCCTGGCGCCCTTTGCCGTTTCCTTTCTTGCCTCCTTCCGGCCGAACAGCGAATCCGGCAGG
CCGCCGCTGCCGCCATGGCCGATCTCGGGGCTGAGTTTCGACGCCTACCGGGCGCTCGACAGCTTCGGTGCCG
GCATCTGGCAGCACATGTTCAATTCGCTCCTCGTGTCGATCGGAACGGTCGTCCTGACCGTCGCGGTCAGCGT
GCTCGCCGGCTACGGCTTCTCCCGCTACCGCTTCCCGTTCAAGAACGCGCTCTTCGTACTGATCATCGCGACG
CTGATGGTCCCGTTCCAATCGATCCTGACGCCGCTCTTCATCATTCTGGCGCGGCTCGGGCTCAACAATTCGC
TCGTCGGGCTGATGCTCGTCTATGTGACGCTGCAGCTTCCCTTTTCCGTCTTCATGATGCGCAACGCCTTCGA
TGCCGTGCCGAAGGAGATCGAGGAAGCGGCGCGCATCGACGGCGCCCGCGACCTGAAACTTCTCGTCCGGGTG
CTGTTGCCGCTGGTCATGCCCGGGATTGCGACCGTGTCGATCTTCGCCTTCCTCAATGCCTGGAACGAATTCT
TCGCCGCGCTCGTCCTCCTGTCCAGCAACGACAATTACACGCTTCCCGTTCTGATGACCGCCGTGCGCGCCGG
CCGTCTCGGCGCGATCAACTGGGGCGCGGTGCAGGCCGGCGTCGCGGTGATGGTCGTTCCCTGCCTCTTCGTG
TTCCTTCTTCTGCAACGCTATTACATGCGCGGGCTGATGGCCGGCGCGGTGAAATGAATGAGCAGTGAACAGA
GCAAACCCGCCGCGGCCCCCGTACTTCCGGGCACGTGGGGCATCAGGGAGACCGCGATCAAGTACGGCTTCCT
CATGCTGCTCGCCGGCATGGTTCTTTATTTCTCGCTCGTAACCGGAGGTTTTGCCTCCCCTCAAAGCGCAGTC
TTCATTCTGCAATCCGTGTCGATCACCGGCATTCTCGCCCTCGGCGTGACGGCGACGCTTGTCGTGGGGGGGT
TCGACCTCTCGATTGGCTCGGTCGCCACCACGGCGATGATGGCTTCCTCCTATGTTATGGTGGTGATGGGAGG
CGACGCGCTTACGGCAGCGCTCGTATGCCTCGCCGTCGGCGCGCTCGTCGGCCTGATCAACGGCTTCATCATC
GTCTACATGCGCGTTCCGGACCTTCTGGCTACCCTCGGCATGATGTTCCTGCTGCTCGGCCTTCAGCGCATCC
CGACCGAGGGCCGCTCGATTGCCGCGGGCATGACGCTGCCCGACGGTTCGGTCGCGACCGGCACTTTCAGTCC
GGCTTTCCTGGCGCTCGGGCGCCACCGCTTCGATCTCGTCCTGCCCAACCTCGTGCCGGTCTCGGTCGTGGTG
CTCGTGGTGCTCGCCATAGCGATCTGGTTCTTCCTCGAATACACCCGCTTCGGCCGCATAATGTATGCGGTCG
GGTCCAACGAGCGCGCGGCCGCGCTCGCAGGGGCGCCGGTCAATGCCTACAAGATTTCGGCCTACGTCATTTC
CGGCGTCTTCGCCTCTATCGGCGGCATTCTGCTTGCAGCACGCCTCGGCCGCGGCGATATCGCCTCCGGAAAC
AACCTGCTGCTCGACGCCGTCGCCGCCGCGCTGATCGGTTTTGCCGTGCTGGGTGCGGCCAAGCCGAACGCTT
TCGGAACGGCCGTCGGCGCGCTTTTCGTCGGGATCCTACTGCAGGGCCTGACGATGATGAATGCGCCTTACTA
CACGCAGGATTTCATCAAGGGCGCCGTGCTCGTCGTCGCCCTGATCTTTACCTTTGCGCTCTCGAAAAGAGGC
AAACGCTGAATGAAAGAGTTCAACGGCCGACGTTTGGAAATCGATGCGCTGCGCGCCTTGTGGGCGATCCGGC
GTCACGGCGGGATAACCCGCGCGGCCGAGGCCCTGGGCCTGTCGCAGTCTGCCGTCAGCCACAAGATCAAACG
GCTCGAGACGAGCCTCGATTGTGACCTGCTCGGCCGGAGCCCGGGCGGAGCGATGTTTACCGCAGCCGGAGAG
GATTTGCTGGATTATGCCGGGAAAATCCTCGGCTTGCATGACGAAGCGCTTCTGAGCTTATCGAAAGCGTCAC
TTGAAGGACGCATCGCGCTCGGTTTGACCGAAGACACGGCGTGCAGCGACCTCGCGCGGATATTGGGGAGATT
TCGCCGACTGCATCCGAATGTCGCCGTCCGCACCAAAGTCCGCATGAGTCTAGTCCTGCGAGCGATGCTGGAG
CGGGGGGAGCTAGACGCCGCGATCCTGCAGGTTTTCGCTCATGAAGTCCGGCCGACCGACGTCGTTCTGTTCC
GGGAGGAGCTCCATTGGGTCAAGCACCCGCAGCTGTCACTGCCGCAGCAGGGCCCGATTCCCTTCCTGTCCTT
CGACGACGAATGTTTCTATCGAAGATGGGCGCTCGACATCGGACAGGACGGCGGAGTGGTTCTTGAAACCGTA
TTCGAATGTGCAAGCGCTGCTGGTATCGTTGCCGCGGTCACTTCGGCCATGGGTGTAGCACTTCTGAGCGGGC
GTCATCTGCGCCCCGAAATGCAGATCGTCACCGAACGCTTGCCCACGCCGCCTGCTTTGGCATATGTCGCCCG
CCGCGCCCGAAAGGTGAGAAATTCTGCGCTAGATACGCTGGTCGCTGAAATCGAAAACGAAATCAGTCGCTAC
GGAGGGCTGGTTCTCGCGAGCTAAATGACGATGGAGGCGGATGAAATTCGCGTATCCGAGAGTGTGCCGGTCG
GTGCAGGGCACCGCCGCGGTACGCGCGCCATGGCGCCCGGCGACATTCCGGCGGTCGGACGGCTGTTCAACAG

GATTTTCCGAAGGCAGGACCACGCGCCCGGGAATGACCTTGGACGTTATCTCGAGACCGTCTTCTTCGGCAGC
CCTCACTATTCTCCCGAACTCGGCAGCGTCGTGCACGAAAACGGTGCGACCGGCATAGACAGCGCAATACTCG
CGGTACCGATGGCGTTCATGGTCAATGGCCGCCCGACCATGGCCCGTCTTCTCTGTGCCTTCATGGCGGATGG
AAAAGCAGGAGCCGTTGGAGCGGCGCGTCTTGCGCGCACCATGCGGGCGTCTCGCCAGGACATGTGCTTCTCC
GACAATGCGTCGCCGGTCAGCGCCGACCATTGGGTTGCGGGAGGTGGCATCGTCCTGCCGATCCAGAGCCTCG
AATGGCAGCGTTCGTTTCTGCCCTTGAGCGCCGCGGCTTTCCTTGCGGGCAGTCACTTCCCGATAGCGCGCTC
GCGCGTTGTCGTCGGCGTTCTCAGGCTGATTGACCGTGCACTGCGCCGGTTGCGGCCCTCCATGCGGCCGAAG
GCGGAGGGCGAATGCAGGATCGAGGCAGCGGGCGTCGAGGAGTTCCTCCAATGCGCGAGCCCGATGCTGGAAC
GCTTTGCGATCCGCCCGGCCTGGTCGCGAGCCGAATTCGATTGGCTCGTGCGCGTCGCTTCGATGAACCGGAG
CCTCGGGACGCTCCAATGCAGGAAAGTCGTTCTCGCGGACGGCCGCACGATCGGCGCCTTTCTTTTTTTCGGC
AAGCAAGGTGCCGTGGCCACGGTGCTCAATCTCGTCTGCGAAGAGGGGCGCGAACGCGACGTCGCCGCTGCGA
TGTTCGCGCGGCTCGATGCGGAAGGCTATGCCGTGGCGGTCGGCATGGCACAGCCGTTCCTGATGAACGCTAT
CTCGCGCCAGCGATGGCTGTCATTCCGGCACCGCGGCTACTTCTGCATGGTGACCCGCCATGCGGACCTCAAG
GAAGCGGCAGAGCGCAACGACATCTATATCGGCGGCCTCGCCTCCGAGACCTGGAGCCGGCTGCTGACGGATT
TTTAGATGTTCTCCGATCCAGATTTTCTGAAGCTCATCTTCGGCCGGCTTACCCTCGACGCGATCCCTTATCA
CGAGCCGATCCTCGTCGTGACCTTTCTCGCCGTCGCGCTCGGCGGCATCGCGCTGATCGGCCTCATCACTTAT
TACCGCTTCTGGGGGACGCTCTGGCGCGACTGGCTGACGAGCGTCGACCACAAGAAGATCGGCATCATGTACA
TCATTCTCGCGCTGGTGATGCTGCTGCGCGGATTTTCCGACGCGATCATGATGCGGCTGCAGCAGGCGATGGC
CTTCGGCGGCTCGGAAGGTTACCTGCCGCCGCATCACTACGACCAGGTCTTCACGGCACACGGCGTGATCATG
ATCTTCTTCATGGCGATGCCCTTCGTGACGGGGCTGATGAACTTCGTCGTGCCGCTGCAGATCGGTGCGCGCG
ACGTCTCCTTCCCCTTCCTCAACAATTTCAGCTTCTGGATGACGACGGCCGGCGCGATCATCATCATGCTGTC
GCTCTTCGTCGGCGAGTTCGCGCGGACCGGATGGCTTGCCTATCCGCCGCTGTCGGGCGCGGACTACAGCCCC
GGCGTCGGGGTCGACTATTACATCTGGGGCCTGCAGATCGCCGGCATCGGAACGACGCTGTCGGGGATAAACC
TCATCGCCACCATCGTGAAGATGCGCGCCCCCGGCATGACGATGATGAAGATGCCGGTCTTCACCTGGACGTC
GCTCTGCACCAATGTGCTGATCGTCGCCACCTTCCCGGTGCTGACCGCGACGCTGGCGCTGCTTACGCTCGAC
CGTTATGCCGGCACGAACTTCTTCACCAACGACCTCGGCGGCAATCCGATGATGTATGTCAACCTCATCTGGA
TCTGGGGCCATCCGGAGGTCTACATCCTCATCCTGCCGGCCTTCGGCATTTTCTCGGAAGTCGTCGCGACCTT
CTGCGGCAAGCGGCTCTTCGGCTATGCCTCGATGGTCTATGCCACGGTCGTCATCACCATCCTCTCCTACATC
GTCTGGCTGCACCACTTCTTCACCATGGGCTCGGGTGCGAGCGTCAACGCCTTCTTCGGCATCACGACGATGA
TCATCTCGATCCCGACCGGAGCGAAGATGTTCAACTGGCTCTTCACCATGTATCGCGGCCGCATCCGCTACGA
GGTGCCTATGCTGTGGACCATCGGTTTCATGGTGACCTTCGTCCTCGGCGGCATGACCGGCGTCCTGCTTGCC
GTTCCGCCGGCCGATTTCGTCCTGCACAATTCGCTGTTCCTCATCGCCCACTTCCACAATGTCATCATCGGCG
GCGTCGTCTTCGGGCTGATGGCAGGCGTCGTCTACTGGTTCCCGAAGGCCTTCGGATTCAGGCTCGATCCGTT
CTGGGGCAAGATGTCCTTCTGGTTCTGGCTGACCGGCTTCTACTTCGCCTTCATGCCGCTCTACGTGCTCGGG
CTCATGGGGGTCACGCGCCGCGTCAGCCAGTTCGAGGATCCCTCGCTGCAGATCTGGTTCGTCATCGCGGCCT
TCGGCGCCTTCCTGATCGCGCTCGGCATCCTGTCGATGCTGATCCAGCTCGTCGTCAGCTTCGTGAGGCGCGA
AGAGCTGCGCGACACGACCGGCGACCCCTGGGACGGGCGCACGCTCGAATGGTCGACCGCCTCGCCGCCACCG
GATTACAACTTCGCCTTCACCCCGCTCGTCCACGATCAGGACTCCTGGTGGGATATGAAAAAGCGCGGCCACA
GCCGGCCGCTTGTAGGCTTTAAGCCGATCCACATGCCGAAGAACACCGGCACAGGCGTCATCCTCGGAGCGAT
CAGCGTCGTCTTCGCCTTCGCCATGGTCTGGTACATGTGGTGGCTGGTGATCCTCTCCTTCGTCGCCATGGCG
GCGATCGCCATCGGCCACACCTTCAATTATGACCGCGACTTCCACATTCCCGCAAACGTCGTCGCCGACACAG
AGGGCAAGCGGACCGAAGCGCTTGCCGCCGAGGTGCAAGCATGAATGGCTCAAAGAGCAGGCAACGCGGATCT
CCCGCTGCATGGCGGCCGCGTGCCGCGATGGCTCGGCGACCGTATGACGCGCCTCGGCGCGTTGGTCACCGAA
GCGGTCGTTCATCACTACGGACGCGACGAGTTTTTAAGGCGGCTTGCGCATCCGTTCTGGTTCCAGTCCTTCG
GTGCCGTCATGGGCATGGACTGGCATTCCTCCGGTATTACCACGAGCGTCATCGGTGCATTGAAACGCGGTCT
GACCCCGCTTGCCGGCGAGCTGGGCATCCATGTCTGCGGCGGCCGCGGCCAGCACTCCCGCAAGACGCCGGGC
GAACTCATATCGATTGGCGATCGGGTCGGTTTCGATGGCGGCGCATTGGCAGAAGCGAGCCGCCTCGTCGCCA
AGGTCGACAGTGCCGCCGTTCAGGACGGCTTCGATCTCTACCTGCACGGTTTCATCGTCACGGACGATGCCAG
GTGGGTGGTCGTCCAGCAGGGCATGAACGGCGACCGCCGGCAGGCGAGGCGCTATCACTGGCTCTCGGAGGGC
CTGACAAGCTTCCTCGACGCGCCGCATAGCGCGATCGAAGGCAGAGAACAGGGCGAAATCGTCAATCTTGCCG
ACCGCCGCGCCGCCGCCTCGCGGACTGCGCAGCTCGATCTCCTCCAGTCCCTCGGGCCCGACGAGCTCCTGCG
CGAGGTCGCCCGGATCGAGTCTCGTGGGGCTCCTGTGGCCGCGCCGGCACAGCCGATGCTGCCGCATCTCTTC
ATGCCCGCCCATCACGACGTGCGCGAAAGCGACGTCAATCTCCGGCGCCTTCACGGCAACTTCGCCGCCGCCG
CCGACCGTGGTCCGGAGGATTTCAAGGACCTGCTGCTGGTGCCAGGCGTCGGCGCGCGGACGGTCAAGGCGCT
GGCGATGGTCGCGGAGGTGGTCCACGGGACGCCGTGCAGGTTCTCGGACCCCGCCCGCTTTTCGCTCGCCCAT
GGCGGCAAGGACCGCCATCCGTTCCCGGTGCCGTTGAAAGTCTATGACGAGACGATCAGGATCATGAAATCAG
CGGTCAGCAAGGGCCGCCTCGGGCGCGAGGAAGAGCTTGCGGCGCTGAAGCGGCTCGACGAGCAGTCGCGCCG
GCTGGAGCGCTACGTCACGGGCCCCGACCTCAAGGAGATCGTCGCGGGCGAATTCAGGGACTCCGCGCGTTTC

GGCGGGCGCAGCATATTCGGCTTGGAACCGCACGACGACGAGGCAACCGCGGCGGAGCCAAGCAACCGCACGC
GGCGCTGAATGACGACGAGCGAGATCAAGAACGTGCAGGGAATAGCAGCGATCGAGGCCCTGCTTGCCGCCGA
ATACGGCATCGCGGGAGAAATCCTGGCGCTGCCCGGCGAGCACGATCTCAATTTCCGCATTCAGGCGAGCGAC
GGTCGCGCGTTCCTGCTGAAGCTGCATGCCCTCGGCGCGCCCGAGGAGCTCGACATGCAGATCGCGGTTCTCG
ACCATCTCGCGCGCGAGGCGACGGAGCTGCCTGTCGCGAAGGTCCTGCCGAGCCGCTCCGGCGCCTCGTTCAC
CCGCGTCGAATTCGAAGGAGAGCGCGTCGCACGTCTTCTGACGTGGCTGCCCGGGGAAACCTGGGCACGAGCC
GCCGACCGCTCGAGCAACAGCGTCGAGACCCTTGGGGCGCTGCTCGGCAAGCTCGACCGCAGCCTCGCCGGTT
TCTCTCATCCCGGGACCAGGAGAGAGTACGCCTGGGATATAGCCCGGGCGGAGATGCACCTCGCCAATGTCGA
TCTGATCGAGGGTATGGAGAAGCGGCGAGCGGTTCGCGCGATCCTCGAGCACTTCGTCTCCACGGTGCTGCCG
CGCCTTAAGGCTTGCCCCCGGCAGGTCATCCATAATGACGCCAATGACTATAACGTGCTCGTCGGCGCCGACG
GCTGCGTCAGCGGACTGCTCGACTTCGGCGATATGGTCGAGAGCAATCGCGTGGTGGAAGTGGCGGTCGCTTC
CGCCTATGCGCTGATCGGTTCGCCGGATCCGATCGGCGCCATCGCGCGGCTGGCTGGCGGCTATCACGGCGTC
AATCCGCTCGGCGAAACGGAGGCCGAGCTGATCTTCGATCTCGTGCGCACCCGCTATGCCGTCAGCATGTGCA
TGGCGGCGAGGCAGATCCGCGACAACCCCGAAAACACCTATCTGCTCGTCAGCCAGGAGGACGTCTGGCGGGA
ACTGAGACGCCTCGAGCAAGAGAACCGGCCCTTTGCCATCGCCCGCCTTCGCGACGCCTGCGGTTTTGCACCG
ATTCCGAAGGCCGCGCGGGTGGTGCGGTGGCTGGAGAGCAATGCTCATGATTTTTCGGGCGTCATCAAGCCGG
GCATCGCAAGACCGAAACCAGCCGTCTTCGATTTTTCCGCGAACAGCTCCGAGACCTGGGCAGGTCTCGACAA
GGACGTGGCGCAAGCGCGCATCGAAGCACATATTCATGCGGAGGGGGCGGATTTCGGCCTTGGCCTCTACGGA
GAGGACAGAGCGGTCTACAAGGGTGACGCTTACCAGGCGACTGCAAGCCTGCGCCGCACGATTCACCTTGGCA
TCGATCTCTTCGCTCCCGCCAACGAGCCGGTGCATGCACCCTTCGCCGGCAAGGTCGCATTTTACCATGACGA
CGCGGTCCCTTACGGTTTCGGACCGACCATTCTGCTCGAACATAGGACGGGCGAGGGCGACGCGTTCTGGACG
CTCTACGGACATCTCTCCCGTGAAAGCGCCTCACGGCTGTCGATCGGCCAGCCGGTCGCCAGGGGCGAAGCTT
TCGCGGCCATGGGGAATCGCGCCGAAAACGGCGGGTGGGTTCCGCATCTGCATTTCCAGATCGTCACCGATCA
TCTCGGCCTCGAGGGTCGCATGCACGGCGTCGGCGTCAAAGAGCAGTGGCAGGTCTGGCGAGAGATCAGCCCC
GATCCGAGCGTCGTGCTCGGCCTTTCCGTTCCGGCGTCGGTGATCGTCGAGCGCGGCAAGGTGTTCCTGGTGC
GCGAACGCCAGCGCCGCATCGGTCGCTCCCTAAGCATCGCCTACGGCTCGGCGCCTCTCAAGATCGTTGCCGG
CGAGGGCGCCTACCTGATCGATGACGAGGGCACGCGCTGGCTCGACATGGTCAACAATGTCTGCCACGTCGGC
CATTGCCATCCGCGCGTCGTCAAGGCGGCGCAGATGCAGATGGCGCGGCTCAATACCAATTCGCGCTATCTGC
ATGACAGTCTGGTGGAATATTCGCGCCGGCTTGCGGCGCTCTTCCCTGATCCGCTGAACGTCTGCTTCTTCGT
CAATTCCGGCAGTGAGGCCAACGATCTCGCGATCCGGCTGGCGCGCGCCTATACCGGCAACCGCGACGTCATT
ACCGTCGATCACGCCTATCACGGCCATCTGACAAGCCTGATCGATGTCAGTCCCTATAAATTCGCGGGCAAGG
GCGGGGAAGGGCGGCCGGCGCATGTGCGGGTCGCCGAAATGCCGGACCTCTATCGTGGACGCTATCGCTATGG
CGATACAGACGCGGGGCGCAAATATGCCGAGGACGTGAAGCGTCAGATCGATGCTCTGGCGGCGGAAGGGCGC
AAACCCGCGCTCTTCTTCAGTGAGGGCATCCTCGGCACGGGCGGTCAACTGGTGCTCCCGGAAGGCTATCTCA
GAGGGGCCTATGCGCATGTGCGTGCGGCCGGTGGGTTGTGCCTCGCTGACGAGGTCCAGGTGGGGTTCGGCCG
TGTCGGCAGTCATATGTGGGCGCATGAAACGCAAGGAGTCGTGCCCGACATAGTCACCATGGGCAAGCCGATC
GGCAACGGCCACCCGATGGCGGCGGTGGTGACCACCGAGGCGATTGCCGCGGCCTTTGCAAACGGAATGGAAT
ATTTCAACACGTTCGGCGGAAACCCGGTATCGGCCGAGATCGGCCTCGCCGTGCTCGACATCATCCGCGATGA
GCGCCTCATGCACCATTGTGCGGTCGTCGGAAACCGTCTGATGGATGGAGCGCGCGAACTCGCCAGCCGCCAT
ACGATCATCGGCGACGTGCGCGGTTACGGGCTCTTCAACGGAATCGAGCTCGTGCGTGATCGCGACACCCTTG
AGCCGGCCGCAGCGGAGCTCGACTTCGTCATTGCCGAAATGAAGGACAGGCACCGCATCCTGCTGTCGAGCGA
TGGGCCGCAGCACAACGTGCTGAAAATCAAGCCGCCTGCGCCCTTCAGCGCAGACGATTGCGACCGTTTCCTC
GATGCCCTCGACGCGGTGCTGGCGCAGGTAAATGCCGCCTGAATGGAACACCAACTGGCCAGACAAATCGCAT
CGCGGAAGCCCTTCGATCCGACCTATGATCCGATCCGCGCGCCCAATCCGGGCGACGGCAAGGACTATGCGCC
GACCTACTGGATCGGCACGGCCGGCACTCCGCCTGCCGACGATGGGCCCGTTAGCGGCGATATGGACGTCGAC
GTCGCTATCGTCGGCTCCGGATATACGGGGCTCTCCTGCGCGATCCATCTGGCCCAGGAGCATGGCATCAAGG
CGACGGTGCTCGAAGCCAATGGCGTCGCCTGGGGCTGCAGCACCCGCAATGGCGGTCAGGCGCAGATTTCCGC
CGGCCGGCTCAAGCGTTCGCAATGGATCGAGCGCTGGGGCGTCGATGTGGCCAAAAGGCTGCATGGCGAAATC
AGCGAAGCGTTCGATCTGTTCCGGGACCTGATCCGCTCGCCCGAGATCGACTGCGACCCTCAGGACGGCGGCC
ATCTTTATATCGCCCATCGGGACAAGGTGATGCCGGCGCTCGAAGCCGAGTCTCGGCTTCTCAACGAGGTTTT
CGGCTACCGGTCGCGTATCGTCGGTCGCGATGAGGTCCACCGCGACTTCGTACGCGACGAGGAGGCGAGGGGG
GCGATGTACGAACCGGACGGCATGGGCATCCACGCGGCCAAGCTCGCCTTCGGCTACCTCAATCTGGCACGCA
AGCTCGGTGCGCGGGTCCACACGGCCAGCCCCGTTCTCGGCTGCGACTGGAAGAACGGCGCCTATCATCTGAC
TACGCCGGGCGGCACCGTTCGTGCGCGCGCCGTCTGCATTGCGACGGCGGGCTATACCTCGCCGGACCTGCAT
ACGCTGACGCGGCACCGGCTGATGCCGATCCTCTCGAACTCGATCGTCACGCGCCCGCTGACCGAGGAAGAAC
GCGCCGAACTGAACTTCAAGACGCGGATCCCGCTCACCGATACGCGCACGCTGCGCCACTACTACCGGATGAT
GCCGGACGGCCGCGTGCAGATCGGCAGCCGCAGCGCCATCACCGGCCGCGATGCGGTCAACCCCAAACATCTC
GATCGGCTGCTCGAAGGGCTCTACCGAAAGTTTCCGATCCTGAAGGGGATCACGATCGACTATTCCTGGTGGG

GCTGGGTCGATGTCAGCCACGACATGATGCCGCGGATCTTCCGGCCCGATCCGAAACAGCCGCTGTTCTATGC
GATGGGCTATGGCGGCAACGGCGTCATGTATTCCGCCCAGGCCGGCCGCCGCATGGCCCAGATGGTGGCGGGC
AAGGGCGGCGCACTCGACCTGCCGATCTTCACGTCGCCGCTCCCGAGCCATGGCCTGCTCACGCCTTTCAGAC
GCCTCGGCCAGTGGGGCATGTATCGCTGGTACTACCTCAAGGACGAAATTCTTTAGATGATCTCCTTCATCCT
TCGCCGGCTGGCGAGTGCGGTGCCGACGCTGTTCATCGTCGTCACCATATCCTTCTTTCTGATGCGTTTTGCC
CCCGGTGGGCCCTTCAACCTCGAGCGTCCTCTCCCGCCCTCCACGATGGAGAACCTGATGAGGACCTATCACC
TCGATCAGCCGCTCTGGCGCCAATACGCTACCTATCTAGGCAATGCGGTCACGGGCGACTTCGGTCCGAGCTA
CATCTACCATGACAACAATGTGGCGCAGCTGATCGGCAAGGGCCTGCCCTATTCGATGGAGCTCGGCTTTTAC
GCGCTGTTACTCGCGGTCGTCGGCGGCGTCATAACGGGAACGATCGCCGCATTGCGGCAGAACAGCTTCCTGG
ATTTCGCGGTCATGTCGGTTTCCACGATCGGGGTCACCGTTCCGAACTTCGTCGTCGGTCCCGTTCTGACGCT
GATCTTTGCGATCGTGCTCGCGTGGCTGCCCGCCGGCGGCTGGGGGGACGGTTCTCTCCGGTTCCTCATCCTG
CCGATGATCGCGCTCGCGCTTCCCCAGCTTGCAGTGTTCGCCAGGCTCACACGCGGCTCGATGATCGAGGCGC
TCAACACGGATCATATCCGCACCGCGAAAGCCTATGGCCTGCCGTCCCGCGCCGTGGTCGTGACGCACGCCAT
GCGCGGTGCCATGCTGCCGGTCGTCTCCTATCTTGCTCCTTGCGCCGCCGCCCTCCTTACGGGCTCGGCCGTG
GTGGAAACGATCTTCACTATACCGGGGGTCGGGCGCTACTTCGTGCTCGGAGCAATCAACCGCGACTATACGC
TGGTAATGGGCACCGTGATCCTCATCGCGATCTTCGTCATCCTCTTCAATCTTCTGGTCGATATTCTCTACGG
CCTGCTCGATCCGAGGGTTCGCCATGACTGAATGATCGAAATCGAGGAAATAACCAAGCGCTATGGCGACACG
ACGGTCGTGAGCGACGTGTCGCTGACGGTCGCGCCGCATACGGTCACCGTCGTCGTCGGAACCTCCGGCTCCG
GCAAGACGACGCTCCTCAGAATGATCAATCGCCTCGTCGAGCCGACGGCAGGGACGATCAAGATAGACGGCGA
GGACAATCGATCGATTCCGGGTTTCGAGCTGCGCCGGCGTATCGGCTACGCCATTCAAGGCCATGGCCTCTTC
CCGCACAGGACAGTCGCGCAGAACATCGCGACCGTGCCGACGCTGCTCGGCTGGGAGAAAGCCCGCATCGATG
CCAAGGTGGAGGAGCTGCTGAAGCTGTTCCAGCTCGATCCCTCGGAATTCGGACCGCGCTACCCTCACGAGCT
TTCCGGCGGGCAGCAGCAGCGCGTGGGCGTTGCAAGGGCACTTGCGGCGGAGCCCAACGTACTCCTGATGGAC
GAGCCCTTCGGCGCGCTCGATCCGATCATCCGCGCCAAGGCGCAGGACGACCTCGCCGCCATCCAGAAACATT
TCGGCACCACGATCATCCTCGTCACCCACGACATGGAAGAGGCCTTCCACCTTGCCGACAGGATCGCGGTGAT
GGACAAGGGCGAGGTGGTGCAATATGCGACGCCGGCCGAAATGCTCGTCAGGCCCGCCACGCCCTTCGTCGAG
ACGCTGGTCGGCGCCAGCGAGCGTCCATTCCGGCTGCTCGACATCGAAACGGTCGGCGAGGCGGTCGAGCCCG
GCTCGGCCGAGGGCCGGCCGGTTCCGGAAGTTCTCAGCCAGCGTGACGCGCTGTCGGAGCTTCTGTGGACGGG
CCGTTCGGCACTGCCGGTCGCGGCGGCGGACGGCGCGATCCTGGGCAAGATCACGCTCGAAGCGATCGTGAAG
CGTGCGGCGAGGCGGCAGTGAATGCGCGTGCCCCCCGGGATTGCCGACGATCTCACCTTTCTCACGGCCTTTC
GCCGCGACCTGCACGCTCATCCCGAACTCGGTTTCGAGGAAGAGCGGACGAGCGATCTTGTGGCGAGAATCCT
CGAAGAAGCAGGCCTCAGAGTCCACCGCGGCCTCGGCAAGACAGGCGTCGTTGGCACGCTGCAGGTCGGCAAC
GGCACGCGCGCGATCGGCCTGCGCGCCGACATGGATGCGCTCGCCATGCCGGAACTCGCCGACCGGCCCTACA
AATCCACGGTAGCGGGGAAGATGCATGCCTGCGGCCATGACGGCCATACGACGATGCTGCTCGGCGCGGCGCG
CCACCTTGCGGCGACGCGCAACTTCTCAGGAATGGTCCATTTCATCTTCCAGCCCGCCGAGGAAGGCCGCGGC
GGCGCCAGGCGCATGGTGGAGGACGGGCTTTTCGATCTTTTCCCTTGCGACGCGGTCTATGGACTGCACAACA
TGCCGGGGCTTGCAGTGGACGAGATGGCCGTCGTGGCGGGGCCGCAGCTTGCCTCCTCGGACAGCTGGCGCCT
CACCTTCCGCGGAGTCGGGACGCACGGCGCCAAGCCGCATCTCGGCCGGGATCCGATCACGGCGGCGGGGACA
TTCCTCGCTTCTCTGCAAACCGTCGTCGGCCGGGTCGTCGACCCGCTGCAGCCGGCGGTCGTCAGCGCCTGCG
CGCTGCAAGCAGGCGACCCCAAGGCGCTGAACGTCATTCCGGACACGGTCGAGATCGGCGGCACGGCGCGTGC
CTATACGCCGCATGTGCGCGACCAGTTGGAAGAAGAGATCGGCCGGTTGGCGCTGGGGACCGCGGCGATGTAC
GGCATCGAGGCCGATTATCGCTTCGAGCGGCGTATTCCCCCGGTCGTCAACGATCCCGACGCGACGGCACGCG
CCCTTTCGGCCGCGCGGTCCGTCTTCGGTGAGAAGGCGCTGACCAGCTTCCCACCCTCGACCGCCGGCGACGA
CTTTGCCTTCTTCGCGCTGGAGGCGCCCGGCTGCTATGTCTGGCTCGGGAATGGTCCCGCCGTCGACGGCGCG
CTGCACCATAACAGCGCCTACGACTTCAACGACGCGGCGATCGGCCCCGGGGCTGCCTTCTGGACCGCGCTGG
TGGAGCAGGAACTCAAGGCCTGAATGAACACTGGTCCTGATGCCGAAAAACTGCTGAACCGCATCCGGACGCG
CACCTTCACGATCGGTATTATGGGGCTTGGATACGTCGGATTGCCGCTCGCTATGAGCTCGCTTGAGGCGGGG
TTCAGGGTAATCGGTTTCGACATCGACCAGTCGCGGGTCGCGCTGCTCAACCGCGGCGAAAGCGGAATAAAGC
ATCTGCCTGGCCCAGCCATTCAGCAGGCTGTTGAGAGCAAGCGGTTTCGCGCCACCAGCGATTTCTCTGAGTT
GAGCATGCCTGATGCGATCCTGATAGCGGTTCCGACGCCGCTTACTAGAAATCGGGAGCCAAACCTGTCGTTT
GTCGAAAACAGCGCAAGGTCGATCGCTGAGCGGCTCCGTCCTGGGCAACTTGTCGTCTTAGAGTCGACCACTT
GGCCAGGCACGACACGAGAAATCGTGCGGCCGCTGCTGGAAGCGACGAACCTTGTCGTTGGAAAGGATTTTTT
TCTTGCATATTCGCCCGAGCGCGAAGACCCCGGAAACGAAGTGCACACTACCACCAGTGTTCCGAAGGTGATC
GGCGCCGACGATCCGTCTTCACTGGAAGTTGCGCAAGCACTGTACAGCCAGATTATCGTGCGGACTGTTCCCG
TGACTTCGACCGCAACCGCGGAGGCGGTCAAGCTAACGGAGAACATATTTCGCTCCGTGAACATTGCCTTGGT
CAACGAGCTGAAGGTCATTTTCGACAGGATGGGCATCGATGTTTGGGAAGTGATCGAAGCCGCCAAGACAAAG
CCGTTCGGATATATGGCGTTCTACCCCGGACCGGGCCTCGGCGGCCACTGCATCCCCATCGACCCGTTCTACC
TGACATGGAAGGCTCGCGAGTACGAAGTAGCAACGCGCTTCATAGAATTAGCTGGAGAGGTCAACACGAACAT

GCCGCACTACGTTGTTGACCGCCTTGTGGAGGCTTTGGACCGCAGAGCCCGCCGCGGCTTGAACGAATCTCGA
ATCCTATTGATCGGGATGGCTTACAAGAAGAATGTGGGGGATACACGAGAGAGCCCGTCGCTACGGCTAATCG
ACCTGATGGAGCGACGGGGCGCAGTCGTTGACTACCACGACCCTTACGTACCTGAGATTCCCGCCACAAGAGA
GTATGCCGAGTTAGCAGGTCGGCGCTCGGTGGATATTTCAGTTGACCGCCTGGCCTCGTTTGATGCCGTTGTC
ATTACGACAGATCATGATGCCATTGATTGGAAAACCGTCGTTGAAGGTGCAAAGCTGGTGGTCGACACCAGAA
ATGTCTGTGCACGCTTCCATGTGAGCGCCCCTAATGTGGTAAAGGCATAAATGCGTATCGTCATCGGTTCCGA
CCATGCTGGCTTCCACCTCAAGGCCACGATCATCGACCATGTCACCTCGCTCGGCCACGAGGTGTACGACGCA
GGCTCATACGATGCGAACCCCGTAGACTTCCCCGACATCGCAAAGATGGTCACTTCTTCGATCATCGAGGGCA
AGGCGGATCGCGGGCTGATGGTCTGCGGCACCGGCGTCGGCGCCTCGATCGCGGCAAACAAGGTGAAAGGTAT
CCGCGCAGCGGTCTGCCATGACGTGCATTCCGCCCATCAGTCGGTGGAGCATGACGACGTCAACGTCATGTGC
ATCGGCGCCCAGATCGTCGGCGCATGGTTGGCCAGGGATCTCGTTGCAGCCTACCTCGCTGCCGAATTCTCCA
CCGACGAGGACTTCCGGCGCCGCGTGCGCAAGCTTGCCGAAATGGACGAGCAGCGCTGAATGGAAGCTCTTGC
AGCCCTGGCGCGAAACTGCATCGTCACGCTGTTGTGCGGTTGCGCGCTTTTCGCACCTTCGCTTGCTGCCGAG
GAGGCCGATGATGTCGCGACCGGGACGCGGCTTGCCGAGCTGTTGCGTGCCGCTCGAAACGTTCTTTCGAATT
ACCAGTCGCTCATCAATGACCCGGCCCTCGGAGACAAGAAACTCGACGGTGAGCGTTTCACTGCCGAAGCGAT
CGCCCTTTATGGCGAGCGCACCGGCCATCCGTTGATGTCGGATGATCTCGGCGATCGCGACCGCAAGCTTCTG
CAGGCGCAGGTCGAGGCGATGCGGGAAGTCGTGAATGAGCAGCAGGACGACATAAACCGCCCCGGGATCGGGT
TCAAAGGTTTCGTGCCGGCCATTTTCGCGCGTCTGATGAACGAGAAATTTGCCGTCAAGGCGGGCAATGAGGC
GCTGGTCCGCGTTACGGCGCCGGAGGTTCTGGTGCGCAACCGCAAGTCGCTTCCCGATGCCTGGGAGGCGCGA
GTCATCAACGAGGTCTTCTCGGATCCGCAGAGGCCGAAGGGTGAACTCTATACGGAAGTGACGCAGGTGAACG
GCCGCCCGGCTTTCCGGATGCTGCTGCCGGAATATTACACGGAGTCCTGCCTTTCCTGTCACGGCTCGCCGAA
GGGTGAAATCGACGTCACCGGCTACCCGAAGGAAGGCGGCAAGGCGGGCGATCTGGGCGGTGCCATCAGCATC
GTCCTGTTCAAATGAATGATTTTCACCCCCAAAGGAAAACACCTCGTCGCGGGCGAATGGCTCGATGGGGCAG
GCACCTTCGCTTCGGCGCCGGCGCATGGCCCGGCGCATGATTTTGCCGTAGGCACCGTAGAGCTGGTGAACCG
GGCCTGCGAGGCGGCTGAGGAAGCCTTCTGGACCTATGGCTATTCCTCGCGCAAGGAGCGCGCCGCCTTCCTG
CGCGCCATCGCCGACGAGATCGAGGCCCGGGCGGAGGCGATTACCGAGATCGGCAGCCAGGAAACCGGCCTGC
CCGAGGCGCGGCTCAACGGCGAACGGGGCCGCACGACCGGCCAGCTCAGGCTCTTTGCCGATCATATCGAAAA
GGGTGATTATCTCGACCGGCGCGTCGATGCGGCGATGCCCGAGCGCCAGCCGGCGCCCCGTCAGGAGATACGG
CTGGTCCAGCGCCCCGTCGGTCCGGTCGCCGTCTTCGGCGCCTCGAATTTCCCGCTGGCCTTTTCGACCGCCG
GCGGCGATACGGCCGCGGCGCTCGCAGCCGGCTGCCCGGTCGTGGTAAAGGGTCATTCGGCGCATCCCGGCAC
CGGCGAGATCGTCGCCGAGGCCGTCGACGCAGCGATCCGCAAGACGGGTGTTCATCCCGGCGTATTCTCGCTG
ATCCAGGGCGGCAGCCGCGACGTCGGTCATGCGCTGGTGCAGCATCCCCATATCAAGGCCGTAGGCTTCACCG
GTTCGCTCGCCGGCGGACGTGCCCTGTTCGATCTTTGCGCGGCGCGCCCCGAACCGATCCCGTTCTTCGGCGA
ACTCGGTTCGGTCAACCCGATGTTCCTGCTGCCGGAAGCGTTGAAGGCACGGGCGGAAACGCTGGGGCAGGGA
TGGGCCGGTTCGCTCACCATGGGCGCCGGGCAGTTCTGCACCAATCCCGGAATCGCCGTCGTCATCGAAGGTG
CGGACGCCGACCGCTTCACCACCGCCGCCGTCGAGGCGCTTGCCAAGGTGGCACCGCAAACCATGCTGACGGA
CGGTATCGCCAAGGCCTACCGCGACGGTCAGGCGCGCTTTGCGACGCGCAACGCCGTCAAACCGCTGCTTGCG
ACCGAGTCGTCCGGCCGTGACGCATCGCCCAATCTCTTCGAGACGACGGGTGCCCAGTTCCTGGCCGACCATG
CGCTCGGCGAAGAAGTGTTCGGCCCGCTTGGACTGGTGGTTCGGGTGGGATCGCCGGCCGAAATGGAAGAGCT
CGCGCGCGGCTTCCAGGGCCAGCTGACCGCAACCATCCATATGGATGCGGGCGATCTGGAGACGGCGCGCCGT
CTGCGTCCGGTGCTCGAGCGCAAGGCGGGCCGGGTGCTGGTCAACGGCTTCCCGACCGGGGTTGAAGTCGTGG
ATTCGATGGTGCATGGAGGCCCCTATCCAGCCTCGACCAACTTCGGCGCAACCAGCGTCGGCACCATGTCGAT
TCGACGTTTCCTGCGTCCCGTCGCTTACCAGAACATGCCGGAAGACCTGCTGCCCGAGGACTTCCTCGGCTGA
ATGCGCACTCGCGCTGCTCTGGCCGTTCAGGCCGGAAAACCCCTTGTCGTGACCGAAGTCGAGCTCGAAGGCC
CGCGGGCCGGCGAGGTGCTGGTCGAGGTGAAGGCGACCGGCATCTGCCATACCGACGATTTCACGCTGTCGGG
CGCCGATCCGGAGGGGCTGTTCCCGGCGATCCTCGGTCATGAGGGCGCCGGCATCGTCGTCGATGTCGGCCCC
GGCGTCACCTCGGTGAAGAAGGGCGACCACGTCATCCCGCTCTATACGCCGGAATGCCGCTCCTGCCCCTCCT
GCCTCAGCCGCAAGACCAATCTGTGCACCGCCATCCGCGCCACCCAGGGGCAAGGGCTGATGCCGGACGGCAG
CTCGCGGTTCTCGATCAACGGCGACAAGATCCACCATTATATGGGCTGCTCGACCTTTTCGAACTTCACCGTG
CTGCCGGAGATCGCGCTCGCCAAGGTCAATCCGGACGCCCCCTTCGACAAGATCTGCTATATCGGCTGCGGCG
TCACCACCGGCATCGGCGCGGTGATCAACACCGCCAAGGTCGAGATCGGCGCCACCGCCATCGTCTTCGGTCT
CGGCGGCATCGGCCTCAACGTCATCCAGGGGCTTCGCCTTGCCGGCGCCGACATGATCATCGGCGTCGACCTC
AACAACGACAAGAAGCCCTGGGGCGAAAAGTTCGGCATGACCCACTTCGTCAATCCGAAGGAGGTCGGCGACG
ACATCGTCCCTTATCTCGTCAACCTGACGAAGCGCGGTGCCGACCAGATCGGCGGCGCCGACTACACCTTCGA
CTGCACCGGCAACACCAAGGTCATGCGCCAGGCGCTCGAAGCCTCGCATCGCGGCTGGGGCAAATCGGTGATC
ATCGGCGTCGCCGGCGCCGGCCAGGAGATTTCGACCAGGCCGTTCCAGCTCGTCACCGGCCGCACCTGGATGG
GCACCGCGTTTGGCGGCGCGCGCGGCCGCACCGATGTGCCGAAGATCGTCGACTGGTACATGGAGGGCAAGAT
CGAGATCGATCCGATGATCACCCACACCATGCCGCTCGAGGACATCAACAAGGGTTTCGAGCTCATGCATTCC

GGCGAGAGCATTCGAAGCGTGGTCCTCTATTGAATGAACACATATGAAAGGATCGATAGAGACCTGTCCGACC
GGGACTTCACCGTCGTGGGCAAGAGCGTCAAGCGTTCCGATACTCTGGAGAAGGTCACGGGGACGGCCAGATA
TGCAGGCGACGTCGCCTTGCCCGGCATGCTCCACGCCAAGATGAAGCGCAGCAACATCGCGCATGCGCGCATC
AGGAGCATCGACACGAGCAAGGCGCTGGCGCTCAAGGGCGTCAAGGCGGTGCTGACGCACGAGGACGTGCCGC
GCGTTTTGCATGCGGGCTCACCGCATCCGCGCTCGGCATCCGTTACCAAGGATCAGTACATCCTGGATGACAG
GGTGCGATATTGGGGCGAGGGCGTCGCCGCAGTCGCTGCCGTCAGCGAGGAAATCGCTGAGCGCGCGGTCGCG
CTCATCGAGGTCGAGTACGAGCCCTTGCCCGGCCTCTTCACCATCGAAGCCGCATCGGTGCCCGGCGCTCCGC
CGATCCATGAGAATGGTCTCGACCGGAACCAGGTGTTGCCGCCGGTCTTCGTCACCCGCGGCGACGTCGACAG
GGGTTTCGCCGAAGCGGATCTGATCCTCGAGCGCGAATACGATCTCGGCCGCCCGACACCGGCCTATATGGAG
CCCAATGTCTGTGTCAGCCAGTGGGACGGCAACGGCAAGCTCACCATGTGGACCTCGACCCAGAGCGCATTCA
TGGTCCGCGGCACGCTTGCCGAAGTGCTCGGCGTGCCGCTGAACAAGGTACGCGTTATCGTCGATCACATGGG
CGGCGGCTTCGGCGCGAAGCAGGATCTGTTTCAGAACGAGTTCCTCTGCGCCCTGCTCGCGCGACAGACGGGG
CGTCCGGTCAAGATGGAGTTCAGCCGCAAGGAAACGTTCCTCGGCGGCCGCTCGCGACACCCCGGCAAGATAT
GGCTGAAGCAGGGCTTCACCAAAGACGGCCGGATCGTCGCACGCGAAGCCAGGGTCACCTTCAACTCCGGCGC
CTACGGCTCGCACGGTCCAGGCGTCACCAATGTCGGCACCGCAGCGCTGACCTCGCTCTACCGTTGCGAGAAT
GTCCGGCTGGAAGGCCGCTGCATCTATACCAATTCGCCGATCGCCGGCGCCTTTCGCGGCTATGGTGTGGTTC
AGACCTACTATGCGCTCGACCTCATGATGGACGAGGCGGCCGAGAAGCTCGGCTTCGACCCGGCCGAGTTCAA
GTTGATGAACGCGGTGCGCGAGGGCGACATCGCACCCTCCGGGCATCCGATCGTCGGACACGGCCTCGGGGAT
TGCATCCGCCGCGTCATGGAGGAGACGAACTGGCACGAATTGCGCAGGCGGGAGAAGCCGGAAACCGTCAAGC
GCCGCGGGATCGGGATCGGCTGCGAAATGCACGGATCCAGTGCCTATCCGGGCATCAAGGAACAGGGCAACGC
AATCGTGAAGATGAACGAGGACGGAACGGTGACACTGATCACCGGCACCGCCGGCCTCGGCACCGGCGCGCAT
ACAGCGCTTTCGCAGATCGTCGCCGAGGAACTCGGCGTGCCCTTCGAAGCCGTCTCGGTCGTTCAAGGGGACA
CCGATATCGTCCCCTGGGATATCGGTGCCTTCGCCAGCCACACGACCTATCTCGGCGGACGGGCGGCTCAGCT
CGCGGCCGCCGACGTGCGCAGACAAGTGCTCGAACACGCCGCGCCCATGCTCAAGACCGAGCCCGAGAATCTC
GCGATCCGGGACGGTTTCGTGGTCGTCACCAACGGCTCCAACCGCAGCATCCGGGTTTCCGAGGCCGTCGGGC
CGCAACGGGGGATGCCGGCGGTGCAACTAGTCGGCGTAGGCACCTACATGCCGACGAAGTCCTACTCCTTCGC
CGCCCATTTCGCCGAAGTCGAGGTGGACACCGAGACCGGCGAGGTGGCCGTTCTCGAGGTCGTTCCGGTACAT
GAGATCGGCAGGGTCATCCATCCGATCGCCGCGGCCGGACAGATCGAGGGTGGCATTCAGCAGGGCATCGGCC
ACACCCTCAGCGAGGATTACGTCATCGACCTTACCGACGGACGTTCCCTCAATCCGAGCTTCGTCGATTACAA
GATGCCCCTGTCGATGGACATGCCGTCCATCCGCACCATCATCCTCGAGACGGCACCGGATCCCGGCGGTCCC
TACGGTGCCAAGGGCGTCGGCGAGGATCCGATCATCGCGATCGGGCCCGCCATCGCCAACGCCATCTACGACG
CCATCGGCGTCCGCTTCCACCACTACCCGATAACGCCCGAGCAGGTGTTGAACGCTCTCAAGTCCAAAGCCAA
CGAAACGAGGCAGTGAATGGCAAACAGTGAAAGTGGACAGCCGCTGGCGGTCGTCCCCCAGGCCTATGGCGCC
CTTTGGCGAGGCCTGCTCGGTCCTTTACGCACGATTATCCAGCCGGCCGCCGCTGTTGTTCTGGCTCTCGCAT
GCGGCGCGGCGCTCCTTGCCTTCAACGGTTACGAAGCACGCGCCGTGATCGATGTGATGGTGCTTGGCGTCTT
CCAGGACACCCGCTCGATCGCCGAAATTCTCCTGAAAGCAACGCCGCTGATCCTGATCGGCGTCGGCCTTTGC
GTCGCCTTCCGCTGCAGCATCTGGAACATCGGCGCCGAGGGGCAATTCTATGCCGGCGCCATCGCCGCAACCG
CCGTCGGCGTTTCCTTCGACGGGTTGACCGCGTGGATCTACGTCCCGCTGGTCATGATTGCAGGCGCTCTTGC
AGGTGCGGCATGGGCGGCGATCGCCGGACTTCTGAAGGTCTATTTCCGGGCCAGCGAGATCGTGACGACGATC
ATGCTGAATTACATCGCCATTATCATGACGAGCTATCTCGTGACCGGCCCGCTGCGGGATGCGGCTGCGGCCT
ATCCGCAATCGGCCCGGCTGGTCCAGGAGGCATGGATGCCGCGCATCCTGCCGCCGACCCGCCTCCATATCGG
CATCCTCGTCGCCCTGGCGCTGGCCGTCGTGATGTACATCTTTCTGTTCCGCAGCACCCGGGGCTATGCGCTG
AGGGTGGTCGGCATCAGCCCGGACGCCGCGCGCTATGCCGGGATCAGCGTTCCCAGGAACATCATGCTGTCGA
TCTGCCTGAGCGGCAGCATGGCCGGGCTCGCAGGGGCCTTCGAGATCGCCGGCGTCACCCGGCGGCTCTTTCA
GTCGATCTCTCCGGGCTATGGCTTCGAAGGCATCGCGGTCGCTCTTCTCGCAAACAACAATCCGATCGGAGCG
GTCTTCTCCGGCCTCTTGTTTGCAATTCTGCGAAGCGGCTCCGAGCTCATGCAGATCACGGCACAGGTGCCTC
AGGTTCTCGTGCTCGTGATTCAGGGCATCGTCATTCTCTCGGTCGTGGGATTTGCCGTGTTGCGGATCCCGTC
GATGCGCCCTTCCGAATAGATGATTGATTTTTCCCGTGCCCGCGAACGGATGATCACGTCGCATCTCTCGCGA
CGGGGGATAAGGGACAGACATGTCGTGGAGGCGATGGGGGTCGTCCCGCGGGAGGCATTCGTCGATCCCGGCT
TCGAGGAATTCGCCTATGAGGATTCGGCCCTTTCGATCAGCCATGGCCAGACCATCTCGCAGCCCTATATTGT
GGCGCTGATGATCGAACGCGCCGAAGTCCAGCCGGGCGATACGGTGCTCGAGATCGGGACCGGATCCGGTTAT
GCGGCGGCGGTGCTCAGTCGGATCGCGGCATATGTCTATACGATCGAGCGCCATGCCGGCCTCGCCGAGGTTG
CCGAGAGACGATTTGCCGAGCTCCGTTACGGGAATATCGATGTGCGTGTCGGCGACGGCACGGCCGGCTGGCC
CGAGGCAGGACCCTTCGACGCCATACTGGTTGCCGCCGGAGGTCCCGAGATACCGTATGCGCTGAAGGAGCAG
CTCGACCTCGGTGGCCATCTCGTCATACCCGTCGGCCCCCCTGAGGAGCAGCGCCTGATGAAGGTCACGCGGG
TCAACGCCACGACATTCGAGGAGCAGGACCTCGGCGGGGTCAGATTCGTGCCGCTCGTCGGCGAACACGGCTG
GCATGAGGAGCGGGTTCAATCCGGGGCAAGGCTCAGAGCGGCGCCCACCTTGCCCGAGCTGGTTGCAGAGGCG
GCCGAGCCGCTGCCCGATCTCGACGATCCGGCGTTCGGGCTGCTTTTCGATCGCTTTGCCGGGCGGCGGATCG

TGCTGCTCGGAGAGGCGAGCCACGGCACGTCGGAATTCTACCGCGCCCGCGCAGCCATCACGCGCCGCCTGAT
CGAGGAACACGGGTTCACGATCGTGGCGGTCGAGGCGGATTGGCCGGATGCGGCGGCAGTCGACCGCTATGTC
CGACATCGAAGTCAAGGCATGCGCCTCGACGCGCCGTTCCAGCGGTTCCCGACCTGGATGTGGCGCAACCGCG
AAGTGATGGATTTCGTCGAGTGGATGCGCGAACATAACGGCCGCAGGAGCTTGCCTGACCGGGCCGGCTTCTA
CGGTCTCGACATCTATAATATGCGCGGCTCCATTTCCTCGGTTCTCCGGTATCTCGATGAGACCGACCCCGAG
GCCGCCGCGGTCGCCCGCGAGCGCTACGGTTGCCTGACCCCCTGGCAGAACGAGCCGTCGACCTATGGCCGTG
CGGCCATGACCGCGGGCTTCCGCAAATGCGAAGAGGCCGTCGTCAGGCAATGCCGCGAACTCCTGGAGAGGCA
GCTCGAAGCCGGGCGCGACAGCGGCGACGAGCTTCTCGACGCCGTGCAGAACGCGCGCCTCGTCGCCTCGGCC
GAGCGGTACTACCGTATCATGTATTATGCTGGGGCCGACTCCTGGAACATGCGCGACACCCATATGTTCGAAA
CGCTCGAGCATCTGTTGAATGCACGCGGCGCTCGCTCGAAGGCGGTCGTGTGGGCGCACAATTCCCATATCGG
CGATGCCCGCCATACCGATATGGGCACGGCCCGCGAGGAGTTGAACATCGGTCAGCTCTGCCGCGAGCGGTTT
GGCGATCAGGCAGCGCTGATCGGCTTCGGCACGCATGGCGGCCATGTGGCTGCCGCAAGCGATTGGGACGGCG
ATATGGAGGTGAAGCCGATCCGGCCGTCGCTCGAAGGAAGCTACGAGCGCGTGATGCACGACTCAGGGGTAGG
GTGCTTCCTCATCGACTTCGCGCGCCACGCGCGTCTGCGCGACGGTCTCCTCAAGCCGATGCTCGAGCGCTTC
ATCGGGGTGATCTACCGGCCGGACACGGAACGCTTCAGCCATTATGCCCACGCTTCGCTGCCCCGGCAGTTCG
ACGCCTTCGTCTGGTTCGATCAGACGACGCCGGTCGAACCGCTCGGCGCGCAACACATAAAGGCGGGCGTGCC
GGACACGTTCCCGTTCGGCCTGTGAGTGAGCCGCGTGCTGACCCTGGCCGGGCGAACGGGGACGGGACGCGTC
GGCGCGGCTCTCGCCGCGCTCGTCATCCTCTTCGCAGCACTCTGGATGGGCGCTGCGATCGGCGAAACGGCGA
TCCCGTTCAAGACCGTTGCGCAGACGGTGGCGAACCGGCTGTGGAACGCCGGCTACCCGCTGGCACCGATCGA
CGAGGGCATCATCTGGAGCTACCGGCTGAGCCGGGCAGTCGTGGCCGCTTCCTGCGGGGCGTCGCTCGCGCTC
TCCGGCGCCGTGCTGCAATCGCTGCTGCGCAATCCGCTCGCCGACCCCTACATTCTCGGCATCTCCGCCGGCG
CCTCGACTGGCGCGGTCAGCGTCGCCATTCTCGGTGTGGGTGCCGGGATGCTGACGCTGCCGCTCGGCGCCTT
CGTCGGCGCGCTGGTCGCTTTCGTCCTGGTGAGCCTGCTGGCGGTGAAGGCCGGCCGCGGCACCGCGGCGATC
ATCCTGGCAGGTGTGGCGGGATCACAGCTCTTCAACGCGCTGACCTCCTTCATTGTCACCAAGGCGGCGACGG
CCGAGCAGGCGCGCGGCATCATGTTCTGGCTGCTCGGCAACCTTTCGGGCGTGCGCTGGCCGGATGCCTGGCT
CGCTGTGCCGGCGACGCTTCTCGGTCTCGTCGTATGTCTCTGGCATGCCCGTCCCCTCGACGCGTTTACCTTC
GGGTCGGAATCGGCTGCCTCGCTCGGCATCTCGGTCAGGCGAACCTATTTCGCCCTCGTCGGCGTCTCGGCGA
TGATGACGGCGGTGATGGTATCCATCGTGGGGTCGATAGGTTTCGTCGGCCTCGTCATCCCCCATGCGGCCCG
CATGCTCGTCGGCGTCCGCCACGGTGTCCTGCTGCCGGCAGCGGCGCTCATCGGCGCCGTATTCATGATCCTC
GCGGACATCCTGTCGCGCGTTCTCATTCCCGGCCAGGTCTTGCCGATCGGCGTGATCACGGCGCTCGTCGGCG
CCCCGGCCTTCGCGTTGATACTCGGACAAAGGAGGGACCGCGCATGAATGACCATCGAAGCCGAAAGCACTCT
TCCGGTGGCGATCGAGCCCGAGGAAAAGCACCACCACGAGAGCTACTCCGCTCTCGTCTGGCGCCGGTTGAAG
CGTTCCTGGACCGGCCTGCTCGGCCTTGTCCTCGTCTGCCTCCTGATCCTGATGGCGGTCTTCGCCGAGTTCC
TGTCGCCCGTCGATCCGAAGGCGACGGACGTAGCCTTCGCTCAGCCGCAGACGATCAGCTTCCGGGACAAGGA
GGGTAATTTCGTCTTTCCGCCGAGGAGCTATCCGGTGCGGGAGACGGAGGAGCTCGATCCCGTTACCTTCCAG
CCGATCATCGGACCGGATTACAACAACCCGCAGGTCCTCGGCTTCTTCGTCAAGGGCGCACCCTACAGGCTCT
TCGGGCTGATCCCGGCCGAGCGGCATCTGTTCGGTGCCGTCGACGGTACGCCGGTGCATCTGCTCGGCACCGA
CAAGTTCGGCCGCGACGTGCTGTCCCGCATCCTCTACGGCTCGCGCATCTCGCTGATGATCGCGCTCACCGTC
GTCTTCATCGTCACCGTGGTCGGGACCACCGTCGGCATGGTGTCCGGCTATTTCGGCGGGCGCTTCGATGCCT
GGGTGCAGCGTTTCGTCGAACTTGTGCTCGCCTTCCCGCAATTGCCGCTCTATCTGGCGCTCGCCTCGCTGAT
CCCGGTCACCGCGCCGACCAATGTCTTCCTCGCCTTCGTCATCATCGTGATGTCGGCGCTCGGCTGGGCGCAG
ATGTCGCGCGAGGTGCGCGGCAAGACCCTGGCGCTTGCGCGGATCGAATATGTGCGGGCGGCGATCGCCATCG
GCGCGACGGACCGGCGCATCATATTCCAGCACATCTTCCCGAATGTGATGAGCCACGTCATCGTCGCGGTGAC
GCTCGCCATCCCGCATGTGGTGCTGCTCGAGTCTTTCCTCGGTTTCCTCGGCTTTGCGGTCAAGCCGCCGCTG
ATCTCCTGGGGGCTGATGCTGCAGGATACGGCGAATTATTCCGCGATTGGTTCCTATCCCTGGATTCTCTCGC
CCGTCGCCTTCGTGCTCGTCACCGTTTTCGCCTTCAATGCGCTGGGCGACGGCCTGCGCGACGCAATCGACCC
CTATTGAGTGAAGCTCGATCCCTTCTATCTCATCGTCGACAGCGCCCAATGGATCGAACGGCTGGTGCCGCTC
GGCGTCAAACTCGTGCAGCTCCGCATCAAGGACCGGAACGAGGAAGACATCCGCCACCAGATACGGGTCGCCA
GGGCAGTCTGCTCCGCCCATGCATGCCAGCTGATCGTCAACGACTACTGGCAGCTGGCGATAGAGGAAGCCTG
CGATTTCATCCATCTCGGTCAGGAGGATCTGGCGGAGGCCGATCTTGCCGCCATCCGTGCCGCCGGGCTCAAG
CTCGGCGTCAGCACCCATGACGAGGCGGAACTGGCGAAGGCGCTCGCAGCCGAACCGGACTACGTCGCGCTCG
GGCCGATCTATCCGACGATCCTCAAGAAGATGAAGTGGGCGCCGCAGGGGCTGGAGCGGCTTTCATGGTGGCG
CGAGCGCGTTCACCCGCTCCCGCTCGTCGCCATCGGCGGCCTCAATACGGAGCGGATCGAAGGCGTCTTCGCG
CATGGCGCCGACAGCGCCGCCGTGGTGACTGACATCACGCTGAATGCCGATCCGGAAGGAAGGACAAGGGAGT
GGATACGGAAGACGGAGGCGTGGAGATAGGTGGGGCAAGCTGAGGCAGCAACGGGAAAAAAGAGACGCAAGGC
GCTTTGGGCCGCAGTTGCGGTGATAGCAATCACCGCCGCCGGCATGGCCTGCTACAAGACCATCGTAGAAAAA
CGCGTGAGCAGGATGATCGCCGAGCGCGGCGGCAAGGCGGGCAGCGTCGAAGCGGATTTCCTTGGGCGAATTC
ATCTGCGCGACGTGGCCCTGCCGCTGAAGGACGGGTCGGACGTTCGTATCGCCGCGGTTGACGGCCGGCCCAG

TATCTTTTTCCTCCAGGGCATGCTCGAAGTAAGCGGCCTTGAACTCAAGACTGCTTTCGGCACGGTCTCTGTC
CCGCGCGCGGTCGTCGAAGAAGCAGATTTTGATCGCGGCACCTTGGAGCAGATGTTCGGCGGGAAGAGCGAAC
TCTCGATCTCCAAGCGAGTAGAACTTTTCACGGCAAGGCGTATCTCCGCCCCGGAGATCGTCTTCGCACAGAT
CGTTGCCGGGACCGAGCAGAAGCTGATCTACAGGAACGTGGTCTCGGAAGGCATCGCCGATGGGCGTATCGCC
CGCTATTCGGCGAGCGGCGGCAGCTTCGAAGTCGGCGTGGACCGTTCGGGGGGAGAGGGCGGCAGGAAGAATA
GGGTGGCGGGTTCCATCGGAGCCGTGACGGCGCAGGATATCGATGCCGCCTATCTGGCGCGGCTCTATACCGA
AAAAGCCGGCTCGGACGACAACGAGGCAAAGCCTGTCTATGGGCCGATTTCGGTAAAGGACATCGCCTTCTCG
GATGGCGAGGCGAGCTTCGGCTATGACGAGGTTCGCAGCAGCGGCTTCAGCATGCGAATGCCGGCCGAGCCGC
TGGTCGAGACGCTCAAAAAGCTGAAATCGGTCTCGGCGCCGGATCAATTGCCGCCGGCGGAACGCCAAGCCTA
TTTCAATCGGGTCTTCTCGATTATCGACATGACGAAGAAGGCCGACATAGAAATGCTCGGCCTTACGATCGAC
GCGCCGATGAAGGACGGTGACGAAGGCGAGAGGATCAAGGTTTCGATAGATCGCCTCGCCCTCGAGGTCGATG
GACGGAAGCTTGACGGCACGCTGAATGGCCTCTCGATGGGAGAGGGCGCGGACTACGTCAAGGTCTCCGAAGC
GAGCATTTCGGGATTTTCCTGGAATTCGACCTTGGAGGGTCTGCAGAGGATGGTCGCGCTCGACGAGCAGCAG
CGCGAGAGATTTGCCTTCACGACACTGTTGCCGGAATTCGGTACGGTCCGGCTGGCGGGTCTGGATGCCGATC
TGCCCGATACAGAAACCGAAGACGCCGAGAGCGACACACAGGATTCGGTGCCGAGGCGCGTTCGCTTCTCGCT
CAAGGATTACGAGTTGGCGCTCACGAAGCCACGCAACGGCATTCCGACCGACATCCGCATCAGATATCAGGAT
CTGACCCTGCCGATCCCGGAGCATATGCACGACGAGCCGTTCGCGCAGCTTCGCAACCTCGGTTTCAATGAGG
TGGTCCTGTCGTCCAACATCGAAGCCGCCTGGGACGAGCCGAACCAGACTCTGGTCATCAAGGATATCTCCGC
ACGTGGAAAAGATATTGGCGGCTTTTCCCTCTCCGGCCTGCTGGGGGGCGTGAGCGGCGACTTCTTTTCCGGC
GACGAGGCGAAGATGCGGGCAGCGCTGTTCGGCCTCACCGCCCGTGAAGCAAAGCTGAAGATCGAGGACAAAG
GTCTCGCCGCCAAGGCCATAAAGGCCTATGCCGAGCAAAACGACATGACGGAGGATCAGGCGCGCGGCATGCT
CACGATGACGGCTTCGGTGGCGTTGCAGCAGTTTGCCGCGCAGGAGCCTCGGTTGCAGGACGTGCTGGACGCA
GCTTCGCAGTTCATTGCCAAACCGGGGATGTTCACGTTGACGGTCAAATCCAAATCCGCAAACGGAATCGGTG
CGCTCGAATTCGCGGTTGCTTCGCAAAATCCGCTCTTGCTTCTCGACAGGGTCGACTTCGAGGCGACGGCGGA
ATAGATGATCACCTCGTTTCTCGTCAAATCGCTGGATCGGACGATCGTCGTCGCTGTCGCGATCCTGCTGGCG
CTGGCCGTTATCGTTCCCGCGCTCAACCTCCTGACCGCGCCGGAACACCCGCTGCATGTGCCGACCTATCTGG
TCTCGCTCTTCGGCAAGTACCTGACCTATGCGCTCTTGGCTCTGGCGCTCGATCTTGTCTGGGGTTTCTGCGG
CATCCTCTCGCTGGGGCATGCGGCCTTCTTCGCGCTCGGCGGCTACGCCATGGGCATGTATCTGATGCGCCAG
ATCGGCGCACGCGGCTCCTACGGCAATCCGCTGCTGCCGGACTTCATGGTCTTTCTCAACTGGAAGGAGCTGC
CCTGGTTCTGGTACGGCTTCGACATGTTCTGGTTCGCAGCGCTGATGGTGGTGCTGGTGCCGGGCCTGCTTGC
CTTCGTCTTCGGCTGGTTCGCCTTCCGCTCGCGCGTCAACGGGGTCTATCTCTCGATCATCACCCAGGCGATG
ACCTATGCGCTGCTGCTCGCCTTTTTCCGCAACGACATGGGCTTCGGCGGCAATAACGGGCTCACCGATTTCA
AGGACATCCTCGGCTTCAACATCCAGGCCGACGGAACGCGTGCGGCACTCTTTGCAGCCTCCGCGCTGGCGCT
TGCCGTTTCGCTCGTCGTCACCTCGGGCATCGTCCGCTCGAAATTCGGCAAGGTGCTGGTGGCGCTGCGCGAC
GCGGAAAGCCGCACACGCTTTCTCGGCTATCGCGTCGAGCACATGAAGCTCTTCGCCTTCACGGTCTCGGCAA
CGATGGCGGGCGTCGCCGGCGCGCTCTACGTGCCGCAGGTCGGCATCATCAATCCGGGCGAGTTCGCCCCCGC
CAATTCGATCGAGGTGGTCATATGGACGGCCGTCGGCGGGCGCGGCACGCTGATCGGTCCGATCGTCGGCGCG
ATCCTCGTCAACGGCGGAAAAAGCATCTTCACCGCCGCCTTCCCCGAATTCTGGCTCTTTGCGCTCGGCGGGC
TCTTCGTCCTCGTCACGCTTTTCCTGCCGAAGGGCGTGGTCGGCACGGCGCAGAACTATCTCGCAAGCCGGCG
CGCATCACACGCGGCGGCCCGGAAGGAAAGCGAGAGCGAAAAAGCGGCGAGCCTTGCGGACGCCGAAACGGTG
GCCGCGGAGTGAATGACGACATCAGGAGCGGACGGAACGAAGGAGCGGCGCCCTGTGATTGCGCTGGCCATGG
GCGACCCCGCGGGAATCAGCCCGGAACTGACGGCGCGGCTGCTGGCCCTTTCCGACATCCGCGACGCGGCGCA
CATCATCGCCATCGGCGATCGCCGCATCCTCGACGAGGGCGCCCGGGTGGCCGGCGTCACGCTGGATCTGGAA
GCCGCTTCGCTCGATGCGCTCGACAACGCCGGCACCGCCCGCCACGTCTTCGTGGATCTCGCCCATCTCGATC
CGGCCGATGTGGTGCGCGGCGAGGCGACGCTGGCCGGCGGCACCTTCGCCACACGCAATTTCCGCACCGCGCT
CGAACTCGCCCATGCGGGCAAGGCCGATGCCGTCTGCTTCACGCCCTTCAACAAGAAGGCGATGCGCTTTGCC
TATCCCGGCTATGACGACGAGATCCGCTTCGTCGCCGACGTGCTGTCCTTCACCGGAAAGGTTCGTGAATTCA
ATGTTCTCGAAAAGGTGTGGAACGCGCGGGTGACGTCGCATATCCCGCTCAAGGACGTGGCATCCCACCTGTC
GGTGGAGGCGATCCTTGCCGAACTCAAACTGACGCAGGCCTGCCTCAAGGATGCCGGCTACGAGGAGGCGAAG
ATCGCGGTTGCGGGACTCAATCCGCATGCCGGCGACGGCGGCAGCTTCGGCATGGAAGAGATCGACATCATCG
AGCCTGCGGTCGAGAAGGCCAGGGCGCTCGGCTTTAATGTCGAGGGGCCGTTCCCTGCCGATACGGTCTTCCT
CCGGGCACTGAAGGAAGGCTTCAACGCGGTGATGACCATGTATCACGACCAGGGTCAGATCGCCATGAAGATC
ATCGGCTTCGACAAGGGTGTGACGATGATGGGCGGACTGCCGTTCCCGCTCTGCACGCCCGCCCACGGCACCG
CCTACGACATCGCCGGCAAGGGTATCGCCGATATAGGCGCAACACGCGAGGCGATCCTGCTTGCAGCCCGCAT
GGCGAAGAAAAAGAGGGCGCTTTCGGCGGCTGCCTGAATGTGGGGAGAAACCGGTTGGGGCATGCCGACGTCG
CTCGCCCTGCACCTGGCGCTCGCCTTCTTGCTGCTGGTTCGGCTGCCGGAATTGTCTGCGCCGGCAAAGGAAC
AAAGCGTCAGCGTCGAGCTCGTTCCGCAGCCGAAGAGTGCGGAAAAGCCCGAGCAAAAGCCGAACGCGGCCAA
GAGCGAGCAGGCGCGCCCTCAGCCTGAGGCGTTCGAGTCGGCGGCAGCGCCGGCCGAAAAGGAAAAGCCGCCT

CGGCCCGAGTTGCCTCCGGCCGCGCCGAAGAAGACCGAAACGCCGAGCGAGACGCCGGAGGCCTCGCGGCCCG
CCCCGGCAAAGACGGAGAACTCCGCCAGCGAAAAGCCGGCGGAAAGCAAGACAACGCTTGCGGAATTGCGCGT
CGATGCCCGGAGCGCAGCGAACGAGGCGGCCGATGCTGCCGCGGCGCCGCCGGCTGCAGCTGTTCCCCAGGAG
AAACCGGTTCTGGAGGAAGCAAGGGCGGAGCCGACGCAAAAGCCGGCCGAGGCGAAGGCCGAGCGCCAGTCGA
GCCAGCTCGTCCAGGCGAAGGAACTCTATTCTGAAAACGCCCTGTCGGACCCGCGCGTGAAACAGGCGATCGG
CAGGCTGCCGCCGAAAAAGCGGATCGTACAGCTCTGCAGCATCGAGGCGCTCGAGCAGGTGCGGCGGGAAATG
CCCGGCGCCTTTCCGGACATGCTCGTTCCCTTCGGCCCTTCCGGGGGCTTCATCTCGGAAACGGGACTAAACG
CCGATGGCGGCGCCTTCCGCAGCCGGTCGACATGGTACGCCATCGACTTCAAATGCGAAGTGAGCCCCGAAAC
GACATCGGTCACTTCCTTCAGCTTCGCGATAGGCAAGGCGATCCCGAAGAGCGAATGGGGTGCGCGACAACTG
CCGAGCAATTAAATGCAAGACGCATCGATACCGCGTCCGCGGATTTCCAATGCCATAGACAACGTAGCCCGCG
CGGTGGGCGGCCTGGCCATCGCCACCTTCACCGCCATCATCGTCTACGTCGTCATTTGCCGGTATGCCCTTTC
CTTCACACCACGCTGGTCGGAGGAAATCCCGCGTCTTCTATTGGTTTGGGTGACGTTCATCGGCGCGGTTTCC
GCCTTTGTCCGCAACACGCATCTTTGCGCCGGCCTGACGGACCTGCTTGTTGCGCCCGGCTGGTTCCGTTCCC
TCCTGGCCGTGCTCGCGCGTCTGGCATCCCTGCTGTTCCTGGTCGTCGTCATCTGGTCCGGATGGAAGATAAC
GGTGCTGACATGGTCGCATGAAACCACGGTCCTAAGCTGGCCCGCGGGGCTCGTCTATCTCGCGCTGCCGGTC
ACGGGTGCCATCGCCTTCGTTGCCCTCGTTGCCGCGGAACTGCGCAAATGAATGGACAACATCATTCTTGAAA
TGCGGGGCATTACGAAGACGTTTCCGGGCGTCAAGGCCCTGGACAATGTCTCGTTCAAGGTCCGCGAAGGCGA
GATCCACGCGCTTGTCGGGGAAAACGGCGCCGGCAAGTCGACCTTGATGAAGGTGCTGAGCGGAGTCTATCCG
GCCGGCACCTATGAGGGCGAGATCCATTACGACGGCGAGCTTCGCCAGTTCAGCACGATCTCGGACAGCGAAG
AGCTCGGCATCATCATCATCCATCAGGAACTGGCGCTCGTTCCGCTGCTTTCGATCGCCGAGAACATCTTTCT
CGGCAACGAGGTGGCCGAAAAGGGCGTGATCCACTGGCCGCAGACCTTTGCCCGCACCCAGGAGCTCCTGAAG
AAAGTGGGCCTGAACGAGTCCCCCGCGACGCTGATCACCGATATCGGCGTCGGCAAGCAGCAGCTGGTGGAGA
TTGCCAAGGCGCTTTCGAAGAAAGTCCGCCTGCTGATCCTCGACGAGCCGACCGCATCCCTCAACGAAAACGA
CTCCGACGCGCTTTTGAAGCTGCTCATGGAGTTCCGCAGCCAGGGCATGACGTCGATCATCATCTCTCACAAG
CTCAACGAGATTAAGAAGGTTGCCGACCAGATCACCATCCTGCGCGATGGCGGAACGGTAGAGACGCTCGACT
GTCACAAGGAAGACATCAGCGAGGACCGGATCATCAACGGCATGGTCGGCCGGGCCATGGAAGACCGCTATCC
GCCGCGTGAACCGAAGATCGGTGACACGCTGCTCGAGGTGAAGAACTGGAACGTCTACCATCAGCATCACCGC
GACCGGCAGTTCCTGCATGACGTCAGCTTCAAGGTTCGCGCCGGCGAGGTCGTCGGCATCGCCGGGCTGATGG
GCGCCGGCCGCACCGAGACGGCGATGAGCGTTTTCGGCAAGTCCTGGGGCCACAAGATCACCGGCGAGGTGAC
GATGCATGGCCGGCCGGTGGACGTGAGCACGATTCCCAAGGCCATCAAGGCAGGTCTTGCCTATGTAACGGAG
GACCGCAAGCAGCTCGGCCTCGTGCTGATCAACAACATCATGCACAACACCACGCTCGCCAATCTGCATGCGG
TCGCATCGAACGGCGTCATCGACGAGCGCAAGGAACGGAAGGTCGCCGCCGAATATCGCTCGAAGCTCCGCAT
CCGTTCCCATTCGATCTATCAGGAGACGGTCAACCTCTCTGGCGGGAACCAGCAGAAGGTCGTCCTGTCCAAG
TGGCTGTTCACCAATCCCGAAGTCCTCATACTCGATGAGCCGACGCGCGGTATCGACATCGGCGCGAAGTATG
AGATCTACACCATCATCAACCAGCTCGCGGCCGAGGGCAAAGGCATCCTGATGATCTCGTCGGAGATGCCGGA
GCTGCTCGGGACCTGCGATCGCATCTATGTCATGAACGAGGGGCGCATGGTGGCCGAGCTTTCCAAGGAGGAA
GCAAGCCAGGAATCGATCATGCGCGCCATCATGCGTTCAGGGGAGAAACACTAAATGATCAATGCGGACAGCA
GCCCGCGCCAGCCATATTCGACTTATCATCAATGGTATGCCAGTCAGGACAGAAACCGGCTCATCGCGAAATC
GAAGGAAGCCGAAAAGATTTTTCGTAAGACGGGCATCACCTTCGCAGTCTACGGACACGCGGATTCCTCCGAA
AAACTGATCCCCTTCGATCTCATACCCCGCATCATCTCCGGGCGCGAATGGCGCCGGCTCGCCCAAGGCATCG
AACAGCGCGTCATCGCGCTCAACGCCTTTCTCGACGACATCTACCACAAGCAGGAGATCATCCGCGCCGGGCG
CATCCCGCGCGAACTGATCGAGAAGAACGACGCCTTCCTGCCGCAGATGATCGGCTTCCGGCCGCCGGGCGGC
GTCTACACCCACATCGTCGGTACCGACATCGTACGCACCGGCGAAGACCAGTACTATGTGCTGGAGGACAATG
CCCGCACGCCGTCCGGCGTCAGCTACATGCTGGAAAACCGGGAAACCATGATGCAGATGTTTCCGGAACTCTT
CCACCAGAACCGCGTGCGGCCGGTGGAGAACTATCCCAATCTGCTTCGCCAGAGCCTTTCCTCGCTCGCTCCG
GCCGGCTGCGAGGGCAAGCCGCGCGTTGCCGTCCTCACGCCCGGAATCTACAACTCCGCCTTCTACGAGCACG
CATTCCTCGCGGATATGATGGGCGTCGAACTGGTGGAGGGCTCGGACCTGCGCGTCATCGACGGCAGGGTAAA
GATGCGCACGACACGCGGCTACGAGGCGATAGACGTGCTTTACCGGCGCGTCGACGACGATTTCCTGGACCCC
CTCACCTTCCGCCCGGATTCCGCCCTCGGGATACCGGGGATCATGGATGTCTATCGCGCCGGCAATATCACCA
TCGCCAATGCCCCCGGCACCGGCATTTCCGACGACAAGGCGATCTATTCCTACATGCCGGAAATCGTCGAATT
CTATACCGGCCGCAAACCCCTCCTCGAGAACGTGCCCACCTGGCGCTGCTCGGAACCGGAGAGCCTCAAATAC
GTGCTCGAGCATCTCGCCGAGCTCGTCGTCAAGGAGGTTCACGGCTCCGGCGGCTACGGCATGCTCGTCGGCC
CGACCGCGACAAAAAAGGAATGTGCAGCCTTCGCGGAGAAGCTCAAATCCCGCCCCGGCAACTACATTGCCCA
GCCGACATTGTCGCTCTCGACGGTGCCCATCATGGTCAACAAGGGCATTGCGCCGCGCCACGTCGATCTGCGT
CCCTATGTGCTCGTCTCCGACAAGGTTCAGATCATTCCGGGCGGCCTGACACGCGTGGCCCTGAAAGAAGGCT
CCCTGGTGGTGAATTCCAGCCAGGGCGGCGGGACCAAGGATACCTGGGTGCTGGAGGACTGAGTGAAGACGCT
CAAATTCCTGGTTCTGGCCGGCTCCCTCGTCGTGGCAGGCGGAGGAGGCTACGTAGCCGGCACAGGCGGCCTT
ACCCAACAGTTCTTCCTTGGGGCAACTGCACGAGAACCCACGCCGCCTGCGGCAAAGCCGGCGGGCAAGATCA

TCTACTACCGGCACCCGGACGGGGTGCCGGAATATTCGGCGGCGCCGAAGCAAACCGCGGACGGGCGCCCGTT
CGTCGCAGTCCGGCAGAGCGAGGATGTCTCTTTCGAGCCGCTGAAGGCGGTTGCCGAGGGCCCCGCCGCCGGC
GAGCGAAAAATCCTCTACTACCGCAACCCCATGGGGCTGCCGGATACCTCCAAGGTGCCGAAAAAGGACTCCA
TGGGAATGGATTACATTCCCGTTTACGAGGGTGAGGAGGCAGACGGCTCGACCGTGAAGGTGTCTCTCGGCAA
GCTCCAGCGGACGGGTGTGAAGACGGCATCGGCGGAGCGTGCCGTCATCGCCCGAAGGGTTCGCGCGCCCGGC
ATGATCGCCTTCGACGAGCGGCGCATCAGGATCGTCTCGACGCGGGTGGATGCGTTCATCGAAGACGTCGCGG
AAATCACGACCGGCGACCGGGTCCAGGAAGGCGAGAACCTCTTCCACTACTATTCGAAGGACGTCGCCAGGGC
GGGAGCGGAATACGCCACGGAACTGCGCGGCGGCGTCAAGCCGGGCCTCGAAGTCGGGGGAGCCCTGCAATTG
CGCAATCTCGGCGTGCAGGAAGAGACGATCCTTTCGATTGCCAGGGATCGTACGGTCCCGCGGAGCATTGCCT
ATCTTTCGCCGAGCGACGGCGTCGTCCTGGAACGCAATGCCATCCCGGGCATGATGGCCAAGGCCGGCGACGT
CCTGTTCCGCATCGCCGACACCTCCAGCATATGGGTCCTGGCCGACATTCCGGAGTATGATGCCGCAGCCGTG
AGAAAAGGTGCTCCGGCGAAGGTCACAGTACGAAACCTTCCGGGCAAGGTCTTCGAGGGCAGCGTCGATCTGA
TCTATCCCGAGCTTCGCTCGCAGACGCGGACGGCGAGGATCCGGATAGAGCTCCCCAATCCGGCGGGGCAGCT
CCTCGCCAACATGTATACCGAGGTCGAGATCGTTACCGGCAAACCGGAGCCCGTCATGACGGTCCCGAACAGC
GCCGTCATCGACACCGGCAACCGCCAGCTCGTCTTCGTGGACAAGGGCGAAGGCCGCTTCGAGCCGCGCGAGG
TCGCGCTCGGGGCACGCGACAGCGAAAGGACCGAGATCACGAAGGGGATCGAAGCCGGCGAGAAGGTCGTGAT
TTCAGCCAATTTCCTGCTCGACGCCGAAAGCAACCTCAACGCAGCGCTGAATGCGCTTGCCTCGGCGGAGGCG
CAGCCATGAATGGCACAGGCATCCGACAGCATCCGCTTCATCCTGAACGACTCCGAGATCGCCCTTTCCGACG
TCGCGCCGACGTCGACGCTGCTCTCTTTCCTGAGGCTGGAGCGCCGCCTCACCGGCACCAAGGAAGGCTGCGC
GGAAGGCGACTGCGGCGCCTGCACGGTGCTCGTGGGCCGGCTCGCCGAGGATGCCAGCGGAGCGAAAAAACTC
GTCTATCAAAGCGTCAACGCCTGCATCCGCTTCACAGGCTCGCTGAATGCCACCCATGTGGTGACGGTCGAGC
ACCTGGCTGCCGCAGACGGCACATTGCATCCGGTGCAGCAGGCACTGGTGGATTTTCACGGCTCCCAATGCGG
CTTCTGCACTCCGGGGATCGTGATGTCGCTTTACGGGCTCTGGCTTGCCGATGAAAACCCGAGCCGCGCGGCG
ATAGAAAAGGCGCTGCAGGGCAATCTCTGTCGCTGCACCGGCTACGAACCGATCGTGCGCGCGGCGGAGGCTG
CGGCCGCGGAGCGCCCGGCCGCTCTCATCGACCCGGTCACCCGCACGCGGGAAGCGGTTACGGCGCGCCTTCA
TGCGATGGGGCAGACGGAGACGATCGTTGTCCGCAGCGGCGCGGACTGTCTGATCGTACCCGTCGACGCTGCG
GACCTGGCAGGTGTTCTCGCTGATCATCCAGGCGCGACGATCGTCGCCGGTTGCACCGATGTCGGGCTCTGGG
TGACGAAGCAGATGCGCTCGCTCAATCCGGTGGTCTTCATCAATGGCATTGCCGAACTGCAGCGGATCGAAAG
CTCGACAGCAGGCCTGACGATCGGTGCAGGCGTCAGCTATACGGCGGCGCACGAGGCCCTGTCTTCAGCCTAT
CCGTCATTCGGCCGGCTGCTCGACCGCATCGGCGGCGACCAGGTGCGCAACATGGGCACGATCGGCGGCAATA
TCGCCAATGGCTCGCCGATCGGCGACAGCCCGCCGCCCCTGATCGTTCTCGGCGCGACGGTTACCCTGCGCTC
GAAGGACGGCCAGCGGACGCTGCCGCTCGAGGATTTCTTCATCGCCTATGGCAAGCAGGATCGCAGGCCCGGT
GAATTCGTCGAGAGCCTCTTCGTCCCCGCTCTGCCCGCGGACGAGCGCTTTGCCGCCTACAAGATCTCGAAGC
GCCGCGACGAGGACATTTCCGCCCTGCTCGGCGCCTTCCGCATCTCGCTCGACGGCGACCGGGTGAAAACCGC
ACGGATAGCCTTCGGCGGTATGGCGGCAACTCCGAAGCGGGCGAAGACCGTCGAAGCAGCGCTGATCGGTCAG
GCCTGGACGGAGGAGACGGTCCGTACTGCGCAGGCCGCCTTCGAGACCGACTATCAGCCGATCACCGACTGGC
GGGCCACCGGTGCCTACCGCATGCTTGCGGCAAAAAACCTGCTGATGCGCTTCTTCCTCGAGAGCATCGGAGA
GACGGTGCAGTTGCAGCGGTTCGAGGAAGTCGCATGAATGTCGTTCCTGAGAAGCGTGCGGCCCAGCAATCTG
ATCATTCTGACAGCGCTGCCGTTCATCATCTATCTTTTCGCCTCCTCCGGAGACTACCAACGTTCGCTGAGAG
CCATTCTCGGAGTCGAGAACGGCTCGTCCGCCTTCGTACCGGGTTTCCTTGCTCTGCTCGTCGCCTTTGGCTC
GGGTGTCGCCGCTCTCGTCTTTTCCCGAGCCAAGGCGCCGCGGCCGCGGCTGGCGCTCGCTGCCGCCGTACTC
AATCTCGCTGCGGCGGCCACCGTGCTTCTGAGCGACGCCGTTCACCCCTACCTTGCGTCGGTCGTCGCCAATG
CCGTCGATTCCTTCACGTCCGATCTCGTTGTGAAGGGGCTTAGCCCTCGGCAACTCACGGACGCCGCGGACAT
GACCCTGCGCGGCATCGAGGCCGTGCTTTTTCCGGCCTATTTCGCGCTGACACTGCTCGGCTTCATGCTTTTC
TTCGGTGCCGGCAAGGCATCCACGCCGGAAAGCATCATGCGCCGCTTGGGAGCGTTAAGCATCGGCCTCGTCA
ACGGTGCGGGGCTCCTGTTCATCCTCTTCTTTGCGCATATCGCCTTTGCCGCCGGCGTGGCGACGACGGTTCG
CGCAGCCATTGCCGCCTATCTTCTCGCCGCGATCATCGGCCTCATCTGGGTCGGATTGCTTCAGTTGCATTAT
ACGGCCCGCACCAGCATCATCTTCGCGCTCGCGACCCTCGGCCTGACGCTCGTCGCCGCCTGGTTCCTGTTGC
AGCCTCGCACGGCCTATGTGCTTGCGGGGCGGCTCGACGGCACGGTCGCGATCGTCAGCAACACGCCGTCCGG
CCTCATAGGCGCGGTGCGCTTCGGCCAATATCCGGGCGCGCCCGGTGAAGAGGCGGCGGTGCGAACCTTCCTT
GGCCCGGCGGAGGCAGTCGCGGCGATGGGAAAGAACGAGGTGACGGCGGCGCTTCTGCCGTCGGCAGCCGTAC
CCGGGGGCTTGCCGCTCCTTTGGGAAACGGCGGCTTTGAACGATCGCGACCGGGCCGCAGGCATCACTTTCGC
CGTCCTGGCAATTATCCTCGGTCTGCTCACCTTCGGCGGCTATCTGCACCGCCGTCATCCCCTGGCGATCGGC
TCGGAGTTCGTCGTCGACACGATTCGCGGCATTCCGATGCTGGTGATCGTGCTCTATGTCGGCCTGCCCTTAA
GCGGGGCGCTGAAGGATGCGACACAAGGCGTGCTCGATCCGCCGAACCTGATGCGCGGCATCGTCGCCATGGC
GCTCGCCTATTCCGCCTATCTGGCGGAAATCTTCCGCTCCGGCATCAATGCCATCCCGATCGGCCAGATCGAA
GCGGCGCGCAGCATCGGGCTCAACCGGTGGCAGACGGCGCGGCTGGTGGTCCTGCCGCAGGCGTTCCGGATCA
TCATTCCACCGCTCGGCAACGAGCTGATCGCGATCCTCAAGGACACGTCGCTCCTGTCGATTCTGTCGATCCG

CGACATCACCCAGCGCATGCGCGAGTTCCAGTCGGCGAGTTTCCTGCCCTTTGCGCCCTATAACTCGGCAGCC
ATCTTCTACATCTTCCTGACGCTCGCGGCGGCGAGCCTCATCAACATGATCGAGCGGAAATATGACATCAAGC
ATCGATAGATGAACCAGATGTCCAAGATTTCGGTGACGGTGAATGGCCGCCGCTATCCGTGGCCCCGCGTACC
GGCAATTGCGGTCTGCCTCGACGGATGCGAGCCGGCCTATCTCGATGCAGCGATCGACGCCGGGCTGATGCCC
GCGCTGAAACGCATCAAGGAACGGGGCGCCGTGCGTCTGGCGCACAGCGTCATCCCGAGCTTTACCAATCCCA
ACAATCTCTCGATTGCAACCGGCAGTCCGCCGGCGGTGCACGGAATTTGCGGGAACTACCTCTACGAGCCGTC
GACGGGCGAAGAGGTGATGATGAATGATCCGAAGTTCCTGCGCGCGCCCACCATTTTCCAGGCTTTCTATGAT
GCAGGCGCCCGGGTCGCGGTCGTCACGGCGAAGGACAAGCTGCGGGCGCTGCTCGGAAAAGGGCTCCGTTTCG
ACGAAGGTCGCGCCGTCTGCTTCTCGTCCGAAAAATCGGACAAGGCGACCCGCGCGGAGCACGGAATCGACAA
TGCTTCCGCCTGGCTCGGCCGGCCGGTTCCCGAGGTCTATTCGGCAGCGCTTTCGGAATTCGTCTTCGCTGCG
GGCGTCAAGCTGCTCAGGGAGTTTCGTCCCGACATCATGTATCTGACGACGACCGACTATGTTCAGCACAAAT
ATGCTCCCGGCGTCCCGGAAGCCAATTCCTTCTACGAAATGTTCGACCGGTATCTCGCCGAACTGGACGGGCT
CGGAGCAGCCATCGTGGTCACCGCCGATCACGGCATGAAGCCGAAGCACAAGGCGGACGGCTCACCCGACGTC
ATCTATGTTCAGGACCTGCTCGACGAATGGCTGGGCAAGGATGCGGCCCGCGTTATCCTGCCGATCACCGACC
CCTATGTCGTGCATCACGGCGCGCTCGGGTCCTTTGCCACCGCCTATCTTCCGGACGGATGCGACCGCAGCGA
GATCATGGCAAGGCTGAAAGCGATCCAAGGCGTCGACGTCGTGCTCGGCCGCGAGGAGGCGTGCCGGCGCTTC
GAGCTGCCGGAAGACCGCATCGGCGACATCGTCCTCGTCTCATCCGAAAACAAGACGCTCGGCACCAGCGAGC
ACCGGCACGACCTGGCCGCACTCGACGAACCGTTGCGCTCCCATGGCGGGCTGACCGAGCAGGAGGTTCCCTT
CATCGTCAACCGCGTGCTGCCCGAACTCCCGAACGCTCCCCGGCTGCGCAACTTCGATGCGTTTTTCTACGCG
GTCACGGCGGCGGCCGAGGCCGGTGCGGAGGGCGGGCTATGAATGACTCTCGTTGCCCTCTCGCTGCATCTCG
CCGGCGCGACCGTACTGCTGCTCTACGCGGTGCATCTCGTCCGCAGCGGCGTCGAGAACGCCTATGGCACGTC
GTTGAAGCGTGTGCTTGCAAGGGGAGGGGAGCGACGCATTTACGCCGCCGCGAGCGGCACGGTCCTTGCAGTC
CTGTTGCAAAGCTCGACCGCCGTGGCAATGCTCGCCTCCGGTCTTTCGGCGAGCGGCTATCTTACCCTGAACA
CCAGCCTTGCCGTCCTGCTGGGAGCGGATCTCGGCTCGGCGCTGGTGGTTCGTGTTCTTTCGTTCGACCTGGG
CTGGCTGTCGCCGCTCCTGCTTGCGGCCGGCGGGCTGCTTTACCTGAAGGGCAGAAGCGACAAGCCCCGCGAA
CTCGGCCGCATGCTGCTCGGCATCGGCTTCGTCCTCATGTCGCTGAAGCTGATCGGCGAGGCGACCTTGCCGT
TGAAGCAGAGCGACGTGTTGCCCGCCGTCGTCGGCTATCTCGCGGAGGACCAGGTGACGGCCTTCGCGGCGGC
GATGCTCTTCACCTGGATCGTGCATTCGAGCGTCGCGGTCCTGATCCTCATCCTGGGTTTTGCCGCACAGGGA
CTTTTGCCGGTCGAGGCAGGTCTGCCGCTGATCCTCGGCGCCAATCTCGGCAGCGGGTTGATCGCCATGTGGC
TGACGCGCGGGTTTTCAGCCGAGGCGCGGCGCGTCGCCGCCGGCAATCTCCTCTTCCGGGCTTTCGGCGCCGC
GATCGCCTTGCTGGTGATCGGCCCCTTGTCGGCAGAACTTCAACATCTTGGCGCCACCGCGGCGGAGCAGCTC
GTTCATTTTCATCTGGCCTTCAACGCCTGCCTCGTGATCTTTTGCCTGCCGCTCTCGCAGCTTGCCGCAAGCG
CCGTTACGTCGCTCATCAAATCGGATCTCAAGAGCTCCGAAGACAGCGACACGCTTGCGCGACGAATCAGCGC
GCTCGACCAGGCTCTGCTCGACAGCCCCGACGCGGCCCTCGCCGCCGCGACGCGCGAGGTGCTGTTCATGGGC
GAGGTGGTCGAGCGGATGCTCTGTCCGGTGATGGAGCTTCTGGAGCATCCGAGCCCGGCGAAGATCGAGGAAA
TCCGCCGGCTCGACGGATATGTCAACCGCGCCCACCGCGACATCAAGCTCTATCTCGCAGCAATCCATCGCGG
CGTGCTCGACGAGCGTCAGTCGCGGCGGGGTGTAGAACTCATCGACATCGCCATCAATCTCGAACATGCCGGC
GACATCGTATCCAAGACCCTGGTCGAAATCGCCCGCGACAAGCTGGAGAACGGCAACAACTTCTCCGACGAGG
GCTGGACCGAGCTCAAGGCGCTTCACGCCAGCGTGCGCGACAACATGCGTCTCGCATTCAACCTGCTGGTGTC
GGATGACCCGGTAATGGCGCGAAAGCTCGTCCGGGAGAAGGAGCGGGTACGCGCGCTCGTGCGGGAAAGTCAC
GAACGGCATCTGGCGAGATTGCGGGCCGGGCGCGCGGAAAGCGTCGAGTCCAGCAACATGCACCTCGAAGTCG
CGCGGGCACTCAAGGAGGTGAACTCCCTCCTGGCCACGCTCGCCTATCCGATCTTGAAGCAGCGAGGGGATCT
GCTCGAGAGCCGGCTTGCGCTTGTCAAAGGGTGAATGAACATGCCCCGCATTCTCAAAACTCTCGTCGCCGGC
ACCGCGCTCACATTGCTGGCGTCCGGCGCCTTCGCCGACGGCATCGGCGCATCGCTCCTGACCCAGCAGCACC
CGTTCTACATCGAGCTCGCCGAGGCGATGAAAGCCGAAGCCAAGGAGAAGAACGTCGCGCTCGAGGTGGCGAT
CGCCAATCAGGACCTCAACAAGCAGCTCGCCGACGTCGAGGACTTCATCGTCAAGGGCGTTGATGTGATCGTC
ATTTCGCCTGTCGACAGCCAGGGCGTACGCGCCGCCATCGCAAAGGCGGAAAAAGCCGGCATCAAGGTGATCA
CCGTCGACGTTCCCGCTAATGGCGCGACGGTGACGTCGCATATCGGCACCGATAATTTCACCGGCGGCGTGAA
GGCGGGCGAGCTCATGGCCGAGGTTCTCGGAAACAAGGGCAAGGTCGCCATTATCGACTATCCGACCGTTCAG
TCGGTGGTCAATCGCGTCAACGGCTTCAAGGAAGCGATCGCCAAGCATCCGGAAATGGAGATCGTCGCGACGC
AGACGGGCATCACCCGCGCCGAAGCCCTCGCTGCCGCACAGAACATGCTGCAGGCCAATCCCGACATTACCGG
CATTTTCGGTTTCGGCGACGATGCGGCCCTTGCCGCGGCCGTTGCCGTCAAGGCCGCCGGACTGGAATCGCAG
GTCAAGGTCATCGGTTTCGACGGCATGAAGGAAGCACGCGATGCCGTCGATGGCAATCCGGTGATGGTCGGCG
TGATCCAGCAATTCCCGGACCAGATGGGCAAGCAGGCGATCGACACCGCCGTAAAGGTCGTCGCCGGCGAGAC
GGTACCGGCCGAGCAGCCGATCGTACCGGGCGTCTATACCGGGAAATAGATGAGCCTGAATTCCGCATCGACT
GCCGCGACCGTCACCACCCCGGTCCATTCGTTGCTCGTGCCGTTTCCAGTCGTCTGTTTTACGCTCGCCCTTC
TGACTGATATCGCCTTCTGGCAGACGGGACATCTCATGTGGCAGAACTTCTCCGCCTGGCTGCTCTTTGCCGG
TATCATCGCCGGCGTGATCGCCGGTCTAGCCAGTGTGTTGGAGTTCCTGTTTCGAAGGGAGCGACGCAGGCAA

GGGGCGGTTTGGCTCCATGTCGTCGGCTACATCCTCGTCTTCGGCCTCGCCTTCGTCAACAACCTCGTCCATG
CCGCCGATGGCTGGACGGCTGTGGTGCCCTACGGGTTGATCCTCTCCGCGGCGACGGTGCTTCTGACGATCCT
CTTCGCTTTGCTTGGCCGCGCCACCCTCTATCGCACGCAATCCGGAGTGAGCGGTTATGAATAGATGTCCGGA
AATAAGGAAAACTCGGACCTGATCGTGGTGGCAATGCCGCTTTATGGCCACGCAGCCCTCGTCCTGGAGGCGA
TCGAGTCCGTGCTCGCTTCGAAGCTTGCCGGGTGCCGGGTGGCAGTGGTGGTGTCCGTCGATGGTGATCCGCG
ACAGGAGACATTCGATCAGTTGCTGCTCTATGCGGCGGCCCATCCTGCGGTTCATGTGCTTTTCGGGGCCAAT
GCCGGGCCGGGCGGGGCGCGCAATCGCGCGATCGACTACGTGCTCGCGAATCTGCCGGAAGCAGAGGCCGTCT
ATTTTCTCGATGCCGACAATCGCGTGCTGCCCGGGACCATCGAGACTCTCTACCGGCAACTGCGCGCCAGCGG
CTGCGGCTGGATCTATACCAATATCGACACGTTTTCGGTGAGCTGGCGCGCCCATTACGGCAATCGCTACTCG
CGGCTTCTGCACTGCATCACGGACAATATCTGCGACACCGGTTCGATGATCTCGATCGACGTTTTCCGGGCGG
GGGTCCGCTTCGACGACGACAGGCAGAACGGTTTCGAGGATTGGGAGTTCTGGCTGTCCTGCATCGAGCACGG
CTTCGTGGGGGCGCCGTGCCACGACACGACCTTCGAATACCGGCTGAGGGCGGAAAGCCGCTTCAAGGAGGCG
AACCGCGATCGCGCCGCCTCGGTGAGCTTCCTCAGAAAGCGCCACCGGGCGCTGTTCCAGCGGCCAATGCTGG
TCGATTTCGAGCACGAGGAATGCCCGCGCTATCTTTTCGCACGGACGGAAGATGCCGCCATTTCGTTCTTTAC
GGATCCGACCAAGACGCCCAGGCGTCTTCGCCTCGACGATATCATTCCAGCCTTCTGGGCGAGCATCGGCGAG
CCTGACAACGTTCACTTCCCGCCCTTCCTGATCGCCGGAAGCGGTGCCACGCTCGATCTGCTGCTGCGCTCCC
GGATGCTGCCGAACGTGCTCTCCCATCTGGAGCGCCTGAGCGAGAAGGCCAACGTGGTCTTCGTCCAGCTCGG
CAACGATGCGGCTCAGCGCAAGATCGAGCCGGTCTTCCTGGAGGCCGGCGCCCAGCACAACGCACCGGCCGAC
CTCATCTTCCTGTCCACTTCGCTCGTGCGCGACGTCATTCATAACAAGGCGCTGGACTGGTTTGCCTCCATCG
GCAACCAGCAGGTCTGGCCGACATCGGCAATTCTGAAAGTCCGCTTCCCCTTCCCGAGAAGCCTGCCGCGCCG
CTCGCTCATCACGCCCCAGCAGGTGATGATCAATTGCGTCAATGCCATCGCGACCAGTCCGCTGCGGCGGACC
GCCGGCAAACGCTGGACCTGGCGTCCGGCACGGCTCGTTCCCTATTCCGACCTCCACAAGGCGCTTCGCCACG
AGATCGGTGGCTCGCCCGTGTTGCCGCTCGGTCATAGCGAGGGAAGCAGGAAGACCGCAGCCCTGCTTGTTCC
GAATGCATCCTTCGGCGGTGCCGAGAAGGTCGTATATGCGGCCTCGCGCGAGCTGAAGGCCGCCGGCTACGAG
ACCCATCTCTTCGTGCTCGGCACATCCAGGATGGATGTGATCGACGAGTTCGATCAGAGCTTCGACTATATTC
ACTTCTGGGACCAAGGCATTCCCGCCTGGGGCGGCTCCGGCTCGTTCCTGGGGCAGGACTTCATCGCCGAGGG
CCACGACGTCGATTGGGCTGCGCTCAAGGGTCAGCTTTCCGGCTTCGACCTCGTGATCAACAATCACGTCATG
GCGGTGCATCCGCTTATCGCGCGGTTACGCTCGGAGGGAACGCGCACCGCCTGCTACCTTCACGTGGTCGACA
ATACCTCCTTCAAAAGGCCCGCCGGTCAGCCCTTCGCGGCGATTGCCCACGAGCATTGCTACGATGCCTTCCT
GACCTGCTCGGAGCAGCTCAAGATCTATCTGCACAGCTTCGGCGTTCCCTATGAAAAGATCTTCGCCGTCCCC
AACGGCGCGAGCTTCTCCGTGCCGCCCAAGGTTCTCGCGGAGGTGCTGTCGGTCAGGCGCATCGAACGCAAGG
ACGATCGGCTTCGGGTCCTCTATATGGGTCGGCTCGATCAGCAGAAGGGGATCGACCGTCTCGCTGCGGCGTT
CGCCGAATTGCGGGCTTCGCGCGTGCCATTCGATGCCCGGGCGATCGGCGGGGAAATCCTCGCGGATGCCACC
TTGTCGTGGACGGATCGCCTGAAGGACCTCGGCGTCGAGGTGCGCCCGCCGGTGTTCGCAAGCAAGGACCTGA
TCAAGGCCCTCGGTTGGGCGGATGTCCTTCTGATGCCGTCGCGCTGGGAGGGCGCTCCCTTGATGATCGCCGA
GGCGCAGCAGCTCGGCTGCGTCCCGATCGCGACGGCAGTCGGTGCCGTCGACGAACTCATAACCGACGGCGAG
GACGGCATCCTCATCGAGGCCGCCGCCGATCCCCAGGTGGTCCGGGACATGGCAAAGGCCATCGAGGAGGTGG
CCAACAACCGCCAGATGCTCGCGCCCCTCATGGAGGGGTGCCTGAGAACGGCGGCACGCCGCTCATGGACGTC
CTCCTTTTCCGAGTTCCTCGGCTGGTGCGACCGTTCCGTGAACAATTCATCGCTTTCGCGCGCAACGGTCATC
CGTGGGCGCGAAGCATCCAATCCCGGAGTTGCGGCCGTGGGCTGAATGGCGGGAGAACTGGAATTCATGAGCC
GGGAGGTCGTTTCGGGCCGCCTGTCGAGGCGCGATTTTCTCGGGCGCGCGGCAGCGCTCGGCGTAACGCTCGC
CTCGGCCCAGGTGCTGCTCGGCAATGCCGCGCGGGCGAGCGGGCCCGTCAAGGGCGGCATATTGCGGGCCGGG
CTCGTCGGCGGCGAATCCACGAACAGCCTCGATCCGGCGACCTGGGCGAGCCAGGTGCCCTATACATTCGGCC
GCTGCTGGGGAGAGCAGTTGCTCGAGGTATCTCCGGCGGGCGAGATAGAATATCGGCTGGCTGAATCCTACGA
AGCGTCGAAGGACGCGAAGACCTGGGCGTTCACGATCCGCAAGGGGGTGACCTTCCATAACGGCAAGGAACTG
ACGCCCGCCGATGTCGTCGCCACATTCGAGCGTCATGCCGACCAGAATTCGAAATCGGCTGCCCTGCCCTTCG
TCCAGGAATTCGAGACCATCAAGGCAGACGGCGACAAGGTCGTCATCACGCTGCGCCAGCCGAACGCCGACAT
GCCGTTCATCGTCAGCGATTATCATCTTATGATCCAGCCGAACGGCGGCAAGGATGACCCGACGGCCGGCATC
GGAACCGGCCCCTACAAAGTCGTCGTCAACGAACCGGGCGTAAAGCATGTCGGTGAGCGTCAGGACGGCTATT
GGGGAGCGGCCGAGCGCGGCCATGCCGATCAGATCGAAATCACTGTCATCAATGACGCAACGGCGCGCACCGC
CTCGCTCCAGGCGGGCGGCGTGCACATCATTAATCGCATCGAGCCGAAGATCGTCGCGCTCGTCAAACGCATT
CCCGGAGTCACGATCCGCAACGTCCCCGGCAAGGGCCACTATGTCCTGATCGCCCATTGCAACACGGCGCCCT
TCGACAACAACGACCTGCGGCTTGCGCTGAAATACGCGGTGGACCGCGAGGAAATGGTGGCGAAGATTCTCGC
CGGATACGGCACGGTCGGCAACGATACGCCGATGATCAAGGGTTACCCGCTCTTCGACGACGATATCGAGCAG
CGCGTCTTTGATCCGGACAGGGCGAAGTTTCACTTCAAGAAATCGGGCCATGACGGTTCGATCCTGCTGCGGA
CCTCCGACGTCGCCTTCCCAGGGGCAGTCGACGCCGCGCAGCTTTTCCAGGCAAGCGCCGCAAAGTGCGGCAT
CGACGTCGAGCTGAAGCGCGAGCCCGGTGACGGCTACTGGTCCGACGTCTGGAACAAGCAGCCCTTCAGCATG
TCTTACTGGACCGGCCGGCCGACGCAGGACCAGGTCTATTCCATCGCCTATCTTTCAAAGGCGGAATGGAACG

ACACCCGCTTCTTCCGCGAGGATTTCGACAAGCTGATCTTTGCGGCGCGCGGCGAGCTGGACGAGGCGAAGCG
CAAGGTTCTCTATCGCGAGGCGGCAATGATCCTGCGCGATGAGGGTGGTGTGATCGCGCCGATGTTCAACAAT
TACATCGATGCCACCAGCGACAAGGTCGGCGGCTGGCTCGACGATCCGAACGGCGAACTCATGGGTGGACATG
CGCTGACCAAGTGCTGGCTCGCCGCCTGAATGCTCCAGCAGATCAAAGGCCTGCATCACGTAACGTCGATGGC
GGACGACGCCCGCACGAACAACCGGTTCTTCACCGACACGCTCGCCCTGCGCCGCGTCAAGAAGACGGTGAAC
TTCGACTCGCCTGACGTCTACCATCTCTACTATGGCGACGCGACCGGCACCCCCGGCACCGTGATGACCTATT
TTCCCTTTCCGCAGATGCCGCGCGGTCGCGCGGGCACGGGAGAGGTGGGGACGACCGTCTTCTCCGTCCCGCG
GGGCTCGCTCGGCTTCTGGCAGGAGCGCTTGGCGGCACTCGGTGCGAGCGGTCTCAAGCAGGCGGAAAGCTTT
GGCGAGAAGCGCCTGAACTTTGCCGGTCCGGACGGCGACGGCTTCGCTCTCGTCGAGGTCGAGGACGACCCGC
GTACGCCCTGGACCGAGGGCGGCATCACCGAGGATCACGCCATCCGCGGCTTCCATTCGGTCGCCATGCGCCT
ACGCGACGAAGGCGCGACGGCGGAACTGCTGAAGTTCATGGGTTACGAGGAACTCGACCGCAAGGACGGCGTC
ACCAGGCTGATCGTGCCCGGCGGCAACGGCGCGAGCCTCGTCGACCTGGAGACGCTGCCAAACATCAGCCGCG
CTGCGCAGGGCGCCGGTTCGGTGCACCACGTCGCATTCGCCGTCGACAACCGCGAGAAGCAGCTGGAGGTGCG
CAAGGCGCTGATGGACACCGGCTACCATGTGACGCCGGTGATCGACCGGGATTATTTCTGGGCGATCTATTTC
CGAACGCCGGGCGGCGTTCTCTTCGAGATCGCCACGAACGAGCCCGGCTTCGACCGGGACGAGGACATCGCGC
ATCTGGGCGAGGCGCTGAAGCTGCCGCAACAGCACACGCATCTGCGCCCGGTTCTCGAAAGCCATTTGCAGAA
GCTGGAAGCCTAGATGGCCACTTCCGTGTCTACACGGCACCTCAGCGAGAACGGCGCTCTTATCGAGCGTCAC
TGGCAGGAGCTCAACAATCGCCGGCGTCTCTATACCTGGCTCGGTCTCGCATTTCTCGCGTTGACGCTTTTCA
GTTCGCTCTGGTTCGCGAACGAGTCGAACGCGGGCAAGTTCTTCGACCGGCTGCCGCACTTCTTCGATTTCGT
CGGACATCTCATGCCGCGCGATCCGATAGAGATCGTTCGCGCCCTCTTCGACCTGCCTTCGCCCTATGACGAC
GGCAGTTTCAAATACAATTACCCGGATGGGCGCCTCTATGTGACCGAGAGCTTCTACATCCCGGAATATGTTC
ACAAGATGCTGGAGACCGTGAACATCGCCATCTTTTCGACTATCATCGGAGTCTTCTTCGGCTTCATCCTGTG
CTTCCTCGCCGCGAAAAATCTGATGCCCAATCCCTGGATCAGGGGCATCGTACGCCGGCTGATGGAAATCCTG
CGCGCCTTTCCGGAAGTCGTCATCGCCGGCTTTTTCCTGGCAATCCTTTCGCTTGGGCCTATTCCCGCCATCG
CTGCGGTGTCGATCCACACGATCGGCGCGCTCGGCAAGCTGTTCTTCGAAGTGGTCGAGAATGCCGACATGAA
ACCGGAGGAAGGTCTGCGTGCCGTCGGCGCCAACTGGATCGAGCGGGTCTGGTTCGGCATGGTCCCCCAGGTG
CTGCCCAATTTCACCAGCTATTTTCTGCTCCGGCTCGAAATCAACGTGCGCGCCTCGACGATCATCGGCGCCG
TCGGCGGGGGCGGCATCGGGGAACTCCTGCGCCTGTCGATCGGCCAGGGCCATGAAGCAAAGACGCTCGCGAT
CGTCCTCCTGCTCTTCGCCACGATCTTCGCCGTCGATCAGTTCTCCGCCTGGCTTCGCCGCCGCCTCGTCGGC
GACCAGGCCTTTCAGCTTGCGCAATAGATGACCACCGCCGATCGAGTATCCGGAACGCTCATCGTCGTCGTCG
GTCCGAGCGGTGCCGGGAAAGACAGCGTCATGGGCTTTGCCGCCCGCCATTTCGCGCAGCGGCCCGATATTCT
CTTCGTCCGGCGGGCGATCACAAGACCGTCGGATGCAGGCGGCGAAGTGCACGAAAGCGTGTCCGACGCCGAA
TTCGAGGAGATGAGCCGGAGCGGCGCTTTCGCCGTTTCGTGGCAGGCGCATGGGTTGAGCTACGCCATCCCGG
CAGAGATCGCCGAAAAGATCGCCAAAGGCATGACCGCGATCGTAAACGGCAGCCGCGCCGCCCTCCCCGCCAT
TCGTCAGGCGTTCGGTACGATCGCCGTTGCGGTGATCACCGCCGAGCCGCCGGTGCTGGCGAAGCGGCTGGCG
GAGCGCGGCCGGGAGAGCGAGGAAGACGTGCTCCGCCGCCTGACGCGGCAGGCACCCGACGTCATCGCCGATG
CCGACGTAACGGTCATCGACAACAGCGGCAGGCTGGATGTGGCGGGCCGGCAATTCGTCGCGCTGGTCGAGCG
GCACTCAGCTAAGCGCCATCACCCTGCCTGAATGTCGATCAAGCGCTATGGTGCCGAACAGGTGGGCGCAGGC
GGAAAAACATTGCCTTTCGCACGTGCCGTGGAGGCGGGCGGCTGGCTTCACGTCTCCGGTCAGGTCGCGATGG
AAAACGGCGAGATCATCGGCGGCAACATCGTCGCCCAGACGCACAAGGCGATCGGCAATCTTCTGGCCATCTT
GAAAGAGGCCGGCTACGGTGTCGAGCACGTGGTGAGAGTTGGCGTCTGGCTGGACGATCCGCGCGATTTCTGG
AGCTTCAACCGGATCTATCAGGATTATTTCGGAGCTCATCCGCCGGCGCGCGCCTGCGTACAGTCCTCCATGA
TGGTCGATTGCAAGGTGGAGATCGATTGCGTCGCCTATAAGCCGAAGGACGCCTGAATGCGACTGAACTTCAC
CAGGACACTGATCGGTGCCGCCATTGCGCTTGCACTCACCACCGCGGCTGCCGAAGCCGAGGTCGTCGTTTCC
TCGAAGATCGACACGGAGGGCGGCGTTCTCGGCAACATCATCCTCTCGGTCCTGAACGCCAACGACATCAAGA
CGACGGATCGCGTGCAACTCGGCGCCACCCCTGTCGTGCGCAAGGCGATCATCGCCGGCGAGATCGACATCTA
TCCCGAATATACCGGCAACGCCGCCTTCTTCTTCGAGAAGGCCAACGATCCGGCCTGGAAGGATGCGGCGAAA
GCCTATGAAGAGGCGAAGAAGCTCGACTATGAAGCCAACAAGATCGTCTGGCTGACGCCGTCTCCGGCCAATA
ACACCTGGGCGATCGCGCTGCGCAAGGACATTGCCGAGAAGAACAACCTCAAGACGCTATCGGATTTCGGCAA
ATTCGTTTCTGGCGGCGGCGAGGTCGTGCTCGCGGCTTCGTCGGAATTCGTCAACTCCGCCGCTGCCTTGCCT
GCGTTCCAGACGACCTATTCCTTCAAGCTGAAACCGGAACAGCTCATCACGCTCTCCGGAGGCGATACCGCCG
CGACGATCGCGGCTGCGGCAAACCAGACGAATGGCGCCAATGCGGCAATGGTCTACGGCACCGATGGAGGCAT
TGCGCCCTCCGGTCTCGTGGTTCTCGAGGACGACAAGGCCGTCCAGCCGGTCTATCAGCCGGCGCCGATCATC
CGCGAAGCGGTTCTCAAGGAAAATCCGGCGATCGAAGAGATCCTGGAGCCCGTCTTCGAAAAGCTCGACCTCA
CCACCCTGCAGGAGCTCAATGGCCGCGTGCAGGTCGGCGGCGAGTCGGCCAAGGCCGTTGCCGAGGACTTTCT
GAAGGCAAACGGCTTCCTGAAATAGATGAGCGTTGCCAATTTCTTCGACGAAATGTCCTATGGCCCGGCCCCC
GAAGCCGATACCGAGGCGCGCGCCTGGCTCACTCGGCATGAAGGCAAGCTCGGCCACTTCATAAACGGCGCAT
TCGTCGGTTCAGCATCAGGCAAATCCTTCGACACGTTCGAGCCCGCAACGGGCAAATTGCTCGCGAAAATCGC

ACATGGCGGGCGCGACGACGTGAACGCGGCGGTGGCGGCAGCGCGCAAGGCGCAGGGCCCGTGGGCAAAGCTC
TCCGGACATGCCCGCGCCCGGCACCTCTATGCGCTCGCGCGCCTGATTCAGCGCCATGCGCGCCTGATCGCCG
TCGTCGAAGCGCTCGACAACGGCAAGCCGATCCGGGAAACGCGCGACATCGACATACCGCTTGCCGCTCGCCA
TTTCTATCATCATGCCGGCTGGGCACAGCTGCAGGAGACGGAATTCGCCGACCAGGTGCCCGTGGGCGTTGTC
GGACAGGTCATCCCCTGGAACTTCCCCTTCCTGATGCTCGCCTGGAAGGTTGCGCCGGCGCTGGCGCTCGGCA
ATTCCGTCATCCTGAAGCCCGCGGAGTTTACGCCCTTGACGGCGCTGCTCTTCGCGGAGCTTGCCGCTGCAGC
GGGGCTCCCCCCGGGCGTGCTGAACGTGGTGACGGGCGAGGGCGAGACGGGGGCGCTGATCGTCGAGCACGAG
GATATCGACAAGATCGCCTTTACCGGCTCGACCGAAGTTGGACGCCTCATCCGCGAGAAGACAGCGGGGAGCG
GCAAGTCGCTGACGCTGGAGCTTGGCGGGAAGTCGCCCTTCATCGTCTTTGACGATGCCGATATCGACGCGGC
GGTCGAAGGCGTCGTGGATGCGATCTGGTTCAATCAGGGCCAGGTCTGCTGCGCCGGTTCACGCCTTCTGGTG
CAGGAGGGCGTGGCGCCGGTCTTTCATGACCGGTTGAAGCGGCGCATGGAGACGCTCCGCGTCGGTCATCCGC
TCGACAAGGCGATCGATATGGCGGCGATCGTCGCGCCGGTGCAGTTGCAGCGAATCGAAGAGCTGGTGGCGAA
GGGCGTCGCCGAAGGCGCATCGATGCATCAGCCGAAGATCGAACTCCCGAAGGGCGGCAGCTTCTTTCGGCCG
ACGCTGTTGACCGGCGTCCAGCCGACTTCCGTCGTCGCGACCGAGGAAATCTTCGGGCCGGTGGCCGTATCGA
TGACCTTCCGTACGCCGGAGGAGGCGATCCAGCTTGCAAATCATAGCCGCTACGGCCTTGCCGCGAGCGTCTG
GAGCGAAACGATCGGTCTTGCCCTGCATGTTGCCGCAAAGCTTGCGGCCGGTGTCGTCTGGGTCAATGCGACC
AACCTCTTCGATGCGTCGAGCGGCTTCGGCGGAAAGCGCGAATCCGGCTTCGGCCGTGAGGGCGGGCGAGAGG
GCTGCTACGAATATCTGAAGCCGAAGGCCTGGATGGGGCGCAAGCTGCGCCCCGAACACGGCTTGCCGGCCCC
GAAGCAGGCCGCCGGTGATTTCGCCGTTCCGGCGCTCGATCGCACTGCCAAGCTCTTCGTCGGCGGCAAGCAG
GCGCGGCCGGACGGGAACTATTCGCGGCCCGTGCTTTCCCCGAAAGGCAAGGTGATCGGCGAAGTCGGCGAGG
GCAACCGCAAGGATATCCGCAATGCCGTCGTTGCGGCGCGGGGCGCCTCGGGCTGGTCTTCGGCAACAGCGCA
TAACCGCGCCCAGATCCTCTATTACATCGCCGAGAATCTGAGCAGCCGCGGGGCGGAATTCGCCGACCGCATC
GCCGCCATGACGGGGGCCTCGGCAGCCAATGCCCGAGCGGAGGTCGAAGCTTCGATTGCCCGGCTGTTCAGCT
ACGGAGCCTGGGCGGACAAATACGAGGGCACCGTGCACCAGCCGCCCCTGCGCGGCGTGGCACTGGCCATGCC
GGAACCCCAGGGCGTCGTCGGCGTCATCTGCCCCCCGGAAGCGCCGCTGCTCGGGCTTGTCAGTCTCGTGGCA
CCGCTGATTGCAGTCGGAAACCGGGTGGTCGCCGTGCCGAGCGAAGCGCATCCTCTGGCTGCGACCGACTTCT
ATTCGGTCCTCGAGACCTCGGATGTGCCGCCCGGCGTCATCAATATCGTCACCGGATCTGCGATCGAACTCGC
CAAGGCACTCGCCGCGCACAACGACGTGGACGCGCTCTGGGCGTTCGGCTCGCCGGAACTTTCGACACTCGTC
GAGAAGCTGTCCGTCGGCAACCTCAAGCGTACCTTCGTGGATTACGGCAAGGCGATCGATTGGACGGATCGCT
CGGCCGCCGAAGGACAGGCGTTCCTGCGGCGAGCGGTCGATGTCAAGAACATCTGGATTCCCTATGGCGAATA
GATGACGATGGCATCGATCTGGCAAACGCTTGCCAACAGGCTCGATCGCCTGCAGGAGACAGGCGGATCGATC
GACTTCTGGCTGCGGGACGACGATGCCGTCGAGCCGACGGCGGCCCTTCACCGACTGCTCGACCTTACCGACC
GCTTTTCGGTGCCGGTGAGCTTGGCCGTCATACCGGCACATACCGACGAGCGCCTCTCGCGCTGCCTCGCCGG
CCGTCCGCATGTCGATGTTGCTGTCCACGGGTGGTCCCATCAAAACCATGCCCCTGTGACTGAAAAACGCCAG
GAACTGGGAGGACACAGGTCGCGCGAGATGGTTGCGGCCGACTTGCGCGCCGGTTGGGCACGCCTGCGCGCGC
TTTATCCCACCGCTATGGTGCCGCTCCTCGTTCCGCCATGGAACCGGATCGATCCGGGGCTGGTCGCCGGATT
GCACGAGATCGGGTTTAGGGCCCTTTCGGTTTTCGGACCGGAGGAGGGGGCAAAATCCGCCGCATTACGCGTT
CCCGGGCTGCAGCTCATAAATGCCCATGTGGATGTGATGGACTGGCACGGCACCCGCGGCTGCCGCCCTTATG
GCGAGATCCTTCGCGACATCGATAGCCGACTGGATGAAATCGAGAAGGGCGGCGGGACGGTGGGAATTCTCAC
CCACCACCTCGTTCACGACGAGAGCGTATGGGAATTCCTCTCCGGACTTTTCGACGTCACCGCCTCGCACGCG
GCCTGCCGCTGGCGCAGGGTCGCCGATCTTATAGATCGATGAATGGGACGCTGGCCGGTTTACGACGAGGAGC
AGATCGAGGATGTCGTGTCGGTCCTCAGATCTGGCGAGGTGAATGCCTGGACGGGACCGCACGTACGCAACTT
CGAGGCTGCCTATGAGCGCTTTCTCGGCGTAAAGCATGCGGTGGCTCTCGCCAACGGAACGGTGGCGTTGGAC
CTCGCGCTCTTCGCGCTCGGACTTGAGCCGGGCGATGAGGTCATCGTGACGCCGCGCAGTTTCATTGCCTCGG
CCTCCACGGTGCCGATGGCGGGTGGAGTTGCCGTCTTCGCCGACGTCGATCGCGACAGCCAGAATATCACCGC
GGAAACCATAAGGGCGAAGCTGACGCCGAGGACCAAGGGCGTCATCGTCGTGCATCTGGCCGGCTGGCCCTGC
GATATGCCCGCAATCATGGCCCTTGCCCGCGAAAAGGGGCTGTGGGTGATAGAGGATTGCGCGCAGGCGCACG
GCGCCGAAATCGACGGACGGCCGGTCGGCAGTTTCGGTGACATCGCGGCATTCTCCTTCTGCCAGGACAAGAT
CATCACCACCGGAGGCGAAGGCGGGCTCGTGGCAATGAACGACGACGAGCTCTGGAAGAAGGCGTGGAGCCGC
AAGGACCATGGCAAGTCGTATGACGCCGTCTACAACAAAGAGCACCCTCCGGGCTTCCGCTGGCTGCATGAGT
CCATCGGTACCAACTGGCGCATGATGTCGATCCAGGCGGTCATGGGATCGCGTCAGTTACAGCGCCTGACGGA
ATGGCGCAGCATAAGAGCGCACAATGCGGCTATTATCGCCAGAGCGGCCGAGGAGATCGAGGCTTTGCGCACG
CCCTTGCCGCCCCGTGGGATTCAGCACGCCTGGTACAGGTTCTATACGTTTCTTCGGCCGGAGCGCCTGCGGC
CCGACTGGAGCCGGGACCGGATCATTGCCGAAATCAACGGCGCCGGCGTCGCATGCTTCAGCGGCAGTTGCTC
GGAAATCTACCTGGAAAAGGCCTTTGCGGACATCGGCATGGCTCCGGCGCAGAGATTGCCCGACGCGCGCGAG
CTCGGCGAGACGAGTCTTGCCTTCCTGGTCGATCCCGCGCTCGATACGGCGGCAATGGAGAAGGCGGCACATG
TCCTGCGGGAGGTGTTTGCAAGGGCCAGCATCGGCAGGCCAGCCCTCGATGCCGAGCGGCGTTAGATGATTCT
GAAGGGAACCAATCTTTCTGACACGATCCTCGGCACTTTGAGTGACGATGAGCTCTGGGGCTATGCGGGGGAT

GATTATCTCGAGGGCCGCGAAGGCAATGACAAGCTTTTCGGCGGCGATGGTAACGACTACATCGTCGGCGGGC
TCGGAGACGACTATGCCGAAGGCGGCGATGGTAACGATCGGTTCGACGGCGGCCTTGGGCACGACACCTTCAA
GGGCGGATTAGGAAACGACATCTTCGACGGCGGAGACGGCAGCGACGTGCTGTACGGCGGCGATGGCCATGAC
CATTTCGTGGGTGGTCGCGGACACGACAAGATCTATGGTGGCGCGGGCGAAGAATACATCGATGCAGGCGACG
GCGACGACATCATCTATGCCGGCGCGGGCGACGACGGCTTCAACAATCGCATAGATCCGGCAACCGGACAGCT
GACGCAGCAGGCCGTCGGCGGGGGCGCCGGCGACGATAGGATCTACGGCGAAGAGGGCAATGACGCGCTGAAG
GGCCAATCCGGCAATGATTGGGTTTATGGCGGCATCGGCGACGACATCGTCGATGGCGGCGACGGGAACAACT
TTCTCGATGGGGGTGACGGCAATGATGTGCTGGACTCGGAGGGCGGCGCGGACGAGGCACATGGCGGCAGCGG
CAATGATCGGATCGCCGTGGGCGCCGGAAACGATCTGGTCTTCGGGGATGACGGTGACGACGTCCTGACCGGT
GAAGGCGGCGACGATGAGCTGAGCGGCGGCCACGACGACGACCGAATCTTTGGCGGCGACGGCAACGACACCC
TGCGTGGCGACGCGGGCCAGGACGTTCTGGTGGGCGAGTCGGGGGCCGACATTCTCTGGGGCGGAACAGAATC
CGATCGTTTCGTCTTCAAAGGCGCCGCAGTGCTCGGCAGTCAGGATACCGTCATGGATTTCCAGGATGGCGTC
GACTTTCTCCTCATCGAAAAGCTCGGGGTCAGCCAATATTCCAGTTCCGGCGCCTCGGGCACGGTCTACGCCT
ATGACACTACAGACGGCGATGTGCAGGTAATGGGCTACGACTCGGCCGGAAACGCCTTCTCCATCCTGGTGGA
CGATCCCAACGGCGCGCTCGCGGCTGCGAACTTTTCGAGGAGTGATTTCCTTTTCGCCTGAATGAGGAAACAC
ATTCCGCCCGCATCGCGACCCGATCCGGAACCGACGTCGCCGGTCGAGCCGATAACGCTCTGGCGCGCCTTCG
TTCCGCGCTGGGCACATATGCCGCTATCCGGCGCGGGTGCCGCTCGCTTCGGTGGCCGCTGGAACCCGATCGG
GATGCCTGCGATTTATGCTGCGCGCGAGCTTTCCACTGCCTGGGCTGAATATAATCAGGGTTTCGTGCAGCAT
CCGGCGCTCATCGTGCAGCTCGAGTTGCGCGGGGCGAGGCTCGCGGACCTCACGGATGCCAGCGTTCTTCTGG
AGCTTGGTGTGGATGAGGCGATCCATCGCTGCGAATGGCGTGATGCGCTCGACAAAGGCGCGGTGCCGGAGAC
CCACAGGCTGCAGTCGGAGCTTCTGGCGCGCGACTATCACGGGGTGATCTATCCATCCTTCATGTCGCCCGGC
GGCACCTGCGTCGCGCTCTGGCGCTGGAACGGTGCGGAAGAACCGCGTCTCGACGTCATCGATCCCGATGGCC
GGCTGCCGAAATCGCCCGCCTCATGGGTTTGAATGTTTTCGAAGCTCCTGATTGCCAATCGCGGTGAAATCGC
CTGCCGCATCATCCGCACCGCGCGACGGCTCGGCATCCGCACCGTCGCCGTCTATTCCGACGCTGACGGAGAC
GCGCTGCACGTGGCGCTCGCCGACGAGGCGATCCGGATCGGGGGGGCGCCCGCCGCCGAAAGCTATCTGGCGA
GCGCTCCTATCGTTCAAGCGGCAAGAAGCGTCGGCGCACAAGCCATTCATCCGGGCTATGGTTTCCTGTCGGA
GAACGCCGATTTCGCGGAGGCTGTCGCAGAAGCAGGCATGATATTCGTCGGGCCGCCGCCGGCCGCAATCCGC
GCCATGGGTCTCAAGGACGCGGCCAAGGCTCTCATGGAACGATCCGGCGTACCGGTCGTGCCCGGCTACCACG
GCGAAGAGCAGGATGCATCCTTTCTTGCCGACAGGGCACGGGAAATCGGCTATCCGGTGCTGATCAAGGCGCG
GGCGGGTGGCGGCGGCAAAGGCATGCGCCGGGTCGAGCGGCAGGAGGATTTCGGCCCCGCGCTGGAGGCCGCA
CGGCGGGAGGCCGAATCCGCCTTCGGCGACGGTTCGGTGCTTCTCGAACGCTATCTGACGAAGCCGCGTCACA
TCGAGATGCAGGTCTTCGGCGACCGGCACGGCAATATCGTCCACCTCTTCGAGCGCGACTGCTCGTTGCAACG
CCGGCATCAGAAGGTGATAGAGGAGGCGCCGGCGCCCGGCATGACGGCCGAGGTCCGCCGTGCGATGGGGGAT
GCGGCGGTCAGGGCCGCCCAGGCGATCGGCTATGTCGGTGCAGGCACCGTGGAGTTCATCGCCGACGTGACCA
ACGGCCTCTGGCCGGATCACTTCTATTTCATGGAGATGAACACGCGGCTGCAGGTGGAGCATCCCGTCACGGA
AGCGATCACCGGCATCGATCTCGTGGAATGGCAGTTGCGGGTCGCTAGCGGCGAGCCGCTGCCGAAGAAACAG
GCCGATATTTCAATGAACGGCTGGGCCTTCGAGGCGCGCCTCTATGCGGAGGATCCGGCCCGGGGCTTCCTGC
CGGCGACCGGCCGGCTGACGGAATTGAGCTTTCCCGAGGGGACTTCGCGGGTCGATTCCGGCGTCCGGCAGGG
GGATACGATCACGCCCTATTACGATCCGCTGATTGCCAAGTTGATCGTACATGGCCAGAACCGTTCGGCTGCG
CTCGGCCGGTTGCAGGATGCTCTCAAGGAATGCCGCATAGGCGGCACGGTCACGAACCGCGACTTTCTCATCC
GCCTGACGGAAGAGCATGATTTCCGCTCTGGCCACCCGGATACGGGGCTGATCGACCGGGAGATCGAGCGACT
GACCGCTCCGGTCGCACCTGGTGACGAGGCGCTGGCGCTGGCGGCGATAGTTTCGACCGGCGCGCTCGACCCG
AACAGGTCGACCGATCCATGGTCGTCGCTCGGCTCCTGGCAGATCTGGGGCGACGCGCACCGCATGGTGGTGA
TCGAACATGCGGACGTGCGCGCAACCGTCACCTTGGCTTCGCGCGGCCGTGACCAGTTCGCCGTGCGTGCCGG
TGCGAGCACGCTGCCCGTCCTCGTGCTCGACCGCTTCGAGGGCGGTGCCCGGCTCGAGGTGGCCGGGCAGAAG
CGCCTGATCCGTTTCTCACGGGATCGCGAGGCGTTGACGCTCTTCCATGGCGGGCGCAACCTGGTGTTCCATG
TGCCGGACGGATTGACCGGCGGGCAGAGCAGCGAGATCGCCGACGACGAGTTGGTTGCGCCGATGCCGGGTCT
CGTGAAGCTGGTGCGGGTCGGCGCCGGCGACGCAGTGACAAAGGGACAGGCGCTTGTTGTGATGGAGGCGATG
AAGATGGAACTGACGCTCTCGGCCTCACGCGAGGGCACGATCGCCAATGTGCATGTGGCGGAAGGCGCTCAGG
TCAGCGAGGGCACGGTGCTCGTGACGCTGATGGAGGAGGCTGCGCAATGATTGATCATCGACACGCATCTGCA
TCTGATCGACAAATCCGCGCTCAACTATCCGTGGCTCGCCGGCGTTCCCGCGCTCGATCGCGATTTTCTCTAT
GCCACCTATGCGGCGGAGGCAAAGCGCGTCGGCGTTGCCGCCTCGCTGCATATGGAAGTGGACGTCGACCCGG
CTGAGATCGAACTCGAAACGCGCGAGGTCGCGCGTCTTGCGGGTGAGCCCGGAAGCCTCCTCAAAGGCGCGAT
CGCGGCTTGCCGCCCGGAAGATGAAGGCTTCGCCGCCTATCTCGAGCGGCAGGAGGAGAACGCTTTCGTCAAA
GGTTTCAGGCGGGTGCTGCACGTCGTTGCCGACGACCTCTCGGAGCAGCCTCTTTTCCGCGAAAACGTCAAGC
GGCTCTCGGGCACCCGCTTCACCTTCGATCTTTGCGTCCTGCCGCATCAGATTCCGAAAGCTATTGCCCTCGC
CGACCTTGCGCCCGACGTTCAGTTCATTCTCGATCATTGCGGCGTTCCGGACATAAAGGGTCACGCGGAGCAC
CCCTGGCGCGACCATATGACCGAGATCGCCCGCCGCCCGAATGTCGTCGCCAAGATCTCCGGCGTGGTCGCCT

ATGCGGAGGAAGACTGGGCGCTCGATTCCATCCGCCCCTATGTCGAGCACACGATCTCGGTTTTCGGATGGGA
CCGGGTGGTCTGGGGCAGCGACTGGCCGGTCTGCACCCTCGGCGGCAACCTCTCGACCTGGGTCGCGGCCACC
CAGGCCCTCATCGAAGGCTGCAGTCCCCAGGAACGGAGGAAGCTTCTCTCCGGCAATGCGCAACGGATCTGGA
ATCTGATCTGAATGAACAAGCACATCGCAGCGCTCACGGTCGCATGCCTCGCCGCAACCTCCGCCAATGCCGC
CGGCCTGCCCGAGCGGATCAAGTCGGCCGGTAAGGTCGTCGTCGCCAACCAGCCGAACTATCCGCCGATGGAA
TATGTCGATCCGGCGACCAACGAACTCAGGGGTGTCGACATCGATCTCGGCGTTGCGCTTGCCAAGGAGCTCG
GCACCACGGTCGAATGGTCGAATATCGGCTTCGAACAGATGATCTCGTCGCTTCAGACAGGCCGCGTCGACCT
TATCCATAGCGGCATGAGCGATCTTCCCAAGCGCCGCGATACGCTCGATTTCATCGATTACATGAAATCCGGC
GCGCAGTTCTACACGTCCTTTGCCCGCAAGGACGAGTTCAAGACGCTCACCGATTTCTGCGGCAAGATAGTCG
GCATGAGCCGCCGCACCTCCTTCCCGGACGAGACGGCGAAATGGAGTACTGCCAATTGCGAAGCCAAGGGGCT
GCCGGCCGTCAAGGTCGTCGGCACGGAAGGCTCCGCCGATGCCCGCGCCCAGCTGAAGCAGGGCCGGCTCGAC
GCCGCCGTCCAGGGTTCCGAAACGCTGCCCTATCTGATGGATCTCGAGAAGGACACCTACGCCGTCGTGGGCG
AGCCCTTCACCGTGGTCTATCAGGGCATCGGTTTCGCCAAGAAGGACACGGAACTGCGCGACGCCTATGCCGC
AGCGCTGAAGGCCCTGATGGAATCAGGCGAATACCAGGCGATTTTCGCCAAGTACGGCCTCGAGGGCACCTTG
CTCGATGGCATTTACGTCAATGGCGAAGCGGTGAAGTAAATGAGCTTAAGTTATTCAGAAAGATTAATTCTCG
CCAATCAATACGCGATTCTTGAGAATCTGGACGTAGGCAACGCACTCCTCCATCGGAGCGCGCGTGAGATCGT
CGAGCGCGGTTATGAATACCTTTATGATACGCTCAATCCGTCGGTGGAAGCAGAAGTCGTTCCCACCGCAATC
GGTCAGGAGGTGTATGATATTCTCGACATGTTCCGCGCGATCGAACGGTCGATGATAGACATCGGCAGGGCCC
CTTCCGAGCTCGAGGCGAATTTTGAAGGCTTTGACCCGAACAACGCCCGCGCTCATTTCCACTTCTGTGAGTT
CGTGCGGCGCTCCCTCGATTATTGGCAGGATCTCGACAAATACCCCGACACCTCGCACGACGCCTCGTCGCTC
ATGCGCTATCGCGCTATGCTGCTGAGATGGAAGTCTCTCGGTGCTCCATCGGCCCTTTCAGAGGCCCAGATCG
CCGAACTCGCGGGTACAGCCAACGCCTTGTCGGGGACCGCACATAAGGTGCACACACTGGTGCCGAAGACAGC
TATGCGGCTGTAGGTGGGCAGATCCGCGAACGAAAAACGAAATGGCGTCCCGCTCATTCATGGGGCCAGCGAT
CTGCCGTCGGTGACGGTTGACGATTATAATCTGGAGCTGCGTGACGGCGACGGCTTCCTTGGCGACAGGGCCA
ACAAATTTGCCTTCCAGGAGAAGCTCGACGCGTGGCGCAAACGCGTGCGCAAAGGCGGCGACGATCCGCTGGG
GCAGGCTTTGACCCAGGATCTGTCCAAAAAGCAAGTCGATGCCCTTCTGCGCGGCGACGACAAGGAGGCGGCC
GCCCTGATCATCGGTGCGGTCGACGATTTCGCCGGCGAACTCGCCAGCGTTCTCGAACGGTTTCTGCAACAAA
AGAACTGGAAGAATACGGAACGCGTGGTAATCGGCGGAGGCTTCCGGGGAAGCGCAGTAGGTGAATTGGCCAT
TGCCCGGGCAATGGTGCTACTGAAGGCTGAAGGCATCAAGATCGAACTCTCCCCTATCGTTCACCATCCGGAT
GATGCGGGACTTATCGGTGCCGCGCATCTCATGCCCGCCTGGATGCTGAAGGGACACAAGGCAATCCTCGCCA
TCGACATCGGCGGCACCAATATCCGCGTGGGCATAGTCGAGCTGCACCTGAAGGACGAAACCGATCTCTCCAA
GGCCAAGGTCTGGAAATCGGATATCTGGCGACATGCGGATGACAAGCCCAACAGAAGCACGACCATCGAGGGG
CTCATCGGCATGATCGAGAAGCTTATAGCCAAGGCGGACAAGGCGGACCTCGCGCCGGCGCCGGTCATCGGCG
TGGCCTGCCCCGGTGTGATCAACGAGGATGGCTCGATCCTTCGCGGCGGCCAGAACCTGCCGGGCGGCAACTG
GGAAAGCGAGCATTTCAACCTGCCCGCCGCCCTTAAGGACGCCATTCCGCAAATCGGCGATCACGAGACCTTC
GTGATCATGCACAACGACGCCGTCGTGCAAGGTCTGTCGCAGATACCCTTCGTGCAGAATGCATCGAGTTGGG
GGATCCTGACGATCGGGACTGGCCTCGGCAACGCGCATTTCAGCAACAAGGCCGAAAACTGAATGGGACAAGC
CGTACGAATCCCCACGAACGAGCGTCAAAGGTTGCTCGCAGTCCGTTCGCTCAATTCAATCGGCAGCGCTCCC
ACGCCCGAACTCGCGACCTTGGCCGAGCTTGCCAAAGGCATCTTCGACGTCTCCTACGCCGCCATCAACATCA
TCGACGAAGACTGGCAGCGGATCGGCGGTCAGGCGGGCTTAAGGATCGGCGAATGCTCGCGCGAGATGTCCAT
ATGCACGCGGGTCGTCTTCGCAGACGAACTCCTCATTGTCCCGGACCTGGCGGAAGACCGTGAGCTGAAGACG
AGACCATATGTGGTCGGAAGCCCGCACTTCCGCTTTTACGCCGGCGCGCCCGTCTATCTCGAGAACCTGCCCG
TCGGCAGCTTCTGCATCCTCGATGTCCGGCCCAGACATCTTTCCGACGGGCAGGCGCAGAACCTGCGCCGATT
TGCGGCTGTCGCCAGTGCGCTTTTGAGCTTGCAGAAGGCAAATCTCGTGATGGGGCTCGCCCATAACCAACTA
CAGAATGCGGCCATTACCGATCCGTTGACCGGCCTGTTCAACCGCTCGGCCCTGTCGTCGATCGTCGACGGGG
CTCTCCACAACGCCATCGCCGCGGGTCAGACCTTCGGCGCTCTTTATCTCGACATGGATGGTTTCAAGGCGAT
CAACGACGAGCTTGGCCATCACGCCGGCGACGAGGTTTTGTACGAAGCCGCCAACCGAATTCGCTCGGTCATC
AGAGCAGGCGATATCGCGATCCGCATGGGCGGCGACGAATTCGCCGTTTTCATTCCAAATCCGCCCAACGCAC
CGGCCCTGGCAGCGGTCTCGCAGCGGCTGGTAGCGGCCTTTCGCGAACCCTTCCAGGTCGACGGCAAGGCGGT
CCTTGCACGCATCAGCATCGGCGGCGCCCTTGCCCCGCTCAACGGGCAGCGACGAGCGGAGCTGCTGAACAGC
TGCGACAAGGCTCTCTACTCGGCAAAGCGTGACGGCCGCGACCGGTTCGTCATCCTCGACGCCTGAATGAGTT
CCTGCCAGTCCAATCTTCCTTCGAACCTCACTGTCAGCCTTCGCGAACTGCGGATGGTCTTCGAGCGGTTGAT
ACAGGTCACGCGGCTCGACGCGGGCCTCGTGCCCTCGCTCAGGGATTGCGCCCTTTATTCCGCAGCGCTCGGC
CTCGGCGGCTTCGCCCGCCTGGCTCACAACCTCGAGGCGGTGCGATCGGCAAATCCGCATGCGCTGAGGCTGA
CAGCGGACGCCGAGCGCCTAACTGTCGACTGCGGCGGGCAGCACGCGTGGATCGCCGCCGATGCGGTGCTCGA
TCTCGCAGCGGAGCGTCTGCGCACGCAGGGTTCGGGGAGGGTCGAGGTCCGTAGCGCCACCGAAATCGGCGAA
CTGAGGGTTGTCGAGGCTCTTGCGCAGCGTTTTCGACTGCAAGCTGAGGTCGCCATCGACGAGACAGCCGGCT
CTGCCGAGATCGTCGTCTCGCCCGCCCGGAAGGGAATGGATGTGACGGTTCTCGAAAGAATCCGGCGCAACGG

GCTGACCGTCGATGCGGCTCTCTGGTGGCAGCTGTTCCACCGGTCCGCCGAAGCGCTCGCGCCGGATTCCTAC
GTGTCGCGCCGCCACGCCGGTCCGATCATCGTCGAGGCCGACGGCAAGGTCATCGGCCGGCAGGACGAGGACG
AGACGGACCTCTCACTGCTGATCGCCGGCGCCGCACAGCACGGCACGACCGCAATCAATAACTGAATGAATCC
GGAAACCACTGCAGTCCCCTCTGAGCGCCTCGGCATACTGATCGATGGCCGCTGGATATACGAGGCAGAGCGG
AGCACCGAGGTGGTGGATCCCGCGACGGGCCAGACGATTGCCCGGCTGCCTTTTGCGGCCGATGGCGAGATTG
CCGAAGCCGTGGCCTCGTCGCAGCGCGCTTTCGAGAGCTGGAAGGATCGCTCGCCGCTCGAGCGGGGCCGGAT
CCTGCGCCGCTTCGCAGACCTCGCGCGCAAGCATGCCGACGAGATCGCCCGTAACATGACGCGCGACCAGGGC
AAGCCGCTCAGCGAGGCGATCGGCGAGATCCGTTTCGCCGCCGACCATGCCGATTGGCATGCGGAGGAAGCGC
GCCGCATCTACGGGCGGGTCATTCCCGCCCGCGATCCGCGCGTGCAGCAGATGGTCCTTCGCGAGCCGGTCGG
GGTCTGCATCGCCTTCACGCCTTGGAATTTCCCCTTCAGCCAGGCGCTGCGCAAGGTCGTGGCGGCGCTTGCG
AGCGGCTGCACCATCATCCTCAAGGGACCGGGGGAATCCCCCTCCTCCACGGTCGCGATCGGCCGGCTGATGC
AAGAGGCCGGGCTGCCGGACGGCTGTCTCAACATCCTCTGGGGCGACCCGGCCCACCTGTCGGAGACGCTGCT
GGCGGCGCCGGAAGTCAGAAAAATCTCCTTCACCGGTTCGGTCGAGGTCGGGAAGCATCTCGCGAGCCTTGCA
GGCAGGCACATGAAACGGTCCACGATGGAGCTCGGGGGGCATGCACCGGTGATCCTCTTCGACGACGCCGACA
TCGAGGCGGCAGCCGATGCGCTTGCCGGCCAGAAGGTGCGTAATGCCGGCCAGGTCTGCATCTCTCCGACGCG
GTTTTACGTGCAGGCGAAAGGGCACGACCGTTTCCTGGCACGCTTTGCCGAGAAGATCGCATCGACAAGGGTC
GGCAACGGTTTCCAGGAGAGCGTACAGATGGGCCCGCTCTGCCACGGCCGACGCGTCGCTGCCATGGAAGGTT
TCGTCCAGGACGCGCGCGAGCAGGGTGCCGAGATCGTCACCGGCGGCGAACGCGTCGGGAACGCGGGCTTTTT
CTATGCGCCAACCGTGGTCGCCGCCGGCACCGACGAACTGCGCCTGATGAAGGAGGAGCCCTTCGGACCGATC
GCCGTCGTCACGCCCTTCGGCGATTTCGACGAGGTGATCCGGCGCGCCAACAGTCTGCCCTTCGGTCTGGCGT
CCTATGTGTTCACAGGCTCGAGCAGCCGCGCACAGAATGCCGCACGCGCTCTTGCAGCCGGAATGGTGAGCGT
CAACCATTTCGGCCTGGCGTTGCCGGAGACTCCCTTCGGCGGAATCAACGACAGCGGCTACGGGAGCGAGGGA
GGCTCCGAGACATTCGACGGGTATCTGAACACGAAGTTCGTCACGCGTTTCGACCAAATCCTGCAGTGATTGC
TCTGCACCGCTGTGGCATGGTCGCTGTTCCAGCTCTGGTACGCGTCTCCCCTACCCTTCGTCTTCGGCTTCGG
CATCCTCAACGATACCGAAGCTCGCGCCATCCATCTCGGTTTCGCGCTGTTCCTGATTTTTCTTGCCTATCCC
GCGCTGAAAAGTTCGCCCCGCGACCGGGTGCCGCTCGCCGATTGGGTGCTCGCGGCGCTCGGCGCCTTCGCGG
GCGCCTATCTGTTCCTCTTTTATGGCGAGCTTGCCGGACGGCCCGGCCAGCCCTCGATGCTCGATCTCGTCAC
CGGCACCGCTGGCATCCTGCTCCTGCTCGAGGCCACGCGCCGCGCGCTCGGTCTGCCGATGGTGTTCGTCGCC
GGCGTCTTCATCTTCTATACCTTCGCGGGGCAGTACATGCCCGACGTCATCCAGCATCGCGGCGCTTCGCTGG
TGAAGTTCATCAACCATCAATGGCTGACGACGGAGGGTGTTTTCGGCATTGCGCTCGGCGTGTCGACGAGTTT
CGTTTTCCTCTTCGTCCTGTTCGGAACGCTGCTCGAGAAGGCCGGTGCCGGCAACTGGATGATGCAGATCTCC
ATCGCGCTGCTCGGACATCTGCGCGGCGGCCCGGCCAAGGTGGCGGTCGTCTCGTCGGCCTTGAACGGCGTCG
TTTCCGGCTCGTCGGTCTCCAACGTCGTGTCGGGCGGCATCTTCACCATTCCGCTGATGAAACGCACCGGGCT
TTCCGGCGTGAAGGCCGGCGCGATCGAAGCCACGGCCTCGATCAACGGCCAGATCATGCCGCCCGTCATGGGC
GCCGCGGCATTCCTGATGGTCGAATATGTCGGCATTCCCTACTCGGAAATCGTCAAGCATGCGCTGCTGCCGG
CGGTCTTCTCCTACATAGCGCTTCTCTACATGGTGCATCTGGAGGCGGTGAAGCTGAACATGCAGCCGATCCC
CCAGCGTCCGACTCCGATGCGCGAGCGGTGGCTTCGCATGGGCCTCGGACTTGCCGGCAGCGTGCTGGCAATC
TGCCTTCTCTATTACGGAATCATCGCCATTCGGGCCGCATTCGGAGAAAGCGCGCCTCTGCTGCTCGCGATTG
CCGGCGTGGCGCTCTACATCGCGACGATCTGGTACTCGTCGCGCTATCCGGACCTGGAGCTCGACGATCCAGA
CGCGCCGATCCTGGAACTGCCGCGGGCATGGGATGTGACGCGGACCGGGCTGGATTTCCTCATCCCGATCGTC
GTCCTGCTCTGGTGCCTGATGGTCGAGCAGCTTTCGCCCGGGCTTTCGGCCTTCTGGGCAACGCTGGCGATCA
TCGCCATCGTCGCCACCCGCAAGCCGCTGATGGCGATCTTCCGGAAGGAGGATGTTGCTGGTTCGGTCCGCGC
CGCCGCCTGGGACCTGACCGACGGCCTGGCGCTCGGTGCCCGCAACATGATCGGCATCGCGGTCGCCACTGCC
ACCGCCGGTATCGTCGTCGGAACGATCACGCTCACGGGCCTCGGCCTGATGATGACGGAGCTCGTCGAGTTCA
TTTCGGGCGGCAACGTCATCATGATGCTGATCCTCATCGCCGCGATCAGCCTAGTGCTCGGCATGGGAATTCC
GACCACCGCCAATTACATCCTGGTGGCCACCCTCATGGCGCCGGTCGTCGTCGAGCTCGGCTCGCAGGCGGGC
CTCGCCATACCGCTGATCGCAGTCCACCTCTTCGTTTTCTATTTCGGAATCATGGCCGACATCACGCCGCCGG
TTGGGCTGGCTGCCTTCGCCGCGGCGGCGATTTCCAAGGAAGACCCGATCGCCACGGGCTTTCAGGGTGCGCT
CTATTCGCTGCGCACCGCGATCCTCCCGTTCGTTTTCATATTCAACCCGGCGATCCTGCTGATCGGCGTCGAC
ACCTGGGCGCATACGATCTGGGTGGCGGCCATATCGCTCGCTGCGATCCTCATTTTCTCGGCCGCGACGATGA
ACTGGTTCGTGACAAAGAGCCGGTTGTGGGAGAGCGCGGTGCTGCTGCTCGTCTGCTTCACGCTGTTCCGTCC
CGATTGGTGGCTCAACCAGATCTCGCCGCCCTATGAAGAACTGCCCGCCTCAGAATTCCTGCGAGCTGTCGAG
GAGGCTCCGGCGAAAAGCCGGATCAACTTCGTGGTTCAGGGCTTCGACCTGATGGGCGACGAGGTCCGCAAGA
CCGTCAACGTGCCGCTCGGCGAGCCCGGCGAACCGCTTCAACGGCTGCAATCGATCGGGCTCACGGTCACACC
GGCGGGCGAGAGTCTGATGATCAGCAATGTCGACTTCGGCTCCTATGCAAAGCGCATCGGCCTCGATGTCGGC
TTTGACATCGTCGCAGTGCTGAGAAAAGCCGAGCAACCGTCAAGCGCCATCCCGGTGGGTATCGCGCTGGCTG
TGACCGCGGGAATTGCCGGCTTACAGTTCGCGCGCAAAAGGGCCGATCGCAGCGAAGCCGGCCTGACGGGGGC
CGCGCGCAAATGAATGAACATCAGCATATCTCAGCGGACGGACACGATGTCCGCCGTTATCGTCACTGGGCCG

GGTACCGTGCGCATAGAGACGTTGCCGTTGCCGCAACCGGGGCGCGGCCAGGTGCGGATAAGGCTCGAAGGCT
GCGGCGTATGCGCCTCCAATCTCGTTCCCTGGGCGGGTCCGGAATGGATGCAGTTTCCGACGGAACCCGGCGC
GCTCGGACACGAGGGCTGGGGAATTATCGACGCGGTCGGCGAGGACGTGCACGGTTTCGCGCCGGGCGATCGC
GTGGCGGCGCTTTCCTACCATGCCTATGCCACGCACGATATCGCCGATCAGAGCGCGATCGCGCCCTTGCCGG
CGTCTCTTGCAGGTCAGCCCTTTCCCGGCGAACCGCTCGGCTGTGCCTTCAACATCTTCCGGCGCAGCGCGAT
CAGTCCGGGAGAGACCGTGGCGATCGTCGGCATCGGTTTTCTCGGGATTCTTCTGACCGAGCTCGCAAGCGCT
GCCGGCGCGCGGGTGATAGCGATATCGCGCAGGCCGTCTTCGCTCGCCGCGGCCAAGCAGGCCGGCGCGAGCC
AAGTGATTCCCATGGACGATCACTGGCGGATCATCGAAAAGGTTAAGCAACTGACCGGCGGGCGCTTCTGCGA
TTGCGTCATCGAGGCGGTCGGCAAGCAATGGCCGCTCGATCTTGCCGGCGAACTGACGAAGGAGCGCGGCAGG
CTCGTCGTCGCAGGATATCACCAGGATGGCCCGCGGCAGGTGAACATGCAGCTCTGGAACTGGCGCGGCCTCG
ACGTGATCAACGCGCATGAACGCGATCCTGCCGTCTACATGCGCGGCATGAACGAGGCGATCGAAGCGACCGC
AGCGGGTCGGTTCTCTCCCTCGCGGCTCTACACCCATCGGTTCCCGCTCGAAGAACTCGGCGCCGCGCTCGAC
ATGACGCGCGACCGGCCGGAAGGTTTCATCAAAGCGCTGGTGATCTGCCATGAGTGAATGACCCGCCTGCGGC
TCCGTTCGCTGGTCCTGCTCCTGCTTGCCGCCTTCCCGGCGGCGGCGGCCGAGGAGCCTGCCGGCTACCGCGA
GAGCGAGTACCGCGCCGAGGTGCCGGCGACGCTTTCCGGTGCGACGGTCGTGTCGACCGAAGCTGCCCATGCA
TTGTGGAAGACCGGTCAGGTCGCTTTCATCGACGTCCTACCGCGGCCGCCGAAGCCGACCAATCTCCCCGAGG
GGACGGTCTGGAACGAAAAGCCGCGCCTGTCCATTCCATCTGCCCTATGGCTGCCGAATGTCGGCTATGGCCA
GCTGGCCGACGCCATGCACGCCTATTTTCGCGCCGGCCTGGAAAAGGCAACCGGTGGCGACAAGGACCGGCCG
GTGCTGTTCTTTTGCCTCGACGGCTGCTGGATGTCCTGGAATGCCGGAAAGCGCGCCCTGGAATATGGCTACA
CACGTGTCTTCTGGTATCCGCAGGGCACCGATGGCTGGACGGCCGCCGGCTATCCCACGGAGCGCATCGAGCC
GGAGCCCGGCGGCCGGTGAATGGCGGGCAAACAACTGCTTTTGATGGATGGAATCCATAAACGATTCGGACCG
CTCATCGTCAGCGCAGGCGTGACCCTCGACGTCGAGGAGAACGAGATTCACGCGCTGCTTGGCGAAAACGGCG
CCGGCAAGACCGTTCTGATGAGCATTCTTTGCGGTGTTCTCTCGCCCAATGAAGGGCAGATCGTCTTCAAGGG
ACGGCCACTCAAGCTAGGCTCCCCCCGCGAAGCCATCAAACATCGCATCGGCATGGTCCACCAGCACTTCATG
CTGGTCCCCAACCTCACGGTCGCGGAAAACTACGTGCTAGGACAGGGCTCGCCCTTCAGCATGATCCGCGACA
TGAGGGCGGTACATGCGCGCATTCTGGAGCTCAGCGACCGCTACGGTCTCGACGTCGCGCCCGACGCGCTCGT
CTCCAGCCTGTCGGTTGGCGAACGGCAGCGGGTCGAGATCTTGAAGGTGCTCTACCACGGCATCGAACTGCTG
ATCCTGGACGAGCCGACCGCGGTCCTGACGCCTCAGGAAACCGACCGTCTCCTGCATCTGCTTCGCCAACTCG
TCGCAGACGGAAAGACGGTCATCTTCATTTCGCACAAACTGGACGAGGTGATGCGCGTCAGTGATCGCGTCAG
CGTCATGCGCGATGCCAAGGTCGTCCGTACGGTGAAAACGTCCGAGACGACCGCGCGCGAGCTGGCGCGCATG
ATGGTCGGCAGAGACGTTCTGATGGATTTGCCGCGGGCACCTCTCGAGCCCGGCCGTGTCGTGCTGTCGGTCG
AGCATCTCAGCTGCGACGGCGAAGCCGGGCTTCCGGCGCTGCGAGACGTATCTTTCGAGGTCAGGGCCAATGA
AATAGTCGGGATCGCAGGCGTCTCCGGCAATGGCCAGAGCGAGCTGGCGCTGGCCCTGACGGGGCTTCTTCCG
GTTGCCTCCGGCAGCGTCACGCTCGACGGGACCCAGCTGGCGGGCCTGTCGTCCTACGAGATCAATCAATTGC
CTATGGCGCATATCCCCGAGGACCGCCACAGGATGGGCATCGTGCTGCCGCTGCCACTGACGGAGAATGTGAT
CCTTCAGCGGTTCGACAAGCCTCCCTTCTCGTCTCGTGGATTGCTCGACCTGAACGAGATCACGAGGCAGACC
CGTGATCTCATGCGCCGCTTCAAGGTCAAGGCGTCGGGACCGGGCGACCGCATCCGGAATTTGTCGGGCGGCA
ATCAGCAGAAGCTCGTCGTCGGGCGCGAGTTGGACCGCCGGTTCGATTTCCTGCTGATCAATCAGCTTACGCG
CGGTATCGACATCGGAGCGACCGAGCTGGTGATGCACAAGATTCTCGAGCAGCGCAGCGCCGGCAAGGCCATT
CTGCTGATATCCACGGAGCTCGAGGAATTGTTCACGCTCTCCGACCGCATCCTCGTCATGTATGAAGGCCGCC
TCGTCGGCGAGATTCCGCCCGACCGCAGCCGTCTCGAAGAGATCGGGCTGCTGATGGCCGGCAAATCTCCGCT
ATCGGCAGCTTGAATGCCGAATGCTTCCGACATCCTCATCGACACGCTTATCGAATGGAAGGTCGACGTCGTC
TTCGGCCTGCCCGGAGACGGCATCAACGGCATCATGGAGGCGCTCAGGAGGCGGCAGGATCGCATCCGGTTCG
TTTCCGTGCGCCACGAGCAATCGGCCGCCTTCATGGCATGCGCCTATGCCAAATTCACCGGCAGACTCGGCGT
CTGCCTCGCCACATCGGGCCCGGGCGGCACAAATCTGCTGACCGGCCTCTACGATGCGAAGCTGGACCAGATG
CCGGTGCTCGCGATCACCGGCATGCAGTATCACGACCTGATCGAGACGTTCTCCCAGCAGGACGTCGATCTCA
CCCGCGTCTTCGAAAACGTCGCCGTCTACAACGCGCAGGTGAACGACGCAGCCCACATGGAAAACCTCGCGAA
CCTTGCCTGCCGGTCGGCGCTCTCCAAGCGCGGCGTGGCGCATCTTTCGATCGCCAACGACGTGCAGGAGCGG
ATGGCTGATGGCGGGCGGTCCCGCCGCAACCGGCCGGGGCACATGCCAAGCCGGTATTTCGCCGGCCAGCTCG
TGCCGCTAGAAAGCGATCTTTCGAGAGCGGCGGATCTCCTGAATGCGGGCAGGAAGGTGGCAATTCTTGCGGG
GCGCGGCGCACTCGAGGCGAAGGAGGCTCTGCGCGAGACGGCGGAACTGCTGGGCGCGCCGGTGGCAAAGGCT
CTTCTCGGCAAGGCGGTGCTGCCCGACGATGATCCGCTGACCACCGGCGGCATCGGCATACTCGGCACCGCGC
CGTCGCAGGAGATCATGCAGCAATGCGACACGCTGCTCATCGTCGGCTCGACCTTCCCCTATATCGAATATTA
TCCCAGGCCGAACACGGCAGCCGGAATCCAGATCGACCGTGACCCGCAGCGCATCGGTCTGCGCTACCCCGTC
GAGGTCGGGCTGGTCGGCGAGGCAGGCGAGACCCTCCGCATGCTCAACAAGCGGCTCAAACGCAAGCAGGACA
GGTCGTTCCTCGAGCGGGCGCAAGAGCAGACGCGAAACTGGCGCCGGGAACTGCGGGCGATCGAAGGCGACAG
GAGTTCTCCCTTGAAACCGCAGGCGGCGGTCGCGGCTTTCGGCCGGCGCATCGCGAAGAAGGGTATCGTCGTC
ACCGATTCCGGACAGAACACCGAGCTTGCGGCCCGTCATGTCGATCTCGGTGCGGACCACATGTTCGCGGTCT

CCGGCGCCCTCGCCTCGATGGCTTCGGGCCTGCCCTATGCTATCGCGGCGGGTATCGCGATGCCGGACCGCCC
GGTCTATGCGGTCGTCGGCGACGGCGGCTTCGCGATGCAGCTCGGCGAATTTGCGACAGCCGTCGGATACCGG
ATTCCTTTGAAGCTCCTGGTGATCCGAAACGACATGCTGAACCAGATCGCCTGGGAGCAGATGATGTTTCTCG
GCAATCCGCAGTTTGCCTGCGAATTGCCGCCGATCGATTTCGCCGCGGCGGCGGAGGCCATGGGCGGCCGGGG
CTTCACCGTCCGGTCCTTCGGCGAACTGGACGGGATCATGGATCAGGCCTTCGCCGTGGATGGGCCGGTGGTC
ATCCAGGCGCTGGTCGACCGCTACGAGCCGCTGATGCCGCCGAAAATGCCGGCGGACTACGCCCGGAACTTCC
GCGCCGCGCTTCCGGAAACGCCGGGGCACGAGAAGATCGAGGAAAACCTCAGGAATTCCTCCGTCGGCAGGAA
GGTAACCGACGAGGAGCCGCAGCCGCCGCATGAGGCGGCGCCGGACGAGAACACCGGCGAATTGCCGGGAATT
CCGTAGATGGACAAGGTCATTCTGATCACCGGCGCCTCCGGCGGCATCGGCGAGGGCATCGCTCGAGAACTTG
GCGTTGCCGGTGCGAAAATATTGTTGGGTGCACGCCGCCAGGCCCGTATCGAGGCCATTGCCACCGAGATTCG
AGACGCAGGAGGTACCGCCCTGGCGCAGGTGCTGGACGTGACCGACCGTCACTCCATGGCAGCCTTTGCGCAG
GCCGCGGTCGACACTTGGGGACGCATCGACGTCCTGGTGAACAATGCCGGCGTCATGCCGCTCTCGCCGCTTG
CCGCAGTTAAGGTGGGCGAGTGGGAGCGCATGATCGACGTGAACATCAAGGGCGTGCTTTGGGGCATCGGGGC
CGTATTGCCAATCATGGAGGCACAGCGTAGCGGGCAGATCATCAATATCGGCTCGATCGGAGCGCTGTCGGTC
GTACCCACAGCCGCGGTATATTGCGCAACGAAGTTCGCAGTCCGCGCGATCTCTGACGGACTGCGCCAGGAGA
GTACCAATATCCGCGTCACCTGCGTCAATCCCGGCGTCGTCGAAAGCGAACTTGCTGCAACCATCACTCACGA
GGAGACCATGGCGGCGATGGACACCTATCGCGCCATCGCGCTTCAGCCTGCCGACATCGCCCGTGCGGTCCGG
CAGGTGATCGAGGCCCCGCATTGCGTGGACACCACCGAGATCACGATCAGACCGACCGCATCGGCAATTGAA
TGAACGCCGTAGAACTGCGCGCGATTCAAGCGCCGATAAAGGAACGCTATCGTGCAGAGCCCGGGGCAGCCCG
TTTCACGCTGAAGGCGCGCGGGACACTTGACGAAAAGAACATCGCCTGCAAGGTCGAGACTGGGCACGCACTG
GCAGTGGCCGGCCTCCACAGGGCTACTGGCGGGACCGGCCTTGAACTTTGCTCGGGCGACATGCTGCTGGAGG
CCCTCGTCGCTTGCGCGGGCGTGACGCTCAAGGCCGTCGCAATGGCCCTCGAGATTCCGCTGTCAGCCGGCAC
AGTATCCGCTGAAGGCGATCTCGACTTTCGCGGCACTCTGGGTGTCGTCAAAGATGTACCGGTGGGTTTCGAC
CAGATCCGCATTATCTTCGAGCTTGAGACGGACGCCCCACAGGAGAAGTTGGATCAGTTGCTAAAGCTGACTG
AACGCTACTGCGTCGTGTATCAGACCATCCGCAACGGGCCCTCGATGGCTGTCGAGATGAGACGCTCGGTGTA
AGTGTCGAGATTTAACCCTACCAGAGAAACAGGTTCGATTTACGATGCCGCTGCGGCGTGGCGAGACCGGTGC
CTCTTGGCGGATGGTTCGGTGCTATCAGATGAGGCCCTCTGGGCGGTCGCCCTGCTCGACGAACTCGACGAGC
GCTTCGTCAAGAATATCGACGCAGGCGAAGGCGATTTCCTTTCCAAGCTGGAGGTGCAGCTGTCGTCCGGCTC
GCCCCAGTGCCGACAGTTGATGGCTGAGAGCCTGTGGTTGCTGCTGCTTTTCCCGTCGAATGTCGGCGCCGCC
AGAAAGCGCGAAAACGTGCGCGAGGTCTGGTCTTGGTCGGGCGACGAACTGGACGAACACCATCCGTTTGTCG
ACGACAAGGTTCTCGCGGGCATCGGCTCGGCCGGTACAGCCTATAACACCCACCGTTGGCGCGAGCTTGTTTT
TCTGATCGGCGCGGTGCGCGACTTCAAGCGACGTGAGAGCGCCCAGCGCCACGACATCCTCCGTGAGCCATGG
GCGTTTTCCGGCTGGCTGAGCAGCCTGTCCGAGGCGCGCCACCGGCAGTTGCTACAGATCCTGCCGCACCTGT
TATTTCCAGAAAATTTCGAGCGGATCAGCTCCGAGGGCGACAAGCGCCTGATCCTAGCCGGGTTCGGCGACGC
GCCGGAGAAGGAAATCCGGAAGTGGAGCACCACCGAGATCGATCGCGCCTTGCTCAACCTGCGCGCTCGGCTT
GAGCAGGAGCAAGGTGTCGACATCGACTTTTACCAGGAAGAGTTCTCCGCCCATTGGAAGACCCAGACGCAAA
ATTGGCTCCTGAGCTGGAACCCTTCCAGGTGGACATGGGACACCCTCGCCGAAGACAGGGCCGCCACGAACAT
TGGCGAAAAGGCTGAGAACCGCTGGCGCTGCGCATCGACGAAGGCGCGCGAGGGCGACCGCGTCTTCCTGGTG
CGGACTGGAGAGCCACCAAAGGGCATCGTCGCCGTTGGCAAGGTCACCCGTGCACCATATGAAGCCCCACATT
GGGAAGCGGCCCGCGCAGAAGCGGGCGAAACGACGCGCTTCATCGACGTCGCTTTCGACGCCGTTCGGGACGC
GTCGATCGACCAGATCGTGAAGCTCGAAGAGTTGGAGCGTCGGGAAGCCGGTCAGGAGTGGAACCCTCAATCA
TCGGGGATAGAGATCAAAAGCAAGCCGGCGCGGACACTCGAGAGGCTGTGGAAGGAGTTACCCCCCGTTGCCG
GACCATCCGCGCCCGATGATGAAACAGCTGCTACCAAGGCGACACGCTCGACCGGATCGCCCCTGAACCTGAT
CCTCTACGGGCCGCCAGGGACCGGAAAAACCTACCGGCTGATGAACGACTATGTGCCGAACTACCGCGATGAG
GCCGAGGACCGCTCCGAATTCATTACCTTCCACCAGAGCTACGCCTACGAGGACTTCGTCGAGGGCATCCGGC
CGTTGGCCAAGAACGGCAGCATCACCTACGAGGTCCGACCCGGGGTACTGAAACGACTCTGCGCACGCGCACG
CCAGTCGCCCCAGAAGCGCTTTGCCCTATTCATCGACGAAATCAACCGGGGAAACATAGCTAAGATCTTCGGC
GAGTTGATATCATTGGTGGAGGTCGACAAGCGGATCCACACCGACACGTCGGGGAAGCGGTTACCCGGCTGCA
AGGGCCTGGAAGTCACGTTGCCCTACTCGGGCGAACGTTTTGGCGTGCCAGCGAATGTCGACGTGATCGGCAC
GATGAACACCGCGGATCGCTCGATCGCGCTCCTCGACAGCGCGCTGCGCCGCCGCTTTCGGTTCGAGGAGCTC
ACACCTAAACCCGAGCTTCTGCAAACGGTGGATGATGACGCCGGCGACACGATCGACCTGCGACAGTTGCTCG
CGGCCATGAATACGCGCCTATCGCGGCTGCTGCATCGGGATCAAACGTTGGGACATAGCTACTTCTGCAACGT
CAAGTCGTTTGGCGATCTTCGCAAAGTCTTCGCACGCGAAATCCTGCCCTTCCTGCAGGAGGCCTTCTACGAC
GACTGGCGACAGATCCGGCTTGTCCTGGCCGACCAGACTGTGGGCGAGGAATTCCAGCTGGTACGGGCGCATA
CGCAAAGCGCCCAGCGGCTCTTCCCCCTTGCCGACGGCAACGAGATAGGCGACGGCGAGACATTCGAAGTCGT
TCGCGAGGACGACATCACGGCCGACGCGATCCGCAAAATCTACGAGCCACCGGAATGAATGAACGACAAGATC
ACCAACTGGACCAGATCCGACGACGCTATGGTCGAAACGGCCATTCGTCGCGGAGCGACACGCCGCGAGCTCC
TGCAGATGATGCTGGCCGGCGGTGCCGCCCTCTCCGCCGGCAGCCTGGTGCTCGGCCGAGCCGGGAATGCGGT

TGCTGCAACGCCGGTCTCCGGCGGTACGCTCAGAGCGGCCGGGTGGTCGGCTTCCACCGCCGACACGCTCGAT
CCGGCCAAGGCATCGCTCTCCACCGACTATGTCCGCTGCTGCTCCTTCTATAACCGCCTCGCCTTCCTCGATA
AAGGCGGCACGCCGCAGATGGAGCTGGCCGAAGCCATCGAAACCAAGGATGCGAAGACCTGGACCGTCAAGCT
GAGGAAGGGCGTCACCTTCCATGATGGCAAGCCGCTAACAGCCGACGACGTGATCTTCTCGCTGAAGCGCCAC
CTCGACCCGTCTGTCGGTTCAAAGGTCGCCAAGATCGCCGCGCAGATGACGGGCTTCAAGGCGGTGGACAAGC
AGACGGTCGAAATCACGCTTGCGAGCCCTAATGCCGACCTGCCGACGATCCTCTCGATGCACCACTTCATGAT
CGTCGCCGACGGCACCACCGACTTCTCAAAAGGGAACGGCACCGGCGCTTTCGTGAGAGAAGTCTTCGAGCCC
GGCGTGCGCTCCGTCGGCATCAAGAACAAGAACTACTGGAAGTCCGGACCGAACGTGGACTCTTTCGAGTACT
TCGCGATCAGCGACGACAGTTCCCGGGTGAATGCGCTCTTGTCCGGCGACATCCACCTCGCCGCCACGATCAA
TCCGCGCTCCATGCGCCTGGTCGAGAGTCAGGGCGCCGGCTTCGTCCTCTCGAAGACGACCTCCGGCAACTAC
ACCAACCTCAACATGCGGCTGGACATGGAACCCGGCAGCAAGCGCGACTTCGTCGAAGGCATGAAGTATCTCG
TCAACCGCGAGCAGATCGTTAAGTCGGCGCTTCGGGGTCTGGGTGAGGTCGGCAACGACCAGCCGGTATCCCC
GGCCAACTTCTATCACAATCCCGACCTGAAGCCGCGCGCCTTCGACCCCGAGAAGGCGAAGTTCCACTTCGAG
AAGGCCGGCATGCTAGGCCAGTCGATCCCGGTGGTTGCCTCCGATGCAGCGAACTCCGCGGTCGATATGGCGA
TGATCATCCAGGCCTCCGCTGCCGAAATCGGATTGAAGCTCGATGTCCAGCGCGTTCCCGCAGACGGTTACTG
GGATAATTACTGGCTGAAGGCGCCGGTCCACTTCGGCAACATCAACCCGCGCCCCACGCCGGATATCCTGTTC
TCTCTGCTCTACTCCTCGGAGGCTCCGTGGAACGAGAGCCAATACAAGTCGGAGAAGTTCGATAAGATGCTGA
TCGAGGCGCGTGGCTCGCTCGACCAGGAGAGGCGCAAGGCGATCTATAATGAGATGCAGGTGATGGTCGCTAG
TGAAGCCGGCACCATCATTCCGGCCTATATCTCCAATGTCGACGCGATCACCGCCAAGCTTAAGGGCCTGGAA
GCCAATCCGCTTGGCGGGCAGATGGGTTATGCTTTTGCGGAATATGTCTGGCTCGAGGCCTGAATGTTCGGCA
AGAAATCCATTTCGGAAGAACGGAGAGCCGAAGCAGCGGTCCAGCCCGTCGTCGAAAACGAACTGCAGGCCGC
GCACGCCCCTGTCCAAGTTCGGAAGCCAGCCCAGGTTCCGGCAACTGCGAAACCCGAGAATGCCGAACAATAT
TACAGCTTGAAGAAGGAAATCTTCAGCGCCCTCATCGCGACGATCGACGTTGCAGCACTCTCCAATATGGATG
GCGAGCAGGCGCGCAACGAAATCGGCGCGATTATAAACGACATCGTCGCGGCCAAGAAGGCCGGCATTTCGAT
GGCCGAGCAGAACGACCTTCTCAGCGACATTTGCAACGACATCTTGGGTTACGGCCCGCTCGAGCCCCTGCTC
GCACGCGACGACATCGCCGACATCATGGTCAATGGCGCAAACCAGGTCTTCATCGAAGTCAACGGCAGGGTGC
AGGAGACGGGAATCCGCTTTCGCGACAACGAGCAGCTACTCAATATCTGCCAGCGCATCGTCAGCCAGGTTGG
CCGGCGCGTCGACGAATCGAGCCCTATCTGCGACGCGCGTCTGGGCGACGGTTCCCGTGTCAACGTGATTGCG
CCTCCGCTGGCAATCGACGGCCCCACGCTGACGATCCGCAAGTTCAAGAAGGAAAAGCTGACCCTCGACCAGT
TGGTCCGGTTCGGCTCGATCTCGCCGGAGGGCGCCGAGGTTCTCAAGATCATCGGACGTGTGCGCTGCAACGT
CCTCATTTCCGGCGGTACCGGCTCCGGCAAGACAACGCTTCTGAACTGCCTGACCGGCTATATCGACCACGGC
GAACGCGTCATCACCTGCGAGGATGCGGCAGAGCTTCAACTGCAGCAACCGCATGTGGTGCGCCTCGAAACGC
GGCCGCCGAACATCGAGGGGCAGGGCGAGATCACCATGCGCAACCTCGTGAAGAACTGCCTGCGCATGAGGCC
CGAGCGGATCATCGTCGGCGAGGTGCGCGGCCCGAGGCCTTCGACCTGCTGCAGGCGATGAACACGGGCCAC
GACGGCTCGATGGGAACGCTCCACGCCAACTCCCCGCGCGAAGCGATGGCACGCATCGAGGCGATGATCACCA
TGGGCGGGAGCTCGCTGCCGGCCAAGACGATCCGGGAGATGCTCGTCTCGTCAGTCGACGTGATCGTGCAGGC
AGCGCGACTTCGCGATGGTTCGCGCCGGATCACCCACATCACCGAAGTGCTCGGCATGGAAGGCGAGGTGGTC
ACGACGCAGGATCTCTTCGTCTACGACATTCTCGGCGAAGACGAGAAGGGAAACATCATCGGAAGACACCGGT
CGACCGGGATAGGTCGCCCGGCATTCTGGGATCGCGCACGCTATTACGGCGAAGAGGCCCGGCTTGCCGCCGC
CCTCGACGCGGCGGAATTGAAGGCCGCCGCCTGAATGTCTATCAGTCTTCGCGGCCGCAGCGTCCTGACGCTC
GACGAATTCACTTCAGGGGAGATCGGGTTTCTGCTGCATATCGCCGCCAGGGTGAAGGCCGAAAAGCGAAGAG
GCAATGAAATCCCCCTTCTCAGCGGCAAGAACATCGCGCTGCTTTGCGAGGAGGGTTCGATGAACGCCCTCTT
CGGATCTGAAGTTGCGGCCTTTGATCAAGGCGCGCGAGTCGTTGGTCTGACGTTGCCCGCCGGCCATGGCGGG
CAATGCGCATCACTGAAGGATATGGCCCGTGTGCTTGGCAGGATCTACAACGCGGTCGAATATTGCGGTCGCA
GCCAGGGCGCCGTCGAAGAACTGGCGGAATACGCCCGCGTTCCCGTCTACAATGGCTCTTCCGAAGAATGCCA
TCCCATCCAGGCTCTGGCAGACTTCTTCACGATGCAGGAAGCTGTCGACAAGCCGTTGCAGGAGGTCACGCTA
GCGTTTCTGGGCGACGGTCGCAGTCCTATCGCCCGTTCGCTCGCGACCGGAGCGGCCAAGCTCGGCATGGACT
TCCGCATCGTCTCGCCGCCAATTTTCTGGCCAGCTCCTTCCTTCATCGACGACGTGACCTCGGAAGCGCATGA
GAATGACGGGAGTTTCACGGTCCTGGAGCATGTCGGCGCCGGGGTCTTAGGCGCCGACTTCGTCTACACGGCT
TCGTGGCTGGGAGAGCAAGAAGCGGGCTGGGCCGAGCGCATCAGGCTGCTGGCGCCTTTTGGCGTCAGAGCCA
ATGTCATGGAAGCCACTGGCAATCCGGGTAGCAAGTTCATGCATTGCCTGCCTGCCTTCCACAACAGTGGCTC
AAAGGCCGGAGCCCGCGTCGCGAAGCAGTTCGGTCTCGACTGCATGGAAGTCTCGGACACGGTTTTTGAATCG
AAAGCTTCTGTGGTGTTCGATCAGGCGGAAAACCGGATGCACACGATCAAGGCCATTTTCATCGCGACCATGG
GGGACCGGGAGACCGATGCAAGAGATAGAGACCCTTAGATGGCACTGAAGGCAAAACCGCATCCGAGCCTGTT
GGCCATTCTCTTCCCCGTCGCCGTCACCGCCGCTGCTGTCTATGTCGTCGGCTGGCGCTGGCCGGGGCTCGCC
GCCGGCATGTCGGGGAAGACGGCATATTGGTTCCTGCGGGCGGCGCCCGTGCCAGCGCTACTGTTCGGGCCGC
TCGCCGGCCTGTTGGCCGTTTGGGCCTTGCCATTGCACCGGCGAAGGCCAGTCGCAATGGCGAGCCTCGCCTG
TTTTCTGACGGTCGCGGGCTTCTACGCGCTCCGTGAATTCGGCCGGCTGTCCCCCTCTGTGGAAAGCGGTGCG

CTATCATGGGACCGGGCGCTGTCCTACCTCGACATGGTCGCTGTCGTCGGCGCGGTCGTTGGCTTTATGGCCG
TCGCTGTCTCCGCGCGCATCTCCACCGTCGTGCCCGAACCGGTAAAGCGCGCTAAGCGCGGAACCTTCGGTGA
CGCGGACTGGCTGCCGATGGCGGCGGCTGGAAAGCTGTTTCCGCCGGACGGCGAGATCGTCATCGGCGAGCGC
TACCGCGTCGACAAGGACATTGTCCACGAACTGCCCTTCGATCCAAACGATCCGGCGACTTGGGGCCAAGGCG
GCAAGGCCCCTTTGCTGACCTACCGCCAGGACTTCGATTCCACCCATATGCTGTTCTTTGCGGGCTCGGGTGG
ATACAAGACGACCAGCAACGTCGTGCCGACGGCGCTGCGTTATACCGGGCCGCTCATCTGCCTCGATCCGTCG
ACCGAGGTCGCGCCCATGGTGGTCGAGCACCGAACCCGCGTGCTCAGCCGCGAGGTGATGGTTCTTGATCCGA
CGAACCCGATCATGGGCTTCAACGTGCTCGACGGGATCGAGCACTCACGGCAAAAGGAAGAGGACATCGTCGG
CATTGCCCACATGCTGCTGTCAGAAAGCGTGCGCTTCGAAAGCTCGACCGGCTCCTACTTCCAGAACCAGGCG
CACAATCTCCTGACCGGTCTGCTCACCCATGTGATGCTGTCGCCCGAATATGCCGGACGGCGGACGTTACGCA
GTCTCCGGCAGATCGTATCCGAGCCAGAGCCGTCGGTGCTGGCGATGCTGCGTGACATTCAGGAGCATTCCGC
GTCCGCTTTCATCCGCGAAACGCTCGGCGTCTTCACCAATATGACCGAGCAGACTTTTTCGGGCGTCTACTCT
ACGGCGTCGAAGGATACGCAGTGGCTGTCGCTCGACAGCTACGCCGCACTCGTTTGCGGCAACGCGTTCAAGT
CGAGCGATATCGTCTCGGGCAAGAAGGACGTGTTCCTGAACATCCCCGCCTCGATCCTGCGCTCCTATCCGGG
CATCGGCCGCGTCATCATCGGCTCGCTGATCAATGCCATGATCCAGGCCGATGGCTCATTCAAGCGCCGGGCG
TTGTTCATGTTGGATGAGGTCGATCTCCTCGGCTACATGCGCCTCCTCGAGGAGGCACGCGACCGCGGCCGCA
AGTACGGCATCAGCATGATGCTGCTGTACCAATCCCTGGGGCAGCTGGAGCGGCATTTCGGGAGGGACGGTGC
GGTGTCGTGGATCGACGGATGCGCCTTCGCCTCCTATGCCGCAGTTAAGGCGCTGGACACGGCGCGCAACATT
TCGGCGCAATGCGGAGAAATGACGGTTGAGGTAAAAGGAAGCTCCCGCAATATCGGCTGGGACACGAAGAACA
GCGCATCGCGCAAATCGGAAAACGTCAATTACCAGCGCCGGCCGCTGATCATACCGCACGAAATCACCCAGTC
GATGCGAAAGGACGAACAGATCATCATCGTCCAGGGCCACAGCCCGATCCGCTGCGGGCGCGCGATCTACTTC
CGACGGAAGGACATGAACGAGGCGGCGAAGGCCAATCGCTTCGTCAAGGCGATCCCTTGAATGGCTGAAAAAC
AACATATCGACTTGGAGGCCGCACCTGCGAGCGCCAAAGACCTGCGTATGCAACTCCTTCAACGACAGATGGA
GGAGATGGAGAAGGAGCGACAGTTCAAAGCGGTACAGGAGCAAAAGCTGACTGACTTTGCCGGCGTATTCCTT
GAGGAGCACGTCACGCCAGAGGAGGTCGCCGTCGTGCGCCGCCTCGTGGCGAACGCCGTACGCGACGGAAAAT
TCGAGGCCATGGTTTACAGCTTCCCCTCGAATCTTTGCACCGATAGTGGCCGCGCAATAAACAGCGCCGACCG
CGATTGGCCGCAGACTCTCCAGGGCAAGGCGAAGGAATTCTACGAACGATATCAAAAATTCGGCAAGCCTCAG
GGCTTCAAGCTGAAAGCTATGATTGTCAACTTTCCGGGTGGAATGCCAGGTGACGTGGGCTTCTTCCTGAACT
GGGCTCCTGATGACGCTTAGGTGCTGGAATTCCTGATCTGCTCGCTGCTCACCATTTTTCCGGACTACCTCGT
CCGCCGCTATGTCCAGGGCAAGCGTGTCGGCCGTGAGATAAATCTCTATTCCATGTGGTTTGAGCTCCGATAC
GGCATAACGGCCTGCCTTATCCTCACCATTTCACTGATCACAATGATCTTCTACTTCCATCCATCTACTAGCA
ACGTCACCGCGGTGTTTCGCACGGTAACGATCATGCCCGAGTCAACCGGGCGAGTAGCAGAGGTTTATGTTGA
CCTCAACGAGAAGGTTTCTGCCGGTGCTCCCCTCTTCCGCCTCGACGATTCCGAGCAACGGGCAGCACTTGAG
ACCGCCCGCCGACGCGTCGCCGAGATCGAGGCAGAGGCAATTGTTGCGCAGAGCGAACTGGCCTCCGCCGACG
GCCTTATCGCCCAAGCCGAGGGCGACTATCTCCAGGCGCTGAATGAACTCGAGACGCAGGTCGAATTGAACCA
GCGCAACCCTAACATCGTCGCAAGGCGCGAAATCGAGCGCCTTCAGGTCGCTGTCGACGGTCGCGATGGTGCG
CGTGCCGCGTCGATCTCCAACAAGCGGACCTTGGAAACGAAAATCGCCTCACTCCTGCCGGCGCAGAAGGCGA
GCGCTGAAGCGGCGCTGACCCAAGCCCAGGTGGAGCTCGACAAGACGATCGTACGGGCTGGAACCGCCGGCGT
AGTCCAGCAGTTCGCGCTCCGCGCTGGAGATATTGTCAATCCGATGATTCGCTCGGCCGGCATCCTGGTTCCC
GAGCAACGCCGCATTGGCTTGATCGCTGGCTTCGGCCAGATCGAGGCACAGGTGATGAAGGCCGGGATGATTG
CCGAGGCGACCTGCATCGGCAAGCCGTTCACCATCATCCCGATGGTGGTGACGGAGGTGCAAGACGTTATCGC
CGCCGGCCAAATTCGTCCGACCGACCAGCTCGTCGACGCGCAGCAACTTGCCAGTCCTGGCACGCTGACTGCC
TATCTGGAGCCGCTCTACGAGGGACAGTTCACCGGCGTACCACCTGGAAGCAGTTGCATTGCCAACGCGTACA
CGAGCAATCACGATGCACTACATGCGCCCGGCATCAGCACACCGCATTGGCTATTTCTGCACGTCGTCGATGC
GGTGGGATTGGTGCACGCCATGATCCTCAGGCTTCAGGCCCTGCTCTTGCCGGTCCAGACGCTAGTCTTCAAG
GGCCATTGAATGGCGAACGACGAACTGAGCGCCGGCTTCACCGGGAAGCCGACCGAACCGCCCACGGGTGTCC
GAAAGATAGCTAAGGTAGCCAGCGGCGCAGTTGGCCGCGAAGCGAACGGTGTCGTGGTAGGGGCCGCAGATCA
CCCATACACTGCAACAGGATTGGCGCTCACAGTCGGCGCCATTGCCTTCGCGGTCGGTTATACTATGGGTCGA
TCGTCAAGCGAAAAGGGCCGAAGTTACTGGCGATAGATGTCGACGCACAAAATTGCGATCGTAGCGATGCAGG
GCGTGCAATTGCTCGATGTGATCGGCCCGTCCGACGTGTTCGCGGAAGCAAACCGCCAGAGTGGCCTCGGCTA
CTACTCGGTAGAGGTCGTCAGCACTTCTGGCAGCCCCGTCAGAGGATCATCTGGATTGGCACTGATTCCCGAT
CGCCTAATCGACGAAGAACCGTCCGAAGTCGATACGCTGCTTGTAGCAGGCGATCCGCTCATCCAGGAGCGGC
CGCTCACCGAAAACCTCCTGCGCTGGGTGAAGCAGACCTCGGCCAGAGCCAATCGCGTTGGGTCCGTCTGCTC
CGGGGCTTTTGTGCTCGGCGAGGCAGGTTTACTTACCGGCAAGCGAGCGACCACGCATTGGTCGCAGGCATCG
GAGTTTGCAAGGCGTTTTCCGGATGTGAAGCTTGAACCAAATCGCATTTTTACAAAGGACGGCAACGTTTGGA
CATCGGCCGGGGTTACCGCTGGGATCGATCTGGCGCTTGCGATTGTCGAGCAAGACCTCGGTTTAGAGATTGC
CCTCAGAGTCGCTCGAGAGCTCGTGGTGTTTCTCAAACGGCCCGGCGGACAGACACAGTTCAGCACGCTCCTG
GCCGGGCAGATGGCCCAGAAGACGCCGATCCGTGCGGCCCAGGACTTCGTCGCCGACAACCTCGCCGCCGACC

TCACCGTGGCGGCCCTGGCGAAGCGCGTGGGAATGAGCGAACGAAACTTTTCAAGGCAGTTCAAACAGGAGGT
CGGCATGACACCGGCCGACTATGTCGAAAGCATGAGGCTAGAGGCGGGTCGCAGATTGTCCGAGGACACCAGC
ATTCCACTCAAGAGGATCGCCATTGATATAGGCTTCAGCGACGATGTCGCCTTCCGGCGATCCTTTGCCCGCA
GATTTGGCACATCCCCGGCTGCCTATCGGAGGAGCTTCGGGTCCTAGATGGGCATGCCCGCGGGAGCGATCTT
TCTCGTCTTCATGACCCTGCAGATTGTCTGCATCGCCGGCGCGCTCCTCTATCCGGACGAGTTCCGCTACCTC
TCGCCGCAGAACCTGACGATCCTGATGAAGGCGATCCCCGTGCTCGGGTGCCTCGCGCTCGGAGCCGGCGTAT
TGATGATCGCCGGTGAATTCGACCTTTCCATCGGCTCCGTGTACACGTTCACGGCCGTGCTGATGGCCTCGCT
CGTCAATGCAGGGCTGAGCGCCTTCATCGCAGCACCTATCGGTATTCTGACCGGCCTGCTGATCGGCTCGCTC
AACGGCCATATCACCCTGCGCTTCGGCCTGCCGTCCTTCATCGTCACGCTCGGTGGGCTCCTCTTCTGGCGCG
GCGCGGTGCTGCTCTACAACGGTGCCGTCCAGGTGCGTTTCGACCCGGAGCCGGTCTTCACCAGCCTCTTTTC
CGGCACTCTGTTCGGCGTGAACGCCGCCTTCATCTGGATCGTCCTCTTCGTCATAGGCTTTCACCTGCTGCTA
CACCGCCACCGCTTCGGCAACCACGTCTTCGCGACGGGTGGAAATCGCGGTGCGGCCGAGGCGATCGGCATCA
ATACCAGCCGCGTCAAACTGATTGCCTTTGCGATTGCGGGCGGCATGGCTGCGGTCGCGGGCATTCTTGCCAC
GGCCCGCGTGGGCAGCGTGCAGCCCGGGCAGGGTGCGGGTCTGGAACTCCAGGCGATCGCCGCCTGCGTCATC
GGCGGCCTTTCGCTACGCGGAGGACGCGGATCGATCATCGGCATCTTTCTCGGCGTGCTGCTCATCCACACGA
TAACCGACGTCCTGCTTCTCCTGCGGGCACCCGGCTTCTATCTCGACATGTTCATCGCCACGCTGATCGTGCT
GGCCGCCATTTTCAACCATCTAATCGAGCGGCGAGGTCTTGCGTGAATGATCAGAGCCTTCGGCTGGAACGAG
TTTCTCGTCATCGTCGCCGCGGCACAGTGGACGATCGCCCTTTCCGCGATCGCCTTTGCCGGCGGCAGCGTCG
GCGGACTGCTGGTCGCATTGATGCGCGTCTCCGAGACGCGAGCGCTGCGCCTTTTCGCCACGGGCTTCATCCG
CGTCTTCCAGGGGACGCCGCTTCTGATGCAGCTCTTTCTGGTATTCTTCGGCATGAAGATCTTCGGCTTTGCG
ATCAATCCCTGGATTGCCGCGACCATTGCGCTCGCGCTCCACGCCAGCGCCTTCCTGGGCGAGATCTGGCGCG
GCTGCATCGAGGCCGTGCCGAAGGGACAGCGCGAGGCGGCGACTGCGCTCGGCCTGCGCTATTTCCGGTCGAT
GCGTCATGTGATCCTGCCTCAGGCGGCCCGTATCGCCGTCGCACCCACCGTCGGTTTCCTGGTGCAGCTCATA
AAGGGCACCTCGCTCGCATCGATCATCGGCTTTACCGAGCTGACCCGGCAAGGGCAGATCATCAACAATGCGA
CGTTCAGTCCGTTCCTGGTGTTCGGTACCGTCGCCGCCGTCTATTTTCTGCTTTGCTGGCCGCTGTCGCTGAT
CGCACGCCGAATGGAAACACGTTTTTCCCGGGCAACGGCACGTTGAATGGGCAACAATGAGAATGGCGGCATA
GGCTTTTGCGTCTTCGTCGTCGTAATTGGATTTAGCACGGGTGCTGTTGCGCAGGAGCCAGCGAATCAATCCG
AAGCTGCGACGAGCCTGGAAGAAATCGTAGTCACCGGCGGACGATCCGCTCAGCAGATTTCTGAAATCGCGCG
AACCATTTACGTCGTCGATTCGGATCAGATCCAGGCCGAGGCCCGGTCGGGCAAGACGCTGCAGCAGATTCTG
GGCGAAACCATTCCGAGCTTCGACCCCGCCAGCGACGGAGCGCGTACGTCATTCGGCCAGAACCTGCGTGGCC
GGCCACCACTGATCCTTATCGACGGTGTCTCGATGAACTCGGCGCGCTCGCTGAGCCGACAATTCGATGCCAT
CGATCCGTTCAACATCGAACGGGTGGAGGTCCTGTCTGGGGCAACTGCAATCTACGGCGGGAACGCAACCGGC
GGCATCATCAACATCATCACGAAGAAGGGCAAAGACGCGGAACCGGGCCTGCATGCGGAAGTGACCGGCGGCA
TGGGCAGCGGCTTTGCCGGCAGCCAGGACTTCGACCGCAATGCGGCGGGTGCGGTCACCTATAACAGCGAAAA
CTGGGATGCCCGCCTTTCGATCGCCGGCAACAGGACCAGCGCCTTTTATGACGGCAGCGGCACGCTGCTGATC
CCCGACATCACGCAGACGTCCACCGCATTCAACGAGCGCATCGACCTGATGGGGTCTATCGGCTACCAGATCG
ACGACGACCGTCGCCTCAAATTTTCCGGGCAATATTTCGACAGCAAGCAGGACTCGGATTACGGGCTTTATTA
CGGGCCCTTCTTTGCAGCGCTCGCCGACCCGAGCCTGTTTGAAACCCGTTCCGGATACGAGTCCGATTTCAAT
CCGCAGACACGCCGCTCGATGTTGAACGTCACCTATACCGATAATGACGTTTTCGGTCAGCAGCTATTGCTTC
AGGGATCTTACCGGACTGAGCGGATAAAGTTCCATCCCTTCCCTGCTTCGGGCAATAGCGAGACGGGTCCTTA
CTTTTACGGCAGTTCGCAGGACACAGATTATTACGGCATCAGGGCTGCCTTGGTTGCGGAACCCACCGACGCG
CTGAAAATCACCTATGGCGTCGATGCGGATATGGATTCCTTCACGGCGCGTCAGAACATCTTCGACATGGTGG
CAGCCGGGCAATCCGGGGGGCTCGATTTCAATACGATCGGCAAGACCGGTCTTTATCCATCCATCGATGTCTC
CACCGTTGCCGGCTTTGCTGAGGCCTCGTACGAGGCTACCGACAGGCTCACTCTGAACGGCGGGGTGCGCTAT
CAGTTCGTCAACACTGAGGTATCCGACTTCATCGGAGCGGCTCAGCAGGTCGCCATCCTGCAGGGCAGAGCAA
CGTCTGCCGACACCATACCTGGCGGCGAAGTCAATTACGACGCCGCTTTGTTCAGTGCAGGCGCAACCTACCA
GTTGACGAATACGCAGCAGGTCTACGCCAATTTCAGCCAGGGCTTCGAACTACCAGATCCGGCCAAGTACTAT
GGAATTGGCAACTACTCCTTCTCCGGTGGACACTACACGCTTGTCAATAGCGTGAACGTGGGCGATTCCGCGC
TCGAGGCGATCAAGACCAACTCATTCGAAATCGGCTATCGTCTCGATGACGGCACCTTCAATCTTGAAACGGC
GGCCTACTACTCGCTCTCCGATCGTTCCATCAATCTCAATCGCTCATCGCTTGCCGTTGAGGTCATCGATCGG
GAGAGACGCGTCTATGGCATCGAAGGAAAGGCGGGCGTAAAGCTCGATCATGGCTTCGATGTTGGAGTTCTGG
GGCACTGGGTCAGAACCGAGGTCAAGGGAGCCGACGGTTGGGAAAAGGACTCCGTCGGCAGCGCGAGCGTGTC
CAAACTCGGCGGCTATGTCGGCTGGACCAATGACGCTCTCAGCCTGAGGTTTTCCGGTCAGCACATCTTCGAA
CTCACCGACGCCCAGAATTTCACGATCGACGATTATACGTTGTTTGATCTAACCGGCGGCTACAGGTTCGAGA
ATACCGACACGACGCTGAATTTCGGTATCCATAATGTCTTCGATACTGATTACACCACCGTCTGGGGCTCTCG
CGCCAAAGCCCTTTACGGCGGCCTAGCCGACGAGTCGGTGTTCGACTACAAGGGCCGCGGCCGCACGTTCGCG
GTCTCGCTGACAAAGGTTTTTTAAGTGCACATCCGGTTGCTTGGCGGACTTGAAGTAAGTTCCGGAGATGGCC
AGCCCGTTCAGTTCACAACGCGCAAGGCGGCACTTCTCTTTGCCGCTCTGGTCCTGGCAGGTCGCCGCGGCCA

GCACCGCGAGCTACTTTCCGAAGGGTTTTGGCCGGGACGAGGCGATGCACAGGCACGTAACAGCTTGCGCCAA
GCCCTTGCCGAGATTAGGCGGTGGTTTCCTCCCGGCCGCAATGCCGACATTTCCGTCGAAAGCAATCAGGAAA
CCGTTGCACTGGTGGTTCCTCCAGAGAAGGTGGACATTTGGCTCTTTGAGAGAAAGCTCGCTGAAGGTGAAAC
CGCGGACCTGGCCTTCGCTGCTGACCTCTATCGCGGTGATCTTCTAGCGGGCGTGGCCGTCCCGGACGAGCTG
GAGGAAAGGTTCCGACCATATCGGAGAAGATATCGGGAGAGAGCACTGCAGTTAGTGGAACGAATGAGCCTTG
CCCTGTCCGATGCTGGATCCGCGGCCGAAGCGGCTTGCGAGCGGCTTGCGGAAACGCTGCTCGCCTCGGACCC
GACTGCCGAGCCGGCTCACCGGGCGTTAATGCGCATCCATGCTGTGAGGGGTCATGAAAACGCGGCCTTGCGG
CAGTTTGAGTCCTGCCGGGCGCTGCTGATGAAGCACCTGGAGGTGGAACCGGAGGCGCAGACGAACTCTCTGG
CGGCGTCGCTGCAATTGCGGCAGGGACAGGGACACCGGCAGACCGGGTCGACCTACAATTTCGCACCGCATAC
GCAGCCCATCCGGGCTCCCACGAGACCGCATGACCAGCCGTCGGTGGCGGTGATGCCCTTCCAGAATCTTGGT
GCGGCGGACGACGAGTATTTTGCGGACGGCGTGGTGGAGGAGATCACTGCCGCGCTATCGCGCGTTCGCGACT
TCTTTGTGATCGCCCGCCAGTCTGCCTTCACCTACAAGGGACGCTTCGTCGACGTGCGTGAGGTCGGCAGGGA
GCTTGGCGTCAGTTATGTGGTCGAGGGCACCGTTCGGAGGGGCGGCGACCGACTGCGCATTTCCGTACAACTG
ATCGATGCCGAGACGCGGACACAATTGTGGTCCGAGCGCTACGAGGGCGCGGTCGAAGACATTTTTGCATTCC
AGGACAGGATCGCGGCGCAGGTGGCCGGGGCTATCCATCCAGCCATCCGGGGCGCTGAGATCGAGTTGGCCAA
GCGCAAGCCGCCAGCTAGCCTGCGCGCCTACGACCTCGTCATGCGAGCCTACCCGCACCTTTGGGGCCGCCGC
AAGGAGGCTAACGACGAGGCTATCGAGCTACTGCGGAAAACGATCGCCATCGATCCGAGCTATGGCCGCGCGC
ATGCACTTTTGGCTTGGTGTCACGCCGCAAACGCCGCATATCTATGGACGAAGCAACCAGAACTCGAGTTGGA
AAAGGCGCTAAAAGCCGTTGAGGCTGCGGCTTCGATCAGCAACGATCCAACCGCGTTGACTGCCGCCGGTGCG
GCAATGAGCATTTGCGGCGACCAGGAGCGCGCCAGCATTTTCATCGAAAAGGCGCTCGCGCTTGATCCAAACA
ACGCATGGGCATGGACGCGCCTGGGCTGGATCGCGATTTACAGGGATGACGCCGTGCGCGCAACGGAATGGTT
CGAGCGAGCGATGATGCTTAGTCCGATGGATCCCCTCACATTCAACATGAGAATAGGATTGGCCGCATCTATG
GCCGTGGCCGGTTCTCTTTCCCAGGCGGTCGCTATCGCCCGTGAACTCATCGCCAGTTATCCGAACGTCATAA
TGTTGTATCGTTTCCTTGCCACTTGGTCGGCGATGAATGGGGATCTTGAGACTGCTCGCTGGGCCGCTCGGAA
GCTGCTGGCAGCCCAGCCAGACTTCACGATCGAGAAGTTCCGCTCACTCCCTATGTTTCGAGACATCCCCAAA
TGGGCAGACAAGGTGTCCGAAGCCTTGAGGTTGGCGGGAGTGCCCGATCACTGAATGTCGCATGAAAAGTTCC
TGTCCGCGCAGATCGGCCGCCGGACGCTGCTTGCGTCCATGGCCGCGGCCGGCGCTTCGGCGGGTCTTTCGAC
CCTCGGCGTCAGCCGCGCCTTTGCCCAGGAGCCGGAAAAGCCGGCCGAGATCATCGTTCGCGCCTGGGGCGGC
AGCTGGGTCGATGCGCTCAAGGCTGGCGTGTCCGACAGCTTTACCAAGATGACCGGGATTGCCGTCCGTCACG
ACCTGACCGAGGACAACGAGATCCAGCCGAAGGTCTGGGCCGCCGTTGCCCAGAAGCGCGTCCCGCCGATCCA
CATCAACTGGGATACGACGACGAACGCCACCAAGTCCGCTTTGCGCGGCGTGACCGAAGACCTGTCCGACCTG
CCGAACCTCAAGAACGCCACGGATCTCGCAAAGCCGGTCGGCCTCGACGGCTATCCGATCGTCAACACCTACG
GTTATGTCTACGTGCTCGCCTATCGCCCTTCGGCCTTCCCGAACGGCGCGCCAAAATCCTGGAAGGATCTGCT
CGACCCCAAGCTCAAGGGCCGGATCGCATTGTATAACGACGGGATCGGCTTCCACTTCCCCGCACAAGTCGCC
GGCGGCGGTAAACTCGAGGACATTCCCGCCAACATGCAGCCGGCCTGGGATTTCATCTCCAAGATCAAGGAGC
AACAGCCGCTGCTCGGCGAGGACCCGGATTTTACCACCTGGTTCCAGAAGGGCGAGATCGACGCCGCCTGCAC
CATCTCGACCAATGCGCGCGAAGCCAAGAAGAACGGCATCGAGATCGCCTGGGTCGTGCCGGAGGAAGGCGCC
AAGTTCGACACCGACGGCCTTTGGATACCGAAGGGTCTGCCCGAGAACGAGCTTTACTGGGCCAAGCAATATA
TCAACCACGCCCTGACCAAGGAAGCGCAGCAGATCTGGCTGGACGGTCTCGGCTTGCCCGGCGTCATCCCCGG
CATCACGCCGCCCGCCGATCTCGTGGGCGATCCCTCCTATCCAACAACGGAGGAGGACTTCAAGCAGCTGATC
CGTATCTCGGCCAAGGTACAGGTCGAGAACGAGAGCCAGTGGTTCGCCAAGTTCAAGGAAATCATGCAGGGCT
GAATGCCGCTACATAGTAGGAAAAAGATGATCGGCCTGGCGCTGTGCCTGGGGGCGGTCGTCGTCGTCGTTGC
CGGGTGGACCTGGGCCCGCGAGAGCGGTGCGGCGTCGACCGACAACGCCTATGTCCGCGGGGATGTTACCTCG
CTTGCCCCAAAGGTCGCAGGCTACGTCACGGCTATCGAAGTCGAGGACAACCAAGCGGTACGGGCGGGCGACG
TGCTCTTCCGGATCGACGATCGGGACTACCGCGCAAAGCTTGCGCAAGCGGTGGCCAATGTCGAAGCGGCAGA
GGCACGGCTGACCAACGTCGATGCGGAGATGGCGCTTCAGCATGCCCTGATCCGCCAGGCCGAAGCGCAGCGA
CGTTCGGTCGTGGCCGAGTTGAACCTGGCGGCCAAGGCCTCTGACCGCCGCCGCGAGCTTATCCGCAGCAACA
CGATCAGCCAGGCCCATGTCGACGAAAGCGACGCGGCGAAATCGAGGGCCGAGGCGAACGTGTTGGCGGCTTC
CGCAACGGTGGAGGCGCAGCAGCAGCGCATCGCCGTGCTTGCCGCGCAGCGCGAAGCCGCCGTCGCTGCGGTG
GCACAGGCGGAGGCCGCGCGCGACCTCGCCGGGATTGATCTGGAGAGCACCGTGGTGCGCGCCCCCGTCGGCG
GCGTCATCGGCAACCGGCAGGTGCGCGTCGGCCGGCTCGTTGCGCCGGGCGCTTCCCTGCTCGATATCGTTCC
GCTCGACAATGTATGGATCGTCGCGAATGTCAAGGAAACCCAGCTCGAACATATCCGGCCCGGTCAGCGCGCC
AGCATCACCATCGATGGTTACCCGAGGGGAGCGCTTGAAGGCGTGGTGGACAGTTTTTCACCCGGCAGCGGCT
CTGCCTTCAGCCTGCTGCCCGCAGACAACGCCACGGGCAACTTCGTCCGCGTCGTCCAGCGCGTTCCGGTGAA
GATCCGGTTCGCCGGCAATCCGCTGTCGGGCCGCCTCCTGCCGGGCCTGTCAGCGCGTGTCGAGATCGATCTG
GAGAGCGGTTCATGAATGCTGCGGGAGGATGTGAAGGATCTGCTGTGGTTCATCGAGGTGGCGAGGGAGCAGA
GCTTCACCAAGGCCGCGGCCACGCTCGGCACCTCTCAGTCGACCCTCAGCCATACGATCAAGCAGTTGGAGGC
GCGGCTGGGAGTACGCCTGCTGACGCGGACGACCCGCAGCGTGGCGACAACGGAGGCTGGCGAGCGCCTGCTA

CAGTCGCTCGCTCCAAGATTTGAGGGGATCGAGGCAGATCTTGCAGATCTGGTGGCTTTCCGGGAGAAACCGT
CGGGCACAGTGCGCCTGACGCTCTCTGATCATGCTCTCCAGACGACGGTCTGGCCTAAGCTGAGCCGTGTCCT
GTCCGACAATCCCGACGTCAAGGTCGAGCTCTACAGCGACAACGGGATGCGCAACATCGTCGAGGAGCGTTTC
GACGCCGGCGTCCGGCTCGGCGAGAGCGTCGACAAGGACATGATCGCCGTGCGCATCGGCCCCGACTGGAGGC
TGCGGGCCGTCGCCTCCCCGGAATACCTCGCCAGGCACGGGATCCCCAAGACACCGCAGGACCTCGTCAGCCA
TGTCTGCATCAACACGCGACAGGCAACATGGGGCGGCTTCTATGCCTGGGAATTCGAGAAGGACGGGCGGGAG
CTTCGGGTCCGGGTCGACGGGCAGCTCAGCTTCAACTCCTCGATCTCCCAGATCGACGCCGCCGTTAAAGGAT
ACGGGATCGCCTATATACCGGAGGATCTCCTGGAAGGACATATCCAGGCGGGAGCGCTGCAGGCTGTCCTCGA
CGATTGGAGCGAGCCGTTCACGGGTTACCACCTTTACTATCCCAGTCGACGTCAGATGTCCCCCGCGATGGCA
CTGATCGTAGATGCTCTGAGACATCGGAGCTGAATGCCCAAGGAAGACTTCAACGATCTGCTGTGGTTTCTCG
CCGTGGCCGAGGAGCGCAGCTTCACCAGGGCCGCTGCCAAGCTCGGCATAACCCAATCGACGCTCAGTCACAC
GATCAAGCGCCTCGAAACGAGGATGGGCCTGAGGCTTCTGACCAGGACCACCCGGAGCGTTGCCGTGACGGAA
GCTGGCGAGCGCCTTCTTCGTTCGCTGGCGCCGCGGATATCGGCCATAAGGGACGAAATCGCGGCCCTTATGG
CCTTTCGCGACAAACCGTCCGGCTCGGTCAAAATCACTTTGTCCGACCACGCCCTGGAGAGCCTCGTCTGGCC
GAAACTACAGCCGGTTCTTCTGGAATACCCCGACATTCGGCTCGAGTTGAGCAGGGACAACGGCTTCAGGAAC
ATCGTGGAAGATGATTTCGACGCAGGCATTCGCCTCGGCGAGAGCGTCGAAAAGGACATGATCGCGGTCCGTA
TCGGACCGGACTGGCGGCTTCTGGCAGTGGCTTCGCCCGACTACCTGGCAGATCGTCCGCTGCCAGAGCATCC
ACAGGAACTGATCCAGCACAATTGCATCAACTCGCGCCAGGCAACAGGCGGCGGACATTATGTATGGGAGTTC
GAGAAGAACGGGCAAGAGCTCCGCGTCAGGGTCGACGGACAACTCACCTTCAATAGCTCATACCCCATGGTCG
ATGCCGCCCTGAAAGGTCACGGCATAGCCTATCTGCCGGAAGATCTCGTCGCGGGGCACATTGCGGCAGGACG
CCTGGTAGCGCTGCTCGAGCCCTGGTCGCCTCCATTTGCCGGGTACTACATTTACTACCCTAGCCGACACCAC
AACTCACCCGCATTCAAGGTGATCGTGGACGCCCTGCGACACCACGGCCCACCAGATTCCGAGGGAACGCGAG
ATGCTTAAATGGGAAACAAATCTGTTCGCGTCATCGCGGGAGCAATGGTGGTCGCCGGCTGGGCCGGCTACGC
CGCTGCGCAAAGTGCCTCGGAAATCAAGATCGTCCTGCCGGAGCAGCCGGCCAATCTCGAGCCCTGCGGTACC
ATCATCACCAATGTCGGCCAGATATTGAGCCGCAACGTGGTCGAGCCGCTGACCATCATCGATCCGAAAAGCG
GCCAGCCGACACCCGGTCTTGCGACCGAGTGGAAGCAGACCGATCCGAACACCTGGCAGCTCAAGCTGCGCGA
AGGCGTCAAGTTCCAGGACGGAGCCGCCTTCGACGCAGAGGCGGTCAAATTCTCCATCGAGCGGATGACCGGC
GGCAAGCTGACCTGCAGCAACATTGCCAAGTTCGGCGATGCCAAGCTGACGGTCACGCCGCTCGATGAGCTGA
CGGTCGAGATCAAGTCGGACAAGCCGCAGCCGATCCTGCCGACGCTGCTCAGCGTGGTCATGATCGTCTCGCC
GAACACGCCGGCGGACAAGGCCGTCAACGACCCGGTCGGTACCGGGCCGTTCAAGCTTTCGAGCTTCACGCCG
CAGACAGTCGTGCTGGAAGCCTTCGACGGCTACTGGGGCGAGAAGCCGGCCATCGCCAAGGCCAGCTATGTCT
GGCGCCCGGAATCCTCGATCCGCGCCGCCATGGTGGAAACCGGCGAGGCCGACCTGACGCCGTCGATCGCGAT
CCAGGATGCGACCAATCCGGAAACGGACTTCGCCTATCTGAACTCCGAAACTACCGCGATCCGCATCGATGCC
GGTTTTGCTCCGCTCGACGACGTCCGAATCCGCAAGGCGCTGAATCTGGCGATCGACTGGGATGGCCTCGCGC
AGCTCTTCGGCGAGGACGTGCAGCGGGCTTCGCAGATGGTCGTCACCGGCATCAACGGCCACGACGACAAGCT
GGCGCCCTGGACGTTCGATGCCGAAAAGGCGCGTGCGCTCATCGCCGAGGCCAAGGCCGCGGGCGTTCCGGTC
GATACCGAAATCGAGCTGATCGGCCGCAACGGCATCTACCCCAACGGTACGGAAGCCATGGAAGCCATGATGG
CCATGTGGCAGGATGTCGGCCTGAACGTGAAGCTGACGATGCTCGACGTGAATGATTGGCTGCGCTACCTGCA
GAAGCCCTTCCCGGAGAGCCGCGGGCCGAACCTGCTGCAGATGATGCATGACAACAACAAGGGTGATGCCGCC
TTCACCATTCCGATCTTCTACACCTCGGCCGGAAGCTATTCGACCTTCAGCGACGCGGCACTCGACAAGGAGG
TCGCCGATGCCATGGCTGCCACCGGTGAGGACCGTACGGCAAAGTTCAAGGCGATCTTCGCGAAGGTGCATGA
CGATCTCGCGGTCGACATCCCGATGTTCCACATGATCGGCTACACCCGGGTCGGCAGCCGCCTGGAGTGGAAG
CCGGACATCACGACCAACAGCGAGATCCCGCTCGCCAATATCGCTCTCAAGGACTGAATGTCGACATTCATCA
AGAACTTCATCGAGCGTGAGACGATCAGCCGTCGCCACTTTCTGCTTGCCTCGGCCGCAGGTCTGGGGGCAGC
GGTCCTGCCAAACCCGCTTGGCGGCAGCGCGCGCGCTGCATTGACGGATCCCGCGATCGCCTGGAGCTATCGC
GACCGCGCTTCGGCCTATTGGAACTCGGTCGTCTCCGGCGGCGAAGCATTCGTCGAATCGCTCGGCAAGCCGA
AGAGCGCGCTCGTAAGTCTGATCAACGAAGGATCATCCGAGAAATCTCTGGCCGACATCAAGGCCTTCCTTGC
CAAGAACAATGGAAATTGCGCCCTGGCCTGCGATGCCAATGACAGCCCGAATGCGCGGCCGATCGTGGAGGCG
GTGGCGGCTGCGGGCGGCTACATCTCCACGATCTGGAACAAGACCGATGATCTCCATCCATGGGATTTCGGCG
ACAACTACGTGTCGCACATGACCTGGTCGGACGAGAAGCCCGCCGAAGAAACCGCGCGCATCCTGTTCGAAGC
CATGGGCGGCGAGGGCGGTGTCGTCCACCTGGGCGGCATTGCCGCCAACAACCCTGCGGTCGAGCGGCTCAAC
GGTCTGAAGAATGCGCTGAAGGACTTTCCCAACATCGAACTGCTCGACGCGCAGCCGGCGGACTGGGACACGC
AGAAGGGCGCGGCTTTGATGTCCTCCTTCCTGACCCGCTACGGCGACAGGATCAAGGGCGTGCATTGCGCCAA
CGACAACATCGCCTACGGCGTCATCGAGGCGCTGCGCGCCGAAGGCATCGAGGGCATGCCGATCGTGTCCTAT
GACGGCAATCCCGAAGCGGTGCAGATGGTCATGGACGGCCAGCTGCTCGCGACGGTCTTCACCAATCCGCACT
GGGGCGGTGGCATCACGTCGGCGCTCGCCTACCATGCGGCAACCGGCAGCTTCAAGCCGTCGGAAGAGCCGAA
AGAACATCGCGAGTTCTACGGACCGACCGTCCTGGTCAGCAAGAAGGATGCCGAGGAGTTCAAGGCCAAATAT
CTGGATTCGGTACCGAAATACGATTGGGCCGACTTCTGGGGTCCCTCGAACGGACAAATTCAGTATCGGTCCT

GAATGAAGCGCATCTACGTCGTTGGCACGGCCGACACGAAGGGGGAGGAACTCGTTTATCTTGCGTCCTGCGT
TGAGGCAGCGGGCGGCCGGCCCGTTTTGGTCGATGTCGGCACGCGTCGCCCCACGGTTCTGGTCGACATCTCC
GCCGAAACCGTGGCCGCGGTGCATCCGGGCGGCGCCGCCGCGGTGCTTTCCGGCAACGATCGGGGAACAGCGA
TCGCTGCCATGGGCGAGGCCTTCGCAAGGTTCCTGCCCGCCCGGGACGACGTCGCCGGCGTGGTGGGCATGGG
TGGCGGCGGCGGGACGTCGATTATCACCGCCGGCATGCGCAGGCTGCCGCTCGGGCTACCGAAGGTCATGGTC
TCGACGCTCGCGTCGGGCGACGTGGGACCTTATGTTGACGTCTCAGACATCATCATGATGCCGTCGGTTACCG
ACATGGCCGGGCTCAACCGCGTGAGCCGCGTGATACTAAAAAACGCGGCCGAGGCGATTACCGCCATGGCGAA
CAGGCCGGCGGAGGAAACAGCCTCGAAGCCCGCTATCGGCCTGACCATGTTTGGCGTGACGACGCCCTGTGTG
ACTGCCATCGTTGAACGACTGAAGGCCGACCATGACTGTTTGGTTTTCCACGCCACAGGCACCGGCGGACGAG
CAATGGAGAAGCTTGCAGACAGCGGGCTTCTATCGGGTGTGCTCGATATCACGACAACGGAGGTGTGCGATCT
TGTCTTCGGCGGCGTGCTGCCGGCCACCGAGGATCGTTTCGGCGCAATTGCGCGCACCGATTTGCCTTATGTC
GGCTCCGTCGGGGCTTTGGATATGGTCAATTTCTGGGCACCAGAGACAGTACCGGAGCGATATTCCGGCCGGC
TCTTGTATCGCCACAACCCCAACGTCACGCTCATGCGCACGACTCCGGAGGAATGCGCCGCGATCGGAAGGTG
GATTGGCGCAAAGCTGAACCTCTGCAGCGGACCTCTCCGGTTCCTGATCCCGGAGCGAGGCGTGTCGGCGCTG
GATATCGAGGGCGGCGCCTTCTTCGATCCTGCGGCGGATGCGGCGCTCTTCGAAGCATTGGAGACCACCGTCA
ATAGAAGTGACCGGCGCCGCATCGAACGCCTTCCGCTCCACATCAACGATCCGCAGTTCGCCGAGGCCGCAGT
CGCCGCCTATCGAGACATTGCCAACCCCTGAATGAAATATCAAACCCAGAAGGTCGCGATGCTGTATTTCTAC
GGTGCGCTCGGCCTGTTTCTCGCCCAGATCCTCTTTGGCGTGCTTGCCGGCACCATCTACGTGCTGCCCAACA
CGCTCTCCGAACTGCTGCCGTTCAACATCGTCCGCATGGTGCACACCAACGCGCTGATCGTCTGGCTGCTGAT
GGGTTTCATGGGTGCCACCTATTACCTCTTGCCGGAAGAGGCGGAGACGGAGCTCTACAGCCCGAAGATCGCC
ATCGCCCAGTTTTGGATCTTCCTGATCGCGGCGGCCGTTGCCGTCGTCGGCTACCTCCTCCACATCCACGAAG
GCCGGGAATTCCTCGAGCAGCCTTTCGCGATCAAGGTCGGCATCGTCGTCGTCGCGCTGATGTTTCTCTTCAA
CATCACCCTGACCGTACTGAAGGGCCGCAAGACAACGGTGACCAACATCCTGATCTTCGGCCTTTGGGGCGTG
GCGATCTTCTTCCTGTTCGCCTTCTACAACCCGGTCAATCTCGCGCTCGACAAGATGTACTGGTGGTATGTCG
TGCATCTCTGGGTCGAAGGGGTCTGGGAGCTGATCATGGCTTCCGTCCTGGCCTTCTTGATGATCAAGCTCAA
TGGCATCGACCGCGAGGTCGTCGAAAAGTGGCTCTATGTCATCGTCGGCCTGGCGCTCTTCTCAGGCATCCTC
GGCACCGGCCACCACTACTACTGGATCGGCGCGCCCGGCTACTGGCAATGGATCGGCTCGCTCTTCTCCACGC
TCGAGGTCGCACCCTTCTTCACCATGGTGGTCTTCACCTTCGTCATGACCTGGAAGGCCGGCCGCAAGCATCC
GAACAAGGCCGCGCTCCTCTGGTCGATCGGCTGCTCGGTGATGGCCTTCTTCGGCGCCGGCGTCTGGGGCTTC
CTGCATACGCTTTCGTCGGTCAACTACTACACCCATGGCACCCAGGTCACCGCCGCGCACGGGCATCTCGCCT
TCTTCGGCGCATACGTCATGCTGAATCTGGCTGTCATGGCCTATGCGATACCGGAGATTCGGGGGCGAGCGCC
CTATAACCAGTGGCTGTCGATCGCCAGCTTCTGGGCCATGTGCAGCGCCATGTCCGTCATGACCTTCGCGCTC
ACCTTCGCCGGTGTCGTTCAGGTCCACCTGCAGCGCGTCCTCGGCGAGAGCTTCATGGCCGTGCAGGAGCAAC
TGGCTCTCTTCTACTGGATCCGCCTCGGGTCCGGTGTCGTCGTACTCATCTCGGCGCTGATGTTCTTCTGGGC
AGTCCTCGTACCCGGCAAGAGCGCTAGCCCGGCCCTCAACCAAGCCGTCCAACCCGCCGAATGAATGACCGGC
GAACTGACGCGGCGTGAAATGTTGAAGGCGCATGCGGCCGGCATCGCCGCAGCTACGGCCGGGATCGCGCTTC
CGGCGGCGGCGCAGCCCGTGCCGGGCGGCGTCGAAGCACTTCAGATAACGTGGTCGAAGGCGCCCTGCCGTTT
CTGCGGTACCGGCTGCGGCGTCATGGTCGGGGTCAAGGAGGGACAGGTCGTCGCCACACATGGCGACATGCAG
GCTGAGGTGAACCGCGGCCTCAACTGCATCAAGGGCTACTTCCTGTCCAAAATCATGTACGGCACCGACCGTC
TGAAGACGCCACTTCTGCGCAAGCGCAACGGCGCCTTTGCCAAGGACGGCGAGTTCGAGCCGGTGAGCTGGGA
CGAGGCCTTCGACGTCATGGCGGAGCAGGCGAAGAAGGTGCTGAAGGACAAGGGCCCGACCGCCGTCGGCATG
TTCGGCTCTGGGCAGTGGACCATCTTCGAGGGTTACGCGGCCACGAAGCTTATGCGCGCCGGGTTCCGCTCCA
ACAATCTCGATCCCAATGCCCGCCACTGCATGGCCTCGGCAGCCTATGCTTTCATGCGCACCTTTGGCATGGA
CGAGCCGATGGGCTGCTACGACGACTTCGAGCACGCGGACGCGTTCGTGCTTTGGGGCTCGAACATGGCGGAG
ATGCACCCGATCCTGTGGACGCGCCTCGCCGACCGTCGGCTCGGGCACGAGCATGTGAAGGTCGCGGTGCTGT
CGACCTTCACCCACCGCAGCATGGATCTTGCGGATATTCCGATCGTCTTCAAACCGGGGACCGATCTGGCGAT
CCTCAACTACATCGCCAATCACATTATTCAGACCGGCCGAGTCAATGAGGATTTCGTCAGGAAGCATACGACC
TTCATGGTCGGCGCGACCGACATTGGCTATGGCCTGAGGCCCGACAATCCGCTCGAGGTCAAGGCCGTGAACG
CCAAGGATGCGGCGAAAATGACGCCGAGTGACTTCGAGAGCTTCAAATCCTTCGTCTCGGAGTACACGCTCGA
CAAGGTGGTCGAACTCACGGGGGTCGAAGCCGGGTTCCTAGAACAATTGGCCGACCTTTATGCCGATCCGAAG
CGCAAGGTCATGTCGCTCTGGACTATGGGGTTCAACCAGCATGTGCGCGGTGTTTGGGTCAACCAGATGGTGT
ACAATCTCCATCTCCTGACCGGGAAGATCTCCGAGCCCGGCAACAGCCCGTTCTCGCTAACCGGTCAACCTTC
CGCCTGCGGCACGGCACGCGAGGTAGGCACCTTCGCGCACCGCCTGCCAGCCGACATGACCGTGACCAATCCG
GAGCACCGCAAGCATGCCGAGGAGATCTGGCGCATTCCCCACGGCATCATTCCTGAGAAGCCCGGATACCATG
CCGTCGAGCAGGACCGAATGCTGAAGGACGGCAAGCTCAATTTCTATTGGGTGCAGGTCAACAACAACGTCCA
AGCGGCTCCCAATACGCAGAACGAGACTTATCAGGGCTACCGCAATCCGGACAATTTTATCGTCGTCTCCGAC
GTCTATCCGACGATCACGGCGATGAGCGCCGACCTCATCCTGCCGGCTGCCATGTGGGTGGAGAAGGAGGGCG
CCTACGGCAATGCCGAGCGTCGTACCCATGTCTGGCACCAGCTCGTCGATGCGCCCGGCGAGGCGCGTTCCGA

TCTCTGGCAGATGGTGGAGTTTTCCAAGCGCTTCACCACCGACGAGGTCTGGTCGACCGACATCCTCGATGCC
AATCCCGGCTATCGTGGCAAGACCCTTTACGACGTGCTCTTCAAGAACGGCAATGTCGACAGCTTCCCGGCAA
GCGAGATCAACAAGGAATACGCCAACCGGGAGGCCGAGGCCTTCGGCTTTTACATCCAGAAAGGCCTGTTCGA
GGAATATGCCTCCTTTGGGCGTGGACACGGGCATGACCTGGCGCCCTATGACCGCTATCACGACGAACGGGGA
CTCCGCTGGCCTGTCGTCGACGGCAAGGAAACGCTCTGGCGCTACCGGGAAGGCTACGATCCCTACGTGAAGC
CGGGGGAGGGCGTCAAGTTCTATGGTCGGCCGGACGGCAAGGCGGTGATCCTCGCCGTTCCTTACGAGCCGCC
GGCGGAATCTCCCGACGACGAATACAATGTGTGGCTGGTGACGGGCCGCGTCCTTGAGCACTGGCACTCCGGC
TCGATGACGATGCGGGTGCCGGAACTCTACAAGGCGTTCCCCGGTGCCGTCTGCTTTATGAATGCCGGTGACG
CCCGCGATCGCGGCATCAACCAGGGGGCCGAGGTCAGGATCGTTTCCCGCCGCGGCGAGATTCGCGCCCGCGT
CGAGACGCGCGGCCGCAACCGCATGCCGCCGGGCGTCATCTTCGTACCCTGGTTCGATGCCAGCAGGCTGATC
AACAAGGTGACACTCGACGCAACCGATCCCATCTCCAAACAGACGGATTTCAAAAAATGCGCGGTCAAAATCG
TCTCTGTCGCATGAATGCTCCACCAACCCAAAACCAAAGCTACAGTCGGACGGCTCGATGCCGAAACCGTCAA
TGCCGCCCGCGTGCGAGGTCCGCTTTACGAGAAGCGTCGGAAGATCTTCCCGAAGCGGGCCGAGGGTCGTTTC
CGCCGGTTCAAATGGCTGGTGATGCTGGTGACGCTCGGCATCTACTATCTGACGCCGTGGATCCGCTGGGACC
GCGGGGCGCATGCGCCGGATCAGGCAGTCCTTATCGACCTTGCTTCCCGGCGCTTCTATTTCTTCTTCATAGA
GATTTGGCCGCAGGAGTTCTTCTTCGTCGCGGGGCTCCTGGTGATGGCGGGCTTCGGGCTTTTCCTCGTGACG
TCGGCAGTTGGGCGGGCCTGGTGCGGCTACGCCTGCCCGCAGACCGTCTGGGTCGATCTCTTCCTCGTCGTTG
AGCGCTTCATCGAGGGCGACCGCAACGCCCGCATGCGCCTCGACGCCGGACCGTGGAGCCTCGACAAGATACG
CAAGCGCGTGGCCAAGCATGCCATTTGGGTGGCGATCGGCGTTGCAACCGGCGGCGCGTGGATATTCTATTTC
GCCGATGCGCCCTCGCTCATGTCGAGCCTAGTCGCGCTCGATGCGCCGCCGGTCGCCTATACCACGATCGGCA
TTCTGACCGCGACCACCTACGTCTTCGGCGGACTGATGCGAGAGCAGGTCTGCACCTATATGTGCCCATGGCC
GCGCATCCAGGCGGCCATGCTAGACGAGAACTCGCTCGTCGTAACCTACAACGACTGGCGGGGCGAGCCGCGC
TCGCGGCATGCGAAGAAGGCGGCTGCGGCCGGCGAGGTGGTCGGGGATTGCGTCGACTGCAACGCCTGCGTTG
CCGTCTGTCCCATGGGCATCGACATCCGCGACGGCCAGCAGCTCGAATGCATCACCTGCGCGCTCTGCATCGA
CGCCTGCGACGGTGTGATGGACAAGCTCGGCCGCGAGCGCGGGCTGATCTCATACGCGACGCTCAGCGACTAT
GCAGCCAATATGGCCCTCGCGACGAGTGGCGGGACTGCGGCGATCGATCCGAGCCGCGTGCGCGACGCCCATG
GCGCTTTCCGTGACAAGGTGAGGCATCTCAACTGGCGCATCGTCTTCCGGCCCCGCGTTCTCGTTTATTTCGG
CGTCTGGGCGACGGTCGGATTCGGTCTTCTTTTCGGCCTGCTGGCGCGCGACCGGCTAGAACTGAACGTCCTG
CACGACCGCAACCCCCAGTTCGTCGTCGAATCCGACGGCTCGGTGCGCAACGGCTACATGGTCAAGCTTCTCA
ACATGATCCCAGAACAGCGCACCATTAGCCTGACGATCGAGGGTATGCCCGCCGCAACCATGCGCGTGGCCGG
ACAGGCGACGGGCGACGGACGCCGGGTCACGATCGGTGTCGAGCCGGACAAGGTCACACCGCTCAAAGTCTTC
GTCACGCTGCCGAAGGGCAGATTTGCCGAGGCGGAAGAGGGCTTCTCCCTCATTGCGGAGGATCCGTCCAGCC
ACGAACGCGATGTGTATCAGGCCAATTTCAATCTACCGGGAGCTGCAGGACGATGAATGGCTCGATTGACCAT
AAACGGCCGAACAAGGGATATCGAAGTGGATCCGGAGACGCCACTTCTCTGGGTGCTACGTGAACAGGCGGGC
CTCACCGGCACGAAATTCGGCTGCGGGGTTGCGCAATGTGGCGCCTGCACCGTGCTTATCGACGGCGTGGCGA
CGCGTTCCTGCACCATGCCGGCAAGCGCGATCGCGCAGGATCAACAGATCGTCACGATCGAGGGCCTGTCCGA
GGACGGCTCTCATCCGGTCCAGCAAGCCTGGCTTGCGCTCGACGTTCCGCAATGCGGCTACTGTCAGGCCGGC
ATGATCATGGCGGCTGCCGCCCTGCTCAACAGCACACCAACCCCAAGCGACGACGATATCAGCGCGGAAATCA
CCAATATCTGCCGCTGTGGCACCTATAACCGCGTCCGTGCGGCGATCAAGCTCGCGGCGGAAACCATGAGCAC
GTCCCAGAAAGGATGAATGCAATCGACGCCTCACATTGCCGTTGTGGATGATCATCGCGATATTCGCGACCTG
GTAGGAAAATATCTGACACAACAGGGTTACCGCGTTTCCCTCGCGGAAAGCGGCGAAGCGCTTCGACGTCTTC
TGGAAAAAGGCGCCCCAGATCTCGTCGTGCTGGACATCATGATGCCCGGCGAGGATGGATTGACCGTTTGCCG
TCAGTTGCGCACGACAACGGAGCTTCCGGTCATCTTTCTGACAGCGATGGCGGACGGGACGGACCGGATCGTC
GGCCTGGAACTCGGCGCCGACGATTACATCACCAAGCCGTTCAACCCGCGCGAACTCCTGGCAAGAATCCGGG
CGGTGCTGCGGCGTGTCAACAGCCTTCCTCCCCGGCGCGGGAGGATCAAGGCCAAGACCATCCGCTTCGATAT
CTGGGAACTCGACACTGGACGCCGGGAGCTCGTCGGACGCGATGGCGTCAGCGTGGCGTTGAGCACGGCGGAG
TTCCGGCTGCTCAATGCCTTCCTGGAACACCCCGGTATGGTCTTGAGCCGTGACCAGCTACTTGATCTGACCG
CAGGGCGAGCCGCCGAGAGCTTCGATCGCAGCATTGACAACCAGGTCAGTCGGTTGCGCAAGAAGATCGAGAG
CGACCCGAAAAATCCATCTTTGATCAAGACGCACTGGGGTGGAGGATACAGCTTCGTGGCGGAGGTAGAAGCC
TACTGAATGGCCACCATCACCCATGTCTTCACCATCAACCATGTCGCCGAAATGCTCGGCGAAGACCGGGAAC
TCCTGGAGGCGATCGTCAGCAATTCCGACAATTTGACCTATGGAAGTATCATCAGCGTCGTACACGGTGACGA
TGAGACAATCACCGCACTGACCGACGATGGCATGGATGAGCTGAGGCAAATGCTTGCCGATGCGCGCCGTTCG
CCTGAAGCCTGGAACGACTTCCTCGACTGCTTCGTCGACGACGAAGAAGTCATCGCTCGCGTCAAGGCGAAAT
CTCCGCGGTAAATGACAGAAACGGCCGCTTCGTCAATAGATAAGCAAGCTCATCTGTTCACCATATCTAATGC
GAGCGGCGGTGAGTACGTTGAGGGTTGGCGCGTCACAGTTCTGCTTGTTACGCTATTTCTTGTCAGCGCCACT
TCCCAGATCGACCGGACGCTTCCCTTCGTCCTCGCCGAGTCGATAAAGGAAGAGCTCGATCTGAGCGATACCG
AAATGGGTTTGCTCACCGGCATCGCCTTTGCGGTATGTTACACGCTGCTGTCGATGCCGCTCGCCCGCTCCGC
TGATCGAGGATCGCCCCGGTTCGTCCTCGTTTTGTGTATCCTATTCTGGAGCGCGATGACATTGCTCGGCGGT

CTCGCCATGAGTTTCCTGTTCCTCGCCTTCACCCGCTTCGGCGTCGCGTTCGGCGAGGCCGGTGGGACGCCCT
CCAGCCACGCGATCATCGCACGCAAGATCCCGCCCGAGCGCCGCGGGCTGGCGCTCGGCATTTTTTCCATGGG
AATTCCACTCGGCACCATGGTGGGTTTCGCTGTTGGCGGCGCTATCGGCGATACGCTCGGCTGGCGCACAGCG
CTGATAGGCGCAGGCGCCATTGGCGTGCTGATCGGTCTGCTGGCGTTCGTCGTCGTCGGTCCGACCCCACCAC
GCAAGAGCTCGGCCGCCAGTAGCGAACCTTTCCTGCGGTCGAGCTTGCAACTGCTTTCGTCCCCGTCATTTCG
CTGGCTGTTCATCGGCTCGGTCGCACTCGGCTTCGCCGCAGCGCCGTTCTACGCTTTTGCAGTTCCCTTCCTA
ATTCGCACTCATGACTACAGCGCCAGCGAGGCAGGACTTGCGTTCGGGCTGCTGCAAGGCCTGATGGGGATTT
GCGGTACGCTGATCGGCGGACGCCTGTTCGACCGCGCCGTGCGCTCCGGCACCGGCCGGGTCCTCGGACCGCC
CGCTATCCTCTTCATGATCGCAAGCCTGACGACGACAGCTGCGCTGTTCGCGCCGGTCGGGTGGCTTTCCATC
CTACTCTTCGTGCCCGGCATGCTGTCCTTCGCATTTCTGCTGCCCTGGTGTTTCGGCGCCGCGCATCTCGTGG
CGGGCAAGGGCAAGGAAGCGATGGCGACGAGCCTTGCGATGATCGGCTCCGGGCTCCTGGGGCCCGCTCTCGG
TCCTCTCATCGTCGGCATGATCAGCGACGCGGCGACCGCGGCACAGGTCTCCAATGGCTTGGGGCTCGGTTTG
GTGACGGTCCCGATCGCAAGCATCCTGACCGGGATCGCCATGCTGATTGCAAACCAGCGCATTGCTGCGGTCC
TTCGAAAACACGACGTCTCTTAAATGACCAAGTGGACGAGCAAGAACATTCCATCGCAGCATGGCCGTACAGC
GGTTGTCACCGGCACCGGAGGATTGGGCTACGAGGGCGCGCTGGCCTTGGCCCGAGCCGGTGCAAGCGTCGTG
ATCGCCGGGCGCAATCCCCAGAAGGGGGCTGAAGCGGTGGCGGCGATCAGACAAGCCGTGCCGGGCGGACAAG
TGCGGTTTGGCAAGTTGGATCTCGCCAGCCTCGCGTCGATCGCTGAGTTCGCCGCGCAGCTTGCACGGGACCA
GGAGGGTCTCGATTTCCTGATCAACAATGCGGGCGTCATGACGCCGCCGGAGCGGCGCCAGACGACCGACGGC
TTCGAATTGCAGTTCGGCACCAACTATCTCGGCCATTTCGCCCTGACCGGACATTTGCTGCCATTGCTGAAGA
AGGGGCGAAATGCGCGCGTCGTCACCCTCGGCAGCGTGGCGGCGAGGAGTGGAGCGATCAATTTCGATGACCT
TCAAGCTCAGCGCGGCTACAAGCCGATGCCGGTTTACAGCCAATCGAAGCTCGCCTGCGTCATGTTCGCGTTC
GAATTGAGCCGGCGCAGCAAGGCGGCCGGGTGGGGCGTCGAGAGCATAGCCGCGCATCCCGGTATCTCGCGCA
CCGATCTGCTTCCGAACGGCGCGGGGCAGACGAGTGCTGCCGGGATGACACGCCGCTTCTTACCGTTTCTGTT
CCAGCCCGCGTGGCAGGGTGCGCTGCCGACGCTGTATGCGGCGACCGATCCGGCGGCGCGCGACGGCGCCTAC
TATGGACCTGACAGGCTGGGCGGGACGCGTGGCTATCCGACCGAGGAGCAGCCTCCCAAACAGGCGCTCGATA
CTTCAGTGGCCGCGCGCCTCTGGGACGCGTCTCAGGAACTGACGAGCGTGACGTTCGTGTGAATGCCTAAGAT
GAAAGCCGCGATCTTCGTCGCGCCTGGTCGTATTGTACTTGACGAGAAGCAGATCCCCGACGTCGGTCCGCTC
GACGCGCTCGTGCGGATCACGACCACCACAATATGCGGCACCGACATCCACATCTTAAGGGGTGAGTATCCGG
TCGCACGCGGCCTGACGATAGGGCACGAGCCGGTGGGCGTGATTGAGAAGCTCGGGTCCGCCGTGCGGGGATA
CAGCGAGGGTCAGCGTGTGATCGCCGGGGCGATCTGCACCTCGGGCCACAGTAATGCGGCCCTGTGCGGCTGC
CATGCCCAGGACGGCCCCGGGACGAACCACGGTTGGAAAGGCATGGGCGGCTGGAAGTTTGGCAACACCATTG
ACGGTTCGCAGGCCGAGTACGTCCTCGTGCCTGACGCCATGGCCAACCTGGCTCCAGTGCCGGACGGACTAGC
CGACGAGCAGGTGCTGATGTGCCCCGATATCATGTCCACGGGCTTCTCCGGGGCGGAGAGCGGTGCCGTCCGC
TTTGGCGACGCCGTGGCGGTGTTCGCGCAGGGGCCCATTGGACTGTGTGCGACCGCGGGTGCGCGTCTCATCG
GCGCAACGACGATTATCGCGGTCGAGAGCGTCCCCGCCCGCATGGAGATGGCCCGCCGCATGGGCGCCGACCA
TGTCGTGGATTTCACGGTATCGGACCCCACTGCAGAAATCATGCGTCTGACCGACGGGCGCGGGGTAGATGTG
GCCATTGAGGCGCTCGGAAGACAGGAGACCTTCGAAGGTGCTCTTCGCGTCCTTAGGCCCGGAGGTACGCTGT
CGTCCCTTGGCGTCTATTCAGGTGATCTAAGAATTCCGCTCGACGGCTTCCTCGCAGGTTTGGGAGACCATAC
TATTCGGACCACCCTCTGCCCGGGCGGCAAGGAACGCATGCGTCGTTTGATGGAGGTGATCGCATCCGGCCGC
GTCGACACGCGTCCTCTGGTGACTCACCGCTTCAAGCTGGACCAAATCGAGGAGGCATACGACCTTTTTGCAA
ACCAGCGTGACGGGGTGCTCAAGGTCGCGATCAATCCATGAATGGATGACGACCTCTCTACTGGGCTTGGCCA
GAAGGAGGCGGAGGTCAGGCTCACGCAATTTGGGCCCAATATGCTTCCCGAACCACAGGCGTCCTCCCTATTC
GCAACATTCTTGCGCCAGTTTCGCAGTCCGCTGATCTATATTCTACTGGCCGCCACGCTTGTGTCGCTCGCGC
TCGGCGATGTGCGCGACGCTCTGTTCATCGCAATCGTCCTCGTCGCGAACGGCACTATAGGCTGCATGCAGGA
ACACTCGGCCGGCAAGGCGGCCCTCGCGCTGCGCAAGCTGGAGCAGCCTAAGGCCACCGTGGTCCGCGACGGA
CACGTGCAGGAGATCGACGCCCGCCTCCTGGTACCGGGTGATCTGGTCCTGATTGAAGCCGGCGGACGCGTGC
CGGCGGATCTCCGGCTTCTCTCAGCCACCGATCTTGTCTGCGACGAATCCCTGCTGACCGGAGAATCGGCACC
GGTCCATAAAACCCTAACTAGTGTTGACGCCACTCCCGAGGTGAATGCCCGCCTCATGGCCTTTGCGGGAACG
CTCGTAACGCGCGGCCGCGGACGGGGGTCAATTGCAGCGACGGGTGCGGCTACCGAGATTGGAAAAATCGCGG
CCGAGATCGGCAAGGCATCCGTCTCCAAGCCCCCCTTGATGATCAGAATGGAACGCTTTTCGCAGTCCATCGC
CTGGGTCGTCGCTGCTGCCGTAGTGCTTCTCATTCTCGTCGGGATCGCTCGGTCGATGTCGCCGAGCGATCTC
TTCATGATGTCGGTTGGCCTGGCGGTGAGCGCGATCCCGGAAGGGCTGCCGATAGCGATCTCGGTCGCGCTGG
CAATCAGCATGCGGCGCATGGCGAAAGCACATGTCATCGTGCGGCGGATGCCGGCCGTGGAAGCGCTTGGCTC
CTGCACCATGATCGCCACCGACAAGACGGGCACCTTGACGCTCAACGAACTGACCGTAACGGATATTCGCTTG
CCGGACGGCAATGAAATCGTCTGCGATACCGGTTCTGATCTGGACTCCTGCACGATCCGTGGCGATGGAACTC
CTCCCGCTGAGGCGCGGGAACGGGCCCTTGCCCTGTTAAAGGCCGCTTCGCTGCCAAATGAAGGATTGCTTAC
CCGAAAAGACAACGGCTGGACCGCCGTAGGGGATACGGTCGACATTGCGCTGCTGGCAGCGGCCTACAAGGGA
GGTCTTCGGCGTGAGGCAATCGAGGATGACTATCCTCTCGTCGCCCGCATTCCTTACGAGCCGGACCTGAAAT

ACGCCGCCTCATTTCACCGCCACGGCGACAGCATCCGCATCTTTGTAAAAGGTGCCGCAGAAACCCTGATCGA
CATGGCCGACAGGATGGACATGGATGGTCGCGCCGAGCCGATCGATCGGGAAGCCCTTCTTCGCCAAAAGGAA
GAGATGGCCGCGCGTGGCTTGCGCGTACTTGCCTTTGCGGAGGGCGAAACGGCGGTCGAGTCGGATGGAGGCT
TCGGTCGGCACCTGCTCGTCGATCTCGTCTTCCTGGGGCTCGCCGGAATGCAGGATCCCGTGCGGCCGGAGGT
CCCGCAGGCAATTCGCGATTGCCACTCAGCAGGGTTGGACGTGGCAATGGTCACTGGCGACGATCCCAAGACA
GCAGCGGCGATCGCCTCCCAGGCCGGGCTAATCTTCACCGAAGATCAGGTAGTCACCGGCGAAGCTGTGCGCA
GAGCAGAGGAGAACGGACAGGAAAGCCTCGACACACTGACCCGCCATGGCCGCATCTATGCACGTGTGGCTCC
TTCGCAAAAACTGGCACTCGTGCTCTCGCTCGCACGCAACGGCCACTTCGTCGCTGTCACGGGCGACGGCGTC
AACGATGCGCCGGCGCTGAAGCACGCGCATATCGGCGTTGCGATGGGACGAAAGGGGACGGAGGTGGCGAAGG
AAAGCGCCGATATCATCATCACCGATGATAACTTCGCTTCGATCGTCAGCGGAATCCGCGAGGGCCGGGTCGC
CTATGCGAATATCCGCAAGGTCATTTTCATGCTGATGTCGACAGGCGCCGCAGAACTGCTGCTCTTTCTGCTG
GCAATTCCACTCGGCTTGCCGATGCCCCTGCTACCCGTGCAATTGCTTTGGCTCAACCTTGTAACCAATGGCA
TTCAGGACATTGCCCTTGCCGGTGAAAGTCCAGAAGGCGACGAACTTAGCCGCGCGCCACGCAGGCCGTCCGA
ACCGATCTTCGATCGGCTGATGATCCGGCGCATATGGCAGTCCACGCTCGTCATGGGAGCCGGCGGTTTCGCG
ATGTTCTATGTTCTGCTCGAGCAGGGTTACGGCGAGAGCGAAGCCCGCAATCTGCTGCTGTTGTTGTTCGTGC
TTTTCGAGAACTTCCAGACGCTCGCCAGCCGCTCCGAGCGCAAGTCCGTCTTTCAGCTTGGCTTTCTTGCCAA
CCCGCTTCTCCTTCTCTCAATCGTAGCGGCCCAGGGCCTTCATATCGCTGCCATGTACACTCCGATCCTCAGT
GAGACGCTGCAAGTTTCACCGATCTCGTTCTCAGAGTGGGCGCTCCTGCTTGTAGCGGCATCGTCTGCCCTCC
TGGTCGTGGAAATTGACAAGTGGCGGGCTCGCCACACGGCGCGCGGACGATCTCGCGGGCAATGAATGAGCAT
TCGCAACCTTCACCATGCGATCGATCCAAGCTCTCTTGCGATAATTGGTGCGTCTGATAGCGACGGATCGCTT
GGCCGGGTGGTTATCGAGAATGTAATCCGGGCTGGATTTGAGGGAGAAATCTGGCCGATCAACCCTAAACATG
ACCAGGTCGCCGGCCATCGCTGCTATCGCCGAGTGGCGGATGTTCCCGGTGTGCCCGACCTTGCCGTCATTGT
CACTCCACCGCAGACCGTGCCGGCTCTGATCCATGATCTGGGTATAAAAGGAACGCGTGCTGCGGTGGTCATC
ACGGCCGGTATCAGCGCCGATCAAGACTTGAGACAAGCGATGCTCGACGCCGCAAAACCATTTCTCCTTCGCA
TAATCGGCCCCAACACAGTGGGCCTCATCGTCCCGTCCGCGAAGCTGAATGCGAGCTTCGCGCATTTGCAGGC
ACAGCCAGGCGGTATCGCGTTGCTTTCGCAATCTGGAGCGATCGCGACTTCGCTGATCGACTGGGCAGCCGAC
AACGATGTCGGGTTCTCCAAGGTCGTTTCCCTCGGCGACATGGCCGATGCAGACGCCGGCGATTTCCTCGATC
TGCTAGCTGGAGATCCCGAGACGCATGCGATCGTCATGTATCTCGAGGCCATCTCCAATCCACGGAAATTCCT
ATCGGCAGCACGGGCCGCGGCTCGGGTCAAACCGATCGTCGCCATCAAGGCGGGCCGCCACGCCGAGGCGGCC
AAAGCGGCCGCCACACATACAGGCGCGCTTTCAGGAGCCGATCGCGTCGTCGACGCGGCTTTGCGTCGTGCCG
GTATCTTGAGAATTGAAGGTCTCGGCGAACTGTTCGATGCGACGGAAACCATCGCGCGCTTTCCCCCTCTTGA
GCACAGCCGTGTCGCAATCGTCACCAATGGCGGCGGTGCCGGCGTTCTCGCCGTGGATCGGCTGATCGACTTC
GGGTGCGCGCTCGCGGATCTTTCTCCAGAGACCGTCGGCACCCTAGATCGAAATCTACCCGCCAATTGGTCGC
GCGCCAACCCCGTCGATATTGTCGGTGATGCACCACCCTACCGCTACAAGACGGCCGTTGAGACGATTGCTCG
GGATGTGGGAGTGGACATCCTGATCGTCATGAATTGCCCGACGGGTCTTGCGTCCCCCGTTGATGCGGCTCAT
GCGATCGCTTCCCTTGCGCAGGCCGGGACAATTTCCGGCAAACCGGTTCTCACATGCTGGCTGGGCGGTCGGA
CCGCTCGTGAGGGTCGCACTGTGCTGCAGCAGGCGGGATTGGCGAGCTACGATACGCCGTCTGACGTTGCCTT
GGCGGCTTCTTACCTCACCAAATGGTCGAAAGCGCAGCGGGTGTTGGTGAGGGTGCCAGAGGGCCGAGACGAC
GAAGTGCATTGCAACCGCGATCTGGCGCGATCCGTTCTCCAGAGGGTGGCGGCCGAAGGTCGCCGAATGCTGA
AGGAACCCGAAGCCAAGGCCGTTCTCGCAGCCTATGGCATCCCCGTCCCTCAAACGATGATCGCAACGTCGCC
GAAGGAGACTGAAGCGATCGCCGGCTTGCTTCTTGCGGGCGCGCCGAAGCTAGTGGTCAAATTGATTTCCAAG
TCGATCACGCACAAATCCGATGTGGGCGGTGTCGTCCTTGACATCCTGTCCCCCGTCGCAGCTCGCGAAGCTG
CCGAGGCAATCGCTGCGCGGCTCAAGGCGCATGACCCGACTGCCGTTGTAGACGGTTTTGCGGTGCAGCCGAT
GATTGAACGCAGACATGCGTGGGAACTGCTCCTCGGCGTCACCCGAGATCCGATCTTCGGTCCGGTCATTCTA
TTCGGATCGGGCGGCGTTTCCGTGGAAGTGGTCGCAGACACCGCGGTGGCGCTTCCCCCACTCGACGCCGTCC
TCGCCGGCGACCTGATCGATGAGACACGAGTTGGCAAGCTCCTCGCCGGTTTCCGCAGCGAGCCGCCGGCAGA
CAGAGCAGCGATCTGCAAAGCGCTCTCGGCTCTATCGCAGCTAATCGTCGATTTCCCATGCGTGCTTTCGATG
GATATCAACCCGCTGGTCGCCAGCGCCGAGGGCGTCATCGCGCTCGACGTGCGTATCGAAGTCGACCCACAGG
CTGTCGCGTGTCCGGGCCCCAATCGCGATCTTGCGATCCGCCCATATCCGGGCGATTGGCAAAAGCAGGTCAC
GCTTGGTGAGCGGATTTATCATCTGCGCCCGATCAGACCCGCAGACGCCGCACTGTATCCGGATTTTATGGCA
GAAACGTCTTCTGGTGACATCCATTTCCGTTTCCTCTCCTCGCGCAAGCACTTTCAGGACCAGATGTTGGTGA
GACTCACGCAGATCGACTACGAGCGCGAGATGGCCTTCGTCGCTCTCGATAATGAAAGTGACGAGCTTGCCGG
AATTGCCAGGCTTTACGCGGATCCCGATCACGAGTCCGCAGAATATGGCTTGCTGGTGAGGACGGATCTGCAG
GGGCGTGGCCTTGGCTGGGCTCTCCTGTCTCATCTGCGGGACTATGCTGCGGCCGATGGTCTGCAACGCATCG
AAGGCCTCGTTCTGAGTGAAAATTCGAAAATGCTGAAAATGTGCAGGGAATTCGGATTCTCAATCTCGACGCA
CCCGGCCGATGCCACCCTGAGGATAGCAACACTGGGACTGCAATGTCAGGCAGGTGTTTCTTGAATGAGCGTT
CAGGTCCGAAGCATCAAGGAAGGTCAACCGGCAGGCATTAGTTCTTCGAGTTTGCCTTCAGCGGATCTTGCTT
GCCTGTTCAGCCGCCAAGCGCTCGAAAAGTTCGAGCCGGGTGAAGCGATCTTCTGGGAGGGCGACGAGGCGAC

AAATCTTTTCGAAGTAGCAGATGGAATGCTGCGAGCACTGCGTTTGCTCGGCGACGGTCGTCGAATTATCGTG
GGCTTCATGCGCCCCGGAGATCTCCTCGGTGTGTCACTCAAGGAGCGTTATCTTTACACGGTCGAGGCAATCT
CCCCGGCGCAAATTCGCCGCTTTCCTCGTCGCGTCTTTGAAGACGAGGTGGCCCGCCATCCACGCTTACAGGA
GCAGCTATTCTCCAAGCTTCGTGATGAGATGACTGCTGCGCAGGATCAAGCCGTCCTTCTTTCCCGCAGGAGT
GCCGAGGAGAAGTTCGCCAACTTCCTGCTGCTGATGAAGGAGAGAGGGAACTCGGAGCATCGTGCCATTGTTG
ACCTCCCCATGACCCGACTGGATATCGCCAATTATCTTGGCATGACGATCGAGACCGTATCTCGCACTATAAC
AAGGCTAGCAAACGGCGGTATTATCGCGGCAGCAGGACGCCGCTCGATCAAGGTCTTGAAGATGGAAACGCTT
CGCCTGCTGGCCGAGGGTGACGAAGGCGAGCAATGGATGCCCTCTCTCGCTCGTCCTGGTCGGTACGCACCCG
CAAACGCTTGAATGCTGTCGCGGTATTTCGAGGAGGAAGGATACCGCGTTACCCTGGCCGGCGACGGACGGGA
GATGCGCGACTGCCTCGATAAACAATCAATCGACATCATTCTCCTCGATCTGGTACTGCCCGGCGAGGACGGG
CTGACGCTTGCGCGCGACCTCCGCGCGCGCTCGGATGTGCCTCTCATCATGCTGACGGGCCGCGACGACGTCG
TCGATCGGGTCGTTGGACTGGAGGTGGGTGCCGACGACTACATTGCCAAACCCTTCCATCTGCGCGAGGTGTT
GGCGCGCGTCAGGGGTGTTCTTCGTGGCCGTCAACCACAAGCGTCGGCGACTGTCGATCTGGCGTCAGAAGTC
TACTCATTTGAGGGGTTGCGCCTCGACATAGCGCGCCGCCAACTCACGTCGGATGATGGCAACGAGATCCTGC
TGACGACCGGCGAATTCGATATGCTCTGTGTCCTAGTGAAGCACGCCGGCCGTGTCTTGCAGCGCGAATTGCT
TATGGATCTGACACGAGGTCGTAATCTCGAAGCTTTCGACCGCACGATCGACGCGCAAATTTCCCGTTTGCGG
CGGAAAATCGAGCGTGACGCCAGCCAGCCAGCTCTGATCAAGTCGGTCCGAGGCGTCGGATATGTCTTCAGTG
CAAGGGCGACACGGCAGGAAGCGTAGATGAAGCGTCGCATTCTTTTGCAAGGCACCACTGCATTGATGGTGCT
GTCGGCACTTCCGGCTTTCGCGCAGGATGACATCAGGGCGGTCGCCCGTGAGGCGTATATCTACACCTATCCA
ATGGTGAAGAACTACCTCACGATGTACCAATACGCGCTGGACCCGGGCGGCAGCCAATACAAGGGGCCACTCA
ACACTCTGGTCAGCATCGCCCGGGTCTACACGCCTGAAGATACGGCAATCATCACGCCGAATTCCGATACGCC
CTATTCCTTCATCGTCTTCGATCTGAGGGCGGAGCCTGTCGTAGTGACGATGCCGCCAATCGAGAAGGATCGC
TACTATTCGCTGCAGCTCATCGATCTATACACCAATAATGTTGACTATCCGGGAACGCGGGTCGACGGCAATG
GCGGCGGCGATTTTCTAATCACCGGCCCCGGCTGGAAGGGCGATGTGCCGAAGGGCATTAAGCGCGTGATCGA
AATGCCGACCACGCTCGCGTTGGGCATCATCAGGACGCAGCTCTTCTCGCCCGACGATCTCGAGAAGGTGAAG
CAGATCCAGGCGCGCTACAAGGCCGCGGTCCTGTCGGCCTATGCGGGGGCTCCGGCTCCGGCTGCTCCTCCGT
CGATCGACTGGTTGCTTATCTCGGACGAGCTGATGGTCACGGACTACTGGTCGATTGCCGCCTTCCTGCTGCA
GTTCGCTCCACCATATTCGGGTGATGAGGCGCAGCGGGAAAACCTCGCCAAGCTTGGTATCCGGGATAGAGGA
GTGTGGCCCGGGACGGATCTTTCACCCGAGACTGTGGCACTGATGAAAGAAGTGGCGGTCGCGACGGAAAAGG
AGATTCGTGACGAGGCTGCCCGGTTGACGGATTCGTCCAAGATATTCGGTACGCCCGAATTCATGAAGGGCCG
GTTCATGGTCCGGGCCGCGGCGGCGCAGGGCGGGATCTATGGTAACTCCGTGCAGGAGGCACTTTATGTCATT
TATGCCTTCGACGCACAGAAGGCGCCACTTGACGGTAAGACAGGAAGATACAAGCTGACTTTCACCCCCAGGA
CGCTTCCACCGGTTGATGCTTTTTGGTCGCTGACCATGTATGATAGGCAAAATCAATTCCTTGTCGACAACCC
CATCGACCGTTATCTGATCAATTCGCCGATGCTTGCCGGACTCAAGAAGAGCAACAAGGGCGAGATCGTCCTG
TACCTGCAGCGCGAAAGCCCAGGAGCGGAGCTGGAATCAAACTGGCTCCCCGCCCCGAGCGAAATCTTCTATG
TGGTCATGAGGCTCTATCTGCCCAGGGCGGAGGCGTTGGACGGGCGCTGGGCGCCGCCGCCGATCGAAGCTCT
TTCGTGAATGCCGGCCGCCAAGGCGCAGGCACTGGTCCAGGGTCTGGTGATGATCGATGCCGATACGGCAGCC
GATCTCGCTGCCCTCGAACGGCTGGCGCTCTGGGCCTTGTCACAATATTCTCCGGTTGTCGCGATGGACCCTC
CCGACGGTATCGTCATGGATACCGAAGGCGCCGATCATCTTCAGGGCGGCGAGGACTTGATGCTGTCCGGCCT
CGTCAATCGCTTCCGCGGCCGGGGTCTCGCTGCCCGCGCGGCAATCGCCGATACCTGGGGTGCCGCGCATGCG
CTTGCCCGGACCACAGATCGCGAGACGGTCATCGTCGGGCGCGGCGATGCCGCCAGGGCCGCGAACCGACTGC
CACTGTCGTCGCTGCGGCTGCCGGCCGAAACTGTTCAAAGCCTTCGAACCCTCGGCTTTACCACCGTCGGCGA
CTTGGCTGCCACACCGCGGGCACCGCTGACGCTGCGCTTCGGCCTGGAGGTCGGCCGCCGTCTCGATCAGATG
TTCGGCCATATCGCTGAGCCGATAGATCCGATCCGGCCGGCGGAACTTGTCGAGGTCCAAAAATCTTTCGCTG
AGCCCATCGGCGCGCCGGAAACGATCGAGAAATATGTGAGCCGCCTGGTGCGGCAGCTCTGTCTTGAGCTCGA
GAAGCGCGGGCTTGGGGTCAGACGCGCCGACCTCATCGTCCATCGCGTCGATAACACCATTCAGGCACTGAGG
GCGGGGACGGCGAAGCCGGTCCGTGACATCGCCTGGCTGACGAAACTCTTCCGCGACCGCATCGAGAGGATCG
AACCAGGCTTCGGCATCGAGAAGCTCGGTCTTGCGGCCATCCTCGCCGAGCCGCTGATTGAGGCACAGTCAGC
CTCGTCACTCATTGAAGAACAGGTAACCGATGTGACGCCGCTCATCGACGTGCTTGGCAATCGCGGTGGACAG
CGGATATTCCGTGTGGCCCCGGTCGCAAGCGACGTCCCGGAACGTAGCGTCCGGCGGATCGCTCCGATCGCCG
ACGAGGACGGCGCCACCTGGCCGCTCAACTGGCCAAGACCGCCGCGGCTTCTGGCGCGTCCCGAACTGATCGA
AGTGATCGCGCTTTTGCCCGATCATCCGCCCGTCTCGTTCACGTGGCGCAGCAAGCGGCGCAGAGTGAAGCGC
GCCGACGGACCGGAGCGGATATTTGGGGAGTGGTGGAAACGCAGCTCAGAATGGGTTGCGGTCCGCGACTATT
TCGTCGTCGAGGATGATAGGGGCGAGCGCTTCTGGATTTTCCGATCCGGCGACGGCGTCGATGCGGAAACAGG
ATCGCATAAGTGGTTCATGCACGGGATGTTTGCGTGAATGAAGCGAATCGTCTTGCTGCTATCACCTCTGGTA
ATCTTGCTTAGCCCGATCATTGACGCGTTTCAGGGAGTGTATATTGACCCCAGAAGCGATGCAGGGTACGCCG
TCATCGCTTGTGTTGCCATAATTGGGCTCGCCCTGGGCATGATTGCAACCGTTTGCTATAAAAGGGGAGCTGT
TGGCAACGTCGTAACAGCAGGCACACTCGCCGTTACCCTTTTCCTTTTTGGCGATCTTTCATACGGTGTTTTC

TGGAGACTAGCAGACCACATCGGAATGGGGGGCGCCGCCGCATTGGCGTTTGCTGGACTGGTTGTACTTATTC
TTATCCTATTCAAGCTCATGGCCGCTGTGCCCCGTATGATGGCCGCATTTGCCGTAGCCCTACTGGGCTCTAC
AGTTCTTGATCGCGGCTTGATTACTGAGGCGAGCGCCGAAGAACCCGCGCCGGCCGTGATTTATATCGTGACT
GATGAAATGATCGGCATTTCCGGGATCGACACAAGATTACCCCATGGAGCAGAAGCCAAAGCCGCATTGTCGC
GGGTTTTCCAGAAACATGGGTTTCGGCTCCATTCCAAGGCTTTCAGCCGCCACATTCTGACTCAGGTTTCAGT
ACCAGCCGCCTTGAACATGGACTATTCTTATAATTTTCCCGGCGATAGATCGCATTATGCCTACCCGGGGGAG
GTAACAAAGTTCAAGGAACTGTCGTTATTCAATCTGTGGCATCGACAAGGATTATCCGTCAATGTCTTCCAGT
CTGCGCATCTTGATTACTGCAAAACTGAAGCGCTAGTCGACTGTCACACCTTCGCATCATTTGACGGAAGTAG
ATTTATTCAACGGCAGCGGATCGAGGGCGGCCCATACCGGGACGTTCCACCCGCTTCCGCTTCGCTGGCGGAT
GTTAAGGCGCTGGTCGAGGGAAACAGGCAAAGCCTTGCAATGGCAGCCGTTGGCTTCGTGGCCTCCAACTTGC
TGGAGACGAAGGAGACGGCACTTCAAGAATGGCACCCGCGATCTTACGACCAACTAGCCTTCCCTTACTGGCT
CGGACAGTTTCAGAACATGATTCTAGATAAAGGTCGTGGAAACGCCTTTTTTGCACATTTTCTATTTCCGCAT
AGTCCCTCGGTCTACAACAAAGATTGCAAACCAACGAACCGTTGGGTTGAGAGGTCCTATCTTACGGAAGTCC
GCGGGTTGGCAGGTCAAGAGCTAGATGAAGCGCGGGTGAAAGAATATGGATTCTATTTCGCTCAAACGCTGTG
TCTCGCCAAGCAACTTGATAAGTTCTTTAATGCGGTTCTCAGCGATAAGAGATTTCAGGACGCAACGATAGTC
GTTCATGGCGACCACGGCTCACGTATTTCTGCGGGTAGAAATGTCGAGACTATGTCTCCGAGGGATTACGTCG
ACAATTATTCCGCTCTTTACGCGGTCAGAAAGCCGGGCGTGGCGACGGGTACGGACTACAAGCTACGCTCGGT
CCAGTGGTTAAACGCTTCTCTGTTCCGTGGCAATGTATCAGAGGTTGCCCCGACCGTAGTAGGATCTATGCAG
GGTAAATCTGACGATCCAGCAGTGTACGCACCCATTACCGACCCAGACCACGTTGCTATAATGCCGATGCATG
ACTTTGACGCATCCCATTGAATGGCAAGCACCAATTTCCGTCCGCTGCACGACCGCGTTGTCGTCCGCCGCGT
CGAGTCTGAAGAAAAGACCAAGGGCGGCGTCATCATTCCGGACACCGCTAAGGAAAAGCCGCAGGAAGGCGAA
ATCGTGGCTGTCGGTTCGGGTGCCCGTGACGAAAGCGGCAAGGTTGTTCCGCTCGATGTGAAGGCTGGCGACC
GCATCCTGTTCGGCAAGTGGTCCGGCACCGAAGTCAAGATCAACGGCGAAGACCTTCTGATCATGAAGGAAGC
CGACATCATGGGTGTCATCGGCTGAATGCTTGTCAAGGATGTGATGACGACAAAGGTTGTCAAACTGTCGCCT
GACAACAGCGTTAGACAGGCAGCCAAGCTGATGTTCGACCACCACGTAAGCGGCGTCCCGGTCGTTGACGACG
ATGGGCGCTTGCTCGGCGTCATCAGTGAGGGCGACCTGATCCGGCGGACGGAACTCTGCAGTGGAGCATCCGT
GTTGATGGCGGAGATGGCGATCGATCCCGATGACAGGGCGAACGCCTTCATCAGGCGATGCTCGTGGCGCGTC
GGCGACGTCATGACGGCCAACCCCGTAACCATTGAGGAGGAGGCGCCCCTTGCGCGCGTTGCAGGGCTCATGC
AGGAACGCGGTATCAAGCGCATCCCCGTAATGCGCGATGGGGAACTGGTCGGCATCGTCAGCCGCGCCGACCT
GCTGCAGGCAATTTTTTCAACGAAGCCAGACGAAACAGCAGCGGGCGACGAAGCCATTCGACGCAGCATCCTT
GTTCGCCTCGGCGAAAACACGGGTCTGGAAGAACTGGACGTGACTGTCACGGTCACCGAGGGGATCGTGCACT
TTTGGGGCCAAGTCGAGACGGCCGCTTGTCGCAGGGCCGCGCGGATCGTGGCTGAGAGCGTCCACGGGGTCAG
GGGCATCGTGGAGCATTTTCCAGATCCCTACACTCAATAGGTGACGCAGTCTGCAGATAATAAAAGACCTGAC
TCCGGCGTGTCCGACGACTCGATCAACATGCTGCTGCGGTTTGCCGTTATGATGCTTCGTTCCGGGGGCACCG
CGATCCGTACCCGCGAACTGGTGGGGGCGATGGCGCACCGCCTCAACCTGGAAACGCCATCGGTGAGTCTCAC
GCTCGAGTCCGTCACCCTCAGTGTTTACCACGGCAGCGAACAGTTCGTAGCGATGCGCGAGGTCGGTCCGCCC
GGAATCGACTTCAGGCGCATCGGTGAGTTGGAGCGGCTTGCGAACGCGTTAGAGCCAACGGCACCGCCACACC
GGATAGCGAAAGAACTTGCTGAAATCGAAGCGCGGGCGCCTCAATATACCGGCTGGCAGATGGCGATCGCGAT
CGGCCTGGCCAGCGGAGGCTTTGCGTTCCTCAACGGTGCGGCCCTGCCGGAAATGGCCAGCGCCGCGATCGGT
GGCGGCATAGGTCAAGGATTGCGCTGGTGGTTGACCCGCCGCCAATTGACACACTTCGGCACGGCGGCACTGG
CTGCCATAACCGCTGCAGGAACTTACGTTCTGGTGGCAGCGCTGGCGCGCCGTGCAGGGTTTGCGTTCTCGCA
CTATGCTGCGGGCTTCATCTCCACCATACTATTTCTCATCCCCGGCGTTTCGCTGATTGCAGGACTGTTCGAT
CTGTTGCAGCATCAGACGGTGGCCGCCTTGAGCCGACTGGCATATGGCGCGCTGATCCTGTTCATCGTCGCTT
CGGGGCTGAGCATCGTGGTGACTGTTGCGGCCATCGAGCTGTTGCCACGCTCGGCGCCGGCCGAGCTTGCCTA
TCCGTTGCGCCTTTCGTTGCGCGCCGTCGCGAGCTTCGTTGCCGGCTGCGGCTTCGCCATGCTGTTCAACAGC
ACGCCACTTTCGTTGGTTGTCGCGGGCATCGTGGCGCTGGCGGCGAATAGCGTGCGCCTCGTCCTGATCGACA
TGGGAATGCTGTTGGCGCCGGCGGCGTTCATCGCCGCGTTGTTGATAGGAATCATCGCCATTCTTGTGAGCCG
GCGGTTGGACGCAGAGCTCATGGCGATTGTTACCGCGCCGGTCGTCATCATGATTCCGGGTCTCTACGTATTC
GAGATGCTTGTTCTGCTCAACCGGGGGCAGACGCTCGAGGCCATGCAGGCCTCGGGGGCAGGGATCTTCGTGA
TCAGCGCGCTCGCGATGGGGTTGAGCGTGGCGCGCCTCGCAGTCCCGTGGGAAAGTTAGATGATGCCGTTCAA
CCGGGCGAACAACGGCGTCGGTGCGCGCTCGTTTCGCGCTAAGTTCATCCTTGTCGTCGGCGGCGCCGTGCTT
TTCGACCTGCTCGTCACCGGCGGCCTTGCTCTCTGGAACGTGCAGCGACTCTCGCGAGACGCGACGGCACAAG
TCGGTGAGGGCCTTGAGGCGGCAAACGAGGACTATATCCGCTCCTACGCGGAAACGACGGCCGAGCGCGTCAA
TCTCCTTTTAGACCAAGTTCACTCTGACGTCGGCGCTCTGGCGGGTGTGCTGCAGGCGCAGATCGACCATCCC
GAAAGGGAAACTCAGATCGGCGAAGCCTTGGCCCGCGGTGCGCCCGAAGCCGTCACGGTCAGCTACAACGAGA
AGGGCGGGTGGGCACAGAACTTGCCTGGATCTTCGTCAGTCGTCAGCGTTTGGGGCTACCTGCTCGGCTCCAA
CCGCGTCCCGTTGCCGGAGGTGCAGCGAGAGATCGAGAACAGCGCGGTAGTTGACCTCGTTGCTCCTAGCCTT
CTGGCAAACGGCCTATCGAAACTGCAAATGTACTATATCGGCCCGAAGGAGCGGCCGATTTTTCGCACATCTC

CGTACACCGACCAGGCCCAGACCTTCGACAGGCTGTACCCGGGCCACAACCAGGCGGAATTCTGGCCGTTCTT
CTTCCCGGGACTGTACGAATCATGGGAGCAATGGGCGGCGGATCCAGCGTCCCGCCCGGTAGAAAACGACATC
ACACAGACTGCACCCTACACGGACGCGATCACCGGCAAGCTGATCGTCAGCTTCTTCCAGCCGCTCTGGTCGC
GCGGCCGTGACCGCGTCGCGGGTGCCGCCGGCGCCGATATCACCCTCGACCAACTGGCAGAGATTGTTGAGAA
TGTCAGGGTCGCTGACAAAGGCTTCGGCTTCCTCACCATGTCCGACGGCAATGTCGTCGCCATTAATCCGGTC
GGCGAAAAGGTGATCGGATTGCAGACCTCGAGCGACGTCAACGGCCAAGGTGTGACGGGTCTAAATCGCTCAT
TGCGCCACAGCGCACAATCGGCGATCGCCAAATTACCCTTGGAGCAGGACGGGCTGCTGCAGCATGTCCTGCT
TGAGGAAAACGGCGAGAAGGTGCCCTATCTCGTCATGCTGAAACAGTTGCAGCCGAGCAACCTTTGGGCCTCC
GGTCCGGTTCGTCGTGAATCCATGCTGCTTGGCATCGTGGTGCCGGAGCGTGAAATCTATGCCTCCCTCTTCG
CCGCACAGGCCGGCATCAACAAGGCGGCGAACAGAATCCTAGTCTATCAAGTGCTAGCTCTGCTCATCTCGCT
CCTTTTTGTCACAGCGGCCGTGTTCGCCATCTCCGGGCGGATCACGAGCGGCCTCAGCGCGCTTGCCGATGCG
GCAAAACGAATCCAGCTCAAGGACTATTCCGTCCGCGTCAACATTCCGACACGCGACGAGGTCGGCGAGGCCG
GAAACGCCTTTAACCGCATGGCCGAACAGATCAGCTATCACACCGAAAACCTGGAGCAACTCGTCCAGGATCG
AACGAAGGAGATTGAAGAGGCAAACCTGCAAATATCCGGCCTGAATGAGCAGCTGCGCAGCGAGAACGTGCGC
CTCGGGGCCGAACTGGACATCGCGCGCCAGATCCAGATGATGGTCCTGCCAAGGGCCAGCGAGCTTGAGGAGA
TCGCGGACCTGGAGATCGCTGCCTACATGCGACCCGCAGACGAGGTAGGCGGTGACTATTATGATGTGCTGAG
GGACGGAGAGCGGCTGAAGATCGGTATTGGCGATGTCACCGGACACGGCCTCGAGAGCGGCGTGCTCATGCTG
ATGGTGCAGTCGGTCGCACGGGCACTGCAGGAATCCGGTGAAATGGACCCCTCGCAGTTCCTCAACAGGCTCA
ATCGAGCGATTTACAAGAACATCGTCAGAACCAGCACGGACAAGCATCTGTCGCTTGCATTTTTGGACTACCA
GGCTGGACAGCTGACATTGTCGGGTCAGCATGAGGAAGTGATCATGGTTCGGGCTGCCGGAGCCGTCGAGCGA
ATTGATACGATCGATCTCGGTTTGCCGATTGGCTTGGAGTCCGACATCTCGGCCTTTGTGGCGACGCACGAGA
TCCCCTTTGCACGCGGCGACATGGCTATTCTTTACACAGACGGCGTGACCGAGGCTGAGAGTTGCAGCAGAGA
ACTGTTTGGAATCGAGCGGCTTTGCGCAAGCGCCCGTCGCCTTTCCGGTCGGAGCGCCGAGGAAGTCAAGACC
GGCATTCTCGACGACCTGGTGGCGCATATCGGCACGCAGAAGATCCATGACGACATCACGTTAGTAGTGATGA
GGCACAGGTAGATGAACCTATTCGGCACACTGAGGGCGCCGCGCGAACTGCTTTTCGGCGCCGGGCAGCGCCA
TGCCCTTGGGGGCATCGCCGCCAAGCTCGGCCATCGCGCGCTGATCGTCACGGATACGCGCCTTGCCGTGGAT
GCCGACTTGCTTGCACTGGTGCGGCGACTCGAAGAGGCAGGGCTTGAGGTGATGGTGGACAGCTCCACCCTGC
CGGACGTCCCGGTGGAATCGGCTATCGTGAGCGCTGCAGCGGCCAGAGGCTTTGCGCCGGACCTCGTCATCGG
CATCGGTGGCGGGTCCTGTCTCGACATGGCGAAATGCGTCACCCTCCTCCTTACCCACGGCGGGCGGCCACAG
GACTATTACGGCGAATATGCCGTGCCGGGACCCGTGATGCCATTGATCGCCATTCCCACCACCGCCGGCACCG
GCTCGGAGGTGACGCCCGTCGCCGTGCTTTCGGATGCCGAGCGTAGCCTGAAAGTTGGCATTTCAAGTCCGCA
CCTGATTCCGGCGGTATCGATCTGCGACCCCGAACTCACGCTCTCCTGCCCATCCGGCCTGACTGCGATCGCC
GGCGCGGACGCCCTGACGCATGCGATCGAAGCCTTCACGGCGATCCGGCGCGAGCCCGTCCCCGGTATCGCGC
AGCAGCGCGTCTTCGTCGGTAAGAACGAGCTTTCCGATCACTTTGCCCTTAGCGCCATTACCCTCCTGTGGCA
GGGGCTGGAGAGGGCCTGCAAGGACGGCGCCGACGCCGGCGCGCGCGAAAAGGTGATGCTCGGCGCAACGCTC
GCCGGTCTTGCTTTCGGGGTGGCGGGTACTGCCGCGGCCCACGCCATCCAGTACCCTGTCGGCGCGCTCACCC
ATACGGCGCATGGCCTCGGGGTCGCCTGTCTAATGCCCTATGTCATGACTTGGAATGCGCCGCTCATCCGCGA
CGAACTCGCGCAGATCGCGCATGCGGCCGGGCTCGGCGGACCGGATGAAGTGATCCCTGCCCTCGTTTTGCTC
TTCGAACGCATCGGCATTCCCGCCACCCTGCGGGACCTCGGCCTCGAGGAAGACCGGATCGATTGGGTGGCGG
AACAAAGCTCGGGTATCGCACGCCTAATCCAGAACAATCCCCGCCCCTTGAACCCGCACGAGATGCGCAACCT
CGTCGCCGCCGCCCATTGCGGCGATCGCTCCCGCCTGAACTGAATGACCATCCGTAAGATGCTTCTGGCATCG
GCCGCGATCACTTGCGCCGCGATGCCCGCCTCCGCATTTGCCGACACCTCGGCCAAGAAGATCGCCCTTTCGA
ACAACTATGCCGGCAATTCGTGGCGCCAGGCCATGCTGACGAGTTGGGAAAAGGTGACGGGCGAAGCCGTGAA
GGCCGGCATCGTCGCCTCGGCCGACGCCTTCACCACCGCCGAGAACCAGGCAACGGAACAGGCCGCGCAGATC
CAGAATATGATCCTGCAGGGCTATGACGCGATCGTGCTGAACGCCGCCTCCCCGACAGCGCTGAACGGCGCGG
TCAAGGAGGCCTGCGATGCCGGCATTACCGTCGTCTCCTTCGACGGCATCGTGACCGAACCCTGCGCCTGGCG
CATTGCCGTCGATTTCAAGGAAATGGGCCGCAGTCAGGTCGAGTATCTTTCGAACAAGCTTCCGGACGGCGGC
AATCTGCTCGAGATCCGCGGCCTTGCCGGCGTATTCGTCGACGACGAGATCTCGGCCGGCATCCATGAGGGCG
TCAAGCAGTTCCCCCAGTTCAAGATCGCCGGCTCCGTGCACGGAGACTGGGCGCAAGACGTGGCGCAGAAGGC
CGTTGCCGGCATCCTGCCGAGCTTGCCTGACATTGTCGGCGTGGTGACGCAGGGCGGAGACGGTTATGGTGCC
GCGCAGGCCATCGCCGCGACCGACCGGAAGATGCCGATCATCGTGATGGGCAATCGCGAGGATGAACTGAAAT
GGTGGAAGGAGCAGAAGGACGCGAACGGCTATGAAACCATGTCGGTCTCGATAGCGCCTGGTGTCTCCACTCT
CGCCTTCTGGGTCGCCCAGCAGATCCTCGACGGCAAGGAGGTAAAGAAGGACCTCGTCGTTCCCTTTCTGCGC
ATAGATCAGGACAATCTCGAAGCGAACCTCGCAAATACGCAGGCCGGTGGCGTCGCCAATGTGGAGTACACGC
AGGAAGACGCGATCAAGGTCATCGAGTCGGCAAAGTGAATGAGCAGTTTTCGACGGAAGTTGACGACTTCCGC
TGTCGCGGCCATCTGCAGCTTGGTTGCTTCCACAGCGGGCGCCCAGACGGTGTTGAAGGCGTCGCACCAATTC
CCGGGGGGCAAGGGCGACATTCGCGACGAGATGGTGCAACTGATCGCCCGCGAGGTCGCCGCCGCCAATGTCG
GCCTCGAAATCCAGGTGTTCCCGGGGTCGTCTCTTTACAAGCCGAACGATCAGTGGAATGCGGTTACACGTGG

CCTGCTCGATATGACTTCGTTCCCCTTGGACTATGCTTCCGGCCGCCACCCGGAATTCTCCGCGACGCTGATG
CCCGGCCTCGTGGGAAACTTCGACCGCGCCATGCGGCTTAACGACTCCGAGTTCATGGGGGACATAAAGAAGG
TCATCGAGAATGCCGGCGCCTTGGTGATCGCCGATGCGTGGCTCTCCGGTGCCTTCGCCTCCAAGAAGAATTG
CATCACCTCCCCCGACACGATCAAGGGACAGGTGATCCGCGCCGCCGGGCCGGCCTTCGAGGAGATGCTCGTG
GAAGCCGGCGCTTCGATCTCATCCATGCCTTCGTCGGAAATCTACACCGGCATGCAGACGGGCGTCCTGGATG
CGGCGAACACCTCGTCGGCGAGTTTCGTCTCCTATCGCCTGTTCGAACAGGCCAAGTGCCTCACGGCGCCGGG
AGAAAATGCCCTCTGGTTCATGTACGAACCGGTGCTGGTATCGAAGCGCGTCTTCGATGGCCTGACGGAAGAG
CAGCAAAAGGCAATCCTCGCTGCCGGCGAGAAAGCGGAAGTCTATTTCAACGAAGAGGTCCGCAAGGGCGATC
AGGTCATGATCGACACCTACAAGAAGGCAGGCGTCGAAGTCGTCGAGATGTCGAAGGAGGATTACGATGCCTG
GCTCGAGCTCGCCAAGGCGTCGTCCTACAAGAACTTCGCCGCCAATGTTCCCGGCGGCGACAAGCTGATCGAA
AAGGCGCTCGCCGTGAAGTAAATGCTCAAGGCCTATTCACAGGCTGTCGGCAATGCTTCGCGTGGCCTCGCGG
CCATTGCGACGGCCCTGCTCATTGCAGCGATGCTCGTCGTCTGCCAGATGATCATGCAGCGCTATGTATTTCG
TCAGGCGACGATCTGGCAGACGGACTTCGTCGTGTTTTCCGCAACCGCAGCCATGTTTCTCGGTGCGCCATAC
GTCTTGCTCAAGGGCGGCCATGTGGGCATCGACGTCGTCGAAATGGTGGTGGGCGAACGTACGCGATACGTTC
TGCGCATCATTGCGGGCCTGCTCGGGCTCCTGTTCTGCGCCGTCATGCTGATTGCCACATGGATTCAGTTCCA
TGACGCGTGGGCGGGAAACTGGAAACACTCCAGCGTCTGGGCGCCGCCGCTTTGGGTGCCGCTCGCTGCCCTG
CCCGTCAGCTTTGCCATGCTGTGCCTGCAATATGTCGCGCAGATTCTGACGCTCCTCACCGCCCCGGCAGTCC
CGGCAGCCATGGGCCACGGCGCGGCCGAACCGGGTTCGGCCCCTGGCGCCGACCGTCATCCACAGGAGATCAT
TCAGTGAATGATGCCGTTCGTCATGGACGAACTGCAGCGGAATTATACGGTTCACAGGCTTTACCAGGCCGCC
GATCGGCCGGCGCTCGAAGCGGCACTCCCATCGATCCGCGCGGTTGCTACGGGCGGCGGCGCGGGCCTTTCCA
ATGAATGGATGGAGAAGCTTCCATCACTGGGGATCATCGCAATCAACGGGGTCGGGACGGACAAGGTGGATCT
CGCCCGGGCGCGCCGTCGCAACATCGATGTGACGACGACGCCGGGTGTCCTCGCGGACGACGTCGCTGACCTC
GGTATGGCGCTCATGCTCGCCGTCCTGCGGAGGGTCGGCGATGGCGACAGGCTGGTGCGCGAAGGCCGCTGGG
CCGCTGGCGAACAACTACCGCTCGGCCATAGCCCGAAGGGAAAGCGGATCGGCGTACTCGGCCTCGGTCAGAT
CGGGCGGGCACTGGCGTCGCGCGCGGAGGCCTTCGGCATGTCGGTGCGATACTGGAACCGGTCCACCCTTTCC
GGCGTCGACTGGATCGCGCATCAGAGCCCGGTCGACCTCGCGCGCGACAGCGACGTTCTGGCCGTCTGCGTGG
CGGCGAGTGCGGCGACGCAGAACATCGTCGACGCGTCCCTTCTTCAGGCGCTCGGTCCCGAGGGTATCGTGGT
AAACGTCGCGCGTGGCAACGTCGTCGACGAGGACGCCCTGATCGAAGCGTTGAGGTCCGGAACGATCGCGGGT
GCCGGGCTCGATGTCTTCGTCAACGAACCCGCAATCCGCAGCGAATTCCATACCACGCCGAATACGGTGCTCA
TGCCGCATCAGGGCAGCGCGACGGTTGAGACGCGCATGGCCATGGGCAAGCTCGTGCTTGCAAATCTCGCCGC
CCACTTTGCCGGCGAAAAAGCGCCGAACACCGTCAATTGA
>5F12 Concatenation of 3 sequences
ATGGACATCAGCATTGCGCGTACGTTCCTCGAGGTGGTCAAGACGGGAAGCTTCGTGAATGCCGCTGCCAATC
TGAACATCTCCCAGACGGCAGTGAGTGCGCGAATTCGCGTACTCGAGGAACAACTCGATCGGCCGATGTTCGT
TCGGAACAAGGCCGGTGCCAGGCTGACCCCCGCCGGAGAGCAGTTTTTCCGTTTTGCAACGAGCCTAGTGCAA
GTATGGGAGGCGGCGCGAACCACGGTCGCTTTGCCCCCGGGACGCGAGGCCATGGTGACGATCGGTGCTGAGC
TCAGCCTGTGGAATCCGCTGTTGAAGCATTGGCTTTTGTGGATGCGCCGCGAATGCGCCAGCGTCGCCATTCG
AACGACGATAGACAGATCGGAGCGGCTGATGGAACTCGTGCAGGACGGTTCACTTGACGCGGCGGTCCTTTAT
GGAGCGCCAAATCGTCCAGGCACGCTTACGGAGCTGCTTTTCGAAGAAAAGCTCGTTCTCGTTCGCACTACAG
CTTTGTCGCAGCCGCCCGGACCGGAAGACCTCGTTGGCATCGATTGGGGAGAGGATTTTGCCGGCAGCTATAG
GGCGGCCTTTCCGGGCGGCCGGAATCCGGTGGTAGCGATCAGTTATGGGCCGCTTGCTCTCGATTACATTCTG
GAAGTCGGCGGATCCGGATATTTTCGGCAGGGCTTCGTTCGGCCCTATCTTGTGGACGGCCGCCTCGAGCTCG
TTCCCAACAGTCCGGAATTTGCCTACCCCGCTTATCTCGTTTCTTCCGCAAAGGCGGATCGGTCTATGCTGGA
TCGCATTCGGGCAGGATTGAGGGCCGCCGCATCCAGTGCCGGCGTCTGAATGTTCGGCATAGACATCACCGTC
CTGGGATTGGCCGGCCTCGTCGCCCTGGCGGCGGCAGCACTCGCCTATGGTGTGCTCTACCCCCAGATCGAGA
CCGAGAAGAAGGCGGAGGGCCGCCTGCGCCGGGTCAGCGCCTCCGAGACGGACCGCACCAAGATCAAGGCGGC
GCGCGACCGCGTCAACGAGATGTCGAAGCGACGCAAATCCGTTCAGGATTCGCTGAAGGAACTCGAGAAAAAG
CAGCAGGAAAAATCCGCCAATACGGCGCCTTCGATGAAGAAGCGCCTGCTGCAGGCCGGTCTTTCGATATCGC
TCGCGCAGTTCTACCTGTTCAGCGCGCTTTTCGGACTGTTCGCCTTCCTTGTCGTCCTCCTGGCAGGGGCGGG
GCTGCTCATTGCCGCGGGCGTTGCCCTGATAGGCGCGGCCGGCCTGCCGCGATGGATCGTCGGCTCCATGGTC
AAACGCCGTTGCAGAAAGTTTCTCGACGAGTTTCCGAATTCGCTCGATGTCATGGTCCGTTCGATCAAGTCGG
GGCTGCCGCTGAACGACGCCTTGCGGCTGATCGCCAGCGACGGGCAGGAGCCGGTCAGGACGGAATTCCGCCG
TGTCGTCGAGTCCCAGCAGGTGGGCCTCAACGTTCCCGAAGCCTGCGCCCGCATGATTCACAGCATCCCGCTG
CCGGAAGTGAACTTCTTCGCGATCGTCATCGCCATTCAGGCGCAGGCGGGCGGCAATCTGTCCGAAGCGCTCG
GCAATCTCTCCAAGGTGCTGCGCGAACGCAGGAAGATGAAGGCGAAGGTCAGCGCGCTGTCGATGGAGGCCAA
GGCGTCCGCCTGTATCATCGGCGCGCTGCCGTTCATCGTCGCAACCCTCGTCTATCTGACTTCGCCCGATTAC
ATGATGGTTCTCTTCACCGACCCGCGCGGCCACATCATCATGGGCGCTTCCGCCGTCTGGATGAGCATCGGCA
TCTGGGTGATGCGCAACATGATCAACTTCGACATCTGAGTGAATTCATCCGCACCCCCGATCCGCGTGCTCAG

CGCCGGGCTCGGCAATATGGGCCGCAGCCACGCGCTCGCCTATCATCAGAACCCCGGTTTCGAAATCGCCGGG
CTGGTCAACCGCTCGAAGGTGGCGCTGCCCGAGGCTCTTGCAGGATACGAGATTCTGCCGTCCTTTCCGGAGG
CGCTCGCCGAACTGGAGCCGGAGCTCTGCTCGATCAACACCTATTCGGACACGCATGCGGACTACGCCGTGAT
GGCGATGGAGGCGGGAGCCCATGTCTTCGTGGAGAAGCCGCTGGCAACGACGGTCGCCGATGCCGAGCGCGTC
GTCGCCTGTGCGCGGGCCAATGGCCGCAAGCTGGTCGTCGGCTATATCCTGCGCCACCACCCGTCCTGGATGC
GGCTGATCGCGGAAGCCCGCAAGCTCGGAGGGCCTTACGTCTTCCGCATGAACCTGAACCAGCAATCGAGCGG
CCCGACCTGGGCGACGCACAAGTCGCTGATGCAGACGACACCGCCGATCGTCGATTGCGGCGTCCACTATGTC
GACGTCATGTGCCAGATCACCGATGCCAACCCCGTCGAGGTGCGCGGCATGGGCTTGAGGCTCTCGAACGAGA
TAGCCGCGGATATGTACAATTACGGGCATCTGCAGGTGATCTTCGAGGACGGCTCGGTGGGCTGGTACGAGGC
GGGCTGGGGGCCGATGATCTCCGAGACGGCCTTCTTCGTGAAGGACGTGATGTCTCCGAACGGCTCCGTGTCG
ATCGTCATGGACCAGAACGCCAAGTCCGACGACATCGACGTGCACACGAAAACCTCCGTCATCCGCGTGCACA
GCGCCGCTACGGGACCGGACGGCCGGTTCCTGAAATCCGACGAGGACCTGGTGATGGAGGGCGAGCCCGGCCA
CCAGGAGCTTTGCGACCGCGAGCAGGCCTTCGTGCTCAAGGCCATCCGAGAAGACATCGATCTTGGCCGGCAC
ATGGACGATGCGGTGCAGTCGCTGAAGATCTGTCTCGCCGCCGACGACAGTGTGCGCACCGGCAAGCCGGTCA
AGCTTTAAATGACGAAATTTCGGAATTTCGTTGTCAGCTGCGCACTCGCCGCCGCCTCCGCGGTTCCCCCTGT
AACGTCCGTTCAGGCCATGCCGCGCCCGGCGCTCGACATTGCGATCAAAGCGCCGGCCGGCGTCGAGAATGCC
ACGCATCGCGGCCGTTACGGGTGGCGCGGCGGCTATTCTCGCCGCGGCTATTATGGAGGATCCGGCTTCTATC
CGGGCTGGAGCCCCTATTACAGGCGGTCCGGGATCAGCTTCTATTTCGGACCCAGCTACCGTTACCGCCCGTA
TTACGGGAACCGCTATTATTACGGACCGCGCTTTTATGGCCCCGGCTATTACAGCTATCCCGGGCCCGCATAT
TACGGTTATCGCTCCTATAGGCCACGCTACTATGGCGGCGGCTACGTCCGGAGCGGCGGCAACAGCCATGTCA
GCTGGTGCCTGTCGCGCTATCGGTCCTATAACCCTGCCTCCAACCGCTACCTGACCTATGGCGGGGTCTACCG
GGTCTGCTACTCGCCCTATCGTTAGATGACTTCGTTCTTTGATTCCGAATCTCCCTCCAACGTCGCCGAATAT
TCGGTGTCGGAATTGTCCGGCTCGATCAAGCGCACGGTCGAGCAGGCCTTCGAGCATGTCCGGGTGCGGGGCG
AGATTTCCGGCTACCGCGGCCCGCATTCCTCGGGACACGCTTATTTCGCCCTGAAGGACGATCGCGCCCGCAT
CGATGCGGTCGTCTGGAAGACCACCTTCGCGCGGCTGAAGTTCCGGCCGGAGGAGGGGATGGAGGTCATCGCG
ACCGGCAGGGTCACGACCTTCCCCGGCTCGTCCAAATATCAGATCGTCATCGACTCGCTTGAGCCGGCCGGTG
CCGGGGCGCTGATGGCGCTTATCGAGGAACGCAAGCGCAAGCTTGCGGCGGAGGGCCTTTTCGATGCGGGCCG
CAAGCGGCCGCTGCCTTTCATGCCGAGGGTCGTCGGCGTCGTGACCTCGCCGACCGGAGCTGTGATCCGCGAC
ATCCTGCACCGCATTGCCGACCGTTTCCCGGTCCATGTCATCGTCTGGCCGGTGCGCGTGCAGGGCGACGGCG
CCTGCGAGGAGATCGTGGCGGCGATCGAGGGTTTCAACGCGCTGCAGCCGGGCGGCTCCATTCCCCGGCCCGA
CGTGCTGATCGTCGCGCGCGGCGGCGGCAGCCTCGAGGATCTCTGGTGCTTCAACGACGAGGCGATGGTGCGC
GCCGCGGCGGCTTCCGCCATCCCTCTGATCTCGGCCGTCGGTCACGAGACGGACTGGACGCTGATCGACCATG
CGGCCGACCAGCGGGCGCCGACGCCGACCGGTGCGGCCGAAATGGCGGTGCCGGTGAAGGCCGATCTCGAGGC
CCAGCTCGCCAGTCTCGCAGCCCGGCTCAAGGGCGCCGCGGCGCGGCAGATGGACAATCGCCGCCAGACGCTG
CGCTCGCTTGCGCGGGCGCTACCCTCGCTCGACCAATTGCTCGCCTTGCCTCGGCGTCGCTTCGACGAGGCGG
CCGCCGGACTCGGCCGCGGCCTGCAGATGAACACCGCCAACAAGCGGCGAAGCTTCGAACGCAATGCGGCCCA
TCTCCGGCCGGAGCTCCTGACGGCAAGGATCGTCGATCGCCGTCAGCGCGTCTTGGATGCCGTCAACCGGGCC
GAGCGCATCGTCGAGCGTAAGGTTCAACGTGGCGCTCAGCGCATTTCGTCTGCCGATGCCTCGCTCCGCGTTC
TGCCGTCGCGGCTGATCGGCCAGATTCATCGCGCATCGGATCGCGTTTCCAGCCTGGGCAGGCGTGGGGATGC
GGCGATCGCCGCCGATCTGCGCAGGATGAAAAGCGCGCTTGCAGCACAGGACCGCGTGCTGCAGTCGCTCTCC
TATCACAGCGTCCTGCAGCGCGGCTTCGCGCTGGTGCGCGATGCCGCGGGCGAGCCGGTTAAACAGGCGGCTG
CCGTGCATCCCGGGATGGCGCTTTCGCTGGAATTCGCCGATGGCCGCATCGCGGCGGTCGCCGGCGAGGACGG
CACTGCGCCGCAGTCGCCGAAGAAGCGGCCTGCACGCTCCGGCGAGCCGACGAAGCAGGGCAGCCTCTTCTGA
ATGAATTTCTCACGCTTCTTCGTCGACCGCCCGGTCTTCGCGGGCGTTCTTTCCGTGATCATCTTCGTCGCGG
GCCTGATCGGCATGACCGGCCTGCCGATTTCCGAATATCCGGAAGTCGTGCCGCCGCAGATCGTCGTCAGGGC
GCAATATCCGGGCGCCAACCCGGCCGTCATCGCCGAAACCGTCGCCACACCGCTCGAAGAGCAGATCAACGGC
GTCGAGGGCATGCTCTACATGCAGAGCCAGGCGACCGCCGATGGCCTGATGACGCTGACCGTCACCTTCGAGC
TCGGAACCGATCCGGATCAGGCCCAGCAGCTGGTGCAGAACCGCGTCTCGCAGGGCGAGCCGCGGCTGCCGGA
AGAGGTGCGCCGGCTCGGCGTCACCACCGTCAAGAGCTCGCCCGACCTGACGCTCGTCGTGCACCTGATTTCA
CCCAACGGGCAATACGACATCAACTATCTGCGCAACTACGGCGTCCTGAACGTCAAGGACCGGCTGGCGCGCG
TAGATGGTGTCGGCCAGGTGCAGATCTTCGGCGGCGGCGACTATTCCATGCGCGTCTGGATCGACCCCGAAAA
GGCCGCAGAGCGCGGGCTTGCCGCGAGCGACATCGCCAATGCCGTCCGCGGGCAGAACGTCCAGGCAGCCGCC
GGCGTCATCGGCGCTTCGCCGTCGGTTCCCGGCCTGGACCTGCAACTCTCCGTCAACGCTCAGGGCCGCCTCA
AGACACCCGAGGATTTTGCCGACATCGTCGTCAAATCGGGCGAGAGCGGGGAGATCACGCGCCTGGGCGACGT
CGCCCGCGTCGAAATGGGAGCCGCTGACTACTCGCTGCGCTCGCTTCTCGACAACAAGGCCGCCGTCGGCATG
GGCGTGTTCCAGGCGCCCGGCTCGAACGCCATCCAGATCTCCGAAAACGTCCACAAGGTCATGGCCGAGCTGA
AGCAGACCATGCCTGATGGCGTCGATTACGAGATCGTCTACGACACGACGCAGTTCGTCCGGGCGTCGATCGA
ATCGGTCATCCACACGCTGCTCGAAGCCATCGCGCTCGTGGTCGTCGTCGTCATCGTCTTCCTGCAGACCTGG

CGCGCCTCGATCATCCCGCTCGTCGCCGTGCCCGTCTCGATCGTCGGTACCTTCGCCGTGATGTATGTCTTCG
GCTTCTCGATCAACGCGCTCAGCCTCTTCGGTCTGGTGCTGGCGATCGGCATCGTCGTCGACGACGCCATCGT
CGTCGTGGAGAACGTCGAACGCAATATCTCCCAGGGCCTCTCACCGGTGCAGGCGACCTATCGCGCCATGCAG
GAGGTCTCTGGGCCGATCATCGCGATCGCGCTGGTCCTCGTCGCGGTCTTCGTGCCGCTCGCCTTCATCACCG
GGCTCACCGGCCAGTTCTACCGCCAGTTCGCGCTGACCATCGCGATTTCGACGGTGATTTCGGCCCTCAACTC
GCTGACGCTCTCGCCGGCGCTCGCCGCCCTTCTCCTGAAGGACCACCACGCCCCGAAGGACCGGCTGACGCGG
GTCATGGATGGGTTGTTCGGCTGGTTCTTCCGCGGCTTCAACCGCTTCTTCGGAGCGAGTTCGGAGGCCTATG
GCCGCGGCGTCGGCGGCATCCTCACCCGCAAGTCGCTGGTCATGGGCGTCTATGTCGTCCTCCTCGGCGTCAC
CTTCGTGCTCTTCCGCGCGGTGCCCGGCGGCTTCGTGCCGGCGCAGGACAAGCAATACCTCATCGGCTTTGCG
CAGCTGCCGGACGCGGCGACGCTCGATCGCTCGGAAGATGTCATCCGGCGCATGAGCGAGATCGCCCTCAAGC
ACCCGGGCGTCGAACACGCCATCGCCTTCCCGGGCCTGTCGATCAACGGCTTCACCAATTCGTCCAACTCCGG
CATCGTCTTCGTGTCGCTGAAGCCGTTCGAGGAGCGTACGACGCCCGAACTTTCGGGCGGCGCCATCGCCATG
CAGTTGAACCAGGAGTTCGGGGCGATACAGGATGCCTTCATCGCCATGTTCCCGCCACCGCCCGTCCAGGGTC
TCGGCACGACCGGCGGCTTCAAGCTGCAGATCGAGGACAGGAACGGCCTCGGCTACCGGACGCTGGACGATGC
GACCAAGGCCGTCCTCGCCAAGGCGATGGCGGCACCGGAACTCGCCGGGCTCTATTCGAGCTTCCAGATCAAC
GTGCCGCAGCTCTATGCCGACCTCGACCGCACCAAGGCCCGTCAGCTCGGCGTCGCGGTCACGGACGTGTTCG
AGACGCTGCAGATCTATCTTGGCTCGCTCTACGTCAACGACTTCAACGCTTTCGGCCGCACCTACAGCGTGCG
CATCCAGGCCGATGCGAGCTATCGCAGCCACGCCGACGACATCGGCAAGCTGAAGGTGCGTTCGCAGACGGGC
GAGATGATCCCGCTGTCGGCCCTGCTCAACGTCGAGCAGACGGTCGGCGCCGAGCGGGCCATCCGCTACAACG
GCTTCCTCGCCGCCGATATCAATGGCGGACCGGCACCCGGCTTCTCTTCCGGTCAGGCGCAGGCGGCCATCGA
GCGGATTGCGGCCGAGACTCTGCCGCCCGGCATTTCATACGAGTGGACGGACCTGACCTACCAGCAAATCCTC
GCCGGCAATTCTGGCATCCTCGTCTTCCCGCTGGCGCTGCTGCTCGTCTATCTCGTGCTTGCGGCCCAGTATG
AGAGCCTGCTGCTGCCGATCGCGATCATCCTGATCGTGCCGATGGGCATCATGGCGGCGCTGACGGGCGTCTG
GCTGACCGGCGGCGACAACAACGTCTTCACCCAGATCGGCCTAATCGTTCTGGTGGGTCTGTCGGCAAAGAAC
GCTATCCTGATCGTCGAATTCGCCCGCGAACTGGAACTGTCCGGCAGCAATGCCGTCAGTGCGGCGATCGAGG
CGAGCCGGCTCCGGCTCAGGCCGATCCTGATGACCTCGATGGCCTTCATCATGGGTGTCGTGCCGCTCGTCAC
CTCCACCGGCGCCGGCGCGGAAATGCGCTCCGCCATGGGCGTGGCGGTCTTTGCCGGCATGATCGGCGTCACG
GCCTTCGGCATCTTCATGACGCCCGTCTTCTATGTGCTCATCCGCAAGCTTTCCGGCGAAAAGCCGCTGAAGC
ACACCGGAGCGCATATAGAAGCCCCGCATCTCGCCCCCGGCGAGTAGATGTCACTGACAGATGGCCCCTATGC
GGGGCCGCCCCAGCCGGATGTGGTCGACCCGAGCGGCGGGGCCAATCAAACGCGCGTGCGCGCCTATAACGAA
CGGCTGGTCATGTCGCTGGTGCGCCGTCACGGCAGCTTGTCCAAGGCCGAGATCGCGCGCCGATCCGGTCTAT
CGGCGCAGACCGCCTCCGTCATCATGCGGTCGCTCGAAGCCGACGGGCTGCTCGTCCGCGGCGCGCCGGTGCG
CGGCCGGGTTGGACAGCCCTCCATCCCCATGCGGCTCAATCCGGATGCGGTCTATTCCTTCGGCGTGAAGATC
GGCCGGCGCAGCGCCGATCTGGTGCTGATGGATTTTCTCGGCACCATCCGGCTGCACCTGCATCAGATCCATA
CCTATCCGCTGCCCGAGGAGATCGTCAGCTTCATCGTCGGGGGCATCGGCAAGCTCGAGGGACAGCTTTGCCC
CGGCGAGCGCGGCCGCATTGCCGGCGTCGGCATCGCCACGCCGTTCGAACTGTGGAACTGGTCGGAGGAAGTC
GGCGCGCCGCGCGAAGAGATGGACAGGTGGCGCGATTTCGACCTGCAGGCGGCGGTCGCCTCGCGCATCCGGC
ATCCGGTTTTCCTTCAGAACGACGGAACCAGCGCCTGCGGTGCCGAACTCGCCTTTGGTGTGGGCGCCAGTTA
TCCCGATTTCGTCTATTTCTATATCGGCTCCTTTATCGGCGGCGGCGTCGTGATCAACTCCGCCCTCTTTTCC
GGACGAACCGGCACCGCCGGCGCCGTCGGCCCGCTGCCTGTCGCCGGCAAGGACGGCAAGTCGACGCAGTTGC
TGAAAATCGCCTCGGTCTTCGTCCTGGAAAAGCTCCTGCGCGAACGCGGCATCGACCCGGAGCCGCTCTGGTA
CTCCGCCGACGACTGGATCGATTTCGGCGAACCGCTCGAAATATGGATTCAGGATGCGGCGGCAGCGCTTGCG
CAAGCCGTCGTCTCCGCGGTTTCCGTCGTCGATTTTTCGGCGGCCGTGATCGACGGCGGCTTTCCGCCCTGGG
TGCGTGCGCGCCTTCTTGCCGCGACGCGAAAGGCCCTCCAGACGCTCGACCTGCAGGGCGTCACGGTTCCGGA
CCTCGTCGAAGGCACCGTCGGCAGCCACGCCCGCGCGATCGGCGGCGCAAGCCTGCCGCTCTTCTCCCGCTAT
CTGCTGGACACCAATGTCCTCTTCAAGGAGCTTTAATTGAGGAACGAGGATATCGTGTTGGATGGCGCGAAGG
TGTTCTGGCGCACCCTGCTGCCGAGGTTGAAGGCGGAGCGCGGGCTTCTCGGCCTGTCCGTCGTGCTCGCCTT
CCTCGCCGCACTCGCGGGCATCCATCCGCTGGCCGTCCTGCCTTTCTGGATCGTGCTCGTCGCGGCCATCCTC
AACCGGGGAGCGCCGCCCGTCCAGACAGCGCCGGCGAGCGTCGGCGCTGCTGTGGAAACCAAGGCCGACCCGC
TTGCTCCCCTGCTCGACGCGCTCGACATGCCGGTGCTCGTCGTGGCGGCCGACGAGACCGTCGTCTTCCAGAA
CGCCGCCGCAGAGAAGGCCTTCGGAACTATCCCCCAGGACAGCTACCTTTCCGCCCGCGTCCGTTCGCCCGGC
ATTCTCGACATGGTGCGCGAGACGATCGCGACAGGCAAGGTCAACCAGATCGAGCATGCGGAAAGGCTGCCTT
CGGAGGCGGTCTACGTGGTTCGCGTCGCGCCCGTGGACATCGGCTCCGACGTTTCCGGCCGGCGCATCTTTCT
GCTGACCTATCGCGATATCTCGCAGGCTCGCCGCATCGACCGGATGCGCTCCGATTTCGTCGCCAATGCCAGC
CATGAGCTGCGCACGCCGCTCGCATCGCTGCGCGGGTTCATCGAGACGCTGCAAGGCCCTGCCCGCGACGACC
GGAAGGCCCAGGAGAAATTCTTCGGCATCATGCACGAGCAGGTCACGCGCATGAGCCGGCTCGTGGACGACCT
CCTGTCGCTGTCGCGCCTCGAGTTGAAGTCGCACATCGCGCCGGACGATGCCGTCGATCTCGGGCCGCTGCTC
GGACACGTGCGCGACAGCCTGGCGCCGCTTGCGGGCGAACTCGACGTGACGATCGCGCTGGAGGTTCCGGAGA
[truncated: 472,153 more chars]
